# Ultrafast photophysics of *para*-substituted 2,5-bis(arylethynyl) rhodacyclopentadienes: thermally activated intersystem crossing†

**DOI:** 10.1039/d4sc04306e

**Published:** 2024-08-15

**Authors:** Zilong Guo, Yaxin Wang, Julia Heitmüller, Carolin Sieck, Andreas Prüfer, Philipp Ralle, Andreas Steffen, Petr Henke, Peter R. Ogilby, Todd B. Marder, Xiaonan Ma, Tobias Brixner

**Affiliations:** a Institut für Physikalische und Theoretische Chemie, Julius-Maximilians-Universität Würzburg Am Hubland 97074 Würzburg Germany tobias.brixner@uni-wuerzburg.de; b Institute of Molecular Plus, Tianjin University Tianjin 300072 P. R. China xiaonanma@tju.edu.cn; c Institut für Anorganische Chemie, Julius-Maximilians-Universität Würzburg Am Hubland 97074 Würzburg Germany todd.marder@uni-wuerzburg.de; d Department of Chemistry and Chemical Biology, TU Dortmund University Otto-Hahn-Str. 6 44227 Dortmund Germany; e Department of Chemistry, Aarhus University Aarhus DK-8000 Denmark progilby@chem.au.dk; f Institute for Sustainable Chemistry and Catalysis with Boron (ICB), Julius-Maximilians-Universität Würzburg Am Hubland 97074 Würzburg Germany; g Faculty of Science, Charles University Hlavova 2030 128 43 Prague 2 Czech Republic

## Abstract

2,5-Bis(phenylethynyl) rhodacyclopentadienes (RCPDs), as a type of Rh(iii) complex, exhibit unusually intense fluorescence and slow intersystem crossing (ISC) due to weak metal–ligand interactions. However, details on their ultrafast photophysics and ISC dynamics are limited. In this work, electronic relaxation upon photoexcitation of two substituted RCPDs with two –CO_2_Me (A-RC-A) or –NMe_2_/–CO_2_Me (D-RC-A) end groups are comprehensively investigated using femtosecond transient absorption spectroscopy and theoretical analysis. Upon ultraviolet and visible excitation, dephasing of vibrational coherence, charge transfer, conformation relaxation, and ISC are observed experimentally. By calculating the spin–orbit coupling, reorganization energy, and adiabatic energy gap of plausible ISC channels, semi-classical Marcus theory revealed the dominance of thermally activated ISC (S_1_ → T_2_) for both D-RC-A and A-RC-A, while S_1_ → T_1_ channels are largely blocked due to high ISC barriers. With weak spin–orbit coupling, such differences in plausible ISC channels are predominately tuned by energetic parameters. Singlet oxygen sensitization studies of A-RC-A provide additional insight into the excited-state behavior of this complex.

## Abstract 2

Full photophysical picture and thermally activated ISC was revealed for rhodacyclopentadienes by fs-spectroscopy and Marcus analysis, while potential application on ^1^O_2_ sensitizing was explored.

## Graphical abstract

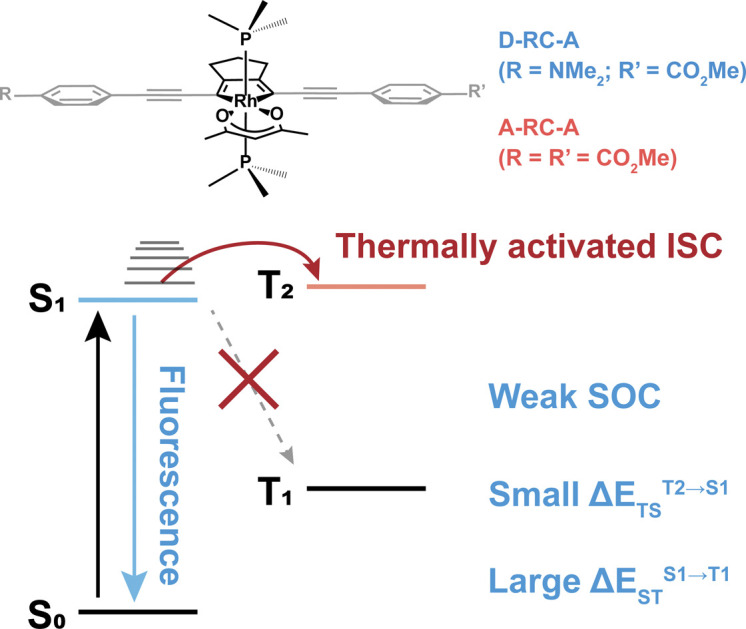

## Introduction

Organometallic complexes have attracted attention for a wide range of applications in photocatalysis,^1–5^ bioimaging,^6–8^ sensing,^9,10^ and organic light-emitting diode (OLED) devices.^11–14^ The spin-forbidden nature of the singlet–triplet transition often leads to slow intersystem crossing (ISC) in organic systems. However, by incorporating heavy transition metals, the ISC rate constant (*k*_ISC_) can be significantly increased to typically greater than 10^10^ s^−1^,^15^ even up to 10^13^–10^14^ s^−1^ in Ru(ii) complexes,^16–19^ leading to significant fluorescence quenching and enhanced phosphorescence emission. Efficient ISC can be primarily attributed to strong spin–orbit coupling (SOC) due to participation of the d-orbitals of heavy metals, which can significantly alter the photophysics due to strong metal–ligand interactions *via* metal-to-ligand charge transfer (MLCT) states that dominate electronic relaxation.^20–24^

Unlike complexes with ultrafast ISC, 2,5-bis(phenylethynyl) rhodacyclopentadienes (RCPDs)^25–27^ incorporating Rh(iii) exhibit up to 69% fluorescence quantum yields (*Φ*_f_) with *k*_ISC_ = 10^7^–10^8^ s^−1^ which is several orders of magnitude slower than that in Ru(ii) complexes.^17^ Intriguingly, by replacing Rh(iii) with much heavier Ir(iii), the corresponding iridacyclopentadienes^28^ are still highly fluorescent. Such photophysics was attributed to weakened SOC due to low d-orbital participation in the frontier orbitals and an enlarged S_1_–T_1_ energy gap (Δ*E*_ST_).^28,29^ Meanwhile, the T_2_ state was reported to be an ISC destination through a thermally activated mechanism in RCPDs,^28^ which increased the complexity of their electronic relaxation. Investigations indicated that the fluorescence properties of RCPDs can be effectively tuned by introducing an electron donor (D) or acceptor (A) at the *para*-positions of the arylethynyl groups.^28,30,31^ In particular, symmetric substitution with A/A groups (–CO_2_Me or –BMes_2_) (Mes = mesityl = 2,4,6-Me_3_C_6_H_2_) at the *para*-positions leads to significantly higher *Φ*_f_ than corresponding D/A (–NMe_2_/–CO_2_Me) and unsubstituted RCPDs (see Table S1†). In a simple excited-state model, enhanced fluorescence emission can be attributed either to slow non-radiative relaxation of the S_1_ state *via* dark states or suppressed ISC to the triplet state.^32,33^ Although *k*_ISC_ of RCPDs was determined to be 10^7^–10^8^ s^−1^ using picosecond time-resolved infrared spectroscopy,^30^ a full picture of their S_1_ relaxation remains unclear. Therefore, a comprehensive picture of the photophysics of RCPDs with weak metal–ligand interactions might be helpful for future rational design of fluorescent emitters.

In general, the ISC rate constant *k*_ISC_ between singlet (S_*m*_, *m* ≥ 1) and triplet (T_*n*_, *n* ≥ 1) excited states can be expressed *via* the Fermi golden rule,^34,35^1
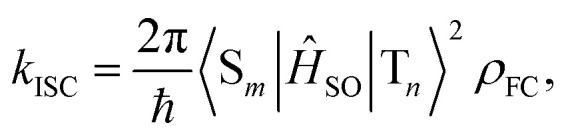
in which *k*_ISC_ is determined by the SOC matrix element 〈S_*m*_|*Ĥ*_SO_|T_*n*_〉, as well as the Franck–Condon-weighted density of states *ρ*_FC_, which can be described in the framework of Marcus–Levich–Jortner theory:^36–39^2
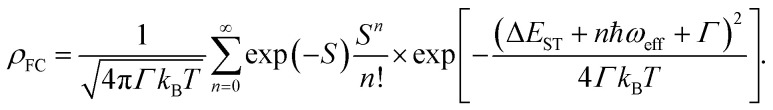
In a semi-classical approximation at high temperature *T*, with *ℏω*_eff_ ≪ *k*_B_*T*, the Huang–Rhys factor (*S*) and effective frequency (*ω*_eff_) of internal vibrational modes can be ignored. The density *ρ*_FC_ follows the standard Arrhenius-type equation and *k*_ISC_ can be obtained from the Marcus-theory equation:^40–43^3
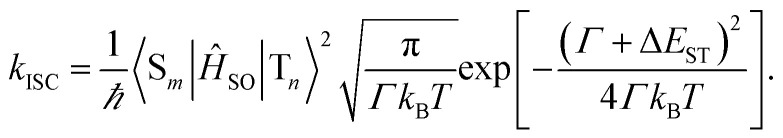
Here, 〈S_*m*_|*Ĥ*_SO_|T_*n*_〉 describes the SOC matrix element,^44,45^ while *Γ* and Δ*E*_ST_ represent the reorganization energy and adiabatic energy gap of the ISC (S_*m*_ → T_*n*_, *m*, *n* ≥ 1) transition, respectively. Eqn (3) has been widely employed for estimating ISC and reverse ISC rates in medium-size organic molecules and complexes.^46–49^

In cases of ultrafast ISC (*k*_ISC_ > 10^12^ s^−1^) of complexes with late transition metals, extremely high *k*_ISC_ is observed due to a large coupling term 〈S_*m*_|*Ĥ*_SO_|T_*n*_〉 up to several thousands of cm^−1^, which greatly amplifies the ISC rate associated with the ISC barrier (*E*_a_) determined by *Γ* and Δ*E*_ST_. However, RCPDs exhibit much slower ISC (*k*_ISC_ = 10^8^–10^9^ s^−1^) due to the lack of strong metal–ligand interactions,^30^*i.e.*, 〈S_*m*_|*Ĥ*_SO_|T_*n*_〉 in eqn (3) is expected to be small and relatively sensitive to *para*-substitution on the aryl ligand. It is hypothesized that the *k*_ISC_ may vary significantly with the reorganization energy and adiabatic energy gap subject to para-substitution, which determine the ISC barrier (*E*_a_) thermodynamically and can be discussed within the framework of Marcus theory.

**Scheme 1:**
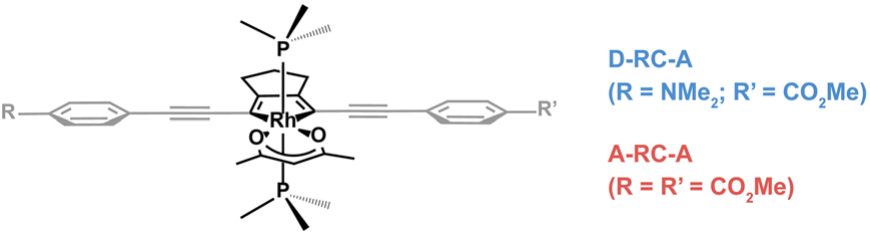
Chemical structures of investigated D-RC-A and A-RC-A. The Rh(iii)–ligand core is illustrated in black while the peripheral groups are in gray. The phosphine ligand is PMe_3_.

In this work, two *para*-substituted 2,5- bis(arylethynyl)rhodacyclopentadiene complexes (Scheme 1) were investigated by using excitation-wavelength-(*λ*_ex_-)dependent femtosecond transient absorption (fs-TA) measurements and time-dependent density-functional theory (TD-DFT) calculations. Compared with A-RC-A (*τ*_S_1__ = 1.7 ns) substituted with two –CO_2_Me groups, the D-RC-A with –CO_2_Me/–NMe_2_ substitution exhibits a shorter (*τ*_S_1__ = 0.8 ns) lifetime of the S_1_ state in toluene solution.^31^ By resolving fs-TA data upon ultraviolet (UV) and visible excitation, the electronic relaxation channels of D-RC-A and A-RC-A are largely revealed. Moreover, by applying semi-classical Marcus theory, we determine that ISC of both D-RC-A and A-RC-A rely on a thermally activated S_1_ → T_2_ transition rather than the channel that directly populates the T_1_ state. We also report studies of singlet oxygen, O_2_(a^1^Δ_g_), sensitization by A-RC-A, which provides additional insight into the excited-state behavior of this complex.

## Results and discussion

### Static absorption spectra

**Fig. 1:**
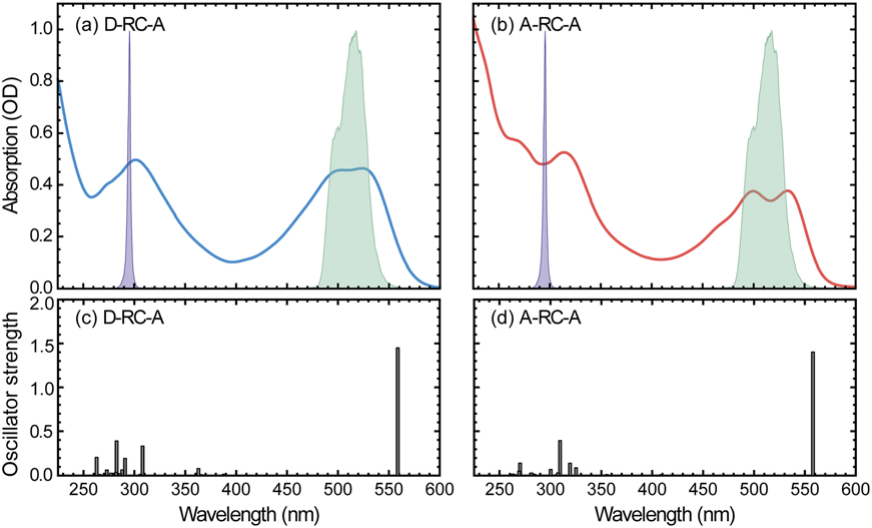
(a and b) Static UV/visible absorption spectra of D-RC-A ((a), blue) and A-RC-A ((b), red) in THF solution (10^−5^ mol L^−1^), overlapped with spectra of UV (violet shaded) and visible (green shaded) excitation pulses employed in the fs-TA experiments. (c and d) TD-DFT-calculated vertical excitation energies and oscillator strengths of D-RC-A (c) and A-RC-A (d).

The static absorption spectra of D-RC-A and A-RC-A in THF are displayed in Fig. 1 along with the calculated vertical excitation energies and oscillator strengths, while the corresponding calculation results are listed in Table S2.† Note that the oscillator strength (*f*) here is not referenced to the transition probability for a freely oscillating electron in a single atom, but reflects only the transition dipole moment and the energy gap between two states of the RCPDs. For both D-RC-A and A-RC-A, the pronounced absorption in the 450–550 nm region can be attributed to the S_1_ excited state, while higher singlet (S_*m*_, *m* > 1) states comprise the intense absorption in the UV region. As a result, upon visible (*λ*_ex_ = 513 nm) and UV (*λ*_ex_ = 295 nm) optical excitation, direct population of the S_1_ and S_*m*_ states can be expected in fs-TA experiments, as described in detail below.

The observed S_1_ band of D-RC-A exhibits less pronounced vibronic progression than that of A-RC-A. Reduced vibronic progression might be attributed to stronger solute–solvent interaction due to excited-state charge transfer (CT) between substituted D and A groups, which was confirmed by natural transition orbital (NTO) analysis of the S_1_ state of both RCPDs. As illustrated in Fig. S1,† A-RC-A exhibits a symmetric distribution of both hole and electron density in the S_1_ state, indicating its locally excited (LE) nature. However, clear CT character is observed in the S_1_ state NTOs of D-RC-A, *i.e.*, hole and electron density are asymmetrically distributed on D and A sides, respectively. A different S_1_ character (CT or LE) of D-RC-A and A-RC-A can also affect the corresponding S_1_ relaxation. The initially populated S_1_ state undergoes rapid decay leading to the relaxed CT state, which is observed in the fs-TA signal of D-RC-A and absent for A-RC-A. Furthermore, NTO analysis on the triplet states indicates that the T_1_ of D-RC-A is slightly mixed with CT character like the S_1_ state, while the corresponding T_2_ state and T_1_/T_2_ states of A-RC-A are all LE dominated.

On the other hand, NTO analysis indicates that the central Rh(iii) in D-RC-A and A-RC-A is barely involved in the S_0_ → S_1_, S_0_ → T_1_, and S_0_ → T_2_ transitions, which is consistent with previous reports on electronic transitions of RCPDs,^28,31^*i.e.*, the low-lying S_1_, T_1_, and T_2_ states of RCPDs are dominated by π–π* transitions of ligands with minimal contributions of Rh(iii). The photophysics indicates the weak SOC of RCPDs, which is consistent with previous reports.^25–28,30,31^ Without pronounced metal–ligand interaction, ISC dynamics of RCPDs are largely determined by energetic parameters (*Γ* and Δ*E*_ST_) and can thus be discussed in the framework of semi-classical Marcus theory.

### Excitation-wavelength-dependent fs-TA

**Fig. 2:**
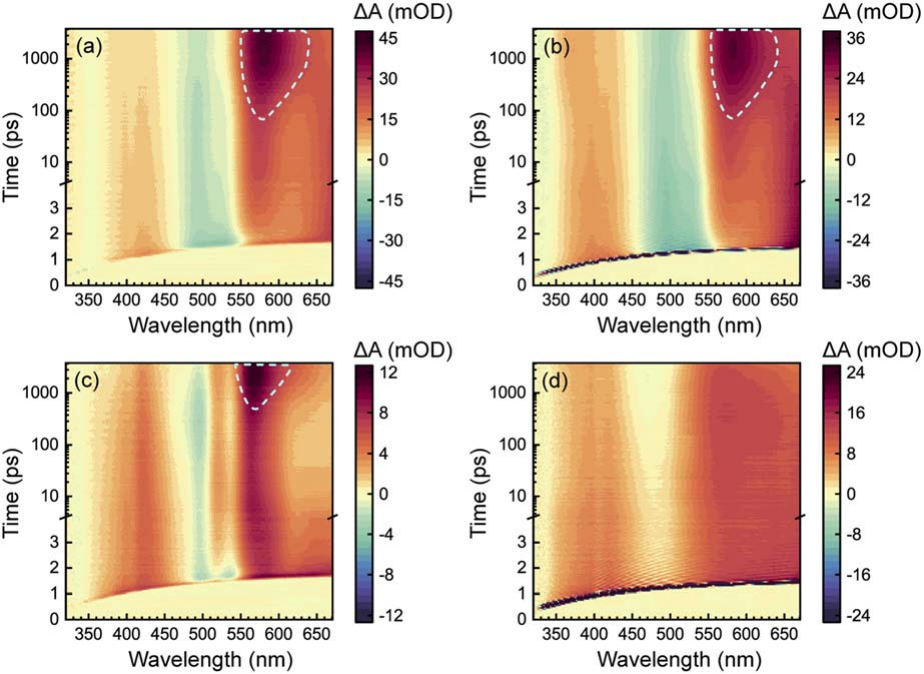
Spectro-temporal maps of *λ*_ex_-dependent fs-TA signals of D-RC-A upon UV (a) and visible (b) excitation as well as of A-RC-A upon identical UV (c) and visible (d) excitation. The spectral signatures of ISC-generated triplet states are highlighted by white dashed lines.

To acquire a full picture of electronic relaxation of RCPDs, we performed fs-TA experiments on D-RC-A and A-RC-A upon excitation by either UV (*λ*_ex_ = 295 nm) or visible (*λ*_ex_ = 513 nm) laser pulses as previously reported.^50^ The resulting TA signal in the probe range of *λ*_pr_ = 320–670 nm was recorded for delay times up to Δ*t* = 3.8 ns and are displayed in Fig. 2.

As shown in Fig. 2a and b, D-RC-A exhibits nearly identical fs-TA signals upon UV (*λ*_ex_ = 295 nm) and visible (*λ*_ex_ = 513 nm) excitation, respectively, in which at least three distinct bands can be distinguished. The negative band appears immediately after excitation in the *λ*_pr_ = 470–550 nm regime and agrees with the static absorption spectrum (S_0_ → S_1_), which can be attributed to the ground-state bleaching (GSB) signal. At the same time, two excited-state absorption (ESA) bands of the S_1_ state appear in the *λ*_pr_ = 360–460 nm and >550 nm regimes which, subsequently, exhibit relaxation simultaneously with the decay of the GSB band. Meanwhile, slow formation of a new positive band is observed with a center at *λ*_pr_ = 580 nm, which is superimposed on the decay of the long-lived ESA band in the *λ*_pr_ > 550 nm region, probably corresponding to ESA of an ISC-generated triplet state. However, the final destination (T_1_ or T_2_) of the observed ISC process is difficult to identify by fs-TA data itself. Instead, ISC rates of S_1_ → T_1_ and S_1_ → T_2_ need to be calculated to assign the observed ISC process. Thus, we tentatively denote the triplet state generated as the T_*n*_ (*n* ≥ 1) state.

Unlike D-RC-A, A-RC-A exhibits *λ*_ex_-dependent relaxation behavior. Upon UV excitation, a similar fs-TA signal as that for D-RC-A was recorded for A-RC-A, although the GSB displays a double peak at *λ*_pr_ = 500 nm and 535 nm, which is consistent with the pronounced vibronic progression exhibited in the static absorption spectrum. Meanwhile, triplet-state formation is also observed at *λ*_pr_ = 570 nm, with a substantially slower *k*_ISC_ than that of D-RC-A. As shown in Fig. S2,† preliminary fitting of triplet formation dynamics of D-RC-A (at *λ*_pr_ = 580 nm) and A-RC-A (at *λ*_pr_ = 570 nm) indicated that the observed ISC of D-RC-A (*τ*_ISC_ < 350 ps) is one order of magnitude faster than ISC of A-RC-A (*τ*_ISC_ > 3.5 ns). Thus, S_1_ state relaxation of D-RC-A might be dominated by ISC as it is much faster than radiative and non-radiative S_1_ → S_0_ channels. In contrast, considering the observed fluorescence lifetime (∼1.7 ns), the radiative and non-radiative S_1_ → S_0_ decay of A-RC-A might be comparable with ISC to triplet states.

Intriguingly, upon visible excitation, the triplet band of A-RC-A is absent in the fs-TA data up to the maximum delay time (3.8 ns), while the decay of ESA bands and refilling of the GSB band are still observable. In general, upon UV excitation, rapid internal conversion (IC) from initially populated S_*m*_ states leads to a vibrationally hot S_1_ state. Compared with the S_1_ state directly populated by resonant visible excitation, excess vibrational energy of the S_1_ state might be helpful to overcome the ISC barrier.^51–54^ Therefore, we believe that the ISC barrier (*E*_a_) for A-RC-A should be higher than that for D-RC-A, which is also indicated by a slower rise of the ISC band of A-RC-A compared to that of D-RC-A upon UV excitation.

### Ultrafast electronic relaxation

**Fig. 3:**
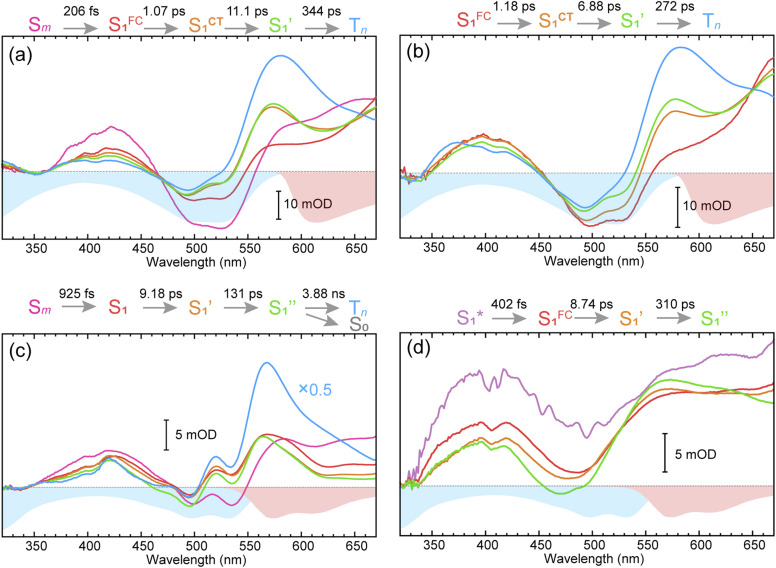
Target-analysis-extracted species-associated spectra (SAS) of *λ*_ex_-dependent fs-TA signals of D-RC-A upon 295 nm UV (a) and 513 nm visible (b) optical excitation, as well as SAS of A-RC-A upon identical UV (c) and visible (d) excitation. Four or five independent species were employed to reproduce the fs-TA signals.

To obtain details on the electronic relaxation of the RCPDs, we performed target analysis on the corresponding *λ*_ex_-dependent fs-TA data. On ultrafast time scales, a sequential kinetic model including several independent species was employed for all fs-TA data, while a branched scheme was considered for the decay of the relaxed (CT and structural) S_1_ state, corresponding to competition between relaxation to the S_0_ state (S_1_ → S_0_) and ISC to triplet states (S_1_ → T_*n*_). As discussed above, S_1_ relaxation of D-RC-A is dominated by the S_1_ → T_*n*_ channel, for which the S_1_ → S_0_ channel was correspondingly ignored in target analysis (Fig. S3†). However, for A-RC-A upon UV excitation, both the S_1_ → S_0_ and S_1_ → T_*n*_ channels were included in target analysis with a branch ratio of 0.5 (Fig. S3†). The extracted species-associated spectra (SAS) are displayed in Fig. 3, while the corresponding fitted time traces at selected *λ*_pr_ and the concentration evolution of each species can be found in Fig. S4 and S5,† respectively, in the ESI.†

As mentioned above, D-RC-A exhibits similar relaxation dynamics upon UV and visible excitation. Upon UV excitation, the populated S_*m*_ states undergo rapid IC with a time constant of *τ*_1_ = 206 fs to reach the Franck–Condon region of the S_1_ state (S_*m*_ → S_1_^FC^), which is absent in the fs-TA data upon resonant visible excitation to the S_1_ state. The subsequent SAS exhibits a spectral depletion in the range *λ*_pr_ = 570–670 nm, which is consistent with fluorescence spectra of D-RC-A and can be attributed to the stimulated emission (SE) band of the S_1_ state. Pronounced solvatochromism of D-RC-A has been reported,^31^ implying CT character of the fluorescent bright state, which is also consistent with the NTO analysis mentioned above. Considering the observed SE signature in the SAS, we assigned the second SAS to the CT-relaxed S_1_ state (noted as S_1_^CT^), corresponding to a local minimum on the S_1_ potential energy surface (PES). Moreover, the fitted formation time of S_1_^CT^ (S_1_^FC^ → S_1_^CT^, ∼1 ps) is consistent with the reported formation time of the solvation-stabilized CT state of organic D–A-type chromophores in THF.^55,56^

During the subsequent relaxation (
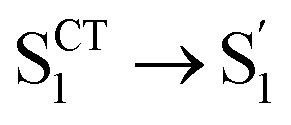
) with a ∼10 ps time constant, the SAS exhibit only minimal changes of spectral features, which might be explained by structural relaxation in the S_1_ state. In order to obtain further details on S_1_ relaxation, we calculated the optimized structures of both D-RC-A and A-RC-A in their S_1_, T_1_, and T_2_ states to be compared with the S_0_ structure. As shown in Fig. S6 and S7,† large S_0_ → S_1_ twisting is observed for the peripheral phenyl and D/A groups of RCPDs, which can be quantified by the calculated twisting angles listed in Table S3.† Apart from the twisting angles, contributions of S_0_ → S_1_ conformational relaxation can be generalized in reorganization energy (*Γ*^S_1_→S_0_^).^57,58^ By summing over contributions from each vibrational mode (Fig. S8†), *Γ*^S_1_→S_0_^ of D-RC-A and A-RC-A were calculated to be 1519 cm^−1^ and 1944 cm^−1^, respectively. However, *Γ*^S_1_→S_0_^ contributed by vibrational modes is not able to provide the relative contribution of each structural part of the RCPDs to conformational relaxation. Therefore, we further calculated the root-mean-square displacement (RMSD) in Cartesian coordinates of the optimized S_0_ (*x*_*i*_, *y*_*i*_, *z*_*i*_) and S_1_ (
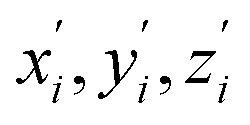
) state structures,4
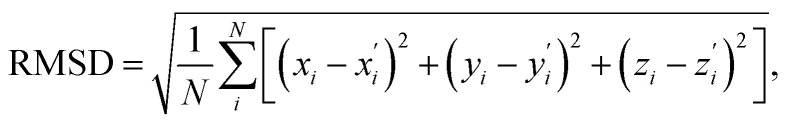


Summing over all atoms, *i* = 1, …, *N*. The calculation leads to RMSD^S_1_/S_0_^ = 0.156 Å for D-RC-A which is lower than that for A-RC-A (0.207 Å), consistent with the calculated *Γ*^S_1_→S_0_^. We further calculated the relative contributions of the peripheral groups (phenyls, C≡C, and D/A, gray part in Scheme 1) and Rh(iii)–ligand core (black part in Scheme 1). For D-RC-A, the RMSD^S_1_/S_0_^ was found to be dominated by twisting of the peripheral parts of the structure with ∼90% contribution. The rotational twisting around C–C single bonds has been widely reported to be nearly barrierless,^59,60^ which is consistent with the observed efficient relaxation (
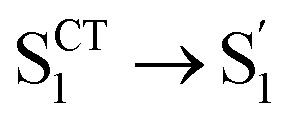
, ∼10 ps). Intriguingly, we found that the Rh(iii)-ligand core contributes ∼40% to the RMSD^S_1_/S_0_^ of A-RC-A *via* its own framework distortion, which is comparable with the twisting of the peripheral parts (∼60%). Compared to the rapid twisting of the peripheral groups, the distortion of the molecular framework is normally much slower due to a high potential barrier. Therefore, a two-step conformational relaxation is expected to be observed in the fs-TA data of A-RC-A, which has been widely reported in organic fluorescent systems.^61–63^

**Fig. 4:**
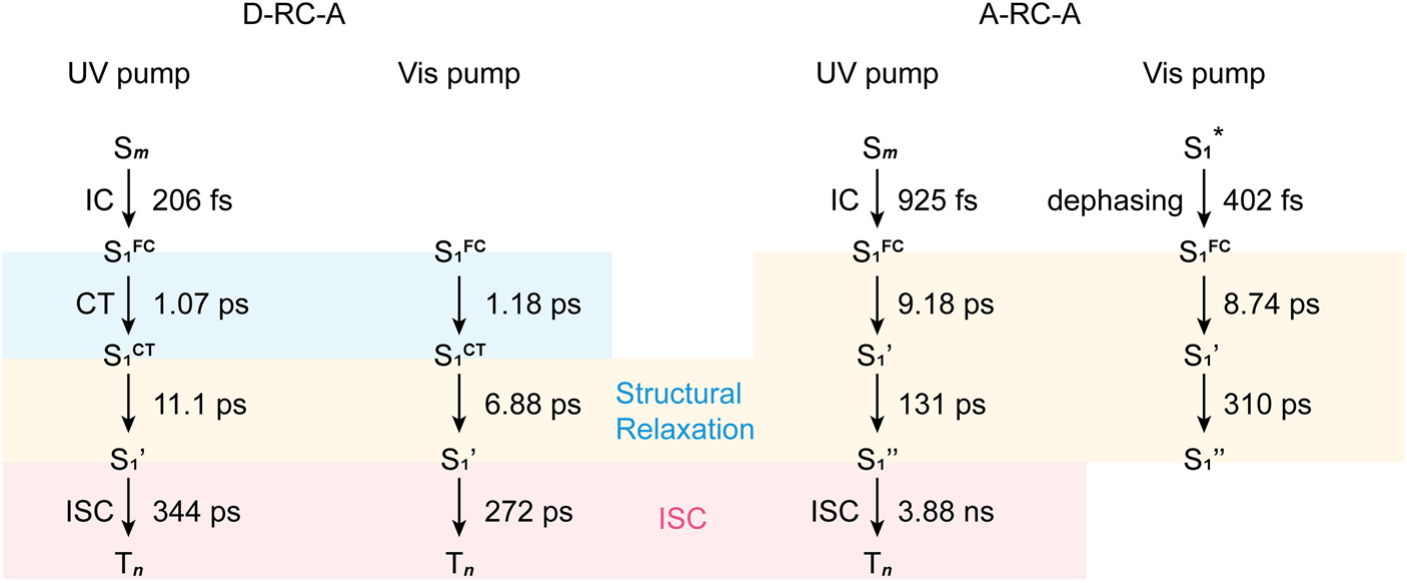
Electronic relaxation of D-RC-A (left two panels) and A-RC-A (right two panels) revealed by *λ*_ex_-dependent fs-TA measurements.

The extracted species 
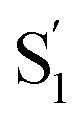
 corresponds to a global minimum on the S_1_-state PES, which subsequently undergoes ISC leading to formation of a triplet state, featured by the rise of intense absorption centered at *λ*_pr_ = 580 nm and the disappearance of SE depletion (*λ*_pr_ = 570–670 nm). The formation time (
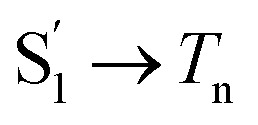
, ∼300 ps) of the triplet state corresponds to the ISC rate constant (*k*_ISC_) of D-RC-A, which is consistent with the relatively low fluorescence quantum yield (*Φ*_f_ = 0.22) and sub-nanosecond fluorescence lifetime (*τ*_S_1__ = 0.8 ns) observed in toluene solution.^31^ Note that relaxation of the S_1_ state is contributed to by non-radiative decay to S_0_ (*k*_NR_^S^), radiative decay to S_0_ (*k*_R_^S^), and ISC to triplet states (*k*_ISC_). Therefore, the S_1_ ESA decay and GSB refilling dynamics could be largely different with formation of the triplet band, *i.e.*, S_1_ ESA and GSB include contributions of *k*_NR_^S^, *k*_R_^S^, and *k*_ISC_, while rising of the triplet band is associated with *k*_ISC_ only. The full picture of the electronic relaxation of the RCPDs is summarized in Fig. 4.

In the fs-TA data for A-RC-A upon UV excitation, we observed electronic relaxation that is significantly different from D-RC-A. Without a D–A structure, the CT state signature was absent in the fs-TA of A-RC-A. After excitation, rapid IC (∼900 fs, S_*m*_ → S_1_^FC^) is followed by two-step conformational relaxation (
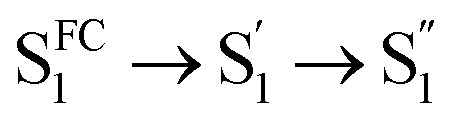
), corresponding to a comparable contribution of the peripheral structure and metal–ligand core to the RMSD^S_1_/S_0_^ of A-RC-A. The first step (
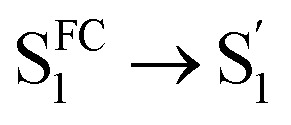
, ∼9 ps) exhibits a time constant similar to that of the corresponding step in D-RC-A (
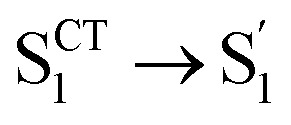
, ∼10 ps), which was attributed to barrierless twisting of the peripheral structure. Note that the observed twisting with a ∼10 ps time constant is unobservable in our previous time-resolved IR investigation due to the limitation of low temporal resolution (10–20 ps).^30^ The subsequent step (
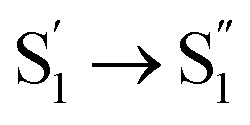
, ∼150 ps) accordingly corresponds to structural distortion of the Rh(iii)–ligand framework. Furthermore, slower triplet formation (S_1_′′ → T_*n*_, ∼3.88 ns) in A-RC-A than in D-RC-A was observed upon UV excitation, corresponding to a long-lived (*τ*_f_ = 1.7 ns) S_1_ state,^31^ which is discussed in detail below. Upon ∼25 fs visible excitation, A-RC-A exhibits largely different electronic relaxation (Fig. 3d) from the case with UV excitation (Fig. 3c). The target analysis extracted a short-lived species(noted as 
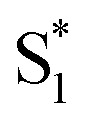
) after excitation with a ∼400 fs time constant. As described above, rapid IC (∼900 fs, S_*m*_ → S_1_^FC^) was identified for A-RC-A upon UV excitation, while the observed visible-excitation-induced ∼400 fs relaxation obviously cannot be assigned to IC from S_*m*_, as the visible excitation at *λ*_ex_ = 513 nm employed is resonant with the S_1_ state of A-RC-A and, correspondingly, incapable of populating the S_*m*_ state. Moreover, as illustrated in Fig. S9,† we observed a beating behavior in the *λ*_pr_ = 400–550 nm wavelength regime of the fs-TA data, which is observed as an oscillatory modulation in both temporal (Fig. S4†) and frequency (Fig. S9†) domains. The beating-modulated TA spectra (Fig. S9†) observed in the initial few picoseconds are highly consistent with the extracted SAS of the 
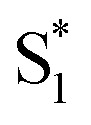
 species with a ∼400 fs relaxation. We therefore attribute 
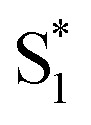
 relaxation to dephasing of the coherent vibrational wave packet (noted as 
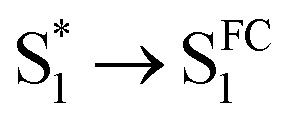
). The dephasing of 
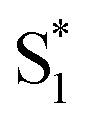
 was not recognized as an independent species in the target analysis of D-RC-A upon visible excitation, which might be associated with a less modulated spectral shape and faster 
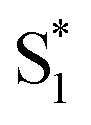
 dephasing (Fig. S9†) of D-RC-A due to intense solute–solvent interactions. We further extracted beating signals by subtracting the fitted exponential components from the measured fs-TA data. The Fourier transformed power spectra of the beating signals (Fig. S10†) revealed a dominant vibrational mode at ∼250 cm^−1^ for both D-RC-A and A-RC-A. Considering that the beating frequency is independent of the 2,5-substitution, we speculate that the observed ∼250 cm^−1^ mode might originate from a distortion of the metal–ligand core.

After rapid dephasing (
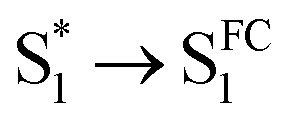
), the S_1_ state of A-RC-A subsequently undergoes a two-step conformational relaxation (
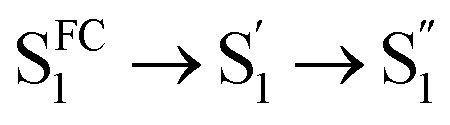
) nearly identical with that of A-RC-A upon UV excitation. However, the rise of the T_*n*_ band observed in the fs-TA of A-RC-A upon UV excitation is absent upon visible excitation. Instead, we observe an isosbestic point at *λ*_pr_ = 530 nm in the fs-TA data of A-RC-A upon visible excitation, indicating that the TA bands located on the blue (GSB, *λ*_pr_ = 430–530 nm) and red (ESA, *λ*_pr_ = 530–670 nm) sides of the isosbestic point belong to an identical process, *i.e.*, S_1_ relaxation. However, note that the fs-TA observation cannot fully exclude the existence of ISC of A-RC-A upon visible excitation due to the limitation of the fs-TA time window. Actually, upon visible excitation, slight spectral rising can be observed between 550 nm and 600 nm at long delays, which is consistent with the triplet band observed upon UV excitation. A plausible explanation is that visible excitation leads to an ultraslow ISC which is unobservable within our time window (3.8 ns).

By measuring the fluorescence quantum yield of A-RC-A upon UV (*Φ*_f_^UV^ = 0.29) and visible (*Φ*_f_^vis^ = 0.33) excitation in THF (Table S4†) and assuming an unchanged radiative decay rate constant (*k*_r_) upon different excitation, the ISC time constant upon visible excitation was estimated to be ∼5.75 ns (ESI,† Section S4), which is accordingly unobservable in the fs-TA with time window of 3.8 ns. We also measured the fluorescence lifetime of A-RC-A upon UV (*τ*_S_1__^UV^) and visible (*τ*_S_1__^vis^) excitation, which leads to an unchanged lifetime of ∼1.6 ns (Fig. S11–S13†). However, quantitative estimation of the S_1_ state decay (ESI,† Section S4) indicated that *τ*_S_1__^vis^ can be only <0.2 ns slower than *τ*_S_1__^UV^, which can be easily concealed by different temporal profiles of excitation sources in the UV and visible regimes. Therefore, we believe an ultraslow ISC that is beyond our fs-TA time window leads to the absence of triplet ESA in the fs-TA of A-RC-A upon visible excitation, which corresponds to an inaccessible barrier for ISC. In the next section, we further analyze the ISC energetic diagram of D-RC-A and A-RC-A in the framework of Marcus theory to understand the fundamental mechanism that leads to the different ISC dynamics of D-RC-A and A-RC-A.

### Marcus analysis of the ISC dynamics

As discussed above, upon identical excitation conditions, D-RC-A exhibited much faster ISC than A-RC-A, which is consistent with the reported lower *Φ*_f_ and shorter *τ*_S_1__ of D-RC-A compared to A-RC-A.^31^ For systems with weak SOC, *i.e.*, 〈S_*m*_|*Ĥ*_SO_|T_*n*_〉 ≪ *E*_a_, ISC can be simplified as a transition between non-adiabatic states and described by semi-classical Marcus theory.^64–66^ As the basis for our Marcus analysis, adiabatic excitation energies of the S_1_ and T_2_ states were calculated with TD-DFT for both D-RC-A and A-RC-A, while the corresponding T_1_ states were calculated using unrestricted DFT (UDFT). UDFT has been reported to be reliable for calculating T_1_ states of organic and organometallic complexes, but is inherently not able to treat higher lying triplet states.^67–69^

Here, we first analyze the S_1_ → T_1_ channel that is regarded as the dominant ISC channel in common organic systems,^70,71^ for which the adiabatic excitation energies of the S_1_ and T_1_ states are required. The ISC barrier of the S_1_ → T_1_ channel (*E*_a_^S_1_→T_1_^) was estimated by a reorganization energy (*Γ*^S_1_→T_1_^) and corresponding adiabatic energy gap (Δ*E*_ST_^S_1_→T_1_^) within the framework of Marcus theory. As listed in Table 1, Δ*E*_ST_^S_1_→T_1_^ of RCPDs are comparable (0.829 eV for D-RC-A and 0.858 eV for A-RC-A) whereas D-RC-A features a higher *Γ*^S_1_→T_1_^ (0.115 eV) than A-RC-A (0.062 eV), leading to *E*_a_^S_1_→T_1_^ for D-RC-A (1.103 eV) and A-RC-A (2.574 eV), which are comparable with the adiabatic energies of the corresponding T_1_ states. Meanwhile, the SOC matrix elements 〈S_1_|*Ĥ*_SO_|T_1_〉 of D-RC-A and A-RC-A were calculated to be 4.045 cm^−1^ and 0.425 cm^−1^, respectively. The large ISC barrier (>1 eV) leads to virtually forbidden transitions of the S_1_ → T_1_ channel of D-RC-A and A-RC-A.

**Table 1:** Summary of the calculated and experimentally measured photophysical parameters associated with plausible ISC channels of D-RC-A and A-RC-A

	D-RC-A	A-RC-A
S_1_ excitation energy^a^/eV	1.952	1.939
T_1_ excitation energy^b^/eV	1.123	1.081
T_2_ excitation energy^a^/eV	2.022	2.213
RMSD^S_1_/S_0_^/Å	0.156	0.207
RMSD^S_1_/T_1_^/Å	0.377	0.302
RMSD^S_1_/T_2_^/Å	0.853	0.325
*Γ* ^S_1_→S_0_^/eV	0.188	0.241
*Γ* ^S_1_→T_1_^/eV	0.115	0.062
*Γ* ^T_2_→S_1_^/eV	0.558	0.257
cΔ*E*_ST_^S_1_→T_1_^/eV	0.829	0.858
cΔ*E*_ST_^S_1_→T_2_^/eV	−0.070	−0.274
cΔ*E*_TS_^T_2_→S_1_^/eV	0.070	0.274
〈S_1_|*Ĥ*_SO_|T_1_〉/cm^−1^	4.045	0.425
〈S_1_|*Ĥ*_SO_|T_2_〉/cm^−1^	4.481	8.697
d *E*_a_^S_1_→T_1_^/eV	1.103	2.574
d *E*_a_^T_2_→S_1_^/eV	0.106	<0.001
e *E*_a_^S_1_→T_2_^/eV	0.177	0.274
*E*_a_^S_1_→T_1_^/*E*_a_^S_1_→T_2_^	6.325	9.387
*k*_ISC_^S_1_→T_2_^ (cal. DA)/*k*_ISC_^S_1_→T_2_^ (cal. AA)	7.774	
*k*_ISC_ (exp.)/ps^−1^	1/350	1/3900
*k*_ISC_ (exp. DA)/*k*_ISC_ (exp. AA)	11.143	

a Adiabatic excitation energy calculated by TD-DFT.

b Adiabatic excitation energy calculated by UDFT.

c Calculated by adiabatic energies of optimized geometries of corresponding electronic states, the adiabatic energy gap of an exothermic process was defined to be positive.

d Estimated by reorganization energy and adiabatic energy gap of the corresponding transition by Marcus theory.

e Calculated by *E*_a_^S_1_→T_2_^ = *E*_a_^T_2_→S_1_^ + Δ*E*_TS_^T_2_→S_1_^.

**Fig. 5:**
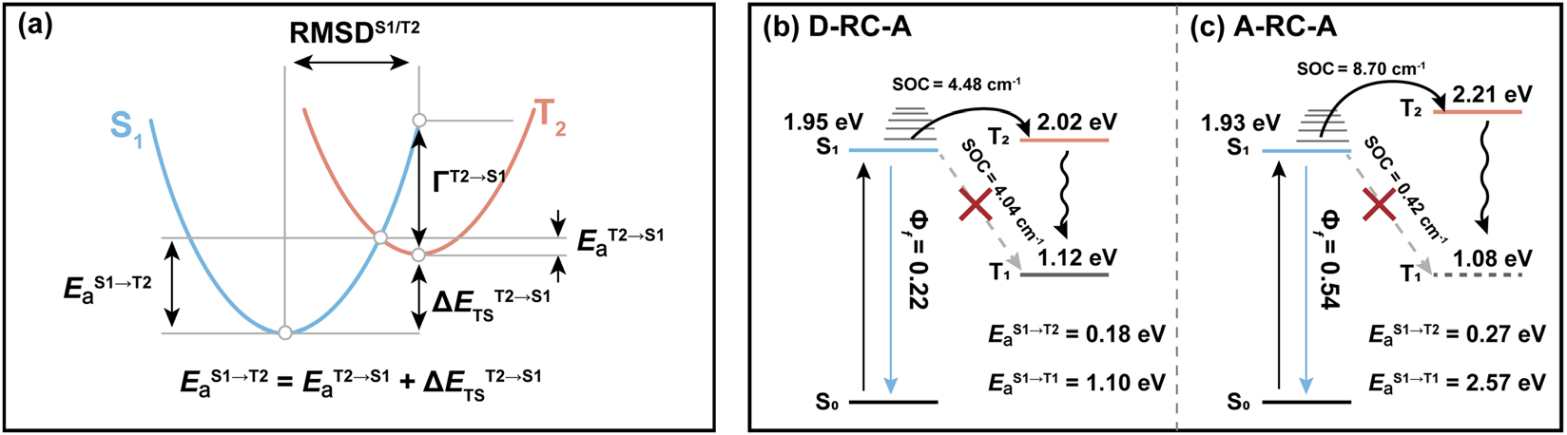
(a) Simplified energetic diagram for calculating the ISC barrier of the S_1_ → T_2_ transition in the framework of Marcus theory, and energy diagrams for plausible ISC channels of D-RC-A (b) and A-RC-A (c), in which both T_1_ and T_2_ states are considered.

Regarding the S_1_ → T_2_ channel, the calculated adiabatic energies of the S_1_ and T_2_ states indicate its endothermic nature with a negative Δ*E*_ST_^S_1_→T_2_^ for both D-RC-A (−0.070 eV) and A-RC-A (−0.274 eV). Nevertheless, the S_1_ → T_2_ transition of RCPDs can still be accessible through a thermally activated mechanism as long as *E*_a_^S_1_→T_2_^ is small enough (normally <0.2 eV), which has been observed *via* temperature-dependent experiments for RCPDs.^28^ To estimate *E*_a_^S_1_→T_2_^, as illustrated in Fig. 5a, we calculated the reorganization energy (*Γ*^T_2_→S_1_^) and adiabatic energy gap (Δ*E*_TS_^T_2_→S_1_^) of its reverse transition T_2_ → S_1_. As an exothermic process, *E*_a_^T_2_→S_1_^ can be estimated by Marcus theory, leading to *E*_a_^T_2_→S_1_^ = 0.106 eV and <0.001 eV for D-RC-A and A-RC-A, respectively. Thus, *E*_a_^S_1_→T_2_^ can be approximated by the sum of *E*_a_^T_2_→S_1_^ and the adiabatic energy gap (Δ*E*_TS_^T_2_→S_1_^), *i.e.*, 0.177 eV and 0.274 eV for D-RC-A (Fig. 5b) and A-RC-A (Fig. 5c), respectively.

With the calculated ISC barriers and SOC matrix elements of plausible ISC channels, we can discuss the ISC dynamics of D-RC-A (Fig. 5b) and A-RC-A (Fig. 5c) in detail. For both D-RC-A and A-RC-A, *E*_a_^S_1_→T_1_^ is substantially higher than *E*_a_^S_1_→T_2_^ (*E*_a_^S_1_→T_1_^/*E*_a_^S_1_→T_2_^ = 6.325 for D-RC-A and 9.387 for A-RC-A), leading to S_1_ → T_2_ as the dominant ISC channel of both D-RC-A and A-RC-A. Although S_1_ → T_2_ ISC is an endothermic process, the thermally accessible barriers (∼0.2 eV) lead to ISC dynamics at room temperature, which is consistent with our previously observed temperature-dependence.^28^ Furthermore, *k*_ISC_^S_1_→T_2_^ of D-RC-A were calculated to be ∼7.7 times faster than *k*_ISC_^S_1_→T_2_^ of A-RC-A, which is perfectly consistent with the ∼11 times faster ISC observed experimentally by fs-TA. The relatively fast ISC of D-RC-A also explains the lower *Φ*_f_ of D-RC-A than A-RC-A due to competition between radiative and non-radiative (including ISC) decay of the S_1_ state. Meanwhile, the calculated ISC barrier (*E*_a_^S_1_→T_2_^) of D-RC-A and A-RC-A also explains the *λ*_ex_-dependent fs-TA data of A-RC-A. For D-RC-A, the thermally accessible S_1_ → T_2_ channel at room temperature (*E*_a_^S_1_→T_2_^ = 0.177 eV) leads to the observed *λ*_ex_-independent ISC signature in the TA-data. However, *E*_a_^S_1_→T_2_^ of A-RC-A (0.274 eV) is ∼0.1 eV higher than that for the ISC channel of D-RC-A, leading to a hindered ISC (S_1_ → T_2_) *via* a thermally activated mechanism. As discussed above, triplet ESA was consequently unobservable in the fs-TA of A-RC-A upon visible excitation due to its slow formation (∼5.8 ns). However, upon UV excitation, initial IC (S_*m*_ → S_1_) decay may lead to more population being distributed in higher vibrational levels of the S_1_ state, leading to faster ISC that can be observed in the fs-TA data. Note that values of all ISC barriers listed in Table 1 were estimated by Marcus theory based on the TD-DFT-calculated energies of S_1_ and T_2_ states, and unrestricted DFT (UDFT)-calculated T_1_ state, which can have errors up to 0.3 eV. Thus, the S_1_ → T_2_ ISC barrier (*E*_a_^S_1_→T_2_^) can possibly be different between D-RC-A and A-RC-A, leading to different observations in the *λ*_ex_-dependent fs-TA data.

Last but not least, note that although the T_2_ state is heavily involved in the ISC dynamics of D-RC-A and A-RC-A, we believe that the observed triplet signal at *λ*_pr_ = 570–580 nm in the fs-TA data must be assigned to the T_1_ rather than the T_2_ state. Spin-allowed T_2_ → T_1_ transitions with ∼1 eV energy gaps are expected to be much faster than the corresponding S_1_ → T_2_ transitions, leading to limited accumulation of T_2_ species. Thus, the observed ESA bands should be assigned to the T_1_ states formed through rapid decay from the T_2_ state. Similarly, the ISC populated T_2_ states are energetically higher than the corresponding S_1_ states of D-RC-A and A-RC-A, leading to potential exothermic reverse ISC (RISC, T_2_ → S_1_). However, such an RISC process cannot be the dominant decay channel of the T_2_ state due to competition of spin-allowed internal conversion (T_2_ → T_1_).

### Singlet oxygen sensitization

Having the above fs-TA and theoretical data in hand, we then examined the ability of photoexcited A-RC-A to sensitize singlet molecular oxygen, O_2_(a^1^Δ_g_), and the associated kinetics for two reasons: (1) this would likely provide additional information on the formation of triplet states upon light irradiation, and (2) a compound which is both fluorescent and can generate O_2_(a^1^Δ_g_) from its triplet state could be potentially useful for both bio-imaging and photodynamic therapy.^72,73^ Moreover, using the fluorescence, one has the advantage of being able to insure that the sensitizing compound was localized at the desired location in a heterogeneous biological system.^74^ We note that in our previous studies,^28,30^ we found that several related complexes are capable of simultaneous fluorescence and O_2_(a^1^Δ_g_) sensitization, with reasonable quantum yields for both processes, often summing to *ca.* 1.

Quantum yields of O_2_(a^1^Δ_g_) production, *Φ*_Δ_, were obtained by monitoring the intensity of the 1275 nm O_2_(a^1^Δ_g_) → O_2_(X^3^Σ_g_^−^) phosphorescence in time-resolved experiments, as described previously.^75,76^ Experiments were performed upon irradiation of A-RC-A at 417 nm. The integrated intensity of the time-resolved O_2_(a^1^Δ_g_) phosphorescence signal, measured as a function of incident laser power, was normalized by the sensitizer absorbance and the O_2_(a^1^Δ_g_) lifetime. Although a bleaching experiment indicates that A-RC-A can react with O_2_(a^1^Δ_g_) (Fig. S14†), all data were recorded under conditions where A-RC-A bleaching was negligible.

We first performed experiments in toluene-h_8_, using a solution that had been bubbled with a gas stream containing 2% oxygen and 98% N_2_ (the reasons for using low concentrations of oxygen will become apparent below when discussing the kinetics of the time-resolved O_2_(a^1^Δ_g_) phosphorescence signal). In this case, we performed one set of experiments using 1*H*-phenalenone (PN) in benzene as the reference O_2_(a^1^Δ_g_) photosensitizer (*Φ*_Δ_ = 0.92 ± 0.03),^77,78^ and another using *m*-tetraphenylporphyrin (TPP) in benzene as the reference photosensitizer (*Φ*_Δ_ = 0.66 ± 0.08).^79^ The results yield a value of *Φ*_Δ_ for A-RC-A of 0.09 ± 0.01. The pertinent data are shown in Fig. S15 and S16.†

**Fig. 6:**
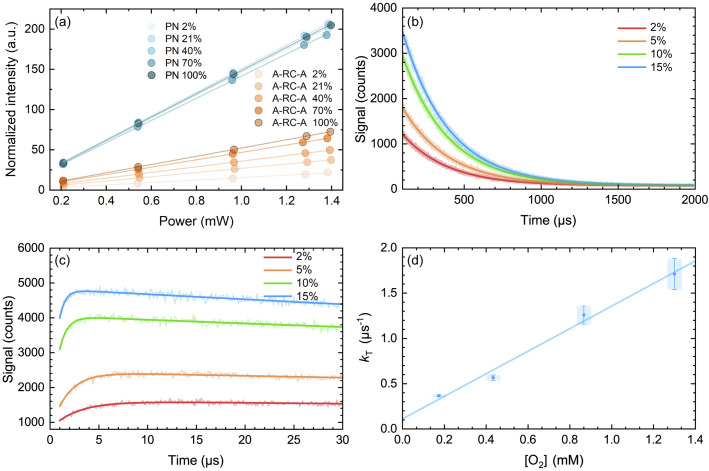
(a) Integrated intensity of the O_2_(a^1^Δ_g_) phosphorescence signal upon irradiation at 417 nm, normalized by the sensitizer absorbance and the O_2_(a^1^Δ_g_) lifetime, plotted as a function of laser power and collected over a range of O_2_ concentrations for both A-RC-A and for the reference standard, phenalenone (PN), in toluene-d_8_. The O_2_ concentration is represented as the percent of O_2_ in the O_2_/N_2_ gas mixture bubbled through the solution. The slopes of the linear fits are proportional to the O_2_(a^1^Δ_g_) quantum yield. Representative time-resolved O_2_(a^1^Δ_g_) phosphorescence traces used to obtain the A-RC-A data are shown in (b and c). (b) The data from 100 μs to 2000 μs were fitted by a single exponential decay function (solid lines) to obtain the lifetime of O_2_(a^1^Δ_g_) (*i.e.*, 1/*k*_Δ_). (c) Using *k*_Δ_ as a fixed parameter, eqn (5) was used as a fitting function (solid lines) to obtain values of *k*_T_ for a time domain where O_2_(a^1^Δ_g_) was formed in the photosensitized reaction. (d) Plot of *k*_T_, obtained from the fits shown in (c) for the A-RC-A data, against the concentration of dissolved oxygen. The slope, (1.3 ± 0.1) × 10^9^ M^−1^ s^−1^, is the bimolecular rate constant for oxygen quenching of the O_2_(a^1^Δ_g_) precursor. The intercept yields a value of ∼9 μs for the lifetime of this O_2_(a^1^Δ_g_) precursor in the absence of oxygen. Both of these numbers are consistent with expectation for oxygen quenching of a triplet state.^74,82^

Singlet oxygen phosphorescence data recorded upon irradiation of A-RC-A show an increase in the value of *Φ*_Δ_ with increasing oxygen concentration (Table 2). In contrast, corresponding data from the PN-sensitized production of O_2_(a^1^Δ_g_) do not show an increase in *Φ*_Δ_ with an increase in the oxygen concentration (Fig. 6a). These data indicate that, in the PN experiment, the lifetime of the O_2_(a^1^Δ_g_) precursor (*i.e.*, the PN triplet state) is sufficiently long that the entire ^3^PN population is effectively quenched at a low oxygen concentration, which is consistent with expectation. For the A-RC-A experiment, the data indicate that the O_2_(a^1^Δ_g_) precursor, presumably the A-RC-A triplet state, is sufficiently short-lived that higher oxygen concentrations are needed to quench a larger fraction of the ^3^A-RC-A population.

**Table 2:** O_2_(a^1^Δ_g_) quantum yields, *Φ*_Δ_, for A-RC-A^a^

Gas	*Φ*_Δ_
2% O_2_	0.10
21% O_2_	0.18
40% O_2_	0.23
70% O_2_	0.32
100% O_2_	0.34

a Errors on *Φ*_Δ_ are ± 10%. Data for the pertinent experiments are shown in Fig. 6.

Given that the lifetime of the S_1_ state of A-RC-A in oxygen-free toluene is 1.7 ns, the S_1_ state is so short-lived that it cannot be quenched by oxygen, even in an oxygen-saturated solution at atmospheric pressure. This can be confirmed in a calculation where one accepts that the quenching will occur at the diffusion controlled limit (*i.e.*, *k*_diff_ = 3 × 10^10^ M^−1^ s^−1^). As such, we infer that the O_2_(a^1^Δ_g_) precursor will be the A-RC-A triplet state (*vide infra*). We then chose to obtain information about the kinetics of the O_2_(a^1^Δ_g_) precursor, again presumably the A-RC-A triplet state, using our time-resolved O_2_(a^1^Δ_g_) phosphorescence data, as described below.

For the triplet-state-photosensitized production of O_2_(a^1^Δ_g_) in homogeneous solutions, the intensity, *I*(*t*), of the time-resolved 1275 nm O_2_(a^1^Δ_g_) → O_2_(X^3^Σ_g_^−^) phosphorescence signal is generally modeled using a fitting function based on eqn (5)^74,75,81^5
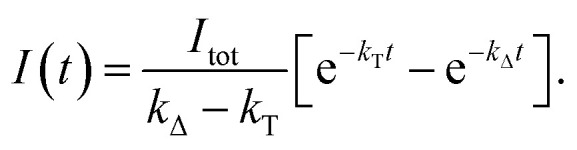
In eqn (5), *I*_tot_ is proportional to the total integrated intensity of O_2_(a^1^Δ_g_) phosphorescence, *k*_T_ is the decay rate constant for the O_2_(a^1^Δ_g_) precursor (*i.e.*, the reciprocal lifetime of the sensitizer triplet state) and *k*_Δ_ the rate constant for O_2_(a^1^Δ_g_) decay (*i.e.*, the reciprocal O_2_(a^1^Δ_g_) lifetime). By performing the O_2_(a^1^Δ_g_) phosphorescence experiment in toluene-d_8_ as opposed to toluene-h_8_, we take advantage of the large H/D solvent isotope effect on the O_2_(a^1^Δ_g_) lifetime.^74^ In this way, we can independently characterize *k*_Δ_ in a time domain where *k*_T_ does not appreciably influence the signal (Fig. 6b). Using this value of *k*_Δ_ as a fixed parameter, we can then re-fit the O_2_(a^1^Δ_g_) phosphorescence data in the time domains where *k*_T_ influences the signal (Fig. 6c). We applied this procedure for experiments performed using a series of comparatively low oxygen concentrations to assess more accurately the decay rate constant of the O_2_(a^1^Δ_g_) precursor.

In Fig. 6d, we plot the values of *k*_T_ thus obtained for A-RC-A against the oxygen concentration to yield the bimolecular rate constant for oxygen quenching of the O_2_(a^1^Δ_g_) precursor. We determined oxygen concentrations using the mole fraction of oxygen in the bubbling gas (controlled by O_2_ and N_2_ flow meters) and Henry's Law constants published by Battino, *et al.*^82^ The value obtained for this quenching rate constant is (1.3 ± 0.1) × 10^9^ M^−1^ s^−1^. As an independent control for this study on A-RC-A, we performed the same experiment using PN as the O_2_(a^1^Δ_g_) sensitizer (Fig. S17†), and the results obtained provide credence for our results on A-RC-A. Specifically, our data for the quenching of ^3^PN by oxygen yield a rate constant of (2.2 ± 0.2) × 10^9^ M^−1^ s^−1^, which is consistent with published data.^78^

The data obtained from Fig. 6d are consistent with the following assignment: the O_2_(a^1^Δ_g_) precursor upon irradiation of A-RC-A is a triplet state whose lifetime in the absence of oxygen is ∼9 μs.

From Fig. 6b, the values of the O_2_(a^1^Δ_g_) lifetime obtained (*i.e.*, *k*_Δ_^−1^ = 296 ± 3 μs) are shorter than what is expected for the solvent-mediated deactivation of O_2_(a^1^Δ_g_ ) in toluene-d_8_. For the latter, we independently recorded a value of 326 ± 3 μs using PN as the sensitizer with the same batch of toluene-d_8_ used for the A-RC-A experiments. Using eqn (6), and with the A-RC-A concentration of 1.7 × 10^−5^ M used in our experiments, we obtain a rate constant of ∼1.8 × 10^7^ M^−1^ s^−1^ for the deactivation/removal of O_2_(a^1^Δ_g_) by A-RC-A. Given the magnitude of this rate constant, it is likely that the mechanism for A-RC-A-mediated deactivation of O_2_(a^1^Δ_g_) involves some charge transfer from A-RC-A to O_2_(a^1^Δ_g_).^74,80^ Moreover, based on an A-RC-A bleaching experiment (Fig. S14†), a small component of this rate constant reflects a chemical reaction between A-RC-A and O_2_(a^1^Δ_g_).

6 *k*^obs.with A-RC-A^_Δ_ = *k*^obs.without A-RC-A^_Δ_ + *k*_q_[A-RC-A]

We also considered the interesting possibility that the O_2_(a^1^Δ_g_) precursor might possibly be the T_2_ state. However, our data indicate that the O_2_(a^1^Δ_g_) precursor upon irradiation of A-RC-A has quite a long lifetime (∼9 μs obtained from the intercept of the plot in Fig. 6d) and, as such, it must be the T_1_ state, as spin-allowed internal conversion from T_2_ to T_1_ is expected to be quite rapid (*vide supra*).

## Conclusions

We investigated the ultrafast photophysics and intersystem crossing (ISC) dynamics of two *para*-substituted 2,5-bis(phenylethynyl) RCPDs (D-RC-A and A-RC-A) using excitation-wavelength-dependent fs-TA measurements and TD-DFT calculations. The electronic relaxation channels of the S_1_ state were revealed in detail, including charge transfer, conformational relaxation, vibrational dephasing, and ISC. By calculating the thermodynamic barrier and spin–orbit coupling, plausible ISC channels were revealed using semi-classical Marcus theory, *i.e.*, ISC is dominated by the thermally activated S_1_ → T_2_ channel. With weak metal–ligand interactions, the plausible ISC channels of RCPDs are largely affected by ISC barriers. Studies of the sensitization of O_2_(a^1^Δ_g_) by A-RC-A give quantum yields *Φ*_Δ_ of up to ∼0.3 at high oxygen concentrations, consistent with the rate of ISC being competitive with that of fluorescence. The relatively long lifetime of the triplet state responsible for sensitization of O_2_(a^1^Δ_g_) of ∼9 μs indicates that it must be T_1_, being formed rapidly from T_2_. Our work, especially the paradigm on ISC dynamics, might provide useful insight into the behavior of other fluorescent emitters.

## Author contributions

Zilong Guo: conceptualization, methodology, investigation, data curation, formal analysis, visualization and writing original draft; Yaxin Wang: data curation, formal analysis and validation; Julia Heitmüller: data curation and formal analysis; Carolin Sieck: investigation and data curation; Andreas Prüfer: investigation and data curation; Philipp Ralle: investigation and data curation; Andreas Steffen: conceptualization, methodology, investigation, writing – review and editing; Petr Henke: methodology, data curation, formal analysis and validation; Peter R. Ogilby: methodology, supervision, and writing – review and editing; Todd B. Marder: conceptualization, formal analysis, funding acquisition, supervision, and writing – review and editing; Xiaonan Ma: conceptualization, formal analysis, funding acquisition, supervision, and writing – review and editing; Tobias Brixner: conceptualization, formal analysis, funding acquisition, supervision, and writing – review and editing.

## Conflicts of interest

There are no conflicts to declare.

## Supplementary Material

SC-015-D4SC04306E-s001

SC-015-D4SC04306E-s002

## Data Availability

The data supporting this article have been included as part of the ESI.†
