# Supplementary material for: Ultrafast photophysics of para-substituted 2,5-bis(arylethynyl) rhodacyclopentadienes: thermally activated intersystem crossing
Source: Chem Sci. 2024 Aug 15;15(36):14746–56. doi: 10.1039/d4sc04306e (PMC11337014; doi:10.1039/d4sc04306e)
Supplement: SC-015-D4SC04306E-s001 [file SC-015-D4SC04306E-s001.pdf]

Supplementary Information (SI) for Chemical Science.
This journal is © The Royal Society of Chemistry 2024

Supporting Information

Ultrafast Photophysics of *para*-Substituted 2,5-Bis(arylethynyl) Rhodacyclopentadienes: Thermally Activated Intersystem Crossing

Zilong Guo,[b] Yaxin Wang,[b] Julia Heitmüller,[a] Carolin Sieck,[c] Andreas Prüfer,[d] Philipp Ralle,[d] Andreas Steffen,[d] Petr Henke,[e,g] Peter R. Ogilby,*[e] Todd B. Marder,*[c,f] Xiaonan Ma,*[b,a] and Tobias Brixner*[a,f]

Contents

1. Experimental Section S3

2. Results and Discussion S5

S1. Photophysical properties and electronic transitions S5

S2. ISC dynamics S8

S3. Target analysis of TA data S9

S4. Estimation of the ISC kinetics of A-RC-A under VIS excitation S22

S5. Studies of $^1O_2$ sensitization by A-RC-A S23

S6. NMR Spectra and Purification of A-RC-A S27

3. References S31

Experimental Section

General information

The complexes D-RC-A and A-RC-A were synthesized according to a previous report.[1] For all measurements, samples were dissolved in spectroscopic-grade solvents purchased from Sigma-Aldrich and used as received. The static UV/visible absorption spectra were performed on a JACSO V-670 spectrometer or an Agilent Cary 60 spectrophotometer using standard 1 cm pathlength quartz cells. Excitation and emission spectra were recorded at right angles to the excitation source on an Edinburgh Instruments FLS1000 spectrometer, equipped with a 450 W Xenon arc lamp, double monochromators for the excitation and emission pathways, and a red-sensitive photomultiplier (PMT-980) as detector. The excitation and emission spectra were corrected using the standard corrections supplied by the manufacturer for the spectral power of the excitation source and the sensitivity of the detector. Photoluminescence quantum yields measurements were performed by using a Quantaurus C11347 integrated sphere (Hamamatsu, Japan) exciting the sample at λ_{ex} between 292 nm and 515 nm. The luminescence lifetime was measured using a FluoTime 300 spectrometer from PicoQuant equipped with a double-grating excitation monochromator, diode lasers (operating at 317 nm, 440 nm, 505 nm, pulse width < 80 ps) operated by a computer-controlled laser driver PDL-828 "Sepia II" (repetition rate up to 80 MHz, burst mode for slow and weak decays), two double-grating emission monochromators along with a PicoHarp 300 detector for TCSPC measurements (minimum base resolution 4 ps). The instrument response function calibration (IRF) was recorded using a diluted Ludox® dispersion. Lifetime analysis was performed using the commercial EasyTau 2 software (PicoQuant). The quality of the fit was assessed by minimizing the reduced chi squared function (χ^2) and visual inspection of the weighted residuals and their autocorrelation.

Femtosecond transient absorption measurements

The details of the employed fs-TA setup have been described elsewhere.[2,3] Briefly, a commercial Ti:sapphire amplifier (Solstice, Spectra-Physics) at 1 kHz repetition rate was employed as the main laser source. The 120 fs pulses at 800 nm were converted to λ_{ex} = 513 nm and 295 nm as pump pulses for λ_{ex}-dependent fs-TA experiments by using a non-collinear optical parametric amplifier (TOPAS White, Light Conversion) and subsequent up-conversion with the fundamental beam. A duration of ~25 fs was extracted for visible pulses by performing frequency-resolved optical gating (FROG) with type-I second-harmonic generation in BBO. For UV pulses (~105 fs), cross-correlation FROG with well-characterized fundamental pulses (800 nm, 100 fs) was employed by type-I difference-frequency generation (DFG) in BBO. The broadband probe pulses were generated by focusing 800 nm pulses into a linearly moving CaF_2 window (2 mm thick). The linearly polarized pump and probe pulses were spatially overlapped in a 0.2 mm thick quartz cuvette with 54.7° mutual polarizations. The delay time was varied up to 3.8 ns by using a commercial translation stage (M-IMS600,

Newport). The probe pulses were eventually dispersed in a spectrometer (Acton SP2500i, Princeton Instruments) and detected shot-to-shot by a CCD camera (Pixis 2K, Princeton Instruments). The transient data measured were evaluated *via* target analysis[4] with the software package Glotaran based on the R-package TIMP.[5]

Theoretical calculations

All electronic structure calculations of D-RC-A and A-RC-A on ground and excited states were performed with the Gaussian 16 package.[6] For the S_0, S_1 and T_2 states of D-RC-A and A-RC-A, a time-dependent density functional theory (TD-DFT) approach was employed for excitation energy calculations and geometric optimizations, while unrestricted density functional theory (UDFT) calculations were performed for optimizing the T_1 states.[7,8] For light elements, PBE0/6-31g* level was employed while LANL2DZ effective core potential basis set was used for Rh(III).[9,10] The natural transition orbital (NTO) analysis of low-lying excited states was performed using the Multiwfn program,[11] and resulting NTO distributions were visualized by the VMD program.[12] MOMAP software[13–15] was employed to estimate the reorganization energy (Γ) of corresponding transitions with a harmonic oscillator approximation,[16,17]

$$\Gamma = \sum_k \Gamma_k = \sum_k \frac{1}{2}\omega_k^2 \Delta q_k^2 \quad . \tag{1}$$

Here, the reorganization energy contribution of each vibrational mode (Γ_k) was calculated by the corresponding frequency (ω_k) and structural difference (Δq_k) with respect to the equilibrium position of the ground state.[18–20] The Δq_k can be further expressed as a linear combination of internal coordinates, *i.e.,*

$$\Delta q_k = \sum_j \zeta_{kj} \Delta D_j \quad , \tag{2}$$

in which ΔD_j represents the displacement with respect to the equilibrium position along internal coordinate *j*. The SOC matrix elements $\langle S_1|\hat{H}_{SO}|T_1\rangle$ and $\langle S_1|\hat{H}_{SO}|T_2\rangle$ were calculated by using a linear response approach implemented in the PySOC program,[21] in which the SOC Hamiltonian can be approximately described as

$$\widehat{H}_{\mathrm{SO}} \approx \sum_i^{N_e} \zeta(r_i)\widehat{\mu}_{\mathrm{L}}\widehat{\mu}_{\mathrm{S}} \quad , \tag{3}$$

where μ_L and μ_S represent magnetic moments resulting from orbital and spin angular momentum with the SOC constant $\zeta(r_i)$.

Results and Discussion

S1. Photophysical properties and electronic transitions

Table S1. Reported photophysical properties of 2,5-substituted RCPDs with two electron acceptors (A-A), two electron donors (D-D), and electron donor/acceptor (D-A) in toluene under oxygen-free conditions.

		Φ_f	τ_{S1} (ns)	k_r (10^8 s^{-1})	k_{nr} (10^8 s^{-1})	Ref.
H-H	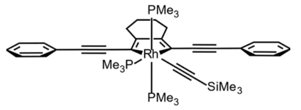	0.33	1.2	2.75	5.5	9
A-A	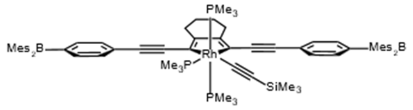	0.69	2.6	-	-	10
A-A	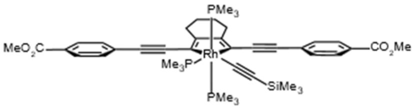	0.69	3.0	2.3	1.0	9
A-A	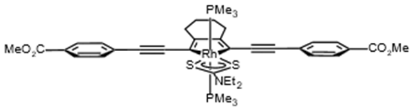	0.46	2.5	1.8	2.2	9
A-A	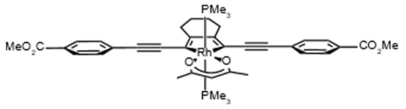	0.50	2.5	2.0	2.0	1
A-A	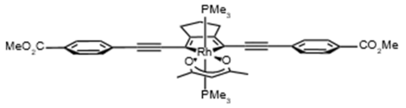	0.54	1.7	3.2	2.7	1
D-D	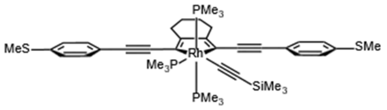	0.34	1.8	1.9	3.7	9
D-D	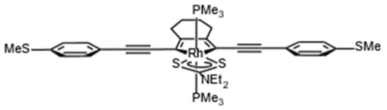	0.16	1.1	1.6	8.5	9
D-D	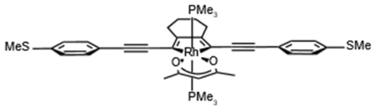	0.13	0.7	1.5	9.9	1
D-D	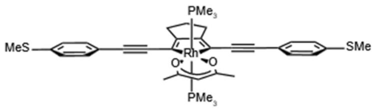	0.07	2.0	2.8	36.6	1
D-A	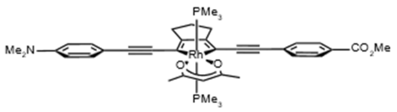	0.22	0.5	2.8	9.8	1

Table S2. Calculated vertical and adiabatic transitions of the lowest-lying excited states (S_1, T_1 and T_2) of D-RC-A and A-RC-A, H = HOMO, L = LUMO, CT = charge transfer, LE = local excited.

		Excitation energy (eV)		*f* (vertical)	Transitions	Character
		vertical	adiabatic			
D-RC-A	S_1	2.2189	1.9518	1.4500	H → L (97.9%)	CT
D-RC-A	T_1	1.1429	1.1228	0.0000	H → L (83.9%)<br>H → L+1 (11.3%)	CT
D-RC-A	T_2	2.3909	2.0222	0.0000	H → L+1 (56.2%)<br>H–1→ L (17.4%)	LE
A-RC-A	S_1	2.2222	1.9392	1.4020	H → L (98.5%)	LE
A-RC-A	T_1	1.0997	1.0809	0.0000	H → L (92.6%)	LE
A-RC-A	T_2	2.3125	2.2131	0.0000	H → L+1 (65.7%)<br>H–2→ L (21.1%)	LE

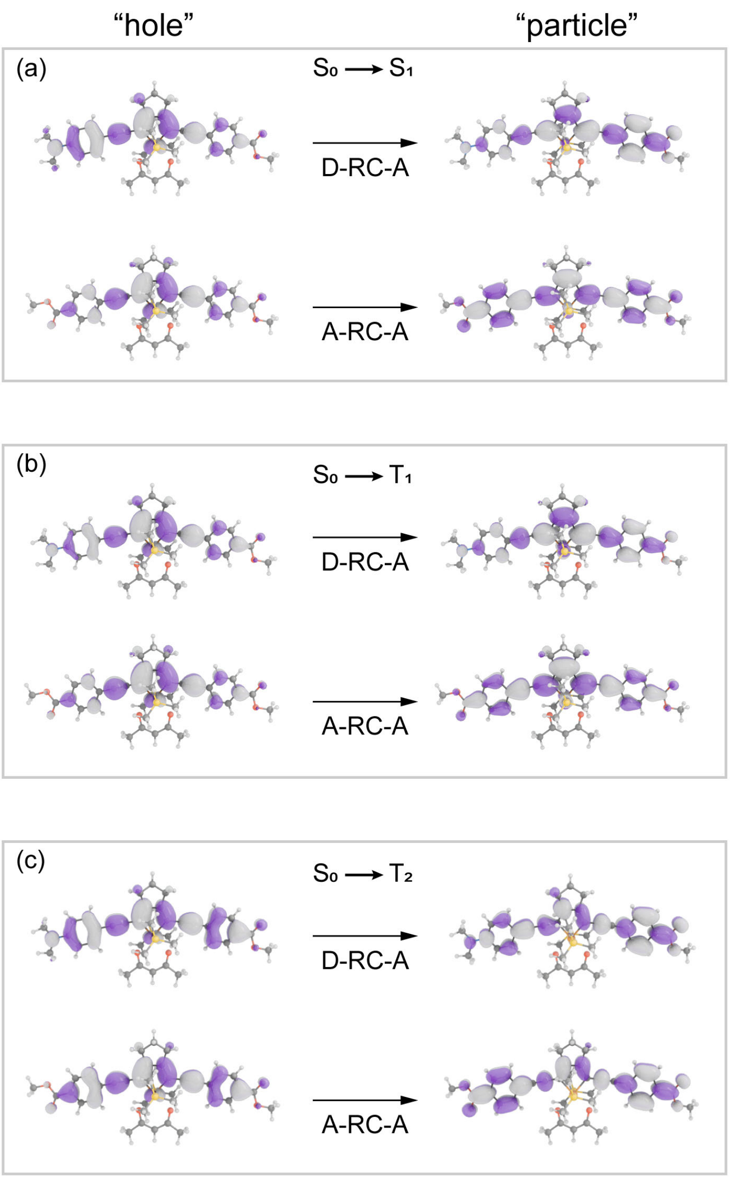

Figure S1. NTO analysis of $S_0 \rightarrow S_1$ (a), $S_0 \rightarrow T_1$ (b), and $S_0 \rightarrow T_2$ (c) transitions for D-RC-A and A-RC-A.

S2. ISC dynamics

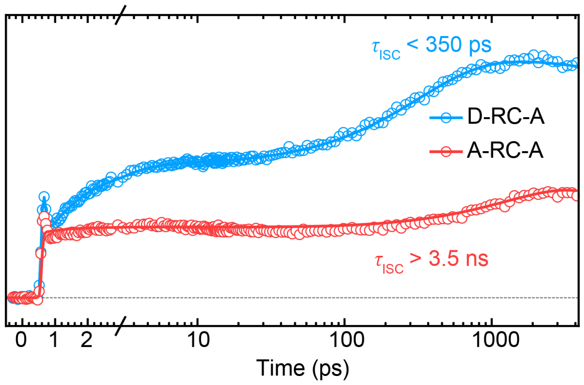

Figure S2. Time traces (blank circles) and multi-exponential fitting (solid lines) of the fs-TA at selected probe wavelengths for D-RC-A (λ_{pr} = 580 nm) and A-RC-A (λ_{pr} = 570 nm) upon UV excitation.

S3. Target analysis of TA data

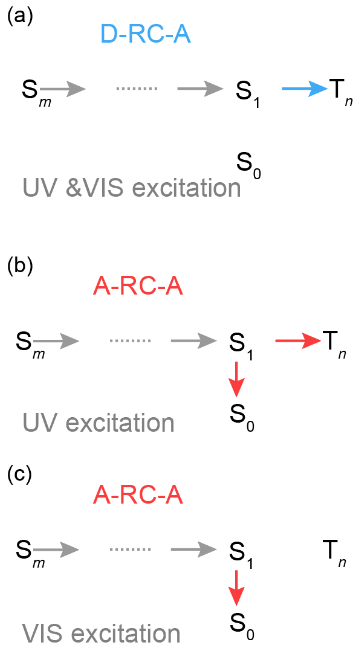

Figure S3. Target analysis models applied to femtosecond transient absorption (fs-TA) data for D-RC-A excited by UV & VIS light (a), and A-RC-A upon UV (b) and visible (c) excitation, in which S_m, S_1, and T_n and S_0 refer to the singlet ($m \geq 1$), relaxed lowest singlet, triplet (T_n, $n \geq 1$) excited states and ground state, respectively. Solid arrows symbolize the kinetic processes considered in the target analysis. These excluded pathways are characterized by slow kinetics when contrasted with parallel, more rapid processes and are therefore neglected in the modeling of the fs-TA data.

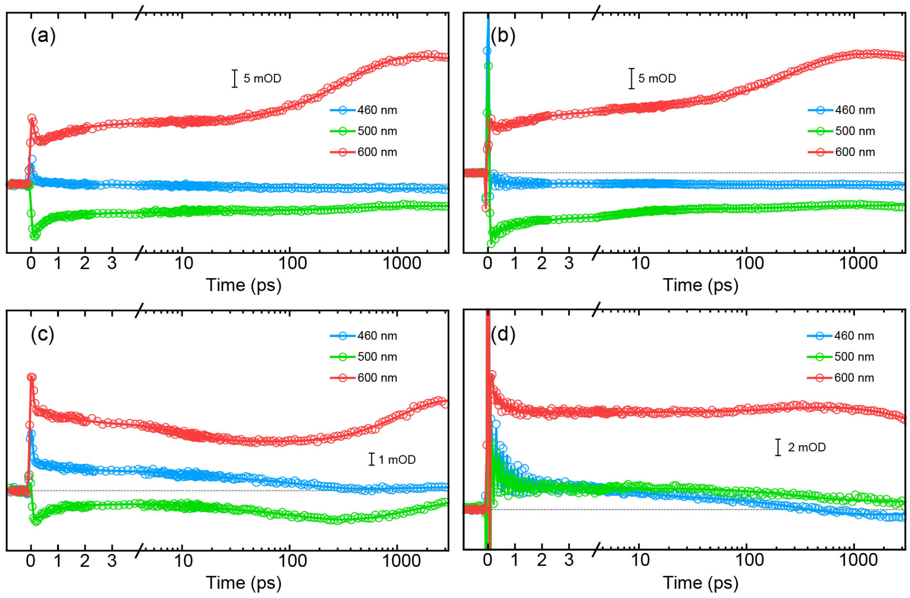

Figure S4. Time traces (open circles) and fitting curves (solid lines) by target analysis of the fs-TA at selected probe wavelengths for D-RC-A upon UV (a) and visible (b) excitation, as well as A-RC-A upon UV (c) and visible (d) excitation.

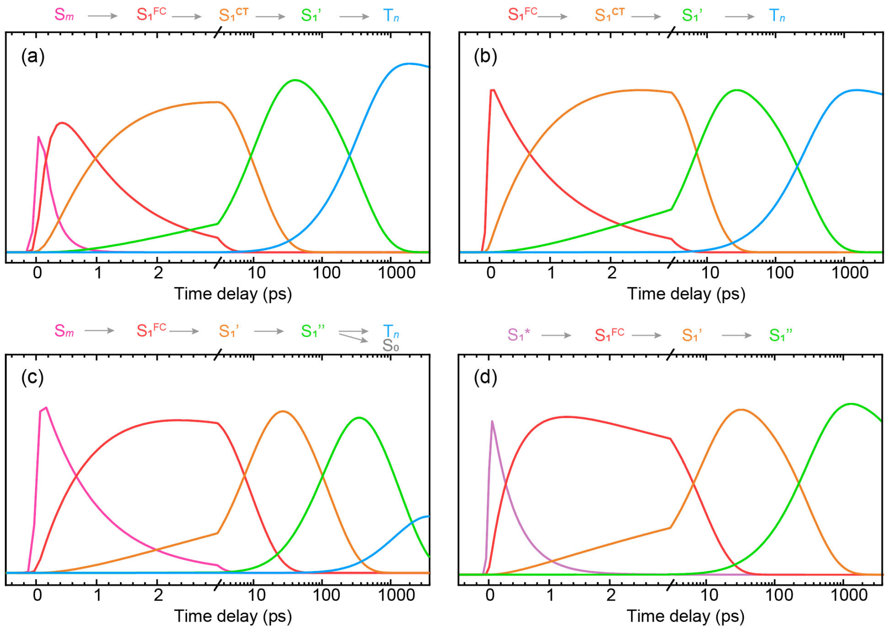

Figure S5. Target-analysis-extracted concentration evolution of transient species for D-RC-A upon UV (a) and visible (b) excitation, as well as A-RC-A upon UV (c) and visible (d) excitation.

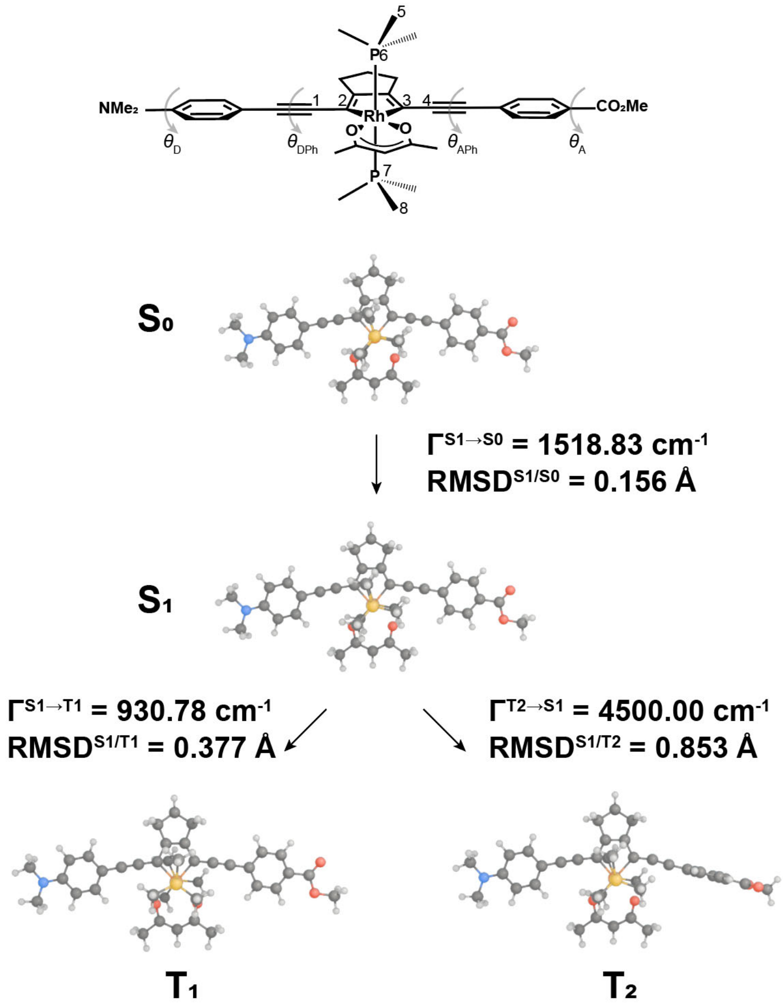

Figure S6. TD-DFT-optimized geometries of D-RC-A at the S_0, S_1, T_1, and T_2 states.

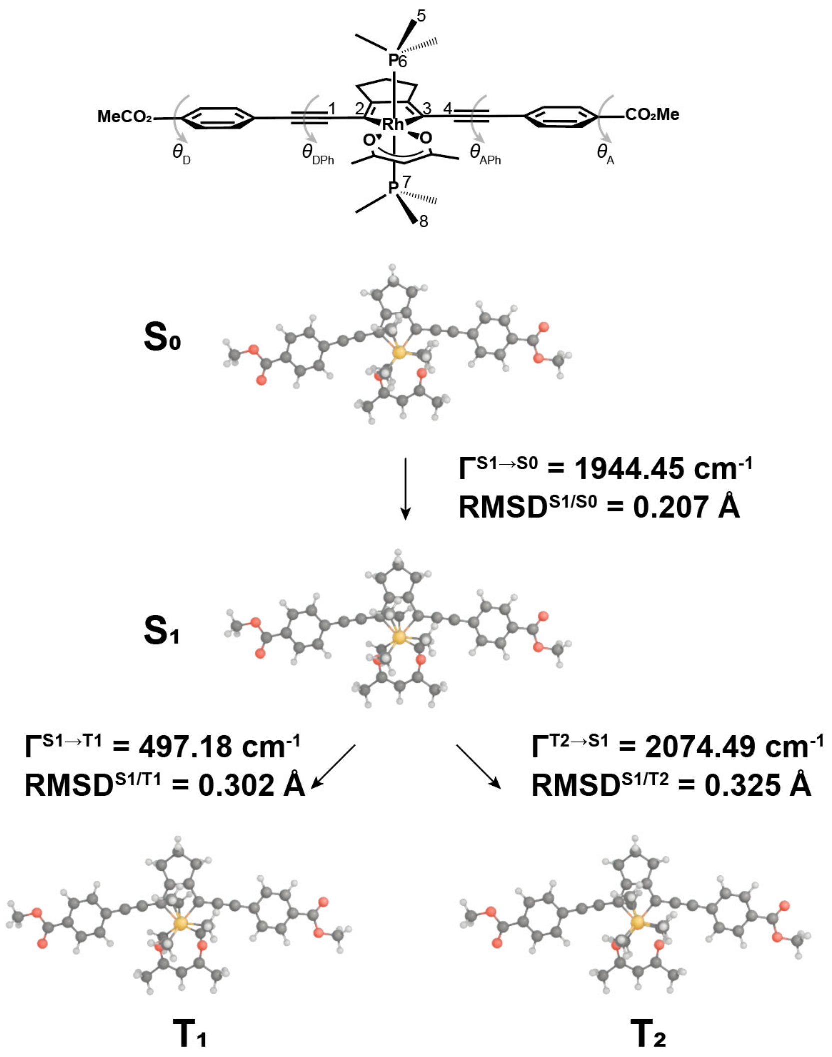

Figure S7. TD-DFT-optimized geometries of A-RC-A at the S_0, S_1, T_1, and T_2 states.

Table S3. TD-DFT-optimized angles and dihedral angles (numbering see Figures S6 and S7) of D-RC-A and A-RC-A at the S_0, S_1, T_1, and T_2 states.

D-RC-A	θ_{D} (°)	θ_{A} (°)	θ_{DPh} (°)	θ_{APh} (°)	θ_{1234} (°)	θ_{5678} (°)
S_0	4.931	0.004	0.090	2.169	2.462	0.099
S_1	0.374	0.053	0.059	0.110	6.016	11.209
T_1	4.729	0.386	0.486	0.190	8.397	30.011
T_2	0.043	0.340	0.0004	90.523	2.629	10.526
A-RC-A	θ_{A1} (°)	θ_{A2} (°)	θ_{A1Ph} (°)	θ_{A2Ph} (°)	θ_{1234} (°)	θ_{5678} (°)
S_0	0.034	0.006	1.198	2.667	2.145	0. 343
S_1	0.157	0.664	0.215	0.234	5.162	13.805
T_1	0.197	0.355	0.065	0.323	4.501	19.924
T_2	0.043	0.087	6.362	6.567	0.646	1.650

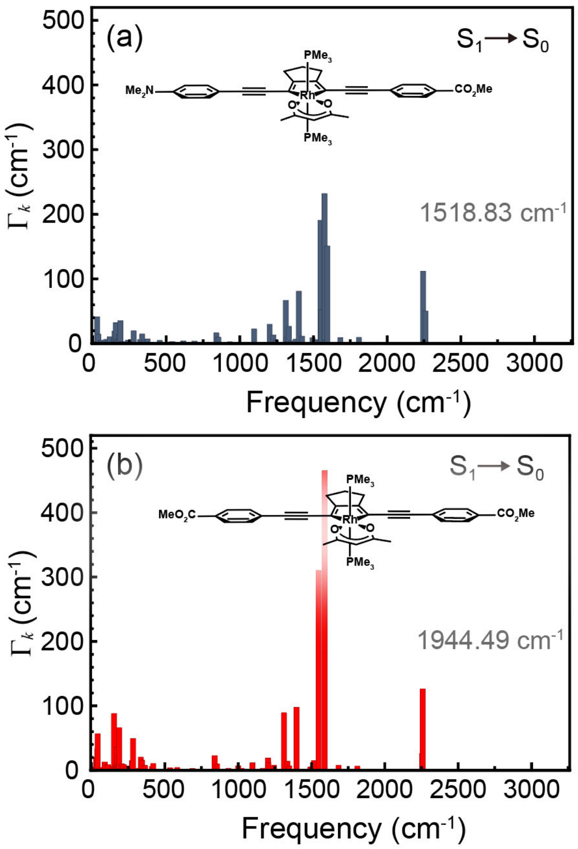

Figure S8. TD-DFT-calculated reorganization energy of each vibrational mode for the $S_1 \rightarrow S_0$ transition of D-RC-A (a) and A-RC-A (b). The values of total reorganization energy are provided for each complex and transition.

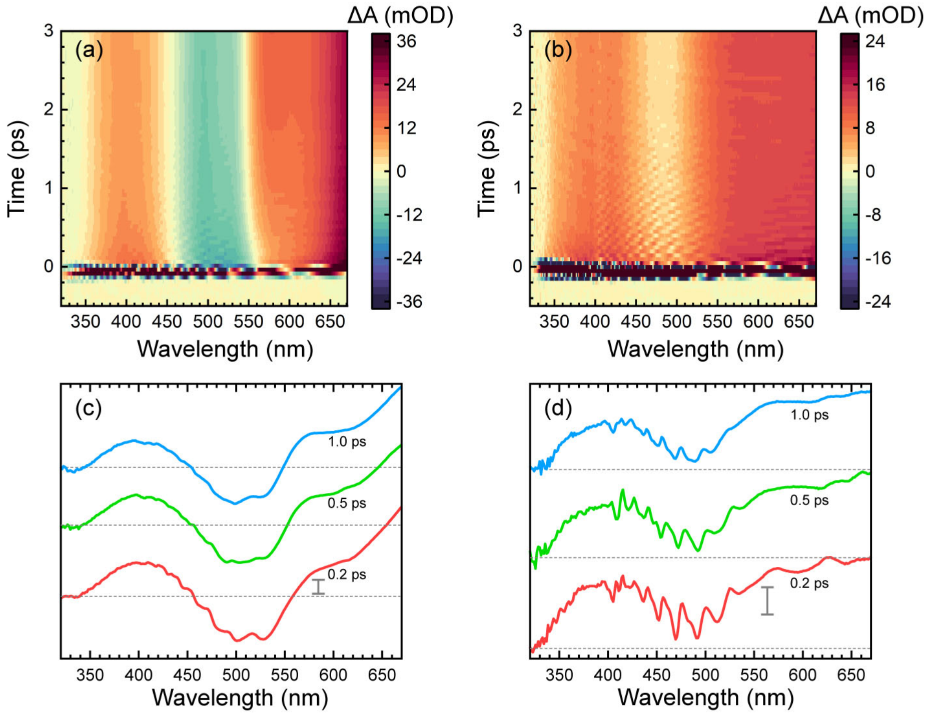

Figure S9. Chirp-corrected spectro-temporal maps of fs-TA signal in –0.5 – 3.0 ps range for D-RC-A (a) and A-RC-A (b) upon visible optical excitation at λ_{ex} = 513 nm; the corresponding TA spectra at selected delay times are illustrated in (c) and (d), respectively.

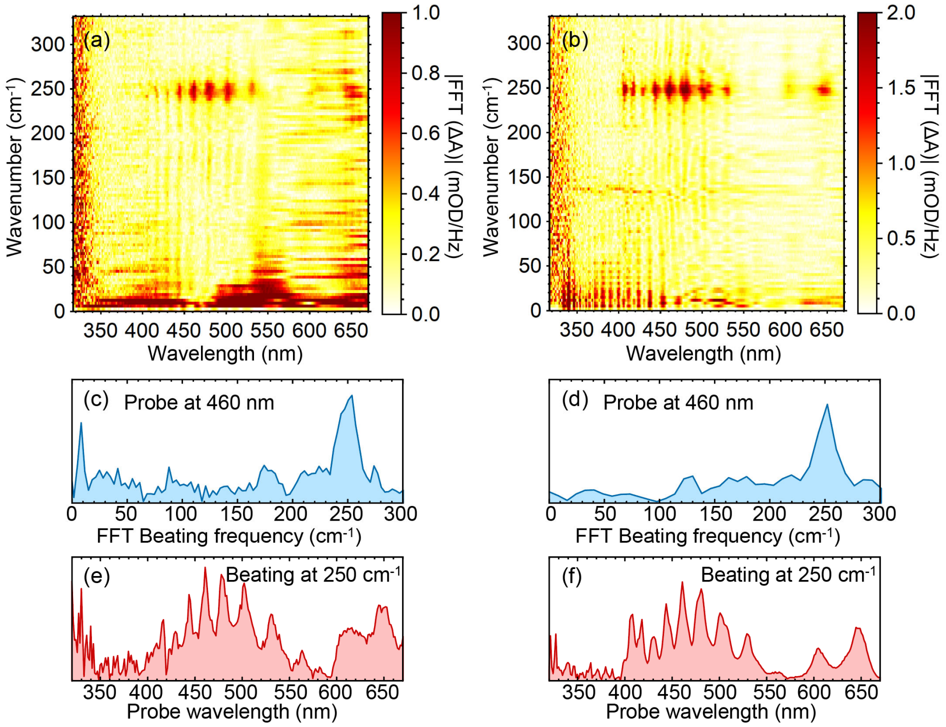

Figure S10. Fast Fourier transform (FFT) of oscillation signal of TA data for D-RC-A (a) and A-RC-A (b); FFT power spectra of oscillation components probed at 460 nm for D-RC-A (c) and A-RC-A (d); distribution of beating at ~250 cm^{-1} along the probe wavelength for D-RC-A (e) and A-RC-A (f).

Table S4. Photoluminescence quantum yields, Φ_f, for A-RC-A collected at different excitation wavelengths.

Excitation wavelength (nm)	Φ_f
295	0.29
422	0.29
473	0.31
513	0.33

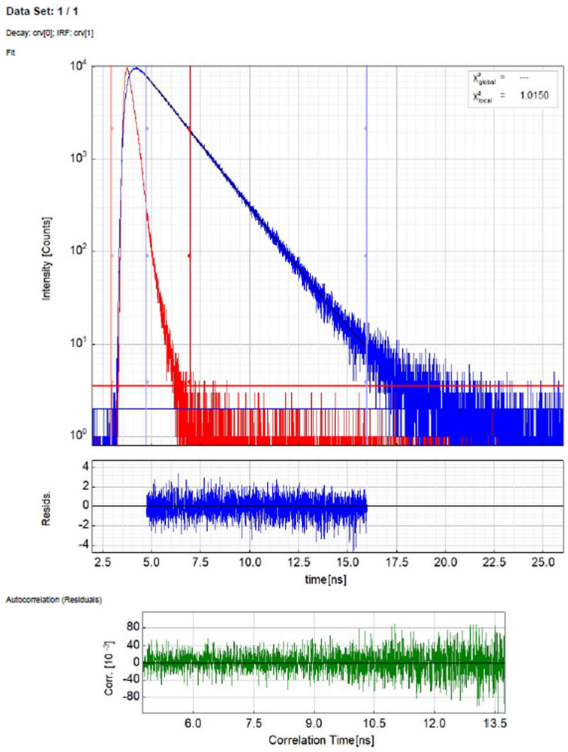

Fitted Parameters

Parameter	Value	Δ	δ
A_1[kCnts/Chnl]	13.68	±0.71	5.1%
τ_1[ns]	1.6050	±0.0062	0.4%
I_1[kCnts]	5 490	±290	5.3%
A_{Rel1}[%]	100.0	---	---
I_{Rel1}[%]	100.0	---	---
$Bkgr_{Dec}$[kCnts]	0.0020	±0.0006	27%
$Bkgr_{IRF}$[Cnts/Chnl]	3.6	±6.4	182%
$Shift_{IRF}$[ps]	78	±89	115%
τ_{AvInt}[ns]	1.6050	±0.0062	0.4%
τ_{AvAmp}[ns]	1.6050	±0.0062	0.4%

Figure S11. Emission decay of A-RC-A in THF collected at 317 nm excitation. A lifetime of 1.6 ns was determined (χ^2 = 1.02).

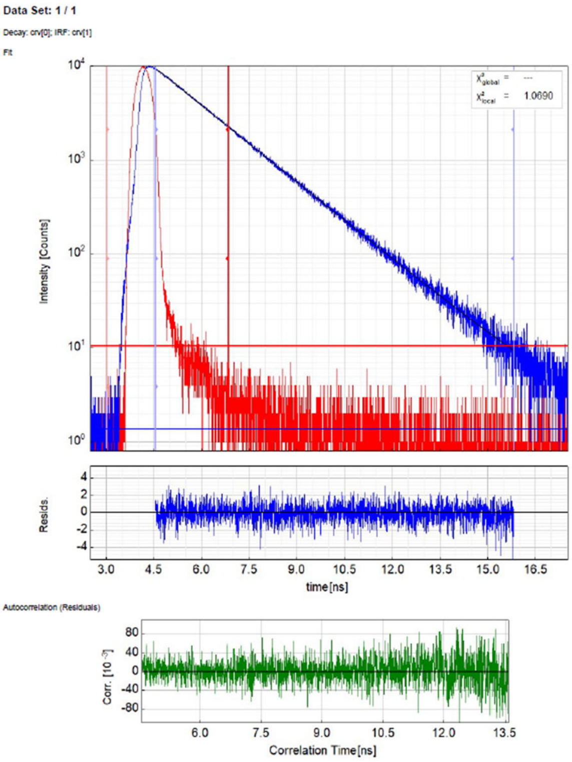

Fitted Parameters

Parameter	Value	Δ	δ
A_1[kCnts/Chnl]	12.06	±0.14	1.1%
τ_1[ns]	1.5943	±0.0054	0.3%
I_1[kCnts]	4 804	±72	1.5%
A_{Rel1}[%]	100.0	---	---
I_{Rel1}[%]	100.0	---	---
$Bkgr_{Dec}$[kCnts]	0.0014	±0.0004	25%
$Bkgr_{IRF}$[Cnts/Chnl]	10.5	±3.1	29%
$Shift_{IRF}$[ps]	171	±23	13%
τ_{AvInt}[ns]	1.5943	±0.0054	0.3%
τ_{AvAmp}[ns]	1.5943	±0.0054	0.3%

Figure S12. Emission decay of A-RC-A in THF collected at 440 nm excitation. A lifetime of 1.6 ns was determined (χ^2 = 1.01).

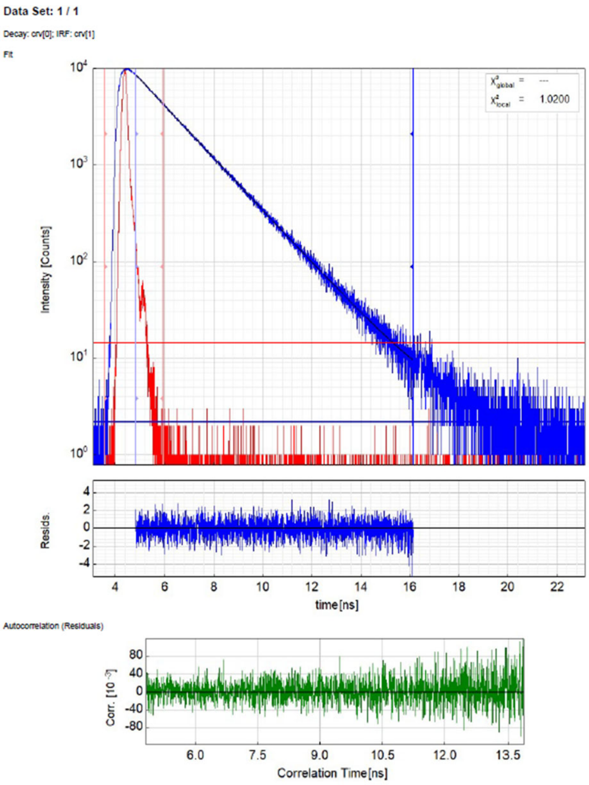

Fitted Parameters

Parameter	Value	Δ	δ
A_1[kCnts/Chnl]	11.6	±1.9	16%
τ_1[ns]	1.5996	±0.0032	0.2%
I_1[kCnts]	4 610	±720	15%
A_{Rel1}[%]	100.0	---	---
I_{Rel1}[%]	100.0	---	---
$Bkgr_{Dec}$[kCnts]	0.0022	±0.0004	16%
$Bkgr_{IRF}$[Cnts/Chnl]	15	±11	73%
$Shift_{IRF}$[ps]	250	±260	101%
τ_{AvInt}[ns]	1.5996	±0.0032	0.2%
τ_{AvAmp}[ns]	1.5996	±0.0032	0.2%

Figure S13. Emission decay of A-RC-A in THF collected at 505 nm excitation. A lifetime of 1.6 ns was determined (χ^2 = 1.02).

S4. Estimation of the ISC kinetics of A-RC-A under VIS excitation

Because the S_1 state experiences a parallel reaction contributed to by three first-order reactions, *i.e.*, radiative transition, internal conversion, and ISC with rate constants k_r, k_{IC}, and k_{ISC}, respectively, the decay of the S_1 state is also a first-order reaction with rate constant of $k_{S1} = k_r + k_{IC} + k_{ISC}$. Meanwhile, the quantum yield of ISC can be calculated as $\Phi_{ISC} = k_{ISC} / (k_r + k_{IC} + k_{ISC}) = k_{ISC} / k_{S1}$, which gives $k_{S1} = k_{ISC} / \Phi_{ISC}$. Similarly, we have $k_{S1} = k_r / \Phi_f$ and $k_{S1} = k_{IC} / \Phi_{IC}$.

Upon UV excitation, the ISC rate of A-RC-A can be calculated as $k_{ISC}^{UV} = 1/\tau_{ISC}^{UV} = 2.58 \times 10^8$ s^{-1}, in which the time constant of ISC (τ_{ISC}^{UV} = 3.88 ns) was determined by fs-TA.

For estimating the corresponding triplet-state quantum yield (Φ_{ISC}^{UV}), we assumed Φ_{ISC}^{UV} is approximately equal to the maximum value of singlet oxygen quantum yield (Φ_Δ) sensitized by the triplet state of A-RC-A, which was determined as 0.34. Considering that ~11% of the singlet-state oxygen will be quenched by A-RC-A in toluene-d_8, the value of Φ_{ISC}^{UV} can be deduced proportionally as ~0.37. So that the decay rate of S_1 state can be calculated as $k_{S1}^{UV} = k_{ISC}^{UV} / \Phi_{ISC}^{UV} = 6.96 \times 10^8$ s^{-1}, the S_1 state lifetime is $\tau_{S1}^{UV} = 1/ k_{S1}^{UV}$ = 1.44 ns, which is highly consistent with the measured fluorescence lifetime (~1.6 ns).

With the measured fluorescence quantum yield (Φ_f^{UV}) upon UV excitation (295 nm, Table S4), the radiative decay rate was estimated by $k_r = k_{S1}^{UV} \times \Phi_f^{UV} = 2.02 \times 10^8$ s^{-1}.

Then, upon visible excitation (513 nm, Table S4), the fluorescence quantum yield (Φ_f^{VIS}) was determined to be 0.33. Since k_r is independent of excitation wavelength, the S_1-state decay rate and lifetime can be calculated as $k_{S1}^{VIS} = k_r / \Phi_f^{VIS} = 6.12 \times 10^8$ s^{-1} and $\tau_{S1}^{VIS} = 1/ k_{S1}^{VIS}$ = 1.63 ns, respectively.

For estimating the ISC rate of A-RC-A upon visible excitation, we assumed that the slowing down of ISC upon visible excitation can be attributed to different S_1-state decay rate, then $k_{ISC}^{VIS} = k_{ISC}^{UV} + k_{S1}^{VIS} - k_{S1}^{UV} = 1.74 \times 10^8$ s^{-1}, while the ISC time constant is $\tau_{ISC}^{VIS} = 1/k_{ISC}^{VIS}$ = 5.75 ns.

S5. Studies of $^1O_2$ sensitization by A-RC-A

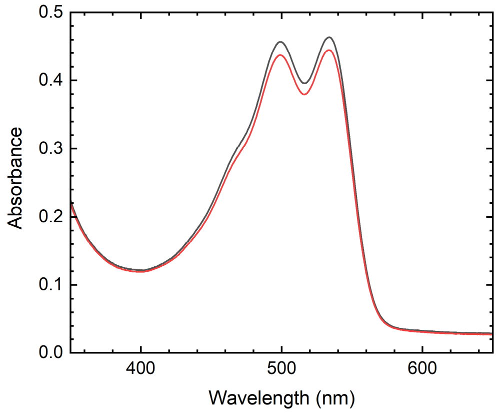

Figure S14. Photobleaching of A-RC-A in air-saturated toluene-d_8 solution before (black) and after (red) 10 min irradiation at 417 nm (fs laser operated at 0.5 kHz repetition, average power of 1.8 mW/cm^2). The solution was not stirred therefore we cannot quantify this change in absorbance. However, the same experiment in toluene-h_8 did not show evidence of bleaching. Therefore, given the appreciably longer lifetime of singlet oxygen in toluene-d_8, and hence the increased probability of reaction with a solute, we infer that A-RC-A bleaching under these conditions is due to singlet oxygen.

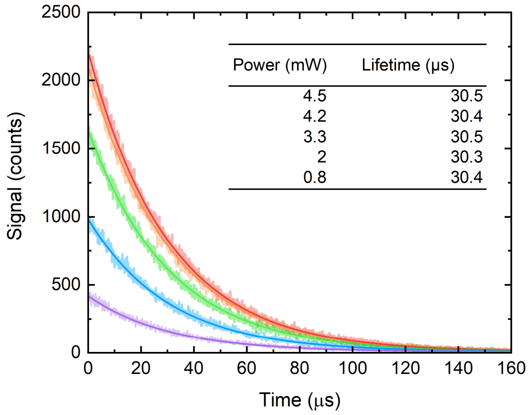

Figure S15. Time-resolved $O_2(a^1\Delta_g)$ phosphorescence traces recorded upon pulsed laser irradiation of A-RC-A at 417 nm in toluene-h_8 for different laser powers (fs laser operated at 1 kHz repetition). Single exponential fits to the data are shown as solid lines. The traces recorded at 4.5 and 4.2 mW overlap appreciably and are almost indistinguishable from each other. In all cases, an intense "spike" coincident with the laser pulse, and likely deriving from A-RC-A fluorescence combined with scattered laser light, was detected at time = 0. We eliminated the data showing this spike for presentation in the Figure. The $O_2(a^1\Delta_g)$ lifetimes obtained from the single exponential fits are consistent with the expectation for toluene-h_8.[22]

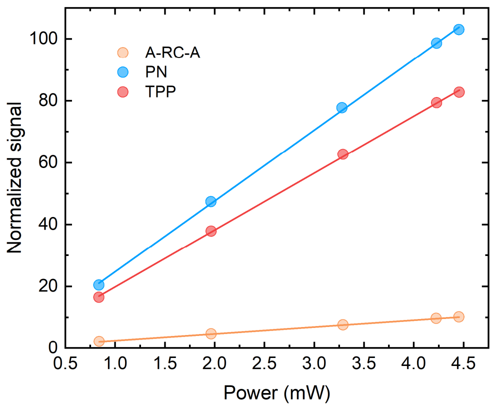

Figure S16. Integrated intensity of the $O_2(a^1\Delta_g)$ phosphorescence signal, normalized by the sensitizer absorbance and the $O_2(a^1\Delta_g)$ lifetime, plotted as a function of laser power for A-RC-A and for the reference standards, phenalenone (PN) and tetraphenylporphyrin (TPP). In such plots, the slopes of the linear fits are proportional to the $O_2(a^1\Delta_g)$ quantum yield.

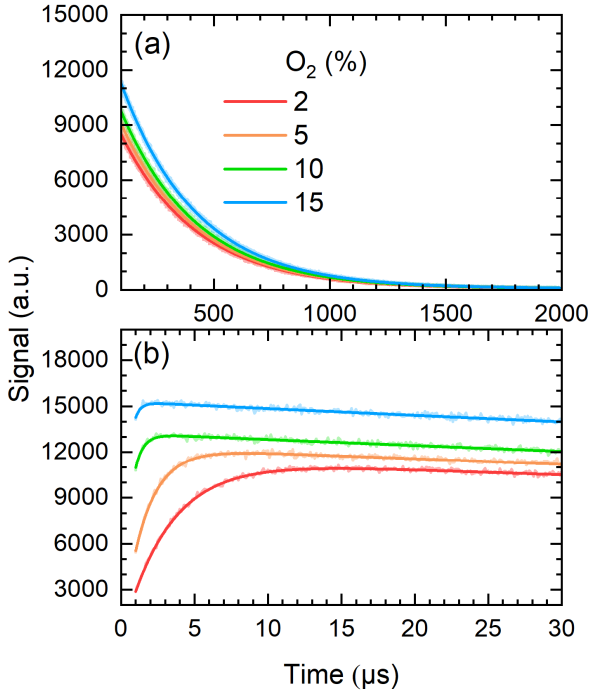

Figure S17. Representative time-resolved $O_2(a^1\Delta_g)$ phosphorescence traces recorded upon irradiation of PN at 417 nm in toluene-d_8. Data were recorded as a function of the O_2 concentration, controlled by the percent of oxygen in a mixture of O_2 and N_2 gas bubbled through the solvent. (a) The data from 100 μs to 2000 μs were fitted by a single exponential decay function to obtain the lifetime of $O_2(a^1\Delta_g)$ (*i.e.*, $1/k_\Delta$). (b) Using k_Δ as a fixed parameter, eq 5 was used as a fitting function to obtain values of k_T for a time domain where $O_2(a^1\Delta_g)$ was formed in the photosensitized reaction. Fits are shown as solid lines superimposed on each trace.

S6. NMR Spectra and Purification of A-RC-A

The compound was purified by column chromatography (Al_2O_3, basic) with n-hexane/THF 30:1 to 0:1 (6 or 7 times). The BHT impurity was reduced by column chromatography (Al_2O_3, basic) flushing with 10 volumes of toluene, and recovering the product in pure THF.

During the course of our current study, we noticed that the NMR spectra and data for A-RC-A presented in our previous paper were likely that for a different derivative which was not discussed in that paper.[1] In particular, the position of the signals for the Me group of the terminal CO_2Me fragment in the ^1H and ^{13}C NMR spectra were upfield from where they would be expected (cf: the directly analogous compound with a $(CH_2)_4$-containing backbone). Thus, we re-recorded the NMR spectra and the new, correct, spectra and data are provided below. We note that the original elemental analysis, mass spectroscopic data, and single-crystal X-ray diffraction data are all correct as are all other NMR spectra and data in the previous paper. In addition, the newly recorded absorption and emission spectra and fluorescence lifetime data are in agreement with the data presented in the previous paper.[1]

^1H NMR (400 MHz, C_6D_6, 294 K) δ: 8.11 (d, J = 8.5 Hz, 4H), 7.66 (d, J = 8.5 Hz, 4H), 5.12 (s, 1H), 3.47 (s, 6H), 2.71 (tt, J = 7, 3 Hz, 4H), 1.90 (s, 6H), 0.99 – 0.90 (m, 18H).

^{13}C NMR (151 MHz, C_6D_6, 294 K) δ: 186.86, 166.47, 141.53, 136.04, 130.82, 129.96, 99.81, 66.98, 51.51, 34.35, 32.56, 30.48, 28.50, 23.75, 21.44, 11.06 (t, J = 14 Hz).

^{31}P NMR (162 MHz, C_6D_6, 294 K) δ: -1.30 (d, J = 113 Hz).

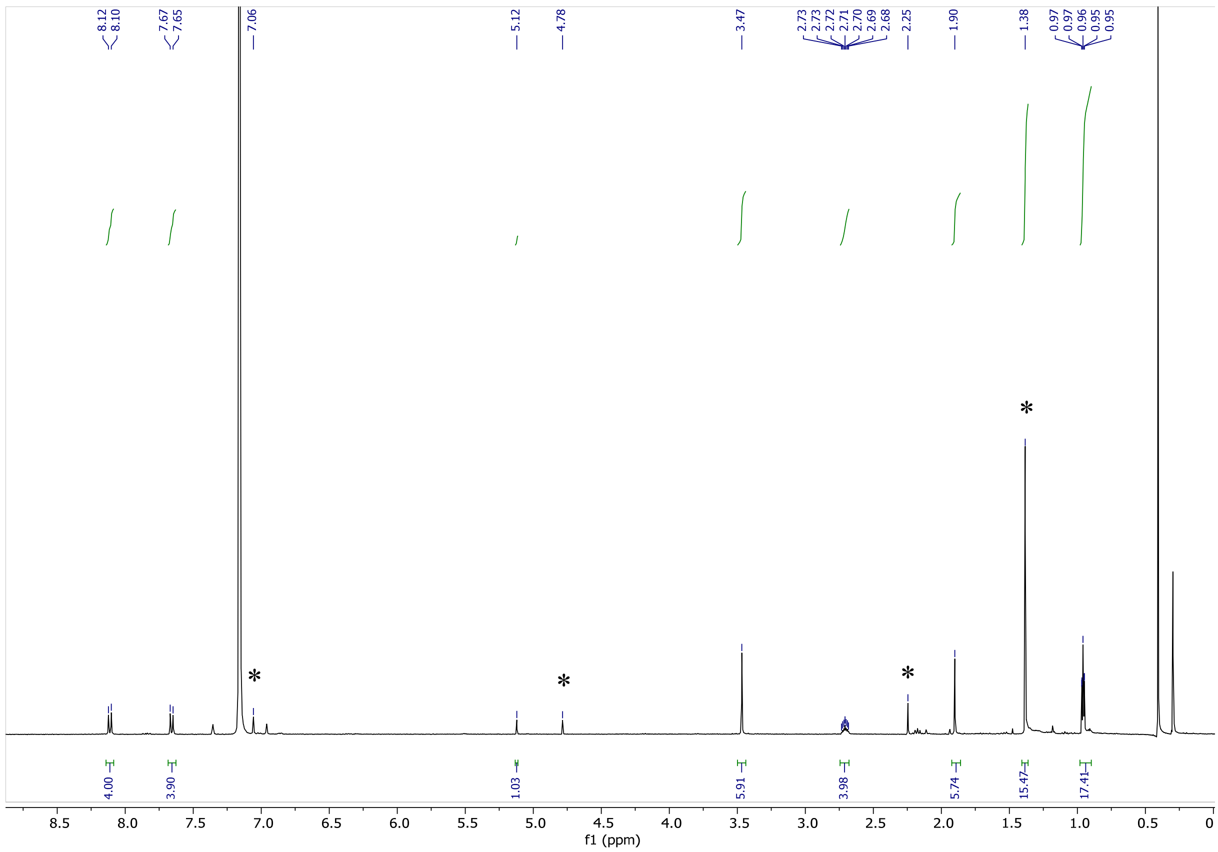

Figure S18. ^{1}H-NMR spectrum (C_6D_6, 400 MHz). Marked with stars are the peaks of the BHT impurity (7.06; 4.78; 2.24, 1.38). The signals at approximately 0.4 and 0.29 ppm can be assigned to water and silicone grease, respectively, originating from the NMR solvent.

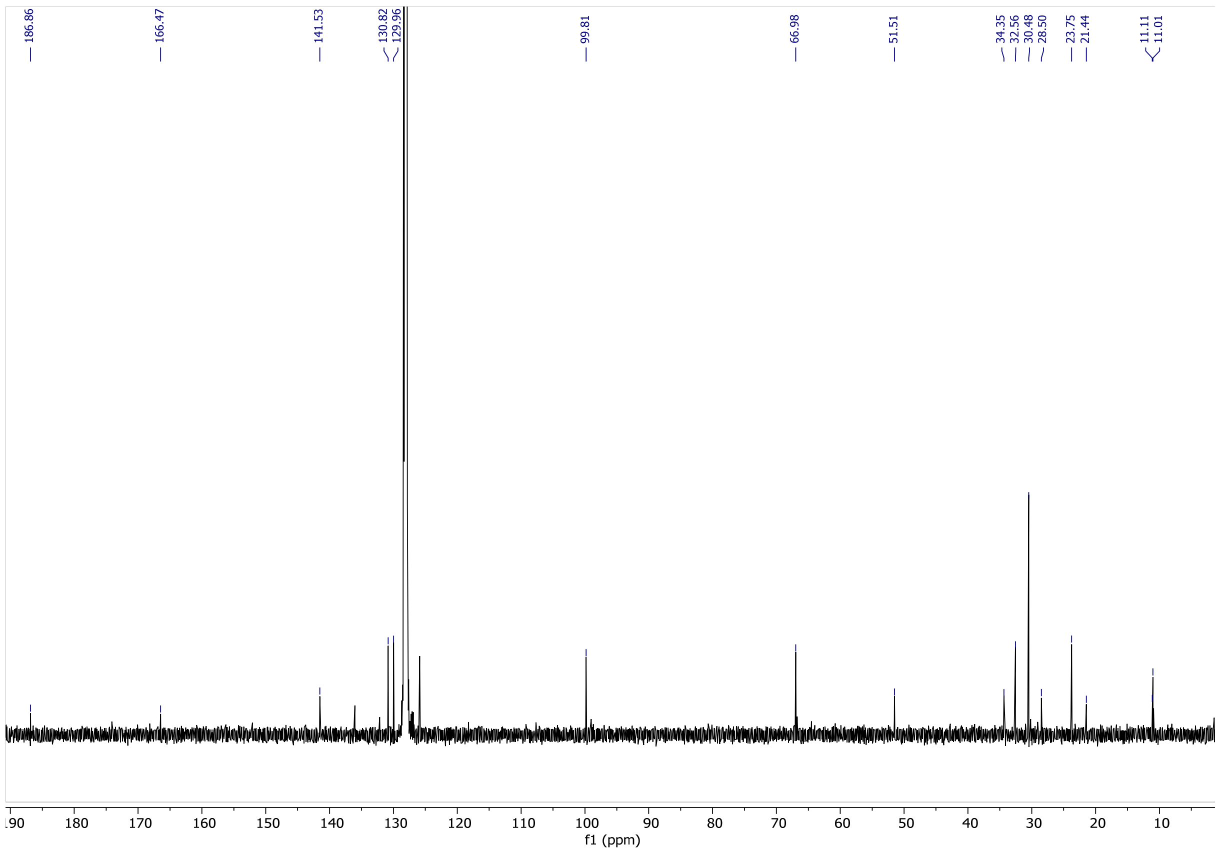

Figure S19. ^{13}C-NMR spectrum (C_6D_6, 101 MHz).

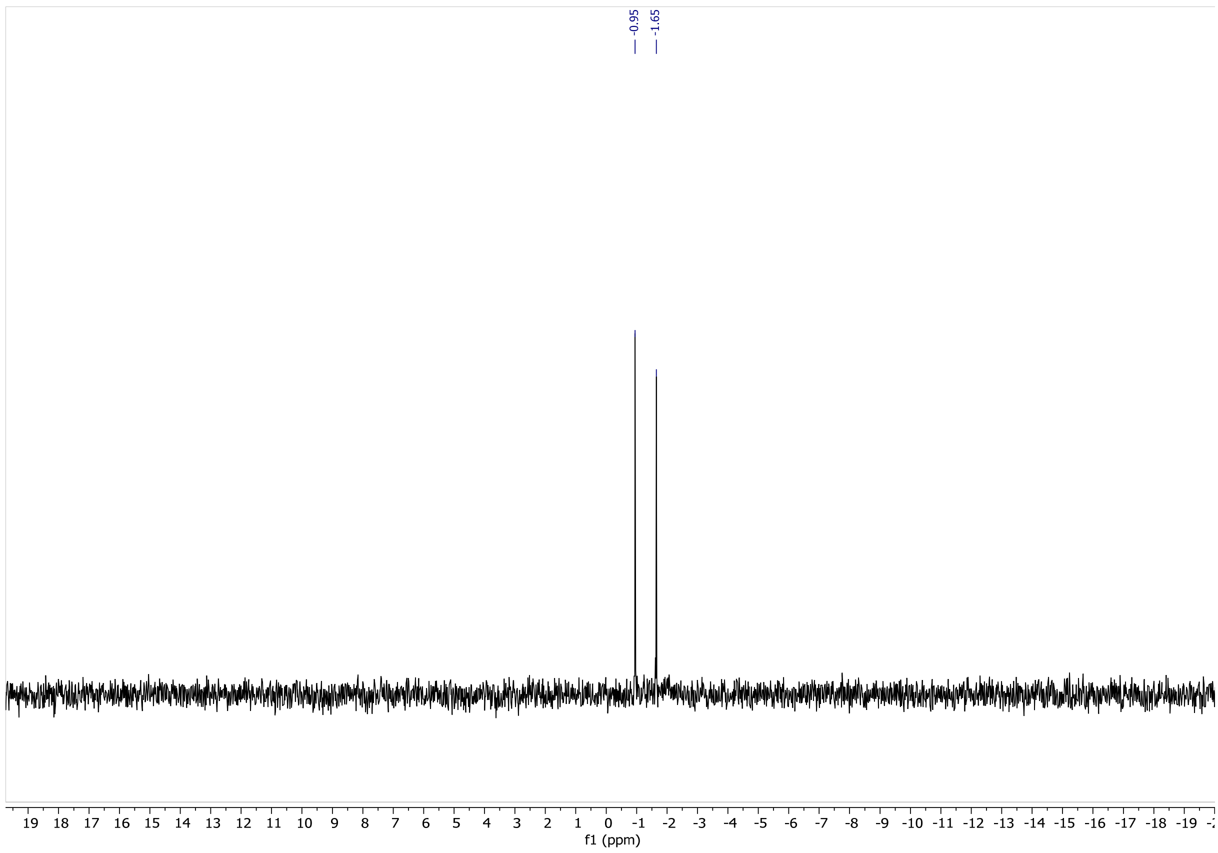

Figure S20. ^{31}P-NMR spectrum (C_6D_6, 162 MHz).

References

[1] C. Sieck, M. G. Tay, M.-H. Thibault, R. M. Edkins, K. Costuas, J.-F. Halet, A. S. Batsanov, M. Haehnel, K. Edkins, A. Lorbach, A. Steffen, T. B. Marder, *Chem. Eur. J.* **2016**, *22*, 10523–10532.

[2] X. Ma, J. Maier, M. Wenzel, A. Friedrich, A. Steffen, T. B. Marder, R. Mitrić, T. Brixner, *Chem. Sci.* **2020**, *11*, 9198–9208.

[3] X. Ma, M. Wenzel, H.-C. Schmitt, M. Flock, E. Reusch, R. Mitrić, I. Fischer, T. Brixner, *Phys. Chem. Chem. Phys.* **2018**, *20*, 15434–15444.

[4] I. H. M. van Stokkum, D. S. Larsen, R. van Grondelle, *Biochim. Biophys. Acta Bioenerg.* **2004**, *1657*, 82–104.

[5] K. M. Mullen, I. H. M. van Stokkum, *J. Stat. Softw.* **2007**, *18*, 1–46.

[6] Gaussian 16, Revision C.01, M. J. Frisch, G. W. Trucks, H. B. Schlegel, G. E. Scuseria, M. A. Robb, J. R. Cheeseman, G. Scalmani, V. Barone, G. A. Petersson, H. Nakatsuji, X. Li, M. Caricato, A. V. Marenich, J. Bloino, B. G. Janesko, R. Gomperts, B. Mennucci, H. P. Hratchian, J. V. Ortiz, A. F. Izmaylov, J. L. Sonnenberg, D. Williams-Young, F. Ding, F. Lipparini, F. Egidi, J. Goings, B. Peng, A. Petrone, T. Henderson, D. Ranasinghe, V. G. Zakrzewski, J. Gao, N. Rega, G. Zheng, W. Liang, M. Hada, M. Ehara, K. Toyota, R. Fukuda, J. Hasegawa, M. Ishida, T. Nakajima, Y. Honda, O. Kitao, H. Nakai, T. Vreven, K. Throssell, J. A. Montgomery Jr., J. E. Peralta, F. Ogliaro, M. J. Bearpark, J. J. Heyd, E. N. Brothers, K. N. Kudin, V. N. Staroverov, T. A. Keith, R. Kobayashi, J. Normand, K. Raghavachari, A. P. Rendell, J. C. Burant, S. S. Iyengar, J. Tomasi, M. Cossi, J. M. Millam, M. Klene, C. Adamo, R. Cammi, J. W. Ochterski, R. L. Martin, K. Morokuma, O. Farkas, J. B. Foresman, D. J. Fox, Gaussian, Inc., Wallingford CT, **2016**.

[7] Z. Lin, T. Van Voorhis, *J. Chem. Theory Comput.* **2019**, *15*, 1226–1241.

[8] K. Schmidt, S. Brovelli, V. Coropceanu, J.-L. Brédas, C. Bazzini, T. Caronna, R. Tubino, F. Meinardi, *J. Phys. Chem. A* **2006**, *110*, 11018–11024.

[9] A. Steffen, K. Costuas, A. Boucekkine, M.-H. Thibault, A. Beeby, A. S. Batsanov, A. Charaf-Eddin, D. Jacquemin, J.-F. Halet, T. B. Marder, *Inorg. Chem.* **2014**, *53*, 7055–7069.

[10] A. Steffen, M. G. Tay, A. S. Batsanov, J. A. K. Howard, A. Beeby, K. Q. Vuong, X.-Z. Sun, M. W. George, T. B. Marder, *Angew. Chem. Int. Ed.* **2010**, *122*, 2399–2403.

[11] T. Lu, F. Chen, *J. Comput. Chem.* **2012**, *33*, 580–592.

[12] W. Humphrey, A. Dalke, K. Schulten, *J. Mol. Graph.* **1996**, *14*, 33–38.

[13] Q. Peng, Y. Niu, Q. Shi, X. Gao, Z. Shuai, *J. Chem. Theory Comput.* **2013**, *9*, 1132–1143.

[14] Y. Niu, W. Li, Q. Peng, H. Geng, Y. Yi, L. Wang, G. Nan, D. Wang, Z. Shuai, *Mol. Phys.* **2018**, *116*, 1078–1090.

[15] Q. Peng, Y. Yi, Z. Shuai, J. Shao, *J. Am. Chem. Soc.* **2007**, *129*, 9333–9339.

[16] J. R. Reimers, *J. Chem. Phys.* **2001**, *115*, 9103–9109.

[17] Z. Pei, Q. Ou, Y. Mao, J. Yang, A. de la Lande, F. Plasser, W. Liang, Z. Shuai, Y. Shao, *J. Phys. Chem. Lett.* **2021**, *12*, 2712–2720.

[18] Y. Wang, Z. Guo, Y. Gao, Y. Tian, Y. Deng, X. Ma, W. Yang, *J. Phys. Chem. Lett.* **2022**, *13*, 6664–6673.

[19] L. Wang, Q. Ou, Q. Peng, Z. Shuai, *J. Phys. Chem. A* **2021**, *125*, 1468–1475.

[20] D. Fan, Y. Yi, Z. Li, W. Liu, Q. Peng, Z. Shuai, *J. Phys. Chem. A* **2015**, *119*, 5233–5240.

[21] X. Gao, S. Bai, D. Fazzi, T. Niehaus, M. Barbatti, W. Thiel, *J. Chem. Theory Comput.* **2017**, *13*, 515–524.

[22] M. Bregnhøj, M. Westberg, B. F. Minaev, P. R. Ogilby, *Acc. Chem. Res.* **2017**, *50*, 1920–1927.
