# Supplementary material for: Ultrafast photophysics of para-substituted 2,5-bis(arylethynyl) rhodacyclopentadienes: thermally activated intersystem crossing
Source: Chem Sci. 2024 Aug 15;15(36):14746–56. doi: 10.1039/d4sc04306e (PMC11337014; doi:10.1039/d4sc04306e)
Supplement: SC-015-D4SC04306E-s002 [file SC-015-D4SC04306E-s002.pdf]

Supplementary Information (SI) for Chemical Science.
This journal is © The Royal Society of Chemistry 2024

Supporting Information

Ultrafast Photophysics of *para*-Substituted 2,5-Bis(arylethynyl) Rhodacyclopentadienes: Thermally Activated Intersystem Crossing

Zilong Guo,[b] Yaxin Wang,[b] Julia Heitmüller,[a] Carolin Sieck,[c] Andreas Prüfer,[d] Philipp Ralle,[d] Andreas Steffen,[d] Petr Henke,[e,g] Peter R. Ogilby,*[e] Todd B. Marder,*[c,f] Xiaonan Ma,*[b,a] and Tobias Brixner*[a,f]

Contents

S7. Raw data of fs-TA measurements S3

Table S5 S3

Table S6 S112

Table S7 S221

Table S8 S331

S8. TD-DFT and UDFT results of D-RC-A and A-RC-A S442

Table S9 S442

Table S10 S444

Table S11 S447

Table S12 S449

Table S13 S452

Table S14 S454

Table S15 S456

Table S16 S459

S7. Raw data of fs-TA measurements.

Table S5. Raw data of fs-TA signals of D-RC-A collected under UV (λ_{ex} = 295 nm) excitation in the λ_{pr} = 320–670 nm regime, the unit of ΔA in the table is mOD.

Wavelength (nm) / Time (ps)	320.70	322.11	323.52	324.93	326.34	327.75	329.16	330.56	331.97	333.38	334.79
-3.28	-0.04	0.89	0.32	0.04	0.49	0.16	0.74	-0.03	0.36	0.56	-0.47
-2.78	0.25	-0.19	-0.02	-0.51	0.10	0.14	-0.35	0.19	-0.11	-0.53	0.04
-2.28	1.17	-0.05	-0.41	-0.30	0.30	0.28	-0.08	0.32	0.03	-0.39	0.41
-1.78	-0.25	0.48	0.32	0.20	-0.10	-0.42	0.17	0.22	0.35	0.06	-0.15
-1.28	0.61	0.34	1.15	0.45	0.14	1.01	1.07	0.57	0.32	0.43	0.45
-0.78	-0.83	0.42	-0.91	0.09	-0.75	0.03	-0.44	0.01	-0.41	0.15	0.01
-0.28	-0.01	-1.14	-0.29	0.24	-0.18	-0.52	-0.74	-0.72	-0.25	0.09	0.05
0.22	-0.91	-0.75	-0.16	-0.21	0.00	-0.69	-0.38	-0.56	-0.29	-0.38	-0.33
0.32	-1.90	-0.65	0.45	0.07	1.03	0.27	0.75	0.16	-0.01	-0.04	0.36
0.42	0.62	-1.69	-3.67	-4.11	-3.96	-3.03	-1.30	0.63	0.73	1.19	0.61
0.52	2.10	1.94	1.61	0.60	1.21	0.03	0.67	0.21	-1.49	-4.62	-6.09
0.62	1.06	2.27	0.84	1.18	-0.22	1.23	0.61	0.90	0.06	0.22	-0.73
0.72	0.72	1.19	1.53	1.47	0.95	1.30	0.96	1.22	1.13	0.99	0.79
0.77	1.61	0.72	0.08	-0.02	1.55	0.08	1.41	0.96	0.64	0.69	0.03
0.82	1.92	1.20	0.48	1.12	0.17	0.44	0.15	1.12	0.24	0.09	0.18
0.87	1.60	1.56	1.64	0.59	1.07	0.82	1.05	0.69	0.64	0.16	0.49
0.92	3.04	2.95	2.61	1.75	2.27	1.56	1.53	1.33	1.11	0.93	0.43
0.97	2.46	1.71	2.67	1.29	1.65	1.58	1.50	1.64	1.45	1.05	0.71
1.02	1.04	1.05	1.85	0.82	1.12	1.13	0.53	0.78	0.60	0.36	0.15
1.07	2.95	1.16	0.11	1.42	0.96	0.92	0.84	1.30	0.32	-0.11	0.07
1.12	2.78	1.09	1.38	1.89	1.35	1.10	2.06	1.70	0.98	0.69	1.06
1.17	2.76	2.43	2.93	2.23	2.89	1.91	1.73	1.48	1.25	0.82	0.32
1.22	3.27	2.50	2.39	1.96	1.81	1.26	2.04	1.61	1.54	1.04	1.06
1.27	3.22	2.66	1.61	1.64	1.81	1.65	1.96	2.32	1.76	0.93	0.58
1.32	2.48	2.02	1.42	1.16	1.00	1.37	1.19	1.64	0.44	1.06	0.68
1.37	1.67	0.92	1.53	1.51	0.60	0.65	0.80	0.67	0.99	0.60	-0.02
1.42	3.25	2.73	2.77	1.83	1.60	1.60	1.34	1.88	1.92	1.22	0.83
1.47	2.82	2.98	2.56	2.05	1.42	1.89	1.79	1.64	0.77	0.88	0.58
1.52	2.00	2.21	1.61	1.37	1.30	1.02	1.76	1.67	1.27	0.34	0.25
1.57	2.07	2.02	1.65	1.81	1.88	1.30	2.03	1.79	0.88	0.90	1.01
1.62	1.78	1.82	1.19	0.96	0.64	0.60	1.56	1.14	1.54	0.66	0.58
1.67	1.82	2.64	2.43	1.27	1.53	1.88	1.70	1.36	1.41	1.03	0.74
1.72	3.56	3.10	2.65	2.84	2.10	2.19	2.46	2.45	1.72	1.82	1.21
1.77	3.49	2.78	3.01	2.64	1.72	1.70	2.69	2.19	1.51	1.50	0.66
1.82	3.17	2.09	1.48	0.62	1.12	0.93	1.55	1.18	1.05	0.62	0.52
1.87	1.12	1.84	0.94	1.36	1.30	0.70	1.27	0.46	0.29	0.48	0.38
1.92	2.09	1.01	2.16	1.44	1.30	1.37	0.49	1.25	1.04	0.70	0.75
1.97	3.43	2.82	3.18	2.03	1.49	2.72	2.39	2.31	1.52	1.82	1.17
2.02	3.41	2.74	2.01	1.60	1.90	2.71	2.04	2.55	1.12	1.78	1.23
2.07	3.38	3.49	3.05	2.85	2.11	2.92	2.35	2.30	2.23	1.65	1.17
2.12	1.76	1.98	2.70	1.93	1.08	1.11	1.16	1.10	1.18	0.45	1.17
2.17	1.31	0.84	1.70	1.57	1.33	1.28	0.49	1.08	0.59	0.97	0.69
2.22	2.80	2.76	0.97	1.76	0.76	1.33	2.31	1.71	2.05	1.67	0.69

2.27	2.11	1.76	1.50	1.42	1.69	1.60	2.40	1.14	0.96	0.63	0.26
2.32	3.92	3.45	3.00	2.69	2.71	2.51	2.59	2.46	2.45	1.58	1.36
2.37	3.40	1.97	3.07	1.84	2.20	1.41	2.08	1.42	1.56	1.12	1.26
2.42	1.41	1.65	-0.38	0.65	0.93	1.27	1.06	1.09	1.00	0.81	0.88
2.47	2.09	2.10	1.33	0.29	0.48	0.98	0.98	1.03	0.76	0.50	0.29
2.52	1.91	2.32	2.85	1.44	1.21	2.46	2.18	1.44	1.42	1.54	1.09
2.57	4.77	2.48	2.65	2.67	1.91	2.00	2.96	2.33	1.60	1.67	1.88
2.62	2.79	2.62	2.07	2.97	2.62	2.97	2.44	1.85	1.41	1.56	1.17
2.67	1.56	1.48	1.20	1.97	1.47	1.35	1.54	1.98	0.88	0.86	0.70
2.72	0.65	0.87	1.61	1.65	0.12	0.44	1.55	0.39	1.18	1.41	0.40
2.77	2.23	1.27	2.01	1.77	1.33	1.96	2.06	2.31	1.87	1.67	1.65
2.82	2.57	2.24	2.92	1.80	1.11	1.78	1.71	1.47	1.07	1.13	0.60
2.87	3.59	3.81	2.76	3.07	2.30	2.75	2.36	1.93	1.58	2.04	1.39
2.92	1.76	2.55	2.36	2.14	2.41	1.68	2.10	1.99	1.37	0.93	1.21
2.97	2.41	2.98	1.38	3.03	1.67	1.66	2.23	2.28	1.97	2.27	0.50
3.02	2.07	2.10	1.87	0.86	2.07	1.14	1.67	1.77	1.95	0.46	0.95
3.07	2.62	2.70	1.77	1.29	2.49	1.64	1.99	1.52	1.54	0.78	0.64
3.12	3.50	2.99	2.59	2.61	3.35	1.96	2.55	2.15	1.33	0.87	1.26
3.17	3.90	4.67	3.28	4.02	2.94	2.73	3.52	2.85	1.98	2.36	2.01
3.22	1.91	1.15	1.98	1.97	0.58	1.61	1.19	1.03	0.89	1.06	0.64
3.27	2.72	3.61	-0.47	0.99	2.69	0.93	-0.51	1.71	1.58	0.19	0.77
3.32	3.60	0.95	1.22	0.82	2.00	1.10	0.75	0.94	1.72	0.90	0.22
3.37	3.11	3.15	2.32	2.89	1.91	2.56	2.41	2.53	1.66	2.20	0.72
3.42	3.28	2.93	2.07	2.16	2.43	2.18	1.90	2.25	2.17	1.36	1.06
3.47	1.45	3.40	2.36	1.61	1.56	1.99	1.86	2.27	1.16	1.09	0.33
3.52	1.68	1.17	2.92	1.37	2.07	1.38	1.34	0.66	1.69	1.24	1.16
3.57	0.82	1.18	1.46	1.04	0.57	0.88	1.02	1.53	0.72	1.47	0.20
3.62	1.74	2.88	3.18	3.03	1.44	2.19	2.45	2.26	1.89	1.66	1.28
3.67	4.55	3.03	3.08	2.32	3.50	2.32	2.88	1.95	1.52	1.68	1.85
3.72	4.03	3.01	2.21	2.74	2.21	2.55	2.92	2.40	1.19	1.57	1.42
3.92	3.26	3.37	2.53	1.96	1.92	1.95	2.92	2.30	1.54	1.55	1.45
4.12	1.41	1.25	2.16	0.77	1.94	1.59	1.42	1.19	1.61	0.71	0.79
4.32	1.30	0.91	1.76	1.52	-0.10	1.15	1.17	0.99	0.38	0.64	0.38
4.52	2.83	1.98	1.60	1.81	2.27	1.90	1.43	2.10	1.59	1.28	0.87
4.72	3.84	3.56	3.24	2.84	2.70	3.11	3.07	2.61	2.00	2.23	1.91
4.92	3.58	4.01	2.72	2.11	2.53	1.75	2.13	2.34	1.78	1.61	0.98
5.12	0.86	2.38	1.77	1.54	1.18	1.54	1.88	2.25	1.66	1.16	0.75
5.32	1.95	2.77	1.05	1.10	1.08	2.04	1.14	1.65	1.81	0.34	0.98
5.52	2.22	1.98	2.00	1.67	1.71	1.66	2.25	0.77	1.74	0.98	0.77
5.72	3.44	3.48	3.75	2.23	2.15	3.29	3.06	1.56	2.54	2.04	1.90
5.92	3.88	2.82	3.26	3.06	2.58	2.62	2.68	2.45	2.03	1.84	1.52
6.12	2.78	2.60	2.36	2.07	2.00	2.37	1.85	2.02	1.93	1.58	0.88
6.32	1.91	1.80	1.36	1.10	1.89	1.62	1.50	1.37	0.57	1.20	0.72
6.52	2.22	0.81	1.37	1.74	1.54	1.16	2.06	1.61	0.66	0.92	1.00
6.72	3.58	4.14	2.18	1.91	2.71	2.48	2.01	2.88	2.05	1.58	1.80
6.92	4.12	3.60	2.55	3.01	2.12	2.54	2.40	2.44	2.43	1.69	0.74
7.12	2.29	2.54	1.85	2.34	2.18	2.08	2.08	1.75	1.58	1.63	1.08
7.32	3.09	2.56	1.92	1.96	2.07	2.35	3.08	1.48	2.11	1.58	1.68
7.52	1.64	0.58	1.61	0.91	0.77	1.07	1.08	1.13	0.97	0.47	-0.20
7.72	1.55	2.16	1.87	1.82	1.55	2.16	2.40	1.04	1.50	1.33	1.09

7.92	2.75	3.24	2.59	2.01	2.54	2.61	2.43	1.89	2.01	1.45	1.12
8.12	2.95	2.58	2.25	2.46	2.19	2.39	2.97	2.24	1.83	1.58	1.02
8.32	2.88	3.20	2.65	2.15	1.52	2.05	2.15	1.83	2.10	1.25	0.52
8.52	2.40	2.17	1.23	1.10	1.51	1.86	1.44	1.81	1.11	0.51	0.64
8.72	1.74	1.44	1.22	1.68	1.60	1.60	1.97	1.28	0.79	1.33	1.35
8.92	3.61	2.58	0.77	2.28	2.45	1.40	1.67	2.20	1.56	1.22	1.96
9.12	3.51	3.37	3.04	2.54	1.90	2.66	2.86	2.12	1.79	1.87	1.70
9.32	3.82	3.98	2.92	3.91	3.02	2.85	3.14	3.31	3.13	2.01	2.39
9.52	2.81	2.65	2.79	2.61	2.84	2.42	2.55	1.75	2.10	1.10	1.85
9.72	1.85	0.65	1.77	1.82	1.87	1.13	2.12	2.01	0.93	0.80	1.40
9.92	2.13	2.40	2.50	1.00	2.55	2.12	1.57	2.28	1.88	1.53	1.33
10.12	3.13	3.66	1.62	2.24	2.35	2.40	1.53	1.90	2.25	1.60	1.18
10.32	3.19	2.81	3.01	2.23	2.23	2.68	1.98	2.70	2.59	2.35	1.35
10.52	3.73	3.14	1.57	1.33	2.27	1.65	1.86	1.98	1.91	1.23	1.34
10.72	2.18	2.60	2.75	1.51	1.93	2.38	2.37	2.43	1.92	1.74	1.44
10.92	1.87	2.07	2.24	1.26	1.81	1.53	1.92	1.70	1.54	1.43	0.58
11.12	2.99	2.47	2.65	1.63	1.90	1.92	2.56	2.49	2.09	1.91	1.92
11.32	2.71	3.49	2.81	2.91	2.01	2.11	2.60	2.48	2.42	1.64	1.48
11.52	4.06	3.57	3.01	2.69	2.29	1.78	1.70	2.24	2.21	1.46	0.89
11.72	1.79	1.27	1.24	1.36	0.66	1.85	1.99	1.59	1.38	0.75	0.71
11.92	1.77	1.39	1.14	0.97	0.76	0.66	1.19	0.40	1.07	0.52	0.82
12.12	1.16	1.23	1.49	1.40	1.02	0.82	0.78	0.76	0.63	0.76	0.87
12.32	2.29	2.30	2.37	2.33	2.36	2.21	1.55	1.54	1.30	1.87	1.51
12.52	2.59	3.15	3.55	2.05	2.62	1.88	2.64	2.67	2.56	1.60	1.48
12.72	3.67	2.98	3.17	2.70	2.56	1.84	2.77	2.55	2.42	1.46	1.31
12.92	2.33	1.87	2.52	1.94	1.66	1.75	2.18	1.99	1.28	1.11	1.47
13.12	1.18	2.03	1.10	0.77	1.45	1.52	1.43	1.29	1.34	1.18	0.97
13.32	2.57	1.69	3.34	2.52	2.44	1.80	2.42	1.89	2.55	1.76	1.86
13.52	3.79	3.33	3.16	3.11	2.76	3.63	2.97	2.17	1.75	1.75	1.59
13.72	3.48	3.45	3.70	3.64	3.06	3.34	3.10	3.30	2.22	1.91	2.62
13.92	2.75	2.82	2.64	2.20	2.28	2.23	2.81	1.61	1.81	1.61	1.26
14.00	1.95	1.23	1.91	1.47	1.29	1.17	0.91	1.38	0.98	1.43	1.11
14.08	2.06	2.73	0.85	1.59	0.94	1.53	1.26	0.73	1.50	0.51	0.60
14.17	2.27	1.75	2.07	1.73	1.83	2.21	0.93	1.13	1.37	0.97	0.80
14.27	3.16	3.33	2.72	3.31	2.42	1.85	3.07	2.38	2.26	1.61	1.84
14.38	3.78	2.93	3.08	1.88	2.44	2.06	2.32	2.54	1.99	1.89	1.30
14.49	2.69	1.69	1.58	2.59	2.49	1.49	2.26	1.63	1.55	1.52	0.82
14.61	1.91	1.58	1.25	1.00	0.96	0.83	2.04	1.51	1.47	0.71	0.65
14.75	3.04	2.71	1.16	0.73	1.97	2.51	2.09	1.52	2.23	1.18	0.93
14.89	2.68	3.17	2.42	1.52	1.78	1.42	2.11	1.59	1.81	1.42	0.81
15.05	3.81	3.19	2.93	1.80	2.91	2.90	2.30	2.89	2.41	1.38	2.12
15.21	2.45	3.24	2.16	1.71	1.95	2.23	2.27	1.53	1.43	1.04	0.53
15.39	2.71	1.96	1.59	1.80	2.22	1.79	2.05	2.02	1.50	0.59	0.95
15.59	1.97	1.61	1.84	1.28	1.80	1.92	1.04	2.27	0.34	1.08	1.09
15.80	3.35	2.61	2.96	2.61	2.09	1.90	2.40	1.91	1.60	1.54	1.77
16.02	3.82	3.68	2.30	3.04	2.69	3.11	2.39	2.11	1.54	1.86	1.12
16.27	4.43	2.91	3.49	3.91	2.94	2.69	3.13	2.74	2.40	2.10	1.52
16.53	1.10	2.93	2.60	1.60	2.47	1.91	1.94	1.52	1.71	1.79	0.64
16.81	3.19	2.40	1.77	2.14	2.17	1.72	1.79	1.50	0.93	1.72	0.98
17.12	2.43	2.01	1.90	0.78	1.37	1.76	1.19	1.19	0.32	1.25	0.62

17.45	3.26	3.51	2.79	2.76	2.69	2.78	2.30	2.37	2.09	2.07	1.57
17.81	4.60	3.16	3.46	2.33	2.79	1.92	3.44	1.79	1.42	2.16	1.74
18.19	3.62	3.15	3.19	2.85	3.23	2.94	2.89	3.02	2.73	1.97	1.45
18.60	2.15	1.63	1.65	2.82	0.86	1.26	1.07	1.90	1.85	1.19	0.94
19.05	2.40	2.70	2.35	1.19	1.34	2.38	2.18	1.92	1.92	0.79	1.80
19.53	3.31	2.76	2.53	1.62	1.57	2.08	2.61	1.81	1.74	1.22	1.13
20.05	3.06	3.29	3.15	2.48	2.90	1.98	2.95	2.52	2.04	1.93	1.95
20.61	3.41	3.20	3.20	2.88	3.03	2.77	2.66	2.34	1.86	1.73	1.47
21.22	2.71	3.12	1.99	2.01	2.16	1.81	2.26	1.59	1.97	1.47	1.61
21.87	2.34	0.60	0.87	1.65	1.40	1.63	1.69	1.13	1.18	0.64	1.04
22.57	1.97	2.41	2.04	2.09	1.93	1.23	2.27	1.89	2.31	1.38	0.51
23.33	3.01	2.73	2.43	2.14	2.52	2.61	2.46	2.03	1.25	1.71	1.56
24.15	4.09	3.58	3.08	2.45	2.19	2.72	2.63	2.37	2.20	1.98	2.10
25.03	3.16	3.03	2.61	3.27	2.85	1.56	2.44	2.41	2.15	1.98	1.49
25.98	1.94	2.22	1.53	2.64	1.07	1.25	2.29	2.02	1.83	1.23	1.38
27.01	1.85	4.10	2.03	2.15	2.09	1.96	2.00	1.76	1.92	0.99	1.92
28.11	2.40	1.40	1.34	1.47	1.00	1.64	1.99	1.96	1.28	1.77	0.44
29.3	4.09	3.64	3.59	3.11	2.60	2.73	3.05	2.40	2.45	2.50	2.02
30.59	3.35	3.12	2.69	3.09	2.64	2.75	2.49	2.29	1.77	2.32	1.61
31.98	2.35	1.98	2.47	1.92	2.40	1.81	1.84	1.63	1.77	1.18	0.84
33.47	3.36	2.98	2.91	2.42	3.14	2.28	2.31	2.14	1.69	1.24	1.78
35.09	2.93	2.55	2.14	1.69	1.53	1.74	2.06	1.44	1.86	1.49	1.25
36.83	3.04	1.74	1.95	1.71	1.57	2.21	1.45	2.00	1.48	1.66	0.77
38.71	3.20	3.52	3.04	3.30	3.05	2.88	3.29	2.42	2.70	1.91	1.55
40.73	3.78	3.51	3.01	2.63	3.40	2.22	2.02	1.68	1.77	1.86	1.45
42.92	2.25	1.85	2.13	2.30	2.81	2.42	2.66	2.60	1.77	1.78	1.14
45.27	2.24	2.10	1.25	2.48	1.29	1.55	2.01	1.84	1.50	0.87	0.54
47.81	3.12	2.95	2.47	1.18	3.11	2.12	2.19	2.42	2.58	2.37	2.06
50.55	3.61	3.84	2.71	2.20	3.01	2.80	2.70	2.88	2.27	1.68	1.20
53.51	4.07	4.03	4.99	4.07	3.56	3.64	3.93	3.66	3.48	3.00	2.24
56.69	3.35	3.34	2.68	2.68	2.50	2.31	2.54	2.69	2.44	2.15	1.30
60.13	2.56	2.35	1.79	1.85	2.43	1.95	1.98	1.93	1.87	0.81	1.06
63.84	2.81	2.12	2.27	1.61	1.57	1.73	2.00	1.74	1.58	1.63	1.29
67.83	2.80	2.88	2.79	2.32	2.95	1.65	2.10	2.41	2.08	1.93	1.39
72.15	3.33	4.63	3.98	2.17	3.27	2.50	2.10	3.15	2.74	2.48	1.80
76.80	3.43	3.15	2.84	2.96	2.91	2.70	3.09	2.53	2.17	1.95	1.74
81.81	2.27	2.75	2.01	2.16	1.90	1.71	1.59	2.21	1.21	1.98	1.14
87.22	1.24	2.04	1.61	1.02	1.42	1.49	2.40	0.77	0.75	1.01	1.02
93.06	2.61	3.15	2.18	2.23	2.45	3.27	2.31	2.31	2.06	1.78	1.07
99.35	2.54	3.53	3.67	2.65	2.11	2.67	2.33	2.79	1.43	1.79	1.41
106.13	3.45	3.65	3.88	4.06	2.64	3.30	2.66	2.51	2.23	2.22	1.88
113.45	3.01	4.50	2.91	3.02	2.74	2.71	2.20	2.16	2.10	1.46	0.75
121.34	2.52	1.88	1.84	2.10	1.94	1.91	2.15	1.76	1.65	0.72	0.68
129.86	2.92	2.61	2.95	2.75	3.32	2.24	2.69	2.84	2.42	1.31	1.39
139.04	3.04	2.38	2.25	1.60	3.49	2.28	2.91	2.16	1.73	2.16	1.14
148.94	4.02	3.83	3.92	3.27	3.04	3.74	2.56	2.91	2.50	2.11	1.54
159.62	3.55	3.44	2.64	2.29	2.05	2.51	2.86	2.10	2.41	1.98	1.40
171.14	2.42	3.53	1.87	2.04	2.16	2.88	2.04	1.83	1.53	0.97	0.72
183.56	2.89	2.60	2.38	2.20	2.19	1.86	1.59	1.73	1.69	0.88	1.80
196.96	2.35	2.85	2.23	1.85	1.54	1.91	2.19	2.29	1.64	1.86	0.80

211.41	2.56	3.13	2.96	3.42	2.97	2.52	2.70	2.35	2.10	1.71	1.42
226.99	3.90	4.40	3.82	3.44	2.63	3.33	3.23	2.52	1.80	2.21	1.65
243.80	2.72	2.95	3.42	2.90	3.17	2.65	2.26	2.26	1.30	1.89	1.53
261.93	3.97	2.40	2.55	2.52	1.76	3.61	1.79	2.09	2.04	2.24	1.72
281.48	2.55	1.40	1.48	2.06	1.89	2.07	2.26	1.32	1.33	1.09	0.79
302.56	4.12	4.75	2.91	3.26	2.34	3.60	2.38	3.31	2.24	1.73	1.54
325.3	4.62	4.20	4.04	3.31	4.06	3.48	3.77	2.67	3.03	2.02	2.08
349.83	3.81	3.79	3.65	3.27	3.90	3.04	2.78	2.77	2.45	1.80	1.67
376.28	2.93	3.37	2.48	3.13	2.99	2.20	2.56	2.05	2.06	1.53	1.26
404.81	3.22	3.30	2.70	2.62	1.95	1.56	1.67	1.83	1.83	0.98	1.48
435.58	1.88	3.28	2.50	2.13	2.84	2.10	2.62	2.29	2.07	1.88	1.44
468.76	3.65	3.37	3.21	2.60	2.63	3.22	2.92	2.36	2.44	2.20	1.62
504.55	4.73	5.12	4.19	3.94	3.79	3.67	3.14	3.22	2.75	2.15	1.78
543.15	4.46	4.43	4.41	4.34	4.16	3.81	3.03	3.82	2.63	1.65	1.89
584.78	3.33	2.37	2.85	2.43	2.44	1.92	2.28	1.76	1.69	1.50	1.29
629.68	2.37	3.55	3.21	2.03	2.67	1.88	2.10	2.29	1.60	1.61	1.40
678.10	3.05	1.39	2.86	0.44	2.07	1.52	1.69	1.26	1.01	0.93	0.21
730.33	5.15	4.46	4.22	3.96	3.47	2.91	3.79	3.40	2.61	2.01	1.54
786.65	4.02	4.99	3.42	3.65	3.02	3.25	2.46	2.94	2.59	2.23	1.52
847.40	3.87	3.17	3.12	3.36	2.93	2.94	2.70	2.99	2.37	2.20	1.73
912.92	3.24	3.34	2.34	2.61	3.27	2.61	2.04	2.04	1.52	1.28	0.74
983.58	2.93	2.65	2.97	2.40	2.70	2.97	2.90	2.01	1.99	1.53	1.07
1059.78	2.95	1.98	2.97	2.21	2.18	1.41	2.72	1.31	1.68	0.94	0.99
1141.98	5.12	3.24	3.73	2.94	3.39	2.45	3.48	1.97	1.82	1.30	0.99
1230.62	3.78	3.50	4.12	2.92	3.35	2.74	2.65	2.68	2.40	1.23	1.78
1326.23	3.81	2.91	3.23	2.42	2.47	2.36	2.17	2.05	1.74	0.70	0.60
1429.34	3.36	2.58	2.45	2.96	1.79	0.92	2.54	1.15	1.35	0.49	1.51
1540.54	2.82	2.95	2.71	2.14	1.62	1.82	2.20	2.27	1.72	1.44	0.90
1660.48	5.21	3.75	3.82	4.22	4.35	2.78	2.74	2.89	2.16	1.99	1.45
1789.83	5.79	4.06	4.63	4.61	3.94	3.57	3.89	2.79	2.52	2.04	1.97
1929.34	4.28	3.99	3.28	3.08	3.23	2.39	2.78	2.52	1.73	1.68	1.74
2079.80	1.96	3.00	2.27	3.23	2.67	1.98	2.07	1.79	1.34	0.86	0.24
2242.08	2.94	3.04	3.15	2.63	2.26	2.17	2.71	2.03	1.44	1.16	0.66
2417.10	4.27	4.25	3.73	3.47	3.00	2.64	2.60	2.69	1.92	1.15	1.50
2605.85	4.17	4.38	4.26	3.92	3.54	2.57	2.74	3.02	1.65	1.84	1.29
2809.43	4.28	3.00	4.42	3.05	3.40	3.48	3.18	2.17	2.38	2.22	1.14
3028.99	4.29	2.47	3.05	3.30	3.49	1.42	3.47	2.46	1.71	1.57	1.11
3265.79	2.44	3.53	3.31	1.95	2.01	2.71	2.40	2.71	2.26	1.75	0.96
3521.18	3.95	3.24	3.56	2.63	2.90	2.47	2.76	2.34	2.05	1.17	1.48
3796.62	5.17	4.07	4.13	3.54	4.19	3.40	3.54	3.52	3.15	1.79	1.90

Wavelength (nm) / Time (ps)	336.20	337.61	339.02	340.42	341.83	343.24	344.65	346.06	347.47	348.87	350.28
-3.28	0.22	-0.09	0.13	0.07	0.19	0.04	0.02	-0.12	0.16	-0.12	-0.03
-2.78	-0.26	0.11	-0.13	-0.11	-0.43	-0.14	-0.06	0.42	0.08	-0.16	0.29
-2.28	0.17	-0.11	-0.06	0.16	0.37	0.28	0.28	-0.14	-0.06	0.20	0.09
-1.78	-0.10	-0.33	0.02	-0.11	-0.05	-0.36	0.18	-0.10	-0.37	-0.07	0.01
-1.28	0.50	0.87	0.93	0.45	0.24	0.36	0.52	0.26	0.60	0.56	0.16
-0.78	-0.05	-0.42	-0.52	0.14	-0.21	-0.17	0.03	0.05	0.11	0.11	0.04
-0.28	-0.26	0.12	0.12	-0.02	-0.01	0.07	-0.62	-0.12	-0.30	0.00	-0.11

0.22	-0.22	-0.14	-0.50	-0.57	-0.09	-0.07	-0.36	-0.26	-0.22	-0.53	-0.46
0.32	-0.23	-0.11	0.10	0.35	-0.07	0.10	0.16	0.48	-0.06	-0.26	0.26
0.42	0.50	0.16	0.21	-0.21	0.34	0.67	-0.26	0.09	0.37	0.10	-0.18
0.52	-7.08	-5.27	-2.47	0.13	0.73	1.22	0.59	0.69	0.51	0.32	-0.03
0.62	-0.16	-1.37	-1.41	-3.13	-4.07	-5.50	-6.36	-6.40	-4.49	-2.90	-1.50
0.72	-0.18	0.01	-0.23	-0.03	-0.09	-0.39	-0.89	-1.71	-1.90	-1.98	-2.04
0.77	-0.22	0.13	0.54	0.39	0.32	0.27	-0.48	-0.09	-0.63	-0.54	-1.31
0.82	0.22	0.01	-0.19	0.02	-0.10	-0.35	-0.47	-0.05	-0.01	-0.15	-0.68
0.87	-0.02	-0.06	-0.57	-0.57	-0.84	-0.71	-1.15	-1.19	-0.94	-1.04	-1.14
0.92	0.17	0.22	0.30	-0.36	-0.13	-0.83	-0.80	-0.89	-0.91	-1.18	-1.09
0.97	0.48	0.14	-0.18	-0.45	-0.13	-0.41	-0.93	-1.10	-0.76	-0.89	-0.85
1.02	0.36	-0.03	-0.33	-0.10	-0.25	-0.36	-0.46	-0.50	-0.48	-0.99	-0.72
1.07	0.12	-0.10	-0.35	-0.57	-0.72	-0.06	-0.39	-0.58	-0.51	-0.72	-0.16
1.12	0.01	-0.07	0.40	-0.28	-0.20	-0.27	-0.15	-0.25	-0.18	-0.16	-0.50
1.17	0.29	0.01	0.36	-0.28	-0.18	-0.44	-0.71	-0.47	-0.68	-0.53	-0.89
1.22	0.40	0.20	-0.07	-0.02	-0.14	-0.42	-0.67	-0.86	-0.89	-1.02	-1.24
1.27	0.75	0.12	0.27	0.38	0.17	-0.40	-0.20	-0.32	-0.05	-0.81	-0.73
1.32	0.87	0.48	0.75	-0.07	-0.01	-0.28	-0.10	0.04	-0.15	-0.40	-0.05
1.37	0.00	0.17	-0.43	0.31	-0.17	-0.17	-1.03	-0.28	-0.76	-0.19	-1.02
1.42	0.30	0.57	0.44	0.04	0.02	-0.51	-0.37	-0.78	-0.57	-0.69	-0.76
1.47	0.09	0.13	0.08	-0.52	-0.22	-0.90	-0.76	-1.23	-0.84	-1.17	-1.13
1.52	-0.15	0.35	-0.11	-0.59	-0.80	-0.89	-1.09	-1.19	-1.09	-1.06	-1.41
1.57	0.66	0.93	0.03	0.42	0.21	-0.04	-0.41	-0.46	-0.27	-0.34	-0.48
1.62	0.18	-0.06	0.06	0.45	-0.12	-0.39	0.16	0.01	0.11	0.15	0.03
1.67	0.38	0.34	0.53	-0.27	0.29	-0.08	-0.74	0.04	0.04	-0.70	-0.46
1.72	1.19	0.66	0.67	0.30	0.24	-0.21	-0.25	-0.36	-0.52	-0.33	-0.55
1.77	0.72	0.69	0.16	0.09	-0.17	-0.29	-0.68	-0.26	-0.69	-0.72	-0.53
1.82	0.03	0.37	0.10	-0.05	-0.03	-0.20	-0.54	-0.72	-0.66	-1.04	-1.12
1.87	0.42	0.39	0.45	0.47	0.18	0.07	-0.66	-0.31	-0.21	-0.36	-0.49
1.92	0.76	0.10	0.21	-0.01	0.42	0.00	-0.57	-0.09	-0.22	-0.57	-0.40
1.97	0.90	0.94	0.57	0.17	0.71	0.22	-0.11	-0.43	-0.15	-0.25	-0.54
2.02	0.82	0.19	0.26	0.00	0.03	0.24	-0.58	-0.33	-0.65	-0.51	-0.83
2.07	1.07	0.82	0.47	0.28	0.47	-0.02	-0.31	-0.57	-0.42	-0.95	-0.90
2.12	0.93	0.54	0.40	0.52	-0.22	-0.16	0.07	-0.18	-0.52	-0.22	-0.47
2.17	0.16	0.47	-0.12	0.01	-0.11	0.31	-0.40	-0.84	-0.59	-0.28	0.14
2.22	0.89	0.52	0.61	0.37	0.36	-0.09	-0.01	-0.21	-0.27	-0.35	-0.02
2.27	0.36	0.00	0.30	-0.22	-0.27	-0.67	-0.83	-0.83	-0.80	-0.98	-1.34
2.32	1.65	1.04	0.58	0.43	0.29	0.15	-0.48	-0.34	-0.35	-0.54	-0.48
2.37	0.71	0.84	0.21	0.46	0.05	0.04	-0.54	-0.43	-0.49	-0.40	-0.74
2.42	0.03	0.43	0.01	-0.11	-0.04	-0.07	-0.35	-0.45	-0.67	-0.65	-0.66
2.47	-0.12	0.42	0.05	0.30	-0.45	-0.11	-0.28	-0.28	-0.25	-0.16	-0.37
2.52	0.45	0.95	0.15	-0.08	0.37	0.01	-0.11	-0.80	-0.21	-0.14	-0.56
2.57	0.86	0.27	0.80	0.73	0.14	0.00	-0.19	-0.59	-0.45	-0.44	-0.72
2.62	0.76	0.43	-0.03	0.13	0.33	-0.51	-0.42	-0.62	-0.39	-0.97	-1.03
2.67	0.20	0.55	-0.63	-0.42	-0.01	-0.68	-0.35	-0.36	-0.47	-0.60	-0.69
2.72	0.28	0.43	0.17	-0.38	-0.07	-0.21	-0.30	-0.39	-0.18	-0.11	-0.20
2.77	1.43	0.48	0.61	0.15	0.55	0.07	0.40	0.08	0.29	0.02	-0.19
2.82	0.38	0.25	0.54	-0.14	0.19	-0.75	-0.44	-0.86	-0.89	-0.87	-0.68
2.87	0.85	0.56	0.56	0.25	0.17	-0.20	-0.32	-0.51	-0.21	-0.62	-0.79
2.92	0.66	0.27	0.27	0.11	0.21	-0.31	-0.56	-1.01	-0.99	-1.15	-1.12

2.97	0.99	0.82	1.51	0.30	0.08	0.53	0.43	0.47	0.37	0.43	0.07
3.02	0.31	0.43	0.32	0.16	0.41	0.29	0.11	-0.24	-0.08	-0.35	0.08
3.07	1.22	0.30	0.36	0.03	0.16	0.30	-0.53	-0.39	-0.44	-0.62	-0.46
3.12	0.52	0.03	0.13	0.21	-0.03	-0.19	-0.55	-0.47	-0.68	-0.63	-0.85
3.17	1.40	0.72	0.92	0.20	0.43	0.32	-0.29	-0.35	-0.20	-0.58	-0.88
3.22	0.56	0.12	0.48	-0.18	-0.40	-0.48	-0.31	-0.61	-0.78	-0.39	-0.77
3.27	0.60	-0.11	-0.21	0.88	-0.14	-0.47	-0.69	0.21	-0.57	-0.46	0.14
3.32	0.67	-0.10	0.16	0.26	-0.02	-1.02	-0.86	-0.61	-0.40	-0.54	-0.43
3.37	0.68	1.01	0.83	0.07	0.43	-0.26	-0.06	-0.27	-0.44	-0.40	-0.73
3.42	0.87	0.50	0.68	0.46	0.41	-0.20	-0.22	-0.43	-0.53	-0.81	-1.28
3.47	-0.16	0.14	0.05	-0.72	-0.38	-0.59	-0.92	-0.67	-1.05	-1.07	-1.30
3.52	0.04	1.26	0.39	0.08	0.28	-0.18	-0.39	-0.20	-0.37	-0.03	-0.99
3.57	-0.31	0.76	0.08	0.03	0.11	-0.21	-0.53	-0.47	-0.44	-0.32	-0.20
3.62	0.82	1.25	0.95	0.51	0.49	0.30	-0.10	-0.02	0.09	-0.53	-0.31
3.67	1.09	0.84	0.51	0.59	0.18	-0.02	-0.02	-0.03	-0.48	-0.51	-0.76
3.72	1.00	0.68	0.69	0.38	0.37	-0.25	-0.48	-0.64	-0.36	-0.81	-0.89
3.92	0.76	0.84	0.77	0.65	0.01	-0.15	0.03	-0.36	-0.29	-0.35	-0.53
4.12	0.51	0.67	0.25	0.13	-0.27	-0.24	-0.26	-0.08	0.04	-0.19	-0.53
4.32	0.22	0.63	0.40	0.09	-0.18	-0.10	0.07	-0.63	-0.26	-0.47	-0.73
4.52	0.32	0.42	1.09	0.28	0.62	-0.30	-0.51	-0.35	-0.20	-0.63	-0.57
4.72	1.19	1.07	0.73	0.31	0.72	0.14	-0.03	-0.13	-0.43	-0.61	-0.61
4.92	0.66	0.47	0.42	0.07	0.05	-0.63	-0.52	-0.38	-0.37	-0.52	-1.02
5.12	0.86	0.03	0.57	0.06	0.55	0.10	-0.18	-0.41	-0.11	-0.39	-0.24
5.32	0.46	1.09	0.67	0.93	0.17	-0.02	-0.15	-0.15	0.03	0.15	-0.38
5.52	0.01	0.37	0.43	0.08	-0.13	0.06	-0.18	-0.42	-0.55	-0.50	-0.57
5.72	1.10	1.31	1.04	0.42	0.55	0.34	-0.22	0.10	-0.01	-0.57	-0.54
5.92	0.23	0.33	0.86	0.19	0.19	0.11	-0.15	-0.49	-0.97	-0.54	-0.78
6.12	0.94	0.72	0.78	0.03	-0.05	-0.02	-0.23	-0.42	-0.19	-0.64	-0.87
6.32	0.52	0.71	0.42	0.39	-0.01	0.06	0.25	-0.16	-0.34	-0.15	-0.34
6.52	0.67	0.00	0.23	0.52	-0.24	-0.69	-0.37	-0.40	-0.81	-0.74	-0.31
6.72	1.47	0.71	0.93	0.97	0.73	0.21	0.29	-0.19	0.03	-0.24	-0.47
6.92	1.09	1.19	0.43	0.35	0.65	-0.15	-0.40	-0.32	-0.41	-0.55	-0.62
7.12	0.85	0.52	0.31	0.15	-0.14	-0.43	-0.58	-0.56	-0.75	-0.94	-1.21
7.32	0.98	0.75	1.07	0.61	0.59	0.43	-0.17	-0.22	-0.23	-0.43	-0.42
7.52	0.54	0.05	-0.10	-0.41	-0.05	-0.79	-0.64	-0.38	-0.38	-0.72	-0.53
7.72	1.29	0.93	0.89	0.64	0.51	0.44	0.38	0.30	-0.21	0.08	0.00
7.92	0.81	1.05	0.65	0.48	0.52	-0.14	-0.75	0.14	-0.30	-0.48	-0.83
8.12	0.96	0.70	0.54	0.48	0.21	-0.31	-0.87	-0.53	-0.84	-0.93	-1.08
8.32	1.00	0.60	0.48	0.31	0.40	-0.27	-0.59	-0.63	-0.95	-1.08	-1.19
8.52	1.33	0.67	0.53	0.63	0.85	0.00	0.19	0.09	-0.15	-0.49	-0.64
8.72	0.24	1.10	0.35	-0.05	0.25	-0.28	-0.27	0.39	-0.62	-0.55	-0.37
8.92	0.77	0.82	0.80	0.23	0.41	-0.21	-0.22	-0.21	-0.32	-0.32	-0.51
9.12	1.38	1.05	0.54	0.07	0.57	0.01	-0.47	-0.43	-0.19	-0.60	-0.89
9.32	1.63	1.18	1.04	0.90	0.68	0.37	0.13	-0.34	-0.25	-0.21	-0.57
9.52	1.25	0.55	0.94	0.70	0.37	0.28	0.03	-0.12	-0.44	-0.38	-0.63
9.72	0.51	0.52	0.81	0.79	0.50	-0.14	-0.27	0.34	0.27	-0.09	-0.24
9.92	0.87	1.69	0.85	0.99	0.71	0.63	0.22	0.35	0.39	0.01	0.07
10.12	0.89	0.91	0.19	0.65	0.61	-0.08	-0.23	-0.15	-0.59	-0.68	-0.61
10.32	1.28	0.98	0.85	0.47	0.48	-0.06	-0.40	-0.47	-0.81	-0.75	-0.64
10.52	0.86	0.65	0.25	0.46	-0.28	-0.28	-0.58	-0.25	-0.64	-0.99	-0.93

10.72	0.98	0.67	0.56	0.53	0.24	0.26	0.00	0.32	0.10	-0.01	-0.43
10.92	0.39	0.88	0.18	0.65	0.28	-0.04	-0.22	0.07	-0.10	-0.28	0.10
11.12	0.87	1.14	0.82	0.56	0.19	0.33	0.13	-0.16	-0.37	-0.14	-0.43
11.32	1.70	0.37	0.78	0.36	0.17	0.01	-0.07	-0.36	-0.72	-0.48	-0.47
11.52	0.35	0.93	0.47	0.46	0.12	-0.43	-0.27	-0.48	-0.59	-1.17	-0.99
11.72	0.69	-0.02	0.34	0.09	0.07	-0.14	-0.65	-0.76	-0.79	-0.59	-1.03
11.92	0.06	-0.33	0.11	-0.37	-0.03	-0.47	-0.79	-0.76	-0.60	-0.94	-0.78
12.12	-0.32	-0.15	-0.05	-0.30	-0.20	-0.32	-0.63	-0.46	-0.20	-0.62	-0.73
12.32	0.86	0.73	0.36	0.35	0.22	-0.07	-0.24	-0.29	-0.59	-0.81	-0.98
12.52	1.36	0.60	0.72	0.58	0.61	-0.14	-0.38	-0.34	-0.25	-0.71	-0.61
12.72	1.42	0.67	0.64	0.43	0.14	0.23	-0.48	-0.20	-0.54	-0.92	-0.87
12.92	0.79	0.95	0.03	0.72	0.42	-0.04	-0.07	-0.51	-0.37	-0.54	-0.25
13.12	0.33	0.44	0.92	0.33	0.06	0.41	-0.01	0.08	0.18	-0.36	-0.21
13.32	1.58	2.03	1.43	0.83	1.46	0.44	0.25	0.26	-0.11	-0.03	-0.41
13.52	1.20	0.96	1.01	0.75	0.83	0.05	0.03	0.01	-0.31	-0.43	-0.27
13.72	1.71	1.48	0.89	0.60	0.74	0.57	0.26	-0.09	-0.16	-0.58	-0.60
13.92	1.21	0.79	1.17	0.40	0.44	-0.09	-0.73	-0.21	-0.23	-0.70	-0.47
14	0.49	0.04	-0.23	0.74	0.26	-0.42	-0.42	-0.56	-0.20	-0.36	-0.65
14.08	0.39	0.51	0.19	0.10	-0.04	-0.20	-0.39	-0.37	-0.65	-0.75	-0.59
14.17	0.46	0.44	0.11	0.03	-0.30	-0.11	-0.30	-0.58	-0.51	-0.86	-0.71
14.27	0.91	0.41	0.71	0.83	0.41	-0.01	-0.24	-0.55	-0.44	-0.70	-0.78
14.38	1.01	1.03	0.73	0.67	0.31	-0.01	-0.68	-0.92	-0.37	-0.84	-1.13
14.49	0.76	0.98	0.46	0.05	0.22	-0.04	-0.38	-0.47	-0.50	-0.56	-0.61
14.61	1.04	1.29	0.57	-0.04	0.39	0.45	0.18	-0.08	-0.18	-0.17	-0.54
14.75	0.93	1.23	0.23	0.55	0.33	0.11	-0.24	-0.11	0.02	-0.38	-0.36
14.89	0.60	0.84	0.85	0.68	0.32	-0.34	-0.50	-0.05	-0.52	-0.82	-0.95
15.05	1.28	1.47	0.98	0.26	0.54	0.25	-0.12	-0.24	-0.38	-0.69	-1.21
15.21	0.91	1.17	0.60	0.44	-0.30	-0.23	-0.35	-0.46	-0.47	-0.82	-0.75
15.39	1.00	0.69	0.97	0.45	0.60	0.17	-0.08	-0.09	-0.31	0.12	-0.64
15.59	0.74	0.49	0.11	0.45	-0.28	-0.25	-0.08	0.12	-0.39	-0.32	-0.27
15.8	0.88	0.90	0.76	0.41	0.87	0.13	0.01	-0.05	-0.12	-0.50	-0.55
16.02	1.15	1.14	0.56	0.51	0.17	0.05	-0.74	-0.26	-0.31	-0.64	-0.76
16.27	1.48	0.95	0.68	0.44	0.43	0.02	-0.20	-0.48	-0.74	-0.99	-1.12
16.53	0.50	0.78	0.44	0.31	0.02	-0.28	-0.73	-0.72	-0.62	-0.74	-0.84
16.81	0.74	0.44	0.54	-0.04	0.32	0.19	0.48	0.03	0.07	0.01	-0.08
17.12	-0.25	0.50	0.69	0.29	0.81	-0.13	-0.38	-0.13	-0.67	-0.64	-0.54
17.45	1.04	1.20	1.07	0.65	0.37	-0.04	-0.08	-0.37	-0.19	-0.40	-0.59
17.81	1.17	0.97	0.78	0.33	-0.01	0.03	-0.21	-0.32	-0.37	-0.88	-0.42
18.19	1.01	0.81	1.07	0.44	0.49	0.38	-0.09	0.52	-0.16	-0.37	-0.33
18.6	0.38	0.59	0.56	0.38	0.40	0.21	0.17	-0.49	-0.08	-0.19	-0.50
19.05	1.23	1.41	1.08	1.05	0.28	0.49	0.61	0.40	0.22	0.36	0.42
19.53	0.77	1.15	0.49	0.38	0.14	0.03	0.01	-0.39	-0.35	-0.74	-0.57
20.05	1.26	0.85	1.08	0.45	0.64	0.04	-0.45	-0.29	-0.22	-0.68	-0.91
20.61	1.04	1.01	0.57	0.54	0.27	0.08	-0.36	-0.58	-0.68	-0.64	-0.28
21.22	0.88	1.07	0.67	0.51	0.63	-0.02	-0.24	-0.13	-0.23	-0.36	-0.78
21.87	0.69	0.42	0.38	-0.18	0.18	-0.22	0.18	0.04	0.08	0.06	-0.83
22.57	0.73	1.28	0.76	0.93	0.16	0.41	-0.12	-0.06	-0.33	-0.32	-0.03
23.33	0.99	0.56	0.64	0.57	0.57	0.10	-0.53	-0.46	-0.54	-0.80	-0.65
24.15	1.59	0.99	0.74	0.55	0.11	-0.26	-0.14	-0.50	-0.48	-0.73	-0.80
25.03	0.93	0.78	0.46	0.46	0.39	0.54	-0.07	-0.53	-0.42	-0.64	-0.74

25.98	1.04	0.80	0.48	-0.10	0.27	0.41	-0.23	-0.24	-0.29	-0.22	-0.42
27.01	1.51	0.98	0.83	0.99	0.51	0.47	0.26	0.05	0.39	0.21	-0.04
28.11	0.48	0.78	0.66	0.60	-0.02	0.14	-0.16	-0.60	-0.41	-0.68	-0.66
29.3	1.52	1.35	0.91	0.60	0.42	0.25	-0.03	0.11	0.22	-0.27	-0.40
30.59	1.46	1.13	1.15	0.67	0.60	0.38	-0.16	0.16	-0.03	-0.66	-0.91
31.98	0.59	0.72	0.85	0.14	0.09	0.24	-0.36	-0.67	-0.93	-0.67	-0.75
33.47	1.54	0.86	0.16	0.61	0.71	0.14	-0.01	-0.68	-0.13	-0.74	-0.30
35.09	0.88	1.04	1.02	-0.06	0.62	0.01	-0.23	-0.05	-0.40	-0.62	-0.47
36.83	0.90	0.76	0.58	0.18	0.07	-0.61	-0.26	-0.38	-0.31	-0.54	-0.38
38.71	1.20	1.27	0.96	0.92	0.36	-0.02	0.10	0.11	0.02	-0.33	-0.34
40.73	1.41	0.85	0.72	0.72	0.38	0.27	-0.21	-0.06	-0.30	-0.62	-0.91
42.92	1.36	0.98	0.46	0.23	-0.02	0.42	-0.10	-0.43	-0.06	-0.53	-0.67
45.27	1.11	0.73	0.73	0.49	0.50	-0.06	0.11	-0.26	-0.36	-0.70	-0.22
47.81	1.30	1.45	1.15	1.03	1.04	0.81	0.38	-0.53	-0.04	-0.09	0.01
50.55	0.94	0.95	0.92	0.54	0.91	0.12	-0.37	-0.54	-0.79	-0.62	-0.84
53.51	2.29	1.84	1.54	0.82	1.15	0.56	0.44	-0.02	0.65	0.04	-0.06
56.69	1.15	1.26	1.02	0.92	0.83	0.44	-0.35	0.19	-0.14	-0.32	-0.68
60.13	1.00	1.24	1.10	0.16	0.63	0.78	-0.32	-0.11	0.27	-0.29	-0.15
63.84	0.89	0.81	0.67	0.68	0.43	0.01	-0.01	0.12	0.01	-0.49	-0.58
67.83	1.62	1.41	1.25	0.67	0.71	0.00	-0.15	-0.30	-0.55	-0.23	-0.35
72.15	1.36	1.23	1.06	0.90	0.71	0.47	-0.29	-0.16	-0.33	-0.51	-0.47
76.8	0.94	1.15	0.65	0.65	0.46	0.22	-0.49	-0.07	-0.16	-0.46	-0.71
81.81	1.10	0.65	0.32	0.12	0.46	-0.39	-0.21	-0.24	-0.33	-0.53	-0.95
87.22	0.13	0.50	0.35	0.44	-0.51	0.24	0.08	-0.12	-0.52	-0.40	-0.58
93.06	1.24	0.44	0.80	0.77	0.59	0.61	-0.27	-0.04	0.19	-0.23	-0.16
99.35	1.04	1.07	1.00	0.26	-0.28	0.32	0.04	-0.43	-0.52	-0.92	-0.83
106.13	1.10	1.27	1.03	0.64	0.41	0.59	0.06	0.12	-0.18	-0.40	-0.77
113.45	0.70	0.67	0.61	0.40	-0.20	-0.03	-0.64	-0.15	-0.45	-1.17	-0.83
121.34	0.65	0.48	0.11	0.46	0.29	0.07	-0.34	-0.32	-0.71	-0.36	-0.66
129.86	0.87	1.40	0.90	0.59	0.73	0.53	0.31	0.33	0.31	-0.37	0.18
139.04	1.01	0.45	-0.12	0.68	0.46	0.22	0.03	-0.13	-0.53	-0.18	-0.57
148.94	1.45	1.21	1.02	0.22	0.85	0.42	-0.47	-0.41	-0.48	-0.62	-0.78
159.62	0.85	0.99	0.54	0.22	-0.03	-0.16	-0.43	-0.36	-0.55	-0.86	-0.99
171.14	0.49	0.59	0.53	-0.41	0.11	-0.26	-0.49	-0.50	-0.47	-0.76	-0.80
183.56	0.79	0.65	0.74	0.45	0.37	0.08	0.18	0.06	-0.39	-0.11	-0.69
196.96	0.93	0.94	0.58	0.03	0.52	-0.46	-0.35	-0.23	-0.66	-0.59	-0.82
211.41	0.68	0.93	0.19	0.23	0.43	0.01	-0.32	-0.41	-0.38	-0.84	-0.73
226.99	0.76	1.27	0.60	0.20	0.10	0.00	-0.55	-0.62	-0.41	-0.94	-0.97
243.8	0.66	0.61	0.94	0.63	0.12	-0.29	0.07	-0.25	-0.37	-0.81	-0.90
261.93	1.34	1.02	0.88	0.51	0.41	0.36	-0.53	-0.35	0.23	-0.06	-0.63
281.48	0.97	0.39	0.39	-0.07	0.56	0.29	-0.15	0.02	-0.15	-0.44	-0.97
302.56	1.28	0.93	1.11	0.63	0.06	-0.09	0.30	-0.22	-0.45	-0.50	-0.50
325.3	1.00	1.31	0.55	0.95	0.36	0.15	-0.50	-0.15	-0.29	-0.43	-0.66
349.83	1.15	0.57	0.50	0.08	-0.29	-0.14	-0.20	-0.34	-0.66	-0.69	-0.90
376.28	1.33	0.66	0.30	0.32	0.28	-0.20	-0.62	-0.35	-0.57	-0.47	-0.84
404.81	0.59	0.00	-0.13	0.29	0.11	-0.10	-0.60	-0.70	-0.45	-0.71	-0.68
435.58	0.95	0.68	0.19	0.88	0.74	0.61	-0.35	-0.15	-0.24	-0.17	-0.45
468.76	0.98	1.12	0.43	0.40	0.26	-0.04	-0.16	-0.56	-0.44	-0.60	-0.84
504.55	1.37	1.37	0.63	0.48	0.40	0.14	-0.10	-0.54	-0.81	-0.86	-0.86
543.15	2.05	0.78	1.26	0.72	0.52	0.30	-0.31	-0.27	-0.45	-0.69	-1.03

584.78	0.45	0.19	0.53	-0.32	0.16	-0.41	-0.74	-0.65	-0.96	-1.02	-1.18
629.68	0.73	0.70	0.51	0.34	-0.33	-0.06	-0.41	-0.41	-0.25	-0.81	-1.12
678.1	-0.05	0.07	0.33	-0.16	-0.71	-0.89	-0.60	-0.94	-0.66	-1.53	-1.60
730.33	0.88	1.10	0.24	0.23	-0.12	-0.15	-0.57	-0.60	-0.97	-0.94	-1.20
786.65	1.38	1.04	0.49	-0.08	0.14	-0.52	-0.68	-0.73	-0.74	-0.98	-0.91
847.4	0.83	0.36	0.62	0.39	-0.21	-0.16	-0.80	-0.87	-0.64	-0.97	-1.15
912.92	0.12	0.39	0.36	-0.04	0.27	-0.21	-0.57	-0.41	-0.49	-0.60	-0.64
983.58	0.82	0.85	0.31	0.08	-0.17	-0.35	-0.03	-0.44	-0.60	-0.51	-1.01
1059.78	-0.02	0.07	-0.20	-0.35	-0.42	-0.64	-1.07	-1.14	-1.17	-1.31	-1.28
1141.98	0.48	0.91	0.58	0.16	-0.62	-0.48	-0.65	-0.77	-0.89	-1.21	-1.03
1230.62	0.27	-0.05	0.22	-0.21	-0.31	-0.66	-1.01	-0.92	-1.45	-1.36	-1.19
1326.23	-0.08	0.73	-0.42	-0.44	-0.09	-0.97	-1.10	-1.24	-1.02	-1.27	-1.26
1429.34	0.77	0.43	0.16	0.14	0.02	-0.07	-0.57	-0.81	-0.84	-0.71	-0.71
1540.54	0.22	0.31	0.57	-0.13	-0.09	-0.67	-1.05	-0.93	-1.11	-0.98	-1.01
1660.48	1.75	0.51	0.50	0.04	0.46	-0.58	-0.89	-1.10	-1.26	-1.27	-1.44
1789.83	1.07	0.52	0.65	0.38	0.00	-0.59	-0.46	-0.70	-0.91	-1.24	-1.25
1929.34	0.37	0.70	0.08	0.13	-0.27	-0.27	-1.02	-0.83	-1.28	-1.58	-1.36
2079.8	-0.06	0.53	0.27	0.41	0.02	-0.56	-0.50	-0.36	-0.20	-0.58	-0.41
2242.08	0.76	0.43	0.60	-0.46	0.28	-0.78	-0.39	-0.75	-0.63	-0.60	-1.22
2417.1	0.68	0.18	0.44	0.13	-0.16	-0.74	-1.03	-0.57	-1.05	-1.50	-1.49
2605.85	0.47	0.25	0.61	-0.15	-0.12	-0.75	-0.85	-1.15	-1.19	-1.51	-1.38
2809.43	0.90	1.16	0.14	-0.39	-0.26	-0.55	-1.25	-1.09	-1.18	-1.65	-1.93
3028.99	0.33	0.03	0.38	-0.34	-0.35	-0.54	-0.65	-1.02	-0.63	-0.75	-1.14
3265.79	0.09	0.08	0.01	-0.04	-0.41	0.39	-0.48	-0.47	0.19	-0.18	-0.76
3521.18	1.57	0.72	0.98	-0.01	0.48	0.00	-0.30	-0.51	-0.62	-0.57	-0.70
3796.62	1.19	0.58	0.48	0.41	-0.12	-0.33	-0.82	-0.81	-1.21	-1.10	-1.21

Wavelength (nm) / Time (ps)	351.69	353.10	354.51	355.92	357.33	358.73	360.14	361.55	362.96	364.37	365.78
-3.28	0.02	-0.30	0.00	-0.15	-0.04	0.01	-0.15	-0.18	0.15	-0.11	-0.27
-2.78	0.13	-0.48	-0.17	0.01	0.02	0.24	0.14	0.01	-0.26	0.07	0.17
-2.28	-0.16	0.23	0.04	0.03	-0.08	-0.22	0.17	0.14	0.06	-0.23	0.05
-1.78	-0.14	0.22	-0.23	0.09	-0.11	0.34	-0.10	-0.08	-0.12	0.05	0.30
-1.28	0.03	0.06	0.13	0.13	0.26	0.27	0.22	0.33	0.22	0.30	0.04
-0.78	0.29	0.34	0.13	0.12	0.01	0.08	-0.19	-0.27	-0.14	0.11	0.07
-0.28	0.08	0.10	0.25	-0.08	0.04	0.00	0.20	0.27	0.38	0.19	0.00
0.22	-0.25	-0.17	-0.16	-0.15	-0.10	-0.71	-0.31	-0.21	-0.31	-0.37	-0.36
0.32	0.29	0.21	-0.45	0.30	0.23	0.11	0.04	-0.03	0.06	0.00	0.02
0.42	0.16	0.06	0.13	-0.11	0.13	0.11	0.39	-0.19	-0.17	0.26	0.02
0.52	0.13	-0.14	-0.18	-0.29	-0.05	0.22	0.33	0.14	0.39	0.28	0.10
0.62	-0.47	-0.30	0.15	0.11	-0.08	0.17	0.14	-0.03	-0.17	-0.33	-0.01
0.72	-2.05	-2.31	-2.45	-2.80	-2.49	-2.19	-1.10	-0.61	0.05	0.44	0.35
0.77	-0.94	-1.35	-1.77	-1.63	-1.54	-1.40	-1.26	-0.96	-0.71	-0.45	-0.33
0.82	-0.47	-0.32	-0.16	-0.50	-0.39	-0.45	-0.64	-1.12	-0.95	-1.38	-1.08
0.87	-0.75	-0.96	-0.61	-0.39	-0.15	-0.21	-0.11	0.14	0.15	0.25	0.29
0.92	-0.95	-1.16	-1.15	-0.95	-0.60	-0.25	0.20	0.29	0.42	0.54	0.74
0.97	-0.95	-0.84	-0.86	-1.08	-0.41	-0.21	0.10	-0.07	0.36	0.54	0.87
1.02	-0.73	-0.43	-0.57	-0.67	-0.08	-0.25	0.07	0.37	0.71	0.95	1.21
1.07	-0.52	-0.20	-0.49	-0.20	-0.35	0.25	0.81	0.46	0.84	1.38	1.37
1.12	-0.54	-0.48	-0.25	-0.24	-0.04	-0.01	0.60	0.42	0.61	0.89	1.00

1.17	-1.10	-1.21	-1.05	-0.58	-0.65	-0.52	-0.18	-0.40	-0.25	-0.02	0.56
1.22	-1.33	-1.46	-1.29	-1.07	-1.09	-1.27	-0.69	-0.63	-0.51	-0.61	-0.43
1.27	-0.73	-0.78	-0.69	-0.75	-0.51	0.00	0.04	0.11	0.16	0.36	0.53
1.32	-0.05	-0.10	0.00	0.12	0.57	0.50	0.75	0.75	1.13	1.01	1.15
1.37	-0.55	-0.53	-0.51	-0.32	-0.14	-0.20	-0.07	-0.15	0.39	0.64	0.79
1.42	-0.85	-0.83	-1.09	-1.06	-0.41	-0.86	-0.59	-0.47	-0.14	-0.07	0.14
1.47	-1.51	-1.35	-1.38	-0.99	-1.15	-1.01	-0.73	-0.76	-0.15	-0.47	-0.01
1.52	-1.99	-1.69	-1.77	-1.46	-1.44	-1.23	-1.31	-0.87	-0.61	-0.55	-0.22
1.57	-0.28	-0.37	-0.39	-0.71	-0.29	-0.11	0.26	0.30	0.35	0.60	0.76
1.62	-0.45	-0.42	-0.16	-0.22	0.20	0.36	0.62	0.70	0.38	1.03	1.08
1.67	-0.16	-0.52	-0.22	-0.44	0.08	0.05	0.33	0.47	0.55	0.55	0.62
1.72	-0.43	-0.83	-0.81	-0.70	-0.54	-0.64	-0.13	-0.13	-0.28	-0.05	-0.06
1.77	-1.10	-0.89	-1.24	-1.12	-1.06	-0.79	-0.84	-0.63	-0.59	-0.50	-0.40
1.82	-1.19	-0.99	-0.79	-0.94	-0.44	-0.70	-0.35	0.06	-0.12	-0.15	0.29
1.87	-0.20	0.01	0.15	-0.09	0.14	0.24	0.67	0.75	0.56	0.91	1.07
1.92	-0.42	-0.79	-0.55	-0.23	0.02	-0.19	0.28	0.30	0.55	0.76	0.84
1.97	-0.46	-0.23	-0.20	-0.11	-0.08	0.20	0.25	0.07	0.34	0.51	0.71
2.02	-0.89	-0.87	-0.96	-0.61	-0.68	-0.63	-0.39	-0.26	-0.05	-0.04	0.39
2.07	-1.12	-1.21	-0.94	-0.83	-0.88	-1.01	-0.73	-0.23	-0.37	-0.28	-0.06
2.12	-0.34	-0.25	-0.19	-0.14	-0.37	-0.10	0.39	0.22	0.54	0.82	1.02
2.17	-0.41	-0.26	-0.18	-0.06	0.02	0.30	0.50	0.68	0.79	0.96	1.35
2.22	-0.24	0.15	-0.24	-0.26	-0.08	-0.18	0.67	0.62	0.98	1.22	0.70
2.27	-1.40	-1.13	-1.34	-1.17	-0.86	-0.75	-0.62	-0.53	-0.34	-0.16	0.29
2.32	-0.73	-1.21	-0.77	-0.85	-0.76	-0.60	-0.32	-0.52	-0.31	-0.12	0.30
2.37	-0.61	-0.84	-0.59	-0.75	-0.39	-0.60	-0.16	-0.33	-0.11	0.06	0.53
2.42	-0.87	-0.71	-0.39	-0.63	-0.25	-0.31	0.10	-0.01	0.44	0.45	0.72
2.47	-0.38	-0.45	-0.27	0.11	-0.05	0.14	0.52	0.59	0.46	0.90	1.28
2.52	-0.66	-0.33	-0.82	-0.61	-0.05	-0.46	-0.32	0.15	0.14	-0.08	0.77
2.57	-0.97	-1.12	-0.94	-0.99	-0.69	-0.45	-0.09	-0.03	0.10	0.07	0.31
2.62	-1.16	-1.31	-1.31	-1.23	-1.35	-1.09	-0.96	-0.62	-0.68	-0.64	-0.36
2.67	-0.47	-0.71	-0.39	-0.58	-0.39	-0.26	0.01	0.16	0.13	0.40	0.48
2.72	-0.44	-0.16	-0.13	-0.02	0.05	0.53	0.62	0.84	0.43	1.00	1.45
2.77	-0.20	-0.09	-0.04	-0.09	0.41	0.38	0.60	0.80	0.67	1.01	1.14
2.82	-1.42	-1.39	-1.18	-1.29	-0.85	-0.83	-0.76	-0.54	-0.40	-0.24	-0.02
2.87	-0.62	-0.73	-0.90	-0.64	-0.43	-0.38	-0.35	-0.09	-0.20	0.06	0.31
2.92	-1.12	-1.27	-1.02	-1.26	-1.08	-0.80	-0.44	-0.67	-0.12	-0.38	-0.28
2.97	0.22	0.02	-0.10	0.07	0.20	0.03	0.40	0.52	0.60	0.92	0.48
3.02	-0.04	-0.23	0.05	0.00	0.25	0.39	0.61	0.80	0.78	1.06	1.25
3.07	-0.60	-0.78	-0.57	-0.37	-0.30	-0.23	0.17	0.16	0.26	0.35	0.60
3.12	-1.04	-1.01	-1.10	-1.08	-0.95	-0.75	-0.50	-0.46	-0.07	-0.19	0.13
3.17	-0.64	-1.19	-0.99	-1.05	-0.53	-0.94	-0.56	-0.53	-0.37	-0.42	-0.27
3.22	-0.92	-0.68	-0.52	-0.60	-0.29	-0.19	-0.04	-0.11	0.26	0.42	0.53
3.27	-0.39	-0.89	-0.24	-0.05	0.22	0.24	0.79	0.65	0.64	1.07	1.34
3.32	-1.17	-0.62	-0.46	-0.34	-0.35	-0.43	0.09	-0.04	0.10	0.58	1.06
3.37	-0.72	-1.03	-0.67	-0.99	-0.36	-0.32	-0.13	-0.32	0.10	0.19	0.44
3.42	-0.85	-1.18	-0.79	-0.97	-0.64	-0.83	-0.22	-0.18	0.14	0.16	0.23
3.47	-1.59	-1.53	-1.69	-1.45	-1.33	-1.16	-0.88	-0.78	-0.88	-0.60	-0.30
3.52	-0.45	-0.50	-0.58	-0.46	0.05	-0.07	0.32	0.16	0.59	0.36	0.56
3.57	-0.41	-0.01	0.05	-0.05	0.12	0.57	0.56	0.82	0.79	0.99	1.35
3.62	-0.58	-0.17	-0.39	-0.53	-0.07	-0.02	0.35	0.04	0.36	0.49	0.61

3.67	-0.64	-0.87	-0.71	-0.96	-0.73	-0.76	-0.35	-0.20	-0.21	0.08	0.57
3.72	-1.42	-1.35	-1.40	-1.25	-1.08	-1.36	-0.92	-1.14	-0.93	-0.39	-0.22
3.92	-0.86	-1.05	-0.62	-0.62	-0.32	-0.47	-0.13	0.23	0.21	0.07	0.32
4.12	-0.07	-0.16	0.08	-0.06	-0.15	-0.01	0.30	0.47	0.65	0.72	1.04
4.32	-0.28	-0.73	-0.71	-0.22	0.14	0.00	0.27	0.15	0.42	1.04	1.13
4.52	-0.50	-0.85	-0.87	-0.96	-0.75	-0.69	-0.30	-0.31	-0.25	0.17	0.40
4.72	-0.91	-0.85	-1.02	-0.94	-0.88	-0.66	-0.27	-0.56	-0.16	0.24	0.28
4.92	-0.84	-0.99	-1.23	-0.93	-0.83	-0.73	-0.37	-0.47	-0.42	-0.56	-0.13
5.12	-0.16	-0.61	-0.66	-0.47	-0.37	-0.05	0.05	-0.10	0.60	0.47	0.68
5.32	-0.58	-0.04	0.11	0.10	0.28	0.39	0.66	0.80	0.83	1.20	1.17
5.52	-0.57	-0.57	-0.92	-0.25	-0.19	-0.27	0.14	0.25	0.34	0.88	1.04
5.72	-0.40	-0.63	-0.63	-0.70	-0.50	-0.53	0.14	-0.36	-0.15	0.16	0.33
5.92	-1.05	-1.02	-0.62	-0.91	-0.64	-0.72	-0.28	-0.43	-0.01	-0.03	0.16
6.12	-1.16	-0.83	-1.00	-1.01	-0.58	-0.44	-0.08	-0.36	-0.02	-0.23	0.19
6.32	-0.23	-0.21	-0.47	-0.34	0.01	-0.06	0.31	0.30	0.52	0.48	1.39
6.52	-0.44	-0.46	-0.08	-0.28	0.04	0.19	0.37	0.28	0.74	0.60	0.83
6.72	-0.20	-0.17	-0.24	-0.39	-0.28	-0.15	-0.05	0.18	0.32	0.42	0.46
6.92	-0.80	-0.87	-0.61	-0.79	-0.42	-0.69	-0.46	-0.21	-0.27	-0.03	0.35
7.12	-1.07	-1.53	-1.42	-1.42	-0.95	-1.16	-0.86	-0.94	-0.77	-0.68	-0.30
7.32	-0.35	-0.56	-0.17	-0.23	-0.44	0.06	0.20	0.33	0.59	0.70	0.61
7.52	-0.38	-0.22	-0.55	-0.26	-0.30	-0.11	0.16	0.52	0.49	0.47	0.69
7.72	-0.56	-0.23	-0.44	-0.09	0.36	0.05	0.38	0.63	0.66	0.57	1.18
7.92	-0.99	-1.13	-1.04	-0.89	-0.91	-0.96	-0.46	-0.37	-0.26	0.08	0.35
8.12	-1.07	-1.02	-1.08	-1.03	-1.08	-0.98	-0.52	-0.46	-0.20	-0.22	0.04
8.32	-1.16	-1.71	-1.29	-1.32	-1.16	-1.16	-0.83	-0.64	-0.58	-0.72	-0.02
8.52	-0.60	-0.49	-0.09	-0.74	-0.26	-0.12	0.03	0.27	0.70	0.45	0.67
8.72	-0.49	-0.46	-0.44	-0.37	-0.25	-0.31	0.55	0.31	0.67	0.60	1.02
8.92	-0.47	-0.18	-0.38	-0.62	-0.24	-0.24	0.15	0.16	0.53	0.45	0.54
9.12	-0.89	-0.83	-1.06	-0.69	-0.65	-0.51	-0.44	-0.62	0.14	0.24	0.16
9.32	-0.62	-0.77	-0.84	-0.97	-0.67	-0.59	-0.52	-0.60	-0.04	-0.15	0.00
9.52	-0.49	-0.35	-0.44	-0.35	-0.53	-0.32	-0.27	-0.24	0.16	0.25	0.57
9.72	-0.16	-0.33	-0.18	-0.27	-0.24	0.25	0.36	0.34	0.40	0.82	1.23
9.92	-0.30	-0.35	0.04	-0.05	0.15	-0.04	0.48	0.59	0.51	0.93	1.12
10.12	-0.86	-0.93	-1.05	-0.52	-0.48	-0.77	-0.30	-0.08	0.02	-0.06	0.45
10.32	-0.85	-0.82	-0.96	-1.04	-0.71	-0.84	-0.44	-0.09	-0.15	-0.13	0.39
10.52	-0.93	-1.10	-1.23	-1.07	-0.98	-1.08	-0.49	-0.82	-0.47	-0.57	-0.27
10.72	-0.35	-0.15	-0.41	-0.29	0.35	0.04	0.56	0.54	0.42	0.90	1.01
10.92	-0.08	-0.58	-0.23	-0.05	0.17	0.37	0.64	0.26	1.01	1.10	1.21
11.12	-0.36	-0.34	-0.02	-0.31	-0.23	-0.04	0.15	0.51	0.58	0.85	0.66
11.32	-0.83	-0.87	-0.38	-0.88	-0.28	-0.63	-0.19	-0.11	0.22	0.14	0.62
11.52	-0.90	-1.19	-1.08	-1.27	-1.02	-0.85	-0.74	-0.40	-0.53	-0.29	-0.05
11.72	-1.14	-0.93	-1.07	-1.16	-0.22	-0.71	-0.62	-0.53	0.06	0.13	0.10
11.92	-0.89	-1.14	-1.15	-0.64	-0.61	-0.45	-0.29	-0.16	0.08	0.35	0.61
12.12	-0.53	-0.78	-0.73	-0.55	-0.39	-0.30	-0.26	-0.07	0.32	0.45	0.73
12.32	-0.53	-0.96	-0.63	-0.48	-0.33	-0.64	0.08	-0.38	0.08	-0.08	0.15
12.52	-0.78	-0.83	-0.76	-0.99	-0.99	-0.44	-0.32	-0.35	-0.11	-0.31	0.17
12.72	-0.80	-1.09	-0.91	-1.19	-0.75	-0.89	-0.66	-0.66	-0.46	-0.53	-0.18
12.92	-0.74	-0.73	-0.58	-0.23	-0.41	-0.25	0.09	0.10	0.39	0.64	0.77
13.12	-0.39	0.09	-0.19	-0.17	0.26	-0.16	0.61	0.33	0.78	0.78	0.97
13.32	-0.41	-0.44	-0.18	-0.39	-0.16	0.21	0.37	0.37	0.53	0.83	0.64

13.52	-0.78	-0.53	-0.90	-0.83	-0.62	-0.58	-0.20	0.10	0.15	0.04	0.40
13.72	-0.75	-0.85	-0.82	-0.98	-0.91	-0.63	-0.33	-0.46	0.06	0.11	0.20
13.92	-0.63	-0.91	-1.14	-0.95	-0.81	-0.75	-0.43	-0.13	0.03	-0.07	0.15
14.00	-0.63	-0.31	-0.27	-0.75	-0.58	-0.16	0.34	0.17	0.39	0.83	0.70
14.08	-0.28	-0.79	-0.51	-0.50	-0.39	-0.11	0.47	0.47	0.56	0.74	1.18
14.17	-0.90	-1.12	-0.93	-0.77	-0.49	-0.51	-0.34	-0.33	-0.07	0.15	0.20
14.27	-0.86	-0.58	-1.03	-0.79	-0.38	-0.63	0.21	0.00	0.20	0.42	0.57
14.38	-0.83	-1.23	-0.61	-1.35	-1.12	-0.92	-1.02	-0.53	-0.51	-0.49	-0.32
14.49	-0.75	-0.64	-0.52	-0.60	-0.52	-0.54	-0.22	-0.17	0.03	0.20	0.46
14.61	-0.19	-0.26	-0.53	-0.34	0.11	0.32	0.33	0.39	0.82	0.88	1.02
14.75	-0.55	-0.47	-0.56	-0.20	-0.20	-0.31	0.24	0.24	0.25	0.53	0.68
14.89	-1.08	-0.72	-0.78	-0.58	-0.57	-0.50	-0.24	-0.06	0.05	0.01	0.32
15.05	-1.19	-1.01	-1.00	-0.69	-0.75	-0.93	-0.57	-0.32	-0.17	-0.06	0.33
15.21	-1.14	-1.28	-1.22	-1.01	-0.88	-1.04	-0.72	-0.49	-0.58	-0.26	-0.16
15.39	0.02	-0.63	-0.34	-0.10	-0.36	0.16	0.53	0.40	0.52	0.37	0.80
15.59	-0.36	-0.46	-0.13	-0.14	-0.19	-0.02	0.40	0.41	0.56	0.14	0.86
15.8	-0.59	-1.02	-0.42	-0.24	-0.34	-0.29	0.04	0.21	0.13	0.28	0.34
16.02	-0.65	-0.78	-0.66	-0.66	-0.64	-0.62	-0.27	-0.10	0.21	0.21	0.51
16.27	-1.26	-0.96	-1.03	-1.29	-1.13	-1.03	-0.70	-0.43	-0.32	-0.44	-0.08
16.53	-0.73	-0.92	-0.85	-1.08	-0.87	-0.60	-0.24	-0.35	-0.32	-0.13	0.29
16.81	0.08	-0.09	-0.11	-0.27	-0.12	0.05	0.31	0.36	0.63	0.61	1.14
17.12	-0.86	-0.70	-0.94	-0.57	-0.72	-0.27	0.12	-0.01	0.27	0.43	0.92
17.45	-0.87	-0.50	-0.39	-0.40	-0.16	-0.20	0.19	-0.01	0.20	0.61	0.63
17.81	-1.26	-0.74	-1.04	-0.77	-0.67	-0.56	-0.29	-0.31	0.20	-0.01	0.33
18.19	-0.40	-0.61	-0.72	-0.31	-0.50	-0.39	-0.54	-0.57	-0.10	-0.29	-0.16
18.6	-0.22	-0.49	-0.13	-0.29	-0.13	-0.09	0.42	0.67	0.67	0.65	0.93
19.05	-0.03	0.07	0.04	0.17	0.18	0.48	0.58	0.87	0.92	1.11	1.25
19.53	-0.63	-0.88	-0.76	-0.83	-0.54	-0.43	-0.12	-0.21	-0.06	0.42	0.28
20.05	-0.99	-0.94	-0.75	-1.04	-0.65	-0.44	-0.56	-0.36	0.02	0.12	0.35
20.61	-1.07	-0.66	-0.85	-0.57	-0.75	-0.47	-0.45	-0.31	0.02	0.05	0.42
21.22	-0.66	-0.47	-1.04	-0.59	-0.41	-0.17	0.03	-0.18	0.08	0.16	0.49
21.87	-0.59	-0.47	-0.69	-0.62	-0.35	-0.16	0.09	0.20	0.39	0.84	0.87
22.57	-0.54	-0.36	-0.34	-0.26	0.27	0.41	0.45	0.42	0.48	0.64	0.85
23.33	-0.83	-0.97	-0.83	-0.93	-0.59	-0.52	-0.71	-0.26	-0.15	-0.27	-0.07
24.15	-0.92	-0.71	-0.85	-1.07	-0.88	-0.59	-0.37	-0.31	-0.16	0.04	0.08
25.03	-0.78	-1.28	-0.96	-0.99	-0.91	-0.65	-0.58	-0.72	-0.53	-0.17	-0.55
25.98	-0.48	-0.42	-0.26	-0.07	-0.10	0.17	0.19	0.41	0.45	0.69	0.86
27.01	-0.27	-0.14	-0.36	-0.01	0.44	0.33	0.67	0.71	0.84	0.79	1.08
28.11	-0.75	-0.87	-0.71	-0.70	-0.48	-0.20	-0.16	-0.21	0.08	0.04	0.53
29.3	-0.43	-0.77	-0.59	-0.72	-0.34	-0.28	-0.19	-0.35	0.20	0.35	0.57
30.59	-0.37	-0.66	-0.83	-0.73	-0.73	-0.29	-0.18	-0.25	0.12	0.07	0.23
31.98	-0.86	-1.12	-0.83	-0.88	-0.61	-0.53	-0.27	-0.16	-0.05	-0.23	0.27
33.47	-0.44	-0.35	-0.28	-0.34	-0.14	-0.17	0.08	0.36	0.46	0.31	0.48
35.09	-0.17	-0.50	-0.46	-0.52	-0.45	-0.21	0.34	0.19	0.38	0.42	0.74
36.83	-0.43	-0.77	-0.50	-0.91	-0.44	-0.61	-0.27	-0.38	-0.03	0.15	0.27
38.71	-0.45	-0.75	-0.60	-0.43	-0.44	-0.07	-0.09	-0.05	0.22	0.27	0.40
40.73	-0.98	-0.78	-1.19	-0.85	-0.63	-0.65	-0.51	-0.37	0.00	-0.25	0.00
42.92	-0.62	-0.75	-0.83	-0.23	-0.51	-0.42	-0.06	-0.07	0.10	0.03	0.31
45.27	-0.19	-0.68	-0.29	0.13	-0.30	0.33	0.34	0.47	0.60	0.79	1.26
47.81	-0.01	-0.39	-0.08	-0.21	-0.34	-0.30	-0.05	0.68	0.53	0.66	0.76

50.55	-0.57	-0.92	-0.80	-0.57	-0.32	-0.56	-0.17	-0.37	0.28	-0.10	0.61
53.51	-0.52	-0.08	-0.34	-0.57	-0.30	-0.40	0.14	-0.06	0.31	0.35	0.54
56.69	-0.80	-0.95	-0.85	-0.86	-0.81	-0.46	-0.33	-0.29	-0.11	-0.01	0.08
60.13	-0.23	-0.18	0.07	-0.10	0.30	0.20	0.58	0.45	0.54	0.86	0.93
63.84	-0.76	-0.42	-0.26	-0.56	0.01	-0.18	0.42	0.44	0.63	0.50	0.79
67.83	-0.29	-0.52	-0.18	-0.31	-0.06	-0.11	0.12	0.14	0.15	0.59	0.54
72.15	-0.65	-1.00	-1.22	-0.67	-0.56	-0.41	0.10	-0.20	0.03	0.03	0.68
76.8	-0.74	-0.86	-0.69	-0.98	-0.61	-0.76	-0.75	-0.37	-0.23	-0.31	0.14
81.81	-0.64	-0.63	-0.83	-0.55	-0.38	-0.05	0.12	0.04	-0.11	0.41	0.37
87.22	-0.59	-0.54	-0.84	-0.71	-0.22	-0.10	0.36	0.20	0.58	0.34	1.03
93.06	-0.24	-0.37	-0.34	-0.30	-0.21	0.06	-0.01	0.22	0.58	0.76	0.50
99.35	-0.92	-1.04	-0.93	-0.99	-0.92	-0.56	-0.50	-0.35	0.00	0.13	0.22
106.13	-0.76	-0.63	-0.94	-0.50	-0.35	-0.45	-0.34	-0.42	0.19	0.25	0.35
113.45	-0.88	-0.92	-0.95	-1.08	-0.89	-0.75	-0.24	-0.09	-0.31	-0.44	-0.10
121.34	-0.79	-0.58	-0.42	0.01	0.12	0.32	0.44	0.53	0.37	0.94	0.67
129.86	-0.39	-0.53	-0.18	0.03	0.01	0.32	0.56	0.20	0.75	0.64	0.98
139.04	-0.97	-0.78	-0.77	-0.87	-0.57	-0.34	0.00	-0.38	-0.20	0.01	0.63
148.94	-0.60	-0.95	-0.60	-1.04	-0.79	-0.79	0.03	-0.37	-0.19	0.19	0.28
159.62	-0.88	-0.97	-1.01	-0.84	-0.88	-0.51	-0.58	-0.19	-0.18	0.22	0.25
171.14	-0.72	-0.82	-1.10	-1.08	-0.80	-0.57	-0.27	-0.20	-0.20	0.15	0.48
183.56	-0.22	-0.61	-0.35	-0.53	-0.01	0.14	0.37	0.83	1.03	0.98	1.59
196.96	-0.79	-0.76	-0.23	-0.47	-0.42	0.07	0.21	0.33	0.47	0.36	0.73
211.41	-0.90	-0.85	-0.82	-0.86	-0.76	-0.76	-0.13	-0.32	-0.07	0.03	0.40
226.99	-1.05	-0.89	-0.98	-0.82	-0.79	-0.68	-0.59	-0.26	0.01	-0.16	0.34
243.8	-1.16	-0.98	-1.02	-0.94	-0.76	-0.48	-0.37	-0.52	-0.34	-0.27	-0.01
261.93	-0.38	-0.42	-0.19	-0.06	0.16	-0.03	0.27	0.33	0.82	0.83	1.07
281.48	-0.41	-0.51	-0.69	-0.38	-0.24	0.10	-0.03	0.33	0.18	0.63	1.18
302.56	-0.33	-0.91	-0.54	-0.19	-0.16	-0.07	0.22	0.22	0.31	0.85	1.10
325.3	-0.81	-1.07	-0.66	-0.62	-0.53	-0.32	-0.15	0.11	0.37	0.24	0.76
349.83	-0.96	-0.93	-1.00	-1.18	-0.54	-0.77	-0.46	-0.55	-0.07	0.00	0.90
376.28	-0.99	-0.96	-0.83	-1.09	-0.83	-0.74	-0.38	-0.35	-0.17	0.32	0.42
404.81	-0.49	-0.83	-0.58	-0.58	-0.27	-0.07	0.34	0.51	0.51	0.92	1.32
435.58	-0.61	-0.59	-0.58	-0.55	-0.28	0.08	0.55	0.51	0.65	1.19	1.44
468.76	-0.91	-1.33	-1.06	-1.08	-0.69	-0.57	-0.41	-0.26	-0.11	0.02	0.32
504.55	-1.09	-0.88	-0.96	-1.01	-0.82	-0.52	-0.31	-0.21	-0.05	0.26	0.75
543.15	-1.16	-1.05	-0.90	-0.73	-0.76	-0.36	-0.26	-0.33	-0.12	0.01	0.19
584.78	-1.17	-0.95	-0.87	-0.98	-0.72	-0.50	-0.15	0.09	0.74	0.57	1.06
629.68	-0.71	-0.76	-0.60	-0.50	-0.04	-0.29	0.39	0.71	0.57	0.76	1.13
678.1	-1.40	-1.31	-0.87	-1.18	-0.60	-0.65	-0.37	-0.30	0.04	-0.10	0.91
730.33	-0.90	-0.96	-1.30	-1.20	-0.89	-0.80	-0.40	-0.47	0.05	0.09	0.28
786.65	-0.92	-1.13	-1.06	-0.89	-0.45	-0.28	-0.04	-0.10	0.47	0.51	0.71
847.4	-1.70	-1.21	-1.38	-1.18	-0.99	-0.96	-0.96	-0.60	-0.46	-0.22	0.25
912.92	-0.69	-0.82	-0.50	-0.41	-0.06	0.00	0.35	0.48	0.82	0.70	1.00
983.58	-1.08	-0.92	-0.64	-0.40	-0.46	-0.03	0.26	0.35	0.77	1.11	1.27
1059.78	-1.43	-1.78	-1.30	-1.31	-0.99	-1.12	-0.41	-0.47	0.11	0.01	0.73
1141.98	-1.37	-1.31	-1.09	-0.98	-0.90	-0.46	-0.08	-0.22	0.14	0.45	0.81
1230.62	-1.44	-1.38	-1.50	-1.32	-1.21	-1.22	-0.94	-0.87	-0.15	-0.24	0.28
1326.23	-1.61	-1.59	-1.25	-1.16	-1.05	-0.94	-0.47	-0.11	0.11	0.16	0.78
1429.34	-0.55	-0.49	-0.42	-0.48	-0.02	-0.24	0.39	0.54	0.61	1.08	1.40
1540.54	-1.00	-1.26	-1.05	-0.64	-0.75	-0.26	-0.23	-0.16	0.40	0.51	0.87

1660.48	-1.31	-1.43	-0.86	-1.34	-0.89	-1.18	-0.73	-0.53	-0.79	-0.11	0.35
1789.83	-1.23	-1.26	-1.23	-1.27	-0.87	-0.56	-0.44	-0.23	0.12	0.18	0.63
1929.34	-1.28	-1.39	-1.68	-1.58	-1.08	-1.04	-0.72	-0.66	-0.58	-0.47	0.23
2079.8	-0.83	-0.63	-0.36	-0.38	-0.12	-0.17	0.29	0.69	0.78	1.14	1.64
2242.08	-0.90	-0.71	-0.84	-0.78	-0.57	0.15	0.42	0.56	0.58	0.81	0.99
2417.1	-1.38	-1.37	-1.41	-1.27	-0.78	-0.59	-0.35	-0.58	-0.18	0.20	0.40
2605.85	-1.30	-1.67	-1.42	-1.45	-1.09	-0.76	-0.29	-0.33	-0.01	0.33	0.73
2809.43	-1.30	-1.99	-1.60	-1.88	-1.26	-0.98	-1.14	-0.75	-0.52	0.09	0.50
3028.99	-1.11	-1.47	-0.73	-1.10	-0.63	-0.59	-0.07	-0.05	0.14	0.53	0.87
3265.79	-0.27	-0.46	-0.52	-0.26	0.12	0.21	0.31	0.58	0.54	1.05	1.38
3521.18	-1.33	-1.04	-0.74	-1.15	-0.78	-0.29	0.02	0.10	0.45	0.52	0.83
3796.62	-1.03	-1.32	-1.17	-1.14	-0.93	-0.97	-0.57	-0.81	-0.19	0.45	0.25

Wavelength (nm) / Time (ps)	367.19	368.59	370.00	371.41	372.82	374.23	375.64	377.05	378.45	379.86	381.27
-3.28	-0.22	-0.03	-0.20	-0.17	-0.24	-0.14	-0.14	-0.23	-0.01	-0.09	-0.16
-2.78	0.22	-0.06	-0.01	-0.17	0.17	-0.12	0.11	0.07	0.03	0.26	0.32
-2.28	-0.07	-0.14	0.49	0.09	-0.16	0.14	0.00	-0.18	-0.12	0.06	0.18
-1.78	-0.02	0.07	-0.16	0.12	-0.15	0.06	-0.04	-0.18	-0.29	-0.25	-0.34
-1.28	0.08	0.27	-0.02	-0.01	0.21	0.05	0.25	0.13	0.19	0.13	-0.05
-0.78	-0.04	0.06	-0.02	-0.04	-0.06	0.01	-0.24	0.02	0.20	0.08	-0.13
-0.28	0.38	0.16	0.22	0.27	0.37	0.41	0.24	0.37	0.16	0.25	0.24
0.22	-0.33	-0.33	-0.31	-0.09	-0.14	-0.42	-0.18	0.01	-0.17	-0.44	-0.04
0.32	-0.04	0.18	-0.09	0.01	-0.07	0.16	0.13	-0.07	-0.14	0.09	-0.10
0.42	-0.10	-0.09	0.02	0.06	0.13	-0.15	0.16	0.29	0.00	0.02	-0.01
0.52	0.21	0.13	0.05	0.33	0.14	0.20	0.43	0.40	0.21	0.16	0.08
0.62	-0.08	-0.22	0.12	-0.07	0.11	0.00	-0.01	0.10	0.03	-0.03	0.08
0.72	0.29	0.44	0.35	0.10	-0.03	0.22	-0.01	0.25	0.27	0.22	0.06
0.77	-0.24	-0.41	-0.08	-0.29	-0.14	-0.10	0.00	0.15	-0.12	0.24	-0.07
0.82	-0.99	-0.29	-0.21	-0.20	0.01	0.07	-0.24	-0.10	-0.17	-0.11	-0.01
0.87	0.39	0.76	0.85	1.18	1.18	1.36	1.50	1.33	0.93	0.53	0.14
0.92	0.55	1.07	1.20	1.35	1.66	1.56	2.24	2.41	2.95	3.44	3.69
0.97	1.22	1.30	1.55	2.17	2.43	2.18	2.69	3.02	3.07	3.27	3.79
1.02	1.97	2.03	2.38	3.15	3.76	4.45	5.22	5.88	6.61	7.12	7.28
1.07	1.75	2.51	2.62	2.95	3.50	4.15	4.50	4.92	5.82	6.22	7.11
1.12	1.34	1.91	1.97	2.42	2.63	3.07	3.11	3.70	4.02	4.14	4.66
1.17	0.83	1.15	1.40	1.83	2.03	2.32	2.60	2.96	3.30	3.70	3.80
1.22	-0.20	0.43	0.48	0.87	1.31	1.62	2.01	2.12	2.56	2.87	3.19
1.27	0.64	1.13	1.42	1.34	1.91	2.17	2.21	2.65	2.96	3.22	3.55
1.32	1.67	1.90	2.30	2.81	3.28	3.10	3.47	3.98	4.40	4.58	4.98
1.37	0.97	1.57	1.71	2.13	2.60	2.31	2.81	3.47	3.68	3.73	4.37
1.42	0.51	0.56	0.81	1.19	1.69	1.77	2.25	2.56	2.92	3.21	3.17
1.47	0.05	0.48	0.77	1.17	1.44	1.55	1.81	2.22	2.42	2.74	3.01
1.52	-0.19	0.22	0.69	0.87	1.03	1.18	1.50	1.92	2.29	2.36	2.75
1.57	0.94	1.32	1.67	2.09	2.26	2.67	2.56	3.01	3.46	3.87	3.73
1.62	1.26	1.99	2.20	2.46	2.91	2.96	3.18	3.65	3.95	4.12	4.12
1.67	1.39	1.44	1.65	1.90	2.18	2.05	2.64	2.91	3.33	3.02	3.44
1.72	0.57	0.78	0.95	1.06	1.46	1.42	1.98	2.20	2.43	2.71	2.88

1.77	-0.15	0.29	0.50	0.89	1.21	1.51	1.78	2.16	2.30	2.74	3.01
1.82	0.57	0.73	1.12	1.48	1.80	1.75	2.26	2.65	2.79	2.86	3.23
1.87	1.35	1.75	1.93	2.39	2.74	2.47	3.24	3.37	3.52	3.80	4.39
1.92	1.05	1.88	1.91	2.13	2.32	2.62	2.83	3.17	3.34	3.88	4.09
1.97	0.83	0.78	1.28	1.57	1.75	2.08	2.21	2.57	2.86	2.90	3.51
2.02	0.49	0.81	1.22	1.48	1.66	1.77	2.18	2.51	2.67	2.73	2.85
2.07	0.16	0.40	0.52	0.82	1.14	1.23	1.50	2.02	2.26	2.51	2.79
2.12	1.49	1.77	1.82	2.29	2.33	2.64	2.90	3.24	3.29	3.63	3.53
2.17	1.32	1.80	2.21	2.07	2.89	2.87	3.21	3.30	3.64	3.87	4.24
2.22	1.26	1.70	1.83	2.11	2.25	2.71	2.92	3.23	3.33	3.31	3.64
2.27	0.59	0.87	1.00	1.40	1.82	2.00	2.47	2.54	2.90	3.28	3.75
2.32	0.06	0.56	0.98	0.98	1.24	1.71	1.85	2.29	2.70	2.83	2.78
2.37	0.42	0.66	0.74	1.24	1.55	1.78	2.16	2.68	2.42	2.90	2.96
2.42	1.23	1.41	1.64	2.07	2.46	2.61	3.04	3.22	3.36	3.72	3.82
2.47	1.13	1.68	2.09	2.13	2.58	2.90	3.13	3.31	3.46	3.54	3.85
2.52	0.74	0.99	1.10	1.20	1.54	1.39	1.72	2.39	2.57	2.51	3.04
2.57	0.26	0.91	1.34	1.41	1.75	1.63	1.87	2.16	2.50	2.74	2.96
2.62	0.06	0.24	0.34	0.77	1.04	1.36	1.30	1.84	2.13	2.43	2.58
2.67	0.69	0.97	1.43	1.64	2.19	2.05	2.09	2.60	2.97	3.06	3.49
2.72	1.46	1.67	1.98	2.43	2.59	2.53	2.98	3.45	3.52	4.16	3.85
2.77	1.60	1.80	2.13	2.26	2.64	2.81	2.92	3.18	3.72	3.80	3.95
2.82	0.20	0.67	0.68	1.16	1.40	1.61	1.83	2.53	2.70	2.88	3.16
2.87	0.66	0.88	0.87	1.32	1.76	1.74	1.84	2.51	2.69	2.80	3.16
2.92	0.16	0.38	0.77	1.11	1.20	1.55	1.88	2.11	2.45	2.69	3.10
2.97	1.11	1.76	1.84	2.31	2.48	2.64	3.03	3.01	3.42	3.50	3.68
3.02	2.07	2.09	2.38	2.56	2.76	2.94	3.52	3.77	3.71	4.03	4.56
3.07	0.94	1.11	1.37	1.24	1.55	1.71	2.00	2.37	2.61	3.05	2.90
3.12	0.51	0.97	1.36	1.26	1.90	2.05	2.40	2.69	2.97	3.29	3.24
3.17	0.19	0.13	0.59	1.35	1.27	1.25	1.82	2.20	2.52	2.77	2.86
3.22	0.89	1.15	1.38	1.55	2.08	1.79	2.50	2.83	3.14	3.33	3.56
3.27	1.26	1.67	2.46	2.54	2.70	2.97	3.23	3.24	3.76	3.96	4.14
3.32	0.96	1.20	1.67	1.90	2.17	2.37	3.00	3.12	3.18	3.71	3.58
3.37	0.50	0.95	1.17	1.36	1.63	1.77	2.14	2.61	2.85	3.02	3.14
3.42	0.61	0.87	1.32	1.33	1.52	1.83	2.27	2.12	2.50	2.88	3.19
3.47	0.09	0.35	0.59	0.79	1.13	1.27	1.74	1.93	2.17	2.35	2.76
3.52	1.31	1.43	1.82	2.26	2.66	2.58	2.92	3.13	3.06	3.54	3.84
3.57	1.74	2.16	2.31	2.56	2.91	3.03	3.33	3.58	3.72	3.73	4.13
3.62	1.11	1.15	1.40	2.02	1.92	2.20	2.41	2.97	2.77	3.14	3.44
3.67	0.38	0.96	1.12	1.54	1.91	2.18	2.54	2.67	2.77	2.86	3.34
3.72	-0.03	0.25	0.57	0.65	0.86	1.36	1.82	2.10	2.46	2.45	2.89
3.92	0.58	1.18	1.23	1.49	1.44	1.91	2.17	2.37	2.84	2.53	2.99
4.12	1.13	1.42	2.14	2.34	2.71	2.54	2.98	3.36	3.40	3.86	4.09
4.32	1.11	1.90	2.03	2.36	2.60	2.68	3.07	3.62	3.58	3.92	4.11
4.52	0.46	0.81	1.20	1.37	1.52	1.76	2.12	2.20	2.66	2.87	3.06
4.72	0.52	1.05	1.17	1.32	1.43	1.81	2.16	2.55	2.46	2.59	3.02
4.92	0.41	0.32	0.99	1.29	1.23	1.43	1.77	2.18	2.47	2.62	2.62
5.12	1.01	1.02	1.33	1.67	2.12	2.13	2.55	2.94	3.12	3.34	3.73
5.32	1.42	1.93	2.13	2.41	2.44	2.71	3.26	3.32	3.67	4.00	4.30
5.52	1.09	1.23	1.59	2.06	2.22	2.19	2.69	2.86	3.17	3.64	3.55
5.72	0.51	0.99	1.30	1.63	1.55	2.02	2.10	2.52	2.80	3.02	3.32

5.92	0.39	0.92	1.07	1.22	1.68	1.71	2.05	2.31	2.58	2.74	2.93
6.12	0.46	0.96	0.83	1.18	1.16	1.67	1.83	2.12	2.14	2.54	2.77
6.32	1.22	1.51	1.75	2.14	2.66	2.75	2.86	3.24	3.65	3.93	3.84
6.52	1.33	1.75	1.52	2.09	2.71	2.65	2.74	3.49	3.51	3.60	3.62
6.72	0.67	1.19	1.16	1.42	1.68	1.95	1.98	2.37	2.48	2.62	3.06
6.92	0.59	0.73	1.02	1.34	1.99	1.86	2.36	2.60	2.61	3.04	3.09
7.12	-0.07	0.28	0.28	0.87	1.16	1.05	1.72	2.13	2.22	2.36	2.66
7.32	1.01	1.46	1.23	1.77	2.14	2.18	2.45	2.57	2.92	3.20	3.14
7.52	1.16	1.28	1.56	2.24	2.43	2.48	2.92	3.18	3.50	3.70	3.92
7.72	1.19	1.63	1.82	2.21	2.45	2.58	2.63	3.07	3.16	3.62	3.75
7.92	0.38	0.48	1.12	1.07	1.68	1.42	2.15	2.31	2.55	2.98	3.03
8.12	0.23	0.66	0.81	0.96	1.41	1.40	1.75	1.93	2.19	2.73	2.61
8.32	0.06	0.18	0.61	0.79	1.18	1.35	1.67	1.76	2.14	2.37	2.52
8.52	1.51	1.42	1.82	2.41	2.49	2.77	2.96	3.47	3.34	3.72	3.87
8.72	1.16	1.28	1.84	1.88	2.20	2.48	3.05	2.93	3.09	3.20	3.36
8.92	0.71	1.40	1.31	1.51	1.62	2.37	2.20	2.65	2.51	3.00	3.02
9.12	0.56	1.06	0.93	1.28	1.81	2.07	2.32	2.95	3.07	2.97	3.46
9.32	-0.02	0.41	0.74	0.82	1.39	1.27	1.66	2.03	2.23	2.56	2.65
9.52	0.63	0.98	1.05	1.38	1.48	2.05	1.93	2.36	2.78	2.84	2.90
9.72	1.20	1.51	1.93	2.17	2.24	2.54	2.95	3.49	3.47	3.79	3.90
9.92	1.17	1.65	1.96	2.15	2.32	2.64	2.61	2.95	3.34	3.48	3.68
10.12	0.51	0.91	1.24	1.56	1.57	2.01	2.47	2.48	2.70	2.79	3.02
10.32	0.67	1.18	1.27	1.47	1.92	2.03	2.43	2.59	2.91	2.95	3.19
10.52	-0.34	0.52	0.63	0.68	1.27	1.33	1.77	2.19	2.34	2.58	2.65
10.72	1.29	1.50	1.67	1.92	2.20	2.53	2.62	2.95	3.02	3.22	3.61
10.92	1.42	1.70	1.96	1.91	2.44	2.74	3.24	3.23	3.36	3.71	3.73
11.12	1.14	1.20	1.74	1.95	1.95	2.14	2.35	2.76	3.20	3.19	3.67
11.32	0.76	1.03	1.23	1.29	1.80	2.10	2.49	2.73	2.81	3.27	3.33
11.52	0.31	0.59	0.83	1.30	1.46	1.59	1.80	2.22	2.59	2.79	2.82
11.72	0.56	0.77	0.64	1.11	1.37	1.61	1.73	2.20	2.36	2.55	2.90
11.92	1.11	1.52	1.55	1.83	2.15	2.55	2.80	3.12	3.49	3.68	3.97
12.12	0.60	1.30	1.07	1.47	2.03	2.18	2.19	2.54	2.47	2.74	3.06
12.32	0.81	0.99	1.06	1.46	1.57	1.61	1.99	2.15	2.68	2.79	3.03
12.52	0.44	0.65	0.97	1.22	1.67	1.77	2.02	2.30	2.75	2.84	2.94
12.72	0.09	0.18	0.88	0.82	1.21	1.53	1.85	1.79	2.21	2.25	2.55
12.92	1.29	1.20	1.12	1.81	2.07	2.25	2.46	2.99	2.97	3.13	3.53
13.12	1.18	1.80	1.79	2.23	2.72	2.75	2.74	3.24	3.42	3.61	3.80
13.32	1.10	1.37	1.99	1.87	2.41	2.60	2.66	3.13	3.31	3.55	3.21
13.52	0.38	0.73	1.21	1.55	1.61	2.10	2.01	2.43	2.57	2.78	3.12
13.72	0.41	0.74	1.18	1.43	1.73	1.54	2.18	2.50	2.48	2.41	2.73
13.92	0.28	0.41	0.78	1.03	1.38	1.85	1.85	2.18	2.62	2.72	2.74
14	1.06	1.48	1.71	1.85	2.60	2.41	3.15	3.31	3.49	3.30	3.98
14.08	1.33	1.62	1.89	1.98	2.49	2.13	2.95	3.23	3.40	3.60	3.51
14.17	0.36	0.87	1.04	1.32	1.66	1.63	2.04	2.43	2.51	2.62	3.16
14.27	0.54	1.01	1.24	1.61	2.12	2.34	2.18	2.98	3.25	3.38	3.68
14.38	-0.08	0.42	0.56	0.69	1.19	1.42	1.76	2.00	2.07	2.37	2.82
14.49	0.84	1.00	1.39	1.32	1.67	1.85	2.41	2.44	2.55	2.77	3.11
14.61	1.68	1.71	2.01	2.29	2.40	2.69	3.20	3.47	3.54	3.86	3.88
14.75	1.19	1.41	1.73	1.68	2.21	2.08	2.06	2.66	2.94	3.21	3.48
14.89	0.75	0.93	1.12	1.66	1.79	1.79	2.17	2.52	2.83	2.85	3.15

15.05	0.65	0.89	1.30	1.35	1.90	1.79	2.13	2.43	2.59	2.66	2.76
15.21	0.01	0.39	0.66	1.08	1.15	1.42	1.75	2.10	2.23	2.27	2.60
15.39	1.32	1.73	1.91	2.00	1.99	2.46	2.69	2.99	3.17	3.46	3.78
15.59	1.11	1.47	1.81	2.27	2.53	2.54	2.65	3.09	3.21	3.49	3.63
15.8	0.49	0.95	1.49	1.60	1.80	2.11	2.25	2.54	2.87	3.25	3.30
16.02	0.65	0.91	1.19	1.85	1.90	2.15	2.47	2.91	2.98	3.26	3.27
16.27	0.19	0.44	0.47	0.95	1.08	1.39	1.54	1.88	2.12	2.49	2.62
16.53	0.34	0.87	1.06	1.25	1.41	1.42	1.99	2.29	2.38	2.83	2.87
16.81	1.21	1.60	2.03	2.18	2.44	2.62	2.83	3.01	3.40	3.56	3.74
17.12	1.16	1.41	2.01	2.02	2.37	2.34	2.40	2.86	3.30	3.08	3.24
17.45	0.87	0.92	1.32	1.75	1.76	2.01	2.35	2.52	2.75	3.27	3.16
17.81	0.56	0.89	1.54	1.39	1.58	1.86	2.22	2.20	2.61	2.70	3.11
18.19	0.03	0.60	0.85	1.21	1.16	1.19	1.51	1.60	2.00	2.18	2.62
18.6	1.22	1.46	1.96	2.08	2.36	2.46	2.82	3.06	3.32	3.34	3.55
19.05	1.57	2.10	2.00	2.26	2.37	2.39	2.55	3.10	3.26	3.50	3.70
19.53	0.57	0.96	1.22	1.24	1.62	2.20	2.11	2.51	2.68	2.74	3.11
20.05	0.43	0.85	1.21	1.34	1.93	2.15	2.28	2.49	2.77	2.81	3.30
20.61	0.26	0.78	0.83	1.18	1.67	1.96	1.98	2.39	2.74	2.61	2.80
21.22	0.56	0.60	1.06	1.22	1.56	1.74	1.97	2.30	2.20	3.07	2.84
21.87	1.17	1.60	1.92	2.48	2.35	2.63	3.14	3.13	3.49	3.89	3.70
22.57	1.23	1.70	1.60	1.75	1.95	2.38	2.50	2.86	3.02	3.14	3.26
23.33	0.36	0.74	1.29	1.25	1.38	1.77	2.24	2.32	2.69	2.98	2.99
24.15	0.19	0.93	0.93	1.46	1.71	1.98	2.14	2.52	2.85	2.85	3.15
25.03	-0.15	0.35	0.38	0.75	0.86	1.28	1.43	1.70	1.89	2.23	2.36
25.98	1.10	1.66	1.73	2.14	2.56	2.51	2.76	2.87	3.22	3.49	3.66
27.01	1.43	1.44	2.17	2.08	2.38	2.76	2.76	3.04	3.02	3.56	3.95
28.11	1.13	1.19	1.41	1.66	2.01	1.75	2.14	2.46	2.69	2.77	3.20
29.3	0.90	1.29	1.58	1.76	1.67	2.32	2.28	2.61	2.90	2.95	3.25
30.59	0.52	0.97	0.94	1.36	1.43	1.77	1.94	2.22	2.48	2.48	2.79
31.98	0.39	0.46	0.88	1.45	1.42	1.85	1.76	2.42	2.43	2.64	2.71
33.47	0.74	1.20	1.63	2.32	2.25	2.49	2.51	3.07	3.26	3.21	3.59
35.09	0.95	1.37	1.56	1.67	1.86	2.00	2.49	2.72	2.95	3.19	3.34
36.83	0.48	1.08	1.06	1.47	1.53	1.90	2.16	2.35	2.52	3.00	2.97
38.71	0.92	1.21	1.17	1.75	1.95	1.95	2.45	2.84	3.19	3.05	3.13
40.73	0.19	0.33	0.63	1.18	1.15	1.56	1.77	2.00	2.40	2.41	2.56
42.92	0.84	0.61	1.32	1.33	1.71	2.22	2.23	2.36	2.75	2.82	3.12
45.27	1.05	1.45	1.78	2.18	2.44	2.73	3.09	3.19	3.47	3.59	3.91
47.81	1.15	1.55	1.84	2.03	2.45	2.31	2.65	2.86	3.17	3.03	3.53
50.55	0.74	0.81	1.12	1.43	1.93	1.85	2.21	2.45	2.66	2.99	3.25
53.51	0.51	1.12	1.31	1.31	1.93	1.80	2.08	2.25	2.41	2.77	3.08
56.69	0.54	0.62	0.64	0.87	1.25	1.36	1.78	1.87	2.22	2.28	2.54
60.13	1.36	1.56	1.71	2.10	2.27	2.52	2.99	3.34	3.25	3.50	3.67
63.84	1.07	1.26	1.51	1.95	2.20	2.24	2.27	2.74	2.91	3.24	3.16
67.83	1.03	1.18	1.29	1.84	1.93	1.93	2.35	2.44	2.92	3.17	3.21
72.15	0.91	0.76	1.16	1.58	1.80	2.15	2.51	2.58	2.83	2.91	3.20
76.8	0.10	0.55	0.81	1.22	1.47	1.42	1.65	1.96	2.23	2.30	2.65
81.81	0.67	1.12	1.15	1.63	1.62	1.84	2.17	2.50	2.70	2.58	3.14
87.22	1.41	1.72	1.88	2.32	2.27	2.68	2.88	3.12	3.37	3.48	3.65
93.06	0.96	1.69	1.40	1.80	2.31	2.34	2.28	2.79	2.76	2.91	2.95
99.35	0.54	0.84	1.05	1.46	1.66	1.88	2.00	2.55	2.72	2.92	2.92

106.13	0.58	1.24	1.15	1.25	1.87	1.86	2.29	2.60	2.96	3.13	3.08
113.45	0.42	0.76	1.07	1.21	1.56	1.58	1.95	2.28	2.43	2.47	3.01
121.34	1.24	1.54	2.23	2.26	2.52	2.75	2.83	3.49	3.60	3.60	4.09
129.86	1.21	1.57	1.98	2.11	2.28	2.63	2.80	3.17	3.34	3.44	3.64
139.04	0.73	1.04	1.09	1.21	1.35	1.84	2.04	2.24	2.50	2.62	2.66
148.94	0.71	0.99	1.20	1.56	1.74	2.08	2.52	2.74	2.75	3.25	3.29
159.62	0.58	0.80	1.22	1.73	1.97	1.73	2.25	2.20	2.50	2.60	2.96
171.14	0.46	0.80	0.98	1.41	1.73	1.81	2.23	2.48	2.60	2.79	2.94
183.56	1.23	1.85	2.29	2.31	2.95	2.83	3.12	3.38	3.49	3.58	3.98
196.96	0.87	1.29	1.83	1.82	2.09	2.17	2.73	3.04	3.06	3.17	3.43
211.41	0.49	0.91	1.48	1.49	1.65	1.84	2.29	2.54	2.95	3.06	3.29
226.99	0.58	0.89	1.34	1.52	1.86	2.33	2.51	2.78	2.99	3.08	3.42
243.8	0.53	0.71	1.25	1.42	1.53	1.66	2.19	2.26	2.59	2.84	2.89
261.93	1.43	1.64	2.18	2.15	2.38	2.52	2.85	3.18	3.10	3.29	3.59
281.48	1.47	1.90	2.02	2.30	2.52	2.72	3.26	3.22	3.60	3.69	3.71
302.56	1.28	1.39	1.89	1.65	1.98	2.44	2.32	2.60	2.80	2.77	2.98
325.3	0.76	1.47	1.74	1.75	2.13	2.32	2.59	2.78	3.02	3.06	3.18
349.83	0.74	1.11	1.64	1.76	2.11	2.38	2.79	2.65	2.73	3.06	3.10
376.28	0.88	0.78	1.32	1.57	1.82	1.79	2.05	2.44	2.49	2.62	2.87
404.81	1.75	2.01	2.11	2.45	3.02	3.05	3.48	3.62	3.78	3.81	3.77
435.58	1.57	1.96	2.19	2.30	2.52	2.70	3.21	3.54	3.63	3.34	3.69
468.76	0.91	0.95	1.26	1.42	2.00	2.09	2.20	2.63	2.94	2.96	3.20
504.55	0.78	1.35	1.79	1.86	2.23	2.49	2.73	3.10	2.94	3.00	3.27
543.15	0.79	1.09	1.44	1.74	1.87	2.12	2.20	2.43	2.71	2.71	2.89
584.78	1.14	1.63	1.74	1.75	2.35	2.77	2.87	2.94	3.21	3.02	3.24
629.68	1.46	1.78	2.07	2.48	2.83	2.87	3.23	3.15	3.49	3.54	3.74
678.1	0.93	1.25	1.33	1.77	2.12	2.22	2.43	2.82	2.89	2.96	3.04
730.33	0.71	1.19	1.33	1.52	1.92	2.11	2.44	2.59	2.86	2.95	3.18
786.65	1.12	1.61	1.95	2.03	2.27	2.56	2.84	3.24	3.30	3.57	3.62
847.4	0.43	0.83	0.78	1.46	1.77	1.87	2.06	2.24	2.46	2.44	2.76
912.92	1.84	1.84	2.27	2.59	2.84	3.02	3.14	3.64	3.77	3.98	4.07
983.58	1.59	1.76	2.15	2.45	3.08	3.12	3.18	3.27	3.60	3.53	3.72
1059.78	0.86	1.68	1.81	2.27	2.32	2.49	2.64	3.15	2.95	2.92	3.32
1141.98	1.20	1.62	1.86	2.35	2.79	2.78	3.08	3.03	3.32	3.36	3.55
1230.62	0.23	0.74	1.07	1.39	1.83	2.20	2.02	2.27	2.54	2.65	2.96
1326.23	0.87	1.45	1.46	1.80	1.75	2.05	2.32	2.85	2.88	3.01	3.35
1429.34	1.86	2.18	2.57	2.76	2.92	3.29	3.56	3.65	3.86	3.90	3.98
1540.54	1.31	1.59	1.89	2.18	2.38	2.66	2.91	3.17	3.24	3.05	3.27
1660.48	0.24	0.99	1.25	2.03	1.99	2.32	2.45	2.60	2.81	3.04	3.06
1789.83	0.90	1.31	1.64	1.91	2.18	2.57	2.91	3.02	3.21	3.30	3.50
1929.34	0.71	0.81	1.20	1.55	1.64	2.05	2.21	2.35	2.74	2.70	2.66
2079.8	1.85	2.32	2.69	2.83	3.43	3.23	3.55	3.96	4.10	4.05	4.35
2242.08	1.60	1.89	2.18	2.66	2.74	2.93	3.34	3.42	3.49	3.61	3.89
2417.1	0.55	1.31	1.41	1.66	2.04	1.98	2.21	2.80	2.60	2.70	2.76
2605.85	0.97	1.36	1.80	2.18	2.37	2.42	2.60	3.05	3.32	3.35	3.48
2809.43	0.62	1.23	1.01	1.55	1.80	1.88	2.30	2.59	2.52	2.79	2.72
3028.99	1.23	1.80	1.90	1.85	2.49	2.47	2.58	2.99	3.22	3.35	3.66
3265.79	1.85	2.07	2.33	2.67	3.05	2.96	3.24	3.49	3.54	3.79	3.73
3521.18	1.39	1.66	2.26	2.03	2.29	2.29	2.53	2.78	2.78	3.01	3.05
3796.62	0.73	1.08	1.62	1.81	1.96	2.28	2.46	2.77	2.88	2.78	2.96

Wavelength (nm) / Time (ps)	382.68	384.09	385.50	386.91	388.31	389.72	391.13	392.54	393.95	395.36	396.77
-3.28	-0.10	-0.18	-0.33	0.08	-0.09	0.05	-0.04	0.10	-0.01	0.12	-0.12
-2.78	0.01	-0.04	0.24	-0.04	0.07	0.09	0.09	-0.12	0.12	0.22	0.08
-2.28	-0.15	-0.03	-0.03	-0.27	-0.02	0.05	0.03	0.07	-0.13	-0.09	-0.05
-1.78	-0.05	-0.12	0.03	-0.25	0.05	-0.22	0.07	-0.13	-0.01	0.18	-0.03
-1.28	0.16	0.07	0.07	-0.08	-0.12	-0.06	-0.12	0.11	-0.16	-0.11	-0.05
-0.78	0.16	0.18	-0.07	0.22	0.10	0.01	-0.01	0.14	0.18	0.03	0.12
-0.28	0.26	0.12	0.19	0.25	0.13	0.34	0.10	0.03	0.05	-0.17	0.15
0.22	-0.29	0.00	-0.09	0.09	-0.12	-0.28	-0.13	-0.20	-0.04	-0.18	-0.09
0.32	0.01	-0.01	-0.26	-0.53	-0.09	0.03	-0.36	-0.30	-0.59	-0.18	-0.04
0.42	0.12	0.15	0.04	-0.24	-0.05	0.00	-0.40	-0.40	-0.26	-0.34	-0.28
0.52	0.10	0.19	0.25	-0.02	0.08	-0.06	0.08	0.25	-0.18	0.11	0.05
0.62	0.01	0.08	0.10	0.13	0.38	0.12	0.24	0.24	0.11	0.07	0.28
0.72	0.15	0.00	0.18	-0.33	0.02	-0.39	-0.08	-0.18	0.02	-0.15	0.03
0.77	0.11	0.11	-0.29	-0.21	0.16	0.04	0.13	0.22	-0.09	0.30	-0.26
0.82	0.19	-0.09	-0.16	-0.49	0.08	0.00	0.09	0.15	-0.06	-0.03	-0.11
0.87	0.04	-0.06	-0.09	0.04	0.04	0.19	0.00	-0.21	0.05	0.26	-0.01
0.92	3.92	3.48	3.21	2.20	1.73	0.83	0.39	0.02	-0.15	-0.23	-0.25
0.97	3.88	3.89	4.52	4.16	4.27	3.99	3.43	2.80	2.30	2.12	1.19
1.02	7.39	7.18	6.48	5.93	5.44	4.73	4.34	3.95	3.68	3.85	3.61
1.07	7.74	8.73	9.15	9.32	10.01	9.65	9.55	9.51	8.56	8.25	7.36
1.12	5.07	5.43	6.24	6.66	7.23	7.45	8.31	8.56	8.75	9.21	9.35
1.17	4.15	4.44	5.11	5.42	6.13	6.01	6.68	7.18	7.58	8.44	8.82
1.22	3.44	4.02	4.55	4.84	5.19	5.39	6.11	6.43	6.82	7.43	7.87
1.27	3.88	4.12	4.68	4.60	4.97	5.51	6.02	6.51	6.92	7.29	7.70
1.32	5.34	5.44	5.93	6.19	6.49	6.87	7.28	7.41	7.83	8.27	8.62
1.37	4.50	4.84	5.40	5.44	5.73	6.18	6.35	6.76	7.10	7.51	7.74
1.42	3.70	3.80	4.57	4.47	4.94	4.93	5.41	5.83	6.02	6.37	6.82
1.47	3.29	3.78	4.11	4.25	4.55	4.93	5.32	5.50	5.93	6.50	6.84
1.52	3.36	3.41	4.11	3.97	4.34	4.60	5.29	5.49	5.78	6.29	6.50
1.57	4.21	4.39	4.74	4.85	5.26	5.15	5.74	5.96	6.44	6.50	6.72
1.62	4.61	4.87	5.42	5.55	5.94	6.22	6.33	6.43	6.75	7.23	7.55
1.67	3.45	3.88	4.37	4.65	5.14	5.29	5.43	5.90	6.02	6.25	6.66
1.72	3.55	3.65	3.93	3.87	4.47	4.84	5.01	5.22	5.64	5.92	6.15
1.77	3.34	3.43	4.10	3.79	4.45	4.53	4.91	4.95	5.37	5.78	6.13
1.82	3.57	3.90	4.02	4.15	4.45	4.73	5.13	5.73	5.79	6.17	6.40
1.87	4.72	4.83	5.35	5.27	5.47	5.96	6.16	6.46	6.42	6.84	7.11
1.92	4.53	4.38	5.08	5.28	5.39	5.43	5.90	6.07	6.34	6.71	6.70
1.97	3.56	3.84	4.13	4.22	4.92	4.97	5.12	5.40	5.48	5.93	6.29
2.02	3.24	3.54	3.77	4.06	4.34	4.50	4.87	5.02	5.43	5.67	5.85
2.07	3.20	3.04	3.88	3.88	4.33	4.32	4.79	4.89	5.29	5.54	5.87
2.12	4.16	4.44	4.64	4.55	4.81	4.92	5.35	5.59	5.85	5.99	6.34
2.17	4.38	4.53	4.86	5.05	5.43	5.66	5.77	6.02	6.57	6.46	6.67
2.22	3.97	3.97	4.65	4.61	4.79	5.07	5.38	5.62	5.89	5.82	6.10
2.27	3.84	3.99	4.50	4.58	5.10	5.19	5.38	5.68	5.91	6.22	6.39
2.32	3.38	3.59	4.30	3.87	4.48	4.61	4.81	5.02	5.38	5.75	5.99

2.37	3.57	3.70	4.13	4.13	4.51	4.58	5.09	5.15	5.37	5.54	5.82
2.42	4.04	4.33	4.88	4.56	5.20	5.47	5.63	5.74	5.89	6.37	6.42
2.47	4.23	4.34	4.80	4.99	5.20	5.04	5.56	5.62	5.89	6.16	6.25
2.52	3.42	3.11	3.79	4.00	4.25	4.28	4.92	5.27	5.15	5.65	5.81
2.57	3.42	3.34	4.07	3.58	4.26	4.37	4.83	5.27	5.28	5.63	5.87
2.62	2.99	3.40	3.42	3.72	3.93	4.05	4.36	4.65	4.89	5.38	5.54
2.67	3.51	3.93	4.47	4.38	4.63	4.81	5.04	5.27	5.48	5.87	5.96
2.72	4.34	4.48	4.91	4.84	5.38	5.51	5.91	5.92	6.16	6.22	6.58
2.77	4.01	4.36	4.83	4.78	5.18	5.16	5.52	5.49	5.70	5.91	5.93
2.82	3.49	3.86	4.26	4.08	4.29	4.78	4.91	5.20	5.29	5.71	5.66
2.87	3.49	3.51	4.18	3.83	4.47	4.39	4.94	4.94	4.93	5.51	5.78
2.92	3.42	3.26	3.94	3.88	4.29	4.48	4.89	5.11	5.27	5.72	5.99
2.97	4.26	4.33	4.67	4.66	5.19	5.08	5.15	5.57	5.82	6.17	5.93
3.02	4.81	4.83	5.11	5.17	5.47	5.73	5.90	6.02	6.16	6.40	6.52
3.07	3.07	3.62	3.94	4.10	4.42	4.58	4.83	5.16	5.31	5.40	5.72
3.12	3.54	3.92	4.40	4.34	4.61	4.68	4.98	5.44	5.45	5.99	5.95
3.17	3.35	3.38	4.13	3.89	4.28	4.41	4.84	4.64	5.02	5.32	5.58
3.22	4.00	3.87	4.38	4.38	4.90	5.00	5.23	5.50	5.45	5.96	6.00
3.27	4.80	4.88	5.29	5.15	5.41	5.69	5.85	5.90	6.38	6.41	6.35
3.32	4.14	4.12	4.66	4.52	4.91	5.13	5.24	5.51	5.86	6.05	6.26
3.37	3.46	3.74	4.38	4.20	4.56	4.56	5.07	5.01	5.52	5.87	5.72
3.42	3.45	3.62	4.25	4.03	4.31	4.39	4.88	4.81	5.09	5.33	5.66
3.47	2.96	3.07	3.65	3.54	3.97	4.22	4.53	4.85	5.14	5.53	5.53
3.52	4.08	4.02	4.40	4.34	4.52	4.83	5.15	5.28	5.48	5.79	6.12
3.57	4.56	4.63	4.87	5.08	5.26	5.32	5.69	5.77	6.12	6.18	6.56
3.62	3.70	3.83	4.30	4.18	4.58	4.70	4.90	4.94	5.35	5.54	5.73
3.67	3.54	3.83	3.97	3.77	4.33	4.51	4.92	5.14	5.21	5.64	5.70
3.72	2.85	3.27	3.80	3.63	3.93	4.24	4.19	4.69	4.89	5.24	5.50
3.92	3.47	3.55	3.82	3.89	4.13	4.39	4.57	4.69	4.90	5.38	5.33
4.12	4.35	4.52	4.84	4.77	4.99	4.94	5.45	5.54	5.86	6.20	6.01
4.32	4.37	4.54	4.82	5.08	5.49	5.25	5.80	5.77	5.81	6.33	6.21
4.52	3.18	3.47	4.28	4.07	4.45	4.57	4.91	5.27	5.21	5.76	5.74
4.72	3.07	3.27	3.85	3.70	4.02	4.33	4.36	4.82	4.84	5.44	5.36
4.92	3.11	3.29	3.66	3.73	4.13	4.36	4.50	5.04	4.99	5.47	5.52
5.12	3.67	4.13	4.48	4.25	4.95	4.56	4.92	5.29	5.06	5.60	5.64
5.32	4.35	4.40	5.11	5.08	5.51	5.19	5.67	5.76	6.00	6.10	6.26
5.52	3.99	4.22	4.34	4.43	4.73	4.69	5.01	5.26	5.38	5.75	6.14
5.72	3.78	3.73	4.29	4.24	4.54	4.70	4.94	5.22	5.35	5.66	5.78
5.92	3.17	3.51	3.99	3.91	4.09	4.41	4.64	4.70	5.09	5.30	5.70
6.12	3.14	3.16	3.55	3.42	3.68	4.03	4.38	4.57	4.72	5.05	5.28
6.32	4.12	4.50	4.69	4.44	4.77	4.83	5.07	5.19	5.41	5.56	5.76
6.52	4.21	4.19	4.67	4.41	4.91	4.80	4.96	5.30	5.40	5.54	5.97
6.72	3.25	3.15	3.88	3.61	4.13	4.44	4.66	4.61	4.80	5.22	5.61
6.92	3.58	3.74	4.07	3.98	4.65	4.27	4.81	4.83	4.96	5.28	5.29
7.12	2.82	2.97	3.69	3.47	3.82	3.99	4.22	4.57	4.79	4.98	5.54
7.32	3.53	3.70	4.25	4.25	4.57	4.48	4.78	4.97	5.15	5.43	5.71
7.52	3.93	4.37	4.80	4.77	5.05	5.13	5.48	5.53	5.51	6.08	6.11
7.72	4.01	4.12	4.53	4.38	4.77	4.84	5.13	5.17	5.34	5.56	5.76
7.92	3.43	3.52	4.11	3.76	4.10	4.49	4.86	4.87	5.33	5.53	5.85
8.12	3.00	3.33	3.65	3.48	4.05	3.99	4.20	4.30	4.97	5.07	5.28

8.32	3.13	2.96	3.26	3.30	3.80	3.97	4.14	4.51	4.53	4.99	5.01
8.52	4.02	4.39	4.55	4.70	4.84	4.95	5.29	5.40	5.58	5.83	5.93
8.72	3.86	3.86	4.55	4.25	4.60	4.85	4.88	5.02	5.31	5.58	5.61
8.92	3.50	3.64	3.65	3.71	4.49	4.23	4.61	4.83	5.15	5.42	5.19
9.12	3.67	3.92	4.35	4.20	4.58	4.69	4.93	4.94	5.37	5.62	5.79
9.32	2.95	3.07	3.67	3.37	3.76	3.96	4.05	4.47	4.59	4.72	4.72
9.52	3.22	3.55	3.74	3.81	4.03	4.23	4.53	4.94	4.76	4.95	5.09
9.72	4.37	4.32	5.05	4.61	5.08	5.24	5.48	5.52	5.83	5.94	6.10
9.92	4.04	3.97	4.21	4.25	4.49	4.57	4.90	4.86	5.12	5.31	5.34
10.12	3.26	3.52	4.02	3.70	4.28	4.48	4.83	4.84	5.36	5.46	5.66
10.32	3.43	3.44	4.10	3.93	4.20	4.33	4.51	4.72	5.25	5.41	5.31
10.52	2.99	3.02	3.85	3.46	3.93	3.89	4.28	4.53	4.66	4.95	5.12
10.72	3.58	3.81	4.34	4.05	4.58	4.37	4.73	4.88	4.97	5.38	5.34
10.92	4.10	4.28	4.59	4.52	4.73	5.07	5.26	5.42	5.56	5.93	6.09
11.12	3.78	3.86	4.18	4.07	4.61	4.60	4.91	4.84	5.02	5.18	5.30
11.32	3.55	3.61	4.21	4.15	4.40	4.66	4.78	5.04	5.17	5.36	5.48
11.52	3.00	3.39	3.88	3.84	3.88	4.29	4.51	4.65	4.83	5.08	5.29
11.72	3.12	3.23	3.59	3.52	4.12	3.91	4.45	4.43	4.70	5.01	5.17
11.92	4.01	3.90	4.56	4.70	5.11	5.11	5.01	5.47	5.29	6.20	5.89
12.12	3.35	3.61	3.94	3.89	4.34	4.32	4.65	4.75	5.14	5.31	5.54
12.32	3.32	3.58	3.91	3.72	3.92	4.27	4.63	4.86	4.93	5.48	5.58
12.52	3.10	3.71	3.92	3.81	4.03	4.33	4.67	4.53	4.96	4.91	5.30
12.72	2.84	3.21	3.58	3.47	3.68	3.89	4.13	4.51	4.89	4.78	5.10
12.92	3.52	3.69	4.01	4.00	4.34	4.47	4.65	4.77	4.95	5.24	5.43
13.12	3.99	4.27	4.71	4.94	4.83	5.26	5.21	5.51	5.36	5.63	5.81
13.32	3.74	3.87	4.25	4.43	4.66	4.35	4.67	5.16	5.04	5.08	5.30
13.52	3.37	3.56	3.91	3.93	4.23	4.31	4.82	4.85	4.98	5.19	5.35
13.72	3.21	3.15	3.51	3.52	3.93	4.12	4.15	4.24	4.52	4.94	5.00
13.92	3.09	3.10	3.75	3.59	3.98	4.05	4.27	4.60	4.68	4.87	5.13
14	4.14	4.04	4.85	4.49	4.83	5.04	5.09	5.23	5.20	5.88	6.07
14.08	4.01	4.34	4.29	4.25	4.48	4.31	5.11	5.06	5.09	5.50	5.59
14.17	3.42	3.42	4.16	4.05	4.10	4.16	4.70	4.57	4.87	4.99	5.24
14.27	3.82	4.00	4.49	4.20	4.51	4.68	5.14	5.36	5.32	5.80	5.73
14.38	2.76	3.08	3.67	3.36	3.88	3.99	4.32	4.45	4.59	4.89	5.09
14.49	3.42	3.63	3.97	3.95	4.31	4.15	4.60	4.58	4.84	5.18	5.15
14.61	4.45	4.33	4.68	4.74	4.91	4.97	5.53	5.47	5.55	5.82	6.02
14.75	3.39	3.54	4.27	4.21	4.34	4.64	4.95	4.93	5.02	5.49	5.57
14.89	3.37	3.69	3.88	3.92	4.38	4.35	4.66	5.00	5.16	5.55	5.64
15.05	3.01	3.37	3.56	3.46	3.94	3.98	4.28	4.66	4.72	5.01	5.07
15.21	2.63	2.92	3.29	3.30	3.72	4.08	4.21	4.16	4.36	4.72	5.02
15.39	3.88	4.08	4.26	4.42	4.62	4.61	4.95	4.91	4.92	5.40	5.65
15.59	3.91	4.09	4.06	4.17	4.56	4.50	4.84	5.05	5.14	5.45	5.62
15.8	3.51	3.49	3.84	3.91	4.30	4.43	4.62	4.75	4.89	5.15	5.21
16.02	3.85	3.77	4.25	4.12	4.39	4.66	4.99	4.84	5.41	5.65	5.56
16.27	2.69	3.04	3.49	3.22	3.79	3.75	4.21	4.28	4.55	4.74	4.91
16.53	3.17	3.21	3.69	3.71	3.98	4.09	4.13	4.51	4.74	5.33	4.97
16.81	4.08	4.50	4.73	4.56	4.67	5.00	5.02	5.39	5.50	5.78	5.90
17.12	3.48	3.78	4.09	3.96	4.35	4.57	4.85	4.85	5.03	5.17	5.21
17.45	3.47	3.55	3.86	4.05	4.38	4.46	4.56	4.70	4.91	5.52	5.46
17.81	3.32	3.52	3.87	3.78	4.08	4.37	4.32	4.61	4.85	5.01	5.17

18.19	2.44	2.67	2.95	3.20	3.46	3.71	3.91	4.01	4.27	4.56	4.77
18.6	3.91	3.64	4.38	4.43	4.51	4.43	5.02	4.88	5.23	5.30	5.65
19.05	4.03	4.06	4.38	4.48	4.75	4.73	5.06	5.19	5.28	5.53	5.56
19.53	3.29	3.60	3.76	4.11	4.42	4.44	4.59	5.02	5.04	5.46	5.52
20.05	3.39	3.76	3.99	3.96	4.35	4.03	4.64	4.74	5.04	5.28	5.50
20.61	3.37	3.35	3.69	3.76	3.87	3.98	4.44	4.48	4.55	4.99	5.08
21.22	3.31	3.38	3.56	3.53	3.86	3.93	4.33	4.62	4.67	4.84	5.25
21.87	4.05	4.30	4.37	4.53	4.76	4.94	5.20	5.20	5.56	5.59	5.73
22.57	3.36	3.66	4.01	3.95	4.16	4.34	4.88	4.79	5.06	5.31	5.24
23.33	3.18	3.07	3.80	3.59	4.10	4.10	4.52	4.31	4.42	5.12	4.99
24.15	3.20	3.61	3.94	3.85	4.13	4.39	4.55	4.70	4.66	5.12	5.07
25.03	2.75	2.96	3.51	3.30	3.68	3.82	3.98	4.42	4.41	4.68	4.90
25.98	3.61	3.81	4.22	3.96	4.52	4.70	4.89	4.89	5.21	5.09	5.36
27.01	4.12	3.97	4.16	4.59	4.59	4.85	4.95	4.92	5.40	5.40	5.64
28.11	3.24	3.38	3.76	3.83	4.09	4.44	4.55	4.64	5.03	5.06	5.09
29.3	3.53	3.49	3.96	3.99	4.05	4.06	4.42	4.64	4.91	5.09	5.17
30.59	2.74	3.02	3.52	3.46	3.78	3.81	4.19	4.28	4.67	4.60	5.20
31.98	2.89	3.36	3.46	3.54	4.02	4.21	4.22	4.41	4.57	4.77	4.97
33.47	3.79	3.81	4.21	4.05	4.21	4.46	4.67	4.80	4.90	5.30	5.36
35.09	3.61	3.73	4.13	4.17	4.53	4.55	5.13	4.98	4.96	5.12	5.30
36.83	3.39	3.38	4.04	3.71	4.13	4.32	4.47	4.62	4.94	5.17	5.35
38.71	3.50	3.60	4.21	3.99	4.46	4.33	4.76	4.82	4.68	5.19	5.15
40.73	2.66	2.61	3.60	3.30	3.75	3.82	4.19	4.28	4.53	4.84	4.74
42.92	3.15	3.59	3.87	3.75	3.94	4.27	4.24	4.36	4.68	5.18	5.38
45.27	3.94	4.15	4.61	4.76	4.85	4.84	4.99	5.32	5.34	5.39	5.68
47.81	3.54	3.60	4.16	4.07	4.12	4.33	4.46	4.66	4.83	5.11	5.15
50.55	3.26	3.23	3.84	3.76	3.90	4.29	4.45	4.58	4.82	5.15	5.12
53.51	3.16	3.11	3.21	3.36	3.96	4.04	4.23	4.45	4.45	4.66	4.82
56.69	2.54	2.75	3.31	3.23	3.64	3.73	4.20	4.12	4.42	4.57	4.85
60.13	3.86	3.88	4.33	4.25	4.56	4.38	4.78	5.01	5.28	5.44	5.44
63.84	3.46	3.70	4.10	4.13	4.06	4.33	4.88	4.57	4.79	5.03	5.12
67.83	3.48	3.62	4.06	3.91	4.25	4.24	4.59	4.69	4.74	4.80	4.97
72.15	3.65	3.69	4.12	4.10	4.25	4.33	4.65	4.65	4.59	5.30	5.27
76.8	2.87	3.17	3.25	3.39	3.76	3.70	4.00	4.01	4.03	4.50	4.62
81.81	3.20	3.13	3.79	3.61	3.99	4.34	4.51	4.74	4.69	4.89	4.94
87.22	4.27	4.04	4.59	4.58	4.56	4.59	5.07	4.80	5.07	5.21	5.39
93.06	3.47	3.16	3.58	3.79	4.04	4.13	4.21	4.55	4.33	4.78	5.02
99.35	3.26	3.41	3.98	3.81	4.30	3.83	4.33	4.61	4.50	4.89	4.82
106.13	3.51	3.34	4.02	3.76	3.98	4.09	4.27	4.51	4.50	4.93	4.84
113.45	3.09	3.12	3.57	3.29	3.75	4.07	4.05	4.30	4.51	4.67	4.92
121.34	4.10	4.11	4.28	4.20	4.33	4.46	4.76	4.72	4.93	5.31	5.29
129.86	3.82	4.14	4.31	4.25	4.25	4.29	4.63	4.72	4.57	5.07	5.30
139.04	3.05	2.91	3.49	3.42	3.44	3.79	4.06	4.15	4.24	4.54	4.78
148.94	3.38	3.33	3.72	3.92	3.90	4.19	4.42	4.51	4.69	4.84	5.29
159.62	3.12	3.04	3.28	3.40	3.49	3.57	3.83	4.20	4.14	4.40	4.68
171.14	3.25	3.20	3.41	3.67	3.74	3.85	3.71	4.17	4.23	4.26	4.37
183.56	3.88	3.75	4.32	4.56	4.66	4.27	4.78	4.74	4.68	5.08	5.20
196.96	3.26	3.38	3.92	3.85	3.86	3.98	4.09	4.46	4.61	4.61	4.41
211.41	3.13	3.47	3.87	3.82	4.10	4.14	4.31	4.51	4.21	4.82	5.09
226.99	3.65	3.81	3.83	4.08	4.29	4.22	4.38	4.60	4.64	4.67	5.01

243.8	3.10	3.29	3.79	3.39	3.86	3.79	3.92	4.35	4.06	4.24	4.54
261.93	3.63	3.51	4.03	3.93	4.09	4.17	4.41	4.38	4.61	4.66	4.79
281.48	4.03	4.07	4.22	4.42	4.32	4.42	4.60	4.65	4.67	4.82	4.87
302.56	3.16	3.22	3.48	3.49	3.83	3.43	3.90	4.08	4.25	4.23	4.47
325.3	3.54	3.46	3.61	3.53	3.86	4.11	4.31	4.13	4.34	4.61	4.42
349.83	3.13	3.32	3.74	3.34	3.83	3.86	4.06	4.20	4.20	4.12	4.41
376.28	2.87	3.22	3.21	3.19	3.50	3.78	3.83	3.83	4.04	4.14	4.08
404.81	4.16	4.21	4.67	4.20	4.45	4.45	4.67	4.51	4.96	4.84	4.94
435.58	4.01	3.72	4.18	3.76	4.29	4.15	4.07	4.37	4.21	4.41	4.32
468.76	3.19	3.16	3.71	3.69	3.90	3.61	3.97	3.94	4.11	4.26	4.32
504.55	3.52	3.54	3.85	3.68	3.90	4.03	4.25	4.25	4.25	4.51	4.48
543.15	3.16	3.14	3.52	3.38	3.34	3.43	4.03	3.75	3.88	3.97	4.02
584.78	3.60	3.72	3.72	3.74	3.84	3.97	3.99	4.22	4.04	4.29	4.34
629.68	3.72	3.54	3.94	3.96	4.16	4.23	4.48	4.47	4.25	4.44	4.52
678.1	3.07	3.20	3.74	3.47	3.62	3.75	3.69	3.70	4.00	4.17	4.18
730.33	3.04	3.00	3.64	3.20	3.75	3.54	3.80	3.83	3.95	4.17	4.15
786.65	3.66	3.74	4.14	3.71	4.21	3.92	4.27	4.27	4.08	4.44	4.48
847.4	2.98	2.83	3.48	3.07	3.39	3.23	3.39	3.41	3.65	3.81	4.04
912.92	3.88	3.94	4.03	4.12	4.45	4.15	4.35	4.33	4.21	4.45	4.54
983.58	3.85	3.79	4.02	3.92	3.86	3.87	4.22	4.04	4.04	4.21	4.31
1059.78	3.28	3.53	3.67	3.32	3.39	3.48	3.73	3.64	3.63	3.91	3.97
1141.98	3.87	3.68	3.87	3.72	3.96	3.94	3.95	3.95	4.04	4.10	4.08
1230.62	3.03	3.04	3.42	2.98	3.07	3.24	3.20	3.40	3.46	3.59	3.55
1326.23	3.16	3.39	3.56	3.34	3.84	3.51	3.71	4.08	3.82	4.01	4.01
1429.34	4.26	4.06	4.34	4.01	3.97	4.20	4.12	4.33	4.22	4.37	4.44
1540.54	3.53	3.36	3.59	3.33	3.50	3.69	3.56	3.67	3.58	3.99	3.79
1660.48	3.22	3.19	3.14	3.25	3.50	3.41	3.65	3.86	3.72	3.97	4.02
1789.83	3.60	3.51	4.02	3.88	3.75	3.73	3.94	3.96	3.98	4.06	4.34
1929.34	2.97	3.03	3.11	3.24	3.57	3.63	3.65	3.91	3.89	4.03	3.89
2079.8	4.15	4.10	4.51	4.07	4.13	4.19	4.40	4.16	4.09	4.44	4.24
2242.08	3.91	3.70	3.88	4.12	4.16	3.84	4.24	4.13	4.09	4.21	4.37
2417.1	2.85	2.91	3.17	3.03	3.44	3.22	3.49	3.57	3.51	3.88	3.87
2605.85	3.52	3.43	3.98	3.71	3.77	3.55	3.62	4.00	4.17	4.27	4.13
2809.43	3.10	3.14	3.28	3.42	3.16	3.27	3.74	3.80	3.45	3.76	3.82
3028.99	3.82	3.65	3.83	3.51	3.65	3.72	3.53	3.71	4.03	3.99	3.99
3265.79	3.65	3.67	4.00	3.97	3.97	3.72	4.03	4.01	3.82	4.16	4.07
3521.18	3.09	3.04	3.50	3.37	3.22	3.35	3.48	3.53	3.58	3.71	3.71
3796.62	3.45	3.40	3.51	3.42	3.66	3.48	3.68	3.61	3.95	3.83	3.95

Wavelength (nm) / Time (ps)	398.17	399.58	400.99	402.40	403.81	405.22	406.63	408.03	409.44	410.85	412.26
-3.28	-0.23	-0.05	-0.13	0.02	-0.04	-0.09	0.07	-0.12	-0.04	0.13	0.16
-2.78	-0.12	0.25	0.05	-0.15	-0.13	0.07	-0.15	-0.05	0.15	-0.10	0.19
-2.28	-0.02	-0.29	0.08	-0.14	0.09	0.19	-0.15	0.03	0.21	0.17	-0.02
-1.78	0.00	0.13	-0.16	0.31	0.12	-0.08	0.01	0.09	0.02	-0.01	-0.24
-1.28	0.01	0.03	0.16	0.17	-0.23	0.06	0.35	-0.22	-0.36	-0.23	-0.18
-0.78	0.24	0.09	0.11	-0.16	0.10	0.18	0.14	0.41	0.08	0.16	0.04
-0.28	0.15	0.00	-0.07	-0.15	0.22	-0.22	-0.02	0.10	-0.22	-0.08	-0.04

0.22	-0.03	-0.16	-0.04	0.09	-0.13	-0.13	-0.23	-0.24	0.15	-0.04	0.09
0.32	-0.03	-0.09	-0.10	0.09	-0.21	0.23	0.34	0.00	-0.31	0.21	-0.03
0.42	-0.25	-0.01	-0.05	-0.24	-0.08	-0.04	-0.06	-0.05	-0.08	-0.01	-0.03
0.52	0.13	0.00	0.03	0.08	0.05	0.12	0.01	-0.02	-0.06	0.03	-0.12
0.62	0.15	0.36	0.09	0.10	-0.09	0.20	0.31	0.45	-0.16	0.52	0.07
0.72	-0.19	-0.05	0.06	-0.01	0.02	0.16	-0.20	-0.06	-0.27	0.14	0.15
0.77	-0.03	-0.13	0.03	0.04	0.02	0.10	-0.01	0.37	-0.07	0.08	0.05
0.82	-0.18	0.07	0.20	-0.05	0.25	0.03	0.08	0.06	-0.13	-0.20	0.04
0.87	0.04	0.25	0.01	0.19	-0.10	-0.02	0.12	0.12	0.08	0.24	0.06
0.92	-0.46	-0.30	-0.43	-0.05	-0.06	0.16	-0.19	-0.24	-0.19	-0.08	-0.14
0.97	0.51	-0.15	-1.27	-1.47	-1.05	-0.61	-0.65	-0.63	-0.18	0.02	-0.41
1.02	3.10	2.21	1.56	0.06	-0.52	-0.46	-0.56	-0.59	-0.46	-0.35	-0.04
1.07	7.03	5.81	4.36	3.34	2.71	2.30	1.84	2.16	1.62	1.61	1.54
1.12	9.75	9.87	10.05	9.77	9.58	9.32	9.27	9.43	8.84	8.84	8.76
1.17	9.00	9.63	9.80	9.91	10.02	10.25	10.30	9.77	10.12	10.34	10.32
1.22	8.19	8.35	8.82	8.81	9.07	9.52	9.62	10.17	10.25	10.54	11.02
1.27	8.02	7.89	8.25	7.88	8.48	8.73	8.61	8.91	9.02	10.07	10.14
1.32	8.89	8.66	8.76	8.79	8.87	8.82	9.15	9.58	9.49	10.05	10.48
1.37	7.93	7.88	7.82	7.55	7.77	7.86	7.95	8.23	8.59	9.47	10.04
1.42	7.03	7.19	7.18	6.93	7.33	7.54	7.33	7.80	8.08	8.60	8.85
1.47	6.82	7.10	7.07	7.42	7.46	7.51	7.78	8.22	7.72	8.44	8.73
1.52	6.70	6.74	7.01	6.45	6.99	6.93	7.18	7.44	7.56	8.26	8.42
1.57	6.85	6.62	6.56	6.83	6.79	6.86	6.71	7.48	7.38	8.12	8.38
1.62	7.51	7.72	7.04	7.19	7.28	7.63	7.38	7.26	7.96	7.75	8.25
1.67	6.83	6.84	6.96	6.89	6.88	6.82	6.96	7.11	7.30	7.67	7.53
1.72	6.18	6.34	6.53	6.49	6.50	6.37	6.37	6.76	6.77	7.45	7.22
1.77	6.29	6.16	6.17	6.11	6.32	6.30	6.20	6.42	6.34	6.88	7.35
1.82	6.48	6.31	6.69	6.36	6.39	6.37	6.86	6.74	6.59	7.40	7.78
1.87	7.15	7.08	6.73	6.50	6.60	6.55	6.09	6.78	6.67	7.15	7.32
1.92	6.69	6.43	6.41	6.52	6.29	6.14	6.55	7.13	6.75	7.06	7.63
1.97	6.25	6.39	6.19	6.36	6.14	6.19	6.07	6.55	6.31	7.19	6.97
2.02	6.14	6.16	6.14	5.86	5.95	6.23	6.33	6.42	6.53	6.77	7.09
2.07	5.91	5.84	5.93	5.74	5.92	6.36	6.03	6.49	6.39	7.20	7.09
2.12	6.46	6.20	6.29	5.86	5.85	6.20	5.79	6.25	6.17	6.42	6.71
2.17	6.71	6.53	6.59	6.30	6.52	6.51	6.33	6.52	6.26	6.93	7.12
2.22	6.26	6.12	6.09	6.11	5.76	5.90	5.96	6.08	6.00	6.46	6.57
2.27	6.50	6.53	6.30	6.26	6.22	6.23	6.29	6.76	6.69	7.05	7.33
2.32	5.91	5.96	5.94	5.93	6.04	6.10	5.76	6.32	6.38	6.81	6.70
2.37	5.98	6.20	6.01	5.68	5.69	5.76	5.95	6.17	6.19	6.59	6.64
2.42	6.44	6.43	6.40	6.01	5.98	5.98	6.27	6.35	6.38	6.81	7.15
2.47	6.29	6.30	6.12	5.98	5.79	5.82	6.42	6.16	6.18	6.44	6.70
2.52	6.03	5.97	6.02	5.60	5.52	5.76	5.65	5.48	5.98	6.59	6.40
2.57	5.78	5.98	5.77	5.93	5.39	5.94	5.79	5.81	5.56	6.20	6.21
2.62	5.48	5.53	5.48	5.55	5.53	5.69	5.72	6.15	6.01	6.18	6.24
2.67	6.32	5.89	5.93	5.95	5.85	5.81	6.11	6.03	6.27	6.63	6.17
2.72	6.46	6.28	6.46	6.06	6.23	6.44	6.05	6.00	5.95	6.49	6.83
2.77	6.20	6.01	6.02	5.53	5.78	5.46	5.46	5.91	6.04	5.85	6.15
2.82	5.82	5.96	5.87	5.72	5.98	5.71	5.72	5.87	6.15	6.08	6.56
2.87	5.81	5.92	5.68	5.43	5.58	5.70	5.75	5.98	6.00	6.34	6.31
2.92	5.90	6.02	5.92	5.80	6.09	5.82	6.20	6.12	5.80	6.19	6.42

2.97	6.08	6.11	5.94	5.66	5.48	5.75	5.46	5.78	5.70	6.10	6.46
3.02	6.26	6.09	6.26	6.02	5.83	5.54	5.83	5.72	5.93	6.32	6.48
3.07	5.97	5.78	5.67	5.34	5.34	5.69	5.13	5.54	5.59	5.95	5.85
3.12	6.36	6.24	6.03	5.77	6.02	6.15	5.90	6.39	6.06	6.52	6.69
3.17	5.74	5.81	5.61	5.40	5.42	5.54	5.38	5.92	5.39	6.19	6.21
3.22	5.94	5.98	5.92	6.03	5.96	5.72	5.59	5.69	6.26	6.73	6.90
3.27	6.60	6.54	6.43	5.93	5.93	5.93	5.60	6.05	6.26	6.52	6.51
3.32	5.88	6.05	5.86	5.53	5.62	5.50	5.33	5.94	5.87	6.56	6.38
3.37	5.86	5.85	5.75	5.68	5.55	5.48	5.20	5.75	5.79	6.08	6.50
3.42	5.69	5.57	5.40	5.53	5.38	5.60	5.64	5.55	5.59	5.90	6.00
3.47	5.86	5.91	5.75	5.55	5.37	5.41	5.54	6.00	5.86	6.27	6.14
3.52	5.86	5.93	5.74	5.53	5.77	5.94	5.99	5.85	6.20	6.50	6.52
3.57	6.56	6.63	6.23	6.06	6.01	5.75	6.29	5.90	5.88	6.43	6.57
3.62	5.76	5.59	5.41	5.33	5.42	5.76	5.81	5.80	5.80	5.95	5.85
3.67	5.90	5.92	5.63	5.41	5.63	5.46	5.49	5.48	5.15	6.01	6.23
3.72	5.70	5.54	5.62	5.40	5.38	5.45	5.58	5.99	5.79	5.88	6.34
3.92	5.33	5.55	5.09	5.37	5.33	5.41	5.42	5.24	5.61	6.07	6.15
4.12	6.43	6.04	6.15	5.88	5.72	5.71	6.05	5.56	5.78	6.54	6.50
4.32	6.29	6.44	6.19	5.59	5.88	5.39	5.63	5.92	5.92	6.36	6.24
4.52	5.81	5.91	5.67	5.71	5.97	5.71	5.40	5.69	6.03	6.19	6.27
4.72	5.51	5.67	5.58	5.58	5.46	5.59	5.68	5.52	5.46	6.08	6.17
4.92	5.53	5.45	5.55	5.07	5.50	5.54	5.67	5.69	5.60	6.13	6.01
5.12	5.83	5.77	5.32	5.23	5.38	5.63	5.40	5.40	5.69	6.00	5.95
5.32	6.31	6.41	5.88	5.56	6.12	5.73	5.45	5.81	5.88	5.92	6.27
5.52	5.80	5.95	5.59	5.63	5.25	5.47	5.39	5.90	5.70	6.27	6.34
5.72	5.91	5.80	5.90	5.65	5.54	5.83	5.32	5.60	5.57	5.80	6.00
5.92	5.62	5.62	5.66	5.70	5.54	5.47	5.73	6.05	5.78	5.87	6.11
6.12	5.45	5.22	5.23	5.40	5.18	5.34	5.32	5.15	5.37	5.92	5.73
6.32	5.81	5.93	5.58	5.41	5.23	5.29	5.59	5.63	5.80	6.11	6.24
6.52	6.14	5.74	5.75	5.51	5.46	5.21	5.14	5.59	5.21	5.76	5.97
6.72	5.51	5.49	5.62	5.22	5.24	5.25	5.29	5.43	4.98	5.99	5.64
6.92	5.36	5.54	5.23	5.14	5.20	5.07	5.46	5.26	5.40	5.84	5.70
7.12	5.22	5.64	5.32	5.23	5.42	5.17	5.27	5.16	5.76	5.78	5.97
7.32	5.73	5.55	5.21	5.33	5.10	4.96	5.18	5.61	5.23	5.87	6.19
7.52	5.97	6.13	6.00	5.89	5.56	5.40	5.56	5.30	5.57	5.71	5.87
7.72	5.35	5.67	5.28	5.19	5.03	5.28	5.13	5.47	5.13	5.65	5.75
7.92	5.52	5.91	5.34	5.10	5.54	5.52	5.29	5.36	5.66	5.82	6.19
8.12	5.35	5.34	5.30	5.22	5.20	5.09	5.18	5.20	5.09	5.49	5.80
8.32	5.16	5.03	5.12	5.19	5.08	5.39	5.21	5.15	5.16	6.06	5.87
8.52	5.84	5.90	5.94	5.21	5.60	5.32	5.48	5.65	5.59	6.10	5.79
8.72	5.60	5.70	5.32	5.45	5.64	5.10	4.82	5.65	5.40	5.84	6.06
8.92	5.77	5.59	5.45	5.58	5.24	5.09	5.20	5.43	5.29	5.43	6.15
9.12	5.52	5.66	5.54	5.47	5.29	5.43	5.56	5.46	5.40	5.49	6.09
9.32	5.05	5.02	5.14	4.96	4.98	4.94	5.05	5.35	4.90	5.47	5.52
9.52	5.26	5.25	5.08	5.07	5.01	5.06	5.56	5.42	5.11	5.77	5.51
9.72	6.13	6.04	5.88	5.78	5.56	5.42	5.11	5.68	5.25	5.77	5.78
9.92	5.44	5.68	5.44	5.12	5.12	5.25	5.16	5.31	5.73	6.03	5.74
10.12	5.60	5.58	5.68	5.41	5.43	5.42	5.24	5.85	5.67	5.65	5.80
10.32	5.35	5.49	5.24	5.22	5.20	5.39	4.98	5.24	5.29	5.50	5.48
10.52	5.20	4.95	5.36	5.15	5.13	5.05	5.02	5.45	5.34	5.33	5.58

10.72	5.21	5.52	5.14	5.19	4.76	4.64	4.81	4.86	5.14	5.33	5.49
10.92	6.14	6.04	5.52	5.54	5.46	5.02	5.50	5.45	5.46	5.59	5.99
11.12	5.41	5.59	5.19	4.99	4.65	5.03	5.10	5.32	5.15	5.46	5.41
11.32	5.62	5.76	5.20	5.08	5.10	5.27	5.00	5.21	5.10	5.82	5.97
11.52	5.51	5.32	5.45	5.11	5.22	5.52	5.22	5.44	4.99	5.67	5.69
11.72	5.38	5.18	5.09	5.22	4.72	4.94	4.79	5.02	5.27	5.89	5.63
11.92	5.73	5.77	5.80	5.14	5.49	5.39	5.54	5.45	5.24	6.03	5.87
12.12	5.49	5.36	5.16	4.99	4.90	5.08	5.03	5.08	5.25	5.60	5.19
12.32	5.60	5.40	5.44	5.28	4.92	5.14	5.12	5.19	4.95	5.36	5.50
12.52	5.06	5.31	5.11	4.94	5.12	5.18	4.80	4.97	5.17	5.60	5.66
12.72	5.14	5.28	4.84	5.01	5.13	4.69	4.87	5.03	5.03	5.22	5.62
12.92	5.56	5.46	5.37	5.18	5.32	5.26	5.26	5.16	5.33	5.42	5.22
13.12	5.90	5.56	5.76	5.55	5.22	5.37	5.29	5.50	5.28	5.61	6.06
13.32	5.46	5.30	5.02	4.94	4.90	4.76	5.06	5.22	5.10	5.40	5.51
13.52	5.50	5.52	5.17	5.23	5.11	5.08	5.18	5.31	5.33	5.49	5.16
13.72	5.20	5.28	5.37	5.10	4.87	5.12	5.25	5.34	5.11	5.44	5.83
13.92	4.92	5.33	5.02	4.83	4.84	5.15	4.99	5.65	5.24	5.47	5.60
14	5.79	5.49	5.52	5.28	5.37	5.02	5.45	5.37	5.05	5.64	5.69
14.08	5.70	5.64	5.47	5.39	4.92	5.15	5.31	5.32	5.12	5.59	6.10
14.17	5.46	5.37	5.10	5.30	5.10	4.82	4.66	5.35	4.99	5.87	5.27
14.27	5.67	5.87	5.56	5.33	5.54	5.12	5.21	5.14	5.37	5.83	5.68
14.38	5.05	5.12	4.77	4.75	4.82	5.09	4.89	5.08	4.67	4.87	5.54
14.49	5.54	5.17	5.36	5.05	5.11	5.15	5.09	5.08	5.00	5.38	5.67
14.61	5.97	5.97	5.73	5.81	5.46	5.30	5.18	5.54	5.33	5.85	5.83
14.75	5.50	5.64	5.43	5.23	4.96	4.95	5.09	5.03	5.28	5.32	5.66
14.89	5.54	5.68	5.51	5.28	5.50	5.13	5.30	5.42	5.48	5.42	5.78
15.05	5.26	5.17	5.49	5.00	5.26	5.23	5.12	5.36	5.24	5.51	5.85
15.21	5.18	5.11	4.89	4.79	4.76	4.78	4.84	4.98	5.08	5.62	5.72
15.39	5.63	5.62	5.56	5.34	5.31	5.09	5.32	5.16	4.91	5.43	5.58
15.59	5.55	5.79	5.41	5.09	5.36	5.35	5.22	4.92	4.91	5.59	5.32
15.8	5.32	5.31	5.16	4.66	4.70	4.81	4.77	4.77	4.94	5.23	5.45
16.02	5.55	5.44	5.49	5.47	5.30	5.35	4.96	4.92	5.46	5.60	5.52
16.27	5.18	5.07	4.88	4.83	4.98	5.05	4.60	5.33	4.94	5.52	5.71
16.53	5.24	5.25	5.24	5.07	4.79	4.78	5.07	4.93	5.02	5.24	5.44
16.81	5.75	5.73	5.34	5.39	5.11	5.33	4.70	5.27	4.89	5.22	5.26
17.12	5.43	5.37	5.19	5.05	5.06	5.02	4.95	5.39	5.00	5.15	5.49
17.45	5.53	5.54	5.47	5.55	5.29	5.24	5.19	5.21	5.10	5.60	5.93
17.81	5.32	5.25	4.96	5.29	4.92	4.76	4.68	5.00	4.85	5.16	5.50
18.19	4.99	4.95	4.74	4.57	4.74	4.61	4.65	4.81	4.61	4.95	5.02
18.6	5.66	5.51	5.39	5.14	4.93	4.88	4.68	5.28	4.84	5.36	5.01
19.05	5.74	5.43	5.23	5.40	5.15	4.69	4.84	5.29	4.71	5.32	5.28
19.53	5.53	5.49	5.05	5.05	4.98	5.41	4.70	5.36	5.21	5.51	5.57
20.05	5.75	5.39	5.25	5.08	4.84	5.12	5.22	5.20	5.27	5.19	5.27
20.61	5.04	5.12	5.16	5.00	4.71	5.05	4.84	5.26	4.87	5.43	5.31
21.22	5.30	5.16	4.89	4.69	4.54	4.60	4.71	4.79	5.15	4.98	5.25
21.87	5.99	5.90	5.22	5.31	5.49	4.96	5.29	5.09	4.96	5.42	5.41
22.57	5.55	5.51	5.26	5.14	5.16	4.92	4.96	4.64	4.99	5.03	5.62
23.33	5.07	5.19	5.12	4.90	4.72	4.87	4.55	5.12	4.94	5.12	5.47
24.15	5.48	5.13	5.13	4.87	4.69	4.84	4.74	5.38	4.83	5.28	5.33
25.03	4.96	4.93	4.80	4.83	4.82	4.69	4.20	4.93	4.89	4.97	5.34

25.98	5.36	5.42	5.35	4.94	4.85	5.01	4.82	4.79	4.70	5.10	5.45
27.01	5.87	5.70	5.39	5.20	4.82	4.97	5.26	4.57	4.81	5.40	5.07
28.11	4.94	5.19	4.83	4.62	4.76	4.61	4.62	5.24	4.75	5.22	5.46
29.3	5.21	5.35	5.12	5.18	4.81	4.78	4.72	4.90	5.22	5.19	5.20
30.59	5.23	5.11	4.97	5.05	4.93	4.81	4.70	4.46	4.75	5.03	4.99
31.98	5.15	4.90	5.13	4.73	4.63	4.52	4.73	4.79	4.67	5.03	5.33
33.47	5.39	5.35	5.15	5.07	4.80	4.99	4.50	4.85	5.05	5.08	5.30
35.09	5.36	5.12	4.96	4.54	4.95	5.14	4.41	5.20	4.78	5.08	5.24
36.83	5.33	5.39	5.09	4.76	4.78	4.88	5.04	5.06	5.07	5.09	5.06
38.71	5.23	5.15	5.10	4.80	4.77	4.94	4.77	4.84	5.13	5.34	4.96
40.73	4.80	4.83	4.93	4.52	4.87	4.95	4.71	4.77	4.48	5.19	5.33
42.92	5.03	5.11	5.14	4.60	4.40	4.96	4.97	5.03	4.90	5.20	5.12
45.27	5.75	5.55	5.47	5.21	4.87	4.99	4.99	4.77	4.76	5.26	5.12
47.81	4.92	5.34	4.79	4.65	4.59	4.79	4.89	4.63	4.55	5.05	5.08
50.55	5.28	5.29	5.12	4.71	4.72	4.68	5.02	4.83	4.83	5.03	5.24
53.51	4.74	4.75	4.57	4.62	4.45	3.99	3.98	4.48	4.14	4.45	4.45
56.69	4.83	4.57	4.67	4.30	4.46	4.57	4.54	4.51	3.98	4.84	4.87
60.13	5.35	5.29	5.28	5.27	4.94	4.86	4.87	4.60	4.95	5.11	4.79
63.84	5.31	4.99	5.02	4.53	4.69	4.63	4.46	4.97	4.78	4.66	4.71
67.83	4.96	4.92	4.97	4.57	4.51	4.36	4.30	4.53	4.76	4.86	4.81
72.15	5.31	5.13	5.05	4.80	4.95	4.95	4.39	4.95	4.68	4.60	4.94
76.8	4.82	4.57	4.53	4.62	4.49	4.60	4.48	4.41	4.24	4.73	4.55
81.81	4.92	4.96	4.89	4.60	4.60	4.39	4.41	4.65	4.56	4.62	5.08
87.22	5.15	5.30	5.24	5.08	4.94	4.49	4.64	4.91	4.61	4.96	5.18
93.06	4.77	4.84	5.05	4.69	4.27	4.44	4.45	4.81	5.01	4.76	4.88
99.35	5.16	4.80	4.92	4.75	4.69	4.93	4.55	4.75	4.64	5.21	5.02
106.13	4.98	4.85	4.58	4.76	4.33	4.64	4.42	4.41	4.35	4.61	5.13
113.45	4.63	4.79	4.88	4.71	4.17	4.69	4.37	4.40	4.63	4.56	4.83
121.34	5.16	5.04	4.79	4.62	4.29	4.72	4.71	4.81	4.69	4.48	5.04
129.86	5.08	4.87	4.55	4.14	4.36	4.33	4.28	4.86	4.11	4.43	4.67
139.04	4.94	4.62	4.57	4.39	4.15	4.39	4.27	4.38	4.55	4.66	4.73
148.94	5.05	5.11	4.68	4.67	4.76	4.47	4.47	4.47	4.50	4.65	4.97
159.62	4.65	4.79	4.64	4.45	4.47	4.46	4.29	4.44	4.65	4.50	4.80
171.14	4.39	4.52	4.36	4.24	4.08	4.38	3.82	3.98	4.04	4.34	4.56
183.56	4.84	5.19	4.91	4.50	4.53	4.26	4.57	4.61	4.18	4.74	4.52
196.96	4.63	4.60	4.48	4.13	4.15	4.42	4.33	4.30	4.25	4.47	4.68
211.41	4.82	4.92	4.68	4.53	4.17	4.52	4.14	4.66	4.36	4.34	4.77
226.99	4.64	4.79	4.68	4.48	4.42	4.34	4.29	4.42	4.53	4.68	4.79
243.8	4.37	4.63	4.27	4.14	4.05	4.16	4.28	4.25	4.10	4.35	4.37
261.93	4.58	4.62	4.46	4.08	3.99	4.31	4.07	4.04	4.23	4.03	4.29
281.48	4.67	4.79	4.65	4.50	4.40	4.37	4.19	4.44	4.30	4.73	4.54
302.56	4.25	4.38	4.11	4.18	4.10	3.64	3.71	3.77	4.11	4.14	4.04
325.3	4.64	4.78	4.37	4.56	4.15	4.18	4.18	4.15	4.38	4.42	4.43
349.83	4.35	4.48	4.20	4.27	3.79	4.19	3.94	4.09	4.19	4.15	4.33
376.28	4.30	4.13	4.10	4.17	3.72	3.60	3.88	3.71	3.74	4.04	4.19
404.81	4.96	4.91	4.61	4.42	4.36	4.04	4.15	4.28	3.87	4.34	4.12
435.58	4.49	4.54	4.34	4.13	3.91	4.01	3.72	3.86	3.83	4.09	3.93
468.76	4.18	4.01	4.18	4.12	4.22	3.74	3.71	3.88	3.62	3.78	3.92
504.55	4.29	4.20	4.22	4.05	4.09	3.80	4.08	3.79	3.46	3.88	4.15
543.15	4.03	3.99	3.76	3.81	3.36	3.41	3.43	3.59	3.55	3.89	3.74

584.78	4.38	4.32	4.06	3.81	3.72	3.84	3.50	3.79	3.61	3.74	3.91
629.68	4.62	4.49	4.29	3.87	3.74	3.92	3.54	3.92	3.41	3.55	4.20
678.1	4.27	4.16	3.65	3.58	3.52	3.44	3.61	3.84	3.61	3.85	3.87
730.33	4.19	3.98	3.99	3.79	4.04	3.88	4.07	3.55	3.35	3.94	3.93
786.65	4.56	4.44	4.05	4.03	3.66	3.52	3.39	3.69	3.63	3.50	4.05
847.4	4.01	4.07	3.71	3.53	3.78	3.51	3.38	3.40	3.50	3.65	3.68
912.92	4.40	4.27	4.02	3.95	4.02	3.69	3.45	3.71	3.83	3.58	3.71
983.58	3.97	4.14	3.85	3.94	3.43	3.51	3.51	3.35	3.44	3.53	3.77
1059.78	4.10	4.01	3.83	3.49	3.74	3.72	3.72	3.76	3.54	3.84	4.02
1141.98	4.33	4.11	3.90	3.63	3.62	3.81	3.56	3.59	3.46	3.82	3.73
1230.62	3.57	3.46	3.67	3.45	3.35	3.19	3.48	3.78	2.91	3.64	3.48
1326.23	4.24	3.97	3.84	3.67	3.62	3.54	3.34	3.58	3.57	3.49	3.74
1429.34	4.29	4.20	4.14	3.70	3.91	3.52	3.34	3.62	3.78	3.57	4.08
1540.54	3.95	3.88	3.64	3.55	3.68	3.39	3.31	3.21	3.00	3.52	3.47
1660.48	3.91	4.07	3.80	3.78	3.51	3.89	3.68	3.86	3.72	3.27	3.56
1789.83	4.02	4.16	3.94	3.62	3.67	3.61	3.46	3.43	3.30	3.15	3.60
1929.34	4.07	4.00	3.99	3.36	3.70	3.26	3.34	3.47	3.42	3.66	3.56
2079.8	4.18	4.00	3.98	3.44	3.64	3.49	3.20	3.52	3.27	3.35	3.66
2242.08	4.25	3.97	3.73	3.55	3.46	3.44	3.21	3.64	3.21	3.69	3.70
2417.1	3.93	3.59	3.64	3.34	3.24	3.36	3.34	3.43	3.57	3.46	3.48
2605.85	4.03	4.01	3.54	3.26	3.64	3.54	2.95	3.39	3.30	3.67	3.43
2809.43	3.80	3.57	3.53	3.35	3.48	3.11	3.19	3.08	3.04	3.54	3.47
3028.99	4.00	4.05	3.62	3.57	3.48	3.22	3.02	3.42	2.97	3.46	3.65
3265.79	4.05	3.93	3.75	3.68	3.13	3.44	3.22	3.18	3.31	3.63	3.56
3521.18	3.74	3.90	3.44	3.28	3.27	3.41	3.29	3.26	3.28	3.27	3.52
3796.62	3.83	3.85	3.68	3.50	3.56	3.29	3.47	3.36	3.10	3.58	3.60

Wavelength (nm) / Time (ps)	413.67	415.08	416.49	417.89	419.30	420.71	422.12	423.53	424.94	426.35	427.75
-3.28	-0.12	0.06	0.00	0.02	0.05	-0.07	0.13	0.13	0.17	-0.01	-0.05
-2.78	0.10	-0.09	0.14	-0.12	-0.01	0.08	0.28	0.14	0.04	0.01	0.05
-2.28	0.13	0.12	0.12	-0.03	0.05	0.04	-0.10	0.04	0.15	-0.01	0.13
-1.78	-0.22	0.05	-0.26	-0.02	-0.01	0.07	-0.12	-0.15	-0.15	-0.14	-0.02
-1.28	-0.05	-0.18	0.02	-0.07	-0.19	-0.10	-0.17	-0.31	-0.35	-0.04	-0.06
-0.78	0.18	0.04	0.21	0.06	0.28	0.15	0.05	0.08	0.26	0.26	0.15
-0.28	0.11	0.03	-0.23	-0.03	-0.11	-0.15	-0.06	-0.05	-0.02	-0.22	-0.18
0.22	-0.14	-0.02	0.01	0.17	-0.06	-0.02	-0.02	0.12	-0.10	0.15	-0.02
0.32	0.44	0.22	0.34	0.18	-0.06	0.24	0.15	0.16	0.20	0.12	0.21
0.42	0.33	0.24	-0.06	-0.02	0.02	0.11	0.04	0.14	0.13	0.18	-0.04
0.52	0.03	0.07	0.08	-0.13	0.00	-0.12	-0.35	-0.05	-0.04	-0.05	0.00
0.62	-0.08	0.10	0.07	-0.04	-0.06	0.08	0.00	0.05	0.32	0.11	0.26
0.72	-0.12	-0.23	-0.15	-0.16	-0.02	-0.08	-0.13	-0.20	0.10	0.00	-0.06
0.77	0.07	0.23	0.40	0.11	0.29	0.14	0.24	0.18	0.27	0.27	0.33
0.82	0.09	0.31	-0.24	-0.04	-0.18	-0.17	-0.09	-0.11	0.06	0.01	0.12
0.87	0.13	0.10	0.14	0.02	0.01	-0.05	0.12	0.25	0.08	0.17	0.03
0.92	0.13	0.00	0.11	-0.04	-0.13	-0.02	0.04	0.13	0.08	0.04	-0.09
0.97	-0.11	-0.14	-0.13	-0.26	-0.09	-0.02	-0.27	-0.21	0.00	-0.30	-0.22
1.02	0.31	0.40	0.21	0.00	-0.09	0.19	0.09	-0.17	0.16	-0.03	0.05

1.07	1.45	1.38	1.19	0.69	0.14	-0.12	-0.40	-0.33	-0.15	-0.22	-0.01
1.12	8.51	8.15	7.88	6.78	5.63	4.33	2.85	1.70	0.74	0.44	0.18
1.17	10.88	11.20	11.33	11.94	12.10	12.43	12.21	11.81	11.45	11.03	9.95
1.22	11.53	11.75	12.00	12.58	12.67	13.09	13.06	12.89	13.07	12.67	12.45
1.27	10.64	11.18	11.37	11.63	11.85	12.29	12.51	12.70	12.75	12.58	12.82
1.32	10.88	11.11	11.75	11.89	11.99	12.22	12.35	12.32	12.43	12.41	12.53
1.37	10.35	10.81	10.95	11.18	11.16	11.30	11.46	11.21	11.39	11.66	11.39
1.42	9.15	9.87	10.06	10.38	10.23	10.52	10.42	10.31	10.75	10.50	10.50
1.47	9.26	9.54	9.79	9.90	10.11	10.11	10.12	10.01	10.07	10.29	10.04
1.52	8.56	9.30	9.58	9.85	9.65	9.78	9.67	9.49	9.62	9.70	9.30
1.57	9.23	9.25	9.57	9.56	9.41	9.53	9.49	9.31	9.50	9.46	9.46
1.62	8.97	9.28	9.34	9.57	9.66	9.70	9.40	9.43	9.46	9.44	9.25
1.67	8.15	8.37	8.65	8.64	8.67	8.74	8.61	8.65	8.82	8.55	8.50
1.72	7.98	7.92	8.12	8.35	8.18	8.21	8.14	8.25	8.21	8.13	7.82
1.77	7.86	8.00	8.02	8.11	8.00	7.99	7.98	8.00	7.97	8.02	8.04
1.82	7.68	8.02	8.45	8.55	8.31	8.36	8.16	8.33	8.31	8.23	8.27
1.87	7.68	7.75	8.29	8.13	8.20	8.23	8.10	7.93	7.98	7.77	8.02
1.92	7.90	8.06	7.95	8.23	8.11	8.36	8.19	8.06	8.33	8.04	8.06
1.97	7.25	7.74	7.84	7.86	7.68	8.06	7.86	7.64	7.79	7.63	7.59
2.02	7.50	7.55	7.55	7.60	7.60	7.61	7.49	7.32	7.85	7.49	7.37
2.07	7.42	7.76	7.68	7.91	7.63	8.02	7.77	7.71	7.82	7.73	7.56
2.12	7.10	7.08	7.46	7.52	7.62	7.65	7.63	7.48	7.50	7.35	7.40
2.17	7.76	7.41	7.79	8.06	8.01	7.90	7.90	7.80	7.77	7.69	7.52
2.22	6.85	7.12	7.32	7.70	7.25	7.25	7.50	7.32	7.19	7.19	7.17
2.27	7.62	7.63	7.84	7.94	7.96	7.88	7.79	7.63	7.67	7.63	7.55
2.32	7.01	7.11	7.59	7.46	7.45	7.44	7.26	7.35	7.27	7.10	7.15
2.37	7.08	7.01	7.37	7.48	7.45	7.63	7.52	7.27	7.39	7.18	7.35
2.42	7.17	7.23	7.46	7.61	7.39	7.49	7.54	7.32	7.40	7.40	7.37
2.47	7.22	7.14	7.58	7.42	7.37	7.45	7.59	7.43	7.28	7.34	7.02
2.52	6.76	7.11	7.21	7.01	6.91	7.08	7.06	6.75	7.15	6.79	6.85
2.57	6.72	6.82	7.06	7.14	6.88	7.03	7.00	6.86	6.95	6.84	6.83
2.62	6.84	6.88	7.12	7.19	7.09	7.18	7.17	6.89	7.14	6.85	6.97
2.67	6.86	7.01	7.21	7.31	6.98	7.17	6.94	6.92	6.99	6.85	6.94
2.72	6.87	7.13	7.14	7.16	7.29	7.49	7.22	7.08	6.98	7.04	6.93
2.77	6.61	6.83	7.05	7.22	6.98	7.15	6.90	6.77	6.68	6.66	6.73
2.82	6.92	7.03	7.31	7.37	7.30	7.25	7.10	7.07	7.11	6.99	6.98
2.87	6.50	6.67	7.01	7.00	7.04	7.02	6.89	6.88	6.83	6.83	6.75
2.92	7.17	6.82	7.27	7.11	7.31	7.07	7.01	6.96	7.12	7.10	7.04
2.97	6.61	6.72	7.21	6.95	6.95	7.20	6.91	6.93	6.84	6.87	6.90
3.02	6.77	7.04	7.12	7.20	7.21	7.29	7.02	7.25	7.31	6.88	6.75
3.07	6.23	6.81	6.69	6.91	6.88	6.70	6.91	6.64	6.71	6.60	6.51
3.12	6.96	7.18	7.42	7.40	7.27	7.49	7.31	7.15	7.07	6.99	6.91
3.17	6.50	6.57	6.96	6.82	6.87	6.90	6.80	6.48	6.88	6.62	6.15
3.22	6.94	7.09	7.28	7.29	7.15	7.27	7.19	7.04	6.99	7.10	6.88
3.27	6.61	7.30	7.17	7.30	7.17	7.13	7.00	7.07	6.93	7.08	7.00
3.32	7.03	7.52	6.97	7.33	7.31	7.28	7.22	7.24	7.20	7.21	6.94
3.37	6.79	7.07	7.02	7.18	7.19	7.13	7.05	6.89	7.05	6.97	6.80
3.42	6.63	6.78	6.79	6.66	6.76	6.64	6.91	6.67	6.52	6.52	6.54
3.47	6.75	6.81	6.88	6.99	7.01	7.13	6.99	6.86	6.89	6.86	6.67
3.52	6.58	7.04	7.30	7.05	6.97	7.31	7.06	6.89	6.91	6.71	6.64

3.57	6.79	7.18	7.18	7.27	7.14	7.37	6.98	7.04	6.89	6.75	6.69
3.62	6.52	6.65	6.88	6.65	6.60	6.78	6.62	6.59	6.75	6.63	6.47
3.67	6.30	6.61	6.86	6.89	6.71	6.90	6.65	6.64	6.89	6.75	6.42
3.72	6.81	6.83	6.96	6.84	6.91	7.10	7.11	6.95	7.03	6.84	6.89
3.92	6.16	6.60	6.60	6.50	6.55	6.63	6.58	6.57	6.53	6.33	6.17
4.12	6.73	6.99	6.78	6.94	6.89	7.21	6.88	6.94	6.55	6.75	6.41
4.32	6.45	6.62	7.22	6.93	7.02	7.33	6.93	7.01	6.97	6.94	6.94
4.52	6.68	6.63	6.93	6.95	7.02	7.08	6.95	6.94	7.17	6.93	6.73
4.72	6.29	6.40	6.53	6.73	6.60	6.87	6.57	6.53	6.68	6.42	6.35
4.92	6.58	6.40	6.70	6.67	6.80	6.69	6.49	6.66	6.76	6.57	6.45
5.12	6.63	6.54	6.87	6.72	6.63	6.70	6.77	6.41	6.55	6.36	6.41
5.32	6.55	6.74	7.04	7.10	6.92	6.92	6.89	6.71	6.73	6.59	6.50
5.52	6.33	6.82	6.78	6.52	6.67	6.81	6.69	6.54	6.54	6.50	6.37
5.72	6.51	6.51	6.51	6.18	6.60	6.47	6.54	6.23	6.23	6.30	6.17
5.92	6.40	6.39	6.55	6.64	6.69	6.87	6.72	6.60	6.67	6.51	6.30
6.12	6.01	6.34	6.56	6.40	6.36	6.50	6.39	6.14	6.25	6.39	6.09
6.32	6.62	6.59	6.65	6.94	6.81	6.60	6.45	6.45	6.30	6.37	6.27
6.52	6.38	6.71	6.75	6.96	6.88	6.84	6.97	6.95	6.66	6.67	6.67
6.72	5.96	6.15	6.29	6.25	6.31	6.39	6.10	6.09	6.14	5.97	6.03
6.92	6.02	6.23	6.12	6.42	6.23	6.38	6.11	6.38	6.13	6.27	6.10
7.12	6.05	6.34	6.42	6.44	6.25	6.49	6.48	6.22	6.27	6.16	6.31
7.32	6.01	6.09	6.57	6.62	6.44	6.60	6.58	6.46	6.59	6.45	6.48
7.52	6.46	6.50	6.64	6.70	6.69	6.70	6.63	6.63	6.64	6.47	6.47
7.72	6.08	6.16	6.34	6.31	6.17	6.34	6.52	6.08	6.26	6.15	5.90
7.92	6.20	6.64	6.60	6.81	6.48	6.56	6.44	6.40	6.52	6.32	6.37
8.12	5.90	6.20	6.18	6.37	6.27	6.21	6.16	6.13	6.01	5.99	5.78
8.32	6.30	6.53	6.57	6.62	6.51	6.58	6.36	6.14	6.50	6.41	6.31
8.52	5.90	6.27	6.39	6.49	6.72	6.54	6.33	6.32	6.37	6.35	6.37
8.72	6.17	6.61	6.38	6.34	6.42	6.63	6.49	6.34	6.57	6.32	6.36
8.92	6.08	6.13	6.13	6.36	6.10	6.24	6.19	5.92	6.19	6.08	5.79
9.12	6.11	6.50	6.43	6.45	6.25	6.21	6.48	6.34	6.27	6.25	6.08
9.32	5.96	5.63	6.26	6.15	5.99	5.99	6.02	5.78	5.87	5.86	5.74
9.52	5.96	6.20	6.34	6.20	6.26	6.43	6.29	6.10	6.14	6.08	5.96
9.72	6.09	6.17	6.70	6.44	6.59	6.50	6.72	6.33	6.29	6.23	6.14
9.92	6.05	6.13	6.18	6.39	6.26	6.32	6.23	6.17	6.26	6.10	5.94
10.12	6.12	6.37	6.54	6.58	6.56	6.64	6.51	6.61	6.40	6.42	6.05
10.32	6.11	5.92	6.23	6.43	6.41	6.37	6.19	6.35	6.31	6.19	6.13
10.52	5.87	6.25	6.11	6.13	6.10	6.03	6.12	6.13	6.17	6.06	5.88
10.72	5.91	5.69	5.77	6.04	5.93	5.84	6.05	5.96	5.94	5.96	5.68
10.92	6.07	6.36	6.33	6.53	6.53	6.51	6.35	6.36	6.36	6.34	6.27
11.12	5.88	6.27	6.38	6.09	6.11	6.30	6.35	6.08	6.28	6.15	5.99
11.32	6.05	6.28	6.19	6.38	6.19	6.30	6.33	6.20	6.21	5.83	6.00
11.52	6.26	6.02	6.21	6.42	6.32	6.43	6.42	6.18	6.36	6.21	6.26
11.72	5.49	6.17	6.35	6.22	6.07	6.10	6.26	6.08	6.15	6.04	5.88
11.92	5.97	6.37	6.44	6.37	6.50	6.28	6.47	6.35	6.25	6.30	6.30
12.12	5.78	6.24	6.27	6.17	6.13	6.02	6.03	6.11	6.23	6.20	5.97
12.32	6.04	6.22	6.03	6.28	6.26	6.53	6.50	6.46	6.34	6.02	5.92
12.52	5.84	6.01	6.03	6.20	6.26	6.14	6.13	6.10	6.22	6.03	5.91
12.72	5.51	6.07	6.08	6.00	6.26	6.23	6.12	6.01	6.12	5.93	5.87
12.92	6.05	5.72	5.94	6.05	6.03	6.22	6.13	5.87	6.07	5.90	5.80

13.12	5.96	6.21	6.52	6.71	6.39	6.32	6.39	6.30	6.44	6.20	6.16
13.32	5.51	5.82	6.10	6.00	6.17	5.86	6.02	5.98	6.04	5.84	5.86
13.52	5.87	6.10	6.36	6.15	6.14	6.20	6.04	6.00	5.91	5.84	5.89
13.72	5.92	5.91	5.99	6.12	5.83	6.21	6.07	5.92	5.88	5.85	5.82
13.92	5.91	6.03	6.17	6.16	6.28	6.19	6.29	5.93	6.33	5.99	5.93
14	5.61	5.82	6.06	6.44	6.11	6.27	6.43	6.32	6.13	6.10	5.92
14.08	6.24	6.27	6.38	6.37	6.56	6.47	6.24	6.02	6.28	6.09	5.98
14.17	6.02	6.00	6.13	6.31	6.05	6.55	6.25	6.16	6.10	6.18	5.91
14.27	6.11	6.02	6.09	6.30	6.14	6.11	6.23	6.28	6.16	6.21	5.95
14.38	5.81	6.06	6.10	6.14	5.91	6.04	5.87	5.65	6.02	5.89	5.78
14.49	5.75	5.93	6.20	6.16	6.01	5.87	6.01	5.91	5.92	5.76	5.59
14.61	6.24	6.20	6.32	6.23	6.21	6.35	6.26	6.05	6.23	5.98	6.20
14.75	5.98	5.73	5.99	6.19	5.78	6.09	6.06	5.94	5.95	5.93	5.91
14.89	6.21	6.17	6.45	6.34	6.36	6.45	6.40	6.23	6.24	6.18	5.97
15.05	5.66	6.16	6.19	6.29	6.19	6.33	6.09	5.96	6.21	5.99	5.98
15.21	5.89	6.18	6.27	6.12	6.09	6.12	6.04	5.96	5.97	5.93	5.88
15.39	5.79	5.93	6.31	6.13	5.68	6.10	6.08	5.95	6.03	5.83	5.77
15.59	6.08	6.20	6.19	6.16	6.05	6.21	6.21	6.19	6.01	6.01	5.92
15.8	5.53	5.63	5.71	5.79	5.84	6.06	5.91	6.07	5.69	5.67	5.83
16.02	6.07	6.21	6.23	6.17	5.99	6.30	6.22	6.03	6.19	5.98	5.70
16.27	5.82	5.92	6.15	5.80	6.04	6.02	5.95	6.07	5.97	5.90	5.81
16.53	5.88	6.05	6.22	5.97	6.11	6.17	6.05	5.75	5.92	5.81	5.66
16.81	5.85	5.86	6.09	5.95	6.15	6.15	6.23	6.20	6.04	6.01	6.06
17.12	5.92	5.81	6.33	6.15	6.27	6.09	5.99	5.74	5.99	5.82	5.87
17.45	6.17	6.19	6.14	6.19	6.46	6.28	6.10	6.02	6.05	6.19	5.94
17.81	5.62	5.91	5.89	6.10	5.95	6.21	6.15	6.00	6.00	5.95	5.65
18.19	5.28	5.45	5.57	5.80	5.86	5.91	5.86	5.62	6.07	5.78	5.62
18.6	5.40	5.76	5.78	5.93	5.67	5.84	5.72	5.77	5.77	5.80	5.56
19.05	5.34	5.59	6.09	5.87	5.88	5.68	5.89	5.81	5.80	5.48	5.62
19.53	6.10	5.99	6.13	6.23	6.15	6.14	6.04	6.06	6.22	5.89	5.81
20.05	5.68	5.58	6.02	5.94	5.80	5.82	5.81	5.87	5.94	5.67	5.62
20.61	5.53	5.87	5.95	5.93	6.09	5.88	6.01	6.12	5.91	5.93	5.66
21.22	5.44	5.78	5.92	5.60	5.79	5.73	5.64	5.73	5.51	5.71	5.47
21.87	5.82	5.96	5.91	5.87	5.89	5.94	6.16	5.96	6.05	5.95	5.81
22.57	5.36	5.73	5.88	6.02	5.74	5.95	5.86	5.94	5.98	5.64	5.66
23.33	5.80	6.03	5.81	6.03	5.95	6.03	5.66	5.69	5.88	5.65	5.86
24.15	5.60	5.67	5.64	5.79	5.86	5.73	5.80	5.53	5.76	5.64	5.54
25.03	5.25	5.31	5.80	5.47	5.87	5.64	5.83	5.67	5.68	5.45	5.54
25.98	5.84	5.96	5.84	5.87	5.82	5.91	5.77	5.62	5.79	5.66	5.43
27.01	5.46	5.30	5.78	5.66	5.63	5.43	5.29	5.39	5.52	5.30	5.27
28.11	5.58	5.84	5.88	6.04	5.90	5.80	6.01	5.63	5.77	5.75	5.57
29.3	5.50	5.70	5.76	6.06	5.73	5.97	5.70	5.62	5.78	5.50	5.45
30.59	5.40	5.54	5.84	5.70	5.61	5.71	5.72	5.56	5.38	5.38	5.11
31.98	5.50	6.12	5.94	5.86	5.86	6.09	5.85	5.86	6.02	5.70	5.51
33.47	5.21	5.76	5.81	5.86	5.83	6.07	5.76	5.70	5.57	5.72	5.56
35.09	5.57	5.62	5.95	5.58	5.91	5.79	5.77	5.81	5.89	5.79	5.52
36.83	5.49	5.57	5.67	5.53	5.60	5.61	5.63	5.62	5.75	5.53	5.41
38.71	5.99	5.75	5.88	5.69	5.53	5.64	5.83	5.58	5.66	5.66	5.37
40.73	5.25	5.34	5.52	5.61	5.61	5.51	5.66	5.38	5.45	5.52	5.39
42.92	5.42	5.58	5.79	5.82	5.81	5.81	5.72	5.62	5.69	5.54	5.56

45.27	5.47	5.64	5.63	5.70	5.78	5.70	5.87	5.79	5.75	5.65	5.80
47.81	5.18	5.41	5.63	5.58	5.59	5.53	5.41	5.47	5.44	5.57	5.02
50.55	5.40	5.84	5.83	5.60	5.50	5.64	5.63	5.43	5.74	5.39	5.17
53.51	4.99	4.63	5.08	5.07	4.92	5.17	4.99	5.15	5.09	5.09	4.91
56.69	5.16	5.25	5.25	5.28	5.10	5.30	5.26	5.24	5.19	5.11	4.86
60.13	5.41	5.35	5.53	5.46	5.63	5.41	5.53	5.30	5.28	5.24	5.04
63.84	5.19	5.33	5.55	5.42	5.50	5.46	5.49	5.11	5.25	5.09	5.12
67.83	5.03	4.95	5.12	5.10	5.36	5.38	5.09	5.26	5.28	5.09	4.91
72.15	5.13	5.27	5.13	5.33	5.21	5.16	5.16	5.13	5.47	5.10	5.04
76.8	4.78	5.07	5.33	5.03	4.98	5.10	5.04	5.06	5.12	5.01	4.82
81.81	5.32	5.24	5.48	5.30	5.37	5.33	5.39	5.34	5.29	5.19	5.05
87.22	5.58	5.66	5.69	5.79	5.66	5.50	5.39	5.46	5.29	5.16	4.99
93.06	5.22	5.45	5.63	5.45	5.52	5.48	5.34	5.44	5.41	5.24	5.19
99.35	5.31	5.45	5.71	5.40	5.52	5.58	5.54	5.32	5.52	5.19	5.05
106.13	5.04	4.90	5.10	5.04	5.21	5.20	5.22	4.92	4.97	5.17	4.87
113.45	5.03	5.25	5.36	5.35	5.37	5.36	5.33	5.29	5.18	5.31	5.18
121.34	5.28	5.38	5.43	5.43	5.06	5.42	5.26	5.12	5.09	5.38	5.13
129.86	4.80	5.06	5.15	5.11	5.03	5.02	4.90	5.01	5.04	4.99	4.69
139.04	4.87	4.75	5.10	5.20	4.82	5.03	5.01	4.94	5.00	4.97	4.83
148.94	4.98	5.18	5.17	5.26	5.22	5.12	5.06	4.99	5.06	4.92	5.01
159.62	4.57	5.18	5.35	5.09	5.05	5.18	5.03	4.91	4.89	4.81	4.84
171.14	4.81	5.06	5.16	5.10	5.08	5.17	5.15	4.96	5.11	4.76	4.76
183.56	4.66	4.78	4.90	4.88	4.74	4.78	5.17	4.76	4.85	4.82	4.58
196.96	4.57	4.97	5.07	5.01	4.98	4.97	5.03	4.86	5.05	4.92	4.74
211.41	4.80	4.98	5.02	5.10	4.93	4.98	4.81	4.81	5.05	4.92	4.70
226.99	4.90	4.95	5.04	4.91	4.91	5.19	5.12	4.99	5.03	4.81	4.81
243.8	4.60	4.89	4.85	5.13	4.80	4.68	4.52	4.68	4.76	4.45	4.53
261.93	4.53	4.65	4.71	5.02	4.77	4.96	4.90	4.75	5.00	4.66	4.63
281.48	4.77	5.10	5.29	5.12	5.13	5.19	5.03	5.06	5.16	5.08	4.88
302.56	4.63	4.63	4.64	4.63	4.57	4.85	4.68	4.48	4.64	4.65	4.47
325.3	4.35	4.79	4.74	4.79	4.94	4.85	4.94	4.71	4.67	4.69	4.45
349.83	4.31	4.30	4.60	4.55	4.58	4.50	4.70	4.44	4.51	4.36	4.36
376.28	4.52	4.38	4.72	4.61	4.35	4.72	4.57	4.47	4.63	4.60	4.32
404.81	4.41	4.66	4.66	4.81	4.65	4.82	4.65	4.58	5.01	4.71	4.60
435.58	4.24	4.26	4.35	4.32	4.47	4.35	4.48	4.32	4.41	4.14	4.12
468.76	4.39	4.55	4.46	4.86	4.39	4.48	4.50	4.51	4.40	4.65	4.49
504.55	4.37	4.39	4.66	4.65	4.54	4.62	4.61	4.71	4.57	4.38	4.50
543.15	4.20	4.33	4.04	4.33	4.26	4.33	4.32	4.33	4.27	4.10	4.15
584.78	4.34	4.29	4.26	4.32	4.54	4.50	4.70	4.35	4.48	4.44	4.30
629.68	4.31	4.32	4.24	4.41	4.25	4.49	4.43	4.40	4.39	4.11	4.25
678.1	4.14	4.35	4.62	4.63	4.41	4.55	4.66	4.49	4.39	4.37	4.21
730.33	4.41	4.16	4.35	4.33	4.27	4.24	4.29	4.26	4.28	4.06	4.03
786.65	3.93	3.97	4.12	4.18	4.15	4.17	4.16	3.95	4.20	3.97	4.05
847.4	3.91	3.93	3.81	3.93	3.89	4.01	4.08	3.83	3.88	3.81	3.70
912.92	3.84	3.94	4.22	4.07	3.95	3.99	4.23	4.11	3.98	4.06	3.73
983.58	4.21	3.73	4.20	4.09	3.96	4.23	4.25	4.03	4.15	4.03	3.99
1059.78	4.37	4.34	4.39	4.29	4.33	4.32	4.19	4.10	4.20	4.22	4.17
1141.98	4.06	4.12	4.25	4.22	4.16	4.13	4.14	4.05	4.31	3.99	3.84
1230.62	3.89	4.02	3.88	4.01	3.93	3.98	3.96	4.08	3.92	3.80	3.76
1326.23	3.99	4.00	4.10	4.31	3.88	4.16	4.33	4.16	4.41	4.10	4.11

1429.34	3.83	4.11	4.17	4.07	4.16	4.13	4.28	4.06	3.92	3.93	3.94
1540.54	3.75	3.96	3.80	3.85	3.84	3.96	3.90	3.66	3.77	3.73	3.56
1660.48	3.69	3.98	3.87	3.95	3.98	3.99	4.06	3.91	3.79	3.87	3.77
1789.83	3.56	3.88	4.05	3.88	3.77	3.97	3.71	3.68	3.64	3.42	3.49
1929.34	3.90	4.08	4.28	4.16	4.05	4.32	4.20	4.02	4.07	4.13	3.89
2079.8	3.84	3.97	3.89	4.01	3.77	3.99	3.75	3.80	4.05	3.78	3.54
2242.08	4.09	4.12	4.09	4.32	4.13	4.27	4.11	3.94	3.87	3.85	3.78
2417.1	3.72	3.79	3.79	3.97	3.68	3.90	3.89	3.85	3.87	3.51	3.58
2605.85	3.85	3.97	4.02	3.86	3.84	4.07	3.77	3.61	3.67	3.61	3.74
2809.43	3.97	3.98	3.89	3.87	3.84	3.85	3.72	3.67	3.74	3.78	3.52
3028.99	3.71	3.74	4.06	4.03	3.74	3.89	4.00	3.73	3.99	3.81	3.81
3265.79	3.78	4.00	3.87	3.83	3.68	3.86	3.83	3.51	3.69	3.57	3.52
3521.18	3.62	3.89	3.75	3.85	3.64	3.65	3.62	3.58	3.47	3.48	3.51
3796.62	3.64	3.48	3.78	4.00	3.67	4.05	3.68	3.74	3.64	3.40	3.26

Wavelength (nm) / Time (ps)	429.16	430.57	431.98	433.39	434.80	436.20	437.61	439.02	440.43	441.84	443.25
-3.28	0.11	0.19	0.02	0.24	0.02	0.05	0.31	0.14	0.21	0.08	0.07
-2.78	0.28	-0.08	0.20	0.14	0.23	0.14	0.08	-0.09	-0.01	0.10	0.15
-2.28	0.02	-0.06	0.10	-0.16	0.01	-0.30	-0.26	-0.07	-0.14	-0.06	-0.01
-1.78	-0.12	-0.11	-0.03	-0.01	0.04	0.11	-0.06	0.08	0.11	0.05	0.07
-1.28	-0.25	-0.06	-0.17	-0.21	-0.26	-0.02	-0.31	-0.17	-0.15	-0.11	-0.25
-0.78	0.03	0.29	0.11	0.01	0.04	0.17	0.15	0.13	0.12	0.17	0.12
-0.28	-0.17	-0.12	-0.31	-0.02	-0.22	-0.18	-0.08	-0.07	-0.27	-0.27	-0.19
0.22	0.11	-0.05	0.08	0.01	0.14	0.03	0.15	0.05	0.13	0.04	0.04
0.32	0.13	0.08	0.11	-0.01	0.16	0.00	0.15	-0.04	0.09	0.05	-0.20
0.42	0.11	-0.07	-0.25	-0.05	0.03	0.07	-0.03	0.08	-0.02	-0.22	-0.09
0.52	-0.22	-0.08	-0.21	-0.25	-0.09	-0.16	-0.26	-0.25	-0.24	-0.18	-0.33
0.62	0.12	0.25	0.35	0.25	0.21	0.30	0.22	0.10	0.12	0.10	-0.03
0.72	-0.24	-0.02	-0.23	-0.25	-0.23	-0.24	-0.16	-0.34	-0.48	-0.28	-0.22
0.77	0.22	0.36	0.13	0.30	0.22	0.05	0.10	0.06	0.06	0.19	-0.01
0.82	-0.07	0.11	-0.01	0.06	-0.05	0.05	0.21	0.10	0.16	0.18	0.16
0.87	0.09	0.13	-0.10	-0.08	0.01	0.15	-0.12	0.10	0.03	-0.06	-0.14
0.92	-0.13	0.09	-0.12	-0.09	0.03	0.00	-0.21	-0.08	-0.04	-0.04	-0.08
0.97	-0.24	-0.29	-0.20	-0.22	-0.17	-0.21	-0.14	-0.30	-0.21	-0.25	-0.12
1.02	0.10	0.30	0.11	-0.08	-0.10	-0.01	-0.03	0.09	-0.01	-0.22	-0.10
1.07	0.01	-0.03	0.12	0.01	-0.04	0.09	0.03	-0.14	-0.11	0.04	0.10
1.12	-0.15	-0.49	-0.49	-0.72	-0.66	-0.54	-0.28	-0.22	-0.25	-0.01	-0.12
1.17	8.85	8.07	6.78	5.54	4.20	3.01	2.01	0.97	0.50	-0.13	-0.38
1.22	12.53	12.22	11.82	11.38	11.04	10.71	10.02	9.25	8.43	7.74	6.77
1.27	12.53	12.79	12.46	12.40	12.28	11.72	11.48	11.07	10.80	10.23	9.57
1.32	12.66	12.67	12.85	12.62	12.63	12.29	12.21	12.19	11.84	11.46	11.04
1.37	11.44	11.48	11.18	10.89	10.82	10.74	10.42	10.32	10.03	9.79	9.57
1.42	10.39	10.40	10.37	10.09	9.94	9.62	9.23	9.41	8.90	8.41	8.07
1.47	10.07	9.92	9.84	9.75	9.35	9.27	8.86	8.82	8.44	8.15	7.70
1.52	9.54	9.36	9.27	9.11	8.79	8.88	8.46	8.21	7.77	7.29	7.01
1.57	9.07	8.98	8.79	8.67	8.40	8.20	8.02	7.60	7.28	6.87	6.63
1.62	9.30	9.08	8.76	8.72	8.34	8.49	7.96	7.68	7.38	7.20	6.80

1.67	8.38	8.28	8.14	8.07	7.70	7.71	7.33	7.10	6.84	6.35	6.24
1.72	7.82	7.71	7.53	7.44	7.35	7.02	6.73	6.45	6.16	6.04	5.71
1.77	7.85	7.79	7.49	7.37	7.15	6.87	6.66	6.63	6.18	5.78	5.48
1.82	7.95	7.96	7.79	7.61	7.31	7.06	6.67	6.77	6.30	5.92	5.43
1.87	7.75	7.62	7.60	7.23	7.04	6.85	6.59	6.32	6.19	5.75	5.45
1.92	7.81	7.69	7.69	7.22	6.91	6.95	6.56	6.54	6.25	5.82	5.47
1.97	7.55	7.36	7.19	6.98	6.76	6.69	6.17	6.08	5.97	5.38	5.05
2.02	7.26	7.29	6.98	6.89	6.52	6.51	6.14	5.86	5.84	5.32	5.11
2.07	7.45	7.48	7.08	6.91	6.74	6.28	6.28	6.05	5.76	5.15	5.04
2.12	7.19	7.23	6.87	6.61	6.82	6.36	6.13	5.98	5.51	5.18	4.90
2.17	7.61	7.63	7.33	7.01	6.82	6.50	6.44	6.18	5.91	5.34	5.15
2.22	7.36	6.89	6.78	6.49	6.26	6.04	6.00	5.48	5.57	5.21	4.88
2.27	7.56	7.32	7.06	6.85	6.72	6.44	6.12	5.83	5.49	5.02	4.86
2.32	7.11	7.03	6.82	6.64	6.45	6.16	5.91	5.79	5.38	5.10	4.74
2.37	7.00	6.98	6.78	6.74	6.55	6.31	6.05	5.83	5.41	5.09	4.88
2.42	7.09	6.97	6.95	6.60	6.43	6.36	6.12	5.75	5.42	5.17	4.92
2.47	7.07	6.86	6.64	6.67	6.47	6.43	6.08	5.79	5.51	5.38	5.13
2.52	6.58	6.53	6.40	6.27	5.81	5.64	5.43	5.40	5.00	4.56	4.30
2.57	6.45	6.35	6.22	6.00	5.83	5.84	5.51	5.33	5.08	4.66	4.22
2.62	6.64	6.61	6.21	6.20	5.94	5.68	5.48	5.30	4.90	4.73	4.46
2.67	6.78	6.52	6.38	6.21	5.86	5.78	5.62	5.33	5.08	4.68	4.45
2.72	6.84	6.61	6.40	6.10	6.04	5.83	5.55	5.20	5.00	4.65	4.38
2.77	6.49	6.29	6.08	5.86	5.63	5.53	5.20	5.04	4.81	4.42	4.29
2.82	6.89	6.80	6.64	6.33	6.05	5.86	5.73	5.42	5.29	4.81	4.53
2.87	6.57	6.47	6.23	5.92	5.87	5.52	5.23	5.22	4.94	4.46	4.22
2.92	6.65	6.62	6.54	6.32	6.10	5.97	5.55	5.26	5.24	4.69	4.48
2.97	6.53	6.46	6.13	5.96	5.91	5.45	5.36	5.17	4.84	4.53	4.31
3.02	6.87	6.69	6.31	6.12	6.04	5.75	5.58	5.40	4.87	4.56	4.33
3.07	6.40	6.12	6.05	6.04	5.66	5.26	5.00	4.83	4.81	4.35	4.26
3.12	6.90	6.85	6.58	6.38	6.09	5.80	5.75	5.51	5.22	4.87	4.53
3.17	6.50	6.25	6.22	5.91	5.74	5.49	5.22	5.17	4.90	4.45	4.17
3.22	6.63	6.69	6.59	6.24	5.92	5.69	5.61	5.18	5.04	4.69	4.22
3.27	6.66	6.33	6.42	6.12	5.80	5.82	5.45	5.39	5.06	4.77	4.54
3.32	6.88	6.83	6.68	6.55	6.13	5.82	5.94	5.46	5.16	4.93	4.72
3.37	6.47	6.37	6.18	6.00	5.79	5.46	5.23	5.03	4.96	4.56	4.17
3.42	6.22	6.18	5.92	5.70	5.34	5.16	5.11	4.83	4.52	4.10	3.91
3.47	6.53	6.70	6.31	6.01	5.87	5.55	5.40	5.11	5.03	4.66	4.13
3.52	6.61	6.42	6.30	5.93	5.58	5.61	5.21	5.07	4.77	4.29	4.20
3.57	6.49	6.33	6.03	5.94	5.85	5.58	5.40	5.34	4.90	4.53	4.17
3.62	6.35	6.21	5.98	5.63	5.51	5.24	5.01	4.89	4.38	4.36	4.10
3.67	6.24	6.10	6.06	5.84	5.55	5.43	5.21	4.91	4.34	4.48	3.97
3.72	6.72	6.40	6.36	6.11	6.02	5.59	5.67	5.13	4.91	4.66	4.14
3.92	6.04	5.84	5.63	5.43	5.19	5.04	4.77	4.55	4.40	3.95	3.50
4.12	6.54	6.29	6.05	5.97	5.53	5.43	4.98	5.01	4.66	4.42	4.11
4.32	6.57	6.59	6.48	6.15	5.82	5.66	5.53	5.05	5.02	4.65	4.13
4.52	6.69	6.78	6.34	6.00	5.81	5.54	5.56	5.06	4.94	4.44	4.23
4.72	6.16	6.08	5.90	5.64	5.41	5.41	4.92	4.73	4.46	4.20	4.13
4.92	6.27	6.28	6.07	5.85	5.44	5.25	5.04	4.90	4.53	4.22	4.13
5.12	6.08	6.23	5.69	5.53	5.53	5.29	4.99	4.72	4.46	4.10	3.72
5.32	6.28	6.20	6.04	5.72	5.65	5.14	5.01	4.91	4.67	4.14	3.70

5.52	6.26	6.03	5.87	5.85	5.43	5.39	5.05	4.72	4.42	4.03	3.69
5.72	5.98	5.96	5.59	5.52	5.18	5.01	4.67	4.53	4.18	3.81	3.55
5.92	6.20	6.35	6.05	5.75	5.51	5.30	5.03	4.75	4.62	4.21	3.95
6.12	5.95	5.89	5.45	5.55	5.19	4.95	4.52	4.57	4.19	3.76	3.72
6.32	5.97	5.93	5.49	5.48	5.25	5.07	4.88	4.44	4.15	3.99	3.69
6.52	6.29	6.23	6.13	5.77	5.46	5.42	5.21	4.64	4.49	4.40	3.85
6.72	5.86	5.59	5.68	5.29	5.22	4.87	4.61	4.47	4.22	3.57	3.60
6.92	5.71	5.59	5.54	5.28	4.95	4.79	4.61	4.30	4.14	3.82	3.60
7.12	6.01	5.89	5.75	5.35	5.25	4.97	4.81	4.66	4.31	3.92	3.81
7.32	6.23	6.04	5.77	5.78	5.29	4.93	4.88	4.63	4.61	4.01	3.87
7.52	6.34	6.31	6.04	5.72	5.53	5.21	5.13	4.68	4.68	4.31	4.23
7.72	5.79	5.97	5.49	5.07	4.79	5.03	4.62	4.32	4.18	3.60	3.31
7.92	6.10	6.04	5.64	5.60	5.05	4.87	4.90	4.56	4.27	4.23	3.80
8.12	5.88	5.79	5.55	5.19	5.18	4.77	4.51	4.49	4.09	3.65	3.51
8.32	6.00	6.19	5.79	5.43	5.41	5.15	4.81	4.70	4.53	4.07	3.85
8.52	6.02	5.91	5.88	5.51	5.21	5.08	5.00	4.55	4.29	3.92	3.72
8.72	6.19	6.00	5.57	5.35	5.19	4.98	4.71	4.56	4.25	3.73	3.52
8.92	5.50	5.74	5.26	4.99	4.91	4.70	4.65	4.15	4.04	3.78	3.46
9.12	5.87	5.77	5.68	5.49	5.12	5.06	4.72	4.42	4.23	3.84	3.71
9.32	5.65	5.44	5.25	4.84	4.84	4.41	4.26	4.09	3.74	3.47	3.11
9.52	5.85	5.77	5.37	5.09	5.10	4.76	4.71	4.38	4.11	3.74	3.34
9.72	6.01	5.84	5.59	5.35	5.31	4.84	4.75	4.65	4.19	3.86	3.44
9.92	5.91	5.65	5.26	5.07	4.94	4.62	4.47	4.10	3.84	3.84	3.25
10.12	6.07	6.09	5.85	5.63	5.27	5.16	4.78	4.65	4.41	4.13	3.83
10.32	5.76	5.60	5.44	5.19	4.96	4.67	4.48	4.33	4.07	3.65	3.39
10.52	5.91	5.71	5.70	5.39	5.13	4.82	4.69	4.39	3.99	3.87	3.50
10.72	5.64	5.49	5.20	5.04	4.87	4.69	4.35	4.26	3.83	3.77	3.36
10.92	6.18	5.89	5.72	5.47	5.43	5.10	4.80	4.65	4.39	4.04	3.66
11.12	5.76	5.35	5.47	5.17	4.68	4.60	4.34	4.01	3.81	3.64	3.32
11.32	5.68	5.56	5.41	5.05	5.06	4.66	4.64	4.24	3.80	3.48	3.27
11.52	6.01	5.82	5.54	5.30	5.30	5.04	4.75	4.40	4.27	3.80	3.65
11.72	5.77	5.51	5.59	5.09	4.96	4.54	4.57	4.16	3.93	3.48	3.33
11.92	6.12	6.06	5.80	5.55	5.41	5.14	4.75	4.40	4.22	4.10	3.75
12.12	6.03	5.87	5.54	5.31	5.16	4.90	4.78	4.67	4.36	4.04	3.78
12.32	5.88	5.73	5.24	5.31	4.85	4.69	4.46	4.21	3.90	3.61	3.37
12.52	5.61	5.69	5.33	5.16	4.94	4.68	4.57	4.10	4.00	3.75	3.44
12.72	5.66	5.55	5.21	5.00	4.80	4.57	4.34	4.07	3.86	3.47	3.07
12.92	5.60	5.59	5.39	5.22	4.90	4.77	4.60	4.17	4.01	3.52	3.43
13.12	6.08	5.81	5.71	5.57	5.32	4.87	4.64	4.50	4.23	3.84	3.73
13.32	5.49	5.43	5.30	5.04	4.82	4.44	4.31	3.93	3.88	3.42	3.10
13.52	5.59	5.46	5.39	5.21	4.81	4.68	4.48	4.09	4.06	3.71	3.49
13.72	5.55	5.64	5.50	5.06	4.62	4.70	4.34	4.27	4.07	3.61	3.43
13.92	5.73	5.61	5.43	5.14	4.88	4.72	4.46	4.31	4.06	3.66	3.36
14	5.62	5.62	5.41	5.25	4.96	4.89	4.65	4.23	3.97	3.69	3.48
14.08	5.79	5.67	5.44	5.28	4.84	4.58	4.35	4.25	3.77	3.67	3.47
14.17	5.82	5.64	5.27	5.20	4.87	4.64	4.28	4.15	3.80	3.65	3.25
14.27	5.77	5.71	5.32	5.24	4.99	4.63	4.59	4.40	3.98	3.54	3.36
14.38	5.75	5.49	5.46	5.10	4.98	4.76	4.28	4.07	4.02	3.66	3.40
14.49	5.32	5.53	4.84	4.90	4.90	4.57	4.33	4.09	3.57	3.45	3.16
14.61	5.86	5.56	5.56	5.43	5.04	4.87	4.74	4.38	4.20	3.85	3.38

14.75	5.52	5.52	5.43	5.23	5.07	4.70	4.50	4.29	4.08	3.76	3.37
14.89	5.77	5.88	5.51	5.27	4.87	4.81	4.35	4.23	4.15	3.60	3.27
15.05	5.71	5.41	5.26	5.06	4.89	4.56	4.46	4.13	3.97	3.61	3.34
15.21	5.65	5.35	5.26	5.16	4.79	4.65	4.43	3.97	3.89	3.47	3.30
15.39	5.57	5.47	5.12	4.87	4.95	4.55	4.32	3.79	3.83	3.53	3.06
15.59	5.74	5.32	5.32	5.19	4.71	4.60	4.25	4.03	3.79	3.32	3.11
15.8	5.45	5.26	5.54	4.97	4.81	4.54	4.34	4.24	3.95	3.53	3.20
16.02	5.66	5.66	5.33	4.98	4.76	4.57	4.21	4.21	3.86	3.61	3.16
16.27	5.69	5.63	5.21	5.07	4.84	4.66	4.51	4.14	3.83	3.60	3.38
16.53	5.82	5.48	5.27	5.07	4.79	4.54	4.37	4.26	3.76	3.59	3.33
16.81	5.60	5.67	5.27	5.21	4.96	4.70	4.20	4.11	4.02	3.61	3.40
17.12	5.59	5.63	5.15	5.15	4.79	4.62	4.43	4.14	3.95	3.52	3.48
17.45	5.55	5.71	5.49	5.18	4.89	4.55	4.59	4.04	4.00	3.57	3.19
17.81	5.47	5.52	5.26	4.90	4.74	4.49	4.26	4.08	3.93	3.73	3.41
18.19	5.39	5.21	5.01	4.80	4.70	4.40	4.46	3.97	3.93	3.47	3.11
18.6	5.49	5.22	5.02	4.81	4.63	4.39	4.14	3.81	3.66	3.35	3.10
19.05	5.51	5.02	4.87	4.78	4.59	4.20	4.13	3.90	3.64	3.29	3.11
19.53	5.43	5.41	5.02	5.13	4.97	4.62	4.44	4.31	3.74	3.68	3.51
20.05	5.48	5.18	5.10	4.97	4.45	4.49	4.08	4.00	3.66	3.31	3.13
20.61	5.42	5.33	5.07	4.80	4.75	4.43	4.32	3.78	3.71	3.45	3.17
21.22	5.46	5.25	5.06	4.80	4.48	4.43	3.96	4.00	3.74	3.40	3.04
21.87	5.73	5.44	5.30	5.02	4.85	4.46	4.17	4.22	3.80	3.54	3.41
22.57	5.40	5.15	5.03	4.79	4.60	4.53	4.22	3.96	3.96	3.60	3.14
23.33	5.63	5.39	5.11	4.93	4.70	4.25	4.12	4.04	3.74	3.34	3.21
24.15	5.25	5.14	5.01	4.72	4.56	4.24	3.96	3.74	3.40	3.12	3.03
25.03	5.35	5.00	5.18	4.79	4.63	4.37	4.22	3.95	3.65	3.42	3.09
25.98	5.30	5.35	5.13	4.61	4.54	4.19	3.94	3.73	3.67	3.18	2.93
27.01	5.04	5.03	4.72	4.56	4.31	4.18	3.95	3.79	3.40	3.07	2.98
28.11	5.43	5.30	5.11	4.79	4.58	4.54	4.12	3.94	3.61	3.36	3.17
29.3	5.29	5.18	4.99	4.50	4.26	4.12	3.77	3.47	3.36	2.94	2.75
30.59	5.14	5.23	5.01	4.47	4.38	4.37	3.98	3.61	3.51	3.20	3.16
31.98	5.38	5.28	4.99	4.77	4.49	4.36	4.21	3.75	3.58	3.20	2.96
33.47	5.27	5.24	4.86	4.75	4.59	4.32	3.99	3.81	3.68	3.03	3.06
35.09	5.47	5.26	5.07	4.85	4.52	4.23	4.06	3.81	3.79	3.39	3.21
36.83	5.18	5.06	4.67	4.49	4.46	4.00	3.77	3.56	3.30	3.08	2.65
38.71	5.25	5.14	4.72	4.53	4.41	4.26	3.92	3.69	3.41	3.26	2.84
40.73	4.98	4.97	4.78	4.61	4.47	4.33	3.97	3.81	3.54	3.21	2.97
42.92	5.28	5.17	4.93	4.77	4.50	4.21	4.23	3.90	3.89	3.30	3.10
45.27	5.56	5.27	5.13	4.89	4.68	4.48	4.34	4.04	3.77	3.36	3.19
47.81	5.13	5.03	4.61	4.53	4.29	4.12	3.96	3.78	3.39	3.23	2.69
50.55	5.16	4.82	4.76	4.52	4.30	3.89	3.55	3.38	3.24	2.90	2.66
53.51	4.64	4.57	4.41	4.20	3.92	3.73	3.46	3.17	2.94	2.52	2.26
56.69	4.83	4.64	4.62	4.52	4.14	3.95	3.62	3.33	3.14	2.79	2.42
60.13	4.81	4.88	4.55	4.21	3.73	3.75	3.55	3.43	3.32	2.92	2.65
63.84	4.96	4.70	4.41	4.31	4.14	3.81	3.64	3.59	3.18	2.93	2.76
67.83	4.80	4.78	4.51	4.26	4.07	3.87	3.58	3.41	2.89	2.79	2.29
72.15	4.95	4.62	4.48	4.22	3.86	3.69	3.55	3.45	3.26	2.77	2.53
76.8	4.77	4.59	4.32	4.11	4.02	3.80	3.59	3.46	3.06	2.69	2.56
81.81	4.91	4.68	4.59	4.32	4.14	3.84	3.69	3.59	3.12	2.83	2.87
87.22	4.72	4.74	4.50	4.38	4.25	4.00	3.59	3.37	2.95	2.61	2.63

93.06	5.10	4.79	4.61	4.43	4.11	3.87	3.64	3.37	2.98	2.75	2.52
99.35	4.86	4.87	4.67	4.29	4.10	3.74	3.65	3.74	3.17	3.02	2.80
106.13	4.66	4.68	4.51	3.96	3.98	3.67	3.41	3.33	2.96	2.52	2.45
113.45	4.73	4.87	4.57	4.29	3.90	3.82	3.59	3.37	3.04	2.95	2.43
121.34	4.94	4.80	4.60	4.46	4.10	3.78	3.61	3.41	3.26	2.75	2.57
129.86	4.60	4.62	4.36	4.17	3.74	3.57	3.40	3.24	3.09	2.77	2.66
139.04	4.58	4.47	4.20	3.95	3.82	3.52	3.46	3.30	2.82	2.77	2.72
148.94	4.43	4.45	4.05	4.08	3.98	3.58	3.24	3.02	2.88	2.77	2.43
159.62	4.55	4.53	4.06	3.89	3.57	3.55	3.44	3.21	2.92	2.40	2.43
171.14	4.62	4.72	4.18	3.96	3.71	3.65	3.36	3.11	2.93	2.89	2.59
183.56	4.24	4.21	4.13	3.82	3.73	3.50	3.43	3.14	2.79	2.46	2.35
196.96	4.61	4.65	4.14	3.90	3.83	3.52	3.27	3.17	2.94	2.57	2.25
211.41	4.35	4.49	4.32	3.91	3.96	3.51	3.52	3.22	2.83	2.78	2.56
226.99	4.49	4.47	4.34	4.04	3.87	3.69	3.43	3.24	2.94	2.69	2.37
243.8	4.16	4.32	3.95	3.70	3.62	3.35	3.20	2.90	2.68	2.35	2.27
261.93	4.46	4.30	3.98	3.72	3.60	3.19	3.18	2.85	2.68	2.35	2.07
281.48	4.70	4.58	4.47	4.21	3.80	3.54	3.50	3.16	3.10	2.82	2.54
302.56	4.46	4.19	3.85	3.91	3.71	3.34	3.28	3.14	2.81	2.49	2.44
325.3	4.27	4.26	4.06	3.65	3.51	3.48	3.21	2.80	2.70	2.27	2.18
349.83	4.04	3.75	3.89	3.62	3.29	3.07	3.15	2.97	2.68	2.31	2.02
376.28	4.13	4.05	3.76	3.73	3.50	3.39	3.12	3.03	2.76	2.44	2.26
404.81	4.44	4.18	4.00	3.70	3.85	3.57	3.42	3.17	2.89	2.82	2.37
435.58	4.04	3.77	3.53	3.43	3.18	3.02	2.80	2.59	2.41	2.18	2.00
468.76	4.20	3.99	3.97	3.83	3.62	3.36	3.05	3.00	2.80	2.69	2.14
504.55	4.15	3.94	4.02	3.85	3.46	3.36	3.24	2.82	2.58	2.39	2.26
543.15	3.84	4.00	3.74	3.44	3.28	2.99	2.82	2.95	2.50	2.32	2.16
584.78	4.14	3.87	4.00	3.53	3.29	3.13	3.13	2.90	2.69	2.28	2.11
629.68	4.02	3.92	3.66	3.52	3.43	3.15	3.02	2.85	2.67	2.27	2.20
678.1	4.12	4.03	3.95	3.49	3.53	3.38	3.22	2.92	3.06	2.42	2.32
730.33	3.83	3.66	3.71	3.38	3.27	3.01	2.75	2.77	2.33	2.29	2.08
786.65	3.74	3.80	3.54	3.48	3.18	2.98	2.79	2.86	2.45	2.34	2.05
847.4	3.54	3.23	3.30	3.18	3.04	2.95	2.65	2.54	2.34	2.03	1.74
912.92	3.77	3.69	3.45	3.39	3.18	2.80	2.80	2.63	2.45	2.34	2.09
983.58	3.78	3.71	3.33	3.32	3.22	3.05	2.99	2.76	2.35	2.17	2.09
1059.78	3.89	3.78	3.61	3.31	3.34	2.97	3.03	2.84	2.61	2.23	2.36
1141.98	3.75	3.85	3.31	3.37	3.20	3.10	2.94	2.66	2.74	2.17	1.99
1230.62	3.73	3.53	3.30	3.21	3.03	2.86	2.82	2.47	2.35	2.21	1.89
1326.23	3.84	3.67	3.51	3.28	3.09	3.08	2.81	2.82	2.48	2.32	1.93
1429.34	3.92	3.57	3.54	3.60	3.44	3.16	2.85	2.93	2.54	2.32	1.97
1540.54	3.41	3.25	3.35	3.12	2.94	2.82	2.57	2.42	2.16	1.95	1.90
1660.48	3.62	3.46	3.26	3.16	2.97	2.81	2.71	2.67	2.43	2.10	2.01
1789.83	3.25	3.06	3.08	2.80	2.68	2.52	2.16	2.07	1.90	1.76	1.45
1929.34	3.86	3.60	3.57	3.41	3.21	3.17	2.86	2.68	2.35	2.31	1.89
2079.8	3.37	3.31	3.25	2.91	2.80	2.83	2.44	2.46	2.24	1.84	1.63
2242.08	3.64	3.65	3.43	3.14	2.86	2.83	2.56	2.56	2.31	2.06	1.71
2417.1	3.34	3.41	3.30	2.98	2.70	2.72	2.79	2.53	2.26	2.00	1.96
2605.85	3.33	3.31	2.98	2.96	2.86	2.67	2.55	2.33	2.16	1.81	1.64
2809.43	3.35	3.39	3.17	2.86	2.90	2.57	2.60	2.21	2.18	1.84	1.78
3028.99	3.50	3.59	3.31	3.20	3.02	3.01	2.52	2.59	2.37	2.09	1.94
3265.79	3.44	3.45	2.92	2.99	2.65	2.64	2.49	2.19	2.01	1.62	1.51

3521.18	3.18	3.10	2.89	2.75	2.69	2.41	2.15	2.26	1.84	1.83	1.47
3796.62	3.25	3.03	2.79	2.69	2.80	2.42	2.15	1.99	1.92	1.67	1.43

Wavelength (nm) / Time (ps)	444.66	446.06	447.47	448.88	450.29	451.70	453.11	454.52	455.92	457.33	458.74
-3.28	0.20	0.40	0.18	0.11	0.21	0.18	0.26	0.10	0.10	0.27	0.35
-2.78	0.24	-0.13	0.14	0.06	0.03	0.07	-0.04	0.04	-0.05	-0.03	0.09
-2.28	-0.05	-0.04	-0.13	-0.12	-0.17	0.11	0.06	-0.06	-0.06	0.11	0.15
-1.78	-0.02	-0.01	-0.01	0.10	-0.06	0.05	-0.06	-0.07	0.04	-0.06	-0.08
-1.28	-0.28	-0.20	-0.12	-0.26	-0.03	-0.23	-0.35	-0.24	-0.20	-0.27	-0.33
-0.78	0.14	-0.01	0.03	0.22	0.11	0.05	0.19	0.03	0.22	0.14	-0.05
-0.28	-0.28	-0.18	-0.24	-0.21	-0.19	-0.38	-0.26	-0.07	-0.22	-0.27	-0.15
0.22	0.05	0.16	0.14	0.09	0.10	0.15	0.20	0.27	0.17	0.10	0.01
0.32	-0.07	0.12	0.12	0.14	0.21	0.08	0.18	0.09	0.21	0.00	0.14
0.42	0.04	-0.30	-0.28	-0.06	-0.02	-0.09	-0.10	-0.15	-0.12	-0.06	-0.07
0.52	-0.38	-0.33	-0.26	-0.25	-0.22	-0.06	-0.23	-0.35	-0.30	-0.32	-0.25
0.62	0.03	0.14	0.19	0.31	0.27	0.01	0.31	0.04	0.11	0.02	0.17
0.72	-0.33	-0.33	-0.36	-0.33	-0.16	-0.15	-0.25	-0.27	-0.31	-0.09	-0.15
0.77	0.15	0.10	0.04	0.13	0.08	0.21	0.07	0.08	0.10	0.27	-0.06
0.82	0.07	0.00	0.03	0.21	0.25	-0.02	0.20	0.06	0.02	0.23	0.08
0.87	-0.14	0.07	-0.07	0.07	0.10	0.09	0.11	-0.03	-0.06	-0.08	0.05
0.92	-0.04	-0.17	-0.06	0.10	0.01	-0.19	-0.16	-0.11	-0.11	-0.13	0.03
0.97	-0.19	-0.22	-0.24	-0.27	-0.17	-0.14	-0.18	-0.10	-0.16	-0.13	-0.16
1.02	-0.07	0.02	-0.18	0.06	-0.06	-0.07	-0.08	-0.14	-0.06	-0.03	-0.07
1.07	0.14	0.18	-0.13	0.11	-0.02	0.06	0.05	0.02	-0.06	0.23	-0.11
1.12	-0.03	-0.15	-0.05	0.07	-0.04	0.14	-0.01	-0.08	0.03	0.00	-0.19
1.17	-0.72	-0.81	-0.87	-0.89	-0.98	-0.86	-0.86	-0.68	-0.89	-0.52	-0.46
1.22	5.88	4.83	3.87	3.01	2.19	1.23	0.77	0.11	-0.16	-0.32	-0.60
1.27	9.23	8.49	7.80	7.47	6.54	5.94	5.11	4.48	3.84	3.22	2.79
1.32	10.86	10.17	9.79	9.39	8.68	8.05	7.23	6.73	6.19	5.70	5.19
1.37	9.58	9.21	9.07	8.72	8.65	8.41	8.11	8.17	7.77	7.85	7.45
1.42	7.81	7.47	7.06	6.67	6.35	5.82	5.32	4.85	4.16	4.00	3.41
1.47	7.22	7.08	6.44	6.14	5.66	5.19	4.73	3.98	3.65	2.98	2.27
1.52	6.59	6.47	5.81	5.42	5.11	4.54	4.01	3.34	2.89	2.53	1.65
1.57	6.38	6.03	5.43	5.00	4.67	4.08	3.51	2.97	2.57	2.16	1.30
1.62	6.20	5.91	5.58	5.27	4.79	4.25	3.75	3.24	2.59	2.11	1.63
1.67	5.58	5.34	4.95	4.41	4.31	3.61	3.08	2.79	2.19	1.76	1.24
1.72	5.31	4.89	4.51	4.33	3.90	3.39	2.85	2.52	2.07	1.48	0.96
1.77	5.17	4.94	4.33	3.88	3.85	3.43	2.79	2.57	1.86	1.32	0.75
1.82	5.40	5.04	4.49	4.20	3.76	3.27	3.00	2.54	2.11	1.59	1.02
1.87	5.24	4.81	4.53	4.19	3.69	3.28	2.86	2.43	2.06	1.35	1.05
1.92	5.23	4.88	4.67	4.20	3.69	3.35	2.98	2.49	2.19	1.73	1.37
1.97	4.95	4.72	4.06	3.81	3.55	2.91	2.60	2.18	1.76	1.37	0.74
2.02	4.76	4.46	4.01	3.82	3.37	3.12	2.42	2.25	1.63	1.26	0.87
2.07	4.77	4.42	4.19	3.78	3.37	2.96	2.58	2.04	1.74	1.32	0.84
2.12	4.65	4.37	4.06	3.77	3.35	2.86	2.46	2.09	1.56	1.23	0.91
2.17	4.80	4.56	4.10	3.93	3.58	3.20	2.82	2.30	1.76	1.33	1.03
2.22	4.64	4.33	3.89	3.65	3.44	2.88	2.41	2.30	1.50	1.20	0.98

2.27	4.69	4.48	3.88	3.54	3.19	2.85	2.47	1.94	1.75	1.30	0.92
2.32	4.43	4.11	3.79	3.51	3.18	2.66	2.36	1.83	1.50	1.05	0.66
2.37	4.36	4.01	3.80	3.43	3.26	2.71	2.34	2.01	1.53	1.00	0.57
2.42	4.76	4.21	3.93	3.73	3.27	2.79	2.50	2.00	1.48	1.20	0.85
2.47	4.94	4.43	3.99	3.91	3.58	3.26	2.81	2.21	1.89	1.46	1.01
2.52	4.23	3.82	3.28	3.24	2.93	2.34	2.22	1.72	1.36	0.70	0.63
2.57	3.84	3.90	3.31	3.18	2.74	2.35	1.90	1.69	1.14	0.80	0.46
2.62	4.14	3.89	3.47	3.06	2.86	2.40	1.99	1.49	1.31	0.86	0.24
2.67	4.12	3.83	3.45	3.03	2.91	2.22	2.08	1.62	1.34	0.88	0.66
2.72	4.02	3.77	3.41	3.17	3.07	2.55	2.16	1.77	1.43	0.82	0.46
2.77	3.89	3.60	3.30	2.91	2.62	2.44	1.98	1.39	0.91	0.59	0.23
2.82	4.27	3.96	3.62	3.32	3.05	2.64	2.20	1.78	1.58	1.14	0.52
2.87	4.07	3.59	3.20	3.09	2.64	2.19	1.90	1.41	0.96	0.73	0.20
2.92	4.37	4.06	3.49	3.37	3.13	2.49	2.21	1.84	1.40	1.05	0.61
2.97	4.05	3.68	3.41	2.97	2.77	2.14	1.86	1.44	1.03	0.88	0.20
3.02	4.15	3.96	3.42	3.35	2.77	2.39	2.10	1.70	1.19	0.85	0.49
3.07	3.82	3.68	3.23	2.84	2.68	2.21	1.60	1.35	0.96	0.54	-0.08
3.12	4.22	4.02	3.35	3.28	2.80	2.55	2.13	1.67	1.27	0.93	0.41
3.17	4.01	3.62	3.16	3.13	2.73	2.34	1.82	1.42	1.26	0.75	0.30
3.22	4.06	3.78	3.37	2.99	2.77	2.33	1.89	1.48	1.13	0.75	0.44
3.27	4.16	3.94	3.65	3.26	3.06	2.42	2.33	1.88	1.42	1.08	0.58
3.32	4.32	3.98	3.64	3.35	3.04	2.67	2.23	1.83	1.44	1.10	0.63
3.37	3.89	3.71	3.16	2.97	2.76	2.20	1.99	1.33	1.20	0.81	0.37
3.42	3.67	3.42	3.02	2.69	2.32	1.90	1.61	1.11	0.81	0.51	0.02
3.47	3.75	3.73	3.39	3.11	2.69	2.37	1.95	1.64	1.26	0.86	0.30
3.52	3.80	3.74	3.19	3.01	2.78	2.39	1.81	1.78	1.14	0.60	0.12
3.57	3.93	3.68	3.25	3.02	2.76	2.20	1.79	1.67	1.06	0.54	0.29
3.62	3.75	3.15	2.98	2.79	2.41	1.85	1.65	1.07	0.79	0.45	0.14
3.67	3.75	3.40	2.99	2.74	2.51	2.11	1.75	1.22	0.86	0.54	0.17
3.72	3.95	3.65	3.31	2.99	2.65	2.17	2.01	1.53	1.04	0.72	0.22
3.92	3.35	3.19	2.76	2.70	2.27	1.66	1.45	1.11	0.69	0.36	0.14
4.12	3.95	3.50	3.22	3.12	2.73	2.22	1.74	1.55	1.15	0.75	0.34
4.32	4.11	3.78	3.32	3.24	2.67	2.39	1.81	1.49	1.01	0.74	0.22
4.52	4.08	3.57	3.29	3.10	2.83	2.22	1.95	1.66	1.15	0.94	0.54
4.72	3.77	3.48	3.10	2.94	2.51	2.17	1.87	1.54	0.85	0.78	0.17
4.92	3.60	3.38	3.02	2.76	2.47	2.00	1.77	1.33	0.98	0.50	0.15
5.12	3.62	3.24	2.79	2.83	2.43	1.87	1.41	1.22	0.96	0.20	0.06
5.32	3.66	3.33	3.03	2.67	2.18	1.74	1.53	1.27	0.90	0.50	-0.16
5.52	3.70	3.37	3.12	2.77	2.52	2.19	1.69	1.28	0.92	0.65	0.11
5.72	3.38	3.10	2.67	2.43	2.07	1.79	1.35	1.02	0.69	0.30	-0.07
5.92	3.70	3.33	2.93	2.74	2.63	2.08	1.65	1.25	0.80	0.48	0.16
6.12	3.47	3.06	2.71	2.57	2.27	1.95	1.62	1.23	0.74	0.40	0.10
6.32	3.52	3.24	2.84	2.60	2.42	1.81	1.54	1.17	0.92	0.45	0.09
6.52	3.75	3.31	3.10	2.83	2.54	2.12	1.78	1.48	1.08	0.52	0.23
6.72	3.38	3.18	2.76	2.65	2.20	1.92	1.60	1.23	0.76	0.45	-0.09
6.92	3.10	2.89	2.66	2.26	1.98	1.70	1.46	1.01	0.60	0.44	-0.09
7.12	3.39	3.23	2.73	2.54	2.22	1.93	1.72	1.29	1.08	0.59	0.21
7.32	3.68	3.35	2.96	2.75	2.52	1.90	1.65	1.29	0.89	0.39	0.20
7.52	3.83	3.46	3.26	2.92	2.49	2.23	1.87	1.44	1.18	0.80	0.37
7.72	3.07	2.87	2.55	2.33	2.25	1.76	1.41	0.80	0.65	0.27	-0.05

7.92	3.55	3.18	2.76	2.58	2.20	1.97	1.73	1.23	1.03	0.40	0.24
8.12	3.39	2.98	2.65	2.38	1.98	1.79	1.30	0.97	0.71	0.42	-0.07
8.32	3.68	3.49	3.02	2.89	2.54	2.04	1.85	1.52	0.98	0.63	0.34
8.52	3.48	3.27	2.87	2.71	2.27	2.03	1.53	1.25	0.77	0.52	0.15
8.72	3.45	3.16	2.83	2.57	2.38	2.04	1.44	1.20	0.81	0.51	0.23
8.92	3.29	2.99	2.63	2.36	2.05	1.72	1.23	0.90	0.57	0.14	-0.13
9.12	3.26	3.02	2.64	2.52	2.33	1.65	1.44	1.09	0.63	0.62	-0.05
9.32	2.84	2.55	2.27	2.07	1.66	1.38	0.81	0.36	0.25	0.02	-0.50
9.52	3.28	2.98	2.56	2.42	2.18	1.75	1.42	1.04	0.72	0.32	0.07
9.72	3.36	2.95	2.77	2.49	2.14	1.69	1.46	1.12	0.87	0.33	-0.05
9.92	3.09	2.86	2.46	2.27	2.02	1.60	1.35	1.02	0.45	0.22	-0.19
10.12	3.46	3.22	2.90	2.91	2.31	1.73	1.66	1.15	0.81	0.47	0.14
10.32	3.21	2.81	2.53	2.42	2.14	1.67	1.30	1.03	0.57	0.19	0.00
10.52	3.24	2.93	2.49	2.33	2.19	1.71	1.20	1.01	0.62	0.22	-0.26
10.72	3.15	2.85	2.41	2.26	1.88	1.47	1.23	1.03	0.66	0.09	-0.18
10.92	3.41	3.21	2.94	2.67	2.26	2.07	1.61	1.30	0.80	0.57	0.14
11.12	3.00	2.89	2.28	2.22	1.96	1.65	1.17	0.61	0.30	0.22	-0.29
11.32	3.09	2.74	2.44	2.16	1.96	1.54	1.23	0.81	0.56	0.27	-0.11
11.52	3.50	3.27	2.66	2.68	2.28	2.14	1.70	1.38	0.83	0.54	0.12
11.72	3.14	2.76	2.41	2.24	2.06	1.51	1.28	0.80	0.62	0.20	-0.25
11.92	3.40	3.25	3.01	2.83	2.22	1.91	1.63	1.26	1.00	0.48	0.17
12.12	3.48	3.27	2.80	2.86	2.58	2.11	1.79	1.39	1.14	0.71	0.24
12.32	2.99	2.98	2.42	2.30	1.88	1.65	1.28	0.80	0.29	0.06	-0.19
12.52	3.16	2.91	2.62	2.42	2.31	1.73	1.50	1.11	0.72	0.41	-0.06
12.72	2.94	2.71	2.34	2.23	1.82	1.46	1.10	0.86	0.39	0.00	-0.24
12.92	3.17	2.88	2.44	2.23	1.88	1.77	1.36	0.95	0.46	0.20	-0.01
13.12	3.38	3.29	2.80	2.65	2.34	1.98	1.66	1.13	0.86	0.52	0.10
13.32	2.92	2.67	2.55	2.25	1.95	1.45	1.19	0.88	0.53	0.16	-0.42
13.52	2.91	2.90	2.55	2.25	1.95	1.55	1.37	0.99	0.56	0.29	-0.14
13.72	3.07	2.98	2.42	2.39	2.00	1.62	1.40	1.23	0.70	0.25	-0.20
13.92	3.02	2.79	2.51	2.29	2.07	1.72	1.29	0.90	0.68	0.17	-0.19
14	3.27	3.08	2.61	2.32	2.21	1.75	1.45	1.04	0.72	0.18	-0.20
14.08	3.19	2.89	2.64	2.48	2.17	1.86	1.28	0.95	0.61	0.29	-0.05
14.17	3.23	2.85	2.47	2.11	1.97	1.56	1.09	0.67	0.63	0.24	-0.33
14.27	3.30	2.80	2.54	2.48	2.08	1.62	1.24	0.86	0.50	0.13	-0.16
14.38	3.19	2.89	2.67	2.40	1.94	1.64	1.21	0.84	0.67	0.35	-0.03
14.49	2.90	2.91	2.36	2.16	1.94	1.69	1.30	1.15	0.37	0.48	-0.15
14.61	3.44	2.87	2.63	2.61	2.12	1.73	1.40	0.96	0.80	0.32	0.03
14.75	3.19	2.84	2.59	2.50	1.99	1.89	1.48	0.94	0.76	0.41	-0.12
14.89	3.09	2.88	2.49	2.34	2.04	1.54	1.28	0.73	0.50	0.25	-0.16
15.05	3.21	2.90	2.58	2.31	2.18	1.67	1.35	1.05	0.43	0.33	-0.03
15.21	3.15	2.68	2.34	2.14	2.14	1.58	1.11	0.87	0.59	0.31	-0.22
15.39	2.99	2.89	2.64	2.29	1.92	1.74	1.05	0.86	0.36	0.23	-0.16
15.59	2.88	2.49	2.33	2.01	1.74	1.30	1.05	0.72	0.35	0.05	-0.51
15.8	3.02	2.92	2.56	2.22	2.04	1.49	1.21	0.82	0.72	0.27	-0.12
16.02	3.07	2.77	2.41	2.09	2.00	1.61	1.20	1.01	0.60	0.15	-0.15
16.27	2.92	2.80	2.51	2.48	2.02	1.66	1.38	0.87	0.61	0.06	-0.16
16.53	3.17	2.87	2.55	2.40	2.16	1.77	1.27	0.98	0.55	0.25	-0.29
16.81	3.08	2.85	2.44	2.38	1.95	1.51	1.30	0.94	0.71	0.21	-0.13
17.12	3.05	2.77	2.55	2.24	1.94	1.61	1.31	0.90	0.71	0.41	-0.35

17.45	3.15	2.93	2.44	2.12	1.87	1.72	1.32	1.00	0.62	0.02	-0.18
17.81	3.01	2.92	2.45	2.18	1.77	1.58	1.08	0.87	0.55	0.23	-0.06
18.19	3.01	2.94	2.56	2.13	1.85	1.76	1.26	0.97	0.50	0.29	-0.26
18.6	2.88	2.59	2.40	1.93	1.94	1.53	1.11	0.70	0.44	0.22	-0.24
19.05	2.92	2.53	2.37	2.07	1.67	1.34	0.96	0.63	0.39	-0.04	-0.40
19.53	3.08	2.84	2.62	2.44	2.25	1.65	1.51	1.08	0.71	0.34	0.07
20.05	2.86	2.47	2.31	2.06	1.77	1.49	1.12	0.58	0.43	0.08	-0.47
20.61	2.90	2.79	2.48	2.11	1.97	1.62	1.27	0.97	0.52	0.23	-0.15
21.22	2.90	2.66	2.33	2.08	1.69	1.29	1.00	0.78	0.46	-0.10	-0.19
21.87	3.16	2.81	2.53	2.57	2.03	1.60	1.24	1.00	0.69	0.33	-0.08
22.57	3.04	2.75	2.67	2.33	1.93	1.64	1.17	1.02	0.59	0.08	-0.15
23.33	2.99	2.76	2.33	2.12	1.94	1.52	1.26	0.71	0.41	0.14	-0.14
24.15	2.91	2.59	2.07	2.01	1.78	1.44	0.93	0.71	0.36	-0.06	-0.39
25.03	2.72	2.65	2.36	2.13	1.82	1.49	1.21	0.87	0.52	0.24	-0.24
25.98	2.70	2.52	2.40	2.03	1.74	1.42	0.86	0.56	0.47	0.08	-0.46
27.01	2.94	2.37	2.25	2.02	1.64	1.27	1.08	0.60	0.25	0.00	-0.33
28.11	2.69	2.62	2.26	2.22	1.89	1.41	1.14	0.84	0.38	0.18	-0.23
29.3	2.56	2.10	1.77	1.69	1.52	0.97	0.80	0.24	-0.02	-0.36	-0.64
30.59	2.83	2.35	2.18	2.13	1.81	1.28	0.93	0.75	0.28	0.10	-0.34
31.98	2.85	2.64	2.38	2.01	1.82	1.19	1.00	0.76	0.29	0.08	-0.43
33.47	2.86	2.54	2.13	1.97	1.66	1.46	1.12	0.72	0.39	0.03	-0.15
35.09	2.93	2.78	2.35	2.22	1.94	1.70	1.32	0.82	0.54	0.30	-0.11
36.83	2.46	2.47	2.13	1.86	1.53	1.09	0.76	0.58	0.12	-0.22	-0.57
38.71	2.59	2.38	2.08	1.83	1.54	1.23	0.93	0.49	0.23	-0.12	-0.49
40.73	2.92	2.64	2.16	2.16	1.76	1.53	1.03	0.76	0.30	-0.05	-0.17
42.92	2.92	2.50	2.37	2.18	1.87	1.39	1.05	0.76	0.28	0.12	-0.26
45.27	2.97	2.74	2.29	2.11	1.84	1.56	1.29	0.67	0.35	-0.05	-0.45
47.81	2.67	2.31	2.00	1.78	1.50	1.26	0.84	0.43	0.02	-0.41	-0.58
50.55	2.34	2.17	2.00	1.82	1.62	1.07	0.76	0.38	0.01	-0.28	-0.61
53.51	2.25	1.94	1.72	1.46	1.06	0.85	0.38	0.02	-0.35	-0.48	-0.93
56.69	2.32	1.98	1.73	1.64	1.32	0.93	0.72	0.23	0.01	-0.26	-0.73
60.13	2.47	2.36	1.93	1.75	1.58	1.15	0.92	0.64	0.16	-0.11	-0.44
63.84	2.48	2.23	1.87	1.84	1.43	1.17	0.62	0.53	-0.17	-0.25	-0.68
67.83	2.40	2.20	1.74	1.65	1.31	0.96	0.71	0.07	-0.13	-0.62	-1.05
72.15	2.33	1.94	1.80	1.65	1.41	1.05	0.60	0.25	-0.18	-0.30	-0.79
76.8	2.25	2.06	1.68	1.45	1.49	0.90	0.49	0.21	-0.13	-0.33	-0.75
81.81	2.57	2.18	1.74	1.65	1.41	0.93	0.72	0.31	0.15	-0.20	-0.71
87.22	2.21	2.14	1.63	1.47	1.25	0.86	0.34	0.12	-0.24	-0.29	-0.90
93.06	2.35	2.19	1.77	1.77	1.26	0.97	0.78	0.40	-0.04	-0.30	-0.87
99.35	2.45	2.29	1.96	1.67	1.58	1.10	0.77	0.35	0.08	-0.29	-0.41
106.13	2.14	1.89	1.58	1.48	1.13	0.83	0.47	0.24	-0.13	-0.63	-1.00
113.45	2.40	2.38	1.85	1.63	1.59	1.05	0.89	0.30	-0.01	-0.33	-0.69
121.34	2.39	2.04	1.76	1.63	1.38	1.00	0.71	0.14	0.15	-0.29	-0.80
129.86	2.30	2.16	1.77	1.54	1.13	0.84	0.50	0.20	-0.23	-0.39	-0.93
139.04	2.42	2.23	1.61	1.61	1.34	1.09	0.73	0.33	0.10	-0.32	-0.81
148.94	2.08	2.00	1.60	1.41	1.06	0.82	0.49	0.04	-0.21	-0.57	-0.98
159.62	2.01	1.82	1.57	1.35	1.16	0.83	0.46	0.23	-0.05	-0.58	-0.80
171.14	2.47	2.24	1.82	1.78	1.46	1.10	0.83	0.28	0.26	-0.13	-0.66
183.56	2.14	1.85	1.53	1.33	0.85	0.69	0.56	0.15	-0.33	-0.57	-0.94
196.96	1.98	1.92	1.60	1.43	1.22	1.02	0.53	0.24	-0.28	-0.39	-0.70

211.41	2.25	2.26	1.85	1.44	1.14	0.87	0.43	0.25	-0.08	-0.39	-0.62
226.99	2.24	2.12	1.64	1.52	1.38	0.88	0.67	0.12	-0.01	-0.38	-0.84
243.8	1.95	1.95	1.54	1.33	1.07	0.70	0.34	0.00	-0.26	-0.41	-0.91
261.93	1.96	1.81	1.47	1.20	1.08	0.81	0.35	0.07	-0.35	-0.54	-1.04
281.48	2.25	2.11	1.82	1.74	1.54	0.98	0.59	0.22	-0.03	-0.46	-0.63
302.56	2.05	1.83	1.62	1.55	1.26	0.93	0.61	0.34	-0.12	-0.54	-0.78
325.3	1.99	1.90	1.38	1.26	1.23	0.66	0.34	-0.02	-0.42	-0.58	-0.91
349.83	1.96	1.77	1.41	1.27	0.97	0.69	0.30	0.07	-0.07	-0.48	-0.95
376.28	2.08	1.85	1.54	1.39	1.20	0.76	0.52	0.08	-0.32	-0.57	-0.94
404.81	2.22	1.96	1.77	1.54	1.39	1.02	0.56	0.48	0.07	-0.31	-0.56
435.58	1.86	1.63	1.19	1.13	0.94	0.54	0.14	-0.22	-0.28	-0.74	-0.91
468.76	2.12	1.90	1.46	1.43	1.26	0.90	0.66	0.23	-0.11	-0.25	-0.81
504.55	1.95	1.97	1.63	1.42	1.28	0.81	0.53	0.36	-0.07	-0.36	-0.72
543.15	1.97	1.64	1.45	1.21	1.12	0.59	0.42	-0.06	-0.22	-0.48	-0.94
584.78	1.77	1.79	1.42	1.27	0.95	0.75	0.35	0.19	-0.31	-0.48	-0.76
629.68	2.08	1.67	1.53	1.32	1.10	0.83	0.45	0.25	-0.03	-0.44	-0.73
678.1	2.11	1.93	1.67	1.55	1.36	1.02	0.76	0.57	0.16	-0.22	-0.35
730.33	2.05	1.84	1.58	1.35	1.07	0.86	0.49	0.11	-0.10	-0.33	-0.56
786.65	1.81	1.80	1.53	1.38	1.00	0.52	0.63	0.10	-0.09	-0.43	-0.72
847.4	1.69	1.51	1.35	1.04	0.92	0.64	0.31	-0.09	-0.45	-0.60	-1.06
912.92	1.94	1.62	1.51	1.33	1.22	0.83	0.47	0.16	-0.20	-0.55	-0.81
983.58	1.91	1.57	1.42	1.36	1.09	0.73	0.34	0.16	-0.13	-0.44	-0.64
1059.78	2.09	1.78	1.75	1.40	1.24	1.06	0.63	0.23	-0.10	-0.36	-0.47
1141.98	1.99	1.88	1.53	1.32	1.00	0.75	0.36	0.30	0.11	-0.09	-0.41
1230.62	1.67	1.38	1.37	1.14	0.85	0.45	0.07	0.06	-0.38	-0.61	-0.82
1326.23	1.78	1.75	1.33	1.24	1.11	0.66	0.44	0.06	-0.16	-0.48	-0.90
1429.34	2.05	1.88	1.51	1.41	1.12	0.81	0.63	0.23	-0.04	-0.42	-0.57
1540.54	1.64	1.33	1.24	1.08	0.93	0.40	0.21	-0.20	-0.41	-0.59	-0.93
1660.48	1.91	1.81	1.33	1.32	1.15	0.67	0.27	0.22	-0.13	-0.42	-0.81
1789.83	1.45	1.10	0.87	0.74	0.56	0.44	-0.07	-0.31	-0.63	-0.89	-1.31
1929.34	1.82	1.64	1.49	1.22	0.91	0.79	0.48	0.04	-0.28	-0.50	-0.75
2079.8	1.29	1.36	1.05	0.90	0.54	0.51	0.14	-0.36	-0.56	-0.75	-1.14
2242.08	1.64	1.42	1.25	1.00	0.76	0.49	0.21	-0.14	-0.39	-0.57	-0.99
2417.1	1.79	1.58	1.43	1.33	1.04	0.56	0.42	0.05	-0.17	-0.44	-0.95
2605.85	1.32	1.22	0.95	0.96	0.58	0.21	-0.05	-0.27	-0.63	-0.84	-1.28
2809.43	1.55	1.35	0.91	1.06	0.73	0.32	-0.01	-0.24	-0.51	-0.79	-1.16
3028.99	1.73	1.61	1.28	1.05	0.92	0.63	0.40	-0.14	-0.13	-0.69	-0.83
3265.79	1.42	1.25	0.90	1.05	0.59	0.24	0.14	-0.23	-0.65	-0.73	-1.28
3521.18	1.41	1.28	0.73	0.77	0.62	0.22	-0.20	-0.49	-0.72	-0.85	-1.52
3796.62	1.43	0.95	0.70	0.81	0.41	0.05	-0.10	-0.56	-0.85	-0.92	-1.33

Wavelength (nm) / Time (ps)	460.15	461.56	462.97	464.38	465.78	467.19	468.60	470.01	471.42	472.83	474.24
-3.28	0.27	0.14	0.20	0.12	0.05	0.31	0.11	0.21	0.13	0.09	0.22
-2.78	0.04	0.11	0.14	0.05	-0.05	-0.05	0.05	0.02	0.21	0.09	0.00
-2.28	-0.13	-0.08	-0.05	0.03	0.01	-0.07	-0.09	0.01	-0.10	-0.06	-0.10
-1.78	0.11	0.11	-0.07	0.02	0.04	-0.04	0.05	0.11	0.04	0.02	-0.01
-1.28	-0.10	-0.15	-0.22	-0.25	-0.12	-0.14	-0.19	-0.22	-0.15	-0.30	-0.23

-0.78	-0.02	0.06	0.01	0.09	-0.10	0.07	0.04	-0.09	0.04	0.07	0.04
-0.28	-0.30	-0.26	-0.22	-0.16	-0.10	-0.20	-0.20	-0.27	-0.28	-0.19	-0.09
0.22	0.14	0.07	0.20	0.11	0.28	0.13	0.25	0.23	0.11	0.27	0.17
0.32	0.13	0.09	0.29	0.16	0.14	0.18	0.17	0.08	0.37	0.16	0.17
0.42	-0.24	-0.09	-0.24	-0.26	-0.39	0.04	-0.21	-0.08	-0.23	-0.32	-0.06
0.52	-0.23	-0.14	-0.34	-0.37	-0.22	-0.36	-0.21	-0.31	-0.28	-0.33	-0.24
0.62	0.07	0.23	-0.03	0.06	-0.03	0.19	0.36	0.12	0.16	0.17	0.02
0.72	-0.46	-0.11	-0.10	-0.21	-0.06	-0.23	-0.13	-0.21	-0.04	-0.29	-0.16
0.77	0.22	0.23	0.21	0.18	0.08	0.25	0.15	0.24	0.26	0.22	0.31
0.82	0.02	0.16	0.27	0.20	0.17	0.24	0.30	0.21	0.09	0.30	0.29
0.87	-0.02	0.26	0.02	0.03	0.07	-0.04	-0.02	-0.07	0.12	-0.10	-0.02
0.92	0.02	0.02	-0.04	-0.09	-0.03	-0.07	0.00	-0.04	-0.07	0.03	0.08
0.97	-0.14	-0.24	-0.23	-0.21	-0.12	-0.13	-0.01	-0.26	-0.23	-0.03	-0.16
1.02	-0.07	0.04	-0.03	-0.03	-0.30	-0.25	-0.05	0.06	0.02	-0.05	-0.07
1.07	0.14	0.27	0.20	0.12	0.14	0.13	-0.03	0.07	0.33	0.14	0.05
1.12	0.21	-0.03	0.02	-0.10	-0.20	-0.11	-0.11	0.00	-0.22	-0.16	-0.08
1.17	-0.33	-0.20	-0.34	-0.30	-0.29	-0.28	-0.33	-0.19	-0.20	-0.15	-0.13
1.22	-0.67	-0.47	-0.49	-0.42	-0.53	-0.43	-0.55	-0.47	-0.38	-0.30	-0.15
1.27	2.04	1.82	1.26	1.08	0.56	0.26	0.05	-0.19	-0.41	-0.67	-0.79
1.32	4.79	4.16	3.82	3.45	2.86	2.73	2.21	1.82	1.49	0.95	0.85
1.37	7.30	7.30	7.13	6.95	6.49	6.49	6.16	5.70	5.45	5.06	4.35
1.42	2.79	2.66	2.00	1.72	1.20	1.02	0.66	0.44	0.22	-0.15	-0.33
1.47	1.78	1.49	0.87	0.30	-0.45	-0.89	-1.50	-1.99	-2.67	-3.22	-3.91
1.52	1.27	0.66	0.04	-0.48	-1.05	-1.59	-2.36	-2.86	-3.50	-4.16	-4.90
1.57	0.81	0.49	-0.34	-0.88	-1.35	-1.96	-2.50	-3.09	-3.89	-4.69	-5.33
1.62	1.19	0.57	0.02	-0.37	-1.26	-1.66	-2.14	-2.97	-3.61	-4.26	-5.10
1.67	0.74	0.30	-0.33	-0.85	-1.56	-1.87	-2.38	-3.13	-3.68	-4.24	-4.93
1.72	0.57	-0.03	-0.42	-0.94	-1.23	-1.80	-2.34	-3.01	-3.50	-4.06	-4.68
1.77	0.34	0.00	-0.31	-0.84	-1.54	-1.92	-2.42	-3.08	-3.44	-4.05	-4.68
1.82	0.62	0.19	-0.31	-0.62	-1.51	-1.88	-2.30	-2.92	-3.42	-3.94	-4.44
1.87	0.75	0.11	-0.23	-0.85	-1.38	-1.75	-2.40	-2.77	-3.32	-4.02	-4.27
1.92	0.61	0.26	-0.11	-0.63	-1.16	-1.60	-2.06	-2.70	-3.01	-3.57	-4.29
1.97	0.54	0.02	-0.26	-0.86	-1.45	-1.74	-2.14	-2.79	-3.19	-3.75	-4.34
2.02	0.56	0.11	-0.45	-0.86	-1.48	-1.82	-2.27	-2.66	-2.95	-3.61	-4.12
2.07	0.41	-0.02	-0.29	-0.98	-1.33	-1.75	-2.23	-2.58	-3.14	-3.72	-4.23
2.12	0.43	-0.06	-0.52	-0.90	-1.46	-1.86	-2.36	-2.63	-3.06	-3.82	-4.10
2.17	0.53	0.12	-0.14	-0.62	-1.26	-1.54	-2.13	-2.41	-2.92	-3.51	-3.89
2.22	0.69	-0.06	-0.16	-0.76	-1.17	-1.59	-2.05	-2.47	-2.85	-3.58	-3.95
2.27	0.52	0.07	-0.31	-0.84	-1.31	-1.64	-2.11	-2.57	-2.94	-3.38	-3.84
2.32	0.17	-0.11	-0.49	-1.09	-1.44	-1.87	-2.27	-2.78	-3.20	-3.67	-4.29
2.37	0.33	-0.08	-0.49	-0.89	-1.36	-1.83	-2.29	-2.57	-2.96	-3.62	-4.12
2.42	0.53	0.10	-0.23	-0.68	-1.13	-1.75	-2.17	-2.45	-3.02	-3.56	-3.94
2.47	0.53	0.35	-0.10	-0.68	-1.06	-1.45	-1.87	-2.37	-2.82	-3.17	-3.84
2.52	0.33	-0.14	-0.64	-0.90	-1.43	-1.80	-2.06	-2.65	-3.29	-3.56	-4.05
2.57	0.09	-0.24	-0.64	-1.16	-1.73	-2.09	-2.47	-2.79	-3.19	-3.71	-4.15
2.62	0.03	-0.33	-0.71	-1.45	-1.63	-2.02	-2.50	-2.89	-3.55	-3.91	-4.40
2.67	0.20	-0.35	-0.59	-0.98	-1.48	-1.92	-2.45	-2.79	-3.22	-3.79	-4.16
2.72	0.10	-0.02	-0.68	-1.08	-1.31	-1.78	-2.27	-2.59	-3.16	-3.43	-3.90
2.77	-0.15	-0.68	-0.88	-1.40	-1.84	-2.27	-2.63	-3.10	-3.54	-4.01	-4.51
2.82	0.30	-0.01	-0.32	-0.86	-1.33	-1.76	-2.19	-2.58	-2.89	-3.47	-3.85

2.87	-0.27	-0.52	-0.93	-1.21	-1.77	-2.04	-2.54	-2.73	-3.43	-3.69	-4.23
2.92	0.25	-0.18	-0.48	-1.03	-1.42	-1.83	-2.24	-2.52	-3.28	-3.57	-4.02
2.97	-0.23	-0.39	-0.87	-1.41	-1.96	-2.19	-2.50	-2.95	-3.40	-3.94	-4.53
3.02	-0.09	-0.36	-0.78	-1.21	-1.63	-1.94	-2.32	-2.90	-3.24	-3.76	-4.18
3.07	-0.06	-0.59	-0.84	-1.20	-1.72	-2.02	-2.38	-2.83	-3.42	-3.69	-4.25
3.12	-0.07	-0.26	-0.60	-1.27	-1.52	-1.96	-2.47	-2.88	-3.22	-3.66	-4.22
3.17	0.02	-0.30	-0.89	-1.09	-1.78	-2.10	-2.48	-2.84	-3.38	-3.94	-4.32
3.22	0.01	-0.41	-0.84	-1.16	-1.63	-2.10	-2.49	-2.72	-3.28	-3.79	-4.21
3.27	0.21	-0.10	-0.58	-1.01	-1.48	-1.88	-2.01	-2.58	-3.10	-3.61	-4.08
3.32	0.40	-0.14	-0.17	-0.89	-1.42	-1.66	-1.97	-2.39	-2.80	-3.39	-4.00
3.37	-0.07	-0.22	-0.65	-1.25	-1.84	-2.09	-2.34	-2.77	-3.35	-3.73	-4.21
3.42	-0.40	-0.71	-1.01	-1.52	-1.90	-2.25	-2.74	-3.10	-3.55	-3.98	-4.43
3.47	-0.02	-0.45	-0.73	-1.12	-1.71	-2.05	-2.40	-3.00	-3.17	-3.76	-3.93
3.52	-0.19	-0.69	-0.87	-1.30	-1.77	-2.14	-2.46	-3.10	-3.09	-3.75	-4.21
3.57	-0.12	-0.58	-1.03	-1.32	-1.73	-2.08	-2.64	-2.88	-3.55	-3.89	-4.30
3.62	-0.25	-0.59	-1.04	-1.35	-1.87	-2.35	-2.66	-3.16	-3.56	-4.05	-4.33
3.67	-0.33	-0.58	-0.98	-1.46	-1.73	-2.14	-2.59	-3.06	-3.37	-3.98	-4.33
3.72	-0.09	-0.38	-0.74	-1.16	-1.66	-2.02	-2.41	-2.69	-3.16	-3.59	-4.17
3.92	-0.34	-0.66	-0.95	-1.32	-1.88	-2.10	-2.43	-2.92	-3.24	-3.71	-4.16
4.12	-0.01	-0.29	-0.75	-1.07	-1.74	-2.03	-2.52	-2.85	-3.09	-3.75	-4.10
4.32	0.25	-0.60	-0.88	-1.22	-1.75	-2.00	-2.39	-3.02	-3.36	-3.77	-4.34
4.52	0.03	-0.31	-0.77	-1.30	-1.52	-1.97	-2.26	-2.77	-3.06	-3.55	-4.03
4.72	-0.10	-0.38	-0.88	-1.15	-1.63	-2.14	-2.56	-2.70	-3.27	-3.75	-4.16
4.92	-0.20	-0.53	-0.94	-1.26	-1.76	-2.18	-2.65	-3.08	-3.47	-3.75	-4.40
5.12	-0.33	-0.74	-0.89	-1.51	-1.99	-2.25	-2.66	-3.09	-3.40	-4.08	-4.33
5.32	-0.51	-0.53	-1.01	-1.44	-1.78	-2.31	-2.78	-3.19	-3.61	-4.06	-4.45
5.52	-0.10	-0.45	-0.88	-1.28	-1.81	-2.19	-2.42	-2.82	-3.42	-3.74	-4.21
5.72	-0.49	-0.95	-1.17	-1.69	-2.05	-2.53	-2.87	-3.29	-3.93	-4.26	-4.66
5.92	-0.25	-0.65	-0.90	-1.40	-1.87	-2.05	-2.54	-2.92	-3.19	-3.72	-4.29
6.12	-0.38	-0.59	-1.02	-1.40	-1.91	-2.33	-2.81	-3.09	-3.37	-4.14	-4.29
6.32	-0.19	-0.59	-0.96	-1.34	-1.87	-2.27	-2.67	-3.04	-3.46	-3.95	-4.13
6.52	-0.03	-0.43	-0.83	-1.26	-1.65	-1.94	-2.68	-2.91	-3.29	-3.78	-4.06
6.72	-0.18	-0.69	-1.01	-1.43	-1.89	-2.33	-2.58	-3.02	-3.57	-3.90	-4.38
6.92	-0.47	-0.63	-0.95	-1.48	-1.94	-2.37	-2.76	-3.02	-3.44	-3.79	-4.22
7.12	-0.09	-0.58	-0.83	-1.21	-1.62	-1.91	-2.25	-2.93	-3.12	-3.66	-4.11
7.32	-0.23	-0.49	-0.91	-1.26	-1.85	-2.25	-2.62	-2.88	-3.12	-3.94	-4.28
7.52	-0.04	-0.38	-0.82	-1.29	-1.62	-2.08	-2.38	-2.92	-3.21	-3.63	-4.13
7.72	-0.58	-0.66	-1.21	-1.69	-2.04	-2.49	-2.72	-3.23	-3.64	-3.95	-4.48
7.92	-0.33	-0.56	-0.81	-1.33	-2.05	-2.21	-2.65	-3.03	-3.33	-3.91	-4.37
8.12	-0.38	-0.76	-1.01	-1.74	-2.00	-2.24	-2.56	-3.09	-3.29	-4.02	-4.36
8.32	-0.16	-0.50	-0.90	-1.17	-1.61	-1.96	-2.58	-2.92	-3.38	-3.73	-4.21
8.52	-0.23	-0.68	-0.92	-1.38	-1.79	-2.22	-2.54	-2.91	-3.56	-3.80	-4.29
8.72	-0.22	-0.61	-0.93	-1.39	-1.92	-2.28	-2.56	-2.87	-3.55	-3.86	-4.27
8.92	-0.56	-1.01	-1.20	-1.69	-2.02	-2.47	-2.86	-3.41	-3.72	-4.26	-4.68
9.12	-0.52	-0.59	-1.24	-1.66	-2.07	-2.21	-2.65	-3.14	-3.50	-3.93	-4.44
9.32	-0.86	-1.18	-1.68	-1.88	-2.42	-2.69	-3.02	-3.50	-3.93	-4.36	-4.67
9.52	-0.54	-0.74	-1.22	-1.43	-1.99	-2.36	-2.83	-3.24	-3.51	-3.92	-4.58
9.72	-0.47	-0.73	-0.86	-1.63	-2.01	-2.31	-2.64	-3.29	-3.63	-4.01	-4.52
9.92	-0.40	-0.89	-1.06	-1.60	-2.01	-2.36	-2.71	-3.24	-3.59	-4.07	-4.52
10.12	-0.16	-0.59	-0.86	-1.26	-1.79	-2.14	-2.49	-2.91	-3.35	-3.64	-4.07

10.32	-0.35	-0.69	-1.09	-1.55	-1.89	-2.29	-2.63	-3.17	-3.55	-3.92	-4.27
10.52	-0.47	-0.65	-1.14	-1.64	-2.15	-2.47	-2.77	-3.16	-3.42	-3.87	-4.33
10.72	-0.65	-0.82	-1.22	-1.59	-2.10	-2.51	-2.68	-2.90	-3.42	-4.03	-4.34
10.92	-0.43	-0.51	-0.98	-1.51	-1.68	-2.27	-2.75	-2.94	-3.31	-3.94	-4.18
11.12	-0.50	-0.94	-1.44	-1.72	-2.20	-2.46	-2.86	-3.27	-3.76	-4.25	-4.38
11.32	-0.49	-0.91	-1.19	-1.72	-2.16	-2.44	-2.95	-3.11	-3.55	-4.16	-4.49
11.52	-0.31	-0.55	-1.05	-1.26	-1.77	-2.16	-2.43	-2.72	-3.20	-3.67	-3.97
11.72	-0.39	-0.96	-1.22	-1.77	-1.94	-2.32	-2.66	-3.15	-3.58	-3.96	-4.52
11.92	-0.25	-0.51	-0.87	-1.15	-1.57	-1.95	-2.30	-2.68	-3.31	-3.64	-4.06
12.12	0.13	-0.42	-0.76	-0.91	-1.47	-1.78	-2.10	-2.51	-2.83	-3.48	-3.77
12.32	-0.55	-0.94	-1.36	-1.70	-2.00	-2.58	-2.91	-3.39	-3.79	-4.16	-4.25
12.52	-0.35	-0.61	-1.05	-1.43	-1.93	-2.20	-2.65	-3.08	-3.32	-3.97	-4.22
12.72	-0.80	-1.18	-1.29	-1.84	-2.12	-2.56	-2.88	-3.47	-3.73	-4.34	-4.59
12.92	-0.58	-0.86	-1.07	-1.60	-2.04	-2.51	-2.78	-3.17	-3.69	-4.12	-4.39
13.12	-0.35	-0.58	-0.86	-1.40	-1.84	-2.35	-2.46	-3.05	-3.27	-3.70	-4.03
13.32	-0.69	-0.87	-1.35	-1.82	-2.23	-2.45	-2.68	-3.47	-3.70	-4.20	-4.47
13.52	-0.42	-0.85	-1.13	-1.71	-1.97	-2.38	-2.84	-3.15	-3.61	-4.05	-4.39
13.72	-0.29	-0.74	-1.09	-1.47	-1.92	-2.31	-2.54	-3.13	-3.32	-3.84	-4.10
13.92	-0.58	-0.96	-1.34	-1.71	-1.94	-2.39	-2.82	-3.37	-3.57	-4.04	-4.67
14	-0.40	-0.72	-1.22	-1.67	-2.06	-2.40	-2.62	-3.08	-3.59	-4.10	-4.55
14.08	-0.37	-0.83	-1.10	-1.50	-1.87	-2.48	-2.60	-3.15	-3.38	-4.10	-4.30
14.17	-0.50	-0.90	-1.11	-1.65	-2.14	-2.29	-2.69	-3.08	-3.52	-3.59	-4.12
14.27	-0.50	-0.96	-1.19	-1.70	-2.09	-2.66	-2.74	-3.22	-3.58	-3.98	-4.47
14.38	-0.56	-0.91	-1.16	-1.47	-2.05	-2.35	-2.83	-3.21	-3.53	-4.02	-4.34
14.49	-0.47	-0.90	-1.37	-1.56	-1.85	-2.42	-2.65	-3.10	-3.52	-3.93	-4.46
14.61	-0.49	-0.78	-1.27	-1.58	-1.89	-2.58	-2.79	-3.11	-3.53	-3.86	-4.27
14.75	-0.40	-0.90	-0.88	-1.34	-1.89	-2.29	-2.73	-3.14	-3.43	-3.96	-4.33
14.89	-0.53	-0.76	-1.22	-1.68	-1.98	-2.40	-2.81	-3.28	-3.56	-4.10	-4.27
15.05	-0.54	-0.72	-1.19	-1.42	-2.02	-2.36	-2.77	-3.15	-3.60	-3.96	-4.34
15.21	-0.53	-0.85	-0.95	-1.70	-2.07	-2.46	-2.90	-3.11	-3.61	-3.95	-4.45
15.39	-0.71	-1.05	-1.16	-1.75	-1.95	-2.36	-2.78	-3.13	-3.55	-3.97	-4.41
15.59	-0.60	-1.15	-1.60	-1.97	-2.27	-2.68	-3.07	-3.47	-3.79	-4.28	-4.60
15.8	-0.45	-0.98	-1.38	-1.58	-1.98	-2.24	-2.91	-3.07	-3.59	-3.78	-4.38
16.02	-0.41	-0.90	-1.17	-1.67	-1.95	-2.43	-2.73	-3.17	-3.53	-3.81	-4.21
16.27	-0.44	-0.69	-1.28	-1.67	-2.10	-2.30	-2.67	-3.15	-3.53	-4.07	-4.42
16.53	-0.55	-0.66	-1.20	-1.50	-1.95	-2.23	-2.58	-3.08	-3.42	-3.82	-4.30
16.81	-0.66	-0.85	-1.14	-1.59	-1.81	-2.28	-2.73	-3.09	-3.61	-3.95	-4.48
17.12	-0.49	-0.86	-1.31	-1.59	-1.90	-2.41	-2.73	-3.09	-3.60	-4.02	-4.43
17.45	-0.59	-0.85	-1.26	-1.79	-2.20	-2.42	-2.85	-3.13	-3.56	-3.96	-4.51
17.81	-0.42	-0.86	-1.35	-1.70	-2.08	-2.21	-2.69	-3.23	-3.73	-4.03	-4.50
18.19	-0.58	-0.87	-1.21	-1.56	-1.96	-2.35	-2.60	-3.07	-3.56	-4.03	-4.32
18.6	-0.38	-0.93	-1.28	-1.89	-1.92	-2.49	-2.78	-3.25	-3.80	-3.87	-4.29
19.05	-0.88	-1.17	-1.59	-1.99	-2.34	-2.65	-2.98	-3.40	-3.86	-4.21	-4.62
19.53	-0.35	-0.53	-0.97	-1.56	-1.89	-2.24	-2.63	-3.05	-3.30	-3.80	-4.11
20.05	-0.78	-1.16	-1.42	-1.94	-2.21	-2.55	-3.00	-3.38	-3.55	-4.01	-4.52
20.61	-0.63	-0.86	-1.16	-1.79	-2.05	-2.46	-2.85	-3.22	-3.51	-4.09	-4.36
21.22	-0.63	-0.99	-1.40	-1.90	-2.17	-2.48	-2.92	-3.27	-3.55	-4.26	-4.52
21.87	-0.37	-0.80	-1.18	-1.62	-1.96	-2.37	-2.54	-3.12	-3.58	-3.90	-4.38
22.57	-0.60	-0.88	-1.29	-1.57	-2.23	-2.38	-2.90	-3.23	-3.53	-3.97	-4.40
23.33	-0.62	-0.92	-1.39	-1.64	-2.04	-2.46	-2.60	-3.28	-3.53	-3.74	-4.24

24.15	-0.90	-0.92	-1.46	-1.59	-2.19	-2.45	-2.72	-3.14	-3.59	-4.11	-4.38
25.03	-0.51	-0.89	-1.40	-1.72	-2.00	-2.45	-2.81	-3.25	-3.53	-3.90	-4.37
25.98	-0.88	-1.27	-1.59	-1.87	-2.26	-2.70	-3.09	-3.45	-3.74	-4.42	-4.63
27.01	-0.89	-1.08	-1.50	-1.83	-2.35	-2.65	-2.99	-3.31	-3.75	-4.25	-4.61
28.11	-0.61	-0.83	-1.19	-1.59	-2.10	-2.32	-2.85	-3.15	-3.59	-3.88	-4.29
29.3	-1.28	-1.43	-1.85	-2.27	-2.79	-3.01	-3.44	-3.68	-3.98	-4.54	-4.91
30.59	-0.52	-1.03	-1.40	-1.73	-2.08	-2.45	-2.81	-3.08	-3.41	-3.94	-4.22
31.98	-0.81	-0.89	-1.31	-1.73	-2.38	-2.44	-2.94	-3.21	-3.65	-4.04	-4.47
33.47	-0.47	-0.84	-1.41	-1.75	-1.90	-2.46	-2.65	-3.08	-3.61	-3.91	-4.52
35.09	-0.67	-0.89	-1.35	-1.68	-2.07	-2.43	-2.76	-3.15	-3.41	-3.89	-4.19
36.83	-0.90	-1.15	-1.67	-1.98	-2.30	-2.54	-3.01	-3.27	-3.72	-4.01	-4.39
38.71	-0.87	-1.10	-1.52	-1.82	-2.23	-2.80	-2.88	-3.36	-3.62	-3.91	-4.49
40.73	-0.62	-0.96	-1.37	-1.68	-2.38	-2.70	-2.84	-3.11	-3.61	-4.02	-4.48
42.92	-0.59	-1.23	-1.29	-1.79	-2.30	-2.52	-2.86	-3.34	-3.64	-4.14	-4.52
45.27	-0.82	-1.23	-1.39	-1.85	-2.44	-2.54	-3.04	-3.52	-3.75	-4.12	-4.65
47.81	-0.96	-1.26	-1.63	-1.90	-2.55	-2.84	-2.96	-3.51	-3.92	-4.21	-4.80
50.55	-0.95	-1.37	-1.71	-2.02	-2.43	-2.82	-3.19	-3.65	-3.83	-4.42	-4.61
53.51	-1.29	-1.53	-2.04	-2.47	-2.67	-3.05	-3.28	-3.84	-4.08	-4.42	-4.80
56.69	-1.18	-1.26	-1.89	-2.34	-2.45	-2.86	-3.24	-3.55	-3.98	-4.46	-4.94
60.13	-0.83	-1.19	-1.41	-1.73	-2.24	-2.59	-3.04	-3.41	-3.72	-4.21	-4.54
63.84	-0.91	-1.45	-1.60	-2.10	-2.43	-2.67	-3.19	-3.57	-3.94	-4.33	-4.63
67.83	-1.25	-1.56	-1.92	-2.13	-2.72	-2.91	-3.34	-3.77	-4.04	-4.67	-4.79
72.15	-1.09	-1.51	-1.83	-2.25	-2.59	-2.93	-3.20	-3.61	-3.88	-4.36	-4.73
76.8	-1.00	-1.24	-1.76	-1.93	-2.60	-2.70	-3.16	-3.53	-3.80	-4.39	-4.64
81.81	-1.13	-1.30	-1.74	-2.24	-2.53	-2.83	-3.02	-3.53	-3.82	-4.20	-4.64
87.22	-0.96	-1.42	-1.94	-2.07	-2.63	-2.91	-3.39	-3.61	-3.73	-4.20	-4.79
93.06	-0.92	-1.25	-1.74	-1.90	-2.47	-2.72	-2.98	-3.48	-3.71	-4.24	-4.59
99.35	-0.95	-1.18	-1.54	-1.98	-2.25	-2.68	-3.12	-3.48	-3.66	-4.17	-4.60
106.13	-1.28	-1.55	-1.73	-2.13	-2.67	-2.97	-3.14	-3.56	-3.77	-4.20	-4.66
113.45	-0.84	-1.33	-1.68	-1.95	-2.38	-2.64	-3.17	-3.38	-3.75	-4.09	-4.58
121.34	-1.01	-1.32	-1.67	-2.08	-2.43	-2.85	-3.16	-3.35	-3.90	-4.20	-4.53
129.86	-1.13	-1.45	-1.83	-2.28	-2.58	-2.93	-3.26	-3.53	-4.21	-4.25	-4.66
139.04	-1.08	-1.24	-1.57	-2.08	-2.39	-2.79	-2.98	-3.31	-3.67	-3.93	-4.43
148.94	-1.29	-1.40	-1.91	-2.27	-2.63	-2.83	-3.22	-3.74	-3.95	-4.30	-4.69
159.62	-1.19	-1.38	-1.80	-2.07	-2.42	-2.92	-3.07	-3.40	-3.71	-4.25	-4.53
171.14	-0.89	-1.20	-1.45	-1.87	-2.22	-2.58	-2.72	-3.14	-3.36	-3.91	-4.11
183.56	-1.39	-1.57	-2.02	-2.33	-2.52	-2.92	-3.26	-3.54	-3.94	-4.25	-4.56
196.96	-1.20	-1.33	-1.82	-1.99	-2.41	-2.74	-3.02	-3.35	-3.77	-4.29	-4.39
211.41	-1.11	-1.30	-1.85	-1.98	-2.50	-2.66	-2.99	-3.35	-3.76	-4.07	-4.27
226.99	-1.14	-1.31	-1.89	-1.95	-2.43	-2.80	-3.12	-3.36	-3.68	-4.13	-4.33
243.8	-1.28	-1.55	-1.75	-2.18	-2.60	-3.00	-3.04	-3.41	-3.68	-4.23	-4.36
261.93	-1.34	-1.50	-1.93	-2.27	-2.62	-2.63	-3.04	-3.34	-3.61	-4.12	-4.37
281.48	-0.95	-1.18	-1.78	-2.02	-2.45	-2.81	-2.93	-3.18	-3.48	-3.99	-4.35
302.56	-1.17	-1.39	-1.71	-2.14	-2.49	-2.40	-2.91	-3.38	-3.42	-3.84	-4.22
325.3	-1.36	-1.73	-1.78	-2.30	-2.59	-2.74	-3.20	-3.51	-3.81	-4.18	-4.38
349.83	-1.30	-1.63	-1.84	-2.02	-2.64	-2.77	-2.87	-3.35	-3.48	-3.93	-4.35
376.28	-1.33	-1.45	-1.87	-1.91	-2.43	-2.54	-3.06	-3.15	-3.49	-3.85	-4.27
404.81	-0.92	-1.21	-1.47	-1.67	-2.18	-2.39	-2.53	-2.97	-3.17	-3.76	-3.88
435.58	-1.25	-1.33	-1.83	-2.13	-2.54	-2.73	-3.06	-3.26	-3.62	-3.92	-4.33
468.76	-1.08	-1.25	-1.48	-2.06	-2.16	-2.29	-2.80	-3.05	-3.41	-3.70	-3.88

504.55	-0.89	-1.23	-1.46	-1.93	-2.34	-2.49	-2.57	-2.90	-3.32	-3.55	-3.88
543.15	-1.32	-1.36	-1.75	-2.06	-2.48	-2.50	-2.82	-3.10	-3.39	-3.74	-3.91
584.78	-1.18	-1.52	-1.72	-1.94	-2.32	-2.53	-2.73	-3.19	-3.48	-3.59	-3.79
629.68	-0.98	-1.38	-1.50	-1.82	-2.30	-2.53	-2.70	-3.11	-3.12	-3.50	-3.87
678.1	-0.74	-0.90	-1.35	-1.51	-1.98	-2.19	-2.44	-2.76	-2.78	-3.03	-3.51
730.33	-0.91	-1.15	-1.35	-1.91	-2.10	-2.37	-2.42	-2.72	-3.10	-3.16	-3.63
786.65	-1.05	-1.21	-1.66	-1.70	-2.18	-2.30	-2.68	-2.84	-3.30	-3.40	-3.69
847.4	-1.41	-1.59	-1.80	-1.97	-2.40	-2.42	-2.82	-3.03	-3.23	-3.79	-3.87
912.92	-1.05	-1.40	-1.69	-1.81	-2.11	-2.46	-2.61	-2.94	-3.00	-3.36	-3.54
983.58	-1.03	-1.22	-1.67	-1.64	-2.11	-2.28	-2.49	-2.88	-2.92	-3.32	-3.69
1059.78	-0.93	-1.22	-1.33	-1.63	-1.93	-2.25	-2.30	-2.76	-2.96	-3.28	-3.44
1141.98	-0.77	-1.14	-1.35	-1.63	-1.77	-2.06	-2.32	-2.48	-2.61	-2.88	-3.19
1230.62	-1.21	-1.39	-1.55	-1.90	-2.16	-2.52	-2.52	-2.71	-2.99	-3.42	-3.58
1326.23	-0.91	-1.20	-1.32	-1.85	-1.98	-2.26	-2.37	-2.70	-2.77	-3.14	-3.43
1429.34	-0.80	-1.08	-1.36	-1.69	-1.98	-2.16	-2.27	-2.62	-2.91	-3.16	-3.34
1540.54	-1.27	-1.35	-1.71	-2.14	-2.45	-2.60	-2.80	-3.11	-3.23	-3.49	-3.84
1660.48	-1.02	-1.40	-1.35	-1.79	-2.12	-2.27	-2.50	-2.60	-2.73	-3.15	-3.36
1789.83	-1.48	-1.75	-1.97	-2.31	-2.47	-2.77	-2.97	-3.12	-3.26	-3.75	-4.11
1929.34	-1.09	-1.48	-1.74	-1.99	-2.35	-2.43	-2.61	-3.07	-3.00	-3.44	-3.71
2079.8	-1.47	-1.67	-1.97	-2.06	-2.55	-2.63	-3.13	-3.06	-3.26	-3.86	-3.85
2242.08	-1.36	-1.52	-1.65	-1.91	-2.29	-2.57	-2.63	-2.98	-3.10	-3.53	-3.85
2417.1	-1.18	-1.27	-1.61	-1.98	-2.08	-2.50	-2.70	-2.94	-3.04	-3.32	-3.67
2605.85	-1.51	-1.79	-2.12	-2.22	-2.57	-2.65	-2.99	-3.36	-3.45	-3.74	-4.01
2809.43	-1.57	-1.71	-1.98	-2.14	-2.48	-2.80	-2.81	-3.12	-3.29	-3.65	-3.71
3028.99	-1.31	-1.42	-1.88	-2.09	-2.22	-2.41	-2.52	-2.90	-3.11	-3.29	-3.63
3265.79	-1.32	-1.65	-1.89	-2.24	-2.53	-2.62	-3.00	-3.01	-3.34	-3.68	-3.84
3521.18	-1.70	-1.69	-2.12	-2.16	-2.48	-2.79	-2.99	-3.35	-3.62	-3.98	-4.03
3796.62	-1.50	-1.81	-2.07	-2.40	-2.56	-2.84	-2.91	-3.26	-3.43	-3.83	-3.98

Wavelength (nm) / Time (ps)	475.64	477.05	478.46	479.87	481.28	482.69	484.10	485.50	486.91	488.32	489.73
-3.28	0.23	0.24	0.10	0.16	0.12	0.13	0.28	0.01	0.22	0.17	0.21
-2.78	0.23	0.15	0.11	0.12	0.13	0.02	-0.06	0.11	0.13	0.06	0.09
-2.28	-0.08	-0.01	0.16	0.00	-0.02	0.07	0.00	0.01	0.01	0.07	-0.04
-1.78	-0.13	-0.02	-0.01	0.20	-0.05	0.09	0.01	0.17	0.00	-0.10	-0.04
-1.28	-0.24	-0.35	-0.30	-0.32	-0.20	-0.25	-0.31	-0.20	-0.31	-0.32	-0.18
-0.78	-0.05	0.13	0.07	-0.02	0.13	-0.01	0.01	0.07	-0.03	0.09	-0.01
-0.28	-0.06	-0.27	-0.40	-0.32	-0.36	-0.32	-0.24	-0.41	-0.25	-0.25	-0.30
0.22	0.10	0.13	0.27	0.17	0.26	0.25	0.29	0.23	0.23	0.27	0.27
0.32	0.14	0.14	0.09	0.12	0.08	0.15	-0.02	0.07	0.20	0.21	0.10
0.42	-0.05	-0.08	-0.04	-0.09	-0.27	-0.10	-0.22	-0.08	-0.16	-0.20	-0.12
0.52	-0.51	-0.15	-0.29	-0.25	-0.32	-0.20	-0.38	-0.26	-0.22	-0.28	-0.34
0.62	0.20	0.17	0.13	0.12	0.20	0.07	0.07	0.18	0.15	0.09	0.15
0.72	-0.28	-0.05	-0.32	-0.17	-0.34	-0.24	-0.28	-0.15	-0.24	-0.27	-0.09
0.77	0.13	0.27	0.12	0.27	0.18	0.21	0.15	0.15	0.12	0.23	0.15
0.82	0.42	0.34	0.25	0.28	0.22	0.40	0.25	0.40	0.10	0.14	0.32
0.87	-0.02	0.03	0.02	0.03	-0.02	-0.06	-0.01	0.00	0.14	-0.14	0.08
0.92	-0.09	0.06	-0.10	0.10	-0.15	-0.12	-0.13	0.06	0.14	-0.03	-0.03

0.97	-0.10	-0.05	-0.33	-0.23	-0.15	-0.15	-0.16	-0.09	-0.20	-0.23	-0.08
1.02	0.05	0.05	-0.07	0.01	-0.06	0.05	0.07	-0.03	0.08	0.03	0.05
1.07	-0.05	0.26	0.08	0.18	-0.02	-0.01	0.08	0.15	-0.05	-0.02	0.33
1.12	-0.06	-0.17	-0.17	-0.16	-0.40	-0.19	-0.16	-0.16	-0.17	-0.18	-0.26
1.17	-0.15	-0.23	-0.39	-0.16	-0.31	-0.30	-0.28	-0.20	-0.34	-0.21	-0.22
1.22	-0.16	-0.18	-0.03	0.09	-0.05	0.06	-0.03	0.06	-0.01	-0.16	0.21
1.27	-0.65	-0.32	-0.54	-0.46	-0.62	-0.54	-0.36	-0.35	-0.37	-0.36	-0.53
1.32	0.42	0.26	0.13	-0.01	-0.07	-0.07	-0.40	-0.39	-0.46	-0.51	-0.74
1.37	3.90	3.55	2.96	2.58	2.30	1.92	1.50	1.23	0.94	0.60	0.24
1.42	-0.61	-0.89	-1.04	-1.26	-1.58	-1.72	-1.60	-1.60	-1.51	-1.45	-1.42
1.47	-4.46	-5.14	-5.83	-6.21	-7.00	-7.53	-7.93	-8.28	-8.63	-8.94	-8.95
1.52	-5.55	-6.27	-6.98	-7.64	-8.50	-9.11	-9.77	-10.37	-10.79	-11.25	-11.76
1.57	-5.88	-6.68	-7.56	-8.18	-8.96	-9.79	-10.43	-11.17	-11.68	-12.41	-12.81
1.62	-5.71	-6.45	-7.18	-8.00	-8.86	-9.42	-10.19	-10.81	-11.31	-12.10	-12.69
1.67	-5.42	-6.14	-7.07	-7.56	-8.31	-8.87	-9.75	-10.27	-10.96	-11.48	-11.95
1.72	-5.33	-5.86	-6.44	-7.19	-7.75	-8.54	-9.09	-9.43	-10.10	-10.69	-11.10
1.77	-5.29	-5.81	-6.28	-7.03	-7.73	-8.17	-8.74	-9.53	-9.88	-10.43	-10.82
1.82	-5.11	-5.67	-6.26	-6.83	-7.49	-8.20	-8.76	-9.28	-9.70	-10.26	-10.72
1.87	-4.88	-5.52	-6.25	-6.81	-7.27	-7.78	-8.45	-9.03	-9.46	-10.00	-10.28
1.92	-4.67	-5.22	-5.91	-6.36	-7.07	-7.48	-8.11	-8.62	-9.19	-9.60	-10.06
1.97	-4.88	-5.41	-6.02	-6.42	-7.19	-7.68	-8.33	-8.77	-9.31	-9.63	-10.03
2.02	-4.74	-5.30	-5.77	-6.47	-7.07	-7.48	-7.96	-8.35	-8.91	-9.29	-9.67
2.07	-4.80	-5.21	-5.89	-6.37	-6.92	-7.64	-7.95	-8.57	-8.90	-9.43	-9.74
2.12	-4.78	-5.15	-5.73	-6.31	-6.93	-7.28	-7.75	-8.18	-8.66	-9.14	-9.61
2.17	-4.42	-4.95	-5.63	-6.02	-6.58	-7.00	-7.65	-8.12	-8.52	-8.89	-9.27
2.22	-4.40	-4.80	-5.49	-5.95	-6.56	-6.99	-7.57	-7.87	-8.26	-8.65	-8.96
2.27	-4.23	-4.92	-5.38	-5.99	-6.57	-6.98	-7.36	-7.80	-8.28	-8.67	-9.02
2.32	-4.58	-5.06	-5.82	-6.31	-6.89	-7.26	-7.82	-8.20	-8.61	-9.01	-9.21
2.37	-4.46	-5.06	-5.73	-6.15	-6.69	-7.14	-7.63	-8.11	-8.38	-8.85	-9.14
2.42	-4.38	-4.99	-5.62	-5.99	-6.62	-7.07	-7.40	-7.75	-8.29	-8.69	-8.87
2.47	-4.37	-4.86	-5.30	-5.82	-6.34	-6.96	-7.40	-7.67	-8.14	-8.58	-8.95
2.52	-4.53	-5.15	-5.60	-5.86	-6.67	-7.00	-7.57	-7.84	-8.24	-8.70	-8.97
2.57	-4.76	-5.16	-5.59	-5.95	-6.72	-7.21	-7.50	-7.83	-8.37	-8.62	-8.94
2.62	-4.93	-5.46	-5.85	-6.35	-6.87	-7.45	-7.79	-8.26	-8.52	-8.86	-9.32
2.67	-4.72	-5.08	-5.65	-6.12	-6.67	-7.01	-7.57	-7.95	-8.38	-8.65	-9.01
2.72	-4.47	-4.88	-5.49	-5.94	-6.51	-6.91	-7.47	-7.71	-8.23	-8.56	-8.77
2.77	-5.00	-5.26	-5.91	-6.36	-6.94	-7.50	-7.90	-8.25	-8.68	-9.03	-9.35
2.82	-4.47	-4.73	-5.52	-5.85	-6.53	-6.73	-7.34	-7.67	-8.13	-8.34	-8.65
2.87	-4.85	-5.24	-5.87	-6.10	-6.70	-7.15	-7.56	-7.91	-8.32	-8.61	-8.97
2.92	-4.65	-5.07	-5.50	-5.94	-6.64	-7.09	-7.53	-8.00	-8.43	-8.59	-8.82
2.97	-5.02	-5.39	-5.94	-6.39	-7.07	-7.50	-7.97	-8.41	-8.56	-9.06	-9.35
3.02	-4.81	-5.12	-5.75	-6.30	-6.72	-7.19	-7.69	-8.03	-8.28	-8.81	-9.08
3.07	-4.72	-5.18	-5.62	-6.04	-6.63	-7.01	-7.49	-7.90	-8.12	-8.36	-8.82
3.12	-4.68	-5.29	-5.75	-6.19	-6.63	-7.20	-7.63	-8.06	-8.33	-8.78	-8.92
3.17	-4.88	-5.36	-5.70	-6.16	-6.82	-7.15	-7.70	-8.06	-8.46	-8.77	-8.97
3.22	-4.74	-5.15	-5.60	-6.08	-6.47	-7.09	-7.54	-7.97	-8.28	-8.65	-8.83
3.27	-4.48	-4.85	-5.60	-5.83	-6.41	-6.88	-7.44	-7.80	-8.00	-8.48	-8.67
3.32	-4.31	-4.90	-5.33	-5.77	-6.36	-6.57	-7.23	-7.60	-7.95	-8.26	-8.45
3.37	-4.59	-5.16	-5.59	-6.05	-6.61	-6.95	-7.46	-7.91	-8.23	-8.56	-8.73
3.42	-4.91	-5.41	-5.86	-6.27	-6.73	-7.09	-7.68	-8.02	-8.25	-8.52	-8.76

3.47	-4.61	-5.04	-5.51	-6.04	-6.56	-6.94	-7.47	-7.88	-8.24	-8.33	-8.48
3.52	-4.84	-5.37	-5.90	-6.26	-6.96	-7.28	-7.72	-7.89	-8.38	-8.68	-8.93
3.57	-4.82	-5.28	-5.88	-6.12	-6.62	-7.17	-7.63	-7.92	-8.34	-8.70	-8.81
3.62	-4.95	-5.44	-5.91	-6.48	-6.83	-7.23	-7.61	-8.05	-8.37	-8.70	-8.97
3.67	-4.76	-5.30	-5.85	-6.17	-6.67	-7.19	-7.48	-8.05	-8.43	-8.56	-8.79
3.72	-4.63	-5.03	-5.62	-5.97	-6.49	-6.87	-7.39	-7.69	-7.95	-8.40	-8.51
3.92	-4.79	-5.19	-5.52	-6.09	-6.59	-7.01	-7.40	-7.71	-8.18	-8.26	-8.46
4.12	-4.50	-5.17	-5.80	-6.08	-6.68	-6.97	-7.47	-7.76	-8.15	-8.40	-8.66
4.32	-4.79	-5.20	-5.77	-6.29	-6.62	-7.25	-7.55	-7.95	-8.48	-8.65	-8.96
4.52	-4.47	-4.83	-5.55	-5.89	-6.51	-6.80	-7.30	-7.63	-7.98	-8.33	-8.60
4.72	-4.57	-5.20	-5.57	-6.07	-6.59	-6.90	-7.27	-7.68	-8.12	-8.41	-8.56
4.92	-4.81	-5.08	-5.81	-6.15	-6.80	-7.03	-7.59	-7.84	-8.14	-8.55	-8.76
5.12	-4.81	-5.29	-5.75	-6.25	-6.80	-7.28	-7.70	-7.94	-8.21	-8.60	-8.76
5.32	-4.91	-5.50	-5.88	-6.45	-6.82	-7.21	-7.64	-8.03	-8.31	-8.82	-8.86
5.52	-4.65	-5.01	-5.61	-5.96	-6.49	-6.89	-7.42	-7.85	-7.86	-8.29	-8.49
5.72	-5.14	-5.66	-6.15	-6.54	-7.10	-7.43	-7.91	-8.22	-8.58	-8.84	-9.03
5.92	-4.66	-5.20	-5.53	-6.01	-6.70	-6.86	-7.32	-7.61	-8.08	-8.37	-8.47
6.12	-4.86	-5.23	-5.79	-6.28	-6.78	-7.25	-7.50	-7.95	-8.20	-8.52	-8.74
6.32	-4.82	-5.16	-5.81	-5.97	-6.64	-7.11	-7.51	-7.78	-8.04	-8.40	-8.77
6.52	-4.66	-5.02	-5.68	-6.08	-6.54	-6.94	-7.39	-7.54	-7.98	-8.13	-8.46
6.72	-4.97	-5.34	-5.72	-6.21	-6.73	-7.21	-7.50	-7.99	-8.31	-8.45	-8.63
6.92	-4.84	-5.16	-5.62	-5.99	-6.52	-7.06	-7.40	-7.64	-8.01	-8.38	-8.48
7.12	-4.47	-4.98	-5.40	-5.77	-6.24	-6.54	-7.02	-7.40	-7.62	-7.87	-8.08
7.32	-4.78	-5.25	-5.58	-6.07	-6.55	-6.95	-7.35	-7.68	-8.12	-8.35	-8.43
7.52	-4.79	-5.23	-5.54	-5.92	-6.52	-6.95	-7.31	-7.58	-7.93	-8.38	-8.52
7.72	-5.08	-5.30	-5.81	-6.30	-6.79	-7.20	-7.61	-7.86	-8.20	-8.58	-8.72
7.92	-4.94	-5.20	-5.59	-6.20	-6.80	-6.93	-7.41	-7.77	-8.03	-8.20	-8.37
8.12	-4.77	-5.26	-5.74	-6.03	-6.48	-7.02	-7.34	-7.54	-8.00	-8.36	-8.33
8.32	-4.46	-4.91	-5.54	-5.97	-6.39	-6.86	-7.17	-7.53	-7.93	-8.20	-8.24
8.52	-4.83	-5.07	-5.70	-6.01	-6.63	-6.99	-7.30	-7.78	-8.05	-8.28	-8.56
8.72	-4.70	-4.98	-5.55	-5.98	-6.55	-7.00	-7.44	-7.77	-7.96	-8.35	-8.44
8.92	-5.04	-5.56	-6.00	-6.23	-6.99	-7.09	-7.72	-7.89	-8.31	-8.43	-8.79
9.12	-4.82	-5.21	-5.81	-6.10	-6.62	-6.99	-7.49	-7.80	-8.09	-8.32	-8.58
9.32	-5.25	-5.63	-6.26	-6.46	-7.07	-7.35	-7.75	-8.27	-8.42	-8.77	-8.98
9.52	-4.87	-5.51	-5.85	-6.15	-6.88	-7.34	-7.60	-7.80	-8.15	-8.31	-8.63
9.72	-4.87	-5.31	-5.78	-6.15	-6.76	-7.06	-7.60	-7.97	-8.16	-8.45	-8.63
9.92	-4.88	-5.48	-5.83	-6.37	-6.80	-7.29	-7.71	-7.97	-8.24	-8.56	-8.83
10.12	-4.70	-5.06	-5.57	-6.02	-6.55	-6.95	-7.28	-7.63	-7.81	-8.09	-8.37
10.32	-4.88	-5.10	-5.66	-6.22	-6.59	-6.91	-7.23	-7.58	-8.00	-8.18	-8.33
10.52	-4.80	-5.31	-5.70	-6.10	-6.68	-6.93	-7.43	-7.60	-7.99	-8.31	-8.54
10.72	-4.98	-5.26	-5.85	-6.23	-6.75	-6.93	-7.33	-7.63	-8.06	-8.21	-8.41
10.92	-4.69	-5.09	-5.73	-6.16	-6.44	-6.86	-7.23	-7.73	-8.08	-8.29	-8.30
11.12	-5.05	-5.44	-5.79	-6.29	-6.82	-7.30	-7.42	-7.88	-8.15	-8.37	-8.71
11.32	-5.00	-5.30	-5.71	-6.00	-6.52	-6.97	-7.34	-7.59	-7.99	-8.17	-8.42
11.52	-4.55	-4.95	-5.46	-5.64	-6.33	-6.72	-6.86	-7.29	-7.63	-7.80	-8.16
11.72	-4.72	-5.27	-5.75	-6.12	-6.61	-6.86	-7.39	-7.52	-7.83	-8.15	-8.34
11.92	-4.64	-4.85	-5.39	-5.81	-6.30	-6.55	-6.88	-7.09	-7.37	-7.73	-8.05
12.12	-4.34	-4.53	-5.20	-5.47	-6.12	-6.47	-6.72	-7.08	-7.18	-7.54	-7.66
12.32	-4.95	-5.53	-5.66	-6.11	-6.77	-7.12	-7.45	-7.79	-8.12	-8.26	-8.50
12.52	-4.66	-5.13	-5.64	-5.86	-6.56	-6.96	-7.14	-7.50	-7.87	-7.99	-8.12

12.72	-5.24	-5.55	-6.13	-6.35	-7.03	-7.33	-7.61	-7.91	-8.15	-8.40	-8.66
12.92	-4.81	-5.22	-5.78	-6.14	-6.76	-6.97	-7.29	-7.72	-7.88	-8.25	-8.36
13.12	-4.55	-5.00	-5.55	-6.11	-6.44	-6.73	-7.13	-7.42	-7.78	-8.14	-8.20
13.32	-5.00	-5.41	-5.98	-6.31	-6.94	-7.22	-7.65	-8.02	-8.33	-8.55	-8.69
13.52	-4.79	-5.27	-5.80	-6.07	-6.62	-7.22	-7.46	-7.71	-7.91	-8.27	-8.54
13.72	-4.62	-5.05	-5.57	-5.98	-6.43	-6.89	-7.31	-7.51	-7.65	-8.05	-8.23
13.92	-4.91	-5.36	-5.87	-6.21	-6.58	-7.18	-7.57	-7.75	-8.10	-8.33	-8.64
14	-4.84	-5.21	-5.63	-5.92	-6.39	-6.91	-7.35	-7.64	-8.00	-8.09	-8.29
14.08	-4.66	-5.17	-5.76	-6.16	-6.55	-6.90	-7.29	-7.80	-8.00	-8.26	-8.47
14.17	-4.80	-5.16	-5.51	-5.86	-6.38	-6.83	-7.14	-7.39	-7.76	-7.97	-8.06
14.27	-4.69	-5.23	-5.72	-6.12	-6.72	-6.98	-7.42	-7.66	-7.88	-8.10	-8.33
14.38	-4.86	-5.31	-5.76	-6.18	-6.57	-6.93	-7.26	-7.67	-7.98	-8.25	-8.35
14.49	-4.83	-5.07	-5.61	-5.88	-6.62	-6.83	-7.25	-7.48	-7.64	-8.14	-8.31
14.61	-4.95	-5.39	-5.95	-6.04	-6.76	-7.12	-7.46	-7.91	-8.15	-8.26	-8.41
14.75	-4.81	-5.15	-5.61	-6.12	-6.45	-6.97	-7.43	-7.68	-7.93	-8.08	-8.37
14.89	-4.99	-5.25	-5.87	-6.19	-6.63	-6.92	-7.43	-7.65	-7.95	-8.36	-8.33
15.05	-4.81	-5.36	-5.67	-6.10	-6.47	-6.99	-7.27	-7.54	-7.79	-8.03	-8.22
15.21	-4.91	-5.20	-5.79	-6.12	-6.77	-7.06	-7.40	-7.70	-7.94	-8.22	-8.29
15.39	-4.96	-5.48	-5.71	-6.18	-6.61	-7.01	-7.50	-7.75	-7.97	-8.22	-8.41
15.59	-5.18	-5.41	-5.98	-6.31	-6.79	-7.24	-7.63	-7.82	-8.25	-8.35	-8.65
15.8	-4.69	-5.13	-5.53	-6.06	-6.45	-6.81	-7.21	-7.50	-7.53	-8.08	-8.17
16.02	-4.74	-5.00	-5.69	-5.91	-6.49	-6.84	-7.08	-7.40	-7.76	-7.93	-8.04
16.27	-4.84	-5.36	-5.81	-6.14	-6.53	-7.01	-7.28	-7.54	-7.77	-7.95	-8.31
16.53	-4.70	-5.03	-5.47	-6.02	-6.53	-6.68	-7.25	-7.45	-7.91	-8.02	-8.26
16.81	-5.01	-5.53	-5.78	-6.26	-6.52	-7.02	-7.45	-7.72	-7.88	-8.14	-8.41
17.12	-4.84	-5.28	-5.55	-6.01	-6.52	-6.97	-7.33	-7.63	-7.80	-8.05	-8.29
17.45	-4.67	-5.26	-5.73	-6.15	-6.65	-6.94	-7.25	-7.60	-7.96	-8.16	-8.53
17.81	-4.82	-5.25	-5.67	-6.02	-6.52	-6.93	-7.16	-7.49	-7.74	-8.10	-8.16
18.19	-4.82	-5.17	-5.64	-5.93	-6.47	-6.77	-7.12	-7.50	-7.87	-7.97	-8.26
18.6	-4.94	-5.16	-5.52	-6.09	-6.45	-6.82	-7.22	-7.40	-7.82	-8.02	-8.14
19.05	-5.05	-5.43	-5.95	-6.37	-7.03	-7.29	-7.66	-7.88	-8.29	-8.47	-8.60
19.53	-4.83	-5.03	-5.57	-6.08	-6.46	-6.77	-7.23	-7.44	-7.78	-7.90	-8.02
20.05	-4.85	-5.34	-5.76	-6.18	-6.55	-7.05	-7.20	-7.50	-7.81	-8.03	-8.20
20.61	-4.73	-5.18	-5.63	-6.00	-6.60	-7.04	-7.33	-7.61	-7.93	-8.07	-8.46
21.22	-4.99	-5.38	-5.71	-6.13	-6.69	-7.13	-7.37	-7.72	-7.92	-8.23	-8.40
21.87	-4.76	-5.20	-5.72	-5.98	-6.44	-6.85	-7.20	-7.58	-7.82	-8.00	-8.25
22.57	-4.86	-5.31	-5.77	-6.27	-6.79	-6.89	-7.41	-7.74	-7.91	-8.16	-8.36
23.33	-4.70	-5.09	-5.57	-5.88	-6.40	-6.61	-7.18	-7.38	-7.61	-7.85	-8.08
24.15	-4.74	-5.22	-5.61	-5.94	-6.43	-6.75	-7.02	-7.37	-7.63	-7.92	-8.07
25.03	-4.90	-5.12	-5.65	-6.10	-6.48	-6.74	-7.05	-7.50	-7.80	-7.90	-8.11
25.98	-5.21	-5.56	-6.00	-6.41	-6.97	-7.25	-7.58	-7.92	-8.22	-8.45	-8.58
27.01	-5.08	-5.51	-5.82	-6.30	-6.76	-7.14	-7.40	-7.75	-8.10	-8.25	-8.56
28.11	-4.65	-5.15	-5.46	-6.05	-6.29	-6.88	-7.03	-7.31	-7.77	-7.94	-8.05
29.3	-5.31	-5.78	-6.09	-6.48	-6.89	-7.20	-7.74	-8.06	-8.17	-8.46	-8.61
30.59	-4.63	-5.13	-5.60	-5.86	-6.36	-6.54	-6.98	-7.16	-7.36	-7.63	-7.86
31.98	-4.75	-5.21	-5.71	-6.01	-6.46	-6.93	-7.34	-7.59	-7.65	-8.14	-8.25
33.47	-4.86	-5.05	-5.57	-5.92	-6.32	-6.78	-7.14	-7.30	-7.60	-7.99	-7.95
35.09	-4.71	-4.95	-5.61	-5.70	-6.37	-6.76	-7.08	-7.34	-7.66	-7.94	-8.03
36.83	-4.91	-5.22	-5.84	-6.08	-6.50	-6.97	-7.40	-7.50	-7.84	-8.11	-8.17
38.71	-4.92	-5.36	-5.74	-6.17	-6.74	-6.93	-7.19	-7.59	-7.78	-7.95	-8.20

40.73	-4.95	-5.40	-5.69	-6.27	-6.66	-6.98	-7.31	-7.68	-7.97	-8.06	-8.26
42.92	-4.71	-5.52	-5.80	-6.21	-6.68	-7.16	-7.43	-7.71	-7.96	-8.12	-8.20
45.27	-5.23	-5.50	-5.87	-6.19	-6.62	-7.07	-7.47	-7.71	-8.06	-8.21	-8.35
47.81	-5.31	-5.57	-6.01	-6.55	-6.91	-7.31	-7.57	-7.95	-8.17	-8.22	-8.46
50.55	-5.28	-5.47	-5.99	-6.28	-6.78	-7.12	-7.49	-7.70	-8.04	-8.29	-8.27
53.51	-5.24	-5.64	-6.11	-6.35	-6.76	-7.12	-7.40	-7.69	-8.12	-8.26	-8.33
56.69	-5.31	-5.43	-6.17	-6.47	-6.91	-7.19	-7.58	-7.86	-8.16	-8.33	-8.51
60.13	-5.02	-5.26	-5.64	-6.16	-6.58	-6.75	-7.32	-7.49	-7.85	-8.07	-8.20
63.84	-5.04	-5.44	-5.95	-6.23	-6.73	-6.95	-7.49	-7.75	-7.93	-8.24	-8.53
67.83	-5.30	-5.70	-6.20	-6.48	-6.81	-7.28	-7.67	-7.93	-8.24	-8.52	-8.72
72.15	-5.11	-5.56	-5.93	-6.33	-6.76	-6.99	-7.38	-7.65	-7.92	-8.10	-8.25
76.8	-5.11	-5.44	-5.75	-6.06	-6.57	-6.93	-7.28	-7.48	-7.71	-7.94	-8.09
81.81	-4.99	-5.50	-5.90	-5.94	-6.68	-6.89	-7.10	-7.62	-7.88	-8.20	-8.24
87.22	-5.06	-5.49	-5.96	-6.28	-6.73	-7.09	-7.38	-7.59	-7.84	-8.33	-8.33
93.06	-5.26	-5.34	-5.78	-6.12	-6.61	-6.93	-7.35	-7.62	-7.84	-8.03	-8.31
99.35	-5.07	-5.20	-5.83	-6.13	-6.62	-6.94	-7.37	-7.77	-7.69	-8.13	-8.26
106.13	-5.10	-5.33	-5.68	-6.07	-6.78	-6.85	-7.30	-7.54	-7.70	-7.97	-8.01
113.45	-4.93	-5.36	-5.73	-6.11	-6.45	-6.90	-7.12	-7.47	-7.74	-8.02	-8.05
121.34	-5.13	-5.12	-5.88	-6.08	-6.73	-6.83	-7.27	-7.47	-7.69	-7.95	-8.22
129.86	-5.25	-5.49	-6.15	-6.39	-6.86	-7.21	-7.55	-7.76	-7.90	-8.18	-8.47
139.04	-4.88	-5.19	-5.54	-5.85	-6.36	-6.65	-7.07	-7.33	-7.49	-7.82	-8.02
148.94	-5.16	-5.57	-5.77	-6.23	-6.87	-7.16	-7.41	-7.52	-7.86	-8.05	-8.19
159.62	-4.83	-5.29	-5.64	-6.04	-6.51	-6.66	-7.04	-7.32	-7.58	-7.80	-8.00
171.14	-4.45	-4.95	-5.30	-5.70	-6.24	-6.39	-6.88	-7.04	-7.29	-7.65	-7.56
183.56	-5.06	-5.31	-5.79	-6.17	-6.54	-6.86	-7.27	-7.62	-7.84	-7.87	-8.11
196.96	-4.73	-5.24	-5.61	-5.98	-6.43	-6.72	-7.19	-7.45	-7.69	-7.88	-8.02
211.41	-4.96	-5.14	-5.59	-5.93	-6.36	-6.75	-7.15	-7.34	-7.56	-7.71	-7.98
226.99	-4.71	-5.07	-5.52	-6.05	-6.52	-6.64	-7.02	-7.32	-7.40	-7.77	-7.92
243.8	-4.63	-5.15	-5.57	-5.84	-6.35	-6.57	-7.05	-7.25	-7.45	-7.69	-7.88
261.93	-4.76	-5.00	-5.48	-5.93	-6.37	-6.51	-6.90	-7.10	-7.47	-7.63	-7.87
281.48	-4.68	-5.04	-5.48	-5.78	-6.22	-6.31	-6.83	-7.09	-7.42	-7.70	-7.81
302.56	-4.74	-4.81	-5.39	-5.74	-5.98	-6.50	-6.75	-7.14	-7.16	-7.41	-7.58
325.3	-4.81	-5.21	-5.58	-5.70	-6.29	-6.57	-6.85	-7.18	-7.52	-7.69	-7.67
349.83	-4.67	-4.92	-5.27	-5.52	-5.91	-6.37	-6.66	-6.71	-7.06	-7.46	-7.49
376.28	-4.62	-4.82	-5.40	-5.59	-6.00	-6.41	-6.81	-7.01	-7.14	-7.46	-7.58
404.81	-4.28	-4.43	-5.12	-5.25	-5.59	-5.86	-6.34	-6.49	-6.72	-7.05	-7.01
435.58	-4.55	-4.84	-5.33	-5.70	-6.07	-6.22	-6.49	-6.92	-7.22	-7.34	-7.54
468.76	-4.42	-4.47	-5.01	-5.36	-5.66	-6.05	-6.32	-6.74	-6.63	-7.13	-7.17
504.55	-4.24	-4.55	-4.89	-5.22	-5.52	-5.91	-6.19	-6.36	-6.80	-6.91	-7.02
543.15	-4.31	-4.70	-4.95	-5.15	-5.81	-6.09	-6.19	-6.54	-6.68	-6.99	-7.03
584.78	-4.37	-4.50	-4.68	-5.21	-5.55	-5.76	-6.12	-6.36	-6.55	-6.77	-6.96
629.68	-4.19	-4.31	-4.71	-5.11	-5.39	-5.58	-6.07	-6.19	-6.67	-6.61	-6.89
678.1	-3.71	-4.04	-4.29	-4.59	-5.18	-5.48	-5.61	-5.78	-6.15	-6.45	-6.50
730.33	-3.99	-4.21	-4.67	-4.91	-5.43	-5.53	-5.90	-6.09	-6.38	-6.63	-6.64
786.65	-3.90	-4.29	-4.68	-4.84	-5.18	-5.57	-5.69	-6.09	-6.21	-6.43	-6.77
847.4	-4.14	-4.60	-4.64	-5.06	-5.56	-5.80	-6.17	-6.25	-6.46	-6.61	-6.78
912.92	-4.03	-4.07	-4.68	-4.72	-5.37	-5.52	-5.85	-6.10	-6.31	-6.43	-6.67
983.58	-3.87	-4.12	-4.28	-4.71	-5.19	-5.40	-5.75	-5.94	-6.15	-6.22	-6.46
1059.78	-3.79	-4.03	-4.44	-4.67	-4.99	-5.33	-5.61	-5.75	-6.23	-6.35	-6.28
1141.98	-3.49	-3.78	-3.96	-4.39	-4.86	-4.93	-5.23	-5.58	-5.72	-5.90	-5.97

1230.62	-3.96	-4.18	-4.37	-4.43	-5.07	-5.34	-5.69	-5.77	-5.83	-6.01	-6.30
1326.23	-3.78	-4.08	-4.31	-4.56	-5.04	-5.40	-5.59	-5.90	-6.03	-6.05	-6.32
1429.34	-3.67	-3.90	-4.38	-4.55	-4.92	-5.19	-5.42	-5.79	-6.00	-6.15	-6.19
1540.54	-4.06	-4.17	-4.54	-4.85	-5.35	-5.58	-5.85	-6.02	-6.22	-6.67	-6.64
1660.48	-3.92	-4.10	-4.19	-4.56	-5.03	-5.24	-5.70	-5.73	-5.96	-6.23	-6.28
1789.83	-4.22	-4.39	-4.77	-4.97	-5.52	-5.56	-5.81	-6.11	-6.35	-6.43	-6.55
1929.34	-3.93	-4.23	-4.43	-4.75	-5.21	-5.51	-5.78	-6.04	-6.27	-6.40	-6.63
2079.8	-4.14	-4.36	-4.77	-5.11	-5.52	-5.65	-5.92	-6.12	-6.24	-6.57	-6.56
2242.08	-3.94	-4.33	-4.64	-4.80	-5.19	-5.55	-5.75	-5.92	-6.23	-6.39	-6.57
2417.1	-3.94	-4.26	-4.76	-4.99	-5.22	-5.50	-5.75	-5.98	-6.21	-6.61	-6.54
2605.85	-4.36	-4.55	-4.78	-5.05	-5.37	-5.85	-5.97	-6.11	-6.49	-6.50	-6.66
2809.43	-4.04	-4.50	-4.66	-4.85	-5.49	-5.43	-5.75	-6.14	-6.35	-6.41	-6.59
3028.99	-3.99	-4.13	-4.59	-4.65	-5.13	-5.39	-5.47	-5.84	-6.08	-6.30	-6.33
3265.79	-4.19	-4.33	-4.67	-4.87	-5.23	-5.48	-5.78	-6.06	-6.38	-6.28	-6.52
3521.18	-4.34	-4.44	-4.82	-5.16	-5.57	-5.70	-6.08	-6.38	-6.53	-6.79	-6.95
3796.62	-4.23	-4.50	-4.86	-5.13	-5.59	-5.78	-5.95	-6.16	-6.42	-6.51	-6.73

Wavelength (nm) / Time (ps)	491.14	492.55	493.96	495.36	496.77	498.18	499.59	501.00	502.41	503.82	505.22
-3.28	0.18	0.14	0.14	0.11	0.19	0.18	0.18	0.30	0.11	0.07	0.16
-2.78	-0.04	0.07	-0.01	0.06	0.03	0.08	-0.05	0.03	0.08	0.12	0.08
-2.28	0.02	0.09	0.06	0.16	0.09	0.11	0.12	0.12	0.18	0.24	0.15
-1.78	0.01	0.01	-0.03	-0.08	-0.01	-0.03	-0.02	0.01	0.02	-0.01	-0.01
-1.28	-0.21	-0.21	-0.25	-0.28	-0.31	-0.28	-0.10	-0.26	-0.27	-0.33	-0.15
-0.78	0.04	-0.04	0.03	0.02	-0.04	-0.02	-0.06	0.03	-0.09	-0.02	-0.08
-0.28	-0.17	-0.25	-0.23	-0.19	-0.28	-0.23	-0.31	-0.38	-0.29	-0.44	-0.43
0.22	0.16	0.18	0.30	0.21	0.33	0.19	0.25	0.16	0.25	0.37	0.27
0.32	0.04	0.12	0.06	0.12	0.18	0.10	0.19	0.07	0.12	0.19	0.02
0.42	-0.23	-0.19	-0.17	-0.26	-0.01	-0.26	-0.32	-0.26	-0.17	-0.11	-0.15
0.52	-0.30	-0.18	-0.28	-0.32	-0.21	-0.36	-0.28	-0.33	-0.23	-0.13	-0.32
0.62	0.15	0.24	0.09	0.18	0.21	0.29	0.07	0.12	0.19	0.09	0.23
0.72	-0.41	-0.37	-0.41	-0.23	-0.28	-0.35	-0.36	-0.28	-0.35	-0.23	-0.38
0.77	0.15	0.18	0.20	0.26	0.17	0.13	0.33	0.19	0.21	0.15	0.29
0.82	0.16	0.23	0.30	0.24	0.30	0.19	0.21	0.25	0.33	0.33	0.19
0.87	0.16	0.10	-0.04	-0.04	0.11	0.02	0.07	0.03	0.00	0.01	-0.04
0.92	0.03	-0.06	-0.06	-0.02	-0.01	0.04	-0.09	0.02	-0.13	-0.04	-0.13
0.97	-0.20	0.02	-0.12	0.00	-0.22	0.04	-0.12	-0.27	-0.06	-0.06	0.03
1.02	0.10	0.20	-0.01	-0.06	0.08	0.02	-0.12	0.01	0.11	0.17	0.04
1.07	-0.05	0.07	0.00	-0.07	0.16	0.17	-0.07	-0.02	-0.04	-0.03	0.00
1.12	-0.17	-0.29	-0.27	-0.19	-0.12	-0.29	-0.11	-0.16	-0.25	-0.17	-0.20
1.17	-0.34	-0.20	-0.07	-0.26	-0.19	-0.26	-0.21	-0.33	-0.22	-0.27	-0.22
1.22	0.03	0.06	0.10	0.09	0.22	0.11	0.08	0.14	0.26	0.00	0.19
1.27	-0.54	-0.29	-0.42	-0.16	-0.24	-0.07	-0.09	-0.17	-0.09	-0.09	-0.09
1.32	-0.63	-0.62	-0.54	-0.56	-0.42	-0.38	-0.42	-0.28	-0.40	-0.36	-0.42
1.37	0.05	0.03	-0.13	-0.15	-0.22	-0.18	-0.24	-0.27	-0.22	-0.41	-0.46
1.42	-1.16	-1.08	-0.91	-0.65	-0.51	-0.40	-0.28	-0.09	0.03	0.29	0.18
1.47	-9.21	-9.24	-9.37	-9.15	-9.01	-8.79	-8.50	-8.19	-7.84	-7.43	-7.02
1.52	-12.17	-12.37	-12.61	-12.84	-12.99	-12.93	-12.96	-12.91	-12.60	-12.46	-12.17

1.57	-13.49	-13.87	-14.30	-14.41	-14.76	-14.94	-14.95	-15.05	-14.99	-15.10	-14.98
1.62	-13.14	-13.71	-14.19	-14.45	-14.66	-14.95	-15.19	-15.26	-15.32	-15.39	-15.38
1.67	-12.66	-13.03	-13.27	-13.61	-13.99	-14.11	-14.43	-14.47	-14.42	-14.50	-14.60
1.72	-11.50	-11.69	-12.23	-12.42	-12.62	-12.67	-12.80	-12.88	-12.86	-13.01	-13.01
1.77	-11.09	-11.45	-11.75	-11.89	-11.99	-12.22	-12.28	-12.24	-12.24	-12.15	-12.32
1.82	-11.13	-11.28	-11.68	-11.68	-11.86	-11.94	-12.08	-12.04	-11.93	-11.83	-11.91
1.87	-10.66	-11.04	-11.14	-11.29	-11.48	-11.43	-11.64	-11.46	-11.55	-11.41	-11.33
1.92	-10.28	-10.44	-10.81	-10.94	-11.06	-11.27	-11.25	-11.18	-10.99	-10.98	-10.92
1.97	-10.45	-10.70	-10.84	-11.03	-10.94	-11.08	-11.15	-11.26	-10.85	-10.79	-10.67
2.02	-9.97	-10.13	-10.36	-10.48	-10.60	-10.55	-10.54	-10.37	-10.34	-10.20	-10.08
2.07	-10.22	-10.29	-10.51	-10.51	-10.61	-10.65	-10.52	-10.60	-10.28	-10.23	-10.12
2.12	-9.78	-9.95	-10.08	-10.22	-10.46	-10.37	-10.49	-10.40	-10.12	-9.85	-9.79
2.17	-9.65	-9.76	-9.95	-9.99	-9.94	-10.14	-10.11	-9.86	-9.71	-9.51	-9.50
2.22	-9.24	-9.38	-9.66	-9.89	-9.75	-9.73	-9.62	-9.60	-9.44	-9.31	-9.15
2.27	-9.11	-9.37	-9.41	-9.55	-9.51	-9.41	-9.55	-9.46	-9.08	-8.95	-8.88
2.32	-9.48	-9.57	-9.83	-9.85	-9.92	-9.88	-9.81	-9.75	-9.42	-9.27	-9.13
2.37	-9.34	-9.54	-9.77	-9.68	-9.79	-9.73	-9.61	-9.36	-9.25	-9.07	-8.98
2.42	-9.33	-9.43	-9.67	-9.50	-9.59	-9.52	-9.54	-9.49	-9.31	-8.92	-8.78
2.47	-9.16	-9.20	-9.42	-9.54	-9.46	-9.40	-9.28	-9.29	-8.95	-8.89	-8.75
2.52	-9.27	-9.44	-9.53	-9.47	-9.50	-9.50	-9.36	-9.16	-9.01	-8.81	-8.62
2.57	-9.25	-9.22	-9.46	-9.35	-9.38	-9.28	-9.27	-9.18	-8.86	-8.61	-8.53
2.62	-9.49	-9.57	-9.73	-9.74	-9.75	-9.83	-9.51	-9.31	-9.14	-8.85	-8.70
2.67	-9.23	-9.32	-9.33	-9.46	-9.52	-9.38	-9.15	-9.10	-8.84	-8.59	-8.41
2.72	-9.01	-9.29	-9.21	-9.39	-9.40	-9.25	-9.18	-9.11	-8.82	-8.50	-8.27
2.77	-9.76	-9.70	-9.82	-9.78	-9.80	-9.73	-9.68	-9.44	-9.25	-9.01	-8.89
2.82	-8.88	-9.00	-9.22	-9.10	-9.02	-8.85	-8.86	-8.66	-8.38	-8.21	-7.98
2.87	-9.09	-9.25	-9.31	-9.35	-9.38	-9.22	-9.19	-8.70	-8.59	-8.40	-8.27
2.92	-9.14	-9.21	-9.36	-9.29	-9.26	-9.22	-9.05	-8.83	-8.53	-8.24	-8.01
2.97	-9.59	-9.73	-9.92	-9.86	-9.83	-9.83	-9.58	-9.44	-9.08	-8.94	-8.70
3.02	-9.34	-9.43	-9.45	-9.58	-9.31	-9.41	-9.28	-9.02	-8.79	-8.64	-8.46
3.07	-9.06	-9.10	-9.20	-9.26	-9.10	-9.16	-8.92	-8.68	-8.44	-8.15	-8.05
3.12	-9.25	-9.25	-9.53	-9.31	-9.23	-9.19	-9.03	-8.80	-8.48	-8.23	-7.98
3.17	-9.20	-9.22	-9.37	-9.50	-9.28	-9.12	-9.06	-8.70	-8.52	-8.29	-8.19
3.22	-9.20	-9.18	-9.30	-9.24	-9.08	-9.06	-8.93	-8.76	-8.47	-8.24	-7.96
3.27	-9.00	-9.10	-9.15	-9.19	-9.17	-9.06	-8.94	-8.65	-8.20	-8.03	-7.85
3.32	-8.78	-8.91	-9.06	-8.89	-8.82	-8.83	-8.63	-8.52	-8.10	-7.99	-7.71
3.37	-8.98	-9.14	-9.28	-9.05	-9.13	-9.06	-8.79	-8.72	-8.21	-8.19	-7.90
3.42	-9.15	-9.13	-9.23	-8.98	-8.91	-8.92	-8.77	-8.42	-8.18	-7.89	-7.69
3.47	-8.87	-9.04	-9.11	-9.04	-8.93	-8.90	-8.65	-8.51	-8.04	-7.87	-7.52
3.52	-9.14	-9.44	-9.49	-9.39	-9.43	-9.21	-8.98	-8.75	-8.52	-8.26	-8.11
3.57	-9.08	-9.18	-9.40	-9.33	-9.20	-9.21	-9.09	-8.65	-8.36	-8.10	-7.92
3.62	-9.20	-9.21	-9.29	-9.30	-9.20	-9.00	-8.95	-8.68	-8.33	-8.04	-7.94
3.67	-9.21	-9.23	-9.17	-9.27	-9.13	-9.07	-8.82	-8.65	-8.35	-8.05	-7.87
3.72	-8.91	-8.91	-8.92	-8.96	-8.64	-8.56	-8.60	-8.21	-7.94	-7.70	-7.31
3.92	-8.76	-8.75	-8.94	-8.92	-8.73	-8.57	-8.48	-8.23	-7.82	-7.52	-7.37
4.12	-8.91	-8.97	-9.02	-8.97	-8.84	-8.81	-8.49	-8.39	-8.03	-7.73	-7.42
4.32	-9.10	-9.19	-9.19	-9.19	-9.06	-8.94	-8.57	-8.65	-8.34	-8.03	-7.72
4.52	-8.71	-8.75	-8.82	-8.85	-8.57	-8.64	-8.47	-8.28	-7.95	-7.47	-7.24
4.72	-8.63	-8.69	-8.89	-8.79	-8.75	-8.61	-8.41	-8.00	-7.85	-7.49	-7.16
4.92	-8.97	-8.94	-8.90	-8.87	-8.92	-8.82	-8.58	-8.35	-7.89	-7.58	-7.41

5.12	-9.04	-9.06	-9.26	-9.06	-9.03	-8.91	-8.76	-8.33	-8.20	-7.78	-7.52
5.32	-9.18	-9.22	-9.26	-9.08	-8.96	-8.95	-8.76	-8.46	-8.11	-7.78	-7.48
5.52	-8.68	-8.64	-8.77	-8.73	-8.66	-8.56	-8.41	-7.99	-7.82	-7.44	-7.14
5.72	-9.23	-9.26	-9.34	-9.33	-9.01	-9.01	-8.75	-8.38	-8.11	-7.72	-7.62
5.92	-8.60	-8.69	-8.77	-8.63	-8.46	-8.18	-8.01	-7.89	-7.52	-7.16	-6.92
6.12	-8.97	-9.02	-8.97	-8.84	-8.82	-8.61	-8.33	-8.15	-7.82	-7.38	-7.19
6.32	-8.91	-8.92	-9.14	-8.83	-8.67	-8.58	-8.48	-8.01	-7.80	-7.55	-7.22
6.52	-8.73	-8.64	-8.85	-8.69	-8.58	-8.48	-8.15	-7.91	-7.59	-7.23	-7.08
6.72	-8.77	-8.98	-8.97	-8.95	-8.69	-8.71	-8.37	-8.01	-7.82	-7.45	-7.21
6.92	-8.65	-8.72	-8.64	-8.60	-8.46	-8.28	-7.97	-7.82	-7.47	-7.21	-6.70
7.12	-8.29	-8.24	-8.28	-8.12	-7.93	-7.80	-7.51	-7.35	-6.98	-6.63	-6.39
7.32	-8.60	-8.53	-8.58	-8.57	-8.44	-8.29	-8.03	-7.84	-7.57	-7.10	-6.87
7.52	-8.65	-8.49	-8.59	-8.50	-8.36	-8.26	-8.06	-7.68	-7.43	-7.21	-6.84
7.72	-8.89	-8.92	-8.89	-8.83	-8.70	-8.58	-8.54	-8.08	-7.69	-7.38	-7.15
7.92	-8.66	-8.63	-8.75	-8.71	-8.32	-8.29	-8.06	-7.77	-7.38	-7.14	-6.79
8.12	-8.58	-8.58	-8.54	-8.41	-8.33	-8.16	-7.88	-7.66	-7.32	-6.94	-6.66
8.32	-8.52	-8.52	-8.53	-8.44	-8.30	-8.17	-7.85	-7.58	-7.19	-6.90	-6.70
8.52	-8.69	-8.68	-8.79	-8.66	-8.43	-8.45	-8.10	-7.74	-7.37	-7.14	-6.87
8.72	-8.64	-8.71	-8.74	-8.62	-8.55	-8.24	-8.10	-7.82	-7.37	-7.16	-6.87
8.92	-8.87	-8.92	-9.02	-8.84	-8.72	-8.63	-8.37	-8.11	-7.76	-7.27	-7.19
9.12	-8.69	-8.79	-8.87	-8.72	-8.54	-8.26	-8.08	-7.95	-7.48	-7.13	-6.89
9.32	-9.10	-9.09	-9.06	-8.96	-8.73	-8.74	-8.49	-8.05	-7.69	-7.42	-7.30
9.52	-8.74	-8.74	-8.75	-8.57	-8.55	-8.38	-8.17	-7.86	-7.48	-7.11	-6.80
9.72	-8.85	-8.85	-8.94	-8.75	-8.58	-8.46	-8.25	-7.91	-7.62	-7.25	-6.91
9.92	-8.94	-8.87	-8.97	-8.91	-8.62	-8.61	-8.35	-8.16	-7.82	-7.48	-7.14
10.12	-8.37	-8.57	-8.51	-8.51	-8.23	-8.06	-7.83	-7.55	-7.17	-6.83	-6.63
10.32	-8.49	-8.39	-8.45	-8.34	-8.13	-7.98	-7.79	-7.52	-7.13	-6.85	-6.64
10.52	-8.63	-8.47	-8.71	-8.67	-8.37	-8.14	-7.87	-7.56	-7.26	-6.97	-6.75
10.72	-8.47	-8.52	-8.50	-8.47	-8.29	-8.13	-8.06	-7.82	-7.32	-7.00	-6.65
10.92	-8.49	-8.54	-8.66	-8.55	-8.43	-8.18	-7.91	-7.82	-7.32	-7.15	-6.68
11.12	-8.77	-8.74	-8.81	-8.72	-8.57	-8.47	-8.20	-7.88	-7.56	-7.31	-6.95
11.32	-8.49	-8.53	-8.49	-8.46	-8.23	-8.05	-7.81	-7.51	-7.21	-6.97	-6.66
11.52	-8.23	-8.07	-8.12	-8.04	-7.89	-7.72	-7.43	-7.15	-6.82	-6.51	-6.21
11.72	-8.29	-8.57	-8.53	-8.28	-8.17	-8.02	-7.66	-7.46	-7.15	-6.91	-6.51
11.92	-8.03	-8.05	-8.22	-8.04	-7.81	-7.66	-7.36	-7.10	-6.82	-6.43	-6.03
12.12	-7.86	-7.75	-7.82	-7.73	-7.65	-7.49	-7.26	-7.00	-6.73	-6.29	-6.06
12.32	-8.60	-8.64	-8.57	-8.53	-8.32	-8.16	-7.88	-7.47	-7.29	-6.88	-6.59
12.52	-8.29	-8.45	-8.25	-8.15	-7.96	-7.82	-7.52	-7.25	-6.89	-6.64	-6.14
12.72	-8.78	-8.67	-8.77	-8.69	-8.56	-8.37	-8.31	-7.89	-7.56	-7.07	-6.90
12.92	-8.51	-8.60	-8.59	-8.52	-8.07	-8.15	-7.85	-7.67	-7.23	-6.93	-6.56
13.12	-8.39	-8.28	-8.30	-8.32	-8.00	-7.93	-7.78	-7.29	-7.10	-6.77	-6.43
13.32	-8.90	-8.96	-9.09	-8.89	-8.75	-8.62	-8.23	-8.09	-7.56	-7.27	-7.01
13.52	-8.55	-8.53	-8.58	-8.41	-8.22	-8.11	-7.74	-7.63	-7.17	-6.81	-6.49
13.72	-8.28	-8.22	-8.37	-8.27	-8.06	-7.87	-7.55	-7.32	-7.00	-6.75	-6.34
13.92	-8.62	-8.80	-8.86	-8.56	-8.43	-8.28	-7.96	-7.79	-7.33	-7.14	-6.76
14	-8.38	-8.35	-8.51	-8.38	-8.14	-8.10	-7.81	-7.37	-7.12	-6.80	-6.42
14.08	-8.46	-8.55	-8.66	-8.44	-8.49	-8.08	-7.93	-7.67	-7.20	-6.96	-6.54
14.17	-8.25	-8.19	-8.19	-8.09	-7.96	-7.78	-7.48	-7.25	-6.91	-6.53	-6.33
14.27	-8.51	-8.58	-8.42	-8.25	-8.34	-7.93	-7.73	-7.61	-7.08	-6.77	-6.42
14.38	-8.54	-8.57	-8.48	-8.39	-8.25	-8.08	-7.85	-7.49	-7.17	-6.88	-6.47

14.49	-8.38	-8.23	-8.44	-8.16	-8.14	-7.99	-7.78	-7.42	-7.13	-6.83	-6.48
14.61	-8.60	-8.66	-8.56	-8.59	-8.62	-8.26	-7.91	-7.78	-7.41	-7.01	-6.75
14.75	-8.54	-8.47	-8.59	-8.43	-8.18	-8.10	-7.79	-7.54	-7.25	-7.01	-6.65
14.89	-8.51	-8.47	-8.43	-8.43	-8.34	-8.21	-7.83	-7.64	-7.21	-6.78	-6.37
15.05	-8.45	-8.39	-8.41	-8.26	-8.16	-7.99	-7.80	-7.55	-7.07	-6.73	-6.43
15.21	-8.52	-8.54	-8.48	-8.34	-8.33	-8.11	-7.97	-7.41	-7.21	-6.88	-6.51
15.39	-8.52	-8.39	-8.46	-8.51	-8.24	-8.10	-7.84	-7.61	-7.23	-6.87	-6.52
15.59	-8.62	-8.66	-8.56	-8.57	-8.26	-8.25	-7.93	-7.57	-7.35	-7.01	-6.70
15.8	-8.38	-8.40	-8.25	-8.19	-8.03	-7.78	-7.62	-7.42	-6.91	-6.65	-6.42
16.02	-8.33	-8.18	-8.19	-8.10	-7.92	-7.70	-7.38	-7.24	-6.90	-6.50	-6.26
16.27	-8.45	-8.38	-8.42	-8.41	-8.01	-7.82	-7.62	-7.46	-6.91	-6.55	-6.40
16.53	-8.31	-8.28	-8.24	-8.16	-7.92	-7.72	-7.56	-7.31	-6.81	-6.53	-6.25
16.81	-8.62	-8.52	-8.59	-8.56	-8.35	-8.17	-7.98	-7.60	-7.22	-7.03	-6.49
17.12	-8.32	-8.48	-8.35	-8.34	-8.11	-7.98	-7.75	-7.34	-7.03	-6.69	-6.38
17.45	-8.39	-8.49	-8.55	-8.43	-8.17	-7.90	-7.76	-7.47	-7.05	-6.82	-6.58
17.81	-8.39	-8.30	-8.24	-8.12	-7.93	-7.92	-7.59	-7.21	-6.95	-6.49	-6.22
18.19	-8.29	-8.37	-8.43	-8.16	-8.11	-7.88	-7.64	-7.45	-6.94	-6.69	-6.32
18.6	-8.33	-8.31	-8.37	-8.13	-7.98	-7.88	-7.57	-7.39	-6.94	-6.65	-6.26
19.05	-8.81	-8.78	-8.78	-8.80	-8.51	-8.37	-8.11	-7.83	-7.38	-7.07	-6.78
19.53	-8.16	-8.36	-8.31	-8.16	-7.93	-7.84	-7.43	-7.25	-7.04	-6.58	-6.23
20.05	-8.39	-8.34	-8.34	-8.10	-7.93	-7.83	-7.67	-7.35	-6.90	-6.62	-6.36
20.61	-8.35	-8.24	-8.26	-8.26	-8.09	-7.81	-7.60	-7.42	-6.99	-6.67	-6.46
21.22	-8.58	-8.52	-8.69	-8.38	-8.33	-8.12	-7.79	-7.41	-7.34	-6.95	-6.49
21.87	-8.42	-8.32	-8.27	-8.11	-8.13	-7.84	-7.63	-7.21	-6.89	-6.57	-6.21
22.57	-8.46	-8.45	-8.46	-8.36	-8.18	-8.03	-7.81	-7.52	-7.23	-6.89	-6.55
23.33	-8.00	-8.18	-8.10	-7.95	-7.87	-7.84	-7.42	-7.13	-6.62	-6.47	-6.13
24.15	-8.14	-8.08	-8.13	-8.09	-7.84	-7.69	-7.35	-7.03	-6.69	-6.36	-6.07
25.03	-8.31	-8.18	-8.30	-8.05	-7.91	-7.88	-7.54	-7.35	-6.80	-6.58	-6.16
25.98	-8.59	-8.64	-8.68	-8.63	-8.41	-8.25	-7.99	-7.65	-7.43	-7.07	-6.67
27.01	-8.65	-8.73	-8.72	-8.56	-8.38	-8.23	-7.94	-7.72	-7.39	-7.07	-6.68
28.11	-8.20	-8.27	-8.20	-8.01	-8.00	-7.72	-7.52	-7.23	-6.76	-6.56	-6.21
29.3	-8.74	-8.80	-8.74	-8.61	-8.38	-8.22	-8.05	-7.76	-7.29	-6.92	-6.53
30.59	-8.04	-7.81	-7.99	-7.90	-7.56	-7.50	-7.28	-7.05	-6.61	-6.33	-5.96
31.98	-8.34	-8.35	-8.22	-8.05	-7.91	-7.84	-7.62	-7.29	-6.95	-6.69	-6.29
33.47	-8.10	-8.06	-8.13	-8.03	-7.91	-7.68	-7.47	-7.26	-6.74	-6.44	-6.00
35.09	-8.26	-8.24	-8.11	-8.19	-7.86	-7.75	-7.43	-7.18	-6.84	-6.40	-6.16
36.83	-8.21	-8.31	-8.35	-8.18	-7.88	-7.76	-7.69	-7.24	-6.93	-6.52	-6.19
38.71	-8.26	-8.32	-8.16	-8.00	-8.01	-7.74	-7.45	-7.22	-6.93	-6.59	-6.11
40.73	-8.36	-8.39	-8.34	-8.14	-8.06	-7.87	-7.67	-7.28	-6.95	-6.72	-6.31
42.92	-8.44	-8.39	-8.48	-8.40	-8.10	-8.01	-7.74	-7.31	-6.96	-6.54	-6.34
45.27	-8.49	-8.45	-8.45	-8.27	-8.27	-7.95	-7.67	-7.47	-7.09	-6.83	-6.55
47.81	-8.73	-8.65	-8.69	-8.48	-8.48	-8.24	-8.07	-7.77	-7.51	-7.03	-6.78
50.55	-8.49	-8.37	-8.46	-8.28	-7.92	-8.00	-7.70	-7.31	-6.96	-6.67	-6.23
53.51	-8.53	-8.38	-8.45	-8.20	-8.18	-8.04	-7.79	-7.45	-6.96	-6.79	-6.43
56.69	-8.51	-8.65	-8.64	-8.47	-8.30	-8.02	-7.87	-7.55	-7.05	-6.73	-6.42
60.13	-8.24	-8.31	-8.29	-8.34	-8.08	-7.98	-7.77	-7.39	-6.97	-6.55	-6.19
63.84	-8.46	-8.46	-8.50	-8.47	-8.10	-8.09	-7.70	-7.50	-7.25	-6.83	-6.39
67.83	-8.81	-8.69	-8.82	-8.64	-8.50	-8.27	-8.10	-7.80	-7.34	-7.04	-6.83
72.15	-8.29	-8.43	-8.39	-8.29	-8.14	-7.94	-7.78	-7.46	-7.21	-6.68	-6.43
76.8	-8.28	-8.24	-8.37	-8.13	-8.02	-7.88	-7.64	-7.34	-6.96	-6.60	-6.34

81.81	-8.17	-8.29	-8.34	-8.20	-7.91	-7.83	-7.57	-7.29	-6.92	-6.55	-6.29
87.22	-8.40	-8.33	-8.38	-8.31	-8.17	-7.79	-7.60	-7.43	-6.99	-6.63	-6.19
93.06	-8.38	-8.28	-8.39	-8.26	-8.18	-7.98	-7.78	-7.38	-7.13	-6.76	-6.49
99.35	-8.29	-8.48	-8.40	-8.28	-8.14	-7.96	-7.74	-7.42	-7.02	-6.72	-6.32
106.13	-8.22	-8.35	-8.15	-8.24	-7.93	-7.81	-7.46	-7.26	-6.88	-6.60	-6.21
113.45	-8.27	-8.20	-8.20	-8.19	-7.98	-7.87	-7.55	-7.21	-6.86	-6.59	-6.22
121.34	-8.30	-8.28	-8.32	-8.21	-8.01	-7.77	-7.66	-7.48	-7.07	-6.82	-6.42
129.86	-8.49	-8.58	-8.59	-8.33	-8.24	-8.24	-7.93	-7.67	-7.24	-7.06	-6.68
139.04	-8.06	-7.99	-8.12	-8.07	-7.89	-7.68	-7.53	-7.23	-6.80	-6.43	-6.16
148.94	-8.44	-8.29	-8.41	-8.30	-7.99	-7.87	-7.74	-7.33	-6.98	-6.61	-6.14
159.62	-8.16	-8.06	-8.03	-8.10	-7.86	-7.66	-7.40	-7.17	-6.72	-6.47	-6.18
171.14	-7.80	-7.78	-7.83	-7.70	-7.64	-7.39	-7.31	-7.00	-6.56	-6.25	-6.04
183.56	-8.31	-8.27	-8.31	-8.20	-8.03	-7.90	-7.64	-7.37	-6.93	-6.74	-6.39
196.96	-8.26	-8.25	-8.19	-8.12	-7.84	-7.83	-7.60	-7.40	-6.98	-6.68	-6.30
211.41	-8.06	-8.15	-8.04	-8.02	-7.97	-7.77	-7.54	-7.27	-6.93	-6.57	-6.32
226.99	-7.90	-7.98	-8.12	-7.93	-7.74	-7.62	-7.55	-7.15	-6.91	-6.45	-6.11
243.8	-8.12	-7.96	-8.09	-7.91	-7.64	-7.65	-7.44	-7.09	-6.83	-6.56	-6.12
261.93	-8.01	-7.95	-7.98	-7.90	-7.71	-7.60	-7.38	-7.25	-6.82	-6.47	-6.25
281.48	-7.83	-7.81	-7.94	-7.79	-7.62	-7.57	-7.21	-7.10	-6.67	-6.39	-5.92
302.56	-7.74	-7.86	-7.69	-7.81	-7.62	-7.42	-7.31	-7.04	-6.74	-6.30	-6.07
325.3	-7.88	-7.93	-7.85	-7.89	-7.68	-7.51	-7.28	-6.98	-6.68	-6.30	-5.99
349.83	-7.57	-7.56	-7.57	-7.54	-7.39	-7.24	-6.97	-6.82	-6.40	-6.07	-5.91
376.28	-7.82	-7.65	-7.73	-7.60	-7.45	-7.44	-7.26	-6.92	-6.62	-6.30	-5.94
404.81	-7.23	-7.31	-7.32	-7.34	-7.26	-7.02	-6.81	-6.51	-6.20	-5.88	-5.65
435.58	-7.57	-7.50	-7.66	-7.63	-7.55	-7.26	-7.12	-6.81	-6.63	-6.28	-5.94
468.76	-7.24	-7.27	-7.35	-7.33	-7.12	-7.13	-6.88	-6.63	-6.27	-5.95	-5.65
504.55	-7.17	-7.05	-7.25	-7.09	-6.85	-7.03	-6.77	-6.45	-6.09	-5.70	-5.58
543.15	-7.17	-7.29	-7.24	-7.14	-7.11	-7.01	-6.71	-6.60	-6.25	-5.77	-5.50
584.78	-7.12	-7.21	-7.09	-7.04	-6.93	-6.87	-6.65	-6.30	-5.97	-5.77	-5.33
629.68	-6.95	-7.07	-7.13	-6.90	-6.81	-6.81	-6.56	-6.36	-6.05	-5.57	-5.42
678.1	-6.69	-6.62	-6.71	-6.66	-6.54	-6.44	-6.14	-5.89	-5.62	-5.25	-4.96
730.33	-6.85	-6.92	-7.04	-6.81	-6.71	-6.62	-6.44	-6.16	-5.81	-5.59	-5.31
786.65	-6.77	-6.86	-6.86	-6.74	-6.73	-6.56	-6.32	-6.12	-5.78	-5.47	-5.29
847.4	-7.02	-6.96	-6.95	-7.00	-6.89	-6.75	-6.48	-6.34	-5.88	-5.67	-5.38
912.92	-6.75	-6.70	-6.94	-6.72	-6.73	-6.38	-6.32	-6.14	-5.79	-5.50	-5.12
983.58	-6.69	-6.52	-6.72	-6.62	-6.60	-6.47	-6.30	-6.11	-5.75	-5.39	-5.14
1059.78	-6.68	-6.57	-6.62	-6.54	-6.56	-6.43	-6.16	-5.89	-5.61	-5.30	-5.08
1141.98	-6.12	-6.12	-6.21	-5.96	-5.98	-6.06	-5.73	-5.52	-5.19	-5.06	-4.69
1230.62	-6.35	-6.40	-6.63	-6.52	-6.32	-6.27	-6.00	-5.75	-5.42	-5.21	-4.90
1326.23	-6.52	-6.51	-6.59	-6.55	-6.56	-6.29	-6.09	-5.96	-5.57	-5.20	-4.96
1429.34	-6.52	-6.37	-6.41	-6.33	-6.26	-6.09	-5.95	-5.68	-5.35	-5.11	-4.75
1540.54	-6.76	-6.85	-6.89	-6.77	-6.78	-6.52	-6.44	-6.22	-5.92	-5.48	-5.25
1660.48	-6.50	-6.54	-6.60	-6.51	-6.41	-6.27	-6.16	-5.77	-5.49	-5.22	-4.92
1789.83	-6.77	-6.70	-6.90	-6.91	-6.65	-6.48	-6.28	-6.15	-5.83	-5.44	-5.20
1929.34	-6.65	-6.77	-6.89	-6.72	-6.58	-6.52	-6.24	-6.00	-5.76	-5.38	-4.99
2079.8	-6.82	-6.89	-6.90	-6.97	-6.68	-6.71	-6.55	-6.20	-5.87	-5.56	-5.50
2242.08	-6.67	-6.72	-6.80	-6.72	-6.71	-6.49	-6.33	-6.02	-5.84	-5.38	-5.07
2417.1	-6.76	-6.73	-6.71	-6.67	-6.62	-6.39	-6.39	-6.07	-5.71	-5.34	-5.13
2605.85	-6.89	-7.00	-7.01	-6.91	-6.76	-6.54	-6.46	-6.08	-5.78	-5.72	-5.14
2809.43	-6.64	-6.76	-6.87	-6.72	-6.56	-6.45	-6.41	-5.98	-5.76	-5.42	-5.21

3028.99	-6.48	-6.41	-6.62	-6.45	-6.35	-6.41	-6.15	-5.99	-5.58	-5.34	-4.99
3265.79	-6.77	-6.75	-6.73	-6.52	-6.58	-6.40	-6.30	-6.07	-5.76	-5.34	-5.15
3521.18	-6.80	-7.06	-7.20	-6.96	-6.80	-6.75	-6.65	-6.42	-6.19	-5.67	-5.39
3796.62	-6.67	-6.90	-6.83	-6.70	-6.78	-6.52	-6.35	-6.12	-5.79	-5.36	-5.17

Wavelength (nm) / Time (ps)	506.63	508.04	509.45	510.86	512.27	513.67	515.08	516.49	517.90	519.31	520.72
-3.28	0.16	0.32	0.33	0.18	0.22	0.23	0.25	0.13	0.19	0.22	0.22
-2.78	0.11	-0.06	0.14	0.09	0.10	0.06	0.02	0.06	0.02	0.02	-0.01
-2.28	0.12	0.14	0.10	0.16	0.20	0.15	0.17	0.20	0.21	0.08	0.21
-1.78	0.02	-0.06	-0.10	-0.02	-0.01	-0.02	0.06	0.03	0.07	0.10	0.02
-1.28	-0.35	-0.29	-0.21	-0.20	-0.32	-0.33	-0.35	-0.30	-0.35	-0.24	-0.34
-0.78	0.05	0.03	0.03	-0.06	0.05	0.10	0.07	0.05	0.01	0.08	0.03
-0.28	-0.31	-0.32	-0.54	-0.32	-0.51	-0.39	-0.47	-0.48	-0.41	-0.47	-0.47
0.22	0.20	0.24	0.26	0.18	0.27	0.22	0.25	0.31	0.26	0.22	0.35
0.32	0.13	0.12	0.15	0.08	0.17	0.07	0.10	0.22	0.12	0.24	0.20
0.42	-0.22	-0.08	-0.18	-0.11	-0.16	-0.12	-0.22	-0.08	-0.16	-0.03	0.01
0.52	-0.40	-0.23	-0.33	-0.33	-0.41	-0.30	-0.31	-0.18	-0.42	-0.33	-0.24
0.62	0.23	0.21	0.26	0.19	0.32	0.17	0.19	0.22	0.20	0.13	0.24
0.72	-0.34	-0.47	-0.37	-0.45	-0.38	-0.41	-0.38	-0.35	-0.39	-0.31	-0.23
0.77	0.15	0.16	0.16	0.18	0.25	0.16	0.22	0.08	0.19	0.14	0.19
0.82	0.31	0.30	0.22	0.06	0.35	0.30	0.21	0.24	0.21	0.26	0.22
0.87	-0.04	0.01	-0.11	-0.02	0.11	0.02	0.00	-0.08	0.04	-0.07	0.04
0.92	-0.14	0.00	-0.10	-0.15	-0.18	-0.11	-0.18	-0.09	-0.05	-0.12	-0.10
0.97	-0.06	-0.02	-0.12	-0.16	0.02	-0.07	-0.04	-0.02	0.02	-0.11	-0.11
1.02	0.02	0.00	0.06	0.02	0.12	0.08	0.13	0.05	0.04	0.05	0.11
1.07	-0.04	-0.04	0.09	0.06	0.07	-0.08	0.02	0.05	0.00	-0.12	-0.05
1.12	-0.18	-0.20	-0.22	-0.24	-0.26	-0.30	-0.29	-0.24	-0.18	-0.21	-0.13
1.17	-0.25	-0.22	-0.24	-0.25	-0.26	-0.23	-0.28	-0.18	-0.22	-0.17	-0.14
1.22	0.12	0.07	0.13	0.14	0.27	0.14	0.10	0.20	0.31	0.17	0.22
1.27	-0.06	-0.09	-0.01	-0.09	-0.04	-0.07	-0.08	-0.02	-0.09	-0.10	-0.02
1.32	-0.39	-0.27	-0.31	-0.35	-0.28	-0.22	-0.25	-0.25	-0.07	-0.19	-0.05
1.37	-0.42	-0.33	-0.42	-0.41	-0.26	-0.40	-0.31	-0.33	-0.34	-0.34	-0.23
1.42	0.33	0.36	0.30	0.22	0.19	0.04	-0.07	0.03	-0.13	-0.23	-0.34
1.47	-6.41	-5.97	-5.55	-5.10	-4.63	-4.26	-3.95	-3.66	-3.46	-3.06	-2.82
1.52	-12.15	-11.82	-11.47	-11.13	-10.97	-10.70	-10.35	-10.19	-9.87	-9.62	-9.24
1.57	-14.94	-14.85	-14.75	-14.69	-14.57	-14.55	-14.56	-14.45	-14.43	-14.24	-14.16
1.62	-15.54	-15.45	-15.52	-15.53	-15.50	-15.60	-15.80	-15.77	-15.97	-15.93	-15.96
1.67	-14.78	-14.80	-14.91	-14.90	-14.90	-15.02	-15.26	-15.39	-15.57	-15.67	-15.82
1.72	-13.15	-13.12	-13.09	-13.11	-13.08	-13.22	-13.41	-13.43	-13.63	-13.88	-13.94
1.77	-12.19	-12.25	-12.30	-12.16	-12.25	-12.28	-12.41	-12.49	-12.65	-12.76	-12.90
1.82	-11.71	-11.80	-11.82	-11.75	-11.69	-11.79	-11.74	-12.03	-12.12	-12.25	-12.37
1.87	-11.29	-11.25	-11.24	-11.14	-11.13	-11.10	-11.09	-11.16	-11.23	-11.45	-11.51
1.92	-10.88	-10.72	-10.67	-10.57	-10.35	-10.46	-10.41	-10.61	-10.72	-10.77	-10.87
1.97	-10.73	-10.50	-10.47	-10.42	-10.34	-10.39	-10.32	-10.36	-10.46	-10.47	-10.52
2.02	-9.98	-9.88	-9.88	-9.68	-9.52	-9.52	-9.51	-9.61	-9.57	-9.56	-9.67
2.07	-9.96	-9.89	-9.75	-9.72	-9.51	-9.45	-9.41	-9.47	-9.41	-9.46	-9.55
2.12	-9.78	-9.50	-9.42	-9.35	-9.21	-9.11	-9.17	-9.17	-9.14	-9.29	-9.22

2.17	-9.36	-9.10	-9.00	-8.77	-8.80	-8.82	-8.70	-8.70	-8.76	-8.77	-8.67
2.22	-9.00	-8.83	-8.69	-8.62	-8.43	-8.45	-8.33	-8.28	-8.27	-8.28	-8.35
2.27	-8.69	-8.49	-8.23	-8.21	-8.02	-7.99	-7.86	-7.84	-7.95	-7.93	-7.84
2.32	-8.80	-8.66	-8.52	-8.34	-8.13	-8.26	-8.12	-7.97	-8.08	-8.17	-8.00
2.37	-8.71	-8.52	-8.28	-8.22	-8.02	-8.01	-7.86	-8.00	-7.78	-7.83	-7.78
2.42	-8.72	-8.32	-8.18	-8.08	-7.78	-7.76	-7.84	-7.70	-7.55	-7.46	-7.48
2.47	-8.54	-8.29	-8.07	-7.95	-7.66	-7.65	-7.62	-7.52	-7.54	-7.41	-7.45
2.52	-8.29	-8.13	-7.99	-7.82	-7.70	-7.46	-7.45	-7.48	-7.32	-7.43	-7.40
2.57	-8.29	-7.90	-7.74	-7.68	-7.44	-7.43	-7.27	-7.36	-7.17	-7.21	-7.16
2.62	-8.50	-8.34	-8.14	-7.77	-7.65	-7.67	-7.32	-7.49	-7.38	-7.39	-7.30
2.67	-8.30	-7.99	-7.78	-7.47	-7.35	-7.21	-7.17	-7.10	-7.05	-6.95	-6.99
2.72	-8.12	-7.76	-7.63	-7.55	-7.31	-7.00	-7.04	-6.99	-6.90	-6.87	-6.75
2.77	-8.61	-8.44	-8.12	-7.86	-7.70	-7.64	-7.49	-7.37	-7.39	-7.30	-7.24
2.82	-7.79	-7.44	-7.34	-7.12	-6.95	-6.68	-6.63	-6.74	-6.48	-6.46	-6.36
2.87	-7.88	-7.68	-7.48	-7.26	-7.21	-7.00	-6.85	-6.80	-6.86	-6.73	-6.69
2.92	-7.74	-7.36	-7.40	-7.14	-6.83	-6.65	-6.56	-6.57	-6.46	-6.61	-6.29
2.97	-8.46	-8.16	-7.98	-7.75	-7.41	-7.33	-7.08	-7.08	-7.00	-6.96	-6.91
3.02	-8.14	-7.76	-7.61	-7.39	-7.17	-7.01	-6.89	-6.82	-6.67	-6.59	-6.45
3.07	-7.69	-7.36	-7.30	-7.08	-6.81	-6.70	-6.39	-6.46	-6.31	-6.24	-6.24
3.12	-7.78	-7.53	-7.26	-6.96	-6.72	-6.57	-6.56	-6.37	-6.37	-6.40	-6.21
3.17	-7.81	-7.44	-7.36	-7.02	-6.74	-6.67	-6.56	-6.52	-6.25	-6.31	-6.28
3.22	-7.72	-7.54	-7.37	-6.92	-6.74	-6.63	-6.53	-6.43	-6.29	-6.33	-6.24
3.27	-7.54	-7.22	-6.99	-6.81	-6.45	-6.36	-6.38	-6.28	-6.14	-6.07	-5.93
3.32	-7.36	-7.11	-6.87	-6.66	-6.36	-6.25	-6.05	-5.98	-5.91	-5.85	-5.84
3.37	-7.58	-7.19	-7.03	-6.76	-6.59	-6.37	-6.36	-6.23	-6.03	-6.06	-5.93
3.42	-7.48	-7.14	-6.93	-6.75	-6.60	-6.36	-6.22	-6.09	-5.98	-6.01	-5.90
3.47	-7.23	-7.06	-6.84	-6.52	-6.24	-6.15	-6.03	-5.95	-5.82	-5.89	-5.65
3.52	-7.83	-7.42	-7.17	-6.95	-6.70	-6.55	-6.36	-6.41	-6.30	-6.29	-6.09
3.57	-7.36	-7.34	-7.12	-6.73	-6.52	-6.26	-6.18	-6.09	-6.00	-5.88	-5.74
3.62	-7.51	-7.25	-6.99	-6.80	-6.53	-6.29	-6.11	-6.06	-5.97	-5.92	-5.92
3.67	-7.56	-7.28	-6.83	-6.74	-6.59	-6.42	-6.17	-6.10	-5.91	-5.90	-5.83
3.72	-7.17	-6.81	-6.49	-6.39	-6.09	-5.88	-5.75	-5.67	-5.54	-5.46	-5.39
3.92	-7.19	-6.74	-6.50	-6.28	-6.07	-5.88	-5.79	-5.66	-5.61	-5.48	-5.38
4.12	-7.28	-6.84	-6.62	-6.28	-6.07	-5.83	-5.74	-5.62	-5.47	-5.50	-5.40
4.32	-7.34	-6.99	-6.76	-6.63	-6.21	-6.03	-5.90	-5.73	-5.69	-5.54	-5.46
4.52	-6.93	-6.43	-6.32	-6.03	-5.76	-5.61	-5.54	-5.30	-5.21	-5.16	-5.19
4.72	-6.77	-6.46	-6.38	-5.96	-5.83	-5.67	-5.42	-5.44	-5.25	-5.23	-5.14
4.92	-7.07	-6.72	-6.40	-6.13	-5.91	-5.69	-5.53	-5.41	-5.36	-5.36	-5.24
5.12	-7.12	-6.92	-6.54	-6.30	-6.06	-5.91	-5.78	-5.58	-5.46	-5.55	-5.40
5.32	-7.16	-6.77	-6.46	-6.19	-5.98	-5.81	-5.51	-5.38	-5.30	-5.40	-5.14
5.52	-6.84	-6.45	-6.29	-6.03	-5.78	-5.49	-5.41	-5.24	-5.11	-5.09	-4.99
5.72	-7.29	-6.92	-6.73	-6.32	-6.08	-5.95	-5.86	-5.63	-5.56	-5.55	-5.39
5.92	-6.59	-6.27	-6.12	-5.83	-5.61	-5.31	-5.21	-5.15	-5.01	-4.97	-4.86
6.12	-6.98	-6.64	-6.37	-5.96	-5.60	-5.47	-5.41	-5.32	-5.12	-5.09	-4.97
6.32	-6.96	-6.68	-6.24	-5.86	-5.74	-5.64	-5.42	-5.24	-5.17	-5.04	-5.04
6.52	-6.72	-6.27	-6.05	-5.81	-5.45	-5.23	-5.10	-5.04	-4.94	-4.89	-4.79
6.72	-6.91	-6.54	-6.16	-6.07	-5.77	-5.47	-5.35	-5.36	-5.10	-5.14	-5.07
6.92	-6.61	-6.21	-5.92	-5.78	-5.43	-5.22	-5.10	-5.00	-4.89	-4.89	-4.67
7.12	-6.05	-5.79	-5.40	-5.20	-4.88	-4.75	-4.49	-4.49	-4.35	-4.34	-4.29
7.32	-6.54	-6.18	-5.87	-5.51	-5.44	-5.22	-5.11	-4.90	-4.79	-4.77	-4.74

7.52	-6.59	-6.22	-5.78	-5.54	-5.25	-5.16	-4.86	-4.83	-4.59	-4.62	-4.50
7.72	-6.95	-6.52	-6.26	-5.88	-5.62	-5.41	-5.34	-5.24	-5.15	-5.11	-4.94
7.92	-6.48	-6.08	-5.82	-5.53	-5.27	-5.23	-5.01	-4.84	-4.83	-4.72	-4.62
8.12	-6.31	-5.84	-5.77	-5.35	-5.19	-5.10	-4.81	-4.76	-4.62	-4.63	-4.50
8.32	-6.37	-5.98	-5.66	-5.32	-5.15	-4.95	-4.81	-4.79	-4.68	-4.55	-4.55
8.52	-6.50	-6.13	-5.93	-5.64	-5.40	-5.06	-4.98	-4.76	-4.74	-4.80	-4.61
8.72	-6.44	-6.15	-5.88	-5.53	-5.25	-5.12	-4.85	-4.70	-4.74	-4.61	-4.61
8.92	-6.73	-6.47	-6.11	-5.85	-5.55	-5.35	-5.10	-5.10	-4.96	-4.96	-4.86
9.12	-6.54	-6.17	-5.96	-5.60	-5.38	-5.20	-5.08	-4.86	-4.75	-4.78	-4.79
9.32	-6.86	-6.47	-6.08	-5.91	-5.64	-5.61	-5.42	-5.32	-5.08	-5.12	-4.94
9.52	-6.50	-6.17	-5.80	-5.43	-5.33	-5.13	-4.93	-4.82	-4.69	-4.69	-4.62
9.72	-6.65	-6.22	-5.88	-5.60	-5.37	-5.17	-4.98	-4.90	-4.74	-4.73	-4.51
9.92	-6.73	-6.38	-6.14	-5.75	-5.46	-5.29	-5.18	-5.04	-4.96	-5.00	-4.80
10.12	-6.21	-5.76	-5.56	-5.28	-4.97	-4.94	-4.77	-4.58	-4.52	-4.51	-4.35
10.32	-6.31	-6.02	-5.69	-5.32	-5.07	-4.98	-4.69	-4.73	-4.56	-4.50	-4.58
10.52	-6.33	-6.01	-5.70	-5.46	-5.12	-4.95	-4.81	-4.87	-4.73	-4.58	-4.60
10.72	-6.31	-5.96	-5.66	-5.43	-5.18	-4.87	-4.78	-4.72	-4.61	-4.53	-4.56
10.92	-6.28	-6.14	-5.84	-5.52	-5.26	-4.96	-4.92	-4.80	-4.70	-4.63	-4.51
11.12	-6.63	-6.22	-5.82	-5.62	-5.39	-5.26	-5.04	-4.92	-4.92	-4.80	-4.73
11.32	-6.30	-5.95	-5.64	-5.34	-5.09	-4.97	-4.82	-4.70	-4.63	-4.58	-4.49
11.52	-5.85	-5.51	-5.21	-4.94	-4.79	-4.61	-4.49	-4.21	-4.23	-4.23	-4.10
11.72	-6.20	-5.94	-5.62	-5.21	-5.05	-4.73	-4.61	-4.51	-4.58	-4.43	-4.41
11.92	-5.87	-5.45	-5.13	-4.88	-4.63	-4.47	-4.30	-4.18	-4.08	-4.03	-3.89
12.12	-5.62	-5.23	-4.80	-4.69	-4.45	-4.21	-4.13	-3.98	-3.89	-3.86	-3.85
12.32	-6.35	-5.87	-5.46	-5.26	-4.93	-4.80	-4.60	-4.45	-4.48	-4.49	-4.29
12.52	-6.04	-5.64	-5.32	-5.09	-4.85	-4.74	-4.55	-4.37	-4.14	-4.28	-4.14
12.72	-6.51	-6.12	-5.99	-5.60	-5.30	-5.19	-4.91	-4.96	-4.90	-4.90	-4.70
12.92	-6.24	-5.88	-5.68	-5.27	-5.12	-4.82	-4.62	-4.60	-4.45	-4.45	-4.37
13.12	-6.09	-5.65	-5.19	-5.09	-4.63	-4.57	-4.42	-4.29	-4.23	-4.24	-4.12
13.32	-6.72	-6.26	-5.98	-5.72	-5.46	-5.23	-5.11	-4.98	-5.03	-4.96	-4.83
13.52	-6.11	-5.88	-5.48	-5.22	-5.02	-4.75	-4.63	-4.68	-4.47	-4.47	-4.41
13.72	-6.02	-5.64	-5.43	-5.14	-4.81	-4.84	-4.51	-4.44	-4.37	-4.32	-4.18
13.92	-6.33	-5.91	-5.76	-5.33	-5.23	-4.94	-4.88	-4.73	-4.54	-4.55	-4.51
14	-6.18	-5.80	-5.49	-5.19	-4.98	-4.76	-4.59	-4.49	-4.49	-4.43	-4.28
14.08	-6.36	-5.89	-5.63	-5.40	-5.05	-4.98	-4.69	-4.63	-4.59	-4.42	-4.42
14.17	-5.89	-5.54	-5.24	-5.03	-4.73	-4.57	-4.46	-4.15	-4.15	-4.19	-3.96
14.27	-6.19	-5.81	-5.58	-5.10	-4.99	-4.72	-4.73	-4.65	-4.55	-4.51	-4.37
14.38	-6.20	-5.74	-5.52	-5.29	-5.01	-4.84	-4.76	-4.50	-4.38	-4.44	-4.35
14.49	-6.12	-5.80	-5.60	-5.21	-4.99	-4.78	-4.71	-4.66	-4.39	-4.38	-4.30
14.61	-6.37	-6.03	-5.73	-5.46	-5.09	-5.00	-4.77	-4.64	-4.66	-4.62	-4.57
14.75	-6.19	-5.97	-5.58	-5.27	-5.00	-4.85	-4.56	-4.61	-4.50	-4.38	-4.37
14.89	-6.16	-5.81	-5.51	-5.25	-5.02	-4.79	-4.76	-4.52	-4.44	-4.35	-4.37
15.05	-6.12	-5.74	-5.46	-5.15	-4.96	-4.70	-4.58	-4.39	-4.41	-4.45	-4.38
15.21	-6.24	-5.79	-5.59	-5.24	-4.96	-4.79	-4.64	-4.41	-4.51	-4.47	-4.24
15.39	-6.08	-5.80	-5.46	-5.30	-4.95	-4.74	-4.61	-4.43	-4.40	-4.32	-4.31
15.59	-6.30	-5.92	-5.64	-5.30	-4.96	-4.92	-4.75	-4.64	-4.59	-4.58	-4.43
15.8	-5.91	-5.67	-5.37	-5.21	-4.81	-4.71	-4.55	-4.53	-4.31	-4.48	-4.32
16.02	-5.87	-5.48	-5.29	-5.01	-4.66	-4.48	-4.39	-4.33	-4.24	-4.15	-4.15
16.27	-6.05	-5.62	-5.34	-5.08	-4.74	-4.73	-4.42	-4.42	-4.35	-4.38	-4.15
16.53	-5.94	-5.52	-5.36	-5.00	-4.75	-4.52	-4.40	-4.37	-4.15	-4.18	-4.04

16.81	-6.20	-5.85	-5.61	-5.11	-5.09	-4.77	-4.61	-4.49	-4.44	-4.34	-4.34
17.12	-6.18	-5.81	-5.38	-5.04	-4.82	-4.63	-4.54	-4.41	-4.39	-4.26	-4.21
17.45	-6.10	-5.75	-5.44	-5.26	-5.01	-4.73	-4.71	-4.52	-4.39	-4.30	-4.23
17.81	-5.91	-5.58	-5.17	-4.96	-4.70	-4.52	-4.39	-4.23	-4.21	-4.27	-4.13
18.19	-6.00	-5.69	-5.46	-5.10	-4.71	-4.69	-4.44	-4.39	-4.29	-4.30	-4.25
18.6	-6.02	-5.42	-5.31	-5.11	-4.78	-4.52	-4.30	-4.33	-4.20	-4.18	-4.11
19.05	-6.43	-6.04	-5.73	-5.47	-5.13	-4.97	-4.76	-4.70	-4.59	-4.62	-4.59
19.53	-5.93	-5.52	-5.25	-4.90	-4.80	-4.42	-4.37	-4.32	-4.20	-4.25	-4.07
20.05	-5.96	-5.66	-5.23	-4.98	-4.78	-4.53	-4.52	-4.37	-4.27	-4.29	-4.18
20.61	-6.05	-5.72	-5.38	-5.06	-4.77	-4.70	-4.54	-4.34	-4.35	-4.33	-4.28
21.22	-6.18	-5.84	-5.50	-5.15	-4.95	-4.78	-4.62	-4.44	-4.46	-4.44	-4.29
21.87	-5.91	-5.55	-5.24	-4.93	-4.53	-4.50	-4.30	-4.21	-4.12	-3.98	-4.07
22.57	-6.21	-5.81	-5.47	-5.20	-4.86	-4.85	-4.51	-4.42	-4.44	-4.35	-4.24
23.33	-5.86	-5.30	-5.04	-4.78	-4.61	-4.42	-4.15	-4.08	-4.04	-4.06	-3.97
24.15	-5.85	-5.37	-5.09	-4.76	-4.56	-4.27	-4.22	-4.08	-3.97	-3.96	-3.93
25.03	-5.78	-5.36	-5.16	-4.99	-4.65	-4.39	-4.26	-4.10	-4.25	-4.05	-3.95
25.98	-6.35	-5.94	-5.75	-5.37	-5.15	-4.91	-4.77	-4.77	-4.67	-4.56	-4.50
27.01	-6.24	-5.97	-5.71	-5.41	-5.05	-4.87	-4.65	-4.61	-4.62	-4.60	-4.54
28.11	-5.84	-5.45	-5.26	-4.90	-4.55	-4.45	-4.30	-4.20	-4.09	-4.13	-4.04
29.3	-6.28	-5.92	-5.63	-5.34	-4.97	-4.82	-4.76	-4.51	-4.65	-4.52	-4.37
30.59	-5.71	-5.29	-5.04	-4.62	-4.37	-4.25	-4.08	-4.13	-4.07	-4.06	-4.12
31.98	-5.89	-5.52	-5.34	-4.97	-4.70	-4.56	-4.47	-4.28	-4.15	-4.15	-4.14
33.47	-5.71	-5.55	-5.21	-4.85	-4.64	-4.41	-4.22	-4.01	-3.90	-3.91	-3.90
35.09	-5.84	-5.47	-5.18	-4.95	-4.55	-4.39	-4.23	-4.16	-4.08	-3.99	-3.94
36.83	-5.85	-5.46	-5.15	-4.92	-4.67	-4.44	-4.37	-4.17	-4.06	-4.09	-3.96
38.71	-5.97	-5.47	-5.20	-4.95	-4.71	-4.51	-4.33	-4.17	-4.07	-4.12	-3.96
40.73	-5.94	-5.54	-5.30	-5.19	-4.71	-4.49	-4.35	-4.31	-4.23	-4.22	-4.13
42.92	-5.94	-5.48	-5.26	-5.01	-4.68	-4.49	-4.36	-4.23	-4.06	-4.11	-3.91
45.27	-6.01	-5.70	-5.38	-5.09	-4.82	-4.59	-4.47	-4.31	-4.25	-4.22	-4.11
47.81	-6.32	-6.02	-5.69	-5.44	-5.16	-4.86	-4.88	-4.74	-4.69	-4.59	-4.56
50.55	-6.05	-5.60	-5.38	-4.99	-4.75	-4.49	-4.33	-4.20	-4.14	-4.17	-4.01
53.51	-6.07	-5.64	-5.44	-5.16	-4.81	-4.66	-4.59	-4.35	-4.40	-4.27	-4.15
56.69	-6.05	-5.75	-5.49	-5.04	-4.80	-4.73	-4.50	-4.35	-4.20	-4.17	-4.00
60.13	-5.99	-5.57	-5.30	-4.91	-4.60	-4.48	-4.32	-4.18	-4.19	-4.03	-3.92
63.84	-6.12	-5.72	-5.47	-5.13	-4.75	-4.82	-4.52	-4.38	-4.21	-4.20	-4.15
67.83	-6.38	-6.04	-5.79	-5.39	-5.23	-5.03	-4.77	-4.67	-4.67	-4.43	-4.38
72.15	-5.94	-5.73	-5.41	-5.18	-4.90	-4.59	-4.47	-4.33	-4.25	-4.17	-4.17
76.8	-6.05	-5.64	-5.25	-4.99	-4.79	-4.51	-4.38	-4.26	-4.21	-4.19	-4.05
81.81	-5.92	-5.46	-5.29	-5.00	-4.67	-4.45	-4.27	-4.11	-3.97	-4.02	-3.88
87.22	-6.01	-5.72	-5.30	-4.99	-4.69	-4.55	-4.21	-4.13	-3.84	-3.97	-3.84
93.06	-6.01	-5.66	-5.31	-4.93	-4.71	-4.46	-4.29	-4.25	-4.13	-4.04	-3.90
99.35	-5.88	-5.69	-5.35	-5.02	-4.75	-4.51	-4.26	-4.27	-4.05	-3.91	-3.92
106.13	-5.84	-5.54	-5.22	-4.94	-4.65	-4.34	-4.23	-4.10	-4.01	-3.91	-3.77
113.45	-5.90	-5.56	-5.35	-4.92	-4.64	-4.55	-4.34	-4.23	-4.02	-4.02	-3.75
121.34	-6.01	-5.66	-5.32	-5.08	-4.72	-4.49	-4.37	-4.17	-4.05	-3.92	-3.78
129.86	-6.29	-5.94	-5.66	-5.28	-4.99	-4.70	-4.61	-4.47	-4.30	-4.23	-4.11
139.04	-5.82	-5.35	-5.21	-4.62	-4.38	-4.10	-3.98	-3.82	-3.66	-3.62	-3.41
148.94	-5.96	-5.56	-5.17	-4.99	-4.54	-4.28	-4.25	-3.91	-3.79	-3.68	-3.57
159.62	-5.75	-5.32	-5.05	-4.73	-4.50	-4.30	-3.96	-3.98	-3.72	-3.71	-3.47
171.14	-5.62	-5.37	-5.02	-4.68	-4.38	-4.12	-3.81	-3.79	-3.72	-3.51	-3.31

183.56	-6.07	-5.60	-5.29	-4.92	-4.56	-4.53	-4.20	-4.10	-3.94	-3.75	-3.60
196.96	-5.93	-5.54	-5.22	-4.92	-4.66	-4.45	-4.10	-3.93	-3.92	-3.68	-3.57
211.41	-5.73	-5.65	-5.12	-4.85	-4.57	-4.29	-3.96	-3.95	-3.77	-3.65	-3.50
226.99	-5.79	-5.24	-4.96	-4.79	-4.43	-4.23	-3.83	-3.72	-3.52	-3.45	-3.36
243.8	-5.84	-5.42	-5.11	-4.84	-4.50	-4.24	-3.99	-3.77	-3.66	-3.49	-3.37
261.93	-5.77	-5.38	-4.98	-4.61	-4.39	-4.17	-3.88	-3.61	-3.53	-3.40	-3.22
281.48	-5.57	-5.34	-4.91	-4.53	-4.28	-3.97	-3.67	-3.46	-3.27	-3.12	-2.88
302.56	-5.56	-5.20	-4.94	-4.57	-4.15	-3.98	-3.77	-3.53	-3.35	-3.14	-2.94
325.3	-5.77	-5.23	-4.93	-4.54	-4.27	-4.03	-3.67	-3.53	-3.24	-3.19	-2.96
349.83	-5.46	-5.05	-4.78	-4.34	-4.09	-3.90	-3.60	-3.41	-3.16	-3.02	-2.68
376.28	-5.61	-5.23	-4.82	-4.55	-4.13	-3.86	-3.68	-3.44	-3.16	-3.11	-2.85
404.81	-5.21	-4.72	-4.48	-4.12	-3.76	-3.42	-3.19	-3.05	-2.80	-2.54	-2.34
435.58	-5.57	-5.24	-4.88	-4.61	-4.03	-3.97	-3.59	-3.35	-3.12	-2.93	-2.59
468.76	-5.30	-4.90	-4.58	-4.08	-3.91	-3.57	-3.19	-2.98	-2.70	-2.57	-2.22
504.55	-5.09	-4.77	-4.37	-4.12	-3.64	-3.49	-3.13	-2.93	-2.71	-2.38	-2.12
543.15	-5.37	-4.79	-4.53	-4.16	-3.76	-3.50	-3.11	-2.94	-2.68	-2.33	-2.16
584.78	-5.01	-4.63	-4.40	-3.96	-3.60	-3.30	-2.94	-2.58	-2.43	-2.21	-1.98
629.68	-5.00	-4.54	-4.23	-3.81	-3.38	-3.29	-2.90	-2.67	-2.34	-2.09	-1.69
678.1	-4.66	-4.27	-3.87	-3.42	-3.09	-2.82	-2.44	-2.26	-1.95	-1.71	-1.42
730.33	-4.84	-4.43	-4.18	-3.95	-3.39	-3.17	-2.79	-2.55	-2.20	-2.01	-1.71
786.65	-4.87	-4.44	-4.10	-3.84	-3.39	-3.17	-2.71	-2.61	-2.23	-2.08	-1.73
847.4	-5.08	-4.73	-4.24	-3.89	-3.49	-3.25	-2.85	-2.70	-2.29	-2.05	-1.61
912.92	-4.71	-4.37	-4.10	-3.63	-3.31	-3.03	-2.66	-2.35	-2.12	-1.85	-1.49
983.58	-4.69	-4.37	-4.12	-3.57	-3.34	-2.96	-2.54	-2.24	-2.01	-1.77	-1.50
1059.78	-4.76	-4.32	-3.98	-3.62	-3.26	-2.91	-2.59	-2.25	-2.04	-1.64	-1.42
1141.98	-4.24	-3.86	-3.55	-3.18	-2.69	-2.42	-2.21	-1.95	-1.53	-1.33	-1.01
1230.62	-4.65	-4.16	-3.80	-3.47	-3.12	-2.81	-2.48	-2.17	-1.93	-1.62	-1.27
1326.23	-4.53	-4.17	-3.88	-3.41	-3.12	-2.79	-2.45	-2.25	-1.92	-1.55	-1.25
1429.34	-4.34	-4.01	-3.67	-3.26	-2.87	-2.50	-2.22	-1.99	-1.58	-1.27	-0.92
1540.54	-4.93	-4.54	-4.20	-3.75	-3.45	-2.98	-2.64	-2.38	-2.03	-1.85	-1.43
1660.48	-4.63	-4.17	-3.82	-3.50	-3.12	-2.73	-2.36	-2.11	-1.75	-1.50	-1.17
1789.83	-4.74	-4.51	-4.05	-3.62	-3.28	-2.96	-2.57	-2.32	-1.93	-1.67	-1.34
1929.34	-4.60	-4.36	-4.00	-3.57	-3.17	-2.80	-2.45	-2.14	-1.80	-1.64	-1.23
2079.8	-5.11	-4.49	-4.33	-3.94	-3.36	-3.29	-2.92	-2.53	-2.16	-1.87	-1.57
2242.08	-4.81	-4.25	-3.94	-3.71	-3.10	-2.92	-2.61	-2.19	-1.90	-1.58	-1.19
2417.1	-4.85	-4.41	-4.01	-3.61	-3.22	-2.94	-2.56	-2.20	-1.92	-1.61	-1.30
2605.85	-4.86	-4.40	-4.13	-3.73	-3.33	-2.95	-2.50	-2.40	-1.98	-1.79	-1.38
2809.43	-4.82	-4.48	-3.94	-3.66	-3.30	-2.92	-2.58	-2.23	-1.90	-1.70	-1.31
3028.99	-4.70	-4.33	-4.00	-3.54	-3.18	-2.88	-2.57	-2.30	-1.92	-1.48	-1.27
3265.79	-4.73	-4.25	-3.95	-3.60	-3.26	-2.87	-2.56	-2.24	-1.97	-1.61	-1.25
3521.18	-5.05	-4.64	-4.32	-3.91	-3.61	-3.23	-2.95	-2.75	-2.29	-2.10	-1.69
3796.62	-4.87	-4.29	-4.02	-3.58	-3.28	-2.95	-2.57	-2.33	-1.86	-1.60	-1.35

Wavelength (nm) / Time (ps)	522.13	523.53	524.94	526.35	527.76	529.17	530.58	531.99	533.39	534.80	536.21
-3.28	0.19	0.22	0.12	0.25	0.22	0.09	0.13	0.19	0.18	0.24	0.15
-2.78	0.09	0.10	0.01	0.19	0.04	0.08	0.09	0.08	0.02	0.03	0.09
-2.28	0.17	0.19	0.28	0.14	0.29	0.28	0.19	0.28	0.26	0.24	0.15

-1.78	0.03	-0.09	0.05	-0.03	0.06	-0.03	0.08	-0.17	-0.01	0.00	0.03
-1.28	-0.36	-0.25	-0.29	-0.31	-0.24	-0.19	-0.27	-0.32	-0.20	-0.29	-0.23
-0.78	0.02	0.07	0.13	0.05	0.04	0.08	0.06	0.09	0.01	0.02	0.05
-0.28	-0.46	-0.51	-0.48	-0.57	-0.63	-0.50	-0.51	-0.46	-0.56	-0.58	-0.58
0.22	0.31	0.26	0.18	0.30	0.23	0.19	0.24	0.31	0.30	0.34	0.34
0.32	0.08	0.11	0.17	0.19	0.19	0.07	0.12	0.17	0.17	0.14	0.28
0.42	-0.08	-0.17	-0.20	-0.11	-0.11	-0.10	-0.04	-0.12	-0.21	-0.22	-0.10
0.52	-0.28	-0.22	-0.25	-0.21	-0.31	-0.22	-0.28	-0.30	-0.35	-0.26	-0.24
0.62	0.15	0.14	0.29	0.24	0.21	0.18	0.15	0.10	0.13	0.17	0.11
0.72	-0.46	-0.46	-0.32	-0.30	-0.42	-0.36	-0.33	-0.41	-0.45	-0.34	-0.31
0.77	0.22	0.14	0.06	0.17	0.17	0.11	0.28	0.12	0.18	0.13	0.14
0.82	0.28	0.23	0.16	0.15	0.16	0.14	0.30	0.26	0.21	0.17	0.18
0.87	0.05	-0.01	0.01	-0.06	-0.11	-0.13	0.01	-0.11	-0.11	-0.01	0.02
0.92	-0.13	-0.19	-0.10	-0.19	-0.12	-0.07	-0.10	-0.06	-0.30	-0.11	-0.04
0.97	-0.04	-0.06	-0.04	-0.01	-0.09	-0.04	-0.14	-0.08	-0.17	0.01	-0.02
1.02	0.00	0.19	0.16	0.15	0.12	0.17	0.15	0.11	0.12	0.19	0.17
1.07	-0.04	0.10	-0.01	-0.10	-0.04	-0.07	0.10	0.07	-0.04	-0.07	-0.05
1.12	-0.28	-0.21	-0.28	-0.17	-0.26	-0.24	-0.19	-0.17	-0.23	-0.33	-0.23
1.17	-0.19	-0.23	-0.26	-0.17	-0.22	-0.20	-0.18	-0.16	-0.19	-0.21	-0.15
1.22	0.30	0.20	0.16	0.29	0.09	0.13	0.25	0.29	0.17	0.21	0.26
1.27	0.04	0.00	-0.03	-0.03	-0.02	0.02	-0.03	0.11	0.04	-0.01	-0.04
1.32	-0.13	-0.18	-0.12	-0.22	-0.27	-0.17	-0.18	-0.15	-0.21	-0.23	-0.19
1.37	-0.39	-0.19	-0.28	-0.24	-0.20	-0.22	-0.11	-0.07	0.01	-0.03	-0.05
1.42	-0.43	-0.47	-0.46	-0.58	-0.49	-0.70	-0.54	-0.43	-0.51	-0.47	-0.41
1.47	-2.45	-2.14	-1.80	-1.51	-1.24	-1.03	-0.60	-0.58	-0.24	-0.01	0.23
1.52	-8.89	-8.57	-8.01	-7.62	-7.13	-6.58	-5.99	-5.51	-4.87	-4.32	-3.46
1.57	-14.00	-13.79	-13.49	-13.07	-12.61	-12.28	-11.60	-11.04	-10.30	-9.50	-8.58
1.62	-16.06	-15.91	-15.87	-15.62	-15.50	-15.21	-14.60	-14.15	-13.65	-12.82	-11.97
1.67	-15.95	-16.03	-16.13	-16.04	-15.99	-15.84	-15.45	-15.17	-14.55	-14.07	-13.29
1.72	-14.14	-14.27	-14.42	-14.38	-14.30	-14.20	-13.92	-13.57	-13.17	-12.72	-11.97
1.77	-13.07	-13.21	-13.13	-13.20	-13.10	-13.06	-12.79	-12.54	-12.12	-11.68	-10.97
1.82	-12.58	-12.64	-12.72	-12.77	-12.64	-12.52	-12.31	-11.95	-11.60	-11.20	-10.70
1.87	-11.64	-11.71	-11.81	-11.78	-11.79	-11.54	-11.33	-11.03	-10.76	-10.33	-9.68
1.92	-10.89	-10.96	-11.10	-10.81	-10.84	-10.72	-10.45	-10.16	-9.81	-9.25	-8.62
1.97	-10.68	-10.61	-10.65	-10.60	-10.57	-10.39	-10.03	-9.54	-9.34	-8.70	-8.13
2.02	-9.66	-9.73	-9.63	-9.60	-9.43	-9.26	-9.00	-8.58	-8.11	-7.59	-6.88
2.07	-9.69	-9.63	-9.61	-9.49	-9.33	-9.03	-8.59	-8.25	-7.82	-7.18	-6.53
2.12	-9.28	-9.28	-9.19	-9.15	-8.91	-8.87	-8.43	-8.09	-7.58	-6.96	-6.35
2.17	-8.70	-8.80	-8.75	-8.70	-8.54	-8.28	-7.91	-7.62	-7.21	-6.53	-5.91
2.22	-8.37	-8.52	-8.36	-8.21	-8.03	-7.83	-7.50	-7.10	-6.75	-6.15	-5.44
2.27	-7.86	-7.79	-7.86	-7.76	-7.54	-7.30	-6.90	-6.44	-6.05	-5.44	-4.78
2.32	-8.02	-7.98	-7.98	-7.68	-7.59	-7.30	-6.78	-6.38	-5.93	-5.23	-4.57
2.37	-7.76	-7.73	-7.83	-7.61	-7.35	-7.10	-6.63	-6.32	-5.72	-5.15	-4.32
2.42	-7.54	-7.59	-7.39	-7.31	-7.16	-6.92	-6.46	-6.06	-5.49	-4.85	-4.05
2.47	-7.45	-7.35	-7.32	-7.13	-7.11	-6.83	-6.24	-5.89	-5.25	-4.71	-4.08
2.52	-7.42	-7.32	-7.22	-7.12	-6.84	-6.70	-6.19	-5.66	-5.21	-4.60	-3.92
2.57	-7.11	-7.14	-7.11	-6.83	-6.74	-6.34	-5.92	-5.53	-4.99	-4.21	-3.55
2.62	-7.31	-7.31	-7.14	-7.03	-6.86	-6.50	-6.02	-5.62	-5.03	-4.28	-3.54
2.67	-6.88	-6.85	-6.80	-6.60	-6.42	-6.11	-5.66	-5.20	-4.72	-4.02	-3.35
2.72	-6.81	-6.78	-6.67	-6.42	-6.34	-6.09	-5.70	-5.18	-4.62	-4.00	-3.17

2.77	-7.24	-7.28	-7.03	-6.92	-6.71	-6.41	-6.01	-5.44	-4.91	-4.36	-3.56
2.82	-6.34	-6.42	-6.13	-6.07	-5.87	-5.53	-5.01	-4.65	-3.99	-3.29	-2.48
2.87	-6.67	-6.65	-6.46	-6.38	-6.15	-5.84	-5.34	-4.82	-4.30	-3.68	-2.90
2.92	-6.31	-6.29	-6.19	-5.99	-5.80	-5.38	-4.95	-4.46	-3.86	-3.12	-2.34
2.97	-6.81	-6.81	-6.58	-6.45	-6.32	-5.95	-5.50	-5.08	-4.36	-3.65	-2.89
3.02	-6.48	-6.46	-6.34	-6.22	-6.01	-5.67	-5.20	-4.78	-4.10	-3.50	-2.74
3.07	-6.11	-6.13	-6.03	-5.76	-5.63	-5.33	-4.83	-4.40	-3.81	-3.06	-2.32
3.12	-6.15	-6.06	-6.00	-5.76	-5.52	-5.23	-4.71	-4.19	-3.65	-2.88	-2.12
3.17	-6.21	-6.12	-6.03	-5.87	-5.47	-5.28	-4.77	-4.15	-3.66	-2.94	-2.06
3.22	-6.20	-6.01	-6.02	-5.73	-5.57	-5.25	-4.76	-4.22	-3.57	-2.93	-2.16
3.27	-5.98	-5.97	-5.75	-5.70	-5.44	-5.07	-4.62	-4.20	-3.54	-2.92	-2.13
3.32	-5.69	-5.68	-5.46	-5.25	-5.06	-4.71	-4.38	-3.88	-3.14	-2.45	-1.60
3.37	-5.84	-5.80	-5.69	-5.56	-5.29	-5.00	-4.40	-3.99	-3.36	-2.62	-1.90
3.42	-5.86	-5.75	-5.65	-5.39	-5.18	-4.75	-4.34	-3.95	-3.35	-2.59	-1.72
3.47	-5.60	-5.49	-5.31	-5.15	-4.86	-4.68	-4.14	-3.77	-3.01	-2.33	-1.38
3.52	-5.99	-5.90	-5.78	-5.58	-5.36	-4.99	-4.54	-4.03	-3.39	-2.60	-1.97
3.57	-5.75	-5.62	-5.49	-5.32	-5.07	-4.76	-4.35	-3.89	-3.31	-2.59	-1.72
3.62	-5.75	-5.77	-5.60	-5.34	-5.13	-4.77	-4.23	-3.72	-3.21	-2.54	-1.64
3.67	-5.78	-5.79	-5.59	-5.44	-5.20	-4.88	-4.37	-3.85	-3.23	-2.44	-1.68
3.72	-5.27	-5.14	-5.04	-4.82	-4.67	-4.22	-3.78	-3.29	-2.64	-1.89	-0.93
3.92	-5.33	-5.35	-5.20	-4.83	-4.67	-4.38	-3.88	-3.37	-2.79	-2.10	-1.23
4.12	-5.21	-5.17	-5.04	-4.91	-4.60	-4.33	-3.78	-3.28	-2.71	-1.92	-1.19
4.32	-5.38	-5.38	-5.22	-4.92	-4.84	-4.44	-3.99	-3.52	-2.78	-2.13	-1.28
4.52	-5.04	-4.93	-4.73	-4.56	-4.29	-4.11	-3.49	-2.98	-2.31	-1.61	-0.84
4.72	-5.04	-5.03	-4.83	-4.63	-4.32	-4.03	-3.53	-2.98	-2.35	-1.63	-0.72
4.92	-5.22	-5.12	-4.95	-4.67	-4.47	-4.16	-3.70	-3.20	-2.48	-1.84	-0.89
5.12	-5.32	-5.28	-5.14	-4.90	-4.58	-4.29	-3.81	-3.36	-2.73	-1.98	-1.01
5.32	-5.09	-5.15	-4.85	-4.67	-4.43	-4.09	-3.63	-3.09	-2.44	-1.64	-0.94
5.52	-4.86	-4.80	-4.77	-4.56	-4.26	-3.90	-3.49	-3.03	-2.31	-1.70	-0.72
5.72	-5.40	-5.30	-5.09	-4.97	-4.76	-4.43	-3.87	-3.30	-2.78	-2.03	-1.02
5.92	-4.75	-4.73	-4.58	-4.47	-4.18	-3.82	-3.33	-2.85	-2.23	-1.44	-0.61
6.12	-5.07	-4.87	-4.81	-4.55	-4.53	-4.04	-3.60	-3.08	-2.33	-1.72	-0.78
6.32	-4.96	-4.85	-4.68	-4.56	-4.38	-3.96	-3.59	-2.96	-2.38	-1.65	-0.82
6.52	-4.68	-4.64	-4.54	-4.41	-4.27	-3.80	-3.38	-2.85	-2.21	-1.45	-0.64
6.72	-4.88	-5.01	-4.74	-4.58	-4.29	-3.91	-3.56	-2.96	-2.39	-1.69	-0.83
6.92	-4.81	-4.57	-4.50	-4.35	-4.20	-3.81	-3.36	-2.70	-2.12	-1.50	-0.62
7.12	-4.20	-4.07	-4.00	-3.91	-3.58	-3.25	-2.82	-2.33	-1.70	-0.97	-0.13
7.32	-4.79	-4.63	-4.45	-4.25	-4.11	-3.67	-3.18	-2.74	-2.08	-1.41	-0.41
7.52	-4.55	-4.41	-4.32	-4.14	-4.00	-3.70	-3.10	-2.59	-2.05	-1.37	-0.48
7.72	-4.93	-4.82	-4.74	-4.48	-4.25	-4.03	-3.60	-3.06	-2.52	-1.73	-0.82
7.92	-4.51	-4.45	-4.36	-4.19	-4.06	-3.65	-3.21	-2.68	-2.05	-1.30	-0.47
8.12	-4.52	-4.38	-4.30	-4.09	-3.98	-3.49	-3.06	-2.61	-2.02	-1.26	-0.45
8.32	-4.50	-4.44	-4.27	-4.12	-3.94	-3.60	-3.12	-2.60	-1.99	-1.34	-0.42
8.52	-4.51	-4.37	-4.34	-4.20	-4.05	-3.78	-3.19	-2.72	-2.17	-1.45	-0.53
8.72	-4.62	-4.52	-4.28	-4.17	-3.96	-3.55	-3.24	-2.68	-2.13	-1.44	-0.59
8.92	-4.87	-4.69	-4.68	-4.42	-4.25	-3.94	-3.38	-2.98	-2.40	-1.53	-0.76
9.12	-4.77	-4.67	-4.51	-4.33	-4.02	-3.81	-3.34	-2.84	-2.23	-1.53	-0.62
9.32	-5.00	-4.86	-4.88	-4.60	-4.41	-4.08	-3.70	-3.12	-2.49	-1.69	-0.82
9.52	-4.54	-4.52	-4.43	-4.35	-4.14	-3.77	-3.33	-2.78	-2.08	-1.39	-0.59
9.72	-4.53	-4.44	-4.33	-4.27	-4.01	-3.75	-3.35	-2.78	-2.28	-1.53	-0.58

9.92	-4.82	-4.79	-4.53	-4.51	-4.18	-3.92	-3.40	-2.93	-2.35	-1.63	-0.74
10.12	-4.34	-4.38	-4.14	-4.14	-3.81	-3.48	-2.96	-2.58	-1.97	-1.19	-0.35
10.32	-4.36	-4.37	-4.27	-4.13	-3.91	-3.62	-3.18	-2.69	-2.14	-1.34	-0.45
10.52	-4.52	-4.38	-4.42	-4.23	-3.94	-3.64	-3.25	-2.72	-2.27	-1.47	-0.67
10.72	-4.42	-4.37	-4.24	-4.12	-3.90	-3.62	-3.12	-2.68	-2.12	-1.39	-0.60
10.92	-4.56	-4.44	-4.38	-4.20	-4.05	-3.68	-3.32	-2.73	-2.18	-1.52	-0.57
11.12	-4.75	-4.72	-4.52	-4.39	-4.15	-3.92	-3.53	-3.01	-2.41	-1.63	-0.77
11.32	-4.37	-4.41	-4.30	-4.09	-3.95	-3.63	-3.14	-2.67	-2.13	-1.38	-0.59
11.52	-4.03	-4.06	-3.93	-3.83	-3.55	-3.27	-2.80	-2.31	-1.71	-1.11	-0.28
11.72	-4.36	-4.34	-4.23	-4.11	-3.87	-3.59	-3.08	-2.63	-2.00	-1.32	-0.43
11.92	-3.90	-3.84	-3.77	-3.63	-3.42	-3.23	-2.65	-2.29	-1.69	-0.91	-0.16
12.12	-3.77	-3.73	-3.65	-3.52	-3.37	-3.15	-2.61	-2.21	-1.64	-1.05	-0.24
12.32	-4.29	-4.20	-4.18	-3.92	-3.76	-3.49	-3.16	-2.53	-1.98	-1.26	-0.47
12.52	-4.23	-4.12	-3.93	-3.78	-3.64	-3.36	-2.88	-2.36	-1.71	-0.99	-0.24
12.72	-4.75	-4.68	-4.51	-4.37	-4.16	-3.83	-3.48	-2.90	-2.27	-1.56	-0.81
12.92	-4.38	-4.25	-4.20	-4.02	-3.73	-3.61	-3.13	-2.57	-2.01	-1.28	-0.51
13.12	-4.13	-4.10	-3.96	-3.83	-3.69	-3.36	-2.80	-2.37	-1.90	-1.25	-0.42
13.32	-4.84	-4.78	-4.70	-4.43	-4.24	-3.98	-3.51	-2.98	-2.42	-1.74	-0.89
13.52	-4.45	-4.34	-4.27	-4.09	-3.87	-3.65	-3.12	-2.63	-2.06	-1.32	-0.51
13.72	-4.34	-4.30	-4.28	-3.88	-3.81	-3.53	-2.98	-2.58	-1.96	-1.23	-0.36
13.92	-4.45	-4.44	-4.33	-4.17	-3.96	-3.57	-3.21	-2.73	-2.14	-1.40	-0.49
14	-4.38	-4.25	-4.19	-4.00	-3.82	-3.56	-3.09	-2.74	-2.08	-1.48	-0.56
14.08	-4.46	-4.30	-4.26	-4.21	-3.97	-3.67	-3.30	-2.78	-2.23	-1.53	-0.77
14.17	-4.11	-3.98	-3.87	-3.75	-3.64	-3.27	-2.89	-2.39	-1.82	-1.06	-0.36
14.27	-4.31	-4.26	-4.15	-4.19	-3.90	-3.60	-3.08	-2.61	-2.07	-1.35	-0.45
14.38	-4.31	-4.39	-4.27	-4.09	-3.87	-3.54	-3.10	-2.69	-2.10	-1.40	-0.48
14.49	-4.36	-4.21	-4.32	-4.08	-3.97	-3.66	-3.16	-2.73	-2.16	-1.47	-0.60
14.61	-4.58	-4.55	-4.32	-4.23	-4.09	-3.72	-3.38	-2.90	-2.29	-1.54	-0.67
14.75	-4.31	-4.33	-4.30	-4.09	-3.90	-3.64	-3.23	-2.66	-2.15	-1.37	-0.60
14.89	-4.31	-4.44	-4.35	-4.15	-3.95	-3.67	-3.17	-2.74	-2.15	-1.50	-0.57
15.05	-4.38	-4.23	-4.21	-4.08	-3.85	-3.53	-3.16	-2.59	-2.04	-1.34	-0.46
15.21	-4.37	-4.30	-4.25	-4.03	-3.88	-3.48	-3.12	-2.63	-1.96	-1.36	-0.44
15.39	-4.34	-4.32	-4.28	-3.98	-3.83	-3.61	-3.15	-2.77	-2.10	-1.33	-0.59
15.59	-4.34	-4.36	-4.26	-4.06	-3.88	-3.65	-3.20	-2.67	-2.12	-1.46	-0.54
15.8	-4.32	-4.33	-4.31	-4.05	-3.89	-3.57	-3.30	-2.71	-2.15	-1.35	-0.62
16.02	-4.08	-4.21	-4.06	-3.91	-3.69	-3.42	-3.00	-2.46	-1.93	-1.21	-0.30
16.27	-4.20	-4.10	-3.99	-3.92	-3.65	-3.38	-2.93	-2.49	-1.92	-1.04	-0.29
16.53	-4.07	-4.06	-3.98	-3.86	-3.72	-3.37	-2.81	-2.48	-1.78	-1.13	-0.21
16.81	-4.26	-4.31	-4.22	-4.05	-3.77	-3.58	-3.20	-2.72	-1.89	-1.37	-0.50
17.12	-4.27	-4.10	-4.13	-3.91	-3.75	-3.50	-3.07	-2.57	-1.91	-1.24	-0.41
17.45	-4.32	-4.21	-4.22	-4.00	-3.92	-3.61	-3.09	-2.63	-2.08	-1.38	-0.60
17.81	-4.09	-4.07	-4.05	-3.84	-3.61	-3.39	-2.92	-2.39	-1.99	-1.17	-0.30
18.19	-4.15	-4.12	-4.03	-3.91	-3.70	-3.44	-3.01	-2.51	-1.96	-1.15	-0.36
18.6	-4.10	-4.09	-4.00	-3.82	-3.63	-3.38	-3.05	-2.49	-1.99	-1.32	-0.41
19.05	-4.50	-4.54	-4.40	-4.26	-4.09	-3.78	-3.31	-2.99	-2.40	-1.67	-0.83
19.53	-4.14	-4.01	-3.98	-3.77	-3.53	-3.27	-2.79	-2.36	-1.78	-1.06	-0.24
20.05	-4.13	-4.13	-4.05	-3.89	-3.83	-3.46	-3.07	-2.51	-1.99	-1.39	-0.49
20.61	-4.33	-4.35	-4.19	-3.98	-3.81	-3.51	-3.09	-2.57	-2.07	-1.32	-0.50
21.22	-4.40	-4.34	-4.26	-4.08	-4.01	-3.70	-3.21	-2.75	-2.21	-1.42	-0.69
21.87	-3.93	-3.88	-3.84	-3.69	-3.56	-3.37	-2.81	-2.39	-1.76	-1.16	-0.25

22.57	-4.27	-4.29	-4.17	-3.95	-3.90	-3.57	-3.21	-2.62	-2.01	-1.42	-0.53
23.33	-3.87	-3.83	-3.82	-3.66	-3.41	-3.23	-2.76	-2.28	-1.69	-1.03	-0.17
24.15	-3.90	-3.78	-3.87	-3.57	-3.41	-3.10	-2.75	-2.26	-1.69	-0.93	-0.09
25.03	-4.08	-3.98	-3.86	-3.64	-3.49	-3.30	-2.86	-2.29	-1.75	-1.06	-0.20
25.98	-4.51	-4.55	-4.46	-4.41	-4.09	-3.82	-3.45	-2.93	-2.37	-1.71	-0.72
27.01	-4.47	-4.45	-4.42	-4.29	-4.06	-3.83	-3.36	-2.94	-2.41	-1.73	-0.78
28.11	-4.03	-3.86	-3.88	-3.80	-3.59	-3.35	-2.90	-2.45	-1.85	-1.18	-0.34
29.3	-4.39	-4.35	-4.29	-4.16	-3.91	-3.66	-3.18	-2.71	-2.10	-1.36	-0.47
30.59	-3.94	-4.09	-3.84	-3.77	-3.50	-3.28	-2.77	-2.30	-1.91	-1.05	-0.38
31.98	-4.17	-4.19	-3.99	-3.85	-3.71	-3.43	-2.90	-2.46	-1.86	-1.17	-0.28
33.47	-3.82	-3.82	-3.77	-3.61	-3.52	-3.15	-2.67	-2.27	-1.74	-1.00	-0.24
35.09	-3.96	-3.92	-3.74	-3.70	-3.50	-3.19	-2.87	-2.46	-1.85	-1.01	-0.16
36.83	-4.03	-3.90	-3.82	-3.65	-3.40	-3.25	-2.77	-2.21	-1.66	-0.94	-0.12
38.71	-3.88	-4.05	-3.79	-3.69	-3.42	-3.24	-2.80	-2.30	-1.69	-1.01	-0.09
40.73	-4.04	-4.05	-3.92	-3.91	-3.60	-3.24	-2.91	-2.24	-1.68	-0.90	-0.12
42.92	-3.91	-4.00	-3.84	-3.64	-3.39	-3.22	-2.70	-2.17	-1.54	-0.82	0.14
45.27	-4.08	-4.08	-3.99	-3.84	-3.64	-3.31	-2.99	-2.41	-1.78	-1.13	-0.21
47.81	-4.53	-4.49	-4.42	-4.22	-3.98	-3.65	-3.22	-2.78	-2.09	-1.42	-0.49
50.55	-4.00	-3.95	-3.94	-3.73	-3.46	-3.11	-2.68	-2.30	-1.65	-0.88	-0.15
53.51	-4.19	-4.18	-3.94	-3.84	-3.58	-3.28	-2.76	-2.42	-1.75	-1.08	-0.31
56.69	-3.97	-3.92	-3.82	-3.64	-3.44	-3.18	-2.66	-2.24	-1.48	-0.78	0.21
60.13	-3.88	-3.83	-3.70	-3.57	-3.24	-3.01	-2.67	-2.09	-1.44	-0.77	0.09
63.84	-4.08	-4.01	-3.90	-3.72	-3.59	-3.30	-2.76	-2.25	-1.58	-0.83	0.06
67.83	-4.43	-4.35	-4.22	-4.06	-3.78	-3.49	-3.01	-2.52	-1.85	-1.13	-0.10
72.15	-4.11	-4.11	-3.91	-3.59	-3.51	-3.19	-2.75	-2.18	-1.62	-0.84	0.01
76.8	-3.97	-4.03	-3.83	-3.62	-3.49	-3.03	-2.64	-2.15	-1.47	-0.74	0.08
81.81	-3.85	-3.76	-3.62	-3.42	-3.22	-2.93	-2.35	-1.84	-1.16	-0.48	0.42
87.22	-3.71	-3.70	-3.57	-3.39	-3.15	-2.93	-2.28	-1.86	-1.24	-0.49	0.47
93.06	-3.84	-3.80	-3.49	-3.34	-3.13	-2.81	-2.30	-1.69	-1.06	-0.30	0.61
99.35	-3.90	-3.74	-3.56	-3.40	-3.07	-2.66	-2.39	-1.64	-1.04	-0.19	0.74
106.13	-3.66	-3.67	-3.36	-3.23	-2.98	-2.66	-2.11	-1.48	-0.93	-0.09	0.77
113.45	-3.67	-3.62	-3.47	-3.20	-2.99	-2.62	-2.09	-1.66	-0.86	-0.06	0.89
121.34	-3.80	-3.79	-3.55	-3.38	-3.16	-2.73	-2.19	-1.69	-1.04	-0.31	0.70
129.86	-3.98	-3.84	-3.79	-3.50	-3.20	-2.80	-2.30	-1.67	-1.11	-0.25	0.65
139.04	-3.43	-3.32	-2.96	-2.78	-2.46	-2.07	-1.48	-0.92	-0.33	0.47	1.44
148.94	-3.52	-3.41	-3.20	-3.00	-2.58	-2.27	-1.63	-1.07	-0.27	0.47	1.40
159.62	-3.28	-3.28	-3.02	-2.77	-2.56	-2.13	-1.60	-0.95	-0.21	0.49	1.43
171.14	-3.26	-3.22	-3.07	-2.69	-2.35	-2.02	-1.43	-0.95	-0.19	0.67	1.58
183.56	-3.40	-3.33	-3.16	-3.00	-2.57	-2.22	-1.65	-1.03	-0.35	0.48	1.42
196.96	-3.39	-3.27	-3.03	-2.84	-2.49	-2.09	-1.56	-0.88	-0.09	0.66	1.75
211.41	-3.22	-3.16	-2.86	-2.63	-2.24	-1.91	-1.31	-0.59	0.19	0.99	2.11
226.99	-3.15	-3.00	-2.80	-2.39	-2.11	-1.61	-0.89	-0.44	0.32	1.34	2.25
243.8	-3.24	-3.00	-2.83	-2.44	-2.17	-1.68	-1.00	-0.43	0.28	1.26	2.33
261.93	-2.88	-2.78	-2.61	-2.32	-1.79	-1.48	-0.78	-0.15	0.65	1.57	2.61
281.48	-2.71	-2.58	-2.24	-1.93	-1.57	-1.20	-0.45	0.20	0.94	1.99	2.93
302.56	-2.76	-2.49	-2.26	-1.92	-1.50	-1.01	-0.36	0.30	1.01	2.02	3.11
325.3	-2.76	-2.55	-2.25	-1.88	-1.43	-0.96	-0.29	0.29	1.14	2.17	3.13
349.83	-2.52	-2.40	-2.06	-1.67	-1.46	-0.79	-0.23	0.53	1.23	2.22	3.24
376.28	-2.53	-2.29	-2.00	-1.64	-1.26	-0.68	-0.07	0.69	1.55	2.46	3.52
404.81	-2.15	-1.90	-1.56	-1.29	-0.92	-0.34	0.43	1.11	1.80	2.85	3.86

435.58	-2.50	-2.19	-1.75	-1.40	-0.89	-0.44	0.23	0.93	1.89	2.77	3.91
468.76	-2.08	-1.76	-1.52	-1.02	-0.67	-0.08	0.61	1.44	2.31	3.30	4.44
504.55	-1.82	-1.64	-1.34	-0.91	-0.47	0.03	0.88	1.67	2.55	3.40	4.51
543.15	-1.81	-1.59	-1.35	-0.88	-0.44	0.08	0.79	1.67	2.56	3.45	4.68
584.78	-1.58	-1.38	-1.10	-0.58	-0.06	0.53	1.32	1.96	2.90	3.90	5.06
629.68	-1.57	-1.26	-0.92	-0.43	0.05	0.59	1.35	2.23	2.99	4.00	5.15
678.1	-1.14	-0.80	-0.48	-0.01	0.45	1.09	1.87	2.56	3.48	4.54	5.68
730.33	-1.35	-1.21	-0.71	-0.35	0.22	0.84	1.64	2.36	3.30	4.28	5.46
786.65	-1.39	-1.21	-0.79	-0.37	0.27	0.83	1.60	2.43	3.27	4.31	5.52
847.4	-1.39	-1.05	-0.62	-0.10	0.33	1.02	1.69	2.70	3.52	4.56	5.78
912.92	-1.15	-0.81	-0.53	-0.01	0.51	1.12	1.86	2.70	3.61	4.56	5.84
983.58	-1.11	-0.90	-0.43	0.11	0.56	1.16	1.90	2.81	3.78	4.71	5.96
1059.78	-1.00	-0.70	-0.36	0.17	0.63	1.32	2.10	2.92	3.86	4.92	6.08
1141.98	-0.76	-0.51	-0.02	0.43	0.92	1.60	2.37	3.15	4.06	5.13	6.34
1230.62	-0.82	-0.56	-0.16	0.34	0.83	1.49	2.22	3.04	4.01	5.06	6.23
1326.23	-0.87	-0.56	-0.22	0.41	0.95	1.48	2.30	3.15	4.14	5.27	6.43
1429.34	-0.60	-0.18	0.18	0.52	1.09	1.78	2.61	3.44	4.40	5.41	6.60
1540.54	-1.05	-0.81	-0.45	0.12	0.68	1.34	2.07	3.00	3.93	5.04	6.23
1660.48	-0.88	-0.46	-0.09	0.40	0.93	1.57	2.43	3.35	4.18	5.36	6.56
1789.83	-0.95	-0.71	-0.28	0.12	0.78	1.49	2.18	3.07	4.05	5.19	6.31
1929.34	-0.98	-0.45	-0.10	0.37	0.99	1.65	2.50	3.41	4.33	5.49	6.68
2079.8	-1.11	-0.83	-0.49	0.01	0.48	1.24	1.95	2.88	3.90	4.93	6.07
2242.08	-0.86	-0.55	-0.05	0.41	0.92	1.77	2.48	3.33	4.28	5.39	6.64
2417.1	-0.95	-0.65	-0.11	0.26	0.93	1.55	2.36	3.15	4.12	5.29	6.55
2605.85	-1.12	-0.80	-0.33	0.22	0.74	1.44	2.30	3.04	3.97	5.12	6.27
2809.43	-0.98	-0.55	-0.25	0.22	0.77	1.50	2.29	3.14	4.13	5.16	6.41
3028.99	-0.89	-0.66	-0.14	0.36	0.82	1.48	2.27	3.04	3.99	5.16	6.29
3265.79	-0.90	-0.44	-0.19	0.32	0.91	1.57	2.31	3.16	4.04	5.15	6.34
3521.18	-1.37	-0.98	-0.60	-0.01	0.44	1.10	2.03	2.78	3.74	4.74	5.95
3796.62	-1.13	-0.52	-0.31	0.31	0.80	1.60	2.26	3.11	4.11	5.21	6.38

Wavelength (nm) / Time (ps)	537.62	539.03	540.44	541.85	543.25	544.66	546.07	547.48	548.89	550.30	551.71
-3.28	0.35	0.26	0.19	0.16	0.28	0.21	0.22	0.18	0.12	0.20	0.24
-2.78	0.09	0.11	0.07	0.12	0.11	0.15	0.13	0.09	0.07	0.10	0.10
-2.28	0.17	0.20	0.24	0.20	0.26	0.21	0.25	0.18	0.19	0.19	0.24
-1.78	-0.01	-0.07	-0.01	0.03	-0.04	0.02	0.00	0.06	0.05	0.05	0.02
-1.28	-0.24	-0.21	-0.12	-0.20	-0.27	-0.26	-0.23	-0.15	-0.18	-0.19	-0.27
-0.78	0.00	0.07	0.02	0.13	0.02	0.04	0.04	-0.01	0.08	0.05	0.03
-0.28	-0.60	-0.63	-0.68	-0.69	-0.61	-0.64	-0.63	-0.61	-0.64	-0.65	-0.65
0.22	0.24	0.27	0.29	0.24	0.24	0.28	0.22	0.25	0.32	0.27	0.29
0.32	0.19	0.16	0.16	0.24	0.20	0.23	0.30	0.21	0.32	0.27	0.19
0.42	-0.09	-0.09	-0.13	-0.06	-0.10	-0.13	-0.10	-0.09	-0.11	-0.01	-0.04
0.52	-0.23	-0.18	-0.18	-0.20	-0.22	-0.27	-0.19	-0.22	-0.20	-0.19	-0.23
0.62	0.15	0.07	0.15	0.16	0.23	0.20	0.11	0.14	0.21	0.18	0.23
0.72	-0.36	-0.31	-0.28	-0.30	-0.24	-0.29	-0.29	-0.22	-0.23	-0.26	-0.26
0.77	0.25	0.12	0.11	0.22	0.20	0.12	0.27	0.17	0.23	0.21	0.19
0.82	0.11	0.08	0.13	0.14	0.12	0.11	0.14	0.08	0.11	0.04	0.10

0.87	-0.10	-0.13	-0.08	-0.08	-0.06	-0.15	-0.08	-0.12	-0.13	-0.16	-0.11
0.92	-0.14	-0.10	-0.10	-0.13	-0.13	-0.16	-0.04	-0.17	-0.13	-0.09	-0.07
0.97	-0.08	-0.05	-0.05	0.10	0.00	-0.05	-0.02	-0.01	0.00	0.00	0.04
1.02	0.22	0.19	0.15	0.27	0.16	0.15	0.18	0.23	0.24	0.27	0.27
1.07	-0.01	-0.06	0.01	0.04	0.00	0.03	0.01	0.05	0.04	0.05	-0.03
1.12	-0.30	-0.21	-0.19	-0.15	-0.22	-0.23	-0.17	-0.17	-0.19	-0.21	-0.18
1.17	-0.17	-0.13	-0.09	-0.12	-0.06	-0.11	-0.09	-0.16	-0.04	-0.14	-0.05
1.22	0.29	0.28	0.33	0.33	0.32	0.30	0.36	0.35	0.31	0.37	0.37
1.27	0.02	-0.03	0.01	0.08	0.11	0.12	0.09	-0.09	0.08	0.12	0.05
1.32	-0.11	-0.12	-0.09	-0.15	-0.11	-0.17	-0.12	-0.16	-0.13	-0.09	-0.15
1.37	0.02	-0.03	-0.09	-0.07	-0.03	-0.07	0.00	-0.09	-0.02	-0.04	0.05
1.42	-0.45	-0.36	-0.37	-0.39	-0.48	-0.44	-0.38	-0.39	-0.30	-0.38	-0.25
1.47	0.50	0.68	0.86	1.16	1.29	1.41	1.48	1.60	1.48	1.56	1.58
1.52	-2.79	-2.02	-1.20	-0.46	0.37	1.15	1.80	2.40	2.92	3.36	3.79
1.57	-7.63	-6.50	-5.32	-4.14	-3.00	-1.69	-0.50	0.71	1.82	2.87	3.84
1.62	-10.97	-9.91	-8.74	-7.43	-6.21	-4.81	-3.40	-1.96	-0.54	0.90	2.27
1.67	-12.50	-11.54	-10.45	-9.36	-8.20	-6.91	-5.68	-4.32	-2.81	-1.49	-0.06
1.72	-11.22	-10.38	-9.47	-8.52	-7.47	-6.27	-5.20	-4.00	-2.75	-1.51	-0.27
1.77	-10.21	-9.42	-8.65	-7.66	-6.72	-5.74	-4.56	-3.45	-2.27	-1.22	-0.04
1.82	-9.92	-9.12	-8.38	-7.45	-6.51	-5.51	-4.43	-3.40	-2.36	-1.23	-0.19
1.87	-9.05	-8.29	-7.48	-6.51	-5.67	-4.65	-3.66	-2.63	-1.65	-0.62	0.36
1.92	-7.96	-7.18	-6.35	-5.48	-4.45	-3.52	-2.46	-1.48	-0.49	0.61	1.70
1.97	-7.30	-6.54	-5.63	-4.68	-3.76	-2.74	-1.72	-0.65	0.38	1.47	2.49
2.02	-6.08	-5.34	-4.37	-3.49	-2.45	-1.43	-0.43	0.66	1.70	2.89	3.87
2.07	-5.68	-4.82	-3.89	-2.81	-1.82	-0.73	0.41	1.43	2.58	3.66	4.76
2.12	-5.56	-4.74	-3.82	-2.83	-1.82	-0.76	0.18	1.31	2.44	3.48	4.51
2.17	-5.14	-4.34	-3.41	-2.47	-1.45	-0.43	0.74	1.78	2.89	3.92	4.98
2.22	-4.59	-3.87	-2.88	-1.94	-0.98	0.15	1.34	2.29	3.42	4.47	5.58
2.27	-3.91	-3.07	-2.09	-1.11	-0.06	1.09	2.17	3.38	4.45	5.48	6.60
2.32	-3.63	-2.75	-1.73	-0.74	0.41	1.50	2.66	3.78	5.00	6.02	7.16
2.37	-3.55	-2.54	-1.61	-0.57	0.47	1.69	2.85	3.97	4.98	6.18	7.35
2.42	-3.32	-2.46	-1.46	-0.45	0.61	1.71	2.90	4.04	5.24	6.40	7.51
2.47	-3.22	-2.30	-1.32	-0.22	0.86	1.96	3.16	4.29	5.44	6.63	7.69
2.52	-3.06	-2.16	-1.16	-0.08	0.96	2.12	3.31	4.40	5.59	6.75	7.83
2.57	-2.69	-1.72	-0.90	0.32	1.43	2.54	3.72	4.92	6.13	7.22	8.42
2.62	-2.64	-1.75	-0.65	0.48	1.57	2.76	3.89	5.10	6.34	7.52	8.62
2.67	-2.41	-1.51	-0.54	0.54	1.74	2.80	4.05	5.22	6.35	7.57	8.77
2.72	-2.30	-1.44	-0.45	0.60	1.68	2.83	4.07	5.21	6.32	7.54	8.71
2.77	-2.66	-1.70	-0.64	0.44	1.62	2.79	3.98	5.14	6.36	7.58	8.76
2.82	-1.63	-0.65	0.34	1.48	2.64	3.78	5.00	6.19	7.44	8.55	9.75
2.87	-2.03	-1.10	0.10	1.14	2.26	3.49	4.73	5.77	7.07	8.31	9.43
2.92	-1.35	-0.44	0.61	1.76	2.86	4.05	5.32	6.55	7.81	9.04	10.20
2.97	-2.00	-0.97	0.13	1.18	2.35	3.63	4.90	6.18	7.47	8.67	9.91
3.02	-1.79	-0.88	0.14	1.36	2.54	3.66	4.98	6.23	7.37	8.62	9.84
3.07	-1.41	-0.39	0.61	1.83	2.84	4.04	5.22	6.53	7.75	9.00	10.07
3.12	-1.02	-0.08	1.01	2.26	3.46	4.67	6.00	7.24	8.57	9.77	11.04
3.17	-1.21	-0.08	0.87	2.07	3.33	4.52	5.78	7.02	8.24	9.55	10.77
3.22	-1.17	-0.25	0.85	1.99	3.18	4.34	5.62	6.86	8.22	9.42	10.63
3.27	-1.28	-0.28	0.74	1.96	3.08	4.31	5.58	6.76	8.06	9.26	10.40
3.32	-0.75	0.16	1.28	2.49	3.70	4.92	6.20	7.41	8.74	10.03	11.26

3.37	-0.74	0.24	1.18	2.42	3.54	4.86	6.03	7.32	8.71	9.88	11.13
3.42	-0.81	0.25	1.21	2.45	3.60	4.82	6.07	7.32	8.53	9.87	11.04
3.47	-0.53	0.53	1.62	2.68	3.83	5.18	6.46	7.69	9.02	10.18	11.50
3.52	-0.95	0.10	1.10	2.40	3.53	4.76	6.14	7.35	8.67	10.00	11.28
3.57	-0.88	0.13	1.33	2.43	3.54	4.83	6.04	7.33	8.70	9.94	11.10
3.62	-0.74	0.29	1.44	2.51	3.80	5.07	6.39	7.54	8.91	10.25	11.47
3.67	-0.68	0.31	1.42	2.66	3.80	5.12	6.38	7.58	9.02	10.30	11.50
3.72	-0.08	0.98	2.16	3.39	4.61	5.85	7.07	8.45	9.78	11.15	12.36
3.92	-0.35	0.73	1.70	2.95	4.12	5.35	6.60	7.87	9.27	10.44	11.71
4.12	-0.21	0.80	1.88	3.12	4.19	5.53	6.83	8.20	9.56	10.88	12.13
4.32	-0.31	0.71	1.87	3.05	4.24	5.58	6.95	8.34	9.65	10.97	12.35
4.52	0.18	1.38	2.44	3.74	4.89	6.12	7.50	8.83	10.22	11.56	12.87
4.72	0.17	1.24	2.34	3.56	4.93	6.16	7.61	8.83	10.25	11.63	12.92
4.92	0.08	1.14	2.28	3.51	4.64	6.01	7.39	8.78	10.11	11.50	12.74
5.12	-0.04	0.95	2.11	3.32	4.64	5.91	7.35	8.65	10.13	11.40	12.82
5.32	0.07	1.13	2.27	3.48	4.79	6.08	7.51	8.88	10.25	11.72	12.97
5.52	0.18	1.17	2.29	3.53	4.82	6.14	7.48	8.88	10.24	11.64	12.96
5.72	-0.01	0.95	2.11	3.43	4.67	5.95	7.43	8.77	10.18	11.56	12.98
5.92	0.52	1.46	2.61	3.87	5.12	6.45	7.82	9.19	10.57	12.04	13.36
6.12	0.13	1.17	2.30	3.64	4.86	6.18	7.54	8.85	10.33	11.61	13.01
6.32	0.17	1.17	2.30	3.56	4.75	6.04	7.44	8.79	10.21	11.60	12.91
6.52	0.27	1.33	2.46	3.64	4.99	6.29	7.74	9.04	10.46	11.86	13.20
6.72	0.27	1.33	2.51	3.75	5.00	6.32	7.69	9.03	10.49	11.86	13.28
6.92	0.20	1.41	2.53	3.72	5.05	6.23	7.64	9.00	10.37	11.78	13.10
7.12	0.76	1.90	3.01	4.18	5.46	6.76	8.11	9.51	10.90	12.20	13.54
7.32	0.53	1.64	2.74	3.91	5.20	6.48	7.97	9.31	10.73	12.04	13.47
7.52	0.34	1.44	2.43	3.77	4.99	6.39	7.82	9.13	10.53	11.88	13.23
7.72	0.06	1.13	2.24	3.53	4.71	6.03	7.50	8.73	10.16	11.65	13.05
7.92	0.49	1.58	2.63	3.93	5.18	6.50	7.95	9.33	10.78	12.13	13.58
8.12	0.59	1.55	2.69	3.95	5.18	6.60	7.85	9.26	10.67	12.11	13.39
8.32	0.56	1.58	2.73	3.93	5.15	6.57	7.96	9.33	10.78	12.21	13.55
8.52	0.31	1.41	2.60	3.76	4.99	6.33	7.73	9.09	10.56	12.01	13.30
8.72	0.34	1.37	2.53	3.71	4.99	6.25	7.75	9.12	10.54	11.93	13.28
8.92	0.28	1.29	2.49	3.63	4.96	6.24	7.65	9.02	10.50	11.88	13.36
9.12	0.38	1.48	2.58	3.86	5.17	6.48	7.82	9.27	10.65	12.01	13.50
9.32	0.08	1.15	2.32	3.57	4.95	6.24	7.70	8.97	10.46	11.98	13.32
9.52	0.49	1.54	2.62	3.96	5.30	6.66	8.06	9.46	10.97	12.38	13.73
9.72	0.33	1.49	2.49	3.80	5.16	6.45	7.88	9.29	10.80	12.14	13.53
9.92	0.29	1.22	2.44	3.69	4.96	6.31	7.79	9.16	10.62	12.08	13.50
10.12	0.66	1.58	2.68	3.98	5.24	6.58	7.98	9.32	10.69	12.21	13.49
10.32	0.47	1.46	2.67	3.88	5.18	6.53	7.84	9.25	10.68	12.11	13.48
10.52	0.39	1.36	2.58	3.80	5.02	6.39	7.82	9.14	10.59	12.02	13.38
10.72	0.29	1.42	2.54	3.70	4.96	6.27	7.62	8.92	10.37	11.77	13.11
10.92	0.30	1.35	2.49	3.71	4.95	6.28	7.72	9.13	10.51	11.92	13.32
11.12	0.18	1.28	2.35	3.66	4.88	6.29	7.59	9.00	10.50	11.96	13.34
11.32	0.31	1.49	2.56	3.89	5.15	6.42	7.85	9.24	10.67	12.02	13.39
11.52	0.73	1.77	2.91	4.21	5.42	6.69	8.12	9.42	10.88	12.24	13.66
11.72	0.42	1.43	2.57	3.81	5.03	6.36	7.78	9.23	10.57	11.96	13.36
11.92	0.69	1.72	2.71	3.85	5.17	6.40	7.78	9.09	10.58	11.87	13.31
12.12	0.70	1.67	2.83	4.03	5.26	6.47	7.99	9.32	10.62	11.99	13.36

12.32	0.58	1.63	2.72	3.96	5.16	6.52	7.89	9.24	10.70	12.09	13.49
12.52	0.77	1.80	2.88	4.25	5.50	6.90	8.26	9.66	11.11	12.56	13.93
12.72	0.35	1.36	2.53	3.80	5.06	6.45	7.95	9.32	10.67	12.16	13.56
12.92	0.40	1.42	2.63	3.86	5.12	6.43	7.77	9.28	10.67	12.11	13.50
13.12	0.54	1.62	2.66	3.90	5.16	6.53	7.91	9.22	10.78	12.14	13.55
13.32	0.14	1.16	2.29	3.56	4.85	6.30	7.63	9.02	10.47	12.05	13.38
13.52	0.55	1.51	2.72	3.82	5.16	6.51	8.01	9.34	10.91	12.22	13.61
13.72	0.57	1.70	2.87	4.14	5.32	6.75	8.18	9.45	11.03	12.43	13.86
13.92	0.39	1.52	2.68	3.88	5.21	6.59	8.02	9.51	10.99	12.32	13.80
14	0.35	1.34	2.50	3.70	4.89	6.18	7.62	9.03	10.46	11.84	13.19
14.08	0.28	1.29	2.37	3.59	5.00	6.20	7.64	9.00	10.46	11.87	13.24
14.17	0.63	1.76	2.79	3.96	5.24	6.49	7.93	9.31	10.72	12.15	13.50
14.27	0.43	1.50	2.70	3.86	5.21	6.52	7.96	9.31	10.69	12.15	13.55
14.38	0.36	1.60	2.64	4.00	5.24	6.53	7.89	9.32	10.78	12.15	13.57
14.49	0.37	1.28	2.45	3.71	4.88	6.22	7.65	9.04	10.41	11.80	13.18
14.61	0.19	1.29	2.43	3.61	4.89	6.27	7.78	9.16	10.63	12.02	13.42
14.75	0.40	1.49	2.64	3.82	5.16	6.49	7.92	9.29	10.71	12.17	13.60
14.89	0.35	1.43	2.49	3.70	5.07	6.44	7.86	9.26	10.72	12.05	13.47
15.05	0.52	1.65	2.76	4.09	5.35	6.74	8.16	9.47	10.99	12.51	13.91
15.21	0.48	1.46	2.66	3.91	5.16	6.55	7.96	9.37	10.84	12.24	13.62
15.39	0.47	1.44	2.51	3.85	5.04	6.38	7.78	9.20	10.60	12.00	13.44
15.59	0.32	1.40	2.60	3.85	5.02	6.32	7.74	9.18	10.62	12.08	13.50
15.8	0.33	1.36	2.45	3.67	4.90	6.21	7.61	8.95	10.43	11.83	13.13
16.02	0.60	1.55	2.67	4.00	5.22	6.57	7.86	9.29	10.76	12.10	13.49
16.27	0.67	1.78	2.96	4.13	5.44	6.78	8.29	9.62	11.15	12.60	13.98
16.53	0.68	1.79	2.79	3.97	5.29	6.68	8.05	9.44	10.98	12.37	13.76
16.81	0.47	1.46	2.66	3.89	5.15	6.46	7.88	9.33	10.77	12.17	13.59
17.12	0.46	1.44	2.56	3.81	5.03	6.43	7.84	9.25	10.76	11.97	13.43
17.45	0.34	1.48	2.68	3.85	5.28	6.62	7.89	9.37	10.79	12.29	13.74
17.81	0.67	1.68	2.88	4.11	5.37	6.75	8.07	9.58	10.95	12.42	13.81
18.19	0.62	1.74	2.78	4.13	5.43	6.74	8.17	9.58	11.05	12.45	13.88
18.6	0.56	1.62	2.73	3.95	5.20	6.52	7.94	9.24	10.74	12.10	13.56
19.05	0.18	1.19	2.41	3.64	4.84	6.27	7.62	9.06	10.51	12.01	13.38
19.53	0.77	1.73	2.91	4.15	5.48	6.78	8.33	9.63	11.07	12.47	13.93
20.05	0.44	1.52	2.66	3.84	5.17	6.50	7.81	9.23	10.56	12.05	13.46
20.61	0.48	1.60	2.81	4.00	5.31	6.65	8.02	9.54	10.89	12.45	13.84
21.22	0.34	1.37	2.53	3.75	5.11	6.36	7.79	9.21	10.66	12.05	13.50
21.87	0.57	1.67	2.82	4.05	5.26	6.52	7.97	9.40	10.88	12.25	13.64
22.57	0.43	1.55	2.58	3.80	5.14	6.55	7.90	9.36	10.80	12.20	13.67
23.33	0.77	1.86	2.94	4.18	5.46	6.72	8.18	9.49	10.90	12.39	13.73
24.15	0.87	1.93	3.07	4.39	5.62	6.95	8.37	9.69	11.20	12.64	13.98
25.03	0.92	1.92	3.05	4.31	5.59	6.87	8.37	9.74	11.17	12.61	14.04
25.98	0.14	1.09	2.21	3.39	4.73	6.17	7.59	9.00	10.43	11.79	13.28
27.01	0.09	1.13	2.28	3.51	4.86	6.13	7.54	9.00	10.33	11.78	13.27
28.11	0.70	1.69	2.87	4.16	5.34	6.58	8.19	9.47	10.83	12.29	13.69
29.3	0.46	1.43	2.64	3.88	5.23	6.57	8.00	9.48	10.92	12.38	13.77
30.59	0.65	1.75	2.83	3.97	5.33	6.66	7.91	9.33	10.84	12.23	13.58
31.98	0.69	1.71	2.92	4.11	5.35	6.81	8.20	9.58	11.03	12.51	13.92
33.47	0.76	1.80	2.97	4.21	5.57	6.84	8.38	9.69	11.11	12.50	14.01
35.09	0.75	1.90	2.92	4.19	5.50	6.90	8.27	9.77	11.16	12.68	14.08

36.83	0.85	1.97	3.11	4.37	5.60	7.02	8.45	9.85	11.35	12.79	14.21
38.71	0.85	1.95	3.18	4.37	5.68	7.03	8.52	9.95	11.41	12.83	14.24
40.73	0.77	1.91	3.05	4.34	5.67	7.03	8.46	9.84	11.39	12.91	14.22
42.92	1.18	2.21	3.26	4.69	6.01	7.38	8.90	10.38	11.91	13.40	14.97
45.27	0.61	1.71	2.95	4.18	5.55	6.90	8.24	9.69	11.12	12.68	14.08
47.81	0.43	1.50	2.82	4.06	5.33	6.81	8.22	9.71	11.28	12.62	14.12
50.55	0.97	2.08	3.27	4.36	5.65	7.14	8.61	9.98	11.47	12.97	14.32
53.51	0.86	1.89	3.09	4.44	5.73	7.07	8.57	9.98	11.42	12.86	14.28
56.69	1.15	2.25	3.49	4.84	6.10	7.50	9.02	10.49	11.98	13.50	14.99
60.13	1.06	2.09	3.36	4.58	5.90	7.23	8.71	10.18	11.68	13.10	14.54
63.84	0.94	2.12	3.37	4.56	5.97	7.39	8.77	10.21	11.78	13.23	14.80
67.83	0.86	1.92	3.19	4.48	5.81	7.26	8.73	10.24	11.73	13.27	14.82
72.15	1.07	2.19	3.46	4.72	6.02	7.46	8.82	10.30	11.84	13.32	14.79
76.8	1.20	2.29	3.36	4.72	6.10	7.49	8.92	10.34	11.87	13.33	14.81
81.81	1.43	2.61	3.83	5.10	6.45	7.81	9.31	10.80	12.26	13.86	15.38
87.22	1.44	2.57	3.76	5.05	6.31	7.69	9.32	10.79	12.26	13.84	15.28
93.06	1.60	2.86	4.02	5.28	6.70	8.14	9.69	11.19	12.72	14.31	15.78
99.35	1.82	2.99	4.17	5.62	6.90	8.36	9.98	11.39	12.99	14.57	16.15
106.13	1.83	3.01	4.25	5.56	6.83	8.34	9.81	11.36	12.81	14.39	15.80
113.45	2.02	3.09	4.36	5.68	7.11	8.52	10.06	11.64	13.11	14.65	16.22
121.34	1.72	2.82	4.14	5.34	6.70	8.13	9.69	11.23	12.69	14.25	15.78
129.86	1.73	2.83	4.10	5.45	6.90	8.31	9.82	11.33	12.95	14.53	16.08
139.04	2.57	3.85	5.07	6.48	7.85	9.33	10.84	12.38	13.96	15.52	17.13
148.94	2.51	3.78	5.07	6.46	7.90	9.38	10.84	12.43	14.05	15.69	17.16
159.62	2.66	3.78	5.10	6.54	7.89	9.29	10.82	12.45	13.91	15.55	17.14
171.14	2.77	3.83	5.14	6.62	7.95	9.47	10.98	12.45	14.10	15.67	17.19
183.56	2.55	3.73	4.95	6.43	7.81	9.26	10.74	12.30	13.92	15.46	17.05
196.96	2.88	4.03	5.40	6.83	8.24	9.80	11.34	12.94	14.55	16.20	17.76
211.41	3.17	4.47	5.79	7.27	8.61	10.24	11.79	13.39	15.01	16.62	18.28
226.99	3.49	4.74	6.22	7.65	9.07	10.65	12.23	13.85	15.57	17.19	18.75
243.8	3.41	4.67	6.00	7.46	8.93	10.46	12.06	13.65	15.25	16.83	18.39
261.93	3.81	5.05	6.35	7.78	9.23	10.85	12.39	14.08	15.77	17.46	19.04
281.48	4.11	5.35	6.70	8.17	9.57	11.22	12.86	14.45	16.07	17.71	19.42
302.56	4.34	5.72	6.95	8.46	10.00	11.50	13.17	14.88	16.55	18.14	19.83
325.3	4.37	5.68	7.08	8.56	10.09	11.63	13.29	14.94	16.63	18.21	19.90
349.83	4.49	5.67	6.93	8.47	9.99	11.50	13.17	14.70	16.32	17.97	19.58
376.28	4.94	6.05	7.49	9.08	10.56	12.12	13.82	15.54	17.18	18.85	20.49
404.81	5.05	6.34	7.69	9.26	10.75	12.28	14.05	15.61	17.31	18.95	20.56
435.58	5.18	6.42	7.76	9.43	10.90	12.56	14.23	15.84	17.59	19.29	20.94
468.76	5.71	7.01	8.44	10.01	11.53	13.22	14.91	16.66	18.23	19.95	21.70
504.55	5.70	7.15	8.61	10.21	11.74	13.35	15.11	16.67	18.38	20.09	21.77
543.15	5.92	7.32	8.74	10.27	11.86	13.46	15.11	16.84	18.62	20.26	21.97
584.78	6.40	7.70	9.08	10.67	12.26	13.84	15.61	17.26	18.97	20.70	22.47
629.68	6.51	7.83	9.30	10.85	12.42	14.09	15.79	17.54	19.23	20.95	22.71
678.1	6.90	8.36	9.65	11.26	12.84	14.44	16.28	17.85	19.60	21.33	23.05
730.33	6.88	8.24	9.68	11.30	12.87	14.47	16.21	17.88	19.63	21.38	23.06
786.65	6.77	8.13	9.74	11.19	12.74	14.46	16.08	17.91	19.48	21.29	22.93
847.4	7.13	8.57	10.07	11.64	13.30	14.91	16.65	18.41	20.17	21.97	23.63
912.92	7.10	8.54	9.98	11.58	13.19	14.88	16.51	18.26	19.97	21.63	23.41
983.58	7.30	8.60	10.07	11.65	13.19	14.86	16.59	18.30	20.01	21.73	23.33

1059.78	7.39	8.74	10.28	11.84	13.39	15.05	16.79	18.48	20.16	21.89	23.61
1141.98	7.56	8.94	10.32	11.90	13.39	15.11	16.78	18.38	20.11	21.84	23.43
1230.62	7.54	8.91	10.44	11.90	13.61	15.22	16.90	18.57	20.39	22.06	23.74
1326.23	7.70	9.18	10.66	12.29	13.89	15.52	17.27	19.02	20.81	22.57	24.25
1429.34	7.87	9.24	10.79	12.40	14.05	15.67	17.44	19.12	20.98	22.65	24.33
1540.54	7.63	9.01	10.50	12.16	13.68	15.41	17.17	18.80	20.62	22.32	24.04
1660.48	7.88	9.25	10.81	12.40	14.03	15.72	17.39	19.06	20.94	22.74	24.40
1789.83	7.66	9.11	10.58	12.25	13.85	15.63	17.25	19.05	20.79	22.60	24.32
1929.34	8.13	9.54	11.04	12.68	14.37	16.10	17.83	19.60	21.33	23.19	24.88
2079.8	7.43	8.77	10.26	11.83	13.43	15.15	16.83	18.66	20.34	22.06	23.82
2242.08	7.89	9.43	10.95	12.51	14.11	15.91	17.65	19.36	21.18	22.97	24.76
2417.1	7.81	9.27	10.72	12.24	14.00	15.67	17.36	19.21	20.90	22.71	24.39
2605.85	7.59	9.04	10.57	12.20	13.88	15.52	17.16	18.90	20.74	22.41	24.05
2809.43	7.80	9.05	10.56	12.19	13.85	15.45	17.23	18.93	20.72	22.43	24.21
3028.99	7.68	8.99	10.39	11.96	13.54	15.27	16.81	18.50	20.28	21.89	23.62
3265.79	7.64	8.90	10.48	11.97	13.59	15.27	17.03	18.59	20.40	22.11	23.73
3521.18	7.22	8.80	10.19	11.83	13.38	14.89	16.66	18.34	20.12	21.86	23.58
3796.62	7.75	9.11	10.52	12.18	13.79	15.47	17.20	18.83	20.59	22.33	23.99

Wavelength (nm) / Time (ps)	553.11	554.52	555.93	557.34	558.75	560.16	561.57	562.97	564.38	565.79	567.20
-3.28	0.22	0.22	0.22	0.21	0.19	0.18	0.21	0.13	0.18	0.12	0.15
-2.78	-0.01	0.05	0.12	0.09	0.04	0.05	0.10	0.07	0.04	0.08	0.03
-2.28	0.22	0.24	0.21	0.19	0.16	0.17	0.22	0.19	0.17	0.15	0.20
-1.78	0.05	0.03	0.02	0.07	0.05	0.08	0.02	0.07	0.14	0.12	0.13
-1.28	-0.12	-0.17	-0.12	-0.11	-0.07	-0.12	-0.07	-0.10	-0.11	-0.11	-0.09
-0.78	0.01	0.05	0.01	0.02	0.05	0.03	-0.02	0.01	0.04	-0.03	-0.04
-0.28	-0.59	-0.69	-0.69	-0.69	-0.64	-0.56	-0.63	-0.59	-0.60	-0.55	-0.56
0.22	0.22	0.27	0.23	0.21	0.21	0.16	0.18	0.24	0.14	0.23	0.16
0.32	0.22	0.26	0.23	0.24	0.22	0.17	0.28	0.27	0.29	0.27	0.37
0.42	-0.06	-0.04	-0.06	-0.11	-0.01	-0.05	-0.05	0.00	0.01	-0.01	0.04
0.52	-0.15	-0.16	-0.17	-0.16	-0.21	-0.20	-0.17	-0.22	-0.17	-0.16	-0.27
0.62	0.11	0.11	0.12	0.03	0.10	0.05	0.09	0.04	0.03	0.09	0.05
0.72	-0.18	-0.18	-0.22	-0.20	-0.23	-0.21	-0.19	-0.18	-0.16	-0.22	-0.18
0.77	0.18	0.23	0.11	0.09	0.19	0.21	0.11	0.23	0.23	0.19	0.13
0.82	-0.01	0.07	-0.01	0.08	-0.03	0.01	0.03	-0.02	0.02	0.03	0.00
0.87	-0.13	-0.15	-0.19	-0.18	-0.14	-0.24	-0.15	-0.20	-0.20	-0.21	-0.23
0.92	-0.09	-0.09	-0.08	-0.12	-0.07	-0.15	-0.11	-0.09	-0.14	-0.05	-0.09
0.97	0.06	0.08	0.15	0.09	0.14	0.19	0.05	0.11	0.14	0.10	0.12
1.02	0.30	0.22	0.25	0.29	0.26	0.22	0.22	0.22	0.21	0.23	0.28
1.07	-0.04	-0.03	0.02	-0.04	-0.03	-0.04	-0.02	-0.02	-0.03	0.00	0.03
1.12	-0.19	-0.18	-0.18	-0.13	-0.11	-0.11	-0.14	-0.12	-0.12	-0.14	-0.13
1.17	-0.02	-0.01	-0.07	0.02	-0.04	0.01	-0.03	-0.01	-0.02	-0.02	0.03
1.22	0.38	0.33	0.34	0.34	0.35	0.38	0.32	0.30	0.34	0.36	0.40
1.27	0.12	0.11	0.05	0.13	0.09	0.14	0.17	0.14	0.15	0.14	0.17
1.32	-0.14	-0.08	-0.19	-0.04	-0.16	-0.08	-0.09	-0.07	-0.12	-0.14	-0.09
1.37	0.01	-0.03	-0.12	-0.10	-0.05	-0.08	-0.05	-0.05	0.03	0.01	0.01
1.42	-0.27	-0.26	-0.20	-0.19	-0.15	-0.19	-0.10	-0.09	-0.04	0.00	-0.06

1.47	1.37	1.33	1.30	1.17	1.22	1.10	0.95	0.88	0.75	0.64	0.57
1.52	4.01	4.27	4.33	4.53	4.53	4.64	4.67	4.66	4.60	4.47	4.34
1.57	4.71	5.55	6.29	6.97	7.51	8.11	8.59	9.02	9.37	9.60	9.79
1.62	3.62	4.98	6.27	7.44	8.49	9.55	10.48	11.38	12.22	12.88	13.51
1.67	1.29	2.68	3.99	5.38	6.54	7.78	8.89	9.89	10.93	11.89	12.75
1.72	1.01	2.14	3.35	4.45	5.51	6.57	7.55	8.52	9.42	10.21	10.98
1.77	1.06	2.18	3.27	4.26	5.21	6.19	7.05	7.89	8.66	9.37	10.04
1.82	0.89	1.97	3.03	4.00	4.92	5.75	6.59	7.33	8.05	8.75	9.30
1.87	1.32	2.45	3.35	4.25	5.06	5.95	6.63	7.33	8.01	8.65	9.11
1.92	2.68	3.62	4.59	5.50	6.31	7.02	7.72	8.43	9.09	9.58	10.16
1.97	3.44	4.44	5.39	6.24	7.06	7.79	8.47	9.14	9.67	10.21	10.55
2.02	4.89	5.72	6.66	7.56	8.31	9.05	9.69	10.32	10.82	11.34	11.69
2.07	5.76	6.80	7.68	8.53	9.35	10.00	10.78	11.35	11.87	12.33	12.75
2.12	5.54	6.54	7.38	8.18	8.97	9.71	10.37	10.92	11.43	11.84	12.25
2.17	6.04	7.04	7.97	8.77	9.52	10.28	10.94	11.45	12.00	12.45	12.82
2.22	6.59	7.49	8.41	9.33	10.07	10.84	11.42	12.09	12.59	12.99	13.36
2.27	7.54	8.51	9.41	10.28	11.04	11.80	12.41	13.01	13.48	13.87	14.25
2.32	8.22	9.23	10.20	11.09	11.82	12.65	13.21	13.82	14.27	14.69	15.01
2.37	8.35	9.33	10.30	11.17	11.94	12.68	13.33	13.91	14.39	14.83	15.07
2.42	8.53	9.54	10.42	11.34	12.04	12.86	13.55	14.09	14.65	15.00	15.34
2.47	8.77	9.82	10.75	11.63	12.46	13.24	13.86	14.51	15.04	15.45	15.73
2.52	8.83	9.93	10.88	11.75	12.55	13.25	13.95	14.59	15.04	15.41	15.72
2.57	9.43	10.41	11.45	12.33	13.14	13.91	14.61	15.15	15.65	16.13	16.49
2.62	9.78	10.74	11.80	12.65	13.42	14.31	14.98	15.56	16.07	16.42	16.90
2.67	9.76	10.81	11.72	12.65	13.45	14.15	14.94	15.51	15.98	16.38	16.71
2.72	9.84	10.82	11.76	12.68	13.58	14.28	14.97	15.51	16.03	16.43	16.66
2.77	9.91	10.93	11.94	12.84	13.65	14.52	15.16	15.76	16.31	16.78	16.98
2.82	10.83	11.92	12.94	13.80	14.64	15.47	16.20	16.72	17.26	17.68	17.93
2.87	10.56	11.66	12.69	13.59	14.41	15.15	15.86	16.43	16.93	17.39	17.78
2.92	11.30	12.45	13.47	14.37	15.15	15.97	16.64	17.27	17.77	18.18	18.49
2.97	11.05	12.15	13.25	14.13	15.11	15.78	16.53	17.11	17.73	18.12	18.39
3.02	10.95	12.01	13.08	14.00	14.98	15.70	16.38	16.89	17.52	17.92	18.25
3.07	11.21	12.30	13.25	14.23	15.08	15.85	16.59	17.19	17.71	18.06	18.45
3.12	12.22	13.35	14.44	15.41	16.30	17.18	17.83	18.44	19.06	19.40	19.78
3.17	11.77	12.98	13.94	14.99	15.71	16.58	17.27	17.93	18.52	18.84	19.17
3.22	11.72	12.85	13.90	14.83	15.75	16.54	17.24	17.88	18.47	18.82	19.15
3.27	11.54	12.70	13.64	14.67	15.55	16.31	17.01	17.69	18.24	18.70	18.99
3.32	12.37	13.52	14.61	15.57	16.43	17.31	18.05	18.75	19.21	19.73	20.05
3.37	12.34	13.43	14.44	15.41	16.32	17.17	17.85	18.46	19.01	19.41	19.78
3.42	12.13	13.31	14.42	15.28	16.21	17.03	17.75	18.39	18.92	19.33	19.70
3.47	12.63	13.75	14.78	15.70	16.69	17.37	18.15	18.77	19.31	19.77	20.17
3.52	12.36	13.48	14.62	15.65	16.55	17.34	18.14	18.80	19.39	19.80	20.17
3.57	12.27	13.42	14.54	15.53	16.40	17.20	17.93	18.58	19.09	19.62	19.97
3.62	12.68	13.75	14.85	15.85	16.74	17.60	18.35	19.03	19.61	20.12	20.41
3.67	12.72	13.86	14.99	15.95	16.95	17.73	18.42	19.12	19.72	20.19	20.50
3.72	13.59	14.69	15.92	16.76	17.69	18.50	19.26	20.03	20.57	21.10	21.36
3.92	12.85	13.99	15.11	15.97	16.91	17.79	18.43	19.08	19.68	20.11	20.55
4.12	13.33	14.44	15.55	16.56	17.55	18.39	19.18	19.88	20.46	20.99	21.33
4.32	13.51	14.72	15.83	16.93	17.89	18.87	19.61	20.39	20.88	21.41	21.79
4.52	14.21	15.35	16.50	17.59	18.52	19.45	20.18	20.90	21.54	22.06	22.42

4.72	14.25	15.34	16.51	17.55	18.55	19.41	20.25	20.89	21.51	22.15	22.52
4.92	14.05	15.25	16.42	17.44	18.40	19.38	20.12	20.91	21.44	21.98	22.38
5.12	14.15	15.32	16.46	17.54	18.59	19.58	20.35	21.12	21.74	22.27	22.69
5.32	14.28	15.50	16.70	17.83	18.83	19.80	20.66	21.39	22.10	22.59	22.92
5.52	14.23	15.41	16.58	17.66	18.66	19.60	20.49	21.17	21.82	22.34	22.79
5.72	14.27	15.50	16.72	17.81	18.83	19.73	20.57	21.31	21.98	22.46	22.89
5.92	14.66	15.85	17.08	18.15	19.14	20.07	20.88	21.63	22.34	22.86	23.30
6.12	14.26	15.56	16.71	17.78	18.79	19.67	20.56	21.35	21.93	22.50	22.89
6.32	14.24	15.38	16.59	17.68	18.70	19.70	20.43	21.22	21.87	22.38	22.81
6.52	14.59	15.75	17.01	18.16	19.13	20.19	20.92	21.69	22.43	22.99	23.37
6.72	14.57	15.84	17.15	18.23	19.20	20.15	21.01	21.73	22.44	22.94	23.34
6.92	14.38	15.58	16.80	17.95	18.90	19.79	20.65	21.42	22.09	22.63	23.06
7.12	14.70	15.98	17.17	18.26	19.21	20.14	20.97	21.73	22.37	22.91	23.36
7.32	14.85	15.99	17.27	18.35	19.34	20.30	21.18	21.96	22.63	23.20	23.57
7.52	14.57	15.71	16.96	18.12	19.12	20.06	20.94	21.71	22.40	22.89	23.39
7.72	14.30	15.48	16.70	17.87	18.86	19.84	20.63	21.52	22.22	22.72	23.14
7.92	14.82	16.10	17.31	18.51	19.50	20.44	21.39	22.08	22.80	23.35	23.67
8.12	14.68	15.91	17.07	18.25	19.25	20.24	21.04	21.78	22.48	22.98	23.45
8.32	14.83	16.09	17.30	18.42	19.44	20.38	21.30	22.03	22.71	23.26	23.68
8.52	14.58	15.94	17.12	18.27	19.30	20.27	21.14	21.92	22.59	23.10	23.63
8.72	14.61	15.86	17.05	18.17	19.22	20.22	21.09	21.86	22.60	23.15	23.56
8.92	14.60	15.85	17.10	18.18	19.32	20.23	21.07	21.91	22.60	23.13	23.62
9.12	14.82	16.10	17.32	18.49	19.52	20.45	21.39	22.20	22.89	23.42	23.89
9.32	14.75	15.99	17.26	18.35	19.45	20.41	21.30	22.08	22.77	23.34	23.79
9.52	15.09	16.41	17.64	18.80	19.86	20.83	21.79	22.60	23.29	23.90	24.29
9.72	14.91	16.22	17.46	18.63	19.75	20.69	21.59	22.42	23.10	23.69	24.10
9.92	14.73	15.94	17.34	18.53	19.55	20.55	21.44	22.29	22.88	23.49	23.97
10.12	14.92	16.14	17.37	18.60	19.58	20.46	21.31	22.17	22.85	23.32	23.88
10.32	14.77	16.01	17.26	18.38	19.46	20.36	21.25	22.03	22.67	23.30	23.76
10.52	14.72	16.08	17.27	18.41	19.50	20.36	21.25	22.00	22.72	23.27	23.66
10.72	14.40	15.76	16.87	18.06	19.12	20.05	20.88	21.68	22.36	22.91	23.33
10.92	14.67	16.00	17.26	18.34	19.46	20.46	21.36	22.15	22.88	23.46	23.89
11.12	14.58	15.92	17.21	18.33	19.33	20.36	21.22	22.01	22.73	23.27	23.74
11.32	14.73	16.05	17.23	18.43	19.41	20.46	21.29	22.08	22.79	23.38	23.77
11.52	14.93	16.15	17.41	18.52	19.57	20.53	21.32	22.21	22.83	23.38	23.83
11.72	14.58	15.86	17.11	18.16	19.19	20.17	21.05	21.78	22.50	23.07	23.63
11.92	14.61	15.71	16.75	17.94	18.95	19.86	20.81	21.46	22.24	22.73	23.16
12.12	14.56	15.89	17.05	18.12	19.22	20.18	20.99	21.81	22.45	22.95	23.40
12.32	14.83	16.09	17.30	18.46	19.47	20.49	21.33	22.11	22.76	23.35	23.69
12.52	15.25	16.59	17.83	18.99	20.02	21.02	21.97	22.71	23.41	24.08	24.45
12.72	14.85	16.18	17.42	18.57	19.69	20.65	21.53	22.33	23.07	23.56	24.08
12.92	14.72	16.08	17.35	18.43	19.52	20.49	21.36	22.14	22.89	23.44	23.86
13.12	14.87	16.14	17.38	18.57	19.62	20.66	21.47	22.27	22.92	23.52	24.01
13.32	14.70	16.04	17.35	18.44	19.55	20.58	21.50	22.30	22.91	23.55	24.03
13.52	15.00	16.28	17.45	18.71	19.79	20.77	21.63	22.36	23.19	23.72	24.26
13.72	15.23	16.58	17.80	18.96	19.99	20.97	21.86	22.72	23.41	23.97	24.47
13.92	15.18	16.52	17.70	18.83	19.97	20.95	21.91	22.64	23.36	23.97	24.43
14	14.52	15.84	17.07	18.23	19.25	20.28	21.14	21.89	22.58	23.12	23.62
14.08	14.62	15.90	17.11	18.27	19.34	20.35	21.23	22.01	22.71	23.30	23.70
14.17	14.81	16.04	17.33	18.44	19.43	20.37	21.27	22.15	22.72	23.31	23.75

14.27	14.86	16.17	17.49	18.56	19.67	20.69	21.48	22.30	23.07	23.59	24.05
14.38	14.96	16.22	17.43	18.62	19.71	20.69	21.58	22.36	23.01	23.63	24.10
14.49	14.53	15.74	17.03	18.17	19.17	20.19	21.04	21.88	22.51	23.13	23.56
14.61	14.76	16.08	17.26	18.46	19.61	20.59	21.50	22.35	22.94	23.54	24.01
14.75	14.92	16.23	17.51	18.61	19.67	20.71	21.57	22.39	23.11	23.64	24.08
14.89	14.81	16.14	17.47	18.57	19.69	20.64	21.49	22.27	23.03	23.66	24.04
15.05	15.25	16.57	17.95	19.10	20.17	21.16	22.00	22.83	23.56	24.13	24.58
15.21	14.88	16.18	17.47	18.61	19.69	20.64	21.52	22.34	23.03	23.65	24.07
15.39	14.74	16.07	17.21	18.33	19.50	20.46	21.31	22.13	22.81	23.42	23.85
15.59	14.85	16.02	17.34	18.50	19.61	20.55	21.50	22.26	22.97	23.56	24.02
15.8	14.42	15.68	16.95	18.03	19.17	20.04	21.04	21.79	22.40	23.02	23.44
16.02	14.77	16.13	17.32	18.41	19.49	20.41	21.29	22.12	22.79	23.32	23.82
16.27	15.28	16.61	17.85	19.05	20.16	21.14	21.98	22.89	23.59	24.25	24.59
16.53	15.13	16.35	17.61	18.74	19.74	20.78	21.66	22.50	23.25	23.83	24.22
16.81	14.98	16.18	17.51	18.70	19.78	20.78	21.68	22.47	23.17	23.75	24.16
17.12	14.71	16.06	17.27	18.37	19.49	20.46	21.41	22.15	22.87	23.44	23.93
17.45	15.06	16.37	17.57	18.75	19.79	20.81	21.68	22.47	23.20	23.85	24.20
17.81	15.22	16.49	17.73	18.93	19.95	21.00	21.90	22.74	23.41	24.02	24.42
18.19	15.20	16.45	17.72	18.97	19.93	20.94	21.90	22.71	23.45	24.01	24.52
18.6	14.73	16.07	17.36	18.36	19.47	20.44	21.35	22.15	22.82	23.39	23.82
19.05	14.78	16.12	17.37	18.56	19.68	20.69	21.54	22.37	23.06	23.70	24.18
19.53	15.24	16.68	17.93	19.06	20.10	21.13	22.08	22.91	23.55	24.23	24.63
20.05	14.79	16.07	17.33	18.51	19.54	20.56	21.39	22.22	22.87	23.49	23.90
20.61	15.14	16.44	17.73	18.88	20.04	20.98	21.87	22.71	23.42	24.02	24.51
21.22	14.85	16.19	17.42	18.50	19.61	20.72	21.62	22.43	23.06	23.67	24.07
21.87	14.93	16.22	17.47	18.64	19.79	20.65	21.62	22.45	23.15	23.72	24.14
22.57	15.04	16.41	17.68	18.85	19.98	21.01	21.86	22.66	23.38	24.08	24.49
23.33	15.09	16.37	17.57	18.68	19.80	20.75	21.73	22.50	23.21	23.78	24.22
24.15	15.44	16.70	17.90	19.12	20.16	21.21	22.07	22.80	23.63	24.20	24.69
25.03	15.42	16.69	17.96	19.10	20.27	21.21	22.19	22.98	23.63	24.22	24.74
25.98	14.62	15.92	17.19	18.32	19.42	20.44	21.38	22.22	22.94	23.56	23.99
27.01	14.67	16.04	17.24	18.39	19.59	20.57	21.45	22.30	23.06	23.60	24.13
28.11	15.06	16.30	17.58	18.79	19.83	20.83	21.76	22.50	23.29	23.83	24.34
29.3	15.22	16.57	17.82	19.01	20.12	21.12	22.04	22.85	23.59	24.16	24.63
30.59	14.86	16.17	17.46	18.52	19.65	20.64	21.55	22.31	22.99	23.59	24.04
31.98	15.25	16.61	17.86	19.06	20.12	21.19	22.12	22.89	23.60	24.22	24.73
33.47	15.30	16.65	17.92	19.07	20.17	21.14	22.10	22.95	23.71	24.26	24.76
35.09	15.43	16.80	18.04	19.18	20.32	21.28	22.23	23.09	23.82	24.44	24.93
36.83	15.57	16.91	18.15	19.37	20.46	21.53	22.49	23.30	24.08	24.68	25.15
38.71	15.55	16.93	18.25	19.40	20.51	21.52	22.40	23.30	24.02	24.68	25.18
40.73	15.71	17.03	18.22	19.51	20.60	21.62	22.50	23.36	24.16	24.74	25.31
42.92	16.29	17.70	19.07	20.19	21.30	22.47	23.39	24.31	25.08	25.77	26.26
45.27	15.44	16.73	18.03	19.22	20.41	21.50	22.46	23.31	24.04	24.73	25.19
47.81	15.51	16.95	18.23	19.54	20.65	21.69	22.63	23.53	24.30	24.94	25.41
50.55	15.71	17.04	18.35	19.55	20.66	21.75	22.60	23.54	24.19	24.89	25.35
53.51	15.75	17.06	18.34	19.54	20.68	21.70	22.65	23.56	24.18	24.85	25.42
56.69	16.38	17.80	19.06	20.30	21.50	22.56	23.50	24.38	25.13	25.75	26.29
60.13	15.89	17.34	18.62	19.87	20.92	21.96	22.97	23.82	24.62	25.24	25.71
63.84	16.07	17.47	18.83	19.97	21.20	22.31	23.28	24.11	24.92	25.50	26.05
67.83	16.20	17.56	18.95	20.18	21.38	22.44	23.38	24.24	25.08	25.76	26.29

72.15	16.25	17.52	18.96	20.14	21.32	22.42	23.33	24.24	25.01	25.60	26.13
76.8	16.25	17.56	18.89	20.08	21.24	22.35	23.32	24.18	24.98	25.62	26.14
81.81	16.68	18.11	19.43	20.59	21.77	22.86	23.82	24.76	25.50	26.15	26.72
87.22	16.65	17.98	19.36	20.58	21.78	22.91	23.89	24.77	25.59	26.22	26.84
93.06	17.22	18.60	19.99	21.28	22.48	23.64	24.56	25.54	26.30	27.02	27.53
99.35	17.67	19.05	20.44	21.73	22.95	24.10	25.12	26.03	26.85	27.55	28.18
106.13	17.32	18.65	20.03	21.22	22.47	23.56	24.56	25.39	26.20	26.93	27.47
113.45	17.62	19.05	20.43	21.73	22.91	24.05	25.04	26.00	26.82	27.52	28.12
121.34	17.18	18.57	19.94	21.19	22.34	23.42	24.38	25.35	26.21	26.88	27.52
129.86	17.51	19.01	20.33	21.65	22.84	24.01	25.02	26.00	26.81	27.51	28.07
139.04	18.62	20.10	21.45	22.75	23.92	25.07	26.06	27.08	27.90	28.59	29.25
148.94	18.74	20.21	21.61	22.96	24.18	25.33	26.39	27.30	28.16	28.92	29.51
159.62	18.58	20.01	21.38	22.63	23.87	24.97	26.03	26.97	27.81	28.59	29.19
171.14	18.69	20.12	21.46	22.70	23.97	25.18	26.23	27.14	28.03	28.75	29.41
183.56	18.47	19.94	21.30	22.63	23.86	25.04	26.08	27.08	27.98	28.70	29.30
196.96	19.29	20.78	22.18	23.59	24.84	26.02	27.08	28.10	29.01	29.74	30.38
211.41	19.72	21.21	22.68	23.99	25.31	26.45	27.50	28.54	29.49	30.17	30.84
226.99	20.41	21.96	23.32	24.72	26.05	27.24	28.39	29.50	30.29	31.16	31.87
243.8	20.00	21.50	22.91	24.35	25.59	26.81	27.84	28.85	29.79	30.53	31.26
261.93	20.62	22.09	23.57	24.98	26.19	27.48	28.57	29.64	30.53	31.37	32.03
281.48	20.87	22.45	23.87	25.28	26.63	27.85	28.91	30.01	30.89	31.76	32.43
302.56	21.45	23.01	24.46	25.82	27.18	28.46	29.56	30.55	31.58	32.38	33.04
325.3	21.44	23.09	24.50	25.85	27.23	28.45	29.52	30.63	31.55	32.37	33.13
349.83	21.12	22.67	24.15	25.55	26.78	28.00	29.08	30.11	31.04	31.84	32.63
376.28	22.08	23.72	25.13	26.59	27.93	29.18	30.37	31.41	32.42	33.24	33.98
404.81	22.10	23.61	25.22	26.54	27.98	29.20	30.41	31.45	32.34	33.15	33.91
435.58	22.55	24.16	25.68	27.07	28.46	29.79	30.94	31.98	32.95	33.84	34.59
468.76	23.24	24.89	26.42	27.88	29.19	30.53	31.69	32.79	33.76	34.63	35.44
504.55	23.38	25.00	26.57	27.98	29.38	30.68	31.77	32.96	33.90	34.76	35.55
543.15	23.58	25.16	26.59	28.09	29.55	30.76	31.99	33.04	34.06	34.95	35.68
584.78	24.02	25.62	27.09	28.51	29.92	31.21	32.41	33.59	34.59	35.45	36.26
629.68	24.33	25.97	27.48	29.01	30.36	31.63	32.86	34.02	35.08	35.99	36.80
678.1	24.63	26.14	27.76	29.20	30.58	31.89	33.02	34.12	35.19	36.06	36.86
730.33	24.79	26.39	27.96	29.44	30.79	32.06	33.27	34.42	35.48	36.40	37.19
786.65	24.73	26.13	27.74	29.22	30.60	31.92	33.14	34.30	35.29	36.24	37.00
847.4	25.33	26.93	28.57	30.02	31.46	32.72	34.03	35.09	36.17	37.10	37.93
912.92	24.99	26.61	28.15	29.64	31.12	32.42	33.63	34.68	35.75	36.71	37.44
983.58	24.98	26.61	28.04	29.59	30.97	32.30	33.45	34.65	35.65	36.53	37.35
1059.78	25.21	26.81	28.32	29.80	31.19	32.50	33.72	34.86	35.88	36.83	37.69
1141.98	25.07	26.61	28.13	29.61	30.95	32.27	33.42	34.54	35.52	36.46	37.28
1230.62	25.41	26.92	28.55	29.91	31.37	32.63	33.90	35.04	36.04	36.97	37.76
1326.23	25.92	27.48	29.08	30.50	31.98	33.25	34.53	35.68	36.71	37.65	38.48
1429.34	25.99	27.57	29.16	30.69	32.13	33.44	34.62	35.81	36.88	37.76	38.68
1540.54	25.75	27.40	28.94	30.48	31.87	33.23	34.46	35.64	36.68	37.54	38.44
1660.48	26.07	27.77	29.29	30.75	32.12	33.53	34.73	35.91	37.01	37.87	38.77
1789.83	26.07	27.58	29.14	30.71	32.12	33.49	34.67	35.83	36.92	37.82	38.68
1929.34	26.59	28.24	29.83	31.42	32.74	34.19	35.41	36.61	37.68	38.60	39.44
2079.8	25.45	27.11	28.55	30.09	31.50	32.88	34.08	35.25	36.29	37.15	38.12
2242.08	26.28	28.04	29.58	31.11	32.54	33.95	35.17	36.33	37.38	38.36	39.27
2417.1	26.07	27.76	29.21	30.73	32.17	33.43	34.73	35.88	36.95	37.81	38.66

2605.85	25.75	27.40	29.02	30.43	31.87	33.15	34.38	35.56	36.61	37.56	38.43
2809.43	25.82	27.42	28.93	30.44	31.85	33.10	34.43	35.62	36.63	37.56	38.42
3028.99	25.17	26.76	28.29	29.75	31.05	32.41	33.55	34.61	35.70	36.60	37.40
3265.79	25.35	26.84	28.41	29.91	31.17	32.53	33.78	34.90	35.93	36.83	37.66
3521.18	25.21	26.82	28.38	29.83	31.19	32.57	33.68	34.84	35.89	36.82	37.66
3796.62	25.64	27.29	28.79	30.30	31.64	32.95	34.14	35.32	36.37	37.32	38.11

Wavelength (nm) / Time (ps)	568.61	570.02	571.43	572.83	574.24	575.65	577.06	578.47	579.88	581.29	582.69
-3.28	0.14	0.20	0.18	0.17	0.16	0.18	0.13	0.12	0.16	0.15	0.15
-2.78	0.03	0.02	-0.01	0.05	0.06	0.08	0.00	0.08	0.00	0.02	-0.01
-2.28	0.15	0.16	0.16	0.15	0.21	0.13	0.16	0.13	0.09	0.07	0.13
-1.78	0.14	0.08	0.10	0.12	0.09	0.14	0.13	0.15	0.12	0.14	0.07
-1.28	-0.09	-0.15	-0.10	-0.08	-0.07	-0.06	-0.06	-0.03	0.00	0.00	0.04
-0.78	-0.02	0.03	0.01	0.00	-0.06	-0.03	0.01	-0.05	-0.02	-0.03	-0.04
-0.28	-0.58	-0.59	-0.54	-0.58	-0.58	-0.58	-0.56	-0.55	-0.51	-0.44	-0.46
0.22	0.21	0.27	0.20	0.18	0.19	0.14	0.18	0.15	0.16	0.09	0.12
0.32	0.29	0.30	0.32	0.34	0.37	0.30	0.29	0.31	0.30	0.28	0.33
0.42	0.03	0.00	0.05	0.07	0.06	0.12	0.15	0.16	0.08	0.12	0.12
0.52	-0.14	-0.20	-0.21	-0.16	-0.20	-0.17	-0.17	-0.10	-0.13	-0.16	-0.12
0.62	0.06	-0.02	0.04	0.02	0.06	0.10	0.07	0.04	-0.02	0.00	0.02
0.72	-0.21	-0.13	-0.14	-0.09	-0.11	-0.07	-0.09	-0.04	-0.10	-0.12	-0.09
0.77	0.24	0.22	0.16	0.20	0.23	0.27	0.27	0.21	0.21	0.21	0.29
0.82	-0.06	-0.05	-0.06	-0.05	-0.07	-0.01	-0.10	-0.10	-0.10	-0.10	-0.11
0.87	-0.26	-0.21	-0.26	-0.23	-0.20	-0.19	-0.21	-0.23	-0.29	-0.26	-0.21
0.92	-0.03	-0.13	-0.09	-0.11	-0.13	-0.09	-0.15	-0.05	-0.02	-0.05	-0.03
0.97	0.10	0.14	0.15	0.13	0.14	0.21	0.17	0.20	0.19	0.17	0.20
1.02	0.32	0.28	0.29	0.27	0.31	0.25	0.30	0.22	0.21	0.22	0.23
1.07	0.02	0.05	0.04	0.05	0.01	0.02	0.05	0.03	0.06	0.02	0.07
1.12	-0.21	-0.13	-0.12	-0.20	-0.15	-0.14	-0.17	-0.18	-0.15	-0.15	-0.17
1.17	0.02	-0.03	0.02	0.03	0.11	0.09	0.11	0.12	0.06	0.07	0.06
1.22	0.38	0.43	0.37	0.37	0.33	0.37	0.42	0.29	0.35	0.28	0.35
1.27	0.13	0.15	0.10	0.14	0.18	0.21	0.20	0.18	0.14	0.18	0.19
1.32	-0.03	-0.05	-0.10	-0.07	-0.10	-0.03	-0.06	-0.03	-0.07	-0.08	-0.09
1.37	-0.01	0.02	0.02	-0.01	0.05	0.02	0.03	0.01	0.01	-0.03	0.02
1.42	-0.06	0.00	0.00	-0.06	-0.01	0.13	0.10	0.08	0.14	0.11	0.16
1.47	0.46	0.36	0.23	0.17	0.19	0.15	0.12	0.11	0.15	0.09	0.08
1.52	4.12	3.90	3.66	3.44	3.09	2.86	2.61	2.38	2.20	1.96	1.86
1.57	9.81	9.71	9.59	9.43	9.19	8.93	8.66	8.35	8.11	7.81	7.56
1.62	14.09	14.57	14.89	15.21	15.37	15.44	15.50	15.54	15.52	15.47	15.39
1.67	13.55	14.35	15.06	15.67	16.29	16.74	17.15	17.62	17.87	18.10	18.46
1.72	11.72	12.39	12.94	13.56	14.13	14.56	14.99	15.29	15.66	15.97	16.27
1.77	10.65	11.27	11.70	12.25	12.65	13.05	13.37	13.67	13.99	14.14	14.40
1.82	9.82	10.28	10.70	11.15	11.49	11.79	12.15	12.35	12.63	12.81	12.97
1.87	9.64	10.08	10.38	10.72	10.97	11.32	11.62	11.75	12.01	12.07	12.20
1.92	10.49	10.92	11.19	11.51	11.78	12.00	12.18	12.39	12.42	12.50	12.55
1.97	10.95	11.27	11.51	11.80	12.03	12.25	12.35	12.48	12.56	12.63	12.73
2.02	12.10	12.35	12.58	12.78	12.98	13.07	13.16	13.28	13.30	13.27	13.33

2.07	13.06	13.30	13.47	13.72	13.81	13.93	14.03	14.08	14.08	14.00	14.02
2.12	12.52	12.87	13.02	13.18	13.30	13.45	13.47	13.57	13.57	13.53	13.49
2.17	13.14	13.43	13.63	13.73	13.88	14.00	13.97	13.99	13.95	13.92	13.88
2.22	13.64	13.87	14.09	14.18	14.35	14.50	14.46	14.41	14.48	14.38	14.40
2.27	14.57	14.70	14.93	15.03	15.03	15.12	15.15	15.00	15.04	14.87	14.81
2.32	15.30	15.55	15.68	15.80	15.92	15.91	15.90	15.78	15.86	15.70	15.65
2.37	15.39	15.61	15.77	15.87	15.97	15.91	15.88	15.86	15.77	15.61	15.51
2.42	15.59	15.80	15.91	16.06	16.05	16.12	16.01	15.93	15.90	15.73	15.61
2.47	15.99	16.26	16.36	16.47	16.46	16.44	16.44	16.37	16.25	16.10	15.98
2.52	16.01	16.20	16.25	16.37	16.43	16.40	16.40	16.29	16.18	16.07	15.94
2.57	16.68	16.86	16.98	17.00	17.09	17.13	16.96	16.93	16.84	16.68	16.51
2.62	17.12	17.30	17.34	17.41	17.39	17.46	17.40	17.26	17.15	16.99	16.87
2.67	16.95	17.13	17.20	17.29	17.31	17.26	17.18	17.11	16.96	16.74	16.53
2.72	16.93	17.16	17.20	17.36	17.30	17.28	17.17	17.12	16.93	16.74	16.56
2.77	17.28	17.47	17.54	17.65	17.62	17.59	17.52	17.47	17.29	17.12	16.99
2.82	18.22	18.36	18.49	18.50	18.49	18.45	18.34	18.16	18.00	17.80	17.63
2.87	17.92	18.13	18.22	18.32	18.25	18.29	18.17	18.02	17.92	17.69	17.50
2.92	18.74	18.91	18.97	19.05	19.02	18.97	18.86	18.72	18.52	18.33	18.04
2.97	18.73	18.93	18.99	19.08	19.12	19.06	18.96	18.86	18.64	18.39	18.20
3.02	18.51	18.73	18.71	18.85	18.81	18.75	18.66	18.49	18.32	18.10	17.85
3.07	18.69	18.85	18.85	19.01	19.00	18.90	18.77	18.63	18.44	18.25	18.00
3.12	20.03	20.24	20.32	20.31	20.38	20.21	20.14	19.99	19.77	19.54	19.28
3.17	19.51	19.58	19.67	19.71	19.69	19.66	19.52	19.41	19.27	19.02	18.68
3.22	19.40	19.63	19.66	19.69	19.73	19.63	19.52	19.35	19.16	18.82	18.63
3.27	19.18	19.37	19.42	19.46	19.47	19.44	19.24	19.08	18.88	18.68	18.48
3.32	20.30	20.47	20.53	20.59	20.59	20.46	20.28	20.09	19.89	19.63	19.37
3.37	20.07	20.20	20.27	20.31	20.31	20.21	20.10	19.98	19.72	19.45	19.22
3.42	19.92	20.12	20.17	20.21	20.23	20.11	19.95	19.80	19.68	19.38	19.17
3.47	20.31	20.54	20.60	20.61	20.55	20.50	20.37	20.13	19.88	19.65	19.38
3.52	20.34	20.60	20.66	20.74	20.57	20.61	20.45	20.31	20.15	19.82	19.59
3.57	20.19	20.32	20.39	20.48	20.45	20.39	20.28	20.01	19.88	19.59	19.28
3.62	20.71	20.77	20.96	21.01	20.99	20.89	20.80	20.57	20.39	20.17	19.89
3.67	20.78	20.98	21.07	21.15	21.14	21.08	20.92	20.76	20.54	20.32	20.06
3.72	21.70	21.87	21.94	22.03	21.98	21.89	21.70	21.55	21.30	21.02	20.76
3.92	20.71	20.91	21.00	21.01	21.04	20.97	20.83	20.65	20.46	20.12	19.87
4.12	21.59	21.79	21.91	21.98	21.96	21.83	21.75	21.53	21.29	21.06	20.78
4.32	22.04	22.27	22.37	22.47	22.43	22.33	22.19	21.99	21.79	21.43	21.18
4.52	22.79	22.94	23.05	23.16	23.08	23.09	22.97	22.73	22.47	22.15	21.88
4.72	22.75	22.98	23.09	23.20	23.15	23.07	23.00	22.77	22.57	22.28	21.98
4.92	22.65	22.95	22.98	23.11	23.07	23.00	22.88	22.64	22.40	22.15	21.87
5.12	22.95	23.20	23.35	23.41	23.42	23.36	23.17	23.03	22.85	22.47	22.17
5.32	23.32	23.60	23.68	23.76	23.69	23.68	23.53	23.34	23.11	22.73	22.47
5.52	23.06	23.29	23.48	23.56	23.51	23.44	23.30	23.17	22.86	22.61	22.35
5.72	23.24	23.46	23.58	23.71	23.74	23.69	23.51	23.31	23.09	22.78	22.46
5.92	23.62	23.83	23.93	24.05	24.00	23.92	23.82	23.64	23.36	23.05	22.83
6.12	23.33	23.50	23.56	23.66	23.64	23.63	23.48	23.25	23.10	22.66	22.42
6.32	23.12	23.40	23.53	23.58	23.68	23.54	23.49	23.27	23.05	22.76	22.44
6.52	23.71	23.88	24.08	24.16	24.10	24.08	23.94	23.76	23.42	23.12	22.82
6.72	23.68	23.95	24.14	24.19	24.28	24.20	24.06	23.89	23.64	23.38	23.10
6.92	23.41	23.61	23.68	23.81	23.76	23.78	23.62	23.48	23.18	22.98	22.61

7.12	23.60	23.87	23.95	24.02	23.96	23.88	23.75	23.54	23.31	23.00	22.69
7.32	23.94	24.19	24.36	24.44	24.40	24.43	24.30	24.06	23.87	23.55	23.22
7.52	23.70	23.88	24.00	24.11	24.10	24.05	23.92	23.68	23.45	23.07	22.81
7.72	23.41	23.71	23.81	23.91	23.91	23.93	23.76	23.55	23.33	23.02	22.71
7.92	24.09	24.27	24.41	24.50	24.50	24.47	24.38	24.11	23.95	23.62	23.27
8.12	23.75	24.05	24.13	24.22	24.24	24.15	24.06	23.80	23.56	23.29	23.02
8.32	24.06	24.28	24.37	24.47	24.48	24.42	24.23	24.05	23.77	23.49	23.14
8.52	23.86	24.21	24.29	24.37	24.34	24.29	24.17	23.98	23.68	23.39	23.10
8.72	23.95	24.14	24.25	24.34	24.36	24.25	24.15	23.95	23.66	23.43	23.09
8.92	23.92	24.23	24.33	24.43	24.48	24.44	24.34	24.09	23.84	23.58	23.27
9.12	24.19	24.43	24.54	24.67	24.68	24.55	24.46	24.30	24.14	23.78	23.48
9.32	24.17	24.42	24.50	24.65	24.76	24.61	24.54	24.37	24.09	23.73	23.45
9.52	24.66	24.90	25.02	25.13	25.05	25.06	24.97	24.79	24.55	24.20	23.90
9.72	24.47	24.64	24.83	25.01	24.87	24.91	24.76	24.53	24.25	23.91	23.64
9.92	24.32	24.56	24.73	24.82	24.83	24.74	24.63	24.44	24.23	23.88	23.59
10.12	24.20	24.44	24.59	24.64	24.70	24.65	24.47	24.24	23.96	23.67	23.37
10.32	24.08	24.32	24.42	24.57	24.58	24.53	24.46	24.20	24.04	23.73	23.41
10.52	24.08	24.29	24.45	24.55	24.51	24.48	24.31	24.16	23.94	23.61	23.24
10.72	23.72	23.98	24.10	24.17	24.20	24.15	23.96	23.81	23.56	23.26	22.99
10.92	24.23	24.55	24.65	24.78	24.79	24.73	24.60	24.43	24.19	23.79	23.53
11.12	24.03	24.32	24.48	24.58	24.58	24.59	24.42	24.27	23.99	23.64	23.34
11.32	24.12	24.39	24.49	24.58	24.56	24.48	24.36	24.18	23.95	23.64	23.40
11.52	24.18	24.40	24.53	24.66	24.62	24.59	24.45	24.15	23.98	23.65	23.37
11.72	23.86	24.09	24.26	24.33	24.28	24.19	24.06	23.92	23.68	23.30	23.01
11.92	23.49	23.72	23.83	23.95	23.92	23.83	23.68	23.48	23.17	22.89	22.51
12.12	23.80	23.96	24.10	24.21	24.17	24.09	23.95	23.68	23.52	23.12	22.83
12.32	24.15	24.44	24.55	24.60	24.64	24.58	24.47	24.25	23.99	23.68	23.35
12.52	24.78	24.97	25.17	25.26	25.26	25.25	25.06	24.86	24.65	24.36	24.02
12.72	24.36	24.68	24.81	24.84	24.91	24.80	24.76	24.49	24.25	23.96	23.63
12.92	24.23	24.43	24.63	24.73	24.72	24.68	24.55	24.36	24.09	23.76	23.50
13.12	24.25	24.57	24.72	24.82	24.79	24.74	24.52	24.31	24.15	23.82	23.45
13.32	24.37	24.68	24.76	24.88	24.91	24.87	24.72	24.61	24.33	24.00	23.66
13.52	24.60	24.79	24.96	25.00	25.04	24.99	24.86	24.64	24.41	24.10	23.81
13.72	24.84	25.10	25.24	25.32	25.31	25.30	25.25	24.99	24.76	24.44	24.07
13.92	24.81	25.03	25.18	25.33	25.31	25.25	25.13	24.94	24.63	24.35	23.99
14	23.98	24.23	24.32	24.48	24.52	24.38	24.22	24.08	23.77	23.44	23.14
14.08	24.08	24.38	24.44	24.57	24.57	24.59	24.45	24.21	23.93	23.59	23.31
14.17	24.13	24.38	24.53	24.62	24.52	24.44	24.34	24.12	23.98	23.53	23.26
14.27	24.37	24.67	24.79	24.85	24.82	24.81	24.68	24.50	24.25	23.88	23.65
14.38	24.42	24.72	24.76	24.87	24.87	24.86	24.74	24.48	24.27	23.90	23.63
14.49	23.95	24.19	24.34	24.35	24.41	24.34	24.26	24.10	23.78	23.54	23.16
14.61	24.33	24.64	24.82	24.86	24.89	24.79	24.73	24.49	24.19	23.92	23.62
14.75	24.46	24.78	24.90	24.98	24.99	24.90	24.77	24.60	24.39	23.94	23.66
14.89	24.39	24.72	24.75	24.88	24.90	24.81	24.75	24.51	24.29	23.97	23.63
15.05	24.93	25.25	25.35	25.47	25.46	25.40	25.25	25.10	24.86	24.57	24.27
15.21	24.43	24.66	24.75	24.86	24.91	24.75	24.66	24.39	24.23	23.88	23.58
15.39	24.22	24.46	24.63	24.73	24.73	24.64	24.48	24.33	24.08	23.75	23.38
15.59	24.31	24.65	24.75	24.84	24.82	24.80	24.71	24.52	24.22	23.96	23.61
15.8	23.89	24.09	24.19	24.32	24.32	24.24	24.15	23.95	23.73	23.41	23.11
16.02	24.18	24.45	24.55	24.63	24.69	24.58	24.43	24.25	24.03	23.73	23.37

16.27	24.96	25.27	25.34	25.39	25.42	25.42	25.28	25.00	24.75	24.49	24.17
16.53	24.60	24.88	24.99	25.12	25.07	24.94	24.87	24.67	24.36	24.08	23.73
16.81	24.50	24.86	24.98	25.15	25.11	25.04	24.90	24.75	24.53	24.15	23.79
17.12	24.26	24.53	24.61	24.73	24.74	24.72	24.55	24.37	24.09	23.75	23.43
17.45	24.63	24.87	25.06	25.14	25.15	25.08	25.00	24.69	24.49	24.12	23.90
17.81	24.90	25.14	25.25	25.39	25.37	25.34	25.15	24.93	24.78	24.42	24.13
18.19	24.81	25.11	25.32	25.40	25.38	25.39	25.26	25.03	24.78	24.38	24.13
18.6	24.18	24.50	24.64	24.77	24.76	24.68	24.54	24.34	24.07	23.77	23.49
19.05	24.48	24.78	24.89	25.05	25.02	24.99	24.91	24.69	24.46	24.11	23.80
19.53	25.01	25.21	25.36	25.44	25.52	25.45	25.30	25.10	24.84	24.56	24.18
20.05	24.28	24.59	24.69	24.75	24.74	24.76	24.64	24.45	24.18	23.93	23.58
20.61	24.83	25.13	25.27	25.41	25.34	25.32	25.23	25.02	24.66	24.37	24.07
21.22	24.48	24.75	24.85	25.06	25.06	25.00	24.82	24.61	24.36	24.06	23.80
21.87	24.55	24.76	24.92	25.02	25.01	25.02	24.82	24.63	24.37	24.02	23.74
22.57	24.88	25.15	25.31	25.43	25.49	25.45	25.31	25.10	24.82	24.53	24.15
23.33	24.61	24.92	25.06	25.20	25.11	25.11	24.90	24.73	24.51	24.19	23.88
24.15	25.00	25.31	25.43	25.57	25.54	25.49	25.41	25.19	25.00	24.68	24.38
25.03	25.19	25.36	25.56	25.67	25.61	25.63	25.48	25.35	25.04	24.66	24.41
25.98	24.37	24.66	24.81	24.94	24.93	24.90	24.78	24.60	24.38	24.02	23.76
27.01	24.46	24.72	24.91	25.07	25.09	25.13	24.93	24.79	24.55	24.19	23.88
28.11	24.66	24.91	25.06	25.21	25.19	25.14	25.11	24.85	24.61	24.24	23.92
29.3	25.01	25.25	25.48	25.59	25.60	25.56	25.44	25.30	25.02	24.72	24.39
30.59	24.38	24.69	24.84	24.93	24.96	24.91	24.76	24.68	24.42	24.13	23.86
31.98	25.06	25.33	25.52	25.66	25.66	25.57	25.46	25.27	25.05	24.66	24.42
33.47	25.17	25.41	25.63	25.76	25.74	25.68	25.59	25.38	25.20	24.81	24.46
35.09	25.35	25.62	25.75	25.92	25.94	25.81	25.74	25.57	25.34	24.96	24.67
36.83	25.54	25.82	26.01	26.12	26.17	26.09	25.98	25.81	25.52	25.22	24.97
38.71	25.59	25.84	26.02	26.08	26.15	26.11	25.97	25.83	25.65	25.27	24.95
40.73	25.55	25.98	26.08	26.18	26.21	26.23	26.06	25.92	25.66	25.41	25.12
42.92	26.58	26.94	27.15	27.29	27.31	27.27	27.16	26.93	26.70	26.37	26.05
45.27	25.53	25.85	26.04	26.15	26.15	26.12	26.08	25.84	25.62	25.32	25.05
47.81	25.82	26.18	26.31	26.49	26.48	26.48	26.43	26.26	26.07	25.80	25.40
50.55	25.82	26.09	26.28	26.37	26.42	26.44	26.31	26.20	25.95	25.67	25.36
53.51	25.85	26.13	26.32	26.46	26.53	26.48	26.39	26.29	26.05	25.74	25.49
56.69	26.75	27.09	27.20	27.40	27.48	27.36	27.32	27.14	26.92	26.57	26.25
60.13	26.13	26.44	26.62	26.76	26.90	26.87	26.83	26.62	26.45	26.14	25.82
63.84	26.49	26.83	27.01	27.19	27.26	27.35	27.18	27.02	26.88	26.55	26.26
67.83	26.73	27.07	27.23	27.47	27.51	27.55	27.48	27.37	27.14	26.85	26.56
72.15	26.61	26.89	27.10	27.32	27.35	27.37	27.34	27.26	27.04	26.74	26.51
76.8	26.58	26.90	27.10	27.26	27.35	27.37	27.28	27.17	27.03	26.74	26.46
81.81	27.14	27.49	27.68	27.83	27.93	27.99	27.84	27.70	27.52	27.20	26.92
87.22	27.22	27.50	27.72	27.93	27.98	27.97	27.91	27.75	27.60	27.30	27.04
93.06	28.06	28.37	28.59	28.83	28.89	28.94	28.92	28.76	28.52	28.21	27.94
99.35	28.55	28.94	29.22	29.40	29.51	29.56	29.48	29.38	29.21	28.91	28.72
106.13	27.92	28.36	28.56	28.74	28.87	28.91	28.86	28.80	28.62	28.37	28.11
113.45	28.53	28.86	29.14	29.36	29.50	29.47	29.47	29.32	29.17	28.94	28.68
121.34	27.92	28.34	28.55	28.79	28.87	28.91	28.87	28.81	28.64	28.34	28.07
129.86	28.61	28.98	29.30	29.51	29.65	29.77	29.75	29.66	29.56	29.23	29.02
139.04	29.72	30.11	30.40	30.65	30.84	30.83	30.84	30.79	30.56	30.28	30.06
148.94	30.05	30.44	30.70	30.98	31.04	31.17	31.13	31.10	31.00	30.77	30.53

159.62	29.66	30.08	30.33	30.55	30.72	30.77	30.80	30.72	30.62	30.33	30.09
171.14	29.88	30.33	30.63	30.85	31.03	31.09	31.10	31.05	30.92	30.65	30.48
183.56	29.83	30.25	30.58	30.86	31.00	31.09	31.11	31.05	30.96	30.70	30.54
196.96	30.94	31.35	31.65	31.93	32.12	32.21	32.33	32.22	32.12	31.92	31.74
211.41	31.41	31.84	32.22	32.41	32.59	32.70	32.78	32.74	32.62	32.41	32.16
226.99	32.46	32.91	33.19	33.51	33.68	33.79	33.84	33.75	33.72	33.54	33.34
243.8	31.82	32.26	32.67	32.95	33.10	33.27	33.29	33.34	33.19	33.02	32.84
261.93	32.64	33.07	33.50	33.84	34.09	34.19	34.28	34.27	34.16	34.00	33.81
281.48	33.06	33.54	33.88	34.28	34.45	34.61	34.63	34.62	34.55	34.29	34.19
302.56	33.69	34.22	34.63	34.99	35.23	35.36	35.41	35.44	35.32	35.16	34.96
325.3	33.75	34.25	34.66	35.02	35.20	35.44	35.47	35.52	35.48	35.33	35.17
349.83	33.18	33.71	34.13	34.42	34.66	34.79	34.93	35.01	34.98	34.77	34.75
376.28	34.59	35.19	35.60	35.95	36.18	36.41	36.55	36.57	36.53	36.36	36.24
404.81	34.54	35.12	35.56	35.92	36.22	36.38	36.44	36.45	36.43	36.35	36.28
435.58	35.24	35.80	36.25	36.61	36.88	37.15	37.28	37.33	37.29	37.14	37.05
468.76	36.10	36.66	37.04	37.44	37.75	38.00	38.09	38.15	38.17	38.00	37.93
504.55	36.25	36.75	37.27	37.67	37.94	38.17	38.31	38.40	38.39	38.30	38.26
543.15	36.41	36.94	37.39	37.84	38.16	38.38	38.50	38.59	38.62	38.53	38.42
584.78	36.97	37.52	37.93	38.38	38.68	39.01	39.14	39.20	39.20	39.14	39.03
629.68	37.44	38.08	38.52	38.99	39.31	39.61	39.79	39.81	39.82	39.75	39.70
678.1	37.53	38.14	38.63	39.07	39.38	39.62	39.78	39.79	39.83	39.73	39.68
730.33	37.95	38.56	38.99	39.46	39.84	40.10	40.25	40.40	40.42	40.36	40.30
786.65	37.71	38.31	38.83	39.31	39.66	39.92	40.12	40.19	40.30	40.28	40.20
847.4	38.67	39.28	39.76	40.20	40.54	40.80	41.03	41.15	41.20	41.13	41.04
912.92	38.24	38.82	39.28	39.79	40.07	40.41	40.57	40.71	40.79	40.65	40.61
983.58	38.09	38.68	39.19	39.68	39.99	40.29	40.40	40.53	40.63	40.51	40.54
1059.78	38.35	38.98	39.43	39.93	40.33	40.58	40.72	40.91	40.90	40.84	40.84
1141.98	37.94	38.58	39.04	39.50	39.81	40.17	40.36	40.42	40.56	40.50	40.42
1230.62	38.43	39.11	39.62	40.06	40.45	40.77	40.98	41.01	41.04	41.13	41.06
1326.23	39.23	39.85	40.40	40.83	41.24	41.53	41.75	41.86	41.94	41.92	41.82
1429.34	39.37	40.04	40.60	41.01	41.36	41.61	41.83	42.02	42.03	42.01	42.01
1540.54	39.18	39.84	40.30	40.82	41.15	41.45	41.64	41.75	41.86	41.80	41.77
1660.48	39.46	40.10	40.66	41.12	41.59	41.82	42.15	42.20	42.28	42.26	42.20
1789.83	39.45	40.03	40.62	41.07	41.43	41.76	41.98	42.15	42.26	42.21	42.26
1929.34	40.13	40.83	41.37	41.85	42.18	42.49	42.79	42.88	42.94	42.89	42.84
2079.8	38.79	39.40	39.93	40.44	40.83	41.10	41.35	41.50	41.55	41.52	41.53
2242.08	39.94	40.66	41.13	41.68	42.01	42.36	42.56	42.72	42.77	42.76	42.67
2417.1	39.39	40.04	40.55	41.09	41.44	41.75	41.91	42.09	42.10	42.06	42.07
2605.85	39.12	39.80	40.27	40.79	41.12	41.47	41.67	41.78	41.75	41.85	41.82
2809.43	39.10	39.72	40.29	40.71	41.14	41.42	41.64	41.76	41.75	41.77	41.75
3028.99	38.07	38.70	39.25	39.70	40.05	40.32	40.56	40.66	40.71	40.67	40.62
3265.79	38.35	39.01	39.52	40.03	40.35	40.62	40.87	40.95	41.02	41.02	40.96
3521.18	38.33	38.96	39.46	39.96	40.32	40.63	40.78	40.96	41.08	41.01	40.91
3796.62	38.87	39.43	39.93	40.43	40.80	41.20	41.30	41.43	41.55	41.53	41.49

Wavelength (nm) / Time (ps)	584.10	585.51	586.92	588.33	589.74	591.15	592.55	593.96	595.37	596.78	598.19
-3.28	0.13	0.11	0.10	0.12	0.11	0.14	0.10	0.12	0.12	0.11	0.14

-2.78	-0.05	-0.01	0.03	0.00	0.11	-0.03	0.04	-0.02	-0.03	0.04	-0.06
-2.28	0.15	0.14	0.11	0.08	0.10	0.14	0.08	0.11	0.09	0.10	0.13
-1.78	0.13	0.10	0.14	0.10	0.03	0.13	0.07	0.13	0.12	0.16	0.10
-1.28	0.04	0.01	0.01	0.06	-0.05	-0.02	-0.01	-0.01	-0.05	-0.02	0.00
-0.78	-0.05	-0.03	0.01	-0.05	-0.09	-0.02	-0.02	-0.08	-0.03	-0.06	-0.02
-0.28	-0.47	-0.45	-0.46	-0.40	-0.34	-0.39	-0.40	-0.36	-0.38	-0.41	-0.40
0.22	0.11	0.13	0.07	0.10	0.12	0.06	0.14	0.12	0.16	0.10	0.11
0.32	0.31	0.30	0.31	0.33	0.36	0.38	0.33	0.35	0.35	0.34	0.33
0.42	0.10	0.15	0.12	0.10	0.12	0.17	0.13	0.11	0.12	0.13	0.12
0.52	-0.15	-0.13	-0.13	-0.17	-0.14	-0.16	-0.19	-0.15	-0.20	-0.16	-0.20
0.62	0.01	-0.04	-0.01	0.01	0.00	0.02	0.03	-0.05	-0.04	0.02	0.03
0.72	-0.09	-0.02	-0.05	-0.07	-0.07	-0.01	-0.06	-0.08	-0.07	-0.04	-0.05
0.77	0.25	0.23	0.21	0.28	0.28	0.27	0.23	0.27	0.23	0.23	0.22
0.82	-0.10	-0.17	-0.06	-0.14	-0.10	-0.08	-0.07	-0.06	-0.07	-0.03	-0.06
0.87	-0.27	-0.25	-0.23	-0.19	-0.18	-0.20	-0.19	-0.22	-0.25	-0.21	-0.26
0.92	-0.02	-0.01	0.00	-0.03	0.03	0.01	0.03	-0.05	-0.01	0.01	-0.01
0.97	0.24	0.23	0.29	0.15	0.21	0.18	0.22	0.17	0.16	0.16	0.23
1.02	0.15	0.18	0.17	0.16	0.18	0.22	0.20	0.17	0.15	0.18	0.18
1.07	0.11	0.01	0.02	0.05	0.03	0.11	0.03	0.05	0.06	0.11	0.08
1.12	-0.10	-0.11	-0.09	-0.06	-0.10	-0.10	-0.13	-0.12	-0.13	-0.06	-0.10
1.17	0.11	0.09	0.11	0.06	0.03	0.05	0.02	0.00	0.00	0.05	0.08
1.22	0.30	0.28	0.27	0.30	0.32	0.31	0.30	0.27	0.30	0.28	0.26
1.27	0.19	0.22	0.23	0.15	0.19	0.19	0.19	0.13	0.13	0.17	0.16
1.32	-0.05	-0.07	-0.04	-0.04	-0.05	-0.03	-0.01	-0.02	-0.01	-0.02	-0.01
1.37	-0.05	0.02	-0.02	0.00	0.00	0.04	-0.01	0.00	0.07	0.05	0.08
1.42	0.15	0.13	0.17	0.13	0.16	0.22	0.12	0.15	0.13	0.18	0.15
1.47	0.09	0.05	0.06	-0.03	-0.03	-0.09	-0.08	-0.12	-0.04	-0.02	0.06
1.52	1.71	1.51	1.38	1.25	1.10	0.96	0.73	0.63	0.56	0.53	0.41
1.57	7.30	7.07	6.70	6.42	6.21	5.93	5.53	5.20	4.90	4.60	4.31
1.62	15.25	15.09	15.05	14.82	14.57	14.39	14.04	13.66	13.30	12.89	12.51
1.67	18.62	18.83	19.01	19.20	19.24	19.40	19.40	19.35	19.38	19.42	19.34
1.72	16.47	16.69	16.85	17.09	17.21	17.43	17.54	17.75	17.86	17.96	18.11
1.77	14.52	14.71	14.88	14.98	15.09	15.24	15.36	15.45	15.52	15.61	15.76
1.82	13.13	13.26	13.35	13.45	13.61	13.65	13.69	13.76	13.81	13.91	13.99
1.87	12.36	12.41	12.47	12.54	12.54	12.59	12.59	12.66	12.68	12.72	12.82
1.92	12.65	12.63	12.68	12.73	12.66	12.71	12.69	12.63	12.66	12.70	12.70
1.97	12.66	12.69	12.68	12.62	12.58	12.60	12.51	12.47	12.46	12.41	12.45
2.02	13.31	13.23	13.17	13.16	13.13	13.00	12.92	12.87	12.76	12.73	12.66
2.07	14.02	13.92	13.95	13.83	13.67	13.63	13.47	13.37	13.33	13.26	13.19
2.12	13.41	13.36	13.32	13.20	13.08	13.04	12.92	12.79	12.66	12.57	12.56
2.17	13.84	13.75	13.62	13.50	13.45	13.39	13.22	13.08	13.00	12.90	12.88
2.22	14.27	14.18	14.07	13.91	13.83	13.66	13.64	13.45	13.31	13.18	13.12
2.27	14.71	14.58	14.51	14.32	14.23	14.13	13.92	13.77	13.69	13.58	13.51
2.32	15.49	15.38	15.24	15.06	14.95	14.73	14.64	14.45	14.35	14.22	14.11
2.37	15.38	15.28	15.06	14.99	14.74	14.63	14.46	14.30	14.23	14.02	13.89
2.42	15.53	15.37	15.18	15.01	14.82	14.69	14.56	14.30	14.21	14.02	13.94
2.47	15.86	15.73	15.47	15.35	15.22	15.02	14.82	14.60	14.46	14.39	14.25
2.52	15.81	15.64	15.38	15.25	15.04	14.85	14.69	14.49	14.36	14.10	13.96
2.57	16.39	16.18	16.01	15.84	15.67	15.47	15.22	15.05	14.80	14.70	14.56
2.62	16.64	16.50	16.27	16.04	15.87	15.69	15.46	15.29	14.96	14.88	14.71

2.67	16.39	16.16	15.93	15.68	15.57	15.34	15.07	14.94	14.67	14.48	14.38
2.72	16.39	16.13	15.96	15.68	15.48	15.28	15.06	14.83	14.71	14.51	14.38
2.77	16.78	16.58	16.36	16.11	15.93	15.61	15.41	15.18	14.95	14.80	14.62
2.82	17.40	17.09	16.93	16.67	16.41	16.15	15.96	15.68	15.48	15.26	15.11
2.87	17.36	17.14	16.86	16.68	16.37	16.21	15.88	15.65	15.45	15.29	15.08
2.92	17.83	17.56	17.32	17.03	16.77	16.51	16.24	15.99	15.80	15.60	15.37
2.97	17.99	17.71	17.45	17.18	16.87	16.63	16.35	16.13	15.92	15.61	15.45
3.02	17.63	17.37	17.12	16.86	16.52	16.24	16.01	15.82	15.53	15.30	15.06
3.07	17.75	17.46	17.19	16.94	16.72	16.42	16.10	15.91	15.65	15.36	15.11
3.12	19.05	18.76	18.50	18.21	17.91	17.65	17.34	17.03	16.78	16.57	16.36
3.17	18.53	18.22	17.93	17.64	17.32	17.10	16.78	16.48	16.24	15.97	15.71
3.22	18.41	18.09	17.86	17.48	17.24	16.92	16.65	16.36	16.06	15.83	15.63
3.27	18.15	17.88	17.61	17.28	17.00	16.69	16.43	16.18	15.89	15.65	15.42
3.32	19.12	18.85	18.49	18.20	17.91	17.60	17.31	17.05	16.73	16.46	16.25
3.37	18.95	18.65	18.31	18.05	17.75	17.39	17.08	16.77	16.49	16.25	16.02
3.42	18.83	18.64	18.34	18.02	17.64	17.37	17.11	16.81	16.44	16.21	15.94
3.47	19.14	18.79	18.49	18.17	17.86	17.54	17.18	16.85	16.63	16.32	16.13
3.52	19.28	19.00	18.72	18.37	18.02	17.73	17.44	17.03	16.76	16.54	16.25
3.57	19.02	18.71	18.41	18.09	17.74	17.43	17.12	16.75	16.54	16.26	15.97
3.62	19.58	19.32	18.97	18.59	18.37	18.00	17.62	17.33	17.05	16.71	16.52
3.67	19.80	19.41	19.13	18.85	18.52	18.16	17.77	17.50	17.20	16.89	16.62
3.72	20.44	20.12	19.78	19.36	19.10	18.72	18.39	18.02	17.75	17.42	17.17
3.92	19.60	19.28	18.99	18.57	18.33	17.90	17.56	17.24	16.92	16.62	16.35
4.12	20.41	20.13	19.72	19.34	19.07	18.69	18.33	18.01	17.66	17.34	17.03
4.32	20.81	20.49	20.14	19.72	19.31	18.99	18.57	18.25	17.91	17.54	17.27
4.52	21.51	21.20	20.86	20.51	20.12	19.67	19.26	18.93	18.57	18.21	17.90
4.72	21.70	21.36	20.97	20.57	20.28	19.83	19.44	19.10	18.78	18.37	18.02
4.92	21.49	21.15	20.79	20.38	19.97	19.64	19.25	18.77	18.45	18.09	17.75
5.12	21.85	21.50	21.15	20.72	20.35	19.92	19.54	19.11	18.78	18.40	18.05
5.32	22.11	21.74	21.38	20.98	20.54	20.21	19.75	19.29	18.95	18.59	18.29
5.52	22.01	21.61	21.18	20.87	20.44	20.02	19.57	19.20	18.82	18.45	18.15
5.72	22.18	21.78	21.39	21.03	20.60	20.15	19.73	19.32	18.98	18.56	18.15
5.92	22.52	22.09	21.76	21.34	20.93	20.51	20.17	19.67	19.31	18.93	18.53
6.12	22.07	21.74	21.34	20.89	20.55	20.10	19.65	19.26	18.83	18.45	18.11
6.32	22.09	21.76	21.32	20.85	20.49	20.06	19.64	19.20	18.79	18.46	18.08
6.52	22.52	22.08	21.69	21.29	20.85	20.42	20.03	19.60	19.22	18.79	18.43
6.72	22.67	22.33	21.91	21.47	21.04	20.59	20.12	19.74	19.33	18.89	18.48
6.92	22.28	21.92	21.55	21.14	20.75	20.36	19.91	19.46	19.11	18.70	18.33
7.12	22.34	21.92	21.52	21.15	20.72	20.25	19.85	19.49	19.00	18.58	18.25
7.32	22.91	22.45	22.10	21.73	21.23	20.88	20.36	19.97	19.52	19.16	18.75
7.52	22.39	22.02	21.62	21.18	20.75	20.35	19.93	19.45	19.00	18.68	18.25
7.72	22.32	22.00	21.58	21.11	20.71	20.24	19.76	19.34	18.88	18.51	18.13
7.92	22.96	22.47	22.07	21.62	21.22	20.86	20.36	19.81	19.45	19.07	18.65
8.12	22.66	22.28	21.89	21.52	21.01	20.64	20.20	19.70	19.32	18.94	18.57
8.32	22.81	22.39	21.94	21.60	21.08	20.63	20.22	19.72	19.29	18.83	18.58
8.52	22.73	22.22	21.92	21.44	21.07	20.56	20.13	19.67	19.20	18.84	18.37
8.72	22.69	22.32	21.87	21.44	21.03	20.56	20.11	19.64	19.28	18.84	18.46
8.92	22.90	22.55	22.06	21.60	21.15	20.75	20.34	19.80	19.36	18.91	18.51
9.12	23.04	22.68	22.25	21.86	21.43	21.02	20.51	20.06	19.61	19.20	18.82
9.32	23.07	22.71	22.36	21.90	21.45	20.93	20.51	20.00	19.58	19.15	18.70

9.52	23.49	23.14	22.78	22.22	21.80	21.34	20.82	20.33	20.00	19.58	19.14
9.72	23.23	22.80	22.41	21.93	21.49	21.02	20.52	20.06	19.62	19.19	18.76
9.92	23.21	22.80	22.40	21.94	21.47	21.06	20.55	20.05	19.61	19.21	18.85
10.12	22.95	22.50	22.17	21.73	21.24	20.84	20.34	19.89	19.46	19.03	18.69
10.32	23.09	22.68	22.31	21.86	21.44	21.02	20.52	20.09	19.66	19.24	18.86
10.52	22.86	22.45	21.97	21.60	21.14	20.68	20.20	19.73	19.29	18.85	18.41
10.72	22.64	22.22	21.81	21.35	20.94	20.43	20.05	19.49	19.09	18.72	18.29
10.92	23.16	22.72	22.32	21.94	21.42	20.96	20.57	20.06	19.75	19.25	18.76
11.12	22.97	22.56	22.17	21.68	21.28	20.77	20.37	19.83	19.38	18.99	18.58
11.32	22.92	22.51	22.18	21.72	21.29	20.84	20.36	19.83	19.43	19.01	18.59
11.52	23.00	22.61	22.19	21.78	21.30	20.82	20.34	19.95	19.48	19.11	18.63
11.72	22.62	22.16	21.83	21.34	20.94	20.51	20.03	19.55	19.15	18.63	18.25
11.92	22.11	21.74	21.31	20.85	20.45	19.99	19.57	19.06	18.65	18.22	17.87
12.12	22.44	22.07	21.61	21.15	20.75	20.29	19.83	19.39	18.95	18.51	18.11
12.32	22.96	22.58	22.12	21.74	21.26	20.74	20.31	19.83	19.35	18.95	18.54
12.52	23.63	23.25	22.88	22.33	21.94	21.47	21.02	20.52	20.10	19.63	19.16
12.72	23.28	22.83	22.42	21.95	21.47	21.01	20.49	20.07	19.62	19.14	18.69
12.92	23.10	22.65	22.22	21.82	21.30	20.93	20.45	19.99	19.52	19.07	18.62
13.12	23.04	22.62	22.19	21.75	21.29	20.78	20.34	19.89	19.44	18.97	18.55
13.32	23.31	22.85	22.40	21.98	21.50	21.05	20.57	20.07	19.64	19.16	18.68
13.52	23.37	23.04	22.51	22.08	21.69	21.26	20.69	20.29	19.78	19.37	18.98
13.72	23.74	23.33	22.92	22.48	22.03	21.60	21.05	20.57	20.19	19.67	19.27
13.92	23.60	23.13	22.72	22.25	21.82	21.31	20.87	20.37	19.93	19.49	19.02
14	22.79	22.33	21.97	21.51	21.01	20.55	20.09	19.61	19.18	18.71	18.31
14.08	22.86	22.49	22.07	21.61	21.16	20.71	20.16	19.74	19.31	18.87	18.52
14.17	22.86	22.50	22.08	21.59	21.13	20.67	20.29	19.73	19.29	18.92	18.46
14.27	23.29	22.87	22.42	21.98	21.53	21.06	20.57	20.09	19.66	19.27	18.75
14.38	23.25	22.83	22.37	21.88	21.48	21.09	20.60	20.06	19.58	19.12	18.72
14.49	22.77	22.38	22.01	21.55	21.15	20.64	20.14	19.73	19.26	18.86	18.46
14.61	23.14	22.76	22.33	21.89	21.43	20.88	20.43	19.89	19.49	19.03	18.63
14.75	23.34	22.90	22.46	22.01	21.50	21.07	20.59	20.03	19.65	19.14	18.73
14.89	23.22	22.85	22.50	21.98	21.52	21.11	20.63	20.14	19.71	19.25	18.79
15.05	23.87	23.51	23.09	22.60	22.15	21.63	21.21	20.69	20.20	19.77	19.32
15.21	23.13	22.67	22.30	21.91	21.38	20.89	20.39	19.95	19.47	19.00	18.62
15.39	23.06	22.60	22.19	21.74	21.24	20.77	20.31	19.84	19.38	18.90	18.44
15.59	23.20	22.75	22.36	21.92	21.35	21.02	20.50	20.00	19.54	19.05	18.72
15.8	22.72	22.30	21.88	21.43	21.05	20.53	20.10	19.61	19.17	18.70	18.30
16.02	23.08	22.61	22.24	21.79	21.36	20.93	20.36	19.92	19.48	19.06	18.59
16.27	23.74	23.33	22.95	22.43	22.02	21.51	21.03	20.57	20.06	19.60	19.19
16.53	23.37	22.94	22.55	22.06	21.61	21.18	20.64	20.18	19.67	19.27	18.82
16.81	23.40	22.96	22.52	22.16	21.66	21.14	20.65	20.18	19.71	19.29	18.82
17.12	23.13	22.71	22.28	21.80	21.35	20.84	20.37	19.86	19.44	18.97	18.55
17.45	23.44	23.06	22.58	22.11	21.69	21.16	20.72	20.17	19.80	19.24	18.84
17.81	23.77	23.33	22.96	22.54	22.03	21.55	21.10	20.64	20.11	19.70	19.31
18.19	23.74	23.25	22.84	22.41	21.93	21.46	20.97	20.48	19.94	19.58	19.12
18.6	23.07	22.67	22.25	21.78	21.29	20.89	20.39	19.84	19.46	19.03	18.51
19.05	23.37	22.95	22.55	22.05	21.51	21.08	20.56	20.03	19.63	19.17	18.69
19.53	23.84	23.43	23.04	22.49	21.97	21.58	21.04	20.53	20.11	19.58	19.20
20.05	23.22	22.75	22.38	21.90	21.48	21.01	20.50	20.07	19.59	19.06	18.65
20.61	23.79	23.31	22.84	22.40	21.94	21.42	20.96	20.49	20.02	19.49	19.08

21.22	23.40	22.94	22.48	22.04	21.59	21.11	20.57	20.09	19.62	19.19	18.70
21.87	23.32	22.84	22.45	21.99	21.55	20.99	20.47	20.04	19.52	19.07	18.64
22.57	23.77	23.26	22.83	22.37	21.92	21.43	21.00	20.43	19.97	19.53	19.07
23.33	23.50	23.12	22.69	22.21	21.77	21.31	20.73	20.32	19.81	19.41	18.94
24.15	24.00	23.55	23.15	22.67	22.23	21.77	21.28	20.79	20.27	19.87	19.36
25.03	23.99	23.56	23.10	22.64	22.21	21.67	21.21	20.73	20.21	19.76	19.33
25.98	23.37	22.93	22.48	22.04	21.65	21.17	20.68	20.14	19.67	19.28	18.81
27.01	23.48	23.10	22.75	22.20	21.70	21.18	20.72	20.18	19.74	19.28	18.86
28.11	23.58	23.13	22.72	22.28	21.79	21.28	20.84	20.28	19.89	19.36	18.92
29.3	24.02	23.59	23.22	22.69	22.25	21.71	21.27	20.72	20.27	19.82	19.32
30.59	23.45	23.07	22.70	22.20	21.74	21.33	20.84	20.35	19.84	19.42	18.99
31.98	23.99	23.56	23.09	22.62	22.12	21.70	21.20	20.67	20.21	19.74	19.23
33.47	24.14	23.71	23.28	22.83	22.38	21.98	21.45	20.91	20.46	20.05	19.54
35.09	24.28	23.84	23.39	22.92	22.47	22.05	21.47	21.00	20.47	20.03	19.57
36.83	24.53	24.05	23.66	23.23	22.76	22.26	21.75	21.30	20.73	20.31	19.85
38.71	24.61	24.27	23.81	23.28	22.84	22.45	21.95	21.42	20.91	20.52	20.08
40.73	24.74	24.31	23.87	23.37	22.88	22.49	21.99	21.46	20.98	20.54	20.01
42.92	25.69	25.27	24.81	24.39	23.84	23.39	22.88	22.36	21.87	21.34	20.93
45.27	24.65	24.23	23.89	23.44	22.87	22.44	21.89	21.45	20.96	20.49	20.00
47.81	25.09	24.76	24.33	23.79	23.36	22.84	22.33	21.80	21.36	20.83	20.36
50.55	25.01	24.58	24.15	23.74	23.23	22.77	22.28	21.76	21.31	20.79	20.45
53.51	25.17	24.84	24.39	23.95	23.45	23.03	22.52	22.00	21.56	21.09	20.55
56.69	25.90	25.43	25.05	24.60	24.06	23.61	23.10	22.56	22.06	21.55	21.13
60.13	25.44	25.09	24.64	24.26	23.78	23.30	22.85	22.35	21.87	21.34	20.92
63.84	25.89	25.50	25.05	24.61	24.15	23.71	23.21	22.68	22.21	21.67	21.20
67.83	26.24	25.81	25.43	24.99	24.47	24.05	23.54	22.97	22.51	22.00	21.59
72.15	26.22	25.77	25.43	24.98	24.56	24.06	23.47	23.05	22.62	22.12	21.63
76.8	26.12	25.76	25.40	24.86	24.44	24.03	23.50	23.02	22.53	22.06	21.55
81.81	26.50	26.19	25.75	25.31	24.90	24.38	23.85	23.32	22.84	22.44	21.86
87.22	26.64	26.24	25.87	25.40	24.98	24.46	24.02	23.49	23.05	22.48	22.01
93.06	27.65	27.23	26.82	26.36	25.95	25.37	24.92	24.40	23.94	23.38	22.94
99.35	28.33	27.97	27.60	27.15	26.72	26.20	25.72	25.19	24.71	24.18	23.71
106.13	27.80	27.43	27.06	26.60	26.22	25.76	25.19	24.75	24.26	23.78	23.31
113.45	28.30	27.92	27.53	27.08	26.64	26.14	25.75	25.22	24.70	24.19	23.75
121.34	27.87	27.50	27.07	26.65	26.18	25.77	25.26	24.80	24.32	23.76	23.35
129.86	28.69	28.33	27.91	27.49	27.12	26.58	26.12	25.62	25.06	24.63	24.10
139.04	29.78	29.36	28.98	28.56	28.11	27.71	27.15	26.65	26.16	25.63	25.18
148.94	30.14	29.77	29.45	29.04	28.50	28.12	27.52	27.06	26.61	26.11	25.57
159.62	29.84	29.48	29.05	28.68	28.27	27.79	27.28	26.78	26.31	25.85	25.31
171.14	30.15	29.89	29.44	29.04	28.60	28.15	27.70	27.19	26.68	26.21	25.70
183.56	30.24	29.92	29.57	29.05	28.72	28.30	27.77	27.27	26.80	26.33	25.80
196.96	31.43	31.07	30.74	30.32	29.85	29.44	28.97	28.40	27.93	27.38	26.90
211.41	31.90	31.54	31.22	30.85	30.41	29.95	29.50	28.91	28.43	27.96	27.38
226.99	33.08	32.74	32.41	32.01	31.59	31.20	30.67	30.11	29.67	29.13	28.60
243.8	32.57	32.29	31.92	31.58	31.15	30.70	30.23	29.79	29.18	28.68	28.24
261.93	33.55	33.24	32.89	32.52	32.09	31.67	31.20	30.63	30.22	29.68	29.21
281.48	33.93	33.61	33.20	32.84	32.52	31.98	31.52	31.01	30.50	29.99	29.49
302.56	34.76	34.40	34.12	33.68	33.33	32.86	32.38	31.81	31.28	30.80	30.24
325.3	34.95	34.71	34.35	33.95	33.57	33.09	32.70	32.20	31.59	31.17	30.65
349.83	34.48	34.19	33.95	33.51	33.16	32.74	32.25	31.80	31.29	30.78	30.31

376.28	36.06	35.81	35.43	35.08	34.69	34.19	33.80	33.26	32.78	32.23	31.72
404.81	36.02	35.77	35.50	35.14	34.78	34.29	33.80	33.35	32.86	32.32	31.84
435.58	36.85	36.63	36.27	35.94	35.53	35.07	34.61	34.10	33.56	33.08	32.50
468.76	37.79	37.45	37.17	36.85	36.48	35.94	35.58	35.07	34.49	34.01	33.47
504.55	38.00	37.80	37.48	37.17	36.85	36.36	35.91	35.46	34.94	34.36	33.89
543.15	38.32	37.98	37.73	37.37	37.02	36.64	36.15	35.63	35.14	34.64	34.05
584.78	38.84	38.57	38.24	37.95	37.56	37.14	36.70	36.16	35.70	35.11	34.67
629.68	39.47	39.26	39.00	38.64	38.33	37.87	37.45	36.90	36.47	35.84	35.35
678.1	39.47	39.23	38.99	38.66	38.34	37.84	37.43	36.93	36.46	35.90	35.38
730.33	40.13	39.84	39.62	39.25	38.94	38.54	38.03	37.55	37.13	36.55	35.94
786.65	40.07	39.89	39.61	39.30	38.96	38.57	38.13	37.66	37.14	36.57	36.05
847.4	40.90	40.65	40.42	40.08	39.74	39.29	38.89	38.36	37.83	37.27	36.75
912.92	40.50	40.30	40.03	39.73	39.42	39.04	38.56	38.07	37.58	37.03	36.48
983.58	40.38	40.14	39.87	39.60	39.25	38.85	38.43	37.90	37.35	36.87	36.28
1059.78	40.63	40.50	40.26	39.91	39.55	39.18	38.77	38.21	37.76	37.23	36.64
1141.98	40.32	40.13	39.90	39.62	39.24	38.88	38.49	37.95	37.53	36.94	36.40
1230.62	40.95	40.71	40.50	40.16	39.87	39.46	39.10	38.55	38.10	37.45	36.96
1326.23	41.67	41.43	41.24	40.94	40.57	40.14	39.78	39.23	38.73	38.17	37.60
1429.34	41.76	41.57	41.34	41.02	40.73	40.33	39.86	39.36	38.86	38.25	37.75
1540.54	41.61	41.41	41.17	40.90	40.61	40.20	39.68	39.20	38.64	38.08	37.57
1660.48	42.07	41.85	41.72	41.33	41.00	40.59	40.12	39.59	39.14	38.60	38.02
1789.83	42.12	41.88	41.67	41.38	41.00	40.60	40.18	39.68	39.17	38.65	38.03
1929.34	42.74	42.43	42.23	41.97	41.48	41.15	40.64	40.17	39.63	39.12	38.48
2079.8	41.38	41.15	41.00	40.65	40.40	40.02	39.56	39.07	38.50	38.02	37.45
2242.08	42.56	42.29	42.08	41.76	41.42	41.03	40.62	40.07	39.54	39.03	38.41
2417.1	41.94	41.76	41.49	41.17	40.79	40.47	39.94	39.44	38.98	38.41	37.76
2605.85	41.69	41.47	41.25	40.97	40.62	40.21	39.84	39.33	38.84	38.27	37.69
2809.43	41.62	41.39	41.17	40.84	40.54	40.18	39.67	39.24	38.73	38.11	37.57
3028.99	40.52	40.33	40.06	39.73	39.43	39.05	38.58	38.10	37.63	37.09	36.54
3265.79	40.87	40.63	40.44	40.13	39.80	39.46	38.98	38.51	37.98	37.42	36.86
3521.18	40.86	40.66	40.43	40.10	39.78	39.37	38.94	38.44	37.97	37.34	36.81
3796.62	41.31	41.13	40.85	40.62	40.24	39.86	39.42	38.91	38.40	37.89	37.32

Wavelength (nm) / Time (ps)	599.60	601.00	602.41	603.82	605.23	606.64	608.05	609.46	610.86	612.27	613.68
-3.28	0.12	0.12	0.11	0.10	0.11	0.11	0.11	0.10	0.08	0.14	0.12
-2.78	-0.02	0.02	0.04	0.00	0.00	-0.03	0.01	-0.03	0.01	-0.02	0.01
-2.28	0.08	0.07	0.05	0.04	0.06	0.07	0.04	0.06	0.03	0.00	0.01
-1.78	0.12	0.12	0.10	0.13	0.15	0.16	0.14	0.12	0.15	0.16	0.09
-1.28	0.00	0.02	0.03	0.06	0.02	0.06	0.05	0.06	0.02	0.10	0.07
-0.78	-0.04	-0.09	-0.04	-0.05	-0.06	-0.07	-0.07	-0.12	-0.07	-0.12	-0.09
-0.28	-0.33	-0.36	-0.35	-0.38	-0.36	-0.38	-0.34	-0.22	-0.30	-0.30	-0.25
0.22	0.06	0.11	0.06	0.10	0.08	0.08	0.06	0.03	0.08	0.04	0.04
0.32	0.35	0.35	0.37	0.35	0.32	0.28	0.26	0.32	0.24	0.23	0.26
0.42	0.18	0.16	0.21	0.24	0.19	0.22	0.20	0.18	0.23	0.21	0.21
0.52	-0.23	-0.17	-0.12	-0.08	-0.13	-0.15	-0.06	-0.10	-0.04	-0.09	-0.09
0.62	0.00	-0.02	-0.02	0.02	0.03	0.03	0.03	-0.04	0.05	0.03	-0.01
0.72	0.03	-0.03	-0.04	0.05	-0.02	0.01	0.07	0.03	0.09	0.12	0.04

0.77	0.23	0.28	0.24	0.28	0.26	0.29	0.31	0.22	0.25	0.27	0.28
0.82	-0.16	-0.10	-0.03	-0.06	-0.09	-0.16	-0.09	-0.08	-0.06	-0.05	-0.09
0.87	-0.24	-0.29	-0.28	-0.18	-0.27	-0.23	-0.24	-0.22	-0.23	-0.21	-0.20
0.92	-0.01	0.02	0.05	0.01	-0.01	0.04	0.06	0.04	0.15	0.13	0.10
0.97	0.26	0.26	0.26	0.26	0.28	0.23	0.23	0.25	0.29	0.31	0.29
1.02	0.18	0.22	0.22	0.20	0.21	0.16	0.19	0.19	0.14	0.14	0.14
1.07	0.02	0.05	0.06	0.05	0.03	0.08	0.01	0.05	0.14	0.08	0.12
1.12	-0.11	-0.13	-0.14	-0.10	-0.17	-0.15	-0.06	-0.11	-0.10	-0.14	-0.10
1.17	0.03	0.05	0.02	0.12	0.10	0.09	0.12	0.15	0.15	0.21	0.17
1.22	0.25	0.29	0.24	0.28	0.25	0.25	0.25	0.22	0.22	0.21	0.16
1.27	0.13	0.15	0.20	0.17	0.19	0.12	0.12	0.15	0.11	0.13	0.12
1.32	-0.01	0.01	0.06	0.01	0.01	0.07	-0.01	-0.05	0.00	0.01	0.01
1.37	0.06	0.11	0.13	0.10	0.12	0.03	0.08	0.08	0.10	0.16	0.15
1.42	0.16	0.17	0.19	0.23	0.16	0.21	0.13	0.21	0.19	0.24	0.20
1.47	0.01	0.06	0.05	0.12	0.17	0.18	0.17	0.14	0.15	0.15	0.12
1.52	0.34	0.35	0.28	0.33	0.30	0.29	0.26	0.20	0.24	0.20	0.12
1.57	4.04	3.76	3.55	3.34	3.20	3.00	2.84	2.72	2.61	2.46	2.42
1.62	12.11	11.64	11.28	10.88	10.61	10.33	10.02	9.80	9.67	9.43	9.30
1.67	19.31	19.20	19.19	19.09	18.99	18.95	18.93	18.98	18.97	18.99	19.01
1.72	18.26	18.38	18.49	18.64	18.85	18.96	19.20	19.37	19.60	19.77	19.97
1.77	15.90	16.08	16.13	16.29	16.49	16.63	16.80	16.98	17.23	17.43	17.64
1.82	14.07	14.19	14.31	14.48	14.54	14.75	14.95	15.06	15.31	15.50	15.73
1.87	12.86	12.93	12.96	13.08	13.22	13.35	13.51	13.57	13.79	13.96	14.12
1.92	12.69	12.70	12.77	12.80	12.88	13.00	13.00	13.09	13.26	13.42	13.55
1.97	12.41	12.41	12.37	12.47	12.49	12.59	12.68	12.69	12.84	12.92	13.05
2.02	12.69	12.67	12.71	12.64	12.65	12.65	12.69	12.76	12.84	12.97	13.03
2.07	13.08	13.11	13.04	13.07	13.04	13.07	13.11	13.11	13.21	13.23	13.31
2.12	12.48	12.45	12.43	12.40	12.38	12.44	12.43	12.45	12.59	12.66	12.72
2.17	12.81	12.72	12.72	12.62	12.62	12.65	12.70	12.69	12.70	12.81	12.79
2.22	13.07	12.95	12.94	12.80	12.85	12.85	12.81	12.87	12.88	12.91	12.97
2.27	13.37	13.29	13.24	13.13	13.15	13.09	13.12	13.13	13.19	13.18	13.20
2.32	14.00	13.93	13.85	13.78	13.79	13.71	13.68	13.68	13.72	13.68	13.79
2.37	13.75	13.74	13.60	13.60	13.50	13.54	13.52	13.47	13.53	13.53	13.56
2.42	13.77	13.71	13.62	13.61	13.50	13.42	13.45	13.45	13.45	13.41	13.42
2.47	14.05	14.01	13.89	13.86	13.82	13.73	13.67	13.64	13.66	13.67	13.62
2.52	13.87	13.77	13.70	13.57	13.51	13.45	13.40	13.39	13.42	13.41	13.36
2.57	14.37	14.28	14.22	14.12	14.05	13.97	13.90	13.83	13.87	13.83	13.80
2.62	14.52	14.42	14.29	14.22	14.16	14.07	14.02	13.98	13.93	13.95	13.92
2.67	14.24	14.11	13.98	13.85	13.79	13.77	13.76	13.71	13.70	13.69	13.66
2.72	14.13	14.01	13.94	13.88	13.76	13.64	13.58	13.56	13.57	13.56	13.49
2.77	14.40	14.32	14.19	14.09	14.01	13.96	13.88	13.85	13.92	13.88	13.91
2.82	14.93	14.77	14.63	14.51	14.43	14.34	14.27	14.14	14.13	14.11	14.10
2.87	14.89	14.72	14.60	14.55	14.43	14.33	14.24	14.19	14.19	14.20	14.14
2.92	15.15	15.06	14.90	14.80	14.68	14.56	14.52	14.44	14.41	14.35	14.30
2.97	15.30	15.16	14.99	14.90	14.84	14.68	14.65	14.59	14.54	14.54	14.47
3.02	14.89	14.78	14.69	14.56	14.41	14.30	14.22	14.15	14.14	14.12	14.13
3.07	15.01	14.83	14.65	14.53	14.44	14.31	14.25	14.15	14.13	14.13	14.12
3.12	16.09	15.90	15.78	15.61	15.47	15.37	15.25	15.18	15.14	15.02	15.04
3.17	15.55	15.41	15.17	15.04	14.91	14.81	14.75	14.72	14.65	14.57	14.50
3.22	15.35	15.18	15.00	14.92	14.74	14.67	14.60	14.51	14.49	14.44	14.34

3.27	15.18	15.01	14.84	14.70	14.57	14.43	14.36	14.30	14.23	14.15	14.13
3.32	16.03	15.84	15.63	15.44	15.33	15.21	15.12	14.99	14.90	14.81	14.78
3.37	15.77	15.59	15.37	15.27	15.12	15.01	14.89	14.77	14.73	14.65	14.67
3.42	15.71	15.54	15.34	15.19	15.08	14.87	14.82	14.73	14.64	14.59	14.55
3.47	15.86	15.65	15.41	15.29	15.14	14.97	14.86	14.78	14.66	14.61	14.55
3.52	16.03	15.86	15.61	15.45	15.30	15.16	15.07	14.98	14.95	14.89	14.85
3.57	15.71	15.49	15.29	15.21	15.03	14.88	14.78	14.70	14.58	14.54	14.46
3.62	16.22	16.00	15.76	15.65	15.47	15.27	15.22	15.13	15.00	14.91	14.82
3.67	16.36	16.18	15.98	15.84	15.62	15.46	15.34	15.26	15.24	15.14	15.05
3.72	16.85	16.71	16.39	16.26	16.09	15.90	15.78	15.63	15.51	15.41	15.31
3.92	16.11	15.85	15.60	15.44	15.30	15.08	14.97	14.87	14.81	14.73	14.65
4.12	16.76	16.54	16.25	16.09	15.94	15.67	15.58	15.46	15.36	15.25	15.11
4.32	16.94	16.68	16.40	16.27	16.02	15.89	15.67	15.53	15.44	15.38	15.25
4.52	17.55	17.37	17.02	16.83	16.65	16.43	16.32	16.12	16.04	15.85	15.78
4.72	17.64	17.45	17.20	17.00	16.79	16.59	16.38	16.18	16.09	15.94	15.81
4.92	17.46	17.16	16.89	16.65	16.42	16.23	16.06	15.91	15.82	15.65	15.56
5.12	17.72	17.45	17.13	16.95	16.70	16.47	16.29	16.09	16.01	15.88	15.76
5.32	17.99	17.63	17.42	17.08	16.87	16.68	16.44	16.30	16.22	16.07	15.97
5.52	17.76	17.43	17.19	16.98	16.67	16.53	16.33	16.14	16.00	15.86	15.72
5.72	17.84	17.55	17.25	16.99	16.81	16.58	16.36	16.24	16.12	15.98	15.88
5.92	18.23	17.91	17.63	17.38	17.08	16.84	16.76	16.54	16.34	16.19	16.08
6.12	17.77	17.48	17.25	16.92	16.69	16.43	16.31	16.12	15.95	15.84	15.67
6.32	17.75	17.49	17.08	16.91	16.71	16.44	16.21	16.10	15.99	15.81	15.64
6.52	18.01	17.73	17.41	17.14	16.90	16.70	16.47	16.29	16.07	15.98	15.79
6.72	18.17	17.73	17.53	17.29	16.99	16.75	16.55	16.35	16.22	16.09	15.92
6.92	17.95	17.64	17.36	17.10	16.80	16.60	16.40	16.18	16.04	15.87	15.73
7.12	17.85	17.57	17.19	16.92	16.69	16.40	16.18	15.94	15.80	15.61	15.43
7.32	18.37	18.05	17.72	17.52	17.19	16.99	16.78	16.54	16.45	16.27	16.13
7.52	17.89	17.55	17.25	16.99	16.74	16.48	16.28	16.08	15.93	15.76	15.68
7.72	17.72	17.43	17.07	16.84	16.60	16.36	16.14	15.92	15.81	15.67	15.53
7.92	18.22	17.93	17.61	17.32	17.03	16.80	16.56	16.42	16.19	16.09	15.91
8.12	18.19	17.81	17.53	17.27	16.98	16.72	16.50	16.31	16.19	16.00	15.93
8.32	18.07	17.78	17.42	17.16	16.98	16.68	16.48	16.28	16.09	15.93	15.75
8.52	18.06	17.68	17.37	17.12	16.84	16.52	16.32	16.16	16.01	15.82	15.72
8.72	18.10	17.73	17.38	17.15	16.79	16.57	16.28	16.15	15.95	15.79	15.69
8.92	18.13	17.83	17.46	17.20	16.95	16.70	16.50	16.28	16.13	15.98	15.77
9.12	18.38	18.06	17.70	17.40	17.14	16.96	16.68	16.49	16.39	16.19	16.02
9.32	18.37	18.01	17.69	17.50	17.15	16.89	16.67	16.52	16.33	16.16	16.09
9.52	18.77	18.37	18.05	17.74	17.46	17.16	16.92	16.76	16.57	16.39	16.27
9.72	18.36	18.00	17.61	17.40	17.17	16.83	16.59	16.37	16.27	16.07	15.94
9.92	18.37	18.08	17.73	17.43	17.13	16.85	16.61	16.41	16.28	16.15	15.99
10.12	18.26	17.88	17.53	17.25	16.93	16.72	16.44	16.24	16.04	15.90	15.78
10.32	18.43	18.11	17.75	17.53	17.21	16.93	16.77	16.60	16.37	16.23	16.06
10.52	18.10	17.70	17.36	17.07	16.82	16.54	16.36	16.12	15.95	15.82	15.65
10.72	17.86	17.53	17.22	16.96	16.64	16.37	16.15	15.96	15.81	15.63	15.45
10.92	18.43	18.07	17.73	17.46	17.15	16.92	16.65	16.42	16.27	16.20	15.97
11.12	18.15	17.77	17.50	17.21	16.94	16.69	16.43	16.21	16.06	15.90	15.70
11.32	18.24	17.86	17.51	17.15	16.93	16.66	16.37	16.22	16.04	15.88	15.73
11.52	18.33	17.96	17.51	17.23	16.99	16.75	16.46	16.20	16.09	15.88	15.70
11.72	17.85	17.51	17.13	16.88	16.64	16.35	16.11	15.90	15.69	15.53	15.39

11.92	17.45	17.10	16.74	16.43	16.17	15.92	15.64	15.45	15.28	15.12	14.97
12.12	17.70	17.33	17.00	16.67	16.34	16.07	15.86	15.63	15.47	15.26	15.10
12.32	18.22	17.80	17.45	17.18	16.86	16.59	16.34	16.14	15.89	15.77	15.62
12.52	18.78	18.40	18.01	17.83	17.45	17.19	16.88	16.63	16.49	16.28	16.12
12.72	18.36	17.94	17.64	17.34	16.98	16.77	16.51	16.38	16.16	15.90	15.74
12.92	18.28	17.93	17.54	17.25	16.95	16.65	16.46	16.23	16.10	15.96	15.76
13.12	18.12	17.84	17.42	17.13	16.82	16.57	16.31	16.12	15.94	15.76	15.65
13.32	18.27	17.98	17.59	17.31	17.00	16.78	16.56	16.35	16.17	16.01	15.85
13.52	18.47	18.18	17.84	17.53	17.23	16.94	16.69	16.43	16.27	16.11	15.89
13.72	18.86	18.51	18.08	17.81	17.52	17.18	16.97	16.77	16.56	16.43	16.18
13.92	18.62	18.24	17.86	17.54	17.26	16.94	16.74	16.47	16.35	16.11	16.02
14	17.90	17.57	17.20	16.89	16.59	16.36	16.14	15.85	15.69	15.58	15.39
14.08	18.01	17.72	17.36	17.05	16.77	16.50	16.26	16.04	15.81	15.76	15.56
14.17	18.04	17.69	17.35	17.04	16.72	16.38	16.25	15.99	15.81	15.61	15.38
14.27	18.37	18.08	17.67	17.37	17.09	16.81	16.61	16.48	16.24	16.07	15.88
14.38	18.31	17.96	17.61	17.30	16.95	16.75	16.46	16.23	16.07	15.86	15.74
14.49	17.99	17.65	17.22	17.00	16.66	16.46	16.25	16.02	15.84	15.66	15.56
14.61	18.17	17.82	17.45	17.20	16.87	16.63	16.40	16.17	16.05	15.83	15.65
14.75	18.27	17.99	17.58	17.29	16.94	16.70	16.46	16.19	16.00	15.85	15.66
14.89	18.38	18.01	17.70	17.36	17.08	16.80	16.52	16.39	16.21	16.08	15.90
15.05	18.89	18.54	18.14	17.86	17.55	17.24	17.06	16.75	16.66	16.41	16.25
15.21	18.20	17.81	17.42	17.07	16.76	16.56	16.23	16.07	15.91	15.69	15.54
15.39	18.13	17.74	17.43	17.14	16.78	16.54	16.29	16.06	15.96	15.73	15.60
15.59	18.19	17.80	17.51	17.20	16.81	16.61	16.30	16.10	15.97	15.78	15.65
15.8	17.91	17.52	17.22	16.87	16.55	16.32	16.07	15.87	15.70	15.51	15.36
16.02	18.25	17.88	17.54	17.23	16.91	16.64	16.38	16.17	16.02	15.86	15.65
16.27	18.74	18.40	18.03	17.68	17.40	17.06	16.84	16.52	16.38	16.22	16.04
16.53	18.44	18.08	17.64	17.37	17.06	16.80	16.56	16.34	16.15	15.91	15.75
16.81	18.39	18.04	17.62	17.32	17.00	16.73	16.53	16.25	16.10	15.88	15.77
17.12	18.13	17.76	17.33	17.07	16.76	16.43	16.19	16.07	15.86	15.67	15.44
17.45	18.45	18.06	17.71	17.39	17.02	16.82	16.57	16.35	16.15	15.97	15.80
17.81	18.80	18.45	18.03	17.76	17.41	17.14	16.92	16.66	16.44	16.21	16.09
18.19	18.67	18.38	17.99	17.59	17.30	17.02	16.75	16.49	16.30	16.09	15.92
18.6	18.15	17.76	17.41	17.10	16.87	16.48	16.30	16.04	15.89	15.63	15.54
19.05	18.26	17.91	17.53	17.17	16.93	16.62	16.41	16.17	16.01	15.85	15.67
19.53	18.72	18.37	17.98	17.62	17.28	17.02	16.78	16.52	16.34	16.12	15.93
20.05	18.29	17.86	17.52	17.18	16.87	16.63	16.39	16.13	15.98	15.81	15.60
20.61	18.60	18.21	17.81	17.54	17.18	16.96	16.67	16.38	16.24	16.04	15.88
21.22	18.29	17.95	17.53	17.21	16.98	16.64	16.43	16.14	15.99	15.81	15.62
21.87	18.18	17.80	17.47	17.11	16.82	16.52	16.24	16.05	15.84	15.67	15.54
22.57	18.63	18.26	17.94	17.57	17.28	16.95	16.68	16.45	16.29	16.14	15.98
23.33	18.46	18.17	17.75	17.44	17.12	16.79	16.54	16.29	16.14	15.93	15.75
24.15	18.94	18.60	18.23	17.89	17.61	17.26	16.99	16.79	16.56	16.36	16.19
25.03	18.86	18.47	18.13	17.77	17.44	17.09	16.85	16.60	16.40	16.17	15.97
25.98	18.40	17.96	17.64	17.34	17.02	16.69	16.50	16.23	16.04	15.86	15.72
27.01	18.37	18.07	17.66	17.39	17.04	16.80	16.56	16.33	16.16	15.94	15.82
28.11	18.46	18.08	17.67	17.38	17.11	16.75	16.48	16.19	16.06	15.86	15.73
29.3	18.89	18.51	18.18	17.82	17.47	17.20	16.89	16.60	16.42	16.22	16.13
30.59	18.54	18.15	17.80	17.44	17.15	16.90	16.58	16.34	16.16	15.95	15.78
31.98	18.87	18.38	18.06	17.75	17.40	17.10	16.83	16.56	16.38	16.15	15.99

33.47	19.11	18.70	18.30	17.99	17.62	17.35	17.04	16.84	16.60	16.37	16.16
35.09	19.16	18.72	18.34	18.03	17.64	17.39	17.10	16.88	16.65	16.39	16.26
36.83	19.40	18.98	18.58	18.22	17.90	17.59	17.27	17.02	16.79	16.54	16.32
38.71	19.64	19.29	18.81	18.47	18.14	17.81	17.55	17.29	17.08	16.88	16.66
40.73	19.60	19.24	18.83	18.39	18.07	17.79	17.44	17.20	17.05	16.78	16.58
42.92	20.44	20.03	19.61	19.26	18.88	18.56	18.24	17.92	17.72	17.44	17.29
45.27	19.54	19.15	18.71	18.35	18.04	17.66	17.41	17.18	16.83	16.68	16.54
47.81	19.91	19.51	19.05	18.66	18.36	18.00	17.77	17.49	17.31	17.09	16.91
50.55	20.01	19.59	19.17	18.80	18.52	18.16	17.86	17.54	17.30	17.10	16.87
53.51	20.13	19.72	19.33	19.00	18.67	18.35	18.02	17.77	17.57	17.33	17.17
56.69	20.63	20.22	19.78	19.44	19.09	18.71	18.40	18.09	17.82	17.57	17.38
60.13	20.43	19.98	19.66	19.26	18.93	18.56	18.27	17.99	17.76	17.51	17.29
63.84	20.78	20.34	19.96	19.53	19.17	18.83	18.56	18.24	18.00	17.77	17.57
67.83	21.07	20.69	20.26	19.87	19.55	19.16	18.84	18.58	18.35	18.06	17.84
72.15	21.21	20.79	20.37	19.98	19.63	19.27	18.98	18.72	18.49	18.22	17.95
76.8	21.11	20.69	20.26	19.87	19.55	19.21	18.83	18.55	18.28	18.00	17.74
81.81	21.43	21.00	20.59	20.23	19.78	19.44	19.13	18.80	18.53	18.28	18.04
87.22	21.51	21.14	20.67	20.31	19.95	19.51	19.22	18.87	18.62	18.36	18.15
93.06	22.44	22.01	21.57	21.18	20.74	20.42	20.01	19.72	19.42	19.14	18.92
99.35	23.18	22.75	22.28	21.90	21.47	21.09	20.71	20.40	20.13	19.83	19.59
106.13	22.83	22.32	21.96	21.58	21.18	20.78	20.49	20.18	19.82	19.53	19.27
113.45	23.19	22.72	22.38	21.88	21.47	21.09	20.69	20.34	20.09	19.74	19.45
121.34	22.88	22.43	21.99	21.59	21.19	20.82	20.49	20.15	19.92	19.61	19.33
129.86	23.64	23.19	22.74	22.34	21.96	21.55	21.21	20.89	20.54	20.22	19.97
139.04	24.66	24.20	23.71	23.28	22.86	22.43	22.05	21.64	21.33	21.01	20.72
148.94	25.08	24.59	24.15	23.75	23.27	22.87	22.52	22.09	21.78	21.49	21.15
159.62	24.83	24.43	23.92	23.44	23.04	22.61	22.22	21.87	21.50	21.17	20.88
171.14	25.15	24.77	24.23	23.83	23.36	22.91	22.62	22.21	21.85	21.54	21.19
183.56	25.27	24.81	24.35	23.97	23.49	23.09	22.71	22.28	21.99	21.69	21.32
196.96	26.37	25.87	25.40	24.96	24.51	24.11	23.67	23.28	22.90	22.46	22.13
211.41	26.91	26.39	25.93	25.50	25.02	24.48	24.13	23.72	23.37	22.93	22.62
226.99	28.10	27.59	27.11	26.63	26.14	25.70	25.25	24.82	24.42	24.02	23.66
243.8	27.67	27.17	26.62	26.14	25.66	25.28	24.82	24.32	24.02	23.57	23.25
261.93	28.65	28.16	27.63	27.15	26.67	26.21	25.73	25.29	24.89	24.44	24.12
281.48	28.93	28.47	27.91	27.43	26.91	26.45	26.01	25.54	25.06	24.71	24.28
302.56	29.73	29.23	28.66	28.16	27.64	27.21	26.75	26.29	25.83	25.39	24.95
325.3	30.11	29.56	29.05	28.52	28.06	27.58	27.09	26.60	26.17	25.73	25.31
349.83	29.76	29.24	28.70	28.23	27.74	27.21	26.74	26.27	25.87	25.41	25.00
376.28	31.13	30.61	29.97	29.59	29.01	28.52	28.02	27.50	27.01	26.58	26.09
404.81	31.35	30.75	30.22	29.74	29.18	28.64	28.14	27.66	27.17	26.71	26.26
435.58	31.88	31.38	30.80	30.32	29.80	29.25	28.70	28.18	27.71	27.26	26.81
468.76	32.89	32.32	31.77	31.18	30.70	30.07	29.54	29.03	28.54	28.05	27.52
504.55	33.32	32.78	32.21	31.67	31.13	30.55	30.00	29.51	28.97	28.41	27.96
543.15	33.51	32.94	32.29	31.81	31.21	30.70	30.17	29.62	29.13	28.55	28.02
584.78	33.99	33.48	32.90	32.33	31.74	31.18	30.68	30.10	29.55	29.05	28.54
629.68	34.77	34.20	33.54	32.99	32.38	31.81	31.26	30.71	30.18	29.60	29.08
678.1	34.74	34.12	33.58	32.95	32.32	31.76	31.21	30.65	30.07	29.49	28.95
730.33	35.43	34.83	34.21	33.68	33.10	32.46	31.89	31.32	30.80	30.20	29.62
786.65	35.45	34.87	34.34	33.74	33.13	32.54	32.00	31.43	30.91	30.31	29.75
847.4	36.16	35.49	34.91	34.37	33.71	33.11	32.47	31.87	31.30	30.75	30.13

912.92	35.84	35.25	34.61	34.04	33.42	32.82	32.22	31.66	31.05	30.48	29.90
983.58	35.68	35.14	34.50	33.94	33.32	32.72	32.13	31.54	30.97	30.39	29.78
1059.78	36.06	35.49	34.86	34.23	33.64	33.01	32.43	31.80	31.26	30.63	30.07
1141.98	35.82	35.30	34.70	34.10	33.52	32.91	32.32	31.65	31.12	30.52	29.92
1230.62	36.33	35.79	35.12	34.56	33.93	33.33	32.73	32.10	31.47	30.92	30.33
1326.23	37.05	36.43	35.80	35.20	34.52	33.95	33.30	32.73	32.10	31.49	30.92
1429.34	37.13	36.51	35.91	35.33	34.68	33.98	33.41	32.82	32.22	31.60	30.98
1540.54	36.87	36.39	35.66	35.13	34.43	33.82	33.22	32.60	32.02	31.43	30.76
1660.48	37.45	36.81	36.17	35.58	34.96	34.32	33.69	33.09	32.46	31.86	31.24
1789.83	37.45	36.89	36.22	35.61	35.00	34.37	33.71	33.12	32.56	31.96	31.37
1929.34	37.85	37.20	36.64	36.03	35.30	34.70	34.04	33.31	32.77	32.14	31.58
2079.8	36.92	36.25	35.65	35.04	34.39	33.79	33.21	32.59	31.97	31.37	30.75
2242.08	37.78	37.16	36.55	35.92	35.24	34.59	33.93	33.30	32.77	32.08	31.53
2417.1	37.16	36.62	35.92	35.34	34.69	34.05	33.40	32.75	32.17	31.60	30.98
2605.85	37.11	36.54	35.84	35.28	34.63	34.04	33.43	32.77	32.21	31.63	30.97
2809.43	36.97	36.40	35.73	35.04	34.45	33.89	33.18	32.63	31.96	31.39	30.74
3028.99	35.96	35.36	34.68	34.14	33.55	32.90	32.27	31.71	31.04	30.50	29.89
3265.79	36.30	35.63	35.13	34.51	33.86	33.24	32.60	31.98	31.40	30.83	30.19
3521.18	36.19	35.58	34.95	34.42	33.77	33.10	32.54	31.94	31.36	30.74	30.16
3796.62	36.72	36.17	35.48	34.87	34.21	33.62	33.00	32.39	31.84	31.20	30.58

Wavelength (nm) / Time (ps)	615.09	616.50	617.91	619.32	620.72	622.13	623.54	624.95	626.36	627.77	629.18
-3.28	0.12	0.08	0.06	0.11	0.10	0.13	0.07	0.10	0.05	0.07	0.02
-2.78	0.00	0.02	0.04	0.00	-0.02	0.00	0.04	-0.02	0.00	0.04	0.02
-2.28	0.07	0.01	-0.01	-0.03	0.00	-0.02	-0.04	-0.07	-0.07	-0.05	-0.03
-1.78	0.12	0.10	0.13	0.16	0.10	0.14	0.12	0.11	0.10	0.06	0.10
-1.28	0.08	0.11	0.05	0.10	0.07	0.10	0.10	0.08	0.13	0.12	0.12
-0.78	-0.12	-0.09	-0.12	-0.10	-0.10	-0.09	-0.09	-0.10	-0.09	-0.15	-0.11
-0.28	-0.26	-0.24	-0.20	-0.18	-0.16	-0.18	-0.12	-0.09	-0.06	-0.04	-0.03
0.22	-0.02	0.01	0.05	-0.05	0.01	-0.07	-0.07	-0.02	-0.06	-0.06	-0.09
0.32	0.27	0.21	0.20	0.21	0.19	0.11	0.15	0.15	0.12	0.09	0.10
0.42	0.25	0.18	0.20	0.19	0.21	0.17	0.17	0.20	0.21	0.15	0.12
0.52	-0.04	-0.02	-0.06	-0.07	0.02	0.00	-0.04	0.00	-0.05	0.03	0.03
0.62	0.01	-0.05	-0.02	-0.05	-0.05	-0.07	-0.04	-0.04	0.00	-0.11	-0.03
0.72	0.11	0.13	0.12	0.10	0.14	0.20	0.18	0.12	0.14	0.14	0.21
0.77	0.22	0.26	0.23	0.19	0.17	0.15	0.17	0.20	0.16	0.18	0.09
0.82	-0.11	-0.13	-0.13	-0.16	-0.08	-0.06	-0.13	-0.12	-0.13	-0.08	-0.08
0.87	-0.21	-0.20	-0.20	-0.21	-0.16	-0.17	-0.14	-0.15	-0.11	-0.17	-0.08
0.92	0.15	0.09	0.14	0.18	0.15	0.11	0.17	0.12	0.17	0.21	0.20
0.97	0.24	0.22	0.24	0.24	0.27	0.22	0.26	0.22	0.20	0.20	0.18
1.02	0.12	0.05	0.12	0.10	0.12	0.08	-0.02	-0.02	0.03	0.00	0.00
1.07	0.07	0.02	0.05	0.10	0.09	0.06	0.05	0.04	0.00	0.06	0.06
1.12	-0.08	-0.05	-0.03	-0.04	0.01	-0.01	0.01	-0.05	-0.02	-0.01	0.01
1.17	0.15	0.17	0.18	0.19	0.19	0.20	0.15	0.19	0.17	0.20	0.21
1.22	0.15	0.12	0.11	0.17	0.15	0.07	0.05	0.08	0.07	0.09	0.07
1.27	0.16	0.09	0.15	0.12	0.13	0.13	0.09	0.13	0.10	0.10	0.14
1.32	0.12	0.07	0.09	0.06	0.09	0.10	0.14	0.09	0.06	0.10	0.14

1.37	0.11	0.05	0.09	0.09	0.10	0.07	0.01	-0.02	-0.01	0.03	0.10
1.42	0.20	0.23	0.13	0.17	0.19	0.15	0.11	0.09	0.14	0.15	0.12
1.47	0.14	0.11	0.08	0.05	0.05	0.01	0.05	0.02	0.07	0.02	0.03
1.52	0.13	-0.03	-0.03	-0.04	-0.15	-0.17	-0.22	-0.18	-0.23	-0.19	-0.19
1.57	2.26	2.09	1.85	1.80	1.60	1.52	1.28	1.16	1.08	1.06	0.94
1.62	9.06	8.86	8.59	8.34	8.12	7.83	7.55	7.21	6.94	6.66	6.43
1.67	19.04	19.05	19.03	19.06	19.03	18.96	18.87	18.71	18.58	18.49	18.36
1.72	20.24	20.50	20.68	20.94	21.15	21.36	21.57	21.71	21.90	22.17	22.36
1.77	17.94	18.16	18.40	18.67	18.99	19.24	19.50	19.81	20.08	20.38	20.68
1.82	15.92	16.22	16.49	16.82	17.04	17.41	17.69	18.01	18.27	18.65	19.02
1.87	14.37	14.53	14.79	15.06	15.27	15.54	15.81	16.16	16.48	16.82	17.14
1.92	13.69	13.82	14.04	14.28	14.55	14.77	15.06	15.24	15.55	15.93	16.28
1.97	13.22	13.40	13.56	13.70	13.95	14.13	14.39	14.67	14.88	15.17	15.54
2.02	13.14	13.22	13.36	13.54	13.79	13.92	14.05	14.33	14.62	14.88	15.16
2.07	13.43	13.49	13.59	13.79	13.93	14.15	14.27	14.49	14.75	15.02	15.30
2.12	12.78	12.89	13.03	13.21	13.42	13.57	13.76	13.90	14.08	14.40	14.76
2.17	12.95	12.98	13.08	13.21	13.33	13.47	13.64	13.83	14.09	14.25	14.54
2.22	13.01	13.06	13.14	13.24	13.40	13.52	13.69	13.83	14.06	14.29	14.49
2.27	13.29	13.34	13.42	13.49	13.61	13.78	13.90	14.07	14.25	14.47	14.64
2.32	13.80	13.78	13.90	13.99	14.06	14.19	14.30	14.48	14.60	14.89	15.14
2.37	13.59	13.61	13.68	13.79	13.90	14.03	14.14	14.22	14.41	14.62	14.86
2.42	13.49	13.56	13.55	13.67	13.78	13.90	13.98	14.11	14.27	14.51	14.74
2.47	13.66	13.66	13.73	13.84	13.90	14.01	14.13	14.23	14.39	14.55	14.75
2.52	13.42	13.42	13.54	13.55	13.64	13.76	13.78	13.96	14.11	14.25	14.51
2.57	13.87	13.85	13.83	13.94	14.00	14.11	14.18	14.25	14.41	14.58	14.80
2.62	14.00	13.92	13.97	14.02	14.07	14.19	14.30	14.38	14.52	14.69	14.81
2.67	13.71	13.71	13.76	13.87	13.93	13.95	14.10	14.21	14.43	14.54	14.70
2.72	13.56	13.58	13.63	13.67	13.70	13.75	13.89	14.04	14.20	14.39	14.49
2.77	13.89	13.87	13.94	14.07	14.11	14.22	14.30	14.36	14.55	14.68	14.88
2.82	14.09	14.05	14.05	14.07	14.21	14.25	14.29	14.39	14.49	14.63	14.81
2.87	14.18	14.17	14.21	14.22	14.28	14.30	14.45	14.48	14.68	14.76	14.92
2.92	14.34	14.30	14.33	14.40	14.40	14.47	14.52	14.57	14.72	14.87	15.04
2.97	14.52	14.52	14.55	14.57	14.63	14.65	14.80	14.77	14.91	15.10	15.28
3.02	14.09	14.14	14.12	14.19	14.26	14.31	14.44	14.49	14.57	14.73	14.98
3.07	14.12	14.08	14.10	14.15	14.19	14.28	14.34	14.40	14.47	14.64	14.79
3.12	15.00	14.99	14.95	14.98	14.98	15.07	15.04	15.14	15.27	15.41	15.52
3.17	14.49	14.39	14.40	14.42	14.39	14.53	14.59	14.61	14.70	14.85	15.01
3.22	14.35	14.34	14.39	14.41	14.40	14.49	14.57	14.64	14.71	14.89	15.07
3.27	14.15	14.12	14.12	14.20	14.22	14.29	14.35	14.39	14.48	14.63	14.80
3.32	14.75	14.64	14.68	14.68	14.71	14.67	14.73	14.80	14.91	15.01	15.12
3.37	14.60	14.54	14.59	14.56	14.58	14.59	14.69	14.76	14.87	14.99	15.10
3.42	14.53	14.50	14.49	14.46	14.53	14.51	14.62	14.67	14.71	14.87	15.00
3.47	14.52	14.46	14.43	14.45	14.47	14.50	14.57	14.67	14.70	14.80	14.96
3.52	14.84	14.79	14.78	14.76	14.83	14.87	14.90	15.02	15.07	15.20	15.35
3.57	14.41	14.41	14.39	14.42	14.42	14.53	14.46	14.59	14.71	14.76	14.89
3.62	14.85	14.74	14.79	14.77	14.82	14.83	14.82	14.89	14.90	15.05	15.22
3.67	15.01	14.96	14.94	14.99	15.05	15.04	15.06	15.12	15.16	15.31	15.44
3.72	15.27	15.17	15.15	15.08	15.10	15.05	15.07	15.13	15.18	15.27	15.41
3.92	14.62	14.53	14.53	14.54	14.55	14.59	14.65	14.72	14.73	14.89	14.95
4.12	15.12	15.03	14.96	14.91	14.91	15.01	14.97	15.06	15.09	15.18	15.26

4.32	15.23	15.13	15.08	15.11	15.05	15.10	15.13	15.18	15.32	15.41	15.48
4.52	15.72	15.64	15.59	15.57	15.52	15.44	15.47	15.53	15.63	15.67	15.72
4.72	15.73	15.64	15.58	15.55	15.52	15.48	15.44	15.50	15.50	15.63	15.71
4.92	15.48	15.36	15.35	15.29	15.35	15.32	15.34	15.36	15.41	15.53	15.73
5.12	15.79	15.59	15.58	15.58	15.54	15.49	15.51	15.58	15.65	15.78	15.88
5.32	15.87	15.77	15.73	15.69	15.66	15.67	15.63	15.69	15.71	15.86	15.98
5.52	15.64	15.58	15.52	15.44	15.46	15.43	15.47	15.48	15.57	15.65	15.72
5.72	15.75	15.70	15.66	15.58	15.59	15.54	15.67	15.72	15.70	15.85	15.94
5.92	15.98	15.89	15.84	15.85	15.74	15.76	15.76	15.75	15.82	15.93	16.03
6.12	15.60	15.48	15.43	15.44	15.39	15.43	15.38	15.36	15.47	15.57	15.65
6.32	15.58	15.50	15.45	15.40	15.43	15.43	15.38	15.41	15.53	15.54	15.66
6.52	15.75	15.62	15.56	15.54	15.52	15.52	15.54	15.59	15.63	15.64	15.78
6.72	15.85	15.73	15.75	15.71	15.68	15.64	15.63	15.72	15.74	15.83	15.86
6.92	15.68	15.57	15.51	15.44	15.47	15.43	15.44	15.45	15.50	15.62	15.71
7.12	15.31	15.17	15.08	15.05	15.00	15.00	14.98	14.96	15.03	15.10	15.22
7.32	16.09	15.93	15.85	15.75	15.77	15.73	15.77	15.79	15.84	15.87	16.01
7.52	15.53	15.42	15.36	15.34	15.29	15.30	15.31	15.34	15.34	15.46	15.52
7.72	15.45	15.37	15.28	15.32	15.25	15.28	15.28	15.29	15.36	15.46	15.65
7.92	15.84	15.71	15.63	15.60	15.62	15.60	15.63	15.63	15.67	15.70	15.85
8.12	15.80	15.61	15.50	15.55	15.49	15.48	15.48	15.49	15.50	15.58	15.69
8.32	15.67	15.53	15.47	15.42	15.40	15.40	15.40	15.41	15.52	15.64	15.69
8.52	15.58	15.50	15.41	15.43	15.44	15.37	15.41	15.54	15.50	15.56	15.68
8.72	15.56	15.45	15.35	15.34	15.34	15.27	15.30	15.33	15.32	15.37	15.54
8.92	15.78	15.62	15.56	15.53	15.57	15.51	15.51	15.54	15.66	15.75	15.82
9.12	15.97	15.91	15.81	15.74	15.78	15.76	15.78	15.78	15.86	15.92	16.09
9.32	15.94	15.85	15.76	15.71	15.73	15.73	15.81	15.76	15.79	15.92	16.05
9.52	16.16	16.02	15.92	15.91	15.86	15.84	15.84	15.87	15.93	16.03	16.17
9.72	15.78	15.71	15.69	15.64	15.62	15.58	15.60	15.64	15.66	15.76	15.94
9.92	15.90	15.75	15.68	15.69	15.67	15.67	15.67	15.71	15.74	15.85	16.01
10.12	15.70	15.57	15.46	15.38	15.41	15.39	15.42	15.48	15.49	15.56	15.65
10.32	15.96	15.86	15.77	15.71	15.69	15.65	15.67	15.63	15.69	15.77	15.92
10.52	15.61	15.45	15.36	15.34	15.37	15.32	15.38	15.36	15.45	15.55	15.65
10.72	15.32	15.22	15.22	15.14	15.13	15.08	15.05	15.16	15.19	15.29	15.35
10.92	15.89	15.74	15.71	15.64	15.64	15.61	15.63	15.65	15.71	15.82	15.91
11.12	15.63	15.58	15.48	15.48	15.47	15.45	15.48	15.49	15.57	15.64	15.80
11.32	15.62	15.48	15.41	15.39	15.37	15.36	15.36	15.40	15.52	15.62	15.63
11.52	15.60	15.48	15.37	15.35	15.30	15.26	15.26	15.34	15.36	15.41	15.59
11.72	15.30	15.18	15.09	15.07	15.08	15.09	15.10	15.14	15.16	15.25	15.40
11.92	14.87	14.74	14.65	14.64	14.58	14.60	14.54	14.61	14.69	14.68	14.84
12.12	15.02	14.89	14.81	14.80	14.70	14.74	14.69	14.73	14.80	14.81	14.95
12.32	15.50	15.41	15.31	15.24	15.18	15.19	15.23	15.28	15.27	15.41	15.51
12.52	16.07	15.89	15.77	15.71	15.71	15.66	15.67	15.65	15.70	15.73	15.91
12.72	15.72	15.53	15.54	15.48	15.49	15.47	15.48	15.56	15.58	15.66	15.80
12.92	15.59	15.54	15.50	15.43	15.44	15.38	15.39	15.44	15.55	15.61	15.73
13.12	15.51	15.35	15.28	15.27	15.28	15.24	15.16	15.28	15.36	15.40	15.53
13.32	15.77	15.75	15.68	15.60	15.63	15.64	15.63	15.76	15.76	15.82	16.00
13.52	15.82	15.69	15.57	15.55	15.55	15.53	15.56	15.55	15.63	15.64	15.80
13.72	16.06	15.95	15.90	15.80	15.76	15.73	15.76	15.77	15.81	15.90	16.01
13.92	15.90	15.76	15.70	15.71	15.67	15.60	15.67	15.64	15.70	15.79	15.93
14	15.30	15.25	15.21	15.12	15.11	15.13	15.10	15.21	15.22	15.31	15.49

14.08	15.45	15.36	15.30	15.29	15.32	15.27	15.27	15.29	15.35	15.45	15.60
14.17	15.33	15.21	15.17	15.05	15.04	14.97	14.99	14.99	15.03	15.14	15.23
14.27	15.82	15.69	15.61	15.54	15.48	15.55	15.59	15.54	15.67	15.72	15.81
14.38	15.57	15.47	15.42	15.41	15.34	15.34	15.35	15.40	15.44	15.52	15.63
14.49	15.40	15.34	15.29	15.28	15.21	15.22	15.23	15.26	15.36	15.44	15.50
14.61	15.63	15.43	15.43	15.36	15.38	15.38	15.40	15.45	15.54	15.66	15.75
14.75	15.56	15.46	15.39	15.36	15.33	15.35	15.41	15.40	15.44	15.52	15.62
14.89	15.78	15.66	15.61	15.60	15.59	15.54	15.54	15.56	15.61	15.70	15.80
15.05	16.12	16.04	15.96	15.91	15.87	15.81	15.84	15.85	15.92	16.06	16.14
15.21	15.47	15.34	15.22	15.24	15.20	15.15	15.18	15.17	15.24	15.34	15.48
15.39	15.53	15.39	15.30	15.30	15.27	15.27	15.30	15.33	15.37	15.48	15.59
15.59	15.58	15.39	15.34	15.31	15.32	15.31	15.29	15.34	15.42	15.53	15.66
15.8	15.25	15.08	15.10	15.06	15.04	15.00	15.04	15.08	15.17	15.30	15.37
16.02	15.56	15.41	15.36	15.31	15.27	15.26	15.26	15.29	15.32	15.43	15.57
16.27	15.88	15.81	15.62	15.56	15.53	15.60	15.59	15.56	15.57	15.70	15.81
16.53	15.64	15.51	15.42	15.41	15.36	15.35	15.35	15.38	15.45	15.47	15.60
16.81	15.65	15.54	15.47	15.44	15.39	15.34	15.38	15.43	15.47	15.59	15.65
17.12	15.40	15.28	15.17	15.13	15.13	15.16	15.10	15.16	15.25	15.33	15.48
17.45	15.72	15.57	15.47	15.42	15.44	15.40	15.46	15.47	15.51	15.61	15.76
17.81	15.99	15.82	15.72	15.61	15.66	15.60	15.59	15.62	15.67	15.76	15.80
18.19	15.83	15.63	15.58	15.51	15.48	15.49	15.46	15.45	15.55	15.62	15.64
18.6	15.33	15.25	15.19	15.12	15.13	15.15	15.15	15.14	15.18	15.33	15.42
19.05	15.66	15.55	15.45	15.43	15.36	15.39	15.45	15.50	15.52	15.62	15.83
19.53	15.80	15.67	15.60	15.60	15.55	15.47	15.51	15.55	15.60	15.65	15.76
20.05	15.53	15.42	15.34	15.30	15.20	15.27	15.24	15.32	15.41	15.50	15.60
20.61	15.75	15.63	15.54	15.55	15.47	15.44	15.45	15.50	15.57	15.64	15.72
21.22	15.53	15.44	15.32	15.30	15.29	15.33	15.25	15.35	15.40	15.54	15.66
21.87	15.37	15.23	15.19	15.12	15.10	15.07	15.06	15.17	15.25	15.31	15.36
22.57	15.85	15.73	15.65	15.61	15.54	15.50	15.55	15.59	15.68	15.76	15.85
23.33	15.60	15.44	15.33	15.28	15.25	15.26	15.22	15.23	15.23	15.30	15.41
24.15	16.08	15.93	15.83	15.73	15.64	15.66	15.64	15.67	15.72	15.73	15.86
25.03	15.90	15.70	15.57	15.51	15.52	15.41	15.46	15.50	15.52	15.57	15.65
25.98	15.61	15.42	15.47	15.39	15.40	15.34	15.38	15.43	15.59	15.64	15.72
27.01	15.70	15.61	15.56	15.56	15.56	15.60	15.58	15.64	15.66	15.79	15.92
28.11	15.59	15.42	15.32	15.33	15.25	15.22	15.26	15.21	15.32	15.43	15.53
29.3	15.94	15.80	15.78	15.74	15.68	15.65	15.66	15.69	15.70	15.77	15.92
30.59	15.63	15.48	15.40	15.37	15.31	15.33	15.26	15.30	15.39	15.44	15.51
31.98	15.90	15.71	15.66	15.55	15.57	15.55	15.52	15.56	15.61	15.71	15.79
33.47	16.10	15.88	15.79	15.78	15.69	15.62	15.64	15.64	15.66	15.81	15.83
35.09	16.08	15.91	15.89	15.80	15.74	15.68	15.67	15.66	15.74	15.85	15.96
36.83	16.21	16.06	15.96	15.89	15.84	15.81	15.79	15.82	15.82	15.91	16.06
38.71	16.49	16.32	16.20	16.13	16.12	16.04	16.02	16.06	16.04	16.14	16.25
40.73	16.38	16.27	16.07	16.05	16.02	15.96	15.89	15.91	15.98	16.02	16.19
42.92	17.09	16.89	16.78	16.68	16.60	16.56	16.51	16.50	16.51	16.54	16.58
45.27	16.40	16.23	16.08	16.02	15.96	15.91	15.90	15.91	15.95	16.04	16.10
47.81	16.75	16.60	16.49	16.42	16.42	16.35	16.36	16.36	16.36	16.48	16.58
50.55	16.75	16.55	16.43	16.30	16.28	16.25	16.10	16.18	16.18	16.20	16.26
53.51	16.96	16.79	16.62	16.51	16.46	16.46	16.42	16.40	16.38	16.49	16.53
56.69	17.23	17.06	16.88	16.79	16.72	16.60	16.62	16.58	16.56	16.60	16.64
60.13	17.11	16.96	16.82	16.74	16.64	16.58	16.51	16.48	16.50	16.57	16.63

63.84	17.43	17.24	17.09	17.00	16.89	16.78	16.77	16.80	16.77	16.83	16.83
67.83	17.62	17.49	17.37	17.24	17.19	17.11	17.10	17.03	17.05	17.08	17.15
72.15	17.81	17.58	17.49	17.32	17.24	17.11	17.10	17.08	17.10	17.09	17.12
76.8	17.56	17.36	17.24	17.11	17.05	16.97	16.85	16.83	16.76	16.81	16.87
81.81	17.76	17.57	17.45	17.33	17.22	17.06	17.02	16.97	16.99	17.02	17.02
87.22	17.93	17.72	17.53	17.39	17.30	17.27	17.20	17.14	17.09	17.08	17.15
93.06	18.66	18.37	18.24	18.16	18.03	17.89	17.73	17.66	17.66	17.67	17.70
99.35	19.32	19.02	18.87	18.73	18.60	18.43	18.37	18.23	18.14	18.12	18.12
106.13	19.05	18.77	18.62	18.46	18.23	18.18	17.99	17.96	17.95	17.80	17.82
113.45	19.28	19.00	18.81	18.60	18.48	18.35	18.25	18.15	18.10	18.03	18.02
121.34	19.03	18.83	18.71	18.53	18.38	18.30	18.21	18.06	17.94	18.02	18.01
129.86	19.78	19.48	19.27	19.11	19.00	18.83	18.71	18.52	18.53	18.49	18.52
139.04	20.38	20.15	19.87	19.70	19.50	19.30	19.15	19.02	18.99	18.89	18.82
148.94	20.90	20.64	20.34	20.14	19.97	19.82	19.63	19.48	19.36	19.32	19.29
159.62	20.54	20.29	19.98	19.79	19.59	19.40	19.27	19.15	19.01	18.92	18.90
171.14	20.89	20.54	20.34	20.08	19.94	19.71	19.54	19.34	19.30	19.21	19.11
183.56	21.03	20.76	20.53	20.30	20.16	19.90	19.69	19.53	19.41	19.36	19.31
196.96	21.92	21.58	21.32	21.04	20.83	20.61	20.40	20.18	20.07	19.97	19.90
211.41	22.29	21.91	21.66	21.30	21.09	20.87	20.66	20.52	20.40	20.25	20.10
226.99	23.31	22.93	22.62	22.30	22.01	21.78	21.57	21.38	21.17	21.04	20.87
243.8	22.91	22.53	22.15	21.85	21.68	21.40	21.13	20.91	20.71	20.57	20.48
261.93	23.67	23.32	22.98	22.64	22.41	22.13	21.89	21.61	21.38	21.28	21.10
281.48	23.82	23.54	23.16	22.85	22.52	22.22	21.95	21.71	21.56	21.30	21.14
302.56	24.53	24.11	23.72	23.44	23.18	22.86	22.57	22.29	22.07	21.84	21.75
325.3	24.89	24.48	24.13	23.75	23.43	23.10	22.88	22.56	22.28	22.07	21.93
349.83	24.61	24.19	23.80	23.50	23.12	22.85	22.50	22.23	22.01	21.81	21.60
376.28	25.75	25.27	24.83	24.45	24.15	23.83	23.44	23.15	22.84	22.61	22.40
404.81	25.86	25.35	25.01	24.64	24.25	23.93	23.53	23.24	22.94	22.65	22.53
435.58	26.40	25.91	25.51	25.06	24.72	24.33	23.97	23.64	23.34	23.11	22.93
468.76	27.08	26.57	26.12	25.71	25.32	24.94	24.49	24.15	23.90	23.62	23.31
504.55	27.47	27.02	26.55	26.15	25.68	25.29	24.90	24.53	24.14	23.88	23.60
543.15	27.54	27.06	26.62	26.18	25.74	25.31	24.84	24.54	24.24	23.92	23.60
584.78	28.01	27.51	26.99	26.60	26.10	25.69	25.29	24.89	24.56	24.20	23.85
629.68	28.58	28.03	27.51	27.02	26.65	26.20	25.70	25.28	24.91	24.59	24.27
678.1	28.38	27.85	27.33	26.85	26.38	25.88	25.45	25.07	24.63	24.29	23.94
730.33	29.12	28.54	28.07	27.57	27.10	26.68	26.16	25.75	25.34	25.01	24.61
786.65	29.28	28.70	28.17	27.70	27.24	26.76	26.30	25.91	25.47	25.14	24.79
847.4	29.62	28.98	28.53	28.00	27.50	27.01	26.59	26.11	25.67	25.34	24.98
912.92	29.36	28.80	28.29	27.77	27.22	26.78	26.26	25.85	25.46	25.09	24.67
983.58	29.21	28.68	28.07	27.62	27.18	26.62	26.16	25.71	25.30	24.92	24.56
1059.78	29.52	28.94	28.40	27.91	27.35	26.81	26.33	25.90	25.49	25.12	24.66
1141.98	29.40	28.78	28.25	27.80	27.29	26.74	26.28	25.79	25.44	25.04	24.66
1230.62	29.70	29.16	28.58	28.12	27.54	27.07	26.56	26.08	25.63	25.23	24.78
1326.23	30.30	29.69	29.16	28.56	28.09	27.60	27.04	26.56	26.10	25.66	25.30
1429.34	30.41	29.78	29.25	28.74	28.14	27.61	27.06	26.62	26.21	25.70	25.34
1540.54	30.21	29.59	29.05	28.50	27.92	27.46	26.96	26.45	26.02	25.61	25.19
1660.48	30.67	30.04	29.48	28.95	28.42	27.90	27.39	26.89	26.33	25.92	25.52
1789.83	30.75	30.16	29.53	29.08	28.48	27.97	27.45	26.94	26.49	26.10	25.68
1929.34	30.94	30.29	29.70	29.18	28.59	28.10	27.56	26.94	26.54	26.10	25.66
2079.8	30.19	29.63	29.07	28.49	27.99	27.43	26.96	26.48	26.09	25.61	25.19

2242.08	30.85	30.22	29.65	29.11	28.61	28.03	27.54	26.99	26.51	26.09	25.62
2417.1	30.34	29.70	29.13	28.58	28.07	27.56	27.01	26.49	26.01	25.56	25.14
2605.85	30.41	29.73	29.24	28.66	28.14	27.56	27.12	26.60	26.07	25.72	25.31
2809.43	30.17	29.58	28.96	28.48	27.94	27.41	26.89	26.34	25.88	25.54	25.08
3028.99	29.38	28.71	28.20	27.69	27.10	26.61	26.09	25.69	25.18	24.77	24.40
3265.79	29.65	29.01	28.41	27.89	27.38	26.84	26.34	25.80	25.35	24.92	24.54
3521.18	29.63	28.96	28.39	27.93	27.40	26.84	26.33	25.86	25.37	24.95	24.57
3796.62	29.99	29.41	28.83	28.30	27.74	27.19	26.71	26.16	25.73	25.34	24.92

Wavelength (nm) / Time (ps)	630.58	631.99	633.40	634.81	636.22	637.63	639.04	640.44	641.85	643.26	644.67
-3.28	0.01	0.02	0.00	0.10	-0.01	-0.01	0.01	0.01	-0.03	-0.07	-0.08
-2.78	0.02	0.00	-0.02	-0.02	0.06	0.01	0.01	0.08	0.01	0.10	0.06
-2.28	-0.07	-0.05	-0.10	-0.14	-0.14	-0.08	-0.09	-0.12	-0.10	-0.12	-0.16
-1.78	0.09	0.12	0.13	0.11	0.07	0.05	0.08	0.07	0.09	0.07	0.00
-1.28	0.12	0.15	0.11	0.11	0.13	0.20	0.10	0.05	0.09	0.13	0.19
-0.78	-0.12	-0.14	-0.09	-0.14	-0.09	-0.17	-0.15	-0.15	-0.07	-0.11	-0.09
-0.28	-0.01	0.02	0.05	0.06	0.05	0.05	0.13	0.17	0.17	0.11	0.20
0.22	-0.04	-0.12	-0.09	-0.09	-0.08	-0.04	-0.09	-0.12	-0.16	-0.11	-0.12
0.32	0.09	0.08	0.09	0.03	-0.01	-0.09	-0.05	-0.04	-0.05	-0.06	0.04
0.42	0.17	0.14	0.12	0.14	0.10	0.10	0.09	0.13	0.15	0.11	0.11
0.52	0.06	0.03	0.03	0.02	0.04	0.08	0.03	0.04	0.02	0.09	0.15
0.62	-0.07	-0.06	-0.04	-0.02	-0.08	-0.05	-0.03	-0.01	0.00	-0.05	0.00
0.72	0.19	0.15	0.22	0.20	0.17	0.21	0.18	0.29	0.27	0.23	0.29
0.77	0.15	0.12	0.09	0.13	0.09	0.15	0.06	0.07	0.13	0.09	0.11
0.82	-0.07	-0.06	-0.08	-0.04	-0.13	-0.10	-0.11	-0.06	-0.05	-0.08	-0.05
0.87	-0.10	-0.06	-0.03	-0.05	-0.02	-0.07	-0.04	-0.04	0.03	0.01	0.04
0.92	0.17	0.20	0.15	0.15	0.16	0.25	0.18	0.17	0.16	0.19	0.17
0.97	0.21	0.21	0.17	0.14	0.17	0.12	0.14	0.14	0.10	0.11	0.10
1.02	-0.06	-0.04	-0.11	-0.05	-0.07	-0.06	-0.07	-0.09	-0.13	-0.13	-0.13
1.07	0.12	0.00	0.05	0.05	0.11	0.14	0.08	0.06	0.02	0.05	0.08
1.12	0.03	0.03	-0.01	0.04	0.02	0.03	0.01	0.10	0.08	0.08	0.10
1.17	0.21	0.23	0.13	0.16	0.06	0.16	0.28	0.25	0.21	0.18	0.21
1.22	0.03	-0.01	0.03	-0.09	-0.08	0.05	-0.05	-0.01	-0.10	-0.14	-0.04
1.27	0.11	0.10	0.06	0.12	0.12	0.10	0.02	0.04	0.08	0.09	-0.01
1.32	0.11	0.14	0.12	0.10	0.10	0.14	0.11	0.16	0.12	0.12	0.14
1.37	0.06	0.05	0.01	0.03	-0.01	0.03	0.05	0.09	0.01	-0.01	-0.02
1.42	0.15	0.07	0.09	0.12	0.07	0.09	0.13	0.08	0.10	0.07	0.10
1.47	-0.01	0.05	0.03	-0.01	-0.05	-0.01	0.01	-0.03	0.02	0.00	0.00
1.52	-0.21	-0.18	-0.24	-0.22	-0.24	-0.19	-0.16	-0.28	-0.24	-0.29	-0.21
1.57	0.89	0.79	0.76	0.66	0.62	0.64	0.60	0.50	0.33	0.26	0.25
1.62	6.21	5.97	5.76	5.61	5.45	5.29	5.11	4.87	4.70	4.55	4.38
1.67	18.18	17.97	17.86	17.75	17.68	17.59	17.47	17.33	17.21	17.01	16.80
1.72	22.43	22.65	22.69	22.86	23.05	23.25	23.36	23.46	23.60	23.69	23.81
1.77	20.90	21.19	21.42	21.76	21.99	22.33	22.61	22.85	23.11	23.43	23.67
1.82	19.31	19.64	20.02	20.33	20.73	21.10	21.36	21.73	22.08	22.40	22.84
1.87	17.49	17.79	18.15	18.60	18.92	19.29	19.68	20.11	20.46	20.82	21.23
1.92	16.60	16.96	17.23	17.61	17.99	18.50	18.81	19.27	19.66	19.99	20.42

1.97	15.91	16.18	16.54	16.85	17.26	17.65	18.07	18.45	18.81	19.21	19.69
2.02	15.48	15.79	16.11	16.47	16.82	17.22	17.51	17.95	18.35	18.81	19.14
2.07	15.55	15.82	16.14	16.46	16.82	17.19	17.51	17.93	18.29	18.67	19.14
2.12	15.00	15.28	15.53	15.84	16.15	16.60	16.95	17.30	17.63	18.04	18.41
2.17	14.78	15.05	15.35	15.68	16.01	16.34	16.64	17.06	17.42	17.74	18.16
2.22	14.76	15.03	15.32	15.56	15.89	16.21	16.59	16.97	17.33	17.58	18.03
2.27	14.88	15.18	15.51	15.80	16.11	16.39	16.70	17.08	17.46	17.94	18.28
2.32	15.32	15.50	15.75	16.06	16.39	16.71	17.08	17.38	17.71	18.05	18.48
2.37	15.01	15.30	15.51	15.89	16.11	16.45	16.75	17.15	17.43	17.82	18.19
2.42	14.91	15.15	15.34	15.71	16.01	16.32	16.60	17.02	17.38	17.69	18.17
2.47	14.96	15.22	15.40	15.70	15.99	16.29	16.59	16.88	17.33	17.65	18.00
2.52	14.75	14.93	15.16	15.48	15.69	16.07	16.33	16.68	17.04	17.41	17.74
2.57	14.94	15.15	15.34	15.63	15.94	16.20	16.50	16.83	17.15	17.43	17.93
2.62	15.02	15.21	15.46	15.72	16.00	16.33	16.53	16.86	17.27	17.54	17.91
2.67	14.89	15.12	15.32	15.63	15.92	16.26	16.47	16.85	17.13	17.50	17.86
2.72	14.69	14.90	15.11	15.43	15.74	16.03	16.25	16.56	16.84	17.25	17.64
2.77	15.06	15.26	15.45	15.74	15.99	16.30	16.51	16.87	17.30	17.59	17.93
2.82	14.92	15.16	15.37	15.60	15.83	16.11	16.34	16.73	17.10	17.39	17.73
2.87	15.09	15.28	15.52	15.70	16.00	16.25	16.54	16.80	17.12	17.38	17.71
2.92	15.18	15.32	15.53	15.79	16.04	16.34	16.51	16.84	17.21	17.57	17.80
2.97	15.42	15.57	15.79	16.01	16.30	16.54	16.82	17.09	17.41	17.67	18.04
3.02	15.11	15.33	15.54	15.74	15.96	16.30	16.53	16.90	17.18	17.46	17.79
3.07	14.94	15.06	15.34	15.55	15.80	16.02	16.28	16.58	16.85	17.20	17.54
3.12	15.68	15.84	16.03	16.22	16.52	16.79	17.06	17.31	17.62	17.97	18.30
3.17	15.14	15.30	15.40	15.65	15.85	16.05	16.30	16.61	16.96	17.19	17.50
3.22	15.19	15.30	15.54	15.79	16.02	16.30	16.47	16.72	17.07	17.39	17.75
3.27	14.91	15.12	15.29	15.51	15.71	15.96	16.20	16.43	16.80	17.12	17.40
3.32	15.28	15.36	15.51	15.78	16.01	16.30	16.38	16.66	16.93	17.31	17.66
3.37	15.22	15.40	15.60	15.80	16.00	16.25	16.47	16.74	16.99	17.34	17.65
3.42	15.09	15.38	15.41	15.63	15.86	16.09	16.33	16.65	16.96	17.21	17.50
3.47	15.08	15.18	15.37	15.56	15.82	16.01	16.29	16.51	16.88	17.10	17.46
3.52	15.47	15.59	15.71	16.03	16.25	16.47	16.66	16.97	17.25	17.56	17.89
3.57	15.03	15.22	15.34	15.55	15.82	16.04	16.25	16.62	16.83	17.12	17.40
3.62	15.28	15.40	15.64	15.83	16.01	16.24	16.47	16.69	16.96	17.26	17.57
3.67	15.57	15.67	15.86	16.03	16.24	16.47	16.70	16.99	17.25	17.51	17.79
3.72	15.47	15.63	15.79	15.96	16.15	16.43	16.58	16.84	17.12	17.32	17.65
3.92	15.04	15.19	15.37	15.54	15.75	16.04	16.24	16.40	16.73	16.89	17.26
4.12	15.45	15.52	15.68	15.83	16.01	16.28	16.51	16.73	16.90	17.19	17.46
4.32	15.63	15.72	15.92	16.22	16.34	16.54	16.77	16.99	17.35	17.54	17.87
4.52	15.80	15.87	16.06	16.22	16.45	16.62	16.82	17.07	17.26	17.56	17.85
4.72	15.79	15.89	16.00	16.16	16.34	16.50	16.69	16.94	17.17	17.37	17.65
4.92	15.81	15.84	15.98	16.19	16.45	16.61	16.80	17.11	17.33	17.56	17.89
5.12	16.01	16.12	16.31	16.45	16.68	16.89	17.04	17.35	17.58	17.85	18.18
5.32	16.09	16.19	16.26	16.49	16.63	16.89	17.09	17.34	17.53	17.87	18.10
5.52	15.83	15.99	16.08	16.24	16.41	16.62	16.92	17.19	17.42	17.66	17.92
5.72	16.03	16.19	16.30	16.51	16.78	16.93	17.11	17.40	17.68	17.89	18.24
5.92	16.08	16.20	16.33	16.48	16.69	16.89	17.07	17.34	17.67	17.87	18.12
6.12	15.66	15.88	16.02	16.25	16.45	16.65	16.82	17.12	17.37	17.66	17.93
6.32	15.80	15.91	15.97	16.22	16.38	16.58	16.76	16.99	17.30	17.51	17.83
6.52	15.84	15.99	16.22	16.38	16.53	16.70	16.90	17.10	17.38	17.61	17.93

6.72	16.05	16.16	16.33	16.49	16.72	16.89	17.05	17.34	17.62	17.84	18.14
6.92	15.76	15.91	16.03	16.24	16.40	16.61	16.79	17.00	17.33	17.54	17.87
7.12	15.28	15.44	15.51	15.60	15.83	15.97	16.15	16.56	16.73	16.98	17.22
7.32	16.06	16.21	16.27	16.50	16.75	16.89	17.09	17.31	17.52	17.74	18.11
7.52	15.68	15.79	15.86	16.19	16.28	16.54	16.78	16.98	17.28	17.52	17.80
7.72	15.72	15.92	15.98	16.17	16.33	16.63	16.90	17.11	17.41	17.66	17.88
7.92	16.00	16.17	16.26	16.38	16.51	16.89	17.08	17.34	17.55	17.79	18.14
8.12	15.82	15.94	16.04	16.27	16.44	16.63	16.82	17.06	17.31	17.51	17.86
8.32	15.75	15.87	16.03	16.27	16.46	16.64	16.87	17.08	17.44	17.68	18.00
8.52	15.84	15.96	16.17	16.35	16.44	16.71	16.91	17.18	17.51	17.78	18.05
8.72	15.67	15.85	15.96	16.17	16.35	16.59	16.82	17.04	17.26	17.56	17.92
8.92	16.01	16.10	16.25	16.44	16.65	16.87	17.05	17.33	17.58	17.94	18.24
9.12	16.21	16.28	16.39	16.59	16.82	17.01	17.29	17.50	17.79	18.07	18.42
9.32	16.27	16.36	16.43	16.66	16.91	17.06	17.29	17.56	17.84	18.08	18.34
9.52	16.25	16.35	16.47	16.73	16.89	17.18	17.41	17.63	17.75	18.06	18.43
9.72	16.02	16.15	16.31	16.52	16.72	16.97	17.20	17.52	17.77	18.11	18.30
9.92	16.03	16.17	16.33	16.45	16.71	16.92	17.14	17.39	17.69	17.92	18.18
10.12	15.79	15.93	16.08	16.27	16.46	16.71	16.90	17.18	17.44	17.74	18.03
10.32	16.01	16.11	16.28	16.46	16.63	16.87	17.02	17.33	17.61	17.81	18.10
10.52	15.69	15.82	16.06	16.25	16.46	16.68	16.91	17.15	17.45	17.66	18.01
10.72	15.47	15.53	15.78	15.96	16.12	16.34	16.56	16.82	17.08	17.39	17.64
10.92	16.05	16.12	16.24	16.51	16.71	16.93	17.13	17.35	17.64	17.98	18.26
11.12	15.92	16.05	16.24	16.36	16.58	16.80	17.01	17.29	17.55	17.85	18.12
11.32	15.78	15.92	16.07	16.27	16.49	16.73	16.92	17.22	17.46	17.79	18.03
11.52	15.67	15.81	15.91	16.04	16.26	16.55	16.70	17.03	17.27	17.50	17.79
11.72	15.52	15.61	15.87	16.01	16.21	16.46	16.70	16.96	17.30	17.57	17.84
11.92	14.95	15.10	15.27	15.49	15.63	15.83	16.08	16.36	16.57	16.84	17.13
12.12	15.12	15.27	15.43	15.58	15.73	15.98	16.16	16.49	16.79	17.03	17.35
12.32	15.65	15.72	15.84	16.11	16.30	16.54	16.76	16.94	17.26	17.60	17.97
12.52	15.98	16.03	16.14	16.40	16.60	16.76	17.01	17.30	17.53	17.85	18.15
12.72	15.94	16.05	16.18	16.41	16.63	16.86	17.11	17.42	17.60	17.91	18.22
12.92	15.79	15.86	16.09	16.24	16.50	16.71	16.88	17.11	17.45	17.75	18.01
13.12	15.62	15.74	15.92	16.17	16.36	16.58	16.73	17.05	17.35	17.60	17.91
13.32	16.11	16.29	16.40	16.62	16.88	17.06	17.33	17.61	17.93	18.14	18.47
13.52	15.90	16.03	16.19	16.38	16.63	16.82	16.97	17.30	17.57	17.83	18.24
13.72	16.07	16.20	16.34	16.57	16.72	16.94	17.15	17.39	17.65	17.94	18.27
13.92	15.96	16.21	16.39	16.52	16.71	16.95	17.22	17.52	17.81	18.02	18.42
14	15.55	15.76	15.87	16.14	16.31	16.54	16.76	17.07	17.36	17.69	17.92
14.08	15.76	15.87	16.02	16.18	16.47	16.77	16.98	17.23	17.53	17.79	18.09
14.17	15.38	15.46	15.62	15.79	15.97	16.29	16.48	16.80	17.07	17.26	17.61
14.27	15.97	16.08	16.26	16.44	16.72	16.89	17.05	17.42	17.70	17.95	18.30
14.38	15.78	15.88	16.00	16.24	16.46	16.67	16.90	17.18	17.45	17.69	18.03
14.49	15.58	15.77	15.90	16.12	16.35	16.57	16.70	17.01	17.22	17.58	17.86
14.61	15.83	16.00	16.24	16.41	16.69	16.88	17.12	17.40	17.71	17.98	18.36
14.75	15.82	16.01	16.16	16.31	16.51	16.74	17.00	17.35	17.62	17.81	18.17
14.89	15.95	16.07	16.22	16.42	16.62	16.92	17.14	17.40	17.68	18.01	18.32
15.05	16.30	16.39	16.56	16.73	16.98	17.24	17.47	17.67	17.95	18.27	18.60
15.21	15.61	15.74	15.92	16.13	16.35	16.54	16.78	17.05	17.38	17.62	17.93
15.39	15.73	15.90	16.08	16.24	16.42	16.72	16.92	17.24	17.54	17.79	18.08
15.59	15.77	15.87	16.03	16.30	16.50	16.75	16.98	17.23	17.56	17.81	18.10

15.8	15.43	15.57	15.72	16.02	16.22	16.39	16.62	16.84	17.17	17.47	17.76
16.02	15.61	15.73	15.92	16.14	16.33	16.60	16.79	17.06	17.26	17.61	17.94
16.27	15.95	16.05	16.19	16.45	16.65	16.85	17.02	17.39	17.59	17.86	18.22
16.53	15.71	15.85	16.00	16.26	16.43	16.68	16.85	17.12	17.42	17.73	18.02
16.81	15.76	15.97	16.05	16.26	16.47	16.72	17.05	17.28	17.56	17.78	18.19
17.12	15.63	15.73	15.90	16.13	16.32	16.57	16.79	17.06	17.39	17.66	17.99
17.45	15.86	16.05	16.20	16.40	16.60	16.90	17.06	17.41	17.69	17.90	18.32
17.81	15.90	16.01	16.22	16.39	16.58	16.83	17.05	17.36	17.57	17.86	18.20
18.19	15.75	15.88	16.05	16.26	16.44	16.70	16.86	17.20	17.45	17.75	17.98
18.6	15.53	15.67	15.78	16.02	16.28	16.50	16.76	16.99	17.23	17.57	17.91
19.05	15.88	16.03	16.26	16.46	16.66	16.85	17.17	17.39	17.70	17.99	18.31
19.53	15.85	15.99	16.21	16.40	16.60	16.84	17.00	17.31	17.65	17.92	18.21
20.05	15.70	15.85	16.04	16.29	16.53	16.75	16.88	17.27	17.49	17.78	18.15
20.61	15.86	16.03	16.14	16.41	16.58	16.81	17.05	17.36	17.61	17.85	18.27
21.22	15.78	15.87	16.01	16.25	16.48	16.68	16.95	17.23	17.50	17.83	18.17
21.87	15.56	15.67	15.83	16.01	16.29	16.55	16.77	17.04	17.31	17.62	17.89
22.57	16.02	16.15	16.30	16.55	16.73	16.94	17.17	17.45	17.78	18.09	18.38
23.33	15.59	15.72	15.80	15.96	16.16	16.41	16.65	16.89	17.24	17.47	17.77
24.15	15.96	16.13	16.25	16.42	16.57	16.82	17.05	17.40	17.66	17.91	18.15
25.03	15.74	15.88	16.06	16.27	16.40	16.68	16.90	17.19	17.55	17.76	18.07
25.98	15.89	15.98	16.20	16.48	16.68	16.97	17.07	17.40	17.67	17.94	18.32
27.01	16.09	16.25	16.42	16.57	16.82	17.14	17.35	17.66	17.93	18.23	18.59
28.11	15.59	15.69	15.94	16.14	16.28	16.56	16.77	17.05	17.35	17.60	17.94
29.3	16.05	16.21	16.35	16.49	16.66	16.97	17.22	17.52	17.82	18.09	18.41
30.59	15.62	15.77	15.95	16.07	16.33	16.53	16.78	17.04	17.29	17.59	17.91
31.98	15.88	16.02	16.18	16.42	16.67	16.86	17.08	17.28	17.57	17.87	18.25
33.47	15.89	16.02	16.21	16.42	16.61	16.80	17.03	17.22	17.54	17.84	18.16
35.09	16.01	16.10	16.26	16.52	16.72	16.88	17.10	17.40	17.69	17.92	18.27
36.83	16.17	16.32	16.43	16.68	16.83	17.05	17.31	17.63	17.90	18.20	18.53
38.71	16.35	16.39	16.54	16.74	16.93	17.19	17.33	17.61	17.86	18.13	18.46
40.73	16.21	16.32	16.44	16.68	16.86	17.04	17.28	17.52	17.87	18.06	18.34
42.92	16.72	16.89	16.92	17.14	17.25	17.46	17.72	18.01	18.17	18.41	18.68
45.27	16.21	16.30	16.41	16.63	16.86	17.11	17.28	17.50	17.82	18.11	18.51
47.81	16.65	16.81	16.96	17.03	17.25	17.49	17.63	17.96	18.24	18.47	18.75
50.55	16.40	16.54	16.61	16.79	16.97	17.13	17.38	17.65	17.89	18.15	18.48
53.51	16.67	16.70	16.81	16.98	17.16	17.39	17.63	17.87	18.10	18.28	18.57
56.69	16.79	16.86	16.97	17.14	17.31	17.58	17.79	18.02	18.27	18.55	18.77
60.13	16.73	16.75	16.81	17.03	17.28	17.48	17.59	17.79	18.02	18.32	18.62
63.84	16.94	17.02	17.13	17.31	17.41	17.59	17.82	18.04	18.24	18.51	18.76
67.83	17.21	17.27	17.45	17.59	17.70	17.95	18.13	18.34	18.59	18.84	19.10
72.15	17.14	17.20	17.35	17.46	17.69	17.85	18.06	18.22	18.47	18.66	18.98
76.8	16.93	16.97	17.05	17.29	17.38	17.56	17.77	17.93	18.15	18.43	18.58
81.81	17.10	17.06	17.21	17.39	17.50	17.74	17.88	18.08	18.30	18.54	18.82
87.22	17.18	17.30	17.38	17.56	17.65	17.86	17.97	18.23	18.51	18.59	18.90
93.06	17.66	17.74	17.78	17.95	18.06	18.20	18.34	18.53	18.79	19.01	19.21
99.35	18.13	18.23	18.27	18.40	18.47	18.65	18.72	18.95	19.15	19.37	19.56
106.13	17.85	17.88	17.98	18.01	18.16	18.22	18.43	18.59	18.79	18.91	19.11
113.45	18.04	18.12	18.14	18.23	18.40	18.45	18.65	18.83	18.94	19.17	19.35
121.34	18.00	18.01	18.06	18.12	18.29	18.48	18.62	18.75	18.97	19.02	19.25
129.86	18.49	18.50	18.49	18.54	18.70	18.81	18.97	19.11	19.18	19.34	19.59

139.04	18.84	18.79	18.80	18.86	18.84	19.03	19.08	19.25	19.44	19.53	19.67
148.94	19.21	19.22	19.21	19.25	19.40	19.45	19.56	19.65	19.85	19.99	20.11
159.62	18.83	18.80	18.77	18.82	18.97	19.03	19.09	19.22	19.29	19.45	19.60
171.14	19.02	19.02	18.96	19.05	19.10	19.22	19.26	19.39	19.51	19.58	19.81
183.56	19.29	19.26	19.24	19.31	19.34	19.37	19.47	19.55	19.73	19.90	19.95
196.96	19.80	19.69	19.69	19.74	19.71	19.79	19.86	19.99	20.01	20.21	20.35
211.41	20.04	20.01	19.96	20.01	20.01	19.98	20.02	20.13	20.23	20.31	20.52
226.99	20.73	20.69	20.66	20.61	20.57	20.56	20.56	20.63	20.81	20.83	21.00
243.8	20.40	20.25	20.23	20.13	20.15	20.16	20.18	20.28	20.31	20.33	20.49
261.93	20.99	20.90	20.72	20.64	20.64	20.64	20.70	20.73	20.78	20.79	20.79
281.48	20.98	20.86	20.78	20.66	20.65	20.61	20.66	20.64	20.64	20.68	20.76
302.56	21.59	21.35	21.20	21.18	21.13	21.13	21.12	21.09	21.14	21.15	21.18
325.3	21.71	21.52	21.39	21.33	21.33	21.23	21.15	21.17	21.14	21.11	21.20
349.83	21.41	21.29	21.08	20.95	20.91	20.88	20.82	20.80	20.82	20.79	20.87
376.28	22.23	22.03	21.86	21.79	21.68	21.62	21.61	21.55	21.50	21.48	21.49
404.81	22.30	22.04	21.87	21.72	21.59	21.56	21.50	21.45	21.40	21.31	21.40
435.58	22.69	22.51	22.29	22.16	22.10	21.98	21.95	21.82	21.82	21.76	21.73
468.76	23.07	22.86	22.58	22.46	22.35	22.34	22.11	22.10	21.97	21.88	21.90
504.55	23.30	23.02	22.82	22.66	22.50	22.40	22.20	22.10	22.00	21.97	21.96
543.15	23.27	23.11	22.81	22.68	22.45	22.34	22.24	22.07	21.94	21.90	21.82
584.78	23.59	23.29	23.08	22.92	22.72	22.53	22.40	22.40	22.16	22.10	22.06
629.68	23.95	23.66	23.40	23.25	22.99	22.82	22.69	22.58	22.44	22.31	22.20
678.1	23.63	23.29	23.00	22.84	22.62	22.37	22.29	22.17	22.00	21.83	21.74
730.33	24.29	24.05	23.68	23.47	23.24	23.10	22.92	22.72	22.65	22.43	22.33
786.65	24.43	24.08	23.82	23.65	23.38	23.12	22.92	22.86	22.69	22.51	22.41
847.4	24.64	24.23	23.97	23.69	23.56	23.36	23.14	22.95	22.80	22.64	22.52
912.92	24.36	24.07	23.71	23.48	23.25	23.03	22.84	22.67	22.46	22.33	22.25
983.58	24.10	23.76	23.45	23.29	23.02	22.81	22.66	22.47	22.28	22.06	21.94
1059.78	24.35	23.96	23.68	23.43	23.18	22.96	22.72	22.53	22.35	22.20	22.02
1141.98	24.26	23.90	23.59	23.32	23.03	22.76	22.61	22.40	22.26	22.03	21.82
1230.62	24.42	24.04	23.66	23.41	23.17	22.93	22.74	22.50	22.32	22.09	21.99
1326.23	24.90	24.52	24.25	23.85	23.68	23.45	23.19	22.95	22.73	22.57	22.42
1429.34	24.95	24.55	24.15	23.91	23.73	23.44	23.17	22.97	22.76	22.57	22.32
1540.54	24.87	24.43	24.17	23.89	23.64	23.39	23.04	22.86	22.70	22.50	22.32
1660.48	25.13	24.77	24.42	24.01	23.76	23.54	23.24	23.08	22.84	22.60	22.46
1789.83	25.30	24.93	24.53	24.29	23.95	23.74	23.49	23.20	23.01	22.77	22.67
1929.34	25.23	24.87	24.48	24.21	23.88	23.58	23.30	23.11	22.94	22.73	22.47
2079.8	24.79	24.49	24.12	23.87	23.57	23.30	23.03	22.85	22.69	22.41	22.29
2242.08	25.26	24.82	24.46	24.19	23.89	23.69	23.40	23.11	22.87	22.68	22.51
2417.1	24.73	24.43	24.03	23.73	23.36	23.17	22.90	22.70	22.51	22.27	22.02
2605.85	24.94	24.54	24.16	23.89	23.60	23.35	23.18	22.94	22.66	22.50	22.30
2809.43	24.64	24.31	23.91	23.62	23.34	23.12	22.84	22.59	22.32	22.19	22.06
3028.99	23.92	23.59	23.27	23.02	22.72	22.44	22.19	22.04	21.78	21.61	21.47
3265.79	24.16	23.77	23.39	23.09	22.80	22.52	22.32	22.04	21.81	21.60	21.46
3521.18	24.22	23.81	23.49	23.19	22.92	22.66	22.37	22.12	21.95	21.74	21.50
3796.62	24.48	24.08	23.72	23.45	23.13	22.86	22.61	22.39	22.16	21.98	21.81

Time (ps) \ Wavelength (nm)	646.08	647.49	648.90	650.30	651.71	653.12	654.53	655.94	657.35	658.76	660.16
-3.28	-0.03	-0.02	-0.05	-0.07	0.01	-0.07	-0.15	-0.10	-0.11	-0.11	-0.12
-2.78	-0.01	0.01	0.05	0.06	0.09	-0.04	-0.02	0.02	0.07	0.09	0.04
-2.28	-0.10	-0.11	-0.11	-0.18	-0.14	-0.12	-0.07	-0.09	-0.10	-0.08	-0.09
-1.78	0.02	0.07	0.05	0.07	0.00	0.03	-0.01	0.02	0.09	0.02	0.04
-1.28	0.14	0.05	0.11	0.09	0.08	0.09	0.08	0.12	0.10	0.05	0.03
-0.78	-0.06	-0.05	-0.10	-0.01	-0.05	-0.02	-0.03	-0.11	-0.09	-0.06	-0.06
-0.28	0.15	0.21	0.16	0.14	0.16	0.20	0.32	0.22	0.20	0.18	0.24
0.22	-0.12	-0.16	-0.11	-0.11	-0.15	-0.08	-0.10	-0.07	-0.16	-0.09	-0.07
0.32	-0.03	-0.05	-0.09	-0.10	-0.14	-0.09	-0.11	-0.12	-0.18	-0.14	-0.17
0.42	0.15	0.15	0.10	0.11	0.11	0.04	0.01	0.04	-0.05	-0.07	-0.06
0.52	0.14	0.06	0.10	0.11	0.10	0.12	0.09	0.02	0.08	0.10	0.16
0.62	0.01	0.00	0.00	-0.05	0.01	-0.02	-0.09	-0.12	-0.18	-0.15	-0.15
0.72	0.17	0.17	0.12	0.24	0.10	0.15	-0.02	0.05	0.10	0.01	-0.01
0.77	0.06	-0.08	-0.15	-0.09	-0.10	0.05	-0.09	-0.13	-0.25	-0.18	-0.11
0.82	-0.06	-0.08	-0.12	-0.04	0.02	-0.07	-0.07	-0.03	0.02	0.08	0.03
0.87	-0.03	-0.01	0.03	0.03	0.00	0.08	0.03	0.06	0.05	0.04	0.08
0.92	0.19	0.14	0.09	0.06	0.06	0.03	0.02	0.00	0.03	0.05	0.06
0.97	0.16	0.03	0.04	-0.02	0.05	-0.02	-0.02	-0.12	-0.06	-0.11	-0.12
1.02	-0.23	-0.25	-0.20	-0.17	-0.20	-0.21	-0.20	-0.25	-0.16	-0.22	-0.17
1.07	0.08	0.11	0.10	0.03	0.10	-0.03	0.10	0.10	0.07	0.02	0.01
1.12	0.06	0.06	0.04	0.20	0.13	0.11	0.09	0.03	0.10	0.10	0.02
1.17	0.21	0.24	0.18	0.16	0.14	0.13	0.07	0.06	0.08	0.07	0.01
1.22	-0.12	-0.04	-0.12	-0.12	-0.13	-0.12	-0.18	-0.18	-0.11	-0.13	-0.11
1.27	0.04	0.01	0.01	-0.03	0.05	-0.05	-0.12	-0.04	-0.11	-0.11	-0.12
1.32	0.14	0.11	0.06	0.08	0.05	0.05	0.06	0.09	0.11	0.02	0.02
1.37	0.05	0.01	0.01	0.06	0.03	0.05	0.00	0.02	0.06	-0.04	-0.02
1.42	0.08	0.06	0.01	-0.01	0.01	0.03	-0.02	-0.13	-0.11	-0.07	-0.07
1.47	0.00	-0.03	-0.05	-0.02	-0.10	-0.08	-0.05	-0.07	-0.09	-0.20	-0.17
1.52	-0.32	-0.28	-0.28	-0.30	-0.25	-0.30	-0.21	-0.26	-0.25	-0.28	-0.17
1.57	0.28	0.21	0.05	0.01	0.01	0.00	-0.02	-0.01	-0.01	-0.07	-0.02
1.62	4.16	3.88	3.65	3.45	3.31	3.12	2.94	2.74	2.64	2.50	2.43
1.67	16.51	16.29	15.91	15.61	15.24	14.94	14.53	14.13	13.68	13.34	13.09
1.72	23.84	23.84	23.79	23.69	23.56	23.33	23.22	23.05	22.95	22.73	22.55
1.77	23.96	24.02	24.26	24.49	24.60	24.69	24.78	24.84	24.93	24.93	25.03
1.82	23.12	23.45	23.77	24.08	24.39	24.62	24.86	25.06	25.36	25.59	25.79
1.87	21.62	22.05	22.28	22.72	23.08	23.42	23.68	24.07	24.36	24.60	24.97
1.92	20.89	21.25	21.70	22.00	22.45	22.78	23.17	23.64	24.01	24.40	24.78
1.97	20.11	20.49	20.85	21.25	21.76	22.22	22.56	22.99	23.36	23.84	24.24
2.02	19.54	19.93	20.41	20.91	21.34	21.67	22.13	22.60	23.10	23.56	23.97
2.07	19.48	19.92	20.32	20.77	21.11	21.59	22.02	22.49	23.02	23.42	23.98
2.12	18.91	19.32	19.61	20.08	20.38	20.90	21.30	21.77	22.21	22.58	23.04
2.17	18.54	18.94	19.43	19.80	20.21	20.65	21.00	21.50	21.95	22.25	22.67
2.22	18.41	18.82	19.15	19.63	19.99	20.35	20.80	21.17	21.65	22.01	22.49
2.27	18.68	19.05	19.48	19.86	20.32	20.76	21.16	21.62	22.13	22.52	22.97
2.32	18.95	19.30	19.63	20.05	20.46	20.87	21.34	21.79	22.30	22.69	23.21
2.37	18.57	18.98	19.36	19.86	20.16	20.66	21.02	21.46	21.95	22.41	22.77
2.42	18.49	18.87	19.25	19.61	20.16	20.49	20.91	21.38	21.69	22.12	22.58

2.47	18.39	18.66	19.19	19.51	20.01	20.35	20.73	21.14	21.65	22.02	22.44
2.52	18.14	18.52	18.86	19.28	19.83	20.11	20.51	20.91	21.33	21.75	22.29
2.57	18.27	18.65	18.99	19.36	19.81	20.24	20.70	21.19	21.55	21.91	22.39
2.62	18.30	18.66	18.98	19.39	19.86	20.30	20.63	21.09	21.56	21.92	22.47
2.67	18.28	18.66	18.99	19.38	19.85	20.25	20.68	21.05	21.48	21.88	22.31
2.72	18.04	18.31	18.68	19.09	19.51	19.93	20.37	20.72	21.09	21.51	21.96
2.77	18.30	18.65	19.05	19.38	19.81	20.16	20.58	20.96	21.40	21.80	22.24
2.82	18.02	18.44	18.85	19.30	19.71	20.09	20.52	20.94	21.37	21.77	22.25
2.87	18.12	18.49	18.89	19.28	19.54	19.99	20.39	20.78	21.28	21.65	22.07
2.92	18.17	18.60	18.94	19.36	19.70	20.14	20.51	20.92	21.42	21.81	22.14
2.97	18.43	18.74	19.04	19.42	19.81	20.25	20.64	20.96	21.38	21.78	22.27
3.02	18.12	18.48	18.85	19.20	19.64	19.98	20.40	20.85	21.20	21.57	21.97
3.07	17.88	18.23	18.58	18.99	19.36	19.77	20.08	20.54	20.86	21.27	21.68
3.12	18.66	18.96	19.31	19.74	20.14	20.49	20.80	21.20	21.71	22.17	22.62
3.17	17.83	18.14	18.54	18.85	19.25	19.64	20.00	20.46	20.86	21.33	21.80
3.22	18.12	18.42	18.72	19.15	19.59	19.95	20.35	20.73	21.13	21.43	21.92
3.27	17.71	17.97	18.40	18.75	19.17	19.57	19.87	20.16	20.63	20.95	21.35
3.32	17.99	18.31	18.49	18.96	19.32	19.76	20.08	20.52	20.86	21.23	21.64
3.37	17.94	18.28	18.55	19.00	19.33	19.69	20.10	20.43	20.94	21.28	21.68
3.42	17.82	18.17	18.51	18.83	19.18	19.56	19.92	20.35	20.77	21.17	21.50
3.47	17.79	18.14	18.44	18.75	19.11	19.52	20.00	20.33	20.73	21.13	21.56
3.52	18.20	18.50	18.84	19.15	19.49	19.87	20.15	20.48	20.92	21.29	21.64
3.57	17.75	18.02	18.44	18.69	19.09	19.44	19.80	20.14	20.55	20.86	21.26
3.62	17.81	18.13	18.52	18.85	19.14	19.52	19.85	20.20	20.57	20.91	21.34
3.67	18.14	18.45	18.75	19.10	19.48	19.82	20.15	20.53	20.94	21.30	21.66
3.72	17.95	18.24	18.51	18.87	19.25	19.63	19.97	20.31	20.68	21.06	21.49
3.92	17.56	17.88	18.17	18.51	18.82	19.14	19.45	19.88	20.25	20.52	20.91
4.12	17.82	18.06	18.34	18.78	19.09	19.37	19.60	19.97	20.40	20.74	21.08
4.32	18.18	18.40	18.72	19.10	19.49	19.86	20.18	20.44	20.90	21.24	21.70
4.52	18.18	18.47	18.71	19.06	19.40	19.70	20.05	20.43	20.79	21.15	21.48
4.72	17.95	18.23	18.47	18.82	19.10	19.44	19.78	20.10	20.54	20.83	21.25
4.92	18.21	18.48	18.84	19.13	19.47	19.80	20.18	20.54	20.94	21.33	21.60
5.12	18.38	18.69	18.98	19.30	19.63	19.91	20.23	20.54	20.98	21.33	21.67
5.32	18.41	18.69	18.94	19.24	19.59	19.94	20.20	20.57	20.89	21.25	21.62
5.52	18.19	18.45	18.76	19.05	19.37	19.72	19.97	20.26	20.65	20.98	21.29
5.72	18.53	18.87	19.06	19.46	19.83	20.11	20.50	20.77	21.16	21.53	21.85
5.92	18.38	18.76	18.98	19.39	19.66	20.03	20.39	20.67	21.13	21.39	21.63
6.12	18.17	18.45	18.77	19.04	19.38	19.70	19.98	20.19	20.65	20.96	21.26
6.32	18.03	18.35	18.58	18.92	19.34	19.57	19.84	20.13	20.50	20.81	21.23
6.52	18.19	18.49	18.87	19.16	19.44	19.79	20.10	20.49	20.86	21.10	21.48
6.72	18.36	18.74	18.97	19.30	19.63	19.97	20.26	20.67	20.93	21.24	21.61
6.92	18.06	18.32	18.74	19.05	19.36	19.58	19.94	20.37	20.64	21.04	21.38
7.12	17.48	17.76	18.11	18.45	18.77	19.09	19.46	19.80	20.20	20.45	20.88
7.32	18.37	18.59	18.86	19.18	19.41	19.76	20.02	20.34	20.62	20.98	21.24
7.52	18.11	18.39	18.69	19.06	19.37	19.66	19.98	20.28	20.59	20.86	21.26
7.72	18.18	18.52	18.81	19.15	19.44	19.69	20.05	20.32	20.61	20.99	21.36
7.92	18.39	18.73	19.02	19.32	19.70	20.07	20.38	20.73	21.15	21.47	21.87
8.12	18.17	18.42	18.72	19.03	19.40	19.76	20.06	20.39	20.78	21.15	21.49
8.32	18.26	18.56	18.87	19.19	19.57	19.84	20.02	20.41	20.79	21.15	21.48
8.52	18.35	18.65	18.89	19.23	19.62	19.96	20.26	20.50	20.85	21.27	21.55

8.72	18.10	18.37	18.71	19.09	19.44	19.73	20.03	20.31	20.60	20.99	21.38
8.92	18.51	18.83	19.08	19.39	19.75	20.12	20.44	20.72	21.01	21.36	21.77
9.12	18.72	19.07	19.36	19.67	20.05	20.38	20.75	21.05	21.41	21.77	22.11
9.32	18.64	18.93	19.28	19.60	19.89	20.25	20.56	20.81	21.30	21.67	22.01
9.52	18.72	19.03	19.26	19.56	19.90	20.24	20.61	20.94	21.29	21.59	21.88
9.72	18.65	19.00	19.25	19.57	19.92	20.25	20.55	20.88	21.20	21.52	21.85
9.92	18.56	18.80	19.11	19.45	19.80	20.05	20.44	20.71	21.10	21.37	21.74
10.12	18.31	18.66	18.91	19.37	19.65	20.06	20.32	20.69	21.00	21.31	21.63
10.32	18.37	18.74	18.99	19.33	19.61	19.94	20.17	20.57	20.89	21.25	21.59
10.52	18.35	18.65	18.92	19.26	19.60	19.90	20.23	20.60	20.94	21.24	21.59
10.72	17.87	18.20	18.44	18.82	19.10	19.43	19.73	20.05	20.35	20.64	20.99
10.92	18.60	18.77	19.10	19.46	19.81	20.16	20.47	20.77	21.05	21.30	21.72
11.12	18.50	18.85	19.08	19.45	19.73	20.04	20.39	20.74	21.13	21.35	21.69
11.32	18.39	18.71	18.98	19.27	19.65	20.05	20.39	20.74	21.08	21.29	21.66
11.52	18.10	18.42	18.74	19.06	19.34	19.74	20.00	20.41	20.77	21.11	21.51
11.72	18.12	18.42	18.75	19.15	19.36	19.72	20.05	20.44	20.85	21.12	21.48
11.92	17.54	17.79	18.06	18.33	18.65	19.03	19.41	19.73	20.10	20.34	20.67
12.12	17.64	17.93	18.25	18.67	18.95	19.26	19.53	19.81	20.22	20.59	20.95
12.32	18.21	18.55	18.83	19.17	19.50	19.93	20.20	20.48	20.88	21.14	21.50
12.52	18.48	18.83	19.08	19.43	19.79	20.13	20.46	20.80	21.11	21.45	21.85
12.72	18.61	18.90	19.20	19.53	19.88	20.20	20.55	20.91	21.34	21.56	21.93
12.92	18.35	18.61	18.89	19.21	19.57	19.84	20.19	20.48	20.77	21.10	21.46
13.12	18.32	18.58	18.86	19.25	19.58	19.80	20.17	20.53	20.82	21.21	21.40
13.32	18.78	19.20	19.45	19.75	20.07	20.44	20.81	21.04	21.36	21.63	22.02
13.52	18.48	18.77	19.04	19.37	19.75	20.14	20.43	20.77	21.11	21.46	21.88
13.72	18.61	18.89	19.19	19.60	19.94	20.28	20.56	20.90	21.26	21.54	21.94
13.92	18.73	19.11	19.46	19.80	20.13	20.38	20.78	21.16	21.44	21.78	22.09
14	18.29	18.60	18.93	19.21	19.66	19.95	20.24	20.68	20.89	21.21	21.65
14.08	18.39	18.73	19.09	19.39	19.75	20.01	20.38	20.68	21.00	21.23	21.52
14.17	17.96	18.28	18.54	18.90	19.24	19.61	19.94	20.24	20.52	20.85	21.17
14.27	18.60	18.93	19.24	19.56	19.94	20.30	20.69	21.01	21.40	21.63	22.00
14.38	18.36	18.77	19.03	19.38	19.69	20.12	20.48	20.79	21.14	21.46	21.76
14.49	18.20	18.53	18.78	19.15	19.43	19.78	20.08	20.44	20.82	21.05	21.31
14.61	18.69	19.06	19.30	19.57	19.98	20.29	20.70	21.08	21.33	21.60	21.98
14.75	18.43	18.78	19.19	19.48	19.82	20.06	20.41	20.78	21.15	21.49	21.82
14.89	18.69	18.91	19.34	19.75	20.00	20.28	20.59	20.99	21.32	21.62	22.01
15.05	18.98	19.22	19.52	19.90	20.21	20.54	20.84	21.22	21.63	21.93	22.27
15.21	18.21	18.61	18.81	19.19	19.54	19.87	20.26	20.64	20.98	21.31	21.66
15.39	18.42	18.69	19.05	19.35	19.71	19.93	20.26	20.62	21.01	21.23	21.57
15.59	18.54	18.84	19.06	19.45	19.69	20.06	20.41	20.83	21.12	21.41	21.69
15.8	18.10	18.37	18.73	19.01	19.37	19.74	19.99	20.37	20.71	20.94	21.28
16.02	18.23	18.63	18.77	19.15	19.50	19.88	20.26	20.60	20.96	21.26	21.53
16.27	18.51	18.88	19.18	19.42	19.85	20.20	20.54	20.86	21.22	21.53	21.85
16.53	18.31	18.58	18.97	19.28	19.63	19.98	20.29	20.54	20.96	21.21	21.55
16.81	18.53	18.76	19.18	19.43	19.78	20.04	20.40	20.81	21.22	21.33	21.71
17.12	18.29	18.63	18.96	19.37	19.62	19.92	20.28	20.59	20.97	21.21	21.55
17.45	18.64	18.91	19.20	19.52	19.89	20.24	20.63	20.90	21.19	21.56	21.91
17.81	18.56	18.80	19.16	19.52	19.86	20.22	20.52	20.86	21.23	21.50	21.78
18.19	18.30	18.61	18.91	19.25	19.56	19.88	20.22	20.55	20.90	21.27	21.43
18.6	18.22	18.59	18.73	19.11	19.43	19.71	20.02	20.34	20.64	20.95	21.26

19.05	18.62	18.96	19.21	19.67	19.88	20.29	20.55	20.88	21.17	21.48	21.83
19.53	18.56	18.89	19.22	19.52	19.88	20.23	20.49	20.85	21.15	21.36	21.65
20.05	18.46	18.74	18.99	19.38	19.69	20.02	20.28	20.66	21.03	21.32	21.69
20.61	18.59	18.96	19.21	19.58	19.97	20.29	20.67	20.97	21.25	21.61	21.99
21.22	18.46	18.82	19.14	19.44	19.87	20.14	20.47	20.81	21.12	21.39	21.65
21.87	18.23	18.58	18.80	19.17	19.48	19.87	20.15	20.48	20.89	21.10	21.45
22.57	18.70	19.06	19.39	19.69	19.96	20.35	20.64	20.92	21.21	21.50	21.84
23.33	18.09	18.38	18.73	19.03	19.34	19.66	19.97	20.26	20.60	20.94	21.18
24.15	18.51	18.85	19.22	19.48	19.83	20.05	20.34	20.73	21.15	21.35	21.72
25.03	18.36	18.73	19.05	19.40	19.73	20.01	20.30	20.64	20.92	21.21	21.56
25.98	18.62	18.91	19.18	19.59	19.96	20.25	20.54	20.93	21.23	21.53	21.89
27.01	18.80	19.14	19.49	19.87	20.19	20.47	20.75	21.06	21.34	21.66	21.87
28.11	18.22	18.59	18.90	19.14	19.56	19.89	20.20	20.50	20.76	21.04	21.32
29.3	18.77	19.02	19.41	19.78	20.11	20.42	20.64	20.97	21.39	21.62	22.05
30.59	18.21	18.48	18.76	19.10	19.45	19.76	20.05	20.34	20.59	20.84	21.23
31.98	18.61	18.95	19.21	19.48	19.83	20.16	20.46	20.79	20.98	21.28	21.57
33.47	18.41	18.69	18.94	19.32	19.57	19.98	20.29	20.56	20.85	21.07	21.36
35.09	18.55	18.87	19.25	19.55	19.88	20.20	20.46	20.75	21.10	21.34	21.61
36.83	18.81	19.09	19.36	19.73	20.12	20.36	20.62	20.90	21.21	21.49	21.80
38.71	18.81	19.10	19.30	19.57	19.91	20.27	20.60	20.86	21.20	21.42	21.71
40.73	18.69	18.96	19.33	19.59	19.83	20.18	20.48	20.77	21.14	21.40	21.66
42.92	18.98	19.23	19.57	19.90	20.19	20.50	20.72	20.97	21.33	21.58	21.85
45.27	18.81	19.04	19.27	19.63	19.93	20.33	20.58	20.75	20.99	21.34	21.58
47.81	19.07	19.34	19.70	19.93	20.27	20.63	20.93	21.20	21.49	21.69	21.94
50.55	18.69	18.97	19.29	19.54	19.92	20.18	20.44	20.68	21.00	21.22	21.56
53.51	18.90	19.21	19.45	19.70	19.93	20.28	20.53	20.81	21.18	21.37	21.61
56.69	19.10	19.29	19.63	19.93	20.09	20.49	20.69	20.98	21.15	21.41	21.70
60.13	18.92	19.18	19.33	19.64	19.94	20.19	20.43	20.68	20.93	21.12	21.40
63.84	19.05	19.26	19.61	19.75	20.06	20.34	20.53	20.88	21.10	21.27	21.61
67.83	19.38	19.61	19.87	20.14	20.48	20.75	20.92	21.21	21.51	21.73	22.05
72.15	19.25	19.47	19.64	19.81	20.15	20.42	20.64	20.79	21.15	21.36	21.63
76.8	18.87	19.13	19.38	19.68	19.93	20.21	20.51	20.67	21.01	21.18	21.47
81.81	19.05	19.32	19.54	19.81	20.07	20.27	20.49	20.82	20.99	21.20	21.47
87.22	19.18	19.40	19.61	19.83	20.09	20.29	20.56	20.77	21.00	21.28	21.50
93.06	19.44	19.64	19.78	20.12	20.39	20.56	20.83	21.00	21.22	21.34	21.64
99.35	19.79	20.07	20.19	20.53	20.73	20.96	21.13	21.34	21.63	21.79	22.01
106.13	19.34	19.55	19.79	20.03	20.18	20.43	20.61	20.85	21.12	21.19	21.33
113.45	19.58	19.78	19.97	20.14	20.39	20.65	20.90	21.08	21.26	21.33	21.62
121.34	19.52	19.81	19.99	20.16	20.22	20.56	20.70	20.92	21.12	21.27	21.43
129.86	19.78	19.97	20.15	20.39	20.60	20.73	20.94	21.12	21.23	21.33	21.47
139.04	19.84	20.04	20.22	20.38	20.58	20.68	20.85	20.99	21.20	21.32	21.42
148.94	20.32	20.40	20.63	20.85	21.00	21.14	21.26	21.42	21.69	21.75	21.97
159.62	19.80	19.93	20.17	20.31	20.51	20.58	20.81	20.93	21.06	21.19	21.32
171.14	19.90	20.10	20.15	20.35	20.57	20.70	20.81	20.98	21.06	21.15	21.32
183.56	20.09	20.19	20.28	20.52	20.63	20.78	20.83	20.95	21.15	21.20	21.29
196.96	20.45	20.56	20.75	20.85	20.93	21.02	21.21	21.32	21.44	21.51	21.51
211.41	20.58	20.67	20.75	20.83	21.00	21.11	21.30	21.31	21.44	21.47	21.64
226.99	21.06	21.10	21.22	21.31	21.45	21.58	21.69	21.63	21.73	21.83	21.83
243.8	20.60	20.67	20.72	20.82	20.93	21.02	21.12	21.15	21.26	21.25	21.36
261.93	20.86	20.92	20.99	21.08	21.19	21.21	21.23	21.27	21.44	21.38	21.47

281.48	20.86	20.95	21.01	21.06	21.13	21.18	21.19	21.22	21.29	21.25	21.42
302.56	21.26	21.35	21.35	21.35	21.36	21.37	21.50	21.48	21.58	21.44	21.43
325.3	21.27	21.31	21.23	21.30	21.37	21.38	21.41	21.44	21.44	21.45	21.44
349.83	20.81	20.87	20.83	20.88	20.95	20.97	20.94	20.98	21.03	20.96	20.92
376.28	21.48	21.49	21.41	21.40	21.46	21.42	21.40	21.34	21.38	21.26	21.19
404.81	21.35	21.37	21.36	21.40	21.27	21.30	21.28	21.21	21.22	21.13	21.00
435.58	21.73	21.67	21.57	21.54	21.51	21.54	21.43	21.42	21.37	21.24	21.20
468.76	21.83	21.80	21.67	21.60	21.52	21.51	21.44	21.36	21.33	21.19	21.15
504.55	21.87	21.81	21.72	21.63	21.56	21.58	21.48	21.40	21.33	21.19	21.09
543.15	21.76	21.72	21.53	21.46	21.34	21.30	21.24	21.13	21.05	20.86	20.77
584.78	21.90	21.85	21.80	21.75	21.73	21.57	21.42	21.33	21.17	21.06	20.92
629.68	22.09	22.03	21.91	21.82	21.66	21.56	21.35	21.25	21.15	20.98	20.84
678.1	21.70	21.60	21.45	21.42	21.30	21.17	21.00	20.94	20.81	20.67	20.47
730.33	22.22	22.08	21.92	21.85	21.72	21.60	21.40	21.20	21.06	20.90	20.70
786.65	22.30	22.17	21.99	21.93	21.74	21.61	21.46	21.28	21.23	21.06	20.89
847.4	22.39	22.12	22.03	21.86	21.67	21.50	21.29	21.15	21.02	20.86	20.64
912.92	22.09	21.93	21.68	21.64	21.51	21.38	21.22	21.01	20.72	20.52	20.43
983.58	21.80	21.71	21.47	21.46	21.29	21.03	20.90	20.79	20.60	20.32	20.12
1059.78	21.94	21.74	21.59	21.41	21.28	21.10	20.91	20.73	20.57	20.34	20.14
1141.98	21.69	21.52	21.27	21.10	21.00	20.82	20.63	20.44	20.34	20.07	19.82
1230.62	21.83	21.58	21.42	21.21	21.04	20.91	20.68	20.52	20.28	20.10	19.92
1326.23	22.24	22.04	21.85	21.67	21.50	21.31	21.11	20.87	20.69	20.49	20.20
1429.34	22.21	22.06	21.87	21.64	21.44	21.26	21.11	20.87	20.69	20.42	20.22
1540.54	22.10	21.90	21.69	21.57	21.40	21.21	20.95	20.75	20.50	20.34	20.14
1660.48	22.22	22.00	21.84	21.69	21.46	21.27	20.94	20.72	20.55	20.29	20.13
1789.83	22.41	22.20	21.99	21.76	21.65	21.39	21.17	20.96	20.78	20.54	20.31
1929.34	22.30	22.05	21.90	21.71	21.61	21.34	21.07	20.80	20.51	20.30	20.05
2079.8	22.13	21.95	21.68	21.56	21.37	21.18	21.02	20.77	20.53	20.32	20.13
2242.08	22.39	22.15	21.92	21.66	21.53	21.33	21.16	20.88	20.73	20.44	20.20
2417.1	21.88	21.65	21.49	21.34	21.25	20.92	20.70	20.53	20.31	20.04	19.88
2605.85	22.14	21.90	21.66	21.51	21.31	21.10	20.83	20.62	20.38	20.21	19.97
2809.43	21.82	21.64	21.42	21.26	21.13	20.91	20.70	20.35	20.18	19.95	19.75
3028.99	21.20	20.98	20.82	20.60	20.49	20.26	19.96	19.81	19.61	19.34	19.17
3265.79	21.27	21.10	20.85	20.68	20.49	20.33	20.08	19.84	19.66	19.38	19.24
3521.18	21.37	21.19	21.01	20.81	20.53	20.36	20.15	20.00	19.84	19.48	19.29
3796.62	21.59	21.42	21.11	20.95	20.80	20.51	20.36	20.13	19.87	19.69	19.52

Wavelength (nm) / Time (ps)	661.57	662.98	664.39	665.80	667.21	668.62	670.02
-3.28	-0.08	-0.08	-0.11	-0.11	-0.14	-0.13	-0.10
-2.78	0.00	-0.02	0.02	0.10	0.12	0.09	0.12
-2.28	-0.11	-0.08	0.00	-0.01	-0.02	0.02	-0.05
-1.78	0.02	0.00	-0.03	-0.13	-0.07	-0.04	-0.03
-1.28	0.05	0.01	0.02	0.00	0.01	-0.05	-0.02
-0.78	0.00	-0.01	-0.04	-0.05	-0.12	-0.07	-0.08
-0.28	0.23	0.26	0.22	0.22	0.20	0.11	0.19
0.22	-0.10	-0.08	-0.07	-0.03	0.02	0.07	-0.03
0.32	-0.08	-0.09	-0.14	-0.23	-0.18	-0.09	-0.03

0.42	-0.04	0.00	0.04	0.00	0.01	-0.05	-0.03
0.52	0.20	0.14	0.13	0.14	0.08	0.10	0.21
0.62	-0.20	-0.22	-0.20	-0.16	-0.14	-0.02	-0.02
0.72	0.00	-0.03	-0.02	0.02	0.08	0.07	0.15
0.77	-0.05	-0.10	-0.09	0.00	0.02	0.17	0.23
0.82	0.05	-0.02	0.00	-0.03	0.00	0.07	0.18
0.87	0.10	0.03	0.09	0.07	0.16	0.14	0.15
0.92	0.04	0.08	0.09	0.12	0.18	0.11	0.17
0.97	-0.12	-0.10	-0.11	-0.14	-0.04	0.04	0.07
1.02	-0.21	-0.19	-0.26	-0.15	-0.07	-0.17	-0.10
1.07	0.07	0.09	0.11	0.12	0.10	0.12	0.11
1.12	-0.02	0.03	0.05	0.13	0.08	0.10	0.14
1.17	0.02	-0.01	0.01	0.05	0.02	0.03	0.07
1.22	-0.11	-0.20	-0.18	-0.09	-0.06	-0.11	-0.11
1.27	-0.05	-0.09	-0.03	0.07	0.07	0.10	0.11
1.32	0.07	0.04	0.09	0.02	0.08	0.04	0.12
1.37	0.02	0.00	-0.04	-0.01	0.03	-0.02	0.01
1.42	-0.07	-0.15	-0.06	-0.08	0.03	0.03	0.03
1.47	-0.20	-0.22	-0.23	-0.21	-0.17	-0.09	-0.03
1.52	-0.19	-0.19	-0.22	-0.21	-0.08	-0.08	0.05
1.57	0.09	0.08	0.07	0.11	0.16	0.13	0.17
1.62	2.35	2.28	2.22	2.08	1.97	1.94	2.03
1.67	12.74	12.40	12.08	11.79	11.51	11.40	11.30
1.72	22.33	22.08	21.97	21.63	21.45	21.38	21.49
1.77	25.09	25.02	25.01	25.05	25.11	25.27	25.51
1.82	26.03	26.23	26.31	26.46	26.55	26.99	27.48
1.87	25.26	25.57	25.82	26.11	26.37	26.94	27.40
1.92	25.16	25.59	26.01	26.37	26.77	27.37	28.00
1.97	24.78	25.18	25.59	26.04	26.50	27.20	28.09
2.02	24.39	24.87	25.34	25.91	26.49	27.17	27.95
2.07	24.43	24.97	25.44	25.84	26.36	27.11	27.97
2.12	23.49	23.86	24.37	24.75	25.21	25.90	26.60
2.17	23.12	23.64	23.95	24.42	24.81	25.41	26.08
2.22	22.92	23.34	23.80	24.26	24.68	25.31	25.90
2.27	23.48	23.98	24.47	24.82	25.33	26.05	26.85
2.32	23.64	24.09	24.58	25.06	25.61	26.26	27.08
2.37	23.28	23.79	24.24	24.70	25.21	25.73	26.46
2.42	23.01	23.34	23.81	24.19	24.59	25.26	25.82
2.47	22.91	23.29	23.73	24.10	24.53	25.07	25.76
2.52	22.70	23.19	23.57	23.96	24.48	25.12	25.70
2.57	22.92	23.41	23.86	24.33	24.84	25.57	26.22
2.62	22.96	23.45	23.88	24.29	24.83	25.45	26.17
2.67	22.77	23.17	23.59	23.88	24.39	25.05	25.73
2.72	22.37	22.75	23.16	23.63	23.91	24.55	25.17
2.77	22.66	23.02	23.42	23.78	24.17	24.78	25.42
2.82	22.68	23.06	23.47	23.91	24.28	24.88	25.59
2.87	22.55	22.98	23.43	23.85	24.23	24.89	25.60
2.92	22.63	23.10	23.55	23.90	24.36	24.95	25.71
2.97	22.63	23.04	23.41	23.83	24.21	24.83	25.39
3.02	22.47	22.74	23.15	23.61	24.06	24.64	25.22

3.07	22.07	22.50	22.87	23.24	23.69	24.32	24.95
3.12	23.10	23.53	23.98	24.38	24.90	25.55	26.19
3.17	22.22	22.68	23.13	23.52	23.97	24.57	25.25
3.22	22.36	22.72	23.10	23.40	23.77	24.42	25.11
3.27	21.79	22.17	22.49	22.81	23.19	23.73	24.33
3.32	22.03	22.44	22.73	23.18	23.50	24.10	24.73
3.37	22.16	22.65	23.03	23.44	23.82	24.38	24.98
3.42	21.97	22.30	22.85	23.19	23.57	24.27	24.89
3.47	21.97	22.40	22.83	23.21	23.54	24.23	24.86
3.52	22.00	22.43	22.65	23.06	23.47	24.09	24.71
3.57	21.68	21.97	22.39	22.67	23.05	23.46	24.07
3.62	21.70	22.02	22.45	22.80	23.24	23.80	24.37
3.67	22.12	22.54	22.92	23.35	23.65	24.27	24.97
3.72	21.95	22.34	22.69	23.14	23.56	24.22	24.95
3.92	21.25	21.66	22.04	22.36	22.69	23.16	23.81
4.12	21.45	21.76	22.04	22.39	22.75	23.23	23.80
4.32	22.07	22.37	22.69	23.01	23.47	23.94	24.43
4.52	21.87	22.21	22.62	22.95	23.40	23.84	24.38
4.72	21.55	21.97	22.40	22.68	23.12	23.66	24.22
4.92	22.06	22.46	22.72	23.13	23.45	23.98	24.60
5.12	22.04	22.33	22.70	23.03	23.37	23.93	24.48
5.32	21.96	22.32	22.60	22.92	23.21	23.76	24.33
5.52	21.64	21.96	22.27	22.62	22.97	23.42	24.00
5.72	22.19	22.52	22.84	23.19	23.50	24.00	24.55
5.92	22.06	22.39	22.71	23.02	23.31	23.88	24.40
6.12	21.56	21.85	22.19	22.55	22.93	23.39	23.95
6.32	21.46	21.76	21.98	22.29	22.57	23.10	23.58
6.52	21.74	22.05	22.37	22.68	23.02	23.40	23.81
6.72	21.99	22.31	22.57	22.89	23.11	23.66	24.08
6.92	21.68	21.99	22.40	22.75	23.03	23.61	24.10
7.12	21.21	21.51	21.87	22.21	22.54	23.03	23.52
7.32	21.62	21.93	22.18	22.48	22.79	23.22	23.80
7.52	21.49	21.73	22.01	22.31	22.63	23.01	23.55
7.72	21.73	22.00	22.25	22.56	22.92	23.37	23.91
7.92	22.31	22.59	22.89	23.14	23.57	24.09	24.62
8.12	21.76	22.24	22.39	22.69	23.01	23.51	24.05
8.32	21.84	22.17	22.47	22.84	23.17	23.60	24.22
8.52	21.92	22.20	22.51	22.82	23.02	23.47	23.87
8.72	21.68	21.95	22.19	22.41	22.71	23.16	23.62
8.92	22.03	22.32	22.60	22.91	23.10	23.67	24.20
9.12	22.46	22.82	23.11	23.45	23.70	24.25	24.82
9.32	22.35	22.64	23.04	23.37	23.70	24.16	24.67
9.52	22.28	22.56	22.88	23.21	23.48	23.93	24.46
9.72	22.14	22.42	22.62	22.95	23.28	23.65	24.16
9.92	22.12	22.36	22.62	22.84	23.11	23.61	24.10
10.12	22.03	22.33	22.66	22.94	23.23	23.70	24.16
10.32	21.97	22.23	22.59	22.87	23.21	23.78	24.25
10.52	21.89	22.22	22.46	22.86	23.17	23.63	24.11
10.72	21.33	21.63	21.92	22.19	22.46	22.97	23.31
10.92	22.05	22.28	22.47	22.76	23.07	23.58	24.08

11.12	22.01	22.34	22.57	22.80	23.14	23.57	23.97
11.32	22.12	22.45	22.72	23.05	23.40	23.88	24.47
11.52	21.72	22.11	22.39	22.75	23.00	23.43	24.02
11.72	21.76	22.01	22.34	22.58	22.85	23.27	23.64
11.92	20.99	21.34	21.65	21.93	22.11	22.61	23.11
12.12	21.25	21.47	21.65	21.97	22.24	22.69	23.06
12.32	21.82	22.14	22.43	22.68	22.91	23.45	23.96
12.52	22.14	22.54	22.84	23.12	23.42	23.95	24.41
12.72	22.27	22.64	22.91	23.26	23.52	24.03	24.53
12.92	21.82	22.00	22.32	22.62	22.88	23.29	23.79
13.12	21.84	22.07	22.42	22.63	22.90	23.28	23.67
13.32	22.36	22.64	22.85	23.04	23.34	23.83	24.38
13.52	22.25	22.56	22.82	23.15	23.40	23.90	24.42
13.72	22.23	22.63	22.84	23.10	23.42	23.86	24.41
13.92	22.57	22.81	23.10	23.36	23.64	24.09	24.55
14	21.88	22.13	22.27	22.63	22.85	23.24	23.72
14.08	21.86	22.14	22.42	22.60	22.91	23.32	23.84
14.17	21.42	21.77	21.98	22.27	22.60	23.03	23.47
14.27	22.37	22.61	22.91	23.19	23.53	23.97	24.43
14.38	22.14	22.43	22.76	22.95	23.16	23.63	24.14
14.49	21.68	21.98	22.24	22.46	22.72	22.98	23.55
14.61	22.31	22.55	22.78	23.04	23.27	23.68	24.16
14.75	22.06	22.20	22.57	22.81	23.15	23.59	24.00
14.89	22.32	22.59	22.87	23.17	23.43	24.00	24.46
15.05	22.58	22.95	23.27	23.64	23.92	24.36	24.90
15.21	22.04	22.35	22.72	22.86	23.21	23.68	24.22
15.39	21.85	22.14	22.38	22.59	22.87	23.21	23.72
15.59	21.97	22.24	22.49	22.79	23.05	23.48	23.96
15.8	21.62	21.82	22.16	22.40	22.66	23.03	23.48
16.02	21.89	22.19	22.49	22.74	23.02	23.46	23.95
16.27	22.20	22.58	22.85	23.10	23.36	23.85	24.39
16.53	21.86	22.17	22.42	22.65	22.86	23.30	23.81
16.81	22.00	22.28	22.52	22.75	22.99	23.37	23.70
17.12	21.84	22.17	22.49	22.70	22.86	23.31	23.77
17.45	22.33	22.61	22.92	23.25	23.49	23.93	24.42
17.81	22.23	22.52	22.75	23.04	23.35	23.79	24.28
18.19	21.79	22.10	22.42	22.70	22.92	23.43	23.86
18.6	21.56	21.83	22.08	22.27	22.60	22.93	23.47
19.05	22.14	22.35	22.61	22.80	23.04	23.39	23.85
19.53	21.97	22.27	22.55	22.83	23.00	23.37	23.92
20.05	21.97	22.31	22.56	22.86	23.16	23.60	24.11
20.61	22.28	22.55	22.79	22.97	23.22	23.69	24.16
21.22	21.99	22.23	22.44	22.73	22.94	23.36	23.76
21.87	21.80	22.06	22.31	22.55	22.89	23.29	23.71
22.57	22.14	22.36	22.65	22.86	23.07	23.43	23.83
23.33	21.60	21.85	21.92	22.30	22.54	23.03	23.49
24.15	21.93	22.20	22.50	22.82	23.14	23.57	24.03
25.03	21.90	22.11	22.39	22.52	22.82	23.18	23.70
25.98	22.19	22.42	22.62	22.85	23.02	23.44	23.75
27.01	22.18	22.36	22.61	22.83	23.04	23.51	23.91

28.11	21.67	21.91	22.14	22.29	22.49	22.94	23.43
29.3	22.26	22.52	22.80	22.99	23.26	23.74	24.19
30.59	21.45	21.67	21.98	22.24	22.45	22.83	23.23
31.98	21.96	22.18	22.43	22.66	22.77	23.14	23.56
33.47	21.57	21.96	22.10	22.30	22.41	22.86	23.26
35.09	21.93	22.09	22.33	22.54	22.72	23.11	23.41
36.83	22.08	22.28	22.57	22.66	22.99	23.35	23.81
38.71	22.00	22.28	22.55	22.78	23.01	23.39	23.86
40.73	21.97	22.16	22.46	22.66	22.87	23.27	23.71
42.92	22.12	22.36	22.59	22.84	22.96	23.32	23.74
45.27	21.90	22.05	22.18	22.39	22.54	22.89	23.25
47.81	22.31	22.44	22.56	22.82	22.92	23.38	23.64
50.55	21.84	22.02	22.22	22.40	22.70	23.10	23.54
53.51	21.91	22.15	22.26	22.51	22.73	23.16	23.55
56.69	21.92	22.11	22.25	22.45	22.61	22.99	23.36
60.13	21.60	21.83	22.01	22.17	22.33	22.62	22.99
63.84	21.76	22.01	22.19	22.34	22.57	22.73	23.13
67.83	22.26	22.50	22.64	22.80	22.87	23.22	23.59
72.15	21.83	22.05	22.30	22.47	22.70	23.05	23.46
76.8	21.70	21.85	21.97	22.18	22.36	22.65	23.02
81.81	21.56	21.78	21.94	22.10	22.25	22.56	22.91
87.22	21.65	21.85	21.95	22.06	22.27	22.60	22.88
93.06	21.82	21.94	22.03	22.07	22.18	22.51	22.89
99.35	22.24	22.46	22.59	22.71	22.81	23.05	23.38
106.13	21.52	21.66	21.79	22.03	22.17	22.61	22.88
113.45	21.78	21.95	22.10	22.17	22.28	22.58	22.98
121.34	21.50	21.63	21.69	21.74	21.90	22.18	22.44
129.86	21.64	21.83	21.89	21.99	21.99	22.15	22.57
139.04	21.53	21.73	21.79	21.89	21.96	22.21	22.46
148.94	22.06	22.17	22.25	22.32	22.46	22.77	23.08
159.62	21.45	21.57	21.63	21.70	21.81	22.09	22.33
171.14	21.37	21.46	21.50	21.54	21.61	21.85	22.07
183.56	21.35	21.42	21.53	21.60	21.67	21.81	22.10
196.96	21.61	21.65	21.68	21.66	21.73	21.88	22.13
211.41	21.69	21.71	21.77	21.75	21.77	21.90	22.28
226.99	21.98	22.06	21.99	22.05	22.04	22.33	22.64
243.8	21.43	21.42	21.47	21.50	21.45	21.62	21.91
261.93	21.48	21.40	21.40	21.33	21.40	21.64	21.76
281.48	21.37	21.31	21.25	21.23	21.18	21.29	21.45
302.56	21.40	21.36	21.32	21.27	21.28	21.33	21.58
325.3	21.50	21.45	21.32	21.28	21.23	21.28	21.50
349.83	20.95	20.92	20.84	20.71	20.67	20.73	20.93
376.28	21.23	21.10	20.96	20.88	20.75	20.92	21.08
404.81	20.95	20.88	20.80	20.60	20.57	20.69	20.78
435.58	21.09	21.01	20.88	20.78	20.71	20.68	20.76
468.76	21.05	20.86	20.79	20.61	20.62	20.71	20.78
504.55	20.97	20.86	20.67	20.56	20.45	20.53	20.52
543.15	20.74	20.65	20.54	20.45	20.30	20.30	20.49
584.78	20.72	20.56	20.38	20.32	20.10	20.15	20.24
629.68	20.78	20.62	20.41	20.24	20.05	20.18	20.35

678.1	20.35	20.14	19.96	19.73	19.66	19.68	19.75
730.33	20.61	20.37	20.22	20.01	19.88	19.85	19.99
786.65	20.67	20.49	20.27	20.05	19.89	19.94	19.97
847.4	20.43	20.19	20.00	19.82	19.74	19.68	19.84
912.92	20.27	20.07	19.84	19.54	19.38	19.34	19.41
983.58	19.92	19.59	19.41	19.17	18.94	18.85	18.91
1059.78	20.04	19.73	19.49	19.23	19.07	19.03	19.20
1141.98	19.65	19.41	19.23	18.99	18.86	18.91	18.99
1230.62	19.76	19.53	19.27	19.04	18.88	18.88	18.84
1326.23	19.98	19.75	19.45	19.24	19.09	18.99	19.04
1429.34	20.02	19.78	19.52	19.33	19.13	19.08	19.07
1540.54	19.95	19.66	19.49	19.26	18.99	18.94	19.03
1660.48	19.85	19.59	19.33	19.09	18.96	18.89	18.91
1789.83	20.19	19.85	19.64	19.37	19.17	19.09	19.11
1929.34	19.88	19.57	19.28	19.09	18.89	18.88	18.84
2079.8	19.89	19.54	19.32	19.04	18.76	18.82	18.88
2242.08	19.93	19.76	19.43	19.15	18.89	18.77	18.67
2417.1	19.68	19.37	19.13	18.86	18.60	18.48	18.49
2605.85	19.78	19.52	19.37	19.08	18.90	18.74	18.86
2809.43	19.52	19.32	19.03	18.76	18.61	18.52	18.56
3028.99	18.97	18.64	18.46	18.22	17.99	17.96	17.95
3265.79	18.98	18.77	18.65	18.31	18.06	17.99	18.05
3521.18	19.12	18.88	18.64	18.41	18.16	18.03	18.09
3796.62	19.23	19.05	18.79	18.52	18.24	18.16	18.27

Table S6. Raw data of fs-TA signals of A-RC-A collected under UV (λ_{ex} = 295 nm) excitation in the λ_{pr} = 320–670 nm regime, the unit of ΔA in the table is mOD.

Wavelength (nm) / Time (ps)	320.70	322.11	323.52	324.93	326.34	327.75	329.16	330.56	331.97	333.38	334.79
-3.28	-0.32	0.43	0.21	0.26	0.44	0.01	0.02	0.10	0.19	0.09	0.10
-2.78	0.59	0.03	0.17	0.03	0.19	-0.03	0.05	-0.23	0.19	0.13	0.33
-2.28	0.15	-0.84	-0.23	-0.31	-0.34	-0.28	-0.26	-0.19	-0.35	-0.21	0.12
-1.78	-0.19	0.15	0.71	-0.68	-0.26	-0.20	0.08	-0.01	-0.49	-0.33	-0.18
-1.28	0.31	0.30	-0.07	0.16	0.25	0.75	0.27	-0.32	0.15	0.28	-0.23
-0.78	0.49	0.51	-0.80	-0.08	-0.01	-0.18	-0.36	0.49	-0.07	0.29	-0.03
-0.28	-1.18	0.00	0.23	0.10	-0.04	-0.07	-0.09	-0.20	0.25	-0.40	0.10
0.22	0.57	-0.87	-0.20	0.37	0.00	-0.25	-0.19	0.05	-0.06	-0.01	-0.17
0.32	-0.61	-0.22	0.20	0.17	0.05	0.51	0.30	0.19	0.16	-0.14	-0.16
0.42	0.21	0.50	-0.22	-0.03	-0.27	-0.28	0.18	0.12	0.03	0.31	0.12
0.52	0.50	0.17	0.71	0.04	0.50	0.72	0.54	0.73	0.45	0.06	-0.52
0.62	0.29	-0.10	0.24	-0.21	-0.42	0.84	0.38	0.52	0.37	0.55	0.44
0.72	0.08	-0.47	0.73	0.08	0.35	0.39	-0.03	-0.30	-0.36	-0.47	-0.30
0.77	0.01	-0.15	0.48	-0.21	0.31	0.43	0.06	-0.05	-0.28	-0.02	-0.36
0.82	-0.77	-0.67	0.21	-0.25	-1.15	0.46	-0.15	-0.78	-0.78	-0.39	-0.70
0.87	0.36	-0.10	-0.06	-0.06	-0.26	0.30	0.04	-0.27	0.00	-0.25	0.02
0.92	-0.10	-0.05	0.04	0.38	-0.17	0.13	-0.03	-0.08	-0.52	0.00	-0.50
0.97	-0.05	-0.77	0.48	-0.25	0.40	0.05	-0.01	-0.21	-0.07	0.11	-0.04
1.02	-0.45	-0.19	0.28	0.29	-0.36	0.21	0.13	-0.13	-0.26	-0.07	-0.14

1.07	-0.33	0.41	-0.48	0.47	0.06	-0.27	-0.60	-0.22	-0.30	-0.79	-0.40
1.12	0.15	0.35	-0.13	0.12	0.45	0.50	0.14	0.25	0.06	-0.43	-0.16
1.17	0.65	-0.34	-0.67	0.12	0.29	0.21	0.09	-0.13	-0.83	-0.59	-0.30
1.22	-1.07	0.11	0.97	-0.22	0.07	0.07	0.03	-0.13	-0.55	0.14	-0.30
1.27	-0.96	0.09	-0.12	-0.54	0.02	0.00	-0.32	-0.46	-0.47	-1.04	-0.47
1.32	-0.89	-0.14	-0.04	0.21	-0.30	-0.50	0.06	-0.10	-0.95	-0.16	-0.35
1.37	-0.28	-0.76	-0.39	-0.05	0.17	0.37	0.24	0.19	-0.63	-0.15	0.15
1.42	0.16	-0.59	-0.04	0.07	0.12	0.05	0.36	-0.16	-0.72	-0.15	-0.06
1.47	0.17	-1.12	0.11	0.58	-0.27	-0.21	0.40	0.32	-0.35	0.10	0.47
1.52	-0.36	-0.38	0.06	-0.18	-0.26	-0.02	-0.75	-0.20	-0.47	-0.09	-0.34
1.57	-0.52	-0.51	0.84	-0.23	-0.58	0.12	0.00	-0.79	0.00	-0.09	-0.62
1.62	-0.68	-0.44	-0.38	0.11	0.42	0.01	-0.53	-0.53	-0.79	-0.19	-0.29
1.67	0.29	0.08	-0.63	0.31	-0.14	-0.06	-0.02	-0.36	0.00	-0.27	-0.53
1.72	-0.31	-0.63	-0.29	-0.31	-0.15	-0.29	-0.64	-0.80	-0.78	-0.56	-0.66
1.77	0.13	-0.23	0.07	-0.03	-0.29	0.62	0.50	-0.16	-0.09	-0.14	-0.17
1.82	-0.19	0.14	-0.36	-0.19	0.15	1.15	-0.84	0.74	-0.13	-0.24	-0.31
1.87	0.43	-1.05	0.09	-0.38	-0.71	-0.18	-0.15	-0.51	-0.81	-0.41	-0.55
1.92	0.56	-0.32	-0.08	-0.18	-0.13	0.32	-0.16	0.11	-0.44	-0.12	-0.02
1.97	0.07	-0.17	-0.11	0.04	0.10	0.62	0.27	0.10	-0.20	0.30	0.22
2.02	-0.08	-0.97	0.53	0.07	-0.24	-0.59	0.15	-0.26	-0.30	-0.09	-0.46
2.07	0.41	-0.82	0.23	0.68	-0.14	-0.09	0.41	-0.59	-0.14	0.00	-0.03
2.12	-0.13	-0.37	-0.15	0.20	-0.87	0.12	-0.01	-0.33	-0.53	-0.39	-0.31
2.17	-0.95	-1.09	-0.15	0.16	-0.14	0.09	-0.55	-0.31	-0.18	-0.26	-0.86
2.22	-0.50	0.21	-0.27	-0.70	-0.22	-0.24	0.21	-0.20	-0.45	-0.96	-0.37
2.27	-0.63	-0.07	-0.25	0.23	-0.54	-0.02	-0.21	-0.15	-0.44	-0.64	-0.13
2.32	0.31	0.00	-0.06	-0.13	0.44	-0.13	-0.04	-0.28	-0.51	0.25	-0.15
2.37	0.27	-0.12	-0.32	0.23	-0.11	0.36	0.04	0.17	-0.16	0.07	-0.07
2.42	-0.38	-0.86	-0.19	0.31	-0.31	-0.09	-0.44	-0.84	-0.17	-0.16	-0.49
2.47	-0.38	-0.76	-0.50	-0.21	0.44	-0.10	-0.61	-0.06	-0.24	-0.35	-0.15
2.52	0.76	0.73	0.21	-0.94	-0.62	0.63	-0.60	-0.14	-0.42	-0.25	-0.06
2.57	0.75	0.03	0.00	0.48	-0.23	-0.40	-0.14	-0.23	-0.18	0.10	-0.17
2.62	0.10	-1.17	-0.29	-0.02	0.55	0.26	-0.17	-0.27	-0.46	-0.42	-0.28
2.67	0.20	-0.18	-0.42	-0.44	-0.23	-0.05	-0.59	-0.72	-0.91	-0.40	-0.36
2.72	-0.44	-0.51	0.30	-0.71	-0.42	-0.21	-0.55	-0.59	-0.38	-0.63	-0.07
2.77	0.09	-0.64	0.25	-0.13	0.08	-0.59	-0.57	-0.61	-0.49	-0.50	-0.36
2.82	-0.96	-0.12	-0.43	-0.34	0.34	0.33	-0.15	-0.31	-0.87	-0.30	-0.23
2.87	0.32	0.05	0.44	-0.02	-0.93	-0.29	0.38	-0.64	-0.24	0.07	-0.16
2.92	0.17	0.30	0.54	-0.17	-0.34	-0.10	-0.32	0.00	-0.71	-0.34	-0.30
2.97	-0.40	0.33	-0.07	-0.93	-0.16	0.13	-1.04	-0.73	-0.14	-0.81	-0.78
3.02	-0.17	-0.10	0.31	-0.52	0.62	0.07	-0.16	0.17	0.10	0.02	-0.08
3.07	-0.66	0.04	-0.63	0.09	-0.01	-0.36	-0.34	-0.65	-0.15	-0.63	-0.41
3.12	-0.56	0.15	-0.11	-0.01	-0.23	-0.40	0.09	-0.16	-0.46	-0.62	-0.22
3.17	0.25	-0.28	-0.51	-0.61	-0.43	-0.30	-0.38	-0.32	-0.87	-0.95	-0.78
3.22	-0.12	-0.52	0.28	-0.25	-0.39	0.38	-0.23	-0.49	-0.81	-0.28	-0.46
3.27	-0.16	-0.26	0.71	0.60	0.37	0.83	-0.04	-0.25	0.02	-0.29	-0.42
3.32	0.30	0.10	-0.76	0.23	0.39	0.45	-0.48	0.36	-0.33	-0.21	-0.12
3.37	0.25	-0.41	-0.74	0.45	-0.11	-0.21	-0.35	-0.09	-1.07	-0.28	-0.43
3.42	-0.37	-0.10	-0.10	-0.24	-0.17	-0.17	-0.55	0.26	-0.18	-0.62	-0.58
3.47	-0.59	-0.40	-0.02	-0.36	-0.19	0.06	-0.55	-0.17	-0.69	-0.39	-0.12
3.52	0.05	-0.67	-0.01	0.25	0.05	0.38	-0.38	0.11	-0.48	0.02	-0.32

3.57	0.57	0.31	-0.49	0.06	0.20	-0.63	-0.39	-0.18	-0.19	-0.46	-0.45
3.62	-0.29	-0.46	-0.24	-0.41	0.32	-0.42	-0.54	-0.19	-0.22	-0.33	-0.71
3.67	0.19	-0.75	0.90	0.70	0.16	-0.05	0.15	0.45	-0.27	-0.08	-0.12
3.72	0.51	-0.98	0.16	-0.38	0.46	-0.24	0.11	-0.28	-0.49	-0.39	-0.15
3.92	-0.18	0.07	0.07	0.14	0.61	0.26	-0.85	-0.43	-0.36	-0.26	-0.36
4.12	0.08	-0.84	0.59	-0.17	-0.47	-0.38	-0.12	-0.92	-0.56	-0.68	-0.82
4.32	-0.13	-0.17	-0.51	0.37	-0.08	0.29	0.00	0.02	0.04	-0.34	-0.47
4.52	-0.22	0.40	-0.02	-0.07	0.42	-0.29	-0.56	-0.84	-0.37	-0.22	-0.14
4.72	-0.29	0.25	-0.50	-1.16	0.18	-0.75	-0.51	0.04	0.05	-0.26	-0.34
4.92	0.21	-0.52	-0.50	-0.44	0.29	-0.21	-0.16	-0.10	-0.21	-0.15	-0.18
5.12	-0.86	0.23	0.34	-0.27	0.14	-0.24	-0.28	-0.65	0.00	-0.57	-0.24
5.32	0.12	-0.37	-0.49	0.75	0.28	-0.54	-0.53	-0.21	-0.42	-0.68	-0.25
5.52	-0.11	-0.43	0.16	-0.10	-0.19	-0.28	-0.07	-0.64	-0.48	-0.16	0.12
5.72	0.49	0.03	0.30	-0.41	0.22	-0.32	-0.69	-0.19	-0.61	-0.79	-0.60
5.92	0.41	1.17	-0.03	-0.34	0.21	0.66	-0.59	-0.23	-0.26	-0.66	0.17
6.12	-0.16	-0.39	-0.26	0.48	0.60	0.16	-0.37	-0.22	-0.34	-0.56	-0.27
6.32	-0.37	-0.04	-0.08	-0.79	0.01	-0.50	-0.35	-0.25	0.02	-0.48	-0.15
6.52	0.08	-0.12	0.03	0.26	-0.11	-0.18	0.01	0.10	0.18	-0.30	-0.20
6.72	0.45	0.53	0.55	-0.19	-0.07	-0.40	0.20	-0.59	-0.35	-0.06	-0.70
6.92	-0.27	-0.33	0.00	-0.88	-0.13	0.44	-0.28	-0.53	-0.43	-0.52	-0.47
7.12	-0.63	-0.20	1.17	0.13	-0.49	0.21	0.39	-0.82	-0.73	0.14	-0.20
7.32	0.34	-0.66	0.03	-0.02	0.47	-0.10	-0.36	-0.17	-0.06	-0.28	-0.49
7.52	0.36	-0.06	0.63	0.09	-0.23	-0.27	-0.21	-0.39	-0.62	-0.35	-0.31
7.72	0.28	-1.18	-0.31	0.50	-0.20	0.50	0.36	0.08	-0.27	0.28	0.00
7.92	0.07	-0.08	-0.34	0.60	-0.37	-0.28	-0.28	-0.72	-0.75	-0.22	-0.63
8.12	0.85	-1.07	0.47	0.68	-0.09	-0.07	0.03	0.06	-0.52	-0.13	0.46
8.32	-0.02	-0.66	0.95	-0.17	-0.31	0.44	-0.34	-0.18	-0.68	-0.16	-0.04
8.52	0.15	-0.05	-1.06	-0.18	0.03	-0.34	-0.57	-0.25	-0.43	-0.38	-0.37
8.72	-0.01	0.39	-0.26	-0.30	-0.15	0.07	-0.63	-0.73	-0.38	-0.46	-0.33
8.92	0.22	-0.03	-0.23	-0.36	0.17	0.02	0.06	-0.59	0.00	-0.02	-0.60
9.12	-0.22	-0.44	0.76	-0.16	-0.43	0.02	-0.38	-0.53	-0.70	-0.12	-0.28
9.32	-0.21	-0.29	-0.03	-0.12	0.09	0.48	-0.23	0.10	-0.22	-0.04	-0.13
9.52	0.75	-0.25	-0.14	-0.34	-0.47	-0.75	-0.14	-0.45	-0.56	-0.23	-0.23
9.72	-0.43	0.12	0.77	-0.24	-0.23	0.58	-0.08	-0.23	-0.36	-0.13	-0.48
9.92	0.07	-0.59	-0.74	-0.60	0.27	-0.49	-0.38	-0.74	-0.41	-0.26	-0.71
10.12	1.04	-0.21	-0.57	0.22	0.40	-0.84	-1.22	-0.05	-0.03	-0.56	-0.01
10.32	-0.24	0.12	-0.94	0.24	-0.52	-0.04	0.03	-0.79	-0.65	-0.64	-0.23
10.52	0.22	0.11	-0.24	-0.26	-0.23	0.53	-0.31	-0.39	-0.46	-0.08	-0.63
10.72	-0.40	-1.61	0.02	0.21	-0.23	-0.16	0.01	-0.03	-0.67	-0.26	-0.20
10.92	0.07	0.20	-0.14	-0.13	-0.56	-0.22	0.09	-0.58	-0.35	-0.49	-0.46
11.12	0.09	-0.21	0.38	-0.67	0.07	-0.68	-0.10	-0.29	-0.42	-0.05	-0.60
11.32	-0.03	-0.29	-0.12	-0.02	0.19	-0.31	-0.13	0.15	-0.24	-0.22	-0.18
11.52	0.16	0.13	0.02	-0.71	-0.12	-0.23	-0.21	-0.38	-0.87	-0.58	-0.38
11.72	0.17	-1.17	0.55	0.37	-0.91	0.76	-0.78	-0.65	-0.43	0.42	-0.22
11.92	-0.02	-0.30	0.12	-0.11	0.44	-0.26	-0.05	0.35	0.19	-0.26	-0.52
12.12	-0.37	0.54	0.20	0.06	-0.02	0.39	-0.43	-0.59	-0.19	-0.27	-0.25
12.32	0.56	-0.40	0.09	-0.09	-0.08	0.23	-0.05	0.22	0.25	-0.29	0.19
12.52	0.13	-0.17	-0.24	-0.03	-0.80	-0.40	0.37	-0.27	-0.74	-0.21	-0.15
12.72	-0.41	-0.26	-0.15	0.15	-0.11	-0.20	-0.70	-0.47	-0.33	-0.83	-0.65
12.92	-0.58	-0.66	0.45	-0.06	-0.23	-0.02	-0.52	-0.45	-0.24	0.09	-0.33

13.12	-1.18	0.18	0.53	-1.07	-0.05	0.20	0.10	-0.80	-0.32	0.15	-0.17
13.32	-0.68	-0.19	-0.58	-0.04	-0.21	-0.55	0.12	-0.14	-0.18	-0.63	-0.31
13.52	-0.41	0.24	-0.12	-0.07	-0.87	-0.27	-0.20	-0.34	-0.49	-0.39	-0.48
13.72	-0.25	-0.16	0.11	-0.23	0.29	0.15	0.02	-0.08	-0.23	-0.08	-0.23
13.92	-0.69	-0.09	0.14	-0.36	0.26	0.39	-0.07	-0.79	0.03	0.38	-0.43
14.00	0.67	0.18	-0.01	0.30	0.01	0.51	-0.06	-0.30	0.28	-0.01	0.30
14.08	1.01	-0.76	-0.02	0.37	-0.76	-0.34	-0.34	-0.47	-0.81	-0.39	-0.19
14.17	0.73	0.05	-0.71	-0.10	0.25	-0.05	-0.49	-0.19	-0.64	-0.36	-0.80
14.27	0.45	-0.29	-0.17	-0.36	-0.14	-0.41	0.00	-0.34	-0.47	-0.52	-0.46
14.38	-0.56	0.35	0.02	-0.98	-0.24	0.25	-0.83	-0.75	-0.74	-0.07	-0.66
14.49	-0.23	-0.50	-0.77	0.39	0.86	0.67	-0.10	0.21	0.12	0.57	0.11
14.61	0.03	-0.98	-0.06	-0.73	-0.07	-0.22	-0.77	-0.67	-0.41	-0.31	-0.22
14.75	0.06	0.22	-0.65	0.20	-0.64	0.15	-0.09	-0.73	-0.09	-0.61	-0.67
14.89	-0.41	-0.64	0.18	-0.42	-0.76	-0.51	-0.08	0.05	-0.31	-0.69	-0.08
15.05	0.44	0.36	0.97	0.19	-0.18	0.31	-0.81	0.12	-0.16	0.00	-0.38
15.21	-0.54	0.09	-0.23	-0.30	-0.20	-0.30	-0.20	0.34	-0.14	-0.01	-0.11
15.39	-0.04	0.29	0.34	0.28	-0.81	0.20	-0.25	-0.42	-0.01	-0.30	-0.26
15.59	0.28	-0.13	0.19	-0.10	-0.57	-0.12	-0.12	0.10	-0.33	-0.23	0.17
15.80	0.36	-0.33	0.19	0.03	-0.52	-0.07	-0.08	0.04	-0.28	-0.60	-0.47
16.02	0.31	0.47	-0.65	-0.52	0.71	-0.28	0.38	-0.32	-0.64	-0.59	-0.31
16.27	-0.25	-0.59	-0.60	0.20	-0.68	0.16	-0.25	-0.49	-0.82	-0.56	-0.72
16.53	-0.29	-0.33	-0.29	0.06	0.03	-0.12	0.01	-0.40	-0.29	-0.61	-0.55
16.81	0.01	-0.11	0.40	0.09	0.05	-0.21	0.22	-0.41	-0.36	-0.38	-0.39
17.12	-0.67	-0.79	-0.83	0.35	0.08	-0.46	-0.48	-0.33	-0.60	-0.23	-0.19
17.45	-0.70	-0.23	-0.08	-0.38	-0.58	-0.09	-0.64	-0.54	-0.12	-0.48	0.00
17.81	-0.47	0.08	0.28	-0.52	0.35	-0.40	-0.19	0.16	-0.40	-0.09	-0.63
18.19	0.35	-0.49	0.39	-0.31	-0.08	0.21	0.10	-0.16	-0.37	-0.30	-0.23
18.60	-0.43	-0.44	-0.28	-0.60	-0.01	0.22	-0.27	0.09	-0.46	0.12	-0.06
19.05	-0.25	0.37	0.50	-0.28	-0.15	0.08	-0.07	-0.14	0.00	-0.08	-0.34
19.53	0.08	-0.41	-0.18	-0.32	-0.43	0.09	-0.12	0.17	0.12	0.06	0.30
20.05	-0.74	0.33	0.74	-0.36	-0.02	-0.09	-0.14	-0.38	-0.09	-0.08	0.03
20.61	0.43	-0.63	-0.56	-0.07	0.01	-0.04	-0.09	-0.05	-0.37	-0.28	-0.51
21.22	-0.43	-0.58	0.60	-0.14	0.31	-0.08	-0.15	-0.17	-0.44	-0.34	-0.31
21.87	0.05	-0.32	0.04	0.01	0.58	0.00	-0.12	-0.68	-0.34	-0.20	-0.38
22.57	0.12	0.89	0.72	-0.13	0.49	0.20	0.14	0.64	0.21	0.34	0.12
23.33	-0.39	0.54	-0.84	-0.24	-0.10	-0.21	-0.68	-0.71	-0.31	-0.22	-0.27
24.15	-0.69	0.04	-0.08	-0.59	-0.09	0.12	-0.16	-0.03	0.13	0.00	-0.34
25.03	-0.01	-0.72	-0.39	-0.41	-0.08	0.03	-0.83	-0.61	-0.51	-0.48	-0.04
25.98	-0.33	-0.04	-0.36	-0.24	-0.37	-0.05	-0.11	0.13	-0.51	-0.04	-0.05
27.01	0.28	-0.33	-0.07	-0.05	-0.27	0.31	0.33	-0.05	-0.33	-0.25	0.13
28.11	-0.19	-0.32	-0.80	-0.48	0.10	0.32	-0.74	-0.25	-0.35	-0.17	-0.45
29.30	-0.23	-0.61	0.11	-0.27	-0.39	-0.42	-0.09	-0.13	-0.66	-0.63	-0.05
30.59	-0.15	0.22	0.59	-0.77	0.34	-0.25	-0.19	0.11	-0.23	0.23	-0.02
31.98	0.46	-0.49	0.23	-0.11	-0.21	0.37	-0.57	-0.45	-0.55	-0.20	-0.13
33.47	0.08	-0.18	-0.10	-0.70	-0.65	0.26	-0.29	-0.41	-0.38	-0.20	-0.49
35.09	0.07	0.11	-0.19	0.39	-0.46	0.29	0.21	-0.19	0.03	0.12	0.17
36.83	-0.46	-0.24	-0.04	-0.47	-0.45	-0.33	-0.88	-0.36	-0.09	-0.01	-0.10
38.71	0.15	0.83	0.56	0.18	0.06	-0.13	-0.02	0.03	-0.50	-0.54	-0.34
40.73	-0.05	-0.89	0.32	0.41	0.06	-0.08	0.23	0.27	-0.34	-0.29	-0.51
42.92	0.13	-0.49	-0.06	-0.23	-0.13	-0.30	-0.07	-0.77	-0.98	-0.27	-0.40

45.27	-0.57	-0.51	-0.72	-0.34	-0.11	0.00	0.00	-0.61	-0.43	-0.34	-0.91
47.81	0.61	-0.12	0.36	0.38	0.26	0.13	0.50	-0.44	-0.03	0.03	-0.07
50.55	-0.10	-0.52	0.16	-0.17	0.22	0.00	-0.31	0.02	-0.32	-0.07	0.04
53.51	-0.39	0.05	0.28	-0.60	0.46	0.70	-0.39	0.14	-0.07	0.02	0.17
56.69	0.41	-0.43	0.75	1.14	0.31	-0.56	-0.62	-0.29	-0.20	-0.39	0.12
60.13	0.09	0.22	0.18	-0.02	0.06	0.58	-0.11	-0.31	-0.46	0.01	0.09
63.84	-0.47	0.35	0.04	-0.22	-0.41	0.16	-0.36	-0.24	-0.09	0.03	0.04
67.83	0.09	-0.05	0.52	0.60	0.50	-0.86	-0.21	0.01	-0.43	0.24	0.18
72.15	-0.17	0.54	-0.03	-0.09	0.02	0.60	-0.11	-0.10	-0.38	-0.22	-0.10
76.80	-0.02	-0.54	-0.47	-0.45	0.52	-0.11	0.13	0.24	-0.28	-0.14	-0.01
81.81	-0.24	-0.01	0.17	0.09	0.49	-0.54	-0.23	-0.44	-0.70	-0.03	-0.17
87.22	-0.97	-0.63	-0.46	0.66	-0.40	-0.78	-0.58	-0.38	-0.85	-0.51	-0.13
93.06	-0.46	-0.79	-0.19	-0.28	0.26	0.46	-0.63	-0.43	-0.52	-0.60	-0.35
99.35	0.08	0.17	-0.03	-0.15	0.39	0.54	0.28	-0.34	-0.06	0.35	-0.09
106.13	-0.94	-0.35	0.68	0.62	-0.40	0.06	0.04	-0.06	-0.36	-0.03	-0.32
113.45	0.56	-0.78	-0.71	-0.37	0.05	-0.30	0.04	-0.43	-0.56	-0.39	-0.15
121.34	-0.35	-0.02	-0.01	-0.78	-0.48	-0.43	0.01	-0.48	-0.47	-0.66	-0.66
129.86	-0.76	-0.29	0.63	0.30	-0.15	0.27	0.41	-0.22	0.22	0.17	0.01
139.04	0.29	-0.74	0.14	0.68	0.76	-0.07	-0.04	0.35	-0.06	-0.10	0.33
148.94	0.07	-0.50	0.37	-0.32	0.23	-0.28	-0.82	-0.28	-0.48	-0.36	-0.01
159.62	0.00	-0.52	-0.61	-0.16	0.13	0.67	-0.39	-0.05	-0.20	0.02	-0.12
171.14	0.01	-0.96	-0.34	-0.16	-0.60	0.17	-0.39	-0.32	-0.43	-0.58	-0.60
183.56	-0.16	-0.55	0.77	0.50	-0.44	0.02	0.29	0.45	-0.20	-0.43	-0.08
196.96	0.26	-0.16	-0.11	0.32	0.08	-0.03	0.53	-0.39	-0.91	-0.54	-0.28
211.41	-0.19	-0.32	-0.17	0.39	0.32	0.30	-0.07	0.05	-0.53	-0.84	-0.14
226.99	-0.02	-0.12	0.93	0.60	0.12	0.46	0.30	0.48	0.47	-0.11	0.45
243.80	-0.10	-0.11	-0.40	0.27	-0.04	-0.58	-0.35	-0.30	-0.61	-0.59	-0.16
261.93	0.41	-0.42	-0.22	-0.52	-0.10	-0.16	0.48	-0.25	0.20	0.20	0.22
281.48	-0.05	0.44	0.10	-0.15	-0.07	-0.46	-0.17	-0.33	-0.36	-0.10	-0.20
302.56	-0.08	0.48	-0.59	0.62	0.12	0.65	-0.42	-0.32	0.26	0.03	-0.41
325.30	-0.45	0.46	0.48	0.66	0.30	0.37	0.13	-0.13	0.27	0.18	-0.23
349.83	0.19	0.12	0.17	-0.04	-0.19	-0.14	-0.48	-0.43	0.04	-0.54	-0.16
376.28	0.68	-0.23	0.52	-0.19	-0.22	-0.21	0.39	-0.11	-0.30	0.11	-0.19
404.81	-0.68	-0.32	-0.07	0.07	0.05	0.52	-0.45	0.13	-0.02	-0.32	-0.09
435.58	0.02	-0.02	-0.20	-0.15	-0.32	-0.02	-0.42	0.19	-0.48	-0.07	0.00
468.76	-0.58	-0.04	-1.04	-0.71	0.24	-0.16	-0.87	-0.49	0.02	-0.37	-0.03
504.55	0.14	-0.43	0.38	0.22	0.18	0.36	0.14	-0.43	0.00	-0.10	0.01
543.15	-0.16	-0.52	0.76	0.00	-0.52	-0.21	-0.17	0.06	-0.10	-0.52	0.13
584.78	0.34	-0.85	0.34	-0.52	0.54	0.09	0.17	-0.36	0.24	-0.23	-0.55
629.68	0.03	-1.02	-0.48	0.10	-0.09	-0.17	-0.51	-0.33	-0.61	-0.32	-0.38
678.10	-0.14	-0.47	1.10	0.24	0.24	-0.28	0.16	-0.23	0.22	0.16	0.03
730.33	-0.60	-0.65	-0.33	0.00	-0.06	0.66	-0.15	-0.33	-0.06	0.14	-0.06
786.65	-0.88	0.01	-0.75	-0.57	0.18	-0.40	-0.87	-0.39	-0.42	-0.71	-0.34
847.40	-0.80	-0.19	0.16	-0.50	-0.53	-0.26	-0.38	-0.42	-0.45	-0.16	-0.42
912.92	0.31	0.00	-0.67	-0.01	0.16	-0.08	-0.12	-0.13	0.23	-0.46	0.05
983.58	-0.05	-1.04	-0.62	-0.29	-0.25	0.08	-0.04	-0.07	-0.65	0.05	-0.02
1059.78	0.71	-1.05	-0.61	-0.03	0.32	-0.09	-0.01	-0.32	-0.51	0.03	-0.44
1141.98	0.32	-0.60	0.17	-0.43	0.18	-0.24	-0.05	0.00	-0.38	-0.43	0.38
1230.62	-0.26	-0.56	-0.66	-0.20	0.50	0.41	-0.17	0.41	0.15	0.06	-0.05
1326.23	-0.20	0.34	0.65	-0.18	0.42	0.45	0.19	-0.10	0.15	0.25	0.44

1429.34	0.36	-0.31	-0.47	0.03	-0.24	-0.36	-0.56	-0.15	-0.62	-0.77	0.22
1540.54	-0.01	-0.94	-0.30	0.00	0.36	-0.05	-0.23	-0.27	-0.55	-0.58	-0.36
1660.48	0.62	-0.35	0.41	-0.34	0.26	0.04	-0.20	-0.02	-0.32	-0.03	0.13
1789.83	-0.89	0.73	-0.15	0.11	-0.08	0.05	-0.26	0.09	0.11	-0.06	0.12
1929.34	0.11	0.08	-0.02	0.02	0.12	0.26	0.29	-0.50	0.61	-0.01	-0.16
2079.80	-0.34	0.58	-0.55	-0.16	-0.49	0.36	-0.52	-0.39	-0.43	-0.02	-0.64
2242.08	0.11	0.04	0.22	0.28	-0.53	0.17	0.00	-0.48	-0.19	-0.19	-0.16
2417.10	-0.17	0.59	0.37	-0.39	-0.32	0.04	-0.43	-0.43	-0.13	-0.40	-0.20
2605.85	-0.84	0.82	0.25	0.13	0.24	0.06	-0.04	-0.06	-0.60	-0.14	-0.23
2809.43	-0.53	-0.50	0.23	-0.37	-0.38	-0.15	0.04	-0.30	-0.29	-0.26	-0.42
3028.99	0.52	0.23	0.11	-0.19	-0.55	0.05	0.22	-0.23	0.05	-0.18	0.20
3265.79	-0.12	-0.18	0.99	0.00	-0.52	-0.08	-0.40	-0.50	-0.22	0.30	-0.33
3521.18	-0.42	-0.77	0.00	0.13	-0.52	0.05	0.07	-0.15	0.43	-0.18	0.04
3796.62	-0.73	-0.32	-0.19	-0.20	0.44	0.26	0.76	0.28	0.06	0.18	0.12

Wavelength (nm) / Time (ps)	336.20	337.61	339.02	340.42	341.83	343.24	344.65	346.06	347.47	348.87	350.28
-3.28	0.19	0.00	0.30	0.22	0.29	0.27	0.18	0.09	0.25	0.20	0.29
-2.78	0.12	-0.13	-0.11	-0.08	-0.19	-0.19	-0.08	-0.03	0.24	-0.18	-0.14
-2.28	-0.22	-0.13	-0.09	-0.01	-0.13	-0.15	-0.02	-0.24	-0.14	-0.02	0.03
-1.78	0.03	-0.17	-0.10	-0.25	0.03	-0.10	0.08	0.27	-0.09	-0.06	-0.05
-1.28	0.11	0.11	-0.26	-0.17	0.03	0.02	-0.23	0.01	-0.05	0.21	0.02
-0.78	-0.25	0.27	0.01	-0.05	0.02	-0.01	0.05	-0.23	-0.04	-0.04	-0.32
-0.28	0.14	0.14	0.24	0.13	-0.03	-0.01	0.08	0.23	-0.04	0.06	0.12
0.22	-0.20	-0.17	-0.23	0.13	-0.04	0.20	-0.11	-0.17	0.01	0.03	0.09
0.32	0.26	-0.02	0.19	0.08	-0.02	0.12	-0.01	0.13	-0.14	0.01	-0.03
0.42	-0.18	0.08	0.04	0.00	0.04	-0.15	0.05	-0.07	0.00	-0.20	0.00
0.52	-0.49	-0.62	-0.08	0.03	0.19	-0.08	0.05	0.04	0.00	0.01	-0.01
0.62	0.27	0.26	0.75	0.59	0.23	0.16	-0.26	-0.25	-0.26	-0.03	0.09
0.72	-0.17	-0.37	-0.23	0.04	0.33	0.78	1.46	1.72	1.53	1.45	1.27
0.77	-0.36	-0.06	0.39	-0.14	0.43	0.26	0.72	0.98	0.96	0.88	1.24
0.82	-0.62	-0.12	-0.49	-1.02	-0.46	-0.28	-0.33	-0.03	0.43	0.61	0.92
0.87	-0.02	-0.29	-0.10	0.07	-0.16	-0.12	0.29	0.37	0.38	0.34	0.75
0.92	-0.10	-0.34	-0.11	-0.22	-0.20	0.09	-0.06	0.10	0.14	0.31	0.43
0.97	0.05	-0.35	-0.04	-0.03	-0.11	-0.26	-0.18	0.33	0.38	0.41	0.41
1.02	-0.17	-0.49	-0.19	-0.09	-0.15	-0.14	0.11	0.13	0.25	0.39	0.54
1.07	-0.51	-0.54	-0.56	-0.03	-0.03	0.00	-0.28	0.03	0.17	0.33	0.55
1.12	0.30	-0.18	0.02	0.10	-0.14	-0.04	0.16	0.29	0.33	0.51	0.40
1.17	-0.29	-0.53	-0.07	-0.23	-0.32	-0.41	-0.02	0.12	0.20	0.19	0.22
1.22	-0.12	-0.01	-0.29	-0.17	0.07	0.09	0.17	0.15	0.38	0.56	0.43
1.27	-0.32	-0.53	-0.42	-0.51	-0.28	-0.23	-0.18	0.10	0.01	0.05	0.37
1.32	-0.70	-0.78	-0.33	-0.25	-0.12	-0.18	0.00	0.07	0.27	0.39	0.56
1.37	-0.30	0.12	-0.11	-0.12	-0.14	0.26	0.21	0.30	0.17	0.35	0.54
1.42	-0.24	-0.49	-0.44	0.00	0.30	0.08	0.15	0.49	0.42	0.47	0.47
1.47	0.13	-0.37	0.14	0.16	-0.20	0.14	0.43	0.35	0.32	0.40	0.60
1.52	-0.22	-0.67	-0.06	-0.26	-0.25	-0.45	0.05	0.29	0.18	0.00	0.50
1.57	-0.74	-0.11	-0.10	-0.33	-0.12	0.01	-0.20	0.16	0.02	0.10	0.22
1.62	-0.38	-0.45	-0.45	0.11	-0.34	-0.35	0.17	0.20	0.16	0.23	0.27
1.67	-0.30	-0.21	-0.53	-0.20	-0.21	0.18	-0.01	0.12	0.23	0.41	0.42

1.72	-0.48	-0.50	-0.44	-0.51	-0.18	-0.32	-0.33	0.11	-0.36	-0.10	0.09
1.77	-0.41	-0.29	0.21	0.11	-0.17	-0.14	-0.13	0.51	0.22	0.31	0.22
1.82	-0.14	-0.50	-0.16	0.05	0.00	-0.17	0.25	0.16	0.20	0.25	0.19
1.87	-0.84	-0.57	-0.41	-0.54	-0.36	0.02	-0.06	-0.09	0.05	0.24	0.14
1.92	-0.47	-0.20	-0.24	-0.17	-0.36	-0.10	0.27	0.23	0.35	0.23	0.51
1.97	0.03	-0.32	-0.07	0.10	-0.05	0.25	0.00	0.11	0.05	0.23	0.45
2.02	-0.26	-0.45	0.04	-0.33	-0.09	-0.28	0.13	-0.13	0.35	0.44	0.33
2.07	-0.56	-0.20	-0.20	-0.30	-0.03	-0.13	-0.15	0.03	0.15	0.23	0.33
2.12	-0.31	-0.57	0.08	-0.20	0.03	-0.08	0.19	0.26	0.27	0.39	0.41
2.17	-0.57	-0.85	-0.33	-0.32	-0.46	-0.14	0.13	0.20	0.12	0.28	0.26
2.22	-0.46	-0.48	-0.63	-0.44	-0.02	-0.43	-0.04	0.17	0.08	0.28	0.21
2.27	-0.70	-0.57	-0.36	-0.05	-0.36	-0.26	-0.24	-0.14	0.05	-0.05	0.17
2.32	-0.57	-0.52	-0.39	-0.40	-0.45	-0.12	-0.27	0.12	0.18	0.14	0.04
2.37	-0.16	-0.14	-0.30	-0.04	-0.14	0.02	0.19	0.26	0.19	0.35	0.36
2.42	-0.61	-0.56	-0.32	-0.55	-0.13	-0.07	0.03	0.12	0.03	-0.02	0.20
2.47	-0.08	-0.16	-0.09	-0.03	-0.06	0.07	-0.07	0.26	0.33	0.45	0.49
2.52	-0.46	-0.52	-0.41	-0.35	-0.26	-0.01	-0.12	0.24	0.32	0.42	0.60
2.57	-0.34	-0.18	-0.22	-0.24	0.18	-0.26	0.07	0.19	0.22	0.15	0.34
2.62	-0.51	-0.58	-0.62	-0.13	-0.30	-0.44	-0.30	0.03	0.10	-0.02	0.02
2.67	-0.59	-0.66	-0.18	-0.35	-0.41	-0.14	-0.06	0.22	0.48	0.49	0.46
2.72	-0.68	-0.56	-0.36	-0.28	-0.22	-0.30	-0.19	0.20	0.03	0.21	0.20
2.77	-0.46	-0.52	-0.09	-0.31	-0.09	0.04	0.10	0.00	0.22	0.37	0.44
2.82	-0.37	-0.31	-0.38	-0.35	-0.55	-0.32	-0.08	-0.09	-0.08	0.14	0.18
2.87	-0.31	-0.31	-0.16	-0.63	0.03	0.06	0.12	0.25	0.05	0.19	0.29
2.92	-0.41	-0.59	-0.30	-0.04	0.14	-0.04	-0.05	0.32	0.25	0.09	0.58
2.97	-0.30	-0.79	-0.86	-0.71	-0.31	-0.26	-0.30	-0.14	-0.06	0.01	0.27
3.02	0.08	-0.31	0.27	0.10	-0.02	0.03	0.09	0.16	0.21	0.24	0.25
3.07	-0.63	-0.72	-0.57	-0.20	-0.20	-0.21	-0.23	0.19	0.24	0.40	0.48
3.12	-0.11	-0.15	-0.26	0.14	-0.15	0.00	0.24	0.27	0.16	0.48	0.51
3.17	-0.66	-0.91	-0.46	-0.38	-0.41	-0.38	-0.20	-0.19	0.04	-0.04	0.36
3.22	-0.10	-0.26	-0.23	-0.41	-0.18	-0.24	-0.15	0.20	0.21	0.19	-0.01
3.27	-0.11	-0.61	-0.40	-0.16	-0.31	-0.02	0.04	0.10	0.17	0.16	0.35
3.32	-0.41	-0.32	-0.30	0.06	-0.12	0.06	0.15	0.45	0.30	0.71	0.54
3.37	-0.61	-0.89	-0.47	-0.40	-0.23	-0.28	0.03	0.21	0.22	0.28	0.32
3.42	-0.13	-0.36	0.05	-0.25	-0.27	-0.07	-0.04	0.21	0.10	0.43	0.25
3.47	-0.66	-0.61	-0.44	-0.31	-0.20	-0.09	-0.16	0.38	0.19	0.15	0.33
3.52	-0.07	-0.29	0.04	-0.32	0.06	-0.19	-0.12	-0.08	0.30	0.16	0.60
3.57	-0.29	-0.29	-0.33	-0.22	-0.17	0.04	0.09	0.24	0.30	0.55	0.51
3.62	-0.38	-0.40	-0.24	-0.42	-0.18	-0.15	-0.01	0.10	0.23	0.26	0.27
3.67	0.16	-0.08	-0.11	0.29	-0.05	0.11	0.40	0.43	0.36	0.59	0.59
3.72	-0.38	-0.47	0.02	-0.15	-0.04	-0.06	0.00	0.36	0.22	0.32	0.23
3.92	-0.61	-0.71	-0.37	-0.49	-0.57	-0.29	0.12	0.14	0.02	0.26	0.24
4.12	-0.76	-0.59	-0.37	-0.49	-0.49	-0.21	-0.40	-0.15	-0.13	-0.09	-0.01
4.32	0.22	-0.19	-0.12	0.17	0.14	0.02	0.24	0.53	0.39	0.41	0.66
4.52	-0.50	-0.50	-0.32	-0.38	-0.29	-0.27	-0.21	0.05	0.12	0.34	0.11
4.72	-0.31	-0.58	-0.16	-0.17	-0.47	-0.21	-0.02	0.19	0.18	0.30	0.34
4.92	-0.08	-0.78	-0.27	0.13	-0.43	0.04	-0.24	-0.03	0.16	-0.01	0.24
5.12	-0.51	-0.19	-0.20	-0.12	-0.17	-0.10	0.01	0.35	0.14	0.39	0.53
5.32	-0.58	-0.54	-0.24	-0.52	-0.58	-0.43	0.06	0.28	0.12	0.18	0.47
5.52	-0.54	-0.42	0.04	-0.63	-0.20	-0.14	0.19	0.26	0.30	0.45	0.46

5.72	-0.06	-0.61	-0.43	-0.10	-0.21	-0.24	-0.01	0.13	0.14	0.27	0.51
5.92	-0.14	-0.30	-0.30	-0.19	-0.20	-0.16	0.17	0.03	0.18	0.10	0.40
6.12	-0.08	-0.61	-0.52	-0.26	-0.11	-0.01	0.05	0.22	0.13	0.25	0.23
6.32	-0.31	-0.34	-0.28	0.00	-0.12	0.14	0.04	0.29	0.22	0.30	0.47
6.52	-0.40	-0.56	-0.27	-0.36	0.27	-0.09	0.19	0.47	0.11	0.25	0.43
6.72	-0.36	-0.33	-0.18	-0.35	-0.07	-0.27	-0.02	0.16	0.22	0.10	0.31
6.92	-0.33	-0.53	-0.19	-0.25	0.07	-0.48	-0.13	0.10	0.25	0.34	0.59
7.12	-0.83	-0.36	-0.15	-0.64	-0.26	0.15	0.00	0.03	0.30	0.41	0.29
7.32	-0.32	-0.55	-0.07	0.05	0.00	-0.11	-0.06	0.37	0.43	0.26	0.35
7.52	-0.45	-0.28	-0.43	-0.42	-0.27	-0.11	0.09	0.24	0.13	0.16	0.22
7.72	-0.05	-0.41	-0.11	0.08	-0.34	0.18	0.15	0.29	0.37	0.29	0.16
7.92	-0.52	-0.65	-0.32	-0.53	-0.36	-0.17	-0.22	0.33	0.15	0.20	0.26
8.12	-0.17	-0.28	0.13	-0.11	-0.14	0.06	0.27	0.16	0.04	0.42	0.40
8.32	-0.32	-0.15	-0.46	-0.14	0.02	-0.19	0.14	0.35	0.19	0.21	0.40
8.52	-0.43	-0.80	-0.58	-0.41	-0.53	-0.48	0.01	-0.25	0.12	0.21	0.02
8.72	-0.78	-0.29	-0.30	-0.71	-0.25	-0.25	-0.15	-0.08	-0.02	0.05	0.05
8.92	-0.16	-0.07	-0.08	0.00	-0.01	0.06	-0.20	0.16	0.31	0.35	0.44
9.12	-0.48	-0.52	-0.24	-0.39	-0.02	-0.23	-0.17	-0.06	0.17	0.33	0.20
9.32	0.32	-0.19	-0.09	-0.18	-0.06	0.18	0.18	0.37	0.48	0.35	0.48
9.52	-0.36	-0.65	-0.14	-0.36	-0.23	-0.11	-0.01	0.32	0.12	0.19	0.32
9.72	-0.32	-0.21	-0.38	-0.33	-0.09	-0.10	0.09	0.35	0.46	0.41	0.46
9.92	-0.06	-0.44	-0.45	-0.21	0.21	-0.01	-0.07	0.41	0.36	0.36	0.19
10.12	0.27	-0.83	-0.26	-0.13	-0.11	0.03	0.37	0.41	0.31	0.43	0.60
10.32	-0.06	-0.26	-0.14	0.08	0.08	-0.12	0.10	0.34	0.49	0.37	0.38
10.52	-0.55	-0.62	-0.41	-0.33	-0.24	-0.17	-0.05	0.19	0.03	0.24	0.32
10.72	-0.04	-0.27	0.14	0.27	0.20	0.18	0.19	0.31	0.38	0.43	0.59
10.92	-0.17	-0.55	-0.04	-0.06	-0.17	-0.18	0.00	0.09	0.23	0.27	0.21
11.12	-0.59	-0.29	-0.16	-0.37	-0.33	-0.20	-0.17	0.04	0.16	0.27	0.23
11.32	-0.34	-0.47	-0.28	-0.12	0.17	-0.03	0.13	0.59	0.58	0.30	0.45
11.52	-0.21	-0.66	-0.46	-0.24	-0.25	-0.24	0.05	-0.01	0.30	0.10	0.15
11.72	-0.09	-0.50	0.14	0.05	-0.34	-0.14	-0.12	0.11	0.25	0.22	0.16
11.92	-0.25	0.11	0.12	0.21	0.36	0.40	0.35	0.69	0.72	0.55	0.55
12.12	-0.27	-0.18	-0.35	0.01	0.07	0.07	0.10	0.36	0.39	0.36	0.35
12.32	0.15	-0.08	0.29	-0.29	0.27	0.20	0.15	0.40	0.35	0.42	0.23
12.52	-0.50	-0.40	-0.20	0.02	-0.12	0.20	0.13	0.12	0.33	0.50	0.35
12.72	-0.21	-0.61	-0.54	-0.33	-0.46	-0.36	-0.02	0.09	0.07	0.14	0.22
12.92	-0.12	-0.31	-0.07	0.06	-0.14	-0.28	0.04	0.37	0.41	0.43	0.43
13.12	-0.30	-0.21	0.02	-0.25	0.22	0.13	0.07	0.15	0.56	0.31	0.51
13.32	-0.24	-0.80	-0.52	-0.16	0.01	-0.20	-0.22	0.35	0.42	0.34	0.41
13.52	-0.39	-0.65	-0.39	-0.55	-0.28	-0.13	-0.25	-0.10	-0.13	-0.01	0.17
13.72	-0.17	-0.38	-0.37	-0.12	0.22	0.00	0.00	0.30	0.45	0.40	0.49
13.92	-0.11	-0.13	-0.25	-0.52	-0.06	0.05	0.17	-0.13	0.40	0.37	0.26
14.00	-0.04	-0.21	0.03	-0.05	0.22	0.02	0.13	0.17	0.20	0.16	0.34
14.08	-0.55	-0.92	-0.08	-0.25	-0.19	-0.16	0.12	-0.44	-0.15	0.11	0.14
14.17	-0.32	-0.56	-0.23	-0.35	-0.33	-0.13	-0.18	0.00	-0.03	0.10	0.05
14.27	-0.16	-0.28	-0.25	-0.17	0.01	0.15	0.06	-0.01	0.24	0.26	0.28
14.38	-0.39	-0.40	-0.12	-0.29	-0.08	0.11	-0.18	0.33	0.20	0.05	0.22
14.49	-0.13	-0.51	0.13	-0.34	0.16	-0.11	0.28	0.10	0.25	0.45	0.53
14.61	-0.41	-0.29	-0.45	-0.07	-0.05	-0.13	0.01	-0.03	0.11	0.18	0.53
14.75	-0.49	-0.65	-0.03	-0.26	-0.18	-0.11	0.08	0.34	0.16	0.16	0.02

14.89	-0.65	-0.33	-0.25	-0.04	-0.40	-0.14	-0.17	-0.23	0.09	-0.01	0.21
15.05	0.28	0.08	0.02	-0.22	0.16	0.15	0.25	0.65	0.31	0.42	0.70
15.21	0.11	-0.49	-0.33	-0.08	0.00	0.13	0.08	0.33	0.22	0.35	0.38
15.39	-0.03	-0.49	-0.06	-0.19	-0.13	-0.02	0.10	0.22	0.46	0.15	0.12
15.59	-0.38	-0.50	-0.28	-0.12	-0.13	-0.23	0.15	0.09	0.28	0.04	0.18
15.80	-0.55	-0.68	-0.36	-0.02	-0.38	-0.24	0.01	0.22	-0.17	-0.04	0.22
16.02	0.06	-0.67	-0.46	-0.65	-0.39	-0.15	0.05	0.15	-0.01	0.01	0.13
16.27	-0.63	-0.85	-0.51	-0.26	-0.43	-0.44	0.08	-0.15	0.03	0.18	0.02
16.53	-0.69	-0.69	-0.32	-0.25	-0.40	-0.09	0.19	0.26	0.16	0.35	0.25
16.81	-0.10	-0.24	0.02	-0.50	-0.01	-0.29	-0.01	-0.11	0.16	0.07	0.33
17.12	-0.60	-0.32	-0.22	-0.40	-0.15	0.02	-0.16	0.05	0.01	0.34	0.31
17.45	-0.48	-0.33	-0.50	-0.44	-0.22	-0.27	-0.14	-0.16	-0.19	-0.02	-0.08
17.81	-0.21	-0.24	-0.48	-0.31	-0.39	-0.20	0.00	-0.03	0.05	0.14	0.14
18.19	-0.34	-0.49	-0.20	-0.04	0.02	0.08	0.04	0.20	0.14	0.38	0.30
18.60	-0.26	-0.27	0.18	-0.49	0.18	-0.28	0.04	0.10	0.13	0.13	0.24
19.05	-0.26	-0.22	-0.21	-0.03	-0.06	-0.08	-0.02	0.18	-0.04	0.03	-0.17
19.53	0.06	-0.24	0.08	0.28	0.01	0.06	0.13	0.32	0.47	0.45	0.50
20.05	-0.18	-0.11	0.47	-0.28	0.00	-0.19	0.23	0.66	0.42	0.53	0.21
20.61	-0.27	-0.34	-0.27	-0.02	0.39	-0.13	-0.14	0.36	0.37	0.36	0.54
21.22	-0.45	-0.52	-0.07	-0.04	-0.11	0.32	0.39	0.19	0.26	0.24	0.17
21.87	-0.29	-0.28	-0.37	-0.13	-0.05	-0.23	0.18	0.10	0.28	0.30	0.36
22.57	0.26	0.04	0.31	0.41	0.26	0.41	0.19	0.47	0.24	0.32	0.43
23.33	-0.49	-0.31	-0.11	-0.27	-0.27	0.03	-0.13	0.09	0.13	0.38	0.36
24.15	-0.12	-0.28	-0.29	0.04	-0.01	0.12	0.16	0.32	0.28	0.53	0.36
25.03	-0.52	-0.49	-0.39	-0.04	-0.22	0.00	0.03	0.18	0.13	0.21	0.39
25.98	-0.28	-0.36	-0.19	-0.28	-0.32	-0.21	0.03	0.06	0.17	0.14	0.23
27.01	-0.25	-0.12	-0.10	0.01	-0.04	0.08	-0.11	0.29	0.28	0.41	0.42
28.11	-0.21	-0.38	-0.05	-0.18	-0.07	-0.01	-0.04	0.24	0.26	0.42	0.50
29.30	-0.40	-0.33	-0.18	0.18	-0.09	-0.08	0.07	0.09	0.32	0.07	0.28
30.59	-0.22	0.08	-0.25	0.28	0.07	0.03	0.29	0.42	0.32	0.57	0.35
31.98	-0.31	-0.31	-0.41	-0.07	0.07	-0.03	0.22	0.23	0.22	0.34	0.37
33.47	-0.66	-0.53	-0.27	-0.48	-0.31	0.11	-0.06	0.24	0.23	0.09	0.43
35.09	-0.16	-0.24	0.05	-0.08	-0.03	0.20	0.27	0.44	0.49	0.54	0.40
36.83	0.09	-0.31	-0.31	-0.44	-0.20	0.01	0.02	-0.01	0.18	0.17	0.31
38.71	-0.29	-0.40	-0.03	0.06	-0.30	-0.07	0.14	0.11	0.22	0.27	0.35
40.73	-0.21	-0.26	-0.26	0.03	-0.11	0.22	-0.33	0.22	0.40	0.25	0.20
42.92	-0.35	-0.21	-0.25	-0.08	0.20	0.29	0.19	0.20	0.30	0.36	0.53
45.27	-0.17	-0.58	-0.56	-0.49	-0.13	-0.09	-0.29	-0.01	0.08	-0.02	0.22
47.81	-0.08	-0.18	-0.26	-0.21	0.09	0.13	0.01	0.37	0.17	0.19	0.27
50.55	-0.21	-0.24	0.07	0.00	0.15	-0.18	0.01	0.35	0.33	0.54	0.37
53.51	-0.21	-0.30	0.06	0.03	-0.07	0.02	0.15	0.56	0.25	0.02	0.50
56.69	-0.63	-0.56	-0.21	-0.18	-0.52	-0.16	0.13	0.18	0.19	0.48	0.20
60.13	-0.60	-0.20	0.16	-0.19	0.31	0.08	-0.06	0.23	0.54	0.14	0.47
63.84	0.02	-0.21	0.05	-0.08	0.02	0.12	0.37	0.37	0.19	0.49	0.61
67.83	-0.40	-0.33	-0.17	0.03	0.13	0.24	0.04	0.40	0.43	0.33	0.47
72.15	0.01	-0.37	-0.12	-0.29	0.12	-0.07	0.06	0.39	0.21	0.45	0.28
76.80	-0.55	-0.11	0.07	-0.32	-0.34	-0.06	-0.05	0.16	0.32	0.19	0.25
81.81	-0.55	-0.58	0.10	-0.34	-0.57	0.05	0.03	0.03	0.14	0.22	0.28
87.22	-0.34	-0.37	-0.69	0.02	-0.25	-0.11	-0.10	0.28	0.12	0.20	0.32
93.06	-0.10	-0.31	-0.11	0.09	0.15	0.08	0.22	0.10	0.30	0.41	0.38

99.35	-0.08	-0.07	0.28	0.00	0.11	0.25	0.14	0.16	0.48	0.29	0.44
106.13	-0.40	-0.15	-0.02	-0.17	-0.10	-0.05	0.44	0.47	0.50	0.73	0.67
113.45	-0.39	-0.43	-0.08	-0.37	-0.34	0.05	-0.03	0.21	0.16	0.48	0.18
121.34	-0.49	-0.42	-0.10	-0.09	0.05	-0.21	0.10	0.25	0.48	0.49	0.35
129.86	0.13	-0.34	-0.11	0.04	0.06	0.06	0.28	0.36	0.46	0.42	0.59
139.04	0.09	0.01	0.07	0.18	0.10	0.09	0.36	0.57	0.43	0.48	0.62
148.94	-0.35	-0.46	-0.32	-0.43	0.01	-0.07	-0.14	0.22	0.17	-0.01	0.25
159.62	-0.15	-0.15	0.00	0.17	0.16	0.11	0.10	0.51	0.24	0.53	0.44
171.14	-0.38	-0.29	-0.37	-0.19	-0.01	0.02	-0.12	0.21	0.19	0.24	0.30
183.56	-0.12	-0.30	0.36	0.30	0.49	0.37	0.30	0.60	0.46	0.57	0.43
196.96	-0.59	-0.40	-0.60	-0.37	-0.11	-0.10	-0.05	0.04	0.21	0.33	0.52
211.41	-0.14	-0.63	-0.30	-0.04	-0.11	-0.02	0.00	0.44	0.20	0.11	0.26
226.99	0.28	-0.03	-0.06	0.14	0.03	0.33	0.18	0.22	0.33	0.11	0.27
243.80	0.03	-0.36	-0.25	-0.32	-0.10	-0.11	0.05	0.25	0.17	0.28	0.34
261.93	0.04	0.24	-0.01	0.13	0.12	0.28	0.29	0.51	0.45	0.14	0.43
281.48	-0.05	-0.25	-0.14	-0.04	-0.13	0.33	0.53	0.43	0.44	0.44	0.52
302.56	0.11	-0.56	-0.19	0.04	0.04	0.26	-0.01	0.35	0.13	0.25	0.21
325.30	0.09	-0.12	-0.11	0.12	-0.02	0.16	0.34	0.25	0.34	0.42	0.39
349.83	-0.25	-0.16	-0.03	0.12	-0.07	-0.05	0.21	0.17	0.20	0.19	0.06
376.28	-0.07	-0.42	-0.13	-0.10	0.26	0.28	0.23	0.30	0.26	0.23	0.31
404.81	-0.28	-0.26	-0.09	-0.46	0.14	-0.16	0.10	0.25	0.51	0.30	0.31
435.58	-0.54	-0.33	0.03	0.01	0.05	0.12	0.48	0.29	0.42	0.45	0.43
468.76	-0.51	-0.44	-0.12	-0.24	-0.03	-0.04	0.02	0.10	0.35	0.01	0.22
504.55	-0.40	-0.32	-0.13	0.06	-0.27	-0.10	-0.05	0.42	0.19	0.07	0.31
543.15	-0.46	0.06	0.22	-0.06	-0.01	0.06	0.17	0.19	0.40	0.09	0.34
584.78	0.07	0.23	0.17	0.00	0.08	-0.07	0.11	0.27	0.31	0.38	0.40
629.68	-0.24	-0.25	-0.02	-0.36	-0.08	0.13	0.17	0.09	0.02	0.14	0.25
678.10	-0.05	-0.02	0.42	0.10	0.15	0.17	0.14	0.22	0.45	0.28	0.56
730.33	-0.16	0.16	0.16	0.00	0.05	0.06	0.11	0.37	0.64	0.46	0.66
786.65	-0.51	-0.53	-0.14	0.05	-0.05	-0.20	-0.32	0.18	0.35	0.15	0.24
847.40	0.03	-0.40	-0.02	-0.40	-0.30	0.03	-0.13	0.20	0.17	0.12	0.10
912.92	-0.05	-0.24	-0.03	-0.29	0.20	-0.33	-0.04	0.38	0.09	0.18	0.46
983.58	-0.57	-0.37	0.07	-0.15	0.05	-0.04	0.05	0.22	0.23	0.34	0.30
1059.78	-0.06	-0.39	-0.60	-0.35	-0.15	-0.29	-0.03	0.34	0.32	0.49	0.46
1141.98	-0.07	-0.22	0.04	0.23	0.22	-0.01	0.19	0.32	0.41	0.34	0.52
1230.62	0.10	0.11	-0.23	0.28	0.06	0.30	0.33	0.33	0.28	0.34	0.42
1326.23	-0.16	0.66	0.25	0.07	0.27	0.41	0.32	0.65	0.56	0.30	0.55
1429.34	-0.34	-0.46	-0.32	-0.41	-0.62	-0.41	-0.23	-0.22	0.07	-0.08	0.27
1540.54	-0.17	-0.36	-0.26	0.25	-0.23	-0.15	0.08	0.31	0.03	0.42	0.52
1660.48	0.09	-0.25	-0.33	0.20	0.09	0.13	0.18	0.32	0.40	0.49	0.57
1789.83	0.11	0.01	-0.02	-0.27	0.05	0.01	0.05	0.32	0.28	0.37	0.42
1929.34	-0.14	0.07	-0.13	0.07	0.29	-0.01	0.27	0.31	0.41	0.39	0.33
2079.80	-0.22	-0.41	-0.31	-0.40	0.04	-0.13	0.14	0.02	0.12	0.29	0.16
2242.08	-0.22	-0.17	-0.26	-0.40	-0.01	-0.11	-0.11	0.13	0.28	0.19	0.24
2417.10	-0.54	-0.39	-0.30	-0.33	-0.28	-0.08	-0.22	0.21	-0.09	0.07	-0.03
2605.85	-0.05	-0.20	-0.10	0.00	-0.01	-0.18	-0.02	0.17	0.22	-0.03	0.14
2809.43	-0.15	-0.24	-0.01	-0.03	0.18	0.20	0.04	0.29	0.35	0.29	0.44
3028.99	-0.45	-0.17	0.07	-0.03	-0.10	0.18	0.32	0.42	0.51	0.38	0.53
3265.79	-0.22	-0.18	-0.16	-0.33	-0.11	0.23	-0.11	-0.01	0.28	0.36	0.18
3521.18	-0.22	-0.14	0.00	0.05	0.31	0.22	0.07	0.27	0.38	0.57	0.21

3796.62	0.14	-0.01	-0.01	-0.08	0.52	0.39	0.26	0.40	0.26	0.32	0.40

Wavelength (nm) / Time (ps)	351.69	353.10	354.51	355.92	357.33	358.73	360.14	361.55	362.96	364.37	365.78
-3.28	0.00	0.00	0.00	0.00	0.00	0.00	0.00	0.00	0.00	0.00	0.00
-2.78	0.00	0.00	0.00	0.00	0.00	0.00	0.00	0.00	0.00	0.00	0.00
-2.28	0.00	0.00	0.00	0.00	0.00	0.00	0.00	0.00	0.00	0.00	0.00
-1.78	0.00	0.00	0.00	0.00	0.00	0.00	0.00	0.00	0.00	0.00	0.00
-1.28	0.00	0.00	0.00	0.00	0.00	0.00	0.00	0.00	0.00	0.00	0.00
-0.78	0.00	0.00	0.00	0.00	0.00	0.00	0.00	0.00	0.00	0.00	0.00
-0.28	0.00	0.00	0.00	0.00	0.00	0.00	0.00	0.00	0.00	0.00	0.00
0.22	0.00	0.00	0.00	0.00	0.00	0.00	0.00	0.00	0.00	0.00	0.00
0.32	0.00	0.00	0.00	0.00	0.00	0.00	0.00	0.00	0.00	0.00	0.00
0.42	0.00	0.00	0.00	0.00	0.00	0.00	0.00	0.00	0.00	0.00	0.00
0.52	0.00	0.00	0.00	0.00	0.00	0.00	0.00	0.00	0.00	0.00	0.00
0.62	0.00	0.00	0.00	0.00	0.00	0.00	0.00	0.00	0.00	0.00	0.00
0.72	0.00	0.00	0.00	0.00	0.00	0.00	0.00	0.00	0.00	0.00	0.00
0.77	0.00	0.00	0.00	0.00	0.00	0.00	0.00	0.00	0.00	0.00	0.00
0.82	0.00	0.00	0.00	0.00	0.00	0.00	0.00	0.00	0.00	0.00	0.00
0.87	0.00	0.00	0.00	0.00	0.00	0.00	0.00	0.00	0.00	0.00	0.00
0.92	0.00	0.00	0.00	0.00	0.00	0.00	0.00	0.00	0.00	0.00	0.00
0.97	0.00	0.00	0.00	0.00	0.00	0.00	0.00	0.00	0.00	0.00	0.00
1.02	0.00	0.00	0.00	0.00	0.00	0.00	0.00	0.00	0.00	0.00	0.00
1.07	0.00	0.00	0.00	0.00	0.00	0.00	0.00	0.00	0.00	0.00	0.00
1.12	0.00	0.00	0.00	0.00	0.00	0.00	0.00	0.00	0.00	0.00	0.00
1.17	0.00	0.00	0.00	0.00	0.00	0.00	0.00	0.00	0.00	0.00	0.00
1.22	0.00	0.00	0.00	0.00	0.00	0.00	0.00	0.00	0.00	0.00	0.00
1.27	0.00	0.00	0.00	0.00	0.00	0.00	0.00	0.00	0.00	0.00	0.00
1.32	0.00	0.00	0.00	0.00	0.00	0.00	0.00	0.00	0.00	0.00	0.00
1.37	0.00	0.00	0.00	0.00	0.00	0.00	0.00	0.00	0.00	0.00	0.00
1.42	0.00	0.00	0.00	0.00	0.00	0.00	0.00	0.00	0.00	0.00	0.00
1.47	0.00	0.00	0.00	0.00	0.00	0.00	0.00	0.00	0.00	0.00	0.00
1.52	0.00	0.00	0.00	0.00	0.00	0.00	0.00	0.00	0.00	0.00	0.00
1.57	0.00	0.00	0.00	0.00	0.00	0.00	0.00	0.00	0.00	0.00	0.00
1.62	0.00	0.00	0.00	0.00	0.00	0.00	0.00	0.00	0.00	0.00	0.00
1.67	0.00	0.00	0.00	0.00	0.00	0.00	0.00	0.00	0.00	0.00	0.00
1.72	0.00	0.00	0.00	0.00	0.00	0.00	0.00	0.00	0.00	0.00	0.00
1.77	0.00	0.00	0.00	0.00	0.00	0.00	0.00	0.00	0.00	0.00	0.00
1.82	0.00	0.00	0.00	0.00	0.00	0.00	0.00	0.00	0.00	0.00	0.00
1.87	0.00	0.00	0.00	0.00	0.00	0.00	0.00	0.00	0.00	0.00	0.00
1.92	0.00	0.00	0.00	0.00	0.00	0.00	0.00	0.00	0.00	0.00	0.00
1.97	0.00	0.00	0.00	0.00	0.00	0.00	0.00	0.00	0.00	0.00	0.00
2.02	0.00	0.00	0.00	0.00	0.00	0.00	0.00	0.00	0.00	0.00	0.00
2.07	0.00	0.00	0.00	0.00	0.00	0.00	0.00	0.00	0.00	0.00	0.00
2.12	0.00	0.00	0.00	0.00	0.00	0.00	0.00	0.00	0.00	0.00	0.00
2.17	0.00	0.00	0.00	0.00	0.00	0.00	0.00	0.00	0.00	0.00	0.00
2.22	0.00	0.00	0.00	0.00	0.00	0.00	0.00	0.00	0.00	0.00	0.00
2.27	0.00	0.00	0.00	0.00	0.00	0.00	0.00	0.00	0.00	0.00	0.00
2.32	0.00	0.00	0.00	0.00	0.00	0.00	0.00	0.00	0.00	0.00	0.00

2.37	0.00	0.00	0.00	0.00	0.00	0.00	0.00	0.00	0.00	0.00	0.00
2.42	0.00	0.00	0.00	0.00	0.00	0.00	0.00	0.00	0.00	0.00	0.00
2.47	0.00	0.00	0.00	0.00	0.00	0.00	0.00	0.00	0.00	0.00	0.00
2.52	0.00	0.00	0.00	0.00	0.00	0.00	0.00	0.00	0.00	0.00	0.00
2.57	0.00	0.00	0.00	0.00	0.00	0.00	0.00	0.00	0.00	0.00	0.00
2.62	0.00	0.00	0.00	0.00	0.00	0.00	0.00	0.00	0.00	0.00	0.00
2.67	0.00	0.00	0.00	0.00	0.00	0.00	0.00	0.00	0.00	0.00	0.00
2.72	0.00	0.00	0.00	0.00	0.00	0.00	0.00	0.00	0.00	0.00	0.00
2.77	0.00	0.00	0.00	0.00	0.00	0.00	0.00	0.00	0.00	0.00	0.00
2.82	0.00	0.00	0.00	0.00	0.00	0.00	0.00	0.00	0.00	0.00	0.00
2.87	0.00	0.00	0.00	0.00	0.00	0.00	0.00	0.00	0.00	0.00	0.00
2.92	0.00	0.00	0.00	0.00	0.00	0.00	0.00	0.00	0.00	0.00	0.00
2.97	0.00	0.00	0.00	0.00	0.00	0.00	0.00	0.00	0.00	0.00	0.00
3.02	0.00	0.00	0.00	0.00	0.00	0.00	0.00	0.00	0.00	0.00	0.00
3.07	0.00	0.00	0.00	0.00	0.00	0.00	0.00	0.00	0.00	0.00	0.00
3.12	0.00	0.00	0.00	0.00	0.00	0.00	0.00	0.00	0.00	0.00	0.00
3.17	0.00	0.00	0.00	0.00	0.00	0.00	0.00	0.00	0.00	0.00	0.00
3.22	0.00	0.00	0.00	0.00	0.00	0.00	0.00	0.00	0.00	0.00	0.00
3.27	0.00	0.00	0.00	0.00	0.00	0.00	0.00	0.00	0.00	0.00	0.00
3.32	0.00	0.00	0.00	0.00	0.00	0.00	0.00	0.00	0.00	0.00	0.00
3.37	0.00	0.00	0.00	0.00	0.00	0.00	0.00	0.00	0.00	0.00	0.00
3.42	0.00	0.00	0.00	0.00	0.00	0.00	0.00	0.00	0.00	0.00	0.00
3.47	0.00	0.00	0.00	0.00	0.00	0.00	0.00	0.00	0.00	0.00	0.00
3.52	0.00	0.00	0.00	0.00	0.00	0.00	0.00	0.00	0.00	0.00	0.00
3.57	0.00	0.00	0.00	0.00	0.00	0.00	0.00	0.00	0.00	0.00	0.00
3.62	0.00	0.00	0.00	0.00	0.00	0.00	0.00	0.00	0.00	0.00	0.00
3.67	0.00	0.00	0.00	0.00	0.00	0.00	0.00	0.00	0.00	0.00	0.00
3.72	0.00	0.00	0.00	0.00	0.00	0.00	0.00	0.00	0.00	0.00	0.00
3.92	0.00	0.00	0.00	0.00	0.00	0.00	0.00	0.00	0.00	0.00	0.00
4.12	0.00	0.00	0.00	0.00	0.00	0.00	0.00	0.00	0.00	0.00	0.00
4.32	0.00	0.00	0.00	0.00	0.00	0.00	0.00	0.00	0.00	0.00	0.00
4.52	0.00	0.00	0.00	0.00	0.00	0.00	0.00	0.00	0.00	0.00	0.00
4.72	0.00	0.00	0.00	0.00	0.00	0.00	0.00	0.00	0.00	0.00	0.00
4.92	0.00	0.00	0.00	0.00	0.00	0.00	0.00	0.00	0.00	0.00	0.00
5.12	0.00	0.00	0.00	0.00	0.00	0.00	0.00	0.00	0.00	0.00	0.00
5.32	0.00	0.00	0.00	0.00	0.00	0.00	0.00	0.00	0.00	0.00	0.00
5.52	0.00	0.00	0.00	0.00	0.00	0.00	0.00	0.00	0.00	0.00	0.00
5.72	0.00	0.00	0.00	0.00	0.00	0.00	0.00	0.00	0.00	0.00	0.00
5.92	0.00	0.00	0.00	0.00	0.00	0.00	0.00	0.00	0.00	0.00	0.00
6.12	0.00	0.00	0.00	0.00	0.00	0.00	0.00	0.00	0.00	0.00	0.00
6.32	0.00	0.00	0.00	0.00	0.00	0.00	0.00	0.00	0.00	0.00	0.00
6.52	0.00	0.00	0.00	0.00	0.00	0.00	0.00	0.00	0.00	0.00	0.00
6.72	0.00	0.00	0.00	0.00	0.00	0.00	0.00	0.00	0.00	0.00	0.00
6.92	0.00	0.00	0.00	0.00	0.00	0.00	0.00	0.00	0.00	0.00	0.00
7.12	0.00	0.00	0.00	0.00	0.00	0.00	0.00	0.00	0.00	0.00	0.00
7.32	0.00	0.00	0.00	0.00	0.00	0.00	0.00	0.00	0.00	0.00	0.00
7.52	0.00	0.00	0.00	0.00	0.00	0.00	0.00	0.00	0.00	0.00	0.00
7.72	0.00	0.00	0.00	0.00	0.00	0.00	0.00	0.00	0.00	0.00	0.00
7.92	0.00	0.00	0.00	0.00	0.00	0.00	0.00	0.00	0.00	0.00	0.00
8.12	0.00	0.00	0.00	0.00	0.00	0.00	0.00	0.00	0.00	0.00	0.00

8.32	0.00	0.00	0.00	0.00	0.00	0.00	0.00	0.00	0.00	0.00	0.00
8.52	0.00	0.00	0.00	0.00	0.00	0.00	0.00	0.00	0.00	0.00	0.00
8.72	0.00	0.00	0.00	0.00	0.00	0.00	0.00	0.00	0.00	0.00	0.00
8.92	0.00	0.00	0.00	0.00	0.00	0.00	0.00	0.00	0.00	0.00	0.00
9.12	0.00	0.00	0.00	0.00	0.00	0.00	0.00	0.00	0.00	0.00	0.00
9.32	0.00	0.00	0.00	0.00	0.00	0.00	0.00	0.00	0.00	0.00	0.00
9.52	0.00	0.00	0.00	0.00	0.00	0.00	0.00	0.00	0.00	0.00	0.00
9.72	0.00	0.00	0.00	0.00	0.00	0.00	0.00	0.00	0.00	0.00	0.00
9.92	0.00	0.00	0.00	0.00	0.00	0.00	0.00	0.00	0.00	0.00	0.00
10.12	0.00	0.00	0.00	0.00	0.00	0.00	0.00	0.00	0.00	0.00	0.00
10.32	0.00	0.00	0.00	0.00	0.00	0.00	0.00	0.00	0.00	0.00	0.00
10.52	0.00	0.00	0.00	0.00	0.00	0.00	0.00	0.00	0.00	0.00	0.00
10.72	0.00	0.00	0.00	0.00	0.00	0.00	0.00	0.00	0.00	0.00	0.00
10.92	0.00	0.00	0.00	0.00	0.00	0.00	0.00	0.00	0.00	0.00	0.00
11.12	0.00	0.00	0.00	0.00	0.00	0.00	0.00	0.00	0.00	0.00	0.00
11.32	0.00	0.00	0.00	0.00	0.00	0.00	0.00	0.00	0.00	0.00	0.00
11.52	0.00	0.00	0.00	0.00	0.00	0.00	0.00	0.00	0.00	0.00	0.00
11.72	0.00	0.00	0.00	0.00	0.00	0.00	0.00	0.00	0.00	0.00	0.00
11.92	0.00	0.00	0.00	0.00	0.00	0.00	0.00	0.00	0.00	0.00	0.00
12.12	0.00	0.00	0.00	0.00	0.00	0.00	0.00	0.00	0.00	0.00	0.00
12.32	0.00	0.00	0.00	0.00	0.00	0.00	0.00	0.00	0.00	0.00	0.00
12.52	0.00	0.00	0.00	0.00	0.00	0.00	0.00	0.00	0.00	0.00	0.00
12.72	0.00	0.00	0.00	0.00	0.00	0.00	0.00	0.00	0.00	0.00	0.00
12.92	0.00	0.00	0.00	0.00	0.00	0.00	0.00	0.00	0.00	0.00	0.00
13.12	0.00	0.00	0.00	0.00	0.00	0.00	0.00	0.00	0.00	0.00	0.00
13.32	0.00	0.00	0.00	0.00	0.00	0.00	0.00	0.00	0.00	0.00	0.00
13.52	0.00	0.00	0.00	0.00	0.00	0.00	0.00	0.00	0.00	0.00	0.00
13.72	0.00	0.00	0.00	0.00	0.00	0.00	0.00	0.00	0.00	0.00	0.00
13.92	0.00	0.00	0.00	0.00	0.00	0.00	0.00	0.00	0.00	0.00	0.00
14.00	0.00	0.00	0.00	0.00	0.00	0.00	0.00	0.00	0.00	0.00	0.00
14.08	0.00	0.00	0.00	0.00	0.00	0.00	0.00	0.00	0.00	0.00	0.00
14.17	0.00	0.00	0.00	0.00	0.00	0.00	0.00	0.00	0.00	0.00	0.00
14.27	0.00	0.00	0.00	0.00	0.00	0.00	0.00	0.00	0.00	0.00	0.00
14.38	0.00	0.00	0.00	0.00	0.00	0.00	0.00	0.00	0.00	0.00	0.00
14.49	0.00	0.00	0.00	0.00	0.00	0.00	0.00	0.00	0.00	0.00	0.00
14.61	0.00	0.00	0.00	0.00	0.00	0.00	0.00	0.00	0.00	0.00	0.00
14.75	0.00	0.00	0.00	0.00	0.00	0.00	0.00	0.00	0.00	0.00	0.00
14.89	0.00	0.00	0.00	0.00	0.00	0.00	0.00	0.00	0.00	0.00	0.00
15.05	0.00	0.00	0.00	0.00	0.00	0.00	0.00	0.00	0.00	0.00	0.00
15.21	0.00	0.00	0.00	0.00	0.00	0.00	0.00	0.00	0.00	0.00	0.00
15.39	0.00	0.00	0.00	0.00	0.00	0.00	0.00	0.00	0.00	0.00	0.00
15.59	0.00	0.00	0.00	0.00	0.00	0.00	0.00	0.00	0.00	0.00	0.00
15.80	0.00	0.00	0.00	0.00	0.00	0.00	0.00	0.00	0.00	0.00	0.00
16.02	0.00	0.00	0.00	0.00	0.00	0.00	0.00	0.00	0.00	0.00	0.00
16.27	0.00	0.00	0.00	0.00	0.00	0.00	0.00	0.00	0.00	0.00	0.00
16.53	0.00	0.00	0.00	0.00	0.00	0.00	0.00	0.00	0.00	0.00	0.00
16.81	0.00	0.00	0.00	0.00	0.00	0.00	0.00	0.00	0.00	0.00	0.00
17.12	0.00	0.00	0.00	0.00	0.00	0.00	0.00	0.00	0.00	0.00	0.00
17.45	0.00	0.00	0.00	0.00	0.00	0.00	0.00	0.00	0.00	0.00	0.00
17.81	0.00	0.00	0.00	0.00	0.00	0.00	0.00	0.00	0.00	0.00	0.00

18.19	0.00	0.00	0.00	0.00	0.00	0.00	0.00	0.00	0.00	0.00	0.00
18.60	0.00	0.00	0.00	0.00	0.00	0.00	0.00	0.00	0.00	0.00	0.00
19.05	0.00	0.00	0.00	0.00	0.00	0.00	0.00	0.00	0.00	0.00	0.00
19.53	0.00	0.00	0.00	0.00	0.00	0.00	0.00	0.00	0.00	0.00	0.00
20.05	0.00	0.00	0.00	0.00	0.00	0.00	0.00	0.00	0.00	0.00	0.00
20.61	0.00	0.00	0.00	0.00	0.00	0.00	0.00	0.00	0.00	0.00	0.00
21.22	0.00	0.00	0.00	0.00	0.00	0.00	0.00	0.00	0.00	0.00	0.00
21.87	0.00	0.00	0.00	0.00	0.00	0.00	0.00	0.00	0.00	0.00	0.00
22.57	0.00	0.00	0.00	0.00	0.00	0.00	0.00	0.00	0.00	0.00	0.00
23.33	0.00	0.00	0.00	0.00	0.00	0.00	0.00	0.00	0.00	0.00	0.00
24.15	0.00	0.00	0.00	0.00	0.00	0.00	0.00	0.00	0.00	0.00	0.00
25.03	0.00	0.00	0.00	0.00	0.00	0.00	0.00	0.00	0.00	0.00	0.00
25.98	0.00	0.00	0.00	0.00	0.00	0.00	0.00	0.00	0.00	0.00	0.00
27.01	0.00	0.00	0.00	0.00	0.00	0.00	0.00	0.00	0.00	0.00	0.00
28.11	0.00	0.00	0.00	0.00	0.00	0.00	0.00	0.00	0.00	0.00	0.00
29.30	0.00	0.00	0.00	0.00	0.00	0.00	0.00	0.00	0.00	0.00	0.00
30.59	0.00	0.00	0.00	0.00	0.00	0.00	0.00	0.00	0.00	0.00	0.00
31.98	0.00	0.00	0.00	0.00	0.00	0.00	0.00	0.00	0.00	0.00	0.00
33.47	0.00	0.00	0.00	0.00	0.00	0.00	0.00	0.00	0.00	0.00	0.00
35.09	0.00	0.00	0.00	0.00	0.00	0.00	0.00	0.00	0.00	0.00	0.00
36.83	0.00	0.00	0.00	0.00	0.00	0.00	0.00	0.00	0.00	0.00	0.00
38.71	0.00	0.00	0.00	0.00	0.00	0.00	0.00	0.00	0.00	0.00	0.00
40.73	0.00	0.00	0.00	0.00	0.00	0.00	0.00	0.00	0.00	0.00	0.00
42.92	0.00	0.00	0.00	0.00	0.00	0.00	0.00	0.00	0.00	0.00	0.00
45.27	0.00	0.00	0.00	0.00	0.00	0.00	0.00	0.00	0.00	0.00	0.00
47.81	0.00	0.00	0.00	0.00	0.00	0.00	0.00	0.00	0.00	0.00	0.00
50.55	0.00	0.00	0.00	0.00	0.00	0.00	0.00	0.00	0.00	0.00	0.00
53.51	0.00	0.00	0.00	0.00	0.00	0.00	0.00	0.00	0.00	0.00	0.00
56.69	0.00	0.00	0.00	0.00	0.00	0.00	0.00	0.00	0.00	0.00	0.00
60.13	0.00	0.00	0.00	0.00	0.00	0.00	0.00	0.00	0.00	0.00	0.00
63.84	0.00	0.00	0.00	0.00	0.00	0.00	0.00	0.00	0.00	0.00	0.00
67.83	0.00	0.00	0.00	0.00	0.00	0.00	0.00	0.00	0.00	0.00	0.00
72.15	0.00	0.00	0.00	0.00	0.00	0.00	0.00	0.00	0.00	0.00	0.00
76.80	0.00	0.00	0.00	0.00	0.00	0.00	0.00	0.00	0.00	0.00	0.00
81.81	0.00	0.00	0.00	0.00	0.00	0.00	0.00	0.00	0.00	0.00	0.00
87.22	0.00	0.00	0.00	0.00	0.00	0.00	0.00	0.00	0.00	0.00	0.00
93.06	0.00	0.00	0.00	0.00	0.00	0.00	0.00	0.00	0.00	0.00	0.00
99.35	0.00	0.00	0.00	0.00	0.00	0.00	0.00	0.00	0.00	0.00	0.00
106.13	0.00	0.00	0.00	0.00	0.00	0.00	0.00	0.00	0.00	0.00	0.00
113.45	0.00	0.00	0.00	0.00	0.00	0.00	0.00	0.00	0.00	0.00	0.00
121.34	0.00	0.00	0.00	0.00	0.00	0.00	0.00	0.00	0.00	0.00	0.00
129.86	0.00	0.00	0.00	0.00	0.00	0.00	0.00	0.00	0.00	0.00	0.00
139.04	0.00	0.00	0.00	0.00	0.00	0.00	0.00	0.00	0.00	0.00	0.00
148.94	0.00	0.00	0.00	0.00	0.00	0.00	0.00	0.00	0.00	0.00	0.00
159.62	0.00	0.00	0.00	0.00	0.00	0.00	0.00	0.00	0.00	0.00	0.00
171.14	0.00	0.00	0.00	0.00	0.00	0.00	0.00	0.00	0.00	0.00	0.00
183.56	0.00	0.00	0.00	0.00	0.00	0.00	0.00	0.00	0.00	0.00	0.00
196.96	0.00	0.00	0.00	0.00	0.00	0.00	0.00	0.00	0.00	0.00	0.00
211.41	0.00	0.00	0.00	0.00	0.00	0.00	0.00	0.00	0.00	0.00	0.00
226.99	0.00	0.00	0.00	0.00	0.00	0.00	0.00	0.00	0.00	0.00	0.00

243.80	0.00	0.00	0.00	0.00	0.00	0.00	0.00	0.00	0.00	0.00	0.00
261.93	0.00	0.00	0.00	0.00	0.00	0.00	0.00	0.00	0.00	0.00	0.00
281.48	0.00	0.00	0.00	0.00	0.00	0.00	0.00	0.00	0.00	0.00	0.00
302.56	0.00	0.00	0.00	0.00	0.00	0.00	0.00	0.00	0.00	0.00	0.00
325.30	0.00	0.00	0.00	0.00	0.00	0.00	0.00	0.00	0.00	0.00	0.00
349.83	0.00	0.00	0.00	0.00	0.00	0.00	0.00	0.00	0.00	0.00	0.00
376.28	0.00	0.00	0.00	0.00	0.00	0.00	0.00	0.00	0.00	0.00	0.00
404.81	0.00	0.00	0.00	0.00	0.00	0.00	0.00	0.00	0.00	0.00	0.00
435.58	0.00	0.00	0.00	0.00	0.00	0.00	0.00	0.00	0.00	0.00	0.00
468.76	0.00	0.00	0.00	0.00	0.00	0.00	0.00	0.00	0.00	0.00	0.00
504.55	0.00	0.00	0.00	0.00	0.00	0.00	0.00	0.00	0.00	0.00	0.00
543.15	0.00	0.00	0.00	0.00	0.00	0.00	0.00	0.00	0.00	0.00	0.00
584.78	0.00	0.00	0.00	0.00	0.00	0.00	0.00	0.00	0.00	0.00	0.00
629.68	0.00	0.00	0.00	0.00	0.00	0.00	0.00	0.00	0.00	0.00	0.00
678.10	0.00	0.00	0.00	0.00	0.00	0.00	0.00	0.00	0.00	0.00	0.00
730.33	0.00	0.00	0.00	0.00	0.00	0.00	0.00	0.00	0.00	0.00	0.00
786.65	0.00	0.00	0.00	0.00	0.00	0.00	0.00	0.00	0.00	0.00	0.00
847.40	0.00	0.00	0.00	0.00	0.00	0.00	0.00	0.00	0.00	0.00	0.00
912.92	0.00	0.00	0.00	0.00	0.00	0.00	0.00	0.00	0.00	0.00	0.00
983.58	0.00	0.00	0.00	0.00	0.00	0.00	0.00	0.00	0.00	0.00	0.00
1059.78	0.00	0.00	0.00	0.00	0.00	0.00	0.00	0.00	0.00	0.00	0.00
1141.98	0.00	0.00	0.00	0.00	0.00	0.00	0.00	0.00	0.00	0.00	0.00
1230.62	0.00	0.00	0.00	0.00	0.00	0.00	0.00	0.00	0.00	0.00	0.00
1326.23	0.00	0.00	0.00	0.00	0.00	0.00	0.00	0.00	0.00	0.00	0.00
1429.34	0.00	0.00	0.00	0.00	0.00	0.00	0.00	0.00	0.00	0.00	0.00
1540.54	0.00	0.00	0.00	0.00	0.00	0.00	0.00	0.00	0.00	0.00	0.00
1660.48	0.00	0.00	0.00	0.00	0.00	0.00	0.00	0.00	0.00	0.00	0.00
1789.83	0.00	0.00	0.00	0.00	0.00	0.00	0.00	0.00	0.00	0.00	0.00
1929.34	0.00	0.00	0.00	0.00	0.00	0.00	0.00	0.00	0.00	0.00	0.00
2079.80	0.00	0.00	0.00	0.00	0.00	0.00	0.00	0.00	0.00	0.00	0.00
2242.08	0.00	0.00	0.00	0.00	0.00	0.00	0.00	0.00	0.00	0.00	0.00
2417.10	0.00	0.00	0.00	0.00	0.00	0.00	0.00	0.00	0.00	0.00	0.00
2605.85	0.00	0.00	0.00	0.00	0.00	0.00	0.00	0.00	0.00	0.00	0.00
2809.43	0.00	0.00	0.00	0.00	0.00	0.00	0.00	0.00	0.00	0.00	0.00
3028.99	0.00	0.00	0.00	0.00	0.00	0.00	0.00	0.00	0.00	0.00	0.00
3265.79	0.00	0.00	0.00	0.00	0.00	0.00	0.00	0.00	0.00	0.00	0.00
3521.18	0.00	0.00	0.00	0.00	0.00	0.00	0.00	0.00	0.00	0.00	0.00
3796.62	0.00	0.00	0.00	0.00	0.00	0.00	0.00	0.00	0.00	0.00	0.00

Wavelength (nm) / Time (ps)	367.19	368.59	370.00	371.41	372.82	374.23	375.64	377.05	378.45	379.86	381.27
-3.28	0.14	0.00	-0.02	0.24	0.14	0.11	0.15	0.08	0.13	-0.06	-0.20
-2.78	-0.05	0.07	0.16	-0.05	-0.04	0.10	0.09	0.04	-0.08	-0.08	-0.01
-2.28	0.03	0.05	-0.05	-0.07	-0.09	-0.25	0.01	-0.04	-0.07	-0.07	0.05
-1.78	-0.04	-0.01	0.10	0.08	0.01	0.15	0.06	0.09	0.24	0.29	0.14
-1.28	0.14	-0.04	-0.09	0.02	-0.03	-0.21	-0.23	-0.02	-0.12	0.05	0.07
-0.78	-0.15	-0.04	-0.02	-0.06	0.10	0.03	-0.09	-0.09	-0.08	-0.14	-0.07
-0.28	0.02	0.08	-0.04	-0.07	-0.06	0.00	-0.07	-0.02	-0.07	0.03	-0.02
0.22	0.02	0.13	-0.06	-0.06	0.07	0.05	0.02	-0.06	0.00	0.09	0.03

0.32	-0.12	-0.10	0.07	-0.05	0.06	0.04	0.04	-0.04	0.06	-0.08	0.01
0.42	0.01	-0.15	-0.04	0.02	-0.16	-0.01	0.03	0.07	-0.02	-0.02	-0.01
0.52	0.02	0.01	0.12	0.09	0.06	0.04	0.12	0.06	0.13	-0.03	0.12
0.62	0.34	0.11	0.08	0.13	0.07	0.08	0.01	0.01	0.15	0.16	0.26
0.72	-0.01	-0.11	0.03	-0.06	-0.08	0.01	-0.08	-0.13	-0.01	-0.03	-0.08
0.77	-0.20	-0.19	-0.07	-0.14	0.07	-0.15	-0.05	-0.03	-0.03	0.03	-0.06
0.82	1.22	0.92	0.70	0.39	0.12	-0.25	-0.17	-0.09	-0.18	-0.17	-0.08
0.87	3.51	3.74	3.40	3.07	2.47	2.06	1.37	0.99	0.61	0.33	0.19
0.92	3.05	3.25	3.46	3.45	3.61	3.20	3.22	3.22	3.00	2.58	2.40
0.97	2.84	3.26	3.60	3.99	4.13	4.08	3.97	3.78	3.66	3.64	3.67
1.02	2.19	2.40	2.79	2.81	2.93	3.22	3.47	3.53	4.18	4.53	5.09
1.07	2.06	2.09	2.50	2.33	2.69	2.89	2.95	3.26	3.37	3.63	4.02
1.12	2.35	2.17	2.61	2.68	2.89	2.98	3.23	3.25	3.61	3.77	3.93
1.17	2.09	2.20	2.26	2.34	2.50	2.83	2.70	2.91	3.12	3.17	3.22
1.22	2.04	2.26	2.37	2.57	2.50	2.72	2.80	3.09	3.16	3.19	3.46
1.27	1.95	2.20	2.18	2.36	2.38	2.56	2.62	2.82	2.92	3.12	3.28
1.32	2.17	2.07	2.29	2.31	2.60	2.51	2.62	2.77	2.99	2.98	3.26
1.37	1.78	1.90	2.17	2.23	2.41	2.30	2.46	2.72	2.81	3.00	2.99
1.42	1.71	1.99	2.20	2.33	2.43	2.65	2.44	2.80	2.73	2.73	3.01
1.47	1.92	2.00	2.15	2.06	2.19	2.40	2.36	2.48	2.60	2.82	3.01
1.52	1.63	1.79	2.09	1.94	2.05	2.17	2.28	2.25	2.60	2.62	2.77
1.57	1.67	1.77	1.81	2.11	2.15	2.27	2.27	2.39	2.48	2.68	2.66
1.62	1.73	1.77	1.89	2.00	2.15	2.06	2.27	2.27	2.37	2.59	2.66
1.67	1.75	1.77	2.13	2.00	2.25	2.21	2.34	2.34	2.66	2.78	2.88
1.72	1.47	1.65	1.72	1.87	1.78	2.03	2.14	2.17	2.13	2.44	2.66
1.77	1.58	1.88	1.87	2.03	1.94	2.01	2.28	2.34	2.42	2.35	2.58
1.82	1.61	1.55	1.89	1.73	2.03	1.95	2.32	2.37	2.44	2.25	2.57
1.87	1.43	1.45	1.72	1.83	1.87	1.94	2.00	2.15	2.33	2.19	2.43
1.92	1.75	1.82	1.81	1.95	1.94	2.09	2.29	2.35	2.27	2.55	2.65
1.97	1.62	1.61	1.73	1.76	1.97	2.13	2.16	2.28	2.31	2.41	2.56
2.02	1.56	1.65	1.71	1.82	2.11	2.14	2.08	2.25	2.19	2.41	2.54
2.07	1.45	1.51	1.84	1.60	1.86	1.91	1.94	2.11	2.24	2.27	2.53
2.12	1.65	1.81	2.09	1.86	1.88	2.10	2.18	2.34	2.56	2.58	2.63
2.17	1.40	1.59	1.95	1.90	1.90	1.99	2.08	2.29	2.33	2.28	2.50
2.22	1.48	1.57	1.87	1.53	1.85	2.06	2.05	1.98	2.13	2.17	2.40
2.27	1.24	1.29	1.48	1.69	1.77	1.76	2.00	2.05	2.20	2.21	2.35
2.32	1.28	1.51	1.45	1.59	1.65	1.68	1.82	2.06	2.06	2.10	2.23
2.37	1.53	1.70	1.74	1.85	2.00	1.96	2.03	2.27	2.30	2.25	2.40
2.42	1.45	1.61	1.65	1.93	1.85	1.89	1.98	2.24	2.23	2.27	2.41
2.47	1.49	1.49	1.85	1.80	1.85	1.96	1.81	2.06	2.23	2.09	2.46
2.52	1.60	1.43	1.77	1.80	1.81	1.95	2.04	2.12	2.14	2.11	2.51
2.57	1.47	1.61	1.75	1.86	1.97	1.72	1.92	2.01	2.06	2.27	2.38
2.62	1.35	1.37	1.50	1.62	1.76	1.78	1.75	1.87	1.92	2.14	2.21
2.67	1.48	1.70	1.73	1.85	1.95	1.79	1.89	2.02	2.14	2.22	2.31
2.72	1.60	1.64	1.69	1.71	1.90	2.07	1.98	2.12	2.10	2.21	2.40
2.77	1.40	1.65	1.81	1.77	1.88	1.97	2.11	2.36	2.22	2.27	2.57
2.82	1.34	1.46	1.71	1.73	1.69	1.80	1.82	2.03	1.99	2.17	2.37
2.87	1.79	1.72	1.69	1.73	1.86	1.74	1.89	1.88	2.12	2.24	2.18
2.92	1.52	1.69	1.71	1.72	1.76	1.81	1.95	2.11	2.06	2.08	2.16
2.97	1.58	1.34	1.55	1.54	1.92	1.73	1.86	1.96	2.07	2.12	2.26

3.02	1.59	1.65	1.88	1.89	1.97	1.84	2.02	2.19	2.22	2.31	2.55
3.07	1.50	1.69	1.79	1.82	1.82	1.94	2.06	2.13	2.16	2.15	2.31
3.12	1.75	1.68	1.81	1.87	1.87	1.78	2.12	2.00	2.16	2.23	2.36
3.17	1.25	1.42	1.51	1.76	1.64	1.87	1.87	1.96	1.95	2.20	2.28
3.22	1.45	1.48	1.72	1.60	1.66	1.88	1.84	1.84	2.09	2.00	2.27
3.27	1.48	1.55	1.63	1.71	1.84	1.83	1.94	1.97	2.10	2.25	2.40
3.32	1.59	1.69	1.91	2.02	1.89	2.01	2.01	2.12	2.34	2.46	2.57
3.37	1.39	1.66	1.81	1.87	1.86	1.94	1.86	2.04	2.24	2.13	2.11
3.42	1.59	1.73	1.79	1.77	1.80	2.08	2.01	1.96	2.20	2.46	2.40
3.47	1.42	1.55	1.76	1.70	1.79	1.77	1.87	1.96	1.99	2.27	2.33
3.52	1.42	1.66	1.76	1.81	1.77	1.81	1.98	1.93	2.20	2.08	2.27
3.57	1.43	1.52	1.78	1.79	1.84	1.84	1.91	1.80	2.20	2.10	2.08
3.62	1.50	1.58	1.64	1.51	1.74	1.88	1.86	1.89	1.91	2.18	2.23
3.67	1.60	1.75	1.81	1.71	1.98	1.87	2.18	2.01	2.10	2.21	2.59
3.72	1.44	1.61	1.59	1.59	1.70	1.77	1.83	1.82	2.01	2.04	2.11
3.92	1.35	1.55	1.51	1.62	2.00	1.86	1.64	2.08	2.24	2.29	2.28
4.12	1.45	1.38	1.58	1.77	1.57	1.77	1.87	1.96	2.00	2.02	2.27
4.32	1.46	1.54	1.54	1.73	1.73	1.87	1.79	2.12	2.03	2.06	2.18
4.52	1.36	1.51	1.68	1.70	1.66	1.83	1.99	2.01	2.06	2.19	2.29
4.72	1.54	1.69	1.74	1.69	1.90	1.76	1.93	1.90	2.06	2.02	2.36
4.92	1.45	1.41	1.68	1.51	1.78	1.67	1.67	1.96	1.95	2.20	2.28
5.12	1.63	1.57	1.66	1.73	1.70	2.01	1.83	1.75	2.10	2.07	2.32
5.32	1.35	1.55	1.64	1.70	1.75	1.97	1.85	2.06	2.12	2.12	2.21
5.52	1.45	1.68	1.61	1.68	1.99	1.77	1.96	1.80	2.13	2.25	2.41
5.72	1.49	1.62	1.82	1.63	1.89	1.95	1.99	1.97	2.08	2.14	2.24
5.92	1.51	1.54	1.58	1.70	1.65	1.81	1.93	1.83	2.00	2.22	2.17
6.12	1.40	1.54	1.51	1.60	1.65	1.61	1.87	2.00	2.00	2.04	2.07
6.32	1.56	1.34	1.65	1.58	1.68	1.72	1.74	1.87	2.07	1.82	2.13
6.52	1.27	1.67	1.67	1.71	1.66	1.87	2.09	2.03	2.15	2.05	2.17
6.72	1.44	1.56	1.72	1.55	1.80	1.94	1.99	1.92	2.18	2.21	2.30
6.92	1.65	1.35	1.78	1.66	1.60	1.74	1.94	1.92	1.86	2.16	2.18
7.12	1.41	1.31	1.37	1.49	1.66	1.74	1.84	1.94	2.06	2.10	2.31
7.32	1.41	1.61	1.66	1.65	1.71	1.70	1.96	1.82	1.98	1.93	2.18
7.52	1.30	1.64	1.60	1.68	1.72	1.64	1.80	1.88	1.99	2.14	2.30
7.72	1.28	1.44	1.41	1.63	1.73	1.75	1.79	1.87	2.01	2.12	2.37
7.92	1.35	1.59	1.75	1.56	1.75	1.85	1.76	1.89	1.85	2.00	2.13
8.12	1.39	1.31	1.43	1.77	1.91	1.68	1.70	1.89	1.82	2.01	2.20
8.32	1.47	1.32	1.38	1.55	1.74	1.75	1.79	1.78	2.05	2.03	2.00
8.52	1.26	1.52	1.31	1.51	1.47	1.54	1.59	1.67	1.63	1.78	1.93
8.72	1.37	1.35	1.45	1.48	1.68	1.79	1.68	1.88	1.90	1.98	2.04
8.92	1.18	1.57	1.56	1.69	1.51	1.81	1.67	1.80	1.92	1.97	1.98
9.12	1.46	1.49	1.52	1.65	1.60	1.81	1.84	1.94	2.02	1.99	2.02
9.32	1.46	1.37	1.55	1.73	1.85	1.74	1.87	2.02	1.82	2.01	2.14
9.52	1.31	1.34	1.42	1.55	1.51	1.79	1.63	1.83	1.93	1.86	2.08
9.72	1.34	1.29	1.70	1.58	1.53	1.69	1.69	1.83	1.83	1.93	2.08
9.92	1.36	1.52	1.77	1.71	1.89	1.90	1.90	2.01	1.95	2.11	2.28
10.12	1.48	1.65	1.88	1.64	1.83	2.04	2.11	2.20	2.10	2.24	2.25
10.32	1.21	1.54	1.61	1.74	1.72	1.75	1.75	2.02	2.14	1.94	2.13
10.52	1.45	1.35	1.49	1.64	1.67	1.68	1.89	1.86	2.09	2.00	2.30
10.72	1.60	1.60	1.47	1.70	1.78	1.66	1.63	1.96	1.93	2.08	2.07

10.92	1.49	1.51	1.57	1.46	1.51	1.63	1.64	1.64	1.83	2.03	1.86
11.12	1.16	1.34	1.51	1.50	1.54	1.69	1.57	1.72	1.76	1.93	2.13
11.32	1.53	1.56	1.75	1.70	1.77	1.63	1.85	1.92	2.28	2.20	2.23
11.52	1.27	1.31	1.51	1.49	1.38	1.55	1.55	1.78	1.91	2.03	1.97
11.72	1.41	1.55	1.50	1.74	1.77	1.63	1.75	1.96	1.99	2.03	2.07
11.92	1.44	1.51	1.61	1.76	1.70	1.65	1.74	1.84	1.90	1.83	2.06
12.12	1.39	1.44	1.55	1.49	1.44	1.72	1.80	1.85	1.62	1.96	2.07
12.32	1.08	1.42	1.47	1.51	1.63	1.70	1.77	1.91	2.04	2.24	2.34
12.52	1.19	1.36	1.73	1.61	1.68	1.64	1.64	1.81	1.78	1.92	2.06
12.72	1.27	1.30	1.48	1.42	1.45	1.56	1.53	1.62	1.90	1.79	2.01
12.92	1.27	1.39	1.58	1.64	1.64	1.71	1.88	1.72	1.88	2.02	2.02
13.12	1.18	1.54	1.46	1.46	1.61	1.50	1.52	1.76	1.74	1.72	1.83
13.32	1.20	1.44	1.60	1.53	1.54	1.81	1.86	2.06	2.04	1.98	2.11
13.52	0.73	1.17	1.20	1.35	1.27	1.51	1.66	1.47	1.64	1.79	1.84
13.72	1.43	1.45	1.76	1.69	1.82	1.87	1.78	1.96	1.96	1.92	2.09
13.92	1.31	1.26	1.34	1.41	1.49	1.53	1.73	1.57	1.93	2.14	2.22
14.00	1.21	1.29	1.42	1.50	1.54	1.65	1.74	1.87	1.99	1.87	2.10
14.08	1.00	1.26	1.29	1.18	1.44	1.49	1.54	1.53	1.67	1.60	1.75
14.17	1.11	1.07	1.13	1.49	1.51	1.52	1.46	1.59	1.70	1.82	1.78
14.27	1.24	1.37	1.43	1.50	1.47	1.69	1.63	1.68	1.77	1.94	2.07
14.38	1.29	1.35	1.54	1.61	1.67	1.62	1.67	1.80	1.85	1.83	2.02
14.49	1.37	1.39	1.63	1.61	1.56	1.69	1.88	1.86	1.96	2.05	2.03
14.61	1.08	1.42	1.45	1.54	1.56	1.46	1.62	1.65	1.76	1.87	1.95
14.75	1.12	1.18	1.49	1.50	1.48	1.43	1.57	1.63	1.69	1.72	1.92
14.89	1.11	1.36	1.40	1.45	1.56	1.54	1.72	1.78	1.81	2.10	2.26
15.05	1.37	1.43	1.62	1.53	1.48	1.59	1.79	1.78	1.88	2.10	2.09
15.21	1.23	1.47	1.59	1.58	1.73	1.72	1.75	1.78	1.90	2.06	2.08
15.39	1.11	1.24	1.35	1.49	1.54	1.49	1.65	1.66	1.84	1.91	2.12
15.59	1.33	1.45	1.54	1.32	1.48	1.43	1.47	1.50	1.63	1.79	1.92
15.80	1.12	1.28	1.47	1.42	1.58	1.55	1.58	1.85	1.78	1.81	2.09
16.02	0.89	1.07	1.30	1.27	1.38	1.55	1.36	1.49	1.71	1.87	1.84
16.27	1.32	1.19	1.50	1.55	1.50	1.62	1.63	1.68	1.96	1.84	1.87
16.53	1.35	1.36	1.50	1.57	1.47	1.58	1.70	1.81	1.78	1.81	1.92
16.81	1.09	1.09	1.26	1.23	1.39	1.32	1.44	1.75	1.83	1.68	1.97
17.12	0.97	1.16	1.08	1.31	1.52	1.45	1.47	1.71	1.69	1.67	1.87
17.45	1.04	0.99	1.19	1.27	1.33	1.40	1.34	1.40	1.55	1.54	1.71
17.81	1.20	1.20	1.21	1.41	1.64	1.45	1.65	1.70	1.75	1.76	1.92
18.19	1.40	1.38	1.44	1.39	1.54	1.57	1.62	1.62	1.95	1.86	2.12
18.60	1.24	1.28	1.55	1.47	1.57	1.64	1.67	1.97	1.99	1.81	2.05
19.05	1.04	1.23	1.31	1.34	1.41	1.40	1.55	1.61	1.72	1.65	1.98
19.53	1.20	1.18	1.43	1.50	1.46	1.71	1.54	1.78	1.62	1.81	1.98
20.05	1.50	1.36	1.49	1.64	1.59	1.65	1.62	1.93	1.96	1.92	2.09
20.61	1.22	1.44	1.73	1.60	1.61	1.69	1.76	1.96	1.89	2.02	2.10
21.22	1.21	1.37	1.51	1.49	1.42	1.46	1.75	1.68	1.90	1.78	2.02
21.87	1.11	1.25	1.35	1.51	1.53	1.42	1.56	1.76	1.76	1.92	2.08
22.57	1.28	1.27	1.60	1.52	1.32	1.51	1.66	1.69	1.84	1.89	2.06
23.33	1.21	1.25	1.35	1.40	1.25	1.64	1.57	1.54	1.81	1.76	2.08
24.15	1.13	1.28	1.38	1.32	1.41	1.64	1.57	1.51	1.63	1.78	2.08
25.03	1.11	1.34	1.34	1.46	1.37	1.37	1.62	1.63	1.63	1.71	2.00
25.98	1.56	1.32	1.41	1.40	1.53	1.61	1.48	1.78	1.78	1.93	1.93

27.01	1.15	1.27	1.24	1.29	1.36	1.49	1.48	1.64	1.63	1.63	1.95
28.11	1.30	1.39	1.34	1.50	1.31	1.48	1.60	1.65	1.89	1.90	2.02
29.30	1.22	1.22	1.30	1.19	1.44	1.47	1.47	1.50	1.60	1.78	1.83
30.59	1.14	1.20	1.39	1.45	1.52	1.56	1.71	1.77	1.83	1.84	2.12
31.98	1.42	1.38	1.49	1.26	1.48	1.43	1.60	1.56	1.64	1.81	2.04
33.47	1.04	1.42	1.30	1.52	1.53	1.50	1.52	1.75	1.70	1.82	1.94
35.09	1.19	1.37	1.36	1.64	1.52	1.61	1.63	1.76	1.86	1.76	2.07
36.83	1.23	1.16	1.38	1.39	1.48	1.54	1.49	1.65	1.84	1.70	1.84
38.71	1.26	1.41	1.38	1.52	1.45	1.47	1.44	1.54	1.72	1.73	1.84
40.73	1.04	1.27	1.48	1.46	1.48	1.43	1.54	1.52	1.74	1.74	1.85
42.92	1.33	1.28	1.50	1.36	1.43	1.47	1.59	1.77	1.76	1.82	2.14
45.27	1.08	1.23	1.31	1.17	1.43	1.41	1.51	1.64	1.78	1.84	1.97
47.81	1.24	1.13	1.21	1.47	1.41	1.48	1.62	1.64	1.66	1.92	1.90
50.55	1.18	1.15	1.23	1.43	1.25	1.53	1.47	1.73	1.78	1.68	1.87
53.51	1.22	1.25	1.28	1.32	1.53	1.50	1.55	1.59	1.78	1.78	1.85
56.69	1.16	1.31	1.21	1.54	1.50	1.59	1.53	1.66	1.80	1.92	1.98
60.13	1.09	1.27	1.33	1.28	1.38	1.54	1.62	1.65	1.72	1.80	1.87
63.84	1.28	1.46	1.44	1.56	1.29	1.61	1.60	1.78	1.66	1.83	1.97
67.83	1.24	1.23	1.25	1.40	1.43	1.44	1.52	1.63	1.62	1.90	1.95
72.15	1.04	1.05	1.29	1.27	1.24	1.35	1.34	1.42	1.52	1.67	1.84
76.80	0.97	1.23	1.13	1.23	1.35	1.48	1.36	1.54	1.49	1.57	1.68
81.81	1.12	1.29	1.23	1.16	1.29	1.38	1.59	1.63	1.67	1.72	1.74
87.22	0.97	0.97	1.06	1.34	1.33	1.29	1.32	1.38	1.57	1.65	1.62
93.06	1.12	1.25	1.30	1.23	1.15	1.28	1.42	1.52	1.63	1.71	1.78
99.35	1.09	1.19	1.31	1.31	1.31	1.40	1.39	1.53	1.66	1.64	1.72
106.13	1.12	1.26	1.34	1.34	1.38	1.39	1.43	1.47	1.61	1.53	1.87
113.45	0.97	1.31	1.24	1.27	1.45	1.50	1.54	1.66	1.60	1.72	1.86
121.34	1.11	1.16	1.40	1.38	1.39	1.18	1.43	1.54	1.72	1.87	1.82
129.86	0.98	1.42	1.24	1.27	1.52	1.36	1.45	1.71	1.60	1.47	1.82
139.04	1.38	1.46	1.54	1.34	1.44	1.43	1.42	1.55	1.63	1.77	1.86
148.94	1.05	0.89	1.19	1.14	1.30	1.31	1.31	1.39	1.46	1.67	1.53
159.62	1.25	1.27	1.28	1.25	1.25	1.25	1.47	1.39	1.64	1.64	1.60
171.14	1.10	1.20	1.33	1.37	1.33	1.47	1.27	1.64	1.54	1.57	1.72
183.56	1.33	1.17	1.35	1.50	1.57	1.61	1.61	1.60	1.60	1.59	1.65
196.96	1.06	1.28	1.36	1.40	1.40	1.59	1.42	1.56	1.62	1.50	1.72
211.41	1.10	1.13	1.32	1.17	1.16	1.32	1.27	1.38	1.45	1.41	1.65
226.99	1.10	1.00	1.10	1.06	1.02	1.14	1.29	1.28	1.50	1.49	1.69
243.80	1.03	1.09	1.14	1.03	1.31	1.42	1.44	1.30	1.58	1.45	1.57
261.93	1.18	1.38	1.40	1.30	1.39	1.52	1.45	1.66	1.72	1.63	1.84
281.48	1.14	1.43	1.35	1.34	1.39	1.36	1.44	1.58	1.67	1.67	1.76
302.56	1.09	1.07	1.28	1.10	1.45	1.47	1.35	1.53	1.51	1.70	1.67
325.30	0.97	1.08	1.19	1.08	1.15	1.17	1.30	1.40	1.20	1.53	1.54
349.83	0.88	0.84	1.09	1.14	1.13	1.06	1.32	1.25	1.20	1.40	1.40
376.28	1.09	1.05	1.09	1.28	1.29	1.35	1.34	1.30	1.45	1.40	1.82
404.81	1.23	1.23	1.38	1.33	1.38	1.38	1.37	1.50	1.67	1.62	1.86
435.58	1.14	1.11	1.18	1.30	1.25	1.32	1.26	1.29	1.47	1.49	1.70
468.76	1.01	0.94	1.08	1.06	1.02	1.22	1.04	1.20	1.34	1.42	1.56
504.55	0.96	1.02	1.14	1.16	1.02	0.85	1.09	1.24	1.29	1.23	1.44
543.15	0.72	0.94	0.98	1.22	1.18	1.25	1.18	1.27	1.42	1.37	1.41
584.78	0.91	0.91	1.03	0.91	1.00	1.06	0.95	1.16	1.19	1.22	1.15

629.68	0.93	1.01	1.20	1.07	1.18	1.18	1.15	1.29	1.34	1.31	1.36
678.10	1.07	1.07	1.13	1.22	1.28	1.26	1.51	1.36	1.55	1.38	1.60
730.33	1.10	1.11	1.03	1.21	1.30	1.20	1.18	1.36	1.29	1.42	1.46
786.65	0.66	0.93	0.98	0.87	1.03	1.05	1.03	1.28	1.14	1.11	1.30
847.40	0.87	0.79	0.90	0.80	0.99	1.04	1.09	0.89	1.09	1.23	1.36
912.92	0.83	1.11	1.04	1.00	1.16	1.12	1.24	1.33	1.29	1.35	1.41
983.58	0.85	0.93	1.09	1.04	0.92	1.00	1.01	1.11	1.15	1.14	1.30
1059.78	0.86	1.03	1.17	0.93	0.92	1.15	1.12	1.15	1.20	1.05	1.21
1141.98	0.85	0.86	0.85	0.92	0.74	1.05	0.80	0.98	0.96	1.08	1.21
1230.62	0.71	0.87	0.90	0.92	1.05	1.07	1.04	1.15	1.08	1.15	1.16
1326.23	1.13	0.96	0.97	1.19	1.08	1.05	1.01	1.13	1.17	1.33	1.24
1429.34	0.47	0.90	0.82	0.75	0.73	0.92	0.84	0.87	1.02	1.10	1.07
1540.54	0.94	0.82	0.88	0.92	0.92	0.76	0.78	1.03	1.07	0.87	1.04
1660.48	0.90	0.90	1.00	1.01	0.81	1.00	0.89	0.89	1.02	1.00	0.99
1789.83	0.77	0.92	0.79	0.91	0.91	0.79	0.87	0.89	0.85	1.05	1.04
1929.34	0.70	0.66	0.69	0.73	0.66	0.80	0.81	0.82	1.03	0.91	0.93
2079.80	0.62	0.74	0.49	0.85	0.69	0.66	0.73	0.84	0.89	0.84	0.97
2242.08	0.81	0.62	0.82	0.82	0.98	0.82	0.95	0.78	0.99	1.00	0.92
2417.10	0.51	0.64	0.73	0.41	0.51	0.55	0.54	0.69	0.77	0.61	0.93
2605.85	0.47	0.39	0.57	0.63	0.54	0.53	0.60	0.64	0.81	0.88	0.91
2809.43	0.67	0.72	0.85	0.77	0.63	0.81	0.56	0.66	0.79	0.78	0.83
3028.99	0.68	0.69	0.62	0.67	0.76	0.55	0.86	0.77	0.84	0.71	0.90
3265.79	0.71	0.62	0.68	0.60	0.64	0.61	0.64	0.60	0.70	0.69	0.85
3521.18	0.73	0.72	0.76	0.60	0.71	0.64	0.62	0.73	0.75	0.87	0.87
3796.62	0.52	0.63	0.82	0.69	0.70	0.68	0.66	0.73	0.93	0.61	0.87

Wavelength (nm) / Time (ps)	382.68	384.09	385.50	386.91	388.31	389.72	391.13	392.54	393.95	395.36	396.77
-3.28	-0.08	-0.06	-0.08	-0.05	-0.09	-0.04	0.02	-0.03	-0.01	0.01	-0.04
-2.78	-0.04	0.01	-0.08	-0.10	0.04	-0.09	-0.03	-0.10	-0.08	-0.11	-0.05
-2.28	0.12	-0.02	-0.05	0.00	-0.05	-0.07	0.05	0.08	0.02	-0.05	0.08
-1.78	0.13	0.09	0.18	0.18	0.28	0.21	0.08	0.25	0.27	0.25	0.31
-1.28	-0.09	-0.06	0.08	-0.03	0.06	-0.07	0.02	-0.10	-0.09	-0.03	-0.05
-0.78	-0.12	0.00	-0.09	-0.10	-0.11	-0.07	-0.16	-0.14	-0.05	-0.10	-0.23
-0.28	0.07	-0.06	0.08	-0.01	0.06	-0.04	0.09	-0.01	-0.05	0.12	0.04
0.22	0.07	0.07	-0.15	-0.03	-0.13	0.08	0.01	-0.05	-0.01	-0.15	-0.05
0.32	-0.05	0.05	0.14	0.06	-0.13	0.11	-0.02	0.04	-0.02	0.10	-0.15
0.42	-0.01	-0.02	-0.04	0.09	0.04	-0.03	-0.05	0.05	0.01	-0.04	0.13
0.52	-0.02	-0.05	-0.03	-0.01	-0.09	-0.09	-0.04	0.01	0.14	-0.09	0.11
0.62	-0.03	0.12	0.25	0.01	-0.12	-0.01	-0.02	0.05	0.10	0.07	-0.01
0.72	0.04	-0.04	-0.08	0.04	-0.08	-0.02	-0.17	-0.15	0.03	0.08	0.03
0.77	0.22	-0.07	0.10	-0.02	0.18	0.04	0.15	0.07	0.14	0.13	0.02
0.82	-0.14	-0.03	0.05	0.13	0.01	-0.06	0.00	-0.01	-0.06	-0.11	0.01
0.87	-0.14	-0.10	-0.13	0.00	-0.01	0.04	0.01	0.15	0.26	0.02	0.00
0.92	1.94	1.28	0.84	0.46	-0.09	-0.11	-0.21	-0.18	-0.22	-0.17	-0.10
0.97	3.51	3.62	3.42	3.32	3.22	2.62	2.14	1.51	0.97	0.58	0.21
1.02	5.32	5.52	5.95	6.19	6.10	5.96	5.90	5.59	5.11	4.70	4.18
1.07	4.28	4.49	4.91	5.23	5.45	5.72	5.76	6.00	6.04	5.96	5.99
1.12	4.16	4.28	4.53	4.86	5.23	5.28	5.56	5.98	6.24	6.40	6.70

1.17	3.53	3.63	3.98	4.06	4.21	4.41	4.75	4.89	5.13	5.13	5.47
1.22	3.61	3.82	3.84	4.03	4.22	4.39	4.71	4.70	4.90	5.08	5.43
1.27	3.39	3.71	3.69	4.09	3.92	4.16	4.28	4.53	4.70	4.92	5.07
1.32	3.30	3.36	3.36	3.72	4.01	3.97	4.21	4.39	4.56	4.74	4.86
1.37	3.28	3.16	3.53	3.60	3.67	3.62	3.86	3.98	4.29	4.27	4.42
1.42	3.12	3.23	3.33	3.50	3.66	3.71	3.94	3.97	4.21	4.28	4.61
1.47	3.06	3.16	3.13	3.39	3.42	3.64	3.66	3.94	4.09	4.26	4.45
1.52	3.15	2.91	3.17	3.31	3.28	3.36	3.52	3.91	3.89	4.02	4.22
1.57	2.82	2.79	3.17	3.26	3.36	3.41	3.62	3.71	3.84	4.01	4.02
1.62	2.91	2.77	3.14	3.34	3.39	3.63	3.66	3.77	3.76	4.01	4.07
1.67	2.97	2.97	3.12	3.31	3.37	3.49	3.69	3.68	3.97	3.91	4.39
1.72	2.84	2.89	2.71	3.09	3.08	3.30	3.46	3.63	3.66	3.99	3.84
1.77	2.70	2.71	2.96	3.13	3.10	3.14	3.51	3.37	3.67	3.80	3.91
1.82	2.59	2.72	2.95	3.05	3.08	3.01	3.11	3.44	3.59	3.55	3.75
1.87	2.62	2.60	2.72	2.98	2.94	3.02	3.25	3.33	3.46	3.71	3.65
1.92	2.60	2.80	3.01	3.07	3.21	3.39	3.51	3.44	3.57	3.87	3.75
1.97	2.77	2.70	2.78	3.12	2.85	3.24	3.31	3.56	3.69	3.74	3.76
2.02	2.65	2.59	2.75	2.95	3.01	3.18	3.27	3.31	3.60	3.59	3.82
2.07	2.52	2.57	2.82	2.87	2.80	2.96	3.13	3.30	3.41	3.49	3.58
2.12	2.86	2.86	2.91	3.28	3.16	3.32	3.59	3.60	3.76	3.88	3.79
2.17	2.68	2.66	2.75	3.05	2.96	3.09	3.19	3.30	3.55	3.57	3.76
2.22	2.63	2.53	2.59	2.91	3.03	3.04	3.00	3.14	3.39	3.59	3.70
2.27	2.45	2.50	2.52	2.73	2.76	3.02	3.06	3.32	3.53	3.61	3.74
2.32	2.33	2.44	2.52	2.75	2.80	2.89	3.03	3.26	3.53	3.42	3.39
2.37	2.60	2.62	2.77	2.99	2.95	3.03	3.24	3.36	3.40	3.58	3.74
2.42	2.54	2.70	2.85	2.83	3.07	2.93	3.32	3.40	3.45	3.45	3.69
2.47	2.47	2.47	2.66	2.81	2.68	3.00	3.18	3.20	3.42	3.36	3.57
2.52	2.55	2.75	2.87	2.86	2.86	2.94	3.23	3.32	3.40	3.48	3.68
2.57	2.42	2.45	2.56	2.91	2.81	2.93	2.96	3.08	3.37	3.48	3.49
2.62	2.27	2.27	2.37	2.69	2.63	2.78	2.90	3.15	3.15	3.33	3.47
2.67	2.44	2.39	2.63	2.73	2.80	2.78	3.02	3.06	3.20	3.29	3.47
2.72	2.50	2.47	2.80	2.69	2.91	2.96	2.99	3.13	3.25	3.37	3.45
2.77	2.57	2.64	2.77	3.08	3.15	3.11	3.32	3.38	3.52	3.53	3.68
2.82	2.38	2.40	2.53	2.62	2.79	2.99	2.90	3.16	3.42	3.25	3.55
2.87	2.37	2.39	2.58	2.78	2.80	2.80	3.04	2.96	3.17	3.31	3.44
2.92	2.39	2.43	2.51	2.75	2.84	2.77	2.92	3.06	3.21	3.41	3.43
2.97	2.41	2.43	2.45	2.64	2.80	2.86	2.85	2.95	3.31	3.29	3.35
3.02	2.51	2.65	2.62	2.87	2.91	2.98	3.09	3.24	3.42	3.55	3.59
3.07	2.51	2.54	2.67	2.87	2.97	3.00	3.06	3.21	3.30	3.38	3.53
3.12	2.51	2.51	2.74	2.85	3.09	2.89	3.14	3.35	3.50	3.45	3.48
3.17	2.26	2.21	2.59	2.77	2.82	2.90	2.88	3.12	3.49	3.56	3.51
3.22	2.22	2.31	2.54	2.81	2.54	2.79	3.01	3.18	3.49	3.36	3.42
3.27	2.35	2.52	2.77	2.76	3.01	2.74	3.14	3.17	3.43	3.42	3.52
3.32	2.52	2.55	2.63	2.83	2.96	2.97	3.20	3.09	3.39	3.46	3.49
3.37	2.38	2.60	2.26	2.66	2.78	2.73	2.90	3.14	3.19	3.24	3.37
3.42	2.42	2.44	2.73	2.94	3.05	3.11	3.13	3.40	3.51	3.54	3.60
3.47	2.44	2.22	2.49	2.66	2.85	2.78	2.91	3.17	3.33	3.36	3.40
3.52	2.30	2.40	2.56	2.81	2.76	2.97	2.96	3.08	3.23	3.41	3.44
3.57	2.33	2.33	2.51	2.75	2.76	2.86	2.95	3.12	3.18	3.32	3.38
3.62	2.26	2.30	2.40	2.58	2.76	2.92	3.06	3.00	3.31	3.38	3.35

3.67	2.47	2.39	2.56	2.79	2.72	2.88	2.99	3.05	3.13	3.27	3.33
3.72	2.34	2.32	2.45	2.69	2.80	2.68	2.98	2.97	3.13	3.35	3.46
3.92	2.37	2.30	2.54	2.50	2.65	2.83	2.84	2.99	3.22	3.36	3.42
4.12	2.28	2.21	2.37	2.55	2.78	2.81	2.96	2.91	3.13	3.33	3.40
4.32	2.28	2.30	2.55	2.52	2.70	2.74	2.78	2.79	3.09	3.03	3.35
4.52	2.35	2.27	2.56	2.69	2.91	2.80	2.91	2.91	3.15	3.24	3.39
4.72	2.30	2.48	2.44	2.74	2.68	2.61	2.86	3.07	3.08	3.21	3.36
4.92	2.41	2.43	2.47	2.53	2.65	2.81	2.95	3.00	3.20	3.27	3.45
5.12	2.28	2.36	2.41	2.65	2.70	2.80	2.73	2.93	3.22	3.19	3.27
5.32	2.43	2.61	2.55	2.80	2.87	2.99	3.07	3.28	3.48	3.40	3.53
5.52	2.49	2.41	2.54	2.93	2.80	2.85	3.04	3.25	3.17	3.39	3.31
5.72	2.41	2.28	2.60	2.76	2.93	2.83	2.90	3.18	3.27	3.30	3.48
5.92	2.34	2.52	2.63	2.55	2.68	2.88	2.99	3.15	3.12	3.28	3.30
6.12	2.33	2.23	2.31	2.59	2.60	2.77	2.73	3.05	3.30	3.36	3.29
6.32	2.29	2.21	2.31	2.60	2.40	2.64	2.75	3.06	3.10	3.18	3.35
6.52	2.39	2.47	2.50	2.70	2.72	2.78	2.97	3.17	3.17	3.23	3.49
6.72	2.52	2.59	2.62	2.67	2.76	2.94	3.01	2.99	3.28	3.37	3.52
6.92	2.20	2.31	2.39	2.57	2.68	2.77	2.94	2.87	3.05	3.26	3.27
7.12	2.28	2.42	2.35	2.70	2.85	2.66	2.93	3.02	3.07	3.11	3.30
7.32	2.30	2.28	2.39	2.59	2.64	2.71	2.81	3.11	3.29	3.21	3.35
7.52	2.18	2.17	2.43	2.49	2.59	2.76	2.92	2.91	3.16	3.29	3.47
7.72	2.29	2.37	2.51	2.64	2.67	2.84	2.91	2.99	3.04	3.20	3.40
7.92	2.13	2.33	2.34	2.56	2.70	2.69	2.85	2.92	2.98	3.39	3.25
8.12	2.37	2.32	2.47	2.53	2.74	2.79	2.80	2.98	3.20	3.35	3.44
8.32	2.10	2.31	2.43	2.78	2.55	2.78	2.90	3.00	3.08	3.17	3.24
8.52	2.10	1.87	2.16	2.42	2.34	2.51	2.39	2.76	2.79	2.99	2.97
8.72	1.99	2.12	2.33	2.53	2.44	2.59	2.60	2.91	2.91	3.07	3.32
8.92	2.20	2.42	2.55	2.58	2.65	2.68	2.96	2.87	2.94	3.17	3.16
9.12	2.13	2.32	2.44	2.76	2.64	2.63	2.74	2.88	3.23	3.34	3.50
9.32	2.17	2.17	2.51	2.52	2.57	2.66	2.68	2.78	2.99	3.18	3.08
9.52	2.19	2.27	2.38	2.53	2.65	2.63	2.88	2.96	3.15	3.05	3.36
9.72	2.29	2.08	2.39	2.38	2.54	2.52	2.78	2.81	3.08	3.07	3.29
9.92	2.44	2.34	2.49	2.50	2.78	2.76	2.86	3.07	3.25	3.32	3.19
10.12	2.50	2.45	2.38	2.80	2.93	2.89	2.98	3.11	3.35	3.34	3.47
10.32	1.93	2.18	2.34	2.58	2.61	2.58	2.74	2.84	3.01	2.97	3.21
10.52	2.16	2.25	2.41	2.41	2.72	2.80	2.73	2.90	3.08	3.27	3.37
10.72	2.14	2.21	2.38	2.73	2.55	2.74	2.74	3.00	3.14	3.01	3.21
10.92	2.12	1.97	2.20	2.50	2.47	2.54	2.67	2.92	2.84	3.00	3.04
11.12	2.32	2.24	2.30	2.30	2.54	2.63	2.56	2.88	3.05	3.20	3.21
11.32	2.56	2.46	2.49	2.75	2.61	2.82	2.83	3.05	3.12	3.34	3.40
11.52	2.07	2.13	2.18	2.41	2.42	2.61	2.72	2.81	2.84	2.92	3.05
11.72	2.38	2.32	2.46	2.50	2.53	2.73	2.89	3.06	3.13	3.18	3.17
11.92	2.17	2.15	2.31	2.46	2.55	2.61	2.83	2.93	2.91	3.03	3.19
12.12	2.43	2.17	2.55	2.48	2.53	2.68	2.77	2.89	3.16	3.11	3.17
12.32	2.32	2.24	2.44	2.58	2.51	2.77	2.88	3.01	2.91	3.07	3.40
12.52	2.13	2.18	2.32	2.63	2.50	2.68	2.72	2.96	3.11	3.12	3.13
12.72	2.15	2.15	2.28	2.49	2.42	2.54	2.69	2.94	3.08	3.07	3.28
12.92	2.06	2.34	2.40	2.55	2.70	2.67	2.82	2.92	3.15	3.20	3.22
13.12	1.87	2.02	2.22	2.49	2.36	2.45	2.61	2.62	2.81	2.89	2.94
13.32	2.30	2.26	2.42	2.39	2.37	2.59	2.86	2.82	3.06	2.96	3.22

13.52	1.99	2.04	2.13	2.46	2.36	2.41	2.55	2.65	2.76	2.92	3.08
13.72	2.30	2.30	2.33	2.68	2.71	2.83	2.83	3.13	3.17	3.33	3.51
13.92	2.04	2.12	2.42	2.58	2.42	2.41	2.85	2.79	2.77	2.98	3.00
14.00	2.27	2.23	2.47	2.67	2.60	2.76	2.85	2.89	3.13	3.08	3.30
14.08	1.94	1.95	2.15	2.16	2.52	2.30	2.54	2.66	2.94	2.90	3.03
14.17	2.01	2.14	2.17	2.24	2.28	2.36	2.50	2.65	2.90	3.12	3.10
14.27	2.15	2.10	2.37	2.57	2.49	2.52	2.59	2.80	3.00	3.04	3.08
14.38	1.97	2.34	2.36	2.44	2.52	2.70	2.75	2.85	3.05	3.16	3.14
14.49	2.18	2.21	2.42	2.41	2.48	2.59	2.88	2.70	2.89	3.14	3.16
14.61	2.04	2.09	2.18	2.36	2.30	2.56	2.54	2.73	2.84	2.99	2.89
14.75	2.15	1.87	2.15	2.12	2.29	2.39	2.48	2.59	2.71	2.96	2.92
14.89	2.28	2.27	2.62	2.70	2.62	2.63	2.92	3.02	3.20	3.34	3.48
15.05	2.25	2.18	2.41	2.72	2.74	2.78	2.79	2.98	3.00	3.28	3.24
15.21	2.22	2.26	2.39	2.62	2.55	2.84	2.83	2.88	3.13	3.19	3.22
15.39	1.96	2.08	2.32	2.44	2.34	2.64	2.71	2.81	3.11	2.87	3.19
15.59	1.98	2.04	2.20	2.18	2.44	2.32	2.47	2.60	2.95	2.92	2.89
15.80	2.28	2.20	2.28	2.47	2.51	2.68	2.64	2.75	3.19	3.14	3.25
16.02	1.90	2.15	2.06	2.30	2.31	2.39	2.59	2.64	2.74	2.97	3.01
16.27	2.11	2.12	2.44	2.53	2.56	2.61	2.76	2.85	3.08	3.23	3.32
16.53	2.20	2.11	2.22	2.43	2.66	2.64	2.65	2.91	3.01	3.11	3.07
16.81	1.95	2.05	2.04	2.26	2.52	2.40	2.60	2.65	2.90	2.93	3.12
17.12	2.01	2.09	2.19	2.33	2.48	2.49	2.63	2.92	2.83	2.95	3.03
17.45	1.89	1.79	2.07	2.13	2.35	2.27	2.39	2.45	2.54	2.82	2.99
17.81	2.05	2.04	2.36	2.28	2.52	2.65	2.63	3.01	3.11	3.12	3.09
18.19	2.18	2.05	2.32	2.59	2.57	2.81	2.78	2.83	3.01	3.28	3.41
18.60	2.23	2.35	2.31	2.54	2.59	2.65	2.85	2.94	3.18	3.26	3.29
19.05	2.10	2.10	2.15	2.38	2.38	2.44	2.68	2.79	3.02	3.09	3.27
19.53	2.14	2.13	2.21	2.39	2.40	2.60	2.62	2.71	2.95	3.07	3.02
20.05	2.34	2.21	2.40	2.63	2.55	2.64	2.67	2.89	2.95	3.30	3.04
20.61	2.29	2.31	2.44	2.49	2.61	2.69	2.81	2.94	3.11	3.21	3.32
21.22	2.20	2.37	2.20	2.53	2.36	2.54	2.66	2.78	2.84	3.16	3.19
21.87	2.12	2.05	2.50	2.42	2.50	2.73	2.78	2.78	3.04	3.14	3.19
22.57	2.09	2.13	2.23	2.45	2.46	2.56	2.65	2.72	3.04	3.11	3.17
23.33	2.15	2.11	2.07	2.39	2.59	2.48	2.72	2.76	2.98	3.03	3.07
24.15	2.00	2.20	2.29	2.51	2.35	2.49	2.52	2.76	2.82	2.94	3.11
25.03	1.96	1.95	2.25	2.33	2.28	2.55	2.62	2.70	2.78	2.92	2.98
25.98	2.08	2.15	2.33	2.46	2.50	2.51	2.76	2.84	3.02	3.11	3.14
27.01	1.95	2.14	2.09	2.26	2.43	2.36	2.65	2.82	2.93	2.94	3.14
28.11	2.08	2.16	2.27	2.28	2.50	2.58	2.58	2.61	2.79	3.00	3.14
29.30	2.06	2.10	1.99	2.23	2.36	2.40	2.50	2.78	2.82	3.01	2.97
30.59	1.96	2.18	2.15	2.57	2.48	2.65	2.66	2.88	2.90	2.93	3.21
31.98	2.07	1.93	2.02	2.48	2.43	2.51	2.57	2.56	2.84	2.90	3.04
33.47	2.03	2.04	2.21	2.53	2.42	2.48	2.66	2.72	2.81	2.89	3.10
35.09	2.13	2.10	2.27	2.47	2.44	2.62	2.69	2.72	2.80	3.18	3.13
36.83	1.90	1.97	2.10	2.30	2.34	2.41	2.42	2.66	2.78	2.83	2.96
38.71	1.87	1.92	2.17	2.25	2.46	2.51	2.44	2.87	2.87	3.11	3.08
40.73	2.00	2.21	2.23	2.34	2.32	2.51	2.54	2.81	2.87	3.00	3.16
42.92	2.03	1.98	2.29	2.38	2.53	2.65	2.62	2.80	2.89	2.97	3.18
45.27	2.03	2.11	2.20	2.51	2.37	2.47	2.52	2.60	2.85	2.87	3.01
47.81	2.09	2.02	2.19	2.50	2.36	2.59	2.60	2.82	2.73	3.08	3.24

50.55	1.96	1.95	1.91	2.15	2.22	2.40	2.35	2.46	2.65	2.69	2.87
53.51	2.09	2.25	2.13	2.36	2.30	2.49	2.67	2.70	2.89	3.02	3.08
56.69	2.06	1.97	2.23	2.39	2.27	2.43	2.53	2.75	2.76	3.04	3.09
60.13	2.06	2.09	1.97	2.24	2.45	2.32	2.40	2.52	2.76	2.63	2.72
63.84	2.06	2.25	2.15	2.35	2.56	2.56	2.76	2.73	2.96	3.15	3.09
67.83	2.01	2.20	2.14	2.30	2.42	2.24	2.57	2.62	2.84	2.77	2.95
72.15	1.82	1.82	2.18	2.11	2.26	2.26	2.44	2.53	2.72	2.85	2.98
76.80	1.90	1.74	2.09	2.16	2.08	2.35	2.44	2.68	2.55	2.62	2.83
81.81	1.85	2.00	2.12	2.27	2.42	2.43	2.49	2.57	2.90	2.87	2.93
87.22	1.87	1.92	1.97	2.20	2.19	2.43	2.51	2.64	2.62	2.71	2.89
93.06	1.69	1.97	2.22	2.10	2.25	2.42	2.42	2.41	2.70	2.61	2.75
99.35	1.85	1.87	2.08	2.03	2.20	2.33	2.43	2.64	2.69	2.81	2.84
106.13	1.96	1.83	2.18	2.41	2.35	2.37	2.36	2.61	2.69	2.69	2.90
113.45	1.90	2.26	2.29	2.38	2.37	2.53	2.60	2.80	2.83	3.04	3.03
121.34	1.92	1.94	2.00	2.20	2.04	2.19	2.30	2.52	2.67	2.75	2.82
129.86	1.81	1.85	1.98	2.28	2.21	2.26	2.29	2.47	2.73	2.64	2.75
139.04	2.00	1.94	2.07	2.19	2.19	2.34	2.46	2.42	2.70	2.72	2.93
148.94	1.66	1.76	1.93	1.97	2.05	2.17	2.09	2.36	2.37	2.50	2.57
159.62	1.80	1.67	2.02	2.09	2.26	2.32	2.40	2.49	2.59	2.61	2.66
171.14	1.95	1.83	1.93	2.17	2.28	2.46	2.49	2.60	2.62	2.74	2.88
183.56	2.07	1.87	2.06	2.18	2.15	2.26	2.31	2.31	2.44	2.62	2.58
196.96	1.82	1.97	1.92	2.32	2.28	2.34	2.25	2.43	2.71	2.72	2.82
211.41	1.72	1.68	1.92	1.97	2.09	2.20	2.20	2.34	2.52	2.53	2.63
226.99	1.64	1.72	1.72	1.99	2.01	2.14	2.20	2.40	2.61	2.49	2.60
243.80	1.67	1.73	1.65	1.99	1.96	2.03	2.20	2.31	2.33	2.40	2.55
261.93	1.83	1.85	1.95	1.94	2.23	2.22	2.31	2.43	2.55	2.61	2.79
281.48	1.85	1.87	2.02	2.20	1.99	2.28	2.23	2.48	2.61	2.68	2.73
302.56	1.63	1.62	1.84	1.87	2.05	2.10	2.07	2.21	2.36	2.45	2.58
325.30	1.70	1.79	1.87	2.06	1.92	2.16	2.00	2.16	2.38	2.44	2.63
349.83	1.47	1.63	1.73	1.89	1.77	1.84	2.19	2.03	2.19	2.19	2.37
376.28	1.68	1.73	1.73	1.88	1.93	2.07	2.05	2.11	2.41	2.44	2.61
404.81	1.69	1.82	1.86	2.13	1.91	2.02	2.34	2.24	2.47	2.44	2.78
435.58	1.73	1.77	1.78	1.91	2.10	2.11	2.12	2.21	2.52	2.40	2.54
468.76	1.45	1.52	1.71	1.86	1.82	1.86	2.06	2.19	2.23	2.31	2.48
504.55	1.44	1.41	1.52	1.78	1.81	1.79	1.73	2.01	2.09	2.17	2.12
543.15	1.48	1.53	1.63	1.79	1.72	1.86	1.82	1.95	2.26	2.28	2.28
584.78	1.44	1.37	1.54	1.52	1.64	1.80	1.93	1.97	1.88	2.11	2.23
629.68	1.41	1.53	1.53	1.68	1.76	1.84	1.67	1.96	2.10	2.34	2.06
678.10	1.64	1.77	1.55	1.75	1.65	1.93	1.99	2.03	2.06	2.25	2.26
730.33	1.59	1.63	1.80	1.79	1.89	2.01	1.87	1.98	2.13	2.30	2.39
786.65	1.52	1.24	1.70	1.59	1.68	1.83	1.88	2.02	2.02	2.17	2.15
847.40	1.15	1.20	1.38	1.65	1.46	1.40	1.60	1.71	1.91	1.95	2.02
912.92	1.23	1.44	1.59	1.58	1.70	1.72	1.90	2.07	2.03	2.14	2.19
983.58	1.29	1.40	1.59	1.58	1.67	1.58	1.73	1.79	2.02	2.10	2.10
1059.78	1.41	1.43	1.44	1.52	1.69	1.66	1.75	1.94	1.97	2.20	2.21
1141.98	1.26	1.30	1.41	1.41	1.46	1.56	1.55	1.70	1.92	1.75	1.91
1230.62	1.24	1.36	1.30	1.39	1.48	1.52	1.39	1.63	1.75	1.87	1.74
1326.23	1.42	1.43	1.67	1.76	1.58	1.64	1.76	1.79	1.93	1.99	1.91
1429.34	1.27	1.07	1.31	1.33	1.37	1.54	1.49	1.73	1.87	1.98	1.98
1540.54	1.22	1.25	1.29	1.45	1.50	1.64	1.74	1.71	1.88	1.92	1.99

1660.48	1.20	1.27	1.10	1.57	1.48	1.49	1.73	1.68	1.89	2.00	2.06
1789.83	1.29	0.98	1.22	1.37	1.31	1.35	1.52	1.46	1.59	1.67	1.79
1929.34	1.03	1.07	1.23	1.38	1.28	1.37	1.53	1.51	1.61	1.66	1.80
2079.80	1.11	1.02	1.25	1.17	1.36	1.43	1.40	1.51	1.60	1.68	1.67
2242.08	1.03	1.09	1.19	1.17	1.39	1.29	1.42	1.55	1.62	1.64	1.68
2417.10	0.89	0.89	0.91	1.12	1.26	1.28	1.30	1.47	1.52	1.56	1.60
2605.85	1.06	1.05	1.15	1.14	1.13	1.24	1.39	1.45	1.53	1.61	1.49
2809.43	0.72	1.02	0.98	1.19	1.05	1.11	1.17	1.28	1.46	1.36	1.53
3028.99	0.88	0.96	1.10	1.11	1.11	1.22	1.14	1.34	1.37	1.39	1.48
3265.79	0.89	0.89	0.84	1.03	1.28	1.09	1.27	1.38	1.40	1.47	1.68
3521.18	1.06	0.98	1.11	1.26	1.34	1.16	1.28	1.38	1.49	1.58	1.57
3796.62	0.79	0.92	0.81	1.00	1.11	1.09	1.11	1.21	1.30	1.35	1.43

Wavelength (nm) / Time (ps)	398.17	399.58	400.99	402.40	403.81	405.22	406.63	408.03	409.44	410.85	412.26
-3.28	-0.04	-0.13	-0.13	0.14	0.04	-0.13	-0.03	0.04	-0.06	-0.16	-0.10
-2.78	-0.01	0.01	-0.09	-0.16	-0.18	0.08	-0.10	-0.07	-0.11	0.00	-0.07
-2.28	-0.06	0.01	0.02	-0.14	0.02	-0.27	-0.19	-0.12	-0.19	-0.02	-0.03
-1.78	0.12	0.05	0.09	0.09	0.18	0.11	0.21	-0.03	0.17	0.22	0.13
-1.28	-0.15	0.02	-0.02	-0.10	-0.15	-0.11	-0.04	-0.01	0.16	-0.26	-0.13
-0.78	-0.03	-0.15	-0.09	0.10	-0.21	-0.02	0.01	-0.03	-0.17	0.22	0.07
-0.28	0.18	0.09	0.08	0.00	0.07	0.17	0.07	-0.12	-0.03	0.04	0.02
0.22	-0.05	0.06	0.12	-0.01	0.09	0.10	0.20	0.26	0.15	0.02	0.07
0.32	-0.04	-0.02	0.00	-0.04	0.08	0.11	-0.22	-0.02	0.11	-0.05	0.03
0.42	0.07	0.06	0.00	0.13	0.05	-0.04	0.08	0.09	-0.04	-0.03	-0.01
0.52	0.07	-0.07	-0.06	-0.06	0.05	0.12	0.19	-0.02	-0.18	0.04	0.10
0.62	0.03	-0.01	0.14	-0.03	-0.14	0.15	0.06	-0.01	-0.03	-0.04	0.19
0.72	-0.02	-0.04	0.16	-0.08	0.06	0.03	0.18	-0.03	0.24	-0.07	0.12
0.77	0.09	0.14	0.07	-0.08	-0.02	0.01	0.06	0.15	0.35	0.15	-0.02
0.82	-0.05	-0.15	-0.13	-0.07	-0.16	0.05	0.10	-0.17	0.04	0.00	-0.13
0.87	0.17	0.10	0.09	0.20	0.27	-0.11	0.06	0.38	0.20	0.04	0.30
0.92	-0.21	-0.31	-0.16	-0.04	0.03	-0.13	0.00	-0.35	-0.04	-0.31	-0.12
0.97	-0.08	-0.42	-0.63	-0.66	-0.32	-0.03	0.01	-0.17	0.38	-0.05	-0.02
1.02	3.30	2.12	1.05	-0.17	-0.46	-0.82	-0.43	-0.57	-0.08	-0.20	-0.06
1.07	6.04	5.95	5.37	4.59	3.87	2.89	2.33	1.69	1.39	0.93	1.03
1.12	7.08	7.19	7.25	6.84	6.46	5.90	5.52	5.48	4.86	4.56	4.62
1.17	5.73	5.95	5.96	6.22	6.10	6.08	6.43	6.34	6.14	6.08	6.21
1.22	5.30	5.31	5.59	5.64	5.44	5.67	5.90	5.64	5.86	5.84	6.05
1.27	5.13	5.20	5.29	5.37	5.21	5.38	5.19	5.25	5.74	5.63	6.05
1.32	4.96	4.92	4.77	4.83	4.75	4.92	5.07	5.25	5.34	5.42	5.88
1.37	4.53	4.43	4.47	4.47	4.62	4.55	4.81	4.84	5.11	5.21	5.27
1.42	4.64	4.56	4.51	4.58	4.76	4.74	4.47	4.82	4.95	5.03	5.29
1.47	4.36	4.49	4.37	4.25	4.25	4.17	4.32	4.85	4.50	4.75	5.15
1.52	4.36	4.40	4.16	4.20	4.29	4.26	4.42	4.64	4.82	4.48	4.84
1.57	4.14	4.12	4.15	4.23	4.18	4.24	4.33	4.57	4.48	4.65	5.07
1.62	4.02	4.10	4.01	4.22	3.91	4.26	4.08	4.56	4.66	4.73	4.79
1.67	4.36	4.20	4.16	4.28	4.47	4.47	4.30	4.50	4.76	4.84	4.90
1.72	4.01	4.02	4.09	3.98	4.24	4.10	4.20	4.20	4.68	4.61	4.85
1.77	3.97	3.86	3.91	3.83	3.93	4.16	4.00	4.07	4.38	4.46	4.61

1.82	3.90	3.80	3.76	3.86	4.01	3.94	3.91	4.07	4.30	4.08	4.57
1.87	3.78	3.67	3.62	3.63	3.79	3.89	3.86	4.21	4.13	4.10	4.57
1.92	3.97	3.86	3.78	3.84	4.01	3.96	4.05	4.01	4.33	4.44	4.53
1.97	3.92	3.99	3.99	4.16	3.97	3.86	4.10	4.24	4.43	4.37	4.63
2.02	3.84	3.79	3.68	3.82	3.69	3.97	4.16	4.03	4.23	4.33	4.47
2.07	3.62	3.61	3.58	3.71	3.62	3.81	3.97	4.05	3.89	4.25	4.46
2.12	3.98	3.95	4.12	3.96	3.92	4.00	3.91	4.21	4.38	4.63	4.81
2.17	3.74	3.75	3.71	3.88	3.73	3.76	3.79	4.10	4.20	4.18	4.41
2.22	3.77	3.59	3.57	3.77	3.70	3.78	3.88	3.74	4.20	4.15	4.39
2.27	3.72	3.50	3.70	3.78	3.65	3.70	3.76	4.00	4.29	4.09	4.44
2.32	3.49	3.55	3.59	3.49	3.46	3.48	3.65	3.76	4.01	4.03	4.21
2.37	3.90	3.74	3.90	3.88	3.85	4.00	4.02	4.04	4.06	4.29	4.61
2.42	3.86	3.83	3.83	3.89	3.99	3.73	3.99	3.99	4.28	4.30	4.29
2.47	3.41	3.48	3.59	3.52	3.57	3.69	3.69	3.86	4.15	3.97	4.29
2.52	3.67	3.50	3.61	3.75	3.85	3.81	4.00	3.91	4.06	4.06	4.29
2.57	3.56	3.49	3.59	3.40	3.55	3.67	3.70	3.70	3.83	3.89	4.09
2.62	3.55	3.57	3.50	3.45	3.47	3.63	3.65	3.79	4.11	4.10	4.26
2.67	3.47	3.42	3.36	3.39	3.43	3.31	3.48	3.63	3.96	3.67	4.08
2.72	3.53	3.75	3.61	3.50	3.37	3.55	3.69	3.75	3.98	3.82	4.31
2.77	3.80	3.85	3.59	4.00	3.69	3.69	4.25	4.15	4.13	4.37	4.48
2.82	3.66	3.53	3.55	3.51	3.57	3.49	3.75	3.64	4.01	3.96	4.14
2.87	3.33	3.34	3.56	3.72	3.55	3.58	3.82	3.52	3.90	3.85	4.20
2.92	3.50	3.60	3.47	3.36	3.40	3.57	3.69	3.79	3.83	3.69	4.06
2.97	3.37	3.39	3.43	3.35	3.61	3.65	3.72	3.63	4.03	3.82	4.04
3.02	3.64	3.73	3.73	3.64	3.93	3.72	4.12	3.86	4.07	4.31	4.70
3.07	3.54	3.63	3.46	3.61	3.65	3.71	3.44	3.86	4.25	4.11	4.37
3.12	3.64	3.80	3.85	3.80	3.91	3.51	3.83	4.03	3.92	4.14	4.48
3.17	3.56	3.70	3.67	3.55	3.79	3.54	3.58	3.80	4.16	4.05	4.28
3.22	3.52	3.31	3.53	3.56	3.63	3.68	3.71	4.15	3.98	4.00	4.22
3.27	3.56	3.63	3.60	3.60	4.00	3.80	3.73	3.80	4.09	4.18	4.26
3.32	3.84	3.70	3.63	3.69	3.64	3.89	3.87	4.29	4.20	4.35	4.50
3.37	3.51	3.59	3.48	3.54	3.56	3.45	3.55	3.91	3.91	4.12	4.20
3.42	3.55	3.62	3.63	3.75	3.82	3.69	3.72	4.12	4.06	4.22	4.46
3.47	3.43	3.62	3.63	3.52	3.64	3.56	3.65	3.76	4.01	3.99	4.17
3.52	3.69	3.62	3.51	3.58	3.55	3.74	3.58	3.84	3.88	3.88	4.15
3.57	3.54	3.53	3.52	3.25	3.34	3.55	3.52	3.67	3.96	3.76	4.27
3.62	3.51	3.59	3.62	3.50	3.34	3.65	3.82	3.66	3.85	3.84	4.32
3.67	3.51	3.44	3.43	3.42	3.61	3.46	3.47	3.69	3.87	3.89	4.14
3.72	3.64	3.51	3.52	3.40	3.39	3.42	3.29	3.82	3.98	3.90	4.11
3.92	3.49	3.54	3.63	3.56	3.53	3.63	3.66	3.77	3.93	4.06	4.14
4.12	3.53	3.51	3.55	3.47	3.51	3.62	3.67	3.78	3.79	4.12	4.26
4.32	3.16	3.44	3.42	3.37	3.71	3.47	3.36	3.60	3.73	3.74	4.08
4.52	3.41	3.40	3.60	3.37	3.36	3.46	3.58	3.75	3.85	4.16	4.09
4.72	3.60	3.50	3.39	3.45	3.31	3.49	3.68	3.72	3.80	3.77	4.01
4.92	3.57	3.52	3.33	3.59	3.35	3.60	3.71	3.79	3.99	3.89	4.13
5.12	3.42	3.36	3.39	3.45	3.51	3.56	3.69	3.67	3.85	3.80	4.22
5.32	3.67	3.56	3.47	3.51	3.56	3.73	3.71	3.80	4.06	4.17	4.48
5.52	3.57	3.46	3.37	3.71	3.62	3.75	4.02	3.84	4.11	4.15	4.39
5.72	3.62	3.61	3.57	3.69	3.68	3.63	3.68	3.92	4.02	4.03	4.50
5.92	3.49	3.40	3.37	3.30	3.37	3.42	3.64	3.70	3.87	3.59	3.89

6.12	3.42	3.46	3.43	3.41	3.44	3.73	3.54	3.78	3.92	3.88	4.12
6.32	3.39	3.30	3.33	3.34	3.41	3.43	3.27	3.71	3.77	3.74	3.97
6.52	3.52	3.49	3.44	3.55	3.55	3.54	3.70	3.69	3.97	3.96	4.17
6.72	3.51	3.57	3.49	3.55	3.62	3.64	3.81	4.04	4.12	4.17	4.49
6.92	3.36	3.35	3.43	3.37	3.38	3.53	3.60	3.67	4.10	3.81	4.45
7.12	3.40	3.36	3.35	3.28	3.41	3.54	3.91	3.72	3.87	3.71	4.14
7.32	3.49	3.40	3.49	3.70	3.68	3.59	3.72	3.71	4.06	4.03	4.30
7.52	3.42	3.35	3.31	3.31	3.49	3.31	3.62	3.69	3.88	3.74	4.18
7.72	3.44	3.35	3.41	3.32	3.27	3.45	3.29	3.88	3.62	3.90	4.04
7.92	3.46	3.36	3.40	3.49	3.51	3.54	3.67	3.68	3.92	3.73	4.11
8.12	3.49	3.63	3.41	3.50	3.59	3.66	3.76	4.03	4.00	4.20	4.51
8.32	3.41	3.40	3.34	3.32	3.34	3.44	3.83	3.71	3.78	3.92	4.22
8.52	3.16	3.28	3.23	3.09	3.42	3.20	3.44	3.46	3.71	3.46	3.91
8.72	3.33	3.46	3.38	3.32	3.49	3.37	3.42	3.76	3.79	3.84	4.12
8.92	3.31	3.14	3.35	3.10	3.45	3.30	3.41	3.32	3.85	3.65	3.96
9.12	3.57	3.42	3.38	3.40	3.43	3.50	3.51	3.71	3.90	4.05	4.31
9.32	3.14	3.20	3.23	3.37	3.40	3.28	3.65	3.36	3.54	3.64	3.99
9.52	3.38	3.37	3.47	3.32	3.22	3.22	3.43	3.47	4.07	3.94	4.18
9.72	3.27	3.20	3.30	3.21	3.41	3.59	3.60	3.79	3.94	3.93	4.14
9.92	3.52	3.48	3.48	3.38	3.51	3.41	3.40	3.67	3.86	3.98	3.99
10.12	3.56	3.58	3.46	3.48	3.59	3.64	3.87	3.88	3.95	3.77	4.26
10.32	3.20	3.04	3.38	3.20	3.27	3.25	3.18	3.54	3.64	3.63	4.09
10.52	3.40	3.39	3.55	3.32	3.53	3.38	3.55	3.72	3.83	4.09	4.16
10.72	3.27	3.20	3.34	3.23	3.53	3.52	3.51	3.39	3.83	3.92	4.25
10.92	3.26	3.10	3.20	3.13	3.21	3.35	3.47	3.53	3.82	3.72	4.13
11.12	3.30	3.41	3.17	3.22	3.35	3.41	3.47	3.64	3.80	3.72	4.02
11.32	3.47	3.54	3.59	3.66	3.53	3.42	3.60	3.81	4.12	3.94	4.48
11.52	3.08	3.19	3.35	3.07	3.14	3.36	3.63	3.56	3.68	3.63	4.26
11.72	3.27	3.38	3.35	3.24	3.33	3.64	3.72	3.70	3.99	4.04	4.35
11.92	3.26	3.21	3.29	3.20	3.11	3.29	3.37	3.50	3.78	3.87	4.13
12.12	3.29	3.30	3.37	3.28	3.36	3.71	3.43	3.82	3.88	4.02	4.31
12.32	3.18	3.36	3.38	3.39	3.38	3.68	3.58	3.73	3.83	4.09	4.21
12.52	3.26	3.27	3.25	3.36	3.33	3.26	3.66	3.57	3.61	3.82	3.92
12.72	3.31	3.37	3.15	3.23	3.44	3.45	3.56	3.72	3.86	4.08	4.21
12.92	3.45	3.37	3.40	3.29	3.40	3.47	3.66	3.74	3.89	4.07	4.06
13.12	3.16	3.13	3.16	3.08	3.10	3.18	3.35	3.35	3.86	3.62	3.75
13.32	3.39	3.37	3.23	3.33	3.16	3.53	3.72	3.48	3.78	3.69	4.04
13.52	2.99	3.07	2.95	3.19	3.06	3.32	3.35	3.56	3.63	3.74	3.98
13.72	3.42	3.33	3.42	3.39	3.60	3.62	3.53	3.65	3.96	4.08	4.17
13.92	3.13	3.10	3.39	3.13	3.34	3.36	3.43	3.54	3.72	3.99	3.88
14.00	3.37	3.37	3.45	3.56	3.47	3.55	3.47	3.64	4.05	4.08	4.30
14.08	3.24	3.17	3.23	3.13	3.29	3.10	3.47	3.75	3.45	3.70	4.14
14.17	3.16	3.16	3.22	3.11	3.00	3.26	3.26	3.52	3.64	3.68	3.91
14.27	3.19	3.29	3.33	3.37	3.36	3.35	3.47	3.62	3.89	3.82	4.08
14.38	3.17	3.35	3.17	3.13	3.15	3.20	3.38	3.56	3.63	3.67	3.86
14.49	3.12	3.25	3.13	3.38	3.13	3.44	3.26	3.47	3.66	3.77	4.06
14.61	3.26	3.15	3.24	3.25	3.26	3.28	3.43	3.49	3.87	3.83	3.98
14.75	3.14	3.07	3.28	3.02	3.27	3.21	3.46	3.77	3.63	3.71	3.98
14.89	3.51	3.56	3.48	3.59	3.66	3.55	3.76	3.74	3.88	4.21	4.38
15.05	3.40	3.53	3.33	3.08	3.56	3.39	3.58	3.27	3.62	3.90	4.15

15.21	3.41	3.37	3.45	3.28	3.45	3.46	3.52	3.58	4.13	3.98	4.27
15.39	3.27	3.29	3.26	3.14	3.55	3.34	3.36	3.44	3.53	3.63	3.93
15.59	3.18	3.12	3.17	3.11	3.22	3.38	3.43	3.40	3.70	3.92	4.04
15.80	3.25	3.25	3.28	3.41	3.55	3.38	3.59	3.77	4.04	3.85	4.32
16.02	3.11	3.10	3.02	3.17	3.12	3.17	3.21	3.59	3.54	3.34	3.83
16.27	3.28	3.38	3.16	3.39	3.58	3.66	3.54	4.03	3.93	3.98	4.27
16.53	3.25	3.25	3.26	3.36	3.24	3.47	3.47	3.63	3.84	3.96	3.97
16.81	3.09	2.96	3.06	2.98	3.03	3.12	3.29	3.55	3.65	3.44	3.90
17.12	3.24	3.10	3.07	3.13	3.23	3.51	3.43	3.46	3.65	3.86	3.99
17.45	2.96	2.95	3.13	3.07	2.96	3.15	3.24	3.39	3.64	3.67	3.79
17.81	3.17	3.25	3.29	3.12	3.47	3.53	3.78	3.70	4.07	4.09	4.20
18.19	3.46	3.42	3.30	3.50	3.33	3.45	3.67	3.56	3.86	3.89	4.24
18.60	3.47	3.41	3.39	3.33	3.48	3.49	3.80	3.82	4.12	4.11	4.27
19.05	3.20	3.34	3.12	3.26	3.31	3.34	3.68	3.66	3.82	3.82	4.00
19.53	3.22	3.09	3.18	3.21	3.21	3.22	3.31	3.61	3.76	3.88	3.96
20.05	3.24	3.32	3.28	3.43	3.41	3.46	3.37	3.62	4.12	3.89	4.18
20.61	3.51	3.45	3.41	3.44	3.17	3.48	3.43	3.49	3.92	4.03	4.33
21.22	3.12	3.17	3.47	3.23	3.35	3.21	3.51	3.60	4.02	3.86	4.26
21.87	3.21	3.30	3.27	3.44	3.08	3.33	3.57	3.52	3.94	3.96	4.15
22.57	3.30	3.35	3.27	3.27	3.35	3.41	3.54	3.34	4.08	3.84	4.14
23.33	3.19	3.07	3.38	3.32	3.56	3.15	3.69	3.68	3.85	3.78	4.30
24.15	3.24	3.08	3.20	3.13	3.26	3.36	3.42	3.31	3.65	3.51	3.98
25.03	3.09	3.16	3.21	3.16	3.08	3.09	3.25	3.58	3.58	3.74	3.88
25.98	3.25	3.19	3.19	3.32	3.09	3.42	3.52	3.66	3.82	3.86	4.32
27.01	3.07	3.06	3.18	3.26	3.33	3.08	3.18	3.60	3.76	3.81	4.24
28.11	3.25	3.24	3.26	3.23	3.17	3.52	3.39	3.55	3.82	3.70	4.18
29.30	3.07	3.13	3.05	3.08	3.27	3.30	3.35	3.49	3.91	3.65	4.04
30.59	3.07	3.11	3.36	3.34	3.35	3.24	3.52	3.64	3.77	3.71	4.20
31.98	3.21	3.11	3.16	3.16	3.12	3.32	3.35	3.27	3.56	3.58	4.02
33.47	3.14	3.07	3.22	3.17	3.05	3.20	3.36	3.75	3.65	3.91	4.16
35.09	3.11	3.19	3.16	3.12	3.37	3.32	3.49	3.42	3.86	3.84	4.11
36.83	3.06	3.11	3.14	3.12	3.25	3.21	3.40	3.29	3.68	3.93	4.13
38.71	3.04	3.19	3.22	2.97	3.18	3.30	3.31	3.35	3.51	3.58	3.96
40.73	3.11	3.20	3.16	2.99	3.24	3.27	3.46	3.52	3.64	3.91	4.05
42.92	3.23	3.21	3.16	3.20	3.36	3.40	3.20	3.52	3.63	3.83	4.04
45.27	3.08	3.02	3.10	3.07	3.11	3.32	3.43	3.55	3.76	3.78	4.05
47.81	3.12	3.27	2.97	3.15	3.07	3.16	3.35	3.21	3.45	3.80	4.07
50.55	2.82	2.83	2.85	2.91	2.92	3.03	3.23	3.30	3.53	3.60	3.57
53.51	3.26	3.10	3.19	3.06	3.23	3.09	3.39	3.67	3.88	3.86	4.20
56.69	3.11	3.24	3.18	3.27	3.23	3.40	3.19	3.53	3.70	3.72	4.19
60.13	2.88	2.97	3.08	3.04	2.94	3.01	3.11	3.33	3.72	3.78	4.06
63.84	2.99	3.28	3.08	3.02	3.13	3.30	3.33	3.59	3.75	3.93	4.04
67.83	3.06	3.08	2.96	3.09	2.95	3.32	3.44	3.40	3.59	3.92	4.20
72.15	3.01	2.87	2.96	2.94	2.90	2.90	3.38	3.43	3.51	3.92	3.92
76.80	2.83	3.01	2.98	3.00	2.96	3.02	3.11	3.44	3.53	3.49	3.81
81.81	3.05	3.08	3.19	2.98	3.19	3.19	3.40	3.56	3.41	3.80	4.24
87.22	2.96	2.94	2.77	3.03	2.69	2.67	3.10	3.33	3.24	3.59	3.70
93.06	2.85	2.98	2.89	2.60	3.05	2.88	3.11	3.29	3.65	3.50	3.91
99.35	2.93	2.88	3.08	2.92	2.91	3.30	3.16	3.38	3.68	3.68	3.75
106.13	2.89	2.95	2.73	3.09	2.93	2.99	3.08	3.29	3.57	3.70	3.87

113.45	3.12	3.05	3.13	3.16	3.03	3.12	3.43	3.46	3.63	3.78	4.01
121.34	2.84	2.97	2.90	2.91	2.97	3.13	3.02	3.42	3.61	3.45	3.78
129.86	2.91	3.02	2.97	2.92	2.77	2.92	3.13	3.21	3.37	3.50	3.64
139.04	2.94	3.08	2.84	2.86	2.96	2.97	2.98	3.04	3.24	3.50	3.59
148.94	2.82	2.67	2.67	2.65	2.71	2.58	3.00	3.09	3.42	3.46	3.51
159.62	2.67	2.81	2.81	2.85	2.97	2.95	3.26	3.23	3.41	3.68	3.97
171.14	2.93	2.86	3.01	2.78	3.03	3.22	3.11	3.35	3.46	3.37	3.76
183.56	2.80	2.63	2.66	2.52	2.93	2.70	2.97	2.98	3.43	3.60	3.52
196.96	2.96	2.95	2.87	2.85	2.92	2.89	3.18	3.46	3.28	3.70	3.78
211.41	2.72	2.63	2.71	2.62	3.00	2.99	2.98	3.21	3.13	3.11	3.66
226.99	2.76	2.80	2.71	2.92	2.98	2.88	3.23	3.31	3.41	3.25	3.76
243.80	2.62	2.70	2.47	2.66	2.70	2.80	2.89	2.84	3.13	3.41	3.45
261.93	2.85	2.79	2.89	2.94	2.94	2.67	3.06	3.02	3.24	3.52	3.69
281.48	2.86	2.98	2.84	2.80	2.80	2.98	2.98	3.11	3.36	3.42	3.57
302.56	2.73	2.63	2.59	2.54	2.39	2.81	2.77	3.01	3.32	3.12	3.53
325.30	2.55	2.54	2.47	2.51	2.73	2.90	3.11	2.88	2.92	3.35	3.50
349.83	2.25	2.43	2.27	2.43	2.49	2.53	2.70	3.05	2.83	3.06	3.42
376.28	2.43	2.58	2.47	2.58	2.52	2.72	2.90	3.06	3.18	3.08	3.56
404.81	2.68	2.79	2.79	2.73	2.91	2.88	3.24	3.01	3.44	3.38	3.56
435.58	2.60	2.52	2.61	2.39	2.64	2.66	2.69	2.61	2.95	3.14	3.39
468.76	2.49	2.40	2.50	2.56	2.35	2.43	2.63	2.76	2.94	2.85	3.27
504.55	2.46	2.32	2.27	2.48	2.42	2.55	2.62	2.78	2.97	2.75	3.23
543.15	2.40	2.32	2.39	2.25	2.43	2.36	2.70	2.71	3.01	2.95	3.29
584.78	2.14	2.34	2.35	2.35	2.31	2.19	2.59	2.44	2.87	2.87	2.96
629.68	2.32	2.20	2.35	2.45	2.38	2.49	2.37	2.66	2.82	2.88	3.13
678.10	2.38	2.36	2.42	2.32	2.44	2.36	2.72	2.70	2.75	3.13	3.39
730.33	2.35	2.43	2.38	2.55	2.38	2.67	2.63	2.81	3.04	3.10	3.39
786.65	2.29	2.26	2.42	2.36	2.31	2.49	2.67	2.66	2.75	3.00	3.13
847.40	2.14	2.14	2.05	2.21	2.31	2.31	2.36	2.35	2.74	2.73	3.15
912.92	2.26	2.27	2.03	2.28	2.12	2.26	2.33	2.88	2.76	2.67	3.21
983.58	2.27	2.08	2.06	2.24	2.08	2.37	2.47	2.57	2.53	2.99	3.16
1059.78	2.26	2.27	2.08	2.21	2.43	2.18	2.43	2.70	2.68	2.67	3.19
1141.98	2.05	1.93	1.89	1.87	1.84	2.16	2.03	2.35	2.36	2.43	2.99
1230.62	1.83	2.01	1.81	1.89	1.97	1.99	1.98	2.45	2.66	2.36	2.73
1326.23	2.01	1.97	2.06	1.97	2.00	2.18	2.14	2.47	2.82	2.64	3.01
1429.34	2.14	2.08	2.06	2.09	2.05	2.27	2.31	2.27	2.56	2.66	3.12
1540.54	2.15	1.87	2.00	2.06	2.07	2.00	2.31	2.31	2.42	2.57	2.94
1660.48	2.05	1.94	1.92	1.83	1.98	2.15	2.19	2.09	2.49	2.73	2.91
1789.83	1.77	2.00	1.78	1.68	1.84	1.96	2.26	1.98	2.41	2.28	2.58
1929.34	1.73	1.95	1.92	1.89	1.90	2.01	2.03	2.07	2.40	2.34	2.57
2079.80	1.74	1.67	1.74	1.69	1.59	1.67	1.76	1.93	2.22	2.32	2.34
2242.08	1.83	1.81	1.64	1.69	1.90	1.79	1.99	2.14	2.39	2.41	2.71
2417.10	1.62	1.58	1.68	1.76	1.75	1.75	1.80	2.10	2.14	2.32	2.77
2605.85	1.62	1.44	1.68	1.63	1.65	1.69	1.57	2.02	2.08	2.15	2.38
2809.43	1.63	1.57	1.75	1.58	1.48	1.57	1.92	2.03	2.05	1.79	2.23
3028.99	1.35	1.58	1.36	1.43	1.57	1.93	1.77	1.68	1.96	2.09	2.45
3265.79	1.61	1.67	1.56	1.61	1.56	1.62	1.70	1.69	1.98	2.11	2.39
3521.18	1.55	1.73	1.67	1.58	1.54	1.60	1.77	2.05	2.05	2.18	2.49
3796.62	1.46	1.55	1.52	1.50	1.52	1.75	1.61	2.09	1.77	1.92	2.23

Time (ps) \ Wavelength (nm)	413.67	415.08	416.49	417.89	419.30	420.71	422.12	423.53	424.94	426.35	427.75
-3.28	0.07	-0.11	-0.24	-0.03	-0.12	-0.15	-0.12	-0.08	-0.08	-0.18	-0.06
-2.78	-0.07	-0.03	-0.07	-0.18	0.01	-0.07	-0.09	0.03	-0.14	-0.11	-0.06
-2.28	-0.14	-0.03	0.07	-0.11	-0.16	-0.04	-0.08	-0.02	-0.04	-0.10	-0.07
-1.78	0.20	0.13	0.22	0.27	0.10	0.11	0.30	0.12	0.16	0.13	0.12
-1.28	-0.17	-0.09	-0.14	0.06	-0.01	0.05	0.04	-0.10	0.04	0.03	-0.10
-0.78	-0.18	-0.07	-0.07	-0.20	0.00	-0.11	-0.05	-0.10	0.00	-0.06	-0.05
-0.28	0.00	0.12	0.06	0.04	0.01	0.00	0.00	-0.03	-0.03	0.04	-0.04
0.22	0.02	0.10	0.12	-0.03	0.06	0.14	0.01	-0.03	0.14	0.06	0.20
0.32	0.11	-0.06	0.07	0.13	-0.07	-0.05	-0.01	0.06	-0.15	0.07	0.03
0.42	0.16	0.04	-0.02	0.05	0.17	0.12	-0.02	0.14	0.09	0.11	0.03
0.52	-0.11	0.05	-0.08	-0.05	0.02	0.04	-0.08	0.00	0.03	0.13	0.08
0.62	0.17	0.06	0.04	0.02	0.11	0.18	0.14	0.09	0.08	0.14	-0.09
0.72	0.09	0.22	0.06	0.12	0.11	0.17	-0.05	-0.02	0.01	-0.02	0.11
0.77	0.01	0.18	0.12	0.11	0.06	0.13	0.15	0.03	0.15	0.11	-0.08
0.82	-0.04	0.12	-0.13	-0.12	0.10	0.12	0.01	0.10	0.15	0.13	0.09
0.87	0.19	0.10	-0.06	0.04	0.04	0.02	0.14	0.06	-0.04	0.02	-0.04
0.92	-0.19	0.02	-0.25	-0.16	0.02	0.02	-0.01	0.03	-0.06	0.07	-0.04
0.97	0.11	0.21	0.13	0.14	0.04	0.06	0.08	0.04	0.02	-0.02	-0.01
1.02	-0.09	0.03	0.05	-0.09	-0.15	-0.13	-0.02	-0.02	-0.05	0.10	0.10
1.07	0.91	0.73	0.35	0.18	-0.13	-0.33	-0.33	-0.23	-0.08	-0.04	-0.14
1.12	4.54	4.21	3.78	3.38	2.73	1.97	1.09	0.45	0.04	-0.20	-0.38
1.17	6.41	6.52	6.73	6.92	7.04	6.90	6.21	5.73	4.88	4.11	3.28
1.22	6.37	6.27	6.70	6.83	7.07	7.19	7.13	6.79	6.68	6.57	6.13
1.27	6.11	6.16	6.44	6.75	6.86	6.99	7.07	7.23	7.18	7.24	7.20
1.32	5.92	6.09	6.15	6.42	6.51	6.38	6.33	6.47	6.48	6.47	6.39
1.37	5.59	5.90	5.84	5.96	5.94	5.94	6.08	6.08	6.05	5.93	5.98
1.42	5.46	5.51	5.66	5.68	5.85	5.84	5.88	5.75	5.82	5.83	5.84
1.47	5.25	5.42	5.61	5.65	5.68	5.88	5.74	5.72	5.66	5.77	5.74
1.52	5.11	5.35	5.63	5.52	5.63	5.59	5.74	5.60	5.49	5.59	5.59
1.57	5.19	5.31	5.50	5.51	5.47	5.53	5.65	5.50	5.50	5.53	5.47
1.62	4.93	5.27	5.49	5.51	5.63	5.55	5.61	5.69	5.60	5.58	5.59
1.67	5.11	5.40	5.55	5.60	5.58	5.64	5.62	5.53	5.57	5.60	5.47
1.72	4.98	5.27	5.32	5.49	5.52	5.51	5.56	5.57	5.53	5.56	5.41
1.77	4.87	5.02	5.16	5.25	5.36	5.35	5.32	5.41	5.44	5.40	5.34
1.82	4.67	4.89	4.93	5.18	5.08	5.30	5.16	5.20	5.10	5.13	5.18
1.87	4.59	4.77	4.89	5.12	5.04	5.23	5.15	5.19	5.30	5.27	5.10
1.92	4.59	5.09	5.14	5.31	5.25	5.31	5.32	5.38	5.37	5.38	5.20
1.97	4.73	4.94	5.14	5.33	5.27	5.20	5.25	5.30	5.33	5.31	5.32
2.02	4.56	4.94	5.04	5.12	4.97	5.30	5.20	5.19	5.17	5.32	5.05
2.07	4.60	4.88	4.79	5.02	5.07	5.22	5.16	5.05	5.10	5.14	5.07
2.12	4.77	5.14	5.30	5.30	5.39	5.56	5.49	5.57	5.53	5.59	5.50
2.17	4.70	5.02	5.02	5.12	5.08	5.32	5.38	5.42	5.39	5.34	5.33
2.22	4.62	4.72	4.92	4.96	5.09	5.06	5.14	5.08	5.06	5.15	5.02
2.27	4.54	4.91	5.13	5.05	5.22	5.06	5.27	5.26	5.23	5.36	5.21
2.32	4.58	4.51	4.69	4.99	4.94	4.99	5.11	5.08	5.14	5.12	4.99
2.37	4.92	4.97	4.99	5.24	5.27	5.36	5.30	5.37	5.23	5.41	5.25
2.42	4.59	4.93	4.83	5.15	5.14	5.22	5.30	5.35	5.23	5.36	5.21

2.47	4.41	4.39	4.73	4.83	4.83	5.04	5.07	5.02	5.01	5.18	4.97
2.52	4.51	4.78	4.74	5.03	5.18	5.15	5.13	5.18	5.25	5.23	5.09
2.57	4.36	4.52	4.59	4.88	4.84	5.05	4.90	4.99	5.00	4.94	5.03
2.62	4.42	4.64	4.67	4.90	4.94	5.05	4.88	5.00	5.07	5.13	5.17
2.67	4.20	4.34	4.77	4.68	4.88	5.01	4.86	4.93	4.99	4.94	5.02
2.72	4.45	4.80	4.80	4.94	4.99	5.05	5.15	5.01	5.09	5.06	5.08
2.77	4.81	5.01	5.20	5.35	5.21	5.35	5.46	5.39	5.42	5.39	5.30
2.82	4.34	4.56	4.92	4.94	4.88	5.05	5.07	5.17	5.07	5.26	5.16
2.87	4.22	4.53	4.72	4.73	4.89	5.02	4.88	4.93	4.93	5.16	4.90
2.92	4.40	4.43	4.73	4.86	4.97	5.00	4.95	5.07	5.09	5.03	4.99
2.97	4.27	4.69	4.80	4.90	4.94	4.93	4.96	5.07	5.10	5.05	5.06
3.02	4.67	4.93	4.94	5.10	5.10	5.14	5.27	5.27	5.32	5.22	5.23
3.07	4.67	4.73	4.93	5.14	5.10	5.11	5.05	5.05	5.19	5.20	5.09
3.12	4.68	4.72	4.88	5.10	5.19	5.25	5.30	5.22	5.31	5.22	5.22
3.17	4.57	4.73	4.85	4.99	5.14	5.16	5.19	5.26	5.30	5.39	5.21
3.22	4.54	4.67	4.67	4.95	5.10	5.01	5.07	5.06	5.03	5.16	4.96
3.27	4.53	4.74	4.85	4.99	5.18	5.09	5.21	5.18	5.18	5.40	5.18
3.32	4.84	4.71	5.08	5.12	5.12	5.28	5.48	5.35	5.37	5.31	5.26
3.37	4.40	4.69	4.64	4.74	4.75	5.01	4.91	4.89	4.93	4.95	4.89
3.42	4.66	4.75	4.87	5.14	5.23	5.32	5.26	5.32	5.34	5.38	5.29
3.47	4.34	4.49	4.74	4.90	4.89	5.16	5.18	5.21	5.08	5.14	5.12
3.52	4.30	4.56	4.76	4.96	4.94	4.91	5.12	5.34	5.12	5.06	4.99
3.57	4.00	4.62	4.47	4.84	4.77	4.96	4.95	5.16	5.10	4.97	5.01
3.62	4.40	4.63	4.72	4.91	4.95	5.07	4.98	5.13	5.15	5.14	4.91
3.67	4.31	4.51	4.63	4.77	4.81	4.97	4.91	4.97	5.03	5.13	4.88
3.72	4.52	4.57	4.79	4.95	4.92	4.95	4.97	4.97	5.04	5.07	5.06
3.92	4.50	4.55	4.86	5.04	5.01	5.22	5.21	5.13	5.14	5.11	5.03
4.12	4.47	4.64	4.71	5.02	5.03	5.10	5.12	5.19	5.06	5.34	5.19
4.32	4.27	4.48	4.50	4.74	4.77	4.76	4.86	4.90	5.02	4.93	4.82
4.52	4.48	4.64	4.84	5.02	5.06	5.11	5.12	5.15	5.14	5.02	5.01
4.72	4.31	4.55	4.74	4.71	4.88	4.96	5.06	4.89	4.84	4.98	4.92
4.92	4.43	4.59	4.80	5.09	5.08	5.10	5.11	5.21	5.25	5.35	5.04
5.12	4.31	4.56	4.61	4.83	4.90	5.08	5.06	5.03	5.11	5.12	5.01
5.32	4.62	4.93	4.98	5.20	5.20	5.27	5.32	5.28	5.28	5.29	5.18
5.52	4.50	4.84	4.95	5.08	5.16	5.17	5.25	5.22	5.25	5.29	5.07
5.72	4.59	4.69	4.90	5.13	5.27	5.35	5.28	5.29	5.34	5.35	5.30
5.92	4.43	4.52	4.82	4.86	4.80	5.06	5.04	5.21	5.02	4.97	5.01
6.12	4.40	4.61	4.79	4.95	5.15	5.15	5.09	5.10	5.21	5.22	5.03
6.32	4.11	4.36	4.52	4.78	4.92	5.06	5.02	4.92	4.97	4.94	4.84
6.52	4.53	4.53	4.70	5.05	4.94	5.12	5.12	4.98	5.24	5.10	5.06
6.72	4.75	4.86	5.11	5.19	5.32	5.41	5.40	5.33	5.28	5.41	5.25
6.92	4.49	4.56	4.73	4.96	4.89	5.03	5.09	4.93	5.05	5.03	4.96
7.12	4.49	4.78	4.99	4.86	4.98	5.16	5.14	5.07	5.22	5.11	4.98
7.32	4.38	4.67	4.90	5.01	4.97	5.15	5.13	5.24	5.22	5.22	5.03
7.52	4.58	4.58	4.61	4.92	4.93	5.01	5.02	5.07	4.93	4.91	4.94
7.72	4.51	4.57	4.91	5.06	5.16	5.20	5.20	5.16	5.18	5.13	4.94
7.92	4.46	4.74	4.60	4.85	4.97	4.89	5.05	5.08	5.12	5.10	4.96
8.12	4.50	4.72	4.90	5.03	5.18	5.25	5.17	5.19	5.24	5.26	5.09
8.32	4.58	4.62	4.83	4.95	5.02	5.10	5.16	5.05	5.18	5.16	5.00
8.52	4.12	4.43	4.46	4.53	4.62	4.73	4.83	4.78	4.80	4.86	4.73

8.72	4.45	4.56	4.54	5.05	5.11	5.09	5.14	5.19	5.12	5.18	5.04
8.92	4.34	4.44	4.68	4.94	5.12	5.07	5.09	5.09	4.97	5.06	5.01
9.12	4.32	4.77	4.74	4.91	5.11	5.11	5.19	5.13	5.12	5.16	5.04
9.32	4.11	4.28	4.64	4.80	4.91	4.81	4.94	4.99	4.85	4.85	4.67
9.52	4.26	4.59	4.83	4.96	4.88	5.10	4.97	5.12	5.02	5.14	4.90
9.72	4.25	4.66	4.71	4.84	4.75	4.84	4.99	5.04	4.94	4.88	4.72
9.92	4.56	4.73	4.65	4.93	5.09	5.12	5.20	4.89	5.05	5.01	4.78
10.12	4.41	4.69	4.98	5.23	5.20	5.48	5.44	5.34	5.21	5.25	5.13
10.32	4.23	4.34	4.49	4.69	4.85	4.86	4.93	4.90	4.86	4.80	4.71
10.52	4.49	4.77	4.76	5.02	5.02	5.06	5.06	5.11	4.94	4.97	4.99
10.72	4.26	4.68	4.74	4.82	4.96	5.09	4.99	5.14	5.07	4.95	4.78
10.92	4.28	4.38	4.45	4.81	4.74	4.75	4.88	4.89	4.87	4.96	4.65
11.12	4.36	4.54	4.79	4.97	4.97	5.06	5.02	4.91	5.05	4.85	4.74
11.32	4.50	4.73	4.92	5.07	5.19	5.21	5.11	5.25	5.13	5.15	4.99
11.52	4.30	4.57	4.60	4.78	4.84	4.90	4.93	4.92	4.88	4.92	4.77
11.72	4.51	4.70	4.89	4.90	5.02	5.12	4.94	5.17	5.07	5.01	4.88
11.92	4.41	4.35	4.69	4.77	4.96	4.87	4.91	4.98	4.96	4.87	4.77
12.12	4.46	4.85	4.97	5.07	5.22	5.18	5.24	5.22	5.15	5.17	5.02
12.32	4.43	4.81	4.77	5.07	5.15	5.18	5.21	5.28	5.18	5.14	5.03
12.52	4.34	4.55	4.72	4.94	5.02	5.12	4.99	4.95	5.05	5.05	4.92
12.72	4.46	4.65	4.99	5.00	4.97	5.09	5.06	5.12	5.06	5.07	4.91
12.92	4.37	4.70	4.75	5.03	5.00	5.10	5.14	5.16	4.97	5.03	4.97
13.12	4.21	4.57	4.55	4.60	4.73	4.79	4.63	4.80	4.71	4.63	4.44
13.32	4.23	4.73	4.68	5.05	5.01	5.19	5.05	5.07	5.05	5.22	4.83
13.52	4.25	4.43	4.68	4.76	4.78	4.84	4.72	4.80	4.90	4.77	4.72
13.72	4.52	4.73	4.93	5.19	5.12	5.23	5.29	5.18	5.25	5.16	5.04
13.92	4.31	4.69	4.73	4.94	4.99	5.01	5.06	5.03	4.97	5.02	4.84
14.00	4.61	4.83	4.90	5.05	5.19	5.25	5.27	5.30	5.22	5.10	5.07
14.08	4.48	4.62	4.75	4.89	4.77	4.94	4.88	4.94	4.84	4.92	4.89
14.17	4.09	4.31	4.63	4.85	4.81	5.05	5.07	4.96	4.86	4.99	4.69
14.27	4.25	4.57	4.70	4.78	4.94	5.00	4.98	5.02	4.98	4.89	4.77
14.38	4.19	4.43	4.64	4.79	4.88	5.06	4.98	4.97	4.95	4.89	4.78
14.49	4.33	4.40	4.67	4.91	4.86	4.89	4.87	4.84	4.98	4.89	4.80
14.61	4.29	4.40	4.54	4.88	4.86	4.79	4.85	4.82	4.85	4.86	4.85
14.75	4.25	4.30	4.52	4.66	4.78	4.81	4.81	4.89	4.76	4.86	4.55
14.89	4.78	5.07	5.19	5.31	5.36	5.49	5.38	5.28	5.27	5.31	5.14
15.05	4.34	4.45	4.63	5.02	5.06	5.01	4.93	4.95	4.98	5.13	4.85
15.21	4.60	4.75	4.85	5.01	5.22	5.29	5.05	5.10	5.19	5.09	4.97
15.39	4.35	4.62	4.74	4.83	4.92	4.93	4.94	4.88	4.88	4.85	4.79
15.59	4.36	4.45	4.62	4.83	4.88	4.94	4.91	4.91	4.90	4.92	4.71
15.80	4.45	4.75	4.93	5.08	5.20	5.29	5.10	5.07	5.06	5.03	4.91
16.02	4.18	4.36	4.67	4.74	4.78	4.83	5.00	4.77	4.88	4.85	4.64
16.27	4.61	4.80	4.83	5.11	5.12	5.17	5.28	5.14	5.06	5.24	5.08
16.53	4.30	4.52	4.68	4.80	4.92	4.98	5.03	5.04	4.96	4.89	4.82
16.81	4.08	4.45	4.55	4.73	4.75	4.78	4.80	4.82	4.84	4.64	4.64
17.12	4.48	4.71	4.78	4.96	4.98	5.02	5.04	5.13	5.08	5.08	4.80
17.45	3.96	4.25	4.56	4.69	4.64	4.77	4.74	4.63	4.76	4.85	4.57
17.81	4.43	4.83	4.96	5.15	5.16	5.34	5.27	5.21	5.13	4.99	4.96
18.19	4.47	4.75	4.83	4.95	5.15	5.18	5.24	5.02	5.12	5.12	4.93
18.60	4.61	4.84	5.02	5.18	5.30	5.36	5.34	5.20	5.21	5.06	5.05

19.05	4.46	4.78	4.88	5.05	5.20	5.10	5.00	5.04	4.95	4.88	4.88
19.53	4.39	4.70	4.76	4.92	5.01	5.01	5.02	4.93	4.94	4.93	4.78
20.05	4.42	4.65	4.77	5.03	5.06	5.09	5.03	5.08	4.94	5.01	4.89
20.61	4.56	4.70	4.74	5.00	5.20	5.04	5.18	5.10	5.12	4.95	4.93
21.22	4.46	4.56	4.72	4.81	4.96	4.99	5.05	5.04	4.93	4.91	4.85
21.87	4.44	4.76	4.89	5.02	5.07	5.11	5.09	4.90	4.88	5.07	4.70
22.57	4.44	4.76	4.93	5.10	5.02	5.05	5.05	5.13	5.00	5.07	4.83
23.33	4.46	4.59	4.77	5.05	5.13	5.02	5.14	5.14	5.10	5.14	4.85
24.15	4.41	4.60	4.53	4.91	4.97	4.95	4.98	4.87	4.86	4.76	4.80
25.03	4.26	4.29	4.60	4.82	4.87	4.98	4.93	4.85	4.87	4.69	4.74
25.98	4.37	4.72	4.88	5.10	5.06	5.12	5.16	5.16	4.99	4.98	4.90
27.01	4.45	4.53	4.64	4.80	4.95	5.00	5.10	5.04	4.90	4.87	4.77
28.11	4.44	4.68	4.77	5.11	4.95	5.16	5.17	5.17	4.97	4.94	4.66
29.30	4.43	4.48	4.76	5.00	4.95	4.98	5.03	4.99	4.84	4.83	4.64
30.59	4.27	4.57	4.66	5.06	5.06	5.06	4.99	4.95	4.91	4.86	4.73
31.98	4.35	4.46	4.67	4.75	4.86	4.91	4.85	4.84	4.90	4.79	4.63
33.47	4.14	4.56	4.62	4.97	4.88	4.95	4.85	4.90	4.92	4.85	4.64
35.09	4.43	4.48	4.79	5.01	4.99	5.12	5.15	5.11	4.97	4.89	4.70
36.83	4.22	4.52	4.55	4.80	4.86	4.83	4.86	4.87	4.83	4.68	4.53
38.71	4.21	4.32	4.74	4.83	4.90	4.76	4.71	4.73	4.72	4.65	4.58
40.73	4.40	4.67	4.66	4.88	5.02	5.05	5.07	4.99	4.87	4.97	4.76
42.92	4.22	4.62	4.55	4.81	4.80	4.99	4.92	4.90	4.90	4.78	4.64
45.27	4.29	4.67	4.83	4.95	4.90	5.06	4.93	4.94	4.80	4.74	4.66
47.81	4.32	4.58	4.85	5.03	4.98	5.01	4.85	4.95	4.78	4.74	4.77
50.55	3.91	4.12	4.46	4.46	4.62	4.62	4.63	4.38	4.40	4.48	4.21
53.51	4.46	4.65	4.77	4.88	5.03	4.99	4.94	4.92	4.79	4.85	4.64
56.69	4.55	4.58	4.95	5.05	4.91	4.98	5.09	4.80	4.79	4.71	4.63
60.13	4.29	4.53	4.74	4.95	4.75	4.79	4.91	4.69	4.74	4.67	4.38
63.84	4.35	4.69	4.77	4.85	5.02	5.08	5.06	4.95	4.68	4.73	4.64
67.83	4.29	4.50	4.67	4.92	4.88	4.86	4.71	4.71	4.77	4.66	4.33
72.15	4.31	4.51	4.51	4.78	4.73	4.77	4.80	4.74	4.73	4.58	4.43
76.80	4.08	4.38	4.68	4.72	4.75	4.85	4.74	4.78	4.65	4.55	4.41
81.81	4.13	4.59	4.62	4.86	4.92	5.04	4.97	4.76	4.87	4.78	4.57
87.22	3.95	4.21	4.43	4.57	4.72	4.77	4.70	4.63	4.61	4.53	4.29
93.06	4.00	4.35	4.39	4.61	4.85	4.76	4.76	4.56	4.72	4.57	4.49
99.35	4.32	4.50	4.53	4.76	4.83	4.86	4.90	4.73	4.68	4.78	4.49
106.13	4.18	4.41	4.48	4.75	4.71	4.78	4.62	4.68	4.72	4.43	4.30
113.45	4.39	4.54	4.83	4.90	4.88	5.04	4.98	4.95	4.88	4.73	4.51
121.34	4.19	4.25	4.53	4.61	4.73	4.81	4.82	4.68	4.56	4.54	4.34
129.86	3.92	4.07	4.28	4.52	4.59	4.53	4.56	4.39	4.40	4.35	4.07
139.04	3.95	4.19	4.30	4.53	4.60	4.58	4.52	4.54	4.61	4.32	4.25
148.94	3.85	4.08	4.35	4.35	4.38	4.42	4.43	4.36	4.22	4.30	4.01
159.62	3.91	4.21	4.34	4.62	4.42	4.61	4.53	4.64	4.33	4.39	4.23
171.14	4.07	4.30	4.43	4.62	4.68	4.67	4.72	4.62	4.56	4.51	4.31
183.56	3.74	4.06	4.24	4.32	4.26	4.32	4.40	4.20	4.29	4.23	4.08
196.96	4.03	4.29	4.50	4.63	4.61	4.78	4.87	4.54	4.56	4.44	4.26
211.41	4.00	4.13	4.32	4.54	4.54	4.50	4.58	4.54	4.31	4.40	4.18
226.99	3.89	4.17	4.33	4.70	4.49	4.55	4.47	4.55	4.53	4.46	4.21
243.80	3.70	3.90	4.05	4.28	4.33	4.32	4.21	4.06	4.18	4.15	3.89
261.93	3.99	4.18	4.43	4.60	4.47	4.57	4.56	4.60	4.48	4.42	4.14

281.48	4.10	4.22	4.40	4.59	4.47	4.67	4.60	4.46	4.42	4.28	4.13
302.56	3.79	3.87	3.98	4.11	4.39	4.20	4.21	4.26	4.17	4.04	3.92
325.30	3.61	4.00	4.07	4.45	4.39	4.43	4.45	4.38	4.20	4.31	4.04
349.83	3.48	3.79	3.87	4.14	4.24	4.23	4.15	4.27	4.13	4.05	3.84
376.28	3.79	4.04	4.05	4.35	4.24	4.31	4.36	4.24	4.12	4.08	3.98
404.81	3.88	4.11	4.26	4.46	4.53	4.32	4.37	4.25	4.15	4.17	3.93
435.58	3.66	3.71	4.03	4.18	4.31	4.22	4.24	4.18	4.13	4.12	3.83
468.76	3.76	3.78	3.87	4.12	4.23	4.05	4.21	4.12	3.98	3.96	3.81
504.55	3.45	3.67	3.66	3.93	3.89	4.03	3.93	4.03	3.76	3.86	3.66
543.15	3.54	3.75	4.00	4.20	4.19	4.05	4.07	3.97	4.01	3.79	3.48
584.78	3.27	3.60	3.81	3.88	3.80	4.01	3.89	3.67	3.81	3.66	3.49
629.68	3.38	3.56	3.63	3.88	3.93	4.01	3.85	3.86	3.76	3.68	3.61
678.10	3.53	3.74	3.91	4.03	4.08	4.19	4.05	4.06	3.89	3.82	3.64
730.33	3.59	3.90	3.89	4.15	4.12	4.14	4.08	4.03	3.98	3.97	3.83
786.65	3.38	3.66	3.66	3.89	3.89	3.86	3.89	3.73	3.68	3.63	3.51
847.40	3.31	3.56	3.72	3.83	3.83	4.00	3.89	3.71	3.77	3.61	3.43
912.92	3.30	3.61	3.80	3.98	4.08	3.96	3.98	3.87	3.83	3.60	3.55
983.58	3.27	3.67	3.89	4.11	4.08	4.05	3.93	3.88	3.71	3.84	3.60
1059.78	3.22	3.40	3.78	3.91	3.99	3.87	3.85	3.91	3.63	3.76	3.54
1141.98	3.16	3.29	3.38	3.78	3.66	3.53	3.61	3.61	3.51	3.50	3.26
1230.62	3.22	3.30	3.25	3.50	3.67	3.54	3.43	3.49	3.26	3.28	3.08
1326.23	3.16	3.54	3.53	3.74	3.85	3.79	3.62	3.67	3.55	3.37	3.39
1429.34	3.37	3.39	3.66	3.76	3.82	3.95	3.83	3.82	3.79	3.70	3.33
1540.54	3.14	3.26	3.48	3.67	3.74	3.81	3.74	3.57	3.49	3.43	3.38
1660.48	2.90	3.36	3.41	3.68	3.66	3.81	3.69	3.58	3.46	3.41	3.21
1789.83	2.90	3.16	3.38	3.44	3.48	3.72	3.54	3.26	3.39	3.38	3.17
1929.34	3.12	3.25	3.28	3.58	3.47	3.59	3.58	3.43	3.55	3.42	3.17
2079.80	2.82	3.24	3.28	3.41	3.48	3.53	3.45	3.48	3.39	3.18	3.00
2242.08	2.80	2.99	3.23	3.52	3.48	3.40	3.45	3.36	3.18	3.14	3.02
2417.10	2.83	3.14	3.18	3.52	3.37	3.59	3.40	3.37	3.35	3.21	2.95
2605.85	2.76	3.13	3.24	3.36	3.43	3.55	3.34	3.36	3.21	3.23	2.87
2809.43	2.52	2.73	2.92	3.06	3.07	3.10	2.97	2.98	2.80	2.90	2.68
3028.99	2.50	2.87	3.02	3.12	3.28	3.13	3.22	3.07	3.03	2.96	2.82
3265.79	2.65	2.84	2.88	3.12	3.17	3.25	3.19	3.04	3.02	3.02	2.81
3521.18	2.65	2.96	3.10	3.23	3.38	3.20	3.25	3.09	3.05	3.07	2.91
3796.62	2.61	2.70	2.85	2.98	2.99	3.10	3.02	2.85	2.78	2.86	2.45

Wavelength (nm) / Time (ps)	429.16	430.57	431.98	433.39	434.80	436.20	437.61	439.02	440.43	441.84	443.25
-3.28	-0.18	-0.08	-0.12	-0.24	-0.16	-0.05	-0.16	-0.07	-0.19	-0.13	-0.02
-2.78	-0.13	-0.09	-0.14	-0.05	-0.13	-0.06	-0.01	-0.09	-0.18	-0.08	-0.02
-2.28	-0.03	-0.01	0.03	-0.02	0.02	-0.02	-0.13	-0.07	0.03	0.02	-0.01
-1.78	0.10	0.15	0.07	0.20	0.03	0.16	0.12	0.07	0.17	0.03	0.05
-1.28	-0.03	0.00	0.01	0.08	0.01	-0.02	-0.02	0.04	-0.05	0.07	0.03
-0.78	-0.12	-0.02	-0.12	-0.07	0.02	0.03	-0.10	-0.13	-0.12	-0.12	-0.06
-0.28	0.01	-0.10	-0.06	-0.04	0.02	-0.05	-0.07	0.02	-0.01	0.01	-0.02
0.22	0.12	0.07	0.25	0.07	0.20	-0.03	0.17	0.07	0.14	0.17	0.03
0.32	0.01	-0.03	0.04	-0.10	-0.04	0.05	0.03	-0.01	0.00	-0.04	-0.02
0.42	0.25	0.11	0.05	0.17	0.01	-0.01	0.18	0.17	0.21	0.07	0.04

0.52	0.05	0.02	-0.01	0.05	0.06	-0.06	-0.05	-0.10	-0.01	0.00	-0.05
0.62	0.04	0.01	0.10	0.09	-0.09	-0.02	0.03	-0.06	0.08	0.03	-0.02
0.72	0.06	0.09	0.25	0.17	0.20	0.27	0.20	0.02	0.18	0.23	0.22
0.77	0.10	0.02	0.01	0.14	0.06	0.04	-0.01	0.14	-0.02	-0.02	0.02
0.82	0.12	0.12	0.00	0.01	0.02	0.00	-0.09	-0.01	0.02	0.04	0.19
0.87	0.01	0.02	0.12	0.02	-0.02	-0.08	-0.06	-0.03	-0.04	0.04	-0.03
0.92	0.09	0.06	-0.01	0.04	0.08	0.11	-0.06	0.06	0.09	-0.01	-0.19
0.97	0.01	-0.11	0.11	0.00	0.00	-0.13	-0.18	-0.04	-0.05	-0.02	-0.03
1.02	0.04	-0.05	-0.01	-0.10	-0.11	-0.12	-0.02	-0.11	-0.08	-0.09	-0.03
1.07	0.02	-0.05	-0.04	-0.01	-0.11	-0.01	-0.05	-0.06	-0.10	0.07	0.01
1.12	-0.39	-0.51	-0.45	-0.48	-0.32	-0.26	-0.21	-0.22	-0.25	-0.09	-0.22
1.17	2.51	1.72	1.26	0.51	0.24	-0.17	-0.28	-0.44	-0.41	-0.47	-0.33
1.22	6.01	5.59	5.38	4.96	4.50	4.22	3.64	3.19	2.61	2.41	1.76
1.27	7.37	7.26	7.32	7.20	7.09	6.96	6.76	6.54	6.30	6.01	5.63
1.32	6.38	6.57	6.49	6.39	6.35	6.49	6.43	6.44	6.43	6.51	6.29
1.37	5.88	5.94	5.83	5.72	5.74	5.72	5.62	5.46	5.38	5.31	5.20
1.42	5.86	5.67	5.75	5.75	5.67	5.48	5.33	5.27	5.19	5.14	5.13
1.47	5.68	5.57	5.58	5.44	5.30	5.21	5.23	5.05	4.92	4.83	4.81
1.52	5.52	5.45	5.37	5.15	5.13	5.04	4.90	4.84	4.67	4.59	4.45
1.57	5.41	5.29	5.17	5.20	5.16	4.94	4.87	4.70	4.65	4.58	4.44
1.62	5.52	5.52	5.41	5.23	5.13	5.01	4.95	4.83	4.69	4.55	4.39
1.67	5.46	5.43	5.38	5.20	5.14	5.12	4.89	4.81	4.72	4.67	4.47
1.72	5.29	5.34	5.24	5.17	5.11	4.93	4.79	4.71	4.64	4.41	4.37
1.77	5.32	5.39	5.19	5.17	5.03	4.85	4.87	4.56	4.56	4.29	4.17
1.82	5.12	5.03	5.05	4.84	4.92	4.81	4.62	4.50	4.43	4.12	4.08
1.87	5.14	5.03	5.05	4.90	4.80	4.73	4.65	4.49	4.42	4.27	4.07
1.92	5.20	5.22	5.28	4.96	4.93	4.72	4.80	4.48	4.48	4.23	4.12
1.97	5.10	5.13	5.08	4.98	4.76	4.73	4.69	4.54	4.25	4.13	4.17
2.02	5.22	5.15	4.99	4.88	4.94	4.88	4.58	4.38	4.29	4.37	4.06
2.07	5.02	4.87	4.88	4.80	4.79	4.59	4.32	4.24	4.17	3.95	4.04
2.12	5.40	5.36	5.31	5.13	5.21	5.02	4.78	4.74	4.62	4.36	4.31
2.17	5.34	5.13	5.05	4.89	5.00	4.78	4.66	4.43	4.42	4.37	4.15
2.22	5.11	4.98	4.81	4.73	4.72	4.64	4.47	4.29	4.22	3.97	4.01
2.27	5.15	5.17	5.05	4.90	4.88	4.85	4.70	4.56	4.38	4.30	4.20
2.32	5.04	5.02	5.02	4.80	4.73	4.52	4.51	4.42	4.23	4.05	4.02
2.37	5.31	5.18	5.04	4.99	4.84	4.68	4.59	4.46	4.35	4.18	4.17
2.42	5.17	5.04	5.11	5.07	5.00	4.83	4.48	4.46	4.28	4.15	4.07
2.47	4.99	4.93	4.79	4.68	4.56	4.45	4.28	4.16	4.02	4.03	3.79
2.52	5.09	4.97	4.97	4.80	4.67	4.56	4.50	4.19	4.21	3.98	3.94
2.57	4.91	4.85	4.75	4.62	4.58	4.34	4.26	4.08	4.00	3.82	3.68
2.62	5.06	4.88	4.89	4.76	4.75	4.44	4.46	4.27	4.27	4.02	3.87
2.67	4.96	4.82	4.71	4.54	4.61	4.35	4.26	4.01	4.07	3.79	3.66
2.72	4.82	4.84	4.81	4.68	4.55	4.42	4.38	4.21	4.07	4.07	3.78
2.77	5.29	5.24	5.26	5.03	4.85	4.67	4.56	4.45	4.29	4.23	4.14
2.82	5.13	4.95	4.97	4.85	4.60	4.47	4.45	4.34	4.06	3.98	3.87
2.87	4.97	5.00	4.73	4.69	4.58	4.47	4.31	4.08	4.01	3.92	3.72
2.92	5.03	4.79	4.98	4.74	4.63	4.58	4.30	4.13	4.16	4.08	3.87
2.97	4.97	4.88	4.78	4.74	4.68	4.53	4.36	4.23	3.94	3.94	3.77
3.02	5.13	5.08	5.04	4.76	4.72	4.63	4.49	4.33	4.16	4.06	3.90
3.07	5.03	5.05	4.99	4.75	4.66	4.54	4.48	4.21	4.05	4.11	3.89

3.12	5.17	5.16	5.00	4.94	4.83	4.74	4.66	4.37	4.35	4.19	4.00
3.17	5.23	5.12	5.05	4.91	4.79	4.61	4.48	4.25	4.19	4.03	3.85
3.22	5.14	4.99	4.97	4.74	4.70	4.44	4.17	4.22	4.07	4.02	3.86
3.27	5.02	5.00	4.86	4.87	4.72	4.62	4.43	4.40	4.15	4.01	3.92
3.32	5.27	5.11	5.08	5.08	4.80	4.83	4.58	4.42	4.17	4.28	3.98
3.37	4.71	4.73	4.67	4.58	4.46	4.36	4.03	3.96	3.94	3.80	3.74
3.42	5.26	5.03	5.13	5.02	4.80	4.73	4.43	4.31	4.15	4.08	3.86
3.47	4.98	4.95	4.99	4.70	4.65	4.49	4.33	4.28	4.05	3.94	3.87
3.52	5.12	4.94	4.93	4.95	4.73	4.52	4.41	4.24	4.18	3.97	3.83
3.57	4.94	4.81	4.86	4.74	4.44	4.49	4.25	4.06	3.94	3.93	3.76
3.62	5.03	5.00	4.89	4.78	4.71	4.45	4.21	4.21	4.04	3.83	3.73
3.67	4.78	4.76	4.74	4.59	4.52	4.20	4.20	3.96	3.88	3.72	3.55
3.72	5.07	4.92	4.92	4.81	4.56	4.48	4.16	4.21	4.04	3.89	3.80
3.92	5.12	4.99	4.84	4.76	4.74	4.55	4.33	4.28	4.02	3.91	3.70
4.12	4.90	4.93	4.88	4.66	4.68	4.59	4.35	4.22	4.06	3.93	3.68
4.32	4.86	4.73	4.71	4.52	4.36	4.26	4.11	3.98	3.85	3.80	3.58
4.52	4.91	4.89	4.80	4.58	4.42	4.46	4.19	4.03	3.87	3.72	3.68
4.72	4.97	4.83	4.71	4.59	4.49	4.41	4.20	3.94	3.97	3.70	3.48
4.92	5.19	5.08	5.05	4.82	4.65	4.44	4.33	4.19	4.04	3.88	3.73
5.12	4.94	4.88	4.80	4.76	4.54	4.45	4.18	3.96	3.84	3.74	3.58
5.32	5.14	5.01	4.85	4.72	4.56	4.53	4.23	4.20	3.99	3.77	3.83
5.52	5.05	4.98	4.80	4.79	4.52	4.47	4.30	4.08	4.03	3.92	3.52
5.72	5.28	5.09	5.08	4.79	4.65	4.59	4.37	4.21	4.18	3.92	3.78
5.92	4.80	4.73	4.71	4.55	4.41	4.12	4.10	3.90	3.69	3.74	3.54
6.12	4.95	4.99	4.83	4.63	4.54	4.27	4.11	3.99	3.82	3.72	3.62
6.32	4.84	4.72	4.54	4.46	4.32	4.05	3.90	3.79	3.65	3.55	3.23
6.52	4.92	4.95	4.74	4.51	4.40	4.31	4.06	4.02	3.82	3.66	3.45
6.72	5.23	4.93	4.91	4.73	4.64	4.40	4.36	4.09	3.90	3.77	3.85
6.92	4.86	4.77	4.74	4.47	4.41	4.25	4.10	3.93	3.81	3.68	3.45
7.12	4.96	4.80	4.59	4.56	4.49	4.30	4.05	3.99	3.82	3.59	3.37
7.32	5.09	4.93	4.83	4.55	4.52	4.44	4.06	3.95	3.91	3.72	3.53
7.52	4.88	4.77	4.63	4.59	4.47	4.27	3.95	3.82	3.77	3.66	3.47
7.72	5.00	4.84	4.63	4.67	4.42	4.23	4.06	3.97	3.83	3.71	3.52
7.92	4.84	4.78	4.64	4.41	4.45	4.24	4.06	3.89	3.75	3.59	3.35
8.12	5.03	4.89	4.84	4.66	4.52	4.31	4.10	3.99	3.95	3.80	3.60
8.32	4.94	4.84	4.68	4.53	4.44	4.31	4.23	3.99	3.74	3.49	3.49
8.52	4.70	4.53	4.47	4.25	4.18	4.07	3.86	3.65	3.59	3.50	3.15
8.72	5.01	4.90	4.80	4.64	4.39	4.27	4.10	3.88	3.76	3.72	3.52
8.92	4.75	4.76	4.70	4.57	4.39	4.10	4.00	3.84	3.77	3.48	3.33
9.12	5.01	4.98	4.51	4.54	4.42	4.25	4.10	4.01	3.74	3.67	3.45
9.32	4.56	4.57	4.27	4.30	4.23	3.90	3.79	3.59	3.52	3.35	3.24
9.52	4.83	4.77	4.66	4.48	4.23	4.08	4.13	3.80	3.68	3.55	3.34
9.72	4.85	4.71	4.48	4.37	4.27	4.06	3.92	3.74	3.58	3.44	3.19
9.92	4.73	4.71	4.55	4.43	4.27	4.13	3.95	3.86	3.59	3.35	3.26
10.12	5.00	4.87	4.74	4.50	4.48	4.23	4.09	3.86	3.73	3.60	3.29
10.32	4.55	4.49	4.35	4.21	4.09	3.90	3.72	3.53	3.43	3.23	3.07
10.52	4.83	4.67	4.64	4.47	4.26	4.05	3.98	3.67	3.52	3.44	3.37
10.72	4.83	4.70	4.54	4.42	4.39	4.13	3.84	3.68	3.57	3.48	3.35
10.92	4.62	4.49	4.34	4.25	4.08	3.98	3.74	3.72	3.52	3.37	3.20
11.12	4.71	4.53	4.44	4.36	4.10	4.07	3.84	3.76	3.64	3.38	3.22

11.32	4.95	4.81	4.64	4.62	4.44	4.36	3.99	3.84	3.81	3.63	3.49
11.52	4.57	4.55	4.46	4.23	4.32	4.05	3.81	3.85	3.63	3.42	3.22
11.72	4.80	4.60	4.53	4.39	4.19	4.17	3.79	3.59	3.56	3.44	3.22
11.92	4.70	4.59	4.42	4.26	4.04	4.09	3.84	3.53	3.47	3.25	3.14
12.12	4.95	4.74	4.62	4.48	4.32	4.26	4.02	3.80	3.69	3.57	3.35
12.32	5.02	4.84	4.74	4.51	4.39	4.15	3.93	3.80	3.64	3.48	3.26
12.52	4.74	4.54	4.64	4.25	4.11	3.87	3.89	3.79	3.49	3.28	3.23
12.72	4.89	4.67	4.72	4.49	4.25	4.16	4.04	3.67	3.70	3.59	3.31
12.92	4.76	4.59	4.57	4.41	4.21	4.05	3.84	3.59	3.48	3.42	3.24
13.12	4.61	4.18	4.17	4.12	4.01	3.77	3.65	3.47	3.45	3.29	3.18
13.32	4.82	4.59	4.42	4.42	4.31	4.04	3.88	3.77	3.49	3.33	3.26
13.52	4.53	4.41	4.42	4.19	3.95	3.94	3.66	3.58	3.46	3.23	3.17
13.72	4.89	4.80	4.68	4.39	4.30	4.16	3.97	3.76	3.50	3.57	3.29
13.92	4.81	4.52	4.47	4.36	4.29	3.94	3.81	3.61	3.64	3.40	3.33
14.00	4.94	4.75	4.70	4.55	4.32	4.18	4.03	3.76	3.63	3.61	3.36
14.08	4.60	4.65	4.46	4.29	4.10	3.95	3.76	3.57	3.57	3.29	3.14
14.17	4.79	4.50	4.42	4.29	4.04	3.92	3.71	3.52	3.50	3.29	3.15
14.27	4.71	4.60	4.44	4.38	3.98	3.99	3.73	3.64	3.46	3.32	3.19
14.38	4.70	4.59	4.44	4.27	4.19	4.02	3.69	3.76	3.59	3.23	3.18
14.49	4.66	4.47	4.36	4.23	4.10	3.90	3.67	3.48	3.34	3.26	3.17
14.61	4.53	4.49	4.30	4.22	4.08	3.90	3.72	3.51	3.31	3.19	3.09
14.75	4.45	4.36	4.19	4.12	4.05	3.99	3.70	3.60	3.38	3.31	3.01
14.89	5.15	5.00	4.75	4.70	4.41	4.39	4.15	3.83	3.69	3.46	3.34
15.05	4.75	4.70	4.41	4.27	4.10	4.02	3.75	3.56	3.42	3.44	3.04
15.21	4.84	4.69	4.49	4.40	4.34	4.18	3.96	3.88	3.56	3.42	3.26
15.39	4.54	4.56	4.30	4.31	4.05	3.96	3.79	3.54	3.36	3.32	3.12
15.59	4.63	4.62	4.30	4.35	3.93	3.74	3.65	3.60	3.39	3.21	3.15
15.80	4.74	4.67	4.64	4.36	4.24	4.00	3.94	3.67	3.59	3.40	3.18
16.02	4.63	4.32	4.38	4.16	3.89	3.88	3.69	3.55	3.30	3.24	3.00
16.27	4.81	4.67	4.54	4.34	4.28	4.06	3.88	3.85	3.64	3.50	3.34
16.53	4.68	4.57	4.39	4.27	4.13	3.84	3.63	3.71	3.46	3.24	3.22
16.81	4.53	4.52	4.41	4.29	4.01	3.99	3.66	3.62	3.38	3.21	2.96
17.12	4.74	4.72	4.55	4.23	4.05	3.95	3.75	3.71	3.47	3.36	3.07
17.45	4.55	4.29	4.19	3.97	3.89	3.56	3.43	3.30	3.14	3.01	2.99
17.81	4.85	4.74	4.55	4.40	4.21	4.11	3.80	3.77	3.55	3.35	3.15
18.19	4.96	4.66	4.54	4.38	4.19	4.12	3.82	3.78	3.66	3.34	3.25
18.60	4.94	4.75	4.61	4.49	4.34	4.20	3.95	3.83	3.66	3.40	3.33
19.05	4.77	4.55	4.62	4.39	4.20	4.05	3.83	3.71	3.52	3.34	3.17
19.53	4.66	4.50	4.31	4.23	4.09	3.85	3.65	3.48	3.33	3.17	3.04
20.05	4.83	4.53	4.52	4.28	4.15	3.91	3.80	3.59	3.42	3.39	3.05
20.61	4.72	4.63	4.57	4.39	4.31	3.89	3.85	3.64	3.61	3.27	3.21
21.22	4.72	4.59	4.27	4.25	4.10	3.87	3.75	3.55	3.56	3.23	3.10
21.87	4.71	4.48	4.38	4.33	4.07	3.94	3.69	3.60	3.42	3.31	3.05
22.57	4.76	4.55	4.45	4.20	4.09	3.98	3.75	3.57	3.47	3.22	2.97
23.33	4.76	4.57	4.47	4.37	4.25	3.98	3.75	3.69	3.41	3.26	3.08
24.15	4.71	4.52	4.35	4.24	4.14	3.88	3.84	3.49	3.33	3.24	2.97
25.03	4.59	4.40	4.27	4.18	3.88	3.84	3.54	3.39	3.25	3.09	2.90
25.98	4.73	4.62	4.44	4.26	4.09	3.85	3.74	3.55	3.32	3.20	3.11
27.01	4.62	4.65	4.26	4.15	4.00	3.85	3.63	3.45	3.41	3.30	3.03
28.11	4.54	4.50	4.47	4.29	4.09	4.00	3.75	3.55	3.39	3.15	3.07

29.30	4.50	4.47	4.49	4.18	3.99	3.92	3.68	3.51	3.31	3.18	3.06
30.59	4.66	4.39	4.34	4.20	4.08	3.74	3.68	3.52	3.26	3.19	3.01
31.98	4.58	4.36	4.23	4.13	3.98	3.80	3.52	3.26	3.25	3.06	2.86
33.47	4.63	4.37	4.33	4.07	3.91	3.79	3.63	3.53	3.34	3.08	2.95
35.09	4.59	4.50	4.33	4.17	3.88	3.82	3.56	3.50	3.26	3.03	3.00
36.83	4.52	4.34	4.09	4.01	3.87	3.66	3.61	3.44	3.29	3.13	2.96
38.71	4.38	4.36	4.25	4.11	3.77	3.71	3.51	3.36	3.22	3.01	2.68
40.73	4.56	4.34	4.18	4.10	3.94	3.73	3.55	3.31	3.26	3.07	2.92
42.92	4.53	4.44	4.37	4.13	4.06	3.78	3.62	3.39	3.25	3.19	2.89
45.27	4.44	4.27	4.25	4.02	3.89	3.67	3.35	3.33	3.26	3.11	2.85
47.81	4.59	4.29	4.18	3.92	3.88	3.75	3.55	3.36	3.25	3.15	2.86
50.55	4.06	4.09	3.89	3.69	3.56	3.38	3.16	2.93	2.79	2.75	2.54
53.51	4.60	4.30	4.30	4.13	3.86	3.71	3.51	3.31	3.15	2.96	2.80
56.69	4.49	4.45	4.27	4.13	3.77	3.81	3.62	3.34	3.13	3.13	3.02
60.13	4.27	4.23	4.12	3.95	3.76	3.57	3.34	3.27	3.00	2.93	2.61
63.84	4.51	4.33	4.24	4.13	3.89	3.61	3.51	3.43	3.25	3.05	2.85
67.83	4.35	4.16	3.99	3.86	3.73	3.55	3.31	3.14	3.00	2.92	2.71
72.15	4.24	4.18	4.21	3.99	3.74	3.49	3.30	3.20	3.03	2.78	2.72
76.80	4.38	4.07	3.99	3.87	3.61	3.52	3.25	3.05	2.88	2.80	2.57
81.81	4.46	4.29	4.19	3.90	3.84	3.59	3.38	3.32	3.15	2.87	2.80
87.22	4.26	3.98	3.97	3.75	3.65	3.44	3.22	3.06	2.95	2.89	2.60
93.06	4.32	4.00	3.98	3.77	3.70	3.47	3.40	3.09	2.92	2.78	2.63
99.35	4.32	4.20	3.97	3.87	3.81	3.52	3.35	2.99	2.91	2.84	2.58
106.13	4.13	3.99	3.94	3.66	3.56	3.32	3.08	3.10	2.80	2.69	2.43
113.45	4.46	4.19	4.12	4.01	3.75	3.55	3.32	3.13	3.15	2.80	2.61
121.34	4.29	4.18	4.00	3.75	3.69	3.46	3.25	3.03	2.94	2.89	2.61
129.86	4.13	3.84	3.71	3.60	3.43	3.28	3.08	2.86	2.59	2.64	2.35
139.04	4.02	4.04	3.71	3.60	3.57	3.19	3.18	2.98	2.71	2.57	2.30
148.94	4.02	3.80	3.62	3.44	3.36	3.09	2.90	2.70	2.65	2.39	2.26
159.62	4.06	3.96	3.78	3.61	3.46	3.33	3.01	2.84	2.85	2.57	2.44
171.14	4.26	3.98	3.88	3.76	3.44	3.34	3.05	2.86	2.86	2.65	2.47
183.56	3.85	3.82	3.63	3.43	3.23	3.09	2.88	2.70	2.60	2.35	2.28
196.96	4.20	4.06	3.89	3.71	3.45	3.26	3.03	2.77	2.69	2.61	2.22
211.41	4.06	3.96	3.77	3.46	3.24	3.15	2.86	2.80	2.68	2.55	2.24
226.99	4.00	3.91	3.84	3.51	3.42	3.21	2.99	2.82	2.67	2.52	2.29
243.80	3.75	3.55	3.56	3.26	3.13	2.92	2.77	2.50	2.30	2.22	2.05
261.93	4.04	3.96	3.88	3.58	3.32	3.34	2.95	2.80	2.73	2.56	2.36
281.48	4.02	3.81	3.57	3.45	3.23	2.98	2.77	2.74	2.61	2.37	2.15
302.56	3.75	3.60	3.55	3.26	3.04	2.88	2.75	2.57	2.39	2.25	2.13
325.30	3.86	3.80	3.54	3.40	3.26	3.09	2.80	2.58	2.49	2.34	2.11
349.83	3.67	3.49	3.44	3.16	3.01	2.95	2.80	2.53	2.40	2.14	1.98
376.28	3.68	3.55	3.49	3.18	3.09	2.85	2.62	2.51	2.42	2.12	2.04
404.81	3.83	3.71	3.47	3.20	3.16	2.89	2.73	2.64	2.40	2.27	1.93
435.58	3.69	3.62	3.38	3.25	3.11	2.73	2.55	2.48	2.27	2.20	2.06
468.76	3.83	3.40	3.31	3.16	2.93	2.78	2.65	2.48	2.25	2.01	1.99
504.55	3.46	3.27	3.12	2.99	2.76	2.71	2.48	2.32	2.16	2.05	1.82
543.15	3.53	3.35	3.16	3.06	2.90	2.75	2.53	2.28	2.18	2.03	1.86
584.78	3.40	3.24	3.03	2.89	2.79	2.61	2.48	2.36	2.22	1.97	1.79
629.68	3.47	3.23	3.14	2.84	2.85	2.57	2.41	2.18	2.13	2.05	1.92
678.10	3.56	3.37	3.21	3.02	2.79	2.73	2.43	2.31	2.20	1.95	1.77

730.33	3.61	3.35	3.16	3.05	2.93	2.80	2.42	2.28	2.17	2.04	1.81
786.65	3.49	3.19	3.07	2.87	2.77	2.70	2.32	2.13	2.16	1.98	1.76
847.40	3.37	3.19	3.01	2.96	2.65	2.40	2.24	2.09	2.01	1.78	1.73
912.92	3.49	3.34	3.32	3.02	2.70	2.58	2.42	2.21	2.01	1.95	1.85
983.58	3.40	3.31	3.16	2.77	2.78	2.58	2.32	2.25	2.14	1.96	1.89
1059.78	3.31	3.17	3.07	2.83	2.65	2.53	2.43	2.17	2.03	1.95	1.73
1141.98	3.12	2.97	2.84	2.55	2.56	2.32	2.09	1.88	1.88	1.64	1.46
1230.62	2.95	2.83	2.72	2.48	2.36	2.19	2.13	1.92	1.66	1.75	1.49
1326.23	3.05	2.89	2.83	2.64	2.48	2.36	2.14	1.99	1.93	1.87	1.58
1429.34	3.21	3.17	2.83	2.78	2.53	2.43	2.35	2.09	1.94	1.79	1.67
1540.54	3.24	2.93	2.84	2.67	2.57	2.44	2.22	1.96	2.02	1.74	1.59
1660.48	3.09	2.86	2.84	2.57	2.42	2.28	1.98	1.90	1.87	1.66	1.54
1789.83	2.99	2.78	2.63	2.38	2.33	2.11	1.90	1.91	1.67	1.63	1.46
1929.34	2.98	2.95	2.83	2.70	2.41	2.34	2.05	1.96	1.82	1.63	1.52
2079.80	2.88	2.85	2.72	2.47	2.35	2.18	1.97	1.94	1.91	1.58	1.49
2242.08	2.95	2.74	2.64	2.46	2.33	2.08	2.04	1.88	1.72	1.51	1.36
2417.10	2.92	2.58	2.62	2.48	2.41	2.24	1.96	1.76	1.70	1.64	1.42
2605.85	2.79	2.77	2.47	2.46	2.29	2.12	1.96	1.67	1.63	1.51	1.41
2809.43	2.67	2.38	2.39	2.24	2.00	1.98	1.76	1.78	1.53	1.52	1.33
3028.99	2.61	2.62	2.35	2.17	2.17	2.06	1.72	1.60	1.66	1.41	1.22
3265.79	2.74	2.59	2.46	2.31	2.23	1.94	1.75	1.63	1.60	1.55	1.32
3521.18	2.75	2.67	2.49	2.20	2.06	1.94	1.82	1.66	1.53	1.44	1.34
3796.62	2.58	2.17	2.18	2.10	1.85	1.89	1.62	1.56	1.38	1.29	1.31

Wavelength (nm) / Time (ps)	444.66	446.06	447.47	448.88	450.29	451.70	453.11	454.52	455.92	457.33	458.74
-3.28	0.01	-0.17	-0.20	-0.12	-0.15	-0.03	-0.07	-0.13	-0.11	-0.07	-0.04
-2.78	-0.04	-0.14	-0.10	-0.10	-0.07	-0.08	0.04	-0.15	-0.03	-0.06	-0.04
-2.28	-0.03	-0.02	-0.08	-0.02	0.12	0.01	0.00	-0.04	-0.02	-0.08	0.07
-1.78	0.08	0.04	0.11	0.07	0.01	-0.01	0.13	0.12	0.02	0.07	0.13
-1.28	0.07	0.09	0.03	0.00	0.01	0.04	0.00	0.01	0.00	0.02	-0.03
-0.78	-0.11	-0.08	-0.01	-0.11	-0.10	-0.10	-0.16	-0.08	-0.06	-0.15	-0.12
-0.28	0.05	0.01	0.10	0.00	0.08	0.01	-0.05	0.00	0.08	0.13	-0.03
0.22	0.09	0.17	0.13	0.16	0.14	0.00	0.10	0.17	0.05	0.10	0.06
0.32	-0.12	-0.07	-0.08	-0.03	-0.16	0.05	-0.07	-0.13	-0.03	-0.09	-0.04
0.42	0.00	0.17	0.10	0.16	0.12	0.12	0.08	0.22	0.10	0.14	0.04
0.52	-0.04	-0.03	0.00	-0.03	0.08	-0.02	0.01	0.05	-0.02	0.07	0.07
0.62	-0.06	-0.06	0.04	-0.11	0.12	0.03	0.16	0.04	-0.07	0.06	0.08
0.72	0.16	0.19	0.28	0.23	0.21	0.28	0.31	0.24	0.28	0.21	0.31
0.77	0.04	0.02	0.10	0.02	0.17	0.14	-0.03	0.05	-0.03	0.04	-0.06
0.82	-0.01	0.05	0.06	0.03	0.09	0.00	0.04	0.09	0.06	0.10	0.15
0.87	0.04	-0.06	0.03	-0.09	0.07	0.00	0.00	0.06	-0.20	-0.09	-0.11
0.92	-0.06	0.00	0.08	-0.08	0.04	0.00	-0.12	0.01	-0.03	0.08	0.06
0.97	-0.11	-0.14	-0.13	-0.10	-0.03	-0.06	-0.08	-0.10	-0.11	-0.08	-0.08
1.02	-0.09	-0.03	-0.01	-0.16	-0.04	-0.02	0.06	0.00	-0.06	0.01	-0.05
1.07	0.00	0.13	0.13	0.05	0.03	0.07	0.07	0.07	0.06	0.13	0.10
1.12	-0.11	-0.05	-0.09	-0.19	-0.07	0.01	-0.12	-0.09	-0.10	-0.07	-0.13
1.17	-0.43	-0.29	-0.26	-0.30	-0.21	-0.33	-0.15	-0.09	-0.05	-0.16	-0.09
1.22	1.46	1.13	0.72	0.30	0.13	-0.12	-0.16	-0.31	-0.32	-0.28	-0.22

1.27	5.35	4.95	4.52	4.06	3.64	3.11	2.73	2.17	1.72	1.55	1.26
1.32	6.41	6.42	6.35	6.29	6.20	6.10	5.86	5.64	5.31	5.08	4.76
1.37	5.15	5.10	4.99	5.04	5.03	5.03	4.96	4.93	4.83	4.78	4.81
1.42	4.94	4.82	4.63	4.56	4.53	4.22	4.08	3.94	3.79	3.84	3.61
1.47	4.64	4.52	4.34	4.16	4.06	4.00	3.70	3.60	3.35	3.20	3.07
1.52	4.30	4.21	4.08	3.85	3.88	3.70	3.38	3.23	3.06	2.96	2.66
1.57	4.23	4.12	3.94	3.83	3.74	3.44	3.27	3.12	2.97	2.82	2.60
1.62	4.25	4.14	3.98	3.82	3.69	3.47	3.47	3.21	3.02	2.77	2.54
1.67	4.28	4.19	4.03	3.70	3.61	3.47	3.22	3.09	2.90	2.65	2.46
1.72	4.26	4.13	4.00	3.77	3.64	3.39	3.13	2.98	2.75	2.66	2.44
1.77	4.08	3.96	3.77	3.54	3.38	3.22	2.97	2.95	2.71	2.51	2.31
1.82	4.10	3.92	3.60	3.56	3.50	3.10	3.11	2.97	2.65	2.58	2.49
1.87	3.95	3.89	3.78	3.60	3.47	3.21	3.08	2.95	2.69	2.48	2.40
1.92	4.03	3.80	3.66	3.53	3.34	3.17	3.02	2.91	2.70	2.58	2.29
1.97	3.92	3.68	3.53	3.44	3.26	3.17	2.86	2.77	2.67	2.41	2.24
2.02	4.05	3.83	3.60	3.54	3.46	3.03	3.01	2.92	2.70	2.37	2.26
2.07	3.73	3.64	3.60	3.21	3.22	2.93	2.76	2.67	2.47	2.29	2.02
2.12	4.22	4.11	3.77	3.59	3.54	3.37	3.10	2.93	2.74	2.54	2.46
2.17	4.08	3.89	3.67	3.54	3.50	3.23	3.02	2.93	2.63	2.55	2.37
2.22	3.80	3.64	3.51	3.32	3.25	3.00	2.79	2.67	2.50	2.23	2.11
2.27	3.96	3.85	3.67	3.47	3.48	3.19	2.88	2.80	2.64	2.48	2.37
2.32	3.87	3.65	3.65	3.35	3.30	2.94	2.92	2.68	2.50	2.37	2.12
2.37	3.93	3.72	3.51	3.27	3.33	3.07	2.85	2.61	2.44	2.28	2.08
2.42	3.93	3.83	3.59	3.43	3.27	3.02	2.89	2.84	2.65	2.45	2.16
2.47	3.58	3.56	3.40	3.14	3.02	3.00	2.69	2.50	2.43	2.23	2.10
2.52	3.73	3.62	3.57	3.36	3.31	3.06	2.97	2.64	2.56	2.39	2.18
2.57	3.53	3.54	3.31	3.19	2.83	2.82	2.63	2.35	2.34	2.08	1.93
2.62	3.72	3.68	3.47	3.23	3.23	2.92	2.73	2.53	2.54	2.31	2.13
2.67	3.63	3.37	3.33	3.18	2.99	2.81	2.68	2.37	2.37	2.16	1.95
2.72	3.66	3.52	3.44	3.18	3.16	2.80	2.74	2.45	2.40	2.39	2.13
2.77	3.88	3.84	3.78	3.56	3.30	3.15	2.92	2.83	2.73	2.48	2.25
2.82	3.74	3.55	3.47	3.26	3.12	3.00	2.77	2.59	2.39	2.22	2.08
2.87	3.54	3.45	3.39	3.10	3.03	2.84	2.64	2.52	2.33	2.16	1.93
2.92	3.76	3.50	3.43	3.18	3.18	2.81	2.74	2.56	2.40	2.12	2.00
2.97	3.54	3.48	3.54	3.18	3.13	2.81	2.66	2.52	2.29	2.16	1.96
3.02	3.82	3.63	3.46	3.16	3.20	3.00	2.78	2.64	2.44	2.24	2.07
3.07	3.81	3.68	3.47	3.19	3.13	2.87	2.80	2.62	2.29	2.27	2.09
3.12	3.86	3.66	3.49	3.29	3.15	2.92	2.81	2.66	2.53	2.33	2.12
3.17	3.71	3.56	3.38	3.28	3.03	2.90	2.69	2.62	2.31	2.19	1.97
3.22	3.68	3.53	3.42	3.25	3.02	2.84	2.63	2.50	2.34	2.19	1.96
3.27	3.70	3.58	3.38	3.21	3.10	2.94	2.80	2.49	2.45	2.18	1.90
3.32	3.90	3.64	3.52	3.39	3.21	3.08	2.84	2.59	2.35	2.22	2.16
3.37	3.59	3.38	3.18	3.11	2.88	2.75	2.54	2.32	2.12	2.09	1.74
3.42	3.75	3.60	3.37	3.29	3.13	2.84	2.82	2.55	2.36	2.18	1.99
3.47	3.61	3.46	3.30	3.15	2.93	2.82	2.58	2.51	2.20	2.12	1.94
3.52	3.56	3.49	3.30	3.09	3.02	2.80	2.62	2.45	2.20	2.07	1.99
3.57	3.53	3.37	3.26	3.11	2.89	2.80	2.64	2.30	2.15	1.97	1.95
3.62	3.67	3.40	3.45	3.13	2.98	2.85	2.65	2.50	2.34	2.10	1.94
3.67	3.50	3.27	3.12	2.95	2.93	2.57	2.45	2.38	2.16	2.04	1.87
3.72	3.60	3.46	3.26	3.00	2.94	2.77	2.69	2.39	2.23	2.19	1.95

3.92	3.73	3.48	3.31	3.05	3.02	2.85	2.68	2.45	2.27	2.12	1.93
4.12	3.66	3.43	3.32	3.01	3.04	2.77	2.56	2.42	2.26	2.02	1.77
4.32	3.39	3.31	3.22	2.92	2.94	2.65	2.46	2.46	2.14	2.07	1.82
4.52	3.44	3.34	3.20	3.01	2.93	2.55	2.62	2.40	2.10	2.10	1.82
4.72	3.50	3.37	3.16	2.90	2.84	2.65	2.46	2.27	2.10	1.90	1.73
4.92	3.65	3.45	3.28	3.05	2.81	2.72	2.67	2.44	2.28	2.13	1.98
5.12	3.49	3.35	3.14	2.88	2.84	2.62	2.47	2.34	2.01	1.99	1.87
5.32	3.47	3.38	3.28	2.96	2.85	2.71	2.48	2.37	2.20	2.09	1.92
5.52	3.56	3.46	3.20	2.92	2.81	2.61	2.62	2.28	2.09	2.04	1.80
5.72	3.69	3.58	3.30	3.15	3.07	2.87	2.59	2.40	2.22	2.10	1.93
5.92	3.31	3.18	2.92	2.73	2.69	2.48	2.28	2.07	2.03	1.76	1.72
6.12	3.35	3.20	3.08	2.89	2.78	2.56	2.50	2.25	2.12	1.95	1.78
6.32	3.09	3.00	2.83	2.62	2.64	2.34	2.12	2.05	1.90	1.74	1.61
6.52	3.32	3.21	3.13	2.91	2.80	2.53	2.29	2.18	2.04	1.90	1.84
6.72	3.61	3.45	3.28	3.01	2.91	2.72	2.53	2.52	2.31	2.11	2.08
6.92	3.22	3.21	2.99	2.80	2.65	2.53	2.36	2.19	2.03	1.79	1.63
7.12	3.31	3.23	3.00	2.83	2.71	2.55	2.37	2.20	2.01	1.84	1.78
7.32	3.28	3.25	3.11	2.88	2.78	2.47	2.23	2.15	2.02	1.78	1.60
7.52	3.31	3.04	3.01	2.83	2.72	2.52	2.29	2.14	1.92	1.83	1.70
7.72	3.42	3.21	3.03	2.91	2.76	2.60	2.52	2.24	2.15	1.91	1.76
7.92	3.30	3.16	3.08	2.80	2.75	2.52	2.31	2.15	1.97	1.78	1.64
8.12	3.44	3.28	3.00	2.76	2.69	2.63	2.42	2.27	2.09	1.91	1.77
8.32	3.50	3.28	3.02	2.86	2.73	2.58	2.36	2.23	2.13	1.85	1.76
8.52	3.16	2.93	2.84	2.68	2.55	2.29	2.20	2.02	1.79	1.76	1.53
8.72	3.33	3.22	3.03	2.82	2.58	2.46	2.19	2.05	1.93	1.79	1.63
8.92	3.23	3.06	2.85	2.71	2.73	2.36	2.23	2.08	1.88	1.69	1.52
9.12	3.43	3.15	3.02	2.82	2.68	2.49	2.27	2.17	1.92	1.85	1.70
9.32	3.13	2.93	2.75	2.62	2.44	2.34	2.02	2.12	1.88	1.74	1.54
9.52	3.30	3.08	2.84	2.79	2.64	2.33	2.25	2.17	1.88	1.79	1.59
9.72	3.07	2.91	2.89	2.78	2.66	2.32	2.12	2.06	1.77	1.61	1.54
9.92	3.15	3.01	2.84	2.68	2.52	2.32	2.19	1.99	1.89	1.64	1.51
10.12	3.25	3.03	3.00	2.73	2.63	2.40	2.27	2.11	1.90	1.71	1.47
10.32	2.92	2.80	2.64	2.49	2.36	2.21	1.96	1.88	1.81	1.50	1.44
10.52	3.09	2.91	2.86	2.69	2.49	2.37	2.19	2.05	1.79	1.65	1.43
10.72	3.27	3.00	2.88	2.81	2.58	2.43	2.29	2.04	1.92	1.74	1.65
10.92	3.16	2.88	2.75	2.73	2.45	2.32	2.21	2.04	1.84	1.74	1.57
11.12	3.20	2.85	2.74	2.64	2.47	2.27	2.22	1.93	1.76	1.71	1.68
11.32	3.22	3.04	2.99	2.86	2.69	2.48	2.39	2.10	1.87	1.77	1.80
11.52	3.05	2.87	2.80	2.66	2.49	2.36	2.02	1.98	1.76	1.64	1.49
11.72	3.08	2.90	2.72	2.62	2.63	2.30	2.14	1.94	1.81	1.79	1.54
11.92	3.01	2.76	2.62	2.55	2.46	2.17	2.04	1.81	1.83	1.60	1.42
12.12	3.27	3.11	2.89	2.72	2.62	2.33	2.19	1.89	1.77	1.68	1.51
12.32	3.25	3.07	2.93	2.73	2.49	2.44	2.27	2.10	1.95	1.63	1.60
12.52	3.05	2.84	2.62	2.47	2.34	2.18	2.15	1.95	1.73	1.64	1.50
12.72	3.24	3.10	2.90	2.64	2.62	2.35	2.22	2.12	1.91	1.78	1.54
12.92	2.97	2.94	2.74	2.59	2.40	2.33	2.22	1.97	1.65	1.59	1.57
13.12	2.95	2.66	2.75	2.45	2.34	2.27	2.06	1.82	1.64	1.49	1.43
13.32	3.16	2.91	2.75	2.59	2.51	2.22	2.06	1.95	1.74	1.50	1.44
13.52	2.96	2.87	2.66	2.58	2.51	2.28	2.09	1.94	1.78	1.58	1.50
13.72	3.22	3.04	2.88	2.64	2.52	2.32	2.28	2.07	1.92	1.67	1.51

13.92	3.07	2.98	2.70	2.68	2.41	2.26	2.09	1.96	1.75	1.68	1.48
14.00	3.18	3.10	2.95	2.58	2.64	2.29	2.32	2.01	1.94	1.70	1.57
14.08	3.06	2.80	2.76	2.42	2.45	2.29	2.06	1.88	1.83	1.64	1.41
14.17	2.94	2.91	2.64	2.57	2.35	2.15	2.10	1.79	1.67	1.50	1.37
14.27	2.98	2.88	2.76	2.55	2.43	2.25	2.05	1.91	1.81	1.58	1.53
14.38	3.05	2.87	2.62	2.59	2.38	2.25	2.04	2.00	1.67	1.58	1.56
14.49	2.90	2.71	2.64	2.45	2.35	2.22	1.99	1.87	1.61	1.62	1.41
14.61	2.94	2.85	2.55	2.54	2.39	2.09	2.06	1.73	1.60	1.62	1.41
14.75	2.91	2.59	2.61	2.43	2.32	2.10	2.04	1.83	1.57	1.62	1.47
14.89	3.34	3.07	2.98	2.80	2.78	2.43	2.28	2.27	1.93	1.83	1.66
15.05	2.96	2.76	2.71	2.47	2.45	2.15	1.98	1.84	1.76	1.69	1.49
15.21	3.07	3.01	2.86	2.64	2.54	2.39	2.24	2.07	1.79	1.71	1.47
15.39	3.09	2.84	2.66	2.58	2.30	2.28	2.10	1.96	1.76	1.67	1.52
15.59	2.86	2.91	2.67	2.49	2.38	2.22	1.97	1.90	1.71	1.54	1.44
15.80	3.18	2.99	2.77	2.56	2.62	2.36	2.10	1.93	1.90	1.77	1.56
16.02	2.95	2.65	2.55	2.41	2.26	2.05	2.03	1.78	1.62	1.59	1.31
16.27	3.04	2.94	2.89	2.57	2.43	2.38	2.23	1.99	1.77	1.71	1.59
16.53	3.00	2.79	2.65	2.56	2.38	2.24	2.05	1.98	1.77	1.62	1.41
16.81	2.86	2.75	2.48	2.38	2.44	2.19	2.06	1.84	1.73	1.50	1.43
17.12	3.02	2.84	2.73	2.51	2.28	2.30	2.06	1.92	1.86	1.58	1.50
17.45	2.93	2.71	2.56	2.35	2.24	2.08	1.93	1.82	1.71	1.43	1.39
17.81	3.10	2.99	2.84	2.54	2.53	2.40	2.17	1.86	1.85	1.68	1.57
18.19	3.07	2.98	2.84	2.61	2.51	2.24	2.27	2.01	1.71	1.73	1.53
18.60	3.12	2.87	2.85	2.68	2.60	2.36	2.18	2.00	1.84	1.67	1.46
19.05	3.14	2.96	2.88	2.61	2.54	2.41	2.17	2.00	1.82	1.58	1.58
19.53	2.95	2.70	2.57	2.46	2.18	2.08	2.06	1.91	1.67	1.50	1.31
20.05	3.03	2.76	2.70	2.43	2.38	2.11	2.03	1.82	1.69	1.52	1.39
20.61	2.99	2.92	2.67	2.43	2.39	2.19	1.98	1.85	1.75	1.55	1.48
21.22	3.02	2.86	2.70	2.57	2.43	2.18	2.15	2.02	1.76	1.59	1.53
21.87	2.89	2.87	2.76	2.47	2.38	2.25	2.02	1.85	1.69	1.51	1.23
22.57	2.98	2.81	2.79	2.47	2.31	2.19	2.05	1.94	1.73	1.59	1.49
23.33	2.91	2.80	2.70	2.45	2.39	2.19	2.08	1.83	1.72	1.48	1.41
24.15	2.95	2.80	2.68	2.44	2.36	2.22	2.07	1.83	1.69	1.48	1.34
25.03	2.94	2.65	2.53	2.27	2.28	1.99	1.98	1.89	1.62	1.47	1.43
25.98	2.98	2.79	2.65	2.47	2.36	2.19	1.98	1.92	1.78	1.54	1.37
27.01	2.72	2.72	2.42	2.36	2.32	2.13	1.78	1.75	1.59	1.34	1.25
28.11	2.96	2.75	2.65	2.36	2.35	2.20	1.99	1.72	1.78	1.45	1.29
29.30	2.83	2.79	2.59	2.42	2.29	2.09	1.97	1.81	1.54	1.45	1.35
30.59	2.95	2.74	2.57	2.41	2.36	2.12	2.00	1.74	1.56	1.42	1.36
31.98	2.75	2.48	2.46	2.23	2.10	1.92	1.79	1.66	1.45	1.30	1.11
33.47	2.71	2.61	2.50	2.39	2.27	1.97	1.88	1.77	1.56	1.43	1.24
35.09	2.79	2.73	2.37	2.12	2.26	1.95	1.84	1.73	1.66	1.36	1.31
36.83	2.69	2.61	2.64	2.35	2.21	2.07	1.91	1.65	1.55	1.47	1.19
38.71	2.68	2.58	2.35	2.24	2.08	1.88	1.75	1.65	1.35	1.35	1.14
40.73	2.72	2.65	2.52	2.40	2.07	1.93	1.90	1.65	1.51	1.29	1.31
42.92	2.79	2.56	2.44	2.40	2.26	1.94	1.83	1.77	1.51	1.33	1.30
45.27	2.76	2.50	2.56	2.29	2.12	2.14	1.79	1.68	1.60	1.45	1.25
47.81	2.80	2.55	2.51	2.20	2.22	2.06	1.84	1.70	1.49	1.35	1.24
50.55	2.45	2.31	2.16	1.93	1.95	1.71	1.55	1.38	1.28	1.09	1.01
53.51	2.72	2.61	2.41	2.27	2.17	1.92	1.80	1.58	1.47	1.44	1.24

56.69	2.86	2.72	2.46	2.37	2.22	1.98	1.78	1.77	1.39	1.23	1.02
60.13	2.47	2.39	2.20	2.02	1.91	1.78	1.65	1.35	1.41	1.13	0.92
63.84	2.62	2.50	2.24	2.10	1.80	1.88	1.70	1.58	1.38	1.11	1.00
67.83	2.57	2.35	2.17	2.03	2.04	1.74	1.62	1.43	1.26	1.26	1.01
72.15	2.54	2.38	2.23	1.99	1.97	1.72	1.58	1.46	1.25	1.10	1.03
76.80	2.36	2.36	2.18	1.88	1.83	1.54	1.44	1.34	1.20	1.04	0.80
81.81	2.69	2.42	2.39	2.18	2.03	1.84	1.59	1.49	1.31	1.20	1.02
87.22	2.49	2.31	2.04	1.89	1.87	1.69	1.64	1.35	1.32	1.01	0.93
93.06	2.44	2.27	2.19	1.92	1.85	1.62	1.46	1.25	1.14	1.09	0.82
99.35	2.49	2.30	2.09	1.95	1.81	1.59	1.68	1.47	1.18	1.00	0.90
106.13	2.29	2.22	2.05	1.81	1.68	1.54	1.45	1.28	1.10	1.00	0.75
113.45	2.64	2.31	2.24	2.07	2.05	1.74	1.53	1.45	1.22	1.14	0.97
121.34	2.45	2.18	2.15	1.98	1.82	1.65	1.48	1.31	1.04	0.96	0.74
129.86	2.20	2.11	1.87	1.72	1.60	1.45	1.28	1.15	0.90	0.85	0.60
139.04	2.34	2.18	1.97	1.83	1.72	1.48	1.36	1.22	1.00	0.92	0.75
148.94	2.12	1.96	1.74	1.56	1.54	1.44	1.21	1.01	0.92	0.71	0.61
159.62	2.33	2.10	1.92	1.79	1.70	1.43	1.25	1.18	1.03	0.87	0.65
171.14	2.28	2.18	1.94	1.68	1.71	1.52	1.32	1.23	0.94	0.83	0.62
183.56	2.09	1.90	1.74	1.59	1.42	1.19	1.17	0.93	0.90	0.66	0.56
196.96	2.16	2.03	1.81	1.72	1.68	1.27	1.19	1.08	0.82	0.62	0.48
211.41	2.19	1.97	1.92	1.72	1.80	1.33	1.12	1.07	0.96	0.75	0.48
226.99	2.21	2.00	1.95	1.69	1.55	1.36	1.24	0.89	0.87	0.71	0.55
243.80	1.96	1.66	1.69	1.40	1.32	1.10	1.04	0.83	0.74	0.65	0.45
261.93	2.20	2.03	1.94	1.61	1.61	1.43	1.22	0.93	0.81	0.64	0.54
281.48	1.95	1.97	1.73	1.63	1.56	1.26	1.14	1.02	0.84	0.65	0.44
302.56	1.93	1.87	1.67	1.54	1.37	1.16	0.99	0.90	0.76	0.54	0.46
325.30	2.00	1.92	1.67	1.47	1.41	1.25	0.93	0.87	0.85	0.59	0.43
349.83	1.89	1.78	1.66	1.37	1.34	1.24	1.05	0.91	0.81	0.55	0.42
376.28	1.84	1.79	1.61	1.30	1.31	1.21	0.97	0.89	0.64	0.48	0.27
404.81	1.84	1.77	1.56	1.42	1.37	1.16	1.00	0.92	0.68	0.56	0.37
435.58	1.90	1.70	1.56	1.39	1.33	1.15	0.90	0.74	0.73	0.56	0.32
468.76	1.71	1.68	1.48	1.27	1.37	1.06	0.83	0.80	0.61	0.51	0.25
504.55	1.68	1.43	1.38	1.29	1.17	0.97	0.89	0.75	0.55	0.42	0.27
543.15	1.70	1.64	1.50	1.31	1.26	0.94	0.89	0.63	0.64	0.45	0.38
584.78	1.61	1.49	1.38	1.30	1.14	1.05	0.93	0.62	0.52	0.45	0.31
629.68	1.62	1.53	1.44	1.27	1.29	1.14	0.75	0.79	0.54	0.41	0.33
678.10	1.77	1.61	1.44	1.20	1.11	0.98	0.82	0.73	0.54	0.45	0.35
730.33	1.74	1.65	1.46	1.26	1.34	1.20	1.01	0.79	0.69	0.57	0.39
786.65	1.70	1.57	1.46	1.32	1.16	1.04	0.88	0.77	0.63	0.46	0.29
847.40	1.61	1.37	1.25	1.08	0.97	0.89	0.87	0.60	0.46	0.40	0.29
912.92	1.66	1.52	1.37	1.24	1.20	0.92	0.84	0.63	0.49	0.41	0.19
983.58	1.66	1.58	1.38	1.17	1.17	0.95	0.81	0.77	0.74	0.45	0.36
1059.78	1.60	1.53	1.40	1.30	1.09	1.08	0.85	0.67	0.55	0.49	0.30
1141.98	1.33	1.32	1.23	1.01	1.05	0.87	0.74	0.67	0.46	0.39	0.34
1230.62	1.48	1.16	1.18	1.01	1.02	0.92	0.67	0.54	0.44	0.29	0.28
1326.23	1.56	1.34	1.29	1.24	1.07	0.94	0.87	0.74	0.57	0.52	0.47
1429.34	1.52	1.41	1.27	1.21	1.07	0.99	0.85	0.77	0.55	0.43	0.41
1540.54	1.48	1.36	1.26	1.07	1.04	0.94	0.80	0.81	0.56	0.54	0.38
1660.48	1.38	1.21	1.17	1.15	1.07	0.93	0.70	0.69	0.50	0.40	0.37
1789.83	1.39	1.33	1.17	1.13	1.05	0.84	0.73	0.74	0.58	0.43	0.37

1929.34	1.51	1.40	1.27	1.09	1.04	0.93	0.81	0.61	0.66	0.53	0.42
2079.80	1.46	1.35	1.22	0.95	1.04	0.84	0.77	0.64	0.62	0.49	0.48
2242.08	1.31	1.26	1.15	1.02	1.00	0.90	0.66	0.70	0.58	0.55	0.38
2417.10	1.40	1.19	1.21	0.96	0.94	0.94	0.91	0.66	0.64	0.46	0.44
2605.85	1.39	1.26	1.18	1.01	1.03	0.93	0.89	0.76	0.69	0.59	0.57
2809.43	1.17	1.06	1.15	0.86	0.96	0.80	0.69	0.79	0.69	0.49	0.42
3028.99	1.31	1.13	1.11	0.99	0.91	0.81	0.74	0.68	0.41	0.48	0.41
3265.79	1.17	1.32	1.09	1.02	0.96	0.91	0.62	0.64	0.69	0.55	0.37
3521.18	1.14	1.22	1.10	0.92	0.88	0.83	0.73	0.71	0.65	0.54	0.44
3796.62	1.15	1.10	1.04	0.91	0.90	0.72	0.65	0.59	0.47	0.34	0.41

Wavelength (nm) / Time (ps)	460.15	461.56	462.97	464.38	465.78	467.19	468.60	470.01	471.42	472.83	474.24
-3.28	-0.13	-0.12	-0.12	-0.17	-0.18	-0.14	-0.10	-0.06	-0.09	-0.10	-0.11
-2.78	-0.06	-0.11	-0.12	-0.08	-0.08	0.02	-0.02	-0.12	-0.12	-0.14	-0.08
-2.28	0.07	-0.03	-0.05	-0.09	0.06	0.06	-0.09	0.06	0.07	-0.08	0.04
-1.78	0.08	0.20	0.21	0.14	0.15	0.13	0.12	0.12	0.11	0.19	0.19
-1.28	-0.01	0.01	0.00	0.07	0.00	-0.09	-0.02	0.00	-0.13	-0.06	-0.11
-0.78	-0.15	-0.10	-0.10	-0.11	-0.08	-0.11	0.05	-0.04	0.00	-0.03	-0.04
-0.28	0.02	-0.03	0.03	0.02	-0.02	-0.07	-0.09	0.02	-0.01	0.00	-0.01
0.22	0.16	0.12	0.10	0.11	0.06	0.17	0.12	-0.03	0.07	0.11	0.07
0.32	-0.01	0.01	-0.08	-0.06	-0.09	-0.04	-0.05	-0.10	-0.07	-0.04	-0.16
0.42	0.03	0.06	0.12	0.18	0.18	0.06	0.08	0.16	0.16	0.15	0.21
0.52	0.03	0.01	-0.03	-0.04	-0.06	0.07	-0.01	-0.01	0.04	0.04	-0.04
0.62	0.01	-0.02	0.04	0.04	0.05	0.04	0.00	0.09	0.00	0.06	0.08
0.72	0.27	0.31	0.25	0.27	0.24	0.28	0.22	0.26	0.21	0.29	0.22
0.77	0.08	0.02	0.13	0.02	0.08	0.10	0.10	0.04	0.09	0.21	0.11
0.82	0.00	-0.01	0.03	0.07	0.18	0.00	-0.01	0.01	0.10	0.16	-0.02
0.87	-0.12	-0.11	-0.01	-0.13	-0.07	-0.06	-0.08	-0.12	-0.05	-0.10	-0.07
0.92	0.03	-0.10	-0.07	-0.12	-0.01	-0.03	0.05	0.05	0.10	-0.01	0.02
0.97	-0.09	-0.12	-0.06	-0.06	-0.03	-0.09	-0.01	-0.15	-0.08	0.01	-0.05
1.02	-0.02	0.07	-0.05	-0.12	-0.02	-0.02	-0.03	-0.06	0.02	-0.12	0.07
1.07	0.10	0.23	0.07	0.12	0.15	0.04	0.10	0.10	0.17	0.07	0.11
1.12	-0.17	-0.20	-0.22	-0.16	-0.14	-0.13	-0.09	-0.19	-0.16	-0.15	-0.14
1.17	-0.03	-0.20	-0.12	0.02	0.03	0.02	-0.05	-0.09	-0.04	-0.09	-0.09
1.22	-0.30	-0.23	-0.15	-0.08	-0.12	-0.15	-0.08	-0.07	-0.01	-0.07	0.02
1.27	0.88	0.78	0.46	0.25	0.20	0.11	-0.10	-0.16	-0.14	-0.25	-0.20
1.32	4.32	4.04	3.41	3.10	2.87	2.39	1.99	1.65	1.27	0.96	0.67
1.37	4.73	4.81	4.63	4.66	4.47	4.44	4.39	4.27	4.22	3.98	3.64
1.42	3.49	3.33	3.35	3.37	3.39	3.18	3.28	3.33	3.24	3.42	3.39
1.47	2.78	2.61	2.39	2.34	2.25	2.00	2.07	1.92	1.84	1.81	1.67
1.52	2.41	2.26	2.06	1.89	1.93	1.65	1.53	1.48	1.39	1.30	1.11
1.57	2.30	2.21	2.09	1.96	1.77	1.66	1.41	1.40	1.30	1.14	1.04
1.62	2.29	2.21	2.03	1.92	1.84	1.66	1.47	1.37	1.24	1.19	0.88
1.67	2.15	2.12	1.73	1.59	1.52	1.44	1.33	1.26	1.03	0.97	0.84
1.72	2.13	2.11	1.92	1.57	1.59	1.43	1.25	1.11	1.03	0.91	0.80
1.77	2.11	2.04	1.69	1.66	1.52	1.39	1.17	1.02	0.93	0.82	0.78
1.82	2.09	2.02	1.73	1.68	1.49	1.58	1.28	1.19	1.05	0.92	0.71
1.87	2.28	1.98	1.91	1.74	1.60	1.54	1.30	1.21	1.14	1.01	0.87

1.92	2.12	1.94	1.74	1.64	1.48	1.37	1.24	1.12	1.07	0.95	0.72
1.97	2.05	1.94	1.60	1.64	1.44	1.28	1.15	0.98	0.92	0.91	0.67
2.02	2.03	1.93	1.67	1.59	1.42	1.35	1.22	1.09	1.07	0.92	0.81
2.07	1.97	1.82	1.59	1.27	1.43	1.16	1.18	1.01	0.95	0.78	0.67
2.12	2.19	1.95	1.78	1.74	1.61	1.50	1.40	1.31	1.14	0.98	0.81
2.17	2.10	2.05	1.92	1.71	1.66	1.55	1.43	1.29	1.19	0.95	0.86
2.22	2.08	1.88	1.68	1.54	1.48	1.22	1.23	1.05	0.96	0.84	0.77
2.27	2.05	2.02	1.80	1.63	1.60	1.33	1.22	1.28	1.14	0.97	0.89
2.32	1.96	1.87	1.63	1.49	1.44	1.35	1.22	1.24	1.06	0.88	0.81
2.37	1.98	1.82	1.67	1.49	1.32	1.24	1.09	1.07	0.99	0.86	0.62
2.42	1.97	1.88	1.72	1.54	1.47	1.34	1.26	1.12	0.94	0.91	0.75
2.47	1.82	1.67	1.44	1.49	1.35	1.18	1.09	1.05	0.99	0.81	0.71
2.52	2.01	1.76	1.72	1.57	1.43	1.32	1.14	0.96	0.96	0.88	0.75
2.57	1.67	1.64	1.43	1.37	1.20	0.99	1.03	0.92	0.86	0.68	0.60
2.62	1.97	1.84	1.66	1.60	1.58	1.34	1.15	1.14	1.05	1.00	0.90
2.67	1.80	1.71	1.46	1.29	1.36	1.15	1.08	0.98	0.73	0.77	0.68
2.72	1.80	1.79	1.56	1.42	1.38	1.21	1.15	1.04	1.01	0.91	0.71
2.77	2.04	1.98	1.75	1.73	1.60	1.48	1.44	1.28	1.21	1.03	0.89
2.82	1.98	1.83	1.58	1.37	1.27	1.22	1.11	1.04	0.98	0.96	0.77
2.87	1.86	1.60	1.47	1.25	1.19	1.12	1.04	0.93	0.79	0.87	0.67
2.92	1.86	1.70	1.60	1.48	1.39	1.15	1.10	1.04	1.01	0.82	0.76
2.97	1.73	1.60	1.45	1.35	1.16	1.24	1.11	0.97	0.78	0.86	0.57
3.02	1.89	1.72	1.58	1.46	1.39	1.23	1.09	0.98	0.92	0.77	0.82
3.07	1.86	1.78	1.52	1.51	1.38	1.24	1.13	0.90	0.99	0.89	0.73
3.12	1.82	1.71	1.51	1.40	1.43	1.21	1.09	0.91	0.81	0.91	0.72
3.17	1.85	1.71	1.58	1.39	1.31	1.08	1.12	0.92	0.86	0.87	0.80
3.22	1.79	1.74	1.50	1.39	1.27	1.14	1.08	0.91	0.85	0.82	0.62
3.27	1.81	1.61	1.44	1.34	1.42	1.10	1.11	1.01	0.78	0.82	0.74
3.32	1.87	1.74	1.58	1.48	1.47	1.12	1.18	1.06	1.02	0.88	0.69
3.37	1.58	1.50	1.56	1.30	1.21	1.00	0.82	0.81	0.81	0.59	0.60
3.42	1.86	1.67	1.62	1.28	1.37	1.14	1.02	0.90	0.83	0.78	0.76
3.47	1.66	1.58	1.39	1.26	1.19	0.95	0.86	0.85	0.72	0.64	0.59
3.52	1.67	1.56	1.37	1.27	1.23	0.93	0.95	0.82	0.76	0.66	0.63
3.57	1.79	1.61	1.43	1.19	1.30	0.99	1.03	0.90	0.81	0.76	0.56
3.62	1.64	1.73	1.52	1.36	1.33	1.24	0.93	0.98	0.89	0.83	0.70
3.67	1.58	1.51	1.34	1.21	1.14	1.02	0.90	0.84	0.80	0.59	0.51
3.72	1.69	1.54	1.44	1.37	1.20	1.15	0.94	0.76	0.86	0.76	0.60
3.92	1.71	1.64	1.45	1.20	1.19	1.00	1.04	0.99	0.93	0.73	0.70
4.12	1.60	1.51	1.35	1.32	1.17	1.14	1.01	0.85	0.78	0.75	0.61
4.32	1.71	1.63	1.32	1.22	1.22	1.10	1.01	0.92	0.79	0.68	0.64
4.52	1.62	1.65	1.21	1.25	1.22	1.04	0.90	0.79	0.70	0.64	0.51
4.72	1.61	1.51	1.18	1.12	1.06	0.88	0.86	0.80	0.76	0.67	0.46
4.92	1.72	1.62	1.46	1.38	1.28	1.19	0.98	0.97	0.93	0.87	0.73
5.12	1.66	1.55	1.39	1.20	1.20	1.08	0.97	0.89	0.78	0.69	0.77
5.32	1.78	1.50	1.42	1.31	1.20	1.04	0.89	0.84	0.85	0.75	0.56
5.52	1.65	1.63	1.43	1.40	1.22	1.11	1.02	0.89	0.93	0.82	0.69
5.72	1.66	1.69	1.46	1.43	1.32	1.13	1.04	0.86	0.90	0.83	0.76
5.92	1.34	1.37	1.19	0.90	1.03	0.95	0.78	0.74	0.68	0.55	0.40
6.12	1.63	1.40	1.27	1.07	1.09	0.90	0.89	0.85	0.69	0.61	0.51
6.32	1.35	1.29	1.08	0.95	0.93	0.90	0.80	0.71	0.56	0.39	0.48

6.52	1.54	1.40	1.23	1.07	1.06	1.00	0.77	0.72	0.72	0.71	0.77
6.72	1.78	1.59	1.45	1.42	1.31	1.14	1.09	0.94	0.94	0.82	0.69
6.92	1.47	1.33	1.24	1.13	1.01	0.88	0.82	0.73	0.70	0.58	0.41
7.12	1.49	1.36	1.24	0.88	1.01	1.04	0.78	0.70	0.66	0.64	0.59
7.32	1.47	1.43	1.28	1.06	0.97	0.81	0.82	0.73	0.69	0.52	0.57
7.52	1.44	1.32	1.21	1.13	1.01	0.91	0.89	0.81	0.67	0.62	0.51
7.72	1.52	1.41	1.24	1.13	1.08	0.95	0.91	0.75	0.70	0.69	0.53
7.92	1.48	1.28	1.23	1.00	0.97	0.85	0.89	0.68	0.74	0.47	0.58
8.12	1.63	1.43	1.16	1.13	1.15	0.92	0.80	0.81	0.78	0.66	0.57
8.32	1.52	1.35	1.20	1.07	1.10	0.92	0.81	0.79	0.59	0.59	0.59
8.52	1.34	1.26	1.04	1.09	0.94	0.77	0.68	0.69	0.53	0.45	0.45
8.72	1.34	1.24	1.10	1.04	0.79	0.73	0.68	0.70	0.67	0.42	0.35
8.92	1.37	1.32	1.12	0.88	0.89	0.67	0.67	0.66	0.54	0.46	0.46
9.12	1.45	1.31	1.15	1.03	0.99	0.83	0.76	0.69	0.57	0.60	0.43
9.32	1.31	1.28	1.17	1.02	0.83	0.80	0.81	0.68	0.61	0.52	0.49
9.52	1.41	1.38	1.10	0.97	0.96	0.74	0.72	0.75	0.56	0.69	0.31
9.72	1.31	1.25	1.02	0.90	0.86	0.86	0.76	0.56	0.62	0.40	0.45
9.92	1.35	1.24	1.17	0.96	0.99	0.78	0.80	0.75	0.72	0.53	0.45
10.12	1.28	1.39	1.06	1.03	0.77	0.75	0.77	0.58	0.53	0.53	0.43
10.32	1.18	1.17	0.96	0.83	0.82	0.63	0.66	0.69	0.41	0.42	0.52
10.52	1.11	1.22	1.06	0.93	0.84	0.74	0.68	0.51	0.54	0.46	0.31
10.72	1.47	1.33	1.11	1.00	0.94	0.78	0.83	0.78	0.63	0.62	0.52
10.92	1.33	1.26	1.10	0.96	0.90	0.71	0.72	0.74	0.50	0.45	0.42
11.12	1.29	1.18	0.95	0.86	0.89	0.73	0.61	0.53	0.47	0.46	0.38
11.32	1.41	1.36	1.30	1.12	1.05	0.95	0.87	0.75	0.75	0.66	0.62
11.52	1.40	1.17	1.08	0.87	0.88	0.71	0.69	0.64	0.53	0.55	0.32
11.72	1.16	1.19	0.99	0.86	0.84	0.86	0.63	0.67	0.58	0.41	0.34
11.92	1.17	1.00	0.92	0.92	0.80	0.64	0.63	0.47	0.52	0.40	0.36
12.12	1.31	1.27	1.14	0.96	0.93	0.77	0.65	0.56	0.51	0.46	0.41
12.32	1.37	1.18	1.09	1.00	0.95	0.93	0.87	0.64	0.61	0.53	0.40
12.52	1.18	1.11	1.00	0.94	0.75	0.72	0.60	0.54	0.67	0.46	0.34
12.72	1.59	1.41	1.21	1.06	1.03	0.86	0.87	0.85	0.76	0.64	0.56
12.92	1.25	1.22	1.16	0.93	0.91	0.79	0.69	0.59	0.60	0.53	0.48
13.12	1.26	1.15	0.94	0.84	0.93	0.78	0.70	0.52	0.47	0.40	0.29
13.32	1.26	1.11	0.93	0.89	0.83	0.61	0.63	0.48	0.32	0.40	0.18
13.52	1.29	1.18	1.15	0.94	0.89	0.78	0.63	0.68	0.57	0.53	0.42
13.72	1.43	1.34	1.05	0.94	0.85	0.78	0.79	0.82	0.62	0.53	0.48
13.92	1.20	1.17	1.00	0.94	0.86	0.74	0.66	0.67	0.44	0.47	0.34
14.00	1.38	1.24	1.13	0.96	0.95	0.82	0.76	0.67	0.71	0.59	0.53
14.08	1.36	1.20	0.87	0.88	0.86	0.80	0.63	0.56	0.51	0.41	0.36
14.17	1.34	1.12	0.92	0.77	0.71	0.56	0.57	0.67	0.37	0.48	0.38
14.27	1.32	1.22	0.98	0.97	0.91	0.68	0.58	0.53	0.59	0.48	0.32
14.38	1.27	1.21	0.96	0.95	0.88	0.77	0.81	0.66	0.57	0.52	0.32
14.49	1.27	1.06	1.01	0.84	0.86	0.69	0.52	0.56	0.56	0.36	0.33
14.61	1.14	1.08	0.94	0.81	0.73	0.58	0.64	0.52	0.40	0.41	0.26
14.75	1.15	1.10	0.94	0.85	0.72	0.60	0.55	0.40	0.49	0.43	0.27
14.89	1.55	1.36	1.24	1.15	1.01	1.00	0.78	0.69	0.73	0.62	0.54
15.05	1.21	1.08	0.95	0.90	0.84	0.70	0.74	0.66	0.45	0.49	0.43
15.21	1.41	1.32	1.20	1.00	1.00	0.97	0.70	0.61	0.63	0.50	0.48
15.39	1.18	1.27	1.08	0.85	0.91	0.72	0.70	0.62	0.52	0.49	0.37

15.59	1.22	1.09	1.09	0.86	0.80	0.65	0.57	0.42	0.35	0.38	0.30
15.80	1.37	1.22	1.12	1.00	0.85	0.70	0.76	0.64	0.52	0.61	0.40
16.02	1.24	1.13	1.03	0.88	0.79	0.61	0.71	0.68	0.40	0.43	0.29
16.27	1.33	1.14	1.06	0.97	0.98	0.76	0.61	0.60	0.58	0.43	0.31
16.53	1.26	1.11	0.99	0.81	0.75	0.67	0.60	0.47	0.46	0.40	0.30
16.81	1.18	1.06	0.91	0.87	0.73	0.50	0.46	0.55	0.38	0.36	0.35
17.12	1.35	1.20	1.03	0.88	0.94	0.75	0.61	0.49	0.50	0.42	0.41
17.45	1.12	1.02	0.89	0.72	0.65	0.59	0.60	0.41	0.37	0.32	0.22
17.81	1.32	1.15	0.98	0.86	0.82	0.70	0.66	0.54	0.45	0.41	0.41
18.19	1.36	1.21	1.02	0.90	0.86	0.77	0.65	0.53	0.55	0.49	0.38
18.60	1.27	1.31	1.05	1.00	0.86	0.66	0.66	0.54	0.54	0.45	0.49
19.05	1.19	1.21	1.08	1.00	0.85	0.71	0.58	0.56	0.41	0.40	0.40
19.53	1.14	1.23	0.94	0.83	0.75	0.66	0.53	0.41	0.40	0.32	0.28
20.05	1.12	1.02	0.82	0.71	0.68	0.50	0.46	0.41	0.31	0.21	0.27
20.61	1.24	1.10	1.09	0.94	0.83	0.64	0.47	0.59	0.47	0.44	0.25
21.22	1.20	1.18	0.98	0.90	0.86	0.80	0.54	0.47	0.48	0.35	0.37
21.87	1.12	1.01	0.88	0.79	0.72	0.58	0.48	0.45	0.43	0.24	0.06
22.57	1.16	1.05	1.00	0.83	0.72	0.64	0.74	0.55	0.51	0.36	0.34
23.33	1.26	1.08	1.06	0.89	0.88	0.69	0.60	0.44	0.51	0.32	0.23
24.15	1.25	1.16	0.85	0.81	0.67	0.64	0.50	0.42	0.32	0.30	0.29
25.03	1.11	1.08	0.92	0.72	0.75	0.56	0.51	0.45	0.53	0.32	0.26
25.98	1.01	1.01	0.77	0.75	0.78	0.54	0.50	0.45	0.36	0.29	0.17
27.01	1.08	0.99	0.80	0.66	0.63	0.45	0.40	0.36	0.36	0.24	0.12
28.11	1.10	0.97	0.92	0.69	0.69	0.65	0.52	0.41	0.34	0.44	0.26
29.30	1.16	1.04	0.89	0.81	0.74	0.57	0.50	0.50	0.42	0.24	0.33
30.59	0.98	0.98	0.78	0.77	0.71	0.42	0.46	0.38	0.20	0.23	0.16
31.98	0.93	0.82	0.64	0.56	0.60	0.44	0.37	0.27	0.11	0.10	0.08
33.47	1.05	0.89	0.90	0.61	0.61	0.49	0.48	0.39	0.37	0.35	0.16
35.09	1.00	0.96	0.80	0.78	0.66	0.48	0.38	0.33	0.27	0.15	0.17
36.83	1.05	0.95	0.87	0.65	0.61	0.48	0.29	0.31	0.21	0.13	0.05
38.71	1.02	0.97	0.78	0.63	0.63	0.46	0.36	0.15	0.32	0.14	0.18
40.73	1.18	0.94	0.89	0.68	0.70	0.57	0.40	0.32	0.31	0.27	0.12
42.92	1.11	0.91	0.80	0.70	0.62	0.50	0.50	0.36	0.27	0.27	0.16
45.27	1.00	1.09	0.82	0.62	0.55	0.42	0.45	0.45	0.37	0.24	0.14
47.81	1.05	0.95	0.68	0.64	0.58	0.47	0.43	0.22	0.29	0.12	0.09
50.55	0.67	0.73	0.49	0.38	0.40	0.24	0.15	0.20	0.14	-0.01	-0.13
53.51	0.99	0.93	0.72	0.63	0.52	0.57	0.36	0.31	0.24	0.25	0.10
56.69	0.99	0.85	0.58	0.52	0.53	0.33	0.24	0.16	0.13	-0.06	-0.09
60.13	0.72	0.65	0.45	0.46	0.38	0.30	0.14	0.12	0.05	-0.03	-0.10
63.84	0.80	0.65	0.54	0.44	0.42	0.22	0.14	0.03	0.11	-0.02	-0.10
67.83	0.72	0.65	0.55	0.30	0.35	0.17	0.04	0.05	-0.04	-0.11	-0.22
72.15	0.71	0.72	0.53	0.28	0.30	0.13	0.24	0.12	-0.02	-0.11	-0.10
76.80	0.60	0.53	0.42	0.30	0.17	0.04	0.04	-0.04	-0.10	-0.17	-0.24
81.81	0.71	0.72	0.59	0.43	0.36	0.28	0.15	0.10	0.04	0.01	-0.27
87.22	0.81	0.64	0.38	0.29	0.30	0.31	0.05	0.05	0.00	-0.02	-0.20
93.06	0.60	0.57	0.34	0.23	0.13	0.03	-0.08	-0.08	-0.18	-0.18	-0.25
99.35	0.69	0.68	0.34	0.28	0.27	0.09	0.05	0.02	-0.16	-0.15	-0.28
106.13	0.61	0.62	0.30	0.12	0.07	0.01	-0.09	-0.11	-0.06	-0.28	-0.32
113.45	0.81	0.62	0.47	0.46	0.37	0.14	0.09	-0.09	-0.11	-0.08	-0.16
121.34	0.62	0.53	0.43	0.23	0.11	-0.01	-0.12	-0.12	-0.21	-0.36	-0.40

129.86	0.48	0.36	0.08	0.03	0.02	-0.21	-0.28	-0.25	-0.43	-0.49	-0.50
139.04	0.55	0.56	0.36	0.12	0.07	-0.03	-0.08	-0.25	-0.23	-0.29	-0.46
148.94	0.43	0.20	0.09	0.07	-0.02	-0.09	-0.24	-0.23	-0.30	-0.40	-0.49
159.62	0.53	0.39	0.24	0.10	-0.07	-0.12	-0.17	-0.24	-0.29	-0.48	-0.46
171.14	0.62	0.36	0.19	0.02	0.09	-0.25	-0.27	-0.27	-0.35	-0.36	-0.45
183.56	0.44	0.19	0.07	-0.03	-0.09	-0.18	-0.29	-0.32	-0.36	-0.51	-0.57
196.96	0.38	0.17	0.11	0.03	-0.10	-0.18	-0.34	-0.32	-0.40	-0.53	-0.59
211.41	0.34	0.20	0.06	0.03	0.02	-0.19	-0.21	-0.40	-0.37	-0.52	-0.52
226.99	0.33	0.20	0.07	-0.04	-0.03	-0.16	-0.23	-0.36	-0.45	-0.54	-0.64
243.80	0.33	0.10	-0.04	-0.03	-0.20	-0.32	-0.40	-0.43	-0.50	-0.55	-0.56
261.93	0.31	0.21	0.05	-0.05	-0.22	-0.19	-0.33	-0.36	-0.40	-0.47	-0.64
281.48	0.27	0.05	-0.04	-0.21	-0.18	-0.32	-0.37	-0.59	-0.50	-0.70	-0.72
302.56	0.15	0.02	-0.04	-0.19	-0.09	-0.36	-0.47	-0.46	-0.44	-0.54	-0.76
325.30	0.18	0.12	-0.12	-0.15	-0.23	-0.30	-0.32	-0.39	-0.47	-0.58	-0.60
349.83	0.16	0.16	-0.06	-0.17	-0.08	-0.26	-0.40	-0.50	-0.44	-0.52	-0.67
376.28	0.21	0.10	-0.08	-0.23	-0.22	-0.42	-0.52	-0.51	-0.51	-0.64	-0.76
404.81	0.13	0.17	-0.11	-0.20	-0.23	-0.30	-0.41	-0.49	-0.43	-0.63	-0.53
435.58	0.16	0.12	-0.01	-0.17	-0.29	-0.39	-0.42	-0.47	-0.47	-0.53	-0.58
468.76	0.04	0.14	-0.14	-0.27	-0.20	-0.36	-0.41	-0.45	-0.45	-0.53	-0.66
504.55	-0.01	-0.08	-0.10	-0.22	-0.26	-0.40	-0.48	-0.48	-0.66	-0.70	-0.67
543.15	0.14	-0.05	-0.11	-0.05	-0.19	-0.30	-0.33	-0.45	-0.37	-0.54	-0.63
584.78	-0.03	-0.12	-0.10	-0.27	-0.32	-0.47	-0.45	-0.61	-0.78	-0.57	-0.61
629.68	0.18	0.02	-0.13	-0.16	-0.21	-0.22	-0.37	-0.37	-0.55	-0.58	-0.49
678.10	0.14	0.01	-0.19	-0.14	-0.30	-0.39	-0.35	-0.46	-0.48	-0.58	-0.65
730.33	0.32	0.22	-0.02	-0.07	-0.17	-0.25	-0.20	-0.35	-0.38	-0.45	-0.49
786.65	0.20	0.07	-0.14	-0.19	-0.07	-0.18	-0.26	-0.36	-0.42	-0.49	-0.35
847.40	0.06	-0.03	-0.22	-0.19	-0.24	-0.31	-0.40	-0.43	-0.53	-0.57	-0.65
912.92	0.06	0.03	-0.10	-0.17	-0.18	-0.40	-0.40	-0.45	-0.48	-0.49	-0.62
983.58	0.22	0.15	0.04	-0.06	-0.11	-0.22	-0.29	-0.38	-0.43	-0.34	-0.40
1059.78	0.13	0.21	-0.04	-0.04	-0.11	-0.10	-0.17	-0.33	-0.28	-0.34	-0.42
1141.98	0.19	0.07	-0.04	-0.16	-0.15	-0.36	-0.30	-0.29	-0.41	-0.43	-0.47
1230.62	0.10	0.09	-0.08	-0.20	-0.14	-0.16	-0.20	-0.31	-0.33	-0.36	-0.47
1326.23	0.24	0.18	0.05	-0.04	-0.02	-0.17	-0.22	-0.18	-0.17	-0.21	-0.38
1429.34	0.19	0.20	0.02	-0.03	0.03	-0.11	-0.20	-0.24	-0.24	-0.29	-0.35
1540.54	0.17	0.15	0.06	0.07	-0.03	-0.03	-0.15	-0.16	-0.19	-0.22	-0.26
1660.48	0.18	0.08	-0.02	-0.03	-0.12	-0.12	-0.24	-0.30	-0.16	-0.24	-0.23
1789.83	0.22	0.18	0.07	-0.04	-0.06	-0.02	-0.15	-0.20	-0.18	-0.13	-0.20
1929.34	0.33	0.22	0.06	0.06	0.05	-0.04	-0.01	-0.08	-0.15	-0.15	-0.06
2079.80	0.31	0.14	0.12	0.00	0.09	0.01	0.00	-0.01	-0.07	-0.07	-0.20
2242.08	0.29	0.32	0.07	0.04	-0.01	0.07	-0.04	-0.09	-0.06	-0.12	-0.16
2417.10	0.32	0.20	0.07	0.24	0.11	-0.08	-0.01	0.11	-0.04	0.00	-0.14
2605.85	0.32	0.26	0.26	0.20	0.24	0.16	0.12	0.04	0.12	0.05	-0.02
2809.43	0.28	0.24	0.14	0.17	0.12	0.12	0.01	-0.02	-0.04	0.00	-0.01
3028.99	0.26	0.23	0.16	0.17	0.12	0.02	0.13	0.06	0.09	-0.02	0.02
3265.79	0.29	0.33	0.19	0.17	0.19	0.06	0.12	0.10	0.05	0.09	0.12
3521.18	0.31	0.27	0.28	0.17	0.23	0.21	0.27	0.21	0.09	0.19	-0.03
3796.62	0.30	0.24	0.12	0.10	0.21	-0.07	0.06	0.02	0.09	0.05	0.05

Time (ps) \ Wavelength (nm)	460.15	461.56	462.97	464.38	465.78	467.19	468.60	470.01	471.42	472.83	474.24
-3.28	-0.13	-0.12	-0.12	-0.17	-0.18	-0.14	-0.10	-0.06	-0.09	-0.10	-0.11
-2.78	-0.06	-0.11	-0.12	-0.08	-0.08	0.02	-0.02	-0.12	-0.12	-0.14	-0.08
-2.28	0.07	-0.03	-0.05	-0.09	0.06	0.06	-0.09	0.06	0.07	-0.08	0.04
-1.78	0.08	0.20	0.21	0.14	0.15	0.13	0.12	0.12	0.11	0.19	0.19
-1.28	-0.01	0.01	0.00	0.07	0.00	-0.09	-0.02	0.00	-0.13	-0.06	-0.11
-0.78	-0.15	-0.10	-0.10	-0.11	-0.08	-0.11	0.05	-0.04	0.00	-0.03	-0.04
-0.28	0.02	-0.03	0.03	0.02	-0.02	-0.07	-0.09	0.02	-0.01	0.00	-0.01
0.22	0.16	0.12	0.10	0.11	0.06	0.17	0.12	-0.03	0.07	0.11	0.07
0.32	-0.01	0.01	-0.08	-0.06	-0.09	-0.04	-0.05	-0.10	-0.07	-0.04	-0.16
0.42	0.03	0.06	0.12	0.18	0.18	0.06	0.08	0.16	0.16	0.15	0.21
0.52	0.03	0.01	-0.03	-0.04	-0.06	0.07	-0.01	-0.01	0.04	0.04	-0.04
0.62	0.01	-0.02	0.04	0.04	0.05	0.04	0.00	0.09	0.00	0.06	0.08
0.72	0.27	0.31	0.25	0.27	0.24	0.28	0.22	0.26	0.21	0.29	0.22
0.77	0.08	0.02	0.13	0.02	0.08	0.10	0.10	0.04	0.09	0.21	0.11
0.82	0.00	-0.01	0.03	0.07	0.18	0.00	-0.01	0.01	0.10	0.16	-0.02
0.87	-0.12	-0.11	-0.01	-0.13	-0.07	-0.06	-0.08	-0.12	-0.05	-0.10	-0.07
0.92	0.03	-0.10	-0.07	-0.12	-0.01	-0.03	0.05	0.05	0.10	-0.01	0.02
0.97	-0.09	-0.12	-0.06	-0.06	-0.03	-0.09	-0.01	-0.15	-0.08	0.01	-0.05
1.02	-0.02	0.07	-0.05	-0.12	-0.02	-0.02	-0.03	-0.06	0.02	-0.12	0.07
1.07	0.10	0.23	0.07	0.12	0.15	0.04	0.10	0.10	0.17	0.07	0.11
1.12	-0.17	-0.20	-0.22	-0.16	-0.14	-0.13	-0.09	-0.19	-0.16	-0.15	-0.14
1.17	-0.03	-0.20	-0.12	0.02	0.03	0.02	-0.05	-0.09	-0.04	-0.09	-0.09
1.22	-0.30	-0.23	-0.15	-0.08	-0.12	-0.15	-0.08	-0.07	-0.01	-0.07	0.02
1.27	0.88	0.78	0.46	0.25	0.20	0.11	-0.10	-0.16	-0.14	-0.25	-0.20
1.32	4.32	4.04	3.41	3.10	2.87	2.39	1.99	1.65	1.27	0.96	0.67
1.37	4.73	4.81	4.63	4.66	4.47	4.44	4.39	4.27	4.22	3.98	3.64
1.42	3.49	3.33	3.35	3.37	3.39	3.18	3.28	3.33	3.24	3.42	3.39
1.47	2.78	2.61	2.39	2.34	2.25	2.00	2.07	1.92	1.84	1.81	1.67
1.52	2.41	2.26	2.06	1.89	1.93	1.65	1.53	1.48	1.39	1.30	1.11
1.57	2.30	2.21	2.09	1.96	1.77	1.66	1.41	1.40	1.30	1.14	1.04
1.62	2.29	2.21	2.03	1.92	1.84	1.66	1.47	1.37	1.24	1.19	0.88
1.67	2.15	2.12	1.73	1.59	1.52	1.44	1.33	1.26	1.03	0.97	0.84
1.72	2.13	2.11	1.92	1.57	1.59	1.43	1.25	1.11	1.03	0.91	0.80
1.77	2.11	2.04	1.69	1.66	1.52	1.39	1.17	1.02	0.93	0.82	0.78
1.82	2.09	2.02	1.73	1.68	1.49	1.58	1.28	1.19	1.05	0.92	0.71
1.87	2.28	1.98	1.91	1.74	1.60	1.54	1.30	1.21	1.14	1.01	0.87
1.92	2.12	1.94	1.74	1.64	1.48	1.37	1.24	1.12	1.07	0.95	0.72
1.97	2.05	1.94	1.60	1.64	1.44	1.28	1.15	0.98	0.92	0.91	0.67
2.02	2.03	1.93	1.67	1.59	1.42	1.35	1.22	1.09	1.07	0.92	0.81
2.07	1.97	1.82	1.59	1.27	1.43	1.16	1.18	1.01	0.95	0.78	0.67
2.12	2.19	1.95	1.78	1.74	1.61	1.50	1.40	1.31	1.14	0.98	0.81
2.17	2.10	2.05	1.92	1.71	1.66	1.55	1.43	1.29	1.19	0.95	0.86
2.22	2.08	1.88	1.68	1.54	1.48	1.22	1.23	1.05	0.96	0.84	0.77
2.27	2.05	2.02	1.80	1.63	1.60	1.33	1.22	1.28	1.14	0.97	0.89
2.32	1.96	1.87	1.63	1.49	1.44	1.35	1.22	1.24	1.06	0.88	0.81
2.37	1.98	1.82	1.67	1.49	1.32	1.24	1.09	1.07	0.99	0.86	0.62
2.42	1.97	1.88	1.72	1.54	1.47	1.34	1.26	1.12	0.94	0.91	0.75

2.47	1.82	1.67	1.44	1.49	1.35	1.18	1.09	1.05	0.99	0.81	0.71
2.52	2.01	1.76	1.72	1.57	1.43	1.32	1.14	0.96	0.96	0.88	0.75
2.57	1.67	1.64	1.43	1.37	1.20	0.99	1.03	0.92	0.86	0.68	0.60
2.62	1.97	1.84	1.66	1.60	1.58	1.34	1.15	1.14	1.05	1.00	0.90
2.67	1.80	1.71	1.46	1.29	1.36	1.15	1.08	0.98	0.73	0.77	0.68
2.72	1.80	1.79	1.56	1.42	1.38	1.21	1.15	1.04	1.01	0.91	0.71
2.77	2.04	1.98	1.75	1.73	1.60	1.48	1.44	1.28	1.21	1.03	0.89
2.82	1.98	1.83	1.58	1.37	1.27	1.22	1.11	1.04	0.98	0.96	0.77
2.87	1.86	1.60	1.47	1.25	1.19	1.12	1.04	0.93	0.79	0.87	0.67
2.92	1.86	1.70	1.60	1.48	1.39	1.15	1.10	1.04	1.01	0.82	0.76
2.97	1.73	1.60	1.45	1.35	1.16	1.24	1.11	0.97	0.78	0.86	0.57
3.02	1.89	1.72	1.58	1.46	1.39	1.23	1.09	0.98	0.92	0.77	0.82
3.07	1.86	1.78	1.52	1.51	1.38	1.24	1.13	0.90	0.99	0.89	0.73
3.12	1.82	1.71	1.51	1.40	1.43	1.21	1.09	0.91	0.81	0.91	0.72
3.17	1.85	1.71	1.58	1.39	1.31	1.08	1.12	0.92	0.86	0.87	0.80
3.22	1.79	1.74	1.50	1.39	1.27	1.14	1.08	0.91	0.85	0.82	0.62
3.27	1.81	1.61	1.44	1.34	1.42	1.10	1.11	1.01	0.78	0.82	0.74
3.32	1.87	1.74	1.58	1.48	1.47	1.12	1.18	1.06	1.02	0.88	0.69
3.37	1.58	1.50	1.56	1.30	1.21	1.00	0.82	0.81	0.81	0.59	0.60
3.42	1.86	1.67	1.62	1.28	1.37	1.14	1.02	0.90	0.83	0.78	0.76
3.47	1.66	1.58	1.39	1.26	1.19	0.95	0.86	0.85	0.72	0.64	0.59
3.52	1.67	1.56	1.37	1.27	1.23	0.93	0.95	0.82	0.76	0.66	0.63
3.57	1.79	1.61	1.43	1.19	1.30	0.99	1.03	0.90	0.81	0.76	0.56
3.62	1.64	1.73	1.52	1.36	1.33	1.24	0.93	0.98	0.89	0.83	0.70
3.67	1.58	1.51	1.34	1.21	1.14	1.02	0.90	0.84	0.80	0.59	0.51
3.72	1.69	1.54	1.44	1.37	1.20	1.15	0.94	0.76	0.86	0.76	0.60
3.92	1.71	1.64	1.45	1.20	1.19	1.00	1.04	0.99	0.93	0.73	0.70
4.12	1.60	1.51	1.35	1.32	1.17	1.14	1.01	0.85	0.78	0.75	0.61
4.32	1.71	1.63	1.32	1.22	1.22	1.10	1.01	0.92	0.79	0.68	0.64
4.52	1.62	1.65	1.21	1.25	1.22	1.04	0.90	0.79	0.70	0.64	0.51
4.72	1.61	1.51	1.18	1.12	1.06	0.88	0.86	0.80	0.76	0.67	0.46
4.92	1.72	1.62	1.46	1.38	1.28	1.19	0.98	0.97	0.93	0.87	0.73
5.12	1.66	1.55	1.39	1.20	1.20	1.08	0.97	0.89	0.78	0.69	0.77
5.32	1.78	1.50	1.42	1.31	1.20	1.04	0.89	0.84	0.85	0.75	0.56
5.52	1.65	1.63	1.43	1.40	1.22	1.11	1.02	0.89	0.93	0.82	0.69
5.72	1.66	1.69	1.46	1.43	1.32	1.13	1.04	0.86	0.90	0.83	0.76
5.92	1.34	1.37	1.19	0.90	1.03	0.95	0.78	0.74	0.68	0.55	0.40
6.12	1.63	1.40	1.27	1.07	1.09	0.90	0.89	0.85	0.69	0.61	0.51
6.32	1.35	1.29	1.08	0.95	0.93	0.90	0.80	0.71	0.56	0.39	0.48
6.52	1.54	1.40	1.23	1.07	1.06	1.00	0.77	0.72	0.72	0.71	0.77
6.72	1.78	1.59	1.45	1.42	1.31	1.14	1.09	0.94	0.94	0.82	0.69
6.92	1.47	1.33	1.24	1.13	1.01	0.88	0.82	0.73	0.70	0.58	0.41
7.12	1.49	1.36	1.24	0.88	1.01	1.04	0.78	0.70	0.66	0.64	0.59
7.32	1.47	1.43	1.28	1.06	0.97	0.81	0.82	0.73	0.69	0.52	0.57
7.52	1.44	1.32	1.21	1.13	1.01	0.91	0.89	0.81	0.67	0.62	0.51
7.72	1.52	1.41	1.24	1.13	1.08	0.95	0.91	0.75	0.70	0.69	0.53
7.92	1.48	1.28	1.23	1.00	0.97	0.85	0.89	0.68	0.74	0.47	0.58
8.12	1.63	1.43	1.16	1.13	1.15	0.92	0.80	0.81	0.78	0.66	0.57
8.32	1.52	1.35	1.20	1.07	1.10	0.92	0.81	0.79	0.59	0.59	0.59
8.52	1.34	1.26	1.04	1.09	0.94	0.77	0.68	0.69	0.53	0.45	0.45

8.72	1.34	1.24	1.10	1.04	0.79	0.73	0.68	0.70	0.67	0.42	0.35
8.92	1.37	1.32	1.12	0.88	0.89	0.67	0.67	0.66	0.54	0.46	0.46
9.12	1.45	1.31	1.15	1.03	0.99	0.83	0.76	0.69	0.57	0.60	0.43
9.32	1.31	1.28	1.17	1.02	0.83	0.80	0.81	0.68	0.61	0.52	0.49
9.52	1.41	1.38	1.10	0.97	0.96	0.74	0.72	0.75	0.56	0.69	0.31
9.72	1.31	1.25	1.02	0.90	0.86	0.86	0.76	0.56	0.62	0.40	0.45
9.92	1.35	1.24	1.17	0.96	0.99	0.78	0.80	0.75	0.72	0.53	0.45
10.12	1.28	1.39	1.06	1.03	0.77	0.75	0.77	0.58	0.53	0.53	0.43
10.32	1.18	1.17	0.96	0.83	0.82	0.63	0.66	0.69	0.41	0.42	0.52
10.52	1.11	1.22	1.06	0.93	0.84	0.74	0.68	0.51	0.54	0.46	0.31
10.72	1.47	1.33	1.11	1.00	0.94	0.78	0.83	0.78	0.63	0.62	0.52
10.92	1.33	1.26	1.10	0.96	0.90	0.71	0.72	0.74	0.50	0.45	0.42
11.12	1.29	1.18	0.95	0.86	0.89	0.73	0.61	0.53	0.47	0.46	0.38
11.32	1.41	1.36	1.30	1.12	1.05	0.95	0.87	0.75	0.75	0.66	0.62
11.52	1.40	1.17	1.08	0.87	0.88	0.71	0.69	0.64	0.53	0.55	0.32
11.72	1.16	1.19	0.99	0.86	0.84	0.86	0.63	0.67	0.58	0.41	0.34
11.92	1.17	1.00	0.92	0.92	0.80	0.64	0.63	0.47	0.52	0.40	0.36
12.12	1.31	1.27	1.14	0.96	0.93	0.77	0.65	0.56	0.51	0.46	0.41
12.32	1.37	1.18	1.09	1.00	0.95	0.93	0.87	0.64	0.61	0.53	0.40
12.52	1.18	1.11	1.00	0.94	0.75	0.72	0.60	0.54	0.67	0.46	0.34
12.72	1.59	1.41	1.21	1.06	1.03	0.86	0.87	0.85	0.76	0.64	0.56
12.92	1.25	1.22	1.16	0.93	0.91	0.79	0.69	0.59	0.60	0.53	0.48
13.12	1.26	1.15	0.94	0.84	0.93	0.78	0.70	0.52	0.47	0.40	0.29
13.32	1.26	1.11	0.93	0.89	0.83	0.61	0.63	0.48	0.32	0.40	0.18
13.52	1.29	1.18	1.15	0.94	0.89	0.78	0.63	0.68	0.57	0.53	0.42
13.72	1.43	1.34	1.05	0.94	0.85	0.78	0.79	0.82	0.62	0.53	0.48
13.92	1.20	1.17	1.00	0.94	0.86	0.74	0.66	0.67	0.44	0.47	0.34
14.00	1.38	1.24	1.13	0.96	0.95	0.82	0.76	0.67	0.71	0.59	0.53
14.08	1.36	1.20	0.87	0.88	0.86	0.80	0.63	0.56	0.51	0.41	0.36
14.17	1.34	1.12	0.92	0.77	0.71	0.56	0.57	0.67	0.37	0.48	0.38
14.27	1.32	1.22	0.98	0.97	0.91	0.68	0.58	0.53	0.59	0.48	0.32
14.38	1.27	1.21	0.96	0.95	0.88	0.77	0.81	0.66	0.57	0.52	0.32
14.49	1.27	1.06	1.01	0.84	0.86	0.69	0.52	0.56	0.56	0.36	0.33
14.61	1.14	1.08	0.94	0.81	0.73	0.58	0.64	0.52	0.40	0.41	0.26
14.75	1.15	1.10	0.94	0.85	0.72	0.60	0.55	0.40	0.49	0.43	0.27
14.89	1.55	1.36	1.24	1.15	1.01	1.00	0.78	0.69	0.73	0.62	0.54
15.05	1.21	1.08	0.95	0.90	0.84	0.70	0.74	0.66	0.45	0.49	0.43
15.21	1.41	1.32	1.20	1.00	1.00	0.97	0.70	0.61	0.63	0.50	0.48
15.39	1.18	1.27	1.08	0.85	0.91	0.72	0.70	0.62	0.52	0.49	0.37
15.59	1.22	1.09	1.09	0.86	0.80	0.65	0.57	0.42	0.35	0.38	0.30
15.80	1.37	1.22	1.12	1.00	0.85	0.70	0.76	0.64	0.52	0.61	0.40
16.02	1.24	1.13	1.03	0.88	0.79	0.61	0.71	0.68	0.40	0.43	0.29
16.27	1.33	1.14	1.06	0.97	0.98	0.76	0.61	0.60	0.58	0.43	0.31
16.53	1.26	1.11	0.99	0.81	0.75	0.67	0.60	0.47	0.46	0.40	0.30
16.81	1.18	1.06	0.91	0.87	0.73	0.50	0.46	0.55	0.38	0.36	0.35
17.12	1.35	1.20	1.03	0.88	0.94	0.75	0.61	0.49	0.50	0.42	0.41
17.45	1.12	1.02	0.89	0.72	0.65	0.59	0.60	0.41	0.37	0.32	0.22
17.81	1.32	1.15	0.98	0.86	0.82	0.70	0.66	0.54	0.45	0.41	0.41
18.19	1.36	1.21	1.02	0.90	0.86	0.77	0.65	0.53	0.55	0.49	0.38
18.60	1.27	1.31	1.05	1.00	0.86	0.66	0.66	0.54	0.54	0.45	0.49

19.05	1.19	1.21	1.08	1.00	0.85	0.71	0.58	0.56	0.41	0.40	0.40
19.53	1.14	1.23	0.94	0.83	0.75	0.66	0.53	0.41	0.40	0.32	0.28
20.05	1.12	1.02	0.82	0.71	0.68	0.50	0.46	0.41	0.31	0.21	0.27
20.61	1.24	1.10	1.09	0.94	0.83	0.64	0.47	0.59	0.47	0.44	0.25
21.22	1.20	1.18	0.98	0.90	0.86	0.80	0.54	0.47	0.48	0.35	0.37
21.87	1.12	1.01	0.88	0.79	0.72	0.58	0.48	0.45	0.43	0.24	0.06
22.57	1.16	1.05	1.00	0.83	0.72	0.64	0.74	0.55	0.51	0.36	0.34
23.33	1.26	1.08	1.06	0.89	0.88	0.69	0.60	0.44	0.51	0.32	0.23
24.15	1.25	1.16	0.85	0.81	0.67	0.64	0.50	0.42	0.32	0.30	0.29
25.03	1.11	1.08	0.92	0.72	0.75	0.56	0.51	0.45	0.53	0.32	0.26
25.98	1.01	1.01	0.77	0.75	0.78	0.54	0.50	0.45	0.36	0.29	0.17
27.01	1.08	0.99	0.80	0.66	0.63	0.45	0.40	0.36	0.36	0.24	0.12
28.11	1.10	0.97	0.92	0.69	0.69	0.65	0.52	0.41	0.34	0.44	0.26
29.30	1.16	1.04	0.89	0.81	0.74	0.57	0.50	0.50	0.42	0.24	0.33
30.59	0.98	0.98	0.78	0.77	0.71	0.42	0.46	0.38	0.20	0.23	0.16
31.98	0.93	0.82	0.64	0.56	0.60	0.44	0.37	0.27	0.11	0.10	0.08
33.47	1.05	0.89	0.90	0.61	0.61	0.49	0.48	0.39	0.37	0.35	0.16
35.09	1.00	0.96	0.80	0.78	0.66	0.48	0.38	0.33	0.27	0.15	0.17
36.83	1.05	0.95	0.87	0.65	0.61	0.48	0.29	0.31	0.21	0.13	0.05
38.71	1.02	0.97	0.78	0.63	0.63	0.46	0.36	0.15	0.32	0.14	0.18
40.73	1.18	0.94	0.89	0.68	0.70	0.57	0.40	0.32	0.31	0.27	0.12
42.92	1.11	0.91	0.80	0.70	0.62	0.50	0.50	0.36	0.27	0.27	0.16
45.27	1.00	1.09	0.82	0.62	0.55	0.42	0.45	0.45	0.37	0.24	0.14
47.81	1.05	0.95	0.68	0.64	0.58	0.47	0.43	0.22	0.29	0.12	0.09
50.55	0.67	0.73	0.49	0.38	0.40	0.24	0.15	0.20	0.14	-0.01	-0.13
53.51	0.99	0.93	0.72	0.63	0.52	0.57	0.36	0.31	0.24	0.25	0.10
56.69	0.99	0.85	0.58	0.52	0.53	0.33	0.24	0.16	0.13	-0.06	-0.09
60.13	0.72	0.65	0.45	0.46	0.38	0.30	0.14	0.12	0.05	-0.03	-0.10
63.84	0.80	0.65	0.54	0.44	0.42	0.22	0.14	0.03	0.11	-0.02	-0.10
67.83	0.72	0.65	0.55	0.30	0.35	0.17	0.04	0.05	-0.04	-0.11	-0.22
72.15	0.71	0.72	0.53	0.28	0.30	0.13	0.24	0.12	-0.02	-0.11	-0.10
76.80	0.60	0.53	0.42	0.30	0.17	0.04	0.04	-0.04	-0.10	-0.17	-0.24
81.81	0.71	0.72	0.59	0.43	0.36	0.28	0.15	0.10	0.04	0.01	-0.27
87.22	0.81	0.64	0.38	0.29	0.30	0.31	0.05	0.05	0.00	-0.02	-0.20
93.06	0.60	0.57	0.34	0.23	0.13	0.03	-0.08	-0.08	-0.18	-0.18	-0.25
99.35	0.69	0.68	0.34	0.28	0.27	0.09	0.05	0.02	-0.16	-0.15	-0.28
106.13	0.61	0.62	0.30	0.12	0.07	0.01	-0.09	-0.11	-0.06	-0.28	-0.32
113.45	0.81	0.62	0.47	0.46	0.37	0.14	0.09	-0.09	-0.11	-0.08	-0.16
121.34	0.62	0.53	0.43	0.23	0.11	-0.01	-0.12	-0.12	-0.21	-0.36	-0.40
129.86	0.48	0.36	0.08	0.03	0.02	-0.21	-0.28	-0.25	-0.43	-0.49	-0.50
139.04	0.55	0.56	0.36	0.12	0.07	-0.03	-0.08	-0.25	-0.23	-0.29	-0.46
148.94	0.43	0.20	0.09	0.07	-0.02	-0.09	-0.24	-0.23	-0.30	-0.40	-0.49
159.62	0.53	0.39	0.24	0.10	-0.07	-0.12	-0.17	-0.24	-0.29	-0.48	-0.46
171.14	0.62	0.36	0.19	0.02	0.09	-0.25	-0.27	-0.27	-0.35	-0.36	-0.45
183.56	0.44	0.19	0.07	-0.03	-0.09	-0.18	-0.29	-0.32	-0.36	-0.51	-0.57
196.96	0.38	0.17	0.11	0.03	-0.10	-0.18	-0.34	-0.32	-0.40	-0.53	-0.59
211.41	0.34	0.20	0.06	0.03	0.02	-0.19	-0.21	-0.40	-0.37	-0.52	-0.52
226.99	0.33	0.20	0.07	-0.04	-0.03	-0.16	-0.23	-0.36	-0.45	-0.54	-0.64
243.80	0.33	0.10	-0.04	-0.03	-0.20	-0.32	-0.40	-0.43	-0.50	-0.55	-0.56
261.93	0.31	0.21	0.05	-0.05	-0.22	-0.19	-0.33	-0.36	-0.40	-0.47	-0.64

281.48	0.27	0.05	-0.04	-0.21	-0.18	-0.32	-0.37	-0.59	-0.50	-0.70	-0.72
302.56	0.15	0.02	-0.04	-0.19	-0.09	-0.36	-0.47	-0.46	-0.44	-0.54	-0.76
325.30	0.18	0.12	-0.12	-0.15	-0.23	-0.30	-0.32	-0.39	-0.47	-0.58	-0.60
349.83	0.16	0.16	-0.06	-0.17	-0.08	-0.26	-0.40	-0.50	-0.44	-0.52	-0.67
376.28	0.21	0.10	-0.08	-0.23	-0.22	-0.42	-0.52	-0.51	-0.51	-0.64	-0.76
404.81	0.13	0.17	-0.11	-0.20	-0.23	-0.30	-0.41	-0.49	-0.43	-0.63	-0.53
435.58	0.16	0.12	-0.01	-0.17	-0.29	-0.39	-0.42	-0.47	-0.47	-0.53	-0.58
468.76	0.04	0.14	-0.14	-0.27	-0.20	-0.36	-0.41	-0.45	-0.45	-0.53	-0.66
504.55	-0.01	-0.08	-0.10	-0.22	-0.26	-0.40	-0.48	-0.48	-0.66	-0.70	-0.67
543.15	0.14	-0.05	-0.11	-0.05	-0.19	-0.30	-0.33	-0.45	-0.37	-0.54	-0.63
584.78	-0.03	-0.12	-0.10	-0.27	-0.32	-0.47	-0.45	-0.61	-0.78	-0.57	-0.61
629.68	0.18	0.02	-0.13	-0.16	-0.21	-0.22	-0.37	-0.37	-0.55	-0.58	-0.49
678.10	0.14	0.01	-0.19	-0.14	-0.30	-0.39	-0.35	-0.46	-0.48	-0.58	-0.65
730.33	0.32	0.22	-0.02	-0.07	-0.17	-0.25	-0.20	-0.35	-0.38	-0.45	-0.49
786.65	0.20	0.07	-0.14	-0.19	-0.07	-0.18	-0.26	-0.36	-0.42	-0.49	-0.35
847.40	0.06	-0.03	-0.22	-0.19	-0.24	-0.31	-0.40	-0.43	-0.53	-0.57	-0.65
912.92	0.06	0.03	-0.10	-0.17	-0.18	-0.40	-0.40	-0.45	-0.48	-0.49	-0.62
983.58	0.22	0.15	0.04	-0.06	-0.11	-0.22	-0.29	-0.38	-0.43	-0.34	-0.40
1059.78	0.13	0.21	-0.04	-0.04	-0.11	-0.10	-0.17	-0.33	-0.28	-0.34	-0.42
1141.98	0.19	0.07	-0.04	-0.16	-0.15	-0.36	-0.30	-0.29	-0.41	-0.43	-0.47
1230.62	0.10	0.09	-0.08	-0.20	-0.14	-0.16	-0.20	-0.31	-0.33	-0.36	-0.47
1326.23	0.24	0.18	0.05	-0.04	-0.02	-0.17	-0.22	-0.18	-0.17	-0.21	-0.38
1429.34	0.19	0.20	0.02	-0.03	0.03	-0.11	-0.20	-0.24	-0.24	-0.29	-0.35
1540.54	0.17	0.15	0.06	0.07	-0.03	-0.03	-0.15	-0.16	-0.19	-0.22	-0.26
1660.48	0.18	0.08	-0.02	-0.03	-0.12	-0.12	-0.24	-0.30	-0.16	-0.24	-0.23
1789.83	0.22	0.18	0.07	-0.04	-0.06	-0.02	-0.15	-0.20	-0.18	-0.13	-0.20
1929.34	0.33	0.22	0.06	0.06	0.05	-0.04	-0.01	-0.08	-0.15	-0.15	-0.06
2079.80	0.31	0.14	0.12	0.00	0.09	0.01	0.00	-0.01	-0.07	-0.07	-0.20
2242.08	0.29	0.32	0.07	0.04	-0.01	0.07	-0.04	-0.09	-0.06	-0.12	-0.16
2417.10	0.32	0.20	0.07	0.24	0.11	-0.08	-0.01	0.11	-0.04	0.00	-0.14
2605.85	0.32	0.26	0.26	0.20	0.24	0.16	0.12	0.04	0.12	0.05	-0.02
2809.43	0.28	0.24	0.14	0.17	0.12	0.12	0.01	-0.02	-0.04	0.00	-0.01
3028.99	0.26	0.23	0.16	0.17	0.12	0.02	0.13	0.06	0.09	-0.02	0.02
3265.79	0.29	0.33	0.19	0.17	0.19	0.06	0.12	0.10	0.05	0.09	0.12
3521.18	0.31	0.27	0.28	0.17	0.23	0.21	0.27	0.21	0.09	0.19	-0.03
3796.62	0.30	0.24	0.12	0.10	0.21	-0.07	0.06	0.02	0.09	0.05	0.05

Wavelength (nm) / Time (ps)	491.14	492.55	493.96	495.36	496.77	498.18	499.59	501.00	502.41	503.82	505.22
-3.28	-0.14	-0.11	-0.12	-0.15	-0.12	-0.09	-0.18	-0.17	-0.11	-0.04	-0.14
-2.78	-0.16	-0.02	-0.12	-0.03	-0.12	-0.15	-0.12	-0.07	-0.08	-0.10	-0.05
-2.28	-0.02	0.02	0.04	-0.08	0.00	-0.02	0.04	-0.03	0.00	-0.05	-0.08
-1.78	0.31	0.19	0.19	0.12	0.26	0.19	0.19	0.19	0.17	0.24	0.16
-1.28	-0.02	-0.11	-0.06	0.00	-0.05	-0.02	0.03	0.01	-0.07	0.03	-0.06
-0.78	-0.09	-0.12	-0.15	-0.16	-0.08	-0.15	-0.09	-0.15	-0.06	-0.08	-0.03
-0.28	-0.01	-0.02	0.03	0.07	0.00	-0.01	-0.01	0.00	0.04	-0.07	0.11
0.22	0.11	0.12	0.12	0.06	0.09	0.16	0.06	0.11	0.04	0.09	0.12
0.32	-0.07	-0.08	-0.07	-0.02	-0.07	-0.04	-0.09	0.04	-0.05	-0.14	-0.10
0.42	0.10	0.13	0.13	0.18	0.10	0.13	0.17	0.07	0.11	0.12	0.07

0.52	-0.09	-0.02	0.01	0.01	-0.02	-0.01	-0.05	0.04	-0.03	0.01	-0.09
0.62	0.07	0.16	0.05	0.09	0.10	0.01	0.13	0.06	0.08	0.10	0.03
0.72	0.30	0.27	0.19	0.25	0.23	0.27	0.21	0.28	0.20	0.24	0.27
0.77	0.17	0.21	0.06	0.08	0.20	0.11	0.08	0.01	0.10	0.11	0.08
0.82	0.08	0.13	0.04	0.15	0.07	0.06	0.10	0.05	0.10	0.14	0.13
0.87	-0.03	0.04	-0.13	-0.01	-0.09	-0.06	0.08	0.02	-0.04	-0.03	0.00
0.92	-0.05	0.03	0.12	0.13	0.10	0.09	-0.03	0.06	0.10	0.08	0.04
0.97	0.00	-0.08	-0.06	0.04	0.04	-0.02	-0.03	0.01	0.02	-0.08	-0.10
1.02	-0.06	-0.07	-0.11	-0.06	-0.06	-0.13	-0.15	-0.07	-0.10	-0.08	-0.14
1.07	-0.01	0.10	0.10	0.14	0.16	0.08	0.12	0.10	0.17	0.14	0.11
1.12	-0.17	-0.14	-0.24	-0.20	-0.22	-0.23	-0.16	-0.28	-0.14	-0.10	-0.21
1.17	-0.17	-0.10	-0.15	-0.11	-0.05	-0.06	-0.04	-0.04	-0.22	-0.11	-0.19
1.22	-0.03	-0.01	0.00	0.02	0.01	-0.01	0.06	-0.04	-0.04	0.09	-0.03
1.27	0.03	0.05	0.01	0.10	0.05	0.05	0.09	0.04	0.02	0.06	0.03
1.32	-0.30	-0.34	-0.29	-0.23	-0.23	-0.20	-0.20	-0.13	-0.09	0.00	-0.12
1.37	0.31	0.15	0.07	0.01	-0.09	-0.27	-0.18	-0.27	-0.34	-0.28	-0.29
1.42	2.23	2.14	1.84	1.66	1.61	1.44	1.33	1.32	1.15	1.11	0.88
1.47	0.22	0.28	0.14	0.30	0.37	0.43	0.63	0.70	0.92	1.20	1.30
1.52	-1.19	-1.27	-1.42	-1.42	-1.44	-1.38	-1.33	-1.23	-1.04	-0.77	-0.52
1.57	-1.74	-1.90	-2.07	-2.19	-2.33	-2.27	-2.29	-2.29	-2.12	-1.88	-1.70
1.62	-1.72	-2.00	-2.11	-2.33	-2.43	-2.54	-2.47	-2.46	-2.35	-2.20	-2.04
1.67	-1.88	-2.18	-2.38	-2.50	-2.65	-2.64	-2.59	-2.57	-2.42	-2.25	-2.16
1.72	-1.87	-2.04	-2.27	-2.36	-2.47	-2.50	-2.48	-2.38	-2.34	-2.11	-2.01
1.77	-1.82	-2.03	-2.16	-2.23	-2.37	-2.42	-2.26	-2.25	-2.20	-2.00	-1.79
1.82	-1.61	-1.76	-1.98	-2.10	-2.20	-2.22	-2.15	-2.04	-1.96	-1.78	-1.61
1.87	-1.45	-1.63	-1.73	-1.85	-1.86	-1.95	-1.90	-1.84	-1.68	-1.55	-1.36
1.92	-1.51	-1.69	-1.90	-1.95	-2.00	-2.05	-1.96	-1.92	-1.79	-1.56	-1.38
1.97	-1.57	-1.79	-1.93	-1.97	-2.14	-2.14	-1.98	-2.03	-1.85	-1.56	-1.40
2.02	-1.45	-1.61	-1.80	-1.88	-1.92	-1.89	-1.76	-1.87	-1.58	-1.41	-1.24
2.07	-1.59	-1.77	-1.84	-1.80	-1.93	-2.01	-1.92	-1.84	-1.72	-1.48	-1.24
2.12	-1.51	-1.55	-1.64	-1.84	-1.83	-1.87	-1.79	-1.70	-1.55	-1.21	-1.04
2.17	-1.25	-1.46	-1.47	-1.53	-1.57	-1.61	-1.49	-1.43	-1.34	-1.01	-0.85
2.22	-1.33	-1.46	-1.76	-1.64	-1.73	-1.77	-1.61	-1.50	-1.35	-1.05	-0.92
2.27	-1.18	-1.38	-1.55	-1.52	-1.58	-1.58	-1.57	-1.43	-1.18	-0.98	-0.76
2.32	-1.27	-1.26	-1.52	-1.59	-1.65	-1.63	-1.49	-1.39	-1.17	-1.00	-0.76
2.37	-1.45	-1.52	-1.71	-1.75	-1.85	-1.71	-1.65	-1.50	-1.35	-1.19	-0.87
2.42	-1.34	-1.31	-1.61	-1.58	-1.65	-1.61	-1.52	-1.41	-1.15	-0.96	-0.68
2.47	-1.26	-1.47	-1.48	-1.60	-1.69	-1.57	-1.53	-1.40	-1.26	-0.94	-0.73
2.52	-1.32	-1.45	-1.40	-1.60	-1.69	-1.55	-1.52	-1.33	-1.26	-0.94	-0.69
2.57	-1.36	-1.48	-1.58	-1.65	-1.74	-1.68	-1.62	-1.30	-1.27	-0.99	-0.77
2.62	-1.00	-1.15	-1.36	-1.32	-1.32	-1.34	-1.25	-1.11	-0.87	-0.58	-0.35
2.67	-1.33	-1.39	-1.53	-1.44	-1.52	-1.63	-1.46	-1.41	-1.14	-0.95	-0.64
2.72	-1.14	-1.16	-1.44	-1.51	-1.42	-1.42	-1.29	-1.17	-1.01	-0.73	-0.48
2.77	-1.04	-1.18	-1.31	-1.36	-1.31	-1.25	-1.19	-1.08	-0.84	-0.58	-0.30
2.82	-1.21	-1.30	-1.36	-1.43	-1.50	-1.36	-1.26	-1.24	-0.85	-0.62	-0.46
2.87	-1.33	-1.37	-1.47	-1.62	-1.63	-1.50	-1.36	-1.25	-1.06	-0.79	-0.51
2.92	-1.25	-1.26	-1.43	-1.46	-1.48	-1.39	-1.26	-1.21	-1.10	-0.74	-0.43
2.97	-1.12	-1.31	-1.47	-1.45	-1.50	-1.49	-1.31	-1.20	-1.06	-0.81	-0.47
3.02	-1.21	-1.26	-1.38	-1.45	-1.50	-1.44	-1.38	-1.27	-1.06	-0.72	-0.48
3.07	-1.20	-1.37	-1.50	-1.54	-1.59	-1.46	-1.36	-1.29	-1.00	-0.79	-0.53

3.12	-1.26	-1.34	-1.49	-1.55	-1.57	-1.44	-1.35	-1.22	-0.98	-0.62	-0.37
3.17	-1.13	-1.31	-1.50	-1.45	-1.48	-1.37	-1.28	-1.21	-0.95	-0.57	-0.34
3.22	-1.20	-1.28	-1.44	-1.40	-1.45	-1.38	-1.24	-1.16	-0.92	-0.60	-0.43
3.27	-1.16	-1.31	-1.31	-1.38	-1.43	-1.40	-1.35	-1.14	-0.92	-0.59	-0.35
3.32	-1.21	-1.31	-1.43	-1.54	-1.53	-1.40	-1.32	-1.15	-0.93	-0.63	-0.38
3.37	-1.37	-1.49	-1.63	-1.60	-1.65	-1.58	-1.45	-1.30	-1.14	-0.76	-0.48
3.42	-1.16	-1.29	-1.42	-1.47	-1.48	-1.35	-1.24	-1.17	-0.91	-0.59	-0.28
3.47	-1.31	-1.41	-1.62	-1.58	-1.55	-1.52	-1.40	-1.26	-1.03	-0.78	-0.55
3.52	-1.33	-1.43	-1.54	-1.64	-1.57	-1.53	-1.39	-1.33	-1.09	-0.75	-0.46
3.57	-1.18	-1.36	-1.50	-1.43	-1.41	-1.37	-1.33	-1.21	-1.00	-0.67	-0.38
3.62	-1.20	-1.23	-1.36	-1.42	-1.34	-1.37	-1.21	-1.00	-0.84	-0.50	-0.19
3.67	-1.28	-1.40	-1.45	-1.52	-1.49	-1.48	-1.29	-1.26	-1.03	-0.69	-0.49
3.72	-1.10	-1.26	-1.35	-1.40	-1.41	-1.32	-1.22	-1.00	-0.85	-0.60	-0.24
3.92	-1.20	-1.35	-1.42	-1.53	-1.41	-1.37	-1.21	-1.12	-0.86	-0.46	-0.34
4.12	-1.23	-1.38	-1.38	-1.52	-1.57	-1.45	-1.29	-1.19	-0.95	-0.61	-0.31
4.32	-1.21	-1.33	-1.39	-1.37	-1.51	-1.37	-1.20	-1.00	-0.82	-0.58	-0.26
4.52	-1.22	-1.38	-1.44	-1.53	-1.46	-1.45	-1.23	-1.00	-0.84	-0.52	-0.26
4.72	-1.25	-1.45	-1.56	-1.55	-1.47	-1.39	-1.24	-1.16	-0.91	-0.64	-0.30
4.92	-1.14	-1.19	-1.44	-1.48	-1.37	-1.30	-1.17	-0.98	-0.75	-0.38	-0.12
5.12	-1.16	-1.26	-1.34	-1.35	-1.44	-1.24	-1.17	-0.97	-0.83	-0.48	-0.14
5.32	-1.15	-1.25	-1.34	-1.25	-1.32	-1.32	-1.08	-1.08	-0.74	-0.40	-0.11
5.52	-1.07	-1.12	-1.29	-1.28	-1.31	-1.25	-1.06	-0.88	-0.62	-0.29	-0.13
5.72	-1.04	-1.29	-1.35	-1.28	-1.36	-1.22	-1.14	-0.98	-0.79	-0.41	-0.01
5.92	-1.24	-1.48	-1.55	-1.60	-1.57	-1.43	-1.34	-1.15	-0.88	-0.61	-0.24
6.12	-1.24	-1.41	-1.44	-1.38	-1.38	-1.36	-1.19	-1.01	-0.83	-0.44	-0.24
6.32	-1.26	-1.43	-1.54	-1.44	-1.59	-1.44	-1.27	-1.15	-0.89	-0.60	-0.32
6.52	-1.06	-1.26	-1.35	-1.44	-1.34	-1.46	-1.17	-0.97	-0.67	-0.26	0.01
6.72	-1.08	-1.13	-1.30	-1.35	-1.29	-1.17	-1.05	-0.89	-0.68	-0.40	-0.02
6.92	-1.28	-1.42	-1.48	-1.48	-1.47	-1.42	-1.29	-0.99	-0.85	-0.45	-0.25
7.12	-1.13	-1.35	-1.50	-1.49	-1.50	-1.38	-1.17	-1.04	-0.80	-0.34	-0.12
7.32	-1.25	-1.34	-1.48	-1.55	-1.59	-1.36	-1.32	-1.08	-0.82	-0.47	-0.19
7.52	-1.26	-1.34	-1.48	-1.46	-1.40	-1.38	-1.30	-1.06	-0.68	-0.44	-0.13
7.72	-1.24	-1.37	-1.50	-1.51	-1.50	-1.45	-1.16	-1.04	-0.82	-0.43	-0.13
7.92	-1.24	-1.27	-1.54	-1.54	-1.43	-1.39	-1.20	-1.10	-0.79	-0.47	-0.15
8.12	-1.28	-1.30	-1.44	-1.45	-1.43	-1.29	-1.16	-0.97	-0.76	-0.35	-0.05
8.32	-1.24	-1.28	-1.47	-1.39	-1.41	-1.29	-1.12	-0.96	-0.68	-0.36	-0.07
8.52	-1.34	-1.30	-1.40	-1.50	-1.53	-1.42	-1.23	-1.12	-0.80	-0.46	-0.18
8.72	-1.42	-1.53	-1.60	-1.71	-1.58	-1.58	-1.39	-1.26	-0.91	-0.64	-0.26
8.92	-1.38	-1.53	-1.59	-1.65	-1.71	-1.46	-1.36	-1.19	-0.91	-0.60	-0.36
9.12	-1.32	-1.46	-1.51	-1.55	-1.51	-1.47	-1.21	-1.10	-0.84	-0.46	-0.16
9.32	-1.30	-1.43	-1.39	-1.51	-1.52	-1.39	-1.28	-1.09	-0.87	-0.47	-0.21
9.52	-1.39	-1.53	-1.58	-1.60	-1.61	-1.47	-1.31	-1.21	-0.97	-0.53	-0.28
9.72	-1.23	-1.41	-1.46	-1.53	-1.42	-1.42	-1.28	-1.12	-0.81	-0.39	-0.14
9.92	-1.15	-1.25	-1.25	-1.33	-1.36	-1.27	-1.09	-0.89	-0.68	-0.32	0.03
10.12	-1.37	-1.49	-1.61	-1.54	-1.67	-1.55	-1.37	-1.11	-0.83	-0.51	-0.29
10.32	-1.27	-1.42	-1.46	-1.51	-1.51	-1.41	-1.26	-1.09	-0.90	-0.57	-0.17
10.52	-1.31	-1.60	-1.68	-1.63	-1.62	-1.51	-1.37	-1.19	-0.92	-0.57	-0.22
10.72	-1.25	-1.47	-1.49	-1.48	-1.51	-1.42	-1.20	-1.14	-0.82	-0.56	-0.09
10.92	-1.25	-1.37	-1.48	-1.48	-1.44	-1.34	-1.24	-1.07	-0.75	-0.47	-0.07
11.12	-1.38	-1.44	-1.64	-1.56	-1.56	-1.53	-1.40	-1.11	-0.74	-0.52	-0.21

11.32	-1.14	-1.23	-1.36	-1.43	-1.32	-1.30	-1.08	-0.93	-0.67	-0.27	-0.08
11.52	-1.31	-1.42	-1.50	-1.58	-1.52	-1.37	-1.36	-1.16	-0.84	-0.59	-0.17
11.72	-1.26	-1.34	-1.47	-1.46	-1.45	-1.40	-1.22	-1.10	-0.82	-0.43	-0.15
11.92	-1.37	-1.55	-1.59	-1.69	-1.64	-1.60	-1.47	-1.17	-0.87	-0.55	-0.27
12.12	-1.51	-1.61	-1.65	-1.58	-1.68	-1.58	-1.52	-1.22	-0.89	-0.52	-0.21
12.32	-1.32	-1.39	-1.54	-1.51	-1.58	-1.56	-1.38	-1.11	-0.81	-0.48	-0.18
12.52	-1.38	-1.45	-1.58	-1.62	-1.49	-1.50	-1.38	-1.23	-0.87	-0.58	-0.16
12.72	-1.18	-1.18	-1.29	-1.32	-1.24	-1.24	-1.03	-0.94	-0.59	-0.28	-0.03
12.92	-1.34	-1.40	-1.53	-1.42	-1.45	-1.40	-1.20	-0.98	-0.83	-0.41	-0.14
13.12	-1.34	-1.40	-1.58	-1.61	-1.54	-1.43	-1.33	-1.15	-0.87	-0.54	-0.12
13.32	-1.48	-1.61	-1.72	-1.74	-1.69	-1.59	-1.50	-1.34	-0.92	-0.53	-0.24
13.52	-1.24	-1.37	-1.41	-1.49	-1.41	-1.30	-1.18	-1.04	-0.80	-0.49	-0.13
13.72	-1.24	-1.24	-1.49	-1.45	-1.41	-1.38	-1.20	-0.92	-0.69	-0.37	0.03
13.92	-1.36	-1.51	-1.61	-1.72	-1.64	-1.52	-1.41	-1.19	-0.91	-0.52	-0.18
14.00	-1.36	-1.43	-1.62	-1.51	-1.60	-1.51	-1.30	-1.08	-0.69	-0.40	-0.09
14.08	-1.38	-1.49	-1.52	-1.58	-1.61	-1.45	-1.24	-1.04	-0.84	-0.43	-0.12
14.17	-1.37	-1.53	-1.55	-1.57	-1.64	-1.47	-1.29	-1.13	-0.98	-0.61	-0.17
14.27	-1.47	-1.56	-1.72	-1.72	-1.71	-1.58	-1.46	-1.20	-0.93	-0.61	-0.27
14.38	-1.25	-1.42	-1.54	-1.58	-1.54	-1.51	-1.33	-1.08	-0.78	-0.43	-0.14
14.49	-1.37	-1.50	-1.58	-1.51	-1.59	-1.47	-1.35	-1.20	-0.91	-0.53	-0.11
14.61	-1.52	-1.57	-1.71	-1.66	-1.67	-1.57	-1.36	-1.15	-1.03	-0.61	-0.38
14.75	-1.44	-1.57	-1.73	-1.65	-1.64	-1.69	-1.42	-1.21	-0.87	-0.60	-0.29
14.89	-1.18	-1.31	-1.40	-1.42	-1.40	-1.33	-1.19	-0.96	-0.66	-0.27	0.13
15.05	-1.33	-1.53	-1.54	-1.58	-1.54	-1.52	-1.39	-1.12	-0.80	-0.54	-0.23
15.21	-1.31	-1.39	-1.46	-1.61	-1.42	-1.32	-1.29	-1.00	-0.79	-0.51	-0.08
15.39	-1.22	-1.29	-1.37	-1.42	-1.47	-1.28	-1.11	-1.11	-0.72	-0.32	-0.04
15.59	-1.43	-1.60	-1.67	-1.72	-1.72	-1.60	-1.31	-1.20	-0.94	-0.59	-0.19
15.80	-1.26	-1.52	-1.53	-1.55	-1.54	-1.56	-1.34	-1.11	-0.89	-0.41	-0.18
16.02	-1.31	-1.43	-1.47	-1.49	-1.41	-1.50	-1.21	-1.03	-0.84	-0.45	-0.10
16.27	-1.39	-1.47	-1.58	-1.54	-1.57	-1.45	-1.26	-1.13	-0.83	-0.43	-0.08
16.53	-1.45	-1.57	-1.75	-1.65	-1.63	-1.60	-1.43	-1.19	-0.89	-0.62	-0.26
16.81	-1.46	-1.63	-1.74	-1.68	-1.67	-1.59	-1.44	-1.20	-0.97	-0.72	-0.31
17.12	-1.36	-1.55	-1.61	-1.65	-1.65	-1.50	-1.33	-1.22	-0.81	-0.49	-0.17
17.45	-1.40	-1.50	-1.60	-1.62	-1.67	-1.46	-1.39	-1.19	-0.88	-0.56	-0.26
17.81	-1.39	-1.59	-1.76	-1.77	-1.57	-1.63	-1.40	-1.22	-0.91	-0.52	-0.16
18.19	-1.47	-1.55	-1.70	-1.77	-1.62	-1.63	-1.44	-1.19	-0.91	-0.47	-0.18
18.60	-1.50	-1.57	-1.60	-1.60	-1.62	-1.54	-1.34	-1.15	-0.84	-0.38	-0.07
19.05	-1.48	-1.55	-1.68	-1.69	-1.62	-1.54	-1.46	-1.31	-0.92	-0.49	-0.21
19.53	-1.60	-1.64	-1.74	-1.64	-1.74	-1.66	-1.47	-1.33	-0.98	-0.63	-0.32
20.05	-1.56	-1.73	-1.75	-1.80	-1.74	-1.75	-1.47	-1.33	-1.05	-0.60	-0.28
20.61	-1.46	-1.55	-1.67	-1.75	-1.66	-1.56	-1.41	-1.18	-0.84	-0.50	-0.16
21.22	-1.33	-1.53	-1.57	-1.62	-1.57	-1.49	-1.38	-1.13	-0.96	-0.50	-0.17
21.87	-1.56	-1.74	-1.76	-1.80	-1.84	-1.74	-1.53	-1.34	-0.91	-0.65	-0.30
22.57	-1.44	-1.57	-1.61	-1.66	-1.57	-1.43	-1.30	-1.29	-0.90	-0.49	-0.18
23.33	-1.56	-1.64	-1.67	-1.72	-1.63	-1.71	-1.42	-1.25	-1.04	-0.59	-0.26
24.15	-1.52	-1.64	-1.69	-1.76	-1.72	-1.52	-1.46	-1.20	-0.94	-0.60	-0.28
25.03	-1.55	-1.66	-1.80	-1.73	-1.75	-1.63	-1.50	-1.25	-0.94	-0.57	-0.33
25.98	-1.58	-1.66	-1.81	-1.88	-1.74	-1.71	-1.53	-1.25	-1.03	-0.57	-0.30
27.01	-1.64	-1.76	-1.84	-1.88	-1.76	-1.73	-1.50	-1.39	-1.02	-0.70	-0.31
28.11	-1.55	-1.70	-1.78	-1.68	-1.78	-1.59	-1.35	-1.19	-0.93	-0.55	-0.23

29.30	-1.64	-1.75	-1.76	-1.79	-1.73	-1.65	-1.49	-1.34	-1.11	-0.66	-0.31
30.59	-1.56	-1.69	-1.75	-1.77	-1.81	-1.76	-1.56	-1.34	-1.02	-0.62	-0.36
31.98	-1.72	-1.92	-2.03	-1.98	-1.97	-1.83	-1.65	-1.43	-1.17	-0.89	-0.46
33.47	-1.64	-1.79	-1.88	-1.87	-1.82	-1.75	-1.58	-1.36	-1.14	-0.65	-0.30
35.09	-1.66	-1.79	-1.89	-1.80	-1.90	-1.65	-1.68	-1.34	-1.05	-0.73	-0.35
36.83	-1.74	-1.82	-1.91	-2.01	-1.94	-1.83	-1.63	-1.48	-1.21	-0.76	-0.46
38.71	-1.60	-1.76	-1.86	-1.80	-1.70	-1.75	-1.55	-1.35	-1.04	-0.64	-0.32
40.73	-1.58	-1.72	-1.76	-1.70	-1.76	-1.68	-1.53	-1.30	-0.97	-0.62	-0.19
42.92	-1.67	-1.76	-1.94	-1.78	-1.84	-1.71	-1.59	-1.33	-1.06	-0.70	-0.36
45.27	-1.68	-1.74	-1.82	-1.92	-1.88	-1.76	-1.56	-1.44	-1.12	-0.74	-0.40
47.81	-1.72	-1.84	-2.01	-1.88	-1.99	-1.91	-1.65	-1.49	-1.14	-0.80	-0.37
50.55	-1.80	-1.81	-2.03	-2.06	-2.01	-1.93	-1.74	-1.55	-1.26	-0.92	-0.54
53.51	-1.76	-1.90	-2.00	-1.92	-1.89	-1.85	-1.65	-1.47	-1.20	-0.80	-0.39
56.69	-1.85	-2.00	-2.14	-2.09	-2.15	-2.00	-1.83	-1.63	-1.32	-0.90	-0.64
60.13	-1.92	-2.05	-2.13	-2.19	-2.09	-2.10	-1.87	-1.68	-1.33	-1.03	-0.64
63.84	-1.94	-1.99	-2.07	-2.05	-2.03	-1.96	-1.78	-1.59	-1.35	-0.89	-0.54
67.83	-2.07	-2.19	-2.33	-2.32	-2.25	-2.11	-2.07	-1.79	-1.42	-1.03	-0.70
72.15	-2.07	-2.09	-2.28	-2.22	-2.16	-2.04	-1.94	-1.77	-1.47	-1.01	-0.72
76.80	-2.04	-2.18	-2.40	-2.27	-2.31	-2.18	-1.98	-1.76	-1.42	-1.10	-0.74
81.81	-2.00	-2.06	-2.23	-2.20	-2.18	-2.08	-1.98	-1.70	-1.39	-0.96	-0.57
87.22	-1.94	-1.98	-2.14	-2.11	-2.17	-2.02	-1.80	-1.67	-1.34	-0.92	-0.57
93.06	-2.05	-2.25	-2.37	-2.35	-2.39	-2.25	-1.99	-1.81	-1.55	-1.13	-0.82
99.35	-2.04	-2.18	-2.28	-2.29	-2.24	-2.18	-2.03	-1.70	-1.51	-1.14	-0.74
106.13	-2.31	-2.32	-2.41	-2.43	-2.40	-2.39	-2.19	-1.95	-1.63	-1.18	-0.85
113.45	-2.09	-2.18	-2.25	-2.33	-2.29	-2.22	-2.01	-1.76	-1.51	-1.02	-0.64
121.34	-2.23	-2.31	-2.51	-2.53	-2.47	-2.40	-2.26	-1.95	-1.66	-1.28	-0.88
129.86	-2.20	-2.40	-2.50	-2.49	-2.57	-2.47	-2.22	-2.08	-1.68	-1.29	-0.97
139.04	-2.12	-2.31	-2.37	-2.48	-2.38	-2.29	-2.07	-1.97	-1.64	-1.20	-0.88
148.94	-2.39	-2.40	-2.45	-2.65	-2.56	-2.40	-2.19	-2.07	-1.83	-1.35	-1.00
159.62	-2.28	-2.43	-2.51	-2.50	-2.55	-2.39	-2.22	-1.98	-1.67	-1.39	-0.87
171.14	-2.36	-2.48	-2.53	-2.56	-2.58	-2.45	-2.25	-2.04	-1.66	-1.36	-0.89
183.56	-2.42	-2.49	-2.68	-2.67	-2.56	-2.50	-2.25	-2.14	-1.81	-1.45	-1.10
196.96	-2.46	-2.50	-2.67	-2.59	-2.63	-2.58	-2.31	-2.08	-1.77	-1.44	-1.02
211.41	-2.42	-2.45	-2.50	-2.58	-2.54	-2.50	-2.26	-2.04	-1.70	-1.27	-0.98
226.99	-2.39	-2.45	-2.68	-2.63	-2.66	-2.61	-2.41	-2.07	-1.80	-1.45	-1.06
243.80	-2.37	-2.52	-2.67	-2.64	-2.57	-2.48	-2.47	-2.16	-1.85	-1.46	-1.09
261.93	-2.44	-2.55	-2.67	-2.68	-2.71	-2.52	-2.43	-2.19	-1.85	-1.43	-1.06
281.48	-2.62	-2.77	-2.81	-2.99	-2.87	-2.71	-2.51	-2.30	-2.04	-1.56	-1.16
302.56	-2.48	-2.56	-2.75	-2.69	-2.71	-2.65	-2.40	-2.25	-1.95	-1.48	-1.13
325.30	-2.34	-2.59	-2.65	-2.59	-2.53	-2.54	-2.29	-2.04	-1.78	-1.41	-1.04
349.83	-2.41	-2.51	-2.63	-2.74	-2.59	-2.53	-2.30	-2.10	-1.74	-1.39	-0.98
376.28	-2.46	-2.60	-2.77	-2.68	-2.62	-2.59	-2.42	-2.17	-1.91	-1.46	-1.10
404.81	-2.25	-2.38	-2.51	-2.44	-2.49	-2.32	-2.15	-1.97	-1.69	-1.34	-0.88
435.58	-2.39	-2.53	-2.58	-2.59	-2.57	-2.54	-2.29	-2.12	-1.74	-1.42	-1.02
468.76	-2.36	-2.43	-2.60	-2.64	-2.53	-2.50	-2.22	-2.04	-1.74	-1.41	-0.97
504.55	-2.36	-2.48	-2.58	-2.50	-2.50	-2.39	-2.28	-2.04	-1.79	-1.36	-1.01
543.15	-2.24	-2.40	-2.50	-2.49	-2.47	-2.38	-2.30	-2.02	-1.72	-1.33	-0.99
584.78	-2.34	-2.46	-2.55	-2.51	-2.46	-2.50	-2.29	-2.05	-1.73	-1.41	-1.00
629.68	-2.14	-2.31	-2.27	-2.33	-2.32	-2.24	-2.04	-1.92	-1.62	-1.18	-0.87
678.10	-2.31	-2.46	-2.65	-2.67	-2.57	-2.53	-2.27	-2.00	-1.75	-1.29	-1.08

730.33	-2.28	-2.23	-2.39	-2.37	-2.36	-2.22	-1.99	-1.79	-1.54	-1.14	-0.75
786.65	-2.13	-2.22	-2.27	-2.21	-2.31	-2.13	-1.99	-1.77	-1.43	-1.08	-0.68
847.40	-2.26	-2.28	-2.41	-2.39	-2.48	-2.29	-2.19	-1.85	-1.58	-1.23	-0.94
912.92	-2.10	-2.22	-2.38	-2.38	-2.18	-2.14	-1.97	-1.72	-1.43	-1.01	-0.73
983.58	-2.04	-2.28	-2.35	-2.24	-2.28	-2.13	-2.03	-1.83	-1.51	-1.06	-0.70
1059.78	-2.02	-2.04	-2.17	-2.20	-2.26	-2.00	-1.84	-1.60	-1.37	-1.00	-0.67
1141.98	-1.83	-2.06	-2.20	-2.11	-2.26	-2.04	-1.90	-1.63	-1.35	-0.93	-0.68
1230.62	-1.79	-1.95	-2.07	-2.04	-2.01	-1.89	-1.73	-1.45	-1.20	-0.87	-0.56
1326.23	-1.79	-1.91	-1.95	-1.93	-2.00	-1.76	-1.74	-1.53	-1.26	-0.76	-0.41
1429.34	-1.78	-1.83	-1.99	-1.85	-1.89	-1.73	-1.65	-1.42	-1.19	-0.68	-0.37
1540.54	-1.62	-1.69	-1.78	-1.85	-1.72	-1.79	-1.52	-1.31	-1.00	-0.63	-0.33
1660.48	-1.68	-1.83	-1.86	-1.93	-1.85	-1.79	-1.53	-1.39	-1.11	-0.70	-0.36
1789.83	-1.60	-1.77	-1.82	-1.77	-1.78	-1.69	-1.58	-1.23	-1.03	-0.62	-0.32
1929.34	-1.37	-1.59	-1.70	-1.64	-1.51	-1.50	-1.26	-1.06	-0.79	-0.45	-0.12
2079.80	-1.42	-1.49	-1.58	-1.50	-1.54	-1.41	-1.30	-1.05	-0.76	-0.40	-0.09
2242.08	-1.42	-1.40	-1.58	-1.50	-1.48	-1.43	-1.25	-1.08	-0.78	-0.37	-0.05
2417.10	-1.31	-1.42	-1.35	-1.52	-1.48	-1.21	-1.10	-0.95	-0.65	-0.27	-0.04
2605.85	-1.13	-1.24	-1.38	-1.29	-1.26	-1.22	-0.99	-0.86	-0.60	-0.21	0.00
2809.43	-1.09	-1.15	-1.28	-1.20	-1.15	-1.06	-0.96	-0.77	-0.54	-0.20	0.12
3028.99	-1.14	-1.25	-1.40	-1.35	-1.35	-1.17	-1.07	-0.74	-0.50	-0.20	0.01
3265.79	-1.02	-1.16	-1.11	-1.19	-1.10	-1.01	-0.80	-0.68	-0.44	-0.09	0.21
3521.18	-0.96	-1.02	-1.04	-1.03	-1.00	-0.98	-0.81	-0.59	-0.37	-0.03	0.24
3796.62	-1.05	-1.13	-1.26	-1.13	-1.16	-1.11	-0.94	-0.78	-0.55	-0.22	0.08

Wavelength (nm) / Time (ps)	506.63	508.04	509.45	510.86	512.27	513.67	515.08	516.49	517.90	519.31	520.72
-3.28	-0.17	-0.06	-0.13	-0.12	-0.13	-0.20	-0.17	-0.15	-0.20	-0.11	-0.09
-2.78	-0.12	-0.07	-0.14	-0.04	-0.08	-0.09	-0.12	-0.10	-0.06	-0.10	-0.05
-2.28	0.03	-0.02	0.01	-0.01	-0.03	0.04	0.00	0.04	0.00	-0.04	-0.02
-1.78	0.18	0.18	0.23	0.13	0.22	0.26	0.24	0.20	0.19	0.20	0.25
-1.28	-0.02	0.06	-0.01	0.00	0.02	0.03	0.06	0.01	-0.02	0.04	-0.01
-0.78	-0.07	-0.10	-0.15	-0.08	-0.08	-0.10	-0.08	-0.14	-0.14	-0.08	-0.14
-0.28	-0.02	-0.01	0.02	0.04	0.03	-0.03	0.02	0.04	0.05	0.09	-0.01
0.22	0.21	0.11	0.12	0.12	0.13	0.11	0.06	0.15	0.14	0.05	0.08
0.32	-0.05	-0.14	-0.06	-0.09	-0.14	-0.12	-0.09	-0.12	-0.07	-0.06	-0.11
0.42	0.03	0.05	0.10	0.05	0.05	0.11	0.06	0.06	0.09	0.02	0.10
0.52	0.04	-0.07	0.03	0.04	-0.02	0.04	-0.02	-0.05	-0.08	-0.02	-0.05
0.62	0.03	-0.06	0.04	0.03	-0.01	0.03	0.05	0.06	-0.01	0.02	-0.03
0.72	0.29	0.26	0.29	0.20	0.20	0.25	0.26	0.28	0.27	0.27	0.25
0.77	0.12	0.05	0.10	0.02	0.12	0.06	0.10	0.17	0.11	0.08	0.04
0.82	0.04	0.07	0.11	0.11	0.05	0.10	0.15	0.12	0.16	0.17	0.13
0.87	-0.04	-0.05	-0.01	-0.01	-0.08	-0.02	0.02	-0.02	-0.03	-0.01	-0.08
0.92	0.09	0.03	0.09	0.07	0.00	0.12	0.05	0.13	0.04	0.04	0.13
0.97	-0.08	-0.07	-0.04	-0.01	-0.06	-0.06	-0.09	0.05	0.03	-0.04	-0.04
1.02	-0.13	-0.10	-0.07	-0.10	-0.17	-0.10	-0.16	-0.05	-0.08	-0.14	-0.13
1.07	0.11	0.13	0.09	0.14	0.06	0.13	0.24	0.08	0.18	0.12	0.10
1.12	-0.22	-0.21	-0.20	-0.12	-0.22	-0.13	-0.23	-0.22	-0.21	-0.21	-0.17
1.17	-0.10	-0.24	-0.13	-0.09	-0.16	-0.07	-0.06	-0.04	-0.14	-0.15	-0.17
1.22	0.01	-0.01	-0.01	0.00	0.00	-0.02	-0.02	-0.07	-0.02	-0.03	-0.03

1.27	0.04	0.08	0.12	0.06	0.05	0.12	0.12	0.12	0.07	0.03	0.12
1.32	-0.06	-0.06	-0.02	-0.09	0.00	-0.01	-0.04	-0.03	0.04	0.02	0.01
1.37	-0.35	-0.38	-0.36	-0.33	-0.29	-0.30	-0.23	-0.25	-0.26	-0.21	-0.19
1.42	0.93	0.78	0.66	0.57	0.38	0.29	0.26	0.18	0.02	-0.11	-0.10
1.47	1.60	1.78	1.96	2.16	2.13	2.17	2.17	2.08	2.00	1.89	1.71
1.52	-0.20	0.06	0.32	0.58	0.81	1.05	1.19	1.35	1.42	1.44	1.51
1.57	-1.46	-1.16	-0.96	-0.68	-0.46	-0.26	-0.10	0.09	0.08	0.15	0.14
1.62	-1.85	-1.59	-1.37	-1.16	-1.05	-0.85	-0.70	-0.54	-0.54	-0.56	-0.67
1.67	-1.90	-1.77	-1.49	-1.36	-1.16	-0.99	-0.85	-0.70	-0.80	-0.84	-0.94
1.72	-1.72	-1.56	-1.29	-1.03	-0.96	-0.83	-0.62	-0.50	-0.55	-0.64	-0.71
1.77	-1.60	-1.42	-1.07	-0.86	-0.66	-0.51	-0.42	-0.33	-0.27	-0.42	-0.43
1.82	-1.33	-1.14	-0.95	-0.67	-0.51	-0.28	-0.18	-0.18	-0.12	-0.16	-0.20
1.87	-1.14	-0.85	-0.59	-0.40	-0.24	-0.01	0.12	0.16	0.18	0.20	0.06
1.92	-1.15	-0.96	-0.60	-0.39	-0.19	0.00	0.17	0.26	0.24	0.31	0.13
1.97	-1.14	-0.95	-0.62	-0.26	-0.16	0.01	0.13	0.26	0.29	0.28	0.22
2.02	-0.92	-0.66	-0.41	-0.17	0.10	0.23	0.44	0.57	0.63	0.60	0.55
2.07	-0.96	-0.75	-0.39	-0.26	0.00	0.23	0.33	0.50	0.55	0.54	0.48
2.12	-0.75	-0.45	-0.15	0.12	0.35	0.57	0.70	0.86	0.97	0.91	0.90
2.17	-0.52	-0.27	0.01	0.34	0.47	0.78	0.82	1.01	1.06	1.07	1.04
2.22	-0.62	-0.48	-0.01	0.22	0.46	0.62	0.90	0.98	1.02	1.02	1.01
2.27	-0.47	-0.15	0.08	0.48	0.63	0.89	1.06	1.15	1.22	1.26	1.19
2.32	-0.46	-0.25	0.17	0.38	0.60	0.86	0.99	1.16	1.26	1.21	1.20
2.37	-0.58	-0.30	0.02	0.27	0.51	0.72	0.94	1.08	1.17	1.19	1.15
2.42	-0.41	-0.18	0.16	0.52	0.76	0.98	1.12	1.26	1.42	1.41	1.43
2.47	-0.44	-0.28	0.12	0.38	0.60	0.90	1.13	1.24	1.21	1.33	1.34
2.52	-0.31	-0.11	0.16	0.55	0.82	1.04	1.16	1.34	1.46	1.48	1.48
2.57	-0.43	-0.16	0.17	0.45	0.67	0.94	1.21	1.22	1.41	1.39	1.41
2.62	-0.14	0.17	0.54	0.81	1.10	1.30	1.45	1.73	1.74	1.81	1.74
2.67	-0.37	-0.15	0.20	0.50	0.75	1.07	1.26	1.43	1.41	1.58	1.48
2.72	-0.21	0.10	0.46	0.66	1.02	1.29	1.46	1.66	1.78	1.78	1.78
2.77	0.03	0.31	0.67	1.00	1.22	1.50	1.71	1.83	1.97	1.99	1.94
2.82	-0.10	0.16	0.55	0.88	1.12	1.42	1.63	1.78	1.81	1.85	1.88
2.87	-0.18	0.03	0.38	0.67	0.99	1.31	1.52	1.60	1.75	1.79	1.80
2.92	-0.18	0.20	0.47	0.78	1.09	1.32	1.55	1.71	1.79	1.82	1.85
2.97	-0.13	0.09	0.41	0.82	1.14	1.37	1.63	1.79	1.83	1.96	1.95
3.02	-0.02	0.26	0.63	0.89	1.16	1.50	1.65	1.87	1.97	2.08	2.04
3.07	-0.20	0.14	0.46	0.90	1.15	1.43	1.59	1.84	1.93	2.05	2.00
3.12	-0.05	0.29	0.68	1.09	1.28	1.55	1.84	2.03	2.13	2.15	2.19
3.17	0.00	0.32	0.58	1.03	1.22	1.54	1.78	1.99	2.13	2.19	2.30
3.22	0.01	0.28	0.66	1.03	1.25	1.58	1.87	2.00	2.16	2.29	2.21
3.27	-0.10	0.37	0.72	1.06	1.35	1.61	1.78	2.04	2.14	2.19	2.28
3.32	-0.01	0.29	0.81	1.10	1.40	1.66	1.85	2.11	2.26	2.32	2.36
3.37	-0.15	0.16	0.54	0.86	1.12	1.37	1.63	1.83	2.09	2.03	2.05
3.42	0.04	0.35	0.80	1.05	1.40	1.67	2.01	2.17	2.31	2.39	2.37
3.47	-0.16	0.23	0.65	0.99	1.26	1.64	1.84	2.03	2.19	2.27	2.27
3.52	-0.16	0.25	0.65	0.99	1.25	1.56	1.76	2.02	2.12	2.18	2.16
3.57	-0.05	0.23	0.63	0.99	1.24	1.61	1.86	2.06	2.20	2.26	2.27
3.62	0.16	0.42	0.79	1.10	1.39	1.76	2.02	2.18	2.29	2.40	2.46
3.67	-0.08	0.20	0.61	0.99	1.29	1.58	1.79	1.93	2.13	2.17	2.21
3.72	0.10	0.35	0.77	1.15	1.45	1.69	2.07	2.19	2.40	2.43	2.45

3.92	0.06	0.39	0.85	1.21	1.49	1.77	2.13	2.30	2.52	2.54	2.57
4.12	0.07	0.49	0.84	1.14	1.52	1.80	2.16	2.33	2.43	2.57	2.51
4.32	0.09	0.36	0.78	1.23	1.53	1.79	2.14	2.33	2.45	2.50	2.50
4.52	0.04	0.40	0.86	1.24	1.44	1.81	2.04	2.27	2.41	2.56	2.63
4.72	0.10	0.40	0.80	1.18	1.41	1.82	2.12	2.30	2.44	2.62	2.56
4.92	0.31	0.67	1.03	1.37	1.75	2.11	2.39	2.68	2.76	2.88	2.87
5.12	0.33	0.62	0.98	1.38	1.75	2.03	2.34	2.60	2.67	2.77	2.78
5.32	0.21	0.58	1.13	1.46	1.81	2.11	2.45	2.72	2.80	2.95	2.99
5.52	0.34	0.79	1.14	1.51	1.84	2.16	2.50	2.75	2.91	3.02	2.94
5.72	0.33	0.69	1.10	1.59	1.90	2.30	2.50	2.75	2.90	3.05	3.08
5.92	0.16	0.53	0.93	1.43	1.67	2.01	2.27	2.49	2.69	2.83	2.86
6.12	0.19	0.57	1.17	1.48	1.82	2.15	2.42	2.72	2.86	2.95	3.01
6.32	0.06	0.47	0.91	1.31	1.58	2.00	2.15	2.48	2.63	2.69	2.72
6.52	0.33	0.65	1.03	1.51	1.83	2.19	2.41	2.70	2.86	2.99	3.00
6.72	0.43	0.83	1.29	1.66	2.10	2.31	2.61	2.89	3.00	3.21	3.24
6.92	0.14	0.56	0.92	1.42	1.71	2.13	2.42	2.61	2.74	2.92	2.92
7.12	0.28	0.63	1.05	1.49	1.83	2.21	2.45	2.73	2.92	2.98	3.05
7.32	0.28	0.60	1.06	1.51	1.91	2.14	2.53	2.78	2.94	2.98	3.04
7.52	0.28	0.60	1.09	1.49	1.88	2.24	2.49	2.77	2.97	3.02	3.03
7.72	0.24	0.69	1.13	1.49	1.82	2.21	2.64	2.77	2.91	3.06	3.06
7.92	0.26	0.59	1.04	1.52	1.81	2.17	2.44	2.76	2.91	3.02	3.10
8.12	0.37	0.70	1.22	1.63	1.96	2.26	2.64	2.87	3.07	3.14	3.22
8.32	0.39	0.79	1.25	1.65	2.04	2.31	2.59	2.85	3.01	3.12	3.25
8.52	0.18	0.58	1.07	1.49	1.79	2.13	2.44	2.72	2.86	2.92	3.02
8.72	0.16	0.53	0.99	1.52	1.78	2.05	2.37	2.74	2.87	3.03	3.02
8.92	0.09	0.56	0.94	1.38	1.74	2.09	2.39	2.66	2.89	2.97	3.01
9.12	0.29	0.70	1.08	1.58	1.92	2.30	2.57	2.80	3.04	3.11	3.17
9.32	0.24	0.60	1.07	1.50	1.73	2.16	2.41	2.67	2.78	2.99	3.04
9.52	0.18	0.62	1.03	1.54	1.86	2.29	2.55	2.82	3.05	3.21	3.18
9.72	0.24	0.63	1.11	1.50	1.84	2.14	2.51	2.82	2.90	3.09	3.06
9.92	0.44	0.76	1.29	1.66	2.02	2.41	2.65	2.98	3.17	3.21	3.31
10.12	0.28	0.69	1.15	1.52	1.86	2.33	2.59	2.85	3.08	3.21	3.24
10.32	0.21	0.62	1.05	1.48	1.80	2.16	2.40	2.61	2.77	2.95	2.99
10.52	0.28	0.57	1.02	1.43	1.82	2.27	2.48	2.73	3.01	2.96	3.13
10.72	0.34	0.77	1.20	1.61	1.91	2.34	2.69	2.96	3.10	3.18	3.24
10.92	0.28	0.62	1.15	1.60	1.87	2.26	2.58	2.90	3.03	3.09	3.09
11.12	0.28	0.60	1.08	1.46	1.84	2.27	2.51	2.82	3.00	3.11	3.17
11.32	0.45	0.77	1.26	1.79	2.13	2.51	2.75	3.03	3.22	3.33	3.34
11.52	0.32	0.61	1.15	1.57	1.83	2.21	2.56	2.77	2.99	3.08	3.08
11.72	0.29	0.67	1.11	1.53	1.87	2.29	2.58	2.86	3.01	3.15	3.23
11.92	0.20	0.50	0.97	1.44	1.87	2.14	2.37	2.75	2.85	2.98	3.00
12.12	0.26	0.64	1.09	1.55	1.87	2.32	2.63	2.94	3.03	3.20	3.23
12.32	0.32	0.71	1.14	1.54	1.93	2.32	2.67	2.88	3.14	3.22	3.25
12.52	0.26	0.57	1.09	1.55	1.81	2.27	2.54	2.80	2.98	3.04	3.11
12.72	0.52	0.87	1.29	1.76	2.09	2.49	2.75	3.09	3.23	3.29	3.31
12.92	0.38	0.75	1.18	1.66	2.03	2.39	2.65	2.96	3.22	3.28	3.36
13.12	0.19	0.70	1.00	1.46	1.78	2.15	2.48	2.70	2.89	3.00	3.05
13.32	0.15	0.54	1.00	1.52	1.89	2.24	2.53	2.78	3.04	3.13	3.12
13.52	0.30	0.71	1.13	1.61	1.89	2.21	2.54	2.79	2.98	3.15	3.14
13.72	0.43	0.85	1.34	1.74	2.04	2.49	2.87	3.02	3.26	3.35	3.36

13.92	0.28	0.64	1.10	1.52	1.86	2.32	2.61	2.85	2.99	3.16	3.17
14.00	0.40	0.77	1.27	1.72	2.03	2.52	2.71	3.08	3.31	3.44	3.41
14.08	0.32	0.70	1.13	1.61	1.93	2.35	2.59	2.87	3.02	3.16	3.18
14.17	0.23	0.67	1.13	1.48	1.90	2.30	2.52	2.85	2.98	3.11	3.16
14.27	0.17	0.59	0.99	1.47	1.83	2.18	2.60	2.75	2.94	3.18	3.14
14.38	0.34	0.72	1.20	1.62	1.90	2.28	2.67	2.92	3.04	3.13	3.17
14.49	0.26	0.71	1.13	1.49	1.83	2.25	2.59	2.84	2.97	3.12	3.16
14.61	0.20	0.57	0.95	1.44	1.84	2.17	2.57	2.65	2.84	3.01	3.07
14.75	0.14	0.53	1.01	1.42	1.80	2.16	2.42	2.72	2.87	3.02	3.09
14.89	0.62	0.96	1.49	1.89	2.34	2.69	3.04	3.32	3.41	3.56	3.65
15.05	0.16	0.53	1.11	1.48	1.84	2.31	2.59	2.84	3.03	3.17	3.10
15.21	0.40	0.72	1.23	1.63	2.01	2.43	2.73	3.12	3.22	3.27	3.35
15.39	0.34	0.78	1.19	1.63	1.98	2.30	2.66	2.95	3.10	3.20	3.25
15.59	0.19	0.63	1.09	1.51	1.85	2.29	2.54	2.80	3.08	3.10	3.17
15.80	0.32	0.74	1.15	1.67	2.03	2.49	2.72	3.06	3.27	3.30	3.36
16.02	0.42	0.72	1.06	1.62	1.91	2.38	2.68	2.89	3.00	3.16	3.20
16.27	0.44	0.83	1.27	1.73	2.08	2.50	2.83	3.13	3.27	3.40	3.36
16.53	0.15	0.56	1.05	1.44	1.89	2.27	2.57	2.74	3.01	3.15	3.12
16.81	0.13	0.52	1.06	1.45	1.81	2.21	2.55	2.84	2.96	3.02	3.08
17.12	0.35	0.80	1.35	1.67	2.04	2.41	2.73	2.98	3.11	3.31	3.37
17.45	0.20	0.53	1.06	1.51	1.75	2.31	2.54	2.78	2.90	2.99	3.10
17.81	0.33	0.73	1.17	1.71	2.03	2.47	2.76	3.05	3.22	3.35	3.40
18.19	0.29	0.78	1.24	1.66	2.04	2.46	2.79	3.09	3.30	3.40	3.44
18.60	0.38	0.70	1.25	1.69	2.06	2.44	2.82	3.03	3.22	3.36	3.46
19.05	0.32	0.75	1.19	1.65	2.06	2.49	2.82	3.03	3.28	3.37	3.33
19.53	0.20	0.50	0.99	1.47	1.85	2.25	2.60	2.86	2.99	3.11	3.11
20.05	0.16	0.61	1.04	1.49	1.84	2.28	2.60	2.88	3.11	3.13	3.24
20.61	0.25	0.59	1.09	1.60	2.00	2.39	2.68	3.03	3.15	3.29	3.36
21.22	0.30	0.61	1.18	1.62	2.05	2.42	2.72	2.98	3.16	3.21	3.26
21.87	0.22	0.56	1.10	1.53	1.91	2.36	2.62	2.95	3.12	3.18	3.32
22.57	0.26	0.77	1.26	1.69	1.93	2.40	2.69	2.94	3.24	3.25	3.31
23.33	0.20	0.67	1.22	1.65	2.08	2.40	2.76	3.00	3.21	3.22	3.28
24.15	0.21	0.62	1.05	1.56	1.95	2.23	2.62	2.94	3.11	3.17	3.19
25.03	0.18	0.49	1.06	1.58	1.90	2.26	2.63	2.77	2.99	3.12	3.22
25.98	0.19	0.62	1.17	1.63	2.00	2.44	2.77	3.05	3.14	3.37	3.34
27.01	0.07	0.56	0.98	1.49	1.93	2.24	2.55	2.86	3.07	3.16	3.15
28.11	0.34	0.68	1.25	1.56	1.98	2.37	2.84	3.07	3.20	3.38	3.40
29.30	0.19	0.65	1.14	1.56	1.89	2.33	2.66	2.93	3.11	3.26	3.23
30.59	0.20	0.61	1.11	1.55	2.02	2.37	2.75	2.88	3.16	3.32	3.29
31.98	-0.14	0.40	0.91	1.33	1.74	2.06	2.44	2.72	2.91	3.05	3.03
33.47	0.17	0.58	1.02	1.50	1.85	2.30	2.60	2.86	2.98	3.19	3.20
35.09	0.11	0.58	0.94	1.41	1.77	2.18	2.55	2.83	3.02	3.13	3.11
36.83	0.02	0.47	0.95	1.38	1.80	2.17	2.50	2.79	3.05	3.07	3.14
38.71	0.19	0.60	0.99	1.52	1.99	2.28	2.62	2.86	3.12	3.22	3.20
40.73	0.18	0.59	1.05	1.58	1.93	2.35	2.72	2.94	3.09	3.18	3.31
42.92	0.15	0.56	0.96	1.51	1.90	2.19	2.50	2.86	2.97	3.10	3.05
45.27	0.10	0.45	0.94	1.40	1.75	2.09	2.47	2.77	2.91	3.06	3.08
47.81	0.02	0.56	0.91	1.53	1.84	2.22	2.58	2.83	3.00	3.23	3.19
50.55	-0.04	0.33	0.71	1.10	1.52	1.94	2.26	2.43	2.65	2.78	2.78
53.51	0.10	0.51	1.07	1.48	1.80	2.30	2.67	2.88	3.01	3.13	3.19

56.69	-0.04	0.32	0.80	1.30	1.69	2.03	2.36	2.74	2.87	2.97	2.96
60.13	-0.15	0.28	0.78	1.26	1.65	1.97	2.34	2.62	2.77	2.89	2.96
63.84	-0.13	0.24	0.85	1.33	1.73	2.19	2.51	2.81	2.98	3.09	3.08
67.83	-0.24	0.20	0.71	1.11	1.57	1.99	2.33	2.60	2.71	2.94	2.87
72.15	-0.19	0.21	0.72	1.14	1.55	1.96	2.33	2.59	2.75	2.85	2.88
76.80	-0.30	0.10	0.61	1.03	1.43	1.94	2.11	2.49	2.66	2.74	2.76
81.81	-0.20	0.32	0.81	1.29	1.75	2.16	2.41	2.81	2.99	3.06	3.06
87.22	-0.20	0.29	0.75	1.25	1.60	2.00	2.32	2.59	2.80	2.86	2.90
93.06	-0.30	0.09	0.64	1.09	1.48	1.97	2.29	2.54	2.67	2.84	2.83
99.35	-0.19	0.19	0.69	1.24	1.62	1.95	2.37	2.63	2.74	2.92	2.92
106.13	-0.41	0.17	0.58	1.05	1.47	1.83	2.23	2.47	2.60	2.75	2.80
113.45	-0.14	0.22	0.77	1.29	1.62	2.14	2.43	2.75	2.86	3.00	2.96
121.34	-0.38	-0.06	0.58	0.94	1.40	1.71	2.12	2.41	2.61	2.71	2.72
129.86	-0.42	-0.04	0.38	0.91	1.30	1.73	1.96	2.32	2.56	2.66	2.62
139.04	-0.33	0.03	0.60	1.07	1.47	1.87	2.16	2.42	2.59	2.75	2.82
148.94	-0.56	-0.09	0.37	0.88	1.26	1.66	1.96	2.23	2.41	2.48	2.49
159.62	-0.39	0.04	0.52	1.02	1.38	1.79	2.20	2.44	2.68	2.78	2.76
171.14	-0.51	-0.04	0.51	1.01	1.34	1.78	2.14	2.45	2.57	2.71	2.70
183.56	-0.63	-0.23	0.22	0.74	1.10	1.50	1.79	2.16	2.28	2.38	2.40
196.96	-0.61	-0.09	0.41	0.86	1.31	1.77	2.01	2.38	2.51	2.68	2.68
211.41	-0.44	-0.04	0.47	0.97	1.38	1.77	2.14	2.42	2.60	2.76	2.66
226.99	-0.52	-0.08	0.38	0.90	1.37	1.74	2.05	2.37	2.56	2.62	2.68
243.80	-0.66	-0.21	0.26	0.76	1.11	1.53	1.79	2.17	2.23	2.39	2.51
261.93	-0.56	-0.04	0.37	0.83	1.30	1.72	2.11	2.40	2.57	2.70	2.67
281.48	-0.72	-0.19	0.21	0.74	1.09	1.58	1.91	2.22	2.43	2.61	2.55
302.56	-0.64	-0.18	0.33	0.80	1.18	1.55	1.92	2.26	2.41	2.54	2.51
325.30	-0.47	-0.16	0.41	0.89	1.36	1.74	2.08	2.40	2.53	2.66	2.65
349.83	-0.58	-0.17	0.33	0.83	1.27	1.75	1.98	2.26	2.39	2.54	2.58
376.28	-0.58	-0.12	0.37	0.86	1.28	1.59	1.97	2.26	2.48	2.56	2.60
404.81	-0.39	-0.03	0.56	1.07	1.34	1.87	2.18	2.32	2.55	2.66	2.79
435.58	-0.53	-0.13	0.43	0.91	1.27	1.64	2.01	2.29	2.41	2.53	2.50
468.76	-0.53	-0.07	0.36	0.94	1.36	1.74	2.01	2.34	2.44	2.57	2.55
504.55	-0.50	-0.11	0.28	0.89	1.25	1.64	1.96	2.23	2.37	2.50	2.51
543.15	-0.46	0.01	0.48	0.87	1.30	1.66	1.99	2.27	2.45	2.56	2.66
584.78	-0.56	-0.07	0.35	0.73	1.25	1.56	1.93	2.21	2.34	2.53	2.57
629.68	-0.36	0.06	0.47	0.95	1.37	1.77	2.01	2.23	2.47	2.58	2.61
678.10	-0.48	0.00	0.39	0.90	1.29	1.72	2.01	2.34	2.50	2.54	2.62
730.33	-0.31	0.13	0.62	1.16	1.58	1.98	2.26	2.59	2.76	2.83	2.85
786.65	-0.29	0.12	0.63	1.06	1.49	1.83	2.21	2.51	2.63	2.76	2.73
847.40	-0.38	0.02	0.55	0.96	1.40	1.81	2.10	2.31	2.60	2.65	2.69
912.92	-0.22	0.23	0.72	1.14	1.61	1.97	2.25	2.54	2.72	2.90	2.88
983.58	-0.24	0.20	0.70	1.19	1.54	1.89	2.20	2.51	2.66	2.78	2.76
1059.78	-0.12	0.24	0.84	1.21	1.59	1.99	2.32	2.53	2.74	2.85	2.90
1141.98	-0.06	0.30	0.70	1.09	1.57	2.01	2.24	2.48	2.70	2.83	2.79
1230.62	-0.17	0.24	0.72	1.13	1.48	1.89	2.15	2.45	2.61	2.69	2.73
1326.23	-0.05	0.40	0.80	1.27	1.76	2.01	2.39	2.69	2.74	2.84	2.85
1429.34	0.02	0.51	0.94	1.39	1.81	2.22	2.56	2.76	2.91	3.06	3.03
1540.54	0.19	0.58	1.01	1.48	1.78	2.19	2.57	2.78	3.01	3.04	3.02
1660.48	0.03	0.49	1.00	1.43	1.75	2.12	2.44	2.78	2.92	2.93	2.98
1789.83	0.11	0.56	0.91	1.47	1.83	2.18	2.44	2.75	2.84	3.03	3.01

1929.34	0.36	0.73	1.18	1.63	2.03	2.37	2.65	2.87	3.08	3.17	3.22
2079.80	0.38	0.78	1.29	1.63	2.01	2.36	2.58	2.94	3.06	3.10	3.20
2242.08	0.37	0.78	1.24	1.55	1.99	2.35	2.60	2.85	3.00	3.13	3.10
2417.10	0.46	0.85	1.22	1.68	2.06	2.44	2.68	2.88	3.12	3.20	3.19
2605.85	0.54	0.84	1.29	1.69	2.07	2.41	2.72	2.99	3.12	3.23	3.17
2809.43	0.50	0.80	1.27	1.68	1.93	2.29	2.55	2.68	2.92	2.99	2.96
3028.99	0.50	0.84	1.34	1.63	1.97	2.31	2.71	2.81	3.00	3.02	3.10
3265.79	0.69	0.94	1.44	1.83	2.14	2.46	2.72	2.94	3.13	3.19	3.20
3521.18	0.62	1.04	1.42	1.73	2.13	2.43	2.72	2.99	3.06	3.22	3.20
3796.62	0.37	0.78	1.25	1.59	1.86	2.21	2.44	2.67	2.83	2.86	2.92

Wavelength (nm) / Time (ps)	522.13	523.53	524.94	526.35	527.76	529.17	530.58	531.99	533.39	534.80	536.21
-3.28	-0.13	-0.18	-0.14	-0.13	-0.12	-0.19	-0.17	-0.12	-0.14	-0.17	-0.15
-2.78	-0.08	-0.11	-0.09	-0.05	-0.11	-0.08	-0.15	-0.09	-0.14	-0.12	-0.13
-2.28	-0.06	0.04	-0.05	-0.12	0.03	-0.03	0.00	-0.05	-0.05	-0.02	-0.06
-1.78	0.31	0.23	0.27	0.25	0.22	0.23	0.21	0.23	0.24	0.25	0.22
-1.28	-0.03	0.00	0.01	-0.02	0.00	0.00	0.03	-0.03	0.03	0.02	0.01
-0.78	-0.13	-0.10	-0.15	-0.06	-0.12	-0.09	-0.12	-0.08	-0.10	-0.09	-0.09
-0.28	0.03	0.01	0.02	0.01	0.03	0.05	0.04	0.06	0.12	0.08	0.06
0.22	0.12	0.10	0.10	0.13	0.08	0.09	0.15	0.07	0.07	0.09	0.12
0.32	-0.10	-0.05	-0.10	-0.07	-0.08	-0.08	-0.08	-0.06	-0.07	-0.06	-0.02
0.42	0.05	0.06	0.12	0.06	0.07	0.12	0.09	0.07	0.05	0.04	0.07
0.52	0.00	-0.10	-0.02	0.00	-0.02	-0.04	-0.05	0.00	-0.02	-0.04	-0.03
0.62	0.05	0.01	0.03	0.05	0.01	0.09	0.05	0.03	0.05	0.10	0.00
0.72	0.27	0.27	0.26	0.22	0.21	0.25	0.22	0.24	0.26	0.12	0.19
0.77	0.07	0.01	-0.02	0.05	0.06	0.07	0.12	0.06	0.05	0.05	0.05
0.82	0.10	0.14	0.07	0.14	0.09	0.03	0.03	0.07	0.19	0.10	0.12
0.87	-0.06	0.01	0.00	0.04	0.07	0.04	0.00	0.00	0.12	-0.04	-0.03
0.92	0.04	0.03	0.10	0.09	0.02	0.04	0.05	0.07	0.00	-0.05	0.11
0.97	-0.02	-0.01	-0.02	0.02	-0.01	-0.07	0.04	-0.08	-0.03	-0.04	-0.04
1.02	-0.12	-0.08	-0.16	-0.16	-0.09	-0.12	-0.13	-0.05	-0.15	-0.14	-0.09
1.07	0.17	0.14	0.17	0.21	0.15	0.16	0.09	0.18	0.28	0.12	0.20
1.12	-0.20	-0.23	-0.25	-0.23	-0.24	-0.26	-0.19	-0.18	-0.23	-0.24	-0.21
1.17	-0.15	-0.10	-0.20	-0.13	-0.12	-0.14	-0.22	-0.17	-0.12	-0.21	-0.11
1.22	-0.03	-0.06	-0.03	-0.03	0.04	-0.08	-0.08	-0.04	-0.04	0.00	0.02
1.27	0.03	0.06	0.09	0.15	0.13	0.10	0.11	0.15	0.07	0.09	0.10
1.32	-0.02	0.00	-0.07	0.00	0.03	0.01	0.00	-0.02	0.00	0.00	-0.02
1.37	-0.25	-0.16	-0.18	-0.16	-0.10	-0.08	-0.19	-0.10	-0.08	-0.12	-0.06
1.42	-0.28	-0.27	-0.34	-0.31	-0.39	-0.34	-0.36	-0.28	-0.27	-0.29	-0.30
1.47	1.50	1.35	1.21	0.99	0.88	0.71	0.50	0.41	0.24	0.22	0.11
1.52	1.43	1.31	1.26	1.18	1.07	0.97	0.77	0.81	0.72	0.73	0.73
1.57	0.16	0.06	-0.02	-0.09	-0.07	-0.23	-0.29	-0.33	-0.24	-0.24	-0.13
1.62	-0.65	-0.84	-0.94	-1.07	-1.20	-1.27	-1.35	-1.43	-1.40	-1.45	-1.25
1.67	-1.03	-1.30	-1.39	-1.59	-1.77	-1.93	-2.09	-2.16	-2.20	-2.25	-2.15
1.72	-0.82	-1.04	-1.16	-1.44	-1.61	-1.77	-1.89	-2.07	-2.20	-2.30	-2.14
1.77	-0.59	-0.76	-0.96	-1.07	-1.34	-1.47	-1.71	-1.83	-1.92	-2.03	-2.03
1.82	-0.31	-0.49	-0.72	-0.85	-1.04	-1.36	-1.46	-1.65	-1.79	-1.80	-1.74
1.87	-0.05	-0.13	-0.35	-0.56	-0.75	-0.90	-1.14	-1.26	-1.35	-1.45	-1.39

1.92	0.10	-0.03	-0.26	-0.48	-0.67	-0.85	-1.11	-1.19	-1.31	-1.35	-1.39
1.97	0.08	-0.11	-0.22	-0.41	-0.59	-0.80	-1.01	-1.14	-1.20	-1.32	-1.28
2.02	0.41	0.24	-0.02	-0.17	-0.31	-0.43	-0.65	-0.72	-0.82	-0.88	-0.87
2.07	0.37	0.30	0.01	-0.14	-0.28	-0.45	-0.64	-0.68	-0.79	-0.87	-0.87
2.12	0.83	0.64	0.48	0.26	0.17	-0.10	-0.28	-0.40	-0.47	-0.51	-0.52
2.17	0.92	0.81	0.63	0.47	0.31	0.05	-0.07	-0.17	-0.27	-0.29	-0.27
2.22	0.94	0.80	0.59	0.44	0.31	0.08	-0.05	-0.18	-0.20	-0.35	-0.24
2.27	1.10	1.03	0.86	0.70	0.50	0.37	0.16	0.09	-0.04	-0.08	-0.05
2.32	1.17	1.08	0.87	0.69	0.57	0.37	0.24	0.16	0.02	0.07	0.05
2.37	1.08	1.00	0.78	0.61	0.52	0.32	0.17	0.10	0.03	-0.10	-0.02
2.42	1.28	1.16	1.05	0.93	0.75	0.59	0.35	0.27	0.27	0.17	0.26
2.47	1.24	1.17	1.00	0.84	0.70	0.48	0.40	0.27	0.16	0.19	0.20
2.52	1.36	1.25	1.09	0.96	0.91	0.67	0.52	0.43	0.35	0.34	0.34
2.57	1.35	1.19	1.03	0.90	0.76	0.61	0.46	0.37	0.24	0.32	0.31
2.62	1.75	1.60	1.49	1.31	1.19	1.01	0.83	0.82	0.72	0.64	0.71
2.67	1.48	1.29	1.20	1.04	0.95	0.81	0.66	0.63	0.45	0.48	0.57
2.72	1.74	1.66	1.46	1.34	1.25	1.04	0.92	0.87	0.77	0.68	0.77
2.77	1.93	1.78	1.71	1.59	1.41	1.26	1.11	1.10	0.96	0.91	0.97
2.82	1.81	1.72	1.65	1.49	1.32	1.20	0.99	1.02	0.88	0.82	0.86
2.87	1.70	1.61	1.48	1.42	1.18	1.05	0.93	0.89	0.77	0.72	0.81
2.92	1.86	1.75	1.60	1.45	1.32	1.26	1.01	0.94	0.93	0.87	0.91
2.97	1.90	1.83	1.70	1.56	1.38	1.19	1.14	1.10	0.95	0.88	1.01
3.02	1.99	1.86	1.82	1.64	1.51	1.35	1.24	1.18	1.02	0.90	1.10
3.07	1.97	1.82	1.69	1.66	1.53	1.36	1.17	1.11	1.00	1.01	0.96
3.12	2.15	2.11	1.94	1.76	1.70	1.52	1.30	1.29	1.22	1.14	1.20
3.17	2.14	2.05	1.94	1.78	1.66	1.54	1.45	1.37	1.27	1.26	1.26
3.22	2.19	2.15	1.98	1.87	1.80	1.56	1.42	1.30	1.30	1.23	1.29
3.27	2.24	2.08	2.02	1.85	1.74	1.59	1.42	1.32	1.29	1.21	1.34
3.32	2.24	2.18	2.06	1.92	1.79	1.66	1.46	1.40	1.36	1.36	1.35
3.37	1.92	1.88	1.75	1.71	1.59	1.40	1.24	1.19	1.09	1.01	1.16
3.42	2.42	2.17	2.11	2.00	1.87	1.72	1.57	1.47	1.49	1.43	1.47
3.47	2.14	2.15	2.02	1.84	1.70	1.56	1.43	1.32	1.31	1.24	1.36
3.52	2.26	2.08	1.97	1.88	1.72	1.60	1.40	1.36	1.28	1.24	1.22
3.57	2.26	2.11	2.04	1.82	1.77	1.56	1.52	1.34	1.35	1.29	1.29
3.62	2.39	2.32	2.22	2.02	1.93	1.79	1.70	1.54	1.45	1.51	1.55
3.67	2.17	2.10	1.97	1.84	1.72	1.60	1.48	1.35	1.32	1.24	1.29
3.72	2.35	2.31	2.20	1.98	1.86	1.77	1.72	1.49	1.50	1.49	1.56
3.92	2.52	2.42	2.32	2.20	2.09	1.93	1.79	1.72	1.64	1.60	1.67
4.12	2.57	2.46	2.31	2.25	2.04	1.90	1.74	1.73	1.66	1.69	1.70
4.32	2.52	2.44	2.27	2.22	2.09	1.90	1.85	1.75	1.67	1.57	1.73
4.52	2.50	2.50	2.41	2.20	2.16	1.95	1.87	1.75	1.68	1.62	1.69
4.72	2.57	2.50	2.43	2.26	2.13	2.00	1.85	1.72	1.71	1.69	1.74
4.92	2.83	2.80	2.68	2.58	2.44	2.36	2.19	2.09	1.99	1.96	1.99
5.12	2.79	2.70	2.68	2.44	2.40	2.22	2.03	1.97	1.91	1.87	1.89
5.32	2.93	2.87	2.82	2.71	2.51	2.34	2.19	2.16	2.06	2.09	2.12
5.52	3.02	2.95	2.84	2.71	2.58	2.46	2.28	2.16	2.05	2.05	2.17
5.72	3.03	2.98	2.87	2.75	2.61	2.49	2.30	2.18	2.16	2.10	2.19
5.92	2.83	2.72	2.65	2.51	2.37	2.26	2.03	2.01	1.94	1.89	1.94
6.12	2.96	2.94	2.74	2.68	2.53	2.40	2.28	2.23	2.08	2.01	2.05
6.32	2.76	2.68	2.62	2.43	2.24	2.22	2.00	1.92	1.82	1.80	1.82

6.52	2.99	2.95	2.84	2.66	2.56	2.36	2.29	2.21	2.08	2.05	1.99
6.72	3.21	3.13	2.98	2.92	2.78	2.56	2.41	2.39	2.23	2.15	2.18
6.92	2.95	2.88	2.70	2.56	2.44	2.25	2.13	2.13	2.04	1.94	1.99
7.12	2.94	2.96	2.82	2.65	2.57	2.29	2.26	2.17	2.06	1.93	2.01
7.32	3.02	2.99	2.85	2.75	2.65	2.43	2.31	2.14	2.05	1.94	2.07
7.52	3.00	2.91	2.83	2.70	2.53	2.37	2.19	2.19	2.09	2.02	2.06
7.72	3.05	3.05	2.92	2.74	2.59	2.36	2.29	2.12	2.09	2.00	2.06
7.92	3.05	2.99	2.89	2.70	2.61	2.38	2.31	2.14	2.05	2.00	2.08
8.12	3.15	3.14	2.95	2.85	2.66	2.59	2.35	2.27	2.21	2.12	2.15
8.32	3.16	3.08	3.00	2.87	2.66	2.55	2.37	2.27	2.17	2.10	2.14
8.52	2.93	2.84	2.81	2.67	2.52	2.32	2.16	2.13	2.01	1.93	1.95
8.72	2.99	2.96	2.84	2.75	2.54	2.37	2.25	2.09	1.96	1.94	1.97
8.92	2.99	2.92	2.74	2.57	2.46	2.29	2.12	2.01	1.84	1.80	1.85
9.12	3.09	3.11	2.94	2.85	2.64	2.51	2.27	2.19	2.14	2.02	2.11
9.32	2.96	2.95	2.81	2.67	2.45	2.21	2.11	2.01	1.95	1.87	1.90
9.52	3.11	3.05	2.96	2.86	2.64	2.44	2.27	2.22	2.03	1.96	2.07
9.72	3.12	3.05	2.84	2.69	2.60	2.42	2.14	2.17	2.07	2.01	1.93
9.92	3.32	3.23	3.07	2.91	2.75	2.60	2.43	2.33	2.21	2.13	2.13
10.12	3.22	3.12	2.91	2.82	2.72	2.48	2.30	2.23	2.09	2.00	2.10
10.32	2.97	2.90	2.82	2.64	2.45	2.27	2.09	2.01	1.91	1.84	1.83
10.52	3.10	2.98	2.89	2.70	2.57	2.37	2.23	2.13	1.99	1.86	1.88
10.72	3.28	3.24	2.98	2.87	2.72	2.52	2.29	2.24	2.13	2.12	2.11
10.92	3.08	3.08	2.90	2.79	2.62	2.41	2.28	2.09	2.03	1.95	2.04
11.12	3.08	3.00	2.85	2.72	2.63	2.48	2.26	2.14	2.04	1.92	1.85
11.32	3.34	3.25	3.12	3.00	2.76	2.58	2.43	2.28	2.12	2.16	2.18
11.52	3.00	2.98	2.86	2.67	2.59	2.38	2.22	2.10	1.96	1.89	1.92
11.72	3.20	3.00	2.93	2.83	2.68	2.48	2.36	2.15	2.03	1.89	1.93
11.92	3.02	2.93	2.76	2.62	2.48	2.31	2.05	1.93	1.91	1.78	1.80
12.12	3.17	3.13	2.95	2.86	2.60	2.40	2.23	2.16	1.95	1.94	1.96
12.32	3.30	3.14	2.96	2.80	2.66	2.44	2.30	2.22	2.03	1.98	1.96
12.52	3.05	2.97	2.84	2.73	2.50	2.32	2.09	2.09	1.91	1.92	1.89
12.72	3.33	3.37	3.08	2.94	2.84	2.58	2.44	2.29	2.17	2.15	2.13
12.92	3.22	3.19	3.07	2.94	2.70	2.52	2.34	2.25	2.07	2.03	2.04
13.12	3.06	2.91	2.77	2.63	2.41	2.26	2.12	2.11	1.87	1.79	1.82
13.32	3.17	3.09	2.85	2.73	2.53	2.42	2.16	2.11	1.93	1.90	1.87
13.52	3.09	3.03	2.89	2.76	2.61	2.36	2.27	2.04	2.01	1.86	1.87
13.72	3.37	3.29	3.12	3.02	2.85	2.62	2.46	2.35	2.17	2.05	2.09
13.92	3.10	2.97	2.88	2.72	2.56	2.36	2.21	2.05	1.94	1.80	1.86
14.00	3.38	3.28	3.21	2.96	2.76	2.60	2.40	2.32	2.13	2.07	2.08
14.08	3.18	3.13	2.98	2.81	2.67	2.54	2.26	2.14	2.05	2.03	1.91
14.17	3.11	3.08	2.87	2.77	2.55	2.37	2.12	2.04	1.93	1.88	1.87
14.27	3.11	2.93	2.83	2.72	2.51	2.35	2.15	1.99	1.88	1.77	1.86
14.38	3.13	3.09	3.01	2.88	2.63	2.42	2.27	2.13	2.02	1.91	1.96
14.49	3.05	3.08	2.85	2.72	2.53	2.29	2.13	2.01	1.92	1.82	1.85
14.61	3.05	2.98	2.89	2.66	2.47	2.28	2.13	2.01	1.82	1.79	1.82
14.75	3.01	2.93	2.75	2.57	2.43	2.27	2.06	2.03	1.85	1.75	1.78
14.89	3.65	3.59	3.39	3.30	3.06	2.85	2.61	2.57	2.39	2.19	2.34
15.05	3.17	3.05	2.83	2.74	2.59	2.42	2.12	2.00	1.86	1.81	1.82
15.21	3.26	3.22	3.08	2.92	2.70	2.53	2.30	2.18	2.05	1.96	1.95
15.39	3.21	3.06	2.94	2.81	2.70	2.47	2.22	2.11	1.99	1.91	2.02

15.59	3.14	3.03	2.84	2.74	2.57	2.32	2.26	2.06	1.93	1.80	1.77
15.80	3.33	3.23	3.11	2.92	2.72	2.55	2.29	2.22	1.99	2.01	1.98
16.02	3.15	3.05	2.90	2.80	2.61	2.38	2.23	2.04	1.95	1.88	1.95
16.27	3.34	3.37	3.07	3.06	2.80	2.71	2.40	2.23	2.13	2.07	2.11
16.53	3.11	2.98	2.87	2.72	2.52	2.37	2.23	2.07	1.86	1.76	1.79
16.81	3.05	2.95	2.78	2.64	2.46	2.24	2.01	1.94	1.83	1.67	1.66
17.12	3.21	3.17	3.05	2.87	2.69	2.52	2.21	2.20	2.08	1.93	1.95
17.45	3.05	2.98	2.81	2.68	2.47	2.25	2.01	1.93	1.76	1.73	1.84
17.81	3.33	3.28	3.09	2.91	2.80	2.56	2.33	2.24	2.04	2.04	2.02
18.19	3.37	3.31	3.15	2.94	2.78	2.50	2.28	2.22	2.09	1.97	1.91
18.60	3.31	3.21	3.12	2.91	2.74	2.51	2.37	2.21	1.99	1.91	1.95
19.05	3.31	3.33	3.08	2.92	2.76	2.48	2.31	2.20	2.06	1.92	1.95
19.53	3.11	2.99	2.86	2.65	2.49	2.30	2.08	1.90	1.87	1.74	1.77
20.05	3.16	3.14	2.97	2.71	2.58	2.32	2.14	1.98	1.88	1.79	1.68
20.61	3.22	3.13	3.05	2.87	2.63	2.45	2.28	2.07	1.93	1.90	1.89
21.22	3.27	3.16	2.95	2.82	2.68	2.39	2.22	2.10	1.91	1.79	1.82
21.87	3.11	3.10	2.97	2.80	2.58	2.42	2.20	2.02	1.85	1.82	1.83
22.57	3.31	3.16	3.01	2.86	2.64	2.38	2.17	2.05	1.90	1.82	1.84
23.33	3.26	3.14	3.02	2.89	2.61	2.40	2.19	2.03	1.92	1.80	1.79
24.15	3.15	3.10	3.01	2.76	2.64	2.37	2.12	2.05	1.88	1.83	1.82
25.03	3.13	2.99	2.85	2.61	2.50	2.34	2.07	1.91	1.76	1.65	1.74
25.98	3.28	3.24	3.04	2.89	2.72	2.44	2.24	2.10	1.92	1.83	1.85
27.01	3.11	3.03	2.78	2.70	2.49	2.20	2.05	1.94	1.81	1.64	1.69
28.11	3.36	3.20	3.07	2.85	2.62	2.45	2.25	2.06	1.93	1.88	1.91
29.30	3.19	3.15	2.92	2.77	2.57	2.37	2.10	2.05	1.87	1.75	1.66
30.59	3.25	3.13	2.88	2.83	2.65	2.41	2.15	2.05	1.85	1.85	1.76
31.98	2.93	2.88	2.72	2.57	2.37	2.09	1.90	1.75	1.65	1.54	1.53
33.47	3.09	2.99	2.87	2.70	2.47	2.23	2.09	1.87	1.74	1.65	1.62
35.09	3.11	2.99	2.82	2.70	2.42	2.21	2.00	1.92	1.67	1.55	1.56
36.83	3.09	2.96	2.71	2.59	2.39	2.09	1.97	1.82	1.68	1.62	1.64
38.71	3.13	3.04	2.88	2.70	2.53	2.24	2.05	1.87	1.81	1.66	1.63
40.73	3.16	3.07	2.97	2.71	2.50	2.28	2.14	1.93	1.82	1.76	1.79
42.92	3.06	2.99	2.79	2.66	2.34	2.20	2.05	1.78	1.66	1.60	1.56
45.27	3.04	2.90	2.79	2.55	2.36	2.17	1.98	1.80	1.61	1.58	1.58
47.81	3.14	3.04	2.96	2.72	2.48	2.25	1.95	1.85	1.73	1.63	1.56
50.55	2.77	2.65	2.48	2.29	2.08	1.86	1.55	1.39	1.35	1.30	1.27
53.51	3.14	3.06	2.90	2.74	2.47	2.32	2.08	1.83	1.73	1.64	1.65
56.69	2.92	2.87	2.64	2.55	2.28	2.04	1.83	1.68	1.46	1.43	1.44
60.13	2.95	2.81	2.63	2.41	2.27	2.03	1.72	1.61	1.51	1.38	1.38
63.84	3.05	2.92	2.77	2.57	2.31	2.10	1.91	1.76	1.59	1.51	1.48
67.83	2.86	2.77	2.54	2.36	2.16	1.97	1.69	1.54	1.38	1.31	1.25
72.15	2.83	2.71	2.52	2.38	2.17	1.88	1.74	1.57	1.41	1.29	1.35
76.80	2.69	2.60	2.42	2.28	2.08	1.84	1.62	1.52	1.34	1.23	1.27
81.81	3.02	2.91	2.82	2.57	2.26	2.19	1.91	1.73	1.53	1.54	1.51
87.22	2.82	2.74	2.59	2.37	2.19	1.98	1.72	1.60	1.46	1.29	1.37
93.06	2.77	2.65	2.44	2.34	2.09	1.84	1.70	1.52	1.30	1.27	1.27
99.35	2.86	2.78	2.61	2.41	2.18	1.92	1.81	1.61	1.46	1.35	1.35
106.13	2.79	2.56	2.42	2.23	2.02	1.82	1.65	1.46	1.32	1.22	1.28
113.45	2.99	2.84	2.71	2.42	2.23	2.01	1.76	1.67	1.52	1.35	1.42
121.34	2.67	2.62	2.38	2.23	2.01	1.78	1.58	1.31	1.28	1.09	1.16

129.86	2.64	2.51	2.34	2.13	1.89	1.73	1.49	1.35	1.22	1.09	1.06
139.04	2.64	2.55	2.45	2.23	1.95	1.73	1.64	1.40	1.22	1.17	1.23
148.94	2.52	2.43	2.16	2.03	1.75	1.55	1.35	1.15	1.02	0.97	0.95
159.62	2.81	2.58	2.46	2.25	2.04	1.76	1.57	1.40	1.27	1.13	1.24
171.14	2.66	2.55	2.38	2.22	1.97	1.76	1.56	1.46	1.23	1.18	1.14
183.56	2.36	2.22	2.07	1.90	1.69	1.46	1.17	1.07	0.96	0.84	0.87
196.96	2.60	2.53	2.32	2.14	1.95	1.61	1.51	1.33	1.17	1.05	1.05
211.41	2.69	2.55	2.38	2.13	1.98	1.77	1.47	1.33	1.17	1.13	1.10
226.99	2.57	2.53	2.29	2.13	1.85	1.61	1.41	1.21	1.10	0.96	1.08
243.80	2.38	2.27	2.12	1.88	1.66	1.45	1.31	1.18	1.00	0.91	0.91
261.93	2.61	2.57	2.40	2.12	1.98	1.73	1.45	1.28	1.17	1.02	1.08
281.48	2.52	2.39	2.20	1.97	1.76	1.59	1.30	1.19	1.05	1.01	0.93
302.56	2.47	2.39	2.21	2.00	1.77	1.62	1.35	1.21	1.05	1.03	0.99
325.30	2.63	2.57	2.43	2.16	1.93	1.66	1.54	1.33	1.15	1.11	1.11
349.83	2.48	2.35	2.20	2.04	1.83	1.62	1.34	1.14	1.05	0.93	0.99
376.28	2.54	2.39	2.21	2.08	1.78	1.62	1.29	1.15	1.07	1.00	1.05
404.81	2.66	2.54	2.41	2.23	1.97	1.74	1.56	1.46	1.29	1.24	1.26
435.58	2.52	2.42	2.24	2.04	1.79	1.57	1.29	1.24	1.11	1.00	1.05
468.76	2.55	2.46	2.31	2.14	1.88	1.71	1.46	1.30	1.15	1.11	1.09
504.55	2.52	2.41	2.15	1.96	1.78	1.59	1.36	1.18	1.09	1.01	1.05
543.15	2.57	2.40	2.22	2.02	1.87	1.61	1.40	1.27	1.16	1.02	1.06
584.78	2.47	2.35	2.12	1.98	1.83	1.55	1.36	1.27	1.07	1.04	1.08
629.68	2.53	2.45	2.20	2.06	1.92	1.70	1.45	1.34	1.28	1.17	1.17
678.10	2.49	2.40	2.24	2.08	1.84	1.60	1.39	1.20	1.16	1.06	1.13
730.33	2.81	2.68	2.46	2.31	2.07	1.86	1.68	1.48	1.44	1.27	1.37
786.65	2.79	2.65	2.42	2.25	2.11	1.79	1.56	1.48	1.45	1.34	1.36
847.40	2.58	2.50	2.33	2.14	1.90	1.70	1.49	1.37	1.29	1.21	1.29
912.92	2.75	2.60	2.52	2.26	2.09	1.86	1.64	1.50	1.46	1.32	1.44
983.58	2.73	2.55	2.39	2.27	2.00	1.80	1.52	1.43	1.43	1.26	1.36
1059.78	2.72	2.78	2.49	2.31	2.12	1.95	1.70	1.66	1.47	1.43	1.52
1141.98	2.70	2.64	2.45	2.26	2.05	1.80	1.71	1.58	1.38	1.37	1.43
1230.62	2.66	2.53	2.38	2.19	2.01	1.76	1.59	1.48	1.34	1.34	1.44
1326.23	2.87	2.77	2.50	2.36	2.14	1.93	1.78	1.69	1.50	1.48	1.61
1429.34	3.00	2.84	2.70	2.52	2.32	2.11	1.98	1.82	1.68	1.67	1.75
1540.54	2.98	2.95	2.67	2.53	2.33	2.07	1.94	1.85	1.71	1.71	1.75
1660.48	2.92	2.83	2.62	2.53	2.33	2.09	1.81	1.71	1.67	1.67	1.69
1789.83	3.00	2.84	2.67	2.44	2.24	2.10	1.89	1.77	1.67	1.62	1.72
1929.34	3.14	2.95	2.84	2.61	2.42	2.27	2.01	1.94	1.91	1.89	1.97
2079.80	3.04	2.95	2.83	2.60	2.52	2.21	2.11	1.99	1.88	1.91	1.98
2242.08	3.11	2.94	2.77	2.60	2.45	2.29	2.02	2.00	1.88	1.79	1.96
2417.10	3.08	3.03	2.83	2.64	2.44	2.36	2.08	2.02	1.93	1.93	1.98
2605.85	3.14	3.02	2.89	2.70	2.48	2.33	2.09	2.09	2.00	1.98	2.06
2809.43	2.87	2.82	2.66	2.57	2.34	2.10	1.99	1.91	1.86	1.88	1.98
3028.99	2.97	2.98	2.75	2.55	2.38	2.21	2.06	1.94	1.92	1.84	1.98
3265.79	3.13	3.02	2.83	2.65	2.57	2.39	2.21	2.14	2.03	1.99	2.12
3521.18	3.06	3.01	2.91	2.61	2.53	2.39	2.17	2.14	2.00	2.02	2.03
3796.62	2.80	2.76	2.52	2.43	2.29	2.11	1.92	1.86	1.74	1.78	1.88

Time (ps) \ Wavelength (nm)	537.62	539.03	540.44	541.85	543.25	544.66	546.07	547.48	548.89	550.30	551.71
-3.28	-0.14	-0.13	-0.08	-0.12	-0.10	-0.07	-0.04	-0.06	-0.14	-0.14	-0.09
-2.78	-0.15	-0.12	-0.07	-0.10	-0.07	-0.10	-0.14	-0.09	-0.08	-0.06	-0.07
-2.28	0.00	-0.02	-0.01	-0.14	-0.04	-0.04	-0.05	-0.02	0.00	-0.03	-0.04
-1.78	0.27	0.23	0.24	0.24	0.23	0.21	0.24	0.24	0.23	0.20	0.18
-1.28	0.03	0.02	-0.01	0.02	-0.07	-0.05	-0.03	0.03	-0.03	0.03	-0.01
-0.78	-0.07	-0.10	-0.14	-0.12	-0.08	-0.09	-0.11	-0.14	-0.12	-0.15	-0.16
-0.28	0.04	0.08	0.04	0.09	0.04	0.06	0.09	0.06	0.07	0.05	0.06
0.22	0.06	0.14	0.07	0.14	0.08	0.12	0.10	0.06	0.10	0.12	0.11
0.32	-0.14	-0.11	-0.07	-0.14	-0.06	-0.11	-0.08	-0.08	-0.06	-0.07	-0.09
0.42	0.09	0.01	0.04	0.14	0.06	0.08	0.02	-0.01	0.04	0.05	0.09
0.52	-0.04	-0.05	-0.09	0.01	-0.05	-0.08	-0.02	-0.07	-0.07	-0.09	-0.04
0.62	0.07	0.01	0.02	0.08	0.07	0.00	-0.05	0.00	0.09	0.05	0.02
0.72	0.27	0.18	0.19	0.18	0.18	0.13	0.11	0.21	0.12	0.15	0.19
0.77	0.09	0.01	-0.02	0.02	0.04	0.09	0.08	0.07	0.03	0.03	0.05
0.82	0.04	0.07	0.04	0.08	0.11	0.01	-0.03	0.02	0.06	0.07	0.07
0.87	0.07	-0.02	0.03	0.07	0.08	0.02	-0.01	0.02	0.00	0.02	0.01
0.92	0.05	0.03	-0.02	-0.01	0.02	0.04	-0.01	0.01	-0.04	-0.06	-0.04
0.97	-0.01	0.01	-0.02	0.05	-0.02	-0.03	-0.02	-0.02	-0.03	0.03	0.00
1.02	-0.12	-0.08	-0.11	-0.11	-0.07	-0.09	-0.15	-0.13	-0.11	-0.10	-0.14
1.07	0.15	0.19	0.12	0.16	0.20	0.13	0.16	0.11	0.12	0.14	0.15
1.12	-0.22	-0.26	-0.28	-0.20	-0.16	-0.21	-0.24	-0.21	-0.31	-0.20	-0.15
1.17	-0.14	-0.16	-0.19	-0.08	-0.12	-0.13	-0.09	-0.11	-0.15	-0.16	-0.14
1.22	-0.07	-0.01	-0.11	-0.04	-0.08	-0.04	-0.04	-0.09	-0.07	-0.07	-0.07
1.27	0.18	0.06	0.09	0.12	0.14	0.11	0.08	0.11	0.11	0.09	0.12
1.32	0.04	-0.02	-0.04	0.00	0.06	0.03	-0.01	-0.04	0.04	-0.01	0.05
1.37	-0.05	-0.10	-0.16	-0.06	-0.03	-0.03	-0.11	-0.08	-0.06	-0.07	-0.08
1.42	-0.20	-0.24	-0.22	-0.22	-0.17	-0.20	-0.21	-0.16	-0.19	-0.11	-0.17
1.47	0.21	0.14	0.12	0.13	0.07	0.04	-0.01	-0.02	-0.01	-0.01	-0.05
1.52	0.79	0.86	1.03	1.18	1.34	1.51	1.67	1.76	1.84	1.94	1.96
1.57	0.10	0.31	0.71	1.09	1.55	1.94	2.38	2.93	3.39	3.80	4.20
1.62	-1.00	-0.76	-0.36	0.03	0.50	1.12	1.69	2.30	2.92	3.63	4.28
1.67	-1.92	-1.73	-1.42	-1.02	-0.51	-0.01	0.54	1.23	1.86	2.59	3.30
1.72	-2.01	-1.86	-1.59	-1.20	-0.77	-0.31	0.22	0.86	1.37	2.00	2.66
1.77	-1.88	-1.74	-1.51	-1.14	-0.83	-0.38	0.08	0.61	1.21	1.83	2.34
1.82	-1.72	-1.57	-1.42	-1.05	-0.73	-0.39	0.07	0.56	1.01	1.62	2.12
1.87	-1.32	-1.24	-1.02	-0.77	-0.42	-0.11	0.28	0.73	1.23	1.79	2.26
1.92	-1.25	-1.23	-0.94	-0.80	-0.39	-0.04	0.35	0.83	1.31	1.85	2.31
1.97	-1.16	-1.05	-0.87	-0.55	-0.25	0.09	0.56	0.96	1.51	1.95	2.50
2.02	-0.80	-0.63	-0.44	-0.12	0.14	0.51	0.92	1.42	1.92	2.38	2.88
2.07	-0.72	-0.65	-0.44	-0.08	0.25	0.60	1.03	1.49	1.92	2.40	2.85
2.12	-0.34	-0.24	-0.02	0.28	0.58	1.03	1.42	1.90	2.42	2.89	3.43
2.17	-0.14	-0.01	0.23	0.44	0.79	1.18	1.60	2.08	2.51	2.98	3.50
2.22	-0.20	-0.03	0.16	0.46	0.79	1.19	1.56	1.96	2.41	2.88	3.41
2.27	0.08	0.17	0.42	0.66	1.02	1.36	1.77	2.19	2.67	3.16	3.63
2.32	0.12	0.25	0.43	0.74	1.14	1.45	1.83	2.30	2.76	3.30	3.67
2.37	0.09	0.30	0.45	0.79	1.14	1.60	1.90	2.45	2.86	3.33	3.79
2.42	0.38	0.43	0.72	1.04	1.34	1.68	2.12	2.60	3.12	3.59	4.02

2.47	0.31	0.41	0.64	0.98	1.26	1.63	2.06	2.53	2.94	3.43	3.89
2.52	0.48	0.60	0.80	1.10	1.45	1.81	2.28	2.72	3.15	3.65	4.07
2.57	0.39	0.53	0.76	1.01	1.37	1.78	2.16	2.59	3.11	3.48	3.95
2.62	0.83	0.94	1.21	1.44	1.82	2.22	2.59	3.00	3.45	3.92	4.40
2.67	0.60	0.76	0.98	1.21	1.59	1.86	2.32	2.77	3.18	3.62	4.01
2.72	0.87	1.03	1.28	1.61	1.89	2.30	2.63	3.09	3.52	4.02	4.45
2.77	1.04	1.20	1.42	1.71	2.06	2.50	2.91	3.35	3.83	4.32	4.69
2.82	1.07	1.18	1.37	1.70	2.00	2.40	2.78	3.24	3.67	4.16	4.58
2.87	0.91	1.06	1.21	1.62	1.94	2.28	2.66	3.20	3.62	4.05	4.52
2.92	1.02	1.13	1.40	1.74	2.08	2.44	2.80	3.26	3.67	4.22	4.67
2.97	1.08	1.20	1.43	1.76	2.05	2.48	2.91	3.34	3.77	4.21	4.68
3.02	1.20	1.34	1.61	1.93	2.30	2.67	3.08	3.52	4.05	4.51	4.94
3.07	1.07	1.22	1.53	1.84	2.26	2.65	3.05	3.48	3.95	4.44	4.95
3.12	1.31	1.49	1.72	2.05	2.36	2.80	3.27	3.73	4.20	4.65	5.13
3.17	1.37	1.61	1.84	2.12	2.47	2.82	3.23	3.79	4.19	4.75	5.18
3.22	1.39	1.53	1.81	2.13	2.50	2.94	3.39	3.74	4.21	4.77	5.23
3.27	1.37	1.57	1.83	2.07	2.49	2.81	3.24	3.66	4.17	4.68	5.02
3.32	1.47	1.67	1.90	2.20	2.65	3.11	3.43	3.91	4.42	4.94	5.41
3.37	1.27	1.46	1.66	1.98	2.38	2.75	3.14	3.67	4.08	4.55	5.04
3.42	1.55	1.70	1.89	2.31	2.68	3.04	3.47	3.94	4.43	4.86	5.33
3.47	1.38	1.60	1.89	2.19	2.56	2.99	3.41	3.89	4.36	4.88	5.31
3.52	1.39	1.61	1.87	2.14	2.54	2.91	3.28	3.79	4.24	4.70	5.19
3.57	1.49	1.59	1.82	2.17	2.54	2.90	3.35	3.78	4.20	4.63	5.13
3.62	1.66	1.82	2.10	2.35	2.76	3.09	3.51	4.01	4.50	4.95	5.43
3.67	1.49	1.59	1.78	2.18	2.59	2.97	3.34	3.78	4.27	4.69	5.19
3.72	1.63	1.78	2.02	2.37	2.77	3.09	3.56	3.98	4.47	4.94	5.39
3.92	1.75	1.95	2.26	2.55	2.96	3.38	3.85	4.29	4.73	5.28	5.76
4.12	1.76	1.98	2.22	2.55	2.93	3.33	3.72	4.17	4.66	5.14	5.53
4.32	1.80	1.97	2.29	2.56	2.91	3.21	3.64	4.14	4.59	5.07	5.54
4.52	1.82	2.07	2.22	2.59	3.00	3.36	3.79	4.21	4.69	5.20	5.69
4.72	1.84	2.01	2.25	2.56	2.97	3.34	3.70	4.17	4.71	5.17	5.60
4.92	2.13	2.32	2.52	2.90	3.27	3.67	4.07	4.57	5.02	5.58	5.99
5.12	2.06	2.19	2.49	2.76	3.15	3.57	4.02	4.46	4.91	5.45	5.93
5.32	2.22	2.36	2.61	2.93	3.31	3.70	4.18	4.65	5.13	5.62	6.10
5.52	2.17	2.39	2.61	2.93	3.31	3.71	4.18	4.67	5.14	5.66	6.12
5.72	2.22	2.39	2.70	3.00	3.42	3.79	4.29	4.79	5.29	5.76	6.28
5.92	2.03	2.22	2.40	2.76	3.14	3.58	4.02	4.45	5.01	5.44	5.91
6.12	2.21	2.37	2.55	2.97	3.36	3.71	4.17	4.68	5.20	5.71	6.16
6.32	1.99	2.11	2.39	2.66	3.10	3.46	3.86	4.43	4.88	5.39	5.85
6.52	2.13	2.36	2.54	2.86	3.20	3.63	4.03	4.57	5.01	5.50	6.01
6.72	2.31	2.51	2.76	3.04	3.48	3.92	4.36	4.84	5.38	5.90	6.38
6.92	2.03	2.26	2.46	2.83	3.20	3.59	4.03	4.51	5.03	5.55	5.98
7.12	2.20	2.27	2.55	2.84	3.17	3.61	4.06	4.49	5.00	5.52	5.99
7.32	2.21	2.37	2.50	2.95	3.28	3.74	4.19	4.73	5.17	5.74	6.21
7.52	2.22	2.26	2.58	2.86	3.21	3.61	4.13	4.61	5.02	5.63	6.07
7.72	2.19	2.32	2.56	2.84	3.23	3.68	4.12	4.61	5.10	5.53	6.10
7.92	2.15	2.30	2.55	2.87	3.27	3.67	4.10	4.63	5.07	5.61	6.08
8.12	2.22	2.39	2.64	3.00	3.45	3.73	4.19	4.68	5.12	5.69	6.24
8.32	2.29	2.44	2.58	2.88	3.31	3.70	4.15	4.66	5.13	5.65	6.07
8.52	2.08	2.24	2.49	2.72	3.19	3.56	4.03	4.48	4.98	5.43	5.89

8.72	2.05	2.26	2.51	2.81	3.19	3.63	4.12	4.58	5.10	5.62	6.07
8.92	2.03	2.12	2.34	2.65	3.08	3.46	3.92	4.40	4.92	5.39	5.93
9.12	2.18	2.37	2.53	2.91	3.30	3.71	4.15	4.66	5.18	5.75	6.25
9.32	2.01	2.15	2.34	2.66	3.00	3.46	3.87	4.32	4.83	5.31	5.77
9.52	2.16	2.24	2.52	2.89	3.24	3.62	4.10	4.56	5.10	5.59	6.09
9.72	2.17	2.35	2.50	2.81	3.24	3.58	4.05	4.56	5.04	5.57	6.03
9.92	2.24	2.44	2.68	2.99	3.29	3.75	4.15	4.58	5.05	5.60	6.08
10.12	2.20	2.35	2.59	2.92	3.30	3.75	4.20	4.66	5.13	5.72	6.23
10.32	2.07	2.10	2.40	2.64	3.02	3.36	3.85	4.31	4.80	5.26	5.71
10.52	2.01	2.17	2.35	2.71	3.16	3.47	3.93	4.47	4.89	5.40	5.92
10.72	2.22	2.31	2.55	2.88	3.32	3.63	4.13	4.60	5.14	5.65	6.09
10.92	2.08	2.27	2.46	2.77	3.18	3.55	4.03	4.46	5.02	5.52	5.99
11.12	2.01	2.25	2.37	2.73	3.18	3.50	3.91	4.44	4.86	5.37	5.88
11.32	2.27	2.37	2.59	2.91	3.29	3.73	4.17	4.63	5.19	5.65	6.12
11.52	2.05	2.11	2.36	2.73	3.02	3.42	3.93	4.41	4.89	5.40	5.86
11.72	2.08	2.18	2.47	2.73	3.12	3.47	3.90	4.45	4.89	5.40	5.90
11.92	1.92	2.04	2.24	2.60	3.02	3.40	3.85	4.36	4.83	5.40	5.87
12.12	2.03	2.22	2.42	2.73	3.19	3.57	4.06	4.53	5.07	5.59	6.12
12.32	2.09	2.25	2.42	2.76	3.11	3.59	4.01	4.50	5.03	5.56	6.01
12.52	1.98	2.09	2.31	2.64	3.00	3.43	3.84	4.30	4.84	5.39	5.87
12.72	2.25	2.35	2.57	2.89	3.23	3.67	4.04	4.53	5.03	5.55	6.05
12.92	2.20	2.31	2.50	2.80	3.13	3.58	4.03	4.52	5.00	5.55	6.01
13.12	1.90	2.10	2.29	2.60	2.93	3.35	3.76	4.26	4.70	5.28	5.71
13.32	2.01	2.09	2.39	2.69	3.15	3.47	3.94	4.46	4.96	5.45	5.92
13.52	1.96	2.15	2.39	2.62	3.12	3.38	3.83	4.24	4.75	5.25	5.75
13.72	2.26	2.39	2.61	2.94	3.30	3.70	4.14	4.69	5.18	5.67	6.20
13.92	1.96	2.04	2.32	2.59	2.97	3.38	3.83	4.30	4.75	5.28	5.84
14.00	2.19	2.29	2.50	2.83	3.18	3.66	4.12	4.61	5.13	5.65	6.15
14.08	2.06	2.24	2.44	2.80	3.12	3.53	3.96	4.43	4.94	5.51	5.94
14.17	1.96	2.06	2.32	2.67	3.04	3.44	3.86	4.33	4.77	5.33	5.79
14.27	1.91	2.11	2.28	2.58	2.97	3.42	3.80	4.26	4.77	5.26	5.80
14.38	2.02	2.19	2.36	2.70	3.12	3.56	3.96	4.44	4.95	5.49	5.94
14.49	1.96	2.08	2.24	2.54	2.95	3.40	3.71	4.25	4.76	5.21	5.70
14.61	1.90	2.04	2.21	2.58	2.96	3.43	3.87	4.32	4.77	5.31	5.76
14.75	1.87	2.05	2.24	2.54	2.95	3.30	3.74	4.26	4.72	5.25	5.70
14.89	2.41	2.55	2.68	3.09	3.50	3.92	4.32	4.85	5.35	5.90	6.39
15.05	1.89	2.11	2.27	2.63	2.97	3.39	3.76	4.30	4.80	5.36	5.87
15.21	2.09	2.20	2.49	2.76	3.12	3.58	3.99	4.52	5.01	5.53	6.06
15.39	2.00	2.17	2.27	2.63	3.04	3.44	3.80	4.29	4.80	5.27	5.72
15.59	1.92	2.09	2.30	2.60	3.01	3.38	3.86	4.38	4.90	5.38	5.86
15.80	2.09	2.26	2.48	2.81	3.18	3.61	4.03	4.51	5.05	5.58	6.08
16.02	1.93	2.11	2.29	2.63	3.01	3.34	3.72	4.31	4.72	5.22	5.70
16.27	2.20	2.33	2.61	2.96	3.25	3.70	4.08	4.64	5.18	5.69	6.19
16.53	1.90	2.05	2.28	2.62	2.94	3.38	3.84	4.31	4.82	5.34	5.86
16.81	1.83	1.90	2.18	2.50	2.79	3.26	3.68	4.13	4.66	5.13	5.61
17.12	2.09	2.21	2.41	2.79	3.13	3.55	4.07	4.52	5.01	5.57	6.00
17.45	1.89	1.93	2.19	2.48	2.83	3.24	3.69	4.21	4.65	5.14	5.64
17.81	2.08	2.17	2.46	2.80	3.18	3.59	4.01	4.49	5.01	5.59	6.06
18.19	2.02	2.15	2.43	2.85	3.21	3.63	4.08	4.59	5.13	5.64	6.18
18.60	2.02	2.21	2.44	2.77	3.10	3.51	4.02	4.53	5.06	5.58	6.10

19.05	2.11	2.13	2.35	2.76	3.09	3.54	4.00	4.47	4.91	5.53	5.98
19.53	1.76	1.98	2.20	2.52	2.91	3.33	3.75	4.26	4.73	5.31	5.85
20.05	1.88	2.06	2.17	2.56	2.91	3.32	3.77	4.30	4.82	5.33	5.82
20.61	1.98	2.04	2.33	2.63	3.07	3.40	3.89	4.40	4.92	5.42	5.98
21.22	1.92	2.08	2.36	2.62	3.02	3.41	3.87	4.41	4.94	5.37	5.92
21.87	1.90	2.01	2.21	2.54	2.99	3.41	3.83	4.39	4.90	5.36	5.89
22.57	1.90	2.07	2.32	2.60	2.97	3.37	3.84	4.32	4.87	5.36	5.87
23.33	1.93	2.07	2.28	2.60	3.06	3.42	3.92	4.39	4.94	5.52	5.95
24.15	1.87	2.03	2.22	2.54	2.95	3.31	3.78	4.28	4.80	5.29	5.77
25.03	1.81	1.96	2.15	2.55	2.91	3.24	3.71	4.20	4.75	5.26	5.77
25.98	1.93	2.06	2.31	2.71	3.09	3.53	4.00	4.54	5.02	5.56	6.07
27.01	1.75	1.85	2.13	2.46	2.79	3.17	3.63	4.14	4.68	5.20	5.69
28.11	1.95	2.13	2.32	2.62	3.00	3.49	3.94	4.43	5.01	5.57	6.05
29.30	1.82	1.99	2.21	2.49	2.95	3.33	3.79	4.37	4.90	5.37	5.87
30.59	1.90	2.02	2.21	2.55	2.99	3.38	3.80	4.32	4.86	5.38	5.82
31.98	1.65	1.78	2.04	2.36	2.82	3.14	3.62	4.08	4.66	5.24	5.72
33.47	1.74	1.93	2.13	2.46	2.87	3.27	3.79	4.32	4.77	5.30	5.81
35.09	1.72	1.81	2.02	2.36	2.80	3.22	3.60	4.10	4.59	5.18	5.62
36.83	1.67	1.90	2.08	2.43	2.83	3.30	3.74	4.27	4.73	5.30	5.81
38.71	1.74	1.87	2.08	2.42	2.83	3.27	3.68	4.20	4.72	5.29	5.80
40.73	1.78	1.92	2.15	2.47	2.82	3.25	3.67	4.20	4.74	5.25	5.75
42.92	1.68	1.79	2.06	2.41	2.82	3.15	3.66	4.16	4.67	5.22	5.71
45.27	1.59	1.79	2.01	2.35	2.71	3.08	3.59	4.08	4.59	5.06	5.63
47.81	1.81	1.92	2.11	2.45	2.81	3.29	3.68	4.24	4.74	5.34	5.85
50.55	1.36	1.53	1.76	2.09	2.57	2.83	3.29	3.88	4.32	4.90	5.43
53.51	1.75	1.89	2.10	2.48	2.82	3.28	3.71	4.29	4.90	5.44	5.92
56.69	1.54	1.70	1.90	2.19	2.66	3.12	3.55	4.11	4.63	5.15	5.70
60.13	1.48	1.62	1.85	2.20	2.59	3.08	3.52	4.07	4.58	5.15	5.72
63.84	1.57	1.70	1.96	2.31	2.68	3.18	3.59	4.20	4.77	5.30	5.81
67.83	1.42	1.56	1.78	2.14	2.58	2.99	3.46	4.02	4.56	5.18	5.63
72.15	1.40	1.60	1.83	2.23	2.62	3.02	3.54	4.04	4.58	5.12	5.75
76.80	1.29	1.50	1.72	2.04	2.45	2.91	3.42	3.88	4.50	5.05	5.52
81.81	1.60	1.78	1.92	2.33	2.76	3.16	3.66	4.20	4.78	5.36	5.90
87.22	1.45	1.58	1.91	2.21	2.63	3.02	3.53	4.02	4.61	5.16	5.67
93.06	1.37	1.46	1.79	2.08	2.49	2.92	3.42	3.96	4.53	5.11	5.64
99.35	1.41	1.62	1.84	2.21	2.64	3.12	3.60	4.12	4.73	5.31	5.86
106.13	1.28	1.54	1.80	2.17	2.55	3.02	3.50	4.08	4.62	5.23	5.79
113.45	1.47	1.70	1.81	2.29	2.70	3.15	3.69	4.23	4.76	5.45	5.93
121.34	1.22	1.44	1.66	2.03	2.43	2.94	3.44	3.97	4.60	5.16	5.73
129.86	1.17	1.40	1.66	1.95	2.43	2.91	3.46	3.96	4.56	5.09	5.69
139.04	1.33	1.43	1.70	2.07	2.48	2.95	3.42	3.96	4.57	5.16	5.64
148.94	1.13	1.23	1.53	1.81	2.30	2.78	3.26	3.90	4.38	4.98	5.52
159.62	1.32	1.44	1.75	2.07	2.51	2.92	3.48	4.09	4.68	5.29	5.89
171.14	1.31	1.43	1.65	2.05	2.50	2.97	3.48	4.08	4.63	5.18	5.81
183.56	1.03	1.20	1.39	1.79	2.26	2.71	3.21	3.68	4.31	4.94	5.52
196.96	1.22	1.38	1.73	2.10	2.52	3.04	3.49	4.13	4.71	5.34	5.90
211.41	1.23	1.39	1.66	1.99	2.44	2.97	3.39	4.00	4.65	5.28	5.76
226.99	1.19	1.41	1.64	2.05	2.44	2.99	3.55	4.09	4.76	5.40	6.04
243.80	1.05	1.24	1.49	1.83	2.31	2.78	3.23	3.82	4.43	5.01	5.62
261.93	1.21	1.41	1.61	2.01	2.52	2.97	3.50	4.16	4.76	5.40	5.97

281.48	1.11	1.24	1.56	2.00	2.41	2.98	3.51	4.14	4.80	5.45	6.03
302.56	1.06	1.25	1.63	1.97	2.44	2.94	3.45	4.03	4.68	5.30	5.91
325.30	1.27	1.43	1.66	2.11	2.56	3.07	3.65	4.17	4.86	5.52	6.15
349.83	1.06	1.27	1.55	1.95	2.47	2.92	3.49	4.15	4.72	5.37	6.03
376.28	1.12	1.31	1.61	2.04	2.56	2.95	3.55	4.25	4.84	5.59	6.13
404.81	1.39	1.57	1.82	2.23	2.74	3.29	3.80	4.38	5.00	5.68	6.31
435.58	1.16	1.33	1.64	2.08	2.54	3.01	3.58	4.28	4.90	5.59	6.18
468.76	1.32	1.51	1.78	2.18	2.60	3.18	3.61	4.33	4.98	5.63	6.31
504.55	1.16	1.37	1.70	2.07	2.51	3.10	3.66	4.27	4.89	5.52	6.22
543.15	1.26	1.36	1.71	2.13	2.66	3.12	3.73	4.36	5.08	5.65	6.38
584.78	1.22	1.34	1.73	2.17	2.57	3.05	3.61	4.28	4.92	5.62	6.31
629.68	1.29	1.57	1.84	2.20	2.68	3.17	3.74	4.32	5.02	5.68	6.31
678.10	1.22	1.48	1.78	2.21	2.64	3.28	3.92	4.58	5.27	5.96	6.63
730.33	1.49	1.71	2.08	2.45	2.97	3.52	4.15	4.79	5.47	6.21	6.91
786.65	1.46	1.68	2.12	2.48	3.01	3.52	4.08	4.70	5.45	6.14	6.77
847.40	1.44	1.65	1.96	2.38	2.90	3.47	4.07	4.77	5.43	6.15	6.83
912.92	1.58	1.86	2.11	2.56	3.05	3.64	4.24	4.96	5.60	6.32	7.00
983.58	1.48	1.73	2.03	2.50	3.15	3.63	4.25	4.90	5.60	6.36	7.08
1059.78	1.69	1.88	2.23	2.68	3.22	3.77	4.37	5.13	5.82	6.63	7.28
1141.98	1.60	1.88	2.21	2.58	3.17	3.73	4.29	5.02	5.73	6.45	7.12
1230.62	1.53	1.75	2.09	2.51	3.04	3.54	4.13	4.83	5.51	6.18	6.91
1326.23	1.76	1.97	2.28	2.74	3.27	3.82	4.46	5.12	5.93	6.63	7.32
1429.34	1.91	2.19	2.55	3.02	3.57	4.11	4.75	5.52	6.26	6.99	7.74
1540.54	1.94	2.16	2.46	3.02	3.51	4.07	4.71	5.45	6.16	6.91	7.63
1660.48	1.93	2.09	2.50	2.90	3.49	4.13	4.76	5.43	6.24	7.01	7.71
1789.83	1.92	2.13	2.54	2.92	3.52	4.12	4.77	5.42	6.25	6.97	7.75
1929.34	2.11	2.37	2.67	3.23	3.76	4.33	4.96	5.68	6.46	7.24	7.99
2079.80	2.16	2.42	2.72	3.17	3.73	4.34	4.97	5.65	6.36	7.19	7.90
2242.08	2.10	2.42	2.70	3.19	3.70	4.32	4.96	5.71	6.45	7.21	7.96
2417.10	2.13	2.47	2.88	3.39	3.83	4.43	5.01	5.74	6.56	7.26	7.98
2605.85	2.25	2.47	2.76	3.29	3.87	4.39	5.07	5.75	6.52	7.21	7.97
2809.43	2.13	2.40	2.67	3.11	3.60	4.15	4.79	5.40	6.15	6.90	7.56
3028.99	2.14	2.32	2.69	3.19	3.76	4.33	4.95	5.68	6.37	7.14	7.92
3265.79	2.32	2.57	2.86	3.36	3.92	4.49	5.12	5.81	6.46	7.24	8.02
3521.18	2.29	2.55	2.88	3.27	3.87	4.43	5.11	5.75	6.48	7.21	7.90
3796.62	2.06	2.29	2.75	3.14	3.59	4.19	4.73	5.45	6.14	6.91	7.66

Wavelength (nm) / Time (ps)	553.11	554.52	555.93	557.34	558.75	560.16	561.57	562.97	564.38	565.79	567.20
-3.28	-0.09	-0.06	-0.09	-0.10	-0.04	-0.06	-0.12	-0.05	-0.06	-0.06	-0.04
-2.78	-0.07	-0.10	-0.12	-0.11	-0.09	-0.11	-0.10	-0.03	-0.11	-0.11	-0.07
-2.28	-0.06	-0.09	-0.07	-0.02	-0.05	-0.04	-0.05	-0.05	-0.08	-0.01	-0.05
-1.78	0.24	0.28	0.25	0.18	0.24	0.23	0.23	0.21	0.21	0.20	0.23
-1.28	0.01	-0.02	-0.08	0.01	-0.04	-0.02	-0.04	-0.06	-0.03	-0.02	-0.07
-0.78	-0.17	-0.11	-0.10	-0.06	-0.09	-0.10	-0.07	-0.08	-0.09	-0.15	-0.11
-0.28	0.10	0.01	0.06	0.09	0.06	0.05	0.09	0.05	0.11	0.08	0.07
0.22	0.10	0.12	0.10	0.05	0.08	0.07	0.10	0.09	0.08	0.09	0.07
0.32	-0.11	-0.06	-0.05	-0.12	-0.08	-0.07	-0.07	-0.05	-0.07	-0.05	-0.03
0.42	0.07	0.04	0.08	0.08	0.02	0.04	0.03	-0.03	0.03	0.03	0.00

0.52	-0.03	-0.07	-0.03	-0.06	0.02	0.04	-0.06	-0.03	0.01	-0.09	-0.08
0.62	0.08	0.00	0.05	0.03	0.08	0.02	0.10	0.03	0.02	0.06	0.07
0.72	0.18	0.08	0.16	0.10	0.14	0.17	0.08	0.10	0.08	0.09	0.09
0.77	0.04	0.03	0.03	0.08	0.02	0.05	0.06	0.06	0.07	0.03	0.03
0.82	0.11	0.04	0.02	0.06	0.06	0.05	0.07	0.03	0.03	0.00	0.05
0.87	0.03	0.07	0.08	0.02	0.06	0.09	0.05	0.03	0.11	0.08	0.01
0.92	0.00	-0.06	-0.06	-0.07	-0.04	-0.06	-0.10	-0.07	-0.02	-0.10	-0.09
0.97	0.01	-0.02	0.02	0.01	0.02	0.08	0.06	0.04	0.05	0.02	0.06
1.02	-0.17	-0.15	-0.12	-0.09	-0.07	-0.09	-0.10	-0.13	-0.13	-0.09	-0.10
1.07	0.14	0.13	0.15	0.13	0.12	0.12	0.13	0.10	0.15	0.10	0.10
1.12	-0.20	-0.18	-0.21	-0.25	-0.18	-0.19	-0.16	-0.19	-0.21	-0.19	-0.15
1.17	-0.15	-0.14	-0.09	-0.14	-0.07	-0.08	-0.14	-0.09	-0.11	-0.12	-0.11
1.22	-0.07	-0.11	-0.05	-0.15	-0.07	-0.06	-0.03	-0.06	-0.02	-0.06	-0.05
1.27	0.09	0.10	0.09	0.14	0.07	0.11	0.09	0.08	0.11	0.12	0.08
1.32	0.05	0.08	0.07	0.04	0.04	0.08	0.05	0.05	0.04	0.06	0.03
1.37	-0.01	-0.06	-0.06	-0.10	-0.02	0.00	-0.07	-0.05	-0.05	-0.06	-0.05
1.42	-0.13	-0.10	-0.08	-0.03	-0.08	-0.07	-0.07	-0.05	-0.10	-0.13	-0.09
1.47	-0.07	-0.14	-0.13	-0.14	-0.13	-0.12	-0.19	-0.21	-0.19	-0.30	-0.30
1.52	1.92	1.93	1.89	1.79	1.76	1.69	1.60	1.46	1.34	1.21	1.05
1.57	4.59	4.84	5.20	5.31	5.49	5.61	5.71	5.71	5.70	5.66	5.57
1.62	4.90	5.51	6.08	6.57	7.03	7.54	7.89	8.22	8.45	8.69	8.89
1.67	3.91	4.64	5.32	5.91	6.49	7.02	7.52	7.94	8.40	8.76	9.12
1.72	3.31	3.95	4.59	5.18	5.74	6.25	6.73	7.14	7.57	7.88	8.19
1.77	2.99	3.51	4.13	4.64	5.16	5.69	6.13	6.54	6.88	7.22	7.50
1.82	2.69	3.24	3.73	4.19	4.72	5.10	5.53	5.88	6.24	6.53	6.80
1.87	2.77	3.26	3.77	4.21	4.61	5.03	5.39	5.72	6.01	6.31	6.55
1.92	2.87	3.36	3.86	4.30	4.68	5.10	5.47	5.76	6.12	6.33	6.63
1.97	2.96	3.45	3.93	4.38	4.86	5.24	5.58	5.90	6.19	6.43	6.72
2.02	3.34	3.82	4.28	4.74	5.16	5.49	5.82	6.11	6.38	6.63	6.84
2.07	3.36	3.82	4.32	4.75	5.15	5.51	5.82	6.09	6.37	6.57	6.79
2.12	3.98	4.41	4.89	5.32	5.74	6.08	6.48	6.69	6.98	7.18	7.42
2.17	3.97	4.46	4.84	5.26	5.67	6.00	6.28	6.56	6.76	6.99	7.18
2.22	3.88	4.33	4.79	5.17	5.63	5.95	6.23	6.49	6.77	6.93	7.08
2.27	4.10	4.59	5.02	5.35	5.74	6.03	6.38	6.61	6.86	7.00	7.22
2.32	4.18	4.57	5.03	5.40	5.74	6.03	6.34	6.62	6.77	7.00	7.16
2.37	4.28	4.73	5.19	5.57	5.96	6.30	6.57	6.75	7.06	7.20	7.37
2.42	4.49	4.93	5.37	5.74	6.13	6.42	6.68	6.93	7.11	7.30	7.46
2.47	4.38	4.78	5.26	5.58	5.89	6.25	6.52	6.72	6.95	7.09	7.22
2.52	4.63	5.03	5.40	5.79	6.21	6.51	6.74	7.02	7.19	7.33	7.50
2.57	4.40	4.83	5.31	5.65	5.97	6.29	6.56	6.72	6.94	7.13	7.27
2.62	4.82	5.26	5.64	5.98	6.33	6.61	6.87	7.05	7.28	7.41	7.54
2.67	4.54	4.94	5.40	5.68	6.04	6.33	6.56	6.74	6.94	7.03	7.18
2.72	4.91	5.31	5.73	6.07	6.46	6.69	6.96	7.12	7.24	7.45	7.56
2.77	5.20	5.61	6.01	6.37	6.65	6.98	7.20	7.41	7.59	7.70	7.83
2.82	5.08	5.46	5.80	6.16	6.50	6.79	7.02	7.20	7.33	7.48	7.60
2.87	4.93	5.43	5.81	6.08	6.45	6.73	7.01	7.17	7.30	7.45	7.58
2.92	5.08	5.52	5.89	6.21	6.53	6.87	7.04	7.28	7.37	7.57	7.69
2.97	5.10	5.53	5.91	6.32	6.59	6.90	7.10	7.32	7.45	7.56	7.66
3.02	5.45	5.87	6.22	6.62	6.92	7.18	7.41	7.61	7.79	7.88	8.02
3.07	5.40	5.78	6.24	6.53	6.86	7.19	7.34	7.53	7.71	7.80	7.84

3.12	5.60	6.01	6.43	6.78	7.10	7.39	7.58	7.78	7.93	8.00	8.10
3.17	5.70	6.09	6.54	6.85	7.14	7.41	7.63	7.82	7.98	8.04	8.14
3.22	5.65	6.04	6.46	6.82	7.13	7.37	7.61	7.75	7.93	8.01	8.07
3.27	5.53	5.99	6.31	6.65	7.01	7.21	7.44	7.60	7.75	7.86	7.93
3.32	5.90	6.31	6.73	7.03	7.38	7.66	7.83	8.04	8.22	8.29	8.36
3.37	5.50	5.91	6.30	6.65	6.91	7.22	7.41	7.55	7.69	7.78	7.88
3.42	5.83	6.24	6.65	6.96	7.28	7.55	7.76	7.93	8.10	8.15	8.24
3.47	5.80	6.16	6.60	6.96	7.24	7.54	7.78	7.97	8.04	8.09	8.23
3.52	5.63	6.08	6.46	6.82	7.13	7.34	7.57	7.76	7.89	8.01	8.02
3.57	5.55	5.94	6.34	6.71	7.01	7.22	7.42	7.63	7.72	7.78	7.85
3.62	5.86	6.30	6.66	7.00	7.30	7.57	7.72	7.91	8.01	8.07	8.15
3.67	5.62	6.08	6.45	6.73	7.04	7.38	7.48	7.64	7.82	7.84	7.95
3.72	5.87	6.27	6.61	7.00	7.27	7.54	7.77	7.86	8.01	8.08	8.14
3.92	6.25	6.66	7.11	7.46	7.72	8.02	8.25	8.40	8.54	8.54	8.59
4.12	6.05	6.44	6.86	7.21	7.44	7.79	7.90	8.03	8.20	8.28	8.30
4.32	5.92	6.40	6.81	7.12	7.39	7.69	7.88	7.99	8.07	8.08	8.15
4.52	6.11	6.54	6.98	7.28	7.61	7.83	8.03	8.11	8.26	8.29	8.34
4.72	6.14	6.54	6.82	7.19	7.50	7.79	7.97	8.05	8.17	8.22	8.24
4.92	6.49	6.95	7.33	7.70	8.01	8.23	8.41	8.55	8.61	8.68	8.72
5.12	6.32	6.80	7.16	7.46	7.75	8.01	8.20	8.31	8.37	8.46	8.48
5.32	6.54	6.97	7.37	7.66	7.98	8.24	8.42	8.55	8.68	8.66	8.72
5.52	6.61	7.01	7.44	7.76	8.07	8.24	8.43	8.55	8.68	8.69	8.71
5.72	6.75	7.17	7.58	7.89	8.19	8.45	8.63	8.71	8.80	8.85	8.85
5.92	6.45	6.85	7.27	7.56	7.85	8.09	8.31	8.42	8.55	8.54	8.53
6.12	6.67	7.07	7.48	7.80	8.15	8.40	8.42	8.53	8.66	8.69	8.66
6.32	6.32	6.72	7.20	7.46	7.78	8.02	8.22	8.34	8.41	8.47	8.43
6.52	6.44	6.85	7.28	7.57	7.93	8.10	8.30	8.40	8.51	8.49	8.48
6.72	6.89	7.31	7.70	8.06	8.37	8.60	8.78	8.90	8.95	8.96	9.00
6.92	6.48	6.92	7.25	7.60	7.91	8.18	8.33	8.42	8.52	8.58	8.54
7.12	6.46	6.89	7.28	7.62	7.85	8.09	8.32	8.39	8.52	8.50	8.49
7.32	6.75	7.18	7.57	7.86	8.19	8.40	8.62	8.72	8.82	8.84	8.85
7.52	6.57	7.04	7.42	7.66	8.07	8.20	8.39	8.53	8.61	8.63	8.66
7.72	6.58	7.01	7.42	7.78	8.13	8.28	8.46	8.54	8.66	8.67	8.64
7.92	6.55	6.97	7.36	7.66	7.99	8.21	8.40	8.46	8.53	8.56	8.58
8.12	6.67	7.12	7.53	7.78	8.05	8.31	8.44	8.61	8.70	8.70	8.71
8.32	6.59	7.00	7.39	7.70	7.99	8.26	8.41	8.51	8.57	8.61	8.58
8.52	6.47	6.86	7.24	7.56	7.79	8.00	8.21	8.31	8.38	8.37	8.39
8.72	6.56	7.02	7.43	7.77	8.10	8.34	8.45	8.59	8.71	8.66	8.70
8.92	6.37	6.85	7.26	7.58	7.87	8.14	8.29	8.41	8.42	8.47	8.46
9.12	6.71	7.17	7.57	7.83	8.14	8.35	8.53	8.62	8.72	8.72	8.71
9.32	6.24	6.64	7.07	7.36	7.62	7.88	8.01	8.12	8.18	8.13	8.19
9.52	6.55	6.95	7.41	7.77	8.02	8.26	8.40	8.52	8.58	8.63	8.63
9.72	6.52	6.97	7.30	7.66	7.94	8.14	8.29	8.41	8.41	8.47	8.42
9.92	6.53	6.96	7.32	7.66	7.94	8.19	8.31	8.40	8.49	8.48	8.52
10.12	6.66	7.12	7.53	7.88	8.18	8.40	8.58	8.70	8.78	8.76	8.73
10.32	6.21	6.57	6.97	7.30	7.62	7.83	7.93	8.05	8.16	8.12	8.08
10.52	6.33	6.78	7.19	7.51	7.87	8.04	8.23	8.31	8.39	8.35	8.37
10.72	6.58	7.07	7.43	7.73	8.06	8.29	8.45	8.54	8.64	8.65	8.56
10.92	6.53	6.91	7.26	7.61	7.87	8.09	8.23	8.31	8.43	8.42	8.41
11.12	6.40	6.82	7.23	7.54	7.80	8.03	8.21	8.25	8.36	8.34	8.31

11.32	6.65	7.06	7.46	7.83	8.04	8.32	8.46	8.51	8.60	8.59	8.58
11.52	6.30	6.78	7.12	7.45	7.72	7.98	8.12	8.21	8.34	8.31	8.29
11.72	6.38	6.77	7.16	7.42	7.76	7.95	8.15	8.21	8.26	8.30	8.28
11.92	6.33	6.77	7.24	7.45	7.76	7.92	8.12	8.19	8.27	8.22	8.27
12.12	6.63	7.09	7.50	7.79	8.13	8.33	8.43	8.56	8.69	8.64	8.60
12.32	6.51	6.96	7.32	7.63	7.98	8.16	8.31	8.44	8.47	8.45	8.42
12.52	6.36	6.70	7.17	7.45	7.72	7.90	8.09	8.21	8.25	8.27	8.24
12.72	6.51	6.88	7.27	7.54	7.82	8.09	8.15	8.26	8.31	8.30	8.28
12.92	6.51	6.95	7.29	7.62	7.86	8.12	8.28	8.39	8.44	8.40	8.38
13.12	6.18	6.61	6.97	7.32	7.62	7.86	7.99	8.07	8.17	8.15	8.13
13.32	6.45	6.91	7.26	7.61	7.93	8.18	8.34	8.37	8.45	8.49	8.43
13.52	6.21	6.63	7.04	7.30	7.59	7.76	7.94	8.03	8.06	8.06	8.03
13.72	6.69	7.11	7.51	7.78	8.04	8.31	8.42	8.50	8.56	8.57	8.51
13.92	6.29	6.71	7.11	7.37	7.70	7.96	8.06	8.10	8.23	8.17	8.15
14.00	6.66	7.11	7.42	7.77	8.06	8.28	8.42	8.52	8.65	8.55	8.55
14.08	6.45	6.84	7.28	7.57	7.83	8.04	8.14	8.32	8.36	8.32	8.30
14.17	6.31	6.66	7.12	7.41	7.67	7.90	8.07	8.13	8.20	8.15	8.15
14.27	6.26	6.63	7.07	7.38	7.69	7.86	8.07	8.17	8.18	8.17	8.06
14.38	6.42	6.85	7.19	7.52	7.82	8.07	8.16	8.28	8.31	8.26	8.27
14.49	6.20	6.64	6.98	7.25	7.56	7.78	7.92	7.96	8.03	8.04	7.98
14.61	6.23	6.64	7.09	7.38	7.72	7.90	8.02	8.10	8.21	8.18	8.14
14.75	6.19	6.63	7.06	7.34	7.58	7.84	7.97	8.11	8.13	8.10	8.10
14.89	6.88	7.30	7.71	8.08	8.32	8.59	8.71	8.80	8.83	8.78	8.75
15.05	6.23	6.64	7.06	7.39	7.68	7.92	8.05	8.14	8.22	8.16	8.12
15.21	6.46	6.96	7.27	7.64	7.91	8.10	8.28	8.38	8.39	8.39	8.34
15.39	6.20	6.56	6.96	7.26	7.57	7.76	7.90	7.96	7.93	8.05	7.96
15.59	6.31	6.81	7.19	7.49	7.77	8.04	8.15	8.22	8.23	8.28	8.24
15.80	6.55	7.03	7.39	7.74	8.01	8.22	8.40	8.47	8.52	8.50	8.47
16.02	6.12	6.55	6.86	7.25	7.49	7.69	7.82	7.94	7.96	7.97	7.95
16.27	6.67	7.12	7.51	7.85	8.13	8.35	8.46	8.51	8.58	8.57	8.55
16.53	6.31	6.71	7.15	7.46	7.73	7.90	8.09	8.18	8.24	8.22	8.12
16.81	6.10	6.50	6.90	7.23	7.49	7.75	7.89	7.96	8.02	7.98	7.98
17.12	6.52	6.97	7.42	7.64	8.00	8.15	8.29	8.40	8.47	8.41	8.38
17.45	6.09	6.47	6.92	7.27	7.53	7.73	7.82	7.93	8.02	7.95	7.90
17.81	6.55	7.07	7.41	7.75	8.02	8.26	8.35	8.45	8.54	8.44	8.48
18.19	6.64	7.07	7.50	7.80	8.11	8.29	8.51	8.59	8.62	8.56	8.53
18.60	6.52	6.97	7.33	7.65	8.03	8.14	8.34	8.43	8.45	8.41	8.39
19.05	6.52	6.97	7.31	7.67	7.96	8.19	8.29	8.35	8.42	8.39	8.33
19.53	6.28	6.77	7.17	7.50	7.77	8.01	8.16	8.23	8.31	8.27	8.18
20.05	6.38	6.80	7.21	7.50	7.80	8.03	8.10	8.22	8.25	8.26	8.19
20.61	6.39	6.81	7.21	7.55	7.78	8.01	8.12	8.20	8.23	8.24	8.21
21.22	6.34	6.79	7.14	7.48	7.73	7.93	8.09	8.14	8.21	8.13	8.11
21.87	6.40	6.86	7.23	7.54	7.78	8.05	8.18	8.28	8.26	8.23	8.25
22.57	6.34	6.80	7.21	7.48	7.76	8.01	8.09	8.20	8.24	8.17	8.15
23.33	6.47	6.88	7.35	7.63	7.89	8.16	8.27	8.36	8.34	8.35	8.31
24.15	6.22	6.65	7.03	7.36	7.59	7.82	8.00	8.04	8.11	8.06	7.99
25.03	6.24	6.67	7.07	7.34	7.67	7.86	7.98	8.04	8.07	8.03	8.01
25.98	6.60	7.05	7.45	7.85	8.06	8.31	8.43	8.53	8.56	8.48	8.42
27.01	6.16	6.62	6.99	7.31	7.54	7.75	7.88	7.96	8.01	7.97	7.91
28.11	6.53	6.96	7.40	7.67	7.96	8.18	8.36	8.39	8.36	8.39	8.33

29.30	6.39	6.85	7.23	7.59	7.84	8.10	8.21	8.28	8.31	8.32	8.20
30.59	6.38	6.79	7.23	7.52	7.74	8.01	8.12	8.16	8.19	8.19	8.15
31.98	6.22	6.68	7.08	7.38	7.67	7.88	8.01	8.08	8.10	8.09	8.03
33.47	6.30	6.72	7.14	7.47	7.73	7.98	8.06	8.14	8.16	8.13	8.08
35.09	6.18	6.63	7.03	7.30	7.64	7.82	7.93	8.02	8.07	7.97	7.97
36.83	6.32	6.76	7.16	7.48	7.85	8.07	8.19	8.24	8.33	8.23	8.21
38.71	6.24	6.70	7.08	7.38	7.61	7.86	7.95	8.05	8.10	8.08	8.01
40.73	6.28	6.65	7.07	7.36	7.67	7.83	7.98	8.01	8.06	8.04	7.98
42.92	6.23	6.71	7.03	7.34	7.61	7.87	7.99	8.04	8.02	8.03	7.98
45.27	6.10	6.51	6.89	7.18	7.47	7.71	7.79	7.87	7.92	7.89	7.76
47.81	6.37	6.78	7.22	7.53	7.79	7.99	8.14	8.20	8.26	8.17	8.06
50.55	5.92	6.32	6.76	7.01	7.34	7.57	7.65	7.74	7.75	7.72	7.68
53.51	6.45	6.89	7.34	7.66	7.95	8.14	8.31	8.38	8.41	8.38	8.33
56.69	6.21	6.65	7.06	7.40	7.70	7.85	8.01	8.08	8.16	8.07	8.06
60.13	6.22	6.74	7.12	7.43	7.71	7.95	8.11	8.17	8.25	8.13	8.11
63.84	6.34	6.84	7.25	7.54	7.79	8.09	8.20	8.30	8.34	8.30	8.26
67.83	6.11	6.69	7.08	7.39	7.68	7.95	8.11	8.15	8.18	8.13	8.07
72.15	6.29	6.72	7.21	7.48	7.82	8.05	8.15	8.25	8.30	8.29	8.17
76.80	6.09	6.48	6.92	7.25	7.56	7.78	7.92	8.02	8.07	8.02	7.93
81.81	6.46	6.89	7.32	7.69	7.97	8.22	8.32	8.41	8.48	8.40	8.32
87.22	6.19	6.70	7.08	7.41	7.72	7.96	8.07	8.13	8.17	8.12	8.08
93.06	6.17	6.67	7.03	7.44	7.72	7.96	8.11	8.23	8.23	8.19	8.13
99.35	6.41	6.91	7.33	7.70	8.01	8.24	8.39	8.44	8.52	8.45	8.44
106.13	6.32	6.82	7.30	7.60	7.93	8.16	8.30	8.41	8.39	8.45	8.33
113.45	6.47	7.03	7.44	7.81	8.13	8.38	8.53	8.58	8.62	8.62	8.55
121.34	6.28	6.78	7.24	7.63	7.92	8.16	8.31	8.38	8.46	8.40	8.27
129.86	6.26	6.67	7.13	7.47	7.86	8.07	8.16	8.31	8.31	8.31	8.28
139.04	6.18	6.66	7.06	7.51	7.80	8.05	8.18	8.26	8.31	8.27	8.22
148.94	6.11	6.62	7.05	7.36	7.74	8.01	8.15	8.25	8.35	8.29	8.24
159.62	6.44	6.97	7.42	7.76	8.10	8.36	8.54	8.64	8.71	8.69	8.66
171.14	6.37	6.82	7.32	7.66	7.99	8.29	8.39	8.55	8.57	8.57	8.52
183.56	6.06	6.59	7.04	7.37	7.73	7.97	8.13	8.24	8.27	8.25	8.20
196.96	6.48	7.02	7.54	7.90	8.27	8.49	8.70	8.80	8.87	8.86	8.81
211.41	6.35	6.90	7.36	7.78	8.09	8.35	8.51	8.63	8.66	8.65	8.57
226.99	6.58	7.12	7.61	7.94	8.32	8.60	8.76	8.93	8.92	8.91	8.85
243.80	6.16	6.67	7.16	7.52	7.83	8.15	8.31	8.38	8.46	8.41	8.39
261.93	6.56	7.14	7.64	8.04	8.40	8.62	8.85	8.98	9.00	9.00	8.95
281.48	6.71	7.22	7.77	8.12	8.48	8.82	9.01	9.14	9.22	9.19	9.13
302.56	6.51	7.05	7.56	7.98	8.29	8.61	8.79	8.96	9.02	8.95	8.92
325.30	6.75	7.35	7.72	8.17	8.54	8.85	9.07	9.15	9.25	9.23	9.18
349.83	6.55	7.15	7.67	8.05	8.49	8.68	8.95	9.04	9.14	9.08	9.09
376.28	6.81	7.37	7.85	8.30	8.66	8.97	9.16	9.35	9.47	9.45	9.38
404.81	6.98	7.46	7.97	8.38	8.77	9.06	9.28	9.44	9.46	9.47	9.47
435.58	6.77	7.39	7.85	8.32	8.70	9.03	9.22	9.40	9.48	9.48	9.42
468.76	6.90	7.50	8.03	8.43	8.82	9.15	9.32	9.47	9.53	9.58	9.55
504.55	6.83	7.37	7.91	8.36	8.75	9.06	9.29	9.44	9.52	9.50	9.49
543.15	6.96	7.54	8.07	8.59	8.93	9.30	9.52	9.67	9.82	9.79	9.80
584.78	6.91	7.48	7.98	8.48	8.91	9.16	9.40	9.57	9.66	9.70	9.67
629.68	6.85	7.47	7.99	8.41	8.81	9.11	9.37	9.54	9.62	9.61	9.62
678.10	7.33	7.97	8.50	9.01	9.41	9.78	10.02	10.14	10.25	10.36	10.34

730.33	7.56	8.13	8.70	9.20	9.62	10.01	10.25	10.45	10.55	10.57	10.57
786.65	7.42	8.11	8.63	9.17	9.56	10.00	10.22	10.38	10.52	10.54	10.52
847.40	7.55	8.17	8.75	9.25	9.71	10.11	10.38	10.59	10.69	10.69	10.73
912.92	7.70	8.37	8.94	9.45	9.92	10.30	10.60	10.73	10.88	10.90	10.89
983.58	7.81	8.48	9.07	9.59	10.06	10.46	10.72	10.91	11.07	11.11	11.12
1059.78	8.01	8.59	9.23	9.68	10.16	10.57	10.86	11.09	11.25	11.29	11.26
1141.98	7.88	8.52	9.06	9.63	10.13	10.49	10.80	10.98	11.19	11.21	11.28
1230.62	7.59	8.25	8.81	9.35	9.75	10.13	10.45	10.63	10.79	10.82	10.84
1326.23	8.02	8.74	9.31	9.88	10.29	10.73	11.02	11.27	11.41	11.50	11.51
1429.34	8.49	9.19	9.78	10.36	10.90	11.32	11.60	11.86	12.02	12.07	12.15
1540.54	8.39	9.05	9.66	10.25	10.75	11.21	11.50	11.70	11.86	11.94	11.97
1660.48	8.46	9.20	9.87	10.38	10.89	11.30	11.63	11.87	12.08	12.15	12.22
1789.83	8.53	9.16	9.85	10.44	10.97	11.34	11.65	11.93	12.11	12.15	12.19
1929.34	8.72	9.42	10.04	10.58	11.18	11.59	11.89	12.18	12.36	12.45	12.48
2079.80	8.62	9.27	9.96	10.51	11.00	11.49	11.76	12.08	12.21	12.35	12.39
2242.08	8.69	9.38	10.05	10.60	11.16	11.59	11.88	12.17	12.36	12.41	12.50
2417.10	8.75	9.47	10.11	10.64	11.21	11.68	12.01	12.22	12.41	12.53	12.57
2605.85	8.67	9.38	9.99	10.56	11.13	11.59	11.94	12.16	12.32	12.44	12.44
2809.43	8.28	8.95	9.61	10.09	10.59	11.02	11.38	11.59	11.80	11.91	11.95
3028.99	8.61	9.36	10.02	10.53	11.06	11.50	11.90	12.15	12.37	12.48	12.51
3265.79	8.72	9.45	9.97	10.61	11.14	11.59	11.85	12.18	12.37	12.44	12.60
3521.18	8.65	9.29	9.99	10.51	11.08	11.49	11.80	12.09	12.30	12.46	12.49
3796.62	8.38	9.01	9.66	10.18	10.75	11.16	11.46	11.77	11.97	12.10	12.10

Wavelength (nm) / Time (ps)	568.61	570.02	571.43	572.83	574.24	575.65	577.06	578.47	579.88	581.29	582.69
-3.28	-0.04	-0.06	-0.07	-0.07	-0.05	-0.04	-0.04	-0.03	-0.04	-0.01	0.00
-2.78	-0.07	-0.08	-0.08	-0.11	-0.08	-0.07	-0.07	-0.06	-0.03	-0.04	-0.05
-2.28	-0.06	-0.06	-0.02	-0.04	-0.06	-0.07	-0.08	-0.07	-0.05	-0.05	-0.04
-1.78	0.20	0.19	0.21	0.18	0.22	0.21	0.19	0.16	0.14	0.16	0.14
-1.28	-0.04	-0.05	0.01	-0.02	-0.03	-0.04	-0.03	-0.02	-0.01	-0.06	-0.07
-0.78	-0.12	-0.06	-0.09	-0.11	-0.13	-0.08	-0.10	-0.04	-0.07	-0.05	0.01
-0.28	0.06	0.08	0.03	0.05	0.04	0.08	0.07	0.04	0.02	0.04	0.04
0.22	0.08	0.04	0.04	0.13	0.10	0.06	0.09	0.07	0.07	0.06	0.00
0.32	-0.06	-0.06	-0.02	-0.06	-0.05	-0.09	-0.02	-0.05	-0.06	-0.01	-0.01
0.42	0.06	0.04	-0.01	0.05	0.03	0.02	-0.01	-0.01	0.04	-0.03	-0.02
0.52	-0.01	-0.05	-0.01	-0.03	-0.01	-0.02	-0.03	-0.02	-0.05	0.03	0.00
0.62	0.08	0.05	0.07	0.07	0.06	0.04	0.01	0.04	0.06	0.03	0.04
0.72	0.11	0.06	0.10	0.06	0.03	0.05	0.08	0.01	0.05	0.01	-0.03
0.77	0.07	0.04	0.03	0.06	0.07	0.05	0.02	-0.01	0.05	0.03	0.01
0.82	0.02	0.00	0.00	0.02	0.01	0.05	-0.01	-0.03	0.03	-0.03	0.02
0.87	0.04	0.02	0.05	0.10	0.04	0.06	0.08	0.04	0.07	0.06	0.07
0.92	-0.13	-0.11	-0.08	-0.06	-0.08	-0.07	-0.10	-0.10	-0.08	-0.11	-0.14
0.97	0.05	0.03	0.04	0.00	0.05	0.09	0.05	0.06	0.08	0.04	0.04
1.02	-0.15	-0.13	-0.12	-0.09	-0.08	-0.13	-0.09	-0.06	-0.09	-0.09	-0.06
1.07	0.13	0.11	0.14	0.11	0.13	0.11	0.09	0.09	0.08	0.06	0.09
1.12	-0.15	-0.19	-0.15	-0.20	-0.18	-0.15	-0.18	-0.12	-0.21	-0.18	-0.16
1.17	-0.09	-0.15	-0.10	-0.16	-0.14	-0.15	-0.12	-0.06	-0.09	-0.09	-0.08
1.22	-0.11	-0.09	-0.05	-0.07	-0.08	-0.10	-0.12	-0.14	-0.08	-0.08	-0.08

1.27	0.12	0.08	0.06	0.12	0.09	0.15	0.09	0.10	0.08	0.04	0.07
1.32	0.08	0.08	0.08	0.03	0.02	0.06	0.08	0.07	0.07	0.03	0.05
1.37	-0.03	-0.04	-0.09	-0.09	-0.06	-0.04	0.02	-0.01	-0.02	-0.02	-0.01
1.42	-0.05	-0.12	-0.07	-0.10	-0.04	-0.04	-0.04	-0.10	-0.09	-0.02	-0.07
1.47	-0.28	-0.27	-0.26	-0.31	-0.23	-0.25	-0.20	-0.18	-0.19	-0.16	-0.17
1.52	0.90	0.76	0.68	0.57	0.44	0.39	0.28	0.29	0.19	0.13	0.14
1.57	5.45	5.20	5.02	4.74	4.55	4.28	4.00	3.75	3.58	3.35	3.14
1.62	9.00	9.04	9.06	9.03	8.98	8.88	8.77	8.60	8.40	8.24	8.10
1.67	9.37	9.57	9.79	9.94	10.05	10.16	10.21	10.27	10.31	10.35	10.29
1.72	8.46	8.73	8.97	9.17	9.32	9.43	9.58	9.63	9.69	9.72	9.80
1.77	7.83	7.96	8.19	8.41	8.53	8.63	8.77	8.82	8.91	8.92	8.90
1.82	7.04	7.26	7.38	7.55	7.67	7.80	7.95	7.91	7.98	8.04	8.07
1.87	6.78	6.96	7.14	7.31	7.42	7.54	7.61	7.69	7.70	7.70	7.68
1.92	6.84	7.01	7.22	7.34	7.44	7.53	7.67	7.66	7.74	7.73	7.69
1.97	6.91	7.02	7.21	7.36	7.53	7.59	7.66	7.68	7.79	7.75	7.76
2.02	7.07	7.14	7.34	7.45	7.61	7.66	7.71	7.79	7.79	7.78	7.81
2.07	6.98	7.09	7.27	7.40	7.52	7.56	7.67	7.73	7.75	7.75	7.76
2.12	7.61	7.69	7.87	7.97	8.05	8.14	8.17	8.24	8.27	8.24	8.17
2.17	7.35	7.46	7.58	7.69	7.77	7.80	7.91	7.90	7.88	7.88	7.89
2.22	7.34	7.47	7.56	7.65	7.73	7.83	7.87	7.86	7.86	7.85	7.81
2.27	7.36	7.45	7.62	7.69	7.79	7.80	7.88	7.89	7.92	7.90	7.81
2.32	7.27	7.40	7.51	7.60	7.66	7.67	7.73	7.72	7.73	7.75	7.69
2.37	7.53	7.59	7.69	7.78	7.87	7.90	7.93	7.97	7.96	7.93	7.95
2.42	7.59	7.63	7.82	7.86	7.92	7.95	7.97	7.97	8.01	7.95	7.91
2.47	7.34	7.42	7.49	7.58	7.58	7.68	7.68	7.64	7.63	7.62	7.58
2.52	7.62	7.72	7.79	7.89	7.90	7.95	7.94	7.95	7.95	7.94	7.87
2.57	7.38	7.43	7.53	7.61	7.66	7.67	7.70	7.69	7.71	7.70	7.69
2.62	7.64	7.71	7.76	7.79	7.79	7.83	7.86	7.88	7.84	7.83	7.74
2.67	7.24	7.32	7.42	7.45	7.50	7.50	7.53	7.54	7.50	7.50	7.44
2.72	7.65	7.69	7.76	7.80	7.81	7.85	7.85	7.80	7.81	7.81	7.68
2.77	7.93	7.96	8.02	8.04	8.07	8.04	8.06	8.00	7.99	7.99	7.92
2.82	7.69	7.72	7.77	7.80	7.92	7.81	7.86	7.79	7.76	7.73	7.69
2.87	7.63	7.67	7.76	7.78	7.75	7.78	7.76	7.76	7.73	7.70	7.65
2.92	7.77	7.75	7.81	7.79	7.85	7.87	7.87	7.82	7.78	7.77	7.70
2.97	7.74	7.70	7.78	7.82	7.86	7.83	7.82	7.84	7.78	7.74	7.69
3.02	8.06	8.09	8.15	8.16	8.14	8.13	8.13	8.09	8.02	7.97	7.91
3.07	7.99	8.01	8.02	8.04	8.00	7.98	8.02	7.98	7.94	7.89	7.83
3.12	8.20	8.19	8.22	8.28	8.27	8.26	8.23	8.14	8.14	8.09	8.01
3.17	8.22	8.25	8.29	8.26	8.27	8.25	8.24	8.18	8.13	8.08	8.01
3.22	8.15	8.15	8.20	8.19	8.18	8.20	8.12	8.10	8.04	7.95	7.88
3.27	8.06	7.97	7.98	8.00	8.02	7.99	7.97	7.89	7.84	7.79	7.73
3.32	8.42	8.39	8.46	8.45	8.38	8.37	8.36	8.25	8.17	8.14	8.04
3.37	7.94	7.94	7.93	7.91	7.86	7.87	7.88	7.80	7.76	7.71	7.66
3.42	8.26	8.26	8.31	8.27	8.27	8.21	8.20	8.08	8.07	8.01	7.90
3.47	8.29	8.20	8.23	8.25	8.23	8.18	8.17	8.08	8.06	7.99	7.95
3.52	8.00	8.01	8.05	8.04	8.00	7.96	7.97	7.86	7.85	7.80	7.67
3.57	7.85	7.89	7.89	7.88	7.82	7.75	7.74	7.69	7.62	7.56	7.46
3.62	8.22	8.14	8.14	8.14	8.06	8.05	7.98	7.91	7.85	7.73	7.65
3.67	7.96	7.97	7.93	7.92	7.91	7.87	7.77	7.70	7.66	7.61	7.53
3.72	8.17	8.10	8.10	8.05	8.09	8.02	7.93	7.88	7.83	7.74	7.61

3.92	8.65	8.59	8.58	8.54	8.52	8.43	8.39	8.33	8.20	8.14	8.00
4.12	8.30	8.26	8.21	8.21	8.13	8.06	8.01	7.89	7.84	7.79	7.66
4.32	8.17	8.10	8.09	8.03	7.95	7.88	7.89	7.71	7.64	7.54	7.41
4.52	8.39	8.27	8.25	8.19	8.12	8.02	7.96	7.84	7.81	7.69	7.56
4.72	8.24	8.22	8.14	8.15	8.04	7.96	7.93	7.80	7.71	7.58	7.46
4.92	8.66	8.62	8.54	8.53	8.46	8.35	8.23	8.11	8.02	7.90	7.76
5.12	8.41	8.41	8.32	8.27	8.17	8.05	7.97	7.85	7.73	7.62	7.51
5.32	8.74	8.67	8.63	8.53	8.41	8.27	8.19	8.07	7.98	7.81	7.65
5.52	8.70	8.66	8.50	8.44	8.39	8.29	8.16	8.03	7.90	7.81	7.64
5.72	8.85	8.77	8.67	8.62	8.48	8.41	8.24	8.11	8.01	7.89	7.71
5.92	8.54	8.48	8.42	8.29	8.17	8.11	8.00	7.89	7.74	7.62	7.51
6.12	8.68	8.55	8.51	8.42	8.33	8.16	8.07	7.95	7.78	7.69	7.55
6.32	8.44	8.38	8.27	8.20	8.08	8.01	7.85	7.74	7.63	7.52	7.34
6.52	8.46	8.40	8.34	8.22	8.14	7.96	7.88	7.76	7.65	7.51	7.36
6.72	8.99	8.87	8.82	8.69	8.57	8.45	8.36	8.17	8.04	7.91	7.69
6.92	8.58	8.46	8.35	8.30	8.17	8.03	7.92	7.75	7.65	7.51	7.37
7.12	8.46	8.37	8.29	8.21	8.08	7.96	7.84	7.72	7.60	7.44	7.25
7.32	8.78	8.71	8.62	8.50	8.43	8.25	8.15	8.00	7.82	7.70	7.54
7.52	8.61	8.53	8.40	8.32	8.21	8.04	7.89	7.73	7.66	7.51	7.32
7.72	8.60	8.51	8.40	8.30	8.21	8.08	7.92	7.80	7.63	7.53	7.41
7.92	8.55	8.41	8.31	8.22	8.08	7.94	7.83	7.65	7.55	7.38	7.24
8.12	8.64	8.51	8.44	8.32	8.21	8.03	7.90	7.78	7.64	7.48	7.33
8.32	8.56	8.47	8.38	8.24	8.11	7.97	7.86	7.65	7.56	7.40	7.24
8.52	8.33	8.22	8.16	8.01	7.89	7.81	7.68	7.54	7.41	7.25	7.07
8.72	8.65	8.54	8.44	8.35	8.20	8.08	7.97	7.82	7.66	7.54	7.36
8.92	8.42	8.26	8.19	8.12	7.95	7.86	7.74	7.57	7.44	7.30	7.17
9.12	8.65	8.54	8.44	8.33	8.22	8.04	7.93	7.76	7.60	7.43	7.29
9.32	8.14	8.02	7.92	7.84	7.74	7.61	7.47	7.28	7.11	6.98	6.87
9.52	8.54	8.44	8.37	8.23	8.13	7.96	7.84	7.67	7.55	7.41	7.18
9.72	8.42	8.34	8.22	8.08	7.96	7.82	7.70	7.55	7.37	7.22	7.06
9.92	8.48	8.34	8.23	8.11	8.00	7.87	7.73	7.54	7.39	7.23	7.10
10.12	8.70	8.55	8.47	8.35	8.20	8.04	7.90	7.76	7.63	7.46	7.30
10.32	8.05	7.93	7.84	7.71	7.58	7.44	7.33	7.18	7.07	6.91	6.75
10.52	8.32	8.20	8.11	7.97	7.86	7.74	7.63	7.45	7.31	7.20	7.00
10.72	8.53	8.45	8.29	8.17	8.00	7.88	7.69	7.59	7.45	7.26	7.11
10.92	8.32	8.19	8.15	8.06	7.89	7.66	7.60	7.44	7.28	7.12	6.96
11.12	8.28	8.17	8.08	7.95	7.77	7.65	7.51	7.35	7.23	7.07	6.96
11.32	8.53	8.39	8.26	8.15	8.00	7.84	7.68	7.56	7.36	7.23	7.06
11.52	8.19	8.07	7.97	7.84	7.70	7.53	7.41	7.28	7.11	6.98	6.84
11.72	8.16	8.09	8.00	7.88	7.74	7.55	7.46	7.32	7.14	7.01	6.85
11.92	8.20	8.10	8.03	7.83	7.70	7.54	7.39	7.25	7.13	7.03	6.87
12.12	8.54	8.44	8.34	8.18	8.04	7.88	7.74	7.61	7.44	7.24	7.11
12.32	8.39	8.31	8.15	8.03	7.86	7.72	7.57	7.39	7.28	7.10	6.92
12.52	8.15	8.04	7.92	7.71	7.64	7.44	7.33	7.16	7.02	6.87	6.69
12.72	8.20	8.06	7.97	7.85	7.69	7.60	7.37	7.21	7.07	6.91	6.75
12.92	8.35	8.26	8.12	7.94	7.84	7.71	7.56	7.37	7.17	7.04	6.87
13.12	8.04	7.95	7.82	7.64	7.53	7.42	7.27	7.10	6.97	6.83	6.66
13.32	8.35	8.25	8.12	7.98	7.88	7.70	7.63	7.42	7.27	7.11	6.98
13.52	7.99	7.89	7.73	7.62	7.47	7.33	7.21	7.04	6.85	6.73	6.58
13.72	8.43	8.34	8.21	8.03	7.89	7.71	7.63	7.40	7.24	7.07	6.92

13.92	8.11	7.92	7.80	7.70	7.56	7.40	7.27	7.13	6.99	6.83	6.67
14.00	8.47	8.37	8.22	8.10	7.93	7.76	7.59	7.43	7.28	7.12	6.93
14.08	8.22	8.10	8.01	7.89	7.70	7.59	7.42	7.25	7.08	6.93	6.78
14.17	8.09	7.95	7.89	7.72	7.55	7.40	7.28	7.13	6.99	6.83	6.66
14.27	8.04	7.92	7.84	7.68	7.55	7.40	7.25	7.07	6.98	6.83	6.66
14.38	8.18	8.05	7.96	7.75	7.66	7.51	7.35	7.19	7.05	6.86	6.73
14.49	7.95	7.78	7.70	7.55	7.38	7.30	7.13	7.00	6.81	6.67	6.48
14.61	8.10	7.93	7.85	7.69	7.53	7.36	7.22	7.07	6.97	6.81	6.66
14.75	8.03	7.90	7.83	7.62	7.55	7.32	7.27	7.07	6.98	6.79	6.57
14.89	8.67	8.56	8.40	8.25	8.11	7.98	7.79	7.62	7.42	7.24	7.04
15.05	7.99	7.96	7.83	7.67	7.47	7.30	7.23	7.01	6.87	6.72	6.65
15.21	8.28	8.15	8.02	7.87	7.70	7.55	7.42	7.22	7.05	6.90	6.71
15.39	7.90	7.78	7.65	7.57	7.37	7.21	7.10	6.92	6.83	6.62	6.45
15.59	8.13	8.04	7.89	7.74	7.63	7.42	7.28	7.14	7.01	6.88	6.69
15.80	8.38	8.23	8.12	7.99	7.84	7.70	7.51	7.36	7.21	7.06	6.85
16.02	7.86	7.74	7.63	7.50	7.36	7.19	7.04	6.91	6.72	6.59	6.44
16.27	8.49	8.30	8.19	8.01	7.89	7.72	7.57	7.38	7.21	7.06	6.88
16.53	8.06	7.96	7.83	7.69	7.52	7.38	7.22	7.08	6.90	6.75	6.61
16.81	7.85	7.77	7.66	7.53	7.33	7.21	7.09	6.88	6.77	6.62	6.45
17.12	8.34	8.16	8.04	7.86	7.69	7.57	7.38	7.21	7.10	6.85	6.75
17.45	7.87	7.77	7.60	7.50	7.35	7.17	7.04	6.85	6.73	6.56	6.47
17.81	8.37	8.23	8.14	7.95	7.81	7.68	7.51	7.31	7.16	6.98	6.82
18.19	8.40	8.28	8.15	8.03	7.80	7.67	7.51	7.29	7.15	6.93	6.81
18.60	8.34	8.14	8.02	7.88	7.71	7.54	7.39	7.24	7.02	6.89	6.72
19.05	8.27	8.13	8.00	7.84	7.65	7.50	7.36	7.15	7.00	6.85	6.66
19.53	8.15	7.96	7.87	7.70	7.52	7.40	7.21	7.00	6.89	6.75	6.61
20.05	8.14	7.94	7.86	7.68	7.55	7.34	7.22	6.98	6.89	6.72	6.55
20.61	8.11	7.99	7.89	7.69	7.52	7.37	7.20	7.07	6.92	6.72	6.58
21.22	8.03	7.87	7.78	7.59	7.38	7.23	7.14	6.90	6.78	6.59	6.43
21.87	8.15	8.01	7.88	7.68	7.57	7.37	7.20	7.04	6.85	6.71	6.60
22.57	8.08	7.92	7.77	7.63	7.48	7.29	7.13	6.93	6.79	6.63	6.45
23.33	8.20	8.08	7.93	7.75	7.61	7.42	7.25	7.11	6.92	6.77	6.59
24.15	7.89	7.76	7.67	7.50	7.36	7.16	6.99	6.81	6.70	6.45	6.34
25.03	7.93	7.76	7.67	7.52	7.31	7.14	7.02	6.81	6.66	6.49	6.33
25.98	8.34	8.17	8.00	7.86	7.71	7.52	7.36	7.19	7.01	6.86	6.66
27.01	7.85	7.69	7.59	7.39	7.27	7.07	6.92	6.74	6.60	6.41	6.24
28.11	8.27	8.05	7.95	7.76	7.57	7.43	7.26	7.07	6.85	6.72	6.57
29.30	8.12	7.96	7.81	7.63	7.49	7.27	7.16	6.95	6.78	6.65	6.44
30.59	8.07	7.86	7.72	7.57	7.42	7.22	7.07	6.86	6.68	6.56	6.37
31.98	7.91	7.76	7.61	7.50	7.24	7.11	6.92	6.76	6.61	6.44	6.25
33.47	8.00	7.85	7.63	7.47	7.34	7.16	6.99	6.86	6.65	6.49	6.31
35.09	7.87	7.69	7.57	7.35	7.25	7.07	6.91	6.70	6.57	6.42	6.21
36.83	8.11	7.93	7.80	7.59	7.44	7.27	7.07	6.95	6.78	6.59	6.46
38.71	7.92	7.74	7.61	7.42	7.25	7.05	6.88	6.70	6.51	6.36	6.20
40.73	7.89	7.75	7.59	7.41	7.27	7.05	6.87	6.67	6.55	6.35	6.23
42.92	7.89	7.68	7.56	7.40	7.23	7.05	6.90	6.67	6.51	6.35	6.17
45.27	7.73	7.52	7.39	7.20	7.07	6.88	6.73	6.55	6.35	6.19	6.02
47.81	8.06	7.89	7.77	7.54	7.34	7.16	7.00	6.79	6.63	6.47	6.31
50.55	7.60	7.44	7.30	7.11	7.02	6.80	6.63	6.45	6.27	6.16	5.96
53.51	8.22	8.08	7.92	7.76	7.52	7.39	7.23	6.98	6.80	6.65	6.44

56.69	7.96	7.74	7.67	7.43	7.30	7.12	6.93	6.80	6.57	6.41	6.25
60.13	8.00	7.83	7.70	7.50	7.32	7.16	6.95	6.77	6.62	6.41	6.21
63.84	8.09	7.94	7.86	7.63	7.45	7.27	7.02	6.88	6.70	6.48	6.35
67.83	8.00	7.84	7.64	7.51	7.31	7.09	6.92	6.76	6.60	6.44	6.22
72.15	8.04	7.91	7.79	7.61	7.39	7.22	7.07	6.87	6.70	6.50	6.34
76.80	7.86	7.73	7.58	7.34	7.22	6.98	6.84	6.70	6.49	6.30	6.15
81.81	8.20	8.04	7.91	7.73	7.54	7.31	7.14	6.92	6.76	6.58	6.36
87.22	7.99	7.81	7.70	7.50	7.28	7.12	6.94	6.71	6.56	6.34	6.18
93.06	8.02	7.86	7.72	7.54	7.34	7.18	7.04	6.83	6.59	6.43	6.29
99.35	8.33	8.16	8.05	7.85	7.64	7.39	7.27	7.05	6.81	6.68	6.40
106.13	8.22	8.09	7.90	7.77	7.58	7.41	7.15	6.93	6.78	6.62	6.41
113.45	8.41	8.27	8.17	7.92	7.78	7.56	7.37	7.13	6.91	6.74	6.59
121.34	8.27	8.10	7.93	7.76	7.58	7.34	7.20	6.93	6.80	6.63	6.44
129.86	8.19	7.99	7.81	7.65	7.48	7.26	7.10	6.91	6.68	6.53	6.36
139.04	8.14	8.01	7.85	7.67	7.45	7.25	7.05	6.86	6.69	6.51	6.34
148.94	8.17	7.94	7.84	7.70	7.45	7.29	7.12	6.92	6.72	6.57	6.32
159.62	8.50	8.32	8.19	8.00	7.77	7.58	7.40	7.19	6.98	6.80	6.60
171.14	8.37	8.23	8.07	7.91	7.76	7.52	7.34	7.11	6.96	6.76	6.55
183.56	8.11	7.97	7.78	7.65	7.41	7.25	7.01	6.89	6.71	6.49	6.32
196.96	8.69	8.54	8.33	8.17	8.01	7.80	7.63	7.40	7.23	6.97	6.86
211.41	8.52	8.38	8.24	8.02	7.86	7.62	7.44	7.21	7.04	6.81	6.67
226.99	8.80	8.60	8.48	8.24	8.10	7.82	7.66	7.47	7.25	7.05	6.79
243.80	8.36	8.20	8.05	7.86	7.66	7.52	7.33	7.12	6.92	6.73	6.51
261.93	8.85	8.71	8.51	8.36	8.15	7.95	7.76	7.53	7.26	7.11	6.90
281.48	9.03	8.88	8.73	8.48	8.30	8.07	7.91	7.73	7.45	7.30	7.08
302.56	8.88	8.68	8.51	8.35	8.18	8.00	7.79	7.52	7.36	7.13	6.94
325.30	9.09	8.91	8.79	8.59	8.41	8.18	8.01	7.80	7.55	7.32	7.09
349.83	9.01	8.78	8.67	8.49	8.30	8.10	7.92	7.66	7.52	7.26	7.02
376.28	9.31	9.15	9.00	8.85	8.62	8.38	8.26	8.00	7.77	7.54	7.34
404.81	9.42	9.25	9.14	8.89	8.73	8.53	8.27	8.04	7.83	7.62	7.41
435.58	9.40	9.24	9.06	8.88	8.64	8.48	8.27	8.01	7.77	7.59	7.38
468.76	9.52	9.32	9.21	9.01	8.81	8.63	8.40	8.20	7.99	7.71	7.49
504.55	9.43	9.27	9.17	8.96	8.76	8.56	8.32	8.12	7.93	7.71	7.47
543.15	9.71	9.54	9.40	9.25	9.02	8.82	8.60	8.36	8.12	7.92	7.76
584.78	9.59	9.46	9.31	9.14	8.95	8.74	8.50	8.27	8.08	7.82	7.63
629.68	9.55	9.38	9.27	9.15	8.90	8.66	8.46	8.23	8.07	7.82	7.61
678.10	10.26	10.13	9.93	9.80	9.55	9.34	9.10	8.88	8.60	8.38	8.15
730.33	10.51	10.32	10.22	10.02	9.78	9.57	9.37	9.09	8.84	8.60	8.40
786.65	10.47	10.36	10.18	10.00	9.81	9.58	9.36	9.08	8.93	8.64	8.40
847.40	10.62	10.50	10.40	10.20	9.99	9.80	9.54	9.29	9.10	8.83	8.59
912.92	10.89	10.72	10.59	10.37	10.16	9.93	9.73	9.46	9.27	8.97	8.76
983.58	11.10	10.94	10.76	10.59	10.40	10.18	9.91	9.73	9.49	9.22	8.97
1059.78	11.26	11.06	11.00	10.76	10.57	10.34	10.06	9.82	9.59	9.35	9.07
1141.98	11.19	11.06	10.91	10.74	10.51	10.30	10.05	9.82	9.60	9.34	9.08
1230.62	10.90	10.72	10.58	10.40	10.24	10.04	9.77	9.56	9.31	9.09	8.82
1326.23	11.45	11.32	11.23	11.03	10.81	10.65	10.35	10.09	9.80	9.59	9.33
1429.34	12.08	12.03	11.81	11.67	11.43	11.20	10.96	10.67	10.39	10.17	9.90
1540.54	11.96	11.78	11.67	11.50	11.26	11.06	10.82	10.54	10.26	10.01	9.73
1660.48	12.16	12.06	11.89	11.72	11.50	11.26	11.03	10.75	10.52	10.22	9.92
1789.83	12.18	12.06	11.89	11.68	11.54	11.30	11.05	10.78	10.55	10.24	10.01

1929.34	12.46	12.33	12.23	12.02	11.80	11.57	11.31	11.03	10.78	10.47	10.24
2079.80	12.31	12.18	12.09	11.94	11.69	11.50	11.29	10.94	10.71	10.42	10.21
2242.08	12.48	12.33	12.25	12.07	11.85	11.63	11.40	11.09	10.86	10.56	10.31
2417.10	12.58	12.49	12.29	12.15	11.95	11.73	11.49	11.19	10.97	10.67	10.42
2605.85	12.45	12.37	12.20	12.02	11.82	11.62	11.39	11.12	10.82	10.55	10.28
2809.43	12.00	11.86	11.74	11.57	11.38	11.17	10.97	10.69	10.45	10.18	9.92
3028.99	12.50	12.39	12.29	12.09	11.90	11.71	11.46	11.20	10.92	10.66	10.37
3265.79	12.50	12.38	12.25	12.14	11.93	11.71	11.44	11.20	10.89	10.68	10.37
3521.18	12.45	12.36	12.22	12.10	11.92	11.64	11.37	11.15	10.88	10.59	10.31
3796.62	12.13	12.00	11.90	11.70	11.55	11.35	11.12	10.86	10.65	10.32	10.07

Wavelength (nm) / Time (ps)	584.10	585.51	586.92	588.33	589.74	591.15	592.55	593.96	595.37	596.78	598.19
-3.28	0.00	0.02	-0.01	0.06	0.02	0.03	-0.02	0.02	0.03	0.01	0.00
-2.78	-0.04	-0.07	-0.04	-0.06	-0.03	0.02	-0.03	-0.01	-0.06	-0.02	-0.03
-2.28	-0.03	-0.03	-0.01	-0.08	-0.02	-0.07	-0.06	-0.06	-0.03	-0.08	0.00
-1.78	0.12	0.16	0.15	0.13	0.11	0.11	0.16	0.11	0.11	0.13	0.14
-1.28	-0.04	-0.06	-0.06	-0.06	-0.02	-0.07	-0.03	-0.05	-0.07	-0.06	-0.05
-0.78	-0.06	-0.04	-0.04	-0.01	-0.06	-0.04	-0.04	-0.06	-0.05	-0.03	-0.02
-0.28	0.07	0.03	0.04	0.05	0.03	0.02	0.04	0.01	0.03	0.05	0.01
0.22	0.02	0.05	0.02	-0.01	0.02	0.05	0.05	0.04	0.03	0.00	-0.01
0.32	-0.01	-0.03	-0.03	0.02	0.02	-0.01	-0.04	0.00	0.03	0.02	0.00
0.42	-0.03	-0.03	0.00	-0.06	-0.07	-0.04	-0.03	-0.01	-0.02	-0.02	-0.04
0.52	0.02	0.00	-0.01	-0.02	-0.01	0.02	-0.05	-0.01	-0.01	0.01	-0.10
0.62	0.06	0.04	0.01	0.04	0.03	0.01	0.04	0.02	0.04	0.02	0.01
0.72	0.01	0.03	-0.05	-0.02	-0.06	-0.03	-0.04	-0.04	-0.10	-0.06	-0.04
0.77	0.02	0.04	0.02	0.03	0.01	-0.01	-0.02	-0.01	0.00	0.05	-0.03
0.82	-0.01	0.01	0.00	-0.02	0.00	-0.05	-0.01	-0.04	-0.01	-0.01	-0.04
0.87	0.05	0.07	0.03	0.05	0.06	0.06	0.04	0.04	0.04	0.05	0.02
0.92	-0.09	-0.10	-0.11	-0.17	-0.14	-0.12	-0.15	-0.14	-0.14	-0.13	-0.14
0.97	0.06	0.05	0.07	0.05	0.06	0.10	0.10	0.08	0.08	0.10	0.01
1.02	-0.08	-0.06	-0.06	-0.08	-0.03	-0.07	-0.03	-0.06	-0.07	-0.02	-0.03
1.07	0.07	0.04	0.04	0.06	0.05	0.05	0.02	0.03	0.03	0.03	0.03
1.12	-0.11	-0.12	-0.07	-0.12	-0.09	-0.12	-0.06	-0.09	-0.10	-0.04	-0.06
1.17	-0.06	-0.04	-0.02	-0.10	-0.03	-0.04	-0.01	-0.06	-0.04	-0.03	-0.02
1.22	-0.09	-0.02	-0.06	-0.10	-0.09	-0.09	-0.11	-0.07	-0.04	-0.08	-0.08
1.27	0.09	0.07	0.09	0.05	0.08	0.03	0.09	0.07	0.07	0.09	0.06
1.32	0.08	0.09	0.06	0.02	0.03	0.02	0.09	0.07	0.03	0.09	0.04
1.37	0.01	-0.03	-0.03	-0.05	-0.01	0.02	-0.02	-0.03	-0.05	0.01	-0.02
1.42	-0.09	-0.05	-0.08	-0.06	-0.04	-0.04	-0.07	-0.07	-0.08	-0.05	-0.12
1.47	-0.14	-0.14	-0.18	-0.15	-0.15	-0.14	-0.15	-0.14	-0.15	-0.09	-0.12
1.52	0.07	0.05	-0.01	0.00	-0.07	-0.14	-0.14	-0.17	-0.19	-0.21	-0.20
1.57	2.94	2.79	2.60	2.43	2.27	2.08	1.90	1.67	1.58	1.46	1.33
1.62	7.95	7.76	7.56	7.39	7.20	6.96	6.76	6.52	6.22	6.02	5.72
1.67	10.32	10.33	10.23	10.17	10.20	10.12	10.01	9.95	9.81	9.70	9.57
1.72	9.84	9.80	9.79	9.76	9.74	9.72	9.65	9.65	9.63	9.56	9.51
1.77	8.93	8.92	8.85	8.82	8.77	8.73	8.63	8.57	8.50	8.47	8.41
1.82	8.06	8.04	7.98	7.91	7.86	7.77	7.71	7.63	7.52	7.48	7.38
1.87	7.68	7.62	7.55	7.48	7.44	7.30	7.23	7.10	7.02	6.95	6.80

1.92	7.69	7.63	7.57	7.46	7.38	7.28	7.16	7.01	6.91	6.81	6.70
1.97	7.76	7.73	7.63	7.55	7.50	7.40	7.28	7.13	6.94	6.84	6.69
2.02	7.76	7.74	7.62	7.55	7.40	7.35	7.22	7.08	6.87	6.78	6.67
2.07	7.76	7.75	7.63	7.52	7.46	7.28	7.21	7.04	6.92	6.82	6.63
2.12	8.13	8.13	8.02	7.93	7.82	7.72	7.62	7.44	7.28	7.10	6.92
2.17	7.83	7.78	7.69	7.58	7.50	7.37	7.24	7.08	6.87	6.79	6.57
2.22	7.80	7.77	7.68	7.56	7.43	7.32	7.20	7.02	6.87	6.75	6.49
2.27	7.83	7.76	7.65	7.53	7.45	7.29	7.17	7.01	6.86	6.68	6.53
2.32	7.68	7.63	7.55	7.39	7.31	7.21	7.06	6.89	6.71	6.58	6.36
2.37	7.92	7.85	7.76	7.65	7.51	7.43	7.27	7.13	6.96	6.79	6.66
2.42	7.87	7.82	7.69	7.54	7.49	7.34	7.23	7.06	6.89	6.72	6.51
2.47	7.59	7.50	7.39	7.29	7.17	7.07	6.90	6.78	6.55	6.43	6.20
2.52	7.86	7.81	7.74	7.63	7.47	7.33	7.18	7.05	6.83	6.68	6.48
2.57	7.62	7.54	7.49	7.38	7.26	7.16	6.97	6.82	6.64	6.53	6.31
2.62	7.71	7.62	7.53	7.42	7.34	7.21	7.05	6.90	6.73	6.53	6.34
2.67	7.42	7.36	7.23	7.16	7.01	6.92	6.78	6.64	6.44	6.35	6.15
2.72	7.67	7.60	7.48	7.38	7.26	7.11	6.97	6.84	6.67	6.47	6.24
2.77	7.85	7.74	7.64	7.52	7.46	7.28	7.12	6.96	6.75	6.60	6.35
2.82	7.62	7.55	7.48	7.34	7.22	7.10	6.94	6.75	6.57	6.48	6.22
2.87	7.57	7.52	7.45	7.30	7.17	7.03	6.88	6.73	6.57	6.35	6.20
2.92	7.61	7.57	7.48	7.35	7.22	7.02	6.99	6.83	6.61	6.45	6.22
2.97	7.65	7.56	7.48	7.32	7.21	7.09	6.91	6.75	6.57	6.42	6.24
3.02	7.88	7.79	7.69	7.55	7.43	7.25	7.14	6.97	6.77	6.64	6.42
3.07	7.75	7.65	7.56	7.43	7.32	7.14	6.98	6.84	6.61	6.46	6.30
3.12	7.91	7.84	7.74	7.62	7.47	7.28	7.17	7.01	6.75	6.64	6.42
3.17	7.90	7.84	7.73	7.62	7.46	7.34	7.16	7.00	6.79	6.64	6.39
3.22	7.83	7.70	7.60	7.48	7.36	7.23	7.04	6.88	6.65	6.51	6.26
3.27	7.62	7.52	7.43	7.29	7.16	7.06	6.89	6.70	6.51	6.36	6.18
3.32	8.01	7.91	7.82	7.64	7.49	7.36	7.19	7.03	6.84	6.62	6.43
3.37	7.58	7.49	7.36	7.26	7.11	6.98	6.81	6.62	6.46	6.28	6.10
3.42	7.82	7.73	7.57	7.46	7.38	7.19	7.02	6.87	6.67	6.47	6.31
3.47	7.81	7.74	7.64	7.48	7.36	7.22	7.00	6.85	6.65	6.54	6.28
3.52	7.61	7.53	7.42	7.28	7.10	6.97	6.82	6.64	6.49	6.28	6.11
3.57	7.40	7.33	7.20	7.05	6.91	6.78	6.57	6.42	6.28	6.14	5.92
3.62	7.63	7.47	7.36	7.22	7.10	6.95	6.77	6.59	6.40	6.24	6.00
3.67	7.44	7.34	7.24	7.11	6.96	6.82	6.63	6.49	6.28	6.11	5.94
3.72	7.54	7.47	7.35	7.19	7.06	6.92	6.73	6.58	6.39	6.19	5.98
3.92	7.93	7.79	7.68	7.56	7.40	7.24	7.06	6.85	6.60	6.43	6.22
4.12	7.57	7.49	7.31	7.16	7.03	6.90	6.72	6.55	6.36	6.19	6.00
4.32	7.33	7.22	7.08	6.92	6.78	6.63	6.47	6.29	6.09	5.91	5.75
4.52	7.48	7.34	7.18	7.08	6.90	6.71	6.56	6.35	6.19	6.02	5.78
4.72	7.36	7.30	7.14	6.99	6.84	6.65	6.47	6.35	6.08	6.01	5.77
4.92	7.63	7.46	7.39	7.16	7.04	6.86	6.64	6.48	6.29	6.12	5.85
5.12	7.41	7.23	7.09	6.93	6.80	6.62	6.46	6.24	6.07	5.87	5.66
5.32	7.60	7.45	7.32	7.14	7.00	6.79	6.65	6.45	6.26	6.07	5.82
5.52	7.50	7.42	7.23	7.04	6.88	6.75	6.57	6.32	6.13	5.94	5.73
5.72	7.57	7.42	7.26	7.07	6.95	6.73	6.55	6.38	6.21	5.97	5.76
5.92	7.36	7.19	7.06	6.85	6.74	6.54	6.39	6.18	5.99	5.82	5.61
6.12	7.35	7.27	7.12	6.96	6.79	6.62	6.48	6.24	6.08	5.86	5.64
6.32	7.24	7.11	6.94	6.76	6.63	6.44	6.26	6.08	5.91	5.73	5.47

6.52	7.21	7.05	6.91	6.74	6.59	6.44	6.20	6.06	5.86	5.65	5.43
6.72	7.61	7.40	7.23	7.07	6.89	6.74	6.55	6.32	6.11	5.88	5.69
6.92	7.26	7.15	6.92	6.79	6.65	6.41	6.27	6.09	5.89	5.72	5.49
7.12	7.17	7.00	6.85	6.67	6.53	6.36	6.14	5.95	5.78	5.59	5.40
7.32	7.40	7.27	7.11	6.94	6.75	6.60	6.38	6.20	6.02	5.82	5.57
7.52	7.27	7.07	6.92	6.74	6.59	6.41	6.21	6.00	5.83	5.64	5.43
7.72	7.22	7.08	6.93	6.71	6.55	6.40	6.22	6.02	5.83	5.60	5.40
7.92	7.12	6.99	6.78	6.64	6.45	6.26	6.09	5.89	5.72	5.51	5.28
8.12	7.20	7.00	6.90	6.69	6.54	6.35	6.11	5.96	5.76	5.56	5.33
8.32	7.08	6.92	6.80	6.58	6.45	6.28	6.11	5.88	5.65	5.47	5.26
8.52	6.98	6.81	6.63	6.44	6.33	6.11	5.95	5.77	5.61	5.38	5.17
8.72	7.21	7.12	6.90	6.76	6.58	6.41	6.25	6.04	5.77	5.66	5.46
8.92	7.02	6.85	6.66	6.54	6.39	6.18	5.97	5.83	5.60	5.40	5.23
9.12	7.16	7.01	6.78	6.60	6.47	6.29	6.12	5.90	5.71	5.47	5.32
9.32	6.73	6.62	6.44	6.25	6.14	5.91	5.77	5.57	5.39	5.22	4.98
9.52	7.07	6.94	6.76	6.52	6.39	6.24	6.04	5.87	5.65	5.43	5.22
9.72	6.97	6.77	6.62	6.45	6.28	6.10	5.93	5.74	5.56	5.38	5.12
9.92	6.95	6.80	6.62	6.39	6.28	6.10	5.90	5.74	5.52	5.36	5.15
10.12	7.20	6.98	6.80	6.66	6.46	6.25	6.11	5.91	5.70	5.50	5.26
10.32	6.61	6.48	6.32	6.14	5.97	5.81	5.65	5.48	5.23	5.09	4.85
10.52	6.92	6.69	6.53	6.43	6.26	6.06	5.90	5.73	5.51	5.35	5.10
10.72	6.95	6.84	6.66	6.48	6.33	6.12	5.93	5.77	5.52	5.34	5.08
10.92	6.88	6.66	6.49	6.28	6.19	6.00	5.81	5.61	5.45	5.24	4.99
11.12	6.80	6.64	6.46	6.29	6.19	5.98	5.76	5.55	5.39	5.22	4.99
11.32	6.91	6.75	6.59	6.40	6.22	6.03	5.87	5.63	5.49	5.28	5.05
11.52	6.71	6.55	6.38	6.22	6.02	5.86	5.68	5.49	5.32	5.13	4.89
11.72	6.71	6.55	6.37	6.23	6.03	5.87	5.70	5.51	5.33	5.15	4.94
11.92	6.73	6.58	6.42	6.24	6.03	5.84	5.74	5.51	5.27	5.12	4.89
12.12	6.96	6.79	6.62	6.42	6.28	6.09	5.93	5.70	5.51	5.33	5.12
12.32	6.83	6.61	6.49	6.33	6.13	6.02	5.77	5.58	5.35	5.17	4.98
12.52	6.57	6.40	6.27	6.09	5.97	5.78	5.59	5.38	5.19	5.03	4.74
12.72	6.63	6.48	6.28	6.07	5.93	5.74	5.57	5.42	5.20	5.03	4.78
12.92	6.74	6.60	6.38	6.30	6.09	5.88	5.77	5.56	5.36	5.21	4.95
13.12	6.53	6.38	6.24	6.06	5.93	5.73	5.56	5.38	5.19	5.01	4.83
13.32	6.84	6.70	6.50	6.34	6.18	5.99	5.82	5.61	5.41	5.23	5.02
13.52	6.42	6.30	6.10	5.95	5.81	5.66	5.47	5.25	5.09	4.87	4.69
13.72	6.79	6.59	6.44	6.24	6.11	5.90	5.70	5.48	5.28	5.13	4.91
13.92	6.51	6.36	6.18	5.99	5.83	5.67	5.50	5.31	5.10	4.96	4.74
14.00	6.81	6.65	6.46	6.27	6.13	5.92	5.79	5.58	5.35	5.23	4.94
14.08	6.67	6.51	6.35	6.16	6.00	5.89	5.67	5.50	5.28	5.12	4.88
14.17	6.55	6.36	6.20	6.01	5.88	5.73	5.55	5.35	5.14	4.96	4.73
14.27	6.51	6.36	6.21	6.04	5.89	5.73	5.52	5.36	5.18	4.96	4.75
14.38	6.57	6.45	6.28	6.05	5.90	5.75	5.52	5.36	5.15	4.94	4.76
14.49	6.34	6.19	6.04	5.90	5.69	5.55	5.39	5.19	5.00	4.85	4.60
14.61	6.53	6.37	6.20	6.04	5.89	5.75	5.53	5.33	5.15	4.95	4.76
14.75	6.48	6.35	6.14	6.00	5.88	5.65	5.50	5.32	5.16	4.96	4.74
14.89	6.91	6.76	6.50	6.37	6.21	6.04	5.83	5.60	5.43	5.21	4.99
15.05	6.44	6.28	6.11	5.98	5.81	5.61	5.45	5.28	5.06	4.89	4.66
15.21	6.58	6.41	6.21	6.02	5.87	5.70	5.52	5.36	5.14	4.96	4.69
15.39	6.32	6.16	5.98	5.79	5.69	5.51	5.37	5.13	4.97	4.84	4.61

15.59	6.55	6.39	6.19	6.09	5.90	5.65	5.50	5.33	5.16	4.98	4.74
15.80	6.74	6.55	6.36	6.23	6.04	5.88	5.68	5.51	5.30	5.12	4.89
16.02	6.31	6.15	6.00	5.85	5.67	5.51	5.32	5.15	4.98	4.82	4.60
16.27	6.74	6.57	6.38	6.24	6.06	5.84	5.63	5.45	5.24	5.05	4.84
16.53	6.52	6.33	6.17	5.98	5.84	5.61	5.47	5.29	5.07	4.92	4.67
16.81	6.36	6.19	6.01	5.80	5.66	5.50	5.32	5.14	4.95	4.81	4.58
17.12	6.57	6.43	6.25	6.07	5.96	5.73	5.58	5.36	5.15	5.03	4.75
17.45	6.31	6.14	6.01	5.82	5.64	5.51	5.31	5.12	4.96	4.79	4.58
17.81	6.68	6.49	6.32	6.13	5.97	5.83	5.63	5.39	5.24	5.09	4.87
18.19	6.63	6.46	6.28	6.11	5.98	5.76	5.60	5.38	5.18	5.01	4.75
18.60	6.55	6.37	6.23	6.07	5.89	5.72	5.56	5.32	5.13	4.94	4.74
19.05	6.52	6.33	6.16	5.98	5.86	5.69	5.46	5.30	5.09	4.91	4.65
19.53	6.48	6.31	6.15	5.95	5.82	5.64	5.49	5.24	5.06	4.88	4.66
20.05	6.40	6.25	6.07	5.89	5.74	5.57	5.38	5.22	4.98	4.81	4.63
20.61	6.40	6.26	6.07	5.85	5.71	5.58	5.36	5.17	4.96	4.79	4.59
21.22	6.30	6.14	5.99	5.76	5.65	5.47	5.29	5.09	4.88	4.76	4.51
21.87	6.41	6.25	6.07	5.86	5.66	5.57	5.35	5.23	4.98	4.78	4.59
22.57	6.30	6.20	5.98	5.81	5.72	5.49	5.35	5.18	4.95	4.78	4.53
23.33	6.44	6.32	6.11	5.94	5.78	5.61	5.40	5.24	5.00	4.86	4.67
24.15	6.17	6.02	5.84	5.66	5.48	5.32	5.20	4.98	4.78	4.64	4.40
25.03	6.20	6.00	5.90	5.73	5.56	5.39	5.24	4.98	4.85	4.67	4.46
25.98	6.50	6.37	6.18	5.96	5.79	5.62	5.47	5.25	5.06	4.89	4.66
27.01	6.13	5.99	5.79	5.64	5.45	5.30	5.16	4.94	4.79	4.59	4.37
28.11	6.38	6.24	6.07	5.85	5.70	5.54	5.32	5.17	4.97	4.76	4.56
29.30	6.28	6.15	5.97	5.76	5.61	5.40	5.28	5.10	4.91	4.73	4.49
30.59	6.19	5.98	5.88	5.67	5.54	5.39	5.20	5.01	4.81	4.61	4.41
31.98	6.15	6.02	5.84	5.70	5.47	5.35	5.14	5.00	4.78	4.60	4.36
33.47	6.16	6.00	5.84	5.69	5.52	5.32	5.13	4.98	4.82	4.60	4.39
35.09	6.08	5.92	5.81	5.61	5.43	5.27	5.04	4.93	4.75	4.53	4.36
36.83	6.27	6.13	5.96	5.80	5.61	5.48	5.29	5.08	4.91	4.70	4.45
38.71	6.06	5.89	5.72	5.57	5.38	5.23	5.05	4.86	4.71	4.49	4.26
40.73	6.07	5.87	5.73	5.53	5.35	5.20	5.05	4.89	4.66	4.51	4.30
42.92	6.04	5.87	5.68	5.48	5.33	5.16	4.99	4.79	4.60	4.45	4.25
45.27	5.91	5.76	5.62	5.41	5.24	5.09	4.91	4.75	4.54	4.39	4.17
47.81	6.12	5.95	5.77	5.57	5.44	5.26	5.06	4.88	4.73	4.53	4.30
50.55	5.80	5.66	5.51	5.34	5.18	5.07	4.88	4.69	4.52	4.35	4.14
53.51	6.30	6.09	5.91	5.75	5.54	5.40	5.21	5.03	4.85	4.70	4.43
56.69	6.09	5.95	5.70	5.57	5.38	5.20	5.07	4.87	4.66	4.54	4.34
60.13	6.08	5.93	5.75	5.56	5.42	5.20	5.05	4.83	4.68	4.47	4.28
63.84	6.19	5.99	5.84	5.61	5.48	5.32	5.12	4.98	4.75	4.60	4.36
67.83	6.09	5.92	5.76	5.58	5.39	5.21	5.12	4.85	4.69	4.47	4.29
72.15	6.14	5.99	5.81	5.61	5.46	5.28	5.10	4.89	4.70	4.54	4.35
76.80	5.98	5.84	5.65	5.46	5.30	5.11	4.98	4.75	4.57	4.41	4.23
81.81	6.23	6.06	5.85	5.65	5.51	5.29	5.11	4.92	4.73	4.52	4.35
87.22	5.98	5.86	5.69	5.44	5.33	5.14	4.98	4.75	4.57	4.45	4.18
93.06	6.08	5.94	5.76	5.59	5.39	5.27	5.06	4.84	4.66	4.52	4.30
99.35	6.31	6.12	5.94	5.74	5.58	5.40	5.20	5.06	4.79	4.61	4.38
106.13	6.27	6.11	5.94	5.73	5.51	5.34	5.18	5.01	4.79	4.59	4.37
113.45	6.39	6.19	5.99	5.82	5.65	5.45	5.32	5.05	4.87	4.71	4.49
121.34	6.24	6.10	5.90	5.68	5.52	5.36	5.14	4.95	4.72	4.58	4.36

129.86	6.20	6.03	5.81	5.64	5.49	5.31	5.12	4.89	4.73	4.53	4.30
139.04	6.16	5.99	5.83	5.66	5.42	5.28	5.08	4.98	4.72	4.55	4.30
148.94	6.19	6.04	5.87	5.69	5.51	5.32	5.16	4.93	4.71	4.57	4.37
159.62	6.45	6.22	6.04	5.85	5.72	5.48	5.29	5.13	4.89	4.68	4.47
171.14	6.35	6.19	6.00	5.81	5.60	5.45	5.27	5.07	4.87	4.65	4.46
183.56	6.15	5.94	5.79	5.61	5.46	5.22	5.08	4.88	4.65	4.47	4.29
196.96	6.64	6.46	6.28	6.01	5.87	5.67	5.43	5.26	5.05	4.86	4.63
211.41	6.48	6.28	6.08	5.89	5.71	5.47	5.33	5.08	4.94	4.74	4.51
226.99	6.63	6.42	6.23	6.00	5.86	5.64	5.46	5.22	5.02	4.84	4.61
243.80	6.32	6.17	5.99	5.76	5.63	5.42	5.27	5.05	4.84	4.66	4.47
261.93	6.71	6.49	6.29	6.08	5.90	5.68	5.47	5.31	5.07	4.92	4.64
281.48	6.86	6.66	6.49	6.19	6.02	5.81	5.64	5.43	5.18	5.04	4.73
302.56	6.74	6.56	6.33	6.10	5.91	5.74	5.53	5.34	5.10	4.92	4.73
325.30	6.93	6.75	6.49	6.29	6.11	5.89	5.69	5.50	5.27	5.11	4.92
349.83	6.83	6.65	6.47	6.22	6.05	5.85	5.64	5.42	5.19	5.07	4.83
376.28	7.13	6.94	6.74	6.55	6.33	6.13	5.89	5.69	5.45	5.28	5.04
404.81	7.21	6.96	6.78	6.55	6.39	6.14	5.91	5.76	5.55	5.35	5.12
435.58	7.13	6.95	6.72	6.53	6.33	6.15	5.93	5.71	5.49	5.29	5.10
468.76	7.29	7.04	6.86	6.63	6.38	6.24	6.01	5.84	5.60	5.44	5.20
504.55	7.26	7.11	6.83	6.65	6.43	6.26	6.02	5.83	5.60	5.41	5.24
543.15	7.54	7.31	7.07	6.80	6.68	6.43	6.26	6.03	5.82	5.63	5.39
584.78	7.44	7.23	6.96	6.72	6.51	6.35	6.12	5.91	5.72	5.57	5.26
629.68	7.38	7.19	6.93	6.70	6.52	6.31	6.11	5.90	5.72	5.51	5.32
678.10	7.95	7.76	7.48	7.25	7.02	6.81	6.60	6.39	6.17	5.92	5.73
730.33	8.11	7.96	7.62	7.46	7.20	7.00	6.78	6.52	6.32	6.11	5.90
786.65	8.19	7.95	7.75	7.49	7.29	7.06	6.84	6.61	6.37	6.22	5.95
847.40	8.39	8.17	7.92	7.66	7.50	7.23	6.98	6.81	6.55	6.37	6.11
912.92	8.52	8.33	8.08	7.78	7.56	7.35	7.12	6.93	6.61	6.45	6.26
983.58	8.75	8.48	8.27	7.98	7.71	7.54	7.35	7.05	6.81	6.64	6.38
1059.78	8.88	8.63	8.35	8.07	7.89	7.64	7.40	7.19	6.95	6.72	6.52
1141.98	8.81	8.61	8.36	8.11	7.89	7.66	7.40	7.18	6.98	6.78	6.55
1230.62	8.58	8.37	8.06	7.84	7.67	7.40	7.19	7.02	6.75	6.60	6.37
1326.23	9.10	8.82	8.60	8.33	8.09	7.87	7.66	7.39	7.16	6.97	6.78
1429.34	9.62	9.36	9.10	8.80	8.60	8.34	8.11	7.85	7.63	7.42	7.20
1540.54	9.50	9.24	9.00	8.67	8.49	8.21	7.97	7.70	7.51	7.30	7.08
1660.48	9.67	9.43	9.20	8.88	8.65	8.42	8.16	8.01	7.71	7.51	7.30
1789.83	9.75	9.47	9.20	8.93	8.69	8.44	8.21	7.98	7.70	7.54	7.33
1929.34	9.94	9.70	9.38	9.11	8.84	8.62	8.36	8.12	7.95	7.70	7.49
2079.80	9.90	9.62	9.34	9.04	8.84	8.64	8.34	8.11	7.89	7.71	7.48
2242.08	10.05	9.77	9.48	9.21	8.94	8.73	8.50	8.23	8.06	7.86	7.61
2417.10	10.11	9.85	9.58	9.31	9.08	8.81	8.56	8.37	8.12	7.94	7.64
2605.85	10.02	9.76	9.48	9.18	8.99	8.72	8.51	8.26	8.03	7.87	7.59
2809.43	9.68	9.38	9.16	8.93	8.66	8.45	8.20	7.97	7.79	7.60	7.37
3028.99	10.13	9.81	9.55	9.33	9.04	8.80	8.60	8.35	8.08	7.89	7.69
3265.79	10.08	9.81	9.55	9.27	9.03	8.77	8.54	8.31	8.10	7.92	7.70
3521.18	10.06	9.82	9.56	9.28	9.11	8.80	8.57	8.35	8.11	7.93	7.72
3796.62	9.84	9.60	9.34	9.05	8.81	8.62	8.39	8.16	7.92	7.75	7.53

Time (ps) \ Wavelength (nm)	599.60	601.00	602.41	603.82	605.23	606.64	608.05	609.46	610.86	612.27	613.68
-3.28	-0.02	-0.03	-0.02	0.01	0.01	-0.03	0.02	0.02	0.00	0.04	0.02
-2.78	-0.05	-0.01	-0.02	-0.01	-0.01	0.02	0.01	-0.04	0.00	0.02	0.04
-2.28	-0.01	0.00	-0.01	-0.03	-0.06	-0.04	0.00	-0.03	0.00	-0.03	-0.01
-1.78	0.12	0.12	0.09	0.08	0.06	0.09	0.06	0.08	0.06	0.01	0.03
-1.28	-0.05	-0.04	-0.05	-0.06	-0.03	-0.03	-0.03	-0.04	-0.02	-0.02	-0.02
-0.78	-0.06	-0.04	-0.03	-0.02	0.00	-0.01	0.01	-0.01	0.01	0.05	0.04
-0.28	0.00	-0.01	0.01	0.01	0.02	-0.02	-0.02	0.01	-0.02	-0.02	-0.05
0.22	0.01	0.01	0.01	-0.01	-0.01	0.02	-0.01	0.02	-0.02	-0.04	-0.02
0.32	0.03	0.03	0.01	0.00	0.03	0.00	0.01	0.00	0.00	-0.01	0.01
0.42	0.02	-0.03	0.01	0.01	-0.01	0.00	-0.04	-0.01	0.00	0.01	-0.04
0.52	-0.02	-0.01	-0.05	-0.03	0.00	0.01	-0.05	-0.03	0.02	-0.02	-0.03
0.62	0.01	-0.03	0.03	0.04	-0.02	-0.01	-0.03	-0.07	-0.02	0.00	-0.06
0.72	-0.03	-0.05	0.01	-0.07	-0.05	-0.05	-0.08	-0.05	-0.08	-0.07	-0.06
0.77	-0.01	0.00	0.01	0.00	0.01	-0.03	-0.02	-0.10	-0.04	-0.06	-0.05
0.82	-0.07	0.01	0.02	0.01	0.02	0.01	-0.03	-0.02	0.02	-0.02	-0.03
0.87	0.04	0.03	0.03	0.04	0.06	0.01	0.03	0.04	0.03	0.01	0.01
0.92	-0.12	-0.12	-0.14	-0.13	-0.09	-0.13	-0.11	-0.12	-0.12	-0.09	-0.09
0.97	0.03	0.02	0.05	0.08	0.06	0.02	0.06	0.00	0.02	0.06	0.04
1.02	-0.01	-0.07	-0.04	-0.03	-0.04	-0.01	0.00	-0.02	0.03	0.00	0.01
1.07	0.03	0.05	0.05	0.02	0.04	-0.03	0.01	-0.03	0.04	-0.02	-0.03
1.12	-0.10	-0.08	-0.07	-0.06	-0.04	0.00	-0.01	-0.04	-0.05	-0.01	-0.02
1.17	-0.03	-0.03	-0.03	-0.07	-0.01	-0.03	-0.02	-0.03	-0.02	-0.02	0.00
1.22	-0.07	-0.10	-0.08	-0.05	-0.10	-0.08	-0.05	-0.11	-0.06	-0.03	-0.07
1.27	0.02	0.10	0.10	0.03	0.05	0.04	0.04	0.02	0.03	0.05	0.00
1.32	0.06	0.04	0.05	0.07	0.10	0.07	0.07	0.01	0.05	0.06	0.00
1.37	-0.02	0.00	-0.01	-0.04	-0.01	-0.02	-0.03	-0.04	-0.03	-0.04	-0.02
1.42	-0.06	-0.08	-0.06	-0.05	-0.07	-0.05	-0.03	-0.05	-0.01	-0.05	-0.03
1.47	-0.14	-0.11	-0.06	-0.05	-0.05	-0.06	-0.03	-0.06	-0.03	-0.03	-0.05
1.52	-0.19	-0.16	-0.14	-0.12	-0.12	-0.10	-0.05	-0.06	-0.03	-0.07	-0.07
1.57	1.18	1.08	1.02	0.93	0.89	0.80	0.76	0.72	0.68	0.62	0.53
1.62	5.46	5.23	4.99	4.78	4.64	4.43	4.26	4.08	3.96	3.77	3.63
1.67	9.44	9.32	9.23	9.09	8.96	8.84	8.76	8.64	8.60	8.55	8.48
1.72	9.42	9.43	9.47	9.41	9.39	9.40	9.37	9.32	9.39	9.37	9.37
1.77	8.33	8.30	8.29	8.25	8.23	8.23	8.21	8.24	8.23	8.23	8.25
1.82	7.28	7.25	7.19	7.13	7.10	7.08	7.02	7.02	7.03	7.05	7.03
1.87	6.72	6.64	6.56	6.45	6.42	6.32	6.33	6.24	6.26	6.28	6.26
1.92	6.53	6.47	6.36	6.28	6.20	6.09	6.07	6.01	6.01	5.94	5.91
1.97	6.56	6.46	6.35	6.26	6.12	6.05	5.98	5.93	5.89	5.85	5.81
2.02	6.53	6.39	6.25	6.18	6.06	5.94	5.91	5.75	5.76	5.71	5.63
2.07	6.50	6.40	6.27	6.11	6.04	5.92	5.85	5.74	5.76	5.66	5.59
2.12	6.76	6.60	6.52	6.37	6.21	6.12	5.97	5.88	5.82	5.77	5.66
2.17	6.46	6.27	6.13	6.00	5.90	5.76	5.67	5.54	5.42	5.37	5.28
2.22	6.39	6.22	6.08	5.93	5.87	5.64	5.57	5.45	5.37	5.30	5.21
2.27	6.35	6.26	6.06	5.92	5.79	5.62	5.50	5.43	5.33	5.25	5.21
2.32	6.21	6.09	5.94	5.79	5.63	5.51	5.45	5.26	5.20	5.07	5.07
2.37	6.43	6.28	6.13	5.97	5.84	5.70	5.58	5.43	5.37	5.24	5.18
2.42	6.38	6.16	6.03	5.90	5.71	5.58	5.46	5.28	5.21	5.09	5.01

2.47	6.03	5.94	5.75	5.60	5.43	5.28	5.17	5.00	4.96	4.81	4.73
2.52	6.29	6.11	6.03	5.83	5.66	5.52	5.38	5.21	5.11	5.03	4.90
2.57	6.17	5.98	5.83	5.67	5.50	5.37	5.20	5.06	4.99	4.85	4.76
2.62	6.15	6.00	5.81	5.66	5.45	5.31	5.17	5.00	4.87	4.81	4.70
2.67	6.00	5.82	5.63	5.49	5.34	5.16	5.05	4.89	4.82	4.74	4.61
2.72	6.10	5.92	5.74	5.60	5.39	5.23	5.10	4.95	4.83	4.70	4.58
2.77	6.19	6.05	5.87	5.69	5.52	5.29	5.19	4.99	4.86	4.74	4.66
2.82	6.02	5.93	5.74	5.54	5.39	5.17	5.04	4.92	4.80	4.67	4.57
2.87	6.05	5.87	5.64	5.45	5.31	5.16	5.01	4.85	4.76	4.62	4.48
2.92	6.06	5.88	5.73	5.51	5.32	5.15	5.06	4.85	4.71	4.60	4.46
2.97	6.03	5.80	5.67	5.48	5.29	5.16	5.02	4.88	4.77	4.64	4.50
3.02	6.21	6.01	5.81	5.64	5.53	5.26	5.13	4.93	4.81	4.69	4.54
3.07	6.07	5.92	5.73	5.54	5.38	5.21	4.99	4.83	4.72	4.58	4.44
3.12	6.22	6.06	5.84	5.63	5.43	5.31	5.10	4.92	4.81	4.65	4.52
3.17	6.23	6.04	5.83	5.66	5.50	5.31	5.13	4.97	4.79	4.71	4.54
3.22	6.07	5.92	5.73	5.51	5.32	5.17	5.01	4.84	4.69	4.56	4.41
3.27	5.95	5.77	5.58	5.39	5.20	4.98	4.86	4.69	4.54	4.41	4.25
3.32	6.21	5.98	5.80	5.62	5.41	5.20	5.03	4.86	4.71	4.56	4.42
3.37	5.89	5.71	5.56	5.33	5.17	5.01	4.81	4.66	4.54	4.41	4.27
3.42	6.06	5.87	5.71	5.53	5.33	5.12	4.94	4.76	4.64	4.49	4.34
3.47	6.09	5.87	5.71	5.50	5.30	5.11	4.94	4.78	4.63	4.51	4.33
3.52	5.91	5.70	5.52	5.26	5.14	4.94	4.81	4.60	4.43	4.34	4.17
3.57	5.74	5.52	5.38	5.16	4.97	4.81	4.66	4.47	4.37	4.21	4.12
3.62	5.82	5.60	5.47	5.22	5.05	4.87	4.70	4.52	4.39	4.24	4.06
3.67	5.70	5.52	5.33	5.16	4.97	4.82	4.67	4.49	4.32	4.22	4.06
3.72	5.79	5.62	5.43	5.25	5.03	4.87	4.72	4.47	4.36	4.22	4.06
3.92	6.01	5.83	5.60	5.39	5.22	5.03	4.83	4.62	4.52	4.35	4.20
4.12	5.78	5.55	5.43	5.20	5.00	4.85	4.65	4.46	4.32	4.19	4.02
4.32	5.51	5.33	5.15	4.90	4.76	4.53	4.35	4.22	4.05	3.90	3.81
4.52	5.65	5.40	5.24	5.00	4.81	4.66	4.45	4.28	4.12	3.99	3.87
4.72	5.55	5.36	5.13	4.98	4.79	4.63	4.40	4.26	4.10	3.92	3.83
4.92	5.65	5.49	5.26	5.09	4.91	4.68	4.51	4.32	4.17	4.01	3.83
5.12	5.49	5.25	5.05	4.89	4.64	4.48	4.29	4.10	3.91	3.82	3.68
5.32	5.61	5.47	5.22	5.03	4.80	4.59	4.41	4.24	4.10	3.91	3.72
5.52	5.53	5.29	5.12	4.92	4.70	4.47	4.27	4.09	3.93	3.74	3.61
5.72	5.54	5.30	5.14	4.87	4.73	4.51	4.28	4.10	3.92	3.74	3.60
5.92	5.35	5.12	4.95	4.80	4.54	4.40	4.22	3.99	3.89	3.69	3.54
6.12	5.43	5.25	4.98	4.85	4.61	4.41	4.28	4.04	3.90	3.76	3.57
6.32	5.32	5.13	4.92	4.73	4.50	4.32	4.05	3.95	3.82	3.63	3.50
6.52	5.25	5.06	4.82	4.61	4.42	4.21	4.09	3.82	3.69	3.55	3.35
6.72	5.47	5.28	5.02	4.83	4.64	4.44	4.23	3.99	3.87	3.70	3.52
6.92	5.25	5.08	4.87	4.65	4.51	4.26	4.09	3.89	3.76	3.57	3.42
7.12	5.15	4.99	4.82	4.56	4.37	4.18	4.01	3.82	3.65	3.50	3.35
7.32	5.35	5.15	4.92	4.75	4.56	4.32	4.10	3.93	3.73	3.57	3.43
7.52	5.21	5.02	4.81	4.62	4.40	4.20	4.02	3.80	3.63	3.49	3.35
7.72	5.22	5.06	4.79	4.60	4.39	4.17	4.01	3.73	3.61	3.51	3.30
7.92	5.11	4.90	4.69	4.44	4.25	4.09	3.85	3.70	3.52	3.36	3.18
8.12	5.15	4.92	4.70	4.52	4.28	4.08	3.91	3.74	3.54	3.38	3.23
8.32	5.05	4.84	4.68	4.44	4.25	4.02	3.88	3.65	3.46	3.37	3.16
8.52	5.01	4.81	4.62	4.39	4.22	4.00	3.84	3.70	3.50	3.34	3.21

8.72	5.27	5.02	4.85	4.62	4.44	4.26	4.03	3.85	3.68	3.57	3.38
8.92	4.98	4.81	4.62	4.41	4.22	4.02	3.81	3.64	3.53	3.38	3.21
9.12	5.06	4.91	4.68	4.45	4.26	4.07	3.88	3.64	3.51	3.33	3.17
9.32	4.79	4.56	4.38	4.22	4.03	3.82	3.64	3.45	3.32	3.18	3.02
9.52	5.01	4.80	4.59	4.38	4.22	3.99	3.82	3.63	3.49	3.37	3.16
9.72	4.89	4.71	4.51	4.35	4.12	3.89	3.73	3.53	3.36	3.26	3.06
9.92	4.92	4.75	4.52	4.30	4.13	3.92	3.74	3.50	3.36	3.20	3.05
10.12	5.08	4.85	4.65	4.44	4.21	4.01	3.82	3.65	3.50	3.31	3.19
10.32	4.67	4.48	4.26	4.10	3.90	3.69	3.51	3.37	3.20	3.07	2.89
10.52	4.87	4.71	4.50	4.30	4.14	3.98	3.72	3.54	3.42	3.28	3.05
10.72	4.92	4.67	4.49	4.32	4.09	3.85	3.68	3.52	3.32	3.15	3.02
10.92	4.84	4.60	4.41	4.22	4.03	3.79	3.63	3.46	3.31	3.14	2.99
11.12	4.78	4.56	4.41	4.20	4.02	3.81	3.62	3.45	3.32	3.10	2.98
11.32	4.83	4.65	4.40	4.22	4.00	3.82	3.59	3.39	3.25	3.09	2.94
11.52	4.70	4.53	4.32	4.10	3.87	3.71	3.55	3.35	3.19	3.06	2.92
11.72	4.72	4.54	4.36	4.15	3.97	3.73	3.59	3.36	3.22	3.04	2.91
11.92	4.68	4.50	4.29	4.11	3.91	3.75	3.59	3.40	3.24	3.05	2.96
12.12	4.95	4.72	4.51	4.29	4.07	3.92	3.75	3.55	3.40	3.19	3.07
12.32	4.75	4.55	4.35	4.11	3.95	3.74	3.61	3.39	3.21	3.08	2.92
12.52	4.55	4.42	4.21	3.99	3.77	3.60	3.41	3.26	3.08	2.90	2.78
12.72	4.56	4.37	4.18	3.98	3.76	3.56	3.40	3.21	3.06	2.90	2.77
12.92	4.73	4.56	4.35	4.16	3.92	3.73	3.59	3.32	3.20	3.06	2.88
13.12	4.60	4.38	4.25	4.02	3.85	3.65	3.45	3.27	3.13	2.99	2.84
13.32	4.76	4.62	4.36	4.16	3.97	3.84	3.65	3.46	3.31	3.15	3.02
13.52	4.51	4.30	4.12	3.94	3.74	3.58	3.39	3.18	3.09	2.91	2.77
13.72	4.67	4.50	4.29	4.08	3.85	3.67	3.47	3.29	3.10	2.95	2.76
13.92	4.55	4.35	4.18	3.93	3.76	3.53	3.41	3.20	3.03	2.90	2.79
14.00	4.79	4.56	4.35	4.14	3.91	3.73	3.56	3.32	3.17	2.99	2.86
14.08	4.71	4.52	4.31	4.06	3.89	3.71	3.53	3.34	3.19	3.03	2.91
14.17	4.60	4.34	4.13	3.97	3.74	3.61	3.43	3.23	3.06	2.92	2.78
14.27	4.52	4.36	4.16	3.97	3.74	3.58	3.40	3.25	3.10	2.91	2.75
14.38	4.54	4.34	4.15	3.95	3.80	3.60	3.39	3.19	3.08	2.94	2.77
14.49	4.42	4.21	4.07	3.83	3.61	3.47	3.26	3.09	2.99	2.78	2.68
14.61	4.51	4.35	4.20	3.98	3.73	3.58	3.38	3.22	3.05	2.93	2.77
14.75	4.53	4.37	4.18	4.00	3.80	3.60	3.43	3.24	3.13	2.93	2.81
14.89	4.78	4.56	4.38	4.12	3.95	3.74	3.57	3.37	3.20	3.05	2.84
15.05	4.45	4.24	4.09	3.88	3.71	3.50	3.31	3.15	2.93	2.77	2.68
15.21	4.52	4.33	4.10	3.92	3.71	3.52	3.36	3.15	3.04	2.83	2.66
15.39	4.44	4.19	4.08	3.81	3.64	3.39	3.28	3.10	2.93	2.82	2.65
15.59	4.55	4.36	4.16	3.96	3.75	3.59	3.40	3.23	3.04	2.96	2.83
15.80	4.67	4.48	4.29	4.06	3.90	3.74	3.52	3.28	3.13	2.98	2.82
16.02	4.38	4.21	4.06	3.83	3.66	3.45	3.28	3.10	2.96	2.85	2.66
16.27	4.56	4.43	4.23	3.98	3.79	3.59	3.44	3.26	3.09	2.93	2.76
16.53	4.48	4.25	4.08	3.86	3.71	3.51	3.34	3.16	3.00	2.88	2.73
16.81	4.39	4.22	4.00	3.81	3.65	3.47	3.31	3.12	2.99	2.80	2.69
17.12	4.58	4.34	4.21	3.97	3.79	3.55	3.39	3.23	3.05	2.87	2.70
17.45	4.37	4.20	4.02	3.86	3.64	3.47	3.31	3.09	2.96	2.80	2.67
17.81	4.65	4.45	4.28	4.04	3.85	3.65	3.45	3.26	3.16	3.01	2.81
18.19	4.54	4.34	4.17	3.91	3.69	3.51	3.36	3.16	2.99	2.84	2.68
18.60	4.55	4.36	4.15	3.96	3.74	3.57	3.40	3.17	3.04	2.88	2.77

19.05	4.51	4.30	4.11	3.89	3.69	3.53	3.34	3.14	3.01	2.84	2.68
19.53	4.50	4.22	4.08	3.87	3.71	3.49	3.32	3.14	2.99	2.83	2.72
20.05	4.37	4.21	4.04	3.84	3.63	3.41	3.27	3.10	2.93	2.79	2.64
20.61	4.42	4.24	3.99	3.80	3.59	3.38	3.23	3.02	2.87	2.72	2.56
21.22	4.34	4.11	3.94	3.71	3.55	3.34	3.15	2.99	2.83	2.69	2.53
21.87	4.38	4.17	4.00	3.79	3.61	3.43	3.19	3.06	2.92	2.79	2.63
22.57	4.39	4.14	3.98	3.81	3.56	3.35	3.20	2.99	2.87	2.70	2.57
23.33	4.46	4.25	4.03	3.82	3.64	3.49	3.29	3.12	2.98	2.82	2.67
24.15	4.19	4.01	3.82	3.62	3.42	3.25	3.05	2.88	2.75	2.62	2.45
25.03	4.27	4.01	3.91	3.69	3.50	3.35	3.14	2.99	2.84	2.66	2.53
25.98	4.43	4.26	4.04	3.83	3.64	3.44	3.25	3.10	2.93	2.79	2.58
27.01	4.21	4.07	3.86	3.63	3.49	3.27	3.13	2.92	2.83	2.65	2.49
28.11	4.39	4.15	3.97	3.73	3.55	3.34	3.18	2.98	2.82	2.66	2.56
29.30	4.28	4.10	3.90	3.75	3.50	3.32	3.18	2.95	2.83	2.70	2.52
30.59	4.20	3.98	3.83	3.64	3.42	3.24	3.06	2.92	2.74	2.59	2.45
31.98	4.15	4.00	3.79	3.62	3.42	3.25	3.11	2.92	2.80	2.60	2.54
33.47	4.21	4.03	3.79	3.59	3.46	3.26	3.06	2.89	2.76	2.60	2.47
35.09	4.17	3.99	3.79	3.59	3.38	3.19	3.04	2.87	2.74	2.58	2.45
36.83	4.27	4.10	3.88	3.71	3.54	3.35	3.17	2.97	2.83	2.67	2.54
38.71	4.09	3.94	3.70	3.50	3.31	3.13	2.97	2.80	2.58	2.45	2.33
40.73	4.09	3.89	3.72	3.50	3.34	3.18	2.98	2.82	2.66	2.52	2.39
42.92	4.08	3.87	3.68	3.52	3.32	3.15	2.94	2.76	2.64	2.49	2.33
45.27	4.01	3.81	3.65	3.47	3.29	3.05	2.95	2.75	2.62	2.48	2.34
47.81	4.10	3.93	3.74	3.51	3.30	3.16	2.99	2.83	2.65	2.53	2.35
50.55	3.97	3.80	3.60	3.44	3.29	3.11	2.94	2.74	2.65	2.47	2.33
53.51	4.24	4.04	3.84	3.65	3.43	3.23	3.09	2.89	2.73	2.58	2.42
56.69	4.11	3.95	3.76	3.58	3.37	3.22	3.03	2.87	2.74	2.57	2.45
60.13	4.06	3.88	3.69	3.45	3.28	3.13	2.92	2.74	2.63	2.49	2.34
63.84	4.14	4.00	3.76	3.58	3.39	3.19	3.02	2.81	2.69	2.48	2.32
67.83	4.10	3.93	3.72	3.48	3.31	3.16	2.98	2.74	2.69	2.47	2.37
72.15	4.15	3.98	3.77	3.57	3.41	3.24	3.04	2.83	2.70	2.61	2.42
76.80	3.98	3.87	3.68	3.45	3.26	3.07	2.95	2.72	2.60	2.48	2.33
81.81	4.12	3.91	3.77	3.55	3.37	3.19	3.02	2.83	2.63	2.48	2.32
87.22	4.00	3.82	3.66	3.43	3.24	3.07	2.88	2.71	2.54	2.41	2.26
93.06	4.10	3.94	3.72	3.51	3.36	3.16	2.96	2.78	2.66	2.48	2.41
99.35	4.17	3.99	3.82	3.62	3.41	3.24	3.09	2.85	2.73	2.60	2.41
106.13	4.18	3.99	3.80	3.55	3.36	3.20	3.01	2.84	2.64	2.49	2.37
113.45	4.25	4.06	3.90	3.67	3.43	3.25	3.13	2.93	2.76	2.55	2.38
121.34	4.13	3.99	3.79	3.61	3.42	3.26	3.04	2.88	2.74	2.60	2.46
129.86	4.11	3.93	3.74	3.53	3.35	3.13	2.99	2.77	2.65	2.49	2.38
139.04	4.14	3.92	3.75	3.55	3.33	3.20	3.00	2.77	2.63	2.51	2.36
148.94	4.16	3.99	3.81	3.60	3.40	3.23	3.11	2.94	2.78	2.61	2.53
159.62	4.30	4.08	3.87	3.64	3.51	3.26	3.09	2.93	2.81	2.61	2.46
171.14	4.27	4.08	3.87	3.68	3.50	3.35	3.16	2.94	2.85	2.65	2.52
183.56	4.06	3.84	3.68	3.51	3.29	3.11	2.98	2.78	2.63	2.52	2.42
196.96	4.45	4.19	4.04	3.84	3.67	3.48	3.30	3.15	2.95	2.78	2.62
211.41	4.29	4.14	3.90	3.72	3.55	3.37	3.13	3.02	2.84	2.70	2.56
226.99	4.37	4.23	3.99	3.80	3.59	3.45	3.21	3.07	2.92	2.74	2.63
243.80	4.27	4.10	3.92	3.73	3.55	3.37	3.22	3.03	2.89	2.76	2.60
261.93	4.47	4.29	4.08	3.85	3.68	3.51	3.35	3.11	2.99	2.85	2.70

281.48	4.55	4.39	4.17	4.00	3.81	3.60	3.42	3.23	3.08	2.91	2.78
302.56	4.52	4.32	4.17	3.95	3.74	3.54	3.37	3.20	3.05	2.90	2.77
325.30	4.66	4.47	4.31	4.06	3.89	3.68	3.48	3.31	3.16	3.02	2.86
349.83	4.64	4.43	4.19	4.04	3.82	3.66	3.55	3.32	3.16	3.00	2.83
376.28	4.85	4.67	4.43	4.28	4.07	3.89	3.71	3.50	3.36	3.20	3.07
404.81	4.94	4.68	4.50	4.29	4.10	3.89	3.72	3.58	3.39	3.21	3.08
435.58	4.83	4.64	4.47	4.30	4.09	3.88	3.70	3.55	3.36	3.23	3.10
468.76	5.00	4.83	4.64	4.45	4.23	4.05	3.91	3.69	3.53	3.33	3.22
504.55	5.02	4.79	4.64	4.43	4.28	4.07	3.92	3.77	3.59	3.48	3.31
543.15	5.17	4.98	4.82	4.60	4.45	4.25	4.05	3.91	3.74	3.61	3.46
584.78	5.08	4.94	4.74	4.58	4.39	4.20	4.02	3.82	3.71	3.58	3.45
629.68	5.10	4.92	4.77	4.56	4.38	4.21	4.04	3.87	3.77	3.61	3.48
678.10	5.49	5.34	5.08	4.93	4.70	4.52	4.36	4.20	4.06	3.89	3.78
730.33	5.69	5.52	5.34	5.09	4.91	4.72	4.54	4.35	4.26	4.10	3.93
786.65	5.79	5.59	5.40	5.21	4.98	4.84	4.66	4.55	4.34	4.20	4.01
847.40	5.92	5.75	5.54	5.36	5.16	4.97	4.85	4.65	4.54	4.40	4.23
912.92	6.04	5.86	5.64	5.48	5.30	5.09	4.94	4.79	4.62	4.50	4.37
983.58	6.23	6.01	5.82	5.66	5.47	5.31	5.13	4.98	4.85	4.66	4.50
1059.78	6.32	6.12	5.94	5.72	5.54	5.39	5.22	5.05	4.90	4.73	4.58
1141.98	6.32	6.14	5.92	5.78	5.57	5.46	5.28	5.07	4.96	4.80	4.72
1230.62	6.19	6.00	5.84	5.67	5.48	5.32	5.17	4.99	4.84	4.75	4.58
1326.23	6.57	6.36	6.17	6.02	5.83	5.68	5.47	5.34	5.17	5.06	4.89
1429.34	7.00	6.78	6.60	6.42	6.25	6.07	5.93	5.77	5.59	5.47	5.38
1540.54	6.86	6.69	6.46	6.31	6.13	5.98	5.75	5.62	5.52	5.37	5.22
1660.48	7.07	6.90	6.69	6.50	6.34	6.20	6.03	5.88	5.71	5.58	5.48
1789.83	7.10	6.91	6.77	6.55	6.42	6.25	6.12	5.93	5.81	5.71	5.56
1929.34	7.27	7.07	6.92	6.74	6.58	6.37	6.23	6.02	5.92	5.76	5.66
2079.80	7.27	7.08	6.95	6.74	6.60	6.41	6.29	6.08	5.96	5.85	5.71
2242.08	7.41	7.24	7.02	6.84	6.67	6.58	6.44	6.31	6.12	6.00	5.89
2417.10	7.51	7.33	7.13	7.00	6.80	6.67	6.52	6.36	6.25	6.15	5.98
2605.85	7.43	7.24	7.08	6.90	6.76	6.61	6.47	6.31	6.20	6.11	5.90
2809.43	7.19	7.04	6.83	6.71	6.57	6.45	6.30	6.17	6.06	5.96	5.84
3028.99	7.49	7.34	7.17	6.98	6.87	6.71	6.60	6.44	6.35	6.23	6.11
3265.79	7.48	7.32	7.17	7.02	6.91	6.73	6.57	6.44	6.34	6.22	6.08
3521.18	7.54	7.38	7.17	7.01	6.88	6.73	6.59	6.46	6.36	6.24	6.14
3796.62	7.34	7.19	7.04	6.90	6.78	6.60	6.53	6.43	6.29	6.21	6.11

Wavelength (nm) / Time (ps)	615.09	616.50	617.91	619.32	620.72	622.13	623.54	624.95	626.36	627.77	629.18
-3.28	0.03	0.03	0.06	0.10	0.11	0.09	0.10	0.12	0.11	0.11	0.13
-2.78	0.05	0.02	0.01	0.01	0.04	0.04	0.02	0.05	0.07	0.08	0.05
-2.28	-0.02	-0.01	-0.01	0.04	-0.01	0.00	0.01	0.01	0.04	0.02	0.05
-1.78	0.06	0.01	-0.02	-0.04	-0.05	-0.07	-0.09	-0.11	-0.13	-0.11	-0.14
-1.28	-0.04	-0.03	0.00	-0.04	-0.02	-0.02	-0.01	-0.06	-0.02	-0.04	-0.03
-0.78	0.04	0.02	0.06	0.03	0.05	0.07	0.08	0.07	0.06	0.07	0.04
-0.28	-0.06	-0.03	-0.04	-0.05	-0.06	-0.03	-0.03	-0.05	-0.02	-0.02	-0.01
0.22	-0.02	-0.01	-0.04	-0.05	-0.05	-0.05	-0.07	-0.04	-0.11	-0.06	-0.06
0.32	0.00	0.00	0.00	-0.01	0.02	-0.01	0.01	0.03	0.01	0.00	0.02
0.42	-0.04	-0.01	-0.02	0.02	-0.02	-0.01	-0.02	-0.02	-0.02	-0.04	-0.04

0.52	-0.03	-0.02	-0.04	-0.06	-0.06	-0.01	0.00	-0.03	-0.03	-0.02	0.00
0.62	-0.05	-0.03	-0.09	-0.08	-0.09	-0.06	-0.10	-0.08	-0.09	-0.06	-0.02
0.72	-0.06	-0.13	-0.10	-0.13	-0.11	-0.12	-0.11	-0.13	-0.07	-0.09	-0.09
0.77	-0.07	-0.10	-0.09	-0.09	-0.07	-0.05	-0.06	-0.07	-0.05	-0.04	-0.09
0.82	-0.04	0.00	0.00	0.00	-0.02	-0.04	-0.06	-0.01	-0.01	0.00	-0.02
0.87	0.07	0.06	0.02	-0.02	0.00	0.02	0.02	0.04	0.05	0.02	0.02
0.92	-0.09	-0.08	-0.11	-0.13	-0.09	-0.07	-0.05	-0.06	-0.08	-0.07	-0.05
0.97	0.04	0.04	0.04	0.02	0.06	0.06	0.02	-0.03	0.02	0.03	0.05
1.02	0.00	0.03	0.00	0.01	0.03	0.03	0.03	0.06	0.02	0.04	0.02
1.07	0.00	-0.02	-0.04	-0.04	-0.08	-0.06	-0.03	-0.03	-0.04	-0.05	-0.05
1.12	0.02	0.03	-0.01	0.03	0.01	0.05	0.06	0.04	0.08	0.04	0.08
1.17	0.01	0.01	0.03	-0.03	0.01	0.02	0.02	0.04	0.05	0.08	0.11
1.22	-0.11	-0.07	-0.05	-0.04	-0.07	-0.07	-0.10	-0.07	-0.09	-0.02	-0.03
1.27	0.05	0.02	0.04	0.01	0.02	0.03	-0.01	0.04	-0.02	-0.05	-0.03
1.32	0.02	0.01	0.05	0.03	0.02	-0.01	0.00	0.05	-0.02	0.00	0.01
1.37	-0.05	-0.02	-0.05	-0.02	-0.04	-0.03	-0.03	0.04	0.02	0.04	0.07
1.42	-0.03	-0.03	-0.02	-0.04	-0.03	-0.03	-0.02	0.02	0.03	0.04	-0.04
1.47	-0.05	-0.05	-0.04	-0.04	-0.05	-0.06	-0.05	-0.05	-0.03	0.00	-0.02
1.52	-0.08	-0.06	-0.05	-0.05	-0.11	-0.09	-0.01	-0.01	0.02	0.00	0.02
1.57	0.44	0.45	0.34	0.26	0.18	0.12	0.10	0.09	0.03	0.04	0.03
1.62	3.46	3.34	3.19	3.03	2.85	2.69	2.54	2.37	2.25	2.11	2.01
1.67	8.38	8.33	8.28	8.16	8.07	7.98	7.85	7.81	7.68	7.57	7.44
1.72	9.42	9.47	9.50	9.50	9.52	9.52	9.56	9.56	9.56	9.53	9.56
1.77	8.28	8.36	8.40	8.45	8.51	8.58	8.63	8.75	8.76	8.80	8.94
1.82	7.07	7.11	7.16	7.20	7.26	7.30	7.41	7.50	7.53	7.61	7.65
1.87	6.23	6.24	6.29	6.32	6.41	6.48	6.51	6.58	6.71	6.72	6.79
1.92	5.91	5.95	5.92	6.00	6.03	6.08	6.13	6.23	6.26	6.34	6.44
1.97	5.78	5.82	5.85	5.84	5.89	5.93	6.01	6.08	6.16	6.23	6.29
2.02	5.64	5.64	5.65	5.67	5.70	5.71	5.73	5.83	5.91	5.96	6.03
2.07	5.57	5.58	5.61	5.61	5.64	5.66	5.74	5.79	5.87	5.92	6.03
2.12	5.62	5.60	5.61	5.63	5.66	5.68	5.67	5.76	5.78	5.88	5.96
2.17	5.28	5.26	5.19	5.19	5.22	5.22	5.23	5.31	5.33	5.38	5.41
2.22	5.14	5.16	5.16	5.09	5.09	5.14	5.15	5.19	5.23	5.30	5.36
2.27	5.11	5.06	5.01	5.02	5.03	5.04	5.05	5.12	5.17	5.14	5.22
2.32	4.92	4.91	4.88	4.85	4.82	4.90	4.90	4.97	4.99	5.08	5.09
2.37	5.11	5.08	5.01	4.99	5.02	5.02	5.05	5.08	5.10	5.14	5.22
2.42	4.92	4.87	4.82	4.83	4.79	4.79	4.82	4.84	4.92	4.99	5.02
2.47	4.67	4.62	4.53	4.53	4.55	4.55	4.58	4.59	4.64	4.67	4.73
2.52	4.84	4.79	4.74	4.68	4.70	4.70	4.70	4.72	4.71	4.80	4.86
2.57	4.68	4.65	4.61	4.58	4.57	4.58	4.58	4.66	4.69	4.72	4.75
2.62	4.61	4.55	4.50	4.47	4.48	4.46	4.47	4.48	4.56	4.58	4.62
2.67	4.54	4.45	4.38	4.38	4.36	4.34	4.35	4.35	4.36	4.45	4.48
2.72	4.51	4.49	4.42	4.38	4.33	4.31	4.34	4.39	4.38	4.42	4.46
2.77	4.50	4.45	4.33	4.33	4.30	4.29	4.26	4.29	4.34	4.33	4.35
2.82	4.46	4.43	4.36	4.28	4.26	4.24	4.23	4.24	4.25	4.28	4.31
2.87	4.43	4.38	4.32	4.26	4.26	4.25	4.24	4.30	4.30	4.32	4.41
2.92	4.38	4.29	4.23	4.21	4.16	4.16	4.18	4.21	4.22	4.26	4.26
2.97	4.42	4.35	4.34	4.26	4.19	4.20	4.18	4.24	4.26	4.29	4.27
3.02	4.43	4.36	4.26	4.20	4.17	4.20	4.15	4.16	4.20	4.21	4.24
3.07	4.36	4.32	4.23	4.11	4.09	4.07	4.13	4.15	4.14	4.17	4.19

3.12	4.44	4.33	4.25	4.22	4.16	4.13	4.14	4.14	4.17	4.17	4.25
3.17	4.40	4.36	4.26	4.21	4.14	4.12	4.12	4.14	4.14	4.15	4.16
3.22	4.31	4.19	4.11	4.09	4.02	4.00	3.97	4.01	4.00	4.06	4.08
3.27	4.20	4.10	3.98	3.93	3.91	3.85	3.89	3.91	3.89	3.90	3.93
3.32	4.32	4.26	4.13	4.06	4.02	4.00	3.98	3.97	3.99	4.03	4.07
3.37	4.19	4.09	4.03	3.95	3.93	3.89	3.89	3.90	3.88	3.91	3.97
3.42	4.22	4.10	4.01	3.96	3.91	3.92	3.85	3.86	3.90	3.89	3.94
3.47	4.24	4.12	4.07	3.98	3.99	3.94	3.93	3.96	3.92	3.94	4.03
3.52	4.05	3.99	3.93	3.85	3.82	3.77	3.77	3.81	3.78	3.82	3.84
3.57	4.00	3.89	3.82	3.77	3.75	3.70	3.67	3.73	3.71	3.79	3.80
3.62	3.94	3.87	3.81	3.72	3.65	3.64	3.65	3.61	3.64	3.66	3.67
3.67	3.98	3.89	3.77	3.76	3.68	3.71	3.64	3.65	3.65	3.73	3.72
3.72	3.95	3.87	3.73	3.70	3.65	3.67	3.60	3.62	3.62	3.66	3.68
3.92	4.07	3.96	3.88	3.81	3.74	3.76	3.73	3.69	3.71	3.70	3.72
4.12	3.90	3.79	3.73	3.62	3.58	3.51	3.44	3.46	3.46	3.48	3.47
4.32	3.66	3.59	3.50	3.41	3.37	3.32	3.27	3.28	3.30	3.32	3.35
4.52	3.67	3.56	3.50	3.41	3.34	3.38	3.27	3.29	3.27	3.30	3.29
4.72	3.70	3.55	3.47	3.40	3.35	3.34	3.29	3.28	3.25	3.29	3.34
4.92	3.73	3.65	3.50	3.45	3.37	3.34	3.32	3.31	3.30	3.32	3.37
5.12	3.51	3.43	3.31	3.21	3.18	3.13	3.09	3.05	3.05	3.08	3.09
5.32	3.61	3.46	3.39	3.26	3.20	3.16	3.11	3.10	3.09	3.10	3.06
5.52	3.49	3.35	3.26	3.20	3.09	3.06	3.05	3.04	3.01	3.03	2.98
5.72	3.45	3.29	3.22	3.12	3.06	3.00	2.99	3.01	2.97	2.99	3.03
5.92	3.42	3.29	3.23	3.15	3.10	3.03	3.01	3.02	3.02	3.04	3.05
6.12	3.46	3.36	3.23	3.14	3.10	3.04	3.00	3.03	2.96	3.02	3.03
6.32	3.33	3.27	3.21	3.10	3.03	2.96	2.92	2.96	2.90	2.98	2.96
6.52	3.22	3.14	3.03	2.93	2.90	2.85	2.78	2.77	2.80	2.79	2.78
6.72	3.37	3.26	3.13	3.04	2.97	2.93	2.92	2.90	2.83	2.86	2.85
6.92	3.27	3.20	3.06	2.98	2.96	2.89	2.83	2.85	2.81	2.88	2.85
7.12	3.19	3.14	3.03	2.93	2.92	2.84	2.76	2.79	2.76	2.77	2.81
7.32	3.27	3.18	3.08	2.97	2.94	2.91	2.87	2.85	2.84	2.84	2.89
7.52	3.20	3.05	2.98	2.90	2.86	2.80	2.75	2.77	2.76	2.70	2.74
7.72	3.19	3.09	2.99	2.90	2.85	2.86	2.75	2.76	2.74	2.77	2.78
7.92	3.03	2.94	2.87	2.78	2.72	2.68	2.68	2.69	2.67	2.67	2.74
8.12	3.11	3.02	2.89	2.77	2.70	2.66	2.66	2.62	2.62	2.62	2.69
8.32	3.04	2.93	2.82	2.71	2.68	2.63	2.57	2.59	2.58	2.62	2.62
8.52	3.06	2.95	2.86	2.75	2.76	2.71	2.66	2.68	2.67	2.67	2.70
8.72	3.25	3.14	3.05	3.02	2.91	2.87	2.85	2.82	2.84	2.81	2.81
8.92	3.08	3.01	2.91	2.78	2.75	2.72	2.74	2.73	2.74	2.73	2.75
9.12	3.07	2.96	2.85	2.73	2.72	2.68	2.65	2.61	2.62	2.65	2.64
9.32	2.89	2.77	2.70	2.65	2.59	2.57	2.52	2.56	2.53	2.53	2.55
9.52	3.03	2.97	2.86	2.78	2.68	2.68	2.62	2.64	2.65	2.63	2.64
9.72	2.93	2.88	2.76	2.66	2.62	2.57	2.54	2.54	2.57	2.56	2.56
9.92	2.90	2.79	2.68	2.60	2.52	2.49	2.42	2.43	2.38	2.45	2.45
10.12	3.00	2.93	2.80	2.74	2.66	2.61	2.60	2.59	2.56	2.55	2.54
10.32	2.77	2.70	2.57	2.55	2.46	2.44	2.43	2.39	2.41	2.41	2.44
10.52	2.98	2.86	2.76	2.69	2.61	2.59	2.54	2.59	2.58	2.59	2.58
10.72	2.89	2.78	2.69	2.56	2.52	2.49	2.49	2.48	2.48	2.47	2.50
10.92	2.91	2.76	2.67	2.56	2.52	2.47	2.45	2.48	2.44	2.46	2.44
11.12	2.87	2.72	2.64	2.60	2.55	2.47	2.47	2.43	2.47	2.46	2.46

11.32	2.82	2.74	2.58	2.51	2.46	2.43	2.36	2.37	2.37	2.40	2.40
11.52	2.80	2.68	2.63	2.53	2.47	2.46	2.44	2.45	2.41	2.43	2.48
11.72	2.79	2.64	2.56	2.48	2.44	2.37	2.36	2.34	2.38	2.37	2.38
11.92	2.83	2.73	2.63	2.61	2.51	2.53	2.46	2.50	2.48	2.50	2.48
12.12	2.96	2.85	2.71	2.61	2.64	2.60	2.54	2.53	2.52	2.56	2.49
12.32	2.81	2.72	2.60	2.52	2.45	2.43	2.40	2.42	2.44	2.44	2.43
12.52	2.67	2.56	2.50	2.40	2.42	2.37	2.34	2.36	2.34	2.39	2.44
12.72	2.62	2.52	2.42	2.36	2.35	2.27	2.26	2.20	2.24	2.24	2.28
12.92	2.77	2.67	2.58	2.48	2.44	2.39	2.41	2.41	2.38	2.39	2.39
13.12	2.70	2.62	2.53	2.46	2.46	2.43	2.37	2.32	2.35	2.42	2.42
13.32	2.91	2.82	2.73	2.66	2.59	2.58	2.53	2.54	2.51	2.55	2.57
13.52	2.64	2.53	2.46	2.40	2.34	2.34	2.27	2.30	2.29	2.29	2.34
13.72	2.65	2.49	2.44	2.34	2.30	2.25	2.23	2.26	2.21	2.23	2.25
13.92	2.70	2.54	2.45	2.40	2.38	2.33	2.34	2.34	2.33	2.35	2.40
14.00	2.77	2.65	2.52	2.44	2.36	2.35	2.26	2.31	2.37	2.35	2.35
14.08	2.78	2.64	2.55	2.51	2.46	2.39	2.37	2.34	2.34	2.40	2.35
14.17	2.69	2.58	2.49	2.42	2.39	2.38	2.33	2.31	2.34	2.38	2.34
14.27	2.69	2.59	2.49	2.41	2.39	2.39	2.33	2.35	2.35	2.37	2.39
14.38	2.65	2.53	2.48	2.37	2.32	2.30	2.28	2.25	2.24	2.26	2.27
14.49	2.57	2.46	2.36	2.29	2.26	2.22	2.20	2.20	2.22	2.25	2.26
14.61	2.66	2.63	2.51	2.42	2.37	2.40	2.36	2.37	2.35	2.38	2.42
14.75	2.68	2.61	2.54	2.43	2.38	2.39	2.32	2.37	2.35	2.36	2.36
14.89	2.72	2.57	2.48	2.40	2.35	2.30	2.21	2.23	2.26	2.23	2.24
15.05	2.55	2.45	2.39	2.31	2.23	2.23	2.21	2.22	2.23	2.23	2.26
15.21	2.58	2.47	2.35	2.31	2.23	2.18	2.20	2.17	2.19	2.23	2.22
15.39	2.51	2.44	2.35	2.24	2.22	2.18	2.22	2.18	2.16	2.16	2.19
15.59	2.70	2.61	2.55	2.45	2.40	2.31	2.32	2.35	2.30	2.31	2.38
15.80	2.70	2.65	2.49	2.39	2.37	2.34	2.32	2.35	2.29	2.30	2.35
16.02	2.56	2.45	2.38	2.27	2.19	2.19	2.19	2.21	2.21	2.19	2.21
16.27	2.66	2.55	2.41	2.40	2.33	2.26	2.24	2.31	2.29	2.26	2.28
16.53	2.62	2.50	2.38	2.35	2.34	2.31	2.24	2.25	2.30	2.30	2.35
16.81	2.57	2.53	2.42	2.32	2.30	2.30	2.25	2.26	2.31	2.34	2.33
17.12	2.62	2.52	2.44	2.37	2.30	2.26	2.24	2.25	2.23	2.24	2.25
17.45	2.59	2.51	2.43	2.31	2.32	2.28	2.26	2.26	2.27	2.28	2.31
17.81	2.71	2.64	2.47	2.39	2.38	2.35	2.30	2.34	2.29	2.33	2.32
18.19	2.56	2.46	2.38	2.30	2.27	2.23	2.24	2.25	2.22	2.23	2.24
18.60	2.62	2.51	2.42	2.33	2.30	2.28	2.25	2.22	2.21	2.24	2.23
19.05	2.57	2.49	2.40	2.35	2.30	2.25	2.21	2.23	2.23	2.28	2.28
19.53	2.59	2.51	2.43	2.32	2.31	2.29	2.27	2.24	2.27	2.25	2.29
20.05	2.53	2.45	2.38	2.28	2.26	2.23	2.19	2.26	2.23	2.23	2.26
20.61	2.48	2.32	2.29	2.18	2.14	2.12	2.08	2.07	2.10	2.11	2.10
21.22	2.44	2.33	2.22	2.17	2.16	2.14	2.07	2.11	2.15	2.12	2.15
21.87	2.52	2.35	2.29	2.21	2.17	2.16	2.14	2.17	2.09	2.16	2.17
22.57	2.43	2.33	2.26	2.12	2.11	2.11	2.03	2.06	2.07	2.08	2.11
23.33	2.56	2.44	2.32	2.25	2.22	2.24	2.15	2.16	2.14	2.19	2.17
24.15	2.34	2.27	2.20	2.11	2.10	2.02	2.02	1.98	2.00	2.06	2.05
25.03	2.44	2.35	2.22	2.18	2.18	2.12	2.08	2.07	2.07	2.09	2.12
25.98	2.53	2.41	2.30	2.24	2.17	2.15	2.13	2.14	2.11	2.11	2.13
27.01	2.41	2.31	2.17	2.14	2.12	2.08	2.07	2.08	2.06	2.05	2.06
28.11	2.39	2.32	2.19	2.16	2.10	2.07	2.04	2.06	2.04	2.07	2.13

29.30	2.43	2.33	2.24	2.16	2.12	2.06	2.08	2.07	2.08	2.08	2.08
30.59	2.33	2.25	2.19	2.07	2.04	2.02	1.97	1.99	1.99	1.98	2.05
31.98	2.41	2.28	2.21	2.14	2.12	2.13	2.08	2.11	2.06	2.11	2.11
33.47	2.36	2.29	2.18	2.15	2.08	2.04	2.01	2.00	2.05	2.06	2.00
35.09	2.35	2.24	2.16	2.10	2.03	2.01	1.95	1.99	1.94	1.99	1.99
36.83	2.41	2.35	2.26	2.20	2.14	2.12	2.09	2.10	2.11	2.11	2.12
38.71	2.22	2.12	2.01	1.97	1.93	1.92	1.86	1.88	1.87	1.87	1.91
40.73	2.27	2.13	2.06	2.01	1.94	1.88	1.87	1.87	1.81	1.83	1.82
42.92	2.22	2.12	2.04	1.98	1.84	1.89	1.83	1.85	1.83	1.82	1.80
45.27	2.19	2.14	1.99	1.95	1.91	1.89	1.86	1.82	1.82	1.82	1.81
47.81	2.25	2.11	2.03	1.94	1.90	1.88	1.84	1.88	1.86	1.85	1.89
50.55	2.21	2.16	2.07	1.99	1.94	1.90	1.90	1.86	1.89	1.89	1.91
53.51	2.27	2.20	2.09	2.01	1.94	1.90	1.86	1.86	1.87	1.84	1.85
56.69	2.38	2.24	2.15	2.10	2.02	2.01	1.95	1.95	1.95	1.99	1.95
60.13	2.21	2.10	2.03	1.96	1.94	1.89	1.87	1.89	1.86	1.92	1.88
63.84	2.25	2.12	2.07	1.92	1.92	1.86	1.82	1.78	1.83	1.82	1.78
67.83	2.21	2.11	2.10	2.04	1.93	1.92	1.89	1.94	1.88	1.91	1.91
72.15	2.29	2.19	2.08	2.07	1.97	1.94	1.94	1.88	1.88	1.90	1.92
76.80	2.22	2.15	2.06	1.99	1.96	1.96	1.91	1.93	1.94	1.90	1.95
81.81	2.25	2.13	2.05	1.97	1.91	1.86	1.83	1.82	1.78	1.82	1.77
87.22	2.17	2.04	1.94	1.84	1.82	1.76	1.73	1.72	1.73	1.71	1.72
93.06	2.26	2.15	2.05	2.00	1.96	1.93	1.89	1.88	1.87	1.88	1.84
99.35	2.29	2.18	2.13	1.99	1.93	1.89	1.86	1.87	1.83	1.79	1.83
106.13	2.25	2.10	2.02	1.97	1.87	1.86	1.84	1.80	1.79	1.82	1.79
113.45	2.23	2.23	2.10	1.99	1.92	1.84	1.84	1.84	1.84	1.77	1.76
121.34	2.33	2.21	2.12	2.05	2.05	2.00	1.95	1.95	1.94	1.94	1.98
129.86	2.24	2.15	2.05	2.00	1.94	1.90	1.84	1.86	1.87	1.88	1.83
139.04	2.26	2.09	2.04	1.95	1.90	1.81	1.81	1.79	1.74	1.78	1.77
148.94	2.44	2.26	2.21	2.09	2.10	2.05	2.03	2.00	1.94	1.96	1.97
159.62	2.33	2.25	2.15	2.05	1.99	1.94	1.92	1.86	1.86	1.92	1.84
171.14	2.39	2.26	2.16	2.10	2.03	1.97	1.93	1.92	1.90	1.89	1.85
183.56	2.30	2.17	2.08	2.01	1.97	1.93	1.88	1.92	1.89	1.91	1.92
196.96	2.54	2.43	2.33	2.23	2.19	2.15	2.09	2.05	2.03	2.05	1.98
211.41	2.42	2.32	2.23	2.14	2.08	2.04	2.00	1.97	1.97	1.93	1.95
226.99	2.48	2.38	2.29	2.15	2.12	2.07	2.03	2.02	2.03	2.01	2.00
243.80	2.46	2.38	2.29	2.21	2.14	2.09	2.07	2.04	2.03	1.99	2.01
261.93	2.56	2.46	2.38	2.25	2.27	2.16	2.11	2.13	2.14	2.05	2.06
281.48	2.67	2.57	2.45	2.38	2.29	2.26	2.19	2.19	2.16	2.18	2.14
302.56	2.64	2.53	2.38	2.34	2.25	2.22	2.18	2.18	2.15	2.11	2.14
325.30	2.72	2.61	2.53	2.39	2.37	2.33	2.24	2.24	2.23	2.18	2.12
349.83	2.72	2.65	2.54	2.45	2.34	2.32	2.27	2.17	2.20	2.16	2.18
376.28	2.88	2.80	2.69	2.60	2.53	2.45	2.43	2.43	2.39	2.35	2.34
404.81	2.91	2.84	2.74	2.57	2.50	2.43	2.39	2.37	2.30	2.27	2.23
435.58	2.98	2.88	2.77	2.66	2.58	2.57	2.48	2.43	2.38	2.38	2.37
468.76	3.09	3.01	2.91	2.78	2.72	2.62	2.56	2.53	2.50	2.42	2.40
504.55	3.23	3.11	2.99	2.88	2.83	2.76	2.75	2.71	2.64	2.64	2.61
543.15	3.29	3.18	3.11	2.98	2.89	2.86	2.76	2.74	2.71	2.65	2.64
584.78	3.32	3.21	3.11	3.05	2.99	2.92	2.86	2.79	2.78	2.75	2.71
629.68	3.37	3.26	3.15	3.01	3.00	2.95	2.86	2.80	2.75	2.69	2.69
678.10	3.64	3.48	3.43	3.37	3.26	3.20	3.12	3.07	3.03	3.01	3.01

730.33	3.77	3.65	3.56	3.43	3.40	3.32	3.27	3.19	3.15	3.07	3.03
786.65	3.91	3.80	3.72	3.59	3.48	3.45	3.36	3.32	3.27	3.22	3.17
847.40	4.10	4.01	3.85	3.77	3.65	3.64	3.54	3.51	3.45	3.38	3.32
912.92	4.22	4.14	3.97	3.88	3.82	3.76	3.64	3.60	3.55	3.49	3.46
983.58	4.38	4.30	4.18	4.04	3.98	3.92	3.85	3.80	3.70	3.69	3.65
1059.78	4.48	4.34	4.24	4.13	4.09	3.92	3.88	3.85	3.73	3.67	3.62
1141.98	4.55	4.46	4.36	4.26	4.20	4.07	4.02	3.98	3.91	3.88	3.77
1230.62	4.46	4.37	4.25	4.14	4.08	4.02	3.92	3.88	3.85	3.77	3.71
1326.23	4.79	4.70	4.58	4.45	4.37	4.25	4.21	4.11	4.06	4.00	3.92
1429.34	5.16	5.02	4.93	4.83	4.70	4.60	4.54	4.42	4.39	4.30	4.26
1540.54	5.08	4.93	4.82	4.76	4.65	4.58	4.48	4.36	4.35	4.27	4.17
1660.48	5.34	5.23	5.13	5.00	4.94	4.83	4.74	4.65	4.62	4.53	4.41
1789.83	5.47	5.36	5.23	5.11	5.05	4.94	4.85	4.77	4.69	4.64	4.59
1929.34	5.51	5.39	5.29	5.16	5.05	4.97	4.86	4.78	4.71	4.59	4.50
2079.80	5.61	5.42	5.35	5.20	5.12	5.04	4.94	4.85	4.78	4.72	4.62
2242.08	5.72	5.60	5.53	5.39	5.34	5.22	5.11	5.06	4.96	4.87	4.78
2417.10	5.88	5.79	5.66	5.54	5.41	5.37	5.23	5.19	5.06	4.99	4.87
2605.85	5.82	5.68	5.59	5.50	5.37	5.28	5.15	5.03	4.97	4.91	4.80
2809.43	5.71	5.64	5.54	5.40	5.33	5.19	5.18	5.05	4.94	4.85	4.79
3028.99	6.01	5.91	5.81	5.68	5.59	5.49	5.42	5.34	5.27	5.17	5.10
3265.79	6.02	5.87	5.79	5.67	5.62	5.50	5.41	5.30	5.19	5.14	5.06
3521.18	5.96	5.83	5.75	5.62	5.55	5.46	5.35	5.29	5.17	5.10	5.01
3796.62	6.01	5.90	5.79	5.69	5.63	5.51	5.42	5.37	5.26	5.14	5.04

Wavelength (nm) / *Time (ps)*	*630.58*	*631.99*	*633.40*	*634.81*	*636.22*	*637.63*	*639.04*	*640.44*	*641.85*	*643.26*	*644.67*
-3.28	0.12	0.12	0.11	0.11	0.17	0.17	0.10	0.10	0.05	0.06	0.12
-2.78	0.05	0.05	0.09	0.07	0.05	0.07	0.05	0.03	0.07	0.05	0.06
-2.28	0.04	0.02	0.02	0.00	-0.01	0.04	0.04	0.06	0.04	0.07	0.04
-1.78	-0.14	-0.18	-0.15	-0.15	-0.14	-0.17	-0.15	-0.18	-0.19	-0.17	-0.15
-1.28	-0.05	-0.04	-0.07	-0.02	-0.02	0.03	0.01	0.00	-0.03	0.02	0.00
-0.78	0.08	0.11	0.11	0.09	0.13	0.09	0.10	0.13	0.15	0.13	0.11
-0.28	-0.02	0.00	0.00	0.03	-0.02	-0.03	0.00	0.00	0.01	-0.05	-0.02
0.22	-0.07	-0.08	-0.06	-0.08	-0.10	-0.10	-0.11	-0.09	-0.09	-0.08	-0.10
0.32	0.04	0.04	0.03	0.01	-0.03	-0.01	0.05	0.00	0.02	0.01	0.00
0.42	-0.04	-0.04	-0.09	-0.06	-0.03	-0.09	-0.08	-0.06	-0.03	-0.04	-0.04
0.52	0.01	0.04	-0.03	0.02	0.01	0.05	0.03	0.05	0.07	0.07	0.04
0.62	-0.04	-0.06	-0.04	-0.02	0.00	-0.01	-0.01	0.03	0.01	0.02	-0.01
0.72	-0.11	-0.12	-0.15	-0.11	-0.09	-0.14	-0.15	-0.16	-0.14	-0.11	-0.15
0.77	-0.07	-0.09	-0.07	-0.07	-0.06	0.01	-0.09	-0.08	-0.07	-0.07	-0.08
0.82	-0.05	-0.04	-0.05	-0.04	-0.05	-0.06	-0.05	-0.11	-0.12	-0.05	-0.06
0.87	0.01	0.01	0.06	0.04	0.01	-0.05	-0.04	-0.03	-0.02	-0.01	-0.03
0.92	-0.03	-0.07	-0.01	-0.04	-0.07	-0.06	0.01	0.02	0.04	-0.02	-0.01
0.97	0.01	0.03	-0.03	0.00	0.02	-0.01	0.02	0.00	-0.03	-0.02	-0.01
1.02	0.08	0.07	0.10	0.07	0.08	0.05	0.03	0.10	0.07	0.09	0.10
1.07	-0.10	-0.07	-0.09	-0.10	-0.11	-0.12	-0.11	-0.10	-0.11	-0.11	-0.10
1.12	0.06	0.12	0.12	0.09	0.11	0.12	0.12	0.17	0.14	0.13	0.06
1.17	0.07	0.08	0.13	0.08	0.12	0.12	0.18	0.15	0.14	0.17	0.17
1.22	-0.04	-0.07	-0.04	-0.03	-0.05	0.00	-0.02	0.01	-0.01	-0.03	-0.02

1.27	-0.08	-0.05	-0.03	-0.07	-0.06	-0.08	-0.08	-0.07	-0.08	-0.08	-0.07
1.32	-0.03	-0.04	0.00	0.01	-0.01	-0.04	-0.02	0.01	0.02	0.08	0.08
1.37	0.03	0.06	0.11	0.10	0.11	0.10	0.10	0.09	0.05	0.09	0.07
1.42	0.00	0.01	0.06	0.04	0.01	0.03	0.01	0.07	0.05	0.07	0.01
1.47	-0.02	0.02	0.01	0.06	0.02	0.04	0.05	0.04	0.10	0.07	0.06
1.52	0.02	0.02	0.06	0.10	0.12	0.06	0.01	0.02	0.07	0.15	0.10
1.57	-0.01	-0.01	-0.01	-0.03	0.01	-0.02	-0.05	-0.04	-0.12	-0.10	-0.09
1.62	1.86	1.79	1.71	1.60	1.54	1.49	1.37	1.23	1.18	1.11	1.03
1.67	7.32	7.24	7.13	7.04	6.97	6.83	6.73	6.64	6.49	6.33	6.22
1.72	9.53	9.53	9.56	9.55	9.54	9.56	9.56	9.51	9.52	9.52	9.49
1.77	8.97	9.01	9.06	9.11	9.15	9.26	9.30	9.33	9.35	9.35	9.41
1.82	7.72	7.79	7.88	7.97	8.06	8.11	8.16	8.18	8.25	8.32	8.38
1.87	6.86	6.93	7.05	7.10	7.18	7.20	7.28	7.33	7.38	7.41	7.46
1.92	6.50	6.58	6.65	6.73	6.81	6.83	6.91	6.94	7.01	7.05	7.09
1.97	6.36	6.44	6.53	6.56	6.64	6.68	6.78	6.82	6.82	6.89	6.89
2.02	6.09	6.11	6.24	6.27	6.37	6.35	6.44	6.46	6.50	6.55	6.55
2.07	6.08	6.10	6.18	6.23	6.31	6.39	6.39	6.45	6.43	6.55	6.50
2.12	5.98	6.00	6.13	6.16	6.22	6.24	6.36	6.35	6.37	6.38	6.47
2.17	5.51	5.55	5.58	5.67	5.71	5.78	5.81	5.86	5.91	5.94	5.91
2.22	5.41	5.48	5.52	5.54	5.62	5.65	5.73	5.80	5.85	5.84	5.87
2.27	5.24	5.30	5.42	5.44	5.46	5.49	5.54	5.65	5.70	5.72	5.70
2.32	5.15	5.24	5.26	5.37	5.43	5.48	5.53	5.56	5.58	5.61	5.67
2.37	5.26	5.36	5.38	5.38	5.46	5.53	5.57	5.63	5.72	5.68	5.71
2.42	5.08	5.16	5.16	5.26	5.28	5.39	5.44	5.45	5.51	5.53	5.56
2.47	4.75	4.90	4.95	5.00	5.07	5.11	5.17	5.21	5.33	5.35	5.33
2.52	4.85	4.91	4.99	5.01	5.07	5.14	5.16	5.20	5.20	5.26	5.26
2.57	4.80	4.86	4.91	5.02	5.09	5.10	5.14	5.18	5.18	5.26	5.27
2.62	4.65	4.69	4.76	4.84	4.86	4.88	4.91	4.98	5.00	5.03	5.07
2.67	4.53	4.53	4.62	4.70	4.76	4.81	4.89	4.91	4.95	4.98	4.97
2.72	4.52	4.59	4.65	4.62	4.69	4.72	4.75	4.81	4.81	4.89	4.89
2.77	4.41	4.42	4.50	4.57	4.59	4.67	4.63	4.74	4.76	4.78	4.79
2.82	4.37	4.38	4.49	4.51	4.60	4.63	4.66	4.72	4.78	4.79	4.77
2.87	4.43	4.47	4.51	4.55	4.66	4.66	4.69	4.76	4.77	4.79	4.84
2.92	4.32	4.36	4.47	4.50	4.52	4.59	4.64	4.69	4.71	4.79	4.77
2.97	4.32	4.35	4.44	4.48	4.54	4.51	4.59	4.61	4.69	4.70	4.69
3.02	4.27	4.35	4.42	4.44	4.52	4.54	4.62	4.62	4.70	4.71	4.70
3.07	4.24	4.29	4.40	4.43	4.45	4.51	4.58	4.61	4.68	4.71	4.75
3.12	4.27	4.33	4.35	4.38	4.45	4.49	4.51	4.57	4.62	4.65	4.61
3.17	4.20	4.25	4.28	4.33	4.39	4.44	4.48	4.54	4.59	4.59	4.58
3.22	4.11	4.13	4.19	4.24	4.27	4.34	4.37	4.40	4.42	4.50	4.46
3.27	4.01	4.06	4.12	4.16	4.20	4.24	4.28	4.30	4.34	4.36	4.38
3.32	4.08	4.07	4.17	4.19	4.27	4.29	4.37	4.36	4.38	4.39	4.44
3.37	3.97	4.03	4.08	4.16	4.19	4.22	4.26	4.26	4.29	4.35	4.37
3.42	3.94	3.99	4.00	4.07	4.15	4.15	4.19	4.21	4.24	4.30	4.33
3.47	4.03	4.10	4.18	4.21	4.27	4.34	4.40	4.41	4.40	4.40	4.49
3.52	3.92	3.99	3.98	4.08	4.12	4.14	4.23	4.26	4.25	4.31	4.33
3.57	3.79	3.81	3.90	3.96	4.04	4.11	4.12	4.14	4.13	4.19	4.25
3.62	3.71	3.75	3.76	3.86	3.90	3.95	3.96	4.01	4.06	4.05	4.04
3.67	3.72	3.76	3.84	3.89	3.94	3.93	4.01	4.01	4.03	4.09	4.11
3.72	3.72	3.77	3.82	3.85	3.88	3.91	3.96	4.04	4.07	4.05	4.02

3.92	3.73	3.77	3.81	3.87	3.93	3.94	3.97	4.02	4.06	4.10	4.05
4.12	3.53	3.55	3.62	3.65	3.71	3.70	3.80	3.80	3.82	3.88	3.94
4.32	3.41	3.41	3.45	3.44	3.50	3.56	3.63	3.62	3.68	3.66	3.69
4.52	3.34	3.35	3.43	3.41	3.48	3.54	3.59	3.57	3.60	3.57	3.60
4.72	3.37	3.37	3.43	3.44	3.48	3.55	3.59	3.65	3.62	3.63	3.67
4.92	3.34	3.38	3.41	3.42	3.52	3.50	3.50	3.54	3.57	3.60	3.59
5.12	3.05	3.10	3.17	3.18	3.23	3.23	3.25	3.25	3.34	3.39	3.37
5.32	3.09	3.11	3.12	3.20	3.20	3.20	3.22	3.28	3.33	3.40	3.36
5.52	2.99	3.05	3.10	3.11	3.08	3.16	3.15	3.14	3.20	3.18	3.20
5.72	3.02	3.04	3.08	3.17	3.16	3.20	3.22	3.22	3.27	3.32	3.27
5.92	3.06	3.11	3.12	3.16	3.23	3.23	3.25	3.29	3.33	3.33	3.38
6.12	3.04	3.08	3.06	3.10	3.18	3.20	3.23	3.22	3.27	3.27	3.25
6.32	2.95	3.00	3.08	3.06	3.11	3.13	3.16	3.21	3.20	3.26	3.26
6.52	2.80	2.85	2.94	2.96	2.94	3.01	3.03	3.04	3.07	3.13	3.14
6.72	2.85	2.87	2.92	2.95	2.96	3.01	3.05	3.04	3.04	3.08	3.06
6.92	2.90	2.90	2.95	2.95	2.95	2.98	3.01	3.03	3.07	3.08	3.06
7.12	2.80	2.83	2.89	2.92	2.95	2.96	2.99	3.01	3.00	3.06	3.06
7.32	2.89	2.91	2.93	2.97	3.03	3.04	3.04	3.02	3.06	3.06	3.06
7.52	2.76	2.79	2.87	2.90	2.90	2.89	2.92	3.01	3.02	3.05	3.00
7.72	2.82	2.78	2.85	2.85	2.92	2.95	3.00	3.06	3.02	3.04	3.05
7.92	2.73	2.75	2.75	2.83	2.86	2.92	2.92	2.88	2.91	2.97	2.97
8.12	2.68	2.69	2.71	2.71	2.76	2.80	2.81	2.81	2.86	2.84	2.90
8.32	2.63	2.65	2.67	2.73	2.78	2.80	2.77	2.81	2.81	2.85	2.86
8.52	2.69	2.71	2.73	2.81	2.82	2.83	2.85	2.85	2.88	2.89	2.93
8.72	2.88	2.86	2.95	2.92	2.92	3.00	3.00	3.03	3.00	2.96	3.04
8.92	2.78	2.78	2.89	2.87	2.90	2.88	2.98	2.99	2.96	3.01	3.01
9.12	2.67	2.69	2.70	2.75	2.79	2.82	2.84	2.85	2.86	2.89	2.88
9.32	2.60	2.63	2.62	2.66	2.72	2.73	2.74	2.74	2.78	2.81	2.79
9.52	2.68	2.70	2.75	2.75	2.79	2.78	2.80	2.84	2.84	2.83	2.83
9.72	2.59	2.59	2.66	2.64	2.70	2.67	2.66	2.71	2.72	2.71	2.69
9.92	2.42	2.44	2.52	2.49	2.54	2.54	2.52	2.56	2.60	2.62	2.55
10.12	2.57	2.65	2.67	2.69	2.76	2.78	2.77	2.80	2.86	2.83	2.86
10.32	2.45	2.52	2.51	2.55	2.58	2.56	2.58	2.65	2.64	2.68	2.64
10.52	2.61	2.64	2.70	2.72	2.71	2.81	2.81	2.80	2.80	2.81	2.81
10.72	2.48	2.54	2.57	2.56	2.59	2.65	2.67	2.69	2.66	2.69	2.59
10.92	2.49	2.45	2.48	2.55	2.55	2.56	2.57	2.61	2.58	2.57	2.60
11.12	2.45	2.49	2.48	2.55	2.61	2.58	2.59	2.59	2.62	2.60	2.64
11.32	2.42	2.45	2.45	2.47	2.52	2.53	2.57	2.60	2.58	2.62	2.61
11.52	2.46	2.51	2.51	2.52	2.51	2.53	2.63	2.55	2.61	2.62	2.57
11.72	2.39	2.42	2.47	2.52	2.52	2.54	2.57	2.54	2.58	2.59	2.59
11.92	2.53	2.54	2.58	2.63	2.60	2.62	2.65	2.62	2.67	2.73	2.67
12.12	2.54	2.59	2.63	2.64	2.68	2.66	2.72	2.74	2.73	2.72	2.72
12.32	2.42	2.49	2.52	2.54	2.56	2.59	2.61	2.65	2.68	2.70	2.69
12.52	2.41	2.46	2.51	2.52	2.56	2.58	2.61	2.60	2.65	2.64	2.64
12.72	2.29	2.31	2.35	2.31	2.38	2.42	2.42	2.38	2.40	2.37	2.40
12.92	2.39	2.44	2.45	2.45	2.52	2.50	2.56	2.54	2.56	2.54	2.53
13.12	2.36	2.42	2.46	2.46	2.56	2.52	2.53	2.53	2.50	2.54	2.55
13.32	2.58	2.55	2.58	2.62	2.63	2.64	2.66	2.65	2.63	2.61	2.62
13.52	2.28	2.31	2.36	2.36	2.43	2.37	2.36	2.39	2.43	2.42	2.46
13.72	2.25	2.27	2.32	2.31	2.36	2.38	2.40	2.40	2.39	2.47	2.48

13.92	2.41	2.41	2.44	2.45	2.49	2.54	2.58	2.56	2.60	2.55	2.54
14.00	2.40	2.41	2.50	2.50	2.48	2.49	2.53	2.54	2.54	2.57	2.56
14.08	2.30	2.33	2.39	2.48	2.45	2.43	2.41	2.46	2.46	2.52	2.47
14.17	2.39	2.41	2.43	2.47	2.48	2.53	2.49	2.51	2.52	2.55	2.51
14.27	2.45	2.49	2.49	2.49	2.52	2.59	2.62	2.57	2.57	2.57	2.65
14.38	2.32	2.30	2.30	2.35	2.33	2.39	2.44	2.39	2.42	2.45	2.41
14.49	2.26	2.30	2.35	2.38	2.38	2.43	2.44	2.46	2.47	2.49	2.47
14.61	2.38	2.39	2.50	2.49	2.53	2.52	2.56	2.60	2.55	2.54	2.58
14.75	2.39	2.42	2.44	2.43	2.46	2.48	2.47	2.52	2.49	2.49	2.52
14.89	2.21	2.27	2.31	2.32	2.33	2.40	2.37	2.38	2.39	2.44	2.43
15.05	2.26	2.28	2.34	2.38	2.40	2.39	2.46	2.45	2.46	2.50	2.49
15.21	2.22	2.23	2.29	2.28	2.34	2.36	2.39	2.34	2.34	2.39	2.42
15.39	2.21	2.23	2.30	2.28	2.27	2.30	2.23	2.29	2.29	2.28	2.29
15.59	2.38	2.43	2.42	2.44	2.42	2.49	2.50	2.52	2.50	2.46	2.48
15.80	2.38	2.37	2.43	2.44	2.39	2.44	2.48	2.49	2.46	2.44	2.46
16.02	2.23	2.23	2.34	2.28	2.33	2.33	2.31	2.39	2.42	2.41	2.36
16.27	2.32	2.33	2.39	2.39	2.41	2.39	2.39	2.42	2.42	2.39	2.43
16.53	2.31	2.27	2.37	2.46	2.45	2.48	2.40	2.47	2.46	2.51	2.54
16.81	2.35	2.35	2.43	2.43	2.44	2.44	2.49	2.53	2.49	2.48	2.54
17.12	2.28	2.33	2.37	2.40	2.38	2.36	2.35	2.37	2.37	2.45	2.39
17.45	2.31	2.38	2.36	2.43	2.38	2.44	2.40	2.44	2.46	2.44	2.41
17.81	2.26	2.31	2.40	2.39	2.41	2.37	2.37	2.36	2.40	2.44	2.43
18.19	2.28	2.26	2.34	2.36	2.35	2.36	2.41	2.42	2.44	2.45	2.40
18.60	2.24	2.28	2.29	2.31	2.36	2.36	2.39	2.37	2.32	2.35	2.35
19.05	2.26	2.30	2.32	2.34	2.32	2.37	2.40	2.42	2.45	2.39	2.38
19.53	2.29	2.33	2.32	2.36	2.38	2.38	2.43	2.44	2.48	2.44	2.44
20.05	2.28	2.28	2.32	2.36	2.42	2.39	2.39	2.41	2.42	2.50	2.46
20.61	2.11	2.22	2.20	2.20	2.23	2.25	2.24	2.29	2.30	2.31	2.27
21.22	2.10	2.15	2.20	2.21	2.25	2.20	2.25	2.22	2.24	2.30	2.25
21.87	2.20	2.21	2.29	2.30	2.30	2.32	2.34	2.39	2.38	2.38	2.39
22.57	2.15	2.20	2.20	2.17	2.27	2.30	2.30	2.29	2.31	2.28	2.28
23.33	2.22	2.20	2.22	2.28	2.27	2.27	2.25	2.26	2.24	2.27	2.28
24.15	2.07	2.09	2.14	2.14	2.15	2.16	2.15	2.16	2.15	2.11	2.12
25.03	2.07	2.10	2.14	2.14	2.19	2.17	2.16	2.15	2.17	2.14	2.19
25.98	2.19	2.15	2.18	2.19	2.19	2.21	2.24	2.26	2.30	2.28	2.25
27.01	2.13	2.15	2.13	2.17	2.14	2.16	2.20	2.23	2.26	2.22	2.21
28.11	2.10	2.12	2.15	2.18	2.24	2.18	2.19	2.17	2.15	2.15	2.17
29.30	2.08	2.11	2.16	2.15	2.16	2.14	2.11	2.13	2.18	2.20	2.09
30.59	2.02	2.01	2.04	2.06	2.16	2.08	2.11	2.08	2.11	2.13	2.11
31.98	2.10	2.08	2.16	2.18	2.21	2.13	2.16	2.22	2.17	2.17	2.20
33.47	2.06	2.07	2.10	2.12	2.16	2.10	2.12	2.12	2.10	2.13	2.14
35.09	1.96	1.97	2.02	2.01	2.05	2.03	2.06	2.06	2.04	2.10	2.09
36.83	2.11	2.17	2.11	2.14	2.17	2.15	2.14	2.13	2.11	2.07	2.09
38.71	1.95	1.92	1.93	1.96	2.00	1.98	2.00	1.99	1.96	1.99	1.94
40.73	1.82	1.83	1.87	1.91	1.90	1.89	1.88	1.92	1.89	1.95	1.93
42.92	1.85	1.86	1.91	1.85	1.86	1.88	1.83	1.89	1.91	1.91	1.88
45.27	1.85	1.85	1.89	1.87	1.88	1.90	1.93	1.92	1.93	1.94	1.88
47.81	1.86	1.92	1.92	1.93	1.99	1.96	1.93	1.96	1.97	1.99	1.93
50.55	1.89	1.85	1.94	1.91	1.95	1.92	1.91	1.94	1.93	1.94	1.94
53.51	1.85	1.80	1.87	1.87	1.87	1.88	1.82	1.85	1.82	1.85	1.89

56.69	1.90	1.96	1.95	2.00	1.98	1.97	1.92	1.91	1.88	1.88	1.93
60.13	1.92	1.93	1.97	1.96	1.95	1.95	1.98	1.97	1.96	1.96	1.95
63.84	1.83	1.82	1.84	1.86	1.83	1.85	1.84	1.86	1.89	1.84	1.83
67.83	1.88	1.90	1.92	1.97	2.00	1.92	1.89	1.92	1.90	1.96	1.90
72.15	1.87	1.85	1.91	1.93	1.92	1.96	1.93	1.91	1.94	1.93	1.90
76.80	1.91	1.94	2.01	1.93	1.92	1.94	1.93	1.87	1.89	1.89	1.89
81.81	1.77	1.83	1.89	1.87	1.84	1.85	1.82	1.82	1.85	1.83	1.75
87.22	1.70	1.72	1.77	1.77	1.75	1.77	1.76	1.73	1.73	1.73	1.76
93.06	1.88	1.86	1.87	1.87	1.89	1.86	1.87	1.84	1.81	1.79	1.79
99.35	1.82	1.84	1.85	1.83	1.82	1.89	1.82	1.82	1.84	1.82	1.81
106.13	1.76	1.82	1.80	1.80	1.79	1.83	1.80	1.80	1.80	1.80	1.79
113.45	1.80	1.80	1.81	1.82	1.83	1.81	1.78	1.78	1.78	1.77	1.74
121.34	1.91	1.94	1.96	2.00	1.91	1.87	1.95	1.92	1.91	1.88	1.83
129.86	1.82	1.86	1.84	1.86	1.84	1.86	1.85	1.82	1.81	1.77	1.77
139.04	1.73	1.75	1.75	1.75	1.78	1.81	1.76	1.76	1.73	1.73	1.79
148.94	1.98	1.97	1.94	1.95	1.95	1.95	1.94	1.91	1.88	1.87	1.82
159.62	1.85	1.80	1.82	1.86	1.82	1.79	1.76	1.77	1.73	1.73	1.74
171.14	1.88	1.85	1.85	1.84	1.81	1.80	1.80	1.78	1.81	1.78	1.81
183.56	1.86	1.91	1.92	1.85	1.87	1.92	1.90	1.86	1.85	1.85	1.81
196.96	1.98	1.96	1.96	1.94	1.92	1.91	1.88	1.87	1.89	1.86	1.77
211.41	1.90	1.91	1.89	1.90	1.87	1.87	1.85	1.87	1.81	1.79	1.79
226.99	1.99	2.03	1.95	1.94	1.97	1.96	1.94	1.94	1.89	1.88	1.80
243.80	1.97	1.99	1.97	1.97	1.99	1.95	1.92	1.95	1.89	1.86	1.84
261.93	2.00	2.03	2.04	2.06	1.99	1.99	1.94	1.96	1.91	1.91	1.87
281.48	2.15	2.10	2.12	2.12	2.12	2.10	2.09	2.06	2.07	2.07	2.03
302.56	2.12	2.11	2.12	2.11	2.10	2.04	2.01	2.00	1.94	1.99	1.92
325.30	2.10	2.10	2.14	2.06	2.09	2.07	2.01	1.99	1.95	1.92	1.90
349.83	2.14	2.13	2.11	2.09	2.03	2.07	2.01	1.99	2.00	1.98	1.92
376.28	2.28	2.24	2.27	2.23	2.24	2.20	2.16	2.18	2.10	2.14	2.14
404.81	2.23	2.20	2.16	2.16	2.13	2.03	2.02	2.01	1.97	1.95	1.89
435.58	2.35	2.29	2.27	2.27	2.27	2.27	2.21	2.22	2.15	2.16	2.14
468.76	2.39	2.43	2.36	2.32	2.26	2.27	2.23	2.23	2.18	2.18	2.10
504.55	2.55	2.54	2.53	2.48	2.49	2.44	2.39	2.38	2.34	2.30	2.26
543.15	2.58	2.57	2.53	2.49	2.48	2.40	2.34	2.30	2.33	2.28	2.27
584.78	2.66	2.61	2.59	2.60	2.49	2.48	2.47	2.47	2.41	2.38	2.27
629.68	2.64	2.62	2.56	2.53	2.48	2.47	2.47	2.37	2.31	2.29	2.26
678.10	2.93	2.87	2.90	2.84	2.80	2.79	2.75	2.75	2.70	2.61	2.63
730.33	2.98	2.97	2.93	2.89	2.82	2.74	2.73	2.64	2.62	2.56	2.51
786.65	3.13	3.07	3.03	2.98	2.96	2.92	2.83	2.74	2.67	2.63	2.56
847.40	3.30	3.23	3.21	3.07	3.09	3.03	2.98	2.94	2.84	2.81	2.74
912.92	3.41	3.34	3.27	3.26	3.18	3.09	3.06	3.02	2.97	2.93	2.85
983.58	3.55	3.50	3.42	3.37	3.36	3.31	3.24	3.18	3.22	3.12	3.01
1059.78	3.52	3.50	3.46	3.34	3.33	3.28	3.20	3.11	3.08	3.04	2.96
1141.98	3.71	3.63	3.60	3.55	3.47	3.45	3.38	3.31	3.23	3.17	3.15
1230.62	3.62	3.59	3.54	3.53	3.45	3.37	3.29	3.25	3.21	3.17	3.09
1326.23	3.87	3.81	3.77	3.64	3.59	3.55	3.54	3.48	3.43	3.33	3.22
1429.34	4.13	4.06	3.97	3.92	3.89	3.80	3.69	3.63	3.54	3.47	3.44
1540.54	4.14	4.05	3.96	3.91	3.86	3.81	3.76	3.63	3.55	3.48	3.45
1660.48	4.30	4.29	4.23	4.16	4.06	3.99	3.89	3.81	3.75	3.69	3.60
1789.83	4.45	4.41	4.30	4.27	4.17	4.13	4.02	3.94	3.84	3.85	3.74

1929.34	4.43	4.36	4.32	4.22	4.15	4.07	4.00	3.93	3.84	3.73	3.64
2079.80	4.52	4.44	4.37	4.32	4.19	4.11	4.02	3.93	3.88	3.79	3.71
2242.08	4.70	4.59	4.58	4.48	4.37	4.31	4.20	4.11	4.02	4.01	3.92
2417.10	4.77	4.73	4.58	4.49	4.41	4.33	4.25	4.20	4.04	3.98	3.89
2605.85	4.68	4.63	4.56	4.50	4.41	4.29	4.26	4.17	4.09	4.03	3.89
2809.43	4.71	4.56	4.53	4.43	4.32	4.17	4.11	4.08	3.96	3.92	3.80
3028.99	4.96	4.88	4.81	4.73	4.67	4.60	4.46	4.34	4.26	4.22	4.14
3265.79	4.94	4.87	4.79	4.71	4.60	4.51	4.42	4.35	4.30	4.17	4.02
3521.18	4.92	4.79	4.71	4.62	4.57	4.48	4.38	4.31	4.25	4.16	4.08
3796.62	4.99	4.91	4.85	4.76	4.70	4.58	4.48	4.43	4.32	4.30	4.17

Wavelength (nm) / Time (ps)	646.08	647.49	648.90	650.30	651.71	653.12	654.53	655.94	657.35	658.76	660.16
-3.28	0.07	0.08	0.06	0.09	0.08	0.02	0.05	0.03	0.04	0.03	0.04
-2.78	0.07	0.01	0.03	0.04	0.04	0.06	0.02	0.01	0.02	0.11	0.07
-2.28	0.09	0.04	0.07	0.04	0.01	0.00	0.03	-0.01	0.03	-0.03	0.00
-1.78	-0.14	-0.12	-0.18	-0.21	-0.12	-0.09	-0.06	-0.06	-0.11	-0.12	-0.13
-1.28	0.03	0.02	0.00	0.00	-0.01	-0.02	0.03	0.00	-0.04	0.00	0.01
-0.78	0.10	0.09	0.13	0.12	0.12	0.09	0.11	0.09	0.10	0.07	0.03
-0.28	-0.01	-0.04	-0.01	0.02	0.01	0.01	-0.04	-0.03	-0.02	-0.01	-0.05
0.22	-0.15	-0.06	-0.06	-0.04	-0.09	-0.02	-0.12	-0.04	0.01	-0.01	-0.01
0.32	0.01	0.01	0.01	-0.01	0.04	0.03	0.00	0.02	0.04	0.02	0.06
0.42	-0.08	-0.04	-0.06	-0.06	-0.09	-0.08	-0.01	-0.02	-0.07	-0.06	-0.02
0.52	0.04	0.00	0.08	0.07	0.10	0.03	-0.01	-0.01	-0.02	-0.05	-0.01
0.62	0.01	0.01	-0.01	-0.06	-0.08	-0.03	-0.06	-0.08	0.00	-0.12	-0.10
0.72	-0.21	-0.21	-0.16	-0.16	-0.13	-0.17	-0.20	-0.16	-0.12	-0.10	-0.03
0.77	-0.07	-0.13	-0.12	-0.10	-0.10	-0.09	-0.15	-0.13	-0.15	-0.12	-0.12
0.82	-0.09	-0.12	-0.07	-0.03	-0.08	-0.12	-0.05	-0.09	-0.04	-0.04	0.00
0.87	-0.09	-0.05	-0.04	-0.02	-0.03	-0.03	-0.02	0.01	0.03	0.03	0.00
0.92	-0.01	0.02	0.00	0.01	-0.02	0.01	0.04	0.04	0.07	0.07	0.06
0.97	-0.01	0.03	-0.03	0.03	-0.03	0.03	0.00	0.06	0.06	-0.01	-0.01
1.02	0.08	0.09	0.08	0.10	0.11	0.11	0.11	0.09	0.14	0.12	0.06
1.07	-0.13	-0.07	-0.11	-0.06	-0.08	-0.10	-0.04	-0.06	-0.03	-0.08	-0.03
1.12	0.11	0.10	0.15	0.15	0.11	0.14	0.11	0.12	0.10	0.11	0.06
1.17	0.19	0.20	0.13	0.13	0.11	0.07	0.10	0.04	0.07	-0.01	0.04
1.22	0.04	-0.04	0.02	0.00	-0.03	0.03	-0.03	0.02	0.09	0.02	0.01
1.27	-0.10	-0.10	-0.06	-0.08	-0.05	-0.09	-0.04	-0.05	-0.04	0.03	-0.01
1.32	0.03	0.00	-0.01	0.05	0.05	0.06	0.03	0.03	0.02	0.01	-0.01
1.37	0.09	0.09	0.02	0.03	0.01	-0.03	-0.08	-0.02	-0.03	-0.03	-0.07
1.42	0.05	-0.03	-0.01	0.03	0.03	-0.01	-0.06	-0.03	-0.01	-0.01	0.04
1.47	0.07	0.12	0.10	0.12	0.08	0.12	0.10	0.13	0.08	0.06	0.06
1.52	0.06	0.02	0.02	0.09	0.10	0.05	-0.01	-0.01	0.02	0.02	0.04
1.57	-0.11	-0.16	-0.13	-0.16	-0.17	-0.13	-0.07	-0.14	-0.08	-0.13	-0.10
1.62	0.91	0.85	0.69	0.68	0.67	0.55	0.50	0.44	0.46	0.43	0.40
1.67	5.99	5.89	5.71	5.52	5.33	5.06	4.92	4.76	4.63	4.45	4.34
1.72	9.39	9.31	9.24	9.19	9.04	8.97	8.79	8.63	8.56	8.36	8.20
1.77	9.42	9.40	9.37	9.34	9.34	9.30	9.27	9.16	9.07	9.02	8.95
1.82	8.40	8.34	8.40	8.42	8.46	8.45	8.44	8.39	8.42	8.39	8.43
1.87	7.51	7.57	7.60	7.64	7.66	7.63	7.68	7.70	7.76	7.72	7.80

1.92	7.14	7.20	7.22	7.19	7.23	7.27	7.30	7.39	7.36	7.35	7.39
1.97	6.95	6.95	6.94	7.00	7.02	7.04	7.07	7.08	7.03	7.08	7.08
2.02	6.62	6.61	6.64	6.69	6.62	6.65	6.70	6.72	6.78	6.76	6.81
2.07	6.53	6.52	6.56	6.53	6.58	6.55	6.57	6.57	6.61	6.58	6.62
2.12	6.46	6.47	6.46	6.51	6.53	6.57	6.58	6.59	6.65	6.61	6.66
2.17	5.99	6.04	6.04	6.05	6.06	6.10	6.10	6.09	6.08	6.15	6.14
2.22	5.91	5.95	5.94	5.93	5.96	5.94	5.97	6.01	6.04	5.98	6.01
2.27	5.71	5.74	5.80	5.86	5.82	5.80	5.78	5.81	5.80	5.86	5.83
2.32	5.70	5.66	5.69	5.73	5.71	5.71	5.68	5.71	5.64	5.64	5.63
2.37	5.75	5.75	5.79	5.79	5.79	5.80	5.77	5.80	5.82	5.83	5.80
2.42	5.63	5.61	5.59	5.57	5.56	5.63	5.62	5.64	5.57	5.50	5.53
2.47	5.35	5.40	5.43	5.42	5.44	5.34	5.36	5.33	5.38	5.29	5.33
2.52	5.32	5.32	5.31	5.37	5.31	5.27	5.34	5.34	5.35	5.26	5.31
2.57	5.27	5.23	5.27	5.26	5.23	5.22	5.19	5.17	5.17	5.13	5.17
2.62	5.03	5.05	5.05	5.05	5.04	5.03	4.99	5.02	5.01	5.02	5.01
2.67	5.05	5.13	5.11	5.08	5.06	5.11	5.12	5.12	5.09	5.00	4.99
2.72	4.93	4.91	4.92	4.93	4.95	4.95	4.88	4.94	4.90	4.92	4.92
2.77	4.87	4.85	4.89	4.92	4.91	4.89	4.85	4.88	4.88	4.92	4.90
2.82	4.84	4.91	4.90	4.89	4.93	4.93	4.86	4.92	4.88	4.83	4.78
2.87	4.84	4.83	4.80	4.83	4.82	4.82	4.82	4.88	4.86	4.84	4.77
2.92	4.75	4.77	4.79	4.78	4.74	4.72	4.73	4.70	4.68	4.64	4.66
2.97	4.72	4.72	4.75	4.75	4.76	4.74	4.72	4.67	4.72	4.70	4.71
3.02	4.76	4.72	4.76	4.75	4.73	4.67	4.69	4.70	4.68	4.68	4.64
3.07	4.74	4.78	4.76	4.83	4.82	4.79	4.78	4.78	4.75	4.73	4.74
3.12	4.60	4.64	4.65	4.68	4.69	4.62	4.62	4.63	4.70	4.65	4.67
3.17	4.59	4.64	4.70	4.65	4.63	4.59	4.60	4.56	4.55	4.55	4.49
3.22	4.54	4.50	4.53	4.47	4.44	4.50	4.48	4.49	4.46	4.42	4.41
3.27	4.39	4.41	4.37	4.45	4.39	4.36	4.33	4.37	4.40	4.35	4.34
3.32	4.42	4.49	4.47	4.49	4.49	4.49	4.54	4.49	4.53	4.46	4.53
3.37	4.39	4.42	4.44	4.44	4.44	4.45	4.45	4.47	4.44	4.41	4.40
3.42	4.33	4.35	4.36	4.31	4.31	4.28	4.36	4.36	4.35	4.30	4.32
3.47	4.47	4.51	4.50	4.49	4.45	4.45	4.43	4.41	4.39	4.38	4.36
3.52	4.31	4.34	4.37	4.41	4.32	4.30	4.26	4.28	4.26	4.23	4.24
3.57	4.25	4.25	4.22	4.23	4.20	4.24	4.26	4.21	4.18	4.12	4.21
3.62	4.04	4.10	4.11	4.11	4.13	4.04	4.11	4.09	4.10	4.04	4.02
3.67	4.10	4.14	4.12	4.14	4.12	4.06	4.09	4.11	4.12	4.10	4.01
3.72	4.08	4.12	4.12	4.15	4.11	4.09	4.06	4.08	4.04	4.03	4.01
3.92	4.05	4.10	4.10	4.21	4.12	4.10	4.06	4.07	4.11	4.10	4.08
4.12	3.92	3.94	4.00	3.97	3.94	3.92	3.88	3.92	3.86	3.79	3.81
4.32	3.70	3.73	3.75	3.74	3.74	3.69	3.75	3.75	3.77	3.76	3.78
4.52	3.68	3.70	3.65	3.65	3.67	3.67	3.66	3.68	3.64	3.62	3.65
4.72	3.67	3.66	3.72	3.68	3.67	3.67	3.67	3.61	3.64	3.61	3.64
4.92	3.56	3.52	3.56	3.54	3.57	3.54	3.57	3.56	3.56	3.57	3.51
5.12	3.35	3.34	3.37	3.42	3.42	3.43	3.34	3.32	3.36	3.39	3.43
5.32	3.36	3.41	3.42	3.47	3.45	3.46	3.46	3.38	3.40	3.41	3.45
5.52	3.22	3.24	3.24	3.24	3.25	3.23	3.25	3.24	3.26	3.28	3.29
5.72	3.28	3.27	3.28	3.35	3.27	3.30	3.28	3.25	3.24	3.24	3.28
5.92	3.41	3.37	3.44	3.39	3.37	3.36	3.39	3.38	3.43	3.35	3.33
6.12	3.29	3.27	3.28	3.24	3.27	3.20	3.22	3.26	3.23	3.22	3.14
6.32	3.25	3.31	3.31	3.30	3.28	3.24	3.25	3.26	3.21	3.29	3.23

6.52	3.11	3.09	3.12	3.15	3.13	3.10	3.09	3.08	3.10	3.03	3.05
6.72	3.08	3.09	3.09	3.08	3.09	3.09	3.11	3.08	3.12	3.10	3.08
6.92	3.00	3.01	3.08	3.04	3.04	2.98	2.97	2.94	3.02	3.04	2.99
7.12	3.07	3.09	3.08	3.06	3.07	3.10	3.16	3.11	3.11	3.07	3.10
7.32	3.04	3.04	3.03	2.99	3.05	2.97	2.98	2.97	2.96	2.97	2.97
7.52	3.02	2.99	3.03	3.08	3.05	3.00	2.93	2.94	2.97	3.00	3.00
7.72	3.06	3.09	3.14	3.13	3.10	3.03	3.08	3.13	3.14	3.06	3.09
7.92	2.92	2.95	2.94	2.95	2.92	2.91	2.94	2.88	2.86	2.85	2.85
8.12	2.84	2.85	2.88	2.85	2.81	2.81	2.77	2.80	2.83	2.81	2.83
8.32	2.91	2.85	2.88	2.81	2.89	2.89	2.85	2.82	2.82	2.79	2.83
8.52	2.94	2.86	2.87	2.88	2.91	2.89	2.88	2.80	2.85	2.80	2.86
8.72	3.00	3.03	3.03	3.01	2.98	2.97	2.92	2.99	2.97	2.96	2.99
8.92	3.03	3.03	3.08	3.09	3.04	3.00	3.00	3.02	3.08	3.00	2.99
9.12	2.92	2.91	2.89	2.84	2.78	2.79	2.88	2.83	2.81	2.78	2.80
9.32	2.81	2.77	2.72	2.76	2.79	2.76	2.74	2.75	2.71	2.75	2.80
9.52	2.87	2.89	2.92	2.90	2.91	2.90	2.85	2.88	2.89	2.86	2.88
9.72	2.66	2.71	2.66	2.68	2.64	2.70	2.70	2.71	2.71	2.69	2.71
9.92	2.56	2.57	2.58	2.63	2.59	2.52	2.50	2.49	2.51	2.50	2.52
10.12	2.88	2.91	2.91	2.88	2.84	2.89	2.86	2.91	2.83	2.84	2.84
10.32	2.64	2.66	2.69	2.69	2.69	2.66	2.59	2.60	2.61	2.61	2.59
10.52	2.82	2.79	2.75	2.77	2.76	2.74	2.70	2.67	2.68	2.71	2.71
10.72	2.61	2.67	2.71	2.69	2.70	2.63	2.65	2.70	2.71	2.71	2.77
10.92	2.60	2.66	2.63	2.59	2.58	2.59	2.60	2.57	2.63	2.63	2.59
11.12	2.62	2.63	2.62	2.67	2.68	2.60	2.60	2.67	2.67	2.66	2.73
11.32	2.57	2.58	2.61	2.55	2.57	2.56	2.55	2.52	2.57	2.56	2.57
11.52	2.57	2.61	2.61	2.57	2.57	2.54	2.53	2.57	2.57	2.60	2.59
11.72	2.57	2.61	2.63	2.63	2.60	2.53	2.55	2.50	2.53	2.53	2.52
11.92	2.70	2.68	2.68	2.74	2.74	2.69	2.67	2.74	2.77	2.78	2.76
12.12	2.72	2.83	2.76	2.81	2.72	2.80	2.78	2.82	2.83	2.78	2.78
12.32	2.68	2.73	2.66	2.65	2.68	2.69	2.67	2.63	2.63	2.67	2.64
12.52	2.68	2.67	2.62	2.66	2.57	2.59	2.54	2.55	2.56	2.51	2.52
12.72	2.39	2.44	2.42	2.36	2.37	2.34	2.40	2.41	2.40	2.37	2.38
12.92	2.49	2.50	2.53	2.54	2.49	2.47	2.49	2.44	2.49	2.50	2.52
13.12	2.52	2.55	2.52	2.49	2.50	2.47	2.50	2.48	2.45	2.41	2.49
13.32	2.64	2.63	2.62	2.62	2.65	2.61	2.60	2.65	2.66	2.64	2.65
13.52	2.44	2.44	2.50	2.42	2.39	2.46	2.44	2.42	2.43	2.42	2.47
13.72	2.44	2.46	2.48	2.45	2.45	2.44	2.45	2.37	2.41	2.44	2.41
13.92	2.60	2.57	2.59	2.56	2.58	2.53	2.51	2.52	2.56	2.58	2.59
14.00	2.56	2.56	2.58	2.58	2.56	2.57	2.53	2.49	2.49	2.50	2.50
14.08	2.43	2.41	2.39	2.47	2.42	2.42	2.39	2.43	2.43	2.43	2.44
14.17	2.55	2.48	2.46	2.53	2.45	2.46	2.42	2.39	2.48	2.44	2.46
14.27	2.63	2.66	2.59	2.55	2.53	2.54	2.52	2.53	2.46	2.43	2.46
14.38	2.47	2.44	2.45	2.43	2.46	2.40	2.44	2.44	2.45	2.43	2.44
14.49	2.47	2.46	2.47	2.42	2.42	2.40	2.45	2.47	2.46	2.44	2.41
14.61	2.60	2.61	2.54	2.53	2.55	2.54	2.51	2.51	2.48	2.50	2.55
14.75	2.55	2.51	2.53	2.52	2.53	2.49	2.48	2.55	2.52	2.51	2.48
14.89	2.45	2.42	2.45	2.47	2.44	2.42	2.43	2.40	2.41	2.38	2.39
15.05	2.46	2.49	2.48	2.46	2.45	2.40	2.43	2.44	2.43	2.37	2.40
15.21	2.41	2.41	2.42	2.39	2.38	2.36	2.37	2.38	2.40	2.38	2.40
15.39	2.29	2.27	2.26	2.22	2.23	2.25	2.25	2.22	2.24	2.24	2.23

15.59	2.50	2.58	2.54	2.50	2.51	2.48	2.46	2.51	2.54	2.53	2.50
15.80	2.48	2.45	2.45	2.43	2.42	2.45	2.43	2.43	2.39	2.47	2.40
16.02	2.34	2.31	2.34	2.34	2.31	2.29	2.31	2.23	2.26	2.23	2.23
16.27	2.39	2.35	2.29	2.37	2.34	2.26	2.31	2.24	2.31	2.30	2.30
16.53	2.48	2.48	2.44	2.43	2.42	2.45	2.41	2.38	2.35	2.33	2.37
16.81	2.54	2.47	2.50	2.47	2.49	2.48	2.48	2.46	2.51	2.42	2.44
17.12	2.38	2.36	2.40	2.45	2.42	2.37	2.38	2.39	2.43	2.37	2.39
17.45	2.33	2.41	2.37	2.36	2.33	2.30	2.25	2.29	2.27	2.27	2.25
17.81	2.38	2.38	2.37	2.38	2.41	2.39	2.34	2.35	2.35	2.37	2.42
18.19	2.37	2.35	2.37	2.40	2.37	2.29	2.27	2.37	2.41	2.39	2.43
18.60	2.32	2.31	2.35	2.37	2.36	2.32	2.33	2.38	2.35	2.34	2.34
19.05	2.40	2.44	2.45	2.45	2.42	2.40	2.43	2.42	2.45	2.39	2.41
19.53	2.45	2.45	2.51	2.48	2.45	2.42	2.41	2.42	2.44	2.41	2.44
20.05	2.42	2.43	2.46	2.48	2.44	2.45	2.39	2.39	2.41	2.41	2.39
20.61	2.27	2.26	2.25	2.27	2.22	2.24	2.17	2.18	2.19	2.18	2.12
21.22	2.24	2.23	2.14	2.20	2.20	2.21	2.22	2.20	2.20	2.15	2.24
21.87	2.39	2.39	2.35	2.34	2.32	2.33	2.30	2.26	2.26	2.24	2.22
22.57	2.26	2.31	2.26	2.19	2.24	2.20	2.19	2.18	2.23	2.16	2.14
23.33	2.23	2.18	2.18	2.26	2.26	2.27	2.16	2.20	2.22	2.17	2.26
24.15	2.11	2.11	2.11	2.09	2.05	2.01	2.09	2.05	2.11	2.07	2.09
25.03	2.18	2.19	2.14	2.16	2.18	2.20	2.16	2.17	2.18	2.16	2.18
25.98	2.28	2.31	2.35	2.32	2.27	2.22	2.27	2.28	2.27	2.23	2.28
27.01	2.23	2.20	2.21	2.23	2.21	2.21	2.23	2.19	2.19	2.13	2.22
28.11	2.20	2.13	2.12	2.09	2.08	2.08	2.05	2.10	2.04	2.08	2.06
29.30	2.13	2.12	2.16	2.16	2.10	2.08	2.09	2.11	2.10	2.15	2.15
30.59	2.14	2.07	2.07	2.08	2.07	2.02	2.09	2.03	2.04	2.03	2.08
31.98	2.14	2.14	2.19	2.16	2.19	2.14	2.20	2.16	2.11	2.16	2.22
33.47	2.10	2.05	2.08	2.09	2.10	2.07	2.08	2.10	2.05	2.07	2.05
35.09	2.07	2.10	2.09	2.08	2.07	2.10	2.09	2.07	2.07	2.02	2.11
36.83	2.09	2.07	2.03	2.04	2.07	2.04	2.01	1.99	1.98	2.05	2.04
38.71	1.96	1.96	1.90	1.93	1.89	1.92	1.89	1.87	1.88	1.88	1.91
40.73	1.91	1.86	1.88	1.91	1.93	1.86	1.90	1.86	1.85	1.85	1.87
42.92	1.88	1.89	1.88	1.91	1.90	1.92	1.91	1.89	1.90	1.87	1.92
45.27	1.91	1.96	1.96	1.92	1.95	1.95	1.95	1.99	1.96	1.91	1.92
47.81	1.92	1.95	1.93	1.92	1.85	1.86	1.84	1.86	1.89	1.86	1.87
50.55	1.91	1.87	1.88	1.90	1.88	1.91	1.88	1.95	1.91	1.86	1.92
53.51	1.82	1.81	1.74	1.82	1.83	1.82	1.78	1.76	1.74	1.78	1.88
56.69	1.88	1.85	1.84	1.77	1.84	1.83	1.85	1.88	1.84	1.84	1.91
60.13	1.91	1.96	1.98	1.95	1.85	1.85	1.87	1.93	1.88	1.84	1.85
63.84	1.79	1.76	1.80	1.80	1.80	1.77	1.77	1.78	1.74	1.75	1.78
67.83	1.89	1.84	1.87	1.89	1.86	1.79	1.77	1.82	1.84	1.87	1.87
72.15	1.89	1.90	1.91	1.88	1.92	1.89	1.84	1.87	1.88	1.85	1.84
76.80	1.88	1.84	1.84	1.86	1.85	1.85	1.80	1.81	1.91	1.86	1.83
81.81	1.78	1.74	1.77	1.82	1.77	1.74	1.76	1.78	1.72	1.72	1.76
87.22	1.73	1.71	1.68	1.73	1.69	1.70	1.68	1.65	1.73	1.67	1.67
93.06	1.80	1.80	1.77	1.72	1.74	1.76	1.77	1.82	1.78	1.79	1.81
99.35	1.81	1.77	1.78	1.79	1.71	1.73	1.73	1.72	1.72	1.68	1.70
106.13	1.71	1.70	1.71	1.77	1.75	1.76	1.73	1.72	1.71	1.69	1.71
113.45	1.76	1.70	1.74	1.75	1.75	1.74	1.67	1.68	1.71	1.64	1.70
121.34	1.80	1.83	1.77	1.77	1.72	1.74	1.74	1.77	1.79	1.69	1.77

129.86	1.78	1.77	1.69	1.69	1.64	1.61	1.62	1.64	1.68	1.63	1.65
139.04	1.78	1.73	1.75	1.73	1.71	1.70	1.67	1.64	1.63	1.61	1.64
148.94	1.82	1.82	1.81	1.82	1.74	1.74	1.74	1.79	1.75	1.75	1.75
159.62	1.69	1.65	1.65	1.68	1.68	1.68	1.62	1.66	1.75	1.70	1.68
171.14	1.80	1.71	1.76	1.69	1.74	1.76	1.73	1.73	1.68	1.61	1.69
183.56	1.80	1.78	1.73	1.78	1.78	1.72	1.71	1.66	1.69	1.66	1.73
196.96	1.78	1.76	1.77	1.82	1.72	1.64	1.69	1.68	1.71	1.70	1.71
211.41	1.72	1.71	1.71	1.65	1.66	1.65	1.64	1.64	1.65	1.65	1.65
226.99	1.78	1.76	1.75	1.70	1.65	1.68	1.57	1.61	1.60	1.60	1.59
243.80	1.80	1.80	1.83	1.76	1.72	1.73	1.71	1.69	1.67	1.57	1.60
261.93	1.86	1.81	1.84	1.79	1.80	1.76	1.76	1.74	1.78	1.70	1.76
281.48	2.01	2.01	2.00	1.95	1.91	1.95	1.91	1.89	1.86	1.82	1.81
302.56	1.89	1.81	1.82	1.78	1.77	1.75	1.69	1.72	1.72	1.64	1.72
325.30	1.89	1.84	1.84	1.84	1.83	1.81	1.77	1.78	1.79	1.77	1.77
349.83	1.87	1.87	1.89	1.87	1.86	1.86	1.76	1.77	1.80	1.78	1.72
376.28	2.11	1.96	1.99	1.94	1.95	1.98	1.88	1.88	1.85	1.75	1.79
404.81	1.79	1.76	1.73	1.71	1.71	1.66	1.62	1.56	1.53	1.54	1.56
435.58	2.10	2.11	2.03	1.99	1.97	1.99	1.94	1.91	1.90	1.86	1.88
468.76	2.07	2.00	2.01	2.03	1.99	1.92	1.84	1.79	1.82	1.78	1.79
504.55	2.14	2.15	2.11	2.08	2.05	2.00	1.94	1.93	1.90	1.88	1.86
543.15	2.18	2.18	2.10	2.13	2.06	2.04	2.05	2.00	1.95	1.90	1.94
584.78	2.26	2.27	2.19	2.20	2.10	2.07	2.06	2.00	2.04	2.00	1.93
629.68	2.22	2.18	2.14	2.11	2.05	1.99	2.00	1.91	1.91	1.90	1.83
678.10	2.51	2.49	2.41	2.37	2.29	2.24	2.25	2.19	2.20	2.13	2.10
730.33	2.46	2.38	2.37	2.32	2.29	2.20	2.18	2.13	2.13	2.08	2.07
786.65	2.49	2.46	2.40	2.35	2.27	2.20	2.15	2.11	2.08	2.05	2.02
847.40	2.70	2.68	2.58	2.52	2.45	2.42	2.39	2.39	2.27	2.22	2.21
912.92	2.78	2.70	2.61	2.59	2.54	2.52	2.43	2.45	2.39	2.25	2.28
983.58	3.01	2.92	2.88	2.79	2.77	2.75	2.69	2.60	2.58	2.49	2.47
1059.78	2.93	2.83	2.74	2.73	2.67	2.63	2.63	2.49	2.50	2.40	2.35
1141.98	3.09	2.97	2.92	2.90	2.86	2.83	2.75	2.70	2.63	2.57	2.56
1230.62	3.01	2.95	2.91	2.85	2.76	2.68	2.61	2.55	2.49	2.47	2.42
1326.23	3.17	3.14	3.10	3.01	2.95	2.86	2.81	2.77	2.76	2.61	2.59
1429.34	3.36	3.30	3.18	3.10	3.03	3.01	2.95	2.86	2.81	2.69	2.64
1540.54	3.40	3.33	3.18	3.02	3.04	2.99	2.90	2.82	2.71	2.61	2.58
1660.48	3.50	3.46	3.35	3.30	3.28	3.15	3.09	3.05	3.01	2.89	2.82
1789.83	3.65	3.56	3.48	3.40	3.32	3.27	3.18	3.15	3.02	2.98	2.88
1929.34	3.56	3.49	3.41	3.40	3.28	3.13	3.10	3.02	3.02	2.88	2.87
2079.80	3.60	3.52	3.43	3.41	3.32	3.23	3.14	3.06	3.04	2.94	2.89
2242.08	3.76	3.69	3.65	3.56	3.50	3.36	3.28	3.19	3.07	2.98	2.98
2417.10	3.80	3.73	3.62	3.55	3.47	3.40	3.30	3.22	3.22	3.09	3.07
2605.85	3.85	3.76	3.66	3.57	3.51	3.40	3.28	3.21	3.12	3.04	2.99
2809.43	3.69	3.57	3.50	3.49	3.44	3.29	3.20	3.22	3.13	3.10	3.01
3028.99	4.03	3.97	3.86	3.75	3.70	3.59	3.47	3.45	3.34	3.29	3.19
3265.79	3.91	3.83	3.76	3.68	3.60	3.48	3.35	3.32	3.17	3.13	3.08
3521.18	3.96	3.83	3.81	3.69	3.61	3.54	3.37	3.31	3.24	3.10	3.04
3796.62	4.05	4.00	3.93	3.85	3.69	3.63	3.51	3.39	3.35	3.25	3.18

Time (ps) \ Wavelength (nm)	661.57	662.98	664.39	665.80	667.21	668.62	670.02
-3.28	0.03	0.06	0.13	0.04	0.07	0.02	0.04
-2.78	0.11	0.07	0.01	0.05	0.11	0.10	0.16
-2.28	-0.03	0.00	0.03	0.00	-0.04	0.01	-0.04
-1.78	-0.11	-0.11	-0.10	-0.13	-0.17	-0.15	-0.18
-1.28	0.02	-0.02	-0.02	0.00	-0.04	0.03	0.04
-0.78	0.05	0.02	0.01	0.06	0.05	0.03	-0.01
-0.28	-0.02	-0.05	-0.02	0.03	0.01	-0.01	-0.01
0.22	0.01	0.01	0.01	0.01	-0.06	0.00	0.03
0.32	0.00	0.00	-0.02	0.01	0.07	-0.02	-0.04
0.42	-0.05	0.01	-0.03	-0.08	0.00	0.00	-0.01
0.52	-0.02	-0.04	-0.02	-0.07	-0.08	-0.11	-0.09
0.62	-0.14	-0.11	-0.09	-0.14	-0.16	-0.18	-0.14
0.72	-0.06	-0.01	-0.04	0.00	0.00	0.02	0.00
0.77	-0.19	-0.15	-0.19	-0.12	-0.12	-0.14	-0.19
0.82	-0.03	-0.02	0.03	-0.05	-0.06	-0.04	-0.06
0.87	0.04	0.02	0.03	0.02	0.08	0.08	0.06
0.92	0.03	0.04	-0.02	-0.01	-0.01	-0.02	0.01
0.97	-0.03	0.01	0.03	0.05	-0.01	-0.02	-0.05
1.02	0.04	0.08	0.07	0.03	0.01	-0.05	-0.06
1.07	-0.05	-0.03	-0.01	-0.07	-0.07	-0.08	-0.09
1.12	0.13	0.05	0.06	0.09	0.07	0.09	0.19
1.17	0.05	0.04	0.05	-0.03	-0.04	-0.02	-0.06
1.22	0.02	0.08	0.02	0.00	0.01	-0.02	-0.05
1.27	0.01	0.03	0.01	0.03	-0.01	0.01	-0.09
1.32	-0.03	-0.05	-0.03	-0.09	-0.07	-0.05	-0.08
1.37	-0.09	-0.06	-0.03	-0.06	-0.08	-0.07	-0.09
1.42	-0.04	0.00	0.01	-0.06	-0.02	-0.02	0.00
1.47	0.07	0.08	0.05	-0.01	-0.07	-0.02	0.04
1.52	0.02	0.02	-0.01	-0.01	0.04	0.00	-0.03
1.57	-0.08	-0.09	-0.06	-0.11	-0.09	-0.11	-0.11
1.62	0.40	0.42	0.40	0.35	0.35	0.27	0.29
1.67	4.12	4.00	3.89	3.81	3.68	3.58	3.50
1.72	7.98	7.85	7.66	7.49	7.28	7.19	7.15
1.77	8.85	8.79	8.70	8.63	8.55	8.52	8.54
1.82	8.35	8.35	8.27	8.31	8.35	8.39	8.53
1.87	7.78	7.84	7.90	7.86	7.86	8.00	8.14
1.92	7.46	7.51	7.51	7.51	7.55	7.67	7.83
1.97	7.10	7.23	7.26	7.27	7.37	7.46	7.64
2.02	6.85	6.89	6.97	6.99	7.00	7.12	7.34
2.07	6.67	6.71	6.73	6.73	6.78	6.85	6.96
2.12	6.72	6.75	6.77	6.74	6.75	6.89	7.00
2.17	6.14	6.19	6.21	6.21	6.25	6.35	6.49
2.22	5.97	6.02	6.03	6.03	6.06	6.11	6.24
2.27	5.79	5.82	5.87	5.96	5.98	6.07	6.08
2.32	5.64	5.66	5.70	5.70	5.69	5.79	5.93
2.37	5.82	5.84	5.80	5.79	5.86	5.95	6.01
2.42	5.53	5.58	5.58	5.59	5.63	5.62	5.79

2.47	5.33	5.31	5.29	5.31	5.33	5.38	5.42
2.52	5.29	5.36	5.33	5.31	5.29	5.40	5.47
2.57	5.16	5.14	5.15	5.20	5.22	5.30	5.39
2.62	4.98	4.98	5.06	5.05	5.09	5.13	5.26
2.67	4.98	5.00	4.96	4.94	4.95	4.97	5.04
2.72	4.87	4.92	4.98	4.94	4.97	5.00	5.09
2.77	4.94	4.96	4.89	4.90	4.90	4.90	5.02
2.82	4.81	4.82	4.78	4.82	4.79	4.86	4.93
2.87	4.80	4.82	4.83	4.84	4.89	4.90	4.96
2.92	4.68	4.69	4.62	4.62	4.64	4.73	4.77
2.97	4.67	4.71	4.69	4.64	4.64	4.75	4.83
3.02	4.62	4.64	4.68	4.67	4.62	4.67	4.75
3.07	4.69	4.75	4.72	4.74	4.69	4.72	4.81
3.12	4.65	4.64	4.65	4.70	4.72	4.78	4.84
3.17	4.53	4.55	4.57	4.52	4.50	4.51	4.64
3.22	4.42	4.44	4.44	4.37	4.37	4.40	4.53
3.27	4.31	4.33	4.32	4.38	4.38	4.43	4.45
3.32	4.51	4.50	4.51	4.50	4.49	4.57	4.66
3.37	4.34	4.37	4.42	4.42	4.35	4.44	4.47
3.42	4.35	4.35	4.39	4.36	4.32	4.36	4.47
3.47	4.33	4.32	4.34	4.30	4.32	4.38	4.43
3.52	4.21	4.28	4.22	4.26	4.26	4.30	4.41
3.57	4.23	4.21	4.18	4.12	4.18	4.18	4.29
3.62	4.06	4.07	4.13	4.15	4.10	4.09	4.20
3.67	4.09	4.06	4.09	4.03	4.02	4.10	4.27
3.72	4.05	3.99	4.09	4.09	4.07	4.11	4.21
3.92	4.05	4.07	4.08	4.10	4.08	4.09	4.15
4.12	3.84	3.80	3.82	3.72	3.74	3.77	3.83
4.32	3.75	3.80	3.77	3.80	3.82	3.89	4.00
4.52	3.67	3.64	3.67	3.66	3.66	3.72	3.82
4.72	3.67	3.66	3.67	3.60	3.63	3.73	3.78
4.92	3.56	3.55	3.62	3.52	3.57	3.61	3.68
5.12	3.39	3.38	3.37	3.39	3.44	3.46	3.55
5.32	3.41	3.40	3.42	3.37	3.35	3.44	3.55
5.52	3.27	3.27	3.30	3.36	3.35	3.35	3.44
5.72	3.25	3.31	3.30	3.38	3.29	3.37	3.44
5.92	3.27	3.32	3.36	3.32	3.34	3.30	3.32
6.12	3.17	3.21	3.25	3.26	3.22	3.26	3.26
6.32	3.24	3.23	3.22	3.19	3.18	3.20	3.25
6.52	3.04	3.06	3.03	3.03	3.04	3.06	3.14
6.72	3.05	3.08	3.09	3.09	3.16	3.17	3.26
6.92	3.02	3.05	3.01	3.04	3.15	3.15	3.29
7.12	3.13	3.15	3.18	3.14	3.09	3.16	3.24
7.32	2.99	2.99	3.07	3.08	3.10	3.14	3.27
7.52	2.95	2.96	2.98	3.00	3.01	3.04	3.09
7.72	3.07	3.11	3.07	3.14	3.12	3.18	3.18
7.92	2.86	2.86	2.89	2.92	2.95	3.01	3.02
8.12	2.83	2.90	2.89	2.89	2.89	2.98	3.07
8.32	2.94	2.92	2.91	2.86	2.89	2.99	3.04
8.52	2.87	2.86	2.92	2.83	2.90	3.00	3.04

8.72	3.00	3.00	3.00	3.05	3.05	3.12	3.20
8.92	2.99	2.96	2.95	2.96	2.98	2.97	3.02
9.12	2.82	2.84	2.87	2.82	2.85	2.91	3.02
9.32	2.78	2.84	2.85	2.82	2.79	2.89	2.98
9.52	2.84	2.92	2.96	2.94	2.97	2.99	3.02
9.72	2.69	2.75	2.79	2.79	2.81	2.84	2.87
9.92	2.51	2.51	2.51	2.53	2.55	2.57	2.64
10.12	2.78	2.78	2.77	2.78	2.79	2.88	2.93
10.32	2.58	2.62	2.57	2.59	2.60	2.59	2.65
10.52	2.70	2.63	2.73	2.71	2.75	2.75	2.78
10.72	2.77	2.79	2.79	2.85	2.86	2.87	2.92
10.92	2.61	2.59	2.62	2.69	2.75	2.80	2.86
11.12	2.71	2.68	2.70	2.76	2.78	2.76	2.83
11.32	2.55	2.62	2.61	2.64	2.64	2.69	2.81
11.52	2.59	2.64	2.62	2.64	2.67	2.74	2.78
11.72	2.57	2.51	2.55	2.56	2.61	2.63	2.78
11.92	2.78	2.77	2.79	2.80	2.88	2.88	2.84
12.12	2.83	2.77	2.82	2.82	2.85	2.90	2.92
12.32	2.63	2.65	2.70	2.66	2.64	2.71	2.74
12.52	2.53	2.56	2.63	2.59	2.61	2.65	2.63
12.72	2.40	2.43	2.43	2.42	2.45	2.55	2.62
12.92	2.53	2.52	2.55	2.57	2.63	2.63	2.70
13.12	2.46	2.54	2.60	2.55	2.54	2.56	2.62
13.32	2.65	2.66	2.67	2.67	2.70	2.86	2.89
13.52	2.50	2.49	2.52	2.48	2.50	2.59	2.69
13.72	2.46	2.44	2.42	2.44	2.48	2.51	2.53
13.92	2.53	2.55	2.59	2.62	2.61	2.66	2.82
14.00	2.59	2.57	2.61	2.58	2.55	2.64	2.79
14.08	2.45	2.48	2.47	2.49	2.49	2.59	2.72
14.17	2.48	2.45	2.48	2.47	2.50	2.54	2.66
14.27	2.44	2.56	2.53	2.52	2.45	2.57	2.61
14.38	2.47	2.45	2.52	2.47	2.47	2.54	2.63
14.49	2.42	2.46	2.48	2.50	2.48	2.53	2.58
14.61	2.53	2.54	2.61	2.53	2.55	2.57	2.62
14.75	2.50	2.46	2.50	2.41	2.51	2.54	2.63
14.89	2.43	2.43	2.43	2.40	2.45	2.48	2.59
15.05	2.42	2.37	2.42	2.38	2.42	2.41	2.51
15.21	2.41	2.46	2.48	2.45	2.49	2.50	2.61
15.39	2.23	2.33	2.39	2.36	2.39	2.41	2.50
15.59	2.46	2.46	2.50	2.52	2.60	2.55	2.70
15.80	2.47	2.43	2.47	2.48	2.53	2.60	2.68
16.02	2.31	2.30	2.30	2.25	2.32	2.37	2.48
16.27	2.33	2.35	2.34	2.40	2.43	2.56	2.56
16.53	2.41	2.42	2.39	2.44	2.41	2.48	2.56
16.81	2.51	2.53	2.51	2.48	2.56	2.57	2.65
17.12	2.35	2.43	2.45	2.42	2.46	2.42	2.49
17.45	2.30	2.27	2.38	2.40	2.37	2.40	2.49
17.81	2.39	2.46	2.44	2.40	2.44	2.50	2.55
18.19	2.36	2.41	2.43	2.48	2.50	2.57	2.61
18.60	2.37	2.34	2.38	2.40	2.42	2.47	2.52

19.05	2.45	2.42	2.44	2.44	2.44	2.53	2.61
19.53	2.46	2.41	2.44	2.40	2.33	2.42	2.47
20.05	2.38	2.39	2.43	2.40	2.47	2.45	2.54
20.61	2.19	2.19	2.23	2.20	2.21	2.27	2.34
21.22	2.29	2.30	2.28	2.29	2.30	2.40	2.50
21.87	2.25	2.32	2.25	2.28	2.28	2.38	2.43
22.57	2.19	2.21	2.21	2.28	2.23	2.30	2.38
23.33	2.22	2.24	2.27	2.29	2.33	2.43	2.46
24.15	2.13	2.14	2.18	2.26	2.27	2.32	2.37
25.03	2.19	2.24	2.26	2.23	2.26	2.23	2.30
25.98	2.25	2.23	2.29	2.25	2.23	2.24	2.27
27.01	2.19	2.17	2.13	2.15	2.19	2.28	2.33
28.11	2.14	2.14	2.20	2.21	2.17	2.24	2.27
29.30	2.15	2.16	2.17	2.17	2.21	2.28	2.35
30.59	2.08	2.08	2.11	2.12	2.16	2.19	2.22
31.98	2.17	2.24	2.22	2.17	2.22	2.26	2.34
33.47	2.10	2.10	2.10	2.14	2.11	2.13	2.22
35.09	2.12	2.07	2.06	2.09	2.11	2.16	2.20
36.83	2.09	2.07	2.10	2.18	2.13	2.19	2.28
38.71	1.85	1.90	1.87	1.93	1.95	2.04	2.05
40.73	1.86	1.83	1.86	1.87	1.89	1.93	1.99
42.92	1.93	1.97	1.97	1.94	2.00	2.06	2.09
45.27	1.96	2.00	1.97	1.93	1.97	1.91	1.93
47.81	1.87	1.92	1.92	1.93	1.97	2.00	2.08
50.55	1.96	1.97	1.91	1.97	1.98	1.99	2.09
53.51	1.88	1.88	1.86	1.82	1.84	1.91	2.00
56.69	1.93	1.96	1.98	1.95	2.02	2.09	2.12
60.13	1.88	1.90	1.94	1.91	1.96	1.94	2.00
63.84	1.80	1.77	1.78	1.83	1.90	1.93	1.98
67.83	1.87	1.91	1.94	1.96	1.94	1.97	2.01
72.15	1.85	1.87	1.87	1.83	1.83	1.85	1.85
76.80	1.89	1.92	1.92	1.95	1.96	2.01	2.00
81.81	1.78	1.79	1.80	1.77	1.79	1.86	1.90
87.22	1.65	1.71	1.67	1.68	1.70	1.72	1.76
93.06	1.87	1.91	1.92	1.89	1.93	1.98	1.99
99.35	1.72	1.68	1.70	1.74	1.72	1.79	1.84
106.13	1.73	1.76	1.77	1.82	1.80	1.86	1.87
113.45	1.67	1.68	1.67	1.70	1.72	1.74	1.81
121.34	1.75	1.80	1.81	1.79	1.81	1.90	1.95
129.86	1.70	1.65	1.66	1.78	1.76	1.84	1.82
139.04	1.60	1.61	1.66	1.62	1.62	1.65	1.60
148.94	1.74	1.79	1.80	1.74	1.76	1.82	1.88
159.62	1.71	1.70	1.70	1.76	1.80	1.83	1.85
171.14	1.66	1.72	1.70	1.65	1.65	1.71	1.81
183.56	1.76	1.78	1.71	1.72	1.69	1.75	1.86
196.96	1.65	1.71	1.71	1.74	1.71	1.77	1.82
211.41	1.65	1.66	1.75	1.74	1.72	1.71	1.74
226.99	1.60	1.64	1.64	1.66	1.75	1.78	1.88
243.80	1.57	1.65	1.66	1.62	1.61	1.62	1.65
261.93	1.77	1.78	1.74	1.73	1.78	1.80	1.80

281.48	1.81	1.85	1.78	1.75	1.74	1.77	1.85
302.56	1.70	1.75	1.73	1.75	1.71	1.72	1.79
325.30	1.74	1.80	1.77	1.75	1.76	1.73	1.76
349.83	1.71	1.72	1.73	1.73	1.70	1.68	1.76
376.28	1.82	1.78	1.73	1.70	1.69	1.75	1.78
404.81	1.56	1.57	1.55	1.57	1.60	1.62	1.69
435.58	1.83	1.90	1.87	1.82	1.80	1.80	1.86
468.76	1.76	1.77	1.70	1.69	1.74	1.75	1.78
504.55	1.85	1.83	1.77	1.77	1.74	1.87	1.89
543.15	1.94	1.89	1.90	1.86	1.84	1.87	1.83
584.78	1.96	1.97	1.92	1.93	1.91	1.97	2.01
629.68	1.81	1.91	1.87	1.84	1.79	1.84	1.86
678.10	2.05	2.02	2.02	2.03	1.98	2.05	2.01
730.33	2.02	2.06	2.04	2.03	1.99	2.06	2.08
786.65	1.97	2.03	2.01	2.01	2.00	2.09	2.09
847.40	2.18	2.24	2.18	2.11	2.10	2.07	2.14
912.92	2.23	2.21	2.20	2.20	2.12	2.19	2.20
983.58	2.42	2.38	2.35	2.28	2.26	2.24	2.24
1059.78	2.34	2.33	2.30	2.23	2.12	2.15	2.13
1141.98	2.55	2.48	2.40	2.35	2.31	2.29	2.25
1230.62	2.39	2.29	2.21	2.22	2.18	2.22	2.25
1326.23	2.48	2.45	2.46	2.36	2.38	2.31	2.25
1429.34	2.56	2.60	2.56	2.49	2.39	2.40	2.41
1540.54	2.52	2.47	2.45	2.37	2.34	2.34	2.33
1660.48	2.77	2.74	2.67	2.57	2.54	2.52	2.56
1789.83	2.90	2.78	2.73	2.66	2.63	2.65	2.69
1929.34	2.70	2.72	2.68	2.63	2.65	2.58	2.54
2079.80	2.83	2.73	2.73	2.69	2.61	2.58	2.54
2242.08	2.87	2.83	2.79	2.69	2.63	2.60	2.61
2417.10	3.01	2.94	2.88	2.80	2.75	2.76	2.81
2605.85	2.89	2.83	2.82	2.69	2.69	2.62	2.68
2809.43	3.00	2.91	2.82	2.78	2.76	2.68	2.69
3028.99	3.15	3.03	2.99	2.92	2.85	2.77	2.82
3265.79	3.01	2.94	2.84	2.80	2.75	2.77	2.79
3521.18	2.94	2.88	2.82	2.74	2.65	2.61	2.61
3796.62	3.08	2.98	2.90	2.83	2.75	2.73	2.68

Table S7. Raw data of fs-TA signals of D-RC-A collected under VIS (λ_{ex} = 513 nm) excitation in the λ_{pr} = 318–670 nm regime, the unit of ΔA in the table is mOD.

Wavelength (nm) / Time (ps)	318.42	319.85	321.27	322.70	324.12	325.54	326.97	328.39	329.82	331.24	332.67
-3.78	0.08	-0.55	-1.77	0.50	-0.31	0.50	-1.07	-0.85	-0.25	-0.61	0.51
-3.28	-1.61	1.74	0.24	-0.50	1.37	-0.04	-0.49	0.62	0.34	0.55	-0.14
-2.78	0.85	-0.58	1.20	-0.09	-0.17	0.34	-0.32	-0.28	-0.01	0.90	-0.37
-2.28	0.58	0.81	0.78	-0.72	0.48	1.74	-0.37	0.00	-0.12	0.20	-0.57
-1.78	0.18	-0.25	-0.65	0.23	0.19	-0.32	1.27	-0.44	-0.56	-0.10	0.97
-1.28	0.34	-1.06	0.34	-0.02	-0.41	-0.22	0.61	0.19	-0.29	-0.71	-0.30
-0.78	0.47	-1.28	0.45	0.70	-0.71	-2.21	-0.17	0.68	-0.03	-0.47	-0.54

-0.28	-0.89	1.16	-0.59	-0.11	-0.46	0.22	0.52	0.08	0.92	0.24	0.43
-0.18	-0.35	-1.44	0.92	-0.35	-0.19	-0.95	0.83	0.39	-0.53	0.56	0.26
-0.08	0.17	-2.21	0.13	-1.57	0.74	0.32	-0.11	0.77	0.25	0.76	0.37
0.02	0.73	-0.44	-0.72	-0.58	-0.86	-1.48	-1.04	0.04	0.38	-0.30	-0.07
0.12	-1.09	0.81	-1.07	-1.97	-1.36	-0.42	0.50	-0.40	0.28	0.22	0.18
0.22	-0.32	-1.21	-0.23	-0.42	0.58	-1.91	0.45	1.07	0.02	0.58	-0.42
0.27	1.25	-0.31	-2.10	-2.87	-1.09	-1.53	-1.59	0.31	0.08	0.49	0.41
0.32	4.02	2.98	4.09	4.22	4.12	-1.91	-5.40	-6.48	-4.53	-2.99	-0.38
0.37	-2.38	-1.04	3.07	5.65	8.69	11.13	11.03	4.72	-3.24	-5.84	-7.49
0.42	-2.78	-3.38	-2.66	-5.97	-6.22	0.61	10.72	20.34	25.22	26.07	18.72
0.47	-1.11	1.41	-1.73	-6.14	-7.03	-11.63	-14.16	-11.11	-5.81	6.92	23.29
0.52	1.98	-0.39	1.19	-0.57	-0.47	-1.78	-4.62	-10.01	-15.61	-24.34	-28.53
0.57	-0.86	0.62	-0.07	0.75	-1.11	-0.14	-0.73	1.45	0.29	-2.30	-6.70
0.62	-1.59	-0.87	0.44	-0.01	-1.45	0.11	-0.20	0.02	-1.24	-0.67	0.63
0.67	-0.22	-0.59	-0.80	-0.07	0.06	0.54	0.37	-0.93	-0.29	0.45	0.12
0.72	-1.05	0.51	-0.14	0.75	0.02	-0.59	-0.26	0.15	0.11	0.21	-0.30
0.77	-0.71	-0.49	-0.28	0.42	-0.71	-0.65	-1.19	-1.27	0.03	-0.12	-0.03
0.82	-0.48	0.36	-0.96	-1.67	0.36	-2.24	-0.93	1.41	-0.40	-1.26	-1.79
0.87	-0.61	-0.76	-0.56	-2.41	-1.79	-0.05	-1.03	-0.77	-0.34	-0.21	-0.96
0.92	-2.29	-0.96	-0.53	-1.91	-0.52	0.18	-0.88	-1.60	-1.68	-0.72	-0.11
0.97	-0.88	1.51	-0.93	-0.04	-0.99	-0.14	0.03	-0.79	-1.21	-0.78	-0.86
1.02	0.16	0.14	-0.32	-1.32	-0.60	-0.98	0.28	-0.26	-1.58	-0.99	-0.92
1.07	-1.65	-1.18	0.45	-0.41	-0.28	0.59	-1.08	-0.49	-1.78	-0.32	-1.14
1.12	-1.66	-0.98	-1.00	-1.71	-0.19	-1.53	-0.80	-1.32	-1.85	-1.27	-0.97
1.17	-1.51	-0.18	0.25	0.60	-0.72	-0.62	-1.31	-0.61	-2.17	-1.42	-1.50
1.22	0.41	-1.07	0.34	-0.48	-0.97	-0.34	-2.58	-1.10	-1.42	-1.45	-1.11
1.27	-0.23	-0.09	0.28	-1.80	-0.19	-0.84	-2.22	-0.82	-0.93	-1.08	-1.21
1.32	0.01	-0.10	-0.29	-1.80	0.17	-1.39	0.32	-0.17	-1.87	-0.74	-1.70
1.37	-0.90	0.17	-0.75	0.25	0.32	-1.30	-0.67	-1.12	-1.47	-0.82	-0.60
1.42	-0.92	0.49	0.49	-0.02	-1.13	-1.97	-0.99	-0.41	-0.98	-2.22	-1.23
1.47	-0.10	-0.15	-1.30	0.48	0.31	-0.39	-1.12	-1.06	-1.03	-0.87	-0.23
1.52	-0.70	-1.07	-0.07	-0.99	-0.90	0.08	-1.46	-1.33	-1.07	-1.92	-2.19
1.57	-0.05	-1.15	1.18	-0.58	-0.37	-1.03	-1.84	-1.75	-1.31	-0.04	-1.27
1.62	0.46	-1.86	-1.75	-1.47	-0.08	-1.06	-1.83	-1.57	-1.91	-1.61	-1.59
1.67	-0.71	-2.01	-0.58	-1.86	-0.83	-2.04	-1.17	-1.41	-1.83	-1.09	-1.74
1.72	-1.18	-0.14	-1.04	-0.95	-1.27	-0.68	-1.07	-0.15	-1.75	-1.52	-1.22
1.77	-2.01	0.04	-2.64	-2.05	-0.34	-1.50	0.46	-1.49	-1.92	-1.19	-1.63
1.82	-1.07	-0.74	-1.12	0.22	-1.35	-0.79	-1.83	-1.53	-1.57	-2.10	-0.92
1.87	-1.01	0.49	-1.13	-0.10	-0.34	-0.32	-0.88	-2.54	-1.36	-1.07	-2.65
1.92	-2.77	-1.94	-1.82	-1.40	0.24	-1.09	-1.07	-0.96	-1.62	-1.02	-1.39
1.97	-0.08	-1.89	0.16	-1.41	-0.66	-1.05	-0.12	-0.94	-1.62	-0.63	-1.01
2.02	-1.46	-0.21	-1.00	-1.26	-1.27	-1.06	-0.79	-0.12	-0.80	-1.08	-1.02
2.07	-0.15	-0.46	-0.94	-0.32	-0.15	-0.47	-1.44	-1.49	-0.57	-2.13	-1.28
2.12	-0.09	-1.54	0.53	-1.08	-0.38	-0.96	-1.77	-2.27	-1.24	-2.07	-1.13
2.17	-1.71	-0.66	-0.48	-0.93	-0.50	-1.98	-0.09	-1.33	-1.20	-0.83	-1.35
2.22	-0.82	-1.87	-0.26	-0.95	0.13	-1.07	-0.84	-0.47	-0.92	-0.43	-0.14
2.27	-1.68	0.08	0.72	-1.63	-0.04	-0.98	-0.24	-0.66	-1.00	-1.71	-2.12
2.32	0.04	-1.83	-4.40	-0.42	-0.06	-1.60	-2.43	-0.99	-1.06	-2.37	-0.62
2.37	-0.29	0.48	-1.53	-0.60	0.23	-0.46	-3.06	-0.36	-1.11	-2.59	-1.41
2.42	-0.82	0.66	-0.79	-0.96	-0.36	-2.11	-1.32	-2.33	-0.51	-1.65	-0.77

2.47	-2.31	0.76	-1.30	-2.34	0.51	-0.68	-1.47	-0.83	-2.00	-2.26	-1.26
2.52	0.68	-0.74	-0.98	-1.87	-0.28	-1.51	-1.12	-2.13	-2.28	-1.24	-2.18
2.57	0.29	-0.12	0.41	-0.56	-0.85	-1.12	-0.86	-1.58	-2.42	-1.00	-2.48
2.62	-0.73	-1.73	-2.77	-0.31	-0.03	-0.06	-1.33	-1.55	-1.19	-1.55	-1.38
2.67	-0.39	-1.21	-0.66	-0.35	-0.80	-1.63	-1.53	-1.52	-1.76	-0.85	-1.00
2.72	-0.61	-1.11	1.29	-2.17	0.34	-1.10	0.07	-1.56	-1.46	-0.46	-1.19
2.77	-2.26	-1.29	-1.19	-2.08	0.24	-2.39	-1.03	-1.20	-1.34	-1.95	-1.84
2.82	0.54	-0.19	-2.38	-1.62	-1.23	-0.99	-2.16	-2.33	-1.15	-1.71	-1.03
2.87	-0.41	-0.66	-0.06	-0.15	-1.15	-1.84	-1.06	-1.38	-1.57	-1.19	-1.73
2.92	-1.08	-1.57	-0.38	-1.71	-0.70	-2.03	-0.11	-1.48	-0.24	-0.50	-1.44
2.97	-0.86	-1.02	-1.34	-1.16	-0.46	-1.90	-1.48	-1.06	-1.29	-2.00	-1.50
3.02	0.90	-1.41	-2.17	-1.26	0.03	-0.62	-0.45	-0.44	-1.61	-1.98	-0.99
3.07	-0.38	-1.80	1.03	-1.59	-0.49	-0.65	-0.76	-1.51	-1.08	-1.53	-1.51
3.12	0.00	-0.51	-0.53	-2.02	-1.73	-2.47	-0.40	-1.72	-1.99	-1.84	-1.26
3.17	1.07	-0.68	0.01	-3.33	0.18	-1.22	-0.75	-1.07	-0.77	-2.01	-2.34
3.22	-0.90	-0.25	-0.83	-1.40	-0.83	-1.99	-2.19	-0.27	-1.67	-1.23	-0.71
3.42	-0.61	-1.33	-1.21	-1.07	-2.06	-1.22	-0.87	-1.12	-1.50	-1.95	-2.04
3.62	-0.02	-0.85	-1.53	-2.13	-1.02	-2.25	-1.68	-0.45	-1.19	-1.45	-0.97
3.82	-0.58	-1.80	-1.27	0.29	-0.77	-1.26	-1.18	-1.02	-1.42	-0.72	-2.07
4.02	0.05	-0.90	-0.65	-1.96	0.09	-1.91	-1.85	-1.15	-2.01	-1.26	-2.13
4.22	-0.18	-0.12	-1.42	-1.38	-1.99	0.05	-0.56	-1.59	-1.54	-0.74	-1.72
4.42	-0.43	-0.06	-1.16	-1.77	-1.05	-0.27	0.01	-2.29	-1.01	-0.98	-1.67
4.62	0.63	-0.68	-1.90	-1.27	-0.61	-0.11	-0.68	0.35	-0.85	-2.16	-1.44
4.82	-1.25	-0.12	0.07	-1.14	0.37	-1.71	-1.78	-1.89	-2.18	-2.41	-1.95
5.02	0.15	0.07	-0.24	-1.02	-1.42	-2.29	-1.43	-1.99	-1.32	-1.34	-1.82
5.22	-1.37	0.49	-1.05	-0.56	-0.70	-0.48	-1.38	-0.37	-2.56	-2.12	-1.03
5.42	-1.83	-1.42	-1.87	-0.59	-0.66	-1.44	-0.58	-1.76	-2.07	-2.53	-1.87
5.62	0.67	-1.38	-2.31	0.49	0.77	-2.43	-1.26	0.78	-2.71	-1.85	-1.28
5.82	0.36	-1.77	-0.93	-0.34	-0.05	-0.89	-1.64	-1.35	-2.24	-1.35	-1.32
6.02	0.06	-1.44	-0.81	-1.40	-2.40	-0.52	-1.13	-1.32	-1.85	-1.51	-1.55
6.22	-1.13	-0.95	0.14	-2.03	-0.78	-2.34	-2.15	-1.42	-2.12	-1.06	-2.10
6.42	-2.09	-0.47	-1.53	-0.91	-0.54	-1.14	-1.35	-1.76	-0.30	-1.22	-2.38
6.62	-0.81	-1.18	-0.50	-1.49	-2.69	-0.70	-2.42	-1.38	-1.59	-0.88	-1.77
6.82	-0.76	-1.66	-1.17	-0.89	-0.93	-0.72	-2.27	-1.78	-3.11	-2.61	-1.56
7.02	-1.99	-1.53	-1.75	-0.60	-1.46	-1.15	-2.27	-1.99	-1.30	-1.44	-1.15
7.22	-1.50	-2.10	-2.04	-0.27	-1.44	-3.06	-1.67	-1.18	-0.38	-0.78	-1.20
7.42	-1.03	-0.73	-0.17	-1.41	-2.26	0.14	-0.88	-1.94	-2.63	-1.58	-2.56
7.62	-1.28	-0.70	-1.90	-0.80	-0.90	-2.67	-1.67	-0.82	-1.99	-2.38	-2.19
7.82	0.91	-1.97	-0.74	-0.98	-2.94	-1.96	-1.98	-1.26	-2.13	-2.01	-2.64
8.02	-2.59	-0.28	-1.14	-0.74	-1.72	-2.77	-0.30	0.31	-1.98	-1.51	-1.93
8.22	-2.79	-1.02	-0.44	-0.44	-0.39	-1.05	-0.74	-1.08	-1.55	-2.16	-2.48
8.42	-0.93	-0.25	-0.07	-2.02	-1.37	-3.32	-1.01	-1.01	-2.59	-1.79	-2.31
8.62	-0.36	-0.28	-0.29	-0.61	0.31	-1.42	-0.49	-1.73	-2.14	-1.85	-1.90
8.82	-0.65	0.47	-2.03	-1.17	-0.48	-0.91	-3.57	-1.20	-1.24	-1.97	-2.05
9.02	-1.23	-2.20	-0.65	-0.65	-0.27	-1.03	-0.86	-1.39	-2.72	-2.15	-1.47
9.22	-0.09	-1.42	0.04	-2.05	-0.65	-1.92	-1.70	-1.36	-1.47	-2.29	-2.02
9.42	-2.42	0.48	-0.87	0.43	-1.89	-0.77	-1.11	-1.50	-1.55	-2.38	-2.33
9.62	1.16	-1.05	-0.69	-0.51	-1.51	0.02	-1.68	-0.52	-3.05	-1.80	-1.65
9.82	-0.71	-1.40	-1.19	-0.47	-1.29	-1.96	-0.21	-1.99	-2.66	-0.88	-2.67
10.02	0.58	0.02	-1.16	-2.56	-0.70	-1.86	-1.63	-0.84	-0.96	-2.90	-1.41

10.22	-2.01	-0.02	-1.94	-1.32	-1.98	-1.17	-2.31	-0.99	-0.80	-1.61	-3.06
10.42	0.07	-0.19	-1.07	-1.68	-1.73	-1.12	-0.61	-0.59	-1.91	-1.77	-2.61
10.62	-1.14	-0.93	-0.49	-2.15	0.25	-0.64	-1.13	-0.76	-1.62	-1.87	-1.90
10.82	-1.97	-0.50	-1.24	0.04	-1.37	-1.75	-1.48	-0.34	-2.31	-1.42	-2.70
11.02	-0.31	-2.13	-1.94	-0.80	0.53	-0.28	-0.24	-3.06	-2.64	-3.35	-2.08
11.22	-0.48	-2.14	-0.20	-2.18	-1.57	-1.38	-0.71	-2.05	-2.07	-2.29	-2.05
11.42	-2.01	-1.21	-0.52	-1.56	-0.86	-0.91	-0.71	-1.28	-1.09	-1.40	-2.05
11.62	-2.67	-1.00	-1.31	-1.76	-1.81	-1.54	-1.20	-0.18	-3.11	-3.07	-1.60
11.82	-0.79	-0.31	-1.35	-2.28	-0.55	-1.43	-1.62	-2.28	-1.70	-1.58	-1.52
12.02	-0.51	-0.34	0.02	-0.77	-1.08	-0.53	-2.68	-0.73	-1.94	-1.20	-1.56
12.22	0.68	-0.28	-1.33	-2.14	-1.52	-2.51	-1.91	-0.63	-1.91	-2.67	-1.54
12.42	-1.27	-1.44	0.18	-1.18	-0.58	-1.29	-2.12	-0.70	-1.79	-1.10	-2.27
12.62	-0.94	-0.15	-1.35	-1.07	-0.41	-0.66	-1.55	-2.44	-1.11	-2.10	-1.57
12.82	-0.91	-0.97	-1.26	-2.09	-1.73	0.64	-1.61	-0.83	-2.03	-1.04	-2.28
13.02	-1.40	-1.25	-1.93	-0.60	-0.57	-2.05	0.62	-1.76	-1.62	-1.09	-0.84
13.22	-0.90	0.42	-0.98	-1.17	-2.04	-0.95	-1.80	-0.63	-2.42	-3.10	-1.56
13.42	-1.21	-2.23	1.41	-1.84	-0.22	-1.08	-2.45	-0.81	-1.96	-3.07	-1.62
13.50	-0.88	-0.32	-0.99	-1.90	-1.19	-2.08	-1.15	-2.22	-1.64	-1.72	-1.71
13.59	-0.71	0.09	-0.20	-1.37	0.11	-1.72	-1.05	-0.78	-1.61	-1.39	-1.99
13.68	-2.05	-1.57	-1.63	-1.88	-1.82	-1.36	-1.61	-1.80	-2.15	-1.09	-1.64
13.78	-0.63	-0.94	-1.18	-2.97	-0.99	-1.52	-1.58	-1.02	-1.45	-1.98	-2.37
13.88	-0.57	-0.44	-0.84	-1.82	-1.24	-0.76	-2.46	-2.97	-0.50	-2.00	-0.95
14.00	-1.92	-0.25	-0.98	-3.40	-0.84	-1.52	-1.24	-2.05	-1.96	-2.36	-0.96
14.12	-0.70	-1.28	-0.55	-2.13	-0.79	-1.61	-1.24	-0.61	-1.86	-2.35	-1.93
14.25	-0.44	-1.22	-0.96	-2.57	-1.30	-1.11	-0.22	-1.36	-1.20	-1.74	-1.91
14.40	-0.47	-0.32	0.74	-0.86	-0.71	-0.87	-1.05	-2.47	-2.18	-2.07	-2.22
14.55	-3.25	-2.08	-0.94	-0.98	-0.93	-1.39	-1.97	-1.11	-1.80	-0.95	-1.99
14.72	-0.01	-1.34	-0.90	-2.57	-0.68	-2.40	-2.24	-1.70	-2.50	-2.29	-2.07
14.90	-0.47	0.54	-1.77	-0.63	-1.39	-2.99	-0.59	-1.85	-1.97	-1.58	-2.15
15.09	0.06	-1.67	-0.40	-1.34	-0.84	-0.15	-2.14	-0.80	-2.02	-3.16	-2.04
15.30	-0.39	-2.19	-1.37	-0.52	-0.59	-2.31	-1.59	-1.71	-2.11	-2.42	-2.00
15.53	-1.16	-0.85	0.47	-1.56	-1.44	-2.33	-1.25	-1.06	-2.68	-1.74	-1.91
15.77	0.55	-0.73	-3.45	-1.73	0.08	-1.48	-2.03	-0.94	-2.17	-2.61	-3.14
16.04	-0.43	-1.76	-0.13	-2.70	-0.19	-0.91	-2.18	-1.42	-1.15	-2.10	-1.96
16.32	-1.16	-1.03	0.08	-1.96	-0.72	-2.42	-1.56	-0.72	-0.96	-2.72	-1.62
16.63	-1.57	-0.49	-0.94	-2.60	-1.75	-0.96	-0.45	-2.15	-2.63	-1.61	-3.02
16.96	0.02	-2.82	-2.47	-2.54	-0.45	-3.05	-2.11	-0.41	-3.13	-3.24	-1.60
17.31	-1.05	0.82	-2.91	-2.42	0.09	-1.42	-2.08	-1.91	-0.06	-2.48	-1.81
17.70	-0.92	-1.14	0.46	-2.31	-2.23	-1.46	0.18	-2.38	-2.98	-1.76	-2.13
18.11	-1.04	0.05	-2.02	-3.03	0.32	-2.97	-2.43	-0.16	-2.62	-2.69	-2.30
18.56	-2.47	0.76	0.18	-2.24	-1.21	-0.54	-0.79	-1.01	-1.77	-1.31	-2.73
19.04	-0.25	1.10	-0.32	-1.24	-0.06	-1.65	-1.53	-1.76	-1.86	-2.16	-0.95
19.56	0.24	-1.01	-2.02	-0.98	-2.13	-1.32	-1.02	-1.11	-2.75	-1.58	-1.35
20.12	-0.70	-0.93	-1.57	-0.02	-0.05	-1.64	-1.39	-2.65	-1.98	-2.16	-3.51
20.72	-0.40	-0.67	0.07	-1.20	-1.53	-2.64	-3.18	-1.98	-1.17	-2.27	-2.03
21.37	-1.65	-0.39	-0.75	-1.22	-2.51	-2.28	-2.38	-1.82	-2.54	-1.24	-3.06
22.08	-0.95	-1.98	-0.09	-0.55	-1.87	-2.15	-1.35	-1.58	-2.48	-1.96	-0.46
22.84	-1.33	-1.73	-0.30	-0.26	-1.43	-0.74	-0.27	-1.05	-2.07	-2.19	-1.94
23.65	0.79	-0.31	-1.29	-1.36	-1.33	-0.25	-0.30	-1.32	-2.10	-2.22	-1.76
24.54	1.09	-1.30	-2.51	-1.37	-1.08	-0.70	-0.63	-2.08	-1.85	-2.13	-2.29

25.49	-1.99	-0.23	-1.07	-1.18	-1.04	-1.36	-2.26	-1.54	-1.99	-1.14	-2.31
26.51	-1.27	-1.29	-0.79	-1.44	-1.44	-1.41	-2.25	-1.46	-1.80	-2.34	-2.43
27.62	-1.51	-0.50	-2.15	-1.30	-2.31	-1.97	-2.64	-1.17	-2.19	-2.76	-1.22
28.81	0.69	-0.95	0.70	-0.46	-1.64	-0.76	-1.65	-1.65	-2.40	-2.21	-3.11
30.10	-1.64	0.49	-2.24	-0.05	-1.59	-1.44	-1.52	-1.67	-2.29	-2.84	-2.51
31.49	-0.64	-1.25	0.30	-1.77	-0.59	-1.66	-1.73	-1.07	-3.06	-2.05	-1.14
32.98	-0.76	-1.33	-1.14	-0.58	-1.00	-2.31	-2.36	-1.25	-1.55	-2.05	-2.39
34.60	-1.66	0.07	0.04	-2.77	-0.06	-1.16	-2.06	-1.18	-1.63	-2.01	-1.69
36.34	-0.40	-0.89	-1.06	-1.18	-0.63	-1.89	-2.37	-1.27	-2.74	-2.53	-2.87
38.21	-1.66	0.09	-0.59	-0.43	-2.44	-1.47	-2.52	-2.35	-2.52	-1.47	-2.37
40.24	-1.87	-1.21	-1.16	-1.79	-1.58	-1.67	-1.94	-2.04	-0.90	-1.84	-1.07
42.42	-1.98	1.37	0.44	-1.68	-0.68	-0.99	-1.53	-1.75	-2.75	-0.93	-2.04
44.78	-1.07	-0.82	-0.23	-1.49	-0.67	-1.04	-1.21	-1.85	-1.43	-1.42	-0.88
47.32	-1.82	-1.11	-0.45	-1.49	-1.16	-1.85	-1.90	-1.15	-1.75	-1.88	-2.34
50.06	-0.65	-1.28	-2.56	0.38	-1.01	-0.90	-1.64	-0.01	-2.13	-0.86	-2.58
53.01	-0.61	0.98	-0.41	-0.32	-1.31	-2.03	-1.40	-0.56	-2.11	-1.82	-1.33
56.20	-0.33	-1.14	0.01	-1.05	-2.24	-2.50	-1.49	-1.75	-2.47	-1.75	-2.68
59.64	-0.13	-0.98	-1.24	-0.69	-1.38	-1.52	-2.64	-2.21	-2.22	-1.52	-2.11
63.34	-0.48	-0.33	-1.74	-2.77	-0.30	-0.41	-0.30	-2.35	-2.39	-2.12	-2.04
67.34	0.23	-0.81	-0.55	-1.58	-1.00	-2.74	-2.35	-1.63	-1.53	-2.41	-1.92
71.65	-1.96	-0.98	-0.89	-0.41	-2.11	0.27	-0.67	-0.99	-1.37	-1.89	-3.07
76.30	0.40	-0.46	-0.27	-0.77	-1.51	-1.92	-0.92	-1.72	-1.83	-1.57	-1.27
81.32	-1.36	-0.47	-1.41	-2.37	-0.76	-2.13	-1.45	-1.59	-1.90	-1.92	-2.50
86.73	-1.71	-0.77	0.38	-1.94	-0.27	-2.37	-1.28	-2.06	-2.11	-2.98	-2.63
92.56	-2.12	0.05	-0.29	-1.81	-0.96	-0.88	-1.57	-0.69	-1.83	-1.82	-2.35
98.85	-0.51	0.35	-1.07	-2.00	-1.58	-1.81	-2.10	-1.20	-2.70	-1.81	-2.06
105.64	0.34	0.03	-0.11	-1.03	0.23	-0.94	-1.25	-1.39	-2.08	-1.30	-1.92
112.96	-0.30	-0.84	-0.96	-2.13	-0.66	-1.39	-0.94	-2.06	-2.03	-2.07	-2.51
120.85	-0.13	0.64	0.25	-0.59	-1.23	-0.90	-1.25	-0.90	-1.40	-1.07	-1.68
129.36	-0.78	0.00	-0.13	-1.53	-1.52	-2.00	-1.57	-0.35	-3.08	-2.93	-1.71
138.55	-1.09	-0.52	-2.07	-1.71	-0.61	-1.32	-1.96	-2.56	-2.43	-1.47	-2.19
148.45	0.32	-1.55	-1.59	-1.34	-0.71	-1.53	-2.11	-1.15	-2.47	-2.27	-1.73
159.13	-1.14	-1.31	-2.14	0.03	-1.04	-2.15	-0.41	-2.06	-1.70	-0.79	-1.29
170.65	-1.37	0.38	-1.89	-1.58	-0.55	-2.58	-1.64	-2.89	-3.21	-1.40	-2.32
183.07	-0.83	-2.68	0.81	-2.21	-0.59	-2.39	-1.75	-1.83	-2.08	-2.81	-2.10
196.47	-0.34	-0.12	-0.62	-0.95	-1.87	-1.35	-3.23	-2.70	-2.70	-2.46	-2.11
210.91	-1.49	-1.14	-1.32	-0.32	-1.50	-1.25	-1.12	-2.18	-2.31	-2.59	-1.70
226.50	-1.58	-0.38	-1.16	-1.76	-1.17	-1.26	-2.11	-2.28	-2.86	-1.84	-1.68
243.31	-0.14	-0.43	0.21	-0.99	-1.13	-1.26	-2.21	-2.89	-2.86	-0.75	-2.07
261.43	-0.69	-1.28	-1.11	-0.08	-2.02	-2.59	-2.21	-2.09	-1.32	-2.54	-1.94
280.98	-0.78	-0.40	-0.55	-0.78	-1.54	-2.71	-2.22	-1.12	-2.10	-2.15	-3.90
302.07	-2.10	0.18	-0.50	-3.27	-0.02	-2.80	-1.52	-1.19	-1.76	-1.71	-2.80
324.81	-0.90	-2.19	-0.46	-2.28	-1.34	-1.07	-1.96	-0.98	-1.81	-3.08	-2.85
349.33	-0.16	0.44	-1.97	-1.38	-1.04	-0.06	-0.86	-1.26	-1.78	-1.83	-1.90
375.79	-2.28	-0.53	-0.03	-3.06	-1.74	-2.28	-1.08	-3.46	-2.23	-2.59	-1.47
404.31	-1.66	0.19	-0.50	0.46	-1.59	-1.69	-2.01	-1.60	-3.24	-1.79	-2.38
435.08	-0.27	0.27	-1.83	-1.95	-2.61	-1.44	-2.62	-1.78	-4.21	-2.61	-2.56
468.27	0.40	-0.83	-0.79	-0.84	-0.25	-0.27	-1.19	-1.60	-1.88	-1.74	-2.06
504.06	-0.57	-1.69	-1.36	-0.96	-1.01	-1.24	-1.00	-2.65	-2.37	-1.88	-1.53
542.66	0.23	-1.23	-2.03	-1.42	-2.07	-1.42	-2.02	-2.18	-2.61	-2.59	-2.66

584.29	-1.56	-1.11	-0.22	-1.60	-0.73	-1.74	-1.28	-2.38	-2.67	-1.88	-1.54
629.18	0.32	-0.80	0.69	-0.54	-0.43	-1.68	-2.52	-1.54	-2.30	-2.01	-2.03
677.61	0.23	-1.20	-1.55	-1.77	-1.56	-1.63	-0.56	-2.20	-2.36	-1.86	-3.23
729.83	-1.39	-0.08	-0.51	-0.99	-1.02	-0.90	-1.21	-1.80	-1.13	-2.12	-1.36
786.16	0.80	-0.08	-1.05	-2.38	-1.55	-1.23	-1.57	-3.11	-1.48	-2.42	-1.97
846.90	-1.50	-1.47	-1.12	-1.90	-2.54	-1.45	-1.59	-0.96	-3.02	-1.84	-2.18
912.42	0.42	-0.50	0.53	-1.92	-0.99	-1.80	-1.38	-2.21	-1.76	-1.34	-1.55
983.08	-1.59	0.17	-0.24	-1.43	-1.20	-1.54	-0.98	-1.70	-1.94	-1.79	-1.40
1059.29	-2.18	-2.56	-0.43	-1.90	-0.57	-3.33	-2.53	-1.18	-2.67	-2.02	-2.52
1141.48	-0.93	-1.25	-0.67	-1.25	-1.87	-1.37	-1.67	-1.62	-2.87	-2.32	-2.35
1230.13	-3.03	-1.90	-2.19	-1.73	-0.73	-1.22	-2.47	-2.51	-2.43	-1.94	-1.37
1325.73	-0.80	0.01	-1.84	-1.85	-0.65	-1.41	-0.16	-1.59	-1.79	-1.66	-1.22
1428.84	-0.24	-1.63	-1.13	-1.91	-1.52	-1.30	-0.80	-1.96	-1.84	-2.49	-2.10
1540.05	-0.55	-2.20	-0.68	-1.05	-0.98	-3.15	-2.75	-1.49	-2.38	-2.16	-2.23
1659.99	-1.73	-0.90	0.43	-0.30	-1.46	-2.09	-1.70	-1.32	-2.26	-1.24	-1.82
1789.34	-1.52	0.12	-1.14	-1.25	-1.41	-0.79	-1.01	-2.13	-3.58	-1.52	-1.72
1928.85	-1.04	-0.03	-1.27	-2.47	-1.27	-0.82	-0.15	-1.95	-2.83	-1.99	-1.90
2079.31	-0.90	-0.70	-1.29	-1.48	-1.32	-1.88	-2.38	-2.43	-2.45	-1.48	-2.12
2241.59	-3.27	-0.17	-1.88	-0.65	-1.22	-0.65	-3.75	-2.97	-2.53	-1.23	-1.81
2416.60	-0.52	-1.50	-0.15	-2.00	-1.46	-1.96	-1.99	-2.80	-1.99	-1.81	-1.92
2605.36	-0.01	0.09	-1.79	-1.83	-1.36	-2.03	-0.93	-0.48	-2.45	-2.40	-2.34
2808.94	-0.24	-0.43	-0.81	-1.68	-2.65	-3.03	-1.94	-2.31	-1.90	-2.14	-1.94
3028.50	-0.70	-1.24	-0.16	-1.61	-1.46	-1.20	-1.70	-0.44	-2.16	-1.95	-1.91
3265.30	-1.88	-1.19	-0.55	-1.73	-1.51	-2.84	-1.66	-1.09	-3.17	-2.88	-1.63
3520.69	-1.25	-0.16	-1.00	0.24	-1.61	-2.37	-1.81	-2.09	-1.79	-2.53	-1.89
3796.13	0.55	-1.23	-0.84	-1.46	0.54	-1.90	-2.13	-1.35	-2.66	-0.82	-1.60

Wavelength (nm) / Time (ps)	334.09	335.51	336.94	338.36	339.79	341.21	342.63	344.06	345.48	346.91	348.33
-3.78	0.13	0.19	-0.71	-0.15	0.00	-0.28	0.13	0.01	0.00	-0.25	-0.09
-3.28	0.29	0.84	0.98	0.42	0.16	0.25	0.36	0.26	0.40	0.35	0.04
-2.78	-0.49	-0.03	0.15	-0.21	0.42	0.20	0.17	0.49	0.04	0.22	0.20
-2.28	0.18	-0.18	0.09	-0.04	-0.03	-0.09	0.13	0.39	0.10	0.04	0.27
-1.78	0.16	0.05	-0.39	0.55	-0.31	0.23	0.41	0.01	-0.10	0.09	0.06
-1.28	0.36	-0.18	-0.30	-0.05	-0.10	0.15	-0.23	-0.10	-0.15	-0.12	0.04
-0.78	-0.03	-0.37	0.19	-0.53	-0.33	-0.31	-0.59	-0.63	-0.17	-0.04	-0.50
-0.28	-0.60	-0.32	-0.01	0.00	0.18	-0.16	-0.39	-0.44	-0.12	-0.29	-0.03
-0.18	-0.15	0.13	0.02	0.43	-0.14	0.14	0.73	0.24	0.08	-0.46	-0.20
-0.08	-0.95	0.64	0.01	0.33	0.86	0.03	0.30	-0.09	-0.07	-0.19	-0.07
0.02	-0.04	0.89	0.45	-0.16	-0.06	0.19	-0.18	-0.23	0.13	-0.17	-0.09
0.12	0.70	-0.25	-0.24	0.17	-0.25	0.13	0.26	0.08	0.17	-0.04	0.36
0.22	0.38	0.00	-0.22	-0.98	-0.26	-0.29	-0.15	-0.37	-0.12	-0.49	0.08
0.27	0.32	0.18	-0.44	0.05	-0.76	-0.01	-0.08	-0.20	0.10	-0.12	0.02
0.32	-0.22	0.60	0.26	0.17	0.22	-0.05	-0.07	-0.61	0.47	0.11	0.28
0.37	-8.11	-5.50	-1.57	-0.54	-0.05	-0.03	-0.07	0.11	0.00	0.29	-0.11
0.42	7.32	-7.77	-15.76	-13.25	-6.96	-3.25	-1.67	-0.55	0.09	0.07	0.37
0.47	40.27	47.04	39.25	23.02	7.50	-5.43	-15.27	-17.56	-13.06	-5.85	-1.74
0.52	-20.78	-1.52	20.89	41.93	57.50	66.38	60.70	39.92	11.96	-10.11	-20.11

0.57	-13.31	-21.52	-31.05	-36.65	-31.71	-12.58	19.08	52.55	76.53	83.91	76.21
0.62	1.93	0.09	-2.91	-8.85	-15.91	-24.75	-35.64	-41.23	-32.69	-12.04	17.60
0.67	-0.42	-0.42	0.17	0.76	2.51	2.71	-0.59	-4.99	-10.74	-19.76	-33.42
0.72	0.33	-0.10	0.33	0.88	0.37	-0.04	1.36	1.99	3.94	4.38	3.04
0.77	-0.67	-0.02	-0.33	0.11	0.24	0.64	1.24	1.68	2.20	2.13	1.75
0.82	-0.02	-0.17	-0.08	-0.32	0.08	0.78	1.36	1.46	1.73	2.37	2.39
0.87	-0.87	-0.01	-0.60	-0.56	-0.46	0.42	0.57	0.30	1.38	1.36	2.57
0.92	-0.49	-0.26	-0.55	0.27	-0.74	-0.09	0.67	0.75	0.83	1.41	1.97
0.97	-0.72	-0.66	-0.25	-0.70	-0.40	0.03	0.53	1.26	1.27	1.54	1.51
1.02	-0.31	-1.40	0.31	-0.45	-0.69	0.07	0.10	0.23	0.90	1.18	1.50
1.07	-1.01	-0.68	-0.13	-1.51	-0.13	0.05	0.48	0.81	1.12	0.79	1.77
1.12	-0.94	-0.86	-0.65	-1.30	-0.78	-0.12	0.22	0.51	0.02	1.26	1.51
1.17	-0.60	-1.56	-0.63	-0.78	-1.49	-0.58	-0.29	0.39	0.54	0.70	1.21
1.22	-1.96	-1.39	-0.37	-1.35	-1.07	-0.35	0.12	0.06	0.49	0.64	1.18
1.27	-0.53	-0.45	-0.84	0.00	-0.44	0.14	0.35	0.39	0.31	0.90	0.83
1.32	-0.87	-1.27	-1.05	-1.36	-0.88	-1.14	-0.06	-0.39	0.34	0.68	0.69
1.37	-0.82	-0.91	-0.90	-0.67	-1.45	-0.27	0.20	-0.01	0.56	0.66	0.98
1.42	-1.94	-1.01	-0.80	-0.97	-1.72	-0.35	-0.15	-0.50	0.16	0.79	0.98
1.47	-1.47	-0.25	-1.30	-1.02	-0.82	-0.33	-0.22	0.12	0.46	0.89	1.28
1.52	-2.19	-0.76	-1.03	-1.61	-0.87	-0.63	-0.35	-0.18	0.12	0.42	0.80
1.57	-1.57	-0.96	-0.44	-1.04	-1.13	-0.30	-0.28	-0.37	0.30	0.71	0.73
1.62	-0.98	-0.74	-0.89	-0.65	-0.52	-0.64	0.08	0.04	0.30	0.68	0.94
1.67	-1.89	-1.80	-1.08	-0.78	-1.17	-0.65	-0.12	-0.29	0.26	0.79	0.76
1.72	-0.32	-1.35	-0.60	-1.01	-0.35	-0.05	-0.04	-0.03	0.56	0.68	0.97
1.77	-0.62	-0.40	-0.74	-1.39	-1.12	-0.65	0.30	0.17	0.26	0.43	0.79
1.82	-1.06	-1.22	-1.06	-1.84	-1.01	-0.84	0.10	-0.43	0.00	0.44	0.68
1.87	-1.50	-0.66	-0.78	-1.90	-1.50	-0.33	0.00	-0.07	0.14	0.71	0.88
1.92	-1.33	-0.82	-0.52	-0.96	-0.57	-0.36	-0.44	0.39	0.44	0.61	1.08
1.97	-0.76	-0.91	-0.95	-0.69	-0.94	-0.16	-0.12	-0.02	0.70	0.75	1.20
2.02	-1.35	-1.49	-0.97	-1.17	-0.62	-0.04	-0.39	-0.19	0.29	0.59	1.23
2.07	-0.97	-1.25	-0.76	-0.89	-1.44	-0.26	-0.21	0.02	0.46	0.66	1.13
2.12	-1.38	-0.82	-0.74	-1.31	-1.58	-0.74	-0.53	-0.44	0.50	0.53	0.76
2.17	-1.72	-0.54	-0.42	-1.57	-1.68	-0.52	-0.36	-0.56	-0.01	0.61	1.17
2.22	-1.87	-0.86	-0.91	-0.86	-1.16	-0.44	-0.25	-0.17	0.35	0.96	1.06
2.27	-1.75	-1.72	-0.63	-1.50	-1.42	-0.57	-0.09	-0.29	0.08	0.43	1.20
2.32	-1.54	-2.11	-1.79	-0.52	-1.34	-0.84	-0.50	-0.93	-0.22	0.38	0.79
2.37	-0.82	-1.37	-1.04	-1.68	-0.98	-0.47	-0.12	-0.06	-0.04	0.83	1.00
2.42	-1.42	-1.34	-1.54	-1.06	-0.78	-0.28	-0.58	0.13	0.16	0.09	0.42
2.47	-1.59	-0.67	-1.57	-1.11	-0.94	-0.76	-0.06	-0.15	0.15	0.32	0.76
2.52	-1.61	-1.05	-1.93	-1.07	-1.36	-0.81	-0.25	-0.45	0.14	0.32	0.77
2.57	-1.72	-1.63	-1.64	-1.38	-0.39	-0.77	-0.23	-0.24	0.28	0.71	0.90
2.62	-2.24	-1.82	-1.84	-1.74	-1.19	-0.80	-0.61	-0.28	-0.17	0.58	1.00
2.67	-2.30	-1.57	-0.70	-1.69	-0.79	-0.64	0.42	-0.14	0.54	0.92	0.63
2.72	-1.81	-1.71	-1.43	-0.72	-0.69	-0.44	0.25	-0.02	0.36	0.39	1.02
2.77	-1.41	-0.99	-1.69	-0.97	-1.27	-0.82	-0.36	-0.08	0.07	0.55	1.14
2.82	-0.96	-1.96	-1.03	-1.88	-0.75	0.19	0.09	-0.64	-0.35	0.45	0.88
2.87	-1.17	-1.15	-1.10	-1.28	-1.24	-1.01	-0.51	0.17	0.12	0.40	0.55
2.92	-1.53	-1.40	-0.88	-0.89	-1.91	-0.47	-0.22	-0.72	0.29	0.27	0.86
2.97	-1.39	-1.34	-0.55	-1.78	-1.03	-0.59	-0.45	0.18	0.21	0.58	0.61
3.02	-0.96	-1.43	-1.32	-1.24	-0.61	-0.76	-0.01	0.02	0.13	0.76	0.88

3.07	-1.90	-1.32	-1.03	-1.91	-1.79	-0.63	-0.53	0.03	-0.08	0.58	0.95
3.12	-1.89	-1.26	-1.17	-0.64	-1.54	-1.22	-0.09	-0.18	0.27	0.35	0.67
3.17	-0.47	-1.15	-0.60	-1.56	-1.41	0.10	-0.21	-0.13	0.16	0.88	0.63
3.22	-2.11	-0.93	-1.28	-0.94	-1.69	-1.02	-0.19	-0.04	0.28	0.66	0.84
3.42	-1.22	-1.67	-1.48	-2.10	-1.55	-1.06	-0.45	-0.02	-0.65	-0.36	0.99
3.62	-2.42	-1.54	-0.54	-1.00	-1.60	-0.51	-0.55	-0.42	0.27	0.28	0.79
3.82	-1.56	-0.71	-0.22	-1.09	-0.90	-0.60	0.31	0.00	0.04	-0.12	0.78
4.02	-1.03	-1.84	-1.36	-0.99	-1.67	-0.83	-0.57	-0.11	0.07	0.65	0.86
4.22	-1.51	-1.14	-1.38	-1.22	-1.03	-0.61	-0.60	-0.15	0.27	0.12	1.06
4.42	-1.09	-0.74	-1.59	-1.66	-1.04	-0.78	-0.49	-0.05	0.41	0.39	0.59
4.62	-0.95	-1.63	-1.39	-1.86	-0.58	-0.93	-0.54	-0.42	-0.07	0.16	0.72
4.82	-1.40	-2.09	-1.25	-1.89	-1.36	-1.15	-0.25	-0.44	-0.04	0.09	0.65
5.02	-2.25	-1.90	-1.55	-2.06	-1.73	-0.91	-0.86	-0.32	-0.09	0.29	0.39
5.22	-1.82	-0.63	-1.31	-0.96	-1.59	-0.97	-0.51	-0.14	0.05	0.07	0.81
5.42	-0.63	-1.92	-1.72	-1.05	-1.67	-0.89	-0.54	-0.57	-0.26	0.22	0.57
5.62	-3.00	-2.11	-1.33	-1.14	-1.27	-1.06	-0.37	-0.83	-0.19	0.44	0.54
5.82	-2.01	-1.96	-0.84	-1.70	-1.57	-1.56	-0.56	-0.53	-0.03	0.20	0.56
6.02	-2.00	-0.87	-1.60	-2.07	-1.09	-1.51	-0.64	-0.51	-0.28	0.15	0.57
6.22	-1.72	-1.24	-1.14	-1.49	-1.80	-1.07	-0.45	-0.46	-0.06	-0.04	0.29
6.42	-1.75	-1.55	-1.48	-1.93	-1.37	-0.88	-1.05	-0.55	-0.37	0.10	0.85
6.62	-2.40	-2.17	-1.68	-2.37	-1.35	-0.90	-0.63	-0.43	0.11	0.70	0.46
6.82	-1.86	-2.39	-1.57	-1.78	-2.05	-0.26	-0.81	-0.61	-0.04	0.30	0.57
7.02	-2.31	-2.22	-1.95	-1.32	-1.61	-0.72	-0.53	-0.67	0.22	0.14	0.71
7.22	-1.05	-1.36	-1.09	-2.25	-1.76	-0.83	-0.93	-0.26	0.10	0.17	0.64
7.42	-2.55	-1.65	-2.16	-1.70	-1.71	-0.76	-0.58	-0.50	-0.42	-0.04	0.42
7.62	-2.17	-1.18	-2.09	-1.67	-1.59	-0.90	-0.41	-0.66	-0.03	0.45	0.48
7.82	-2.23	-2.01	-1.71	-1.86	-2.05	-1.24	-0.28	-0.04	-0.37	0.43	0.50
8.02	-1.10	-1.64	-1.36	-1.82	-1.90	-0.77	-0.78	-0.89	-0.29	0.00	0.42
8.22	-1.19	-2.45	-1.47	-1.55	-2.10	-0.93	-0.78	-0.76	-0.24	-0.37	0.34
8.42	-2.83	-1.40	-1.83	-1.83	-1.40	-1.33	-0.40	-0.67	-0.95	-0.05	0.27
8.62	-1.66	-1.81	-0.93	-1.22	-1.66	-1.29	-0.51	-0.40	-0.04	-0.10	0.23
8.82	-1.27	-1.76	-1.87	-1.41	-1.75	-1.55	-0.88	-0.37	-0.65	-0.17	0.79
9.02	-1.73	-1.76	-1.11	-1.82	-1.94	-0.65	-0.55	-0.68	-0.34	-0.03	0.08
9.22	-1.92	-1.32	-1.40	-1.10	-1.62	-1.17	-0.97	-0.62	-0.29	0.23	0.61
9.42	-1.13	-2.22	-1.76	-2.30	-1.74	-1.19	-0.60	-0.74	-0.33	-0.33	0.11
9.62	-2.16	-2.11	-2.14	-2.09	-1.89	-1.05	-0.63	-0.77	-0.79	0.30	0.37
9.82	-2.19	-1.13	-1.62	-2.16	-0.76	-1.07	-0.75	-0.77	-0.30	0.20	0.54
10.02	-1.67	-1.67	-1.90	-1.49	-1.47	-1.21	-0.62	-0.78	-0.36	-0.31	0.38
10.22	-1.94	-1.37	-2.32	-2.42	-1.47	-1.51	-1.07	-0.74	-0.47	-0.10	0.40
10.42	-2.44	-1.18	-1.12	-1.73	-2.26	-1.06	-1.00	-0.94	-0.47	0.06	0.70
10.62	-1.96	-1.65	-1.44	-1.61	-1.78	-1.44	-1.01	-0.75	-0.13	-0.01	0.19
10.82	-1.51	-1.20	-1.67	-1.60	-1.91	-1.40	-0.43	-0.63	-0.56	0.29	0.25
11.02	-1.64	-2.37	-1.53	-1.29	-1.91	-1.18	-0.72	-0.68	-0.46	-0.29	0.00
11.22	-1.77	-1.53	-1.60	-1.70	-1.69	-1.36	-1.04	-0.60	-0.74	-0.14	-0.10
11.42	-1.89	-1.19	-1.60	-1.58	-2.21	-0.86	-0.52	-1.04	-0.62	-0.36	0.04
11.62	-2.24	-1.44	-0.98	-2.13	-1.62	-0.88	-0.91	-0.95	-0.30	-0.14	0.23
11.82	-2.39	-1.73	-1.71	-2.06	-1.36	-1.76	-0.55	-0.89	-0.06	0.14	0.28
12.02	-1.98	-1.45	-1.52	-1.88	-1.57	-1.10	-0.91	-0.58	-0.46	0.03	0.59
12.22	-2.54	-1.23	-1.95	-2.05	-2.01	-1.24	-0.62	-0.56	-0.52	0.30	0.29
12.42	-2.42	-1.50	-2.39	-1.91	-1.98	-0.71	-1.11	-0.68	-0.22	0.02	0.39

12.62	-2.54	-1.78	-1.41	-2.02	-1.91	-1.15	-0.59	-0.71	-1.12	0.26	0.32
12.82	-1.72	-1.64	-1.40	-1.81	-1.49	-1.09	-0.58	-0.93	-0.37	-0.12	0.27
13.02	-1.35	-1.76	-1.41	-1.34	-1.84	-0.76	-1.04	-0.45	-0.17	0.33	0.23
13.22	-1.42	-1.79	-1.12	-1.75	-1.95	-1.62	-0.84	-0.71	-0.97	-0.02	0.11
13.42	-2.15	-2.00	-2.12	-1.91	-1.76	-1.68	-1.48	-0.76	-0.83	-0.08	-0.03
13.50	-2.36	-2.12	-1.43	-1.57	-1.78	-1.42	-0.89	-0.74	-0.86	-0.48	0.48
13.59	-1.46	-2.11	-1.75	-1.55	-2.28	-0.91	-1.17	-1.17	-0.45	0.01	0.29
13.68	-1.63	-2.06	-1.94	-2.11	-1.87	-1.40	-1.08	-0.86	-0.81	-0.48	0.23
13.78	-2.60	-2.10	-1.76	-1.94	-2.54	-1.48	-1.31	-1.00	-0.65	-0.30	0.15
13.88	-2.09	-0.77	-1.88	-2.05	-1.77	-1.41	-0.90	-0.91	-0.07	0.10	0.14
14.00	-1.63	-1.28	-1.87	-2.13	-1.67	-1.73	-0.88	-0.44	-0.32	0.09	0.32
14.12	-2.30	-2.02	-2.42	-2.27	-1.90	-1.33	-1.03	-0.44	-0.69	-0.03	0.12
14.25	-1.65	-2.00	-0.65	-2.07	-1.89	-1.27	-1.03	-0.83	-0.33	0.09	-0.11
14.40	-2.35	-1.17	-2.13	-1.78	-2.20	-0.92	-0.52	-1.21	-0.48	-0.04	0.15
14.55	-3.07	-2.29	-1.97	-2.12	-1.40	-1.41	-1.35	-1.01	-0.75	-0.32	0.06
14.72	-2.07	-2.33	-1.94	-1.70	-2.44	-1.83	-1.15	-1.25	-0.93	-0.24	-0.17
14.90	-1.61	-1.20	-1.09	-2.15	-1.95	-1.55	-0.82	-0.85	-0.56	0.01	0.14
15.09	-1.76	-2.72	-2.65	-2.14	-1.92	-1.44	-0.93	-0.84	-0.66	-0.24	0.34
15.30	-2.73	-2.57	-1.58	-2.15	-1.67	-0.79	-0.95	-1.06	-0.15	-0.42	0.09
15.53	-1.54	-2.04	-1.58	-2.52	-1.29	-1.02	-0.96	-1.01	-0.62	-0.18	0.15
15.77	-2.37	-2.08	-2.52	-1.42	-1.97	-1.82	-0.54	-0.90	-0.99	-0.48	0.17
16.04	-1.84	-1.63	-2.63	-2.22	-1.72	-1.24	-1.28	-1.25	-0.84	-0.28	0.27
16.32	-1.16	-2.08	-1.97	-2.50	-1.99	-1.40	-1.42	-0.85	-0.62	0.08	-0.25
16.63	-2.36	-1.89	-1.43	-2.07	-2.17	-1.73	-1.14	-1.36	-0.08	-0.21	0.22
16.96	-1.22	-1.94	-2.52	-1.43	-2.46	-2.11	-0.81	-1.18	-1.31	-0.25	0.30
17.31	-1.41	-1.41	-2.08	-1.52	-1.15	-1.59	-1.29	-0.54	-0.46	-0.50	0.33
17.70	-2.09	-1.51	-1.79	-2.43	-2.24	-0.79	-0.82	-1.10	0.05	0.08	-0.23
18.11	-1.66	-2.79	-2.83	-1.73	-2.20	-1.68	-1.03	-0.48	-0.82	-0.54	-0.12
18.56	-1.72	-2.22	-1.35	-1.63	-1.96	-1.04	-1.43	-1.10	-0.73	-0.19	-0.32
19.04	-1.80	-2.16	-1.75	-1.66	-1.85	-1.89	-1.37	-0.89	-0.10	-0.51	0.17
19.56	-2.65	-2.11	-1.64	-1.89	-2.10	-1.18	-0.88	-0.79	-0.44	-0.24	0.06
20.12	-1.71	-2.13	-2.61	-2.44	-2.83	-1.29	-1.54	-0.88	-0.95	-0.42	0.24
20.72	-1.68	-1.30	-1.57	-2.34	-2.12	-1.25	-0.82	-1.24	-0.42	-0.15	0.25
21.37	-1.98	-1.81	-1.78	-1.74	-1.50	-1.79	-1.67	-1.12	-0.69	-0.44	0.25
22.08	-2.33	-1.85	-1.14	-1.89	-2.78	-1.39	-0.67	-1.29	-0.63	0.16	0.17
22.84	-1.57	-1.72	-1.56	-1.79	-1.46	-2.02	-0.97	-1.58	-0.86	-0.21	0.12
23.65	-1.24	-1.73	-1.32	-2.18	-1.29	-0.56	-0.76	-1.22	-0.73	-0.04	0.24
24.54	-2.14	-1.81	-1.83	-2.39	-1.38	-1.06	-0.64	-0.76	-0.97	-0.43	0.05
25.49	-2.38	-2.29	-1.42	-1.91	-2.07	-1.22	-1.28	-1.01	-0.72	0.15	0.18
26.51	-2.68	-2.04	-2.08	-1.87	-1.77	-1.96	-0.93	-1.45	-0.53	0.00	-0.01
27.62	-1.90	-2.21	-1.73	-2.70	-2.12	-1.66	-0.94	-1.57	-0.31	-0.40	0.06
28.81	-1.27	-2.12	-2.38	-2.13	-2.04	-1.87	-0.67	-1.21	-0.49	-0.45	0.28
30.10	-2.03	-2.20	-1.73	-2.38	-1.77	-1.39	-1.24	-0.94	-0.53	0.19	0.23
31.49	-2.14	-1.83	-1.21	-1.93	-2.23	-1.52	-0.88	-0.99	-0.30	-0.03	0.42
32.98	-1.84	-2.56	-1.79	-1.95	-1.94	-0.85	-0.88	-0.66	-0.82	-0.21	0.49
34.60	-2.43	-2.27	-1.96	-1.89	-1.90	-1.43	-1.30	-1.20	-0.78	-0.50	-0.11
36.34	-2.05	-2.51	-2.14	-2.15	-2.76	-1.25	-1.35	-1.28	-0.49	-0.03	0.06
38.21	-1.80	-2.04	-1.40	-1.99	-1.47	-1.69	-1.18	-1.11	-0.61	-0.24	0.16
40.24	-2.43	-2.69	-1.65	-2.07	-2.52	-1.31	-1.30	-0.83	-0.50	-0.01	-0.32
42.42	-2.18	-2.11	-1.67	-2.39	-2.02	-1.76	-0.93	-1.20	-0.56	-0.83	0.05

44.78	-2.50	-1.81	-2.48	-1.53	-1.89	-1.19	-1.29	-1.16	-0.63	-0.24	0.24
47.32	-2.19	-2.33	-2.90	-2.45	-2.02	-1.11	-1.47	-0.94	-0.39	-0.54	0.36
50.06	-1.00	-1.77	-1.31	-1.92	-2.10	-1.51	-0.91	-1.10	0.16	0.16	0.28
53.01	-1.89	-1.71	-1.64	-2.18	-2.01	-1.84	-1.04	-1.30	-0.37	0.33	0.13
56.20	-2.34	-3.33	-2.67	-2.03	-2.14	-1.38	-1.31	-1.11	-0.37	0.40	0.31
59.64	-1.84	-1.57	-2.41	-2.12	-2.31	-0.85	-1.03	-0.79	-0.42	-0.33	0.17
63.34	-2.03	-1.89	-1.22	-1.88	-1.75	-1.06	-0.83	-0.67	-0.25	0.39	0.13
67.34	-3.03	-2.03	-1.62	-1.35	-1.94	-1.22	-0.62	-1.01	-0.34	0.27	-0.06
71.65	-1.89	-1.90	-1.62	-2.10	-2.16	-1.14	-0.61	-0.97	-0.84	-0.37	0.43
76.30	-2.10	-2.61	-1.43	-1.85	-1.39	-0.77	-0.37	-0.63	-0.42	0.02	0.84
81.32	-2.04	-2.11	-1.04	-1.92	-2.02	-1.23	-0.85	-0.52	-0.37	-0.03	0.49
86.73	-2.97	-2.12	-2.36	-1.13	-1.74	-1.09	-0.84	-0.76	-0.11	0.00	0.20
92.56	-1.27	-2.25	-2.32	-1.78	-1.81	-1.13	-0.29	-0.85	-0.45	0.30	0.54
98.85	-1.48	-2.38	-1.05	-1.93	-1.11	-1.07	-1.09	-0.98	-0.41	-0.15	0.45
105.64	-1.73	-1.96	-1.71	-1.75	-1.72	-1.25	-0.76	-0.79	-0.30	-0.17	0.41
112.96	-2.02	-1.22	-2.55	-1.07	-1.87	-1.54	-0.72	-0.85	-0.14	-0.15	0.21
120.85	-2.22	-1.75	-1.15	-1.97	-1.66	-0.86	-0.73	-0.76	-0.23	0.46	0.57
129.36	-1.85	-2.92	-1.75	-1.80	-1.89	-1.29	-0.35	-0.30	-0.13	0.62	0.86
138.55	-2.26	-2.15	-1.54	-1.53	-1.67	-0.42	0.17	-0.41	0.30	0.50	1.09
148.45	-1.87	-1.71	-1.30	-1.82	-1.13	-0.92	-0.59	-0.14	0.25	0.75	0.67
159.13	-2.12	-1.67	-1.19	-1.45	-1.85	-0.85	0.06	-0.09	-0.15	0.89	1.21
170.65	-2.24	-2.42	-1.67	-1.85	-1.51	-1.09	-0.55	-1.05	-0.51	0.36	1.00
183.07	-1.76	-1.56	-1.81	-1.95	-1.77	-1.11	-0.58	-0.75	0.02	0.74	0.74
196.47	-2.27	-2.03	-1.31	-1.98	-1.86	-0.31	-0.10	-0.02	0.19	0.78	0.74
210.91	-3.10	-1.44	-0.93	-2.05	-2.34	-0.42	-0.44	-0.49	0.42	0.74	1.35
226.50	-1.77	-1.29	-1.78	-1.08	-1.82	-1.30	-0.08	0.19	0.69	0.76	1.04
243.31	-2.28	-0.75	-1.41	-1.33	-1.34	-0.86	-0.10	0.34	0.36	0.29	1.61
261.43	-2.10	-2.26	-2.21	-2.11	-1.63	-1.04	-0.12	0.35	0.45	0.93	1.28
280.98	-2.19	-2.09	-1.79	-1.73	-2.10	-0.53	-0.29	-0.04	0.67	0.98	1.46
302.07	-2.03	-1.60	-1.64	-1.13	-0.74	-0.20	-0.02	0.16	0.30	1.11	1.58
324.81	-1.64	-1.96	-1.49	-1.59	-1.79	-1.14	-0.04	0.05	0.55	0.73	1.84
349.33	-1.90	-1.93	-0.59	-1.53	-1.00	-0.82	0.10	-0.03	0.55	1.80	1.37
375.79	-1.24	-1.48	-1.35	-1.42	-1.53	-0.51	0.23	0.37	0.75	1.19	1.88
404.31	-1.62	-1.49	-1.38	-1.49	-0.98	-0.72	-0.11	0.50	0.49	1.24	2.01
435.08	-2.48	-1.69	-1.95	-1.69	-1.81	-0.13	-0.60	0.11	0.46	0.83	1.29
468.27	-1.99	-1.70	-1.04	-1.22	-1.24	-0.68	0.25	0.03	0.76	1.76	2.15
504.06	-1.89	-1.43	-0.98	-1.50	-1.03	-0.36	0.84	0.60	0.60	1.25	2.12
542.66	-1.58	-1.14	-1.29	-1.37	-0.65	-0.23	-0.43	0.72	1.09	1.42	2.35
584.29	-2.76	-1.34	-0.38	-1.44	-0.62	-0.17	0.28	0.70	1.23	1.90	2.34
629.18	-2.49	-1.77	-1.36	-1.25	-0.53	-0.51	-0.02	0.53	0.95	1.45	2.11
677.61	-2.24	-1.55	-1.29	-1.41	-1.63	-0.41	0.19	0.80	0.77	1.77	1.86
729.83	-1.80	-1.18	-1.78	-1.23	-0.93	-0.15	0.53	0.43	1.61	1.49	2.17
786.16	-1.73	-1.12	-0.83	-0.76	-0.53	-0.03	0.59	0.56	1.51	2.04	2.21
846.90	-1.97	-1.27	-1.11	-1.82	-0.98	-0.31	0.49	0.85	1.10	1.44	1.83
912.42	-2.18	-1.65	-1.49	-1.36	-0.54	0.13	0.43	0.43	1.00	1.95	2.40
983.08	-2.00	-1.26	-1.36	-0.70	-0.35	-0.48	0.87	0.77	1.18	1.77	2.38
1059.29	-0.98	-1.67	-0.93	-0.72	-0.88	-0.46	0.33	0.74	1.61	1.49	1.90
1141.48	-2.07	-0.84	-1.31	-1.32	-1.58	-0.10	0.51	0.15	1.48	1.71	2.34
1230.13	-2.23	-2.08	-1.69	-0.35	-0.58	0.48	0.80	0.65	1.01	2.00	2.39
1325.73	-1.37	-0.37	-1.35	-0.93	-1.39	-0.32	0.71	1.10	1.24	2.03	2.89

1428.84	-1.81	-1.70	-1.29	-1.82	-0.62	-0.15	0.35	0.76	1.43	2.23	2.48
1540.05	-0.99	-1.06	-0.89	-0.80	-0.87	0.06	0.55	0.79	1.42	2.07	2.35
1659.99	-0.91	-2.10	-1.26	-1.67	-1.37	-0.19	0.58	0.41	1.21	1.82	2.49
1789.34	-2.63	-2.10	-1.11	-1.17	-0.76	-0.06	0.24	0.58	1.37	1.93	2.36
1928.85	-1.70	-0.88	-0.36	-0.59	-0.24	-0.14	0.62	0.92	1.22	2.07	2.45
2079.31	-2.76	-1.46	-0.48	-0.56	-1.08	-0.02	1.06	0.23	1.24	2.66	2.83
2241.59	-1.54	-2.07	-0.57	-1.79	-0.22	-0.44	0.07	0.73	1.69	2.07	2.82
2416.60	-1.66	-1.58	-1.81	-0.43	-0.85	0.44	0.98	0.70	1.16	1.86	2.51
2605.36	-1.78	-1.44	-0.77	-1.13	-0.07	0.32	0.58	1.19	1.70	2.47	2.70
2808.94	-3.29	-1.10	-0.80	-1.31	-0.85	-0.10	0.31	0.47	1.49	1.83	2.45
3028.50	-2.18	-0.84	-1.16	-1.90	-0.48	0.17	0.26	0.71	1.15	2.18	2.62
3265.30	-1.76	-1.78	-1.34	-1.27	-0.70	-0.45	0.94	0.60	1.22	1.88	2.70
3520.69	-1.31	-1.29	-0.42	-1.17	-0.74	0.11	0.49	1.07	1.82	2.04	2.39
3796.13	-1.47	-1.02	-1.15	-0.89	-0.67	0.37	0.71	0.71	1.49	1.98	2.33

Wavelength (nm) / Time (ps)	349.75	351.18	352.60	354.03	355.45	356.87	358.30	359.72	361.15	362.57	363.99
-3.78	0.34	-0.05	-0.15	-0.02	-0.11	-0.09	0.03	0.03	-0.07	-0.09	-0.08
-3.28	0.48	-0.26	0.21	-0.19	0.08	-0.12	-0.02	-0.06	0.03	0.05	0.16
-2.78	-0.09	0.00	0.05	0.05	0.14	0.23	0.16	0.06	-0.03	-0.23	0.00
-2.28	-0.05	-0.03	0.08	0.26	-0.21	0.12	-0.23	-0.17	0.18	-0.01	-0.12
-1.78	-0.01	0.36	0.22	0.03	0.01	-0.18	0.07	0.22	-0.01	-0.07	-0.05
-1.28	0.03	0.13	0.13	0.06	0.08	0.10	0.25	0.02	0.11	0.30	0.18
-0.78	-0.63	-0.07	-0.30	-0.25	0.02	-0.18	-0.09	-0.05	-0.16	0.01	0.07
-0.28	-0.08	-0.09	-0.23	0.06	0.00	0.13	-0.18	-0.07	-0.04	0.03	-0.16
-0.18	-0.28	-0.21	0.25	0.11	-0.05	0.03	-0.10	-0.04	-0.17	0.14	-0.06
-0.08	0.19	0.26	0.10	0.11	-0.43	-0.02	-0.09	-0.30	-0.07	0.07	0.13
0.02	-0.03	0.28	-0.22	0.08	0.03	-0.25	0.11	-0.41	0.13	-0.20	-0.12
0.12	-0.09	0.02	0.43	0.16	0.09	0.06	-0.06	0.05	0.20	0.07	0.03
0.22	0.25	-0.44	-0.14	-0.26	0.06	-0.18	-0.10	-0.07	-0.04	-0.22	-0.25
0.27	0.38	-0.01	0.04	-0.13	0.02	0.20	0.14	0.04	0.28	0.13	0.00
0.32	0.05	-0.26	-0.07	0.03	-0.16	0.05	-0.06	0.00	-0.03	-0.10	-0.17
0.37	0.01	0.16	0.23	0.10	-0.18	0.22	0.19	0.08	0.10	0.09	0.10
0.42	0.02	0.33	0.07	0.17	0.12	0.20	0.09	-0.09	0.40	0.20	-0.24
0.47	-0.08	-0.11	0.13	0.22	0.21	0.42	0.09	-0.03	0.18	-0.12	-0.08
0.52	-20.01	-14.95	-9.25	-4.42	-1.79	-0.24	0.16	-0.16	0.24	0.16	-0.06
0.57	56.09	29.36	3.33	-16.70	-25.85	-25.29	-18.23	-9.77	-3.35	-0.49	0.18
0.62	51.16	81.46	98.64	96.18	75.54	42.18	4.93	-25.27	-39.18	-35.48	-21.84
0.67	-45.39	-44.04	-25.88	6.24	46.70	86.23	113.26	118.14	99.92	67.43	33.12
0.72	-0.21	-4.20	-10.10	-19.70	-32.64	-38.25	-29.45	-5.23	28.00	62.29	90.21
0.77	2.33	3.63	5.29	6.16	5.83	3.22	0.60	-2.47	-12.05	-28.51	-43.72
0.82	2.84	3.89	4.14	3.54	3.72	4.64	5.81	7.11	8.05	7.94	5.83
0.87	2.64	3.02	3.28	3.62	4.12	4.77	5.36	5.44	5.45	5.66	6.19
0.92	2.32	2.59	2.74	3.50	4.11	4.60	4.64	4.98	5.47	6.22	6.58
0.97	1.80	2.52	2.71	3.32	3.65	4.41	4.50	4.50	5.46	5.94	6.26
1.02	2.26	2.24	2.91	2.85	3.19	3.81	4.17	4.91	5.27	5.62	5.96
1.07	2.08	2.51	2.71	2.86	3.54	3.91	4.26	4.57	5.04	5.31	5.32
1.12	1.70	1.96	2.24	2.80	3.41	3.92	4.50	4.30	4.98	5.35	5.90

1.17	1.43	1.61	2.41	2.61	3.07	3.36	3.41	4.02	4.29	4.68	5.10
1.22	1.69	1.99	2.51	2.55	3.14	3.68	3.65	4.13	4.93	5.35	5.38
1.27	1.20	2.11	2.09	2.57	2.55	3.29	3.81	4.13	4.38	4.80	4.86
1.32	0.98	1.54	1.94	2.17	2.78	3.22	3.39	3.73	4.19	4.55	5.01
1.37	1.43	1.78	1.97	2.14	2.49	3.24	3.61	3.75	4.12	4.62	4.91
1.42	1.07	1.58	2.01	2.34	3.04	3.35	3.80	3.92	4.52	5.08	5.43
1.47	1.48	1.89	2.03	2.41	3.10	3.29	3.80	3.91	4.59	4.90	4.77
1.52	1.21	1.81	2.15	2.64	2.71	3.29	3.76	3.80	4.22	4.62	4.89
1.57	1.31	1.72	1.73	2.63	2.84	3.40	3.70	4.18	4.65	4.95	5.17
1.62	1.24	1.91	2.04	2.48	2.74	3.22	3.38	3.99	4.34	4.59	4.64
1.67	1.12	1.27	2.05	2.19	2.58	3.18	3.56	4.16	4.23	4.48	5.02
1.72	1.43	1.56	2.03	2.02	2.76	3.18	3.51	3.99	4.37	4.42	4.67
1.77	1.57	1.72	2.17	2.33	2.60	2.99	3.56	3.80	4.54	4.85	5.07
1.82	1.01	1.42	1.88	2.14	2.46	3.18	3.41	3.69	4.01	4.42	4.92
1.87	1.59	1.81	1.87	2.40	2.70	3.69	3.58	3.82	4.37	4.51	5.05
1.92	1.50	1.88	2.19	2.83	2.86	3.38	3.68	3.90	4.28	4.57	4.95
1.97	1.28	1.67	2.04	2.47	2.72	2.94	3.14	3.84	4.01	4.44	4.75
2.02	0.93	1.55	2.01	1.82	2.49	3.14	2.88	3.52	3.83	4.10	4.34
2.07	1.13	1.68	2.02	2.51	2.73	3.55	3.71	3.83	4.08	4.81	4.95
2.12	1.07	1.65	1.88	2.19	2.54	3.28	3.34	4.10	4.01	4.68	4.73
2.17	1.03	1.27	1.71	2.35	2.63	3.06	3.52	3.70	4.14	4.54	4.58
2.22	1.39	1.66	2.27	2.18	2.92	3.36	3.63	3.84	4.01	4.35	4.85
2.27	1.28	1.47	2.17	2.47	2.73	3.13	3.63	3.73	4.35	4.33	4.98
2.32	1.06	1.49	1.83	1.90	2.19	2.86	3.30	3.30	3.72	4.34	4.72
2.37	1.32	1.71	2.26	2.55	2.67	3.27	3.67	3.71	4.02	4.41	4.99
2.42	1.40	1.70	1.93	2.37	2.77	3.31	3.28	3.80	4.15	4.32	4.83
2.47	1.11	1.85	2.20	2.57	2.54	3.26	3.74	3.77	4.32	4.67	4.87
2.52	1.02	1.96	1.94	2.47	2.57	3.05	3.64	4.03	4.39	4.57	4.84
2.57	1.47	1.53	2.04	2.48	2.77	3.62	3.63	3.73	4.19	4.49	4.83
2.62	1.23	1.55	1.86	2.40	2.84	3.14	3.55	3.90	4.20	4.42	4.97
2.67	1.36	1.47	2.01	2.42	2.56	2.85	3.41	4.02	4.23	4.42	4.45
2.72	1.66	1.72	2.09	2.35	2.69	3.23	3.44	3.71	4.06	4.62	4.79
2.77	1.31	1.41	1.75	2.48	2.89	3.31	3.43	3.77	4.04	4.29	4.68
2.82	0.86	1.50	1.72	2.14	2.79	3.05	3.20	3.78	4.12	4.28	4.68
2.87	1.02	1.58	1.93	1.90	2.74	2.98	3.41	3.62	4.25	4.27	4.54
2.92	1.11	1.30	2.15	1.99	2.55	3.20	3.44	3.77	4.05	4.40	4.96
2.97	0.76	1.25	2.05	2.01	2.65	3.20	3.17	3.69	3.91	4.41	4.77
3.02	0.85	1.51	2.21	2.22	2.70	3.04	3.45	3.81	4.39	4.64	5.07
3.07	1.02	1.15	1.66	2.34	2.53	3.13	3.47	3.45	4.03	4.30	4.51
3.12	1.27	1.54	1.64	2.26	2.71	2.87	3.05	3.49	3.99	4.16	4.75
3.17	1.08	1.43	2.02	2.26	2.70	3.07	3.71	3.66	4.44	4.51	4.77
3.22	1.33	1.66	1.96	2.38	2.62	3.40	3.51	3.85	4.37	4.70	4.93
3.42	0.92	1.11	1.75	1.83	2.38	3.07	3.12	3.49	3.95	4.33	4.63
3.62	0.93	1.77	1.82	1.88	2.65	2.98	3.30	3.71	4.24	4.45	4.72
3.82	1.18	1.50	1.60	2.23	2.54	2.71	3.20	3.42	3.87	4.08	4.46
4.02	1.25	1.71	1.73	2.35	2.88	3.34	3.71	3.87	4.16	4.72	4.83
4.22	1.09	1.48	1.84	1.96	2.54	2.71	3.29	3.44	3.98	4.35	4.60
4.42	1.19	1.38	1.80	2.39	2.45	3.35	3.46	3.71	4.26	4.16	4.77
4.62	0.76	1.12	1.72	1.88	2.58	2.66	3.12	3.23	3.71	3.93	4.74
4.82	1.39	1.51	1.87	2.43	2.35	3.04	3.24	3.61	4.07	4.40	4.65

5.02	1.06	1.64	1.90	2.20	2.74	2.99	3.32	3.61	3.89	4.48	4.50
5.22	1.31	1.21	1.61	2.45	2.54	2.84	3.12	3.37	3.86	4.16	4.52
5.42	0.65	1.46	1.65	2.20	2.32	2.89	3.31	3.40	3.95	4.44	4.63
5.62	0.77	1.35	1.84	1.99	2.35	2.85	3.27	3.40	3.87	4.29	4.90
5.82	0.63	1.43	1.73	1.91	2.28	2.76	3.09	3.49	4.12	4.27	4.57
6.02	1.12	1.48	1.44	2.13	2.23	2.97	3.24	3.54	3.76	3.97	4.40
6.22	0.82	1.40	1.95	2.27	2.33	2.87	3.32	3.40	4.06	4.39	4.54
6.42	0.98	1.06	1.48	2.10	2.19	3.09	3.29	3.47	3.99	4.18	4.54
6.62	0.90	1.38	1.86	2.08	2.21	2.96	3.14	3.35	4.11	4.42	4.52
6.82	0.68	1.02	1.60	1.83	2.27	2.66	2.76	3.31	3.75	4.14	4.46
7.02	0.91	1.64	1.70	2.41	2.23	2.99	3.22	3.56	3.84	4.43	4.69
7.22	1.41	1.26	1.71	1.81	2.25	2.66	3.05	3.14	3.71	4.21	4.43
7.42	1.41	1.98	1.86	2.14	2.62	2.99	3.17	3.85	4.30	4.41	4.69
7.62	1.21	1.20	1.32	2.19	2.38	2.95	3.31	3.39	3.72	3.93	4.64
7.82	0.96	1.02	1.59	1.98	2.11	2.69	3.05	3.35	3.89	4.12	4.36
8.02	0.66	0.93	1.74	1.76	2.27	2.65	2.84	3.57	3.62	4.13	4.53
8.22	0.95	1.00	1.49	1.97	2.33	3.02	2.99	3.17	3.78	4.26	4.28
8.42	0.77	1.20	1.60	1.80	2.61	2.65	2.95	3.42	3.63	4.02	4.51
8.62	0.68	1.15	1.64	1.40	2.13	2.64	3.04	3.31	3.72	4.10	4.50
8.82	0.52	1.09	1.63	2.01	2.14	2.55	3.08	3.31	3.78	3.96	4.36
9.02	0.85	1.50	1.56	1.93	2.07	2.82	2.99	3.35	3.63	4.11	4.16
9.22	0.95	1.14	1.41	1.89	2.22	3.01	2.99	3.34	3.86	3.84	4.61
9.42	0.61	0.75	1.44	1.46	2.09	2.27	2.99	3.16	3.75	3.81	4.29
9.62	0.64	1.14	1.48	1.95	2.18	2.93	2.89	3.21	3.84	4.25	4.24
9.82	0.69	1.18	1.39	1.61	2.28	2.73	2.71	3.06	3.57	3.74	4.07
10.02	0.96	0.98	1.09	1.73	2.17	2.48	2.43	2.96	3.42	3.89	3.96
10.22	0.99	0.77	1.39	1.63	2.04	2.67	2.91	3.14	3.64	4.07	4.16
10.42	0.95	1.40	1.55	1.79	2.24	2.59	2.91	3.15	3.62	3.83	4.11
10.62	0.82	1.06	1.23	1.83	2.31	2.62	2.73	3.52	3.70	3.99	4.46
10.82	0.55	1.23	1.51	1.93	1.84	2.87	2.75	3.11	3.70	3.71	4.15
11.02	0.79	0.88	0.95	1.74	1.75	2.38	2.76	3.17	3.58	3.77	4.21
11.22	0.58	0.98	1.23	1.49	1.90	2.09	2.57	2.87	3.46	3.62	3.95
11.42	0.57	0.44	1.01	1.53	1.90	2.37	2.77	2.94	3.15	3.66	3.88
11.62	0.93	1.21	1.26	1.84	2.01	2.95	3.08	3.18	3.56	3.85	4.08
11.82	0.79	1.06	1.31	1.78	2.00	2.53	2.68	3.23	3.60	3.82	4.31
12.02	0.50	1.22	1.50	1.55	2.16	2.54	2.60	2.95	3.50	3.63	4.15
12.22	0.71	0.89	1.49	2.00	2.30	2.60	3.02	3.53	3.51	4.05	4.35
12.42	0.52	1.15	1.29	1.58	2.21	2.77	2.99	3.22	3.75	3.99	4.24
12.62	0.69	0.80	1.21	1.72	1.90	2.41	2.75	3.08	3.31	3.78	3.93
12.82	0.46	1.11	1.35	1.59	1.76	2.36	2.40	2.63	3.27	3.62	3.65
13.02	0.83	1.35	1.31	1.51	1.98	2.22	2.93	3.00	3.41	3.87	4.03
13.22	0.39	1.00	1.64	1.69	1.95	2.25	2.95	3.06	3.63	4.00	4.27
13.42	0.53	0.80	1.19	1.71	2.13	2.61	2.74	2.98	3.42	4.01	4.28
13.50	0.45	0.87	1.34	1.50	1.76	2.35	2.82	2.96	3.39	3.58	3.89
13.59	0.75	0.90	1.38	1.41	2.23	2.41	2.68	3.23	3.64	3.73	4.08
13.68	0.56	0.84	1.18	1.52	1.78	2.50	2.81	3.12	3.41	3.74	4.05
13.78	0.21	1.10	1.27	1.83	1.82	2.56	2.83	3.19	3.35	3.85	4.20
13.88	0.50	1.04	1.47	1.76	2.15	2.80	2.87	3.31	3.57	3.90	4.14
14.00	0.42	0.81	1.44	1.62	2.01	2.31	2.83	3.10	3.48	3.80	3.99
14.12	0.70	0.84	1.46	1.79	2.01	2.71	3.08	3.40	3.64	3.70	4.38

14.25	0.37	1.02	1.13	1.76	2.17	2.27	2.96	3.14	3.56	3.83	4.18
14.40	0.57	0.90	1.12	1.94	2.38	2.65	2.96	3.19	3.72	4.16	4.17
14.55	0.62	0.95	1.44	1.57	2.07	2.37	2.93	3.17	3.79	3.73	4.23
14.72	0.75	0.58	0.87	1.59	1.84	2.50	2.71	3.28	3.67	4.06	4.17
14.90	0.40	0.79	1.25	1.32	1.93	2.28	2.51	2.71	3.26	3.50	3.85
15.09	0.37	0.76	1.40	1.76	1.99	2.23	2.59	3.02	3.12	3.81	3.94
15.30	0.68	0.89	0.93	1.75	1.92	2.57	2.76	3.17	3.38	3.83	4.12
15.53	0.74	1.11	1.55	1.86	2.34	2.54	2.87	3.42	4.17	4.00	4.34
15.77	0.73	0.88	1.38	1.71	2.02	2.47	3.01	2.97	3.70	3.95	4.13
16.04	0.56	0.90	1.44	1.88	2.18	2.41	3.00	3.16	3.66	3.82	4.23
16.32	0.32	1.15	1.34	1.54	1.84	2.28	2.67	2.73	3.47	3.56	4.10
16.63	0.85	1.09	1.27	1.50	1.90	2.49	2.74	3.11	3.64	3.74	4.20
16.96	0.27	0.47	1.26	1.71	1.75	2.50	2.69	2.93	3.34	4.03	4.03
17.31	0.46	0.85	1.31	1.63	2.05	2.14	2.97	3.00	3.61	4.09	4.03
17.70	0.34	1.21	1.41	1.57	2.05	2.42	2.69	3.05	3.61	3.74	4.25
18.11	0.36	0.64	1.54	1.55	1.85	2.24	2.75	2.88	3.50	3.71	4.02
18.56	0.93	1.25	0.85	1.68	1.87	2.32	2.64	3.06	3.51	3.62	3.84
19.04	0.67	0.50	1.43	1.80	2.04	2.37	2.75	2.68	3.34	3.62	3.96
19.56	0.04	1.39	1.09	1.70	1.95	2.58	2.69	3.20	3.45	3.82	4.12
20.12	-0.01	0.90	0.88	1.67	1.91	2.42	2.71	3.28	3.54	3.76	4.12
20.72	0.83	1.03	1.16	1.92	2.13	2.65	2.70	3.36	3.79	3.96	4.27
21.37	0.29	0.93	1.21	1.50	1.74	2.64	2.66	3.18	3.61	4.13	4.11
22.08	0.60	1.31	1.79	1.65	2.51	3.02	3.38	3.66	4.06	4.14	4.49
22.84	0.58	1.01	1.14	1.54	1.90	2.50	2.87	3.16	3.61	3.72	4.01
23.65	0.63	1.26	1.22	1.86	1.97	2.65	2.89	3.13	3.47	3.70	4.05
24.54	0.41	0.63	1.43	1.56	2.14	2.18	2.86	2.87	3.59	3.77	4.16
25.49	0.61	1.04	1.46	2.02	2.12	2.69	3.13	3.11	3.69	3.89	4.52
26.51	0.14	0.85	1.33	1.63	1.82	2.24	2.93	3.03	3.57	3.76	3.95
27.62	0.61	0.79	1.08	1.60	1.90	2.21	2.78	3.10	3.81	3.82	4.22
28.81	0.42	0.87	1.34	1.97	2.04	2.52	2.93	3.37	3.66	3.75	4.29
30.10	0.30	0.92	1.11	1.59	1.77	2.47	2.70	2.88	3.59	3.63	3.94
31.49	1.05	0.88	1.24	1.16	2.07	2.30	2.82	3.18	3.43	3.76	4.20
32.98	0.33	0.69	1.49	1.41	2.10	2.65	2.82	3.17	3.35	3.84	4.07
34.60	0.31	0.73	1.89	1.58	1.81	2.66	3.00	3.12	3.51	3.76	3.96
36.34	0.61	0.87	1.61	1.86	2.14	2.53	2.54	3.13	3.73	4.16	4.31
38.21	0.73	0.94	1.27	1.64	1.96	2.71	2.85	3.22	3.46	3.70	4.10
40.24	0.80	1.01	1.30	1.72	2.14	2.70	2.89	3.19	3.76	3.77	4.18
42.42	0.63	0.60	1.02	1.51	1.79	2.36	2.75	2.82	3.23	3.89	4.16
44.78	0.72	0.87	1.36	1.88	2.19	2.96	2.91	3.21	3.54	3.95	4.11
47.32	0.38	1.06	1.12	1.94	2.37	2.60	3.11	3.40	3.67	4.23	4.38
50.06	0.90	0.96	1.52	1.97	2.43	2.71	3.26	3.43	3.81	4.29	4.54
53.01	0.46	1.21	1.75	1.77	2.05	2.80	3.07	3.07	3.53	3.91	4.43
56.20	0.85	1.01	1.44	1.74	2.19	2.94	2.93	3.45	3.70	4.20	4.45
59.64	0.45	0.97	1.10	1.68	2.16	2.47	2.81	3.28	3.71	3.79	4.28
63.34	0.65	1.38	1.73	2.07	2.22	2.95	3.36	3.43	3.86	4.12	4.56
67.34	0.64	0.90	1.29	1.88	2.38	2.75	2.94	3.38	3.68	4.05	4.19
71.65	0.55	1.27	1.31	1.76	2.24	2.76	3.32	3.24	3.85	4.20	4.45
76.30	0.78	0.98	1.94	1.93	2.52	2.95	3.23	3.39	4.03	3.98	4.75
81.32	0.81	1.47	1.84	2.05	2.69	3.23	3.75	3.55	3.96	4.20	4.75
86.73	0.74	0.92	1.54	2.14	2.46	3.00	3.60	3.55	3.94	4.29	4.69

92.56	0.70	1.52	1.95	1.95	2.71	2.79	2.99	3.82	4.21	4.25	4.43
98.85	1.08	1.27	1.74	2.11	2.53	3.13	3.31	3.56	4.14	4.35	4.41
105.64	0.81	1.50	1.89	2.28	2.88	3.33	3.30	3.93	4.17	4.56	4.65
112.96	1.07	1.69	1.87	2.38	2.91	3.23	3.73	3.69	4.11	4.51	4.81
120.85	1.14	1.47	1.60	2.35	2.23	3.00	3.34	3.54	4.10	4.32	4.57
129.36	1.01	1.83	2.24	2.80	2.91	3.88	3.99	4.23	4.47	4.80	5.09
138.55	1.41	1.55	2.27	2.65	3.08	3.59	3.54	3.96	4.57	4.66	5.04
148.45	1.35	1.87	2.43	2.75	2.96	3.58	4.00	4.21	4.43	4.93	5.31
159.13	1.34	1.83	2.28	2.46	3.34	3.64	4.07	4.33	4.76	5.20	5.19
170.65	1.40	1.48	2.41	2.80	3.07	3.47	3.84	4.10	4.69	4.98	5.18
183.07	1.68	2.20	2.30	2.81	3.53	3.79	3.78	4.44	4.62	5.23	5.15
196.47	1.57	1.95	2.65	2.76	3.22	3.55	4.02	4.14	4.66	5.04	5.32
210.91	1.61	2.08	2.55	3.03	3.42	3.67	4.07	4.56	4.92	4.97	5.45
226.50	1.70	2.11	2.65	3.09	3.61	3.75	4.39	4.30	4.77	4.95	5.19
243.31	1.61	2.52	2.85	2.98	3.41	4.00	4.54	4.66	4.79	5.23	5.55
261.43	1.96	2.38	2.83	3.33	3.44	4.08	4.61	4.73	5.15	5.66	5.71
280.98	1.79	2.04	2.83	3.35	3.62	4.39	4.62	4.76	5.37	5.58	5.77
302.07	1.88	2.31	3.07	3.50	3.86	4.07	4.62	4.64	5.29	5.55	5.85
324.81	2.18	2.43	2.90	3.24	3.67	4.17	4.73	5.14	5.40	5.44	6.01
349.33	2.33	2.54	3.15	3.44	4.18	4.70	4.85	4.92	5.51	5.53	5.74
375.79	1.96	2.78	3.52	3.57	4.02	4.32	4.98	5.20	5.73	5.94	5.98
404.31	2.38	3.03	3.24	3.57	3.95	4.82	5.03	5.27	5.68	5.97	5.78
435.08	2.26	2.67	3.14	3.69	4.39	5.06	5.23	5.51	5.80	6.11	6.67
468.27	2.74	2.96	3.67	4.16	4.50	4.86	5.29	5.54	6.21	6.41	6.38
504.06	2.42	2.89	3.34	3.84	4.48	4.78	4.99	5.58	5.80	6.16	6.45
542.66	2.33	2.96	3.77	4.02	4.71	5.19	5.27	5.59	6.11	6.14	6.85
584.29	2.79	3.32	3.72	4.33	4.52	5.09	5.50	5.38	6.14	6.41	6.66
629.18	2.59	3.08	3.78	4.04	4.70	4.87	5.69	5.56	5.81	6.43	6.51
677.61	2.53	3.26	3.41	3.83	4.30	4.85	5.20	5.42	5.91	6.15	6.41
729.83	2.65	3.07	3.56	4.31	4.49	5.29	5.27	5.77	6.21	6.30	6.35
786.16	2.90	3.55	3.99	4.20	4.93	5.12	5.37	5.59	6.08	6.15	6.67
846.90	2.87	3.40	3.75	4.14	4.84	5.44	5.49	5.69	5.91	6.46	6.73
912.42	2.62	3.34	3.59	4.45	4.63	5.14	5.56	5.57	6.36	6.42	6.80
983.08	2.81	3.34	4.07	4.61	4.81	5.23	5.63	5.89	6.12	6.61	6.76
1059.29	2.69	3.58	3.87	4.32	4.86	5.24	5.65	5.47	6.48	6.39	6.91
1141.48	2.75	3.60	3.80	4.31	4.94	5.22	5.60	5.90	6.18	6.76	6.57
1230.13	3.05	3.60	4.18	4.52	5.13	5.50	5.66	6.22	6.38	6.64	6.87
1325.73	3.17	3.55	4.33	4.64	5.12	5.63	5.68	6.24	6.85	6.76	6.98
1428.84	2.83	3.69	4.28	4.42	4.88	5.46	5.66	6.35	6.77	6.84	7.09
1540.05	2.85	3.58	4.05	4.60	4.99	5.53	5.90	6.30	6.48	6.93	6.97
1659.99	2.59	3.38	4.01	4.51	5.11	5.30	5.82	5.86	6.11	6.39	6.81
1789.34	2.91	3.44	4.07	4.50	4.89	5.54	6.09	6.01	6.75	6.81	7.02
1928.85	2.95	3.68	4.11	4.57	4.99	5.12	5.59	6.11	6.42	6.54	6.88
2079.31	2.89	3.53	4.06	4.18	4.88	5.40	5.70	6.09	6.38	6.67	6.79
2241.59	3.11	3.78	4.31	4.49	5.02	5.70	5.62	6.20	6.52	6.68	7.18
2416.60	3.12	3.39	4.24	4.50	5.02	5.79	6.33	6.40	6.74	6.82	7.01
2605.36	3.06	3.28	4.01	4.28	4.78	5.41	5.64	6.19	6.47	6.50	7.07
2808.94	2.69	3.39	4.21	4.47	4.96	5.49	5.67	6.02	6.49	6.76	6.84
3028.50	3.20	3.61	4.03	4.54	4.87	5.24	5.57	6.01	6.49	6.44	6.53
3265.30	2.86	3.24	3.86	4.16	4.62	5.15	5.48	5.66	5.95	6.20	6.56

3520.69	3.45	3.53	4.05	4.51	4.83	5.54	5.71	5.81	6.12	6.57	6.72
3796.13	3.17	3.85	4.28	4.46	4.74	5.36	5.52	5.89	6.36	6.81	6.65

Wavelength (nm) / Time (ps)	365.42	366.84	368.27	369.69	371.11	372.54	373.96	375.39	376.81	378.23	379.66
-3.78	0.05	-0.14	-0.09	-0.11	-0.30	-0.06	0.06	-0.20	-0.06	-0.35	-0.18
-3.28	-0.02	-0.02	-0.11	-0.13	-0.32	-0.18	-0.26	-0.02	-0.23	-0.20	0.05
-2.78	-0.23	0.01	-0.03	0.05	0.05	-0.03	0.00	-0.19	-0.02	-0.08	-0.25
-2.28	-0.03	-0.08	-0.05	0.07	0.03	0.03	-0.12	0.16	-0.05	0.08	-0.06
-1.78	0.11	0.18	-0.07	-0.06	0.05	0.03	0.01	-0.03	0.00	0.14	0.12
-1.28	0.31	0.09	0.27	0.27	0.31	0.30	0.13	0.20	0.09	0.29	0.32
-0.78	0.09	-0.02	0.11	0.09	0.10	0.09	0.22	0.21	0.30	0.20	0.04
-0.28	-0.28	-0.02	-0.04	-0.19	0.08	-0.19	-0.05	-0.12	-0.03	-0.09	-0.05
-0.18	0.00	0.13	0.00	0.03	0.16	-0.11	0.12	-0.02	0.12	-0.07	-0.05
-0.08	0.13	-0.04	-0.06	0.13	0.01	-0.19	0.06	-0.05	-0.13	0.21	0.06
0.02	0.05	0.06	-0.12	0.07	-0.08	0.10	0.01	0.12	0.11	0.00	0.07
0.12	-0.09	0.24	0.05	0.02	0.08	0.00	-0.04	-0.07	-0.25	0.20	-0.15
0.22	0.03	-0.15	-0.12	-0.17	-0.17	-0.11	-0.06	-0.14	0.07	-0.06	-0.16
0.27	0.22	0.17	0.05	0.08	0.07	0.04	0.01	0.21	0.23	0.16	-0.05
0.32	-0.29	-0.30	-0.29	0.04	-0.18	-0.38	-0.18	-0.36	-0.27	-0.24	-0.42
0.37	0.01	0.11	-0.03	0.05	0.24	-0.03	0.16	0.14	-0.01	0.01	0.11
0.42	0.13	0.07	0.34	0.18	0.11	0.00	0.04	0.04	0.26	-0.01	-0.03
0.47	0.36	0.42	0.30	0.23	0.26	0.37	0.34	0.17	0.20	0.07	0.26
0.52	0.10	-0.12	0.21	0.05	0.10	-0.05	0.09	0.05	0.07	-0.15	-0.04
0.57	-0.01	0.12	-0.02	0.05	-0.11	-0.25	-0.11	-0.16	-0.12	-0.12	-0.04
0.62	-9.30	-3.72	-2.43	-1.67	-0.27	0.41	0.22	-0.08	-0.15	0.00	-0.20
0.67	4.91	-15.59	-29.24	-34.44	-29.95	-17.93	-6.41	-1.03	-0.08	0.13	0.38
0.72	108.36	113.73	103.51	76.76	37.68	-1.68	-30.35	-43.37	-41.32	-31.59	-19.08
0.77	-47.08	-32.88	-1.74	42.05	86.06	118.67	132.00	122.81	96.73	59.48	19.15
0.82	3.63	1.85	-3.82	-16.79	-32.23	-39.71	-32.47	-9.31	26.21	67.01	103.55
0.87	7.26	8.73	9.73	10.10	9.64	8.05	6.93	4.90	-2.41	-16.94	-32.29
0.92	7.65	7.91	7.78	7.74	8.51	9.22	10.13	10.88	11.98	12.51	11.58
0.97	6.76	7.06	7.62	8.08	8.59	8.76	9.60	9.73	9.66	9.81	10.97
1.02	6.42	6.77	7.31	7.99	8.49	8.66	9.24	9.30	9.93	10.09	10.47
1.07	6.27	6.55	6.99	7.78	8.01	8.21	8.40	8.88	9.60	10.22	10.31
1.12	5.83	6.48	7.04	7.39	7.55	7.82	8.29	8.80	9.12	9.42	10.01
1.17	5.57	6.02	6.45	6.72	7.41	7.64	8.22	8.05	8.63	8.94	8.99
1.22	5.58	6.37	6.76	7.14	7.32	8.06	8.51	8.39	9.10	9.61	9.96
1.27	5.51	5.57	6.12	6.87	7.17	7.32	7.54	8.02	8.34	8.43	8.83
1.32	5.55	5.92	6.29	6.37	7.09	7.23	7.66	7.73	8.29	8.56	8.64
1.37	5.30	5.74	6.06	6.27	6.74	7.07	7.74	7.74	8.06	8.41	8.82
1.42	5.54	5.87	6.38	6.87	7.08	7.40	7.63	7.87	8.41	8.46	8.74
1.47	5.28	6.10	6.29	6.57	6.84	7.51	7.46	7.82	8.11	8.55	8.81
1.52	5.57	6.09	6.35	6.74	6.73	7.24	7.58	7.88	8.20	8.43	8.82
1.57	5.34	5.80	6.08	6.67	7.11	7.02	7.62	7.80	8.19	8.56	8.40
1.62	5.30	5.70	6.06	6.19	6.45	6.98	7.33	7.30	7.86	7.96	8.34
1.67	5.23	5.70	6.23	6.56	6.79	6.94	7.49	7.39	7.80	8.05	8.27
1.72	5.36	5.47	5.92	6.32	6.52	6.64	7.07	7.27	7.74	7.93	8.08

1.77	5.43	5.83	6.04	6.47	6.62	7.17	7.44	7.57	7.74	8.11	8.52
1.82	4.88	5.48	5.91	6.28	6.26	6.65	7.19	7.44	7.47	7.77	7.97
1.87	5.40	5.82	6.03	6.58	6.83	7.16	7.47	7.98	7.87	8.34	8.20
1.92	5.23	5.92	6.00	6.50	6.49	6.77	7.36	7.55	7.38	7.77	8.17
1.97	5.11	5.40	5.71	6.01	6.43	6.57	6.85	7.24	7.34	7.70	7.77
2.02	4.89	5.04	5.42	5.85	5.90	6.42	6.54	6.80	7.11	7.51	7.63
2.07	5.37	5.72	5.97	6.17	6.51	6.86	7.26	7.26	7.60	7.83	8.23
2.12	5.08	5.44	5.66	6.31	6.41	6.84	7.05	7.24	7.57	7.83	8.02
2.17	4.89	5.47	5.81	5.93	6.24	6.75	6.85	6.96	7.50	7.72	7.86
2.22	5.21	5.67	5.86	6.17	6.41	6.82	7.07	7.26	7.61	7.91	7.96
2.27	4.99	5.56	5.93	6.26	6.43	6.71	7.02	7.27	7.60	7.89	8.09
2.32	4.72	5.18	5.75	5.92	6.22	6.58	6.76	6.96	7.14	7.58	7.86
2.37	5.40	5.57	5.85	6.46	6.75	6.53	7.10	7.12	7.50	7.75	7.57
2.42	5.24	5.72	5.87	6.11	6.51	6.79	7.23	7.28	7.53	7.78	8.02
2.47	5.45	5.46	6.11	6.31	6.44	6.78	7.13	7.40	7.49	8.00	7.96
2.52	5.42	5.55	6.07	6.28	6.73	6.73	7.28	7.10	7.75	7.91	7.82
2.57	5.36	5.71	6.18	6.18	6.63	6.61	7.06	7.34	7.49	7.72	7.98
2.62	5.09	5.44	5.94	6.31	6.46	6.59	6.93	7.16	7.54	7.69	7.95
2.67	5.15	5.47	5.62	6.12	6.45	6.29	6.78	7.08	7.28	7.55	7.76
2.72	5.04	5.30	5.85	5.92	6.26	6.31	6.88	6.75	7.26	7.37	7.63
2.77	5.05	5.59	5.77	5.81	6.45	6.59	6.86	7.01	7.34	7.44	7.63
2.82	5.05	5.39	5.63	6.19	6.36	6.60	6.91	6.91	7.47	7.69	7.67
2.87	4.96	5.40	5.68	5.90	6.25	6.60	6.69	6.95	7.18	7.55	7.60
2.92	5.10	5.40	5.78	6.31	6.29	6.59	6.76	7.20	7.54	7.71	7.68
2.97	4.92	5.27	5.65	5.99	6.04	6.41	6.74	7.12	7.07	7.28	7.68
3.02	5.25	5.65	6.04	6.23	6.45	6.85	7.09	7.10	7.30	7.50	8.09
3.07	5.08	5.59	5.62	6.17	6.61	6.77	7.02	6.92	7.46	7.63	7.60
3.12	4.83	5.26	5.48	5.66	6.13	6.37	6.61	6.87	7.31	7.31	7.41
3.17	5.07	5.76	5.86	6.32	6.15	6.50	7.04	7.09	7.33	7.63	7.66
3.22	5.02	5.49	5.90	6.38	6.56	6.66	7.04	7.21	7.38	7.71	7.89
3.42	4.85	5.36	5.64	6.22	6.27	6.54	7.10	7.00	7.25	7.46	7.76
3.62	5.31	5.64	5.99	6.52	6.44	6.62	7.03	7.19	7.55	7.54	8.02
3.82	4.83	5.13	5.45	5.74	6.08	6.38	6.58	6.77	7.23	7.27	7.64
4.02	5.01	5.72	5.96	6.35	6.55	6.70	7.11	7.16	7.41	7.86	7.91
4.22	4.91	5.16	5.42	5.98	6.12	6.41	6.63	6.97	7.00	7.25	7.40
4.42	5.16	5.41	5.97	6.22	6.40	6.66	6.97	6.89	7.09	7.73	7.66
4.62	4.84	5.31	5.60	5.77	6.13	6.27	6.70	6.81	6.99	7.31	7.40
4.82	4.75	5.37	5.66	6.02	6.34	6.33	6.69	6.86	7.14	7.29	7.57
5.02	4.98	5.50	5.74	6.15	6.38	6.43	6.95	6.93	7.30	7.47	7.63
5.22	4.71	5.13	5.67	5.83	6.11	6.24	6.60	6.65	6.95	7.16	7.29
5.42	5.01	5.25	5.56	5.68	6.14	6.40	6.58	6.75	7.06	7.16	7.19
5.62	4.74	5.21	5.44	5.66	5.87	6.30	6.70	6.68	7.00	7.39	7.36
5.82	5.17	5.46	5.52	5.95	6.16	6.38	6.65	6.94	6.91	7.21	7.43
6.02	4.84	5.43	5.30	5.76	6.04	6.20	6.49	6.50	6.82	7.08	7.02
6.22	4.94	5.32	5.63	5.87	6.26	6.71	6.74	6.89	7.14	7.25	7.45
6.42	4.83	5.10	5.48	5.69	6.05	6.32	6.80	6.95	7.08	7.32	7.23
6.62	5.11	5.14	5.57	5.91	5.96	6.34	6.54	6.89	6.97	7.10	7.32
6.82	4.66	5.21	5.36	5.81	5.87	6.13	6.45	6.63	6.63	7.23	7.23
7.02	5.10	5.40	5.62	5.99	6.04	6.53	6.81	7.07	7.07	7.32	7.57
7.22	4.71	5.00	5.17	5.85	5.83	6.18	6.39	6.77	6.75	7.04	7.12

7.42	4.99	5.53	5.71	6.07	6.43	6.59	6.95	7.08	7.17	7.33	7.51
7.62	4.61	5.20	5.33	5.74	6.01	6.21	6.62	6.87	6.85	7.00	7.21
7.82	4.85	5.19	5.44	5.79	6.07	6.25	6.22	6.76	6.87	7.11	7.06
8.02	4.87	5.05	5.34	5.50	6.09	6.36	6.27	6.65	6.85	7.08	7.03
8.22	4.53	4.98	5.28	5.63	5.98	6.22	6.41	6.49	6.87	6.92	7.08
8.42	4.90	5.05	5.11	5.56	5.95	5.97	6.42	6.50	6.79	6.87	7.14
8.62	4.26	5.12	5.09	5.52	5.87	5.95	6.19	6.49	6.58	6.82	6.87
8.82	4.61	5.18	5.24	5.42	5.89	6.26	6.32	6.23	6.64	7.02	7.30
9.02	4.72	5.18	5.20	5.56	5.94	6.02	6.39	6.64	6.77	6.78	7.04
9.22	4.86	5.13	5.36	5.55	5.81	6.01	6.55	6.55	6.98	7.22	7.19
9.42	4.51	4.81	5.17	5.47	5.66	5.86	6.38	6.51	6.73	6.64	6.82
9.62	4.75	4.96	5.46	5.70	5.87	6.04	6.21	6.47	6.68	7.02	7.24
9.82	4.37	4.88	5.24	5.48	5.61	5.91	6.23	6.26	6.62	6.78	6.83
10.02	4.53	4.85	5.31	5.44	5.71	5.97	6.17	6.42	6.47	6.81	7.05
10.22	4.47	4.95	5.24	5.52	5.71	5.78	6.28	6.21	6.77	6.85	7.03
10.42	4.62	4.98	5.05	5.39	5.59	5.93	6.22	6.38	6.56	6.81	6.87
10.62	4.68	4.98	5.09	5.56	5.52	5.79	6.20	6.15	6.78	6.92	6.97
10.82	4.52	4.94	5.17	5.42	5.63	5.83	6.05	6.24	6.65	6.91	6.65
11.02	4.58	4.98	5.11	5.49	5.46	5.74	6.04	6.45	6.55	6.83	7.10
11.22	4.30	4.71	5.17	5.30	5.44	5.85	6.19	6.33	6.50	6.73	6.89
11.42	4.20	4.76	4.87	5.17	5.50	5.54	6.09	6.03	6.20	6.43	6.39
11.62	4.67	5.19	5.42	5.62	5.99	6.14	6.21	6.41	6.62	7.09	7.16
11.82	4.46	4.85	5.05	5.44	5.76	6.16	6.42	6.57	6.42	6.66	7.00
12.02	4.17	4.69	5.01	5.21	5.49	5.84	6.07	6.10	6.37	6.72	6.80
12.22	4.51	4.96	5.05	5.41	5.71	5.80	6.26	6.31	6.82	6.96	7.02
12.42	4.59	4.84	5.36	5.49	5.75	6.06	6.27	6.48	7.08	7.08	6.99
12.62	4.42	4.77	4.74	5.26	5.50	5.79	6.00	6.20	6.37	6.61	6.70
12.82	4.26	4.64	4.58	5.26	5.31	5.57	5.86	5.83	6.00	6.50	6.53
13.02	4.29	4.75	4.84	5.25	5.33	5.59	5.91	6.02	6.31	6.53	6.80
13.22	4.38	4.71	4.94	5.42	5.87	5.93	6.19	6.28	6.35	6.79	6.88
13.42	4.43	4.74	5.15	5.36	5.80	5.87	6.23	6.42	6.56	6.82	6.83
13.50	4.14	4.77	5.01	5.26	5.51	5.84	6.07	6.11	6.42	6.50	6.70
13.59	4.35	4.99	5.00	5.37	5.43	5.76	5.85	6.24	6.42	6.36	6.60
13.68	4.31	4.87	5.03	5.37	5.78	5.96	6.06	6.19	6.81	6.73	6.90
13.78	4.47	4.86	4.96	5.35	5.47	5.71	6.05	6.21	6.39	6.79	6.70
13.88	4.59	4.91	5.13	5.63	5.58	6.09	6.07	6.02	6.81	6.81	7.01
14.00	4.48	4.85	5.03	5.37	5.44	6.01	6.19	6.14	6.49	6.76	6.74
14.12	4.48	4.76	5.06	5.22	5.75	6.06	6.18	6.35	6.67	6.91	6.80
14.25	4.28	4.89	5.16	5.24	5.45	5.86	5.97	6.18	6.17	6.73	6.78
14.40	4.49	4.90	5.11	5.36	5.77	5.88	6.30	6.51	6.64	6.84	6.85
14.55	4.55	5.02	5.09	5.40	5.72	5.71	6.24	6.14	6.55	6.68	6.82
14.72	4.65	5.07	5.11	5.58	5.83	6.43	6.50	6.36	6.90	7.02	7.20
14.90	4.08	4.48	4.84	4.75	5.40	5.63	5.80	5.93	6.18	6.33	6.55
15.09	4.38	4.60	5.05	5.17	5.40	5.72	6.09	6.14	6.45	6.65	6.77
15.30	4.29	4.64	4.91	5.42	5.59	5.76	6.11	6.21	6.53	6.62	6.70
15.53	4.73	5.27	5.60	5.72	5.85	6.32	6.56	6.79	6.85	7.06	7.32
15.77	4.52	5.08	5.20	5.60	5.81	6.01	6.45	6.39	6.73	6.85	6.97
16.04	4.49	4.95	5.19	5.64	5.81	5.98	6.40	6.58	6.79	6.91	6.96
16.32	4.38	4.65	5.17	5.35	5.66	5.84	6.00	6.07	6.38	6.76	6.72
16.63	4.59	4.74	4.98	5.49	5.78	5.84	6.27	6.19	6.52	6.65	6.81

16.96	4.30	4.80	5.04	5.54	5.54	5.90	6.31	6.23	6.60	6.81	6.90
17.31	4.36	4.80	5.25	5.29	5.43	5.79	6.14	6.25	6.41	6.77	6.89
17.70	4.50	5.01	5.31	5.59	5.93	6.15	6.13	6.33	6.67	6.85	6.78
18.11	4.18	4.58	5.21	5.41	5.56	5.95	6.24	6.35	6.35	6.81	6.73
18.56	4.46	4.63	4.72	5.30	5.49	5.72	6.09	6.15	6.45	6.28	6.62
19.04	4.51	4.52	5.09	5.38	5.50	5.68	6.09	6.15	6.61	6.67	6.69
19.56	4.35	5.06	4.95	5.33	5.48	5.81	5.96	6.10	6.30	6.64	6.65
20.12	4.45	4.89	5.28	5.43	5.72	5.87	6.17	6.26	6.60	6.88	7.00
20.72	4.62	5.02	5.07	5.45	5.65	6.06	6.17	6.33	6.52	6.77	6.72
21.37	4.47	4.98	5.13	5.53	5.63	5.80	6.18	6.16	6.50	6.83	6.66
22.08	4.91	5.20	5.61	5.65	6.32	6.30	6.63	6.70	6.98	7.18	7.29
22.84	4.27	4.92	5.29	5.42	5.58	5.86	6.24	6.21	6.59	6.61	6.95
23.65	4.30	4.84	5.18	5.24	5.74	6.01	6.33	6.11	6.43	6.80	6.79
24.54	4.43	4.69	5.06	5.41	5.62	5.80	6.11	6.30	6.40	6.70	6.98
25.49	4.61	5.16	5.24	5.49	5.82	6.03	6.27	6.23	6.58	6.74	6.83
26.51	4.21	4.87	5.35	5.48	5.56	5.84	6.30	6.32	6.51	6.67	6.69
27.62	4.44	4.95	5.17	5.57	5.82	6.04	6.28	6.49	6.66	6.72	6.80
28.81	4.41	5.13	5.32	5.68	5.92	6.01	6.38	6.43	6.66	6.84	6.98
30.10	4.43	4.84	5.15	5.30	5.57	5.82	6.02	6.30	6.38	6.66	6.82
31.49	4.40	4.58	5.05	5.34	5.50	5.58	6.07	5.92	6.20	6.61	6.68
32.98	4.22	4.75	5.08	5.27	5.44	5.62	5.83	5.89	6.32	6.45	6.52
34.60	4.68	5.00	5.09	5.30	5.87	5.90	6.17	6.43	6.47	6.60	6.75
36.34	4.74	5.15	5.23	5.59	5.77	6.13	6.13	6.20	6.57	6.74	6.78
38.21	4.57	4.74	4.99	5.15	5.67	5.41	5.94	6.08	6.45	6.51	6.61
40.24	4.53	5.06	5.27	5.42	5.75	5.90	5.98	6.41	6.62	6.60	6.81
42.42	4.36	4.80	4.89	5.28	5.33	5.66	5.83	6.14	6.30	6.46	6.57
44.78	4.62	4.89	5.28	5.52	5.86	5.88	6.29	6.51	6.44	6.63	6.79
47.32	4.79	5.03	5.23	5.54	5.90	6.07	6.37	6.24	6.40	6.71	6.61
50.06	4.93	5.30	5.54	5.89	6.19	6.08	6.45	6.61	6.63	6.83	6.92
53.01	5.07	5.21	5.61	5.72	6.03	6.29	6.52	6.51	6.83	6.92	7.13
56.20	4.63	5.20	5.32	5.83	5.77	6.10	6.26	6.45	6.69	6.89	6.93
59.64	4.64	4.90	5.43	5.63	5.67	5.84	6.44	6.29	6.57	6.74	6.60
63.34	4.69	5.10	5.64	5.64	5.96	5.94	6.30	6.38	6.60	6.64	6.73
67.34	4.51	5.10	5.26	5.49	5.83	6.00	6.38	6.21	6.49	6.51	6.74
71.65	4.84	5.09	5.54	5.90	5.96	6.29	6.42	6.43	6.72	6.83	6.65
76.30	5.02	5.39	5.36	5.76	5.89	6.24	6.45	6.40	6.68	6.83	6.64
81.32	4.96	5.42	5.79	5.79	6.06	6.14	6.61	6.31	6.55	6.81	6.86
86.73	4.89	5.36	5.57	5.75	5.92	6.21	6.56	6.48	6.82	7.07	7.08
92.56	4.90	5.16	5.44	5.97	6.03	6.26	6.33	6.53	6.46	6.90	6.79
98.85	4.93	5.40	5.79	6.00	6.02	6.17	6.61	6.44	6.73	6.85	6.81
105.64	4.99	5.65	5.61	5.95	5.93	6.19	6.36	6.65	6.78	6.68	6.95
112.96	5.19	5.38	5.46	6.04	6.23	6.27	6.48	6.73	6.80	6.79	7.04
120.85	4.93	5.28	5.79	5.84	6.03	6.02	6.18	6.42	6.35	6.68	6.69
129.36	5.46	5.77	6.12	6.44	6.85	6.76	6.97	6.97	7.18	7.27	7.26
138.55	5.18	5.74	5.85	6.03	6.22	6.31	6.45	6.69	6.68	6.76	6.60
148.45	5.51	5.85	6.15	6.45	6.46	6.60	6.66	6.91	7.13	7.31	7.12
159.13	5.61	6.05	6.31	6.23	6.48	6.60	6.80	6.90	6.96	7.04	6.95
170.65	5.40	5.94	6.07	6.23	6.64	6.68	6.73	6.96	7.09	7.19	7.27
183.07	5.57	5.94	6.26	6.63	6.33	6.63	6.95	6.88	7.06	6.94	7.02
196.47	5.87	5.98	5.92	6.52	6.67	6.90	7.04	6.97	7.03	7.32	7.22

210.91	5.72	5.99	6.26	6.54	6.72	6.74	6.90	7.00	6.89	7.06	6.94
226.50	5.54	5.73	6.02	6.28	6.27	6.44	6.66	6.70	6.79	6.73	6.77
243.31	5.88	6.20	6.37	6.65	6.63	6.85	6.94	6.98	6.92	7.08	7.12
261.43	6.20	6.29	6.40	6.72	7.07	6.99	7.00	7.24	7.38	7.30	7.29
280.98	6.11	6.44	6.46	6.66	6.66	7.07	7.30	6.96	7.24	7.10	7.23
302.07	5.99	6.41	6.57	6.84	6.82	6.97	7.14	7.13	7.13	7.25	7.22
324.81	6.22	6.32	6.61	6.90	7.17	7.14	7.29	7.41	7.21	7.50	7.49
349.33	6.26	6.56	6.74	6.93	6.99	7.09	7.35	7.32	7.26	7.12	7.14
375.79	6.33	6.81	6.68	6.91	7.05	7.09	7.43	7.41	7.46	7.35	7.45
404.31	6.33	6.59	6.93	6.85	7.23	7.19	7.35	7.28	7.23	7.31	7.09
435.08	6.69	7.07	7.11	7.52	7.48	7.56	7.91	7.71	7.95	7.81	7.69
468.27	6.78	7.24	7.37	7.42	7.78	7.79	7.70	7.89	7.80	7.93	7.61
504.06	6.50	7.05	7.15	7.22	7.27	7.51	7.58	7.40	7.58	7.37	7.46
542.66	6.98	7.42	7.35	7.78	7.57	7.69	7.88	7.93	7.90	7.83	7.68
584.29	6.74	7.12	7.46	7.51	7.55	7.71	7.63	7.63	7.46	7.69	7.63
629.18	6.76	7.17	7.34	7.47	7.64	7.61	7.37	7.88	7.54	7.52	7.45
677.61	6.65	7.03	7.26	7.30	7.45	7.53	7.78	7.66	7.77	7.81	7.65
729.83	6.58	6.81	7.25	7.22	7.26	7.41	7.56	7.42	7.56	7.34	7.11
786.16	6.82	7.20	7.06	7.34	7.49	7.66	7.66	7.39	7.57	7.54	7.24
846.90	6.98	7.32	7.34	7.74	7.73	7.58	7.74	7.67	7.78	7.72	7.34
912.42	6.88	6.93	7.07	7.13	7.44	7.49	7.67	7.44	7.62	7.40	7.25
983.08	7.09	7.39	7.42	7.67	7.64	7.62	7.67	7.70	7.69	7.41	7.39
1059.29	6.98	7.40	7.40	7.59	7.39	7.59	7.70	7.51	7.58	7.29	7.20
1141.48	6.93	7.34	7.45	7.69	7.81	7.87	7.74	7.72	7.72	7.63	7.35
1230.13	7.06	7.38	7.58	7.58	7.66	7.70	7.74	7.86	7.69	7.66	7.42
1325.73	7.26	7.57	7.84	7.91	7.88	8.05	7.91	7.78	7.83	7.67	7.71
1428.84	7.14	7.56	7.61	7.85	8.18	8.06	8.06	7.90	7.82	7.95	7.59
1540.05	7.33	7.35	7.80	7.87	7.93	8.12	8.11	7.91	7.92	7.87	7.58
1659.99	7.10	7.33	7.52	7.59	7.61	7.56	7.90	7.85	7.62	7.51	7.44
1789.34	7.17	7.85	7.84	7.99	7.93	7.83	8.13	7.99	7.84	8.07	7.78
1928.85	6.93	7.30	7.39	7.79	7.77	7.68	7.62	7.66	7.69	7.52	7.56
2079.31	7.17	7.67	7.43	7.57	7.74	7.58	7.89	7.69	7.74	7.55	7.38
2241.59	7.08	7.52	7.73	7.66	7.87	7.92	8.00	7.89	7.77	7.81	7.51
2416.60	7.35	7.38	7.63	7.86	7.86	7.87	8.05	7.88	7.74	7.63	7.64
2605.36	6.92	7.29	7.39	7.49	7.52	7.77	7.74	7.61	7.64	7.42	7.17
2808.94	7.03	7.30	7.51	7.89	7.77	7.73	7.87	7.87	7.72	7.92	7.45
3028.50	6.68	7.33	7.43	7.34	7.45	7.32	7.73	7.38	7.42	7.57	7.26
3265.30	6.80	7.09	7.27	7.37	7.38	7.65	7.63	7.55	7.51	7.48	7.33
3520.69	6.93	7.29	7.45	7.44	7.54	7.69	7.82	7.55	7.38	7.39	7.31
3796.13	6.82	7.10	7.12	7.46	7.39	7.45	7.46	7.46	7.45	7.42	7.09

Wavelength (nm) / Time (ps)	381.08	382.51	383.93	385.36	386.78	388.20	389.63	391.05	392.48	393.90	395.32
-3.78	-0.14	-0.22	-0.03	-0.22	-0.14	-0.11	-0.09	0.00	0.05	0.00	-0.19
-3.28	-0.04	-0.12	-0.03	-0.03	0.00	-0.04	0.04	-0.07	-0.11	-0.07	0.05
-2.78	-0.10	-0.34	-0.17	-0.18	-0.08	-0.19	-0.32	-0.25	-0.37	-0.29	-0.18
-2.28	-0.01	0.05	-0.19	-0.02	-0.11	0.08	0.07	0.03	-0.05	-0.15	-0.01
-1.78	-0.03	0.05	0.00	0.10	0.07	0.14	0.11	0.17	0.10	0.23	0.11

-1.28	0.28	0.43	0.34	0.16	0.18	0.13	0.11	0.14	0.38	0.38	0.36
-0.78	0.15	0.23	0.05	0.09	0.11	0.05	0.15	-0.01	-0.02	-0.10	-0.08
-0.28	-0.12	-0.08	0.03	0.10	-0.02	-0.07	-0.07	0.00	0.02	0.00	-0.05
-0.18	0.07	0.01	-0.24	0.01	0.01	-0.14	0.08	-0.05	0.17	0.22	-0.01
-0.08	-0.12	0.11	0.02	0.00	0.01	0.13	0.04	0.08	0.05	0.19	-0.02
0.02	-0.07	-0.02	-0.03	-0.28	0.08	-0.18	-0.24	-0.08	-0.22	-0.17	-0.26
0.12	-0.23	-0.09	-0.23	0.04	0.09	0.01	0.16	0.15	0.03	0.06	-0.11
0.22	-0.27	-0.01	0.00	-0.09	-0.18	-0.01	0.07	-0.01	-0.03	0.01	-0.16
0.27	-0.12	0.26	0.19	-0.10	0.05	0.04	0.02	0.02	0.08	0.13	-0.04
0.32	-0.24	-0.25	-0.25	-0.42	-0.29	-0.30	-0.25	-0.07	-0.22	-0.21	-0.19
0.37	0.24	0.29	0.12	0.05	0.09	0.10	-0.04	0.25	0.29	0.34	0.10
0.42	0.00	0.15	0.35	0.42	0.12	0.22	0.25	0.19	0.22	0.18	0.10
0.47	0.18	0.39	0.10	0.05	0.16	0.22	0.15	0.11	0.18	0.35	0.24
0.52	-0.18	0.08	0.02	0.09	0.05	-0.05	0.08	0.05	-0.06	-0.06	-0.04
0.57	-0.32	0.00	-0.05	-0.22	-0.19	-0.22	-0.06	0.17	0.03	0.07	-0.15
0.62	0.08	-0.03	0.03	-0.01	-0.04	-0.03	-0.06	-0.09	0.08	0.05	0.04
0.67	0.43	-0.09	-0.03	0.14	0.21	0.08	0.09	0.38	0.18	0.24	0.14
0.72	-9.15	-2.84	-0.40	0.27	0.30	0.34	0.30	0.34	0.28	0.37	0.37
0.77	-16.25	-39.67	-48.16	-43.11	-29.98	-15.53	-5.26	-0.71	0.92	1.44	0.54
0.82	127.08	132.51	118.81	89.73	53.45	18.61	-5.58	-16.42	-16.68	-14.87	-17.66
0.87	-39.99	-33.38	-11.70	20.72	56.39	87.15	107.17	112.52	106.83	93.70	73.98
0.92	10.40	10.94	10.41	2.46	-14.88	-36.40	-53.06	-56.62	-44.01	-15.00	29.05
0.97	11.19	11.47	12.10	12.83	13.67	13.78	13.39	13.80	14.24	11.90	2.64
1.02	11.17	11.44	11.37	11.42	11.43	12.15	12.40	12.60	13.08	13.58	14.34
1.07	10.39	10.73	11.34	11.46	11.74	11.71	12.18	12.54	12.74	12.89	13.31
1.12	10.11	10.65	10.64	10.89	11.09	11.61	11.95	11.91	12.47	12.74	12.88
1.17	9.46	9.89	10.33	10.53	10.65	10.97	11.14	11.06	11.48	11.90	11.98
1.22	9.95	9.82	10.29	10.52	10.75	10.71	11.09	11.45	11.59	11.86	12.02
1.27	9.39	9.54	9.81	9.87	10.41	10.82	10.92	10.86	11.05	11.43	11.42
1.32	8.95	9.31	9.72	9.81	9.86	10.03	10.35	10.53	10.66	11.02	11.26
1.37	8.84	9.37	9.21	9.39	9.84	9.93	10.05	10.24	10.48	10.90	10.83
1.42	9.17	9.65	9.80	9.72	9.79	10.15	10.19	10.46	10.72	10.99	11.15
1.47	8.91	9.02	9.46	9.58	9.95	9.75	10.31	10.33	10.54	10.65	10.79
1.52	9.15	8.99	9.17	9.50	9.82	9.58	9.94	9.99	10.35	10.52	10.59
1.57	8.98	9.13	9.32	9.37	9.56	9.62	10.03	10.23	10.25	10.45	10.73
1.62	8.37	8.75	9.03	9.17	9.36	9.50	9.57	9.83	10.05	10.17	10.28
1.67	8.57	8.69	8.72	8.92	9.25	9.50	9.72	9.72	9.94	10.23	10.19
1.72	8.20	8.65	9.02	8.92	9.23	9.26	9.50	9.61	9.82	10.00	10.14
1.77	8.59	8.69	9.05	9.09	9.18	9.40	9.48	9.77	10.01	10.12	10.16
1.82	8.32	8.56	8.43	8.57	8.90	9.01	9.21	9.38	9.34	9.61	9.67
1.87	8.50	8.57	9.13	9.34	9.22	9.24	9.35	9.56	9.70	9.87	10.13
1.92	8.26	8.43	8.61	8.92	9.11	9.19	9.43	9.57	9.84	10.04	10.06
1.97	7.94	8.31	8.53	8.57	8.85	8.88	9.08	9.25	9.36	9.67	9.70
2.02	7.67	7.85	7.94	8.20	8.66	8.55	8.93	8.97	9.20	9.19	9.25
2.07	8.34	8.50	8.40	8.80	9.08	9.14	9.21	9.35	9.53	9.85	10.01
2.12	8.18	8.51	8.52	8.88	8.95	9.02	9.18	9.39	9.51	9.70	9.88
2.17	8.14	8.23	8.17	8.70	8.70	8.78	8.94	9.24	9.44	9.64	9.51
2.22	8.31	8.49	8.52	8.66	8.70	8.98	9.30	9.31	9.58	9.78	9.74
2.27	8.25	8.56	8.68	8.75	8.91	8.84	9.21	9.17	9.32	9.54	9.68
2.32	8.00	8.16	8.26	8.31	8.67	8.67	8.68	8.82	9.15	9.01	9.09

2.37	7.91	8.37	8.40	8.55	8.81	8.89	9.21	9.06	9.43	9.75	9.72
2.42	8.11	8.15	8.61	8.43	8.72	8.86	8.93	8.99	9.27	9.16	9.30
2.47	8.48	8.31	8.28	8.42	8.89	8.90	9.23	9.21	9.36	9.68	9.73
2.52	8.13	8.35	8.59	8.55	8.71	9.02	9.16	9.43	9.44	9.56	9.80
2.57	8.11	8.16	8.57	8.65	8.84	8.97	9.02	9.26	9.23	9.69	9.61
2.62	8.35	8.43	8.45	8.63	8.83	8.74	8.81	9.13	9.34	9.44	9.47
2.67	7.97	8.06	8.13	8.35	8.63	8.61	8.65	8.94	9.09	9.22	9.30
2.72	7.86	7.98	8.24	8.30	8.53	8.63	8.72	8.82	9.12	9.34	9.40
2.77	7.81	7.94	8.27	8.25	8.29	8.46	8.89	8.97	9.09	9.29	9.21
2.82	7.88	8.13	8.21	8.41	8.72	8.52	8.76	8.86	9.08	9.30	9.13
2.87	7.92	8.11	8.14	8.26	8.51	8.64	8.77	8.81	9.03	9.17	9.31
2.92	7.93	8.21	8.34	8.44	8.59	8.83	8.77	9.00	9.05	9.32	9.33
2.97	7.68	8.04	8.13	8.34	8.59	8.35	8.65	8.69	8.78	8.90	9.02
3.02	7.97	8.22	8.30	8.56	8.66	8.82	8.94	9.06	9.26	9.47	9.31
3.07	7.81	8.20	8.13	8.30	8.49	8.54	8.71	8.78	8.99	9.15	9.21
3.12	7.68	8.01	8.03	8.08	8.28	8.44	8.54	8.48	8.94	8.98	9.07
3.17	7.75	7.88	8.21	8.21	8.30	8.32	8.65	8.63	8.96	8.93	9.05
3.22	7.94	8.02	8.29	8.51	8.60	8.57	8.71	9.13	9.26	9.42	9.41
3.42	8.01	8.10	8.40	8.57	8.50	8.44	8.72	8.73	8.88	9.16	9.00
3.62	8.30	8.31	8.64	8.40	8.69	8.93	8.94	9.14	9.16	9.35	9.34
3.82	7.82	8.02	8.08	8.43	8.64	8.65	8.78	8.94	9.06	9.34	9.19
4.02	8.01	8.36	8.19	8.56	8.60	8.66	8.98	9.16	9.22	9.30	9.32
4.22	7.49	7.87	7.94	8.13	8.30	8.39	8.56	8.65	8.61	8.93	9.00
4.42	7.89	8.06	8.10	8.19	8.41	8.61	8.77	8.96	8.88	9.18	9.17
4.62	7.53	7.90	7.93	8.05	8.11	8.34	8.32	8.63	8.63	8.72	8.86
4.82	7.71	7.85	8.01	8.07	8.34	8.35	8.59	8.56	8.90	8.93	8.98
5.02	7.79	8.11	8.18	8.13	8.29	8.50	8.48	8.76	8.86	8.91	9.01
5.22	7.55	7.69	7.86	8.14	8.06	8.20	8.54	8.42	8.70	8.89	8.93
5.42	7.46	7.72	7.86	8.13	8.30	8.36	8.57	8.64	8.82	8.90	8.98
5.62	7.70	7.97	7.97	8.04	8.23	8.03	8.47	8.46	8.68	8.83	8.80
5.82	7.63	7.97	7.79	7.84	8.12	8.21	8.43	8.57	8.61	9.01	8.78
6.02	7.17	7.57	7.54	7.69	7.83	7.72	8.19	8.34	8.49	8.56	8.60
6.22	7.82	7.71	7.67	7.89	8.07	8.26	8.38	8.50	8.61	8.79	8.87
6.42	7.54	7.88	8.03	8.01	8.30	8.44	8.48	8.60	8.70	8.98	8.86
6.62	7.40	7.85	7.82	7.88	8.21	8.18	8.23	8.54	8.47	8.73	8.73
6.82	7.40	7.58	7.70	7.97	7.85	7.97	8.13	8.29	8.43	8.58	8.72
7.02	7.75	7.71	7.89	7.90	8.07	8.06	8.49	8.85	8.71	8.86	8.84
7.22	7.48	7.69	7.67	7.81	7.89	8.06	8.23	8.36	8.44	8.66	8.78
7.42	7.87	8.17	8.08	8.15	8.27	8.48	8.70	8.66	8.85	8.99	9.04
7.62	7.42	7.51	7.80	7.77	7.95	8.06	8.31	8.50	8.42	8.47	8.40
7.82	7.36	7.62	7.69	7.84	8.02	7.96	8.21	8.22	8.38	8.59	8.58
8.02	7.26	7.46	7.54	7.52	7.65	7.76	7.99	8.12	8.15	8.46	8.57
8.22	7.39	7.53	7.57	7.92	8.10	7.87	8.19	8.11	8.30	8.34	8.60
8.42	7.40	7.43	7.51	7.99	8.11	8.08	8.54	8.33	8.54	8.83	8.79
8.62	7.17	7.46	7.42	7.62	7.78	7.77	7.86	8.05	8.21	8.38	8.37
8.82	7.18	7.73	7.62	7.68	8.04	7.95	8.06	8.16	8.22	8.57	8.41
9.02	7.26	7.45	7.48	7.78	7.82	7.93	8.14	8.08	8.34	8.62	8.57
9.22	7.17	7.53	7.75	7.62	7.97	7.97	8.11	8.37	8.37	8.58	8.55
9.42	7.01	7.29	7.39	7.51	7.74	7.73	7.92	8.04	8.06	8.33	8.41
9.62	7.42	7.63	7.70	7.73	7.98	8.20	8.11	8.31	8.24	8.46	8.63

9.82	7.19	7.40	7.50	7.48	7.66	7.71	8.02	7.86	8.21	8.57	8.44
10.02	7.02	7.30	7.49	7.55	7.66	7.65	7.78	7.88	7.99	8.30	8.24
10.22	7.17	7.31	7.49	7.48	7.56	7.66	7.81	7.78	8.05	8.27	8.19
10.42	7.01	7.29	7.32	7.81	7.82	7.98	8.12	8.09	8.22	8.49	8.36
10.62	7.00	7.27	7.41	7.64	7.70	7.91	7.92	7.98	8.38	8.52	8.59
10.82	7.06	7.16	7.30	7.45	7.66	7.68	7.82	8.07	8.28	8.45	8.51
11.02	7.20	7.42	7.35	7.53	7.78	7.85	7.99	8.07	8.11	8.28	8.41
11.22	7.08	7.25	7.31	7.40	7.52	7.53	7.79	7.88	8.09	8.10	8.13
11.42	6.74	6.99	7.17	7.09	7.24	7.18	7.44	7.68	7.78	7.90	7.83
11.62	7.23	7.64	7.58	7.62	7.77	7.93	8.21	8.23	8.40	8.45	8.50
11.82	6.99	7.33	7.47	7.67	7.65	7.72	7.93	7.95	8.07	8.34	8.31
12.02	6.80	7.07	7.49	7.32	7.42	7.58	7.72	7.90	8.06	8.25	8.27
12.22	7.06	7.20	7.47	7.69	7.45	7.81	8.14	8.16	8.36	8.48	8.48
12.42	7.18	7.45	7.43	7.63	7.64	7.77	7.94	8.16	8.17	8.26	8.45
12.62	6.93	7.07	7.15	7.31	7.50	7.46	7.58	7.80	7.93	8.03	7.99
12.82	6.71	6.87	7.00	7.14	7.31	7.22	7.35	7.59	7.67	7.89	7.98
13.02	7.11	6.97	7.23	7.58	7.68	7.68	8.00	8.07	8.25	8.27	8.33
13.22	6.81	7.29	7.31	7.48	7.74	7.61	8.05	8.01	8.01	8.24	8.35
13.42	7.26	7.45	7.48	7.58	7.66	7.77	7.99	7.82	8.13	8.48	8.37
13.50	6.93	7.11	7.20	7.18	7.31	7.53	7.63	7.82	8.01	8.07	8.07
13.59	6.95	7.30	7.12	7.46	7.44	7.57	7.84	7.87	7.98	8.12	8.14
13.68	7.14	7.22	7.17	7.30	7.33	7.57	7.66	7.74	7.78	8.06	7.98
13.78	7.00	7.13	7.10	7.46	7.53	7.70	7.74	7.82	7.94	7.99	8.12
13.88	6.97	7.29	7.57	7.58	7.73	7.52	7.69	7.95	8.03	8.31	8.20
14.00	6.99	7.33	7.35	7.51	7.55	7.58	7.80	7.99	8.01	8.36	8.12
14.12	6.97	7.42	7.40	7.34	7.40	7.65	7.93	8.01	8.20	8.39	8.35
14.25	7.07	7.35	7.42	7.55	7.50	7.62	7.81	7.80	8.14	8.17	8.18
14.40	7.13	7.22	7.42	7.64	7.65	7.90	8.12	8.17	8.32	8.50	8.54
14.55	7.05	7.15	7.40	7.38	7.50	7.59	7.66	7.75	8.01	8.15	8.22
14.72	7.35	7.47	7.71	7.58	7.87	7.97	8.11	8.04	8.33	8.45	8.55
14.90	6.65	6.76	7.09	6.91	7.25	7.17	7.43	7.49	7.68	7.92	7.88
15.09	6.89	6.99	7.21	7.28	7.45	7.44	7.50	7.76	7.97	8.02	8.06
15.30	6.77	7.12	7.24	7.28	7.34	7.51	7.83	7.71	7.83	7.99	8.12
15.53	7.17	7.49	7.58	7.70	7.79	7.75	7.76	8.00	8.28	8.53	8.50
15.77	7.12	7.23	7.25	7.41	7.60	7.62	7.78	8.12	8.03	8.53	8.23
16.04	7.05	7.23	7.46	7.53	7.59	7.60	7.83	8.04	8.29	8.30	8.43
16.32	6.80	7.05	7.09	7.28	7.29	7.27	7.59	7.77	7.84	7.98	7.99
16.63	6.98	7.08	7.34	7.36	7.52	7.65	7.65	7.83	8.06	8.15	8.27
16.96	7.21	7.42	7.33	7.39	7.56	7.55	7.76	8.00	8.22	8.19	8.25
17.31	6.67	7.24	7.24	7.36	7.45	7.62	7.61	7.80	8.02	8.16	8.20
17.70	6.93	7.27	7.28	7.60	7.77	7.46	7.84	7.97	8.08	8.21	8.28
18.11	6.86	7.22	7.20	7.13	7.30	7.61	7.61	7.60	7.95	7.88	8.09
18.56	6.88	6.94	7.19	7.00	7.30	7.57	7.46	7.69	7.93	8.06	8.11
19.04	6.89	6.99	7.31	7.31	7.40	7.48	7.51	7.67	7.92	7.75	7.83
19.56	6.89	6.86	7.02	7.24	7.37	7.43	7.43	7.87	7.89	8.02	7.98
20.12	7.03	7.27	7.31	7.38	7.40	7.49	7.80	7.79	8.00	8.25	8.11
20.72	6.86	7.30	7.34	7.41	7.64	7.78	7.85	8.00	8.08	8.26	8.43
21.37	6.97	7.07	7.45	7.44	7.55	7.57	7.61	7.82	7.93	8.16	7.99
22.08	7.51	7.60	7.66	7.80	7.90	8.10	8.30	8.43	8.41	8.69	8.78
22.84	7.16	7.24	7.20	7.29	7.33	7.57	7.82	7.82	7.98	8.06	8.08

23.65	6.95	7.10	7.08	7.58	7.38	7.58	7.74	7.88	7.89	8.17	8.10
24.54	7.06	6.95	7.20	7.37	7.22	7.28	7.56	7.81	7.87	8.03	8.10
25.49	7.10	7.16	7.48	7.44	7.37	7.63	7.87	7.96	8.06	8.21	8.23
26.51	6.86	7.02	6.86	7.18	7.35	7.24	7.51	7.53	7.83	7.84	7.91
27.62	7.16	7.18	7.11	7.32	7.40	7.57	7.70	7.70	7.93	8.09	8.03
28.81	7.30	7.32	7.52	7.20	7.40	7.61	7.78	7.90	7.94	8.29	8.16
30.10	6.84	6.79	7.09	7.14	7.19	7.32	7.46	7.62	7.63	7.88	7.72
31.49	6.73	6.89	7.03	7.10	7.21	7.50	7.54	7.70	7.81	7.70	7.80
32.98	6.67	6.89	6.76	6.96	7.03	7.39	7.14	7.55	7.53	7.73	7.74
34.60	7.09	7.08	7.09	7.18	7.29	7.42	7.40	7.51	7.79	7.93	7.92
36.34	6.94	7.21	7.40	7.31	7.53	7.40	7.46	7.58	7.77	7.97	8.06
38.21	6.58	6.88	6.96	7.14	7.09	7.24	7.51	7.19	7.65	7.59	7.50
40.24	7.00	7.17	7.10	7.39	7.57	7.55	7.81	7.83	7.90	8.16	8.27
42.42	6.57	6.80	6.87	7.02	6.99	7.17	7.29	7.45	7.48	7.62	7.69
44.78	6.77	6.83	6.99	7.04	7.24	7.27	7.34	7.54	7.57	7.75	7.78
47.32	6.85	7.06	7.12	7.25	7.31	7.29	7.51	7.63	7.78	8.00	7.88
50.06	7.07	7.20	7.11	7.33	7.13	7.54	7.76	7.85	8.03	8.07	8.02
53.01	7.14	7.25	7.29	7.31	7.51	7.21	7.65	7.59	7.95	8.07	7.97
56.20	7.10	7.08	7.19	7.27	7.35	7.28	7.23	7.60	7.66	7.81	7.76
59.64	7.07	7.07	7.12	7.09	7.21	7.30	7.18	7.58	7.54	7.52	7.63
63.34	6.91	6.76	7.14	7.02	7.09	7.19	7.31	7.47	7.59	7.76	7.71
67.34	6.78	6.79	6.81	6.95	7.31	7.08	7.22	7.21	7.40	7.52	7.43
71.65	6.98	7.19	7.18	7.36	7.37	7.10	7.33	7.42	7.54	7.72	7.57
76.30	6.97	6.94	7.06	7.17	7.45	7.18	7.44	7.52	7.65	7.82	7.79
81.32	6.92	7.13	7.08	7.06	7.24	7.40	7.33	7.43	7.56	7.66	7.71
86.73	7.12	7.15	7.31	7.25	7.42	7.40	7.56	7.88	7.81	7.96	7.86
92.56	6.75	7.12	7.23	7.15	7.16	7.29	7.32	7.35	7.42	7.45	7.57
98.85	6.74	6.83	6.87	6.82	7.29	6.95	7.18	7.14	7.41	7.43	7.46
105.64	6.99	7.11	7.17	7.06	7.30	7.05	7.35	7.40	7.66	7.73	7.56
112.96	6.95	6.95	7.19	7.37	7.45	7.48	7.46	7.60	7.66	7.83	7.85
120.85	6.83	6.88	6.90	6.89	7.05	7.05	7.05	7.13	7.13	7.09	7.12
129.36	7.40	7.52	7.33	7.52	7.35	7.70	7.70	7.86	7.68	7.69	7.65
138.55	6.88	7.07	7.32	6.96	7.27	7.28	7.25	7.40	7.52	7.68	7.52
148.45	7.03	7.23	7.26	7.29	7.16	7.26	7.58	7.55	7.72	7.84	7.72
159.13	7.31	7.04	7.27	6.95	7.14	7.08	7.18	7.37	7.37	7.51	7.57
170.65	7.23	7.26	7.19	7.34	7.12	7.19	7.27	7.23	7.30	7.45	7.31
183.07	7.07	7.20	7.12	7.34	7.32	7.31	7.23	7.36	7.36	7.34	7.38
196.47	7.02	7.23	7.17	7.13	7.17	7.04	7.21	7.07	7.49	7.52	7.37
210.91	7.23	7.19	7.28	7.20	7.28	7.02	7.23	7.19	7.15	7.33	7.22
226.50	6.88	6.92	6.98	6.98	6.88	7.15	7.01	7.01	7.26	7.16	7.14
243.31	6.89	7.16	7.07	7.08	6.92	7.23	7.37	7.28	7.19	7.47	7.36
261.43	7.36	7.39	7.38	7.19	7.17	7.35	7.53	7.33	7.30	7.36	7.38
280.98	7.26	7.16	7.18	7.16	7.14	7.38	7.33	7.21	7.32	7.35	7.15
302.07	7.05	7.34	7.16	7.01	7.05	7.03	7.02	7.04	6.95	6.93	7.02
324.81	7.22	7.28	7.22	7.17	7.14	7.14	7.02	7.15	7.22	7.24	6.95
349.33	7.14	7.25	6.90	6.96	7.01	6.97	6.83	7.03	7.06	6.95	6.94
375.79	7.09	7.50	7.23	7.12	7.14	6.97	7.16	7.10	7.12	7.20	6.98
404.31	6.87	7.14	7.11	7.02	7.09	6.81	6.87	7.05	7.07	6.96	6.94
435.08	7.45	7.55	7.55	7.49	7.44	7.38	7.43	7.48	7.47	7.42	7.31
468.27	7.58	7.68	7.49	7.33	7.36	7.33	7.37	7.32	7.40	7.36	7.17

504.06	7.39	7.28	7.26	7.17	7.13	6.99	7.25	6.94	7.03	7.08	7.21
542.66	7.54	7.37	7.38	7.34	7.11	7.20	7.17	7.10	7.13	7.24	6.97
584.29	7.36	7.36	7.19	7.12	7.16	6.96	7.06	7.03	6.97	7.04	7.17
629.18	7.37	7.32	7.15	7.09	7.05	6.96	7.01	7.01	7.05	7.08	6.89
677.61	7.27	7.57	7.51	7.33	7.25	7.23	7.14	7.19	6.99	7.23	7.13
729.83	7.00	7.06	6.94	6.78	6.79	6.68	6.74	6.55	6.56	6.65	6.54
786.16	7.37	7.32	7.08	7.02	6.79	6.87	6.79	6.89	6.75	6.70	6.65
846.90	7.37	7.35	7.27	7.14	6.91	6.77	6.75	6.77	6.77	6.70	6.65
912.42	7.20	7.43	7.08	6.86	6.72	6.65	6.82	6.70	6.60	6.57	6.60
983.08	7.20	7.45	6.99	7.12	7.08	6.95	6.89	7.00	6.73	6.75	6.55
1059.29	7.31	7.27	7.04	6.82	6.84	6.84	6.85	6.68	6.56	6.66	6.51
1141.48	7.27	7.38	6.99	6.94	6.89	6.79	6.85	6.67	6.73	6.77	6.46
1230.13	7.48	7.52	7.46	7.18	7.04	7.06	7.00	6.87	6.92	6.87	6.81
1325.73	7.63	7.45	7.27	7.22	7.13	7.15	7.12	7.13	7.06	7.19	6.92
1428.84	7.43	7.43	7.22	7.25	7.13	7.03	6.95	6.85	6.97	6.86	6.82
1540.05	7.53	7.60	7.47	7.23	7.11	7.00	7.21	7.04	7.04	6.92	6.89
1659.99	7.25	7.36	7.09	6.95	6.97	6.77	6.72	6.80	6.85	6.76	6.71
1789.34	7.62	7.54	7.21	7.13	7.03	7.03	7.04	6.84	6.84	6.85	6.62
1928.85	7.22	7.25	7.06	7.26	7.05	6.63	6.72	6.83	6.55	6.69	6.60
2079.31	7.24	7.43	7.11	7.11	6.86	6.69	6.73	6.70	6.77	6.74	6.64
2241.59	7.46	7.21	7.08	7.21	6.79	6.81	6.98	6.81	6.77	6.73	6.67
2416.60	7.11	7.26	7.35	7.01	7.10	7.02	6.88	6.82	6.88	6.77	6.84
2605.36	7.31	7.08	6.94	7.00	7.01	6.85	6.61	6.68	6.77	6.80	6.74
2808.94	7.45	7.26	6.92	7.18	7.03	6.73	6.65	6.79	6.76	6.77	6.63
3028.50	7.05	7.12	6.79	6.88	6.52	6.57	6.45	6.40	6.56	6.60	6.39
3265.30	7.34	7.38	7.09	7.00	7.01	6.88	7.01	6.94	6.88	6.82	6.76
3520.69	7.18	7.06	7.17	7.00	6.75	6.88	6.82	6.71	6.68	6.84	6.59
3796.13	7.08	6.88	6.84	6.60	6.79	6.81	6.71	6.51	6.57	6.58	6.58

Wavelength (nm) / Time (ps)	396.75	398.17	399.60	401.02	402.44	403.87	405.29	406.72	408.14	409.56	410.99
-3.78	-0.08	-0.03	-0.02	-0.06	-0.11	-0.03	-0.30	-0.13	-0.09	-0.21	-0.19
-3.28	-0.06	0.03	0.15	0.09	-0.10	-0.30	-0.06	0.07	0.00	0.13	-0.22
-2.78	-0.23	-0.17	-0.32	-0.04	-0.20	-0.20	-0.11	-0.14	-0.11	0.14	0.07
-2.28	0.04	0.03	-0.04	-0.09	-0.05	0.25	0.05	-0.11	-0.02	0.14	0.06
-1.78	0.20	0.22	0.00	0.15	0.16	-0.05	0.17	0.18	0.03	-0.12	0.17
-1.28	0.26	0.20	0.19	0.18	0.18	0.35	0.22	0.12	0.08	0.12	0.18
-0.78	-0.08	-0.16	0.02	-0.23	-0.02	0.14	0.01	-0.01	0.11	0.01	0.00
-0.28	-0.06	-0.12	0.01	0.00	0.14	-0.17	0.01	0.01	0.01	-0.21	-0.07
-0.18	-0.03	-0.15	-0.15	0.10	-0.10	-0.05	-0.02	-0.07	0.12	0.09	0.08
-0.08	0.06	0.05	0.15	0.13	-0.03	-0.11	0.11	0.15	0.00	-0.05	-0.12
0.02	-0.12	-0.19	-0.22	-0.04	-0.18	-0.02	-0.06	-0.09	-0.05	0.01	0.11
0.12	-0.01	-0.08	0.01	0.10	-0.33	-0.12	-0.06	-0.19	-0.08	0.14	-0.12
0.22	-0.05	-0.07	-0.20	-0.05	-0.13	-0.12	0.01	-0.24	-0.05	-0.27	-0.40
0.27	0.04	0.05	0.02	-0.01	0.08	0.24	0.14	-0.10	0.05	0.07	0.03
0.32	-0.22	-0.10	-0.33	-0.23	-0.21	-0.23	-0.18	-0.34	-0.38	-0.35	-0.31
0.37	0.18	0.21	0.12	0.29	-0.09	0.29	0.13	-0.01	-0.01	0.21	-0.02
0.42	0.26	0.12	0.20	0.01	-0.04	-0.01	0.08	0.13	-0.27	0.05	-0.01

0.47	0.29	0.17	0.20	0.45	0.29	0.51	0.27	0.24	0.28	0.16	0.17
0.52	0.01	0.11	-0.01	0.14	-0.11	0.25	0.13	0.08	-0.04	0.09	0.07
0.57	-0.04	-0.02	-0.07	-0.12	-0.07	-0.10	0.12	-0.19	-0.39	-0.12	-0.20
0.62	-0.14	-0.04	-0.07	-0.02	-0.13	0.12	-0.01	-0.27	-0.07	-0.12	-0.22
0.67	0.17	0.40	0.15	0.12	0.14	0.16	0.22	0.08	0.04	-0.04	-0.16
0.72	0.31	0.29	0.22	0.32	0.21	0.10	0.27	0.25	0.33	0.19	0.10
0.77	-0.94	-0.71	0.74	0.64	-0.03	0.30	0.19	0.05	0.06	0.19	0.14
0.82	-24.41	-27.57	-19.51	-3.42	4.93	2.32	-0.61	-0.14	0.10	-0.15	-0.08
0.87	44.51	1.34	-46.86	-79.41	-81.99	-58.15	-27.23	-7.27	-0.16	0.36	0.05
0.92	83.11	135.14	167.92	167.00	134.80	88.39	43.74	11.49	-5.11	-9.00	-5.04
0.97	-12.30	-26.60	-31.38	-21.02	3.05	35.21	66.58	92.09	109.45	119.21	122.66
1.02	14.96	14.55	13.77	14.89	15.65	10.16	-6.85	-34.92	-65.20	-88.91	-99.40
1.07	13.56	13.60	13.14	12.70	12.11	12.87	14.59	16.58	17.52	16.33	14.97
1.12	12.78	12.57	12.73	12.88	12.91	12.85	12.79	12.47	12.61	13.06	13.26
1.17	12.27	12.22	11.92	11.87	12.09	12.67	12.93	12.64	11.84	11.16	11.11
1.22	12.30	12.82	12.73	12.51	11.62	11.58	11.68	11.85	12.41	12.77	12.18
1.27	11.37	11.24	11.44	11.87	11.82	11.58	10.92	10.52	10.64	11.31	12.15
1.32	11.74	11.41	10.83	10.57	10.51	10.96	11.42	11.43	10.84	10.43	10.04
1.37	10.89	11.01	11.34	11.41	10.78	10.66	10.53	10.21	10.74	11.02	11.02
1.42	11.17	11.03	10.79	10.99	11.13	11.12	10.73	10.33	10.03	10.10	10.35
1.47	11.07	11.10	10.69	10.47	9.89	10.16	10.01	10.81	10.57	10.02	9.71
1.52	10.76	10.82	10.95	10.87	10.43	10.10	9.82	9.82	10.28	10.53	10.58
1.57	10.81	10.41	10.22	10.19	10.50	10.81	10.49	9.98	9.55	9.12	9.52
1.62	10.49	10.75	10.55	10.24	9.43	9.61	9.70	9.64	9.94	9.64	8.91
1.67	10.15	10.07	10.23	10.42	10.16	9.72	9.49	9.25	9.24	9.61	9.90
1.72	10.32	10.16	9.87	9.74	9.52	9.94	9.70	9.65	9.37	8.98	8.92
1.77	10.28	10.37	10.28	10.12	9.77	9.55	9.05	9.47	9.67	9.68	9.39
1.82	9.77	9.55	9.36	9.68	9.54	9.54	9.19	8.48	8.41	8.53	9.04
1.87	10.37	10.30	10.01	9.85	9.41	9.78	9.56	9.79	9.29	9.07	8.96
1.92	10.09	10.02	10.05	10.00	9.81	9.26	9.16	9.01	9.03	9.20	9.10
1.97	9.78	9.46	9.34	9.33	9.28	9.35	9.25	8.56	8.34	8.22	8.31
2.02	9.41	9.48	9.45	9.02	8.94	8.77	8.63	8.69	8.88	8.90	8.86
2.07	9.96	9.88	9.82	9.80	9.36	9.15	9.12	8.69	8.92	8.99	9.03
2.12	9.94	9.83	9.38	9.38	9.32	9.53	9.25	9.14	8.66	8.68	8.44
2.17	9.65	9.68	9.55	9.52	8.97	8.77	8.83	8.91	8.84	8.62	8.62
2.22	9.92	9.53	9.50	9.48	9.45	9.47	8.98	8.59	8.39	8.61	8.72
2.27	9.76	9.81	9.62	9.15	9.08	9.24	9.26	8.96	8.85	8.55	8.42
2.32	9.31	9.23	8.98	9.08	8.81	8.72	8.19	8.31	8.34	8.52	8.28
2.37	9.88	9.68	9.46	9.27	9.35	9.34	9.02	8.60	8.43	8.33	8.39
2.42	9.68	9.66	9.28	9.10	9.01	8.95	9.18	8.68	8.83	8.58	8.39
2.47	9.77	9.73	9.74	9.65	9.31	9.23	8.68	8.73	8.70	8.68	8.61
2.52	9.93	9.75	9.37	9.30	9.18	9.32	9.12	8.79	8.57	8.35	8.36
2.57	9.77	9.67	9.72	9.43	9.20	8.77	8.70	9.12	8.85	8.75	8.44
2.62	9.47	9.49	9.60	9.20	9.12	9.12	8.61	8.25	8.28	8.45	8.59
2.67	9.39	9.23	9.16	9.15	8.73	9.11	8.70	8.53	8.23	8.13	7.91
2.72	9.40	9.53	9.36	9.17	8.91	8.64	8.52	8.29	8.33	8.41	8.14
2.77	9.31	9.41	9.16	9.04	8.91	8.84	8.66	8.52	8.07	8.10	8.15
2.82	9.25	9.41	9.05	9.12	8.88	8.59	8.55	8.70	8.47	8.30	8.16
2.87	9.45	9.29	9.16	9.17	8.75	8.70	8.56	8.06	8.29	8.30	8.06
2.92	9.46	9.27	9.02	9.08	8.59	8.94	8.66	8.32	8.31	8.09	8.10

2.97	9.08	9.14	8.85	8.83	8.52	8.47	8.42	8.18	8.13	8.38	8.02
3.02	9.57	9.50	9.50	9.31	9.03	8.98	8.71	8.31	8.14	8.48	8.24
3.07	9.42	9.23	9.06	8.98	8.86	8.70	8.64	8.36	8.27	8.16	8.00
3.12	9.10	9.17	9.03	8.85	8.59	8.57	8.55	8.33	8.09	8.09	7.96
3.17	9.18	8.99	8.96	8.96	8.79	8.65	8.43	8.26	8.43	8.17	8.13
3.22	9.55	9.51	9.23	9.10	8.93	8.68	8.65	8.54	8.48	8.25	8.07
3.42	9.22	9.19	8.95	8.92	8.69	8.59	8.39	8.13	8.11	8.24	8.13
3.62	9.52	9.47	9.30	8.96	9.11	8.75	8.82	8.40	8.43	8.29	8.10
3.82	9.22	9.31	9.22	9.00	8.70	8.47	8.29	8.20	8.14	8.03	7.98
4.02	9.42	9.32	9.15	9.06	8.76	8.74	8.60	8.39	8.24	7.90	7.91
4.22	8.97	8.98	8.94	8.63	8.57	8.37	8.26	7.98	7.99	7.94	7.87
4.42	9.32	9.10	8.93	8.70	8.50	8.61	8.55	8.10	8.10	7.96	7.89
4.62	8.92	9.07	8.75	8.80	8.29	8.35	8.29	7.88	8.08	8.12	7.91
4.82	9.28	8.93	9.05	8.72	8.42	8.52	8.17	8.11	8.10	7.75	7.70
5.02	8.96	8.97	8.95	8.75	8.82	8.58	8.47	8.13	7.98	8.25	7.89
5.22	9.02	9.06	8.85	8.70	8.22	8.56	8.22	7.91	7.74	7.76	7.71
5.42	9.01	8.98	8.71	8.70	8.45	8.47	8.19	7.88	7.89	7.93	7.66
5.62	8.94	8.96	8.78	8.71	8.60	8.33	8.21	8.09	7.77	7.76	7.64
5.82	9.17	8.85	8.70	8.48	8.24	8.42	8.20	7.82	7.64	7.77	7.60
6.02	8.75	8.70	8.53	8.27	7.97	7.92	7.97	7.71	7.53	7.68	7.48
6.22	9.03	8.90	8.77	8.62	8.24	8.37	7.91	7.83	7.84	7.67	7.61
6.42	9.11	8.98	8.83	8.81	8.41	8.40	8.23	8.16	7.81	7.63	7.61
6.62	8.93	8.83	8.68	8.37	8.25	8.17	8.07	7.93	7.79	7.60	7.57
6.82	8.82	8.76	8.68	8.30	8.18	8.19	8.08	7.81	7.84	7.67	7.61
7.02	9.06	8.83	8.84	8.57	8.51	8.41	8.19	8.08	7.80	7.82	7.77
7.22	8.73	8.77	8.55	8.37	8.25	8.13	8.05	7.72	7.54	7.63	7.53
7.42	9.25	9.02	8.99	8.98	8.49	8.50	8.27	7.94	7.88	8.02	7.76
7.62	8.61	8.48	8.35	8.21	7.87	7.84	7.84	7.57	7.32	7.61	7.30
7.82	8.68	8.73	8.51	8.50	8.22	8.14	7.94	7.74	7.72	7.67	7.56
8.02	8.52	8.56	8.35	8.18	7.97	7.94	7.86	7.60	7.53	7.44	7.35
8.22	8.59	8.55	8.34	8.22	8.14	7.88	7.81	7.74	7.64	7.51	7.33
8.42	8.86	8.63	8.52	8.45	8.32	8.39	8.19	7.72	7.74	7.52	7.55
8.62	8.50	8.24	8.08	8.09	7.97	7.92	7.82	7.56	7.47	7.32	7.36
8.82	8.76	8.49	8.32	8.45	8.09	8.06	7.94	7.68	7.53	7.52	7.29
9.02	8.63	8.36	8.42	8.24	7.87	8.03	7.74	7.45	7.52	7.53	7.27
9.22	8.66	8.72	8.54	8.33	8.04	7.98	7.80	7.53	7.60	7.59	7.36
9.42	8.41	8.21	8.10	8.13	7.79	7.95	7.70	7.37	7.41	7.37	7.20
9.62	8.77	8.63	8.48	8.15	8.11	7.94	7.76	7.78	7.56	7.29	7.41
9.82	8.53	8.44	8.48	8.26	8.19	7.89	7.54	7.43	7.32	7.28	7.19
10.02	8.31	8.24	8.15	8.11	7.67	7.83	7.71	7.38	7.42	7.38	7.19
10.22	8.42	8.31	8.16	8.01	7.88	7.69	7.61	7.45	7.32	7.27	7.25
10.42	8.43	8.45	8.40	8.24	7.70	7.92	7.91	7.55	7.31	7.37	7.15
10.62	8.47	8.63	8.35	8.22	7.94	8.23	7.59	7.32	7.42	7.18	7.18
10.82	8.48	8.37	8.26	8.20	7.79	7.86	7.66	7.29	7.15	7.16	7.09
11.02	8.51	8.38	8.16	8.16	7.95	7.83	7.53	7.51	7.22	6.95	7.13
11.22	8.11	8.13	8.02	7.95	7.69	7.78	7.93	7.26	7.14	7.35	7.16
11.42	8.20	8.10	7.75	7.65	7.51	7.40	7.42	6.86	7.09	6.98	6.97
11.62	8.57	8.44	8.29	8.30	7.94	8.00	7.81	7.63	7.28	7.41	7.18
11.82	8.53	8.30	8.12	8.03	7.78	7.81	7.37	7.32	7.38	7.30	7.17
12.02	8.42	8.37	7.92	7.83	7.56	7.75	7.41	7.20	7.26	7.07	6.95

12.22	8.60	8.59	8.54	8.43	7.95	7.93	7.81	7.66	7.35	7.37	7.21
12.42	8.50	8.40	8.24	8.17	7.88	7.76	7.76	7.54	7.31	7.33	7.01
12.62	8.16	7.90	7.84	7.91	7.48	7.61	7.23	7.23	7.13	6.99	7.03
12.82	7.83	7.95	7.77	7.52	7.36	7.19	7.32	7.06	6.90	6.74	6.86
13.02	8.60	8.61	8.36	8.05	7.91	7.94	7.47	7.41	7.20	7.00	7.08
13.22	8.38	8.40	8.13	8.10	7.81	7.59	7.67	7.40	7.33	7.25	7.00
13.42	8.31	8.31	8.26	8.08	7.84	7.80	7.66	7.50	7.34	7.10	7.19
13.50	7.99	8.07	7.77	7.75	7.54	7.56	7.28	7.21	7.00	7.19	6.76
13.59	8.22	8.27	8.10	7.94	7.54	7.52	7.42	7.45	7.14	7.09	6.92
13.68	8.20	8.12	8.20	7.91	7.64	7.55	7.59	7.18	7.33	7.18	7.02
13.78	8.29	8.11	8.00	7.89	7.70	7.67	7.60	7.09	7.10	7.01	6.91
13.88	8.45	8.16	8.27	7.93	7.71	7.67	7.50	7.18	7.18	7.30	7.05
14.00	8.35	8.35	8.01	8.09	7.75	7.51	7.30	7.28	7.21	7.17	6.93
14.12	8.35	8.36	8.24	8.12	7.78	7.49	7.53	7.30	7.22	7.12	7.04
14.25	8.35	8.34	8.08	8.11	7.65	7.60	7.46	7.16	6.80	6.99	6.77
14.40	8.56	8.60	8.28	8.20	7.86	7.90	7.73	7.61	7.32	7.20	7.15
14.55	8.35	8.33	8.18	7.97	7.86	7.64	7.48	7.27	7.10	7.09	6.89
14.72	8.42	8.53	8.29	8.10	7.86	7.73	7.48	7.26	7.31	7.09	6.82
14.90	7.96	7.85	7.72	7.65	7.30	7.21	7.17	6.99	6.85	6.80	6.49
15.09	8.16	8.08	7.86	7.69	7.57	7.63	7.35	7.17	7.04	6.88	6.83
15.30	8.08	7.98	7.77	8.06	7.62	7.35	7.40	7.08	7.18	7.05	6.91
15.53	8.42	8.42	8.37	8.31	7.97	7.71	7.68	7.49	7.39	7.36	7.27
15.77	8.42	8.37	8.16	8.13	7.72	7.78	7.37	7.22	7.33	7.16	7.04
16.04	8.51	8.59	8.37	8.21	7.78	8.07	7.64	7.27	7.17	7.22	7.12
16.32	8.13	8.02	8.06	7.92	7.64	7.39	7.23	7.17	6.96	6.81	6.91
16.63	8.34	8.17	8.10	7.83	7.53	7.46	7.35	7.22	6.99	6.88	7.14
16.96	8.34	8.30	8.03	8.00	7.64	7.78	7.55	7.23	7.17	7.05	7.03
17.31	8.40	8.33	8.05	7.82	7.77	7.65	7.42	7.24	7.53	7.09	6.99
17.70	8.36	8.22	7.96	8.02	7.74	7.83	7.51	7.08	7.20	7.24	7.13
18.11	8.07	8.22	7.92	8.05	7.55	7.42	7.30	7.04	7.04	7.03	6.87
18.56	8.01	8.04	7.78	7.87	7.53	7.48	7.46	7.05	7.03	7.00	6.76
19.04	8.13	7.98	7.74	7.83	7.52	7.35	7.28	7.18	7.06	6.81	6.83
19.56	8.22	8.06	7.93	7.83	7.54	7.59	7.14	7.25	7.02	6.96	6.83
20.12	8.18	8.17	7.84	7.85	7.59	7.64	7.47	7.16	6.96	7.14	6.93
20.72	8.42	8.29	8.18	8.00	7.64	7.56	7.42	7.21	7.20	6.90	6.90
21.37	8.19	8.07	8.07	7.92	7.49	7.48	7.33	7.20	7.04	6.97	6.88
22.08	8.88	8.69	8.62	8.41	8.11	8.07	7.80	7.79	7.47	7.36	7.24
22.84	8.26	8.20	8.00	7.80	7.66	7.67	7.33	7.11	7.24	6.98	7.01
23.65	8.38	8.22	7.86	7.92	7.51	7.47	7.37	7.20	7.06	6.97	6.70
24.54	8.07	7.92	7.89	7.73	7.66	7.41	7.25	7.08	7.03	6.89	6.83
25.49	8.37	8.33	8.08	8.10	7.79	7.32	7.40	6.96	7.25	7.14	6.94
26.51	8.03	7.90	7.74	7.74	7.39	7.14	7.07	6.94	6.88	6.92	6.83
27.62	8.20	8.11	7.89	7.84	7.36	7.36	7.35	6.88	6.86	6.89	6.99
28.81	8.18	8.25	8.15	8.04	7.69	7.47	7.27	7.18	6.99	6.96	6.77
30.10	7.87	7.80	7.59	7.37	7.57	7.36	6.92	6.66	6.96	6.72	6.56
31.49	8.01	7.94	7.78	7.68	7.40	7.09	6.92	6.79	6.82	6.80	6.40
32.98	7.93	7.89	7.59	7.66	7.29	7.11	7.10	6.97	6.60	6.77	6.44
34.60	7.97	7.88	7.78	7.48	7.21	7.21	7.10	6.96	6.65	6.66	6.65
36.34	7.95	7.92	7.91	7.54	7.32	7.48	7.22	6.87	7.05	6.76	6.63
38.21	7.80	7.73	7.46	7.52	7.12	7.17	6.89	6.62	6.52	6.68	6.54

40.24	8.18	8.18	7.98	8.01	7.45	7.32	7.29	7.11	6.93	6.78	6.75
42.42	7.58	7.47	7.45	7.38	7.11	7.03	6.91	6.64	6.57	6.67	6.31
44.78	8.02	7.88	7.66	7.37	7.21	7.25	6.87	6.77	6.59	6.55	6.49
47.32	7.99	8.00	7.58	7.67	7.23	7.26	6.94	6.82	6.67	6.38	6.39
50.06	8.11	7.98	7.81	7.77	7.57	7.15	7.07	6.87	6.92	6.69	6.58
53.01	8.11	7.99	7.78	7.79	7.51	7.26	7.19	6.95	6.80	6.81	6.55
56.20	7.93	7.87	7.64	7.48	7.25	7.11	6.88	6.62	6.51	6.66	6.55
59.64	7.58	7.56	7.24	7.34	6.99	7.14	6.92	6.62	6.46	6.60	6.44
63.34	7.78	7.71	7.53	7.23	7.05	6.96	6.73	6.57	6.39	6.55	6.31
67.34	7.56	7.39	7.23	7.00	6.84	6.82	6.62	6.49	6.52	6.32	6.28
71.65	7.65	7.55	7.32	7.31	6.87	6.88	6.72	6.72	6.54	6.47	6.51
76.30	7.81	7.67	7.47	7.25	7.26	7.04	6.81	6.85	6.45	6.41	6.32
81.32	7.79	7.59	7.51	7.56	6.99	6.93	6.64	6.76	6.31	6.34	6.40
86.73	7.77	7.83	7.61	7.39	6.97	6.95	6.80	6.50	6.43	6.37	6.25
92.56	7.54	7.54	7.43	7.21	6.72	6.87	6.60	6.44	6.27	6.13	6.19
98.85	7.53	7.47	7.37	6.91	6.92	6.81	6.54	6.36	6.14	6.31	6.18
105.64	7.61	7.61	7.31	7.32	7.09	6.85	6.75	6.45	6.38	6.37	6.21
112.96	7.67	7.56	7.38	7.43	6.95	6.89	6.88	6.52	6.43	6.34	6.12
120.85	7.14	7.13	6.92	6.75	6.45	6.48	6.15	6.10	6.02	5.96	5.93
129.36	7.76	7.75	7.48	7.33	6.95	6.91	6.70	6.43	6.50	6.46	6.17
138.55	7.56	7.51	7.21	7.06	6.76	6.67	6.38	6.31	5.93	6.21	6.12
148.45	7.75	7.64	7.51	7.41	6.88	6.42	6.52	6.38	6.23	6.41	6.10
159.13	7.59	7.29	7.23	7.23	6.96	6.84	6.46	6.35	6.26	6.18	6.05
170.65	7.52	7.44	7.51	7.08	6.81	6.73	6.48	6.54	6.43	6.26	6.17
183.07	7.38	7.25	7.04	6.94	6.70	6.75	6.27	6.32	6.07	6.01	6.01
196.47	7.39	7.33	7.18	7.03	6.65	6.59	6.54	6.06	5.89	6.20	6.07
210.91	7.36	7.24	7.14	6.84	6.71	6.42	6.43	6.31	5.88	6.13	5.94
226.50	7.13	6.96	6.94	6.81	6.52	6.36	6.13	5.94	6.03	5.78	5.74
243.31	7.24	7.10	7.00	6.82	6.63	6.45	6.35	6.13	5.85	5.79	5.67
261.43	7.35	7.17	7.11	6.99	6.62	6.59	6.44	6.17	6.08	5.87	6.04
280.98	7.24	7.18	6.95	6.85	6.51	6.49	6.17	5.87	6.07	5.87	5.86
302.07	7.06	6.98	6.81	6.39	6.31	6.25	5.93	5.97	5.84	5.85	5.77
324.81	6.99	6.95	6.71	6.55	6.34	6.52	6.02	5.81	5.89	5.93	5.97
349.33	6.95	6.73	6.43	6.45	6.28	6.13	5.87	5.74	5.65	5.69	5.65
375.79	7.19	6.90	6.82	6.60	6.33	6.41	5.98	5.87	5.85	5.69	5.78
404.31	6.93	6.86	6.64	6.47	6.46	6.07	5.98	5.92	5.69	5.58	5.66
435.08	7.42	7.39	7.00	6.94	6.53	6.52	6.21	5.98	6.02	5.95	5.70
468.27	7.14	7.07	6.75	6.78	6.28	6.34	6.22	5.95	5.90	5.68	5.83
504.06	6.97	6.88	6.70	6.52	6.16	5.93	5.80	5.80	5.71	5.68	5.60
542.66	7.13	6.90	6.76	6.64	6.28	6.01	6.14	5.92	5.92	5.71	5.76
584.29	7.03	6.80	6.67	6.47	6.14	6.01	5.84	5.80	5.55	5.41	5.54
629.18	6.89	6.66	6.40	6.43	6.16	6.11	5.81	5.76	5.56	5.47	5.42
677.61	7.18	6.89	6.70	6.53	6.43	6.10	5.82	5.74	5.74	5.75	5.56
729.83	6.42	6.23	6.29	6.15	5.80	5.58	5.47	5.16	5.45	5.21	5.32
786.16	6.77	6.51	6.22	6.18	5.71	5.73	5.74	5.28	5.53	5.31	5.38
846.90	6.84	6.52	6.31	6.05	5.77	5.66	5.57	5.57	5.19	5.31	5.33
912.42	6.44	6.26	6.14	5.86	5.74	5.67	5.55	5.44	5.22	5.52	5.35
983.08	6.69	6.47	6.31	6.21	5.97	5.80	5.62	5.37	5.26	5.25	5.38
1059.29	6.56	6.35	6.16	5.84	5.78	5.55	5.23	5.37	5.26	5.27	5.15
1141.48	6.34	6.49	6.16	5.94	5.87	5.87	5.55	5.28	5.29	5.30	5.14

1230.13	6.69	6.49	6.34	6.05	5.82	5.64	5.65	5.36	5.27	5.28	5.09
1325.73	6.99	6.82	6.70	6.38	6.09	6.06	5.82	5.77	5.52	5.43	5.32
1428.84	6.78	6.39	6.22	6.22	6.05	5.82	5.72	5.52	5.48	5.36	5.43
1540.05	6.84	6.75	6.42	6.21	5.88	5.70	5.71	5.66	5.50	5.66	5.52
1659.99	6.54	6.44	6.23	5.94	5.76	5.67	5.43	5.34	5.19	5.33	5.28
1789.34	6.70	6.44	6.41	6.14	5.92	5.88	5.71	5.47	5.45	5.50	5.41
1928.85	6.61	6.27	6.10	5.91	5.85	5.61	5.48	5.21	5.18	5.37	5.29
2079.31	6.62	6.47	6.28	5.96	5.75	5.71	5.58	5.55	5.45	5.37	5.09
2241.59	6.59	6.48	6.27	6.01	5.79	5.71	5.84	5.37	5.34	5.39	5.26
2416.60	6.78	6.52	6.36	6.10	5.76	5.69	5.89	5.73	5.48	5.41	5.13
2605.36	6.65	6.42	6.17	6.14	5.63	5.54	5.59	5.36	5.14	5.33	5.25
2808.94	6.65	6.43	6.16	5.95	5.72	5.64	5.55	5.35	5.37	5.24	5.36
3028.50	6.29	6.15	5.86	5.75	5.73	5.28	5.11	4.90	5.03	5.05	4.94
3265.30	6.61	6.47	6.08	6.02	5.75	5.81	5.52	5.25	5.25	5.13	4.97
3520.69	6.77	6.40	6.34	6.11	5.88	5.58	5.39	5.33	5.16	5.26	5.33
3796.13	6.71	6.49	6.14	5.71	5.52	5.40	5.32	5.15	5.14	5.05	4.97

Wavelength (nm) / Time (ps)	412.41	413.84	415.26	416.68	418.11	419.53	420.96	422.38	423.80	425.23	426.65
-3.78	-0.11	-0.03	-0.03	0.00	0.01	-0.12	-0.01	-0.11	-0.12	0.06	-0.02
-3.28	0.07	-0.05	-0.13	-0.13	-0.22	-0.14	-0.15	-0.10	-0.10	-0.06	-0.10
-2.78	0.06	0.05	0.13	0.17	0.11	0.13	0.24	0.09	0.19	0.18	0.26
-2.28	0.01	-0.12	0.02	-0.03	-0.04	-0.01	-0.06	-0.01	0.06	-0.11	0.00
-1.78	-0.01	0.02	-0.02	0.00	-0.07	-0.01	-0.03	-0.01	0.01	0.01	-0.09
-1.28	0.03	0.14	0.17	0.09	0.04	0.01	-0.08	0.05	0.09	-0.02	-0.02
-0.78	-0.03	0.12	0.03	-0.10	0.17	0.11	0.07	0.02	-0.03	-0.03	-0.06
-0.28	-0.01	-0.12	-0.16	0.01	0.01	0.01	0.01	0.06	-0.10	-0.03	0.03
-0.18	0.08	0.13	0.11	0.01	0.15	0.10	0.01	0.06	-0.01	-0.03	-0.01
-0.08	0.16	0.11	0.05	0.00	-0.06	0.02	-0.04	-0.09	-0.07	-0.11	-0.18
0.02	0.10	0.12	0.01	0.14	0.11	0.13	0.09	0.06	0.03	0.05	0.02
0.12	-0.02	-0.12	-0.25	-0.15	-0.12	-0.12	-0.11	-0.17	-0.07	-0.17	-0.06
0.22	0.11	-0.05	-0.09	-0.15	-0.06	-0.01	0.02	0.02	-0.03	-0.08	-0.09
0.27	-0.03	0.08	-0.05	0.01	0.03	0.09	-0.01	-0.13	-0.04	-0.08	-0.14
0.32	-0.37	-0.23	-0.23	-0.23	-0.13	-0.13	-0.14	-0.04	0.07	-0.16	-0.04
0.37	0.10	0.05	-0.02	0.00	-0.08	-0.06	-0.13	-0.08	-0.08	-0.08	-0.13
0.42	0.07	-0.02	0.02	-0.09	-0.03	0.00	-0.02	-0.06	0.05	-0.03	-0.01
0.47	0.12	0.24	0.16	0.21	0.16	0.12	0.14	0.07	0.12	0.13	-0.04
0.52	0.14	0.09	-0.01	0.03	0.07	0.06	0.03	-0.04	0.02	-0.05	-0.09
0.57	-0.08	-0.09	-0.15	-0.10	-0.13	-0.14	-0.04	-0.03	-0.05	-0.08	-0.16
0.62	-0.06	-0.15	-0.09	-0.12	0.08	-0.03	0.00	0.01	0.03	-0.17	0.01
0.67	0.00	-0.09	0.00	-0.03	0.05	0.00	0.03	-0.03	-0.01	0.03	0.01
0.72	0.25	0.05	0.06	0.17	0.02	0.14	0.17	0.07	0.13	-0.06	0.04
0.77	0.20	0.20	0.02	-0.07	-0.01	0.01	-0.03	-0.08	-0.10	-0.08	-0.11
0.82	-0.02	0.09	-0.02	0.02	0.06	0.02	0.09	0.02	0.07	-0.13	-0.12
0.87	0.13	0.50	0.65	-0.04	-0.97	-0.79	0.14	0.21	-0.16	-0.22	-0.03
0.92	1.20	3.86	1.08	-6.74	-15.43	-19.75	-15.92	-6.48	0.97	2.71	1.05
0.97	121.03	115.68	106.82	92.61	69.57	36.33	-0.61	-31.81	-49.41	-52.56	-45.87
1.02	-93.95	-73.21	-39.46	4.48	54.52	102.37	139.31	158.41	159.76	146.81	124.90

1.07	16.36	19.20	17.89	7.92	-10.81	-32.54	-49.71	-56.26	-49.61	-30.68	-1.84
1.12	13.16	12.35	11.86	12.21	13.53	15.27	16.42	15.69	14.22	13.80	14.72
1.17	12.01	12.91	13.10	12.80	12.42	12.39	12.49	12.41	11.98	10.99	10.03
1.22	11.66	11.89	12.75	13.26	13.23	12.31	11.17	10.57	10.74	11.30	11.60
1.27	12.27	11.72	11.05	10.81	10.94	11.56	11.71	11.21	10.71	10.24	10.35
1.32	10.47	11.18	11.48	11.12	10.49	10.12	10.35	10.78	11.01	10.57	9.75
1.37	10.39	10.04	9.99	10.33	10.81	10.92	10.53	9.85	9.47	9.14	9.32
1.42	10.94	10.96	10.59	10.23	10.11	10.32	10.31	10.03	9.55	8.70	8.23
1.47	9.70	10.06	10.57	10.61	10.31	9.64	9.34	9.28	9.58	9.61	9.34
1.52	10.42	9.64	9.54	9.74	10.14	10.16	9.80	9.04	8.66	8.45	8.54
1.57	9.96	10.21	9.93	9.36	9.15	9.43	9.56	9.50	9.10	8.38	7.58
1.62	8.76	9.32	9.84	10.09	9.92	9.21	8.75	8.58	8.64	8.75	8.46
1.67	9.75	9.24	9.02	9.02	9.24	9.46	9.11	8.57	8.12	7.85	7.84
1.72	9.12	9.49	9.56	9.23	8.82	8.83	8.67	8.69	8.74	8.46	8.04
1.77	9.04	8.86	8.96	9.34	9.63	9.30	8.81	8.31	8.12	8.00	7.79
1.82	9.39	9.29	8.79	8.52	8.55	8.75	8.80	8.52	7.96	7.34	7.05
1.87	9.02	9.39	9.40	9.35	8.89	8.61	8.43	8.39	8.50	8.23	7.81
1.92	9.16	8.65	8.39	8.46	8.92	8.91	8.57	8.13	7.82	7.62	7.61
1.97	8.68	8.86	8.75	8.41	8.26	8.27	8.40	8.31	8.03	7.52	7.09
2.02	8.66	8.57	8.69	8.99	9.08	8.74	8.42	8.18	7.91	7.85	7.68
2.07	8.91	8.50	8.32	8.45	8.69	8.68	8.38	8.01	7.47	7.33	7.28
2.12	8.62	8.77	8.83	8.63	8.31	8.22	8.22	8.16	7.91	7.57	7.02
2.17	8.28	8.42	8.50	8.60	8.46	8.22	7.83	7.40	7.44	7.25	7.04
2.22	8.96	8.88	8.42	8.19	8.33	8.31	8.24	7.89	7.53	7.22	6.94
2.27	8.60	8.65	8.90	8.71	8.51	8.20	8.02	8.05	7.88	7.61	7.28
2.32	8.23	8.05	7.89	8.06	8.26	8.05	7.83	7.45	7.22	6.95	6.87
2.37	8.74	8.73	8.53	8.25	8.20	8.05	8.12	8.00	7.70	7.07	6.73
2.42	8.26	8.43	8.46	8.56	8.24	8.05	7.80	7.59	7.47	7.34	6.99
2.47	8.51	8.48	8.21	8.25	8.37	8.16	7.96	7.61	7.26	7.01	6.95
2.52	8.43	8.59	8.50	8.34	8.12	8.02	7.94	7.85	7.54	7.11	6.73
2.57	8.34	8.29	8.39	8.36	8.50	8.17	7.80	7.45	7.36	7.15	6.95
2.62	8.66	8.42	8.22	8.19	8.18	8.20	8.00	7.70	7.16	7.03	6.73
2.67	8.28	8.39	8.27	8.23	7.93	7.82	7.75	7.54	7.48	7.19	6.74
2.72	8.01	8.00	8.01	8.05	8.22	8.03	7.77	7.27	7.10	6.90	6.78
2.77	8.25	8.27	8.19	7.98	7.87	7.77	7.97	7.59	7.32	6.93	6.68
2.82	8.21	8.21	8.32	8.32	8.12	7.90	7.79	7.46	7.40	7.06	6.79
2.87	8.20	8.17	8.09	8.03	8.02	7.86	7.59	7.26	7.10	6.91	6.64
2.92	8.14	8.43	8.45	8.10	8.02	7.85	7.79	7.60	7.46	7.05	6.66
2.97	8.24	8.06	8.15	8.17	8.01	7.94	7.78	7.41	7.24	7.05	6.79
3.02	8.44	8.23	8.12	8.08	7.96	7.82	7.67	7.44	7.07	6.86	6.69
3.07	8.12	8.26	8.18	8.15	7.99	7.71	7.67	7.43	7.22	6.94	6.61
3.12	7.95	7.84	7.92	7.99	7.86	7.72	7.54	7.21	6.96	6.84	6.65
3.17	8.26	8.52	8.20	8.07	8.06	7.98	7.90	7.66	7.37	6.98	6.68
3.22	8.02	8.21	8.24	8.11	8.14	7.84	7.67	7.54	7.44	7.06	6.81
3.42	8.05	8.20	8.02	7.99	7.99	8.00	7.75	7.40	7.14	6.84	6.52
3.62	8.19	8.22	8.23	8.11	7.90	7.92	7.70	7.57	7.30	6.97	6.75
3.82	7.92	7.85	7.86	7.91	7.81	7.68	7.61	7.30	6.97	6.81	6.55
4.02	8.02	8.11	8.06	8.03	7.85	7.62	7.47	7.25	7.08	6.89	6.56
4.22	7.84	7.99	7.75	7.73	7.68	7.63	7.53	7.21	7.09	6.70	6.49
4.42	7.95	7.89	7.72	7.74	7.78	7.52	7.47	7.14	6.97	6.66	6.45

4.62	8.01	7.92	7.95	7.93	7.80	7.80	7.64	7.21	7.02	6.72	6.42
4.82	7.80	7.77	7.57	7.66	7.55	7.38	7.26	7.08	6.87	6.52	6.33
5.02	7.91	7.88	7.81	7.93	7.90	7.68	7.50	7.24	7.05	6.69	6.44
5.22	7.70	7.69	7.72	7.66	7.61	7.40	7.30	7.14	6.94	6.53	6.47
5.42	7.71	7.70	7.75	7.52	7.59	7.40	7.35	7.14	6.85	6.56	6.32
5.62	7.76	7.70	7.59	7.56	7.46	7.41	7.38	7.01	6.72	6.50	6.29
5.82	7.72	7.67	7.61	7.53	7.48	7.34	7.12	6.83	6.63	6.35	6.05
6.02	7.52	7.59	7.50	7.55	7.43	7.27	7.21	6.85	6.77	6.56	6.16
6.22	7.73	7.59	7.63	7.60	7.51	7.40	7.16	6.98	6.66	6.45	6.11
6.42	7.65	7.67	7.61	7.45	7.41	7.35	7.14	6.99	6.66	6.45	6.11
6.62	7.67	7.40	7.67	7.61	7.39	7.37	7.26	6.89	6.71	6.52	6.17
6.82	7.65	7.62	7.53	7.60	7.53	7.40	7.10	6.90	6.71	6.44	6.19
7.02	7.67	7.67	7.66	7.62	7.68	7.38	7.19	6.88	6.71	6.45	6.32
7.22	7.51	7.45	7.49	7.44	7.40	7.23	7.11	6.81	6.64	6.42	6.18
7.42	7.55	7.71	7.59	7.56	7.35	7.41	7.13	6.81	6.69	6.36	6.07
7.62	7.47	7.43	7.52	7.50	7.37	7.25	7.11	6.90	6.67	6.37	6.12
7.82	7.50	7.53	7.53	7.50	7.25	7.20	7.07	6.74	6.62	6.24	6.10
8.02	7.56	7.39	7.29	7.39	7.20	7.15	7.00	6.70	6.52	6.22	5.96
8.22	7.32	7.30	7.37	7.23	7.20	7.11	6.95	6.65	6.43	6.20	6.00
8.42	7.54	7.49	7.40	7.32	7.25	7.14	7.01	6.74	6.49	6.25	6.04
8.62	7.32	7.27	7.32	7.25	7.15	7.14	6.97	6.75	6.54	6.34	5.93
8.82	7.36	7.33	7.27	7.36	7.33	7.23	7.01	6.84	6.62	6.39	6.05
9.02	7.23	7.39	7.24	7.14	7.14	7.03	6.96	6.63	6.35	6.24	5.88
9.22	7.48	7.36	7.35	7.20	7.28	7.15	6.85	6.72	6.42	6.14	5.90
9.42	7.21	7.29	7.06	7.23	7.17	7.04	6.74	6.64	6.45	6.21	5.91
9.62	7.40	7.22	7.25	7.20	7.15	6.99	6.82	6.53	6.49	6.12	5.96
9.82	7.26	7.26	7.25	7.18	7.20	6.92	6.96	6.54	6.40	6.30	5.98
10.02	7.24	7.29	7.33	7.28	7.24	7.03	6.94	6.77	6.49	6.23	5.87
10.22	7.31	7.25	7.28	7.17	7.22	7.08	6.95	6.62	6.53	6.32	5.99
10.42	7.16	7.12	7.11	6.95	7.08	6.92	6.82	6.52	6.34	6.03	5.81
10.62	7.00	7.38	7.33	7.15	7.16	7.04	6.85	6.65	6.41	6.11	5.93
10.82	7.13	7.08	7.03	7.02	6.96	6.94	6.86	6.55	6.35	6.02	5.85
11.02	7.22	7.21	7.14	7.13	7.03	7.03	6.85	6.56	6.36	6.06	5.91
11.22	7.15	7.14	7.02	7.03	7.00	6.91	6.76	6.62	6.29	6.00	5.79
11.42	6.96	7.04	7.05	6.96	6.95	6.85	6.66	6.42	6.28	6.00	5.71
11.62	7.34	7.27	7.20	7.11	6.91	6.88	6.67	6.45	6.34	5.98	5.80
11.82	7.34	7.30	7.22	7.24	7.02	6.87	6.81	6.56	6.36	6.12	5.75
12.02	6.93	6.96	6.92	6.87	6.85	6.61	6.50	6.37	6.12	5.97	5.77
12.22	7.19	7.24	7.09	7.08	7.04	6.96	6.82	6.54	6.41	6.23	5.96
12.42	7.28	7.27	7.04	7.12	7.09	6.92	6.75	6.54	6.20	5.99	5.73
12.62	7.11	7.08	7.07	6.94	7.07	6.85	6.69	6.46	6.23	6.01	5.77
12.82	6.91	6.78	6.84	6.90	6.78	6.68	6.65	6.44	6.21	5.88	5.63
13.02	6.93	6.87	6.95	6.94	6.79	6.77	6.64	6.43	6.28	5.94	5.76
13.22	7.15	7.10	7.15	6.98	6.92	6.90	6.78	6.51	6.26	5.98	5.65
13.42	7.00	7.16	7.07	7.04	7.00	6.84	6.71	6.46	6.19	6.05	5.71
13.50	7.01	6.91	7.01	6.85	6.85	6.72	6.64	6.33	6.09	5.88	5.58
13.59	6.91	6.94	6.87	6.94	6.96	6.72	6.70	6.49	6.21	5.96	5.79
13.68	7.05	6.94	6.96	6.91	6.84	6.82	6.50	6.34	6.12	5.89	5.57
13.78	7.04	7.03	6.97	6.89	6.79	6.71	6.52	6.32	6.01	5.73	5.59
13.88	7.18	7.04	6.94	6.97	6.93	6.83	6.73	6.37	6.17	5.97	5.66

14.00	6.98	7.11	6.84	6.80	6.86	6.75	6.67	6.34	6.24	5.91	5.70
14.12	7.19	7.01	6.95	7.02	6.96	6.76	6.65	6.35	6.25	6.03	5.62
14.25	6.86	6.88	6.83	6.74	6.65	6.62	6.44	6.26	5.98	5.70	5.56
14.40	7.17	7.18	7.03	6.99	6.92	6.84	6.61	6.38	6.20	5.90	5.70
14.55	7.05	6.98	6.95	6.85	6.83	6.71	6.59	6.34	6.03	5.95	5.63
14.72	7.01	6.95	6.88	6.83	6.81	6.73	6.50	6.20	6.07	5.70	5.38
14.90	6.58	6.58	6.70	6.59	6.62	6.48	6.31	6.20	6.01	5.73	5.48
15.09	6.87	6.75	6.88	6.82	6.80	6.70	6.55	6.25	6.08	5.75	5.47
15.30	6.88	6.85	7.01	6.87	6.77	6.75	6.63	6.33	6.14	5.89	5.61
15.53	7.30	7.26	7.20	7.10	6.90	6.84	6.76	6.45	6.30	5.98	5.75
15.77	7.00	6.94	6.80	6.87	6.76	6.54	6.46	6.25	6.02	5.83	5.54
16.04	7.21	7.12	7.06	7.05	6.95	6.77	6.60	6.46	6.18	6.00	5.76
16.32	6.95	6.99	6.94	6.78	6.82	6.68	6.46	6.29	6.06	5.83	5.57
16.63	6.94	7.17	7.05	7.05	7.00	6.85	6.61	6.44	6.24	5.94	5.71
16.96	7.16	7.23	7.09	6.98	6.98	6.83	6.68	6.44	6.21	5.87	5.63
17.31	7.05	6.96	6.80	6.87	6.81	6.63	6.57	6.36	6.14	5.85	5.57
17.70	7.02	7.15	7.14	7.06	6.97	6.97	6.74	6.39	6.32	5.93	5.71
18.11	7.01	6.98	6.92	6.86	6.75	6.68	6.50	6.25	6.00	5.78	5.54
18.56	6.72	6.78	6.74	6.88	6.64	6.74	6.47	6.24	5.99	5.77	5.55
19.04	7.03	7.00	6.78	6.83	6.78	6.66	6.53	6.26	6.12	5.84	5.56
19.56	6.81	6.89	6.67	6.82	6.76	6.75	6.44	6.24	6.14	5.89	5.69
20.12	6.96	7.01	6.89	6.92	6.82	6.69	6.62	6.25	6.12	5.79	5.57
20.72	7.02	6.89	6.83	6.78	6.75	6.69	6.62	6.29	6.05	5.92	5.68
21.37	6.99	6.94	6.98	6.85	6.75	6.64	6.49	6.36	6.15	5.82	5.60
22.08	7.24	7.25	7.10	7.01	6.95	6.81	6.76	6.47	6.20	5.94	5.69
22.84	7.08	6.99	7.07	6.92	6.89	6.77	6.66	6.45	6.19	5.89	5.73
23.65	6.81	6.91	6.70	6.66	6.58	6.48	6.34	6.07	5.90	5.59	5.38
24.54	6.83	6.73	6.68	6.74	6.83	6.59	6.49	6.29	5.96	5.69	5.45
25.49	7.24	7.00	6.92	6.96	6.87	6.75	6.46	6.25	6.13	5.81	5.59
26.51	6.94	6.73	6.79	6.77	6.63	6.60	6.41	6.05	5.97	5.54	5.29
27.62	6.97	6.94	6.82	6.82	6.72	6.64	6.57	6.26	6.03	5.74	5.54
28.81	6.80	6.74	6.81	6.68	6.68	6.62	6.46	6.18	6.06	5.58	5.47
30.10	6.82	6.85	6.75	6.56	6.57	6.52	6.26	6.01	5.91	5.58	5.31
31.49	6.58	6.65	6.48	6.54	6.44	6.39	6.35	6.08	5.93	5.60	5.36
32.98	6.65	6.66	6.70	6.65	6.56	6.52	6.31	5.96	5.78	5.70	5.48
34.60	6.71	6.68	6.72	6.63	6.61	6.49	6.39	6.13	5.81	5.64	5.42
36.34	6.69	6.75	6.70	6.62	6.65	6.52	6.35	6.15	5.87	5.58	5.31
38.21	6.50	6.59	6.62	6.70	6.65	6.41	6.43	6.15	5.96	5.73	5.43
40.24	6.71	6.85	6.74	6.64	6.56	6.39	6.33	6.11	5.85	5.60	5.31
42.42	6.50	6.49	6.48	6.35	6.35	6.27	6.17	5.93	5.71	5.39	5.29
44.78	6.49	6.45	6.46	6.43	6.33	6.13	6.08	5.83	5.65	5.38	5.17
47.32	6.42	6.42	6.41	6.36	6.31	6.07	5.99	5.63	5.51	5.29	5.00
50.06	6.64	6.63	6.58	6.53	6.53	6.40	6.12	5.98	5.61	5.44	5.18
53.01	6.68	6.62	6.62	6.48	6.47	6.39	6.31	5.92	5.81	5.49	5.14
56.20	6.46	6.64	6.45	6.47	6.39	6.25	6.16	5.93	5.70	5.42	5.20
59.64	6.46	6.39	6.60	6.55	6.53	6.31	6.17	5.88	5.66	5.30	5.17
63.34	6.46	6.30	6.22	6.23	6.14	6.07	6.02	5.78	5.48	5.35	5.11
67.34	6.37	6.30	6.26	6.23	6.23	6.05	6.00	5.67	5.41	5.32	4.98
71.65	6.47	6.36	6.44	6.36	6.49	6.26	6.18	5.81	5.66	5.38	5.14
76.30	6.33	6.35	6.32	6.31	6.24	6.06	5.98	5.74	5.49	5.33	4.98

81.32	6.43	6.33	6.42	6.29	6.28	6.21	6.02	5.82	5.66	5.42	5.21
86.73	6.23	6.35	6.20	6.21	6.12	6.25	5.93	5.75	5.57	5.33	5.06
92.56	6.19	6.24	6.17	6.24	6.20	6.12	6.03	5.85	5.68	5.32	5.01
98.85	6.09	6.14	6.15	6.20	6.06	6.00	5.81	5.70	5.54	5.21	5.05
105.64	6.23	6.19	6.18	6.18	6.12	5.97	5.96	5.60	5.34	5.30	4.96
112.96	6.13	6.31	6.08	6.13	6.17	5.94	5.86	5.66	5.40	5.17	5.03
120.85	5.97	5.96	6.07	6.19	6.05	5.96	5.87	5.71	5.54	5.28	5.12
129.36	6.33	6.21	6.17	6.17	6.09	6.00	5.88	5.72	5.57	5.21	5.02
138.55	6.03	6.06	6.07	6.11	6.09	6.00	5.92	5.64	5.37	5.29	5.08
148.45	6.05	6.17	6.12	6.17	6.00	5.86	5.73	5.51	5.36	5.15	4.98
159.13	6.34	6.20	6.19	6.10	6.18	5.93	5.77	5.66	5.37	5.27	4.97
170.65	6.30	6.50	6.27	6.27	6.19	6.14	5.82	5.64	5.48	5.21	5.02
183.07	6.17	6.10	6.16	6.11	6.17	6.01	6.02	5.76	5.51	5.21	4.97
196.47	5.91	6.13	5.97	5.94	6.00	5.89	5.82	5.51	5.39	5.15	4.94
210.91	6.12	6.21	6.07	6.12	6.16	5.99	5.89	5.68	5.50	5.21	5.06
226.50	5.84	5.90	5.82	5.94	5.93	5.82	5.77	5.42	5.36	5.11	4.80
243.31	5.90	5.82	5.87	5.90	5.78	5.81	5.67	5.41	5.32	5.06	4.84
261.43	6.01	6.03	6.09	6.05	5.98	5.92	5.75	5.40	5.31	5.17	4.84
280.98	5.75	5.92	5.96	5.82	5.77	5.70	5.70	5.50	5.16	5.06	4.83
302.07	5.86	5.89	5.91	5.96	5.90	5.75	5.73	5.55	5.37	5.11	4.90
324.81	5.85	5.85	5.98	5.89	5.80	5.81	5.71	5.47	5.33	5.02	4.78
349.33	5.44	5.68	5.70	5.86	5.79	5.69	5.57	5.48	5.26	5.07	4.77
375.79	5.79	5.75	5.86	5.78	5.73	5.67	5.54	5.40	5.19	5.10	4.76
404.31	5.66	5.62	5.71	5.64	5.67	5.50	5.51	5.29	5.05	4.84	4.70
435.08	5.93	5.94	5.89	5.73	5.83	5.67	5.58	5.33	5.20	4.84	4.72
468.27	5.69	5.90	5.86	5.77	5.78	5.75	5.59	5.38	5.14	5.05	4.83
504.06	5.70	5.73	5.72	5.78	5.66	5.75	5.44	5.29	5.19	4.93	4.66
542.66	5.82	5.87	6.03	5.94	5.86	5.91	5.72	5.55	5.26	4.98	4.73
584.29	5.64	5.76	5.73	5.60	5.56	5.56	5.52	5.27	5.07	4.98	4.71
629.18	5.64	5.48	5.56	5.73	5.56	5.58	5.44	5.31	5.10	4.90	4.61
677.61	5.74	5.73	5.77	5.76	5.65	5.67	5.50	5.35	5.09	4.93	4.70
729.83	5.44	5.50	5.63	5.54	5.66	5.55	5.48	5.27	5.13	4.91	4.73
786.16	5.36	5.45	5.45	5.51	5.59	5.57	5.42	5.32	5.14	4.98	4.72
846.90	5.38	5.56	5.55	5.64	5.65	5.63	5.44	5.32	5.09	4.78	4.55
912.42	5.46	5.53	5.55	5.55	5.66	5.52	5.49	5.25	5.06	4.93	4.57
983.08	5.49	5.43	5.40	5.50	5.55	5.54	5.35	5.25	4.93	4.84	4.62
1059.29	5.41	5.39	5.38	5.58	5.41	5.49	5.38	5.22	4.97	4.75	4.62
1141.48	5.35	5.48	5.58	5.46	5.66	5.51	5.40	5.23	5.10	4.73	4.63
1230.13	5.36	5.37	5.45	5.45	5.42	5.35	5.28	5.15	4.87	4.71	4.53
1325.73	5.43	5.61	5.53	5.49	5.57	5.48	5.24	5.20	4.97	4.65	4.43
1428.84	5.54	5.63	5.50	5.49	5.46	5.40	5.31	5.23	5.01	4.84	4.54
1540.05	5.62	5.68	5.66	5.65	5.62	5.52	5.38	5.34	5.05	4.88	4.58
1659.99	5.39	5.36	5.40	5.43	5.43	5.53	5.36	5.18	4.86	4.81	4.51
1789.34	5.53	5.55	5.54	5.49	5.52	5.41	5.31	5.14	4.95	4.67	4.49
1928.85	5.27	5.48	5.46	5.43	5.47	5.37	5.33	5.15	4.95	4.65	4.56
2079.31	5.35	5.32	5.40	5.43	5.42	5.44	5.28	5.09	4.91	4.70	4.56
2241.59	5.31	5.44	5.43	5.40	5.50	5.45	5.37	5.17	4.97	4.75	4.60
2416.60	5.26	5.43	5.27	5.41	5.34	5.30	5.15	4.88	4.84	4.63	4.48
2605.36	5.19	5.28	5.27	5.33	5.25	5.24	5.15	4.87	4.84	4.57	4.41
2808.94	5.12	5.40	5.42	5.37	5.37	5.31	5.17	4.98	4.73	4.48	4.32

3028.50	5.07	5.11	5.08	5.17	5.24	5.15	5.06	4.90	4.66	4.48	4.26
3265.30	5.03	5.08	5.26	5.08	5.19	5.15	5.20	4.90	4.76	4.52	4.42
3520.69	5.16	5.17	5.20	5.27	5.15	5.27	5.02	4.92	4.72	4.48	4.30
3796.13	5.00	5.30	5.26	5.18	5.26	5.11	5.08	4.89	4.76	4.58	4.43

Wavelength (nm) / Time (ps)	428.08	429.50	430.92	432.35	433.77	435.20	436.62	438.05	439.47	440.89	442.32
-3.78	-0.06	-0.05	0.01	-0.02	0.06	0.01	0.01	-0.08	0.05	0.06	0.07
-3.28	-0.07	0.07	-0.04	0.00	-0.09	-0.11	-0.03	-0.03	0.08	0.06	0.01
-2.78	0.08	0.28	0.08	0.03	0.11	0.07	0.11	0.13	0.09	-0.07	-0.07
-2.28	0.01	-0.07	-0.08	0.01	-0.01	0.05	-0.02	-0.01	-0.10	-0.03	0.02
-1.78	0.01	-0.05	0.10	0.06	0.05	0.04	0.08	0.04	0.06	0.11	-0.08
-1.28	-0.04	-0.12	-0.02	0.04	-0.07	0.02	-0.04	0.00	-0.06	-0.09	0.01
-0.78	0.00	-0.08	-0.03	-0.12	-0.04	-0.01	-0.08	-0.04	-0.10	-0.10	-0.01
-0.28	0.07	0.02	-0.01	-0.01	-0.02	-0.07	-0.03	0.00	-0.02	0.06	0.06
-0.18	-0.05	-0.06	0.05	0.09	0.01	-0.05	0.00	0.02	0.02	0.06	0.10
-0.08	-0.02	-0.01	-0.15	-0.02	0.13	0.02	-0.02	0.01	0.06	-0.04	0.00
0.02	-0.06	-0.01	0.07	0.07	-0.09	0.04	0.06	-0.11	0.04	0.08	0.05
0.12	-0.06	-0.08	-0.03	-0.03	0.02	0.02	0.04	-0.04	0.06	0.11	0.07
0.22	0.00	-0.09	0.06	-0.04	-0.06	-0.05	0.04	-0.08	-0.07	-0.17	-0.09
0.27	0.00	-0.05	0.01	-0.02	0.12	-0.09	-0.09	0.08	-0.07	0.05	0.14
0.32	0.02	0.00	0.05	0.01	0.14	0.04	0.11	0.07	0.17	0.08	0.16
0.37	0.03	0.03	-0.07	0.08	0.12	0.07	0.01	0.00	0.03	0.00	-0.02
0.42	0.08	0.11	0.02	0.11	0.03	-0.04	0.12	0.07	0.10	0.07	0.05
0.47	0.09	0.04	0.01	0.07	-0.08	0.02	-0.09	-0.09	-0.03	-0.06	0.03
0.52	-0.09	-0.14	-0.01	-0.09	-0.03	-0.12	-0.09	0.08	-0.15	-0.02	-0.11
0.57	-0.09	-0.03	0.00	0.12	0.01	0.17	0.09	0.03	0.15	0.09	0.15
0.62	-0.05	0.03	-0.03	0.04	0.12	0.03	-0.02	0.01	0.02	0.06	0.00
0.67	0.06	-0.15	0.05	0.03	0.03	0.12	-0.04	0.03	-0.02	-0.05	-0.04
0.72	-0.06	0.04	0.07	0.03	0.10	0.02	0.16	0.02	0.04	0.02	0.16
0.77	-0.05	-0.04	0.02	0.09	0.05	-0.06	-0.10	0.01	-0.16	0.00	0.03
0.82	0.09	-0.05	0.08	-0.09	0.08	-0.01	-0.04	-0.03	-0.05	0.01	0.06
0.87	-0.10	-0.10	0.02	0.11	0.00	0.00	0.01	0.06	-0.04	0.03	0.06
0.92	-0.52	-0.66	-0.31	-0.05	-0.04	-0.12	-0.06	-0.13	0.00	-0.22	-0.14
0.97	-35.54	-25.68	-17.61	-10.49	-4.24	-0.34	1.03	0.76	0.25	-0.06	-0.16
1.02	95.86	63.64	30.41	-0.87	-25.81	-42.37	-49.72	-49.43	-44.06	-36.21	-27.83
1.07	33.04	70.77	107.85	140.15	163.99	175.37	173.65	159.22	135.12	105.72	74.30
1.12	13.58	5.49	-12.17	-36.06	-59.23	-74.43	-76.83	-65.56	-42.81	-12.34	22.14
1.17	9.56	9.82	11.27	13.09	14.34	13.81	11.74	10.21	10.65	11.99	11.31
1.22	11.63	11.07	10.30	9.58	9.41	9.37	9.30	8.31	6.69	5.02	4.25
1.27	10.66	10.77	10.43	9.39	8.03	6.74	6.24	6.90	7.50	7.87	7.63
1.32	8.80	8.12	7.99	8.31	8.76	8.39	7.49	6.31	5.69	5.67	6.22
1.37	9.28	9.02	8.32	7.48	7.00	6.74	7.20	7.55	7.32	6.34	5.05
1.42	8.21	8.14	8.29	8.15	7.65	6.68	5.82	5.37	5.44	5.52	5.29
1.47	8.76	8.02	7.42	7.24	7.45	7.49	7.08	6.01	4.71	3.95	3.78
1.52	8.61	8.39	7.61	6.72	6.15	5.75	5.96	6.19	6.24	5.52	4.55
1.57	7.23	7.20	7.34	7.38	7.13	6.36	5.27	4.80	4.69	4.97	5.14
1.62	7.88	7.19	6.67	6.67	6.68	6.97	6.69	5.91	4.53	3.53	3.01

1.67	8.07	7.95	7.50	6.80	5.90	5.33	5.21	5.21	5.33	4.82	4.00
1.72	7.29	6.86	6.57	6.67	6.53	6.07	5.41	4.60	4.08	3.89	4.27
1.77	7.78	7.26	6.65	5.96	5.72	5.52	5.83	5.75	5.21	4.31	3.32
1.82	7.07	7.05	7.15	6.79	6.06	5.32	4.53	4.32	4.42	4.48	4.03
1.87	7.25	6.69	6.41	6.39	6.39	5.95	5.45	4.59	3.94	3.50	3.53
1.92	7.64	7.24	6.72	6.02	5.51	5.10	5.10	5.20	5.10	4.41	3.69
1.97	6.78	6.63	6.48	6.63	6.39	5.64	4.93	4.39	4.16	4.11	4.04
2.02	7.35	6.98	6.51	6.11	5.89	5.58	5.46	5.00	4.33	3.61	3.01
2.07	7.18	7.07	6.52	5.94	5.46	4.94	4.78	4.91	4.81	4.34	3.59
2.12	6.60	6.25	6.09	5.99	5.82	5.35	4.79	4.05	3.62	3.47	3.44
2.17	6.84	6.26	5.97	5.58	5.40	5.21	5.10	4.70	4.09	3.31	2.74
2.22	6.84	6.70	6.56	6.20	5.78	4.98	4.51	4.23	4.17	4.10	3.76
2.27	6.66	6.30	5.83	5.83	5.64	5.43	5.15	4.32	3.81	3.39	3.23
2.32	6.78	6.58	6.10	5.56	5.15	4.89	4.81	4.77	4.42	3.82	3.11
2.37	6.44	6.33	6.19	6.03	5.74	5.10	4.47	4.05	3.71	3.72	3.57
2.42	6.66	6.14	5.77	5.68	5.43	5.20	4.89	4.19	3.68	3.15	2.79
2.47	6.72	6.45	5.97	5.62	5.19	4.77	4.60	4.60	4.23	3.76	3.25
2.52	6.45	6.25	5.94	5.89	5.63	5.17	4.72	4.14	3.69	3.46	3.35
2.57	6.72	6.31	5.95	5.59	5.38	5.16	4.91	4.49	4.03	3.38	2.92
2.62	6.53	6.43	6.17	5.76	5.24	4.72	4.39	4.17	4.02	3.64	3.33
2.67	6.40	6.05	5.85	5.54	5.42	5.09	4.61	3.96	3.64	3.29	3.22
2.72	6.71	6.25	5.95	5.43	5.17	4.99	4.73	4.58	4.10	3.68	2.98
2.77	6.45	6.34	6.06	5.61	5.33	4.84	4.40	4.09	3.70	3.52	3.32
2.82	6.32	6.10	5.70	5.47	5.25	4.95	4.54	4.06	3.64	3.25	2.98
2.87	6.36	6.22	5.88	5.40	4.96	4.73	4.44	4.18	3.90	3.60	3.04
2.92	6.32	5.99	5.85	5.69	5.50	5.03	4.47	3.96	3.66	3.35	3.19
2.97	6.59	6.31	5.77	5.38	5.19	4.95	4.69	4.27	3.82	3.20	2.99
3.02	6.42	6.20	5.97	5.58	5.23	4.77	4.44	4.19	3.94	3.67	3.19
3.07	6.36	5.97	5.78	5.51	5.24	4.83	4.40	4.00	3.67	3.36	2.98
3.12	6.37	5.95	5.56	5.24	4.92	4.77	4.41	4.23	3.74	3.20	2.76
3.17	6.51	6.21	5.90	5.65	5.35	4.82	4.28	3.90	3.70	3.43	3.12
3.22	6.49	6.16	5.80	5.53	5.31	4.99	4.66	4.30	3.77	3.34	3.02
3.42	6.36	6.15	5.81	5.40	5.05	4.66	4.26	4.04	3.78	3.46	3.02
3.62	6.55	6.03	5.75	5.54	5.21	4.92	4.38	4.03	3.57	3.24	3.01
3.82	6.37	5.96	5.76	5.45	5.11	4.67	4.50	4.06	3.92	3.47	3.01
4.02	6.28	5.92	5.67	5.47	5.20	4.82	4.45	4.05	3.61	3.21	2.95
4.22	6.24	5.97	5.70	5.34	5.06	4.71	4.32	4.01	3.74	3.16	2.90
4.42	6.23	6.03	5.79	5.40	5.15	4.79	4.38	3.95	3.68	3.18	3.01
4.62	6.32	5.83	5.54	5.24	4.84	4.49	4.26	3.97	3.55	3.05	2.81
4.82	6.12	5.81	5.57	5.31	4.98	4.60	4.20	3.91	3.59	3.23	2.93
5.02	6.13	5.87	5.55	5.36	4.89	4.49	4.26	3.89	3.49	3.06	2.85
5.22	6.07	5.90	5.53	5.23	4.95	4.65	4.35	3.92	3.67	3.30	2.92
5.42	5.90	5.72	5.45	5.25	4.97	4.47	4.21	3.75	3.51	3.11	2.78
5.62	6.03	5.67	5.45	5.13	4.79	4.47	4.16	3.71	3.36	3.10	2.72
5.82	5.89	5.58	5.32	5.03	4.68	4.40	4.00	3.74	3.38	3.02	2.64
6.02	5.95	5.60	5.38	5.10	4.88	4.46	4.06	3.68	3.46	3.07	2.86
6.22	5.87	5.70	5.33	5.14	4.86	4.39	4.12	3.69	3.43	2.95	2.73
6.42	5.87	5.62	5.21	4.97	4.75	4.25	4.00	3.60	3.30	2.98	2.68
6.62	5.90	5.78	5.52	5.09	4.82	4.48	4.26	3.92	3.52	3.09	2.69
6.82	5.82	5.54	5.26	5.01	4.78	4.23	4.10	3.67	3.31	2.80	2.63

7.02	6.05	5.77	5.37	5.13	4.91	4.45	4.12	3.68	3.35	3.01	2.64
7.22	5.93	5.56	5.36	5.07	4.78	4.39	4.05	3.70	3.38	3.06	2.81
7.42	5.89	5.61	5.29	4.95	4.74	4.33	4.00	3.75	3.36	2.96	2.67
7.62	5.89	5.71	5.37	4.97	4.71	4.41	4.10	3.86	3.48	3.10	2.79
7.82	5.71	5.56	5.21	4.96	4.61	4.24	3.96	3.56	3.25	2.97	2.65
8.02	5.83	5.50	5.31	4.88	4.61	4.23	3.95	3.59	3.26	2.93	2.50
8.22	5.64	5.43	5.23	4.96	4.58	4.17	4.02	3.59	3.27	2.87	2.67
8.42	5.74	5.43	5.19	5.04	4.58	4.21	3.82	3.61	3.22	2.74	2.45
8.62	5.61	5.39	5.16	4.96	4.50	4.25	3.93	3.72	3.46	3.00	2.61
8.82	5.79	5.46	5.23	4.99	4.59	4.24	4.05	3.70	3.35	3.02	2.63
9.02	5.76	5.44	5.18	4.79	4.57	4.20	3.83	3.56	3.28	2.90	2.61
9.22	5.65	5.55	5.17	4.76	4.54	4.20	4.01	3.54	3.17	2.86	2.59
9.42	5.76	5.49	5.05	4.78	4.49	4.19	3.90	3.43	3.11	2.76	2.50
9.62	5.70	5.35	5.14	4.87	4.63	4.23	3.96	3.62	3.13	2.87	2.51
9.82	5.59	5.54	5.23	4.85	4.62	4.25	4.00	3.63	3.31	2.87	2.60
10.02	5.69	5.24	5.06	4.83	4.50	4.08	3.84	3.45	3.17	2.69	2.45
10.22	5.76	5.51	5.12	4.86	4.57	4.17	3.91	3.57	3.24	2.81	2.45
10.42	5.52	5.34	5.11	4.83	4.49	4.10	3.83	3.61	3.21	2.90	2.47
10.62	5.72	5.51	5.21	4.87	4.65	4.23	3.95	3.58	3.27	3.02	2.62
10.82	5.81	5.54	5.05	4.95	4.53	4.29	3.90	3.58	3.25	2.88	2.59
11.02	5.59	5.33	5.11	4.91	4.48	4.24	3.76	3.53	3.18	2.80	2.55
11.22	5.50	5.22	4.89	4.61	4.37	4.00	3.65	3.48	2.97	2.70	2.41
11.42	5.51	5.15	4.84	4.63	4.44	4.02	3.69	3.45	3.00	2.70	2.40
11.62	5.46	5.29	4.98	4.67	4.46	3.98	3.82	3.41	3.11	2.78	2.46
11.82	5.49	5.24	5.01	4.65	4.45	3.98	3.69	3.44	3.06	2.80	2.40
12.02	5.52	5.23	5.06	4.71	4.45	4.21	3.87	3.52	3.07	2.80	2.50
12.22	5.64	5.39	5.09	4.87	4.59	4.21	4.01	3.56	3.32	2.88	2.57
12.42	5.52	5.23	4.99	4.69	4.52	4.04	3.75	3.37	3.05	2.81	2.45
12.62	5.62	5.28	4.98	4.64	4.40	4.03	3.72	3.32	3.13	2.84	2.59
12.82	5.42	5.21	4.92	4.65	4.25	3.97	3.67	3.28	2.89	2.63	2.36
13.02	5.60	5.34	5.07	4.64	4.52	4.09	3.90	3.46	3.19	2.76	2.54
13.22	5.45	5.13	4.96	4.66	4.43	3.97	3.55	3.48	3.01	2.78	2.39
13.42	5.47	5.28	4.96	4.56	4.37	4.00	3.83	3.47	3.10	2.82	2.47
13.50	5.41	5.11	4.78	4.50	4.30	3.89	3.58	3.25	3.01	2.65	2.40
13.59	5.60	5.32	5.05	4.63	4.49	4.13	3.83	3.53	3.15	2.77	2.53
13.68	5.38	5.18	4.83	4.55	4.30	3.86	3.65	3.34	2.95	2.59	2.44
13.78	5.35	5.19	4.84	4.52	4.25	3.96	3.56	3.31	3.03	2.58	2.29
13.88	5.43	5.15	4.89	4.62	4.34	4.16	3.74	3.42	3.09	2.81	2.45
14.00	5.46	5.21	4.95	4.63	4.43	3.98	3.73	3.44	3.09	2.83	2.51
14.12	5.39	5.18	5.00	4.65	4.26	3.91	3.64	3.21	3.06	2.64	2.35
14.25	5.26	4.94	4.79	4.44	4.28	3.95	3.52	3.34	2.95	2.66	2.22
14.40	5.53	5.35	4.99	4.63	4.38	3.96	3.82	3.38	3.00	2.82	2.42
14.55	5.35	5.07	4.81	4.60	4.32	3.95	3.67	3.30	2.98	2.62	2.33
14.72	5.29	4.93	4.78	4.42	4.13	3.77	3.63	3.31	2.97	2.69	2.36
14.90	5.28	5.13	4.78	4.47	4.28	3.82	3.69	3.23	3.05	2.67	2.38
15.09	5.30	5.04	4.74	4.47	4.20	3.91	3.53	3.23	2.91	2.58	2.27
15.30	5.28	5.13	4.87	4.47	4.28	3.95	3.68	3.31	2.94	2.75	2.41
15.53	5.48	5.14	5.00	4.63	4.34	4.01	3.70	3.30	2.96	2.63	2.35
15.77	5.28	5.11	4.70	4.49	4.25	3.84	3.54	3.26	2.95	2.68	2.26
16.04	5.62	5.25	4.94	4.63	4.46	4.06	3.76	3.47	3.19	2.81	2.46

16.32	5.33	4.95	4.79	4.48	4.23	3.97	3.59	3.25	2.98	2.59	2.23
16.63	5.54	5.25	4.85	4.69	4.25	3.91	3.71	3.39	3.15	2.75	2.45
16.96	5.50	5.11	4.94	4.51	4.17	3.81	3.62	3.30	2.96	2.59	2.23
17.31	5.35	5.03	4.85	4.52	4.35	3.96	3.73	3.31	2.96	2.68	2.41
17.70	5.51	5.16	4.94	4.63	4.35	3.96	3.74	3.39	3.07	2.73	2.36
18.11	5.29	4.93	4.69	4.41	4.14	3.73	3.50	3.20	2.86	2.46	2.30
18.56	5.38	5.11	4.71	4.48	4.40	3.91	3.57	3.25	2.85	2.66	2.33
19.04	5.29	4.99	4.78	4.41	4.07	3.80	3.44	3.27	2.82	2.55	2.21
19.56	5.45	5.07	4.90	4.68	4.34	4.01	3.69	3.37	3.01	2.74	2.43
20.12	5.34	5.06	4.85	4.52	4.17	3.92	3.54	3.22	2.90	2.49	2.35
20.72	5.36	5.10	4.91	4.58	4.38	3.97	3.76	3.37	3.08	2.73	2.48
21.37	5.42	5.01	4.66	4.47	4.27	3.87	3.59	3.26	2.91	2.59	2.17
22.08	5.54	5.18	4.89	4.54	4.26	3.94	3.60	3.17	2.98	2.46	2.32
22.84	5.50	5.10	4.84	4.65	4.27	3.97	3.64	3.31	3.01	2.63	2.33
23.65	5.24	4.87	4.57	4.30	4.04	3.88	3.49	3.15	2.88	2.45	2.23
24.54	5.26	4.94	4.61	4.32	4.17	3.75	3.51	3.13	2.77	2.51	2.23
25.49	5.32	5.00	4.87	4.45	4.26	3.86	3.66	3.22	3.03	2.56	2.25
26.51	5.08	4.76	4.47	4.18	3.91	3.60	3.29	2.91	2.69	2.27	1.95
27.62	5.25	4.94	4.71	4.45	4.26	3.73	3.47	3.12	2.84	2.45	2.17
28.81	5.13	4.86	4.70	4.40	4.11	3.71	3.51	3.12	2.82	2.52	2.28
30.10	5.03	4.75	4.63	4.15	3.93	3.65	3.28	3.00	2.80	2.44	2.01
31.49	5.25	4.98	4.75	4.41	4.14	3.73	3.55	3.17	2.98	2.57	2.27
32.98	5.23	4.88	4.66	4.35	4.06	3.71	3.41	3.20	2.89	2.55	2.24
34.60	5.13	4.86	4.58	4.32	4.06	3.72	3.47	3.07	2.73	2.39	2.05
36.34	5.05	4.85	4.47	4.24	4.01	3.62	3.33	2.97	2.69	2.38	2.08
38.21	5.18	4.92	4.60	4.38	4.18	3.80	3.45	3.20	2.81	2.40	2.26
40.24	5.09	4.87	4.58	4.34	3.97	3.61	3.29	3.06	2.77	2.38	2.05
42.42	4.95	4.66	4.41	4.19	3.89	3.61	3.25	2.97	2.56	2.27	1.96
44.78	4.96	4.59	4.45	4.08	3.88	3.41	3.26	2.92	2.72	2.30	2.05
47.32	4.81	4.49	4.26	4.06	3.77	3.34	3.19	2.89	2.58	2.27	1.88
50.06	4.99	4.75	4.50	4.23	3.96	3.62	3.38	3.22	2.82	2.47	2.14
53.01	4.97	4.72	4.37	4.09	3.83	3.42	3.15	2.91	2.55	2.18	1.96
56.20	5.00	4.73	4.46	4.12	3.92	3.57	3.26	2.98	2.56	2.24	2.03
59.64	5.01	4.70	4.51	4.10	3.79	3.51	3.22	2.90	2.64	2.31	2.00
63.34	4.86	4.48	4.26	3.98	3.84	3.36	3.11	2.76	2.56	2.18	1.86
67.34	4.91	4.59	4.28	3.99	3.88	3.50	3.18	2.90	2.63	2.34	1.97
71.65	4.89	4.55	4.24	3.99	3.66	3.41	3.08	2.79	2.47	2.12	1.84
76.30	4.85	4.62	4.30	4.03	3.84	3.46	3.11	2.84	2.51	2.21	2.00
81.32	4.99	4.61	4.34	4.14	3.79	3.54	3.20	2.92	2.63	2.29	1.96
86.73	4.80	4.52	4.31	4.02	3.75	3.51	3.18	2.81	2.50	2.32	2.04
92.56	4.70	4.55	4.34	4.14	3.78	3.45	3.15	2.93	2.59	2.28	1.98
98.85	4.84	4.49	4.30	4.07	3.74	3.35	3.13	2.75	2.56	2.13	1.90
105.64	4.74	4.47	4.30	4.04	3.70	3.38	3.21	2.87	2.56	2.21	1.98
112.96	4.68	4.51	4.21	4.00	3.80	3.34	3.17	2.88	2.57	2.26	1.90
120.85	4.82	4.46	4.28	4.01	3.77	3.25	3.05	2.75	2.48	2.22	1.91
129.36	4.79	4.67	4.34	4.01	3.78	3.47	3.14	2.93	2.57	2.29	2.10
138.55	4.88	4.75	4.38	4.11	3.94	3.54	3.30	2.94	2.56	2.26	2.10
148.45	4.72	4.53	4.33	4.01	3.72	3.44	3.00	2.77	2.49	2.15	1.96
159.13	4.73	4.45	4.33	3.99	3.71	3.42	3.11	2.79	2.53	2.14	1.90
170.65	4.70	4.44	4.21	3.85	3.62	3.20	2.87	2.72	2.39	2.09	1.82

183.07	4.79	4.52	4.25	4.05	3.79	3.41	3.09	2.86	2.42	2.26	1.92
196.47	4.71	4.47	4.02	3.79	3.58	3.31	3.03	2.84	2.42	2.14	1.88
210.91	4.67	4.44	4.25	3.89	3.65	3.26	3.05	2.65	2.41	2.21	1.90
226.50	4.62	4.39	4.21	3.89	3.76	3.35	3.00	2.73	2.47	2.27	1.97
243.31	4.64	4.31	4.06	3.88	3.56	3.34	3.06	2.73	2.43	2.14	1.87
261.43	4.70	4.42	4.19	3.99	3.69	3.37	3.06	2.78	2.48	2.08	1.88
280.98	4.62	4.50	4.12	3.84	3.60	3.26	2.98	2.89	2.48	2.24	2.00
302.07	4.72	4.46	4.27	3.92	3.76	3.35	3.08	2.84	2.48	2.26	2.12
324.81	4.62	4.37	4.17	3.84	3.52	3.22	2.95	2.70	2.36	1.99	1.70
349.33	4.55	4.36	4.11	3.78	3.57	3.30	3.07	2.73	2.47	2.12	2.01
375.79	4.64	4.29	4.18	3.87	3.61	3.24	3.00	2.73	2.47	2.11	1.84
404.31	4.41	4.26	3.90	3.81	3.48	3.13	3.00	2.72	2.40	2.02	1.86
435.08	4.52	4.23	3.99	3.74	3.45	3.16	2.91	2.63	2.31	2.08	1.84
468.27	4.60	4.20	4.13	3.80	3.51	3.27	2.99	2.71	2.39	2.09	1.82
504.06	4.52	4.26	4.04	3.81	3.50	3.12	2.96	2.62	2.43	2.01	1.86
542.66	4.51	4.28	4.07	3.79	3.47	3.20	2.97	2.61	2.42	2.14	1.72
584.29	4.48	4.26	4.08	3.78	3.52	3.20	2.94	2.72	2.37	2.23	1.79
629.18	4.43	4.30	4.05	3.75	3.57	3.16	2.87	2.66	2.36	2.17	1.84
677.61	4.39	4.18	3.97	3.67	3.44	3.16	2.92	2.58	2.44	2.02	1.81
729.83	4.54	4.14	3.95	3.67	3.54	3.22	2.95	2.63	2.44	2.10	1.83
786.16	4.57	4.40	4.10	3.74	3.61	3.29	3.03	2.77	2.53	2.17	1.96
846.90	4.46	4.16	3.98	3.67	3.44	3.12	2.77	2.59	2.15	2.08	1.67
912.42	4.43	4.17	3.90	3.65	3.38	3.03	2.81	2.43	2.29	1.99	1.75
983.08	4.31	4.21	3.92	3.74	3.53	3.13	2.98	2.64	2.39	1.97	1.78
1059.29	4.46	4.17	3.97	3.66	3.49	3.19	2.98	2.70	2.54	2.08	1.78
1141.48	4.25	4.17	3.91	3.65	3.22	3.01	2.80	2.52	2.19	1.88	1.62
1230.13	4.28	4.11	3.81	3.61	3.31	3.09	2.77	2.59	2.29	2.03	1.82
1325.73	4.32	4.10	3.91	3.65	3.44	3.05	2.83	2.56	2.24	1.96	1.79
1428.84	4.26	4.06	3.95	3.55	3.33	3.04	2.75	2.42	2.28	1.96	1.68
1540.05	4.35	4.09	3.81	3.71	3.39	3.10	2.83	2.51	2.28	1.95	1.68
1659.99	4.38	4.10	3.81	3.54	3.28	2.96	2.77	2.39	2.24	1.92	1.62
1789.34	4.24	3.97	3.72	3.45	3.22	2.90	2.58	2.38	2.03	1.78	1.52
1928.85	4.24	3.97	3.75	3.50	3.33	2.86	2.68	2.55	2.16	1.87	1.55
2079.31	4.26	4.16	3.91	3.48	3.37	2.99	2.68	2.31	2.17	1.91	1.68
2241.59	4.22	4.08	3.82	3.57	3.32	2.98	2.71	2.45	2.11	1.86	1.54
2416.60	4.23	3.91	3.76	3.42	3.27	2.91	2.68	2.45	2.05	1.85	1.60
2605.36	4.23	4.00	3.73	3.34	3.34	2.99	2.59	2.34	2.06	1.76	1.60
2808.94	4.05	3.90	3.66	3.32	3.08	2.74	2.49	2.22	1.97	1.64	1.37
3028.50	4.04	3.82	3.62	3.41	3.25	2.77	2.48	2.25	1.94	1.68	1.50
3265.30	4.16	3.88	3.70	3.32	3.23	2.83	2.57	2.27	2.00	1.75	1.48
3520.69	4.05	3.81	3.64	3.40	3.12	2.97	2.62	2.48	2.16	1.96	1.65
3796.13	4.21	3.99	3.77	3.56	3.32	2.98	2.75	2.44	2.25	1.83	1.65

Wavelength (nm) / Time (ps)	443.74	445.17	446.59	448.01	449.44	450.86	452.29	453.71	455.13	456.56	457.98
-3.78	0.01	0.12	0.08	0.11	0.04	0.15	0.07	0.06	0.07	0.09	0.00
-3.28	0.04	-0.02	0.06	0.14	0.15	0.02	0.14	0.07	0.01	0.03	0.04
-2.78	-0.02	-0.01	-0.04	-0.13	-0.09	-0.06	0.02	-0.01	0.00	-0.07	-0.12

-2.28	0.01	0.02	0.00	0.00	0.03	-0.08	-0.02	-0.04	-0.05	-0.07	0.02
-1.78	0.10	-0.04	0.04	0.10	0.06	0.09	0.02	0.01	0.05	0.01	0.16
-1.28	-0.12	-0.09	-0.07	-0.14	-0.13	-0.02	-0.12	0.03	-0.08	-0.05	-0.03
-0.78	-0.06	-0.12	-0.08	-0.06	-0.14	-0.12	-0.13	-0.03	-0.02	0.02	-0.10
-0.28	0.05	0.13	0.00	-0.02	0.08	0.01	0.01	-0.08	0.02	0.04	0.03
-0.18	0.04	0.02	0.03	0.02	0.03	0.05	0.01	-0.05	-0.08	-0.01	0.00
-0.08	0.00	-0.06	0.03	0.13	0.05	0.03	0.01	0.02	-0.05	0.11	0.12
0.02	-0.08	0.01	-0.06	0.08	-0.06	-0.02	-0.08	-0.03	-0.02	0.12	0.01
0.12	0.13	0.02	0.05	0.09	0.07	0.12	0.04	0.01	0.00	0.08	0.00
0.22	0.00	-0.19	-0.09	-0.12	-0.14	-0.13	-0.19	-0.06	-0.06	-0.12	-0.23
0.27	0.13	-0.04	-0.06	0.02	0.04	0.08	0.01	0.01	0.01	0.04	0.02
0.32	0.12	0.19	0.15	0.02	0.05	0.04	0.10	0.09	0.12	0.07	0.11
0.37	0.04	-0.04	-0.07	0.02	0.04	-0.06	-0.13	-0.04	0.01	-0.03	-0.01
0.42	0.03	0.03	0.04	-0.01	0.05	0.03	0.04	0.06	0.08	0.06	-0.01
0.47	-0.04	-0.01	-0.18	0.01	-0.01	-0.06	-0.08	-0.01	-0.06	-0.09	-0.09
0.52	0.00	-0.01	0.03	-0.09	-0.10	-0.05	-0.06	-0.04	-0.03	0.01	-0.12
0.57	0.16	0.00	0.07	0.14	0.19	0.19	0.02	0.10	0.13	0.07	0.01
0.62	0.06	0.08	0.04	-0.05	-0.14	0.03	0.00	0.09	0.02	0.04	-0.10
0.67	-0.08	-0.02	-0.12	-0.01	0.06	0.00	-0.06	0.02	-0.06	0.04	-0.03
0.72	0.11	0.09	0.01	-0.03	0.00	-0.02	0.00	0.01	0.08	0.12	-0.01
0.77	-0.08	-0.05	-0.09	-0.05	-0.05	-0.05	-0.11	0.02	-0.06	-0.07	-0.11
0.82	0.10	0.00	0.02	-0.08	-0.06	0.00	-0.06	0.06	-0.03	-0.01	-0.07
0.87	0.03	-0.07	-0.03	0.08	0.09	0.06	0.04	-0.02	-0.07	0.05	0.06
0.92	-0.05	-0.27	-0.01	-0.07	-0.20	0.05	-0.08	0.04	0.01	-0.08	-0.02
0.97	-0.12	0.02	0.04	0.06	0.11	0.20	0.03	0.09	0.17	0.19	-0.02
1.02	-19.30	-11.83	-6.08	-2.49	-0.92	-0.47	-0.23	0.16	0.30	-0.02	-0.36
1.07	43.89	16.91	-5.66	-23.07	-34.97	-41.66	-44.13	-43.23	-39.67	-33.49	-25.01
1.12	57.61	91.30	121.08	145.37	161.31	167.82	164.66	152.65	132.71	108.08	81.33
1.17	4.64	-8.74	-26.97	-45.72	-59.84	-66.34	-63.87	-52.82	-35.77	-14.64	8.30
1.22	4.72	6.42	8.78	10.63	10.75	8.58	5.21	3.15	3.13	4.70	5.44
1.27	6.41	5.29	4.42	4.12	4.11	4.01	3.18	1.82	-0.01	-1.68	-2.63
1.32	6.57	6.03	4.74	2.84	1.20	0.05	0.03	0.77	1.61	1.99	1.52
1.37	3.74	3.08	3.09	3.38	3.72	3.35	2.38	1.14	0.07	-0.56	-0.69
1.42	4.78	3.75	2.64	2.08	1.94	2.25	2.39	2.33	1.38	0.13	-1.30
1.47	4.28	4.59	4.45	3.77	2.50	1.09	0.01	-0.40	-0.11	0.24	0.15
1.52	3.45	2.87	2.75	2.82	2.97	2.55	1.52	0.04	-1.20	-1.89	-2.09
1.57	4.77	3.66	2.26	1.22	0.72	0.79	1.24	1.52	1.33	0.27	-1.06
1.62	3.21	3.55	3.73	3.25	2.19	0.99	-0.13	-0.56	-0.43	-0.10	-0.07
1.67	3.10	2.50	2.12	2.23	2.38	2.14	1.37	0.29	-0.92	-1.96	-2.49
1.72	4.30	4.01	3.12	2.13	0.92	0.31	-0.04	0.18	0.30	0.11	-0.68
1.77	2.59	2.27	2.38	2.45	2.08	1.50	0.36	-0.58	-1.31	-1.53	-1.28
1.82	3.32	2.42	1.51	1.13	1.09	1.25	1.24	0.90	0.01	-1.16	-2.21
1.87	3.73	3.57	3.03	2.05	1.05	0.29	-0.26	-0.27	-0.26	-0.37	-0.88
1.92	2.88	2.19	2.03	2.07	2.15	1.83	0.95	-0.09	-1.10	-1.70	-2.05
1.97	3.70	2.86	1.91	1.14	0.64	0.55	0.63	0.69	0.32	-0.35	-1.35
2.02	2.67	2.57	2.49	2.30	1.77	1.08	0.10	-0.50	-0.84	-1.14	-1.24
2.07	2.69	2.12	1.81	1.83	1.70	1.47	0.83	-0.18	-1.18	-1.87	-2.17
2.12	3.25	2.94	2.20	1.33	0.60	0.12	-0.11	-0.16	-0.28	-0.49	-1.19
2.17	2.36	2.19	2.26	1.96	1.38	0.69	-0.14	-0.77	-1.21	-1.35	-1.50
2.22	3.09	2.41	1.76	1.28	1.16	1.14	0.87	0.47	-0.25	-1.17	-2.16

2.27	3.14	3.01	2.47	1.82	1.00	0.10	-0.31	-0.42	-0.59	-0.71	-1.22
2.32	2.40	2.06	1.89	1.72	1.64	1.12	0.54	-0.28	-1.09	-1.54	-1.82
2.37	3.13	2.53	1.75	1.13	0.77	0.51	0.29	0.24	-0.11	-0.95	-1.73
2.42	2.77	2.57	2.23	1.75	1.02	0.26	-0.19	-0.64	-0.80	-1.00	-1.28
2.47	2.66	2.06	1.77	1.71	1.33	0.96	0.41	-0.26	-0.91	-1.58	-2.03
2.52	3.02	2.60	2.03	1.23	0.70	0.38	0.04	-0.10	-0.29	-0.74	-1.45
2.57	2.50	2.41	2.21	1.85	1.36	0.71	-0.13	-0.53	-1.02	-1.23	-1.48
2.62	2.64	2.15	1.51	1.19	1.14	0.85	0.58	-0.11	-0.83	-1.44	-2.16
2.67	2.95	2.47	1.99	1.39	0.72	0.30	-0.09	-0.26	-0.45	-0.84	-1.43
2.72	2.41	2.18	1.89	1.75	1.35	0.92	0.16	-0.36	-1.00	-1.43	-1.57
2.77	2.83	2.33	1.75	1.30	0.93	0.67	0.41	0.06	-0.54	-1.14	-1.87
2.82	2.68	2.37	2.08	1.48	0.92	0.34	-0.30	-0.57	-0.90	-1.14	-1.53
2.87	2.61	2.09	1.74	1.43	1.12	0.68	0.28	-0.25	-0.71	-1.31	-1.86
2.92	2.86	2.46	1.90	1.26	0.69	0.30	0.08	-0.14	-0.43	-0.97	-1.67
2.97	2.51	2.32	2.03	1.76	1.18	0.71	0.07	-0.56	-0.96	-1.18	-1.47
3.02	2.69	2.22	1.69	1.32	1.02	0.77	0.39	-0.21	-0.81	-1.37	-1.97
3.07	2.65	2.37	1.82	1.38	0.82	0.33	-0.13	-0.34	-0.65	-1.05	-1.71
3.12	2.30	2.00	1.79	1.45	1.13	0.53	0.03	-0.70	-1.12	-1.38	-1.78
3.17	2.64	2.11	1.49	1.05	0.71	0.48	0.11	-0.25	-0.83	-1.33	-1.92
3.22	2.65	2.43	2.05	1.54	1.03	0.44	-0.05	-0.38	-0.74	-1.07	-1.48
3.42	2.44	1.94	1.69	1.37	0.98	0.59	0.17	-0.28	-0.90	-1.51	-2.10
3.62	2.69	2.30	1.81	1.26	0.80	0.42	-0.08	-0.45	-0.80	-1.07	-1.59
3.82	2.54	2.10	1.74	1.42	1.06	0.54	0.27	-0.19	-0.78	-1.26	-1.84
4.02	2.75	2.27	1.78	1.37	0.89	0.34	-0.06	-0.45	-0.79	-1.12	-1.81
4.22	2.49	2.12	1.59	1.25	0.94	0.59	0.07	-0.42	-0.96	-1.41	-1.81
4.42	2.55	2.16	1.74	1.35	0.83	0.49	0.07	-0.36	-0.75	-1.15	-1.68
4.62	2.35	1.99	1.59	1.19	0.86	0.39	-0.17	-0.55	-0.97	-1.52	-1.98
4.82	2.44	2.06	1.64	1.29	0.80	0.45	-0.15	-0.41	-0.84	-1.31	-1.79
5.02	2.42	1.92	1.61	1.20	0.80	0.33	-0.15	-0.53	-1.03	-1.51	-1.91
5.22	2.55	2.05	1.71	1.25	0.72	0.46	-0.02	-0.32	-0.74	-1.19	-1.73
5.42	2.36	1.98	1.57	1.17	0.76	0.29	-0.14	-0.51	-1.07	-1.43	-1.82
5.62	2.37	2.03	1.54	1.25	0.72	0.32	-0.10	-0.63	-0.93	-1.39	-1.89
5.82	2.17	1.84	1.44	1.10	0.61	0.26	-0.29	-0.59	-1.07	-1.53	-2.04
6.02	2.31	1.94	1.44	1.11	0.69	0.39	-0.05	-0.53	-0.89	-1.27	-1.80
6.22	2.24	1.84	1.43	1.13	0.70	0.31	-0.23	-0.54	-1.01	-1.41	-1.96
6.42	2.15	1.75	1.46	1.03	0.64	0.25	-0.18	-0.59	-1.04	-1.52	-1.98
6.62	2.34	2.04	1.58	1.29	0.76	0.45	-0.10	-0.51	-0.95	-1.37	-1.75
6.82	2.18	1.80	1.50	1.15	0.60	0.26	-0.20	-0.67	-1.03	-1.49	-1.93
7.02	2.24	1.86	1.51	1.10	0.70	0.39	-0.09	-0.51	-0.98	-1.48	-1.77
7.22	2.26	1.93	1.62	1.18	0.74	0.41	-0.07	-0.45	-1.04	-1.28	-1.80
7.42	2.32	1.96	1.56	1.19	0.66	0.28	-0.17	-0.54	-1.05	-1.30	-1.90
7.62	2.44	2.00	1.50	1.22	0.70	0.35	-0.11	-0.48	-0.90	-1.33	-1.91
7.82	2.17	1.84	1.53	1.01	0.68	0.23	-0.24	-0.63	-0.90	-1.48	-1.77
8.02	2.14	1.79	1.40	1.06	0.62	0.27	-0.25	-0.61	-1.00	-1.47	-1.87
8.22	2.27	1.80	1.49	1.01	0.63	0.23	-0.15	-0.47	-1.04	-1.44	-1.88
8.42	2.07	1.76	1.33	0.98	0.52	0.16	-0.26	-0.61	-1.12	-1.49	-2.02
8.62	2.28	2.01	1.55	1.11	0.77	0.27	-0.17	-0.56	-0.95	-1.29	-1.81
8.82	2.36	1.97	1.47	1.10	0.64	0.36	-0.10	-0.53	-0.84	-1.29	-1.84
9.02	2.32	1.84	1.44	1.00	0.69	0.32	-0.07	-0.55	-1.06	-1.27	-1.94
9.22	2.21	1.71	1.38	0.93	0.57	0.20	-0.16	-0.65	-1.06	-1.45	-1.93

9.42	2.21	1.86	1.33	0.96	0.60	0.33	-0.19	-0.67	-1.15	-1.48	-1.86
9.62	2.21	1.86	1.46	1.05	0.75	0.25	-0.20	-0.61	-0.99	-1.35	-1.82
9.82	2.23	1.97	1.44	1.09	0.75	0.35	-0.12	-0.56	-0.88	-1.28	-1.73
10.02	2.10	1.72	1.32	0.88	0.50	0.19	-0.21	-0.65	-1.01	-1.43	-1.90
10.22	2.22	1.76	1.41	1.12	0.64	0.19	-0.19	-0.60	-1.08	-1.51	-1.83
10.42	2.29	1.77	1.38	1.09	0.64	0.21	-0.17	-0.60	-0.91	-1.34	-1.81
10.62	2.18	1.76	1.47	1.13	0.67	0.36	-0.11	-0.54	-0.90	-1.31	-1.87
10.82	2.24	1.94	1.41	1.20	0.82	0.35	-0.06	-0.50	-0.87	-1.37	-1.84
11.02	2.08	1.78	1.37	1.11	0.69	0.26	-0.12	-0.54	-0.90	-1.32	-1.83
11.22	2.01	1.66	1.25	0.82	0.57	0.09	-0.18	-0.70	-1.09	-1.42	-2.05
11.42	1.97	1.60	1.33	0.89	0.61	0.18	-0.30	-0.58	-0.99	-1.39	-1.86
11.62	2.18	1.71	1.36	0.96	0.55	0.10	-0.20	-0.57	-0.99	-1.42	-1.88
11.82	1.95	1.63	1.31	0.99	0.44	0.25	-0.38	-0.66	-1.03	-1.47	-1.89
12.02	2.18	1.84	1.43	1.13	0.74	0.26	-0.22	-0.53	-0.98	-1.38	-1.81
12.22	2.18	1.84	1.36	1.01	0.84	0.21	-0.21	-0.51	-0.81	-1.34	-1.80
12.42	2.07	1.70	1.25	0.96	0.59	0.23	-0.14	-0.67	-1.03	-1.44	-1.87
12.62	2.06	1.77	1.35	1.02	0.59	0.21	-0.32	-0.58	-0.97	-1.38	-1.83
12.82	2.00	1.61	1.30	0.85	0.50	0.25	-0.22	-0.77	-1.11	-1.45	-1.82
13.02	2.21	1.86	1.35	1.07	0.68	0.36	-0.06	-0.49	-0.86	-1.29	-1.75
13.22	2.09	1.73	1.21	0.89	0.68	0.18	-0.28	-0.58	-1.09	-1.42	-1.91
13.42	1.93	1.66	1.34	0.96	0.47	0.14	-0.25	-0.59	-0.98	-1.31	-1.93
13.50	1.94	1.61	1.15	0.88	0.44	0.10	-0.36	-0.69	-1.12	-1.47	-1.94
13.59	2.15	1.82	1.44	1.04	0.66	0.36	-0.19	-0.48	-0.94	-1.37	-1.71
13.68	2.04	1.56	1.21	0.84	0.37	0.08	-0.26	-0.73	-1.05	-1.52	-1.89
13.78	1.95	1.65	1.25	0.93	0.59	0.18	-0.31	-0.76	-1.14	-1.43	-1.92
13.88	2.02	1.68	1.35	0.90	0.59	0.16	-0.23	-0.64	-1.08	-1.45	-1.87
14.00	2.17	1.82	1.34	0.91	0.70	0.24	-0.21	-0.53	-0.98	-1.38	-1.87
14.12	1.91	1.52	1.17	0.84	0.51	0.10	-0.32	-0.69	-1.11	-1.56	-1.99
14.25	2.06	1.57	1.24	0.86	0.55	0.11	-0.30	-0.71	-1.02	-1.52	-1.95
14.40	2.07	1.67	1.29	0.92	0.60	0.18	-0.29	-0.57	-1.08	-1.48	-1.87
14.55	2.02	1.60	1.27	0.82	0.48	0.19	-0.31	-0.69	-1.06	-1.48	-1.92
14.72	1.99	1.55	1.18	1.01	0.57	0.12	-0.18	-0.62	-1.04	-1.34	-1.82
14.90	2.01	1.66	1.32	0.99	0.59	0.26	-0.25	-0.61	-0.91	-1.29	-1.79
15.09	1.94	1.58	1.26	0.92	0.45	0.21	-0.29	-0.67	-1.07	-1.45	-1.90
15.30	2.01	1.56	1.27	0.88	0.49	0.19	-0.25	-0.64	-1.11	-1.52	-1.96
15.53	2.05	1.63	1.23	0.89	0.55	0.17	-0.27	-0.71	-1.10	-1.46	-1.99
15.77	1.97	1.56	1.11	0.89	0.53	0.14	-0.32	-0.72	-1.15	-1.53	-1.90
16.04	2.20	1.72	1.46	1.01	0.64	0.18	-0.20	-0.50	-0.94	-1.42	-1.76
16.32	1.93	1.63	1.26	1.00	0.46	0.12	-0.31	-0.61	-1.01	-1.51	-1.85
16.63	2.02	1.71	1.31	0.95	0.60	0.14	-0.19	-0.65	-0.98	-1.45	-1.86
16.96	1.89	1.56	1.14	0.87	0.41	0.01	-0.37	-0.78	-1.17	-1.56	-2.06
17.31	2.07	1.63	1.27	0.91	0.47	0.10	-0.19	-0.65	-1.10	-1.43	-1.82
17.70	2.12	1.67	1.37	1.04	0.48	0.21	-0.29	-0.60	-1.05	-1.57	-1.93
18.11	1.84	1.62	1.11	0.82	0.42	0.01	-0.38	-0.75	-1.21	-1.50	-2.14
18.56	2.02	1.64	1.28	0.97	0.55	0.12	-0.20	-0.62	-1.02	-1.42	-1.87
19.04	1.98	1.54	1.27	0.86	0.47	0.03	-0.41	-0.75	-1.14	-1.40	-1.95
19.56	2.04	1.70	1.33	1.02	0.55	0.16	-0.21	-0.56	-1.01	-1.45	-1.73
20.12	1.93	1.53	1.14	0.82	0.49	-0.01	-0.29	-0.59	-1.10	-1.51	-2.01
20.72	2.05	1.70	1.32	1.01	0.62	0.23	-0.21	-0.55	-1.08	-1.45	-1.85
21.37	1.97	1.57	1.25	0.93	0.49	0.08	-0.42	-0.82	-1.11	-1.50	-2.00

22.08	1.94	1.60	1.25	1.07	0.61	0.25	-0.10	-0.44	-0.95	-1.34	-1.75
22.84	2.04	1.53	1.26	0.91	0.48	0.16	-0.33	-0.68	-1.13	-1.56	-2.00
23.65	1.94	1.50	1.18	0.83	0.38	0.01	-0.30	-0.78	-1.13	-1.58	-1.95
24.54	1.84	1.54	1.20	0.81	0.50	0.08	-0.35	-0.74	-1.15	-1.61	-2.01
25.49	1.87	1.58	1.21	0.73	0.45	0.06	-0.30	-0.78	-1.12	-1.65	-2.00
26.51	1.71	1.42	0.84	0.71	0.34	-0.09	-0.48	-0.90	-1.30	-1.65	-2.24
27.62	1.98	1.32	1.05	0.80	0.28	-0.05	-0.43	-0.89	-1.29	-1.65	-2.17
28.81	1.87	1.55	1.25	0.80	0.40	0.07	-0.33	-0.71	-1.16	-1.53	-2.04
30.10	1.68	1.39	1.02	0.66	0.25	-0.04	-0.40	-0.86	-1.33	-1.64	-2.11
31.49	1.94	1.63	1.27	0.83	0.47	0.09	-0.31	-0.67	-1.07	-1.53	-2.06
32.98	1.95	1.56	1.21	0.83	0.44	0.12	-0.34	-0.67	-1.16	-1.59	-2.04
34.60	1.81	1.44	1.06	0.79	0.35	-0.05	-0.45	-0.89	-1.20	-1.65	-2.06
36.34	1.77	1.37	0.87	0.57	0.21	-0.03	-0.42	-0.91	-1.23	-1.71	-2.18
38.21	1.92	1.54	1.31	0.85	0.41	0.05	-0.32	-0.70	-1.10	-1.61	-2.01
40.24	1.71	1.46	1.05	0.65	0.27	-0.06	-0.58	-0.77	-1.16	-1.59	-2.14
42.42	1.67	1.28	1.03	0.68	0.33	-0.06	-0.59	-0.93	-1.39	-1.78	-2.26
44.78	1.67	1.32	0.95	0.65	0.36	-0.01	-0.48	-0.93	-1.26	-1.73	-2.21
47.32	1.55	1.41	1.06	0.63	0.23	0.00	-0.49	-0.97	-1.28	-1.65	-2.09
50.06	1.78	1.39	1.06	0.69	0.28	-0.10	-0.43	-0.84	-1.17	-1.62	-2.09
53.01	1.60	1.19	0.97	0.56	0.20	-0.18	-0.57	-1.10	-1.36	-1.92	-2.17
56.20	1.72	1.37	0.89	0.60	0.19	-0.13	-0.57	-0.91	-1.35	-1.66	-2.13
59.64	1.63	1.33	0.93	0.52	0.22	-0.10	-0.48	-0.94	-1.28	-1.69	-2.29
63.34	1.65	1.20	0.93	0.57	0.21	-0.14	-0.52	-0.90	-1.34	-1.63	-2.16
67.34	1.58	1.22	1.00	0.56	0.27	-0.10	-0.50	-0.98	-1.45	-1.83	-2.15
71.65	1.60	1.17	0.90	0.57	0.23	-0.29	-0.57	-1.03	-1.46	-1.87	-2.30
76.30	1.64	1.23	0.95	0.60	0.05	-0.14	-0.60	-0.92	-1.33	-1.74	-2.03
81.32	1.68	1.27	0.98	0.63	0.27	-0.10	-0.54	-0.93	-1.40	-1.64	-2.15
86.73	1.58	1.28	0.87	0.55	0.28	-0.16	-0.57	-1.04	-1.37	-1.66	-2.10
92.56	1.65	1.33	0.96	0.58	0.27	-0.12	-0.51	-0.91	-1.28	-1.64	-2.22
98.85	1.61	1.30	0.85	0.54	0.02	-0.25	-0.55	-1.02	-1.46	-1.71	-2.19
105.64	1.78	1.31	0.92	0.67	0.24	-0.20	-0.55	-0.93	-1.37	-1.74	-2.22
112.96	1.61	1.24	0.81	0.58	0.26	-0.16	-0.62	-0.92	-1.28	-1.77	-2.21
120.85	1.48	1.26	0.85	0.52	0.14	-0.28	-0.69	-0.96	-1.35	-1.75	-2.26
129.36	1.69	1.32	1.02	0.60	0.26	-0.09	-0.53	-0.99	-1.52	-1.70	-2.22
138.55	1.70	1.39	0.97	0.67	0.34	0.07	-0.41	-0.81	-1.30	-1.72	-2.12
148.45	1.61	1.14	0.84	0.52	0.15	-0.18	-0.64	-1.02	-1.44	-1.79	-2.17
159.13	1.54	1.20	0.82	0.58	0.21	-0.25	-0.59	-0.97	-1.28	-1.78	-2.25
170.65	1.46	1.02	0.71	0.40	-0.02	-0.38	-0.71	-1.12	-1.54	-1.83	-2.33
183.07	1.58	1.30	0.91	0.52	0.32	-0.15	-0.67	-0.99	-1.35	-1.81	-2.32
196.47	1.44	1.18	0.75	0.39	0.16	-0.29	-0.59	-1.09	-1.46	-1.81	-2.27
210.91	1.43	1.12	0.85	0.43	0.08	-0.19	-0.61	-0.96	-1.42	-1.78	-2.26
226.50	1.55	1.21	0.95	0.59	0.19	-0.11	-0.51	-0.90	-1.34	-1.79	-2.26
243.31	1.55	1.19	0.87	0.57	0.24	-0.27	-0.44	-0.96	-1.30	-1.76	-2.15
261.43	1.52	1.28	0.80	0.55	0.23	-0.18	-0.64	-0.94	-1.42	-1.80	-2.30
280.98	1.55	1.29	0.83	0.55	0.26	-0.06	-0.58	-0.92	-1.34	-1.84	-2.17
302.07	1.64	1.26	1.00	0.59	0.16	-0.04	-0.58	-0.91	-1.36	-1.71	-2.02
324.81	1.46	1.07	0.81	0.42	0.06	-0.15	-0.66	-0.93	-1.51	-1.97	-2.33
349.33	1.55	1.26	0.96	0.56	0.34	-0.17	-0.49	-0.83	-1.26	-1.63	-2.13
375.79	1.62	1.30	0.80	0.55	0.16	-0.10	-0.68	-0.90	-1.36	-1.73	-2.20
404.31	1.58	1.27	0.81	0.51	0.16	-0.16	-0.51	-1.06	-1.37	-1.79	-2.14

435.08	1.50	1.12	0.83	0.42	0.18	-0.23	-0.65	-0.92	-1.30	-1.76	-2.22
468.27	1.53	1.25	0.80	0.53	0.16	-0.16	-0.68	-0.87	-1.41	-1.79	-2.15
504.06	1.52	1.16	0.89	0.42	0.10	-0.26	-0.55	-1.03	-1.28	-1.71	-2.17
542.66	1.44	1.23	0.83	0.52	0.07	-0.26	-0.67	-1.04	-1.37	-1.79	-2.23
584.29	1.61	1.23	0.78	0.50	0.20	-0.16	-0.55	-0.94	-1.33	-1.74	-2.11
629.18	1.49	1.30	0.91	0.62	0.17	-0.19	-0.54	-0.97	-1.28	-1.68	-2.13
677.61	1.44	1.15	0.83	0.50	0.18	-0.24	-0.61	-0.88	-1.40	-1.71	-2.22
729.83	1.53	1.24	0.86	0.59	0.18	-0.14	-0.67	-0.99	-1.39	-1.60	-2.09
786.16	1.62	1.32	1.04	0.71	0.22	-0.02	-0.50	-0.79	-1.21	-1.58	-2.03
846.90	1.44	1.08	0.76	0.44	0.21	-0.31	-0.65	-1.07	-1.50	-1.78	-2.27
912.42	1.40	1.12	0.63	0.46	0.22	-0.25	-0.62	-1.02	-1.53	-1.78	-2.19
983.08	1.50	1.18	0.72	0.50	0.08	-0.15	-0.59	-1.01	-1.36	-1.71	-2.09
1059.29	1.50	1.08	0.92	0.52	0.17	-0.27	-0.60	-0.95	-1.43	-1.80	-2.14
1141.48	1.27	1.01	0.67	0.41	-0.08	-0.23	-0.66	-1.14	-1.55	-1.96	-2.38
1230.13	1.49	1.13	0.75	0.49	0.20	-0.12	-0.56	-1.04	-1.42	-1.77	-2.15
1325.73	1.43	1.09	0.86	0.43	0.10	-0.19	-0.74	-0.99	-1.40	-1.84	-2.27
1428.84	1.30	0.91	0.58	0.29	0.02	-0.32	-0.79	-1.09	-1.38	-1.93	-2.23
1540.05	1.37	0.98	0.60	0.29	-0.13	-0.42	-0.83	-1.17	-1.38	-1.95	-2.39
1659.99	1.38	0.97	0.59	0.32	-0.07	-0.36	-0.79	-1.15	-1.52	-1.94	-2.35
1789.34	1.20	0.87	0.46	0.12	-0.05	-0.57	-0.93	-1.26	-1.56	-2.06	-2.52
1928.85	1.20	0.92	0.57	0.26	-0.13	-0.41	-0.80	-1.24	-1.66	-1.99	-2.47
2079.31	1.12	0.79	0.57	0.24	-0.13	-0.49	-0.85	-1.26	-1.61	-2.04	-2.44
2241.59	1.27	0.81	0.56	0.19	-0.15	-0.52	-0.95	-1.23	-1.79	-2.12	-2.45
2416.60	1.31	0.91	0.60	0.23	-0.05	-0.46	-0.88	-1.14	-1.65	-1.98	-2.40
2605.36	1.22	0.86	0.59	0.24	-0.06	-0.49	-0.91	-1.35	-1.62	-2.06	-2.45
2808.94	1.00	0.71	0.41	0.08	-0.29	-0.70	-1.08	-1.44	-1.74	-2.22	-2.61
3028.50	1.11	0.75	0.48	0.19	-0.32	-0.55	-0.86	-1.34	-1.75	-2.10	-2.54
3265.30	1.25	0.81	0.47	0.29	-0.14	-0.57	-0.97	-1.33	-1.54	-2.01	-2.53
3520.69	1.21	1.01	0.62	0.30	-0.08	-0.34	-0.79	-1.12	-1.46	-2.03	-2.36
3796.13	1.24	1.02	0.69	0.39	-0.04	-0.29	-0.67	-1.12	-1.48	-1.82	-2.22

Wavelength (nm) / Time (ps)	459.41	460.83	462.25	463.68	465.10	466.53	467.95	469.37	470.80	472.22	473.65
-3.78	0.02	0.03	-0.03	0.07	0.13	0.14	0.08	0.04	0.07	0.07	0.11
-3.28	0.08	0.05	0.07	0.10	0.03	0.10	0.09	0.13	0.14	0.14	0.09
-2.78	-0.02	-0.04	-0.03	-0.05	0.01	-0.10	-0.10	-0.06	-0.01	-0.14	-0.03
-2.28	0.02	-0.01	0.04	0.00	-0.02	-0.04	0.00	0.12	-0.13	-0.03	-0.02
-1.78	0.02	0.07	0.01	0.01	0.01	0.01	0.11	0.01	0.04	0.11	0.07
-1.28	0.06	-0.05	-0.04	-0.11	-0.09	-0.05	-0.06	-0.12	-0.05	-0.02	-0.12
-0.78	-0.14	-0.07	-0.02	-0.06	-0.13	-0.02	-0.13	-0.06	-0.04	-0.14	-0.11
-0.28	-0.04	0.02	0.00	0.06	0.07	-0.04	0.02	-0.06	-0.01	0.00	0.01
-0.18	-0.01	-0.06	-0.03	-0.11	0.03	0.07	-0.02	0.07	0.13	0.10	0.06
-0.08	0.10	-0.07	0.03	-0.03	0.07	0.08	-0.01	0.06	0.11	0.01	0.03
0.02	0.04	0.01	0.09	0.00	0.02	0.10	0.03	0.06	0.00	-0.03	-0.01
0.12	0.05	0.04	0.03	0.04	0.01	0.13	0.03	0.09	0.07	0.10	0.17
0.22	-0.06	-0.12	-0.10	-0.08	-0.06	-0.13	-0.24	-0.05	-0.16	-0.07	-0.10
0.27	-0.05	-0.08	0.08	-0.01	0.05	-0.04	-0.01	0.08	0.05	0.04	-0.02
0.32	0.13	0.08	0.16	0.16	0.10	0.13	0.11	0.22	0.09	0.11	0.06

0.37	0.06	0.04	0.03	0.06	0.08	-0.04	-0.04	0.01	-0.05	-0.03	-0.02
0.42	0.02	-0.09	-0.07	-0.06	0.07	-0.05	0.01	-0.04	-0.04	-0.11	-0.07
0.47	0.04	-0.01	-0.09	-0.06	-0.07	-0.15	-0.11	-0.12	-0.12	-0.01	-0.01
0.52	-0.03	-0.07	-0.02	-0.11	0.03	0.00	-0.09	0.06	-0.09	0.03	-0.06
0.57	0.13	0.05	0.15	0.10	0.08	0.10	0.12	0.11	0.13	0.14	0.14
0.62	0.08	0.09	-0.02	-0.06	-0.08	0.04	-0.07	0.07	0.04	-0.01	0.01
0.67	0.00	-0.11	-0.04	0.16	-0.04	-0.05	-0.19	-0.08	0.02	-0.03	-0.11
0.72	0.04	0.00	0.03	0.05	-0.05	-0.06	-0.06	-0.03	-0.05	0.06	-0.04
0.77	0.05	-0.03	-0.15	-0.04	-0.08	-0.07	-0.13	-0.10	-0.05	-0.08	-0.06
0.82	0.05	0.00	-0.01	0.03	0.05	-0.01	-0.05	-0.01	0.02	-0.09	-0.02
0.87	0.13	-0.01	0.02	-0.03	0.10	-0.02	-0.02	-0.01	0.06	0.15	0.01
0.92	-0.10	-0.05	0.07	-0.12	-0.06	-0.07	-0.14	-0.05	0.02	-0.12	-0.06
0.97	0.10	0.16	0.03	0.04	0.00	0.07	0.15	0.06	0.09	0.12	0.06
1.02	-0.32	-0.08	0.26	0.09	-0.12	-0.16	-0.19	-0.06	0.00	-0.08	-0.02
1.07	-15.49	-6.85	-0.72	1.90	1.92	0.51	-0.75	-1.13	-0.86	-0.45	-0.07
1.12	55.07	30.35	8.96	-8.49	-22.03	-32.25	-39.13	-43.06	-44.04	-41.69	-36.71
1.17	31.78	54.27	75.81	95.50	112.39	124.91	131.66	130.95	123.76	109.80	92.67
1.22	2.63	-5.88	-20.27	-38.25	-56.21	-70.03	-77.74	-77.17	-68.94	-53.77	-33.75
1.27	-2.51	-1.40	0.39	2.24	3.26	2.98	1.13	-1.60	-4.49	-6.10	-5.98
1.32	0.15	-1.49	-2.82	-3.58	-3.87	-3.62	-3.68	-4.18	-5.46	-7.38	-9.42
1.37	-0.39	-0.27	-0.57	-1.66	-3.32	-5.13	-6.65	-7.62	-7.84	-7.40	-6.88
1.42	-2.52	-3.35	-3.35	-3.15	-2.95	-3.08	-3.82	-4.91	-6.03	-7.26	-8.06
1.47	-0.56	-1.77	-3.12	-4.35	-5.03	-5.07	-4.73	-4.26	-4.13	-4.88	-5.99
1.52	-1.77	-1.37	-1.16	-1.93	-3.09	-4.53	-5.94	-6.99	-7.46	-7.50	-7.30
1.57	-2.34	-3.36	-3.54	-3.49	-3.15	-3.04	-3.65	-4.62	-6.20	-7.67	-8.86
1.62	-0.45	-1.51	-3.06	-4.52	-5.63	-5.98	-5.79	-5.26	-4.93	-5.10	-5.85
1.67	-2.53	-2.29	-2.10	-2.50	-3.28	-4.34	-5.73	-6.61	-7.29	-7.43	-7.51
1.72	-1.57	-2.81	-3.72	-4.25	-4.20	-3.99	-4.02	-4.33	-5.12	-6.35	-7.76
1.77	-1.21	-1.29	-1.80	-2.94	-4.14	-5.31	-6.37	-6.61	-6.65	-6.58	-6.61
1.82	-2.98	-3.41	-3.34	-3.21	-3.39	-3.77	-4.79	-5.86	-6.91	-7.73	-8.21
1.87	-1.83	-2.85	-3.74	-4.61	-4.82	-4.71	-4.62	-4.54	-5.13	-5.94	-7.09
1.92	-1.94	-1.82	-1.99	-2.40	-3.30	-4.48	-5.67	-6.53	-6.96	-7.16	-7.02
1.97	-2.44	-3.15	-3.47	-3.70	-3.68	-3.66	-4.08	-4.87	-5.96	-7.02	-7.98
2.02	-1.56	-2.12	-2.87	-3.86	-4.66	-5.27	-5.82	-5.90	-5.88	-6.09	-6.43
2.07	-2.27	-2.19	-2.25	-2.80	-3.48	-4.50	-5.52	-6.19	-6.74	-6.94	-7.07
2.12	-2.11	-2.89	-3.62	-4.14	-4.25	-4.28	-4.55	-4.89	-5.65	-6.63	-7.62
2.17	-1.62	-2.08	-2.82	-3.76	-4.58	-5.26	-5.79	-5.80	-5.95	-6.00	-6.42
2.22	-2.63	-3.02	-3.04	-3.26	-3.47	-3.86	-4.86	-5.62	-6.44	-7.23	-7.68
2.27	-1.77	-2.61	-3.44	-4.13	-4.62	-4.69	-4.73	-4.94	-5.36	-6.00	-6.96
2.32	-1.82	-1.98	-2.34	-2.95	-3.83	-4.72	-5.67	-6.17	-6.44	-6.69	-6.81
2.37	-2.57	-3.19	-3.51	-3.73	-3.83	-4.03	-4.63	-5.25	-6.10	-6.92	-7.70
2.42	-1.90	-2.55	-3.29	-4.17	-4.57	-4.98	-5.24	-5.27	-5.66	-6.17	-6.86
2.47	-2.26	-2.24	-2.52	-3.07	-3.71	-4.49	-5.24	-6.03	-6.43	-6.70	-6.97
2.52	-2.17	-3.01	-3.45	-3.98	-4.09	-4.39	-4.65	-5.15	-5.92	-6.64	-7.38
2.57	-1.81	-2.26	-2.85	-3.75	-4.43	-5.02	-5.44	-5.67	-5.87	-6.13	-6.61
2.62	-2.47	-2.77	-2.98	-3.29	-3.72	-4.30	-5.10	-5.85	-6.51	-7.08	-7.37
2.67	-2.07	-2.84	-3.38	-3.84	-4.11	-4.27	-4.68	-4.99	-5.71	-6.42	-7.13
2.72	-1.84	-2.18	-2.63	-3.25	-3.95	-4.78	-5.41	-5.80	-6.04	-6.32	-6.68
2.77	-2.38	-2.97	-3.34	-3.55	-3.78	-4.14	-4.78	-5.38	-6.10	-6.84	-7.27
2.82	-1.95	-2.59	-3.34	-3.91	-4.33	-4.66	-5.07	-5.30	-5.74	-6.19	-6.87

2.87	-2.14	-2.42	-2.85	-3.34	-3.99	-4.42	-5.13	-5.65	-6.12	-6.50	-6.93
2.92	-2.36	-2.98	-3.47	-3.84	-4.04	-4.25	-4.74	-5.18	-5.81	-6.54	-7.39
2.97	-1.90	-2.36	-2.83	-3.65	-4.21	-4.80	-5.28	-5.63	-5.84	-6.18	-6.66
3.02	-2.40	-2.61	-2.98	-3.28	-3.85	-4.30	-5.00	-5.62	-6.23	-6.71	-7.00
3.07	-2.24	-2.72	-3.38	-3.82	-4.24	-4.53	-4.99	-5.36	-5.78	-6.37	-7.13
3.12	-2.03	-2.47	-2.88	-3.49	-4.13	-4.76	-5.32	-5.64	-5.91	-6.20	-6.67
3.17	-2.51	-2.94	-3.25	-3.68	-4.00	-4.37	-4.91	-5.53	-5.98	-6.70	-7.20
3.22	-1.93	-2.51	-3.12	-3.78	-4.28	-4.70	-5.07	-5.28	-5.75	-6.24	-6.85
3.42	-2.42	-2.74	-3.05	-3.47	-4.02	-4.51	-5.18	-5.78	-6.23	-6.75	-7.10
3.62	-2.15	-2.81	-3.39	-3.83	-4.16	-4.53	-4.93	-5.37	-5.92	-6.43	-7.05
3.82	-2.22	-2.56	-2.96	-3.39	-3.83	-4.32	-5.08	-5.53	-6.00	-6.45	-6.93
4.02	-2.15	-2.74	-3.22	-3.72	-4.17	-4.51	-5.02	-5.30	-5.87	-6.36	-6.88
4.22	-2.24	-2.61	-3.11	-3.59	-4.01	-4.52	-5.08	-5.53	-6.07	-6.59	-6.92
4.42	-2.14	-2.67	-3.14	-3.64	-4.08	-4.37	-4.87	-5.35	-5.83	-6.38	-6.96
4.62	-2.32	-2.76	-3.17	-3.72	-4.12	-4.57	-5.19	-5.51	-6.03	-6.42	-6.99
4.82	-2.20	-2.71	-3.09	-3.66	-4.09	-4.50	-4.97	-5.26	-5.92	-6.26	-6.88
5.02	-2.29	-2.78	-3.23	-3.69	-4.15	-4.65	-5.25	-5.56	-6.12	-6.55	-6.84
5.22	-2.16	-2.65	-3.09	-3.60	-3.94	-4.44	-4.96	-5.33	-5.78	-6.28	-6.88
5.42	-2.19	-2.72	-3.18	-3.60	-4.06	-4.49	-5.11	-5.42	-5.84	-6.37	-6.93
5.62	-2.23	-2.70	-3.22	-3.60	-3.99	-4.55	-4.98	-5.32	-5.79	-6.36	-6.89
5.82	-2.25	-2.87	-3.11	-3.64	-4.11	-4.62	-5.15	-5.51	-5.87	-6.32	-6.97
6.02	-2.22	-2.69	-3.13	-3.54	-4.02	-4.36	-4.95	-5.23	-5.83	-6.30	-6.73
6.22	-2.26	-2.63	-3.19	-3.63	-4.14	-4.48	-4.95	-5.43	-5.87	-6.31	-6.81
6.42	-2.35	-2.84	-3.34	-3.76	-4.14	-4.49	-5.01	-5.51	-5.91	-6.37	-6.88
6.62	-2.21	-2.71	-3.04	-3.53	-3.98	-4.45	-4.97	-5.45	-5.89	-6.39	-6.80
6.82	-2.31	-2.84	-3.16	-3.64	-4.08	-4.55	-4.93	-5.40	-5.82	-6.42	-6.87
7.02	-2.23	-2.65	-3.14	-3.59	-4.06	-4.51	-5.02	-5.30	-5.89	-6.30	-6.76
7.22	-2.17	-2.63	-3.09	-3.53	-3.97	-4.37	-4.91	-5.18	-5.79	-6.15	-6.64
7.42	-2.31	-2.70	-3.10	-3.59	-4.08	-4.58	-4.95	-5.27	-5.86	-6.25	-6.74
7.62	-2.32	-2.67	-3.09	-3.58	-4.00	-4.53	-4.89	-5.28	-5.87	-6.25	-6.68
7.82	-2.29	-2.75	-3.16	-3.63	-4.03	-4.48	-5.09	-5.39	-5.77	-6.21	-6.60
8.02	-2.27	-2.77	-3.18	-3.58	-4.14	-4.53	-5.08	-5.46	-5.85	-6.37	-6.73
8.22	-2.16	-2.69	-3.07	-3.60	-3.95	-4.42	-4.90	-5.28	-5.68	-6.13	-6.60
8.42	-2.43	-2.88	-3.24	-3.59	-4.16	-4.52	-5.11	-5.46	-5.85	-6.14	-6.76
8.62	-2.22	-2.58	-3.10	-3.58	-3.92	-4.37	-4.76	-5.19	-5.59	-6.15	-6.53
8.82	-2.17	-2.62	-3.12	-3.56	-3.96	-4.41	-4.87	-5.29	-5.81	-6.11	-6.63
9.02	-2.12	-2.68	-3.08	-3.56	-3.95	-4.34	-4.76	-5.33	-5.69	-6.13	-6.67
9.22	-2.29	-2.66	-3.09	-3.47	-4.03	-4.40	-5.11	-5.42	-5.71	-6.22	-6.68
9.42	-2.37	-2.67	-3.13	-3.62	-4.02	-4.40	-4.98	-5.31	-5.76	-6.22	-6.59
9.62	-2.16	-2.62	-3.15	-3.53	-3.86	-4.30	-4.96	-5.24	-5.67	-6.21	-6.69
9.82	-2.18	-2.64	-3.03	-3.49	-3.87	-4.26	-4.85	-5.12	-5.50	-6.04	-6.51
10.02	-2.27	-2.83	-3.23	-3.70	-3.96	-4.37	-5.01	-5.30	-5.81	-6.18	-6.56
10.22	-2.35	-2.74	-3.25	-3.62	-4.11	-4.41	-5.00	-5.31	-5.71	-6.24	-6.61
10.42	-2.19	-2.71	-3.12	-3.46	-3.97	-4.40	-4.87	-5.22	-5.59	-5.95	-6.47
10.62	-2.25	-2.60	-2.94	-3.51	-3.85	-4.32	-4.92	-5.16	-5.57	-5.96	-6.52
10.82	-2.18	-2.64	-3.01	-3.47	-3.88	-4.32	-4.76	-5.14	-5.49	-6.06	-6.49
11.02	-2.18	-2.62	-3.13	-3.57	-3.98	-4.32	-4.79	-5.17	-5.62	-6.04	-6.50
11.22	-2.22	-2.73	-3.21	-3.65	-3.91	-4.39	-4.80	-5.28	-5.61	-6.05	-6.47
11.42	-2.39	-2.67	-3.03	-3.60	-3.97	-4.37	-4.71	-5.16	-5.66	-6.03	-6.45
11.62	-2.26	-2.66	-3.04	-3.51	-3.92	-4.34	-4.87	-5.13	-5.58	-6.10	-6.48

11.82	-2.39	-2.80	-3.12	-3.51	-3.97	-4.44	-4.93	-5.19	-5.78	-6.13	-6.54
12.02	-2.11	-2.61	-2.98	-3.49	-3.80	-4.39	-4.81	-5.12	-5.59	-5.94	-6.42
12.22	-2.16	-2.58	-3.05	-3.49	-3.98	-4.28	-4.76	-5.23	-5.70	-6.08	-6.49
12.42	-2.24	-2.71	-3.12	-3.59	-3.92	-4.35	-4.79	-5.18	-5.60	-5.99	-6.45
12.62	-2.16	-2.62	-3.09	-3.42	-3.87	-4.31	-4.92	-5.20	-5.55	-6.04	-6.41
12.82	-2.31	-2.63	-3.14	-3.55	-3.88	-4.34	-4.81	-5.19	-5.57	-5.95	-6.46
13.02	-2.09	-2.56	-2.93	-3.44	-3.82	-4.28	-4.83	-5.16	-5.59	-5.97	-6.39
13.22	-2.30	-2.63	-3.14	-3.49	-3.98	-4.28	-4.77	-5.18	-5.53	-5.95	-6.48
13.42	-2.28	-2.69	-3.12	-3.58	-3.99	-4.37	-4.91	-5.23	-5.50	-6.01	-6.35
13.50	-2.33	-2.72	-3.16	-3.58	-4.03	-4.35	-4.84	-5.23	-5.71	-5.96	-6.45
13.59	-2.20	-2.66	-2.94	-3.46	-3.93	-4.21	-4.76	-4.99	-5.52	-5.96	-6.43
13.68	-2.30	-2.77	-3.13	-3.54	-3.93	-4.45	-4.82	-5.18	-5.58	-6.06	-6.39
13.78	-2.22	-2.73	-3.13	-3.61	-3.97	-4.27	-4.91	-5.20	-5.55	-6.05	-6.53
13.88	-2.28	-2.82	-3.08	-3.53	-3.91	-4.30	-4.78	-5.15	-5.54	-5.97	-6.45
14.00	-2.26	-2.63	-3.05	-3.53	-3.90	-4.36	-4.78	-5.19	-5.53	-6.04	-6.41
14.12	-2.42	-2.76	-3.26	-3.59	-4.05	-4.44	-4.95	-5.27	-5.67	-6.07	-6.45
14.25	-2.35	-2.80	-3.11	-3.55	-3.92	-4.32	-4.85	-5.20	-5.52	-5.94	-6.43
14.40	-2.30	-2.62	-3.11	-3.64	-4.14	-4.40	-4.93	-5.24	-5.67	-6.00	-6.54
14.55	-2.22	-2.68	-3.18	-3.66	-3.93	-4.32	-4.84	-5.23	-5.59	-6.03	-6.56
14.72	-2.23	-2.54	-3.06	-3.49	-3.96	-4.33	-4.87	-5.23	-5.58	-5.95	-6.42
14.90	-2.22	-2.61	-2.92	-3.42	-3.79	-4.29	-4.74	-5.07	-5.49	-5.93	-6.27
15.09	-2.29	-2.85	-3.12	-3.64	-3.90	-4.35	-4.87	-5.15	-5.52	-6.03	-6.43
15.30	-2.30	-2.75	-3.08	-3.58	-3.95	-4.37	-4.83	-5.21	-5.61	-6.04	-6.31
15.53	-2.31	-2.80	-3.31	-3.58	-4.07	-4.59	-4.93	-5.35	-5.66	-6.18	-6.56
15.77	-2.34	-2.76	-3.12	-3.55	-3.92	-4.32	-4.78	-5.16	-5.70	-5.95	-6.41
16.04	-2.22	-2.61	-3.08	-3.51	-3.96	-4.25	-4.87	-5.12	-5.61	-5.99	-6.46
16.32	-2.28	-2.78	-3.18	-3.58	-3.92	-4.37	-4.82	-5.14	-5.55	-6.01	-6.32
16.63	-2.35	-2.69	-3.14	-3.53	-3.96	-4.30	-4.87	-5.32	-5.61	-5.96	-6.29
16.96	-2.51	-2.77	-3.26	-3.73	-4.05	-4.47	-4.96	-5.26	-5.72	-6.00	-6.50
17.31	-2.25	-2.73	-3.09	-3.62	-3.90	-4.31	-4.96	-5.24	-5.52	-6.02	-6.55
17.70	-2.26	-2.67	-3.18	-3.68	-4.05	-4.48	-4.77	-5.28	-5.68	-6.08	-6.45
18.11	-2.32	-2.72	-3.21	-3.63	-3.96	-4.39	-4.82	-5.30	-5.63	-5.92	-6.48
18.56	-2.23	-2.69	-3.03	-3.48	-3.87	-4.30	-4.72	-5.13	-5.49	-5.98	-6.38
19.04	-2.33	-2.78	-3.13	-3.72	-4.03	-4.31	-4.94	-5.19	-5.56	-5.99	-6.39
19.56	-2.17	-2.63	-3.18	-3.62	-3.89	-4.37	-4.75	-5.10	-5.51	-5.96	-6.30
20.12	-2.51	-2.82	-3.29	-3.70	-4.09	-4.52	-4.99	-5.41	-5.70	-6.04	-6.54
20.72	-2.24	-2.64	-3.13	-3.53	-3.82	-4.25	-4.66	-5.19	-5.62	-5.92	-6.41
21.37	-2.23	-2.85	-3.23	-3.72	-4.00	-4.38	-4.79	-5.19	-5.48	-5.92	-6.43
22.08	-2.29	-2.75	-3.20	-3.56	-4.07	-4.47	-5.00	-5.22	-5.72	-6.04	-6.50
22.84	-2.41	-2.75	-3.14	-3.62	-4.02	-4.41	-4.93	-5.24	-5.69	-5.98	-6.46
23.65	-2.40	-2.75	-3.16	-3.50	-3.94	-4.34	-4.84	-5.21	-5.63	-5.97	-6.37
24.54	-2.45	-2.82	-3.27	-3.68	-4.09	-4.51	-5.01	-5.27	-5.68	-5.97	-6.38
25.49	-2.45	-2.84	-3.20	-3.73	-3.98	-4.51	-4.97	-5.27	-5.60	-6.02	-6.50
26.51	-2.55	-2.87	-3.39	-3.74	-4.14	-4.55	-4.95	-5.22	-5.66	-6.04	-6.49
27.62	-2.58	-2.90	-3.29	-3.78	-4.05	-4.48	-4.91	-5.28	-5.70	-6.15	-6.52
28.81	-2.38	-2.83	-3.13	-3.76	-4.00	-4.38	-4.91	-5.25	-5.65	-5.97	-6.37
30.10	-2.57	-2.97	-3.31	-3.77	-4.13	-4.48	-5.00	-5.32	-5.63	-6.05	-6.35
31.49	-2.42	-2.76	-3.13	-3.56	-4.06	-4.36	-4.81	-5.10	-5.56	-5.92	-6.36
32.98	-2.46	-2.81	-3.25	-3.67	-3.92	-4.34	-4.93	-5.14	-5.54	-5.90	-6.33
34.60	-2.44	-2.99	-3.39	-3.68	-3.97	-4.49	-4.91	-5.32	-5.60	-5.96	-6.49

36.34	-2.58	-2.96	-3.26	-3.84	-4.18	-4.48	-5.06	-5.34	-5.69	-6.03	-6.54
38.21	-2.43	-2.84	-3.17	-3.61	-3.97	-4.33	-4.90	-5.10	-5.57	-5.89	-6.31
40.24	-2.50	-2.89	-3.30	-3.77	-4.11	-4.42	-5.01	-5.22	-5.61	-6.06	-6.43
42.42	-2.48	-2.86	-3.37	-3.76	-4.05	-4.45	-4.88	-5.23	-5.55	-5.89	-6.40
44.78	-2.52	-3.02	-3.36	-3.72	-4.11	-4.39	-4.90	-5.18	-5.64	-5.83	-6.29
47.32	-2.48	-2.81	-3.32	-3.76	-4.04	-4.43	-4.88	-5.12	-5.60	-5.94	-6.39
50.06	-2.58	-2.78	-3.25	-3.66	-4.10	-4.48	-4.89	-5.33	-5.60	-5.85	-6.28
53.01	-2.61	-2.96	-3.37	-3.89	-4.13	-4.61	-5.00	-5.30	-5.85	-5.97	-6.45
56.20	-2.51	-3.03	-3.30	-3.79	-4.15	-4.60	-4.95	-5.28	-5.61	-5.97	-6.40
59.64	-2.61	-3.03	-3.43	-3.85	-4.09	-4.55	-4.94	-5.35	-5.69	-6.01	-6.36
63.34	-2.47	-2.99	-3.35	-3.64	-4.11	-4.45	-4.90	-5.07	-5.50	-5.86	-6.27
67.34	-2.51	-2.97	-3.34	-3.88	-4.19	-4.51	-4.91	-5.27	-5.55	-5.83	-6.17
71.65	-2.60	-3.01	-3.43	-3.82	-4.19	-4.62	-5.05	-5.37	-5.61	-6.08	-6.45
76.30	-2.55	-2.96	-3.33	-3.70	-4.07	-4.39	-5.05	-5.22	-5.55	-5.93	-6.29
81.32	-2.58	-3.02	-3.41	-3.74	-4.17	-4.56	-4.96	-5.29	-5.70	-6.03	-6.47
86.73	-2.49	-2.97	-3.32	-3.81	-4.21	-4.47	-5.04	-5.25	-5.64	-5.99	-6.37
92.56	-2.50	-3.00	-3.46	-3.73	-4.14	-4.53	-4.87	-5.15	-5.60	-5.92	-6.26
98.85	-2.57	-3.04	-3.42	-3.79	-4.11	-4.48	-4.94	-5.29	-5.55	-5.99	-6.33
105.64	-2.54	-2.95	-3.28	-3.79	-4.08	-4.48	-4.89	-5.15	-5.43	-5.85	-6.26
112.96	-2.61	-3.04	-3.48	-3.80	-4.10	-4.56	-4.97	-5.29	-5.60	-5.99	-6.36
120.85	-2.67	-2.95	-3.44	-3.78	-4.20	-4.59	-5.11	-5.33	-5.50	-6.00	-6.35
129.36	-2.63	-2.95	-3.39	-3.76	-4.09	-4.51	-4.97	-5.27	-5.59	-5.94	-6.36
138.55	-2.42	-2.83	-3.22	-3.65	-4.11	-4.42	-4.82	-5.27	-5.49	-5.79	-6.27
148.45	-2.57	-3.00	-3.41	-3.86	-4.12	-4.55	-4.97	-5.22	-5.51	-5.89	-6.35
159.13	-2.69	-2.91	-3.36	-3.74	-4.10	-4.44	-4.90	-5.17	-5.53	-5.89	-6.25
170.65	-2.63	-3.14	-3.60	-3.88	-4.20	-4.71	-5.05	-5.34	-5.63	-5.89	-6.32
183.07	-2.52	-2.98	-3.42	-3.77	-4.16	-4.56	-4.91	-5.23	-5.39	-5.82	-6.20
196.47	-2.56	-3.10	-3.46	-3.80	-4.25	-4.49	-4.83	-5.34	-5.61	-5.99	-6.22
210.91	-2.70	-3.06	-3.43	-3.84	-4.26	-4.62	-4.96	-5.19	-5.47	-5.94	-6.25
226.50	-2.58	-2.93	-3.45	-3.77	-4.08	-4.48	-4.82	-5.10	-5.39	-5.70	-6.07
243.31	-2.56	-2.98	-3.38	-3.76	-4.03	-4.32	-4.81	-5.03	-5.43	-5.79	-6.05
261.43	-2.55	-3.03	-3.36	-3.84	-4.07	-4.55	-4.92	-5.11	-5.45	-5.85	-6.19
280.98	-2.42	-2.82	-3.32	-3.68	-4.00	-4.45	-4.82	-5.04	-5.41	-5.73	-6.00
302.07	-2.48	-2.83	-3.21	-3.66	-3.96	-4.30	-4.78	-4.94	-5.28	-5.66	-5.96
324.81	-2.62	-3.00	-3.39	-3.77	-4.21	-4.51	-4.97	-5.11	-5.39	-5.70	-6.09
349.33	-2.59	-2.91	-3.23	-3.68	-4.00	-4.28	-4.71	-5.01	-5.22	-5.51	-5.92
375.79	-2.48	-2.95	-3.35	-3.72	-4.01	-4.30	-4.74	-5.06	-5.33	-5.73	-5.94
404.31	-2.47	-2.92	-3.36	-3.71	-3.95	-4.33	-4.62	-4.96	-5.27	-5.49	-5.87
435.08	-2.67	-2.93	-3.31	-3.70	-4.07	-4.42	-4.84	-5.10	-5.32	-5.57	-5.87
468.27	-2.54	-2.90	-3.24	-3.63	-4.09	-4.27	-4.62	-4.95	-5.13	-5.49	-5.76
504.06	-2.63	-2.99	-3.37	-3.62	-4.04	-4.32	-4.73	-4.95	-5.28	-5.65	-5.80
542.66	-2.61	-3.03	-3.26	-3.73	-3.99	-4.28	-4.66	-4.86	-5.14	-5.50	-5.80
584.29	-2.57	-2.90	-3.32	-3.64	-4.04	-4.26	-4.61	-4.88	-5.14	-5.51	-5.72
629.18	-2.42	-2.85	-3.12	-3.59	-3.82	-4.17	-4.54	-4.83	-5.03	-5.30	-5.71
677.61	-2.47	-2.87	-3.23	-3.70	-4.02	-4.22	-4.64	-4.86	-5.09	-5.33	-5.79
729.83	-2.51	-2.84	-3.18	-3.51	-3.94	-4.18	-4.49	-4.86	-5.01	-5.22	-5.57
786.16	-2.45	-2.74	-3.10	-3.46	-3.84	-4.24	-4.45	-4.80	-5.02	-5.34	-5.57
846.90	-2.62	-2.92	-3.27	-3.60	-4.06	-4.27	-4.60	-4.92	-5.06	-5.33	-5.67
912.42	-2.70	-2.98	-3.23	-3.63	-4.01	-4.24	-4.60	-4.92	-5.12	-5.36	-5.76
983.08	-2.52	-2.95	-3.20	-3.59	-3.84	-4.17	-4.56	-4.77	-5.16	-5.37	-5.67

1059.29	-2.57	-2.93	-3.24	-3.65	-3.89	-4.17	-4.55	-4.76	-5.06	-5.38	-5.64
1141.48	-2.70	-3.08	-3.33	-3.66	-4.01	-4.33	-4.67	-4.85	-5.10	-5.45	-5.73
1230.13	-2.58	-2.89	-3.16	-3.62	-4.00	-4.19	-4.47	-4.80	-5.04	-5.32	-5.56
1325.73	-2.55	-2.91	-3.22	-3.67	-3.93	-4.16	-4.53	-4.73	-5.06	-5.33	-5.65
1428.84	-2.69	-2.98	-3.42	-3.80	-4.10	-4.24	-4.80	-4.93	-5.22	-5.45	-5.74
1540.05	-2.74	-3.12	-3.52	-3.77	-4.14	-4.44	-4.61	-5.00	-5.29	-5.43	-5.69
1659.99	-2.77	-3.08	-3.49	-3.80	-4.06	-4.37	-4.62	-4.92	-5.25	-5.42	-5.73
1789.34	-2.79	-3.20	-3.52	-4.00	-4.15	-4.47	-4.82	-5.12	-5.30	-5.56	-5.80
1928.85	-2.72	-3.07	-3.43	-3.82	-4.02	-4.44	-4.73	-5.09	-5.26	-5.48	-5.77
2079.31	-2.83	-3.17	-3.61	-3.93	-4.27	-4.41	-4.73	-5.12	-5.27	-5.57	-5.86
2241.59	-2.84	-3.32	-3.56	-3.97	-4.30	-4.48	-4.81	-5.09	-5.20	-5.62	-5.82
2416.60	-2.71	-3.06	-3.49	-3.92	-4.20	-4.41	-4.86	-5.09	-5.19	-5.57	-5.86
2605.36	-2.81	-3.14	-3.62	-3.85	-4.13	-4.47	-4.84	-5.07	-5.30	-5.60	-5.82
2808.94	-2.89	-3.36	-3.71	-4.02	-4.25	-4.61	-4.95	-5.04	-5.42	-5.66	-5.91
3028.50	-2.79	-3.12	-3.48	-3.89	-4.26	-4.58	-4.87	-5.08	-5.25	-5.59	-5.86
3265.30	-2.80	-3.13	-3.47	-3.75	-4.19	-4.49	-4.78	-5.05	-5.25	-5.50	-5.74
3520.69	-2.72	-3.07	-3.40	-3.80	-4.00	-4.32	-4.59	-4.86	-5.13	-5.42	-5.67
3796.13	-2.61	-3.05	-3.37	-3.73	-3.94	-4.28	-4.56	-4.76	-5.08	-5.28	-5.58

Wavelength (nm) / Time (ps)	475.07	476.49	477.92	479.34	480.77	482.19	483.61	485.04	486.46	487.89	489.31
-3.78	0.10	0.10	0.07	0.10	0.08	0.06	0.04	0.11	0.15	0.09	0.02
-3.28	0.15	0.01	0.11	0.07	0.02	0.07	0.04	0.11	0.04	0.09	0.08
-2.78	-0.05	-0.09	0.01	-0.06	-0.09	-0.04	0.02	-0.06	-0.08	-0.07	-0.10
-2.28	-0.06	0.01	0.02	-0.09	0.07	-0.03	-0.05	0.04	0.07	0.01	0.01
-1.78	0.02	0.03	-0.01	0.05	-0.01	0.05	0.02	-0.02	-0.05	-0.03	0.00
-1.28	-0.01	-0.08	-0.16	-0.19	-0.06	-0.12	0.00	-0.13	-0.16	-0.09	-0.01
-0.78	-0.15	-0.03	-0.11	0.07	0.01	-0.02	-0.07	-0.04	0.01	0.00	-0.01
-0.28	0.01	0.05	0.07	0.04	-0.02	0.03	0.00	-0.01	0.02	0.00	0.01
-0.18	0.00	0.01	0.05	0.04	0.03	-0.07	0.10	0.08	0.03	0.05	0.01
-0.08	0.03	0.01	0.02	0.04	0.05	0.08	0.05	0.08	0.16	0.03	0.12
0.02	0.08	0.05	0.05	0.07	0.01	-0.08	0.03	0.12	0.07	-0.04	-0.04
0.12	0.10	0.11	0.05	0.06	0.03	-0.05	0.00	0.02	0.07	0.09	0.12
0.22	-0.05	-0.10	-0.14	-0.13	-0.04	-0.11	-0.09	-0.07	-0.01	-0.11	0.01
0.27	0.01	-0.04	0.04	0.13	0.04	0.00	-0.03	0.09	-0.01	-0.06	-0.07
0.32	0.11	0.10	0.09	0.13	0.24	0.10	0.16	0.12	0.15	0.14	0.13
0.37	-0.02	-0.04	0.00	-0.05	0.00	-0.09	-0.04	0.01	-0.09	-0.06	-0.05
0.42	-0.07	-0.05	-0.02	-0.09	-0.08	-0.08	-0.13	-0.11	-0.17	-0.21	-0.08
0.47	-0.08	-0.03	-0.04	-0.11	-0.09	-0.15	-0.10	-0.14	-0.06	-0.06	-0.05
0.52	0.04	0.01	-0.02	0.04	0.10	-0.03	-0.03	0.01	0.01	-0.05	-0.04
0.57	0.15	0.14	0.14	0.02	0.13	0.03	0.10	0.12	0.10	0.10	0.13
0.62	0.03	-0.02	0.05	-0.01	-0.06	-0.03	-0.02	-0.04	0.01	0.01	-0.02
0.67	-0.02	-0.14	-0.02	-0.09	-0.04	-0.08	-0.06	-0.06	-0.04	-0.05	-0.20
0.72	-0.04	0.02	0.01	0.03	-0.10	-0.09	-0.11	-0.08	-0.06	-0.04	-0.09
0.77	-0.07	-0.01	-0.04	-0.09	-0.12	-0.10	-0.08	-0.08	-0.02	-0.04	-0.09
0.82	0.01	-0.01	-0.03	0.00	0.01	-0.11	0.02	0.00	0.07	-0.06	0.01
0.87	-0.11	-0.02	-0.06	0.05	-0.02	-0.05	-0.01	-0.01	-0.01	-0.12	-0.07
0.92	-0.05	-0.02	0.00	0.01	-0.02	0.02	-0.08	-0.03	0.03	-0.03	0.04

0.97	0.04	0.02	0.08	0.06	-0.04	-0.08	0.04	-0.01	0.00	-0.04	-0.05
1.02	-0.01	-0.07	0.02	-0.12	0.05	0.02	-0.01	0.05	-0.07	0.02	-0.06
1.07	0.16	0.05	-0.04	-0.02	0.14	-0.03	-0.12	0.00	0.05	0.04	0.08
1.12	-29.93	-21.84	-13.84	-6.85	-1.85	0.75	1.54	0.89	-0.27	-1.10	-1.40
1.17	72.76	52.27	33.63	17.24	3.84	-7.14	-16.04	-24.05	-30.78	-36.19	-40.13
1.22	-12.57	9.87	32.21	53.14	72.90	90.64	105.96	117.38	125.05	128.15	126.16
1.27	-4.84	-4.44	-6.33	-12.16	-21.72	-34.71	-49.33	-63.83	-76.09	-84.43	-87.70
1.32	-10.96	-11.61	-11.51	-10.93	-10.02	-8.78	-7.26	-6.30	-6.10	-7.26	-9.55
1.37	-6.42	-6.64	-7.30	-8.51	-10.05	-11.39	-12.36	-13.01	-13.38	-13.48	-13.47
1.42	-8.41	-8.56	-8.46	-8.58	-9.26	-10.31	-11.65	-13.22	-14.48	-15.55	-16.09
1.47	-7.68	-9.37	-10.87	-11.81	-12.04	-11.97	-11.68	-11.44	-11.54	-12.09	-12.94
1.52	-7.24	-7.64	-8.42	-9.57	-11.00	-12.10	-13.01	-13.44	-13.29	-13.08	-12.70
1.57	-9.43	-9.56	-9.32	-9.01	-8.88	-9.34	-10.14	-11.47	-12.89	-14.19	-15.07
1.62	-7.13	-8.68	-10.07	-11.06	-11.61	-11.62	-11.41	-11.13	-11.09	-11.60	-12.35
1.67	-7.47	-7.52	-8.20	-9.29	-10.55	-11.83	-12.80	-13.54	-13.73	-13.70	-13.33
1.72	-9.08	-9.90	-10.29	-10.40	-10.24	-10.11	-10.26	-10.63	-11.49	-12.48	-13.43
1.77	-6.94	-7.68	-8.65	-9.79	-10.90	-11.88	-12.20	-12.26	-12.13	-12.03	-12.09
1.82	-8.29	-8.14	-8.16	-8.38	-8.99	-10.01	-11.22	-12.30	-13.06	-13.64	-13.83
1.87	-8.32	-9.40	-10.22	-10.52	-10.75	-10.66	-10.69	-11.02	-11.48	-12.31	-13.11
1.92	-7.17	-7.53	-8.24	-9.26	-10.30	-11.40	-12.16	-12.52	-12.63	-12.42	-12.25
1.97	-8.55	-8.83	-8.78	-8.88	-8.99	-9.43	-10.18	-11.00	-12.03	-12.92	-13.59
2.02	-7.19	-8.03	-9.00	-9.89	-10.54	-11.05	-11.21	-11.33	-11.25	-11.53	-11.93
2.07	-7.26	-7.51	-8.18	-9.15	-10.18	-11.22	-11.90	-12.37	-12.49	-12.56	-12.38
2.12	-8.49	-9.14	-9.47	-9.73	-9.67	-9.89	-10.22	-10.80	-11.35	-12.08	-12.78
2.17	-7.12	-7.98	-8.95	-9.74	-10.46	-10.92	-11.01	-11.21	-11.23	-11.49	-11.66
2.22	-7.85	-8.18	-8.33	-8.66	-9.22	-10.03	-10.83	-11.74	-12.40	-12.90	-13.04
2.27	-7.95	-8.80	-9.51	-9.96	-10.13	-10.34	-10.37	-10.68	-11.08	-11.72	-12.33
2.32	-6.95	-7.61	-8.22	-9.06	-9.90	-10.74	-11.23	-11.52	-11.69	-11.73	-11.62
2.37	-8.21	-8.56	-8.84	-8.90	-9.24	-9.80	-10.50	-11.14	-11.87	-12.45	-12.88
2.42	-7.73	-8.57	-9.36	-9.92	-10.22	-10.43	-10.64	-10.84	-11.17	-11.65	-12.13
2.47	-7.24	-7.66	-8.26	-9.06	-9.74	-10.50	-11.15	-11.65	-11.85	-12.05	-12.08
2.52	-8.15	-8.71	-9.11	-9.39	-9.54	-9.88	-10.28	-10.75	-11.46	-12.17	-12.69
2.57	-7.34	-8.06	-8.78	-9.55	-10.15	-10.76	-11.02	-11.18	-11.27	-11.54	-11.89
2.62	-7.75	-7.93	-8.29	-8.85	-9.45	-10.27	-10.97	-11.54	-11.98	-12.26	-12.33
2.67	-7.98	-8.54	-8.93	-9.24	-9.58	-9.97	-10.08	-10.61	-11.10	-11.70	-12.09
2.72	-7.07	-7.58	-8.29	-9.09	-9.82	-10.46	-10.93	-11.13	-11.37	-11.47	-11.65
2.77	-7.81	-8.15	-8.53	-8.87	-9.16	-9.80	-10.39	-11.01	-11.69	-12.04	-12.35
2.82	-7.55	-8.35	-8.92	-9.43	-9.77	-10.21	-10.39	-10.66	-11.00	-11.37	-11.80
2.87	-7.37	-7.66	-8.26	-8.81	-9.52	-10.10	-10.64	-10.96	-11.34	-11.59	-11.85
2.92	-7.94	-8.40	-8.76	-9.09	-9.30	-9.69	-10.13	-10.68	-11.18	-11.79	-12.21
2.97	-7.21	-7.82	-8.56	-9.20	-9.85	-10.24	-10.56	-10.87	-10.93	-11.23	-11.46
3.02	-7.44	-7.77	-8.17	-8.70	-9.26	-9.86	-10.52	-11.06	-11.33	-11.65	-11.90
3.07	-7.77	-8.34	-8.76	-9.22	-9.46	-9.92	-10.34	-10.71	-11.06	-11.50	-11.99
3.12	-7.18	-7.79	-8.41	-9.04	-9.73	-10.17	-10.47	-10.78	-10.97	-11.24	-11.41
3.17	-7.65	-8.07	-8.40	-8.82	-9.21	-9.91	-10.28	-10.85	-11.28	-11.66	-11.89
3.22	-7.48	-8.13	-8.86	-9.37	-9.72	-10.06	-10.31	-10.59	-10.91	-11.27	-11.66
3.42	-7.39	-7.93	-8.33	-8.88	-9.45	-10.03	-10.57	-10.95	-11.37	-11.50	-11.82
3.62	-7.68	-8.21	-8.65	-9.09	-9.60	-10.01	-10.29	-10.64	-11.11	-11.51	-11.88
3.82	-7.37	-7.82	-8.26	-8.91	-9.41	-9.91	-10.44	-10.84	-11.20	-11.55	-11.66
4.02	-7.52	-7.95	-8.62	-8.97	-9.42	-9.90	-10.14	-10.62	-10.98	-11.36	-11.60

4.22	-7.30	-7.79	-8.22	-8.84	-9.37	-9.88	-10.35	-10.73	-10.96	-11.34	-11.46
4.42	-7.45	-7.89	-8.42	-8.87	-9.28	-9.77	-10.11	-10.46	-10.93	-11.16	-11.51
4.62	-7.26	-7.81	-8.37	-8.82	-9.38	-9.86	-10.25	-10.70	-10.93	-11.26	-11.46
4.82	-7.45	-7.79	-8.38	-8.75	-9.21	-9.60	-10.05	-10.41	-10.77	-11.15	-11.32
5.02	-7.50	-7.94	-8.42	-8.96	-9.37	-9.91	-10.28	-10.54	-10.85	-11.18	-11.38
5.22	-7.23	-7.81	-8.30	-8.79	-9.14	-9.63	-10.02	-10.39	-10.78	-11.20	-11.40
5.42	-7.30	-7.85	-8.30	-8.79	-9.23	-9.85	-10.19	-10.51	-10.84	-11.03	-11.29
5.62	-7.26	-7.78	-8.19	-8.68	-9.04	-9.64	-10.01	-10.34	-10.66	-11.00	-11.22
5.82	-7.27	-7.85	-8.30	-8.78	-9.15	-9.63	-10.01	-10.46	-10.74	-10.96	-11.23
6.02	-7.28	-7.71	-8.13	-8.54	-9.02	-9.47	-9.89	-10.29	-10.51	-10.89	-11.12
6.22	-7.28	-7.79	-8.31	-8.76	-9.13	-9.69	-10.05	-10.32	-10.58	-11.00	-11.17
6.42	-7.27	-7.79	-8.23	-8.70	-9.10	-9.60	-10.00	-10.32	-10.65	-10.91	-11.19
6.62	-7.21	-7.77	-8.20	-8.64	-9.14	-9.56	-9.92	-10.27	-10.56	-10.88	-11.08
6.82	-7.30	-7.81	-8.18	-8.70	-9.19	-9.53	-9.87	-10.19	-10.50	-10.89	-11.07
7.02	-7.19	-7.68	-8.18	-8.55	-9.04	-9.48	-9.77	-10.17	-10.45	-10.80	-10.93
7.22	-7.08	-7.60	-7.94	-8.53	-8.93	-9.44	-9.83	-10.08	-10.37	-10.69	-10.87
7.42	-7.18	-7.69	-8.11	-8.70	-9.10	-9.50	-9.91	-10.20	-10.53	-10.85	-11.01
7.62	-7.21	-7.57	-8.09	-8.54	-8.91	-9.42	-9.86	-10.05	-10.42	-10.69	-10.93
7.82	-7.09	-7.61	-8.01	-8.49	-8.88	-9.42	-9.74	-9.95	-10.30	-10.49	-10.75
8.02	-7.15	-7.64	-8.03	-8.58	-8.99	-9.33	-9.81	-10.11	-10.40	-10.57	-10.80
8.22	-7.05	-7.45	-7.89	-8.44	-8.74	-9.26	-9.57	-9.86	-10.14	-10.39	-10.54
8.42	-7.23	-7.68	-8.07	-8.54	-8.96	-9.48	-9.67	-10.09	-10.35	-10.61	-10.76
8.62	-7.03	-7.52	-7.96	-8.39	-8.74	-9.22	-9.55	-9.81	-10.16	-10.39	-10.50
8.82	-7.07	-7.49	-8.00	-8.43	-8.77	-9.27	-9.64	-9.86	-10.15	-10.47	-10.70
9.02	-7.07	-7.47	-7.91	-8.39	-8.73	-9.26	-9.50	-9.86	-10.12	-10.41	-10.54
9.22	-7.09	-7.47	-7.99	-8.35	-8.86	-9.34	-9.59	-9.93	-10.23	-10.54	-10.62
9.42	-7.03	-7.52	-7.83	-8.37	-8.70	-9.18	-9.52	-9.78	-10.09	-10.41	-10.63
9.62	-7.08	-7.46	-7.92	-8.33	-8.81	-9.25	-9.58	-9.93	-10.22	-10.45	-10.56
9.82	-7.04	-7.42	-7.78	-8.21	-8.69	-9.06	-9.44	-9.60	-10.00	-10.32	-10.50
10.02	-6.92	-7.50	-7.84	-8.36	-8.72	-9.19	-9.50	-9.84	-10.02	-10.38	-10.49
10.22	-7.06	-7.58	-7.97	-8.40	-8.77	-9.18	-9.54	-9.80	-10.19	-10.45	-10.47
10.42	-6.84	-7.36	-7.78	-8.25	-8.75	-9.09	-9.40	-9.81	-10.01	-10.28	-10.58
10.62	-6.95	-7.38	-7.88	-8.16	-8.54	-9.17	-9.48	-9.80	-10.02	-10.40	-10.54
10.82	-6.88	-7.38	-7.83	-8.25	-8.66	-9.01	-9.38	-9.72	-10.00	-10.23	-10.47
11.02	-6.87	-7.26	-7.81	-8.19	-8.68	-9.04	-9.38	-9.69	-9.96	-10.19	-10.33
11.22	-6.89	-7.42	-7.81	-8.13	-8.58	-9.09	-9.37	-9.73	-10.01	-10.21	-10.32
11.42	-6.89	-7.31	-7.71	-8.14	-8.49	-8.95	-9.32	-9.50	-9.79	-10.11	-10.18
11.62	-6.81	-7.28	-7.87	-8.21	-8.56	-9.04	-9.31	-9.63	-9.97	-10.20	-10.33
11.82	-6.97	-7.39	-7.85	-8.32	-8.57	-9.04	-9.41	-9.73	-9.96	-10.20	-10.40
12.02	-6.74	-7.31	-7.72	-8.26	-8.54	-9.01	-9.28	-9.54	-9.80	-10.09	-10.26
12.22	-7.00	-7.35	-7.70	-8.20	-8.64	-9.13	-9.29	-9.67	-10.03	-10.27	-10.46
12.42	-6.93	-7.40	-7.69	-8.17	-8.49	-9.00	-9.27	-9.55	-9.80	-10.08	-10.27
12.62	-6.79	-7.28	-7.77	-8.06	-8.47	-8.90	-9.29	-9.66	-9.81	-10.03	-10.23
12.82	-6.82	-7.22	-7.67	-8.13	-8.36	-8.82	-9.20	-9.35	-9.67	-9.92	-10.01
13.02	-6.86	-7.33	-7.77	-8.17	-8.62	-8.99	-9.33	-9.57	-9.85	-10.07	-10.30
13.22	-6.76	-7.37	-7.60	-8.11	-8.54	-8.91	-9.21	-9.54	-9.78	-10.03	-10.25
13.42	-6.89	-7.35	-7.70	-8.22	-8.45	-9.03	-9.27	-9.58	-9.75	-10.10	-10.32
13.50	-6.91	-7.26	-7.61	-8.11	-8.53	-8.95	-9.28	-9.49	-9.73	-9.89	-9.97
13.59	-6.83	-7.26	-7.73	-8.09	-8.50	-8.87	-9.25	-9.55	-9.86	-10.09	-10.20
13.68	-6.86	-7.27	-7.69	-8.15	-8.46	-8.94	-9.16	-9.55	-9.76	-10.07	-10.05

13.78	-6.89	-7.32	-7.67	-8.04	-8.56	-8.93	-9.22	-9.48	-9.75	-10.00	-10.09
13.88	-6.78	-7.27	-7.72	-8.11	-8.48	-8.95	-9.21	-9.56	-9.77	-10.03	-10.13
14.00	-6.88	-7.29	-7.71	-8.10	-8.49	-9.01	-9.29	-9.55	-9.81	-10.08	-10.21
14.12	-7.01	-7.44	-7.78	-8.23	-8.61	-8.99	-9.35	-9.57	-9.87	-10.13	-10.30
14.25	-6.92	-7.22	-7.61	-8.12	-8.49	-8.83	-9.21	-9.43	-9.72	-9.97	-10.12
14.40	-6.98	-7.44	-7.92	-8.29	-8.60	-9.04	-9.40	-9.67	-9.92	-10.19	-10.40
14.55	-6.82	-7.29	-7.70	-8.20	-8.48	-8.86	-9.16	-9.55	-9.84	-9.93	-10.22
14.72	-6.82	-7.17	-7.54	-8.12	-8.42	-8.83	-9.11	-9.40	-9.65	-9.98	-10.10
14.90	-6.74	-7.05	-7.53	-7.90	-8.31	-8.80	-9.01	-9.27	-9.55	-9.75	-9.99
15.09	-6.82	-7.24	-7.66	-8.01	-8.39	-8.82	-9.17	-9.36	-9.58	-9.88	-10.02
15.30	-6.78	-7.19	-7.51	-8.02	-8.34	-8.80	-9.07	-9.35	-9.61	-9.86	-10.00
15.53	-7.05	-7.41	-7.80	-8.28	-8.66	-9.02	-9.31	-9.67	-9.90	-10.13	-10.33
15.77	-6.88	-7.31	-7.70	-8.11	-8.35	-8.88	-9.15	-9.53	-9.70	-9.88	-10.08
16.04	-6.87	-7.16	-7.65	-8.14	-8.41	-8.98	-9.26	-9.49	-9.76	-10.04	-10.18
16.32	-6.84	-7.19	-7.64	-8.12	-8.44	-8.87	-9.05	-9.36	-9.63	-9.85	-10.06
16.63	-6.82	-7.32	-7.77	-8.15	-8.44	-8.94	-9.24	-9.52	-9.77	-9.92	-10.24
16.96	-6.85	-7.29	-7.74	-8.16	-8.49	-8.96	-9.14	-9.47	-9.71	-9.93	-9.97
17.31	-6.83	-7.20	-7.68	-8.06	-8.40	-8.86	-9.24	-9.46	-9.77	-9.99	-10.03
17.70	-6.87	-7.32	-7.73	-8.12	-8.53	-8.89	-9.17	-9.51	-9.74	-10.02	-10.10
18.11	-6.91	-7.19	-7.64	-8.00	-8.32	-8.72	-9.09	-9.33	-9.61	-9.86	-10.06
18.56	-6.80	-7.20	-7.52	-8.04	-8.39	-8.75	-9.06	-9.37	-9.57	-9.83	-9.97
19.04	-6.80	-7.26	-7.63	-8.05	-8.35	-8.79	-9.05	-9.24	-9.69	-9.81	-9.97
19.56	-6.63	-7.07	-7.50	-7.86	-8.27	-8.64	-9.01	-9.33	-9.52	-9.76	-9.98
20.12	-6.89	-7.30	-7.75	-8.10	-8.47	-8.89	-9.17	-9.58	-9.72	-9.98	-10.20
20.72	-6.73	-7.17	-7.64	-8.00	-8.41	-8.81	-9.18	-9.41	-9.65	-9.91	-10.05
21.37	-6.81	-7.15	-7.52	-7.94	-8.36	-8.67	-9.11	-9.24	-9.57	-9.87	-10.05
22.08	-6.84	-7.20	-7.62	-7.95	-8.33	-8.81	-9.12	-9.42	-9.73	-10.06	-10.16
22.84	-6.74	-7.30	-7.69	-8.09	-8.39	-8.75	-9.12	-9.42	-9.73	-9.98	-10.12
23.65	-6.70	-7.20	-7.58	-8.02	-8.33	-8.77	-9.14	-9.38	-9.63	-9.78	-9.94
24.54	-6.78	-7.20	-7.59	-8.00	-8.35	-8.78	-9.11	-9.35	-9.54	-9.77	-10.00
25.49	-6.89	-7.30	-7.57	-8.12	-8.49	-8.91	-9.13	-9.46	-9.68	-9.89	-10.17
26.51	-6.83	-7.21	-7.58	-7.95	-8.35	-8.79	-9.05	-9.28	-9.47	-9.68	-9.83
27.62	-6.89	-7.22	-7.67	-8.07	-8.46	-8.91	-9.07	-9.41	-9.68	-9.93	-10.02
28.81	-6.80	-7.13	-7.52	-7.87	-8.26	-8.74	-9.07	-9.31	-9.53	-9.85	-10.01
30.10	-6.85	-7.20	-7.58	-8.02	-8.34	-8.67	-9.00	-9.28	-9.52	-9.69	-9.89
31.49	-6.77	-7.06	-7.48	-7.84	-8.20	-8.64	-8.96	-9.22	-9.40	-9.68	-9.83
32.98	-6.70	-7.10	-7.54	-7.84	-8.20	-8.70	-8.90	-9.18	-9.42	-9.64	-9.78
34.60	-6.73	-7.19	-7.64	-7.96	-8.22	-8.72	-9.02	-9.31	-9.49	-9.69	-9.87
36.34	-6.83	-7.24	-7.62	-8.02	-8.40	-8.78	-9.07	-9.34	-9.59	-9.76	-9.82
38.21	-6.69	-7.06	-7.48	-7.84	-8.20	-8.61	-8.83	-9.02	-9.25	-9.51	-9.60
40.24	-6.65	-7.16	-7.64	-7.99	-8.24	-8.67	-8.96	-9.20	-9.46	-9.71	-9.89
42.42	-6.78	-7.08	-7.53	-7.82	-8.14	-8.62	-8.90	-9.15	-9.32	-9.56	-9.65
44.78	-6.62	-7.09	-7.42	-7.87	-8.12	-8.61	-8.85	-9.13	-9.30	-9.59	-9.61
47.32	-6.70	-7.18	-7.51	-7.84	-8.20	-8.61	-8.82	-9.05	-9.25	-9.51	-9.59
50.06	-6.74	-7.05	-7.42	-7.84	-8.17	-8.67	-8.81	-9.12	-9.38	-9.60	-9.72
53.01	-6.73	-7.11	-7.61	-7.94	-8.26	-8.62	-8.94	-9.28	-9.48	-9.70	-9.73
56.20	-6.73	-7.20	-7.50	-7.84	-8.33	-8.70	-8.95	-9.23	-9.42	-9.71	-9.78
59.64	-6.75	-7.12	-7.47	-7.86	-8.15	-8.57	-8.85	-9.08	-9.30	-9.63	-9.72
63.34	-6.59	-6.96	-7.35	-7.73	-8.13	-8.50	-8.78	-9.02	-9.16	-9.46	-9.48
67.34	-6.58	-7.05	-7.41	-7.70	-8.09	-8.48	-8.78	-9.07	-9.25	-9.45	-9.54

71.65	-6.84	-7.15	-7.49	-7.85	-8.19	-8.58	-8.86	-9.14	-9.30	-9.53	-9.72
76.30	-6.62	-7.08	-7.38	-7.82	-8.13	-8.52	-8.79	-9.10	-9.23	-9.52	-9.74
81.32	-6.74	-7.10	-7.55	-7.93	-8.21	-8.60	-8.94	-9.15	-9.36	-9.70	-9.72
86.73	-6.70	-7.12	-7.42	-7.86	-8.18	-8.62	-8.81	-9.09	-9.38	-9.59	-9.76
92.56	-6.65	-6.96	-7.38	-7.70	-7.96	-8.56	-8.74	-9.02	-9.27	-9.49	-9.62
98.85	-6.70	-6.90	-7.44	-7.91	-8.19	-8.44	-8.78	-9.06	-9.22	-9.43	-9.58
105.64	-6.65	-6.97	-7.35	-7.75	-8.15	-8.41	-8.69	-8.93	-9.17	-9.42	-9.56
112.96	-6.70	-6.98	-7.46	-7.85	-8.00	-8.46	-8.83	-9.11	-9.38	-9.47	-9.63
120.85	-6.69	-7.06	-7.31	-7.68	-7.97	-8.37	-8.64	-8.92	-9.13	-9.38	-9.55
129.36	-6.75	-7.07	-7.53	-7.88	-8.09	-8.53	-8.82	-9.11	-9.33	-9.61	-9.76
138.55	-6.58	-6.89	-7.28	-7.68	-8.08	-8.36	-8.71	-8.98	-9.12	-9.42	-9.55
148.45	-6.59	-6.95	-7.34	-7.78	-8.07	-8.43	-8.72	-9.00	-9.29	-9.46	-9.51
159.13	-6.57	-6.95	-7.36	-7.69	-8.06	-8.42	-8.72	-8.95	-9.14	-9.30	-9.55
170.65	-6.73	-7.01	-7.44	-7.71	-8.03	-8.45	-8.77	-9.00	-9.18	-9.48	-9.57
183.07	-6.54	-6.93	-7.32	-7.59	-7.90	-8.34	-8.62	-8.85	-9.10	-9.33	-9.40
196.47	-6.55	-6.89	-7.30	-7.57	-7.97	-8.39	-8.61	-8.86	-9.09	-9.27	-9.42
210.91	-6.59	-6.99	-7.20	-7.62	-7.93	-8.27	-8.57	-8.82	-9.02	-9.22	-9.31
226.50	-6.37	-6.79	-7.17	-7.40	-7.73	-8.20	-8.45	-8.69	-8.90	-9.12	-9.30
243.31	-6.40	-6.76	-7.05	-7.37	-7.69	-8.06	-8.36	-8.58	-8.85	-9.05	-9.24
261.43	-6.48	-6.87	-7.20	-7.54	-7.83	-8.27	-8.53	-8.78	-8.97	-9.20	-9.30
280.98	-6.33	-6.73	-7.10	-7.43	-7.71	-8.15	-8.40	-8.59	-8.82	-9.08	-9.12
302.07	-6.23	-6.59	-6.90	-7.28	-7.57	-7.93	-8.22	-8.42	-8.73	-8.91	-9.06
324.81	-6.39	-6.76	-7.05	-7.41	-7.70	-7.99	-8.30	-8.57	-8.69	-8.97	-9.12
349.33	-6.19	-6.59	-6.91	-7.23	-7.49	-7.79	-8.13	-8.27	-8.54	-8.86	-8.99
375.79	-6.28	-6.61	-6.86	-7.28	-7.46	-7.92	-8.24	-8.48	-8.69	-8.87	-9.02
404.31	-6.16	-6.50	-6.92	-7.15	-7.47	-7.82	-8.15	-8.31	-8.51	-8.76	-8.93
435.08	-6.23	-6.53	-6.95	-7.31	-7.58	-7.98	-8.16	-8.46	-8.72	-8.85	-9.01
468.27	-6.10	-6.48	-6.78	-7.11	-7.47	-7.77	-7.97	-8.32	-8.49	-8.73	-8.84
504.06	-6.07	-6.44	-6.76	-7.04	-7.46	-7.81	-8.02	-8.22	-8.48	-8.65	-8.79
542.66	-6.02	-6.42	-6.79	-7.04	-7.40	-7.75	-7.82	-8.21	-8.41	-8.65	-8.74
584.29	-6.03	-6.37	-6.58	-6.97	-7.30	-7.75	-7.92	-8.23	-8.39	-8.59	-8.71
629.18	-5.93	-6.27	-6.60	-6.91	-7.14	-7.55	-7.67	-8.04	-8.22	-8.41	-8.60
677.61	-5.96	-6.42	-6.67	-6.96	-7.29	-7.60	-7.95	-8.24	-8.31	-8.58	-8.74
729.83	-5.83	-6.21	-6.44	-6.79	-7.12	-7.39	-7.61	-7.93	-8.07	-8.28	-8.40
786.16	-5.93	-6.21	-6.53	-6.81	-7.04	-7.39	-7.64	-7.91	-8.13	-8.31	-8.54
846.90	-5.90	-6.32	-6.50	-6.79	-7.08	-7.49	-7.73	-7.92	-8.13	-8.46	-8.57
912.42	-5.88	-6.27	-6.53	-6.75	-7.08	-7.48	-7.56	-7.81	-8.08	-8.34	-8.46
983.08	-5.90	-6.16	-6.46	-6.78	-7.07	-7.42	-7.64	-7.87	-8.10	-8.33	-8.54
1059.29	-5.89	-6.12	-6.46	-6.79	-7.08	-7.41	-7.51	-7.86	-8.18	-8.46	-8.52
1141.48	-5.91	-6.23	-6.46	-6.79	-7.06	-7.42	-7.69	-7.82	-8.12	-8.30	-8.45
1230.13	-5.68	-6.16	-6.37	-6.83	-7.02	-7.29	-7.56	-7.85	-8.16	-8.35	-8.46
1325.73	-5.96	-6.18	-6.61	-6.76	-7.10	-7.49	-7.70	-7.95	-8.20	-8.46	-8.61
1428.84	-5.97	-6.22	-6.56	-6.75	-7.13	-7.42	-7.63	-7.94	-8.25	-8.42	-8.52
1540.05	-6.07	-6.33	-6.67	-6.97	-7.27	-7.55	-7.93	-8.14	-8.31	-8.61	-8.77
1659.99	-6.05	-6.31	-6.52	-6.92	-7.15	-7.49	-7.78	-8.01	-8.27	-8.53	-8.64
1789.34	-6.12	-6.33	-6.67	-7.00	-7.22	-7.48	-7.77	-8.02	-8.22	-8.49	-8.72
1928.85	-6.00	-6.35	-6.62	-6.88	-7.08	-7.47	-7.73	-8.03	-8.21	-8.44	-8.66
2079.31	-6.06	-6.35	-6.66	-6.85	-7.21	-7.53	-7.80	-8.14	-8.42	-8.59	-8.85
2241.59	-6.06	-6.45	-6.65	-7.00	-7.32	-7.63	-7.80	-8.08	-8.34	-8.67	-8.82
2416.60	-5.99	-6.40	-6.66	-6.85	-7.15	-7.57	-7.82	-8.10	-8.42	-8.79	-8.96

2605.36	-6.16	-6.43	-6.72	-7.05	-7.20	-7.60	-7.80	-8.10	-8.33	-8.69	-8.93
2808.94	-6.13	-6.41	-6.74	-6.97	-7.16	-7.51	-7.81	-8.04	-8.32	-8.73	-8.91
3028.50	-6.09	-6.36	-6.59	-6.77	-7.12	-7.51	-7.69	-7.96	-8.18	-8.49	-8.93
3265.30	-6.09	-6.30	-6.69	-6.87	-7.15	-7.45	-7.64	-7.97	-8.27	-8.60	-8.85
3520.69	-5.87	-6.17	-6.47	-6.73	-7.03	-7.35	-7.59	-7.86	-8.07	-8.46	-8.80
3796.13	-5.80	-6.04	-6.31	-6.56	-6.93	-7.15	-7.42	-7.85	-8.06	-8.40	-8.64

Wavelength (nm) / Time (ps)	490.74	492.16	493.58	495.01	496.43	497.86	499.28	500.70	502.13	503.55	504.98
-3.78	0.11	0.11	0.07	0.18	0.07	0.03	0.08	0.05	0.14	0.07	0.16
-3.28	0.11	0.10	0.03	0.06	0.12	0.04	0.05	0.09	0.08	0.12	0.10
-2.78	-0.10	-0.13	-0.10	-0.01	-0.11	-0.10	-0.17	-0.19	-0.16	-0.12	-0.11
-2.28	0.08	0.00	0.15	-0.02	0.06	-0.03	0.04	0.07	0.02	0.02	0.01
-1.78	-0.09	-0.03	0.01	-0.06	-0.04	0.02	0.05	-0.01	0.02	-0.03	-0.09
-1.28	-0.09	-0.03	-0.10	-0.06	-0.14	-0.04	-0.01	0.02	-0.04	-0.02	-0.10
-0.78	0.01	0.02	-0.03	-0.08	0.02	0.05	0.00	0.00	-0.03	-0.04	0.03
-0.28	-0.02	-0.04	-0.03	-0.01	0.03	0.03	-0.04	-0.03	-0.02	0.01	0.00
-0.18	0.03	0.02	0.06	0.08	0.02	0.08	-0.01	0.01	0.06	0.04	0.00
-0.08	0.11	0.11	0.07	0.15	0.11	0.11	0.04	0.05	0.06	0.09	0.05
0.02	0.02	-0.06	-0.01	0.09	-0.03	-0.04	-0.11	0.06	-0.08	-0.05	-0.01
0.12	0.01	0.10	-0.02	0.08	0.05	0.10	-0.05	0.03	0.09	0.02	0.01
0.22	-0.04	-0.02	-0.09	0.05	-0.03	0.00	0.03	-0.04	-0.02	0.07	0.00
0.27	-0.03	-0.08	-0.07	-0.06	0.06	-0.02	-0.08	-0.12	0.01	-0.02	0.01
0.32	0.03	0.15	0.07	0.09	0.14	0.17	0.06	0.02	0.12	0.16	0.07
0.37	-0.05	-0.04	0.00	-0.10	-0.04	-0.02	-0.10	-0.07	-0.09	-0.08	-0.07
0.42	-0.09	-0.09	-0.21	-0.17	-0.20	-0.14	-0.24	-0.18	-0.18	-0.20	-0.18
0.47	-0.06	-0.11	-0.02	-0.05	-0.10	-0.02	-0.12	-0.09	0.01	-0.05	-0.09
0.52	-0.03	-0.10	-0.15	-0.03	-0.04	-0.01	-0.10	-0.06	0.01	-0.14	-0.10
0.57	0.10	0.08	0.15	-0.03	0.00	-0.04	0.06	-0.06	-0.03	-0.05	0.00
0.62	0.04	0.03	0.02	0.02	-0.03	0.02	0.06	0.05	0.04	0.01	0.04
0.67	-0.17	-0.14	-0.11	-0.01	-0.14	-0.08	-0.12	-0.07	-0.10	-0.16	-0.11
0.72	-0.06	-0.11	-0.10	-0.04	-0.16	-0.03	-0.14	-0.19	-0.18	-0.17	-0.16
0.77	-0.04	-0.05	-0.12	-0.01	-0.04	-0.02	-0.16	-0.05	-0.01	-0.13	-0.12
0.82	0.04	0.11	0.02	0.08	0.03	0.06	0.02	0.02	0.02	-0.04	-0.06
0.87	-0.01	-0.02	-0.07	-0.01	-0.07	-0.14	-0.13	-0.04	0.00	0.00	0.01
0.92	0.03	0.13	0.06	0.06	0.11	0.07	0.12	0.13	0.12	0.06	0.01
0.97	0.00	-0.07	-0.03	0.01	0.07	0.11	-0.03	-0.10	-0.01	-0.06	-0.11
1.02	0.08	0.03	-0.02	0.10	0.10	-0.01	-0.06	0.17	0.04	-0.07	-0.01
1.07	0.03	0.01	0.07	0.10	0.10	0.17	0.17	0.01	0.08	0.05	0.01
1.12	-1.10	-0.59	-0.10	0.12	0.11	-0.10	-0.15	-0.01	-0.03	-0.07	-0.25
1.17	-41.88	-41.40	-38.53	-33.48	-27.02	-19.80	-12.89	-6.80	-2.21	0.56	1.69
1.22	119.53	108.13	92.69	76.12	59.06	43.33	29.39	17.62	7.26	-1.83	-9.72
1.27	-85.28	-77.01	-64.80	-48.66	-30.85	-11.54	8.14	27.77	45.87	61.73	74.81
1.32	-12.46	-14.90	-16.22	-16.27	-15.37	-14.29	-13.77	-14.55	-17.26	-22.16	-29.48
1.37	-13.82	-14.45	-15.40	-16.39	-17.44	-18.10	-18.45	-18.09	-17.21	-16.04	-15.01
1.42	-16.04	-15.56	-14.93	-14.30	-13.83	-13.68	-14.01	-14.49	-15.06	-15.47	-16.01
1.47	-14.08	-14.97	-15.71	-16.05	-16.09	-15.60	-15.20	-14.56	-14.22	-14.19	-14.53
1.52	-12.49	-12.66	-13.07	-13.94	-14.87	-15.74	-16.39	-16.73	-16.61	-16.29	-15.73

1.57	-15.47	-15.46	-15.14	-14.69	-14.13	-13.92	-14.02	-14.33	-14.78	-15.43	-15.76
1.62	-13.39	-14.66	-15.48	-16.01	-16.02	-15.63	-14.94	-14.09	-13.30	-12.91	-12.65
1.67	-13.06	-12.91	-13.07	-13.35	-13.78	-14.48	-15.03	-15.39	-15.32	-15.21	-14.76
1.72	-14.32	-14.82	-14.91	-14.54	-14.10	-13.73	-13.25	-13.10	-13.10	-13.54	-13.98
1.77	-12.33	-12.89	-13.67	-14.38	-14.96	-15.35	-15.43	-15.29	-14.75	-14.16	-13.56
1.82	-13.74	-13.43	-13.13	-12.91	-12.90	-12.99	-13.36	-13.70	-14.05	-14.18	-14.15
1.87	-13.99	-14.42	-14.65	-14.53	-14.16	-13.74	-13.14	-12.82	-12.60	-12.68	-12.87
1.92	-12.13	-12.13	-12.47	-12.92	-13.53	-14.03	-14.50	-14.62	-14.49	-14.14	-13.62
1.97	-13.91	-13.92	-13.65	-13.32	-12.93	-12.72	-12.72	-12.79	-13.05	-13.34	-13.62
2.02	-12.36	-12.85	-13.40	-13.79	-14.00	-13.88	-13.73	-13.34	-12.91	-12.39	-12.12
2.07	-12.23	-12.23	-12.41	-12.82	-13.09	-13.50	-13.70	-13.80	-13.72	-13.36	-12.87
2.12	-13.31	-13.59	-13.69	-13.48	-13.16	-12.84	-12.67	-12.48	-12.51	-12.62	-12.67
2.17	-12.13	-12.76	-13.23	-13.52	-13.73	-13.76	-13.51	-13.12	-12.70	-12.13	-11.81
2.22	-12.87	-12.83	-12.73	-12.61	-12.59	-12.59	-12.79	-12.90	-13.04	-13.10	-13.14
2.27	-12.96	-13.41	-13.56	-13.69	-13.45	-13.00	-12.68	-12.32	-12.16	-11.95	-11.98
2.32	-11.77	-12.05	-12.36	-12.62	-12.92	-13.31	-13.42	-13.30	-12.92	-12.55	-12.18
2.37	-13.06	-13.12	-12.95	-12.70	-12.43	-12.41	-12.33	-12.24	-12.34	-12.48	-12.53
2.42	-12.56	-13.16	-13.30	-13.43	-13.36	-13.05	-12.78	-12.29	-12.03	-11.91	-11.84
2.47	-12.02	-12.23	-12.35	-12.59	-12.78	-13.00	-13.10	-13.08	-12.93	-12.57	-12.23
2.52	-12.97	-13.11	-13.16	-13.05	-12.80	-12.48	-12.37	-12.34	-12.09	-12.21	-12.29
2.57	-12.16	-12.64	-12.95	-13.16	-13.25	-13.12	-12.93	-12.67	-12.24	-11.90	-11.67
2.62	-12.34	-12.36	-12.32	-12.38	-12.41	-12.55	-12.68	-12.83	-12.72	-12.60	-12.29
2.67	-12.54	-12.79	-12.83	-12.65	-12.53	-12.26	-12.05	-11.87	-11.80	-11.69	-11.69
2.72	-11.90	-12.10	-12.32	-12.65	-12.84	-12.89	-12.93	-12.69	-12.35	-11.93	-11.62
2.77	-12.56	-12.47	-12.41	-12.29	-12.19	-12.14	-12.05	-12.16	-12.04	-12.04	-11.95
2.82	-12.11	-12.55	-12.70	-12.69	-12.63	-12.41	-12.12	-11.95	-11.67	-11.48	-11.34
2.87	-11.82	-12.11	-12.20	-12.25	-12.29	-12.41	-12.39	-12.34	-12.06	-11.87	-11.59
2.92	-12.55	-12.57	-12.61	-12.51	-12.22	-12.03	-11.95	-11.78	-11.70	-11.77	-11.59
2.97	-11.73	-12.06	-12.35	-12.42	-12.47	-12.46	-12.26	-11.96	-11.61	-11.34	-11.10
3.02	-11.86	-11.92	-11.93	-11.98	-12.07	-12.08	-12.08	-12.06	-12.07	-11.76	-11.50
3.07	-12.30	-12.43	-12.65	-12.53	-12.34	-12.18	-11.97	-11.71	-11.52	-11.40	-11.31
3.12	-11.59	-11.83	-12.13	-12.21	-12.33	-12.30	-12.18	-11.86	-11.53	-11.28	-11.01
3.17	-12.02	-12.18	-12.22	-12.06	-12.06	-11.86	-11.81	-11.83	-11.72	-11.59	-11.42
3.22	-11.96	-12.31	-12.49	-12.38	-12.44	-12.29	-12.06	-11.85	-11.51	-11.26	-11.14
3.42	-11.82	-11.89	-11.96	-12.05	-12.06	-12.04	-12.05	-11.85	-11.75	-11.63	-11.26
3.62	-12.02	-12.24	-12.41	-12.32	-12.10	-12.05	-11.78	-11.63	-11.39	-11.25	-11.12
3.82	-11.72	-11.88	-11.89	-12.01	-11.95	-11.86	-11.85	-11.80	-11.60	-11.45	-11.14
4.02	-11.83	-12.06	-12.15	-12.14	-12.07	-11.81	-11.73	-11.44	-11.25	-11.05	-10.83
4.22	-11.66	-11.71	-11.78	-11.86	-11.81	-11.85	-11.78	-11.48	-11.35	-11.14	-10.78
4.42	-11.67	-11.73	-11.82	-11.79	-11.72	-11.55	-11.45	-11.34	-11.02	-10.91	-10.64
4.62	-11.57	-11.70	-11.82	-11.82	-11.79	-11.71	-11.56	-11.44	-11.08	-11.00	-10.65
4.82	-11.66	-11.76	-11.88	-11.83	-11.74	-11.61	-11.44	-11.28	-10.97	-10.80	-10.61
5.02	-11.44	-11.62	-11.74	-11.68	-11.60	-11.53	-11.51	-11.28	-10.97	-10.77	-10.53
5.22	-11.56	-11.73	-11.92	-11.76	-11.59	-11.61	-11.36	-11.21	-10.95	-10.80	-10.55
5.42	-11.45	-11.70	-11.74	-11.67	-11.61	-11.59	-11.40	-11.20	-10.94	-10.64	-10.49
5.62	-11.41	-11.53	-11.43	-11.36	-11.37	-11.25	-11.16	-10.91	-10.75	-10.47	-10.23
5.82	-11.34	-11.50	-11.48	-11.49	-11.45	-11.23	-11.19	-10.93	-10.74	-10.45	-10.24
6.02	-11.21	-11.32	-11.41	-11.39	-11.29	-11.12	-10.98	-10.69	-10.49	-10.30	-10.18
6.22	-11.24	-11.43	-11.42	-11.41	-11.34	-11.14	-11.14	-10.82	-10.57	-10.42	-10.16
6.42	-11.29	-11.38	-11.43	-11.36	-11.30	-11.17	-10.96	-10.76	-10.53	-10.29	-10.05

6.62	-11.24	-11.34	-11.45	-11.37	-11.24	-11.16	-10.93	-10.72	-10.48	-10.26	-10.01
6.82	-11.16	-11.17	-11.24	-11.26	-11.11	-10.90	-10.80	-10.63	-10.38	-10.14	-9.95
7.02	-11.10	-11.22	-11.16	-11.23	-11.05	-11.05	-10.82	-10.60	-10.30	-10.11	-9.85
7.22	-11.00	-11.20	-11.19	-11.16	-11.01	-10.90	-10.81	-10.56	-10.30	-10.21	-9.79
7.42	-11.18	-11.26	-11.29	-11.20	-11.18	-11.03	-10.84	-10.59	-10.31	-10.14	-9.84
7.62	-10.92	-11.06	-11.13	-11.06	-11.00	-10.86	-10.64	-10.48	-10.23	-10.04	-9.67
7.82	-10.93	-10.97	-10.98	-10.89	-10.84	-10.69	-10.57	-10.31	-10.09	-9.82	-9.65
8.02	-10.99	-11.07	-11.05	-11.03	-10.90	-10.83	-10.63	-10.44	-10.15	-9.90	-9.58
8.22	-10.63	-10.80	-10.80	-10.81	-10.71	-10.54	-10.40	-10.19	-9.98	-9.66	-9.40
8.42	-10.88	-10.94	-10.95	-10.91	-10.87	-10.60	-10.58	-10.33	-10.03	-9.76	-9.53
8.62	-10.74	-10.75	-10.83	-10.75	-10.49	-10.46	-10.34	-10.04	-9.84	-9.53	-9.33
8.82	-10.83	-10.90	-11.00	-10.92	-10.79	-10.59	-10.52	-10.22	-9.94	-9.80	-9.50
9.02	-10.69	-10.74	-10.78	-10.69	-10.65	-10.55	-10.29	-10.02	-9.79	-9.61	-9.31
9.22	-10.75	-10.87	-11.00	-10.89	-10.78	-10.61	-10.42	-10.22	-9.93	-9.66	-9.48
9.42	-10.69	-10.77	-10.79	-10.72	-10.59	-10.46	-10.36	-10.17	-9.82	-9.56	-9.25
9.62	-10.68	-10.79	-10.73	-10.75	-10.60	-10.41	-10.28	-10.04	-9.81	-9.55	-9.24
9.82	-10.63	-10.65	-10.75	-10.62	-10.60	-10.35	-10.30	-9.99	-9.69	-9.56	-9.22
10.02	-10.70	-10.78	-10.81	-10.75	-10.59	-10.43	-10.21	-9.94	-9.76	-9.50	-9.18
10.22	-10.62	-10.67	-10.69	-10.67	-10.62	-10.30	-10.26	-9.97	-9.65	-9.49	-9.24
10.42	-10.72	-10.77	-10.80	-10.71	-10.56	-10.42	-10.28	-9.97	-9.80	-9.47	-9.27
10.62	-10.59	-10.70	-10.72	-10.72	-10.59	-10.37	-10.22	-10.01	-9.71	-9.52	-9.20
10.82	-10.55	-10.67	-10.67	-10.48	-10.45	-10.38	-10.16	-9.91	-9.73	-9.38	-9.10
11.02	-10.48	-10.56	-10.55	-10.52	-10.39	-10.24	-10.13	-9.84	-9.57	-9.33	-9.13
11.22	-10.50	-10.57	-10.51	-10.40	-10.41	-10.16	-10.06	-9.69	-9.49	-9.23	-8.96
11.42	-10.32	-10.44	-10.38	-10.37	-10.23	-10.08	-9.96	-9.68	-9.37	-9.15	-8.85
11.62	-10.44	-10.56	-10.53	-10.52	-10.34	-10.18	-10.02	-9.83	-9.48	-9.27	-9.04
11.82	-10.56	-10.56	-10.56	-10.54	-10.46	-10.21	-10.09	-9.80	-9.54	-9.28	-9.01
12.02	-10.29	-10.43	-10.45	-10.38	-10.24	-10.13	-9.90	-9.70	-9.37	-9.11	-8.89
12.22	-10.56	-10.70	-10.65	-10.62	-10.50	-10.25	-10.17	-9.82	-9.57	-9.27	-8.94
12.42	-10.28	-10.49	-10.30	-10.26	-10.18	-10.02	-9.83	-9.61	-9.36	-9.10	-8.79
12.62	-10.29	-10.40	-10.39	-10.34	-10.18	-10.02	-9.86	-9.63	-9.38	-9.04	-8.83
12.82	-10.06	-10.04	-10.09	-9.98	-9.92	-9.80	-9.52	-9.35	-9.05	-8.86	-8.48
13.02	-10.39	-10.49	-10.45	-10.34	-10.25	-10.06	-9.87	-9.61	-9.39	-9.18	-8.82
13.22	-10.19	-10.35	-10.23	-10.29	-10.14	-9.98	-9.88	-9.62	-9.29	-9.08	-8.75
13.42	-10.40	-10.38	-10.61	-10.38	-10.32	-10.12	-9.90	-9.68	-9.41	-9.08	-8.87
13.50	-10.16	-10.17	-10.30	-10.17	-9.95	-9.77	-9.60	-9.43	-9.06	-8.89	-8.62
13.59	-10.32	-10.42	-10.42	-10.28	-10.23	-10.02	-9.86	-9.64	-9.26	-9.10	-8.77
13.68	-10.26	-10.30	-10.28	-10.20	-10.13	-9.91	-9.75	-9.49	-9.24	-8.96	-8.75
13.78	-10.22	-10.36	-10.38	-10.33	-10.11	-9.96	-9.82	-9.47	-9.39	-9.00	-8.76
13.88	-10.29	-10.40	-10.39	-10.19	-10.19	-10.02	-9.87	-9.54	-9.32	-9.09	-8.78
14.00	-10.41	-10.44	-10.52	-10.50	-10.27	-10.15	-9.91	-9.66	-9.40	-9.11	-8.76
14.12	-10.33	-10.39	-10.41	-10.25	-10.16	-9.95	-9.84	-9.48	-9.33	-9.02	-8.66
14.25	-10.19	-10.33	-10.25	-10.26	-10.11	-9.94	-9.80	-9.46	-9.21	-9.04	-8.73
14.40	-10.46	-10.61	-10.65	-10.41	-10.34	-10.24	-10.06	-9.80	-9.43	-9.17	-8.97
14.55	-10.26	-10.33	-10.36	-10.25	-10.12	-9.97	-9.74	-9.48	-9.22	-9.01	-8.70
14.72	-10.20	-10.21	-10.22	-10.22	-10.07	-9.84	-9.67	-9.45	-9.13	-8.88	-8.61
14.90	-10.04	-10.18	-10.04	-9.99	-9.93	-9.69	-9.52	-9.29	-9.00	-8.76	-8.50
15.09	-10.14	-10.18	-10.16	-10.04	-9.94	-9.76	-9.59	-9.31	-9.02	-8.85	-8.66
15.30	-10.12	-10.11	-10.09	-10.06	-9.95	-9.79	-9.69	-9.29	-9.02	-8.84	-8.53
15.53	-10.39	-10.44	-10.44	-10.28	-10.25	-10.01	-9.92	-9.55	-9.39	-9.15	-8.89

15.77	-10.08	-10.17	-10.14	-10.12	-9.94	-9.85	-9.69	-9.36	-9.14	-8.81	-8.51
16.04	-10.34	-10.36	-10.30	-10.23	-10.16	-9.99	-9.81	-9.56	-9.27	-9.01	-8.79
16.32	-10.09	-10.15	-10.08	-10.08	-9.94	-9.74	-9.59	-9.31	-9.06	-8.76	-8.53
16.63	-10.30	-10.45	-10.44	-10.24	-10.22	-9.84	-9.74	-9.52	-9.27	-8.92	-8.67
16.96	-10.17	-10.18	-10.21	-10.02	-9.99	-9.76	-9.54	-9.40	-9.09	-8.75	-8.48
17.31	-10.12	-10.16	-10.25	-10.08	-9.97	-9.85	-9.74	-9.39	-9.15	-8.83	-8.54
17.70	-10.28	-10.36	-10.24	-10.16	-10.05	-9.92	-9.71	-9.45	-9.18	-8.89	-8.64
18.11	-10.00	-10.06	-10.09	-9.91	-9.83	-9.69	-9.49	-9.27	-8.90	-8.74	-8.40
18.56	-10.06	-10.10	-9.99	-10.01	-9.83	-9.75	-9.43	-9.22	-8.92	-8.70	-8.36
19.04	-10.15	-10.20	-10.21	-10.03	-9.82	-9.76	-9.55	-9.25	-9.05	-8.75	-8.43
19.56	-10.09	-10.09	-10.07	-10.07	-9.97	-9.71	-9.53	-9.30	-9.07	-8.76	-8.46
20.12	-10.17	-10.23	-10.26	-10.22	-10.06	-9.84	-9.63	-9.46	-9.11	-8.82	-8.53
20.72	-10.20	-10.19	-10.26	-10.08	-10.04	-9.89	-9.64	-9.38	-9.08	-8.93	-8.57
21.37	-10.11	-10.26	-10.20	-9.99	-9.98	-9.79	-9.54	-9.27	-8.99	-8.69	-8.43
22.08	-10.34	-10.44	-10.43	-10.28	-10.16	-10.02	-9.70	-9.49	-9.19	-8.92	-8.61
22.84	-10.21	-10.11	-10.29	-10.12	-10.03	-9.77	-9.68	-9.44	-9.07	-8.87	-8.61
23.65	-10.10	-10.08	-10.18	-10.01	-9.87	-9.69	-9.58	-9.28	-8.96	-8.79	-8.44
24.54	-10.08	-9.97	-10.04	-9.89	-9.81	-9.62	-9.51	-9.17	-9.03	-8.64	-8.40
25.49	-10.18	-10.29	-10.20	-10.14	-10.07	-9.74	-9.62	-9.36	-9.00	-8.78	-8.48
26.51	-9.91	-10.01	-10.01	-9.86	-9.72	-9.52	-9.36	-9.06	-8.75	-8.52	-8.31
27.62	-10.04	-10.18	-10.18	-10.05	-9.92	-9.76	-9.58	-9.24	-8.98	-8.80	-8.43
28.81	-10.05	-10.03	-10.06	-9.97	-9.89	-9.68	-9.46	-9.21	-8.83	-8.63	-8.39
30.10	-9.97	-9.94	-10.02	-9.83	-9.66	-9.52	-9.43	-9.03	-8.82	-8.53	-8.26
31.49	-9.86	-9.95	-9.90	-9.82	-9.62	-9.55	-9.30	-9.03	-8.82	-8.49	-8.23
32.98	-9.85	-9.92	-9.91	-9.74	-9.63	-9.45	-9.37	-9.06	-8.78	-8.49	-8.29
34.60	-9.98	-9.97	-9.97	-9.84	-9.65	-9.61	-9.37	-9.05	-8.76	-8.48	-8.12
36.34	-9.94	-10.06	-9.94	-9.92	-9.70	-9.64	-9.39	-9.17	-8.80	-8.56	-8.28
38.21	-9.72	-9.74	-9.73	-9.68	-9.60	-9.39	-9.13	-8.86	-8.63	-8.26	-7.94
40.24	-9.96	-9.99	-9.95	-9.93	-9.72	-9.58	-9.31	-9.12	-8.77	-8.57	-8.25
42.42	-9.68	-9.75	-9.68	-9.54	-9.55	-9.29	-9.13	-8.81	-8.54	-8.20	-7.99
44.78	-9.83	-9.89	-9.86	-9.73	-9.53	-9.44	-9.32	-8.99	-8.73	-8.44	-8.05
47.32	-9.69	-9.74	-9.69	-9.51	-9.51	-9.17	-9.05	-8.84	-8.55	-8.20	-8.00
50.06	-9.88	-9.89	-9.84	-9.85	-9.57	-9.44	-9.25	-8.94	-8.65	-8.41	-8.12
53.01	-9.91	-9.85	-9.87	-9.75	-9.63	-9.43	-9.26	-8.94	-8.67	-8.43	-8.11
56.20	-9.89	-9.81	-9.82	-9.73	-9.58	-9.46	-9.27	-8.96	-8.66	-8.37	-8.15
59.64	-9.80	-9.89	-9.78	-9.74	-9.57	-9.45	-9.27	-9.02	-8.71	-8.41	-8.13
63.34	-9.61	-9.66	-9.66	-9.56	-9.36	-9.33	-8.96	-8.72	-8.44	-8.15	-7.88
67.34	-9.67	-9.76	-9.67	-9.57	-9.41	-9.21	-9.10	-8.85	-8.52	-8.22	-7.93
71.65	-9.73	-9.83	-9.73	-9.64	-9.62	-9.29	-9.21	-8.98	-8.63	-8.30	-8.06
76.30	-9.84	-9.90	-9.91	-9.76	-9.65	-9.54	-9.27	-8.99	-8.77	-8.44	-8.16
81.32	-9.78	-9.78	-9.80	-9.77	-9.69	-9.43	-9.34	-9.00	-8.72	-8.37	-8.10
86.73	-9.83	-9.86	-9.79	-9.72	-9.63	-9.40	-9.18	-8.96	-8.64	-8.41	-8.14
92.56	-9.71	-9.75	-9.76	-9.64	-9.48	-9.25	-9.07	-8.89	-8.60	-8.27	-7.93
98.85	-9.68	-9.63	-9.71	-9.58	-9.46	-9.19	-9.04	-8.84	-8.54	-8.23	-7.99
105.64	-9.70	-9.79	-9.68	-9.59	-9.46	-9.35	-9.11	-8.88	-8.59	-8.36	-7.97
112.96	-9.73	-9.79	-9.76	-9.66	-9.60	-9.41	-9.32	-9.04	-8.62	-8.35	-8.04
120.85	-9.48	-9.56	-9.51	-9.44	-9.33	-9.12	-8.92	-8.69	-8.28	-8.13	-7.82
129.36	-9.80	-9.87	-9.89	-9.79	-9.72	-9.57	-9.30	-9.07	-8.78	-8.43	-8.17
138.55	-9.68	-9.68	-9.63	-9.48	-9.41	-9.23	-9.17	-8.73	-8.45	-8.15	-7.98
148.45	-9.67	-9.73	-9.76	-9.57	-9.44	-9.20	-9.08	-8.85	-8.45	-8.24	-7.97

159.13	-9.46	-9.63	-9.72	-9.54	-9.50	-9.20	-9.01	-8.82	-8.46	-8.19	-7.91
170.65	-9.64	-9.69	-9.66	-9.59	-9.45	-9.25	-9.13	-8.81	-8.51	-8.15	-7.87
183.07	-9.42	-9.61	-9.51	-9.41	-9.35	-9.19	-8.92	-8.66	-8.27	-8.06	-7.75
196.47	-9.57	-9.53	-9.57	-9.41	-9.40	-9.17	-9.00	-8.79	-8.41	-8.09	-7.84
210.91	-9.31	-9.44	-9.43	-9.29	-9.11	-8.93	-8.91	-8.63	-8.25	-8.03	-7.73
226.50	-9.33	-9.42	-9.34	-9.28	-9.19	-9.06	-8.76	-8.56	-8.31	-7.97	-7.62
243.31	-9.25	-9.33	-9.30	-9.12	-9.12	-8.97	-8.71	-8.48	-8.24	-7.94	-7.67
261.43	-9.48	-9.52	-9.39	-9.41	-9.25	-9.11	-8.99	-8.64	-8.35	-8.09	-7.86
280.98	-9.28	-9.29	-9.30	-9.19	-9.08	-8.94	-8.78	-8.56	-8.23	-7.93	-7.63
302.07	-9.14	-9.19	-9.18	-9.04	-8.93	-8.77	-8.66	-8.40	-8.04	-7.85	-7.56
324.81	-9.17	-9.26	-9.16	-9.08	-9.00	-8.83	-8.70	-8.37	-8.11	-7.77	-7.55
349.33	-9.05	-9.06	-9.14	-9.04	-8.92	-8.82	-8.61	-8.37	-7.98	-7.71	-7.38
375.79	-9.13	-9.22	-9.17	-9.10	-8.88	-8.77	-8.70	-8.30	-8.04	-7.81	-7.49
404.31	-8.89	-8.97	-8.96	-8.88	-8.71	-8.59	-8.35	-8.17	-7.92	-7.63	-7.29
435.08	-9.15	-9.14	-9.13	-8.99	-9.00	-8.80	-8.70	-8.39	-8.10	-7.77	-7.51
468.27	-8.91	-8.95	-9.07	-8.96	-8.77	-8.64	-8.49	-8.29	-7.95	-7.60	-7.33
504.06	-9.03	-9.00	-8.91	-8.90	-8.74	-8.68	-8.42	-8.15	-7.83	-7.65	-7.26
542.66	-8.80	-8.95	-8.92	-8.94	-8.72	-8.57	-8.42	-8.14	-7.79	-7.56	-7.24
584.29	-8.82	-8.89	-8.92	-8.91	-8.79	-8.57	-8.46	-8.07	-7.87	-7.54	-7.30
629.18	-8.66	-8.74	-8.68	-8.67	-8.56	-8.44	-8.22	-7.99	-7.72	-7.50	-7.07
677.61	-8.84	-8.91	-8.98	-8.76	-8.75	-8.59	-8.41	-8.11	-7.78	-7.52	-7.14
729.83	-8.48	-8.60	-8.63	-8.55	-8.40	-8.24	-8.06	-7.90	-7.54	-7.22	-6.98
786.16	-8.60	-8.63	-8.71	-8.62	-8.59	-8.40	-8.19	-7.97	-7.72	-7.43	-7.15
846.90	-8.58	-8.60	-8.65	-8.65	-8.49	-8.41	-8.25	-7.91	-7.60	-7.31	-7.06
912.42	-8.52	-8.58	-8.68	-8.48	-8.41	-8.23	-8.14	-7.91	-7.60	-7.18	-7.11
983.08	-8.53	-8.68	-8.51	-8.50	-8.43	-8.26	-8.07	-7.87	-7.46	-7.27	-7.00
1059.29	-8.67	-8.83	-8.83	-8.75	-8.62	-8.42	-8.39	-8.07	-7.70	-7.47	-7.13
1141.48	-8.54	-8.63	-8.64	-8.52	-8.48	-8.27	-8.16	-7.81	-7.54	-7.26	-6.89
1230.13	-8.58	-8.67	-8.57	-8.57	-8.43	-8.37	-8.15	-7.88	-7.56	-7.32	-7.00
1325.73	-8.79	-8.72	-8.81	-8.64	-8.58	-8.37	-8.28	-8.07	-7.66	-7.37	-7.17
1428.84	-8.73	-8.84	-8.84	-8.74	-8.70	-8.50	-8.32	-8.07	-7.76	-7.45	-7.13
1540.05	-8.93	-8.88	-8.93	-8.78	-8.79	-8.58	-8.47	-8.14	-7.87	-7.55	-7.23
1659.99	-8.88	-8.99	-8.89	-8.93	-8.83	-8.63	-8.50	-8.15	-7.83	-7.59	-7.30
1789.34	-8.81	-8.89	-8.90	-8.86	-8.72	-8.62	-8.42	-8.18	-7.92	-7.55	-7.29
1928.85	-8.87	-8.91	-8.93	-8.90	-8.81	-8.61	-8.52	-8.21	-7.87	-7.57	-7.27
2079.31	-9.01	-9.12	-9.17	-9.14	-9.10	-8.88	-8.64	-8.35	-8.04	-7.75	-7.47
2241.59	-8.97	-9.08	-9.17	-9.02	-8.98	-8.71	-8.68	-8.22	-8.04	-7.76	-7.42
2416.60	-9.03	-9.26	-9.27	-9.27	-9.23	-8.92	-8.82	-8.52	-8.14	-7.94	-7.63
2605.36	-9.16	-9.17	-9.23	-9.22	-9.17	-9.12	-8.87	-8.57	-8.20	-7.95	-7.65
2808.94	-9.09	-9.29	-9.32	-9.36	-9.29	-9.03	-8.94	-8.51	-8.34	-7.95	-7.66
3028.50	-9.04	-9.21	-9.38	-9.31	-9.21	-9.07	-8.85	-8.59	-8.24	-7.94	-7.59
3265.30	-9.14	-9.27	-9.33	-9.26	-9.16	-9.09	-8.83	-8.70	-8.45	-8.05	-7.71
3520.69	-9.08	-9.17	-9.22	-9.23	-9.20	-9.04	-8.92	-8.52	-8.20	-7.97	-7.70
3796.13	-8.89	-9.09	-9.14	-9.18	-9.20	-8.98	-8.82	-8.50	-8.20	-7.97	-7.61

Wavelength (nm) / Time (ps)	506.40	507.82	509.25	510.67	512.10	513.52	514.94	516.37	517.79	519.22	520.64
-3.78	0.07	0.05	0.12	0.11	0.02	0.03	0.13	0.01	0.03	0.10	0.12

-3.28	-0.01	0.05	-0.01	0.09	0.03	0.07	0.07	0.08	0.08	0.05	0.06
-2.78	-0.12	-0.07	-0.15	-0.06	-0.02	-0.13	-0.08	-0.15	-0.02	-0.13	-0.04
-2.28	0.07	0.04	0.07	0.06	0.07	0.01	0.05	0.12	0.04	0.07	-0.01
-1.78	0.01	-0.02	-0.01	-0.12	-0.05	-0.05	-0.03	-0.03	-0.07	-0.11	-0.11
-1.28	-0.02	-0.11	0.00	-0.07	-0.05	-0.06	-0.08	-0.06	-0.05	0.00	-0.06
-0.78	0.00	0.04	0.02	-0.03	0.07	0.05	0.00	0.05	0.02	0.02	0.05
-0.28	-0.01	0.01	-0.04	0.03	-0.08	0.08	-0.06	-0.02	-0.04	-0.01	0.01
-0.18	0.00	-0.01	-0.06	0.02	-0.04	-0.09	0.04	-0.04	0.01	-0.09	-0.06
-0.08	0.11	0.07	0.09	0.13	0.06	0.06	0.09	0.11	0.07	0.06	0.09
0.02	0.01	0.01	-0.03	-0.07	-0.07	-0.17	-0.03	-0.04	0.01	-0.01	-0.05
0.12	0.10	0.04	0.16	0.03	0.09	0.02	0.10	0.11	0.09	0.01	0.00
0.22	0.05	0.12	-0.01	0.03	0.09	-0.04	0.08	0.06	0.03	0.00	0.09
0.27	-0.04	0.00	0.01	-0.08	-0.04	-0.09	0.02	-0.07	-0.03	-0.08	0.04
0.32	0.13	0.05	0.16	0.13	0.10	-0.01	0.13	0.07	0.09	0.07	0.09
0.37	-0.01	-0.04	-0.03	-0.07	-0.02	0.01	-0.07	-0.03	-0.05	-0.08	-0.06
0.42	-0.18	-0.16	-0.18	-0.11	-0.17	-0.13	-0.07	-0.18	-0.13	-0.20	-0.10
0.47	-0.06	-0.07	-0.09	-0.12	0.01	-0.12	0.01	0.01	-0.01	-0.11	-0.10
0.52	-0.09	-0.14	-0.11	-0.07	-0.08	-0.02	-0.02	-0.07	-0.08	-0.08	-0.04
0.57	-0.01	-0.03	0.04	0.01	-0.02	-0.04	-0.06	-0.03	-0.03	-0.04	0.02
0.62	0.03	0.00	0.07	-0.02	0.00	-0.01	0.07	0.02	0.02	-0.05	0.03
0.67	-0.06	-0.09	-0.14	-0.16	-0.11	-0.08	-0.09	-0.08	-0.05	-0.06	-0.12
0.72	-0.04	-0.08	-0.15	-0.12	-0.08	-0.12	-0.12	0.03	-0.18	-0.15	-0.11
0.77	-0.06	-0.03	-0.12	-0.11	-0.09	-0.10	-0.05	-0.05	-0.08	-0.04	-0.01
0.82	0.09	0.03	0.08	0.04	0.00	-0.01	0.09	0.10	0.03	0.00	-0.05
0.87	-0.04	-0.06	0.01	-0.16	0.00	-0.05	0.01	-0.02	-0.11	-0.16	0.00
0.92	0.11	0.06	0.13	0.05	0.05	0.06	0.21	0.10	0.11	0.12	0.09
0.97	-0.06	-0.01	-0.03	-0.04	-0.04	-0.05	-0.01	0.02	0.00	-0.06	-0.09
1.02	0.01	0.04	0.02	0.00	-0.04	-0.09	0.12	0.03	0.01	-0.01	0.06
1.07	0.06	0.09	0.12	0.03	0.15	0.10	0.00	0.10	0.17	-0.03	0.08
1.12	-0.03	-0.03	-0.05	-0.06	-0.02	0.00	0.07	-0.04	-0.13	-0.15	0.05
1.17	1.58	0.68	-0.14	-0.81	-1.03	-1.03	-0.79	-0.44	-0.10	0.03	0.17
1.22	-16.53	-21.94	-26.73	-30.62	-33.21	-35.10	-36.33	-37.04	-36.70	-35.42	-32.61
1.27	84.78	92.18	95.69	95.69	93.72	89.43	84.33	76.17	67.69	57.49	47.55
1.32	-38.60	-49.51	-60.48	-70.88	-79.43	-84.87	-87.00	-85.18	-79.36	-70.45	-58.14
1.37	-13.77	-12.59	-11.54	-10.81	-10.49	-10.69	-11.36	-12.64	-13.98	-15.42	-16.57
1.42	-16.10	-16.20	-15.92	-15.72	-15.49	-15.40	-15.39	-15.71	-16.23	-16.80	-17.32
1.47	-14.93	-15.60	-16.33	-16.96	-17.40	-17.46	-17.36	-17.00	-16.49	-16.02	-15.43
1.52	-14.97	-14.41	-13.99	-13.90	-13.92	-14.19	-14.70	-15.04	-15.47	-15.82	-16.02
1.57	-15.82	-15.80	-15.38	-14.92	-14.26	-13.75	-13.38	-13.22	-13.40	-13.76	-14.13
1.62	-12.79	-13.19	-13.83	-14.47	-14.89	-15.04	-15.22	-15.14	-14.86	-14.53	-14.10
1.67	-14.20	-13.67	-13.11	-13.00	-12.94	-13.18	-13.51	-13.96	-14.34	-14.60	-14.86
1.72	-14.34	-14.73	-14.69	-14.76	-14.40	-13.95	-13.35	-13.00	-12.63	-12.41	-12.34
1.77	-12.93	-12.65	-12.52	-12.63	-12.77	-13.18	-13.56	-13.76	-13.95	-13.98	-13.83
1.82	-13.78	-13.43	-12.89	-12.41	-12.00	-11.80	-11.74	-11.98	-12.14	-12.60	-12.97
1.87	-13.14	-13.44	-13.66	-13.77	-13.81	-13.61	-13.25	-12.85	-12.54	-12.28	-12.06
1.92	-13.00	-12.54	-12.18	-12.09	-11.94	-12.23	-12.35	-12.64	-12.90	-13.04	-12.97
1.97	-13.53	-13.42	-13.16	-12.61	-12.14	-11.70	-11.30	-11.21	-11.24	-11.40	-11.61
2.02	-11.87	-11.78	-11.80	-12.12	-12.16	-12.38	-12.44	-12.39	-12.34	-12.24	-11.91
2.07	-12.37	-11.98	-11.54	-11.45	-11.34	-11.56	-11.73	-11.92	-12.07	-12.25	-12.23
2.12	-12.81	-12.79	-12.85	-12.56	-12.25	-11.98	-11.64	-11.29	-11.15	-10.95	-10.94

2.17	-11.46	-11.32	-11.34	-11.49	-11.57	-11.67	-11.79	-11.73	-11.70	-11.57	-11.36
2.22	-12.64	-12.42	-11.97	-11.57	-11.22	-11.05	-10.92	-11.02	-11.10	-11.24	-11.48
2.27	-11.98	-12.18	-12.23	-12.20	-12.12	-11.88	-11.72	-11.34	-11.23	-10.96	-10.69
2.32	-11.66	-11.40	-11.08	-10.96	-10.92	-11.07	-11.19	-11.34	-11.47	-11.48	-11.34
2.37	-12.25	-12.02	-11.73	-11.44	-11.05	-10.76	-10.51	-10.46	-10.44	-10.56	-10.57
2.42	-11.60	-11.68	-11.82	-11.86	-11.80	-11.64	-11.52	-11.29	-11.06	-10.83	-10.64
2.47	-11.88	-11.49	-11.17	-10.94	-10.82	-10.82	-10.87	-11.01	-11.08	-11.11	-11.15
2.52	-12.11	-12.06	-11.77	-11.68	-11.29	-10.99	-10.71	-10.61	-10.41	-10.42	-10.39
2.57	-11.32	-11.25	-11.10	-11.15	-11.10	-11.17	-11.25	-11.10	-11.06	-10.87	-10.71
2.62	-11.94	-11.60	-11.18	-10.91	-10.57	-10.49	-10.50	-10.61	-10.69	-10.73	-10.90
2.67	-11.57	-11.48	-11.40	-11.13	-10.99	-10.62	-10.43	-10.26	-10.03	-10.03	-9.93
2.72	-11.12	-10.94	-10.72	-10.67	-10.62	-10.74	-10.69	-10.75	-10.71	-10.75	-10.60
2.77	-11.66	-11.44	-11.19	-10.83	-10.47	-10.31	-10.13	-10.04	-10.01	-10.13	-10.18
2.82	-11.20	-11.16	-10.96	-10.93	-10.81	-10.62	-10.55	-10.34	-10.21	-10.07	-9.92
2.87	-11.20	-10.92	-10.64	-10.37	-10.28	-10.20	-10.17	-10.23	-10.25	-10.26	-10.19
2.92	-11.42	-11.40	-11.08	-10.92	-10.51	-10.35	-10.11	-9.86	-9.84	-9.76	-9.84
2.97	-10.69	-10.58	-10.46	-10.36	-10.36	-10.30	-10.23	-10.31	-10.12	-10.01	-9.86
3.02	-11.11	-10.85	-10.52	-10.32	-10.03	-9.96	-9.94	-9.87	-9.95	-9.98	-9.97
3.07	-11.15	-11.00	-10.86	-10.67	-10.49	-10.20	-9.97	-9.96	-9.85	-9.70	-9.57
3.12	-10.61	-10.44	-10.29	-10.12	-10.04	-10.14	-9.99	-10.01	-9.92	-9.92	-9.72
3.17	-11.15	-10.89	-10.62	-10.27	-10.08	-9.89	-9.73	-9.62	-9.66	-9.68	-9.61
3.22	-10.92	-10.72	-10.66	-10.54	-10.32	-10.25	-10.04	-9.97	-9.86	-9.80	-9.55
3.42	-10.96	-10.61	-10.31	-10.08	-9.85	-9.65	-9.62	-9.65	-9.78	-9.64	-9.65
3.62	-10.81	-10.70	-10.51	-10.32	-10.15	-9.93	-9.80	-9.66	-9.55	-9.43	-9.37
3.82	-10.71	-10.45	-10.17	-9.96	-9.75	-9.61	-9.57	-9.54	-9.53	-9.52	-9.39
4.02	-10.66	-10.50	-10.27	-10.13	-9.86	-9.70	-9.60	-9.55	-9.38	-9.29	-9.21
4.22	-10.44	-10.24	-9.97	-9.73	-9.60	-9.48	-9.35	-9.37	-9.44	-9.29	-9.22
4.42	-10.43	-10.11	-9.94	-9.75	-9.58	-9.39	-9.15	-9.10	-9.05	-8.99	-8.80
4.62	-10.39	-10.12	-9.78	-9.69	-9.50	-9.40	-9.25	-9.17	-9.18	-9.04	-9.04
4.82	-10.39	-10.13	-9.83	-9.75	-9.48	-9.35	-9.12	-9.01	-9.01	-8.96	-8.85
5.02	-10.23	-9.97	-9.71	-9.48	-9.35	-9.18	-9.11	-9.13	-8.87	-8.92	-8.80
5.22	-10.26	-10.05	-9.76	-9.51	-9.40	-9.29	-9.11	-8.94	-8.83	-8.87	-8.79
5.42	-10.06	-9.84	-9.60	-9.49	-9.25	-9.13	-8.90	-8.93	-8.83	-8.83	-8.70
5.62	-9.93	-9.73	-9.43	-9.27	-9.01	-8.83	-8.62	-8.69	-8.61	-8.45	-8.47
5.82	-9.83	-9.67	-9.35	-9.12	-9.05	-8.86	-8.67	-8.65	-8.56	-8.59	-8.39
6.02	-9.68	-9.54	-9.30	-9.07	-8.85	-8.71	-8.56	-8.45	-8.42	-8.34	-8.26
6.22	-9.78	-9.52	-9.24	-8.96	-8.72	-8.74	-8.56	-8.37	-8.38	-8.33	-8.29
6.42	-9.75	-9.50	-9.26	-8.99	-8.80	-8.66	-8.58	-8.47	-8.37	-8.32	-8.24
6.62	-9.68	-9.45	-9.13	-9.01	-8.72	-8.63	-8.47	-8.42	-8.21	-8.32	-8.12
6.82	-9.57	-9.28	-9.05	-8.88	-8.63	-8.35	-8.28	-8.25	-8.19	-8.11	-7.99
7.02	-9.49	-9.25	-8.94	-8.72	-8.54	-8.44	-8.26	-8.11	-8.08	-8.02	-7.90
7.22	-9.48	-9.29	-8.99	-8.71	-8.51	-8.43	-8.22	-8.10	-8.00	-8.00	-7.80
7.42	-9.48	-9.31	-9.00	-8.86	-8.61	-8.39	-8.33	-8.21	-8.10	-7.96	-7.94
7.62	-9.35	-9.12	-8.83	-8.66	-8.47	-8.21	-8.13	-8.03	-7.92	-7.86	-7.85
7.82	-9.20	-9.00	-8.69	-8.41	-8.26	-8.12	-7.95	-7.85	-7.78	-7.76	-7.64
8.02	-9.33	-9.08	-8.80	-8.55	-8.24	-8.13	-7.98	-7.91	-7.81	-7.75	-7.54
8.22	-9.01	-8.80	-8.51	-8.27	-8.02	-8.03	-7.85	-7.65	-7.56	-7.51	-7.49
8.42	-9.14	-8.91	-8.63	-8.42	-8.18	-8.09	-7.95	-7.75	-7.69	-7.65	-7.51
8.62	-9.01	-8.74	-8.46	-8.24	-8.07	-7.91	-7.69	-7.59	-7.44	-7.51	-7.41
8.82	-9.19	-8.88	-8.58	-8.36	-8.08	-8.00	-7.85	-7.61	-7.67	-7.56	-7.43

9.02	-8.88	-8.57	-8.35	-8.17	-7.94	-7.75	-7.56	-7.52	-7.51	-7.36	-7.28
9.22	-9.05	-8.78	-8.49	-8.27	-8.08	-7.87	-7.78	-7.63	-7.63	-7.50	-7.42
9.42	-8.98	-8.74	-8.40	-8.28	-7.93	-7.80	-7.68	-7.50	-7.53	-7.40	-7.31
9.62	-8.91	-8.68	-8.44	-8.14	-7.99	-7.72	-7.57	-7.44	-7.43	-7.31	-7.22
9.82	-8.87	-8.57	-8.39	-8.13	-7.88	-7.76	-7.62	-7.47	-7.47	-7.26	-7.20
10.02	-8.96	-8.56	-8.40	-8.16	-7.83	-7.69	-7.60	-7.44	-7.47	-7.30	-7.22
10.22	-8.81	-8.57	-8.28	-8.12	-7.73	-7.72	-7.51	-7.36	-7.37	-7.26	-7.10
10.42	-8.83	-8.63	-8.25	-8.11	-7.87	-7.70	-7.62	-7.47	-7.35	-7.23	-7.23
10.62	-8.78	-8.59	-8.23	-8.10	-7.86	-7.77	-7.48	-7.39	-7.40	-7.22	-7.17
10.82	-8.84	-8.58	-8.16	-8.10	-7.71	-7.63	-7.48	-7.29	-7.18	-7.16	-7.07
11.02	-8.69	-8.47	-8.15	-7.91	-7.70	-7.58	-7.42	-7.26	-7.16	-7.11	-6.98
11.22	-8.59	-8.37	-8.05	-7.76	-7.58	-7.44	-7.22	-7.16	-7.05	-7.02	-6.91
11.42	-8.47	-8.32	-7.99	-7.72	-7.51	-7.39	-7.21	-7.03	-6.98	-6.97	-6.89
11.62	-8.56	-8.41	-8.10	-7.85	-7.69	-7.47	-7.34	-7.17	-7.15	-7.07	-6.97
11.82	-8.54	-8.34	-8.05	-7.89	-7.60	-7.53	-7.19	-7.10	-7.09	-7.04	-6.90
12.02	-8.49	-8.19	-7.91	-7.73	-7.47	-7.34	-7.10	-7.04	-6.91	-6.90	-6.78
12.22	-8.70	-8.44	-8.10	-7.83	-7.67	-7.50	-7.33	-7.28	-7.11	-7.03	-6.89
12.42	-8.40	-8.19	-7.82	-7.60	-7.31	-7.10	-7.05	-6.90	-6.95	-6.75	-6.70
12.62	-8.50	-8.17	-7.87	-7.68	-7.32	-7.28	-7.09	-7.01	-6.97	-6.82	-6.68
12.82	-8.11	-7.92	-7.64	-7.34	-7.10	-6.93	-6.80	-6.73	-6.61	-6.59	-6.49
13.02	-8.57	-8.19	-7.88	-7.62	-7.48	-7.38	-7.11	-7.07	-6.93	-6.84	-6.67
13.22	-8.38	-8.18	-7.84	-7.47	-7.33	-7.19	-7.10	-6.80	-6.87	-6.72	-6.69
13.42	-8.54	-8.23	-7.91	-7.80	-7.44	-7.20	-7.18	-7.02	-7.02	-6.86	-6.76
13.50	-8.23	-7.89	-7.52	-7.49	-7.16	-6.98	-6.84	-6.75	-6.59	-6.60	-6.46
13.59	-8.45	-8.23	-7.90	-7.65	-7.47	-7.21	-7.15	-6.99	-6.85	-6.83	-6.70
13.68	-8.32	-8.14	-7.79	-7.60	-7.27	-7.13	-6.96	-6.79	-6.77	-6.77	-6.59
13.78	-8.39	-8.16	-7.85	-7.56	-7.33	-7.14	-6.94	-6.85	-6.83	-6.75	-6.66
13.88	-8.40	-8.16	-7.92	-7.58	-7.38	-7.18	-7.05	-6.96	-6.93	-6.76	-6.73
14.00	-8.53	-8.21	-7.90	-7.71	-7.51	-7.29	-7.17	-6.98	-6.92	-6.88	-6.72
14.12	-8.39	-8.22	-7.84	-7.50	-7.32	-7.21	-7.00	-6.96	-6.77	-6.79	-6.62
14.25	-8.35	-8.12	-7.79	-7.64	-7.34	-7.21	-7.02	-6.90	-6.73	-6.76	-6.58
14.40	-8.57	-8.32	-8.02	-7.77	-7.45	-7.30	-7.18	-7.03	-7.06	-6.91	-6.70
14.55	-8.31	-8.12	-7.70	-7.53	-7.38	-7.18	-7.02	-6.85	-6.81	-6.73	-6.57
14.72	-8.28	-8.07	-7.68	-7.50	-7.30	-7.10	-6.90	-6.92	-6.77	-6.58	-6.57
14.90	-8.06	-7.82	-7.57	-7.43	-7.07	-6.90	-6.76	-6.70	-6.64	-6.50	-6.45
15.09	-8.25	-7.92	-7.71	-7.39	-7.16	-6.93	-6.79	-6.65	-6.62	-6.54	-6.36
15.30	-8.15	-7.84	-7.54	-7.32	-7.12	-6.99	-6.72	-6.70	-6.53	-6.48	-6.41
15.53	-8.39	-8.12	-7.87	-7.65	-7.29	-7.26	-6.99	-6.89	-6.76	-6.61	-6.57
15.77	-8.14	-7.89	-7.59	-7.29	-7.06	-7.00	-6.78	-6.69	-6.57	-6.51	-6.41
16.04	-8.37	-8.13	-7.83	-7.63	-7.36	-7.18	-6.98	-6.82	-6.72	-6.64	-6.50
16.32	-8.06	-7.90	-7.63	-7.22	-7.08	-6.87	-6.75	-6.58	-6.44	-6.36	-6.32
16.63	-8.29	-8.01	-7.65	-7.43	-7.22	-7.01	-6.84	-6.71	-6.68	-6.44	-6.43
16.96	-8.11	-7.88	-7.51	-7.29	-7.10	-6.92	-6.74	-6.63	-6.54	-6.46	-6.35
17.31	-8.22	-7.95	-7.57	-7.38	-7.16	-6.97	-6.83	-6.65	-6.50	-6.46	-6.36
17.70	-8.13	-7.94	-7.65	-7.38	-7.14	-6.98	-6.79	-6.62	-6.53	-6.53	-6.33
18.11	-8.01	-7.85	-7.35	-7.15	-6.96	-6.79	-6.61	-6.52	-6.39	-6.31	-6.20
18.56	-8.05	-7.67	-7.46	-7.23	-6.94	-6.71	-6.62	-6.48	-6.33	-6.25	-6.25
19.04	-8.10	-7.76	-7.48	-7.27	-7.01	-6.87	-6.78	-6.61	-6.45	-6.35	-6.22
19.56	-8.13	-7.88	-7.55	-7.20	-7.03	-6.84	-6.70	-6.58	-6.39	-6.39	-6.21
20.12	-8.13	-7.86	-7.63	-7.31	-7.04	-6.86	-6.70	-6.55	-6.46	-6.36	-6.36

20.72	-8.25	-7.85	-7.57	-7.32	-7.08	-6.89	-6.72	-6.60	-6.50	-6.50	-6.24
21.37	-8.16	-7.83	-7.46	-7.24	-6.99	-6.85	-6.62	-6.57	-6.46	-6.31	-6.17
22.08	-8.17	-7.95	-7.65	-7.40	-7.19	-6.95	-6.75	-6.65	-6.49	-6.42	-6.40
22.84	-8.20	-7.90	-7.62	-7.38	-7.16	-6.97	-6.68	-6.61	-6.54	-6.38	-6.24
23.65	-8.00	-7.72	-7.42	-7.22	-6.99	-6.81	-6.59	-6.51	-6.38	-6.36	-6.14
24.54	-7.98	-7.66	-7.38	-7.21	-6.87	-6.71	-6.54	-6.40	-6.24	-6.21	-5.98
25.49	-8.02	-7.77	-7.50	-7.27	-7.01	-6.86	-6.59	-6.54	-6.38	-6.31	-6.11
26.51	-7.83	-7.60	-7.24	-7.04	-6.79	-6.58	-6.46	-6.26	-6.19	-6.03	-5.97
27.62	-8.02	-7.73	-7.39	-7.20	-6.89	-6.68	-6.58	-6.38	-6.37	-6.18	-6.00
28.81	-7.98	-7.71	-7.30	-7.09	-6.83	-6.67	-6.51	-6.33	-6.25	-6.13	-5.98
30.10	-7.86	-7.53	-7.14	-7.01	-6.77	-6.56	-6.32	-6.22	-6.08	-6.00	-5.85
31.49	-7.82	-7.54	-7.28	-6.92	-6.69	-6.52	-6.24	-6.22	-6.09	-5.96	-5.83
32.98	-7.86	-7.61	-7.22	-6.95	-6.71	-6.53	-6.35	-6.27	-6.14	-6.03	-5.84
34.60	-7.83	-7.49	-7.09	-6.86	-6.64	-6.46	-6.30	-6.18	-6.00	-5.91	-5.80
36.34	-7.84	-7.52	-7.21	-7.00	-6.74	-6.54	-6.36	-6.22	-6.10	-6.01	-5.81
38.21	-7.61	-7.34	-6.95	-6.78	-6.55	-6.35	-6.18	-5.98	-5.92	-5.77	-5.63
40.24	-7.84	-7.53	-7.18	-6.84	-6.65	-6.42	-6.20	-6.07	-6.02	-5.82	-5.72
42.42	-7.67	-7.32	-6.97	-6.78	-6.51	-6.21	-5.98	-5.87	-5.79	-5.68	-5.55
44.78	-7.73	-7.44	-7.22	-6.84	-6.65	-6.43	-6.19	-6.06	-5.96	-5.80	-5.70
47.32	-7.60	-7.36	-6.90	-6.56	-6.52	-6.26	-6.11	-5.91	-5.79	-5.63	-5.57
50.06	-7.59	-7.39	-7.19	-6.87	-6.57	-6.39	-6.18	-5.96	-5.84	-5.79	-5.68
53.01	-7.70	-7.41	-7.08	-6.84	-6.65	-6.39	-6.23	-5.99	-5.88	-5.78	-5.59
56.20	-7.66	-7.41	-7.05	-6.83	-6.52	-6.33	-6.11	-6.00	-5.87	-5.79	-5.61
59.64	-7.71	-7.42	-7.05	-6.83	-6.61	-6.49	-6.24	-6.03	-5.90	-5.74	-5.64
63.34	-7.37	-7.20	-6.84	-6.63	-6.36	-6.16	-6.00	-5.83	-5.64	-5.53	-5.39
67.34	-7.56	-7.29	-7.02	-6.71	-6.42	-6.20	-6.00	-5.84	-5.77	-5.61	-5.49
71.65	-7.66	-7.37	-7.03	-6.77	-6.48	-6.34	-6.13	-5.91	-5.83	-5.67	-5.49
76.30	-7.78	-7.43	-7.18	-6.89	-6.61	-6.40	-6.24	-6.02	-5.86	-5.69	-5.56
81.32	-7.71	-7.45	-7.07	-6.88	-6.57	-6.32	-6.10	-5.98	-5.77	-5.65	-5.55
86.73	-7.73	-7.49	-7.12	-6.76	-6.57	-6.29	-6.14	-5.90	-5.81	-5.63	-5.50
92.56	-7.57	-7.29	-7.01	-6.74	-6.44	-6.20	-5.96	-5.78	-5.67	-5.56	-5.38
98.85	-7.57	-7.29	-6.91	-6.70	-6.38	-6.22	-6.00	-5.79	-5.59	-5.48	-5.27
105.64	-7.54	-7.29	-6.91	-6.60	-6.36	-6.18	-5.96	-5.82	-5.62	-5.43	-5.31
112.96	-7.67	-7.36	-6.98	-6.78	-6.54	-6.23	-5.99	-5.78	-5.67	-5.57	-5.33
120.85	-7.36	-7.07	-6.79	-6.42	-6.16	-5.92	-5.76	-5.49	-5.35	-5.14	-5.04
129.36	-7.77	-7.46	-7.12	-6.82	-6.56	-6.35	-6.02	-5.96	-5.72	-5.57	-5.34
138.55	-7.57	-7.24	-6.80	-6.62	-6.34	-6.03	-5.81	-5.68	-5.47	-5.28	-5.11
148.45	-7.58	-7.31	-6.99	-6.53	-6.33	-6.15	-5.90	-5.64	-5.47	-5.29	-5.06
159.13	-7.52	-7.17	-6.81	-6.60	-6.32	-6.09	-5.83	-5.62	-5.47	-5.36	-5.05
170.65	-7.57	-7.18	-6.77	-6.62	-6.23	-6.07	-5.85	-5.54	-5.44	-5.20	-4.96
183.07	-7.39	-7.08	-6.72	-6.38	-6.14	-5.92	-5.71	-5.43	-5.25	-5.07	-4.82
196.47	-7.41	-7.10	-6.87	-6.55	-6.20	-5.96	-5.70	-5.52	-5.28	-5.14	-4.82
210.91	-7.30	-7.04	-6.65	-6.39	-6.05	-5.89	-5.49	-5.37	-5.20	-4.96	-4.69
226.50	-7.30	-6.92	-6.63	-6.38	-6.10	-5.83	-5.53	-5.26	-5.09	-4.89	-4.72
243.31	-7.24	-6.94	-6.64	-6.33	-6.06	-5.80	-5.44	-5.33	-5.04	-4.90	-4.56
261.43	-7.42	-7.12	-6.77	-6.49	-6.12	-5.94	-5.59	-5.31	-5.16	-4.93	-4.63
280.98	-7.19	-6.93	-6.59	-6.30	-6.00	-5.68	-5.41	-5.16	-4.85	-4.66	-4.40
302.07	-7.13	-6.77	-6.32	-6.14	-5.77	-5.60	-5.27	-5.09	-4.89	-4.63	-4.36
324.81	-7.09	-6.78	-6.49	-6.13	-5.75	-5.50	-5.20	-4.98	-4.75	-4.65	-4.20
349.33	-7.10	-6.84	-6.40	-6.07	-5.63	-5.46	-5.19	-4.95	-4.69	-4.45	-4.22

375.79	-7.11	-6.75	-6.48	-6.16	-5.78	-5.51	-5.24	-4.94	-4.74	-4.44	-4.10
404.31	-6.99	-6.61	-6.29	-5.97	-5.71	-5.34	-5.02	-4.74	-4.60	-4.30	-3.94
435.08	-7.14	-6.82	-6.41	-6.17	-5.83	-5.59	-5.21	-4.91	-4.65	-4.41	-4.03
468.27	-6.92	-6.74	-6.27	-5.98	-5.63	-5.41	-5.04	-4.84	-4.50	-4.32	-3.91
504.06	-6.88	-6.56	-6.31	-5.95	-5.56	-5.31	-4.91	-4.63	-4.42	-4.19	-3.78
542.66	-6.94	-6.48	-6.15	-5.81	-5.52	-5.30	-4.82	-4.62	-4.39	-4.05	-3.75
584.29	-6.88	-6.55	-6.12	-5.88	-5.52	-5.24	-4.88	-4.67	-4.33	-3.96	-3.63
629.18	-6.72	-6.42	-6.01	-5.69	-5.32	-4.97	-4.72	-4.44	-4.14	-3.86	-3.52
677.61	-6.79	-6.53	-6.19	-5.81	-5.56	-5.20	-4.87	-4.54	-4.24	-3.90	-3.59
729.83	-6.67	-6.28	-5.90	-5.60	-5.19	-4.85	-4.56	-4.26	-4.01	-3.70	-3.27
786.16	-6.76	-6.32	-6.00	-5.66	-5.35	-5.08	-4.77	-4.35	-4.06	-3.76	-3.36
846.90	-6.64	-6.30	-5.97	-5.66	-5.21	-4.98	-4.64	-4.39	-4.04	-3.74	-3.32
912.42	-6.61	-6.27	-5.85	-5.49	-5.19	-4.91	-4.47	-4.23	-3.88	-3.59	-3.27
983.08	-6.46	-6.29	-5.90	-5.59	-5.17	-4.85	-4.53	-4.16	-3.91	-3.56	-3.14
1059.29	-6.70	-6.51	-5.98	-5.68	-5.33	-5.01	-4.67	-4.36	-4.04	-3.80	-3.35
1141.48	-6.61	-6.23	-5.87	-5.53	-5.16	-4.86	-4.53	-4.16	-3.83	-3.47	-3.07
1230.13	-6.59	-6.33	-5.96	-5.59	-5.27	-4.93	-4.67	-4.21	-3.92	-3.64	-3.17
1325.73	-6.72	-6.38	-5.99	-5.60	-5.35	-5.07	-4.57	-4.33	-3.95	-3.62	-3.28
1428.84	-6.68	-6.44	-6.11	-5.69	-5.38	-5.07	-4.66	-4.41	-4.01	-3.64	-3.27
1540.05	-6.93	-6.62	-6.14	-5.81	-5.45	-5.10	-4.75	-4.41	-4.07	-3.78	-3.35
1659.99	-6.87	-6.52	-6.15	-5.87	-5.48	-5.25	-4.83	-4.51	-4.09	-3.81	-3.32
1789.34	-6.92	-6.60	-6.12	-5.88	-5.52	-5.18	-4.76	-4.42	-4.07	-3.73	-3.29
1928.85	-6.92	-6.70	-6.24	-5.92	-5.53	-5.23	-4.81	-4.44	-4.07	-3.77	-3.36
2079.31	-7.14	-6.78	-6.48	-6.15	-5.78	-5.40	-4.99	-4.71	-4.31	-3.98	-3.57
2241.59	-7.01	-6.72	-6.42	-6.04	-5.71	-5.37	-4.96	-4.58	-4.22	-3.95	-3.46
2416.60	-7.17	-6.97	-6.62	-6.21	-5.87	-5.48	-5.16	-4.86	-4.45	-4.07	-3.59
2605.36	-7.25	-7.00	-6.64	-6.29	-5.91	-5.59	-5.23	-4.84	-4.45	-4.04	-3.66
2808.94	-7.26	-6.94	-6.70	-6.40	-5.92	-5.59	-5.24	-4.93	-4.41	-4.07	-3.72
3028.50	-7.32	-7.06	-6.68	-6.43	-6.04	-5.75	-5.30	-4.98	-4.56	-4.26	-3.72
3265.30	-7.41	-7.05	-6.77	-6.47	-6.08	-5.81	-5.46	-5.02	-4.61	-4.24	-3.84
3520.69	-7.29	-7.06	-6.75	-6.43	-6.11	-5.80	-5.37	-4.99	-4.63	-4.32	-3.76
3796.13	-7.22	-7.10	-6.70	-6.46	-6.09	-5.82	-5.43	-4.99	-4.61	-4.29	-3.82

Wavelength (nm) / Time (ps)	522.06	523.49	524.91	526.34	527.76	529.18	530.61	532.03	533.46	534.88	536.30
-3.78	0.03	0.05	0.10	0.01	-0.03	0.02	0.01	0.07	0.04	-0.05	0.04
-3.28	0.07	0.05	0.03	0.05	0.08	0.00	0.07	0.02	-0.05	0.01	-0.02
-2.78	-0.04	-0.09	-0.10	-0.02	-0.08	-0.03	-0.06	0.03	-0.02	0.03	0.06
-2.28	0.02	0.07	0.05	0.08	0.05	0.09	0.05	-0.02	0.06	0.04	0.01
-1.78	-0.08	-0.10	0.00	-0.09	-0.01	-0.11	0.04	-0.05	-0.01	0.06	-0.04
-1.28	-0.01	-0.05	-0.06	-0.05	-0.01	0.00	-0.10	-0.09	0.00	-0.03	0.00
-0.78	0.04	0.09	-0.05	0.05	-0.01	-0.01	-0.04	-0.02	-0.04	-0.04	0.04
-0.28	-0.03	-0.03	0.03	-0.03	0.02	0.05	0.03	0.06	0.01	-0.01	-0.08
-0.18	0.00	-0.02	0.05	-0.01	-0.05	0.03	0.00	-0.04	-0.03	0.00	-0.03
-0.08	0.06	0.06	0.00	0.05	0.15	0.09	0.01	0.01	-0.02	-0.03	-0.06
0.02	-0.06	0.02	-0.09	0.00	0.07	0.03	0.00	-0.03	0.00	-0.01	0.02
0.12	0.04	0.09	0.01	0.09	0.01	0.06	-0.06	0.00	0.11	0.05	0.04
0.22	0.02	0.10	0.02	-0.02	0.00	0.05	-0.01	-0.03	0.02	0.03	-0.05

0.27	0.01	0.02	-0.10	0.03	-0.07	-0.02	-0.04	0.01	0.00	-0.08	-0.11
0.32	0.01	0.00	0.03	0.13	0.10	0.03	0.08	0.05	0.01	0.05	0.05
0.37	-0.13	0.03	0.01	-0.02	-0.01	0.07	-0.12	-0.04	-0.01	0.00	-0.16
0.42	-0.19	-0.18	-0.18	-0.12	-0.04	-0.16	-0.09	-0.13	-0.12	-0.07	-0.16
0.47	-0.08	0.01	-0.12	0.00	-0.08	0.07	-0.07	-0.15	-0.11	-0.07	-0.09
0.52	-0.08	0.03	0.02	-0.03	-0.01	-0.08	-0.16	-0.09	-0.08	-0.01	-0.08
0.57	-0.09	0.09	0.02	0.00	0.05	0.06	0.00	-0.04	-0.02	0.03	-0.07
0.62	0.01	-0.03	0.00	0.06	0.00	0.03	-0.11	-0.06	-0.02	-0.05	-0.01
0.67	-0.05	-0.11	-0.14	-0.09	0.00	0.05	-0.06	-0.05	-0.06	-0.06	-0.14
0.72	-0.14	-0.08	-0.10	-0.08	-0.15	-0.12	-0.02	-0.06	-0.07	-0.07	-0.12
0.77	-0.11	-0.02	0.01	-0.02	-0.03	0.02	-0.02	-0.01	-0.06	0.08	-0.06
0.82	-0.06	0.07	-0.04	-0.02	0.09	0.06	0.02	0.02	0.04	0.04	-0.04
0.87	-0.06	-0.05	0.01	-0.03	-0.06	-0.07	-0.06	0.00	-0.01	0.01	-0.12
0.92	0.02	0.02	0.04	0.03	0.09	0.03	-0.02	0.09	0.08	-0.02	-0.02
0.97	0.05	-0.02	-0.04	-0.01	-0.01	-0.07	-0.10	0.02	-0.05	-0.03	-0.02
1.02	-0.06	0.04	-0.03	-0.04	0.08	0.01	0.03	0.00	-0.06	0.03	0.00
1.07	0.13	0.11	0.05	0.22	0.01	0.10	0.06	-0.01	-0.10	0.12	0.05
1.12	0.01	-0.04	-0.24	-0.05	0.11	0.03	-0.15	-0.06	0.01	-0.01	-0.14
1.17	0.15	0.07	0.00	0.11	0.14	0.06	-0.07	-0.03	-0.07	-0.01	0.00
1.22	-28.39	-23.00	-16.70	-10.28	-4.45	-0.20	2.49	3.40	3.03	1.90	0.48
1.27	37.34	27.42	17.92	9.79	2.94	-2.91	-7.73	-11.59	-14.53	-17.77	-20.17
1.32	-43.99	-28.41	-12.34	4.07	19.51	33.45	45.40	55.07	62.83	67.77	71.83
1.37	-17.58	-18.02	-18.07	-18.00	-18.26	-18.99	-20.22	-22.20	-25.09	-28.80	-33.32
1.42	-17.85	-18.20	-18.13	-17.84	-17.42	-16.85	-15.92	-14.84	-13.78	-12.93	-12.16
1.47	-15.11	-14.82	-14.74	-14.80	-14.99	-15.32	-15.66	-15.65	-15.65	-15.47	-14.94
1.52	-16.07	-15.79	-15.58	-15.35	-15.06	-14.93	-14.82	-14.76	-14.73	-14.60	-14.56
1.57	-14.76	-15.27	-15.76	-15.97	-16.20	-16.18	-15.91	-15.34	-14.73	-14.06	-13.30
1.62	-13.80	-13.61	-13.55	-13.45	-13.69	-13.83	-14.04	-14.03	-13.94	-13.60	-13.21
1.67	-14.89	-14.73	-14.46	-14.00	-13.53	-13.08	-12.79	-12.28	-12.01	-11.60	-11.30
1.72	-12.44	-12.70	-13.03	-13.22	-13.49	-13.58	-13.66	-13.37	-13.03	-12.50	-11.94
1.77	-13.62	-13.19	-12.93	-12.57	-12.33	-12.27	-12.15	-11.95	-11.76	-11.55	-11.27
1.82	-13.27	-13.37	-13.46	-13.32	-13.12	-12.72	-12.32	-11.81	-11.18	-10.63	-9.97
1.87	-12.03	-11.96	-11.99	-12.12	-12.20	-12.28	-12.23	-12.05	-11.72	-11.04	-10.49
1.92	-12.94	-12.64	-12.27	-12.00	-11.59	-11.26	-10.96	-10.69	-10.34	-10.03	-9.64
1.97	-11.87	-12.15	-12.40	-12.43	-12.49	-12.37	-12.01	-11.53	-10.97	-10.20	-9.62
2.02	-11.76	-11.43	-11.27	-11.17	-10.99	-11.02	-10.98	-10.66	-10.43	-10.00	-9.58
2.07	-12.22	-11.99	-11.63	-11.24	-10.98	-10.66	-10.31	-9.83	-9.55	-9.08	-8.68
2.12	-10.90	-11.04	-11.14	-11.17	-11.18	-11.20	-11.00	-10.65	-10.18	-9.56	-8.90
2.17	-11.11	-10.90	-10.72	-10.44	-10.44	-10.28	-10.16	-10.03	-9.75	-9.33	-8.91
2.22	-11.74	-11.70	-11.66	-11.43	-11.23	-10.87	-10.42	-9.88	-9.29	-8.66	-7.97
2.27	-10.66	-10.58	-10.62	-10.54	-10.60	-10.64	-10.45	-10.27	-9.81	-9.20	-8.54
2.32	-11.19	-11.01	-10.67	-10.33	-10.12	-9.88	-9.59	-9.33	-8.94	-8.46	-8.00
2.37	-10.80	-10.78	-10.88	-10.76	-10.79	-10.51	-10.27	-9.82	-9.26	-8.53	-7.88
2.42	-10.53	-10.42	-10.24	-10.09	-10.07	-10.05	-9.94	-9.63	-9.24	-8.72	-8.04
2.47	-11.00	-10.75	-10.58	-10.32	-10.06	-9.77	-9.38	-9.10	-8.64	-8.06	-7.63
2.52	-10.42	-10.36	-10.53	-10.42	-10.41	-10.27	-9.98	-9.56	-9.07	-8.30	-7.69
2.57	-10.56	-10.35	-10.20	-9.94	-9.80	-9.67	-9.45	-9.19	-8.80	-8.27	-7.74
2.62	-10.92	-10.81	-10.60	-10.29	-10.05	-9.79	-9.30	-8.91	-8.31	-7.79	-7.22
2.67	-9.94	-9.98	-9.88	-9.94	-9.88	-9.69	-9.49	-9.04	-8.55	-7.98	-7.29
2.72	-10.54	-10.19	-9.97	-9.71	-9.54	-9.22	-9.01	-8.73	-8.33	-7.81	-7.29

2.77	-10.19	-10.15	-10.11	-9.92	-9.74	-9.57	-9.21	-8.78	-8.22	-7.49	-6.86
2.82	-9.77	-9.74	-9.56	-9.55	-9.36	-9.27	-9.05	-8.66	-8.35	-7.74	-7.13
2.87	-10.09	-9.99	-9.82	-9.53	-9.34	-9.07	-8.69	-8.34	-7.92	-7.26	-6.82
2.92	-9.84	-9.89	-9.80	-9.68	-9.53	-9.42	-9.07	-8.66	-8.17	-7.47	-6.86
2.97	-9.77	-9.50	-9.32	-9.09	-9.01	-8.80	-8.52	-8.24	-7.84	-7.32	-6.75
3.02	-10.03	-9.81	-9.69	-9.40	-9.23	-8.99	-8.64	-8.21	-7.77	-7.17	-6.57
3.07	-9.54	-9.54	-9.45	-9.35	-9.22	-9.08	-8.84	-8.43	-7.91	-7.23	-6.64
3.12	-9.57	-9.42	-9.20	-8.99	-8.80	-8.61	-8.41	-7.97	-7.62	-7.11	-6.53
3.17	-9.67	-9.64	-9.50	-9.37	-9.17	-8.85	-8.50	-8.07	-7.57	-6.94	-6.32
3.22	-9.44	-9.36	-9.19	-9.14	-8.99	-8.81	-8.52	-8.20	-7.79	-7.17	-6.59
3.42	-9.59	-9.52	-9.31	-9.16	-8.86	-8.53	-8.25	-7.88	-7.31	-6.69	-6.02
3.62	-9.36	-9.12	-9.06	-8.97	-8.84	-8.73	-8.49	-8.03	-7.51	-6.81	-6.19
3.82	-9.45	-9.29	-9.15	-8.99	-8.70	-8.45	-8.09	-7.73	-7.15	-6.58	-6.02
4.02	-9.11	-8.95	-8.95	-8.77	-8.73	-8.46	-8.16	-7.72	-7.21	-6.65	-5.93
4.22	-9.15	-9.02	-8.78	-8.60	-8.52	-8.19	-7.82	-7.43	-6.97	-6.35	-5.71
4.42	-8.84	-8.73	-8.62	-8.37	-8.31	-8.14	-7.86	-7.42	-6.96	-6.25	-5.60
4.62	-8.95	-8.81	-8.62	-8.47	-8.28	-7.94	-7.70	-7.27	-6.77	-6.16	-5.59
4.82	-8.82	-8.62	-8.56	-8.37	-8.19	-7.97	-7.71	-7.28	-6.81	-6.16	-5.54
5.02	-8.68	-8.52	-8.35	-8.11	-7.97	-7.72	-7.47	-7.02	-6.55	-5.92	-5.28
5.22	-8.75	-8.52	-8.44	-8.22	-8.08	-7.81	-7.52	-7.10	-6.59	-5.92	-5.23
5.42	-8.60	-8.53	-8.38	-8.09	-7.94	-7.63	-7.32	-6.95	-6.41	-5.80	-5.11
5.62	-8.37	-8.21	-8.09	-7.95	-7.71	-7.46	-7.20	-6.79	-6.30	-5.53	-4.96
5.82	-8.23	-8.18	-8.02	-7.76	-7.62	-7.32	-7.11	-6.64	-6.20	-5.53	-4.91
6.02	-8.14	-8.04	-7.94	-7.71	-7.58	-7.37	-7.01	-6.55	-6.08	-5.39	-4.77
6.22	-8.15	-7.93	-7.82	-7.61	-7.46	-7.22	-6.99	-6.49	-6.04	-5.32	-4.73
6.42	-8.09	-8.06	-7.81	-7.59	-7.43	-7.17	-6.81	-6.49	-5.88	-5.24	-4.69
6.62	-8.06	-7.98	-7.79	-7.56	-7.34	-7.10	-6.78	-6.30	-5.85	-5.19	-4.50
6.82	-7.93	-7.75	-7.65	-7.43	-7.29	-7.05	-6.72	-6.20	-5.71	-5.05	-4.45
7.02	-7.83	-7.63	-7.55	-7.32	-7.21	-6.91	-6.64	-6.22	-5.67	-5.00	-4.43
7.22	-7.77	-7.72	-7.57	-7.32	-7.17	-6.91	-6.63	-6.15	-5.64	-4.91	-4.40
7.42	-7.84	-7.62	-7.57	-7.28	-7.12	-6.89	-6.51	-6.14	-5.64	-4.94	-4.31
7.62	-7.61	-7.58	-7.48	-7.19	-6.91	-6.71	-6.43	-5.96	-5.50	-4.76	-4.13
7.82	-7.52	-7.42	-7.27	-7.03	-6.83	-6.57	-6.34	-5.82	-5.35	-4.78	-4.08
8.02	-7.47	-7.39	-7.28	-7.05	-6.80	-6.65	-6.29	-5.87	-5.39	-4.76	-4.10
8.22	-7.38	-7.22	-7.09	-6.90	-6.75	-6.49	-6.20	-5.80	-5.28	-4.70	-4.03
8.42	-7.42	-7.27	-7.11	-6.96	-6.70	-6.51	-6.19	-5.78	-5.33	-4.67	-4.02
8.62	-7.30	-7.19	-7.07	-6.83	-6.66	-6.42	-6.11	-5.66	-5.24	-4.57	-3.94
8.82	-7.40	-7.18	-7.19	-6.80	-6.70	-6.43	-6.08	-5.68	-5.19	-4.59	-3.90
9.02	-7.19	-7.03	-7.01	-6.71	-6.55	-6.30	-6.06	-5.66	-5.11	-4.47	-3.83
9.22	-7.37	-7.19	-7.08	-6.80	-6.69	-6.44	-6.03	-5.67	-5.14	-4.43	-3.73
9.42	-7.17	-7.06	-6.98	-6.70	-6.48	-6.22	-5.92	-5.53	-4.97	-4.38	-3.68
9.62	-7.09	-7.02	-6.93	-6.66	-6.50	-6.20	-5.94	-5.51	-4.94	-4.35	-3.72
9.82	-7.12	-7.00	-6.78	-6.60	-6.43	-6.16	-5.87	-5.37	-4.91	-4.24	-3.58
10.02	-7.09	-7.06	-6.84	-6.66	-6.47	-6.15	-5.82	-5.44	-4.97	-4.27	-3.68
10.22	-7.07	-6.93	-6.77	-6.61	-6.36	-6.14	-5.79	-5.38	-4.85	-4.23	-3.61
10.42	-7.06	-6.88	-6.82	-6.59	-6.30	-6.09	-5.70	-5.33	-4.87	-4.23	-3.53
10.62	-7.07	-6.83	-6.75	-6.59	-6.36	-6.08	-5.74	-5.31	-4.86	-4.15	-3.49
10.82	-6.89	-6.84	-6.70	-6.41	-6.23	-6.04	-5.80	-5.26	-4.71	-4.10	-3.52
11.02	-6.90	-6.77	-6.53	-6.38	-6.30	-5.91	-5.62	-5.24	-4.69	-4.05	-3.44
11.22	-6.89	-6.74	-6.56	-6.37	-6.20	-5.94	-5.64	-5.20	-4.72	-4.08	-3.49

11.42	-6.76	-6.54	-6.48	-6.25	-6.04	-5.81	-5.53	-5.07	-4.57	-4.06	-3.43
11.62	-6.79	-6.61	-6.49	-6.39	-6.20	-5.93	-5.61	-5.13	-4.70	-4.15	-3.41
11.82	-6.79	-6.75	-6.48	-6.30	-6.15	-5.87	-5.56	-5.14	-4.63	-3.92	-3.35
12.02	-6.68	-6.51	-6.48	-6.29	-6.06	-5.78	-5.52	-5.11	-4.59	-3.94	-3.31
12.22	-6.77	-6.69	-6.57	-6.31	-6.18	-5.86	-5.59	-5.10	-4.64	-3.99	-3.30
12.42	-6.59	-6.46	-6.34	-6.10	-5.95	-5.75	-5.33	-4.92	-4.51	-3.86	-3.28
12.62	-6.58	-6.49	-6.34	-6.15	-5.93	-5.70	-5.37	-4.96	-4.47	-3.83	-3.27
12.82	-6.36	-6.27	-6.08	-5.92	-5.67	-5.39	-5.22	-4.75	-4.33	-3.60	-3.03
13.02	-6.66	-6.41	-6.29	-6.12	-6.05	-5.66	-5.34	-4.90	-4.36	-3.80	-3.13
13.22	-6.50	-6.27	-6.26	-6.11	-5.84	-5.58	-5.30	-4.90	-4.42	-3.75	-3.14
13.42	-6.63	-6.40	-6.27	-6.07	-5.93	-5.62	-5.43	-4.95	-4.46	-3.76	-3.16
13.50	-6.40	-6.22	-6.13	-5.91	-5.73	-5.42	-5.24	-4.84	-4.23	-3.65	-3.04
13.59	-6.64	-6.44	-6.31	-6.07	-5.86	-5.66	-5.32	-4.88	-4.29	-3.66	-3.14
13.68	-6.47	-6.30	-6.20	-5.99	-5.82	-5.44	-5.19	-4.77	-4.30	-3.62	-3.01
13.78	-6.53	-6.35	-6.22	-6.01	-5.85	-5.56	-5.32	-4.80	-4.43	-3.68	-3.09
13.88	-6.52	-6.38	-6.25	-5.97	-5.78	-5.60	-5.26	-4.96	-4.43	-3.74	-3.16
14.00	-6.59	-6.43	-6.33	-6.18	-5.96	-5.63	-5.32	-4.98	-4.38	-3.69	-3.05
14.12	-6.53	-6.39	-6.18	-6.04	-5.82	-5.60	-5.27	-4.76	-4.33	-3.80	-3.09
14.25	-6.51	-6.28	-6.25	-6.02	-5.80	-5.54	-5.23	-4.83	-4.33	-3.70	-3.08
14.40	-6.70	-6.52	-6.36	-6.09	-5.92	-5.63	-5.27	-4.89	-4.45	-3.70	-3.08
14.55	-6.52	-6.28	-6.22	-5.91	-5.74	-5.51	-5.17	-4.72	-4.29	-3.61	-3.03
14.72	-6.41	-6.27	-6.08	-5.85	-5.75	-5.43	-5.08	-4.64	-4.19	-3.58	-2.96
14.90	-6.31	-6.10	-6.02	-5.84	-5.62	-5.30	-5.01	-4.59	-4.15	-3.53	-2.93
15.09	-6.24	-6.12	-6.06	-5.74	-5.62	-5.33	-4.92	-4.56	-4.14	-3.56	-2.90
15.30	-6.33	-6.13	-6.00	-5.78	-5.57	-5.34	-5.05	-4.65	-4.17	-3.49	-2.88
15.53	-6.46	-6.21	-6.12	-5.97	-5.65	-5.44	-5.17	-4.73	-4.21	-3.60	-2.93
15.77	-6.29	-6.13	-6.03	-5.80	-5.58	-5.35	-5.03	-4.65	-4.08	-3.53	-2.85
16.04	-6.40	-6.22	-6.15	-5.92	-5.67	-5.44	-5.14	-4.63	-4.04	-3.43	-2.91
16.32	-6.22	-6.05	-5.89	-5.63	-5.54	-5.22	-4.97	-4.59	-4.07	-3.46	-2.83
16.63	-6.32	-6.19	-6.05	-5.76	-5.65	-5.39	-5.09	-4.60	-4.04	-3.52	-2.87
16.96	-6.26	-6.09	-5.94	-5.66	-5.56	-5.15	-4.86	-4.44	-4.06	-3.41	-2.74
17.31	-6.29	-6.00	-5.89	-5.68	-5.57	-5.32	-4.92	-4.58	-4.09	-3.42	-2.83
17.70	-6.26	-6.10	-5.94	-5.70	-5.49	-5.34	-4.91	-4.51	-4.00	-3.42	-2.71
18.11	-6.12	-5.93	-5.78	-5.54	-5.42	-5.19	-4.89	-4.33	-3.92	-3.24	-2.66
18.56	-6.05	-5.91	-5.86	-5.53	-5.44	-5.10	-4.86	-4.44	-3.89	-3.38	-2.69
19.04	-6.10	-6.00	-5.86	-5.54	-5.42	-5.27	-4.88	-4.43	-3.89	-3.38	-2.69
19.56	-6.11	-5.91	-5.91	-5.55	-5.44	-5.17	-4.79	-4.35	-3.90	-3.24	-2.62
20.12	-6.16	-5.94	-5.84	-5.67	-5.48	-5.15	-4.89	-4.43	-3.84	-3.22	-2.53
20.72	-6.20	-6.03	-5.79	-5.64	-5.39	-5.12	-4.84	-4.31	-3.89	-3.15	-2.59
21.37	-6.11	-5.88	-5.70	-5.53	-5.38	-5.08	-4.74	-4.32	-3.82	-3.16	-2.49
22.08	-6.16	-6.04	-5.92	-5.65	-5.53	-5.17	-4.89	-4.45	-3.83	-3.19	-2.66
22.84	-6.18	-6.04	-5.90	-5.55	-5.44	-5.23	-4.89	-4.39	-3.88	-3.25	-2.60
23.65	-6.07	-5.88	-5.69	-5.61	-5.29	-5.05	-4.71	-4.31	-3.72	-3.08	-2.54
24.54	-5.89	-5.66	-5.69	-5.40	-5.21	-4.90	-4.59	-4.15	-3.69	-3.05	-2.35
25.49	-6.04	-5.87	-5.71	-5.51	-5.24	-4.92	-4.65	-4.19	-3.75	-2.98	-2.31
26.51	-5.85	-5.64	-5.56	-5.20	-5.07	-4.78	-4.57	-4.11	-3.65	-2.97	-2.34
27.62	-5.88	-5.73	-5.59	-5.35	-5.14	-4.84	-4.51	-4.04	-3.67	-2.86	-2.24
28.81	-5.87	-5.68	-5.57	-5.43	-5.11	-4.85	-4.46	-4.05	-3.56	-2.91	-2.22
30.10	-5.74	-5.52	-5.36	-5.23	-5.01	-4.66	-4.38	-3.93	-3.55	-2.81	-2.21
31.49	-5.77	-5.54	-5.45	-5.19	-4.91	-4.67	-4.31	-3.90	-3.43	-2.75	-2.08

32.98	-5.70	-5.59	-5.40	-5.19	-5.00	-4.70	-4.31	-3.90	-3.42	-2.73	-2.03
34.60	-5.70	-5.48	-5.31	-5.03	-4.88	-4.56	-4.28	-3.83	-3.33	-2.64	-1.91
36.34	-5.71	-5.48	-5.37	-5.12	-4.93	-4.55	-4.24	-3.79	-3.26	-2.65	-2.02
38.21	-5.59	-5.38	-5.20	-4.91	-4.75	-4.46	-4.15	-3.70	-3.18	-2.49	-1.75
40.24	-5.62	-5.39	-5.34	-4.99	-4.89	-4.55	-4.24	-3.76	-3.16	-2.51	-1.87
42.42	-5.49	-5.21	-5.10	-4.87	-4.72	-4.38	-4.05	-3.60	-3.12	-2.48	-1.73
44.78	-5.53	-5.42	-5.23	-5.02	-4.79	-4.51	-4.11	-3.66	-3.15	-2.43	-1.82
47.32	-5.48	-5.23	-5.07	-4.80	-4.55	-4.33	-3.96	-3.59	-3.11	-2.48	-1.73
50.06	-5.49	-5.29	-5.17	-4.90	-4.72	-4.44	-4.05	-3.60	-2.98	-2.38	-1.75
53.01	-5.65	-5.24	-5.18	-4.89	-4.68	-4.39	-4.03	-3.54	-3.09	-2.37	-1.75
56.20	-5.44	-5.27	-5.02	-4.83	-4.61	-4.22	-3.98	-3.48	-2.94	-2.22	-1.60
59.64	-5.44	-5.23	-5.06	-4.86	-4.53	-4.22	-3.81	-3.37	-2.91	-2.22	-1.46
63.34	-5.31	-5.04	-4.89	-4.69	-4.41	-4.10	-3.81	-3.26	-2.85	-2.04	-1.51
67.34	-5.37	-5.16	-4.99	-4.70	-4.53	-4.16	-3.80	-3.35	-2.78	-2.18	-1.45
71.65	-5.38	-5.14	-4.91	-4.66	-4.43	-4.11	-3.76	-3.28	-2.69	-2.00	-1.42
76.30	-5.45	-5.13	-5.03	-4.81	-4.53	-4.20	-3.76	-3.29	-2.78	-2.03	-1.30
81.32	-5.24	-5.13	-4.92	-4.61	-4.36	-4.07	-3.69	-3.14	-2.55	-2.02	-1.15
86.73	-5.38	-5.07	-4.82	-4.54	-4.33	-3.93	-3.63	-3.08	-2.43	-1.76	-1.03
92.56	-5.20	-5.00	-4.82	-4.47	-4.21	-3.87	-3.46	-2.98	-2.33	-1.68	-0.98
98.85	-5.17	-4.93	-4.79	-4.49	-4.17	-3.78	-3.41	-2.95	-2.46	-1.66	-0.94
105.64	-5.13	-4.88	-4.70	-4.45	-4.17	-3.83	-3.36	-2.90	-2.32	-1.58	-0.82
112.96	-5.22	-4.96	-4.73	-4.36	-4.05	-3.72	-3.35	-2.76	-2.19	-1.54	-0.79
120.85	-4.84	-4.72	-4.45	-4.10	-3.77	-3.52	-3.06	-2.46	-1.93	-1.15	-0.53
129.36	-5.17	-4.85	-4.61	-4.30	-4.06	-3.61	-3.17	-2.66	-1.98	-1.28	-0.49
138.55	-4.86	-4.65	-4.41	-4.18	-3.89	-3.35	-3.06	-2.47	-1.85	-1.12	-0.33
148.45	-4.90	-4.60	-4.36	-4.10	-3.77	-3.33	-2.87	-2.49	-1.80	-1.05	-0.22
159.13	-4.89	-4.64	-4.37	-4.00	-3.78	-3.34	-2.92	-2.32	-1.73	-0.92	-0.16
170.65	-4.71	-4.49	-4.24	-3.93	-3.58	-3.20	-2.74	-2.21	-1.59	-0.70	-0.04
183.07	-4.72	-4.36	-4.09	-3.78	-3.46	-3.11	-2.57	-2.04	-1.38	-0.64	0.23
196.47	-4.64	-4.33	-4.14	-3.76	-3.46	-3.02	-2.59	-1.95	-1.29	-0.45	0.26
210.91	-4.49	-4.19	-3.89	-3.61	-3.24	-2.80	-2.36	-1.76	-1.15	-0.24	0.51
226.50	-4.42	-4.12	-3.84	-3.49	-3.10	-2.66	-2.20	-1.68	-0.97	-0.10	0.72
243.31	-4.40	-4.10	-3.72	-3.42	-3.09	-2.56	-2.09	-1.50	-0.84	-0.02	0.74
261.43	-4.31	-4.10	-3.79	-3.45	-3.03	-2.52	-2.05	-1.37	-0.71	0.15	0.87
280.98	-4.14	-3.89	-3.58	-3.21	-2.80	-2.34	-1.85	-1.18	-0.51	0.31	1.12
302.07	-4.05	-3.75	-3.46	-3.03	-2.60	-2.26	-1.68	-1.10	-0.31	0.48	1.32
324.81	-4.01	-3.63	-3.35	-2.96	-2.61	-2.00	-1.54	-0.94	-0.15	0.64	1.53
349.33	-3.98	-3.58	-3.28	-2.85	-2.39	-1.94	-1.52	-0.75	0.06	0.77	1.66
375.79	-3.90	-3.55	-3.22	-2.77	-2.26	-1.86	-1.32	-0.76	0.07	0.96	1.78
404.31	-3.68	-3.26	-3.00	-2.47	-2.18	-1.62	-1.11	-0.54	0.29	1.15	1.98
435.08	-3.83	-3.40	-3.05	-2.66	-2.19	-1.67	-1.07	-0.41	0.30	1.21	2.06
468.27	-3.67	-3.30	-2.90	-2.48	-2.05	-1.45	-0.90	-0.26	0.45	1.36	2.31
504.06	-3.56	-3.17	-2.76	-2.30	-1.86	-1.27	-0.79	-0.05	0.78	1.66	2.58
542.66	-3.43	-3.01	-2.71	-2.23	-1.75	-1.15	-0.59	0.06	0.82	1.65	2.60
584.29	-3.38	-2.93	-2.59	-2.16	-1.70	-1.13	-0.52	0.11	1.05	1.93	2.77
629.18	-3.18	-2.84	-2.44	-1.96	-1.50	-0.90	-0.34	0.36	1.17	2.08	2.90
677.61	-3.20	-2.96	-2.49	-1.94	-1.51	-0.91	-0.34	0.44	1.23	2.09	3.09
729.83	-2.98	-2.57	-2.24	-1.70	-1.24	-0.66	-0.03	0.62	1.46	2.37	3.33
786.16	-3.03	-2.66	-2.18	-1.73	-1.26	-0.67	0.05	0.68	1.49	2.40	3.41
846.90	-2.97	-2.59	-2.15	-1.58	-1.06	-0.61	-0.03	0.79	1.59	2.57	3.40

912.42	-2.85	-2.40	-2.12	-1.58	-1.01	-0.53	0.12	0.88	1.64	2.67	3.60
983.08	-2.81	-2.40	-2.01	-1.51	-0.91	-0.48	0.22	0.95	1.77	2.65	3.65
1059.29	-2.93	-2.46	-2.10	-1.63	-1.01	-0.46	0.20	0.94	1.76	2.74	3.62
1141.48	-2.82	-2.39	-1.88	-1.43	-0.87	-0.28	0.34	1.08	1.93	2.89	3.70
1230.13	-2.79	-2.36	-1.96	-1.48	-0.90	-0.37	0.23	1.02	1.85	2.86	3.77
1325.73	-2.88	-2.42	-2.05	-1.46	-0.92	-0.38	0.29	1.13	1.92	2.78	3.78
1428.84	-2.89	-2.49	-2.01	-1.44	-0.95	-0.39	0.20	0.98	1.78	2.82	3.72
1540.05	-2.94	-2.46	-2.03	-1.48	-0.94	-0.32	0.27	1.02	1.86	2.79	3.83
1659.99	-2.94	-2.41	-2.07	-1.47	-0.96	-0.33	0.19	1.05	1.93	2.84	3.84
1789.34	-2.93	-2.37	-2.14	-1.50	-0.94	-0.38	0.26	1.06	1.99	2.90	3.86
1928.85	-2.91	-2.59	-2.05	-1.52	-0.97	-0.46	0.20	1.07	1.98	2.87	3.82
2079.31	-3.08	-2.57	-2.15	-1.65	-1.07	-0.43	0.20	0.97	1.82	2.83	3.82
2241.59	-3.03	-2.59	-2.11	-1.56	-1.04	-0.35	0.18	1.00	1.84	2.81	3.80
2416.60	-3.23	-2.75	-2.25	-1.76	-1.18	-0.60	0.10	0.88	1.73	2.75	3.73
2605.36	-3.22	-2.70	-2.28	-1.74	-1.15	-0.51	0.17	0.89	1.69	2.80	3.72
2808.94	-3.33	-2.74	-2.38	-1.76	-1.22	-0.59	0.02	0.88	1.77	2.73	3.72
3028.50	-3.23	-2.85	-2.34	-1.74	-1.22	-0.68	-0.08	0.86	1.76	2.73	3.68
3265.30	-3.30	-2.72	-2.33	-1.87	-1.16	-0.67	0.03	0.76	1.68	2.65	3.68
3520.69	-3.27	-2.76	-2.43	-1.88	-1.21	-0.58	-0.02	0.84	1.62	2.60	3.67
3796.13	-3.40	-2.81	-2.37	-1.70	-1.29	-0.69	0.00	0.86	1.69	2.74	3.69

Wavelength (nm) / Time (ps)	537.73	539.15	540.58	542.00	543.43	544.85	546.27	547.70	549.12	550.55	551.97
-3.78	0.04	0.01	0.01	0.03	-0.06	0.01	0.03	0.00	0.03	0.02	-0.02
-3.28	0.03	0.07	-0.01	-0.04	0.02	-0.01	0.05	0.07	0.00	-0.01	0.04
-2.78	-0.04	-0.03	0.00	0.06	-0.01	0.05	-0.07	0.04	0.06	0.05	-0.02
-2.28	0.01	0.01	0.06	0.00	0.04	-0.01	0.03	0.05	0.00	0.02	0.05
-1.78	0.00	0.00	-0.03	-0.01	0.02	0.00	0.04	-0.08	0.05	-0.04	0.02
-1.28	-0.03	-0.08	-0.05	-0.06	-0.02	-0.04	-0.02	0.00	-0.09	0.02	-0.03
-0.78	0.01	0.07	0.02	-0.01	0.02	-0.01	0.01	-0.07	-0.03	-0.02	-0.03
-0.28	-0.02	-0.06	0.01	0.04	-0.02	0.01	-0.05	-0.01	-0.02	-0.06	0.00
-0.18	0.04	0.03	-0.01	0.00	0.03	0.08	0.06	-0.01	0.01	0.03	0.08
-0.08	0.02	0.04	-0.07	-0.05	-0.01	-0.03	-0.01	0.02	-0.07	0.04	-0.11
0.02	0.03	-0.04	-0.02	0.03	0.04	0.07	0.04	-0.03	0.05	0.06	0.03
0.12	0.04	0.01	-0.06	-0.02	-0.04	0.02	0.04	0.02	0.05	0.08	0.02
0.22	-0.02	-0.04	0.00	-0.03	0.09	0.03	0.00	0.01	0.05	0.03	0.03
0.27	-0.02	-0.02	-0.03	-0.04	-0.07	0.00	-0.02	0.00	0.02	-0.05	-0.04
0.32	0.07	0.02	0.03	0.05	0.00	0.04	-0.02	0.04	0.00	-0.01	-0.03
0.37	0.02	-0.07	-0.04	-0.02	-0.07	0.03	0.04	0.02	0.07	-0.05	-0.10
0.42	-0.04	-0.16	-0.18	-0.06	-0.13	0.01	-0.07	0.00	-0.08	-0.11	-0.05
0.47	-0.07	0.01	0.04	-0.05	0.01	0.06	0.01	-0.06	-0.02	-0.02	-0.03
0.52	-0.04	-0.08	-0.07	-0.06	0.07	-0.02	0.08	-0.03	-0.04	-0.02	0.02
0.57	0.03	-0.07	0.03	0.03	0.00	0.09	0.06	0.03	-0.01	-0.01	0.00
0.62	-0.04	0.00	0.02	-0.04	0.08	-0.01	0.05	-0.06	-0.04	-0.03	-0.05
0.67	0.04	-0.04	-0.11	0.00	0.00	-0.03	0.08	-0.03	0.06	0.02	0.02
0.72	-0.15	-0.02	-0.13	-0.02	-0.10	-0.05	-0.02	0.03	-0.07	-0.01	-0.10
0.77	-0.02	-0.05	-0.08	0.02	0.03	-0.06	0.03	0.02	-0.03	-0.05	0.01
0.82	-0.04	0.03	0.02	-0.07	-0.09	-0.01	0.05	0.01	-0.04	-0.06	-0.01

0.87	0.04	0.01	-0.03	-0.08	-0.10	0.00	-0.03	-0.07	-0.04	-0.01	0.00
0.92	0.01	0.01	-0.11	-0.03	-0.02	-0.01	0.04	-0.03	-0.06	0.01	-0.04
0.97	-0.03	-0.01	-0.09	-0.02	-0.01	-0.03	0.01	-0.05	-0.04	-0.04	-0.01
1.02	-0.10	-0.05	-0.04	0.01	0.04	0.04	0.05	0.00	0.05	0.00	0.01
1.07	0.00	0.02	-0.08	-0.07	-0.02	-0.04	0.01	-0.02	-0.04	0.01	0.05
1.12	-0.04	-0.08	-0.14	0.03	0.04	-0.04	-0.04	0.01	0.08	0.01	-0.09
1.17	0.19	0.15	0.18	0.09	0.05	-0.02	-0.06	0.01	-0.03	-0.01	0.07
1.22	-0.74	-1.62	-1.97	-1.89	-1.48	-1.06	-0.58	-0.21	-0.02	0.19	0.24
1.27	-22.81	-25.69	-28.64	-31.29	-33.78	-36.10	-37.05	-36.99	-35.60	-32.64	-28.50
1.32	74.00	74.92	74.22	73.93	72.47	68.82	65.79	61.36	56.08	50.93	45.47
1.37	-38.49	-44.17	-49.78	-55.35	-60.07	-64.01	-66.22	-66.85	-65.76	-62.18	-56.66
1.42	-11.31	-10.50	-9.62	-8.81	-7.96	-7.12	-6.50	-6.09	-5.87	-5.81	-5.89
1.47	-14.48	-13.73	-12.88	-11.94	-11.06	-10.11	-9.08	-8.19	-7.29	-6.31	-5.38
1.52	-14.25	-13.93	-13.55	-12.78	-11.99	-11.11	-10.05	-8.94	-7.78	-6.46	-5.19
1.57	-12.41	-11.50	-10.68	-9.88	-9.03	-8.21	-7.49	-6.55	-5.79	-4.98	-4.06
1.62	-12.55	-11.82	-10.84	-10.03	-8.89	-7.88	-6.83	-5.93	-4.94	-4.18	-3.31
1.67	-10.92	-10.39	-9.94	-9.28	-8.58	-7.79	-6.90	-6.02	-4.96	-3.98	-2.92
1.72	-11.07	-10.25	-9.42	-8.54	-7.69	-6.85	-6.04	-5.26	-4.43	-3.65	-2.89
1.77	-10.79	-10.17	-9.40	-8.57	-7.54	-6.62	-5.40	-4.30	-3.26	-2.11	-1.13
1.82	-9.39	-8.63	-7.98	-7.32	-6.71	-5.85	-5.19	-4.32	-3.47	-2.66	-1.78
1.87	-9.58	-8.71	-7.70	-6.67	-5.76	-4.67	-3.78	-2.87	-2.00	-1.09	-0.26
1.92	-9.24	-8.71	-8.05	-7.29	-6.58	-5.64	-4.60	-3.58	-2.58	-1.50	-0.50
1.97	-8.71	-7.83	-7.07	-6.25	-5.32	-4.39	-3.62	-2.74	-1.80	-0.93	0.05
2.02	-8.87	-8.16	-7.20	-6.21	-5.31	-4.18	-3.16	-2.11	-1.09	-0.20	0.78
2.07	-8.20	-7.51	-6.85	-5.99	-5.25	-4.25	-3.18	-2.25	-1.12	-0.16	0.90
2.12	-8.11	-7.27	-6.36	-5.51	-4.60	-3.46	-2.58	-1.74	-0.81	0.10	0.91
2.17	-8.25	-7.48	-6.61	-5.72	-4.72	-3.59	-2.61	-1.58	-0.50	0.51	1.48
2.22	-7.30	-6.56	-5.76	-4.98	-4.10	-3.30	-2.33	-1.53	-0.55	0.47	1.55
2.27	-7.75	-6.90	-5.82	-4.93	-3.89	-2.76	-1.85	-0.89	0.05	1.08	1.97
2.32	-7.45	-6.74	-5.96	-5.15	-4.15	-3.17	-2.13	-1.16	-0.05	1.00	2.04
2.37	-7.09	-6.24	-5.29	-4.44	-3.65	-2.63	-1.69	-0.79	0.20	1.12	2.08
2.42	-7.27	-6.39	-5.50	-4.46	-3.42	-2.30	-1.29	-0.27	0.73	1.58	2.56
2.47	-6.99	-6.22	-5.45	-4.59	-3.71	-2.65	-1.59	-0.59	0.54	1.59	2.58
2.52	-6.86	-6.05	-5.01	-4.05	-3.15	-2.05	-1.15	-0.19	0.86	1.77	2.83
2.57	-7.02	-6.20	-5.20	-4.24	-3.24	-2.04	-1.01	0.04	1.19	2.14	3.17
2.62	-6.47	-5.77	-4.87	-4.02	-3.15	-2.05	-1.11	-0.14	0.99	1.95	3.03
2.67	-6.51	-5.59	-4.70	-3.70	-2.82	-1.72	-0.75	0.17	1.28	2.15	3.16
2.72	-6.54	-5.88	-4.96	-4.02	-3.03	-1.96	-0.84	0.18	1.27	2.35	3.48
2.77	-6.03	-5.33	-4.31	-3.56	-2.62	-1.57	-0.60	0.47	1.49	2.41	3.40
2.82	-6.34	-5.50	-4.49	-3.56	-2.51	-1.45	-0.44	0.62	1.71	2.67	3.61
2.87	-6.00	-5.23	-4.40	-3.53	-2.56	-1.56	-0.50	0.58	1.65	2.70	3.71
2.92	-5.98	-5.06	-4.15	-3.24	-2.30	-1.29	-0.26	0.82	1.89	2.89	3.86
2.97	-5.96	-5.16	-4.23	-3.19	-2.34	-1.08	-0.09	1.01	2.07	3.13	4.14
3.02	-5.86	-5.02	-4.15	-3.33	-2.42	-1.35	-0.22	0.76	1.86	2.94	4.03
3.07	-5.81	-4.98	-3.97	-3.12	-2.04	-0.90	0.04	1.09	2.16	3.13	4.13
3.12	-5.81	-5.01	-4.17	-3.17	-2.19	-1.14	-0.07	1.04	2.16	3.18	4.23
3.17	-5.54	-4.69	-3.86	-2.92	-1.94	-0.90	0.11	1.10	2.24	3.25	4.30
3.22	-5.82	-4.91	-3.92	-2.97	-1.92	-0.79	0.28	1.35	2.44	3.46	4.49
3.42	-5.33	-4.54	-3.56	-2.74	-1.71	-0.58	0.38	1.53	2.54	3.69	4.83
3.62	-5.35	-4.48	-3.52	-2.53	-1.56	-0.43	0.73	1.81	2.74	3.95	4.97

3.82	-5.22	-4.38	-3.49	-2.55	-1.46	-0.42	0.59	1.67	2.83	3.96	5.00
4.02	-5.11	-4.21	-3.26	-2.34	-1.22	-0.24	0.93	2.07	3.15	4.19	5.33
4.22	-4.93	-4.09	-3.13	-2.23	-1.20	-0.13	0.94	1.98	3.11	4.25	5.33
4.42	-4.85	-3.96	-2.98	-2.07	-0.98	0.02	1.14	2.13	3.32	4.46	5.50
4.62	-4.72	-3.90	-3.04	-2.02	-1.02	0.18	1.25	2.45	3.53	4.68	5.76
4.82	-4.70	-3.87	-2.88	-1.91	-0.93	0.21	1.35	2.38	3.56	4.61	5.71
5.02	-4.46	-3.62	-2.70	-1.75	-0.67	0.45	1.51	2.60	3.75	4.87	6.00
5.22	-4.41	-3.61	-2.63	-1.63	-0.61	0.46	1.51	2.69	3.77	4.96	6.03
5.42	-4.35	-3.46	-2.58	-1.50	-0.48	0.63	1.71	2.83	3.94	4.99	6.15
5.62	-4.17	-3.31	-2.27	-1.34	-0.31	0.75	1.88	3.04	4.18	5.17	6.35
5.82	-4.11	-3.22	-2.30	-1.33	-0.29	0.92	1.86	2.95	4.15	5.32	6.48
6.02	-4.00	-3.10	-2.20	-1.17	-0.16	0.97	2.13	3.18	4.30	5.45	6.60
6.22	-3.95	-3.10	-2.14	-1.14	-0.09	1.12	2.05	3.25	4.39	5.45	6.61
6.42	-3.82	-3.02	-1.90	-1.04	-0.01	1.14	2.28	3.37	4.46	5.55	6.73
6.62	-3.77	-2.84	-1.86	-0.85	0.19	1.31	2.42	3.50	4.67	5.80	7.02
6.82	-3.69	-2.79	-1.83	-0.79	0.29	1.34	2.47	3.55	4.65	5.79	6.88
7.02	-3.64	-2.68	-1.76	-0.66	0.23	1.42	2.50	3.65	4.79	5.93	7.05
7.22	-3.55	-2.65	-1.64	-0.67	0.29	1.47	2.61	3.64	4.89	5.99	7.11
7.42	-3.49	-2.56	-1.63	-0.58	0.35	1.56	2.65	3.81	4.98	6.11	7.23
7.62	-3.38	-2.55	-1.47	-0.54	0.37	1.62	2.63	3.88	4.99	6.14	7.17
7.82	-3.29	-2.47	-1.50	-0.44	0.51	1.71	2.74	3.88	5.10	6.18	7.34
8.02	-3.34	-2.45	-1.45	-0.48	0.54	1.63	2.74	3.84	5.01	6.21	7.25
8.22	-3.20	-2.44	-1.48	-0.50	0.62	1.69	2.81	3.90	5.01	6.14	7.21
8.42	-3.18	-2.35	-1.47	-0.50	0.63	1.74	2.75	3.91	5.02	6.13	7.25
8.62	-3.11	-2.15	-1.25	-0.25	0.72	1.76	2.92	4.03	5.16	6.30	7.48
8.82	-2.99	-2.23	-1.20	-0.30	0.89	1.99	3.00	4.18	5.41	6.46	7.60
9.02	-3.03	-2.22	-1.22	-0.27	0.76	1.92	2.97	4.01	5.16	6.26	7.37
9.22	-3.00	-2.17	-1.28	-0.24	0.80	1.94	3.00	4.14	5.31	6.41	7.50
9.42	-2.92	-2.08	-1.12	-0.07	0.96	2.06	3.17	4.27	5.50	6.51	7.69
9.62	-2.90	-2.03	-1.07	-0.11	1.03	2.07	3.13	4.28	5.39	6.47	7.64
9.82	-2.78	-1.90	-1.03	0.05	1.06	2.13	3.28	4.29	5.50	6.69	7.75
10.02	-2.82	-1.98	-0.99	-0.09	1.02	2.12	3.22	4.32	5.53	6.55	7.66
10.22	-2.76	-1.88	-0.92	0.08	1.06	2.21	3.32	4.42	5.62	6.71	7.78
10.42	-2.79	-1.83	-0.93	0.09	1.10	2.18	3.36	4.49	5.65	6.75	7.87
10.62	-2.71	-1.83	-0.88	0.13	1.16	2.25	3.33	4.49	5.65	6.74	7.91
10.82	-2.73	-1.87	-0.88	0.09	1.08	2.29	3.40	4.50	5.68	6.76	7.83
11.02	-2.58	-1.77	-0.82	0.12	1.26	2.37	3.44	4.60	5.66	6.79	7.89
11.22	-2.64	-1.75	-0.92	0.19	1.16	2.32	3.35	4.42	5.54	6.63	7.80
11.42	-2.58	-1.79	-0.82	0.14	1.15	2.27	3.34	4.45	5.55	6.75	7.67
11.62	-2.63	-1.75	-0.85	0.16	1.19	2.30	3.36	4.49	5.65	6.70	7.91
11.82	-2.47	-1.66	-0.72	0.19	1.23	2.47	3.49	4.61	5.73	6.86	7.97
12.02	-2.48	-1.66	-0.60	0.32	1.33	2.38	3.52	4.59	5.73	6.81	7.95
12.22	-2.48	-1.64	-0.72	0.27	1.30	2.49	3.51	4.71	5.85	6.88	8.01
12.42	-2.48	-1.55	-0.71	0.32	1.35	2.37	3.51	4.58	5.70	6.81	7.92
12.62	-2.41	-1.54	-0.58	0.34	1.47	2.54	3.64	4.69	5.75	7.02	8.07
12.82	-2.31	-1.48	-0.48	0.46	1.44	2.53	3.63	4.67	5.74	6.98	8.05
13.02	-2.36	-1.45	-0.49	0.44	1.49	2.63	3.71	4.86	5.93	7.06	8.18
13.22	-2.35	-1.49	-0.50	0.35	1.53	2.58	3.69	4.79	5.89	7.02	8.07
13.42	-2.33	-1.46	-0.57	0.49	1.46	2.59	3.68	4.74	5.86	6.97	8.08
13.50	-2.28	-1.41	-0.44	0.54	1.59	2.59	3.70	4.80	5.99	6.98	8.20

13.59	-2.28	-1.40	-0.45	0.52	1.58	2.72	3.74	4.85	6.05	7.11	8.28
13.68	-2.26	-1.33	-0.46	0.49	1.47	2.65	3.72	4.81	5.89	7.02	8.15
13.78	-2.35	-1.46	-0.51	0.46	1.48	2.61	3.70	4.76	5.87	6.93	8.11
13.88	-2.30	-1.46	-0.48	0.49	1.42	2.52	3.63	4.79	5.89	6.97	8.16
14.00	-2.22	-1.45	-0.50	0.50	1.62	2.66	3.82	4.93	6.01	7.12	8.26
14.12	-2.27	-1.38	-0.50	0.43	1.61	2.58	3.74	4.78	5.94	7.08	8.15
14.25	-2.23	-1.35	-0.52	0.45	1.49	2.66	3.69	4.82	5.88	6.99	8.19
14.40	-2.27	-1.35	-0.38	0.62	1.62	2.73	3.82	5.01	6.10	7.31	8.38
14.55	-2.31	-1.30	-0.48	0.59	1.61	2.68	3.69	4.89	5.97	7.10	8.15
14.72	-2.17	-1.22	-0.36	0.60	1.63	2.73	3.80	4.95	6.12	7.29	8.21
14.90	-2.13	-1.26	-0.33	0.65	1.58	2.75	3.82	4.84	6.01	7.08	8.17
15.09	-2.13	-1.19	-0.34	0.68	1.70	2.79	3.83	4.90	6.03	7.17	8.27
15.30	-2.09	-1.32	-0.28	0.55	1.68	2.69	3.83	4.86	6.08	7.16	8.25
15.53	-2.02	-1.16	-0.18	0.73	1.75	2.95	3.96	5.10	6.29	7.40	8.51
15.77	-2.02	-1.19	-0.26	0.69	1.73	2.81	3.90	4.97	6.13	7.28	8.32
16.04	-2.08	-1.23	-0.31	0.72	1.72	2.92	3.92	5.04	6.18	7.33	8.52
16.32	-2.02	-1.08	-0.23	0.80	1.89	2.89	4.01	5.12	6.19	7.36	8.41
16.63	-1.98	-1.14	-0.19	0.85	1.88	3.05	4.16	5.19	6.30	7.44	8.52
16.96	-1.97	-1.11	-0.09	0.88	1.91	2.96	4.12	5.18	6.32	7.48	8.65
17.31	-2.05	-1.06	-0.14	0.77	1.80	3.00	4.03	5.22	6.27	7.38	8.53
17.70	-1.80	-1.07	-0.10	0.93	2.05	3.10	4.27	5.32	6.50	7.66	8.73
18.11	-1.93	-1.08	-0.10	0.79	1.83	2.86	4.02	5.02	6.23	7.33	8.41
18.56	-1.87	-0.99	-0.09	0.94	1.89	3.08	4.13	5.16	6.29	7.39	8.56
19.04	-1.89	-1.03	-0.06	0.95	1.92	3.07	4.16	5.19	6.38	7.47	8.69
19.56	-1.83	-0.89	0.05	1.06	2.01	3.21	4.33	5.37	6.51	7.70	8.75
20.12	-1.77	-0.89	0.01	1.05	2.09	3.15	4.37	5.46	6.59	7.64	8.77
20.72	-1.67	-0.98	0.08	1.11	2.15	3.24	4.32	5.45	6.59	7.64	8.78
21.37	-1.63	-0.86	0.11	1.13	2.11	3.24	4.42	5.60	6.65	7.80	8.90
22.08	-1.75	-0.92	0.08	1.11	2.12	3.24	4.31	5.39	6.63	7.73	8.80
22.84	-1.78	-0.82	-0.07	1.05	2.18	3.24	4.38	5.48	6.62	7.82	8.85
23.65	-1.72	-0.82	0.07	1.05	2.13	3.26	4.26	5.38	6.58	7.59	8.74
24.54	-1.54	-0.70	0.21	1.33	2.33	3.44	4.55	5.72	6.82	8.00	9.17
25.49	-1.58	-0.67	0.33	1.32	2.45	3.47	4.66	5.76	6.98	8.02	9.15
26.51	-1.61	-0.64	0.22	1.21	2.28	3.41	4.36	5.60	6.64	7.76	8.92
27.62	-1.41	-0.48	0.42	1.48	2.49	3.55	4.81	5.91	7.06	8.21	9.32
28.81	-1.39	-0.51	0.43	1.40	2.42	3.58	4.69	5.80	7.01	8.13	9.24
30.10	-1.39	-0.48	0.49	1.41	2.47	3.59	4.69	5.80	6.88	8.03	9.09
31.49	-1.24	-0.32	0.65	1.62	2.61	3.81	4.95	5.96	7.25	8.33	9.53
32.98	-1.27	-0.32	0.64	1.59	2.69	3.75	4.88	6.05	7.13	8.21	9.35
34.60	-1.12	-0.27	0.71	1.74	2.78	3.92	5.02	6.14	7.38	8.54	9.63
36.34	-1.19	-0.29	0.66	1.61	2.65	3.88	5.04	6.13	7.36	8.43	9.57
38.21	-0.99	-0.14	0.81	1.96	2.89	4.15	5.32	6.36	7.52	8.77	9.82
40.24	-1.09	-0.13	0.84	1.82	2.91	4.10	5.19	6.34	7.44	8.63	9.74
42.42	-0.99	-0.15	0.84	1.84	2.88	3.99	5.04	6.21	7.41	8.51	9.63
44.78	-0.97	-0.15	0.83	1.86	2.91	4.06	5.16	6.32	7.47	8.63	9.70
47.32	-0.94	-0.03	0.91	1.89	2.89	4.13	5.21	6.27	7.51	8.49	9.71
50.06	-0.88	0.00	0.99	2.00	3.12	4.23	5.28	6.44	7.62	8.80	9.95
53.01	-0.84	-0.04	0.95	1.99	3.02	4.20	5.31	6.42	7.70	8.74	9.92
56.20	-0.70	0.21	1.13	2.14	3.18	4.37	5.55	6.68	7.79	8.89	10.17
59.64	-0.61	0.28	1.26	2.25	3.47	4.59	5.71	6.89	8.09	9.29	10.50

63.34	-0.55	0.20	1.18	2.20	3.29	4.41	5.53	6.66	7.84	8.92	10.05
67.34	-0.59	0.29	1.33	2.32	3.32	4.63	5.71	6.85	7.98	9.14	10.29
71.65	-0.38	0.44	1.51	2.49	3.55	4.79	5.91	7.07	8.27	9.49	10.62
76.30	-0.47	0.46	1.47	2.46	3.57	4.73	5.92	7.08	8.29	9.42	10.59
81.32	-0.41	0.67	1.62	2.73	3.76	4.96	6.15	7.23	8.57	9.71	10.98
86.73	-0.24	0.67	1.74	2.80	3.92	5.08	6.32	7.58	8.70	9.91	11.14
92.56	-0.08	0.88	1.88	2.88	4.05	5.29	6.40	7.59	8.88	9.97	11.25
98.85	-0.04	0.80	1.85	2.89	3.97	5.22	6.43	7.52	8.77	9.90	11.11
105.64	0.10	1.06	2.01	3.13	4.25	5.43	6.55	7.76	9.07	10.27	11.44
112.96	0.20	1.20	2.19	3.32	4.44	5.69	6.86	8.11	9.29	10.43	11.74
120.85	0.38	1.32	2.42	3.45	4.56	5.80	6.99	8.13	9.37	10.67	11.81
129.36	0.37	1.40	2.44	3.52	4.71	5.87	7.17	8.29	9.65	10.77	12.02
138.55	0.57	1.55	2.60	3.62	4.92	5.97	7.22	8.55	9.69	10.98	12.19
148.45	0.65	1.55	2.57	3.69	4.92	6.07	7.25	8.49	9.76	10.98	12.21
159.13	0.73	1.64	2.77	3.86	5.06	6.20	7.50	8.70	9.97	11.12	12.35
170.65	0.92	1.91	2.97	4.06	5.22	6.49	7.78	8.90	10.14	11.40	12.60
183.07	1.13	2.17	3.15	4.32	5.48	6.74	7.93	9.24	10.48	11.78	12.95
196.47	1.17	2.21	3.37	4.39	5.60	6.89	8.14	9.37	10.63	11.88	13.11
210.91	1.40	2.47	3.50	4.66	5.83	7.07	8.36	9.61	10.85	12.11	13.41
226.50	1.59	2.59	3.78	4.84	6.04	7.27	8.51	9.82	11.12	12.40	13.63
243.31	1.72	2.72	3.79	4.98	6.16	7.37	8.72	9.91	11.24	12.48	13.71
261.43	1.96	3.01	4.09	5.27	6.51	7.81	9.09	10.28	11.66	12.86	14.23
280.98	2.15	3.22	4.24	5.40	6.68	7.86	9.19	10.49	11.72	13.03	14.34
302.07	2.29	3.29	4.42	5.60	6.80	8.05	9.46	10.63	11.92	13.20	14.51
324.81	2.42	3.52	4.71	5.80	7.11	8.34	9.72	10.90	12.32	13.60	14.87
349.33	2.68	3.70	4.78	6.01	7.31	8.55	9.87	11.17	12.47	13.78	15.09
375.79	2.80	3.91	5.05	6.21	7.43	8.79	10.08	11.41	12.69	14.05	15.33
404.31	2.98	4.09	5.22	6.36	7.63	8.87	10.19	11.52	12.76	14.11	15.41
435.08	3.16	4.25	5.32	6.57	7.91	9.09	10.43	11.79	13.09	14.42	15.75
468.27	3.26	4.38	5.60	6.73	8.00	9.33	10.58	11.98	13.25	14.63	15.91
504.06	3.54	4.63	5.82	6.99	8.25	9.63	10.99	12.32	13.65	14.94	16.29
542.66	3.65	4.76	5.90	7.16	8.42	9.73	11.07	12.43	13.69	15.07	16.33
584.29	3.83	4.94	6.11	7.35	8.58	9.94	11.35	12.63	13.92	15.30	16.57
629.18	3.98	5.21	6.30	7.55	8.83	10.20	11.49	12.79	14.11	15.44	16.78
677.61	4.14	5.26	6.43	7.72	9.05	10.37	11.77	13.08	14.41	15.83	17.18
729.83	4.36	5.48	6.62	7.88	9.14	10.50	11.84	13.15	14.56	15.89	17.20
786.16	4.39	5.54	6.68	8.01	9.23	10.65	12.04	13.39	14.70	16.04	17.46
846.90	4.58	5.59	6.78	8.13	9.37	10.74	12.09	13.42	14.77	16.11	17.45
912.42	4.68	5.73	6.88	8.15	9.50	10.87	12.30	13.63	15.01	16.34	17.64
983.08	4.73	5.76	7.12	8.27	9.55	11.02	12.37	13.72	15.06	16.46	17.77
1059.29	4.80	5.92	7.10	8.42	9.65	11.09	12.50	13.86	15.24	16.65	17.99
1141.48	4.83	6.04	7.15	8.46	9.81	11.14	12.55	13.83	15.24	16.61	17.84
1230.13	4.84	5.96	7.18	8.42	9.74	11.04	12.45	13.80	15.23	16.53	17.91
1325.73	4.83	6.05	7.23	8.45	9.86	11.22	12.53	13.94	15.32	16.68	18.04
1428.84	4.79	6.01	7.21	8.50	9.87	11.21	12.59	13.92	15.29	16.72	17.98
1540.05	4.89	6.07	7.28	8.53	9.87	11.29	12.61	14.01	15.41	16.80	18.12
1659.99	4.94	6.09	7.26	8.67	10.01	11.37	12.64	14.03	15.42	16.73	18.18
1789.34	4.96	6.11	7.25	8.60	9.94	11.28	12.68	14.09	15.42	16.75	18.09
1928.85	4.97	6.06	7.34	8.65	9.95	11.33	12.77	14.01	15.52	16.79	18.14
2079.31	4.97	6.08	7.33	8.59	9.84	11.29	12.70	14.01	15.40	16.87	18.15

2241.59	4.87	6.10	7.26	8.51	9.88	11.22	12.69	13.94	15.39	16.66	18.11
2416.60	4.77	5.99	7.15	8.52	9.78	11.20	12.56	13.91	15.34	16.78	18.08
2605.36	4.77	5.95	7.21	8.49	9.95	11.16	12.57	13.92	15.37	16.71	18.04
2808.94	4.83	5.99	7.21	8.57	9.76	11.15	12.57	13.92	15.29	16.69	17.97
3028.50	4.82	5.96	7.27	8.46	9.77	11.18	12.49	13.95	15.23	16.68	17.89
3265.30	4.72	5.89	7.19	8.37	9.67	11.13	12.51	13.93	15.17	16.59	17.94
3520.69	4.69	5.88	7.06	8.36	9.71	11.05	12.31	13.79	15.08	16.39	17.84
3796.13	4.81	6.01	7.04	8.35	9.70	11.04	12.44	13.75	15.10	16.51	17.79

Wavelength (nm) / Time (ps)	553.39	554.82	556.24	557.67	559.09	560.51	561.94	563.36	564.79	566.21	567.63
-3.78	0.07	-0.04	-0.02	-0.03	-0.05	0.01	-0.09	-0.03	-0.05	-0.05	-0.01
-3.28	0.05	0.09	-0.03	0.06	-0.03	0.03	0.03	-0.01	-0.04	-0.04	-0.06
-2.78	-0.03	-0.04	0.04	0.04	0.03	-0.07	-0.01	0.04	0.06	0.02	0.03
-2.28	-0.01	0.02	0.04	0.02	0.03	-0.05	-0.01	-0.01	-0.01	-0.04	0.00
-1.78	0.08	-0.03	-0.01	-0.04	-0.02	0.02	0.00	-0.01	0.00	0.05	-0.02
-1.28	-0.05	-0.05	0.01	-0.01	0.00	0.02	0.06	0.05	0.04	0.03	0.00
-0.78	-0.05	0.05	-0.02	0.01	0.00	0.03	0.01	0.00	-0.03	0.07	0.02
-0.28	-0.05	-0.01	0.00	-0.05	0.04	0.01	0.01	-0.02	0.02	-0.04	0.03
-0.18	0.05	0.04	0.02	0.02	0.05	0.07	0.04	0.02	-0.05	-0.01	0.02
-0.08	-0.04	0.02	-0.03	0.00	-0.01	-0.06	-0.01	-0.01	0.00	-0.01	-0.01
0.02	0.03	0.01	0.04	0.11	0.11	0.04	0.09	0.03	-0.01	0.02	0.09
0.12	0.04	0.03	0.05	0.03	0.02	0.05	0.03	0.01	0.09	0.04	0.01
0.22	0.06	-0.02	0.06	0.04	0.05	0.08	-0.01	-0.03	0.06	0.05	0.02
0.27	0.01	-0.06	0.08	0.01	0.03	0.06	0.01	0.06	0.00	-0.01	0.05
0.32	0.04	0.05	-0.03	0.02	0.04	0.09	0.00	-0.02	0.04	-0.01	0.11
0.37	0.01	-0.05	-0.08	-0.01	0.03	0.01	0.02	-0.07	-0.09	-0.02	-0.03
0.42	-0.10	-0.08	-0.05	-0.04	-0.05	-0.04	0.03	-0.04	-0.05	-0.06	-0.11
0.47	0.05	0.05	0.05	-0.01	0.04	0.01	0.03	-0.08	0.00	-0.03	0.01
0.52	0.00	-0.04	0.03	0.02	0.02	0.03	0.03	-0.02	-0.05	0.03	-0.03
0.57	-0.02	-0.07	0.07	0.05	0.07	0.01	0.10	0.03	0.07	0.02	0.06
0.62	0.04	-0.07	0.02	0.04	0.05	0.01	-0.03	-0.03	0.00	-0.02	-0.05
0.67	-0.05	-0.05	0.00	-0.02	-0.01	-0.01	0.00	0.00	0.01	0.03	0.01
0.72	-0.04	-0.07	-0.01	-0.07	-0.04	0.03	-0.03	-0.08	-0.06	-0.07	0.00
0.77	-0.03	-0.02	-0.01	-0.01	0.00	0.05	0.09	-0.09	-0.03	-0.01	0.01
0.82	-0.03	-0.05	-0.08	-0.05	0.09	0.04	0.05	0.04	0.00	0.01	0.03
0.87	0.05	-0.02	-0.01	-0.03	-0.08	-0.02	0.07	0.04	-0.02	-0.04	-0.03
0.92	0.10	0.01	0.02	-0.03	0.01	-0.02	0.01	-0.03	0.06	0.06	0.00
0.97	0.06	0.01	0.05	0.01	0.00	0.00	-0.03	-0.03	-0.02	-0.07	0.03
1.02	-0.01	-0.01	-0.01	0.05	0.01	0.08	0.01	0.02	0.07	0.05	0.09
1.07	0.06	0.00	-0.02	-0.01	0.01	0.05	0.04	0.01	0.03	-0.01	0.00
1.12	-0.03	0.04	0.04	-0.02	-0.07	0.05	-0.03	-0.10	-0.04	0.05	0.02
1.17	0.08	-0.01	-0.06	0.02	0.09	0.08	-0.03	-0.09	0.07	0.08	-0.04
1.22	0.19	0.07	0.07	-0.02	-0.10	-0.16	-0.22	-0.32	-0.22	-0.07	0.02
1.27	-23.52	-18.05	-12.63	-7.90	-3.99	-1.26	0.50	1.06	0.99	0.46	-0.14
1.32	40.12	35.16	30.24	25.49	21.25	17.42	13.02	8.72	4.34	0.26	-4.03
1.37	-49.09	-39.25	-27.69	-14.51	-0.30	15.18	30.21	44.95	58.84	71.84	82.55
1.42	-5.79	-5.48	-4.88	-4.08	-3.13	-1.97	-0.97	0.03	0.48	0.58	0.03

1.47	-4.38	-3.35	-2.27	-1.27	-0.16	0.88	2.00	3.09	4.23	5.37	6.35
1.52	-3.98	-2.76	-1.53	-0.39	0.65	1.72	2.57	3.45	4.13	4.86	5.53
1.57	-3.13	-2.29	-1.34	-0.53	0.42	1.26	1.97	2.63	3.45	4.08	4.62
1.62	-2.52	-1.71	-0.93	-0.24	0.49	1.18	1.85	2.47	3.19	3.68	4.41
1.67	-1.92	-0.98	0.06	1.06	1.83	2.64	3.35	4.01	4.61	5.16	5.69
1.72	-2.03	-1.18	-0.38	0.46	1.35	2.20	2.84	3.65	4.36	4.98	5.57
1.77	-0.08	0.84	1.74	2.56	3.38	3.95	4.57	5.08	5.59	5.99	6.48
1.82	-0.83	0.06	1.01	1.85	2.69	3.53	4.24	4.84	5.51	6.05	6.54
1.87	0.63	1.34	2.31	3.00	3.81	4.51	5.15	5.70	6.47	6.95	7.56
1.92	0.59	1.57	2.65	3.48	4.34	5.07	5.72	6.31	6.83	7.36	7.77
1.97	1.04	1.86	2.86	3.69	4.58	5.32	6.03	6.68	7.23	7.79	8.30
2.02	1.73	2.56	3.40	4.17	4.84	5.52	6.12	6.59	7.07	7.52	8.10
2.07	1.98	2.92	3.81	4.75	5.58	6.31	6.99	7.47	8.07	8.50	8.89
2.12	1.79	2.62	3.55	4.33	5.19	5.91	6.51	7.21	7.65	8.20	8.74
2.17	2.40	3.31	4.08	4.93	5.66	6.33	6.87	7.39	7.81	8.24	8.68
2.22	2.44	3.41	4.36	5.30	6.23	6.88	7.53	8.19	8.77	9.20	9.74
2.27	2.84	3.83	4.70	5.49	6.26	7.06	7.74	8.30	8.94	9.39	9.95
2.32	3.03	4.06	4.91	5.80	6.60	7.23	7.95	8.39	9.00	9.26	9.78
2.37	3.10	4.03	4.98	5.80	6.67	7.39	8.02	8.69	9.25	9.78	10.26
2.42	3.55	4.49	5.37	6.15	6.94	7.62	8.30	8.83	9.36	9.82	10.40
2.47	3.64	4.60	5.61	6.47	7.26	8.02	8.73	9.31	9.88	10.29	10.63
2.52	3.79	4.68	5.65	6.56	7.35	8.11	8.77	9.51	9.98	10.52	10.90
2.57	4.17	5.06	6.01	6.85	7.61	8.37	9.09	9.56	10.12	10.52	11.00
2.62	4.12	5.02	6.11	7.10	7.98	8.69	9.36	9.98	10.54	11.06	11.54
2.67	4.11	5.08	5.99	6.80	7.60	8.41	8.93	9.60	10.16	10.72	11.06
2.72	4.58	5.48	6.48	7.36	8.11	8.92	9.54	10.10	10.69	11.13	11.51
2.77	4.48	5.39	6.36	7.25	8.13	8.92	9.65	10.18	10.79	11.20	11.62
2.82	4.67	5.58	6.46	7.41	8.16	8.93	9.62	10.16	10.62	11.20	11.66
2.87	4.76	5.78	6.72	7.58	8.36	9.11	9.77	10.33	10.90	11.31	11.78
2.92	4.86	5.83	6.87	7.66	8.59	9.36	10.13	10.78	11.25	11.76	12.30
2.97	5.19	6.13	7.09	7.90	8.71	9.47	10.10	10.68	11.16	11.64	12.12
3.02	5.13	6.06	7.13	7.95	8.92	9.68	10.42	10.99	11.60	11.99	12.55
3.07	5.20	5.99	7.02	7.91	8.72	9.48	10.12	10.67	11.25	11.76	12.30
3.12	5.30	6.30	7.27	8.12	8.89	9.68	10.30	10.92	11.39	11.90	12.37
3.17	5.40	6.25	7.28	8.14	9.04	9.78	10.49	11.13	11.66	12.13	12.58
3.22	5.61	6.52	7.55	8.46	9.33	10.08	10.77	11.36	11.96	12.47	12.85
3.42	5.81	6.92	7.89	8.88	9.69	10.50	11.16	11.89	12.39	12.91	13.36
3.62	6.03	6.97	8.06	8.97	9.95	10.70	11.44	12.10	12.61	13.22	13.65
3.82	6.16	7.13	8.22	9.18	10.02	10.79	11.54	12.20	12.72	13.23	13.72
4.02	6.45	7.39	8.47	9.40	10.28	11.09	11.88	12.46	13.06	13.68	14.10
4.22	6.41	7.44	8.53	9.44	10.28	11.06	11.89	12.45	13.04	13.47	13.99
4.42	6.60	7.62	8.66	9.58	10.51	11.31	12.01	12.68	13.27	13.78	14.32
4.62	6.92	7.87	8.89	9.83	10.81	11.56	12.29	12.97	13.61	14.09	14.67
4.82	6.81	7.85	8.80	9.82	10.73	11.52	12.21	12.86	13.51	14.02	14.51
5.02	7.14	8.12	9.14	10.10	11.07	11.86	12.66	13.23	13.90	14.37	14.92
5.22	7.12	8.19	9.23	10.20	11.12	11.87	12.67	13.38	14.01	14.47	15.02
5.42	7.30	8.36	9.48	10.35	11.30	12.12	12.76	13.45	14.00	14.65	15.16
5.62	7.52	8.52	9.50	10.47	11.45	12.26	13.02	13.67	14.23	14.89	15.36
5.82	7.49	8.60	9.59	10.55	11.47	12.23	13.05	13.73	14.33	14.83	15.32
6.02	7.72	8.74	9.72	10.79	11.73	12.50	13.30	13.92	14.57	15.12	15.59

6.22	7.72	8.68	9.69	10.70	11.64	12.56	13.20	13.78	14.49	14.95	15.53
6.42	7.78	8.81	9.87	10.86	11.75	12.60	13.35	14.02	14.67	15.21	15.73
6.62	8.08	9.22	10.22	11.29	12.13	12.94	13.69	14.42	14.95	15.62	16.06
6.82	8.06	9.05	10.08	11.05	12.09	12.85	13.60	14.21	14.84	15.42	15.86
7.02	8.22	9.21	10.36	11.28	12.21	13.06	13.91	14.48	15.15	15.76	16.23
7.22	8.22	9.30	10.34	11.32	12.21	13.01	13.81	14.44	15.14	15.59	16.19
7.42	8.33	9.44	10.49	11.47	12.39	13.24	14.07	14.74	15.40	15.89	16.42
7.62	8.32	9.39	10.41	11.45	12.37	13.23	13.96	14.62	15.29	15.75	16.33
7.82	8.45	9.43	10.53	11.48	12.40	13.27	14.02	14.60	15.26	15.81	16.37
8.02	8.47	9.45	10.45	11.44	12.35	13.17	13.85	14.61	15.23	15.78	16.23
8.22	8.30	9.46	10.45	11.51	12.36	13.22	13.94	14.61	15.16	15.87	16.31
8.42	8.38	9.38	10.47	11.36	12.33	13.18	13.84	14.54	15.17	15.67	16.16
8.62	8.59	9.54	10.54	11.64	12.56	13.36	14.11	14.79	15.43	15.98	16.47
8.82	8.72	9.83	10.83	11.75	12.64	13.60	14.32	14.98	15.63	16.17	16.69
9.02	8.55	9.56	10.67	11.57	12.46	13.29	14.08	14.67	15.32	15.87	16.45
9.22	8.67	9.67	10.72	11.75	12.59	13.40	14.24	14.93	15.50	16.12	16.60
9.42	8.89	9.82	10.95	11.88	12.85	13.64	14.44	15.04	15.66	16.24	16.70
9.62	8.78	9.79	10.79	11.86	12.81	13.63	14.39	15.01	15.57	16.21	16.64
9.82	8.90	9.92	11.00	11.94	12.90	13.78	14.50	15.10	15.74	16.37	16.82
10.02	8.87	9.83	10.96	11.83	12.75	13.58	14.40	15.00	15.61	16.19	16.66
10.22	8.95	10.03	11.06	12.05	12.98	13.79	14.50	15.27	15.78	16.44	16.94
10.42	8.97	9.98	11.08	12.08	13.04	13.78	14.56	15.28	15.84	16.43	17.00
10.62	9.11	10.05	11.09	12.04	13.00	13.85	14.55	15.25	15.91	16.56	16.95
10.82	8.98	10.04	11.04	12.00	12.94	13.76	14.52	15.20	15.80	16.33	16.78
11.02	9.01	10.04	11.08	12.10	12.97	13.83	14.57	15.23	15.90	16.38	16.92
11.22	8.85	9.93	11.07	11.91	12.82	13.59	14.38	15.01	15.68	16.23	16.64
11.42	8.81	9.90	10.89	11.86	12.69	13.57	14.33	14.98	15.61	16.09	16.57
11.62	9.01	10.05	11.01	12.05	12.98	13.82	14.58	15.20	15.84	16.36	16.87
11.82	9.04	10.14	11.11	12.11	13.00	13.88	14.64	15.25	15.82	16.42	16.97
12.02	9.09	10.01	11.16	12.22	13.04	13.86	14.66	15.29	15.87	16.45	16.95
12.22	9.21	10.23	11.25	12.31	13.19	14.04	14.85	15.45	16.13	16.64	17.21
12.42	9.05	10.06	11.04	12.00	12.95	13.73	14.43	15.20	15.76	16.29	16.79
12.62	9.16	10.18	11.27	12.21	13.04	13.92	14.65	15.35	15.93	16.58	17.02
12.82	9.15	10.13	11.22	12.14	13.09	13.95	14.62	15.27	15.91	16.46	16.87
13.02	9.42	10.38	11.50	12.40	13.30	14.10	14.98	15.55	16.24	16.78	17.27
13.22	9.24	10.23	11.32	12.34	13.13	14.01	14.77	15.50	16.07	16.64	17.10
13.42	9.17	10.20	11.37	12.24	13.12	13.93	14.71	15.38	16.05	16.60	17.02
13.50	9.30	10.29	11.36	12.28	13.09	13.99	14.71	15.33	15.96	16.54	16.98
13.59	9.38	10.42	11.46	12.38	13.31	14.15	14.93	15.65	16.24	16.76	17.33
13.68	9.22	10.23	11.26	12.31	13.23	13.93	14.79	15.48	15.97	16.52	17.11
13.78	9.15	10.20	11.20	12.15	12.98	13.90	14.54	15.22	15.81	16.41	16.86
13.88	9.24	10.27	11.30	12.39	13.13	13.96	14.82	15.35	15.93	16.54	17.09
14.00	9.41	10.42	11.58	12.45	13.37	14.22	14.99	15.64	16.30	16.80	17.31
14.12	9.30	10.33	11.43	12.41	13.29	14.10	14.81	15.57	16.19	16.70	17.20
14.25	9.21	10.23	11.32	12.26	13.23	13.93	14.77	15.39	16.04	16.56	17.04
14.40	9.51	10.55	11.61	12.56	13.47	14.38	15.15	15.80	16.43	16.94	17.47
14.55	9.29	10.34	11.35	12.37	13.22	14.04	14.86	15.49	16.06	16.63	17.08
14.72	9.45	10.45	11.51	12.44	13.37	14.16	14.96	15.58	16.16	16.78	17.19
14.90	9.26	10.30	11.38	12.27	13.25	14.04	14.88	15.40	16.03	16.67	17.11
15.09	9.39	10.38	11.35	12.37	13.16	14.10	14.79	15.43	16.01	16.60	16.98

15.30	9.37	10.39	11.46	12.40	13.35	14.16	14.98	15.60	16.22	16.71	17.19
15.53	9.63	10.63	11.70	12.72	13.66	14.45	15.27	15.93	16.48	17.02	17.59
15.77	9.40	10.47	11.50	12.50	13.45	14.21	14.96	15.66	16.29	16.86	17.35
16.04	9.58	10.65	11.59	12.65	13.55	14.39	15.12	15.81	16.48	17.01	17.47
16.32	9.49	10.55	11.59	12.62	13.46	14.30	15.07	15.72	16.38	16.96	17.39
16.63	9.69	10.74	11.79	12.73	13.74	14.56	15.36	15.93	16.61	17.14	17.55
16.96	9.78	10.78	11.79	12.74	13.74	14.52	15.31	15.99	16.62	17.15	17.72
17.31	9.62	10.69	11.69	12.74	13.60	14.41	15.15	15.85	16.47	17.06	17.49
17.70	9.89	10.94	12.00	13.02	13.86	14.75	15.62	16.23	16.89	17.43	17.94
18.11	9.51	10.43	11.52	12.52	13.39	14.19	14.98	15.61	16.24	16.76	17.22
18.56	9.68	10.71	11.76	12.58	13.58	14.44	15.18	15.87	16.57	17.03	17.52
19.04	9.73	10.71	11.77	12.82	13.64	14.51	15.23	15.86	16.52	17.04	17.52
19.56	9.88	10.92	12.00	12.96	13.89	14.78	15.55	16.16	16.89	17.43	17.93
20.12	9.92	10.97	12.02	13.08	13.89	14.70	15.52	16.22	16.87	17.33	17.91
20.72	9.96	10.97	12.10	13.03	14.06	14.81	15.64	16.24	16.89	17.46	17.99
21.37	10.01	11.07	12.12	13.07	14.00	14.83	15.57	16.30	16.90	17.50	17.95
22.08	9.98	10.94	12.05	13.04	13.96	14.78	15.54	16.32	16.85	17.44	17.98
22.84	10.02	11.12	12.14	13.14	14.09	14.92	15.68	16.34	17.05	17.55	18.11
23.65	9.84	10.97	11.89	12.88	13.82	14.67	15.36	16.11	16.65	17.23	17.74
24.54	10.20	11.33	12.39	13.35	14.19	15.08	15.85	16.45	17.17	17.67	18.19
25.49	10.39	11.36	12.39	13.40	14.39	15.26	16.03	16.64	17.42	17.95	18.39
26.51	10.06	11.00	12.09	13.07	13.88	14.79	15.46	16.27	16.84	17.34	17.94
27.62	10.42	11.49	12.48	13.60	14.53	15.39	16.08	16.77	17.43	17.98	18.55
28.81	10.48	11.44	12.57	13.53	14.46	15.36	16.10	16.76	17.39	17.90	18.49
30.10	10.27	11.32	12.36	13.29	14.21	15.04	15.80	16.50	17.12	17.60	18.17
31.49	10.66	11.70	12.73	13.78	14.70	15.57	16.35	17.03	17.56	18.28	18.72
32.98	10.50	11.51	12.61	13.64	14.49	15.28	16.17	16.87	17.53	18.05	18.60
34.60	10.81	11.86	12.81	13.83	14.79	15.74	16.49	17.18	17.91	18.39	18.98
36.34	10.73	11.74	12.80	13.76	14.81	15.51	16.44	17.11	17.71	18.26	18.83
38.21	10.99	12.15	13.20	14.31	15.15	16.01	16.80	17.54	18.19	18.76	19.31
40.24	10.97	11.92	13.03	14.10	15.03	15.86	16.63	17.31	18.05	18.61	19.11
42.42	10.77	11.88	12.88	13.83	14.76	15.65	16.42	17.12	17.75	18.38	18.80
44.78	10.86	11.90	13.00	13.91	14.90	15.76	16.58	17.26	17.86	18.47	19.07
47.32	10.90	11.88	12.94	13.95	14.87	15.75	16.54	17.19	17.89	18.51	18.91
50.06	11.08	12.15	13.19	14.27	15.10	15.99	16.81	17.58	18.20	18.72	19.33
53.01	10.99	12.13	13.19	14.21	15.28	16.03	16.83	17.52	18.23	18.82	19.33
56.20	11.32	12.38	13.57	14.52	15.38	16.30	17.09	17.79	18.40	19.08	19.67
59.64	11.64	12.67	13.77	14.77	15.81	16.67	17.42	18.17	18.89	19.50	19.96
63.34	11.25	12.23	13.32	14.30	15.22	16.12	16.88	17.64	18.26	18.86	19.40
67.34	11.54	12.59	13.69	14.64	15.57	16.50	17.32	18.01	18.72	19.31	19.87
71.65	11.79	12.82	13.93	14.96	15.89	16.85	17.69	18.40	19.09	19.65	20.19
76.30	11.78	12.85	14.03	14.98	16.04	16.82	17.64	18.43	19.03	19.67	20.27
81.32	12.16	13.31	14.34	15.40	16.40	17.25	18.13	18.88	19.53	20.15	20.70
86.73	12.30	13.40	14.43	15.54	16.50	17.43	18.31	19.00	19.78	20.34	20.92
92.56	12.37	13.58	14.60	15.65	16.73	17.58	18.52	19.25	19.90	20.50	21.06
98.85	12.28	13.28	14.46	15.45	16.50	17.44	18.27	18.98	19.63	20.32	20.84
105.64	12.73	13.88	14.90	16.04	16.92	17.87	18.78	19.45	20.23	20.83	21.45
112.96	12.94	14.03	15.18	16.24	17.25	18.16	19.02	19.83	20.53	21.16	21.74
120.85	12.96	14.11	15.26	16.36	17.31	18.16	19.04	19.92	20.56	21.24	21.85
129.36	13.25	14.43	15.50	16.60	17.71	18.61	19.50	20.24	20.98	21.61	22.31

138.55	13.47	14.55	15.64	16.70	17.80	18.77	19.57	20.46	21.11	21.81	22.35
148.45	13.42	14.50	15.60	16.65	17.69	18.62	19.48	20.26	21.06	21.72	22.32
159.13	13.66	14.75	15.91	16.98	18.04	19.01	19.83	20.66	21.39	22.06	22.63
170.65	13.92	14.99	16.19	17.32	18.29	19.28	20.13	20.93	21.69	22.33	22.87
183.07	14.27	15.44	16.58	17.61	18.68	19.67	20.50	21.31	22.12	22.81	23.41
196.47	14.30	15.50	16.62	17.77	18.78	19.72	20.62	21.43	22.19	22.84	23.50
210.91	14.67	15.79	16.96	18.12	19.10	20.13	21.07	21.87	22.53	23.23	23.89
226.50	14.94	16.04	17.19	18.30	19.27	20.41	21.23	22.02	22.80	23.46	24.14
243.31	14.96	16.19	17.36	18.43	19.45	20.48	21.37	22.21	22.99	23.61	24.25
261.43	15.49	16.70	17.86	18.94	20.04	21.09	21.95	22.79	23.56	24.22	24.96
280.98	15.53	16.80	17.95	19.02	20.11	21.07	22.05	22.85	23.65	24.38	24.97
302.07	15.75	17.01	18.17	19.26	20.30	21.31	22.18	23.08	23.81	24.50	25.16
324.81	16.11	17.31	18.48	19.62	20.71	21.68	22.69	23.51	24.27	25.00	25.69
349.33	16.35	17.46	18.70	19.87	20.89	21.84	22.91	23.68	24.48	25.20	25.87
375.79	16.60	17.78	19.02	20.16	21.25	22.29	23.17	24.06	24.80	25.55	26.19
404.31	16.63	17.78	19.02	20.20	21.20	22.32	23.16	24.05	24.87	25.58	26.22
435.08	17.02	18.21	19.45	20.58	21.66	22.65	23.56	24.49	25.20	26.04	26.68
468.27	17.17	18.27	19.58	20.71	21.78	22.91	23.80	24.65	25.40	26.16	26.88
504.06	17.53	18.71	19.98	21.14	22.28	23.26	24.22	25.11	25.89	26.58	27.35
542.66	17.68	18.85	20.05	21.21	22.25	23.28	24.35	25.12	26.00	26.72	27.35
584.29	17.90	19.10	20.28	21.43	22.67	23.62	24.65	25.40	26.27	26.89	27.62
629.18	18.10	19.35	20.62	21.71	22.80	23.81	24.80	25.67	26.48	27.21	27.89
677.61	18.48	19.67	20.94	22.12	23.27	24.31	25.35	26.13	26.95	27.75	28.42
729.83	18.55	19.65	20.95	22.12	23.20	24.28	25.22	26.04	26.97	27.63	28.36
786.16	18.71	19.97	21.18	22.39	23.46	24.58	25.45	26.42	27.28	27.98	28.65
846.90	18.71	19.97	21.21	22.40	23.55	24.59	25.48	26.41	27.24	28.04	28.70
912.42	19.02	20.24	21.44	22.62	23.76	24.87	25.73	26.64	27.45	28.26	28.93
983.08	19.04	20.38	21.58	22.76	23.96	24.96	26.04	26.86	27.61	28.38	29.08
1059.29	19.31	20.51	21.74	22.98	24.04	25.16	26.12	26.92	27.77	28.63	29.25
1141.48	19.18	20.46	21.71	22.92	24.01	25.02	26.07	26.96	27.76	28.57	29.22
1230.13	19.24	20.40	21.65	22.89	23.99	25.02	26.00	26.92	27.72	28.42	29.10
1325.73	19.45	20.61	21.84	23.04	24.17	25.25	26.25	27.09	27.90	28.67	29.34
1428.84	19.32	20.50	21.85	23.02	24.07	25.14	26.21	27.02	27.94	28.57	29.32
1540.05	19.53	20.77	22.03	23.19	24.28	25.35	26.43	27.34	28.08	28.86	29.56
1659.99	19.50	20.79	22.07	23.11	24.35	25.33	26.46	27.24	28.15	28.87	29.56
1789.34	19.45	20.64	21.93	23.19	24.21	25.24	26.29	27.15	27.99	28.75	29.47
1928.85	19.50	20.76	22.02	23.30	24.36	25.37	26.40	27.30	28.09	28.88	29.53
2079.31	19.52	20.75	22.02	23.15	24.31	25.47	26.32	27.27	27.98	28.73	29.43
2241.59	19.50	20.77	21.99	23.18	24.20	25.34	26.28	27.14	28.02	28.72	29.47
2416.60	19.47	20.61	21.95	23.04	24.17	25.34	26.31	27.17	27.93	28.71	29.42
2605.36	19.40	20.60	21.91	23.08	24.27	25.40	26.26	27.22	27.92	28.66	29.38
2808.94	19.38	20.61	21.84	23.02	24.10	25.13	26.14	27.03	27.80	28.55	29.19
3028.50	19.27	20.49	21.77	22.96	24.05	25.13	26.05	26.86	27.81	28.45	29.05
3265.30	19.23	20.50	21.75	22.87	24.01	25.13	26.05	26.91	27.78	28.51	29.11
3520.69	19.10	20.37	21.59	22.78	23.81	24.88	25.86	26.66	27.48	28.20	28.84
3796.13	19.07	20.23	21.51	22.69	23.77	24.85	25.73	26.54	27.45	28.07	28.64

Time (ps) \ Wavelength (nm)	569.06	570.48	571.91	573.33	574.75	576.18	577.60	579.03	580.45	581.87	583.30
-3.78	-0.03	-0.03	-0.04	-0.02	-0.03	0.02	0.00	-0.07	0.05	-0.04	0.00
-3.28	0.00	-0.01	-0.02	-0.01	-0.01	-0.06	-0.05	-0.03	-0.06	0.08	-0.03
-2.78	0.01	-0.08	0.00	-0.03	0.00	0.01	0.00	-0.02	0.01	0.02	-0.04
-2.28	-0.03	-0.01	0.04	0.03	0.05	0.04	0.06	0.00	0.05	0.05	-0.02
-1.78	-0.03	0.07	-0.02	0.00	-0.01	-0.06	0.00	0.03	-0.01	0.02	0.02
-1.28	0.10	0.02	0.01	0.02	0.04	0.03	0.05	0.04	0.05	0.03	0.05
-0.78	-0.03	0.02	0.00	0.03	-0.03	0.04	0.03	0.04	-0.02	-0.09	-0.01
-0.28	0.01	0.01	0.02	-0.02	-0.01	-0.01	-0.09	0.03	-0.06	-0.06	0.03
-0.18	-0.01	0.02	0.02	0.03	-0.04	0.02	0.06	0.04	0.00	-0.05	0.05
-0.08	-0.07	0.02	0.00	0.01	-0.02	-0.02	0.01	-0.04	-0.04	-0.01	-0.02
0.02	0.05	-0.01	0.06	0.08	0.05	0.05	0.12	0.12	0.05	0.06	0.04
0.12	-0.07	0.06	0.06	-0.02	0.05	0.05	0.08	0.05	0.07	0.06	0.03
0.22	-0.01	0.00	0.01	0.03	-0.01	0.05	0.00	-0.04	0.04	0.00	-0.03
0.27	0.06	0.03	0.11	0.09	0.06	0.06	0.03	-0.01	0.00	0.03	0.00
0.32	0.01	0.07	0.05	0.08	0.10	0.06	0.02	0.01	-0.05	-0.01	0.04
0.37	0.02	-0.03	-0.05	0.02	0.04	-0.02	-0.01	0.01	-0.05	0.04	0.02
0.42	-0.08	-0.02	0.08	0.05	0.05	0.04	0.02	-0.01	0.03	0.00	0.00
0.47	0.03	0.00	0.01	0.04	-0.05	0.07	0.01	-0.01	-0.01	-0.02	-0.06
0.52	-0.06	-0.06	-0.03	0.01	0.06	0.01	0.07	-0.07	0.02	-0.08	-0.09
0.57	0.07	0.07	-0.01	0.06	-0.01	0.03	0.06	0.01	-0.01	0.07	0.08
0.62	0.06	-0.08	-0.02	-0.02	-0.02	0.06	-0.01	-0.06	-0.03	-0.05	-0.06
0.67	-0.03	-0.05	-0.01	0.05	-0.04	-0.01	-0.01	-0.02	-0.02	0.03	-0.04
0.72	0.00	-0.06	-0.01	0.00	0.01	0.01	0.01	-0.11	-0.01	0.04	0.04
0.77	0.00	-0.03	0.00	0.03	-0.01	0.03	0.00	-0.01	0.04	0.02	-0.06
0.82	-0.04	0.02	-0.02	0.05	-0.01	0.07	-0.05	-0.08	-0.04	-0.03	0.04
0.87	-0.02	-0.03	0.03	0.01	0.00	-0.02	0.02	-0.02	0.03	0.00	-0.03
0.92	-0.01	0.02	-0.07	0.03	0.01	-0.03	0.02	0.03	-0.03	0.05	0.04
0.97	0.01	0.00	0.01	0.02	0.05	0.08	-0.06	0.04	-0.02	0.03	-0.05
1.02	0.02	0.00	0.02	0.07	0.08	0.01	0.05	0.01	-0.06	0.05	-0.05
1.07	0.03	-0.06	-0.01	0.09	0.00	-0.02	-0.01	-0.09	-0.02	-0.05	-0.10
1.12	-0.04	0.11	0.00	-0.01	0.04	0.05	-0.06	-0.06	0.02	0.10	-0.03
1.17	-0.19	-0.11	0.08	0.16	-0.05	-0.07	0.00	0.09	0.08	-0.05	-0.14
1.22	-0.02	0.06	0.00	-0.07	0.03	0.04	0.00	-0.04	-0.05	-0.01	-0.04
1.27	-0.90	-1.35	-1.48	-1.46	-1.19	-0.69	-0.32	-0.02	0.19	0.03	-0.24
1.32	-7.71	-10.86	-13.47	-15.20	-16.62	-17.41	-18.26	-19.01	-20.31	-21.67	-23.35
1.37	91.91	99.31	103.99	107.48	108.44	107.90	105.86	103.06	99.06	94.80	89.46
1.42	-1.16	-2.86	-5.06	-7.69	-10.70	-13.83	-17.01	-20.00	-22.70	-24.89	-26.38
1.47	7.41	8.49	9.39	10.29	11.02	11.69	12.34	12.76	13.35	13.68	14.00
1.52	5.97	6.66	7.12	7.60	8.00	8.42	8.74	8.95	9.22	9.50	9.65
1.57	5.21	5.79	6.31	6.78	7.25	7.62	8.01	8.37	8.67	9.02	9.24
1.62	4.95	5.56	6.20	6.85	7.38	7.93	8.32	8.81	9.25	9.63	9.97
1.67	6.17	6.53	7.02	7.44	7.74	8.05	8.34	8.50	8.78	8.95	9.07
1.72	6.21	6.91	7.33	7.92	8.25	8.69	9.03	9.22	9.50	9.66	9.80
1.77	6.93	7.20	7.56	7.91	8.19	8.44	8.70	8.88	9.14	9.23	9.41
1.82	7.04	7.41	7.82	8.23	8.52	8.73	8.98	9.17	9.37	9.46	9.54
1.87	8.06	8.47	8.98	9.34	9.76	10.08	10.40	10.70	10.88	10.96	11.09
1.92	8.14	8.59	8.85	9.19	9.44	9.74	9.91	10.00	10.21	10.34	10.44

1.97	8.73	9.00	9.43	9.78	10.08	10.23	10.44	10.41	10.55	10.64	10.70
2.02	8.41	8.79	9.20	9.57	9.84	10.13	10.34	10.42	10.71	10.91	10.98
2.07	9.33	9.68	10.01	10.31	10.47	10.68	10.87	10.87	11.05	11.09	11.26
2.12	9.11	9.59	9.97	10.33	10.57	10.69	10.92	11.02	11.12	11.19	11.22
2.17	9.01	9.32	9.61	9.89	10.11	10.33	10.53	10.62	10.71	10.76	10.88
2.22	10.01	10.34	10.73	10.95	11.11	11.38	11.48	11.57	11.59	11.58	11.63
2.27	10.37	10.85	11.21	11.47	11.69	12.00	12.20	12.18	12.41	12.51	12.45
2.32	10.09	10.36	10.72	10.95	11.14	11.28	11.45	11.48	11.48	11.52	11.54
2.37	10.63	10.97	11.26	11.60	11.70	11.84	12.00	12.03	12.10	12.08	12.02
2.42	10.74	11.09	11.35	11.71	11.95	12.19	12.31	12.38	12.47	12.50	12.46
2.47	11.06	11.46	11.66	11.95	12.13	12.26	12.39	12.49	12.58	12.48	12.56
2.52	11.36	11.62	12.00	12.27	12.39	12.66	12.70	12.73	12.74	12.75	12.63
2.57	11.32	11.84	11.99	12.32	12.48	12.61	12.80	12.85	12.97	12.99	13.00
2.62	11.82	12.15	12.49	12.74	12.92	13.01	13.14	13.13	13.10	13.15	13.10
2.67	11.44	11.83	12.15	12.43	12.59	12.82	12.81	12.88	12.94	12.90	12.89
2.72	11.88	12.24	12.53	12.73	12.93	13.11	13.12	13.15	13.07	13.12	13.09
2.77	12.02	12.32	12.58	12.84	12.99	13.12	13.20	13.26	13.23	13.20	13.11
2.82	12.08	12.37	12.73	12.94	13.14	13.36	13.37	13.49	13.39	13.42	13.40
2.87	12.15	12.42	12.68	12.89	12.97	13.18	13.15	13.23	13.25	13.24	13.11
2.92	12.68	13.04	13.39	13.58	13.69	13.85	13.98	14.04	14.00	14.00	13.83
2.97	12.44	12.73	13.01	13.25	13.43	13.61	13.63	13.69	13.75	13.67	13.53
3.02	12.86	13.22	13.53	13.84	14.02	14.04	14.11	14.16	14.19	14.12	14.00
3.07	12.53	12.93	13.20	13.40	13.62	13.71	13.83	13.73	13.86	13.68	13.71
3.12	12.64	13.08	13.34	13.61	13.83	13.89	13.96	13.92	13.92	13.98	13.87
3.17	12.95	13.34	13.58	13.79	13.88	14.04	14.11	14.04	14.05	13.95	13.86
3.22	13.31	13.61	13.97	14.32	14.48	14.62	14.69	14.67	14.71	14.64	14.62
3.42	13.73	14.09	14.40	14.66	14.84	14.85	14.99	14.98	14.95	14.88	14.79
3.62	14.03	14.50	14.79	15.00	15.23	15.33	15.42	15.39	15.48	15.40	15.35
3.82	14.09	14.50	14.80	14.97	15.17	15.22	15.33	15.39	15.28	15.22	15.13
4.02	14.55	14.91	15.20	15.49	15.64	15.78	15.83	15.81	15.88	15.80	15.69
4.22	14.34	14.87	14.99	15.31	15.42	15.54	15.65	15.55	15.54	15.53	15.40
4.42	14.65	15.08	15.34	15.57	15.84	15.94	15.97	15.98	15.97	15.86	15.78
4.62	15.02	15.38	15.66	15.94	16.10	16.23	16.29	16.30	16.28	16.17	16.05
4.82	14.96	15.33	15.62	16.00	16.18	16.16	16.28	16.22	16.24	16.15	16.03
5.02	15.28	15.57	15.96	16.25	16.42	16.55	16.58	16.57	16.56	16.42	16.41
5.22	15.40	15.81	16.10	16.38	16.52	16.67	16.71	16.76	16.73	16.63	16.58
5.42	15.54	15.92	16.23	16.55	16.70	16.84	16.82	16.91	16.81	16.71	16.65
5.62	15.77	16.13	16.42	16.73	16.81	17.02	17.05	17.06	17.01	16.95	16.94
5.82	15.79	16.13	16.44	16.72	16.83	17.01	17.06	17.01	17.00	16.96	16.82
6.02	15.96	16.41	16.64	16.84	17.16	17.22	17.27	17.30	17.18	17.11	17.04
6.22	15.98	16.34	16.56	16.86	17.05	17.10	17.25	17.19	17.17	16.99	16.91
6.42	16.04	16.44	16.76	17.02	17.21	17.28	17.41	17.37	17.33	17.27	17.09
6.62	16.48	16.92	17.21	17.56	17.64	17.76	17.91	17.78	17.77	17.63	17.56
6.82	16.33	16.73	17.03	17.30	17.46	17.59	17.68	17.60	17.58	17.45	17.36
7.02	16.65	17.03	17.35	17.65	17.84	17.97	17.97	18.02	17.96	17.77	17.74
7.22	16.52	16.88	17.27	17.51	17.68	17.82	17.86	17.89	17.84	17.71	17.61
7.42	16.76	17.18	17.62	17.83	17.95	18.13	18.14	18.15	18.13	18.01	17.93
7.62	16.73	17.06	17.36	17.69	17.94	17.88	17.96	17.94	17.92	17.87	17.74
7.82	16.72	17.17	17.48	17.82	17.84	18.05	18.07	18.02	18.00	17.96	17.77
8.02	16.64	17.07	17.36	17.67	17.84	17.90	17.97	17.97	17.92	17.76	17.65

8.22	16.72	17.10	17.47	17.75	17.87	17.94	18.03	18.02	17.98	17.85	17.74
8.42	16.59	17.02	17.25	17.55	17.71	17.81	17.92	17.93	17.79	17.69	17.55
8.62	16.85	17.28	17.60	17.87	17.97	18.14	18.18	18.16	18.18	18.04	17.94
8.82	17.14	17.46	17.85	18.11	18.26	18.36	18.35	18.30	18.38	18.25	18.14
9.02	16.80	17.20	17.49	17.75	17.94	17.97	18.03	18.01	18.08	17.87	17.81
9.22	16.97	17.36	17.76	17.98	18.12	18.19	18.23	18.21	18.18	18.05	17.93
9.42	17.21	17.48	17.87	18.17	18.31	18.46	18.44	18.41	18.35	18.31	18.14
9.62	17.11	17.41	17.74	17.98	18.25	18.30	18.34	18.30	18.26	18.14	18.01
9.82	17.23	17.61	18.01	18.24	18.28	18.54	18.51	18.51	18.42	18.36	18.26
10.02	17.09	17.40	17.81	18.04	18.26	18.31	18.38	18.37	18.32	18.19	17.97
10.22	17.25	17.68	18.00	18.34	18.51	18.56	18.63	18.60	18.57	18.42	18.28
10.42	17.35	17.83	18.01	18.27	18.49	18.65	18.76	18.62	18.52	18.47	18.36
10.62	17.38	17.75	18.13	18.45	18.48	18.63	18.64	18.61	18.62	18.42	18.33
10.82	17.26	17.62	17.92	18.19	18.39	18.44	18.53	18.51	18.46	18.26	18.24
11.02	17.32	17.69	18.00	18.33	18.47	18.55	18.64	18.51	18.55	18.45	18.27
11.22	17.18	17.50	17.84	18.09	18.26	18.36	18.41	18.36	18.34	18.20	18.07
11.42	17.09	17.40	17.69	17.97	18.06	18.23	18.20	18.18	18.11	18.02	17.87
11.62	17.32	17.67	17.99	18.29	18.42	18.53	18.61	18.58	18.50	18.42	18.34
11.82	17.32	17.72	17.96	18.30	18.47	18.58	18.54	18.55	18.53	18.43	18.20
12.02	17.49	17.76	18.08	18.30	18.45	18.58	18.65	18.63	18.53	18.42	18.29
12.22	17.60	17.88	18.21	18.48	18.64	18.76	18.90	18.93	18.83	18.64	18.51
12.42	17.21	17.58	17.87	18.22	18.30	18.39	18.48	18.41	18.39	18.24	18.08
12.62	17.36	17.75	18.08	18.35	18.55	18.53	18.65	18.61	18.65	18.42	18.31
12.82	17.33	17.68	18.04	18.33	18.43	18.55	18.63	18.56	18.49	18.42	18.30
13.02	17.63	17.98	18.38	18.67	18.79	18.88	18.99	18.94	18.93	18.79	18.63
13.22	17.51	17.89	18.24	18.49	18.70	18.75	18.83	18.73	18.67	18.57	18.42
13.42	17.54	17.82	18.13	18.48	18.58	18.69	18.76	18.69	18.69	18.52	18.42
13.50	17.47	17.78	18.14	18.42	18.55	18.67	18.74	18.66	18.51	18.44	18.33
13.59	17.69	18.04	18.45	18.68	18.84	18.96	18.92	18.95	18.84	18.82	18.59
13.68	17.52	17.90	18.23	18.47	18.51	18.65	18.77	18.70	18.65	18.58	18.36
13.78	17.23	17.60	17.91	18.21	18.36	18.48	18.53	18.47	18.38	18.23	18.13
13.88	17.44	17.84	18.17	18.40	18.57	18.69	18.69	18.63	18.67	18.50	18.41
14.00	17.72	18.08	18.43	18.71	18.88	19.00	19.03	18.97	18.89	18.83	18.66
14.12	17.62	18.00	18.31	18.59	18.75	18.89	18.83	18.84	18.78	18.61	18.52
14.25	17.48	17.80	18.19	18.41	18.45	18.66	18.77	18.68	18.52	18.43	18.28
14.40	17.94	18.26	18.64	18.81	19.08	19.21	19.21	19.18	19.09	19.01	18.85
14.55	17.49	17.88	18.19	18.46	18.55	18.67	18.69	18.83	18.66	18.51	18.42
14.72	17.71	18.03	18.36	18.57	18.79	18.93	18.84	18.77	18.77	18.61	18.52
14.90	17.50	17.88	18.16	18.46	18.54	18.65	18.77	18.67	18.66	18.54	18.32
15.09	17.48	17.83	18.12	18.46	18.55	18.74	18.72	18.64	18.51	18.51	18.28
15.30	17.61	18.01	18.39	18.63	18.77	18.86	18.90	18.82	18.84	18.72	18.69
15.53	17.97	18.39	18.69	18.94	19.24	19.24	19.23	19.27	19.18	19.14	18.91
15.77	17.77	18.00	18.39	18.63	18.74	18.97	19.04	19.00	18.91	18.74	18.62
16.04	17.91	18.29	18.61	18.87	19.06	19.15	19.16	19.19	19.10	18.99	18.86
16.32	17.83	18.20	18.42	18.83	18.97	18.99	19.06	19.01	18.94	18.88	18.71
16.63	18.09	18.47	18.83	18.97	19.19	19.25	19.32	19.29	19.25	19.13	18.99
16.96	18.13	18.48	18.78	19.14	19.22	19.28	19.38	19.48	19.34	19.24	19.09
17.31	18.02	18.29	18.63	18.91	19.16	19.18	19.17	19.18	19.09	18.90	18.86
17.70	18.38	18.82	19.10	19.36	19.53	19.66	19.59	19.65	19.58	19.44	19.39
18.11	17.64	17.99	18.29	18.59	18.69	18.84	18.88	18.79	18.77	18.69	18.55

18.56	17.91	18.29	18.64	18.88	19.04	19.17	19.20	19.21	19.12	18.99	18.88
19.04	17.96	18.33	18.64	18.93	19.11	19.27	19.26	19.14	19.15	19.10	18.91
19.56	18.29	18.66	19.05	19.25	19.50	19.63	19.60	19.55	19.52	19.40	19.26
20.12	18.28	18.63	19.01	19.26	19.39	19.55	19.57	19.58	19.59	19.38	19.21
20.72	18.40	18.76	19.15	19.48	19.56	19.62	19.65	19.69	19.65	19.58	19.39
21.37	18.42	18.74	19.07	19.32	19.51	19.65	19.72	19.63	19.57	19.46	19.38
22.08	18.36	18.78	19.07	19.39	19.48	19.61	19.67	19.65	19.61	19.52	19.39
22.84	18.53	18.94	19.17	19.54	19.73	19.80	19.88	19.85	19.81	19.78	19.54
23.65	18.17	18.59	18.87	19.11	19.26	19.38	19.34	19.37	19.32	19.20	19.10
24.54	18.65	19.02	19.32	19.58	19.78	19.92	19.95	19.90	19.88	19.73	19.52
25.49	18.89	19.22	19.57	19.79	20.03	20.13	20.14	20.11	20.15	19.94	19.81
26.51	18.30	18.67	19.01	19.23	19.38	19.49	19.54	19.52	19.52	19.46	19.22
27.62	18.97	19.37	19.73	20.02	20.18	20.25	20.34	20.28	20.17	20.14	20.00
28.81	18.98	19.33	19.61	19.93	20.10	20.20	20.29	20.28	20.20	20.09	19.92
30.10	18.58	18.96	19.30	19.56	19.73	19.89	19.95	19.90	19.81	19.65	19.60
31.49	19.24	19.62	19.93	20.15	20.36	20.49	20.53	20.51	20.52	20.42	20.24
32.98	19.00	19.32	19.68	20.02	20.10	20.30	20.33	20.24	20.27	20.16	20.00
34.60	19.39	19.80	20.16	20.41	20.55	20.72	20.81	20.82	20.76	20.61	20.47
36.34	19.26	19.62	19.99	20.29	20.46	20.59	20.59	20.62	20.54	20.41	20.25
38.21	19.77	20.12	20.50	20.76	21.01	21.07	21.15	21.18	21.12	21.06	20.94
40.24	19.55	19.98	20.29	20.54	20.75	20.86	20.87	20.92	20.88	20.74	20.62
42.42	19.28	19.68	20.04	20.31	20.41	20.66	20.70	20.51	20.60	20.51	20.37
44.78	19.42	19.73	20.14	20.44	20.68	20.76	20.77	20.79	20.78	20.79	20.53
47.32	19.44	19.83	20.12	20.48	20.65	20.71	20.79	20.82	20.74	20.68	20.52
50.06	19.78	20.12	20.54	20.84	20.96	21.13	21.21	21.15	21.20	21.03	20.96
53.01	19.72	20.23	20.54	20.92	20.93	21.19	21.26	21.22	21.21	21.11	20.97
56.20	20.09	20.47	20.87	21.12	21.42	21.38	21.54	21.55	21.56	21.41	21.25
59.64	20.59	20.96	21.29	21.53	21.75	21.94	21.98	21.95	22.01	21.90	21.75
63.34	19.91	20.27	20.62	20.89	21.06	21.26	21.39	21.33	21.31	21.19	21.11
67.34	20.31	20.71	21.08	21.39	21.59	21.71	21.84	21.80	21.74	21.70	21.56
71.65	20.72	21.17	21.51	21.83	21.97	22.18	22.31	22.26	22.21	22.14	22.07
76.30	20.64	21.14	21.50	21.78	21.93	22.19	22.25	22.23	22.30	22.22	22.03
81.32	21.17	21.66	21.99	22.27	22.57	22.74	22.80	22.84	22.88	22.79	22.65
86.73	21.45	21.83	22.25	22.58	22.80	22.92	23.03	23.08	23.05	22.93	22.88
92.56	21.54	22.04	22.40	22.73	22.97	23.13	23.20	23.24	23.21	23.18	23.06
98.85	21.24	21.64	22.15	22.40	22.74	22.83	22.91	23.00	22.94	22.92	22.87
105.64	22.00	22.40	22.83	23.15	23.37	23.51	23.67	23.66	23.71	23.60	23.56
112.96	22.27	22.71	23.08	23.43	23.72	23.81	23.95	23.96	23.95	23.96	23.85
120.85	22.31	22.79	23.15	23.46	23.69	23.94	23.94	24.08	24.08	24.03	23.90
129.36	22.79	23.22	23.59	23.90	24.20	24.40	24.50	24.45	24.50	24.59	24.41
138.55	22.88	23.37	23.74	24.08	24.37	24.50	24.62	24.77	24.77	24.75	24.65
148.45	22.78	23.24	23.62	24.02	24.31	24.44	24.53	24.61	24.59	24.58	24.45
159.13	23.14	23.64	24.07	24.38	24.69	24.78	24.89	24.95	25.01	24.90	24.94
170.65	23.49	23.88	24.36	24.69	24.99	25.14	25.25	25.41	25.45	25.40	25.31
183.07	23.89	24.45	24.81	25.22	25.43	25.69	25.80	25.87	25.90	25.92	25.77
196.47	24.00	24.51	24.82	25.27	25.48	25.79	25.91	25.97	25.96	25.94	25.85
210.91	24.44	24.95	25.31	25.72	25.99	26.26	26.34	26.45	26.40	26.49	26.42
226.50	24.65	25.11	25.59	25.91	26.19	26.36	26.61	26.63	26.69	26.61	26.67
243.31	24.82	25.25	25.72	26.10	26.40	26.60	26.75	26.79	26.93	26.89	26.81
261.43	25.49	25.97	26.39	26.84	27.02	27.30	27.54	27.61	27.67	27.65	27.59

280.98	25.50	25.99	26.46	26.78	27.13	27.34	27.40	27.58	27.65	27.68	27.59
302.07	25.77	26.26	26.57	27.07	27.29	27.55	27.71	27.85	27.91	27.85	27.87
324.81	26.24	26.73	27.12	27.55	27.82	28.05	28.26	28.35	28.40	28.36	28.44
349.33	26.51	26.90	27.30	27.73	28.02	28.19	28.42	28.59	28.62	28.68	28.57
375.79	26.74	27.33	27.76	28.10	28.34	28.66	28.82	28.90	29.05	29.05	29.06
404.31	26.83	27.31	27.75	28.13	28.41	28.66	28.79	28.99	29.00	29.02	28.97
435.08	27.14	27.71	28.20	28.66	28.90	29.18	29.35	29.39	29.58	29.62	29.57
468.27	27.48	27.96	28.36	28.83	29.03	29.38	29.52	29.67	29.72	29.70	29.74
504.06	27.91	28.47	28.80	29.24	29.57	29.85	30.02	30.15	30.18	30.17	30.17
542.66	28.00	28.54	28.96	29.29	29.58	29.81	30.09	30.18	30.26	30.35	30.29
584.29	28.29	28.74	29.23	29.61	29.84	30.11	30.32	30.44	30.62	30.61	30.53
629.18	28.43	28.94	29.43	29.83	30.20	30.31	30.65	30.74	30.78	30.79	30.81
677.61	28.90	29.52	29.99	30.37	30.67	31.02	31.20	31.31	31.35	31.39	31.43
729.83	28.92	29.41	29.94	30.30	30.53	30.89	31.08	31.14	31.16	31.29	31.30
786.16	29.25	29.77	30.23	30.60	30.93	31.20	31.36	31.53	31.56	31.68	31.64
846.90	29.26	29.81	30.25	30.64	30.90	31.23	31.41	31.52	31.57	31.67	31.60
912.42	29.55	30.06	30.46	30.95	31.23	31.52	31.56	31.82	31.85	31.90	31.88
983.08	29.67	30.20	30.65	31.10	31.30	31.58	31.77	31.89	31.95	32.09	31.99
1059.29	29.86	30.34	30.84	31.20	31.59	31.84	31.99	32.11	32.21	32.26	32.17
1141.48	29.77	30.26	30.72	31.13	31.46	31.66	31.81	32.02	32.12	32.09	32.09
1230.13	29.70	30.19	30.60	31.08	31.28	31.63	31.83	32.02	32.00	32.00	32.06
1325.73	29.90	30.41	30.91	31.28	31.58	31.81	32.01	32.15	32.24	32.24	32.21
1428.84	29.86	30.36	30.82	31.27	31.54	31.76	31.98	32.07	32.17	32.19	32.15
1540.05	30.02	30.59	31.04	31.49	31.78	32.01	32.21	32.30	32.36	32.38	32.36
1659.99	30.11	30.61	31.10	31.49	31.75	31.99	32.21	32.28	32.37	32.37	32.31
1789.34	30.01	30.50	30.94	31.30	31.61	31.83	32.02	32.12	32.20	32.23	32.18
1928.85	30.07	30.56	31.05	31.37	31.62	31.94	32.08	32.22	32.20	32.31	32.28
2079.31	30.05	30.56	30.89	31.41	31.64	31.90	31.95	32.14	32.17	32.17	32.19
2241.59	29.94	30.47	30.87	31.24	31.51	31.75	31.94	32.01	32.09	32.11	32.10
2416.60	29.87	30.39	30.83	31.23	31.44	31.69	31.78	31.93	32.03	32.04	32.01
2605.36	29.85	30.38	30.82	31.21	31.47	31.60	31.80	31.87	31.96	32.03	31.99
2808.94	29.74	30.23	30.61	31.00	31.29	31.48	31.65	31.73	31.83	31.86	31.77
3028.50	29.71	30.11	30.46	30.82	31.07	31.33	31.45	31.50	31.62	31.63	31.54
3265.30	29.62	30.07	30.45	30.87	31.05	31.37	31.52	31.51	31.59	31.53	31.59
3520.69	29.34	29.82	30.21	30.54	30.78	30.98	31.15	31.21	31.31	31.25	31.22
3796.13	29.20	29.61	30.06	30.48	30.62	30.73	30.93	31.04	31.10	31.08	31.04

Wavelength (nm) / Time (ps)	584.72	586.15	587.57	588.99	590.42	591.84	593.27	594.69	596.12	597.54	598.96
-3.78	0.04	0.01	-0.03	-0.07	-0.07	-0.02	-0.03	-0.03	-0.01	-0.01	-0.01
-3.28	-0.04	-0.02	0.03	-0.04	-0.05	0.05	-0.03	0.06	-0.02	-0.05	0.06
-2.78	0.00	-0.03	-0.01	0.01	0.09	-0.04	-0.01	-0.01	-0.02	0.01	0.01
-2.28	0.02	0.00	-0.01	-0.02	0.03	-0.02	0.02	0.04	-0.01	0.04	0.02
-1.78	-0.02	0.06	0.03	0.05	0.00	0.03	0.02	-0.01	0.03	0.04	-0.04
-1.28	-0.07	0.02	0.05	-0.01	-0.05	0.00	0.03	-0.03	0.03	-0.04	0.00
-0.78	0.01	0.03	0.02	0.05	-0.02	-0.01	0.00	0.02	-0.01	0.04	0.00
-0.28	0.05	-0.07	-0.09	0.04	0.06	0.02	0.00	-0.04	0.00	-0.04	-0.04
-0.18	0.03	0.00	0.03	0.00	-0.03	0.11	-0.01	-0.03	-0.04	0.04	0.02

-0.08	0.00	-0.07	-0.02	0.02	-0.01	-0.08	0.04	-0.02	0.00	-0.03	-0.03
0.02	-0.04	0.01	0.00	0.05	0.03	0.08	0.05	0.05	0.03	0.06	0.11
0.12	0.04	0.08	0.05	-0.01	0.08	0.04	0.00	0.03	-0.04	0.11	0.06
0.22	-0.09	-0.02	-0.01	0.03	-0.07	0.00	0.01	-0.01	0.05	-0.06	-0.06
0.27	-0.01	0.09	-0.01	-0.04	0.01	-0.01	0.07	0.01	-0.02	0.08	-0.05
0.32	0.08	0.07	-0.01	0.03	0.10	0.02	-0.02	0.07	0.01	0.07	0.06
0.37	-0.09	0.03	0.00	-0.03	0.01	0.01	-0.01	0.00	0.03	0.00	0.05
0.42	-0.05	-0.01	-0.08	-0.01	-0.01	0.01	-0.06	-0.10	0.03	0.01	0.07
0.47	0.04	-0.02	0.07	0.04	0.01	-0.04	0.05	0.04	0.06	0.04	0.06
0.52	-0.03	0.03	0.01	0.01	0.03	0.04	-0.04	-0.09	-0.05	-0.02	-0.06
0.57	0.03	0.06	0.06	0.04	0.05	0.05	0.02	0.00	0.01	0.04	0.11
0.62	0.03	0.07	-0.05	0.06	-0.03	0.09	0.05	0.01	0.03	-0.03	-0.05
0.67	0.01	-0.06	0.01	-0.05	0.01	-0.01	0.05	0.02	-0.04	0.01	-0.01
0.72	-0.11	-0.02	-0.05	0.01	0.01	-0.06	0.00	0.02	-0.08	0.01	-0.01
0.77	0.01	0.07	0.00	-0.01	0.02	0.07	0.01	0.04	0.00	0.02	0.05
0.82	-0.07	0.04	0.00	0.04	0.05	0.03	0.06	-0.05	0.01	-0.03	0.02
0.87	0.04	0.07	-0.02	-0.03	-0.01	0.04	0.02	-0.06	0.01	0.04	-0.02
0.92	-0.02	-0.01	0.02	0.06	0.03	-0.02	-0.09	0.06	-0.02	-0.04	0.00
0.97	0.00	0.02	-0.04	-0.04	0.01	0.08	-0.02	-0.02	0.01	0.02	-0.02
1.02	-0.01	0.06	-0.02	0.04	0.03	0.08	0.08	0.03	-0.03	0.00	-0.07
1.07	-0.05	-0.03	-0.03	0.00	0.05	0.04	-0.03	0.06	-0.01	-0.04	-0.03
1.12	-0.06	0.01	-0.04	-0.04	-0.02	0.06	0.04	0.03	0.01	0.02	0.05
1.17	-0.03	0.07	0.06	-0.03	-0.02	-0.05	0.01	0.08	0.02	-0.05	-0.03
1.22	-0.07	-0.08	0.00	0.00	0.01	-0.06	-0.06	0.03	0.04	0.01	-0.04
1.27	-0.40	-0.45	-0.49	-0.49	-0.38	-0.25	-0.06	0.10	0.21	0.26	0.16
1.32	-25.12	-26.73	-28.07	-28.79	-28.68	-27.92	-26.23	-23.84	-20.83	-17.31	-13.39
1.37	84.47	79.38	72.80	66.50	59.99	52.58	44.59	36.99	28.76	21.28	14.25
1.42	-27.28	-27.20	-26.50	-24.86	-22.46	-19.49	-16.05	-12.03	-7.77	-3.02	1.97
1.47	14.27	14.54	14.55	14.57	14.52	14.34	14.14	13.88	13.46	13.10	12.81
1.52	9.89	10.15	10.39	10.66	11.00	11.30	11.62	12.00	12.41	12.75	13.20
1.57	9.42	9.72	9.85	10.02	10.18	10.27	10.47	10.59	10.73	10.96	11.17
1.62	10.31	10.69	10.84	11.15	11.46	11.66	11.79	11.97	11.95	11.97	11.92
1.67	9.28	9.44	9.63	9.76	9.92	10.20	10.37	10.64	10.84	11.09	11.34
1.72	9.91	10.17	10.14	10.17	10.26	10.36	10.31	10.37	10.31	10.35	10.46
1.77	9.60	9.85	9.97	10.22	10.34	10.69	10.78	11.04	11.17	11.34	11.54
1.82	9.65	9.87	9.89	10.07	10.14	10.21	10.38	10.44	10.69	10.78	11.01
1.87	11.30	11.42	11.45	11.52	11.58	11.65	11.71	11.58	11.59	11.57	11.59
1.92	10.56	10.77	10.89	11.06	11.20	11.32	11.53	11.75	11.88	12.16	12.20
1.97	10.65	10.76	10.72	10.71	10.80	10.81	10.91	10.92	10.96	10.99	11.18
2.02	11.10	11.35	11.48	11.52	11.64	11.81	11.90	12.04	12.03	12.04	12.11
2.07	11.27	11.38	11.42	11.44	11.59	11.81	11.83	11.90	12.06	12.17	12.34
2.12	11.18	11.36	11.25	11.27	11.39	11.36	11.36	11.29	11.27	11.35	11.40
2.17	10.90	11.13	11.16	11.19	11.32	11.44	11.52	11.52	11.61	11.65	11.59
2.22	11.65	11.66	11.67	11.69	11.82	11.82	11.83	11.86	11.99	12.01	12.09
2.27	12.46	12.59	12.61	12.56	12.57	12.50	12.55	12.54	12.44	12.42	12.44
2.32	11.59	11.65	11.67	11.73	11.84	11.91	11.98	12.00	12.11	12.17	12.26
2.37	11.96	12.07	12.01	12.02	12.02	12.01	11.97	12.03	11.96	11.98	12.07
2.42	12.52	12.54	12.58	12.47	12.52	12.49	12.55	12.47	12.38	12.40	12.31
2.47	12.61	12.58	12.62	12.64	12.67	12.79	12.85	12.84	12.88	12.96	12.97
2.52	12.67	12.72	12.51	12.49	12.54	12.44	12.36	12.40	12.29	12.23	12.28

2.57	12.98	13.09	13.02	13.03	13.16	13.15	13.15	13.17	13.14	13.18	13.15
2.62	13.08	13.08	13.01	12.95	12.97	13.03	12.94	12.86	12.92	12.96	13.03
2.67	12.81	12.87	12.78	12.76	12.69	12.77	12.65	12.65	12.59	12.50	12.57
2.72	13.14	13.20	13.11	13.12	13.18	13.15	13.13	13.15	13.13	13.13	13.15
2.77	13.10	13.07	13.07	12.96	12.90	12.90	12.90	12.89	12.85	12.87	12.92
2.82	13.29	13.32	13.23	13.13	13.14	13.07	13.06	12.95	12.87	12.88	12.86
2.87	13.09	13.13	13.08	13.04	13.07	13.03	13.00	13.02	13.00	13.03	13.06
2.92	13.73	13.69	13.64	13.60	13.51	13.43	13.41	13.32	13.29	13.24	13.12
2.97	13.52	13.56	13.50	13.48	13.40	13.37	13.33	13.29	13.31	13.23	13.33
3.02	13.98	13.92	13.94	13.83	13.83	13.70	13.75	13.72	13.62	13.62	13.63
3.07	13.55	13.54	13.47	13.33	13.34	13.28	13.20	13.05	13.11	13.01	12.92
3.12	13.77	13.78	13.73	13.64	13.61	13.58	13.52	13.46	13.43	13.36	13.33
3.17	13.79	13.77	13.74	13.62	13.63	13.50	13.56	13.38	13.34	13.36	13.33
3.22	14.57	14.58	14.47	14.37	14.24	14.27	14.18	14.06	13.98	13.93	13.86
3.42	14.71	14.71	14.51	14.47	14.40	14.31	14.25	14.16	14.12	14.04	14.14
3.62	15.29	15.27	15.15	15.03	14.94	14.90	14.79	14.69	14.62	14.48	14.33
3.82	15.10	14.99	14.90	14.86	14.67	14.60	14.52	14.40	14.40	14.31	14.26
4.02	15.58	15.53	15.43	15.37	15.24	15.23	15.05	14.94	14.81	14.71	14.72
4.22	15.33	15.22	15.11	14.97	14.94	14.84	14.75	14.59	14.48	14.44	14.39
4.42	15.75	15.69	15.43	15.36	15.33	15.20	15.06	14.97	14.82	14.63	14.61
4.62	15.91	15.89	15.79	15.64	15.59	15.44	15.37	15.23	15.17	15.02	14.97
4.82	15.96	15.87	15.82	15.71	15.52	15.47	15.31	15.14	15.05	14.99	14.84
5.02	16.27	16.23	16.11	15.87	15.83	15.74	15.62	15.46	15.36	15.29	15.15
5.22	16.42	16.43	16.27	16.20	16.06	15.95	15.83	15.73	15.61	15.39	15.29
5.42	16.59	16.55	16.36	16.27	16.19	16.02	15.83	15.78	15.60	15.50	15.41
5.62	16.79	16.63	16.54	16.34	16.24	16.16	16.05	15.95	15.78	15.61	15.53
5.82	16.73	16.66	16.49	16.34	16.25	16.15	16.06	15.86	15.78	15.68	15.47
6.02	16.85	16.80	16.67	16.50	16.43	16.39	16.05	15.97	15.88	15.67	15.60
6.22	16.89	16.75	16.51	16.42	16.27	16.17	16.00	15.86	15.76	15.61	15.51
6.42	16.97	16.95	16.74	16.67	16.54	16.30	16.15	16.04	15.81	15.81	15.64
6.62	17.43	17.35	17.20	17.04	16.98	16.80	16.65	16.56	16.39	16.24	16.10
6.82	17.21	17.10	16.92	16.82	16.61	16.48	16.37	16.27	16.01	15.95	15.79
7.02	17.65	17.59	17.35	17.28	17.09	16.99	16.85	16.71	16.57	16.42	16.28
7.22	17.44	17.33	17.17	16.96	16.89	16.70	16.63	16.44	16.28	16.12	16.01
7.42	17.73	17.68	17.55	17.35	17.21	17.06	16.90	16.73	16.63	16.48	16.29
7.62	17.64	17.52	17.34	17.25	17.03	16.92	16.80	16.60	16.46	16.32	16.18
7.82	17.70	17.58	17.37	17.30	17.17	17.00	16.79	16.76	16.48	16.26	16.14
8.02	17.54	17.50	17.25	17.17	16.95	16.85	16.62	16.52	16.29	16.19	15.99
8.22	17.67	17.53	17.45	17.23	17.12	17.00	16.83	16.67	16.52	16.36	16.23
8.42	17.38	17.32	17.12	16.90	16.75	16.66	16.48	16.37	16.17	15.96	15.86
8.62	17.76	17.70	17.47	17.35	17.18	17.11	16.91	16.83	16.63	16.38	16.24
8.82	18.04	17.90	17.77	17.49	17.43	17.27	17.13	16.89	16.77	16.65	16.43
9.02	17.66	17.61	17.34	17.16	17.09	16.87	16.76	16.55	16.37	16.19	16.07
9.22	17.79	17.73	17.57	17.37	17.23	17.06	16.93	16.72	16.58	16.39	16.27
9.42	17.97	17.95	17.78	17.65	17.42	17.33	17.14	16.99	16.83	16.62	16.47
9.62	17.82	17.85	17.59	17.37	17.27	17.16	16.96	16.73	16.55	16.41	16.19
9.82	18.05	17.97	17.77	17.67	17.49	17.29	17.17	16.98	16.79	16.64	16.50
10.02	17.91	17.84	17.59	17.47	17.32	17.16	17.01	16.81	16.55	16.44	16.32
10.22	18.18	18.04	17.92	17.68	17.61	17.40	17.28	17.08	16.86	16.73	16.53
10.42	18.15	18.08	17.95	17.77	17.61	17.46	17.32	17.09	16.95	16.77	16.58

10.62	18.21	18.13	17.91	17.74	17.66	17.38	17.24	17.09	16.93	16.77	16.60
10.82	18.13	17.90	17.80	17.49	17.45	17.18	17.12	16.89	16.70	16.52	16.39
11.02	18.09	18.04	17.85	17.65	17.55	17.38	17.18	16.97	16.80	16.63	16.46
11.22	17.97	17.83	17.63	17.45	17.28	17.15	17.00	16.80	16.58	16.44	16.23
11.42	17.74	17.60	17.46	17.24	17.13	16.93	16.84	16.49	16.30	16.17	16.02
11.62	18.04	18.05	17.90	17.68	17.58	17.38	17.20	16.96	16.87	16.65	16.53
11.82	18.04	17.96	17.81	17.60	17.44	17.34	17.15	16.94	16.73	16.56	16.40
12.02	18.19	18.04	17.86	17.66	17.57	17.36	17.22	17.01	16.85	16.63	16.49
12.22	18.41	18.30	18.09	17.90	17.79	17.54	17.40	17.22	17.01	16.82	16.71
12.42	17.98	17.83	17.59	17.46	17.25	17.14	16.97	16.73	16.62	16.40	16.26
12.62	18.09	18.00	17.85	17.67	17.53	17.31	17.09	16.99	16.74	16.54	16.44
12.82	18.11	17.98	17.90	17.67	17.59	17.44	17.17	16.99	16.76	16.60	16.47
13.02	18.47	18.32	18.08	17.99	17.85	17.63	17.41	17.29	17.00	16.79	16.61
13.22	18.24	18.14	18.01	17.78	17.69	17.44	17.34	17.17	16.98	16.76	16.57
13.42	18.24	18.17	17.95	17.76	17.57	17.49	17.30	17.06	16.92	16.70	16.56
13.50	18.12	18.07	17.89	17.74	17.48	17.38	17.21	16.92	16.80	16.53	16.37
13.59	18.48	18.37	18.15	17.99	17.86	17.69	17.45	17.25	17.07	16.83	16.69
13.68	18.30	18.09	18.00	17.81	17.65	17.46	17.33	17.11	16.93	16.71	16.59
13.78	17.96	17.82	17.61	17.44	17.27	17.19	16.98	16.80	16.56	16.39	16.20
13.88	18.22	18.14	17.97	17.72	17.55	17.41	17.33	17.03	16.85	16.72	16.45
14.00	18.49	18.45	18.20	17.99	17.89	17.67	17.53	17.32	17.10	16.89	16.80
14.12	18.44	18.27	18.07	17.98	17.78	17.44	17.32	17.11	16.97	16.77	16.56
14.25	18.25	18.16	17.93	17.65	17.60	17.46	17.18	17.08	16.84	16.66	16.49
14.40	18.76	18.56	18.44	18.19	18.11	17.99	17.66	17.53	17.30	17.12	16.97
14.55	18.20	18.11	17.89	17.67	17.51	17.38	17.20	17.02	16.81	16.60	16.39
14.72	18.35	18.22	18.05	17.88	17.65	17.47	17.37	17.11	16.94	16.77	16.51
14.90	18.24	18.05	17.88	17.74	17.57	17.41	17.21	16.98	16.82	16.61	16.41
15.09	18.20	18.02	17.92	17.66	17.46	17.22	17.09	16.88	16.70	16.50	16.32
15.30	18.42	18.30	18.15	17.97	17.80	17.64	17.47	17.25	17.03	16.83	16.66
15.53	18.72	18.68	18.44	18.26	18.13	17.96	17.72	17.52	17.35	17.10	16.88
15.77	18.48	18.34	18.18	18.01	17.86	17.65	17.48	17.23	17.10	16.91	16.66
16.04	18.70	18.54	18.38	18.16	18.04	17.80	17.61	17.36	17.22	17.01	16.88
16.32	18.56	18.45	18.30	18.09	17.89	17.75	17.64	17.41	17.11	16.86	16.76
16.63	18.80	18.69	18.49	18.30	18.17	17.96	17.81	17.60	17.40	17.11	16.96
16.96	18.99	18.91	18.67	18.38	18.31	18.09	17.97	17.73	17.51	17.38	17.14
17.31	18.67	18.58	18.34	18.14	17.95	17.78	17.60	17.41	17.18	17.01	16.77
17.70	19.15	19.07	18.86	18.66	18.48	18.37	18.17	17.91	17.78	17.54	17.33
18.11	18.39	18.31	18.07	17.83	17.63	17.49	17.27	17.15	16.90	16.71	16.53
18.56	18.66	18.60	18.42	18.16	18.12	17.87	17.71	17.49	17.29	17.03	16.79
19.04	18.72	18.64	18.41	18.21	18.00	17.85	17.70	17.45	17.17	17.03	16.94
19.56	19.08	19.03	18.81	18.64	18.49	18.25	18.08	17.82	17.63	17.42	17.18
20.12	19.02	18.93	18.64	18.46	18.33	18.13	17.95	17.81	17.52	17.34	17.12
20.72	19.16	19.04	18.91	18.68	18.51	18.35	18.15	17.88	17.74	17.51	17.33
21.37	19.18	19.06	18.81	18.68	18.62	18.29	18.14	17.95	17.71	17.45	17.29
22.08	19.20	19.01	18.80	18.70	18.49	18.32	18.18	17.87	17.67	17.54	17.30
22.84	19.46	19.36	19.08	18.88	18.88	18.61	18.36	18.24	18.06	17.76	17.61
23.65	18.83	18.80	18.62	18.34	18.24	18.11	17.92	17.59	17.41	17.29	17.04
24.54	19.48	19.31	19.07	18.88	18.72	18.60	18.35	18.06	17.97	17.63	17.49
25.49	19.71	19.51	19.30	19.17	18.99	18.80	18.54	18.35	18.17	17.89	17.69
26.51	19.00	19.01	18.76	18.61	18.36	18.20	18.00	17.72	17.61	17.47	17.17

27.62	19.82	19.75	19.55	19.28	19.13	18.96	18.70	18.49	18.20	18.04	17.83
28.81	19.81	19.67	19.46	19.35	19.17	19.04	18.78	18.53	18.31	18.02	17.80
30.10	19.40	19.27	19.02	18.87	18.70	18.45	18.33	18.11	17.85	17.68	17.46
31.49	20.04	19.97	19.77	19.54	19.43	19.20	19.00	18.70	18.58	18.33	18.06
32.98	19.85	19.77	19.52	19.33	19.11	18.97	18.72	18.63	18.30	18.14	17.89
34.60	20.31	20.21	19.95	19.76	19.60	19.47	19.23	18.98	18.81	18.62	18.25
36.34	20.07	20.04	19.77	19.59	19.42	19.24	19.04	18.75	18.54	18.39	18.20
38.21	20.82	20.59	20.48	20.31	20.11	19.91	19.74	19.48	19.21	18.95	18.88
40.24	20.51	20.35	20.17	20.03	19.85	19.66	19.38	19.12	18.91	18.68	18.50
42.42	20.24	20.12	19.94	19.75	19.60	19.39	19.19	18.86	18.71	18.48	18.27
44.78	20.48	20.31	20.10	19.99	19.70	19.50	19.32	19.14	18.97	18.67	18.53
47.32	20.42	20.30	20.05	19.86	19.73	19.54	19.33	19.13	18.82	18.66	18.43
50.06	20.79	20.67	20.54	20.27	20.15	19.95	19.71	19.54	19.27	19.00	18.81
53.01	20.89	20.79	20.58	20.48	20.23	20.03	19.84	19.61	19.34	19.15	18.98
56.20	21.21	21.02	20.87	20.60	20.48	20.31	20.15	19.83	19.61	19.43	19.15
59.64	21.56	21.56	21.37	21.15	21.00	20.82	20.53	20.39	20.14	19.89	19.62
63.34	20.92	20.85	20.77	20.49	20.34	20.14	19.96	19.71	19.54	19.19	19.07
67.34	21.42	21.34	21.16	20.95	20.82	20.64	20.42	20.24	19.95	19.70	19.50
71.65	22.00	21.80	21.70	21.52	21.31	21.15	20.99	20.70	20.47	20.21	20.05
76.30	21.95	21.84	21.68	21.57	21.33	21.18	20.97	20.71	20.43	20.23	19.96
81.32	22.48	22.44	22.24	22.07	21.86	21.65	21.51	21.19	21.07	20.84	20.57
86.73	22.75	22.63	22.44	22.35	22.15	21.94	21.80	21.55	21.31	21.04	20.82
92.56	22.90	22.91	22.78	22.59	22.38	22.23	22.03	21.83	21.53	21.22	21.06
98.85	22.71	22.60	22.41	22.31	22.12	21.92	21.69	21.51	21.23	21.03	20.80
105.64	23.37	23.29	23.19	23.05	22.96	22.76	22.48	22.28	22.04	21.71	21.55
112.96	23.76	23.67	23.49	23.32	23.18	23.01	22.79	22.55	22.27	22.05	21.81
120.85	23.85	23.80	23.58	23.42	23.29	22.98	22.84	22.62	22.35	22.16	21.87
129.36	24.28	24.26	24.13	24.05	23.81	23.65	23.42	23.11	22.81	22.62	22.45
138.55	24.52	24.51	24.28	24.10	23.97	23.75	23.55	23.45	23.04	22.89	22.58
148.45	24.46	24.40	24.24	24.05	23.84	23.70	23.45	23.24	22.98	22.82	22.52
159.13	24.81	24.81	24.63	24.44	24.30	24.11	23.90	23.69	23.50	23.21	22.93
170.65	25.21	25.09	24.98	24.85	24.62	24.57	24.31	24.09	23.81	23.58	23.24
183.07	25.67	25.75	25.54	25.43	25.26	25.03	24.85	24.58	24.33	24.08	23.85
196.47	25.79	25.77	25.65	25.46	25.36	25.18	24.94	24.67	24.46	24.22	23.93
210.91	26.30	26.22	26.20	25.99	25.85	25.64	25.51	25.18	24.93	24.68	24.37
226.50	26.57	26.52	26.39	26.21	26.08	25.93	25.70	25.46	25.16	24.89	24.70
243.31	26.79	26.75	26.55	26.49	26.26	26.13	25.89	25.60	25.39	25.08	24.91
261.43	27.50	27.50	27.31	27.18	27.07	26.85	26.59	26.37	26.20	25.78	25.55
280.98	27.54	27.53	27.42	27.30	27.07	26.97	26.69	26.37	26.17	25.91	25.64
302.07	27.84	27.74	27.60	27.56	27.37	27.13	26.95	26.72	26.36	26.11	25.83
324.81	28.28	28.27	28.18	28.03	27.89	27.68	27.46	27.24	26.98	26.65	26.42
349.33	28.57	28.54	28.35	28.24	28.06	27.88	27.69	27.40	27.13	26.83	26.60
375.79	28.98	28.93	28.78	28.71	28.48	28.33	28.13	27.83	27.56	27.32	27.00
404.31	28.93	28.89	28.79	28.58	28.45	28.34	28.06	27.89	27.66	27.27	27.02
435.08	29.57	29.51	29.37	29.25	29.12	28.96	28.73	28.49	28.12	27.89	27.58
468.27	29.73	29.71	29.55	29.34	29.29	29.02	28.84	28.60	28.24	27.97	27.72
504.06	30.18	30.20	30.02	29.88	29.73	29.52	29.30	29.00	28.78	28.47	28.15
542.66	30.25	30.19	30.08	30.00	29.79	29.63	29.41	29.11	28.86	28.57	28.28
584.29	30.58	30.46	30.41	30.28	30.10	29.88	29.72	29.44	29.17	28.82	28.57
629.18	30.80	30.80	30.63	30.48	30.32	30.14	29.89	29.67	29.33	29.10	28.77

677.61	31.24	31.27	31.14	31.00	30.92	30.66	30.43	30.17	29.89	29.63	29.32
729.83	31.27	31.16	31.10	30.99	30.81	30.60	30.39	30.12	29.77	29.52	29.23
786.16	31.67	31.54	31.46	31.31	31.13	30.88	30.77	30.45	30.20	29.89	29.61
846.90	31.59	31.58	31.48	31.27	31.12	30.98	30.75	30.51	30.13	29.85	29.53
912.42	31.87	31.84	31.73	31.50	31.37	31.15	30.93	30.68	30.43	30.06	29.77
983.08	32.02	31.90	31.82	31.70	31.51	31.29	31.12	30.80	30.53	30.21	29.90
1059.29	32.18	32.18	32.01	31.87	31.75	31.54	31.27	30.96	30.63	30.45	30.12
1141.48	32.03	32.03	31.87	31.80	31.62	31.37	31.20	30.91	30.55	30.32	29.96
1230.13	32.01	31.93	31.85	31.71	31.56	31.29	31.09	30.84	30.43	30.17	29.83
1325.73	32.16	32.13	32.05	31.91	31.70	31.51	31.29	31.06	30.71	30.40	30.14
1428.84	32.14	32.05	31.94	31.85	31.57	31.43	31.23	30.86	30.60	30.26	29.99
1540.05	32.34	32.29	32.15	31.98	31.83	31.70	31.29	31.15	30.77	30.54	30.21
1659.99	32.26	32.20	32.12	31.95	31.75	31.60	31.37	31.07	30.78	30.41	30.14
1789.34	32.06	32.03	31.96	31.82	31.67	31.39	31.15	30.94	30.60	30.20	29.98
1928.85	32.24	32.15	32.05	31.90	31.72	31.55	31.25	31.10	30.65	30.35	30.00
2079.31	32.17	32.09	31.94	31.82	31.70	31.45	31.22	30.95	30.55	30.29	29.97
2241.59	32.01	32.03	31.79	31.74	31.58	31.35	31.02	30.75	30.44	30.09	29.92
2416.60	31.96	31.88	31.82	31.63	31.47	31.16	30.97	30.72	30.42	30.15	29.70
2605.36	31.86	31.81	31.75	31.60	31.38	31.18	30.86	30.62	30.29	30.04	29.70
2808.94	31.69	31.61	31.50	31.30	31.12	30.88	30.69	30.40	30.05	29.73	29.45
3028.50	31.50	31.46	31.30	31.10	30.94	30.81	30.44	30.20	29.84	29.63	29.26
3265.30	31.46	31.36	31.26	31.06	30.97	30.70	30.48	30.13	29.74	29.51	29.16
3520.69	31.13	31.05	30.98	30.76	30.61	30.34	30.05	29.72	29.43	29.20	28.86
3796.13	30.82	30.88	30.73	30.57	30.38	30.16	29.91	29.58	29.23	28.98	28.62

Wavelength (nm) / Time (ps)	600.39	601.81	603.24	604.66	606.08	607.51	608.93	610.36	611.78	613.20	614.63
-3.78	0.01	0.01	0.02	-0.06	0.03	-0.05	-0.06	-0.04	-0.05	0.03	-0.02
-3.28	0.00	-0.01	-0.01	0.03	-0.03	-0.05	-0.06	-0.03	0.00	-0.05	-0.06
-2.78	0.04	-0.02	0.02	-0.04	0.00	0.04	-0.02	-0.02	0.01	-0.03	0.03
-2.28	-0.01	0.03	-0.02	-0.04	0.03	0.02	0.02	0.06	0.03	-0.03	-0.03
-1.78	-0.01	0.03	-0.02	0.03	0.01	0.04	0.07	-0.02	-0.04	-0.03	-0.04
-1.28	-0.02	-0.06	0.03	0.04	-0.02	-0.01	0.00	0.02	0.06	0.03	0.09
-0.78	-0.01	0.04	0.04	0.03	0.00	0.05	-0.01	0.04	0.01	0.05	0.04
-0.28	0.01	-0.01	-0.06	0.00	-0.01	-0.04	0.05	-0.02	-0.02	0.03	-0.01
-0.18	0.01	0.04	-0.04	0.04	-0.03	-0.01	-0.06	0.06	0.02	0.06	0.07
-0.08	-0.09	-0.01	-0.03	-0.06	-0.03	0.01	0.03	0.01	-0.04	0.06	0.01
0.02	0.06	0.07	0.08	0.03	-0.01	0.10	0.04	0.13	0.10	0.10	-0.01
0.12	-0.01	0.01	0.05	0.01	-0.01	0.06	0.02	0.09	0.00	0.05	0.03
0.22	-0.04	0.09	-0.01	0.02	0.01	0.06	0.01	0.07	0.07	0.03	-0.01
0.27	0.00	0.08	0.06	0.01	0.04	-0.04	0.01	0.11	-0.06	-0.01	0.02
0.32	-0.02	0.01	0.07	0.04	0.07	0.04	0.04	-0.01	0.00	-0.02	0.00
0.37	-0.05	0.07	0.02	-0.04	0.01	0.00	-0.04	0.02	0.01	-0.02	-0.01
0.42	0.02	0.06	-0.06	0.00	-0.06	-0.06	-0.05	-0.01	-0.10	-0.08	-0.06
0.47	-0.11	0.06	0.00	0.03	0.00	0.01	0.00	0.04	0.08	0.05	-0.07
0.52	-0.09	0.01	-0.01	-0.04	0.04	0.01	0.02	0.02	0.01	0.00	-0.02
0.57	-0.01	0.00	0.01	-0.02	0.09	0.07	-0.06	0.03	-0.06	-0.02	-0.01
0.62	-0.06	0.03	0.03	-0.01	0.01	0.00	-0.01	0.07	0.03	-0.04	-0.04

0.67	-0.06	-0.03	0.00	-0.07	-0.03	0.04	-0.04	-0.03	-0.06	-0.08	-0.02
0.72	-0.02	0.00	0.04	-0.04	-0.07	-0.08	-0.06	-0.04	-0.03	-0.04	0.01
0.77	-0.06	-0.03	-0.07	-0.02	0.03	0.02	-0.01	-0.03	0.03	0.02	0.05
0.82	-0.05	-0.02	-0.04	0.06	0.01	-0.03	-0.02	0.07	0.06	-0.03	0.03
0.87	0.00	0.08	0.01	0.01	0.02	0.02	-0.03	0.05	0.00	-0.01	0.01
0.92	-0.06	0.05	0.03	-0.04	0.04	0.03	-0.08	0.03	0.00	0.07	0.07
0.97	-0.02	-0.01	0.03	-0.02	0.01	0.10	0.00	0.03	0.00	0.02	-0.01
1.02	-0.01	0.09	0.02	0.06	0.01	-0.01	0.08	0.02	0.05	0.05	0.04
1.07	-0.05	0.04	0.06	0.00	0.02	-0.01	-0.02	-0.05	0.03	0.05	0.01
1.12	-0.03	0.08	0.01	0.02	0.04	-0.02	0.03	0.11	0.04	0.01	0.06
1.17	0.07	0.07	-0.04	-0.08	-0.01	0.02	-0.05	0.06	0.08	-0.02	-0.01
1.22	-0.07	0.05	0.10	-0.01	-0.08	-0.04	0.09	0.15	0.01	-0.10	-0.08
1.27	-0.10	-0.16	-0.28	-0.24	-0.14	-0.05	0.03	-0.01	0.01	-0.10	0.00
1.32	-9.55	-5.75	-2.43	0.45	2.65	4.12	4.77	4.78	4.04	2.86	1.50
1.37	7.16	1.98	-3.96	-7.55	-10.61	-12.33	-13.73	-13.71	-14.10	-14.01	-14.83
1.42	7.06	13.19	18.84	25.73	32.47	40.18	47.81	56.37	64.34	72.68	79.63
1.47	12.53	12.33	12.13	12.11	12.31	12.58	13.00	13.52	13.84	14.20	14.41
1.52	13.57	14.06	14.29	14.69	15.07	15.35	15.66	16.02	16.20	16.51	16.77
1.57	11.41	11.90	12.12	12.60	13.05	13.57	14.06	14.55	14.98	15.45	15.66
1.62	11.85	11.90	11.78	11.78	11.71	11.90	12.05	12.17	12.36	12.73	13.11
1.67	11.61	11.85	12.07	12.36	12.50	12.67	12.78	12.92	12.98	13.07	13.13
1.72	10.44	10.67	10.81	11.07	11.34	11.70	12.05	12.45	12.80	13.16	13.53
1.77	11.60	11.81	11.82	11.95	12.00	12.10	12.16	12.30	12.34	12.46	12.53
1.82	11.18	11.46	11.74	12.01	12.22	12.46	12.67	12.93	12.99	13.11	13.28
1.87	11.52	11.63	11.65	11.80	11.96	12.16	12.47	12.83	13.08	13.31	13.60
1.92	12.35	12.48	12.64	12.72	12.77	12.89	12.90	12.90	12.95	12.92	12.94
1.97	11.26	11.50	11.66	11.90	12.29	12.55	12.81	13.18	13.39	13.53	13.84
2.02	12.06	12.11	12.07	12.18	12.25	12.24	12.33	12.58	12.65	12.76	13.02
2.07	12.45	12.63	12.68	12.82	12.88	12.97	12.99	13.05	13.10	13.10	13.15
2.12	11.34	11.41	11.49	11.61	11.74	12.05	12.28	12.53	12.71	12.92	13.11
2.17	11.63	11.72	11.75	11.72	11.76	11.77	11.85	12.03	12.07	12.21	12.40
2.22	12.20	12.50	12.62	12.71	12.93	13.04	13.24	13.38	13.51	13.58	13.66
2.27	12.28	12.43	12.44	12.56	12.65	12.79	12.92	13.21	13.36	13.67	13.89
2.32	12.29	12.40	12.40	12.48	12.50	12.61	12.65	12.75	12.74	12.82	12.88
2.37	12.20	12.28	12.33	12.55	12.65	12.94	13.08	13.31	13.40	13.53	13.70
2.42	12.22	12.25	12.30	12.22	12.39	12.50	12.59	12.80	12.92	13.18	13.27
2.47	12.96	13.18	13.19	13.24	13.33	13.37	13.48	13.60	13.63	13.63	13.68
2.52	12.30	12.31	12.41	12.57	12.64	12.81	12.97	13.24	13.42	13.48	13.64
2.57	13.05	13.14	13.12	13.13	13.18	13.30	13.21	13.44	13.49	13.64	13.71
2.62	13.11	13.20	13.25	13.41	13.46	13.51	13.66	13.84	13.80	13.86	13.91
2.67	12.50	12.63	12.62	12.71	12.82	13.01	13.15	13.30	13.47	13.61	13.74
2.72	13.14	13.22	13.22	13.22	13.21	13.23	13.28	13.36	13.42	13.44	13.54
2.77	12.83	13.03	13.05	13.21	13.30	13.42	13.51	13.71	13.83	13.90	14.01
2.82	12.69	12.71	12.63	12.71	12.74	12.76	12.94	13.12	13.17	13.34	13.47
2.87	13.02	13.03	13.07	13.14	13.25	13.34	13.36	13.52	13.54	13.59	13.70
2.92	13.09	13.11	13.16	13.26	13.31	13.52	13.59	13.82	13.91	14.04	14.16
2.97	13.22	13.22	13.23	13.15	13.27	13.29	13.31	13.48	13.55	13.54	13.72
3.02	13.65	13.74	13.69	13.82	13.86	13.92	13.97	14.05	14.08	14.12	14.21
3.07	12.86	12.98	12.93	12.98	13.14	13.19	13.30	13.51	13.59	13.69	13.77
3.12	13.13	13.22	13.23	13.18	13.27	13.25	13.29	13.37	13.36	13.47	13.53

3.17	13.38	13.37	13.41	13.44	13.52	13.64	13.71	13.85	13.95	13.97	14.06
3.22	13.74	13.77	13.65	13.67	13.68	13.72	13.84	13.90	13.94	14.13	14.16
3.42	14.00	14.08	14.03	14.11	14.17	14.20	14.20	14.35	14.41	14.43	14.42
3.62	14.33	14.40	14.31	14.28	14.41	14.45	14.53	14.65	14.72	14.78	14.90
3.82	14.14	14.26	14.15	14.13	14.13	14.24	14.22	14.36	14.40	14.43	14.47
4.02	14.61	14.61	14.55	14.57	14.56	14.62	14.68	14.82	14.88	14.92	15.00
4.22	14.35	14.34	14.27	14.28	14.29	14.30	14.31	14.44	14.36	14.55	14.46
4.42	14.52	14.49	14.43	14.39	14.45	14.45	14.53	14.58	14.60	14.62	14.70
4.62	14.89	14.85	14.73	14.78	14.82	14.76	14.81	14.89	14.89	14.93	15.02
4.82	14.70	14.75	14.66	14.69	14.67	14.59	14.68	14.71	14.73	14.80	14.91
5.02	14.98	15.01	14.87	14.88	14.81	14.82	14.80	14.92	14.94	15.03	15.06
5.22	15.23	15.20	15.10	15.06	15.05	15.10	15.06	15.12	15.19	15.16	15.16
5.42	15.36	15.30	15.16	15.11	15.14	15.09	15.07	15.19	15.18	15.21	15.22
5.62	15.33	15.35	15.21	15.21	15.22	15.23	15.13	15.28	15.29	15.26	15.31
5.82	15.35	15.37	15.24	15.22	15.25	15.17	15.14	15.24	15.22	15.24	15.29
6.02	15.49	15.37	15.28	15.31	15.16	15.23	15.22	15.24	15.28	15.29	15.32
6.22	15.32	15.32	15.20	15.08	15.12	15.09	15.01	15.09	15.10	15.12	15.14
6.42	15.47	15.52	15.40	15.34	15.31	15.29	15.24	15.29	15.31	15.36	15.40
6.62	16.05	15.90	15.84	15.72	15.75	15.69	15.65	15.73	15.73	15.66	15.73
6.82	15.69	15.58	15.39	15.39	15.35	15.36	15.33	15.47	15.44	15.39	15.40
7.02	16.16	16.09	15.99	15.91	15.80	15.81	15.85	15.89	15.80	15.81	15.89
7.22	15.84	15.71	15.71	15.56	15.54	15.54	15.42	15.53	15.51	15.51	15.43
7.42	16.18	16.12	15.94	15.91	15.81	15.84	15.80	15.98	15.81	15.84	15.86
7.62	16.07	15.97	15.78	15.72	15.69	15.67	15.64	15.67	15.71	15.69	15.72
7.82	15.97	16.04	15.83	15.74	15.72	15.74	15.72	15.68	15.64	15.71	15.68
8.02	15.84	15.82	15.62	15.57	15.50	15.41	15.44	15.53	15.44	15.45	15.44
8.22	16.05	15.98	15.85	15.74	15.65	15.68	15.67	15.68	15.68	15.59	15.67
8.42	15.66	15.59	15.47	15.39	15.26	15.24	15.20	15.30	15.26	15.25	15.24
8.62	16.12	16.01	15.88	15.80	15.74	15.83	15.74	15.70	15.69	15.72	15.72
8.82	16.20	16.18	16.08	15.94	15.93	15.96	15.84	15.91	15.78	15.81	15.78
9.02	15.95	15.89	15.64	15.60	15.53	15.48	15.50	15.53	15.45	15.49	15.39
9.22	16.03	16.01	15.80	15.72	15.70	15.66	15.62	15.59	15.59	15.52	15.65
9.42	16.27	16.22	16.06	16.01	15.94	15.88	15.83	15.89	15.87	15.84	15.89
9.62	16.08	16.05	15.85	15.77	15.73	15.55	15.58	15.55	15.49	15.49	15.52
9.82	16.33	16.26	16.07	15.98	15.94	15.86	15.80	15.84	15.74	15.78	15.78
10.02	16.10	16.09	15.91	15.85	15.70	15.67	15.64	15.70	15.66	15.73	15.65
10.22	16.27	16.34	16.10	16.08	15.97	15.94	15.86	15.92	15.93	15.86	15.82
10.42	16.41	16.35	16.15	16.10	16.01	15.97	15.88	15.82	15.89	15.85	15.79
10.62	16.33	16.28	16.10	16.04	15.90	15.90	15.81	15.87	15.82	15.77	15.77
10.82	16.15	16.14	15.93	15.87	15.75	15.71	15.61	15.63	15.54	15.56	15.52
11.02	16.27	16.17	16.06	15.95	15.78	15.78	15.78	15.75	15.68	15.69	15.70
11.22	16.06	16.00	15.79	15.83	15.62	15.60	15.53	15.54	15.54	15.54	15.53
11.42	15.85	15.69	15.56	15.47	15.36	15.29	15.33	15.26	15.19	15.16	15.20
11.62	16.27	16.19	16.07	15.95	15.86	15.82	15.80	15.70	15.68	15.61	15.63
11.82	16.23	16.06	15.88	15.81	15.77	15.70	15.59	15.60	15.62	15.57	15.59
12.02	16.33	16.26	16.02	15.92	15.83	15.75	15.72	15.69	15.61	15.65	15.56
12.22	16.50	16.40	16.18	16.08	16.06	15.92	15.91	15.98	15.88	15.83	15.85
12.42	16.06	15.92	15.71	15.67	15.60	15.50	15.45	15.49	15.41	15.38	15.39
12.62	16.23	16.09	15.88	15.80	15.70	15.71	15.60	15.63	15.55	15.52	15.52
12.82	16.34	16.21	16.03	15.91	15.79	15.78	15.72	15.70	15.65	15.62	15.63

13.02	16.54	16.37	16.19	16.07	15.91	15.87	15.86	15.83	15.72	15.67	15.69
13.22	16.41	16.34	16.09	15.95	15.95	15.77	15.76	15.81	15.69	15.62	15.71
13.42	16.32	16.28	16.10	16.00	15.80	15.75	15.69	15.77	15.61	15.63	15.55
13.50	16.25	16.09	15.87	15.85	15.70	15.76	15.53	15.56	15.56	15.50	15.49
13.59	16.43	16.35	16.19	16.08	16.02	15.97	15.90	15.82	15.81	15.73	15.75
13.68	16.32	16.23	16.06	15.88	15.89	15.79	15.73	15.77	15.67	15.57	15.64
13.78	15.97	15.92	15.66	15.54	15.42	15.39	15.33	15.38	15.23	15.22	15.18
13.88	16.26	16.15	15.93	15.95	15.83	15.76	15.59	15.72	15.54	15.53	15.53
14.00	16.57	16.36	16.21	16.13	16.04	16.06	15.87	15.84	15.86	15.84	15.79
14.12	16.42	16.26	16.10	16.00	15.93	15.85	15.64	15.74	15.75	15.68	15.53
14.25	16.25	16.15	15.96	15.87	15.74	15.71	15.68	15.64	15.53	15.50	15.49
14.40	16.71	16.60	16.47	16.36	16.21	16.14	16.15	16.04	16.01	15.97	15.96
14.55	16.25	16.09	15.93	15.82	15.71	15.60	15.59	15.59	15.54	15.49	15.45
14.72	16.31	16.25	16.07	15.93	15.87	15.77	15.70	15.76	15.64	15.58	15.61
14.90	16.18	16.12	15.98	15.80	15.74	15.63	15.60	15.56	15.52	15.41	15.43
15.09	16.13	16.01	15.81	15.68	15.56	15.63	15.49	15.45	15.40	15.36	15.33
15.30	16.48	16.32	16.14	16.05	15.87	15.84	15.80	15.81	15.69	15.76	15.71
15.53	16.68	16.60	16.37	16.29	16.18	16.19	16.00	16.02	16.04	15.98	15.87
15.77	16.43	16.39	16.19	16.06	15.96	15.95	15.85	15.77	15.76	15.76	15.64
16.04	16.67	16.48	16.32	16.22	16.12	16.03	15.95	15.91	15.83	15.83	15.69
16.32	16.63	16.44	16.18	16.08	15.96	15.91	15.83	15.80	15.72	15.73	15.74
16.63	16.76	16.67	16.46	16.31	16.21	16.05	16.04	16.04	15.92	15.90	15.91
16.96	16.97	16.83	16.64	16.50	16.48	16.46	16.25	16.28	16.20	16.15	16.13
17.31	16.63	16.48	16.32	16.18	15.95	16.01	15.85	15.88	15.82	15.78	15.69
17.70	17.06	17.03	16.82	16.73	16.56	16.41	16.32	16.35	16.25	16.28	16.26
18.11	16.27	16.18	15.99	15.86	15.70	15.64	15.72	15.62	15.56	15.46	15.45
18.56	16.61	16.53	16.30	16.16	16.03	15.98	15.86	15.85	15.74	15.74	15.64
19.04	16.68	16.55	16.33	16.26	16.14	15.90	15.87	15.87	15.76	15.79	15.80
19.56	17.02	16.87	16.70	16.59	16.40	16.32	16.25	16.21	16.13	16.07	15.98
20.12	16.83	16.79	16.58	16.47	16.34	16.21	16.15	16.16	16.03	16.00	15.94
20.72	16.99	16.97	16.79	16.67	16.53	16.35	16.33	16.30	16.18	16.13	16.19
21.37	17.04	16.95	16.74	16.57	16.53	16.43	16.29	16.29	16.21	16.23	16.12
22.08	17.09	16.95	16.75	16.55	16.45	16.37	16.27	16.24	16.14	15.98	16.03
22.84	17.40	17.28	17.03	16.97	16.81	16.70	16.57	16.60	16.51	16.39	16.39
23.65	16.82	16.65	16.44	16.40	16.22	16.05	15.97	15.94	15.81	15.85	15.74
24.54	17.28	17.09	16.91	16.74	16.67	16.60	16.40	16.47	16.36	16.24	16.26
25.49	17.52	17.38	17.20	17.00	16.91	16.73	16.71	16.62	16.52	16.44	16.40
26.51	16.96	16.84	16.59	16.49	16.30	16.21	16.12	16.13	16.01	15.98	15.87
27.62	17.61	17.48	17.21	17.12	17.03	16.84	16.77	16.76	16.68	16.59	16.54
28.81	17.63	17.54	17.33	17.12	17.06	16.92	16.85	16.73	16.68	16.64	16.52
30.10	17.18	17.06	16.84	16.75	16.52	16.46	16.26	16.29	16.18	16.12	16.10
31.49	17.92	17.74	17.43	17.41	17.26	17.14	16.97	16.88	16.84	16.76	16.70
32.98	17.63	17.50	17.32	17.15	16.92	16.90	16.74	16.70	16.56	16.48	16.40
34.60	18.07	18.02	17.76	17.60	17.43	17.29	17.21	17.17	17.03	16.98	16.90
36.34	17.86	17.81	17.49	17.34	17.18	17.08	16.93	16.92	16.88	16.74	16.66
38.21	18.56	18.46	18.20	18.08	17.93	17.85	17.63	17.58	17.48	17.38	17.34
40.24	18.16	18.05	17.79	17.67	17.52	17.41	17.32	17.26	17.09	17.05	16.93
42.42	18.04	17.94	17.63	17.41	17.34	17.14	17.05	17.10	16.87	16.81	16.74
44.78	18.20	18.09	17.83	17.65	17.55	17.41	17.28	17.24	17.07	17.00	16.95
47.32	18.12	18.00	17.74	17.58	17.44	17.28	17.15	17.19	17.02	16.91	16.84

50.06	18.52	18.35	18.18	17.99	17.87	17.67	17.57	17.49	17.32	17.20	17.22
53.01	18.61	18.47	18.23	18.00	17.90	17.80	17.64	17.55	17.43	17.40	17.26
56.20	18.85	18.74	18.42	18.32	18.19	18.06	17.93	17.78	17.62	17.50	17.43
59.64	19.37	19.28	18.95	18.87	18.67	18.51	18.34	18.37	18.17	18.05	17.97
63.34	18.77	18.58	18.34	18.20	18.03	17.84	17.70	17.65	17.47	17.37	17.28
67.34	19.24	19.02	18.84	18.59	18.40	18.29	18.13	18.04	17.92	17.78	17.65
71.65	19.71	19.58	19.31	19.09	18.98	18.83	18.65	18.56	18.46	18.29	18.27
76.30	19.78	19.55	19.24	19.11	18.98	18.75	18.64	18.55	18.36	18.20	18.08
81.32	20.36	20.17	19.89	19.68	19.44	19.27	19.11	19.09	18.83	18.70	18.64
86.73	20.50	20.32	20.06	19.87	19.69	19.52	19.32	19.29	19.09	18.96	18.85
92.56	20.73	20.58	20.31	20.12	19.93	19.71	19.53	19.44	19.34	19.13	18.98
98.85	20.44	20.28	20.02	19.77	19.67	19.49	19.33	19.17	19.03	18.84	18.67
105.64	21.26	21.01	20.82	20.56	20.35	20.22	20.07	19.95	19.73	19.49	19.33
112.96	21.56	21.36	21.09	20.86	20.64	20.43	20.26	20.16	19.98	19.86	19.69
120.85	21.58	21.38	21.18	20.86	20.75	20.52	20.33	20.17	20.01	19.86	19.66
129.36	22.11	21.92	21.72	21.40	21.17	21.01	20.77	20.69	20.48	20.26	20.07
138.55	22.34	22.16	21.84	21.62	21.42	21.17	20.94	20.84	20.58	20.38	20.27
148.45	22.17	22.04	21.73	21.50	21.23	21.04	20.87	20.73	20.47	20.27	20.08
159.13	22.69	22.41	22.12	21.92	21.70	21.56	21.23	21.10	20.90	20.70	20.52
170.65	22.98	22.74	22.46	22.25	22.03	21.83	21.53	21.45	21.21	20.95	20.84
183.07	23.53	23.31	23.00	22.76	22.53	22.33	22.07	21.94	21.72	21.45	21.30
196.47	23.62	23.43	23.09	22.84	22.59	22.42	22.19	21.95	21.70	21.50	21.38
210.91	24.13	23.83	23.54	23.31	23.06	22.78	22.61	22.45	22.19	21.96	21.73
226.50	24.39	24.08	23.87	23.58	23.23	23.07	22.80	22.64	22.40	22.12	21.90
243.31	24.50	24.33	24.06	23.83	23.50	23.27	23.01	22.79	22.51	22.28	22.02
261.43	25.28	24.99	24.73	24.44	24.17	23.89	23.59	23.51	23.21	22.91	22.78
280.98	25.33	25.07	24.72	24.39	24.18	23.88	23.70	23.44	23.17	22.97	22.73
302.07	25.54	25.32	24.97	24.69	24.45	24.17	23.89	23.71	23.37	23.13	22.85
324.81	26.05	25.92	25.56	25.19	25.00	24.60	24.40	24.19	23.89	23.67	23.34
349.33	26.31	26.02	25.69	25.38	25.04	24.83	24.52	24.39	24.08	23.71	23.49
375.79	26.69	26.51	26.11	25.79	25.54	25.19	24.90	24.68	24.41	24.06	23.83
404.31	26.65	26.39	26.10	25.73	25.48	25.22	24.87	24.63	24.33	24.06	23.73
435.08	27.15	26.89	26.53	26.24	25.91	25.57	25.29	25.06	24.70	24.48	24.18
468.27	27.36	27.07	26.80	26.40	26.19	25.85	25.48	25.31	24.87	24.71	24.35
504.06	27.82	27.51	27.22	26.92	26.51	26.27	25.87	25.69	25.30	25.00	24.80
542.66	27.95	27.66	27.25	26.99	26.68	26.34	26.06	25.77	25.37	25.11	24.82
584.29	28.24	27.90	27.52	27.23	26.92	26.60	26.30	26.01	25.56	25.27	25.06
629.18	28.37	28.14	27.78	27.38	27.10	26.80	26.38	26.10	25.81	25.45	25.10
677.61	28.91	28.64	28.27	27.95	27.57	27.31	26.88	26.58	26.26	25.87	25.59
729.83	28.91	28.64	28.17	27.87	27.47	27.23	26.83	26.58	26.19	25.84	25.55
786.16	29.20	28.93	28.64	28.17	27.85	27.53	27.16	26.87	26.52	26.14	25.88
846.90	29.14	28.87	28.45	28.19	27.76	27.47	27.17	26.79	26.44	26.15	25.78
912.42	29.37	29.10	28.78	28.37	28.04	27.69	27.31	27.05	26.67	26.35	26.02
983.08	29.55	29.33	28.82	28.42	28.20	27.79	27.43	27.14	26.74	26.36	26.07
1059.29	29.75	29.39	29.03	28.66	28.26	27.97	27.57	27.35	26.91	26.52	26.19
1141.48	29.57	29.27	28.89	28.57	28.24	27.92	27.54	27.25	26.82	26.47	26.07
1230.13	29.50	29.29	28.82	28.49	28.11	27.75	27.36	27.13	26.68	26.29	25.93
1325.73	29.73	29.37	29.01	28.74	28.32	28.01	27.48	27.34	26.82	26.44	26.15
1428.84	29.60	29.29	28.91	28.52	28.11	27.84	27.37	27.21	26.82	26.39	26.06
1540.05	29.81	29.50	29.12	28.75	28.45	28.05	27.66	27.36	26.99	26.67	26.28

1659.99	29.78	29.45	29.03	28.76	28.39	27.95	27.59	27.26	26.86	26.58	26.18
1789.34	29.57	29.33	28.87	28.55	28.19	27.86	27.46	27.17	26.76	26.43	26.08
1928.85	29.66	29.39	28.96	28.63	28.23	27.92	27.52	27.16	26.83	26.47	26.11
2079.31	29.55	29.32	28.83	28.48	28.18	27.79	27.48	27.15	26.72	26.43	26.10
2241.59	29.43	29.14	28.77	28.43	27.98	27.73	27.32	27.06	26.63	26.24	25.96
2416.60	29.36	29.08	28.70	28.24	27.95	27.53	27.17	26.89	26.47	26.11	25.78
2605.36	29.30	29.06	28.62	28.24	27.87	27.48	27.19	26.80	26.45	26.06	25.74
2808.94	29.03	28.81	28.37	28.05	27.61	27.34	26.96	26.68	26.27	25.87	25.56
3028.50	28.90	28.64	28.20	27.83	27.47	27.12	26.75	26.50	26.15	25.65	25.47
3265.30	28.81	28.50	28.04	27.71	27.43	26.99	26.65	26.36	25.90	25.61	25.25
3520.69	28.47	28.21	27.72	27.39	27.06	26.70	26.27	26.01	25.67	25.20	24.90
3796.13	28.28	27.99	27.56	27.28	26.88	26.53	26.15	25.82	25.45	25.10	24.80

Wavelength (nm) / Time (ps)	616.05	617.48	618.90	620.32	621.75	623.17	624.60	626.02	627.44	628.87	630.29
-3.78	-0.02	0.00	-0.02	-0.07	0.02	-0.07	0.03	-0.08	-0.07	0.03	-0.10
-3.28	-0.02	0.01	-0.03	-0.07	-0.06	0.01	-0.08	-0.04	-0.03	-0.04	-0.08
-2.78	0.05	0.03	-0.01	0.02	0.02	-0.01	0.01	0.01	-0.01	0.04	0.03
-2.28	-0.05	0.00	0.01	0.04	-0.01	0.04	0.01	0.02	-0.04	0.03	-0.01
-1.78	-0.02	-0.06	-0.02	-0.07	-0.09	-0.12	-0.07	-0.11	-0.08	-0.14	-0.08
-1.28	0.06	0.04	0.03	0.10	0.00	0.01	0.05	0.05	0.11	0.06	0.05
-0.78	0.04	0.01	0.06	0.05	0.14	0.12	0.08	0.10	0.17	0.10	0.19
-0.28	-0.05	-0.02	-0.03	0.00	-0.03	0.02	-0.03	0.04	-0.04	-0.08	0.00
-0.18	-0.01	0.03	0.08	0.01	0.05	-0.03	0.07	-0.03	0.00	0.00	0.04
-0.08	-0.05	0.01	0.00	0.07	0.04	0.09	0.06	0.03	0.05	0.11	0.05
0.02	0.07	0.07	0.09	0.09	0.17	0.12	0.17	0.03	0.08	0.14	0.08
0.12	-0.01	0.04	-0.01	0.02	0.05	-0.04	0.06	0.02	0.00	0.10	0.07
0.22	0.00	0.12	0.07	0.08	0.05	0.00	0.04	0.07	0.03	0.04	0.06
0.27	-0.03	0.02	0.04	0.03	0.08	0.02	0.11	-0.05	0.07	0.12	0.07
0.32	-0.03	0.05	0.05	-0.09	-0.03	-0.09	-0.05	-0.02	-0.05	-0.03	-0.13
0.37	-0.02	0.02	-0.04	-0.08	-0.05	0.00	0.00	-0.06	-0.07	0.04	-0.09
0.42	-0.09	-0.04	-0.06	-0.10	-0.09	-0.10	-0.09	-0.07	-0.09	-0.02	0.01
0.47	0.02	0.04	0.03	0.03	0.02	0.05	0.12	0.12	0.15	0.13	0.11
0.52	-0.01	0.02	0.08	0.05	0.07	0.03	0.08	0.06	0.08	0.03	0.04
0.57	-0.06	-0.05	-0.04	-0.05	-0.09	-0.14	-0.11	-0.13	-0.04	-0.07	-0.11
0.62	-0.02	-0.03	-0.03	0.01	-0.03	-0.12	-0.03	-0.01	-0.06	-0.03	-0.02
0.67	0.05	0.02	0.01	0.00	0.00	-0.05	0.12	-0.01	0.01	0.06	0.06
0.72	-0.02	-0.08	0.05	0.02	0.01	-0.09	0.10	-0.01	0.00	0.03	0.03
0.77	0.08	0.03	0.00	-0.03	-0.01	-0.03	0.07	-0.07	0.02	-0.02	0.07
0.82	-0.01	0.07	0.00	0.00	0.02	0.02	0.02	0.00	-0.02	0.04	0.06
0.87	0.05	0.03	0.04	-0.03	0.01	-0.04	-0.02	-0.01	0.06	0.04	-0.05
0.92	0.00	0.12	0.07	0.18	0.10	0.06	0.10	0.11	0.09	0.15	0.18
0.97	-0.06	-0.03	-0.02	-0.10	-0.09	-0.10	-0.07	-0.05	-0.05	-0.10	-0.10
1.02	0.10	0.05	0.08	0.08	0.09	0.09	0.04	0.04	0.10	0.05	0.06
1.07	0.04	0.00	0.07	0.10	0.11	0.05	0.11	0.04	0.13	0.14	0.08
1.12	0.10	0.06	0.02	0.02	0.13	-0.01	0.14	0.05	0.07	0.11	0.03
1.17	0.04	0.19	0.13	0.00	0.05	0.10	0.11	0.06	0.03	0.11	0.10
1.22	0.08	0.20	0.07	-0.07	-0.10	0.05	0.24	0.20	0.06	-0.09	0.02

1.27	-0.03	0.12	0.10	-0.01	-0.01	-0.14	-0.02	-0.02	-0.04	-0.01	-0.02
1.32	0.07	-1.23	-2.13	-2.70	-2.86	-2.65	-2.04	-1.44	-0.58	0.10	0.47
1.37	-15.49	-15.84	-16.46	-17.31	-18.19	-18.31	-17.81	-16.85	-15.21	-13.30	-11.26
1.42	86.81	93.99	100.81	105.52	108.59	111.45	113.12	113.72	114.03	113.65	113.05
1.47	14.31	13.98	13.19	11.94	10.29	8.07	5.49	2.34	-0.85	-4.15	-7.53
1.52	17.20	17.58	18.16	18.67	19.32	19.85	20.52	21.17	21.82	22.45	23.00
1.57	15.89	16.36	16.72	17.04	17.20	17.33	17.69	18.14	18.61	18.91	19.16
1.62	13.41	13.79	14.41	14.99	15.46	15.83	16.20	16.81	17.56	18.08	18.30
1.67	13.20	13.51	13.83	14.04	14.31	14.75	15.23	15.65	16.12	16.68	17.20
1.72	13.87	14.20	14.55	14.83	15.12	15.31	15.66	16.08	16.35	16.67	17.10
1.77	12.82	13.05	13.41	13.71	14.15	14.44	14.98	15.33	15.89	16.32	16.77
1.82	13.28	13.57	13.63	13.73	13.90	14.16	14.41	14.62	15.07	15.40	15.72
1.87	13.96	14.30	14.73	15.03	15.25	15.59	16.04	16.24	16.65	17.07	17.46
1.92	12.93	13.07	13.32	13.44	13.70	14.03	14.50	14.73	15.20	15.65	15.98
1.97	13.87	14.07	14.25	14.37	14.49	14.74	14.98	15.11	15.38	15.82	16.20
2.02	13.19	13.47	13.80	14.04	14.33	14.59	14.93	15.10	15.47	15.86	16.18
2.07	13.23	13.41	13.66	13.78	14.03	14.29	14.65	14.91	15.26	15.69	16.17
2.12	13.32	13.61	13.91	14.06	14.31	14.48	14.82	15.07	15.36	15.72	15.94
2.17	12.53	12.88	13.17	13.43	13.65	13.91	14.33	14.62	14.96	15.35	15.67
2.22	13.67	13.82	13.97	14.09	14.22	14.27	14.65	14.83	15.13	15.43	15.82
2.27	14.02	14.35	14.62	14.83	15.10	15.29	15.64	15.84	16.14	16.50	16.83
2.32	12.94	13.05	13.24	13.53	13.76	13.92	14.35	14.53	14.88	15.19	15.59
2.37	13.83	13.97	14.11	14.25	14.44	14.58	14.77	14.97	15.27	15.55	15.88
2.42	13.49	13.71	13.99	14.12	14.40	14.66	15.00	15.22	15.55	15.80	16.16
2.47	13.80	13.99	14.18	14.28	14.44	14.74	15.10	15.29	15.61	15.97	16.32
2.52	13.86	14.03	14.24	14.38	14.48	14.65	14.98	15.14	15.42	15.73	15.97
2.57	13.90	14.10	14.37	14.61	14.81	15.02	15.41	15.62	15.90	16.23	16.48
2.62	13.94	14.17	14.25	14.38	14.54	14.65	14.97	15.14	15.45	15.83	16.16
2.67	13.85	14.00	14.24	14.44	14.65	14.78	15.00	15.29	15.48	15.76	16.04
2.72	13.62	13.81	13.96	14.17	14.37	14.53	14.82	14.99	15.27	15.63	15.87
2.77	14.15	14.24	14.43	14.43	14.70	14.76	15.08	15.22	15.52	15.77	16.06
2.82	13.58	13.84	14.03	14.23	14.44	14.63	14.97	15.11	15.36	15.62	15.88
2.87	13.71	13.90	14.04	14.22	14.45	14.59	14.84	15.07	15.40	15.72	15.98
2.92	14.23	14.42	14.60	14.68	14.88	15.06	15.23	15.42	15.68	15.96	16.17
2.97	13.77	14.02	14.18	14.37	14.49	14.65	14.97	15.13	15.48	15.72	16.00
3.02	14.29	14.38	14.58	14.71	14.81	15.02	15.30	15.43	15.72	16.08	16.29
3.07	13.91	14.12	14.20	14.44	14.62	14.75	14.99	15.16	15.48	15.69	15.97
3.12	13.64	13.76	14.04	14.18	14.30	14.48	14.78	15.01	15.28	15.54	15.87
3.17	14.19	14.36	14.48	14.68	14.74	14.93	15.18	15.35	15.66	15.91	16.18
3.22	14.25	14.44	14.63	14.89	14.97	15.15	15.40	15.56	15.85	16.23	16.33
3.42	14.48	14.66	14.81	14.87	15.07	15.17	15.45	15.65	15.88	16.16	16.42
3.62	15.03	15.15	15.36	15.49	15.58	15.82	16.03	16.19	16.49	16.77	16.96
3.82	14.42	14.58	14.74	14.85	15.00	15.13	15.42	15.48	15.76	16.06	16.27
4.02	15.10	15.24	15.40	15.55	15.74	15.84	16.11	16.31	16.52	16.83	17.04
4.22	14.61	14.70	14.88	15.04	15.08	15.27	15.50	15.65	15.96	16.15	16.43
4.42	14.77	14.93	15.05	15.18	15.39	15.46	15.66	15.75	16.04	16.28	16.59
4.62	15.11	15.20	15.41	15.46	15.60	15.86	16.00	16.14	16.44	16.70	16.85
4.82	14.89	15.04	15.19	15.36	15.41	15.63	15.82	15.96	16.27	16.43	16.76
5.02	15.13	15.21	15.36	15.50	15.70	15.75	16.09	16.21	16.46	16.70	16.90
5.22	15.24	15.37	15.51	15.57	15.77	15.93	16.09	16.28	16.43	16.73	16.95

5.42	15.39	15.49	15.57	15.70	15.82	15.97	16.15	16.31	16.59	16.85	16.99
5.62	15.40	15.51	15.67	15.71	15.88	16.03	16.22	16.36	16.61	16.90	17.10
5.82	15.27	15.45	15.62	15.65	15.84	15.96	16.18	16.30	16.52	16.76	17.06
6.02	15.38	15.47	15.60	15.65	15.81	15.98	16.09	16.25	16.50	16.78	16.96
6.22	15.22	15.31	15.44	15.45	15.63	15.77	15.95	16.07	16.34	16.58	16.78
6.42	15.43	15.58	15.70	15.78	15.86	15.95	16.19	16.38	16.52	16.79	16.98
6.62	15.74	15.88	15.93	16.02	16.17	16.30	16.47	16.57	16.78	17.04	17.24
6.82	15.43	15.51	15.71	15.76	15.90	15.92	16.23	16.31	16.48	16.78	17.07
7.02	15.91	15.99	16.12	16.15	16.32	16.45	16.60	16.79	17.00	17.27	17.50
7.22	15.51	15.66	15.72	15.79	15.90	16.03	16.26	16.32	16.53	16.78	17.01
7.42	15.90	16.01	16.11	16.29	16.24	16.43	16.59	16.73	16.99	17.23	17.46
7.62	15.70	15.77	15.86	15.96	16.08	16.17	16.37	16.54	16.73	16.91	17.12
7.82	15.70	15.77	15.95	16.10	16.11	16.22	16.44	16.57	16.88	17.11	17.29
8.02	15.45	15.63	15.67	15.81	15.94	16.00	16.23	16.33	16.53	16.71	16.94
8.22	15.70	15.75	15.88	15.88	16.03	16.14	16.39	16.49	16.66	16.94	17.17
8.42	15.24	15.28	15.40	15.55	15.60	15.69	15.91	16.01	16.17	16.40	16.67
8.62	15.75	15.86	15.89	15.94	16.06	16.19	16.40	16.44	16.70	16.91	17.16
8.82	15.87	15.88	16.03	16.16	16.20	16.28	16.51	16.57	16.77	17.03	17.21
9.02	15.45	15.53	15.64	15.75	15.79	15.88	16.11	16.19	16.36	16.60	16.74
9.22	15.55	15.71	15.81	15.88	15.98	16.03	16.21	16.35	16.56	16.82	16.97
9.42	15.90	15.91	16.07	16.11	16.17	16.27	16.55	16.65	16.78	17.11	17.31
9.62	15.50	15.56	15.74	15.75	15.86	15.96	16.16	16.30	16.50	16.72	16.89
9.82	15.78	15.84	15.95	16.00	16.18	16.13	16.38	16.47	16.67	16.92	17.08
10.02	15.71	15.78	15.82	15.99	16.07	16.14	16.37	16.44	16.68	16.87	17.08
10.22	15.81	15.88	16.04	16.07	16.22	16.30	16.45	16.58	16.76	16.98	17.21
10.42	15.82	15.93	15.96	16.10	16.13	16.28	16.49	16.62	16.75	16.99	17.20
10.62	15.75	15.82	15.92	16.00	16.00	16.12	16.36	16.44	16.64	16.86	17.06
10.82	15.56	15.57	15.71	15.77	15.83	15.89	16.04	16.18	16.40	16.56	16.73
11.02	15.73	15.74	15.90	15.92	15.99	16.11	16.37	16.39	16.60	16.75	16.98
11.22	15.50	15.62	15.66	15.78	15.86	15.87	16.13	16.21	16.44	16.66	16.91
11.42	15.21	15.29	15.44	15.35	15.53	15.59	15.79	15.81	16.07	16.22	16.40
11.62	15.63	15.65	15.80	15.91	15.89	16.06	16.29	16.36	16.63	16.88	17.00
11.82	15.59	15.62	15.74	15.77	15.94	15.93	16.10	16.27	16.51	16.77	16.90
12.02	15.63	15.66	15.72	15.73	15.82	15.96	16.19	16.19	16.39	16.63	16.80
12.22	15.76	15.89	15.92	16.07	16.04	16.13	16.40	16.43	16.68	16.94	17.09
12.42	15.32	15.40	15.55	15.58	15.57	15.78	15.94	16.04	16.29	16.52	16.66
12.62	15.47	15.54	15.63	15.63	15.72	15.82	16.04	16.09	16.25	16.53	16.63
12.82	15.67	15.65	15.77	15.83	15.82	15.95	16.16	16.29	16.46	16.69	16.86
13.02	15.66	15.71	15.82	15.87	15.95	16.05	16.17	16.27	16.45	16.59	16.83
13.22	15.69	15.75	15.75	15.92	15.95	15.98	16.27	16.37	16.53	16.76	17.05
13.42	15.54	15.65	15.75	15.78	15.86	16.00	16.18	16.20	16.43	16.67	16.86
13.50	15.48	15.56	15.66	15.74	15.85	15.89	16.07	16.15	16.36	16.58	16.75
13.59	15.71	15.75	15.88	15.82	15.99	16.01	16.21	16.30	16.50	16.67	16.87
13.68	15.66	15.66	15.77	15.87	15.91	15.98	16.21	16.34	16.47	16.68	16.88
13.78	15.25	15.21	15.36	15.39	15.54	15.56	15.81	15.87	16.05	16.29	16.47
13.88	15.54	15.61	15.70	15.74	15.78	15.87	16.04	16.17	16.36	16.52	16.76
14.00	15.79	15.90	15.91	16.03	16.05	16.11	16.32	16.43	16.67	16.87	17.04
14.12	15.61	15.73	15.82	15.82	15.93	16.01	16.19	16.34	16.46	16.65	16.88
14.25	15.57	15.62	15.65	15.72	15.79	15.87	16.07	16.24	16.38	16.59	16.81
14.40	15.99	15.97	16.13	16.15	16.20	16.32	16.37	16.53	16.74	16.98	17.16

14.55	15.52	15.53	15.59	15.59	15.73	15.82	15.96	16.07	16.28	16.50	16.66
14.72	15.51	15.58	15.67	15.74	15.80	15.86	16.04	16.14	16.31	16.55	16.74
14.90	15.44	15.35	15.57	15.61	15.65	15.69	15.92	16.03	16.15	16.40	16.56
15.09	15.30	15.34	15.47	15.48	15.58	15.69	15.85	16.03	16.19	16.38	16.52
15.30	15.69	15.67	15.76	15.85	15.92	15.99	16.27	16.31	16.51	16.70	16.99
15.53	15.89	16.06	16.03	16.23	16.26	16.31	16.57	16.60	16.82	17.01	17.20
15.77	15.67	15.75	15.82	15.84	15.97	16.09	16.21	16.35	16.60	16.78	16.99
16.04	15.76	15.80	15.85	15.90	15.97	16.00	16.27	16.34	16.52	16.67	16.85
16.32	15.70	15.75	15.85	15.90	16.03	16.03	16.28	16.38	16.49	16.74	17.00
16.63	15.86	15.83	15.93	15.97	16.06	16.11	16.35	16.41	16.60	16.84	17.07
16.96	16.10	16.21	16.23	16.33	16.46	16.50	16.67	16.78	16.91	17.18	17.35
17.31	15.67	15.75	15.84	15.84	15.91	16.00	16.19	16.23	16.47	16.58	16.76
17.70	16.20	16.25	16.38	16.35	16.45	16.50	16.71	16.79	17.04	17.15	17.37
18.11	15.39	15.38	15.56	15.63	15.68	15.64	15.90	15.96	16.14	16.37	16.58
18.56	15.71	15.67	15.75	15.81	15.85	15.88	16.05	16.19	16.35	16.57	16.71
19.04	15.75	15.81	15.95	15.86	15.99	16.01	16.26	16.31	16.52	16.71	16.93
19.56	15.98	16.02	16.05	16.10	16.22	16.25	16.44	16.58	16.71	16.95	17.04
20.12	15.95	15.98	16.05	16.08	16.16	16.23	16.36	16.47	16.66	16.91	17.07
20.72	16.09	16.16	16.08	16.18	16.25	16.28	16.47	16.53	16.69	16.90	17.13
21.37	16.06	16.16	16.25	16.33	16.33	16.31	16.63	16.60	16.82	17.01	17.25
22.08	15.90	15.97	16.05	16.10	16.09	16.19	16.41	16.51	16.71	16.88	17.05
22.84	16.44	16.37	16.51	16.58	16.59	16.64	16.85	16.96	17.12	17.33	17.54
23.65	15.76	15.83	15.82	15.91	15.93	15.94	16.13	16.32	16.41	16.57	16.77
24.54	16.20	16.22	16.30	16.35	16.42	16.39	16.60	16.65	16.89	17.07	17.22
25.49	16.38	16.43	16.48	16.52	16.57	16.65	16.85	16.89	17.14	17.29	17.45
26.51	15.90	15.96	16.07	16.06	16.10	16.20	16.31	16.45	16.62	16.81	17.00
27.62	16.53	16.58	16.60	16.65	16.68	16.71	16.88	16.99	17.16	17.35	17.49
28.81	16.46	16.45	16.57	16.54	16.67	16.69	16.80	16.85	17.08	17.34	17.48
30.10	16.00	16.03	16.14	16.18	16.23	16.28	16.42	16.46	16.68	16.84	17.04
31.49	16.60	16.65	16.68	16.68	16.71	16.81	16.94	16.93	17.10	17.30	17.51
32.98	16.38	16.45	16.41	16.45	16.39	16.49	16.65	16.79	16.83	17.03	17.24
34.60	16.87	16.85	16.90	16.88	16.96	16.97	17.13	17.19	17.40	17.52	17.70
36.34	16.62	16.63	16.65	16.65	16.71	16.78	16.95	17.00	17.16	17.34	17.50
38.21	17.24	17.30	17.28	17.32	17.33	17.40	17.49	17.55	17.73	17.87	18.09
40.24	16.85	16.91	16.92	16.99	16.97	16.95	17.12	17.14	17.35	17.40	17.65
42.42	16.65	16.70	16.71	16.66	16.69	16.68	16.84	16.93	17.06	17.18	17.29
44.78	16.85	16.81	16.88	16.87	16.86	16.96	17.00	17.07	17.18	17.38	17.57
47.32	16.74	16.75	16.79	16.73	16.73	16.76	16.96	16.95	17.10	17.35	17.47
50.06	17.01	17.03	17.03	17.06	17.08	17.01	17.19	17.25	17.39	17.55	17.66
53.01	17.17	17.19	17.19	17.21	17.27	17.21	17.35	17.46	17.66	17.75	17.87
56.20	17.35	17.29	17.36	17.32	17.35	17.36	17.46	17.50	17.58	17.80	17.85
59.64	17.78	17.86	17.88	17.86	17.84	17.92	18.07	18.03	18.13	18.31	18.40
63.34	17.20	17.17	17.16	17.16	17.11	17.14	17.25	17.29	17.44	17.59	17.63
67.34	17.56	17.52	17.51	17.44	17.48	17.42	17.59	17.59	17.69	17.80	17.91
71.65	18.17	18.13	18.15	18.05	18.06	18.02	18.16	18.15	18.26	18.34	18.51
76.30	18.03	17.95	17.96	17.90	17.76	17.88	17.95	17.93	18.00	18.18	18.22
81.32	18.50	18.47	18.43	18.38	18.35	18.37	18.39	18.30	18.50	18.59	18.71
86.73	18.70	18.68	18.62	18.57	18.55	18.44	18.57	18.61	18.66	18.73	18.83
92.56	18.83	18.82	18.82	18.71	18.68	18.67	18.71	18.75	18.84	18.88	19.00
98.85	18.57	18.46	18.46	18.40	18.31	18.23	18.30	18.33	18.39	18.55	18.56

105.64	19.30	19.20	19.16	19.03	18.98	18.94	19.03	18.99	19.08	19.12	19.26
112.96	19.55	19.43	19.41	19.36	19.29	19.16	19.24	19.25	19.22	19.27	19.33
120.85	19.48	19.45	19.39	19.26	19.22	19.11	19.18	19.15	19.18	19.23	19.26
129.36	19.86	19.81	19.81	19.66	19.54	19.49	19.57	19.50	19.52	19.61	19.66
138.55	20.07	19.90	19.85	19.69	19.60	19.56	19.58	19.51	19.53	19.56	19.63
148.45	19.95	19.78	19.66	19.64	19.45	19.40	19.44	19.30	19.38	19.38	19.42
159.13	20.27	20.25	20.11	19.98	19.91	19.77	19.76	19.75	19.70	19.87	19.86
170.65	20.70	20.46	20.38	20.28	20.20	20.05	20.05	20.03	20.06	20.08	20.05
183.07	21.09	20.95	20.85	20.77	20.63	20.47	20.45	20.40	20.26	20.41	20.37
196.47	21.12	20.98	20.88	20.75	20.56	20.47	20.46	20.32	20.31	20.35	20.31
210.91	21.54	21.43	21.26	21.15	21.04	20.89	20.84	20.68	20.69	20.67	20.63
226.50	21.68	21.47	21.34	21.18	21.01	20.86	20.85	20.68	20.65	20.64	20.56
243.31	21.83	21.60	21.54	21.41	21.22	21.05	20.98	20.86	20.80	20.80	20.77
261.43	22.52	22.35	22.10	22.02	21.79	21.62	21.57	21.43	21.43	21.37	21.30
280.98	22.40	22.20	22.14	21.92	21.73	21.54	21.50	21.28	21.30	21.20	21.12
302.07	22.58	22.41	22.16	21.99	21.91	21.62	21.58	21.42	21.31	21.23	21.12
324.81	23.10	22.97	22.68	22.53	22.32	22.18	22.10	21.83	21.72	21.73	21.62
349.33	23.23	23.06	22.84	22.58	22.42	22.12	22.03	21.85	21.80	21.65	21.58
375.79	23.51	23.31	23.03	22.81	22.68	22.45	22.38	22.11	21.97	21.93	21.84
404.31	23.48	23.18	23.02	22.77	22.59	22.31	22.19	22.03	21.82	21.71	21.62
435.08	23.96	23.68	23.56	23.38	23.21	23.02	22.80	22.57	22.44	22.28	22.14
468.27	24.03	23.78	23.59	23.31	23.10	22.93	22.76	22.55	22.39	22.35	22.17
504.06	24.42	24.19	23.90	23.75	23.43	23.17	22.96	22.77	22.67	22.50	22.26
542.66	24.57	24.27	24.06	23.76	23.55	23.29	23.14	22.88	22.72	22.57	22.42
584.29	24.69	24.38	24.11	23.90	23.55	23.27	23.10	22.85	22.77	22.56	22.30
629.18	24.84	24.58	24.22	23.89	23.72	23.41	23.20	22.95	22.70	22.63	22.40
677.61	25.17	25.01	24.69	24.37	24.11	23.83	23.60	23.31	23.23	23.08	22.85
729.83	25.25	24.96	24.74	24.37	24.09	23.73	23.56	23.32	23.14	22.96	22.75
786.16	25.48	25.21	24.86	24.54	24.24	23.85	23.71	23.34	23.21	23.05	22.79
846.90	25.45	25.09	24.89	24.61	24.33	23.99	23.71	23.56	23.30	23.07	22.90
912.42	25.70	25.36	24.97	24.76	24.45	24.21	23.96	23.67	23.47	23.29	23.09
983.08	25.62	25.34	25.11	24.74	24.47	24.24	24.02	23.65	23.50	23.25	23.10
1059.29	25.80	25.56	25.22	24.95	24.60	24.24	24.10	23.82	23.52	23.39	23.08
1141.48	25.77	25.51	25.18	24.90	24.61	24.28	24.07	23.77	23.49	23.30	23.07
1230.13	25.57	25.26	24.94	24.71	24.39	23.98	23.82	23.46	23.22	23.03	22.78
1325.73	25.83	25.56	25.20	24.86	24.55	24.20	24.01	23.69	23.59	23.30	23.15
1428.84	25.74	25.36	25.04	24.70	24.48	24.13	23.89	23.61	23.39	23.11	22.91
1540.05	25.87	25.55	25.30	25.01	24.64	24.36	24.21	23.79	23.59	23.33	23.14
1659.99	25.83	25.51	25.18	24.93	24.53	24.31	24.01	23.75	23.46	23.26	22.99
1789.34	25.66	25.35	25.10	24.86	24.55	24.16	23.96	23.67	23.41	23.16	23.01
1928.85	25.69	25.40	25.11	24.81	24.47	24.16	23.93	23.61	23.37	23.20	22.95
2079.31	25.65	25.27	25.02	24.73	24.40	24.12	23.86	23.47	23.22	23.11	22.87
2241.59	25.58	25.25	24.92	24.63	24.27	23.92	23.80	23.40	23.22	22.99	22.78
2416.60	25.36	25.06	24.77	24.43	24.16	23.81	23.44	23.19	23.06	22.74	22.59
2605.36	25.34	24.99	24.77	24.33	24.05	23.70	23.47	23.19	22.94	22.80	22.50
2808.94	25.18	24.79	24.61	24.25	24.01	23.67	23.37	23.20	22.94	22.71	22.51
3028.50	25.09	24.76	24.52	24.12	23.81	23.48	23.27	22.98	22.77	22.54	22.35
3265.30	24.90	24.59	24.25	23.98	23.62	23.35	23.10	22.72	22.57	22.38	22.08
3520.69	24.52	24.27	24.00	23.63	23.33	23.01	22.81	22.47	22.36	22.12	21.86
3796.13	24.37	24.07	23.83	23.38	23.15	22.75	22.63	22.27	22.10	21.85	21.65

Time (ps) \ Wavelength (nm)	631.72	633.14	634.56	635.99	637.41	638.84	640.26	641.68	643.11	644.53	645.96
-3.78	-0.02	-0.02	-0.12	-0.07	-0.04	-0.13	-0.07	-0.06	-0.04	-0.04	-0.05
-3.28	-0.04	-0.04	-0.06	0.05	0.00	0.03	-0.08	-0.05	-0.04	-0.05	0.00
-2.78	-0.04	0.03	0.04	-0.01	-0.03	-0.07	0.03	-0.04	-0.04	-0.05	-0.05
-2.28	-0.06	-0.07	0.00	-0.03	0.02	-0.01	-0.01	0.00	-0.02	-0.03	-0.01
-1.78	-0.08	-0.09	-0.09	-0.11	-0.07	-0.03	0.02	-0.06	0.01	0.05	0.08
-1.28	0.06	0.12	0.07	0.09	0.09	0.14	0.04	0.11	0.10	0.08	0.10
-0.78	0.16	0.16	0.16	0.15	0.10	0.11	0.13	0.08	0.12	0.08	0.07
-0.28	0.03	-0.09	-0.01	-0.06	-0.06	-0.03	-0.06	0.02	-0.10	-0.04	-0.13
-0.18	0.03	-0.01	0.00	0.08	0.13	0.06	0.02	0.03	0.05	0.06	0.00
-0.08	0.06	0.07	0.07	0.03	0.09	0.05	0.06	0.01	0.12	0.02	0.11
0.02	0.08	0.08	0.07	0.05	0.12	0.09	0.05	0.00	0.05	-0.02	0.00
0.12	0.06	0.10	0.00	0.14	0.04	0.13	0.18	0.15	0.15	0.12	0.15
0.22	0.01	-0.07	-0.01	-0.08	0.00	-0.05	-0.03	0.00	-0.04	-0.05	-0.02
0.27	0.13	0.09	0.12	0.08	0.15	0.16	0.12	0.13	0.15	0.19	0.16
0.32	-0.13	-0.04	-0.09	-0.07	-0.07	-0.03	-0.04	-0.08	-0.08	-0.07	-0.09
0.37	-0.08	-0.01	-0.04	-0.01	0.03	0.02	0.00	0.05	0.06	0.10	0.08
0.42	-0.08	-0.03	-0.02	-0.03	0.02	-0.03	0.07	0.16	0.13	0.10	0.13
0.47	0.09	0.12	0.16	0.12	0.09	0.21	0.16	0.18	0.20	0.12	0.12
0.52	0.03	0.05	0.08	0.02	0.05	0.04	0.08	0.06	-0.03	0.08	-0.01
0.57	-0.07	-0.11	-0.07	-0.15	-0.05	-0.05	-0.03	0.01	0.05	0.03	0.05
0.62	-0.02	-0.03	-0.07	-0.07	-0.12	-0.04	-0.09	-0.06	0.02	0.02	0.05
0.67	-0.07	0.03	0.09	0.00	0.03	0.05	0.06	0.09	0.04	0.09	0.09
0.72	0.00	0.05	0.11	0.06	0.05	0.07	0.12	0.13	0.23	0.19	0.24
0.77	0.04	0.04	0.07	0.08	0.06	0.03	0.11	0.15	0.11	0.08	0.12
0.82	0.04	0.03	-0.04	0.08	-0.01	0.02	-0.05	0.07	0.08	-0.02	0.02
0.87	-0.03	-0.01	-0.06	-0.05	-0.02	0.02	0.02	0.03	0.05	-0.02	0.08
0.92	0.11	0.12	0.16	0.08	0.14	0.10	0.07	0.09	0.06	0.04	0.09
0.97	-0.16	-0.13	-0.10	-0.02	-0.04	-0.02	-0.04	-0.01	0.08	0.02	0.05
1.02	0.06	0.08	0.04	0.02	0.00	0.03	-0.06	-0.09	-0.03	-0.06	-0.07
1.07	0.10	0.11	0.03	0.11	0.13	0.12	0.08	0.07	0.04	0.01	-0.03
1.12	0.03	0.05	0.07	0.08	0.09	0.08	-0.01	0.03	0.03	0.01	0.01
1.17	0.03	-0.02	0.06	0.10	0.00	0.03	-0.04	-0.05	-0.06	-0.08	-0.14
1.22	0.10	0.17	0.14	-0.03	-0.09	-0.01	0.01	0.08	0.04	0.03	-0.08
1.27	-0.14	-0.18	-0.19	-0.20	-0.07	-0.05	-0.07	-0.02	-0.12	-0.13	-0.15
1.32	0.75	0.81	0.73	0.51	0.16	-0.08	-0.33	-0.59	-0.71	-0.73	-0.58
1.37	-9.44	-7.85	-7.36	-7.20	-7.67	-8.67	-10.28	-12.21	-14.52	-16.86	-18.85
1.42	112.28	111.97	110.92	110.20	109.39	109.15	108.13	106.64	104.43	100.54	97.07
1.47	-10.54	-12.89	-14.83	-15.84	-15.76	-14.84	-12.92	-9.98	-6.15	-1.72	4.21
1.52	23.52	24.00	24.53	25.05	25.48	25.94	26.40	26.84	27.35	27.69	28.16
1.57	19.57	20.23	20.78	21.24	21.58	22.00	22.62	23.29	23.77	24.00	24.45
1.62	18.50	19.24	20.17	20.91	21.21	21.55	22.17	23.34	24.40	24.98	25.14
1.67	17.70	18.26	18.77	19.21	19.76	20.38	20.86	21.45	22.06	22.65	23.34
1.72	17.51	18.00	18.44	18.91	19.48	20.04	20.47	20.98	21.49	21.84	22.36
1.77	17.20	17.71	18.19	18.68	19.17	19.80	20.34	21.05	21.67	22.24	23.07
1.82	16.16	16.57	17.06	17.47	17.93	18.43	18.81	19.29	19.82	20.29	20.86

1.87	17.92	18.38	18.85	19.28	19.86	20.43	20.85	21.36	21.89	22.36	22.78
1.92	16.45	16.87	17.35	17.86	18.33	18.87	19.46	19.97	20.65	21.31	21.98
1.97	16.53	16.94	17.37	17.81	18.26	18.70	19.09	19.56	19.98	20.38	20.81
2.02	16.49	16.88	17.28	17.68	18.14	18.66	19.16	19.68	20.21	20.69	21.27
2.07	16.52	16.93	17.33	17.82	18.29	18.75	19.19	19.79	20.36	20.89	21.57
2.12	16.31	16.75	17.13	17.54	17.93	18.44	18.83	19.37	19.76	20.17	20.65
2.17	15.98	16.43	16.91	17.34	17.81	18.37	18.85	19.40	20.02	20.61	21.20
2.22	16.19	16.49	16.86	17.38	17.74	18.25	18.70	19.12	19.56	20.03	20.54
2.27	17.22	17.64	18.10	18.50	19.01	19.45	19.92	20.39	20.86	21.29	21.78
2.32	15.90	16.33	16.74	17.08	17.54	17.97	18.40	18.92	19.50	19.95	20.59
2.37	16.22	16.52	17.00	17.31	17.71	18.24	18.59	19.02	19.49	19.93	20.42
2.42	16.39	16.93	17.22	17.62	18.02	18.53	19.02	19.56	19.98	20.42	20.92
2.47	16.66	17.00	17.41	17.84	18.30	18.74	19.21	19.74	20.18	20.73	21.22
2.52	16.37	16.67	17.05	17.46	17.86	18.29	18.73	19.21	19.63	20.00	20.52
2.57	16.84	17.24	17.60	17.97	18.48	18.90	19.40	19.89	20.41	20.92	21.44
2.62	16.36	16.71	17.19	17.51	17.96	18.39	18.74	19.24	19.67	20.11	20.66
2.67	16.32	16.74	17.16	17.47	17.93	18.26	18.72	19.10	19.55	19.92	20.38
2.72	16.30	16.59	17.04	17.34	17.73	18.23	18.65	19.14	19.68	20.14	20.77
2.77	16.36	16.75	17.03	17.36	17.83	18.22	18.64	19.08	19.45	19.89	20.34
2.82	16.18	16.53	16.92	17.24	17.65	18.08	18.55	18.97	19.30	19.71	20.16
2.87	16.25	16.66	17.04	17.36	17.73	18.18	18.67	19.03	19.47	19.99	20.48
2.92	16.48	16.76	17.20	17.46	17.91	18.23	18.66	19.07	19.40	19.78	20.27
2.97	16.26	16.57	16.93	17.26	17.66	18.14	18.50	18.94	19.35	19.69	20.26
3.02	16.62	16.94	17.31	17.68	18.08	18.50	18.85	19.36	19.80	20.22	20.74
3.07	16.23	16.57	16.89	17.28	17.66	18.05	18.42	18.89	19.18	19.57	20.05
3.12	16.16	16.46	16.77	17.16	17.49	17.98	18.39	18.88	19.34	19.73	20.29
3.17	16.54	16.77	17.06	17.40	17.74	18.13	18.49	18.82	19.26	19.55	19.97
3.22	16.69	17.04	17.36	17.72	18.12	18.47	18.95	19.33	19.76	20.25	20.66
3.42	16.76	17.02	17.35	17.69	18.00	18.40	18.86	19.20	19.52	19.95	20.32
3.62	17.24	17.64	17.94	18.24	18.60	18.95	19.41	19.74	20.19	20.55	20.98
3.82	16.56	16.85	17.13	17.55	17.84	18.23	18.64	19.05	19.52	19.83	20.31
4.02	17.38	17.70	18.00	18.28	18.66	19.06	19.44	19.79	20.14	20.55	21.00
4.22	16.61	16.95	17.30	17.53	17.89	18.29	18.63	18.99	19.40	19.72	20.20
4.42	16.80	17.08	17.38	17.71	18.09	18.52	18.88	19.22	19.67	20.06	20.39
4.62	17.25	17.47	17.74	18.07	18.39	18.69	18.99	19.33	19.68	20.09	20.43
4.82	16.93	17.26	17.61	17.96	18.30	18.65	19.01	19.41	19.77	20.16	20.51
5.02	17.13	17.50	17.73	18.08	18.39	18.71	19.02	19.37	19.70	20.13	20.48
5.22	17.22	17.43	17.75	18.15	18.41	18.81	19.15	19.50	19.93	20.30	20.60
5.42	17.32	17.53	17.84	18.19	18.47	18.84	19.20	19.53	19.88	20.24	20.60
5.62	17.33	17.62	17.93	18.20	18.61	18.96	19.27	19.58	19.93	20.32	20.71
5.82	17.25	17.54	17.79	18.04	18.46	18.79	19.08	19.44	19.85	20.26	20.61
6.02	17.15	17.47	17.70	18.03	18.30	18.74	19.00	19.37	19.76	20.02	20.41
6.22	17.02	17.29	17.60	17.95	18.28	18.56	18.94	19.28	19.64	20.01	20.37
6.42	17.23	17.51	17.80	18.09	18.44	18.77	19.07	19.37	19.73	20.15	20.50
6.62	17.51	17.79	18.12	18.39	18.62	19.02	19.35	19.75	20.08	20.43	20.84
6.82	17.23	17.49	17.80	18.00	18.40	18.70	18.98	19.37	19.73	19.99	20.41
7.02	17.68	18.08	18.30	18.59	18.95	19.25	19.60	19.96	20.31	20.66	21.00
7.22	17.19	17.45	17.76	18.05	18.37	18.65	19.01	19.36	19.75	20.08	20.44
7.42	17.66	18.04	18.27	18.57	18.89	19.22	19.64	19.95	20.35	20.70	21.07
7.62	17.36	17.64	17.89	18.23	18.53	18.88	19.13	19.56	19.95	20.23	20.51

7.82	17.48	17.81	18.10	18.34	18.67	19.01	19.32	19.70	19.99	20.29	20.72
8.02	17.20	17.43	17.78	18.00	18.29	18.58	18.92	19.19	19.62	19.88	20.28
8.22	17.38	17.71	17.95	18.20	18.60	18.93	19.36	19.63	20.01	20.33	20.70
8.42	16.80	17.18	17.40	17.77	18.04	18.44	18.69	19.08	19.43	19.80	20.22
8.62	17.34	17.60	17.98	18.20	18.56	18.88	19.15	19.45	19.84	20.14	20.55
8.82	17.46	17.74	17.98	18.24	18.60	18.88	19.21	19.56	19.86	20.20	20.55
9.02	16.97	17.33	17.61	17.87	18.18	18.54	18.82	19.14	19.44	19.89	20.25
9.22	17.24	17.51	17.83	18.06	18.35	18.71	19.05	19.41	19.75	20.08	20.46
9.42	17.45	17.78	18.05	18.26	18.61	18.86	19.12	19.57	19.86	20.18	20.54
9.62	17.17	17.41	17.67	18.00	18.26	18.72	18.94	19.34	19.71	20.03	20.37
9.82	17.33	17.64	17.86	18.20	18.49	18.84	19.20	19.56	19.85	20.25	20.63
10.02	17.22	17.47	17.84	18.10	18.29	18.62	18.89	19.22	19.51	19.86	20.25
10.22	17.37	17.66	17.87	18.20	18.43	18.77	19.07	19.42	19.75	20.01	20.44
10.42	17.44	17.74	18.01	18.30	18.56	18.96	19.31	19.64	20.00	20.29	20.73
10.62	17.27	17.56	17.86	18.12	18.39	18.74	19.10	19.48	19.79	20.09	20.46
10.82	16.99	17.26	17.43	17.80	18.08	18.51	18.78	19.12	19.46	19.86	20.27
11.02	17.23	17.55	17.70	17.99	18.36	18.69	18.95	19.29	19.69	20.00	20.42
11.22	17.06	17.34	17.59	17.85	18.20	18.49	18.68	19.14	19.51	19.75	20.14
11.42	16.63	16.94	17.17	17.46	17.70	18.10	18.33	18.69	19.02	19.34	19.74
11.62	17.32	17.59	17.89	18.25	18.48	18.82	19.26	19.58	19.94	20.27	20.67
11.82	17.15	17.37	17.67	17.96	18.22	18.60	18.85	19.17	19.55	19.88	20.22
12.02	17.11	17.30	17.57	17.84	18.13	18.53	18.91	19.28	19.61	19.90	20.35
12.22	17.32	17.57	17.87	18.11	18.49	18.76	19.13	19.49	19.83	20.19	20.59
12.42	16.86	17.24	17.50	17.74	18.02	18.37	18.71	18.93	19.36	19.69	20.01
12.62	16.87	17.15	17.36	17.70	17.94	18.27	18.61	18.89	19.27	19.61	19.90
12.82	17.08	17.36	17.55	17.85	18.15	18.43	18.70	19.03	19.38	19.69	20.06
13.02	17.06	17.34	17.59	17.81	18.12	18.49	18.78	19.18	19.45	19.91	20.32
13.22	17.21	17.48	17.74	18.00	18.34	18.71	19.01	19.30	19.65	20.02	20.43
13.42	17.04	17.33	17.63	17.88	18.28	18.57	18.89	19.23	19.63	19.93	20.30
13.50	17.00	17.23	17.48	17.76	18.08	18.39	18.68	19.04	19.37	19.71	20.01
13.59	17.14	17.41	17.63	17.94	18.25	18.56	18.82	19.29	19.59	19.87	20.34
13.68	17.15	17.47	17.62	17.94	18.26	18.55	18.91	19.23	19.54	19.77	20.19
13.78	16.68	16.96	17.26	17.49	17.81	18.17	18.42	18.85	19.13	19.47	19.83
13.88	16.98	17.27	17.51	17.80	18.10	18.48	18.76	19.21	19.41	19.81	20.12
14.00	17.32	17.57	17.78	18.13	18.40	18.78	19.05	19.44	19.75	20.08	20.48
14.12	17.13	17.32	17.60	17.88	18.18	18.59	18.75	19.17	19.53	19.87	20.15
14.25	17.08	17.36	17.53	17.91	18.17	18.55	18.82	19.17	19.48	19.87	20.22
14.40	17.35	17.65	17.91	18.25	18.54	18.81	19.13	19.50	19.87	20.25	20.53
14.55	16.88	17.12	17.36	17.74	17.97	18.28	18.57	18.97	19.32	19.61	19.97
14.72	16.84	17.17	17.40	17.70	18.01	18.32	18.65	18.96	19.32	19.61	20.02
14.90	16.81	17.00	17.29	17.58	17.84	18.26	18.50	18.87	19.13	19.46	19.91
15.09	16.74	17.04	17.23	17.55	17.87	18.19	18.48	18.79	19.11	19.42	19.77
15.30	17.16	17.42	17.65	17.96	18.31	18.60	18.89	19.23	19.56	19.89	20.27
15.53	17.49	17.69	17.95	18.30	18.54	18.92	19.20	19.52	19.91	20.19	20.56
15.77	17.19	17.49	17.68	18.02	18.33	18.62	18.91	19.36	19.72	19.99	20.44
16.04	17.12	17.38	17.69	17.93	18.29	18.56	18.88	19.36	19.59	20.00	20.33
16.32	17.17	17.36	17.72	17.97	18.28	18.54	18.89	19.18	19.53	19.90	20.29
16.63	17.20	17.49	17.67	18.00	18.33	18.66	18.90	19.27	19.67	19.93	20.37
16.96	17.60	17.92	18.14	18.41	18.77	19.07	19.33	19.66	19.97	20.26	20.65
17.31	17.06	17.30	17.57	17.87	18.16	18.48	18.82	19.15	19.48	19.85	20.18

17.70	17.55	17.84	18.05	18.39	18.70	19.04	19.33	19.63	20.01	20.39	20.69
18.11	16.73	16.99	17.26	17.56	17.86	18.19	18.46	18.83	19.15	19.48	19.81
18.56	16.98	17.22	17.51	17.75	18.09	18.43	18.69	19.03	19.45	19.81	20.15
19.04	17.08	17.44	17.56	17.88	18.18	18.48	18.78	19.19	19.50	19.75	20.12
19.56	17.33	17.58	17.81	18.22	18.46	18.76	19.14	19.45	19.82	20.11	20.59
20.12	17.28	17.52	17.82	18.05	18.34	18.68	18.96	19.36	19.67	19.97	20.40
20.72	17.38	17.59	17.89	18.19	18.49	18.83	19.11	19.53	19.79	20.19	20.60
21.37	17.46	17.62	17.96	18.27	18.47	18.80	19.12	19.44	19.76	20.05	20.49
22.08	17.26	17.52	17.81	18.10	18.43	18.76	19.08	19.49	19.84	20.23	20.65
22.84	17.68	18.05	18.28	18.58	18.84	19.17	19.48	19.91	20.17	20.51	20.91
23.65	16.94	17.24	17.52	17.78	18.17	18.45	18.72	19.06	19.43	19.76	20.16
24.54	17.47	17.73	18.00	18.23	18.49	18.82	19.01	19.47	19.72	20.03	20.41
25.49	17.65	17.98	18.15	18.41	18.75	19.06	19.36	19.70	19.95	20.40	20.77
26.51	17.17	17.44	17.70	17.89	18.19	18.47	18.76	19.14	19.42	19.74	20.01
27.62	17.75	17.96	18.20	18.45	18.75	19.09	19.35	19.69	19.94	20.25	20.64
28.81	17.63	17.89	18.20	18.45	18.74	19.07	19.39	19.73	20.02	20.31	20.67
30.10	17.19	17.42	17.68	17.85	18.20	18.46	18.71	19.11	19.37	19.71	20.02
31.49	17.67	18.00	18.17	18.45	18.77	19.10	19.33	19.69	20.06	20.31	20.69
32.98	17.38	17.60	17.83	18.08	18.45	18.72	19.08	19.27	19.60	19.93	20.35
34.60	17.90	18.17	18.39	18.61	18.85	19.19	19.40	19.73	20.00	20.39	20.65
36.34	17.65	17.89	18.17	18.39	18.69	19.01	19.21	19.50	19.79	20.11	20.43
38.21	18.19	18.42	18.70	18.91	19.18	19.49	19.74	20.01	20.33	20.57	20.89
40.24	17.83	18.11	18.30	18.48	18.85	19.14	19.43	19.73	20.02	20.25	20.69
42.42	17.43	17.70	17.87	18.16	18.40	18.69	18.98	19.28	19.53	19.84	20.14
44.78	17.76	17.96	18.13	18.41	18.63	18.99	19.23	19.46	19.75	20.08	20.38
47.32	17.65	17.80	18.06	18.34	18.62	18.90	19.15	19.42	19.77	20.08	20.37
50.06	17.85	18.07	18.29	18.47	18.74	19.09	19.31	19.62	19.94	20.28	20.55
53.01	18.04	18.25	18.48	18.77	19.04	19.26	19.48	19.77	20.09	20.29	20.62
56.20	18.03	18.20	18.40	18.70	18.89	19.12	19.46	19.65	19.99	20.23	20.49
59.64	18.57	18.75	18.92	19.18	19.42	19.66	19.83	20.16	20.39	20.59	20.90
63.34	17.73	18.03	18.17	18.43	18.66	18.89	19.20	19.42	19.69	19.90	20.26
67.34	18.04	18.32	18.45	18.65	18.86	19.15	19.32	19.62	19.87	20.16	20.42
71.65	18.59	18.81	18.94	19.15	19.39	19.59	19.81	20.06	20.27	20.52	20.82
76.30	18.44	18.61	18.74	18.92	19.23	19.49	19.70	19.96	20.18	20.40	20.67
81.32	18.77	18.92	19.11	19.30	19.50	19.73	19.92	20.25	20.43	20.69	20.98
86.73	18.95	19.05	19.17	19.35	19.63	19.76	20.03	20.21	20.43	20.65	20.95
92.56	19.08	19.31	19.43	19.56	19.80	20.03	20.19	20.44	20.67	20.96	21.15
98.85	18.64	18.73	18.95	19.05	19.31	19.49	19.65	19.90	20.10	20.34	20.58
105.64	19.31	19.53	19.66	19.82	20.02	20.22	20.41	20.66	20.82	21.06	21.35
112.96	19.34	19.54	19.74	19.84	20.01	20.19	20.32	20.57	20.84	20.97	21.19
120.85	19.31	19.44	19.56	19.69	19.82	20.05	20.18	20.36	20.51	20.76	21.05
129.36	19.82	19.81	19.99	20.14	20.30	20.53	20.66	20.90	21.16	21.24	21.54
138.55	19.68	19.73	19.80	19.95	20.13	20.22	20.44	20.64	20.84	21.06	21.26
148.45	19.48	19.57	19.63	19.77	19.90	20.11	20.26	20.45	20.64	20.84	21.07
159.13	19.91	19.94	20.05	20.21	20.35	20.45	20.68	20.80	21.01	21.17	21.36
170.65	20.11	20.26	20.31	20.32	20.51	20.60	20.74	20.89	21.03	21.12	21.33
183.07	20.33	20.40	20.48	20.61	20.72	20.90	20.94	21.05	21.18	21.33	21.51
196.47	20.35	20.34	20.36	20.47	20.47	20.74	20.79	21.01	21.11	21.21	21.36
210.91	20.66	20.71	20.74	20.78	20.87	21.04	21.13	21.18	21.28	21.42	21.55
226.50	20.52	20.66	20.61	20.72	20.80	20.91	20.94	21.11	21.17	21.38	21.45

243.31	20.70	20.77	20.80	20.87	20.97	21.05	21.14	21.20	21.34	21.44	21.58
261.43	21.31	21.26	21.27	21.31	21.39	21.44	21.57	21.56	21.78	21.71	21.91
280.98	21.13	21.08	21.09	21.15	21.19	21.26	21.33	21.39	21.48	21.67	21.71
302.07	21.15	21.11	21.12	21.15	21.18	21.25	21.21	21.31	21.44	21.44	21.49
324.81	21.55	21.54	21.54	21.48	21.54	21.54	21.51	21.56	21.62	21.63	21.72
349.33	21.52	21.50	21.41	21.47	21.39	21.38	21.43	21.52	21.55	21.59	21.57
375.79	21.70	21.71	21.67	21.63	21.63	21.70	21.70	21.71	21.77	21.78	21.88
404.31	21.53	21.43	21.40	21.41	21.34	21.34	21.36	21.41	21.42	21.44	21.58
435.08	22.04	22.04	21.93	21.95	21.87	21.86	21.86	21.88	21.83	21.89	21.86
468.27	22.04	22.02	21.92	21.88	21.86	21.90	21.86	21.88	21.92	21.86	21.99
504.06	22.19	22.15	22.12	22.06	21.96	21.94	21.92	21.91	21.93	21.88	21.92
542.66	22.30	22.26	22.18	22.11	22.03	22.05	22.02	21.96	21.93	21.88	21.89
584.29	22.11	22.09	22.03	21.93	21.92	21.88	21.84	21.82	21.84	21.74	21.82
629.18	22.28	22.23	22.12	22.01	21.94	21.94	21.96	21.94	21.94	21.90	21.89
677.61	22.69	22.51	22.48	22.39	22.30	22.32	22.23	22.25	22.21	22.17	22.15
729.83	22.55	22.39	22.32	22.20	22.08	22.07	21.92	21.87	21.79	21.79	21.70
786.16	22.65	22.53	22.36	22.34	22.18	22.14	22.04	22.05	21.97	21.90	21.89
846.90	22.69	22.58	22.47	22.32	22.31	22.17	22.08	21.95	21.88	21.92	21.82
912.42	22.84	22.72	22.55	22.49	22.28	22.28	22.06	22.03	21.90	21.85	21.76
983.08	22.85	22.75	22.57	22.52	22.36	22.27	22.21	22.06	22.11	22.04	21.91
1059.29	22.90	22.74	22.67	22.60	22.40	22.27	22.21	22.09	22.03	21.92	21.96
1141.48	22.89	22.74	22.60	22.39	22.27	22.18	22.11	21.95	21.91	21.81	21.70
1230.13	22.62	22.53	22.31	22.17	22.06	21.99	21.91	21.80	21.77	21.64	21.62
1325.73	22.94	22.81	22.69	22.55	22.46	22.39	22.25	22.17	22.15	22.04	21.98
1428.84	22.66	22.54	22.43	22.30	22.18	22.03	21.90	21.91	21.75	21.68	21.61
1540.05	22.93	22.83	22.63	22.58	22.45	22.35	22.18	22.11	22.05	21.94	21.91
1659.99	22.78	22.57	22.46	22.31	22.18	22.10	21.96	21.85	21.76	21.66	21.67
1789.34	22.77	22.66	22.54	22.39	22.21	22.11	22.01	21.92	21.74	21.60	21.54
1928.85	22.77	22.64	22.49	22.30	22.24	22.00	21.98	21.82	21.74	21.59	21.52
2079.31	22.69	22.53	22.27	22.19	22.04	21.93	21.81	21.72	21.59	21.52	21.50
2241.59	22.55	22.43	22.25	22.02	21.99	21.87	21.75	21.57	21.51	21.41	21.36
2416.60	22.35	22.19	22.08	21.96	21.84	21.72	21.67	21.55	21.45	21.40	21.32
2605.36	22.27	22.19	22.03	21.86	21.72	21.57	21.54	21.42	21.35	21.20	21.11
2808.94	22.31	22.13	22.02	21.86	21.73	21.54	21.46	21.40	21.23	21.11	21.09
3028.50	22.18	21.95	21.86	21.66	21.54	21.39	21.30	21.10	21.09	20.88	20.80
3265.30	21.83	21.80	21.68	21.52	21.43	21.32	21.16	21.12	20.99	20.92	20.82
3520.69	21.73	21.60	21.40	21.29	21.17	21.12	21.02	20.94	20.85	20.79	20.72
3796.13	21.47	21.30	21.17	21.07	20.92	20.83	20.68	20.63	20.49	20.49	20.34

Wavelength (nm) / Time (ps)	647.38	648.80	650.23	651.65	653.08	654.50	655.93	657.35	658.77	660.20	661.62
-3.78	-0.08	-0.03	-0.05	-0.05	-0.03	-0.06	-0.05	0.01	-0.02	0.00	0.00
-3.28	-0.01	0.03	0.01	-0.01	-0.01	0.03	-0.02	-0.02	-0.06	-0.04	-0.06
-2.78	-0.04	-0.12	-0.13	-0.10	-0.08	-0.06	-0.14	-0.11	-0.08	-0.11	-0.07
-2.28	-0.02	-0.04	-0.02	-0.04	-0.01	-0.01	-0.06	-0.04	0.07	-0.02	0.04
-1.78	0.04	0.07	0.07	0.11	0.18	0.11	0.18	0.21	0.25	0.13	0.18
-1.28	0.09	0.13	0.10	0.11	0.07	0.07	0.13	0.18	0.06	0.17	0.12
-0.78	0.05	0.02	-0.01	-0.03	-0.06	-0.08	-0.09	-0.24	-0.17	-0.16	-0.22

-0.28	-0.03	-0.06	0.03	0.01	-0.06	0.01	0.04	0.00	-0.05	0.03	0.01
-0.18	0.08	-0.04	0.17	-0.02	-0.04	-0.02	0.04	0.08	0.02	0.01	0.00
-0.08	0.16	0.10	0.03	0.04	0.10	0.07	0.10	0.03	0.09	0.00	0.00
0.02	0.07	0.02	-0.08	0.00	-0.04	-0.04	-0.09	-0.03	-0.14	-0.13	-0.15
0.12	0.12	0.19	0.18	0.20	0.19	0.23	0.22	0.29	0.21	0.17	0.21
0.22	-0.04	-0.06	-0.09	-0.08	-0.10	-0.11	-0.07	-0.09	-0.14	-0.13	-0.05
0.27	0.26	0.17	0.20	0.23	0.24	0.14	0.27	0.24	0.19	0.22	0.15
0.32	-0.03	-0.12	-0.03	0.01	-0.11	-0.11	0.00	0.02	-0.02	-0.04	-0.09
0.37	0.14	0.13	0.15	0.22	0.13	0.24	0.21	0.30	0.23	0.31	0.25
0.42	0.20	0.16	0.26	0.26	0.25	0.27	0.28	0.22	0.25	0.30	0.20
0.47	0.23	0.16	0.24	0.24	0.20	0.18	0.30	0.23	0.26	0.20	0.26
0.52	0.04	0.02	0.05	0.01	0.02	0.09	0.04	0.07	0.10	0.08	0.02
0.57	0.11	0.17	0.15	0.15	0.16	0.11	0.22	0.31	0.27	0.23	0.26
0.62	0.05	-0.02	0.05	0.07	-0.03	0.04	0.10	0.12	0.07	0.03	0.08
0.67	0.11	0.06	0.08	0.09	0.06	0.11	0.08	0.18	0.11	0.13	0.06
0.72	0.26	0.31	0.30	0.35	0.32	0.34	0.44	0.43	0.39	0.39	0.40
0.77	0.13	0.13	0.17	0.14	0.19	0.18	0.24	0.19	0.14	0.17	0.13
0.82	0.04	0.00	-0.01	0.00	-0.05	-0.03	-0.03	0.05	-0.02	-0.09	-0.11
0.87	0.08	0.07	0.06	0.09	0.13	0.08	0.11	0.13	0.11	0.09	0.12
0.92	0.05	0.01	0.06	0.01	-0.03	-0.05	-0.03	-0.13	-0.14	-0.24	-0.25
0.97	0.08	0.23	0.22	0.16	0.16	0.23	0.21	0.33	0.32	0.33	0.33
1.02	-0.03	-0.09	-0.06	-0.11	-0.15	-0.07	-0.05	-0.16	-0.14	-0.15	-0.16
1.07	0.05	0.03	0.01	0.02	-0.05	-0.09	-0.11	-0.12	-0.05	-0.12	-0.11
1.12	-0.01	-0.06	-0.03	0.02	0.05	-0.04	0.01	-0.02	0.04	-0.01	0.03
1.17	-0.17	-0.14	-0.24	-0.26	-0.30	-0.38	-0.44	-0.40	-0.48	-0.50	-0.56
1.22	-0.06	-0.11	0.05	0.06	-0.03	-0.14	-0.12	-0.14	-0.14	-0.10	-0.11
1.27	-0.13	-0.19	-0.06	-0.12	-0.05	-0.07	-0.05	-0.07	-0.10	-0.11	-0.15
1.32	-0.35	-0.17	0.01	0.04	0.18	0.12	0.04	-0.06	-0.16	-0.20	-0.25
1.37	-20.58	-21.84	-22.50	-22.46	-21.69	-20.28	-18.15	-15.97	-13.58	-11.21	-9.00
1.42	92.75	87.55	81.41	74.18	67.06	60.11	53.34	46.50	39.17	31.71	24.98
1.47	10.53	17.48	24.73	31.86	39.75	48.05	56.52	64.95	72.62	79.61	86.37
1.52	28.34	28.45	28.62	28.70	28.75	28.93	29.35	29.91	30.60	31.47	32.30
1.57	25.12	25.86	26.63	27.16	27.53	27.96	28.91	29.97	31.17	32.01	32.58
1.62	25.42	26.21	27.48	28.55	28.57	28.32	28.15	28.82	30.12	31.17	31.48
1.67	23.95	24.58	25.37	26.12	26.87	27.60	28.32	29.08	29.66	30.33	30.95
1.72	22.80	23.25	23.78	24.21	24.62	25.11	25.74	26.40	27.05	27.71	28.36
1.77	23.71	24.39	25.17	25.81	26.38	27.05	27.54	28.11	28.61	29.15	29.39
1.82	21.42	21.87	22.66	23.22	23.95	24.68	25.52	26.31	27.15	27.94	28.61
1.87	23.28	23.66	24.09	24.45	24.83	25.25	25.75	26.07	26.66	27.11	27.54
1.92	22.59	23.38	24.14	24.86	25.58	26.37	27.13	27.86	28.52	29.05	29.55
1.97	21.28	21.74	22.26	22.82	23.31	24.00	24.69	25.40	26.09	26.90	27.50
2.02	21.82	22.32	22.83	23.41	23.77	24.36	24.89	25.33	25.71	26.14	26.47
2.07	22.24	22.84	23.54	24.17	24.82	25.50	26.12	26.83	27.38	27.95	28.50
2.12	21.06	21.52	22.07	22.51	22.94	23.45	24.07	24.56	25.19	25.93	26.40
2.17	21.79	22.37	22.96	23.45	24.10	24.50	25.13	25.60	26.05	26.37	26.76
2.22	21.12	21.59	22.23	22.76	23.36	24.04	24.77	25.56	26.16	26.90	27.53
2.27	22.22	22.59	23.13	23.59	23.97	24.33	24.86	25.40	25.81	26.36	26.73
2.32	21.20	21.74	22.35	22.97	23.52	24.10	24.74	25.36	25.84	26.31	26.74
2.37	20.90	21.36	21.91	22.52	22.96	23.58	24.20	24.86	25.58	26.23	26.78
2.42	21.35	21.90	22.33	22.85	23.26	23.67	24.20	24.66	25.17	25.57	25.99

2.47	21.83	22.40	22.98	23.59	24.15	24.74	25.36	25.99	26.43	27.05	27.40
2.52	20.99	21.46	21.90	22.42	22.82	23.42	23.98	24.66	25.23	25.89	26.33
2.57	21.97	22.54	22.97	23.57	23.99	24.46	25.01	25.44	26.02	26.40	26.79
2.62	21.15	21.67	22.26	22.85	23.38	23.95	24.60	25.30	25.87	26.51	27.00
2.67	20.89	21.32	21.76	22.28	22.67	23.19	23.75	24.21	24.73	25.34	25.83
2.72	21.35	21.90	22.50	23.05	23.62	24.18	24.83	25.39	25.81	26.33	26.74
2.77	20.77	21.14	21.74	22.26	22.67	23.34	23.86	24.47	25.16	25.68	26.19
2.82	20.66	21.09	21.56	22.00	22.35	22.87	23.38	23.86	24.34	24.76	25.22
2.87	20.94	21.45	22.09	22.56	23.06	23.63	24.17	24.66	25.23	25.71	26.20
2.92	20.71	21.11	21.63	22.04	22.59	22.97	23.66	24.22	24.71	25.28	25.80
2.97	20.70	21.14	21.64	22.20	22.58	23.09	23.57	24.09	24.49	24.93	25.29
3.02	21.30	21.86	22.41	22.99	23.52	24.17	24.73	25.34	25.93	26.48	26.86
3.07	20.46	20.91	21.37	21.82	22.28	22.74	23.25	23.72	24.25	24.75	25.22
3.12	20.78	21.22	21.78	22.35	22.82	23.32	23.79	24.31	24.82	25.28	25.66
3.17	20.42	20.79	21.34	21.76	22.18	22.70	23.22	23.79	24.19	24.84	25.22
3.22	21.19	21.59	22.07	22.59	23.05	23.42	24.02	24.48	24.97	25.47	25.89
3.42	20.82	21.33	21.68	22.23	22.67	23.14	23.68	24.20	24.69	25.16	25.58
3.62	21.43	21.79	22.28	22.72	23.12	23.66	24.12	24.62	25.11	25.61	25.89
3.82	20.76	21.26	21.77	22.18	22.67	23.19	23.73	24.30	24.81	25.32	25.78
4.02	21.32	21.73	22.19	22.62	23.04	23.50	23.99	24.40	24.81	25.31	25.69
4.22	20.57	21.04	21.44	21.97	22.33	22.73	23.33	23.73	24.27	24.76	25.12
4.42	20.92	21.32	21.88	22.27	22.70	23.16	23.53	24.23	24.67	25.20	25.49
4.62	20.93	21.20	21.69	22.03	22.44	22.86	23.32	23.79	24.16	24.52	24.81
4.82	20.95	21.33	21.81	22.20	22.64	23.10	23.55	24.04	24.42	24.91	25.27
5.02	20.89	21.20	21.67	22.03	22.45	22.86	23.33	23.76	24.03	24.54	24.81
5.22	21.13	21.49	21.97	22.42	22.84	23.35	23.76	24.30	24.76	25.25	25.63
5.42	21.07	21.38	21.96	22.34	22.81	23.25	23.63	24.09	24.56	25.10	25.32
5.62	21.13	21.53	21.97	22.42	22.79	23.17	23.61	24.06	24.52	24.93	25.27
5.82	21.00	21.38	21.83	22.27	22.67	23.12	23.59	24.04	24.44	24.80	25.23
6.02	20.83	21.20	21.65	21.95	22.36	22.79	23.31	23.75	24.21	24.48	24.92
6.22	20.79	21.14	21.69	22.03	22.48	22.85	23.27	23.73	24.22	24.69	25.03
6.42	20.92	21.25	21.70	22.08	22.53	22.83	23.36	23.81	24.23	24.67	25.05
6.62	21.29	21.64	22.08	22.50	22.85	23.37	23.82	24.22	24.64	25.18	25.51
6.82	20.77	21.16	21.58	21.96	22.32	22.78	23.12	23.52	24.02	24.38	24.73
7.02	21.50	21.90	22.35	22.73	23.09	23.56	24.08	24.50	24.92	25.36	25.65
7.22	20.84	21.15	21.75	22.14	22.41	22.82	23.39	23.80	24.24	24.70	24.97
7.42	21.46	21.85	22.30	22.63	23.06	23.52	24.03	24.40	24.86	25.29	25.60
7.62	20.98	21.30	21.79	22.14	22.50	22.94	23.39	23.78	24.16	24.61	24.97
7.82	21.05	21.39	21.86	22.20	22.45	22.90	23.34	23.78	24.13	24.56	24.82
8.02	20.67	21.04	21.42	21.84	22.21	22.56	23.05	23.37	23.83	24.27	24.54
8.22	21.10	21.48	21.94	22.32	22.69	23.13	23.66	24.13	24.49	24.86	25.16
8.42	20.57	20.87	21.27	21.64	22.06	22.52	23.03	23.36	23.73	24.18	24.51
8.62	21.02	21.34	21.73	22.15	22.52	22.92	23.31	23.77	24.19	24.57	24.94
8.82	21.00	21.32	21.77	22.13	22.51	22.97	23.38	23.79	24.17	24.65	24.89
9.02	20.72	20.99	21.48	21.94	22.25	22.62	23.15	23.61	24.07	24.44	24.80
9.22	20.80	21.24	21.63	22.03	22.38	22.85	23.27	23.68	24.12	24.49	24.88
9.42	20.90	21.25	21.62	22.06	22.38	22.75	23.12	23.60	24.05	24.43	24.71
9.62	20.80	21.15	21.58	21.98	22.31	22.76	23.14	23.63	24.02	24.42	24.70
9.82	21.04	21.38	21.81	22.23	22.64	22.97	23.47	23.91	24.33	24.75	25.11
10.02	20.55	20.92	21.29	21.62	22.01	22.35	22.77	23.10	23.55	23.92	24.23

10.22	20.78	21.12	21.56	21.94	22.25	22.68	23.12	23.52	23.91	24.34	24.65
10.42	21.16	21.49	21.98	22.37	22.77	23.16	23.56	24.08	24.52	24.83	25.22
10.62	20.85	21.27	21.73	22.10	22.41	22.86	23.33	23.78	24.14	24.58	24.82
10.82	20.62	21.05	21.52	21.87	22.30	22.69	23.14	23.55	24.01	24.46	24.73
11.02	20.71	21.14	21.58	21.91	22.23	22.74	23.10	23.57	24.05	24.37	24.60
11.22	20.57	20.86	21.33	21.63	22.01	22.38	22.85	23.25	23.57	24.03	24.33
11.42	20.12	20.40	20.81	21.17	21.54	21.95	22.39	22.74	23.18	23.53	23.83
11.62	21.07	21.47	21.89	22.29	22.72	23.19	23.50	24.09	24.54	24.87	25.15
11.82	20.61	20.92	21.32	21.69	22.08	22.41	22.89	23.27	23.58	24.05	24.36
12.02	20.78	21.13	21.53	21.98	22.37	22.78	23.24	23.65	24.02	24.54	24.84
12.22	21.02	21.38	21.78	22.18	22.57	22.99	23.41	23.98	24.31	24.75	25.07
12.42	20.44	20.82	21.34	21.77	22.04	22.50	22.96	23.37	23.73	24.13	24.57
12.62	20.28	20.64	21.03	21.48	21.73	22.21	22.62	23.02	23.39	23.74	24.06
12.82	20.43	20.67	21.15	21.51	21.83	22.21	22.68	23.10	23.43	23.86	24.19
13.02	20.71	21.07	21.52	22.01	22.33	22.83	23.21	23.68	24.02	24.57	24.81
13.22	20.75	21.17	21.60	21.99	22.37	22.79	23.23	23.58	24.11	24.37	24.74
13.42	20.72	21.06	21.42	21.91	22.24	22.65	23.05	23.53	23.99	24.37	24.75
13.50	20.50	20.78	21.22	21.63	21.90	22.25	22.77	23.16	23.46	23.87	24.17
13.59	20.70	21.13	21.49	21.90	22.30	22.78	23.12	23.65	24.10	24.42	24.82
13.68	20.59	20.98	21.40	21.77	22.07	22.51	22.90	23.32	23.71	24.15	24.39
13.78	20.19	20.58	20.94	21.37	21.70	22.17	22.53	22.88	23.25	23.71	24.02
13.88	20.52	20.94	21.43	21.78	22.10	22.58	22.93	23.42	23.86	24.29	24.49
14.00	20.86	21.22	21.59	21.99	22.35	22.74	23.22	23.59	23.99	24.40	24.68
14.12	20.61	20.90	21.33	21.73	22.10	22.47	22.93	23.38	23.78	24.17	24.41
14.25	20.59	21.02	21.43	21.89	22.19	22.56	23.03	23.48	23.88	24.34	24.64
14.40	20.94	21.34	21.79	22.15	22.50	22.93	23.34	23.79	24.22	24.58	24.91
14.55	20.37	20.70	21.17	21.56	21.85	22.27	22.68	23.08	23.43	23.87	24.21
14.72	20.36	20.74	21.16	21.56	21.91	22.31	22.78	23.19	23.57	24.00	24.28
14.90	20.36	20.65	21.07	21.54	21.85	22.24	22.71	23.10	23.50	23.89	24.24
15.09	20.22	20.52	20.87	21.25	21.60	22.02	22.36	22.78	23.15	23.48	23.82
15.30	20.73	21.09	21.48	21.84	22.22	22.61	23.03	23.49	23.83	24.32	24.46
15.53	20.88	21.24	21.61	22.06	22.34	22.71	23.18	23.52	23.93	24.26	24.62
15.77	20.76	21.13	21.56	21.99	22.22	22.63	23.16	23.60	23.87	24.26	24.61
16.04	20.74	21.13	21.60	21.92	22.33	22.74	23.25	23.61	24.12	24.49	24.87
16.32	20.61	20.93	21.48	21.78	22.11	22.54	22.96	23.41	23.69	24.05	24.39
16.63	20.67	20.90	21.38	21.80	22.15	22.60	22.97	23.40	23.77	24.12	24.43
16.96	20.94	21.34	21.76	22.09	22.48	22.82	23.26	23.68	23.98	24.41	24.65
17.31	20.58	21.00	21.36	21.79	22.10	22.55	22.93	23.38	23.79	24.11	24.48
17.70	21.07	21.47	21.88	22.18	22.53	22.98	23.39	23.70	24.18	24.53	24.80
18.11	20.19	20.59	20.99	21.39	21.69	22.12	22.57	22.93	23.34	23.74	24.05
18.56	20.60	20.92	21.45	21.82	22.16	22.57	23.02	23.45	23.84	24.32	24.62
19.04	20.48	20.82	21.23	21.57	21.89	22.30	22.76	23.11	23.42	23.83	24.16
19.56	20.91	21.24	21.65	22.08	22.41	22.85	23.22	23.65	23.93	24.36	24.61
20.12	20.68	20.96	21.44	21.79	22.12	22.53	22.92	23.32	23.66	24.11	24.35
20.72	20.99	21.40	21.84	22.20	22.57	22.94	23.40	23.83	24.33	24.64	24.95
21.37	20.88	21.16	21.59	21.89	22.27	22.59	23.07	23.49	23.85	24.21	24.46
22.08	21.05	21.42	21.86	22.19	22.63	23.05	23.47	23.95	24.29	24.67	25.07
22.84	21.27	21.64	22.06	22.45	22.79	23.16	23.64	24.06	24.40	24.82	25.01
23.65	20.59	20.91	21.30	21.69	21.98	22.50	22.81	23.30	23.62	24.12	24.35
24.54	20.80	21.09	21.53	21.83	22.08	22.52	22.92	23.25	23.61	23.99	24.21

25.49	21.13	21.43	21.77	22.23	22.54	22.90	23.34	23.66	24.03	24.39	24.76
26.51	20.33	20.68	21.02	21.43	21.73	22.09	22.46	22.83	23.09	23.48	23.80
27.62	20.92	21.25	21.67	22.00	22.28	22.57	23.09	23.36	23.72	24.05	24.26
28.81	21.02	21.45	21.79	22.22	22.47	22.94	23.35	23.76	24.11	24.51	24.75
30.10	20.44	20.72	21.07	21.49	21.76	22.12	22.44	22.85	23.06	23.42	23.80
31.49	21.06	21.45	21.84	22.24	22.55	22.98	23.30	23.76	24.05	24.45	24.71
32.98	20.69	20.96	21.36	21.71	22.08	22.35	22.86	23.13	23.59	23.85	24.22
34.60	21.09	21.36	21.75	22.12	22.39	22.73	23.09	23.42	23.72	24.08	24.30
36.34	20.74	21.01	21.45	21.68	22.03	22.38	22.72	23.04	23.36	23.62	23.91
38.21	21.30	21.62	21.97	22.30	22.66	22.98	23.34	23.72	24.07	24.28	24.61
40.24	21.05	21.36	21.72	21.96	22.40	22.75	23.08	23.45	23.77	24.15	24.34
42.42	20.54	20.83	21.20	21.49	21.86	22.14	22.57	22.90	23.25	23.53	23.78
44.78	20.72	21.04	21.39	21.69	21.94	22.36	22.60	22.89	23.35	23.60	23.81
47.32	20.73	21.09	21.41	21.69	22.00	22.40	22.71	23.10	23.41	23.72	23.92
50.06	21.01	21.24	21.67	21.94	22.22	22.64	22.94	23.31	23.63	23.96	24.20
53.01	20.94	21.24	21.56	21.90	22.27	22.46	22.84	23.13	23.45	23.74	23.92
56.20	20.85	21.11	21.49	21.90	22.08	22.41	22.68	23.05	23.40	23.56	23.81
59.64	21.23	21.47	21.80	22.04	22.35	22.62	22.96	23.25	23.51	23.71	23.86
63.34	20.64	20.83	21.19	21.53	21.80	22.04	22.48	22.78	23.06	23.31	23.53
67.34	20.73	20.88	21.29	21.54	21.82	22.04	22.43	22.72	22.94	23.12	23.42
71.65	21.16	21.24	21.61	21.85	22.03	22.36	22.69	22.94	23.23	23.55	23.60
76.30	20.99	21.31	21.67	21.94	22.15	22.45	22.79	23.04	23.33	23.65	23.78
81.32	21.24	21.47	21.80	22.02	22.32	22.55	22.85	23.19	23.38	23.67	23.90
86.73	21.24	21.51	21.78	22.02	22.20	22.47	22.68	23.07	23.22	23.38	23.65
92.56	21.48	21.61	21.92	22.19	22.38	22.70	22.98	23.19	23.49	23.55	23.76
98.85	20.90	21.02	21.38	21.67	21.85	22.08	22.43	22.65	22.81	23.08	23.24
105.64	21.64	21.83	22.09	22.29	22.65	22.86	23.11	23.40	23.53	23.66	23.84
112.96	21.47	21.68	21.88	22.11	22.29	22.59	22.84	23.04	23.29	23.47	23.49
120.85	21.24	21.44	21.62	21.86	22.08	22.26	22.43	22.72	22.85	23.13	23.22
129.36	21.85	22.03	22.31	22.52	22.72	22.96	23.18	23.50	23.62	23.80	23.94
138.55	21.51	21.67	22.01	22.23	22.39	22.66	22.92	23.15	23.32	23.49	23.64
148.45	21.24	21.42	21.71	21.92	22.12	22.36	22.55	22.80	22.92	23.14	23.30
159.13	21.54	21.71	22.02	22.19	22.40	22.66	22.82	23.02	23.18	23.30	23.35
170.65	21.50	21.60	21.90	22.00	22.10	22.29	22.50	22.66	22.78	22.87	22.97
183.07	21.67	21.79	22.00	22.20	22.34	22.46	22.67	22.81	22.93	22.97	23.06
196.47	21.54	21.63	21.85	22.00	22.05	22.19	22.43	22.49	22.63	22.79	22.81
210.91	21.59	21.74	21.94	22.03	22.10	22.24	22.40	22.43	22.59	22.66	22.62
226.50	21.67	21.74	21.96	22.05	22.11	22.25	22.43	22.56	22.57	22.79	22.78
243.31	21.73	21.83	21.99	22.02	22.20	22.30	22.42	22.54	22.55	22.63	22.69
261.43	22.02	22.14	22.23	22.39	22.39	22.45	22.58	22.71	22.84	22.86	22.83
280.98	21.89	21.93	22.11	22.15	22.26	22.36	22.51	22.55	22.63	22.73	22.71
302.07	21.63	21.72	21.87	21.94	21.91	22.06	22.17	22.23	22.31	22.26	22.24
324.81	21.81	21.82	21.95	21.92	21.98	21.99	22.10	22.15	22.10	22.16	22.15
349.33	21.76	21.72	21.85	21.84	21.88	21.97	21.97	22.01	22.07	22.08	22.00
375.79	21.93	21.96	22.04	22.09	22.11	22.14	22.30	22.31	22.31	22.34	22.26
404.31	21.65	21.61	21.74	21.77	21.82	21.86	21.96	22.05	22.02	22.01	21.99
435.08	21.96	21.92	22.07	22.09	22.07	22.04	22.10	22.08	22.09	22.03	21.88
468.27	21.96	22.00	22.04	22.03	22.00	21.98	22.04	22.07	21.94	22.00	21.86
504.06	22.00	21.81	22.01	21.97	21.91	22.03	21.99	21.95	21.95	21.94	21.78
542.66	21.93	21.89	21.97	21.88	21.86	21.84	21.83	21.77	21.73	21.76	21.63

584.29	21.85	21.79	21.92	21.87	21.84	21.84	21.83	21.85	21.80	21.74	21.67
629.18	21.92	21.84	21.95	21.94	21.84	21.95	21.91	21.98	21.81	21.83	21.73
677.61	22.13	22.03	22.08	22.06	22.07	22.05	22.12	22.07	21.90	21.84	21.77
729.83	21.65	21.56	21.63	21.57	21.46	21.45	21.37	21.34	21.24	21.17	20.89
786.16	21.90	21.87	21.92	21.87	21.81	21.84	21.77	21.77	21.69	21.58	21.54
846.90	21.67	21.64	21.63	21.53	21.44	21.36	21.24	21.31	21.08	21.01	20.81
912.42	21.71	21.61	21.59	21.40	21.34	21.24	21.16	21.03	20.92	20.82	20.66
983.08	21.97	21.84	21.85	21.72	21.70	21.63	21.56	21.51	21.39	21.30	21.09
1059.29	21.89	21.80	21.70	21.67	21.56	21.60	21.47	21.50	21.30	21.19	21.02
1141.48	21.60	21.56	21.41	21.28	21.24	21.09	21.10	20.98	20.80	20.73	20.51
1230.13	21.61	21.49	21.49	21.46	21.39	21.35	21.25	21.26	21.19	21.05	20.98
1325.73	22.00	21.89	21.91	21.84	21.75	21.68	21.69	21.56	21.46	21.33	21.13
1428.84	21.64	21.50	21.45	21.36	21.20	21.22	21.12	21.03	20.91	20.82	20.72
1540.05	21.87	21.64	21.65	21.59	21.50	21.41	21.31	21.20	21.16	20.99	20.90
1659.99	21.54	21.49	21.44	21.48	21.19	21.15	21.05	21.01	20.89	20.86	20.60
1789.34	21.43	21.34	21.22	21.16	20.97	20.94	20.86	20.78	20.61	20.47	20.25
1928.85	21.50	21.30	21.28	21.19	21.08	20.96	20.88	20.79	20.68	20.52	20.31
2079.31	21.31	21.30	21.13	21.09	21.00	20.87	20.81	20.70	20.51	20.44	20.34
2241.59	21.24	21.11	21.10	20.95	20.80	20.77	20.67	20.53	20.45	20.30	20.17
2416.60	21.29	21.23	21.14	21.04	20.92	20.91	20.90	20.89	20.73	20.59	20.46
2605.36	21.17	21.04	20.96	20.94	20.78	20.77	20.61	20.64	20.53	20.44	20.25
2808.94	20.99	20.89	20.82	20.63	20.57	20.47	20.36	20.17	20.07	19.89	19.78
3028.50	20.76	20.54	20.47	20.40	20.23	20.03	20.00	19.90	19.67	19.55	19.27
3265.30	20.80	20.71	20.60	20.58	20.44	20.35	20.24	20.23	20.07	19.95	19.86
3520.69	20.67	20.55	20.56	20.44	20.43	20.32	20.18	20.17	20.00	19.84	19.76
3796.13	20.25	20.22	20.18	20.03	19.94	19.89	19.82	19.70	19.67	19.47	19.38

Wavelength (nm) / Time (ps)	*663.05*	*664.47*	*665.89*	*667.32*	*668.74*	*670.17*
-3.78	-0.01	0.02	-0.05	0.00	-0.11	-0.14
-3.28	-0.11	-0.10	-0.05	-0.09	-0.18	-0.14
-2.78	0.00	-0.02	-0.01	-0.01	-0.01	0.08
-2.28	-0.09	-0.07	-0.04	-0.08	-0.05	-0.03
-1.78	0.20	0.24	0.16	0.27	0.27	0.20
-1.28	0.20	0.21	0.24	0.18	0.29	0.26
-0.78	-0.17	-0.28	-0.19	-0.19	-0.31	-0.27
-0.28	-0.01	0.00	-0.05	-0.08	0.09	0.03
-0.18	-0.09	-0.03	-0.02	-0.01	0.00	0.00
-0.08	-0.01	0.02	-0.11	-0.02	-0.15	-0.12
0.02	-0.07	-0.13	-0.20	-0.12	-0.08	-0.18
0.12	0.05	0.16	0.07	0.04	-0.13	-0.22
0.22	-0.07	-0.10	-0.13	-0.11	-0.17	-0.16
0.27	0.23	0.17	0.20	0.21	0.06	-0.04
0.32	-0.13	-0.01	-0.11	-0.09	-0.17	-0.13
0.37	0.35	0.29	0.35	0.24	0.25	0.27
0.42	0.36	0.24	0.21	0.23	0.18	0.20
0.47	0.14	0.11	0.17	0.02	-0.03	-0.12
0.52	0.02	0.07	0.04	0.10	0.05	0.04

0.57	0.29	0.31	0.20	0.29	0.29	0.30
0.62	0.05	0.09	0.07	0.22	0.05	0.12
0.67	0.20	0.17	0.06	0.12	0.10	0.04
0.72	0.38	0.41	0.35	0.34	0.33	0.30
0.77	0.18	0.15	0.22	0.13	0.12	0.18
0.82	-0.06	-0.06	-0.11	-0.06	-0.04	-0.20
0.87	0.11	0.13	0.13	0.07	0.09	0.07
0.92	-0.20	-0.26	-0.33	-0.24	-0.23	-0.28
0.97	0.40	0.44	0.41	0.39	0.38	0.30
1.02	-0.15	-0.19	-0.18	-0.11	-0.11	-0.21
1.07	-0.22	-0.16	-0.22	-0.17	-0.30	-0.19
1.12	-0.02	-0.08	0.00	0.00	0.01	0.02
1.17	-0.48	-0.57	-0.66	-0.65	-0.65	-0.58
1.22	-0.21	-0.12	-0.06	-0.06	-0.05	-0.09
1.27	-0.15	-0.14	-0.28	-0.17	-0.12	-0.16
1.32	-0.29	-0.44	-0.42	-0.39	-0.33	-0.26
1.37	-7.12	-5.72	-4.64	-3.80	-3.13	-2.41
1.42	19.10	13.94	9.42	5.14	1.38	-1.45
1.47	93.39	99.99	106.20	110.30	112.40	112.79
1.52	33.11	33.66	33.98	33.91	33.53	33.27
1.57	32.90	33.56	34.67	35.76	36.24	36.33
1.62	30.78	29.93	30.07	31.37	33.46	35.18
1.67	31.44	31.86	32.19	32.60	32.85	33.46
1.72	29.10	29.88	30.57	31.29	32.04	32.65
1.77	29.70	29.98	30.21	30.52	30.78	31.20
1.82	29.36	30.09	30.63	31.17	31.59	32.17
1.87	28.05	28.71	29.21	29.76	30.23	30.71
1.92	29.98	30.29	30.69	30.96	31.21	31.43
1.97	28.21	28.88	29.62	30.15	30.70	31.20
2.02	26.83	27.30	27.73	28.16	28.61	29.13
2.07	28.87	29.21	29.54	29.86	29.99	30.37
2.12	27.01	27.58	28.30	28.74	29.30	30.00
2.17	27.07	27.33	27.66	28.01	28.37	28.69
2.22	28.09	28.65	29.18	29.54	30.04	30.54
2.27	27.30	27.58	28.15	28.53	28.86	29.20
2.32	27.15	27.51	27.88	28.27	28.47	28.92
2.37	27.45	27.99	28.56	29.12	29.52	30.02
2.42	26.55	26.98	27.40	27.84	28.21	28.68
2.47	27.82	28.21	28.51	28.69	28.82	29.10
2.52	26.97	27.54	28.06	28.65	29.07	29.69
2.57	27.12	27.45	27.78	28.13	28.26	28.56
2.62	27.44	27.79	28.22	28.59	28.91	29.31
2.67	26.29	26.81	27.30	27.72	28.09	28.53
2.72	27.05	27.47	27.73	28.01	28.14	28.42
2.77	26.81	27.30	27.72	28.26	28.61	28.99
2.82	25.54	26.08	26.50	26.88	27.21	27.68
2.87	26.48	26.87	27.23	27.41	27.60	27.89
2.92	26.33	26.83	27.26	27.72	28.03	28.51
2.97	25.67	26.02	26.43	26.89	27.15	27.48
3.02	27.33	27.65	27.95	28.26	28.25	28.49

3.07	25.64	26.06	26.56	27.13	27.43	27.87
3.12	25.95	26.39	26.75	27.07	27.31	27.63
3.17	25.73	26.11	26.46	26.77	27.06	27.44
3.22	26.30	26.70	27.07	27.43	27.71	28.14
3.42	25.95	26.33	26.79	26.99	27.38	27.73
3.62	26.36	26.79	27.11	27.41	27.67	27.94
3.82	26.14	26.49	27.04	27.32	27.60	28.00
4.02	26.10	26.44	26.76	26.98	27.22	27.45
4.22	25.42	25.83	26.17	26.47	26.80	27.13
4.42	25.98	26.30	26.62	26.97	27.11	27.49
4.62	25.18	25.50	25.92	26.07	26.41	26.70
4.82	25.64	25.91	26.23	26.44	26.60	26.93
5.02	25.16	25.47	25.83	26.17	26.30	26.83
5.22	26.00	26.40	26.74	27.02	27.25	27.46
5.42	25.66	26.05	26.39	26.78	27.08	27.35
5.62	25.65	25.87	26.28	26.54	26.69	26.94
5.82	25.63	25.88	26.20	26.51	26.71	26.92
6.02	25.36	25.69	25.93	26.20	26.50	26.79
6.22	25.36	25.80	26.11	26.44	26.68	27.04
6.42	25.44	25.71	26.14	26.47	26.67	27.10
6.62	25.85	26.20	26.47	26.79	27.04	27.34
6.82	25.01	25.42	25.66	26.13	26.28	26.68
7.02	25.94	26.28	26.60	26.73	26.88	27.01
7.22	25.36	25.71	26.08	26.40	26.62	27.00
7.42	25.96	26.32	26.55	26.88	27.06	27.24
7.62	25.29	25.71	25.95	26.38	26.49	26.79
7.82	25.08	25.31	25.60	25.90	25.95	26.22
8.02	24.96	25.20	25.54	25.90	26.14	26.56
8.22	25.51	25.69	26.00	26.10	26.18	26.11
8.42	24.87	25.17	25.49	25.83	26.04	26.38
8.62	25.23	25.51	25.73	26.03	26.13	26.31
8.82	25.25	25.53	25.75	26.13	26.27	26.55
9.02	25.15	25.53	25.73	26.09	26.32	26.58
9.22	25.18	25.49	25.81	26.13	26.45	26.58
9.42	25.02	25.31	25.55	25.83	26.07	26.43
9.62	25.05	25.45	25.68	25.97	26.15	26.57
9.82	25.45	25.72	26.02	26.22	26.32	26.49
10.02	24.58	24.86	25.15	25.44	25.73	26.03
10.22	24.90	25.29	25.65	25.94	26.20	26.53
10.42	25.57	25.86	26.07	26.28	26.33	26.65
10.62	25.17	25.52	25.81	26.08	26.27	26.52
10.82	25.10	25.48	25.87	26.15	26.39	26.63
11.02	24.96	25.33	25.61	25.81	25.96	26.27
11.22	24.71	25.10	25.22	25.49	25.72	25.96
11.42	24.29	24.42	24.75	25.08	25.40	25.67
11.62	25.51	25.77	25.98	26.17	26.08	26.29
11.82	24.61	24.90	25.26	25.55	25.75	26.09
12.02	25.21	25.48	25.77	26.02	26.10	26.32
12.22	25.37	25.64	26.00	26.18	26.35	26.59
12.42	24.91	25.06	25.58	25.80	25.97	26.26

12.62	24.37	24.77	25.01	25.33	25.56	25.88
12.82	24.51	24.82	25.06	25.28	25.47	25.66
13.02	25.15	25.48	25.89	26.18	26.42	26.78
13.22	25.04	25.28	25.65	25.81	25.92	26.18
13.42	24.92	25.38	25.52	25.83	25.93	26.24
13.50	24.53	24.87	25.07	25.29	25.46	25.74
13.59	25.12	25.48	25.73	26.01	26.14	26.50
13.68	24.58	24.95	25.20	25.48	25.62	25.76
13.78	24.33	24.59	24.95	25.11	25.28	25.72
13.88	24.83	25.13	25.42	25.67	25.78	26.10
14.00	24.98	25.31	25.54	25.73	25.92	26.11
14.12	24.75	25.12	25.46	25.73	26.00	26.26
14.25	24.87	25.07	25.33	25.49	25.64	25.83
14.40	25.24	25.49	25.76	26.06	26.30	26.57
14.55	24.54	24.82	25.13	25.41	25.62	25.91
14.72	24.62	24.90	25.21	25.35	25.57	25.91
14.90	24.54	24.82	25.09	25.37	25.50	25.80
15.09	24.14	24.40	24.65	25.00	25.23	25.49
15.30	24.86	25.01	25.29	25.45	25.35	25.56
15.53	24.88	25.05	25.33	25.63	25.91	26.08
15.77	24.96	25.11	25.42	25.52	25.68	25.90
16.04	25.10	25.44	25.76	25.98	26.15	26.42
16.32	24.71	24.90	25.19	25.32	25.53	25.72
16.63	24.70	25.05	25.35	25.67	25.76	26.09
16.96	25.02	25.20	25.38	25.64	25.77	25.85
17.31	24.77	25.05	25.39	25.62	25.72	26.00
17.70	25.12	25.47	25.65	25.82	25.94	26.25
18.11	24.35	24.72	24.91	25.34	25.48	25.66
18.56	24.87	25.22	25.36	25.64	25.89	26.10
19.04	24.38	24.61	24.87	25.20	25.37	25.66
19.56	24.95	25.27	25.44	25.63	25.59	25.91
20.12	24.75	24.92	25.23	25.50	25.62	26.02
20.72	25.26	25.55	25.80	26.11	26.23	26.39
21.37	24.85	25.17	25.38	25.71	25.78	26.09
22.08	25.43	25.74	26.01	26.27	26.42	26.69
22.84	25.42	25.61	25.85	26.03	26.13	26.17
23.65	24.54	24.88	25.19	25.42	25.60	25.89
24.54	24.45	24.83	24.95	25.25	25.42	25.76
25.49	24.99	25.30	25.48	25.69	25.80	25.97
26.51	24.01	24.30	24.44	24.65	24.78	25.17
27.62	24.59	24.79	25.00	25.20	25.35	25.67
28.81	25.00	25.26	25.47	25.61	25.62	25.80
30.10	24.10	24.27	24.54	24.75	25.00	25.23
31.49	24.99	25.25	25.43	25.57	25.65	25.79
32.98	24.42	24.70	24.85	25.04	25.11	25.34
34.60	24.55	24.74	24.94	25.20	25.35	25.45
36.34	24.23	24.39	24.51	24.72	24.83	25.22
38.21	24.80	25.09	25.17	25.32	25.39	25.63
40.24	24.64	24.83	25.03	25.31	25.39	25.55
42.42	24.10	24.28	24.51	24.76	24.93	25.04

44.78	24.06	24.25	24.49	24.62	24.56	24.76
47.32	24.13	24.33	24.50	24.59	24.66	24.68
50.06	24.37	24.63	24.75	24.89	24.98	25.08
53.01	24.06	24.29	24.45	24.57	24.63	24.85
56.20	24.06	24.21	24.47	24.55	24.75	24.85
59.64	24.02	24.20	24.35	24.45	24.57	24.70
63.34	23.77	23.96	24.09	24.20	24.27	24.34
67.34	23.53	23.82	23.82	23.95	24.04	24.12
71.65	23.80	23.99	24.11	24.28	24.37	24.60
76.30	23.97	24.16	24.23	24.40	24.35	24.53
81.32	24.09	24.17	24.35	24.35	24.49	24.76
86.73	23.78	23.91	24.08	24.23	24.22	24.50
92.56	23.96	24.00	24.04	23.99	24.03	24.13
98.85	23.42	23.61	23.68	23.84	23.88	24.02
105.64	23.92	24.02	24.02	23.91	23.94	23.91
112.96	23.69	23.82	23.85	23.88	23.95	24.02
120.85	23.27	23.31	23.45	23.51	23.44	23.52
129.36	24.10	24.18	24.25	24.13	24.15	24.19
138.55	23.79	23.82	23.85	23.88	23.88	24.00
148.45	23.42	23.53	23.60	23.66	23.66	23.81
159.13	23.49	23.53	23.48	23.37	23.26	23.33
170.65	23.08	23.13	23.07	23.14	23.07	23.25
183.07	23.13	23.15	23.23	23.23	23.12	23.26
196.47	22.86	22.83	22.79	22.80	22.65	22.66
210.91	22.70	22.82	22.71	22.67	22.55	22.57
226.50	22.81	22.85	22.86	22.67	22.69	22.56
243.31	22.59	22.60	22.54	22.40	22.28	22.17
261.43	22.88	22.81	22.72	22.59	22.57	22.55
280.98	22.68	22.78	22.67	22.45	22.41	22.27
302.07	22.21	22.20	22.12	21.92	21.79	21.75
324.81	22.20	22.13	22.03	22.05	21.86	21.89
349.33	21.98	21.89	21.80	21.61	21.47	21.33
375.79	22.24	22.18	22.07	21.90	21.71	21.71
404.31	21.97	21.91	21.89	21.73	21.42	21.59
435.08	21.83	21.75	21.70	21.54	21.50	21.51
468.27	21.77	21.68	21.49	21.31	21.08	21.05
504.06	21.70	21.60	21.51	21.31	21.24	21.12
542.66	21.48	21.31	21.12	20.95	20.70	20.53
584.29	21.57	21.55	21.33	21.19	21.13	21.07
629.18	21.65	21.50	21.38	21.19	20.95	20.83
677.61	21.67	21.48	21.33	21.15	20.88	20.82
729.83	20.84	20.74	20.54	20.31	20.14	20.07
786.16	21.46	21.40	21.24	21.07	20.80	20.72
846.90	20.69	20.55	20.42	20.18	19.93	19.80
912.42	20.43	20.29	20.16	19.95	19.72	19.64
983.08	21.10	20.81	20.64	20.36	20.09	19.93
1059.29	20.97	20.81	20.55	20.36	20.19	20.06
1141.48	20.35	20.22	20.07	19.85	19.68	19.61
1230.13	20.82	20.62	20.53	20.32	20.21	20.06
1325.73	20.98	20.76	20.58	20.30	19.98	19.76

1428.84	20.54	20.34	20.23	19.95	19.77	19.66
1540.05	20.71	20.50	20.39	20.18	19.89	19.74
1659.99	20.46	20.34	20.16	19.96	19.82	19.75
1789.34	20.09	19.94	19.77	19.63	19.41	19.26
1928.85	20.25	20.02	19.83	19.59	19.37	19.27
2079.31	20.26	20.07	19.78	19.73	19.45	19.45
2241.59	20.07	19.87	19.67	19.52	19.29	19.22
2416.60	20.38	20.19	20.14	19.91	19.69	19.57
2605.36	20.23	20.07	19.93	19.68	19.57	19.42
2808.94	19.61	19.39	19.27	19.08	18.94	18.76
3028.50	19.14	18.94	18.76	18.53	18.21	18.27
3265.30	19.72	19.56	19.42	19.24	19.19	18.92
3520.69	19.61	19.39	19.15	18.91	18.57	18.47
3796.13	19.11	19.04	18.85	18.71	18.54	18.39

Table S8. Raw data of fs-TA signals of A-RC-A collected under VIS (λ_{ex} = 513 nm) excitation in the λ_{pr} = 318–670 nm regime, the unit of ΔA in the table is mOD..

Wavelength (nm) / Time (ps)	318.42	319.85	321.27	322.70	324.12	325.54	326.97	328.39	329.82	331.24	332.67
-2.74	-0.57	0.14	0.08	-0.76	0.32	-1.01	1.54	-0.66	0.38	-0.50	0.19
-2.24	-0.50	0.44	-1.64	0.48	-0.92	-0.82	-1.05	-0.34	-0.82	1.02	-0.47
-1.74	1.18	-0.38	0.41	-0.09	-1.11	0.95	-0.22	0.94	-0.71	-1.52	0.63
-1.24	-0.34	-1.29	-0.26	1.68	0.48	-0.16	-0.65	0.03	0.42	1.19	-0.51
-0.74	1.47	1.74	0.76	-0.99	-0.20	-0.16	0.81	0.95	0.98	-0.52	0.78
-0.24	-2.15	-0.29	0.99	-1.48	0.10	0.10	0.61	0.63	-0.74	-0.72	-0.56
-0.14	0.72	-0.28	0.18	-0.03	1.00	1.04	-0.44	-0.13	1.26	1.18	0.23
-0.04	0.19	-0.07	-0.53	1.19	0.32	0.06	-0.60	-1.41	-0.78	-0.13	-0.27
0.06	-1.27	-0.99	-0.69	-0.71	-0.73	-1.07	0.98	1.19	0.17	-0.37	1.73
0.16	-0.48	-1.06	-0.62	0.08	0.22	0.78	0.10	1.08	0.79	-0.82	2.19
0.26	-0.22	-0.02	-0.36	2.01	-1.39	0.94	1.32	-0.74	0.34	-0.81	-0.52
0.31	1.90	0.50	-0.52	-1.46	-3.06	0.75	-0.13	0.68	0.43	-1.45	0.55
0.36	-1.76	0.18	0.41	0.01	0.28	0.76	-3.76	-4.32	-4.31	-3.03	-0.83
0.41	2.46	1.03	-0.85	2.39	1.83	5.46	5.85	3.81	-2.32	-8.97	-14.76
0.46	-0.65	-0.72	0.80	0.08	0.77	4.81	7.64	15.38	21.10	31.34	36.27
0.51	0.15	0.37	-0.36	-1.52	-5.26	-5.17	-5.56	-1.13	2.81	16.64	42.17
0.56	-1.34	-0.13	0.26	-1.53	0.75	-1.01	-4.20	-9.24	-19.90	-30.95	-25.30
0.61	-1.00	0.28	-0.60	-0.60	0.44	1.11	1.54	0.64	-1.36	-9.10	-25.45
0.66	0.62	-0.99	0.25	-0.77	-0.24	0.52	0.54	0.99	1.97	0.50	2.83
0.71	-0.37	-1.43	-0.36	-0.04	0.64	1.91	-0.48	1.44	0.11	1.62	2.15
0.76	0.33	0.20	1.96	-1.36	-1.80	1.09	0.77	2.40	0.32	0.15	1.58
0.81	-0.67	2.11	0.50	0.99	-0.30	-1.06	0.59	0.53	0.80	0.10	0.25
0.86	-1.60	0.91	-0.92	0.01	0.58	-0.17	0.19	-0.08	0.65	0.07	1.10
0.91	0.83	1.28	-0.52	-1.21	0.18	0.23	0.57	2.22	1.53	-0.30	1.94
0.96	-0.38	-0.29	-0.45	-0.36	-0.94	-2.27	1.62	1.35	-0.95	0.58	0.40
1.01	-0.52	1.61	-0.98	1.02	-0.83	1.41	-0.23	2.39	2.36	0.34	0.67
1.06	0.34	-0.37	0.61	0.93	-0.08	1.83	0.38	0.86	0.86	1.16	2.45
1.11	-1.78	-1.75	-1.07	0.22	-0.38	1.59	0.75	1.75	1.05	0.59	0.93
1.16	-0.69	1.43	-0.63	-0.94	-0.32	0.10	2.09	0.38	0.61	-0.21	0.87

1.21	-0.85	2.34	1.39	-0.67	0.30	1.64	2.84	1.55	-0.25	0.01	1.18
1.26	-0.26	-0.68	0.25	-0.39	0.85	0.34	-0.14	1.33	1.80	-0.05	1.95
1.31	0.68	-0.55	2.26	-0.43	-0.37	-0.06	0.26	1.57	0.77	0.53	0.78
1.36	1.91	-0.07	0.92	0.18	-0.10	1.58	0.71	1.68	0.09	0.04	1.62
1.41	-2.12	0.61	-0.29	-0.63	-0.99	1.35	-1.40	1.00	1.29	-0.56	1.40
1.46	3.25	0.97	1.29	0.80	-0.12	0.33	0.14	0.28	0.93	-0.83	0.23
1.51	1.11	1.37	-0.45	-1.02	0.34	0.76	0.25	0.20	1.57	-1.15	1.25
1.56	-1.61	2.05	0.74	-0.63	1.83	1.05	-0.94	2.02	-0.25	1.95	2.36
1.61	0.25	1.16	-0.78	-0.09	0.33	0.73	1.17	1.95	0.40	0.11	0.77
1.66	1.38	-1.02	-2.42	0.58	-0.62	-1.80	-0.40	0.43	-0.24	-0.72	1.73
1.71	1.53	0.84	2.06	0.45	-1.28	1.18	0.04	0.26	0.45	0.82	0.60
1.76	-0.21	0.01	1.29	-1.52	1.53	0.46	0.08	0.22	1.27	0.76	0.96
1.81	-0.15	-0.82	0.49	-1.35	0.48	-1.07	0.58	1.41	-0.67	-0.38	0.62
1.86	1.31	0.65	-1.19	1.35	-1.37	-0.20	0.12	2.89	1.02	1.02	0.63
1.91	0.25	2.39	-0.51	0.33	-0.07	0.30	1.03	1.59	0.86	0.85	1.79
1.96	-0.36	0.11	-0.10	-0.57	0.46	2.00	-0.29	0.34	2.10	-0.57	0.57
2.01	0.32	1.09	-1.30	-0.81	-0.58	-0.20	-0.84	0.19	0.21	0.33	0.50
2.06	0.13	0.31	0.72	1.39	0.74	0.72	0.61	0.45	0.58	0.63	1.63
2.11	-0.50	-2.31	2.22	-0.23	-2.88	0.26	2.23	-1.84	-0.44	1.43	1.21
2.16	-1.48	-0.18	1.75	-0.94	-0.06	1.84	-0.35	-0.53	0.62	1.18	0.71
2.21	-0.69	2.13	-1.60	0.84	0.43	-0.75	0.25	2.16	1.20	-1.17	1.31
2.26	0.90	-0.28	1.24	0.65	0.07	0.14	0.65	2.57	0.13	-0.11	1.38
2.31	1.00	1.24	-0.93	-0.86	0.34	-0.26	0.46	1.39	-0.43	-1.76	0.74
2.36	-0.49	2.25	-2.95	-0.53	2.41	0.58	-1.83	1.17	2.76	-0.40	-0.14
2.41	0.71	1.75	0.32	1.02	-2.67	0.47	0.97	-0.68	0.51	-0.16	1.22
2.46	0.79	-0.89	0.64	1.73	-0.41	-1.72	3.00	1.81	0.06	0.07	1.85
2.51	-0.88	1.91	0.42	-2.66	1.87	2.79	0.05	0.24	0.85	-0.07	-1.85
2.56	-0.60	3.66	0.28	-2.37	1.18	1.22	-2.07	-0.82	1.07	-0.17	-0.05
2.61	-0.13	-0.07	0.58	-0.25	-1.80	-0.18	-1.18	0.69	0.18	2.28	0.33
2.66	-0.58	-2.06	1.87	-0.39	-0.38	1.60	-0.03	1.33	-0.36	-0.78	0.19
2.71	2.07	-1.36	-0.37	0.19	-0.68	-0.86	0.79	3.02	-0.15	-1.28	2.02
2.76	-0.30	-2.11	0.70	1.58	-1.49	0.89	1.20	1.49	-0.76	0.76	0.82
2.81	0.92	0.16	0.65	0.09	0.89	1.40	3.43	2.96	-2.32	-0.76	2.68
2.86	2.28	0.58	-0.37	1.26	-0.30	-0.68	-0.35	0.01	1.36	-0.53	1.37
2.91	1.29	0.70	-1.44	-0.51	0.53	0.45	-0.19	2.54	2.35	-1.25	1.01
2.96	-1.17	2.72	-1.10	-1.51	-0.18	1.75	-0.37	0.37	0.99	0.41	0.08
3.01	1.19	0.38	-0.26	0.02	-1.34	-0.29	-0.27	2.07	-0.63	-0.51	0.29
3.06	0.11	0.98	0.82	-1.34	-0.96	-0.29	0.40	0.52	1.31	-1.25	1.20
3.11	0.48	1.29	1.62	-0.56	0.37	-0.29	0.14	-0.07	-0.54	-0.72	1.90
3.16	1.34	-0.24	-1.39	0.02	1.39	-0.91	0.81	1.34	0.97	-0.26	0.40
3.21	-0.22	-1.83	1.04	-0.06	-1.37	0.76	1.24	1.44	0.71	0.32	1.80
3.26	0.54	-1.56	-0.63	1.34	0.45	0.39	0.70	1.39	1.73	0.02	1.47
3.31	1.12	1.91	0.29	1.03	-0.54	1.58	0.82	1.03	0.27	0.24	-0.11
3.36	0.21	0.87	0.48	-1.18	-0.35	0.73	0.52	-1.09	0.72	0.57	-0.93
3.41	0.76	-1.24	0.77	-1.06	-1.81	1.33	-0.03	0.36	0.94	-0.46	0.49
3.46	-1.85	0.01	0.31	0.94	0.55	1.34	1.19	0.39	1.02	-0.63	-0.61
3.51	-0.12	3.06	0.09	0.20	-2.65	1.18	0.81	2.50	1.28	0.01	0.15
3.56	0.64	-0.73	-0.93	-2.67	0.32	2.03	-1.02	2.26	0.21	-0.11	0.71
3.61	1.62	-1.51	-1.13	2.68	1.77	-2.37	2.67	0.93	-1.26	-0.60	1.38
3.66	0.07	0.69	-0.04	-1.74	-1.52	-0.49	1.34	1.33	-0.65	-1.12	1.01

3.71	-0.16	-1.45	-0.13	-0.70	-0.37	2.40	-2.50	-0.66	1.81	0.55	1.53
3.76	0.65	-0.51	-0.24	-1.84	1.02	0.15	1.26	0.54	0.45	-0.03	1.11
3.81	-3.87	1.10	1.21	-1.41	-1.33	2.01	-0.14	-0.84	2.50	0.44	-0.32
3.86	-0.26	0.62	-0.63	-1.40	0.41	-0.23	1.94	0.77	1.19	1.40	0.88
3.91	-0.79	-0.19	0.68	-0.11	-0.92	0.50	0.26	0.95	-0.21	-0.53	-0.34
3.96	-0.45	2.89	-0.41	-3.76	3.29	1.03	-1.93	2.53	3.91	-1.46	-0.94
4.01	0.18	0.35	0.30	1.57	-1.71	-0.17	-1.36	0.60	-0.03	-0.13	0.44
4.06	1.34	0.77	-0.16	-0.83	-0.53	-0.24	-1.76	0.70	0.98	0.12	-0.48
4.11	-1.62	0.06	0.10	-1.40	1.63	2.22	-0.62	0.62	1.39	-0.99	0.25
4.16	-2.03	-1.56	2.10	-0.61	-0.27	2.26	1.66	-2.00	1.64	3.04	0.13
4.21	-0.79	-0.45	1.77	-0.79	-1.12	-0.39	0.22	0.57	-0.58	-1.01	0.78
4.26	1.48	-1.71	1.13	2.10	-0.95	0.99	0.18	-0.08	1.88	0.73	0.96
4.31	0.21	1.09	-1.32	0.09	0.58	0.47	1.43	0.96	0.85	0.22	1.17
4.36	-2.15	0.10	-1.26	-0.18	0.69	0.85	-0.52	0.50	0.95	-0.06	0.33
4.41	-0.59	-0.20	0.28	-0.49	1.12	-0.53	-0.40	1.93	1.33	-0.51	-0.91
4.46	-0.23	-0.70	0.10	0.24	1.15	-1.23	-0.65	-1.30	1.51	0.59	0.74
4.51	0.42	-0.87	-0.37	-1.58	1.05	-1.00	0.01	1.14	0.01	0.43	0.96
4.56	0.05	1.19	0.90	-0.70	-0.09	1.35	0.42	1.21	0.58	-0.34	-0.21
4.61	-0.84	0.22	-1.92	-1.55	2.19	1.70	1.12	1.03	1.32	-0.45	-0.09
4.66	-1.19	-1.22	-0.33	0.80	-0.87	0.40	0.50	-0.49	0.29	0.63	-0.06
4.71	-0.83	-0.58	-1.50	-0.82	-0.04	-0.11	1.12	0.67	1.30	-0.21	-0.26
4.76	-1.40	2.74	-0.96	-0.25	0.92	0.51	0.76	1.28	1.57	1.16	1.03
4.81	1.71	-0.49	-0.53	-0.73	1.68	1.00	1.91	2.89	0.78	-0.63	2.17
4.86	0.14	2.26	1.19	0.55	-0.46	0.56	-0.05	0.24	1.86	-0.87	0.78
4.91	0.22	0.28	1.13	0.37	-0.37	-0.80	-0.08	2.30	-0.61	0.10	0.37
4.96	-0.71	1.37	-0.56	-1.01	0.11	0.77	0.43	0.56	1.01	0.28	0.28
5.01	-0.41	-0.98	1.30	-0.17	0.05	0.50	1.04	1.19	-0.96	0.22	2.57
5.06	-0.13	-0.17	-1.25	0.01	0.02	0.06	-0.11	1.58	0.25	-2.05	0.55
5.11	0.11	1.21	0.12	-0.24	0.92	0.90	-0.50	-0.90	1.56	-0.44	-0.04
5.16	1.06	1.10	0.21	1.63	0.43	-1.70	0.44	0.43	0.47	-0.79	0.83
5.21	-1.29	1.62	-0.96	-0.09	-0.97	-0.08	0.31	0.42	1.42	0.05	0.51
5.26	0.67	-1.94	0.04	1.08	-0.28	-0.73	1.07	2.71	-0.79	-0.11	0.52
5.31	-0.43	-0.63	-0.61	-0.04	0.39	1.10	0.41	-1.58	0.51	-0.59	-0.71
5.38	-0.06	1.69	0.04	1.04	1.70	1.61	0.02	0.80	0.11	0.76	-0.17
5.45	1.99	-0.63	1.09	0.49	-0.36	0.22	1.51	-0.16	0.24	0.11	0.62
5.53	0.87	1.38	-1.39	-0.98	1.06	0.49	0.55	0.45	0.15	-0.72	0.67
5.61	-0.44	-0.49	-1.16	0.94	-1.64	1.90	1.79	0.44	1.17	0.40	1.45
5.70	0.25	1.64	0.57	-1.37	-0.08	0.89	-0.23	-0.57	1.99	-1.22	0.59
5.80	0.07	0.14	1.34	0.73	0.51	0.36	-1.24	1.28	1.10	0.12	-0.48
5.90	0.43	-0.35	-0.47	1.09	-2.11	-0.52	1.91	1.19	-0.56	1.07	1.94
6.01	0.32	0.03	-1.01	0.15	-0.09	-0.10	0.06	0.84	0.27	-0.64	1.17
6.13	1.66	0.54	-0.29	0.57	-0.51	0.85	0.67	0.81	0.02	0.44	0.29
6.25	-1.01	0.62	-1.47	0.06	-0.40	-0.03	-0.39	1.47	0.23	-0.69	0.32
6.39	-0.24	1.82	-0.20	0.68	-1.08	0.34	0.12	0.80	1.32	-2.40	0.51
6.53	0.68	1.52	-0.99	0.50	-1.33	1.81	-0.33	0.75	0.83	-0.58	0.10
6.69	0.89	0.59	0.34	-0.20	-0.93	-0.72	1.61	-0.46	0.47	0.09	1.55
6.85	-0.65	1.11	-0.03	1.48	-0.35	-0.26	0.54	2.14	-1.54	-0.07	0.57
7.03	0.50	2.48	0.27	1.00	0.09	0.29	-0.42	0.98	1.39	0.21	-0.57
7.21	1.07	0.66	-0.50	-0.28	-2.32	1.12	0.70	2.07	-1.06	-1.35	-0.08
7.41	-1.61	1.83	-1.09	-0.27	-1.62	1.73	0.66	2.19	1.83	-1.58	-0.19

7.63	1.01	0.81	1.07	-1.37	1.32	1.10	2.38	0.36	0.16	0.38	1.08
7.86	0.07	-2.00	1.16	0.28	-1.37	1.07	2.15	0.13	1.14	-0.22	1.78
8.10	0.47	0.63	0.50	-1.98	-1.63	1.16	-0.03	0.47	0.95	1.79	0.30
8.36	0.42	0.09	-0.44	0.99	-0.18	1.81	-0.76	1.68	0.59	0.50	0.08
8.64	0.19	-1.01	-0.71	0.84	0.67	-1.14	0.91	1.14	0.21	0.57	-0.15
8.94	-0.57	-0.25	-0.92	1.48	1.99	-1.47	1.67	0.68	-0.07	0.64	0.87
9.26	0.43	-0.46	-0.98	0.76	-0.01	-1.78	1.07	1.30	0.12	0.31	2.02
9.60	-0.15	-0.46	1.29	1.63	0.00	-1.68	1.33	-0.60	0.23	1.70	-0.03
9.97	-0.78	-0.83	-0.30	0.07	-1.61	1.75	0.52	0.40	0.95	1.38	-0.37
10.36	-0.27	2.07	0.50	-1.91	-0.46	0.79	-0.39	2.33	-0.24	-0.67	0.64
10.77	-0.87	-0.32	0.75	0.29	0.23	-0.25	0.39	0.52	1.03	-0.30	0.71
11.22	0.30	0.43	-1.59	-0.13	-0.46	0.36	1.05	0.77	0.39	-0.60	1.23
11.70	-0.30	0.27	-0.08	-0.49	0.76	0.04	0.44	0.10	0.00	-1.38	0.68
12.21	-1.29	-1.87	1.77	-0.12	-2.11	1.66	1.61	-0.62	-1.43	1.94	0.20
12.75	1.84	-0.43	-1.20	1.16	-0.61	-0.37	-0.44	0.70	-0.31	-0.41	2.28
13.33	-0.39	-1.39	0.33	0.40	0.03	0.17	0.00	1.39	1.59	0.61	0.06
13.95	0.56	1.32	-2.04	-0.75	-0.23	-0.94	1.02	1.58	0.46	-0.32	-1.45
14.62	2.85	-0.54	0.03	-1.28	0.83	-0.66	0.20	0.54	-0.52	1.11	0.06
15.33	-0.21	-0.37	0.96	0.01	0.08	1.52	0.18	0.71	0.79	-0.91	0.34
16.09	0.20	0.26	-0.59	0.26	-1.08	-1.45	-1.07	-0.10	0.15	-1.07	1.35
16.90	0.62	-0.85	1.05	-0.77	0.08	-0.21	-0.39	1.85	1.36	0.47	-0.16
17.77	1.14	-0.30	0.03	0.76	-0.51	0.66	1.57	0.75	-0.21	-2.33	1.02
18.70	1.63	0.76	-0.01	1.37	-0.28	-1.34	1.80	-0.67	0.03	-0.06	0.56
19.69	-1.10	0.55	-0.96	0.23	0.63	-0.51	0.17	-0.78	-0.45	0.33	1.68
20.75	0.62	-0.04	0.65	1.11	0.96	0.47	-0.17	-0.17	1.90	0.04	-0.17
21.89	0.92	0.59	1.46	0.19	-0.25	0.41	1.56	0.83	-0.95	-1.19	0.52
23.10	1.15	0.74	0.91	-0.69	-1.38	-2.05	-1.47	-0.29	1.75	-1.83	0.47
24.39	-0.34	-0.51	0.57	-0.46	0.77	1.39	-0.73	0.40	1.05	-0.42	-1.61
25.78	0.92	-1.31	0.58	-0.48	-0.28	-0.80	1.11	1.36	0.99	-1.08	0.16
27.26	-1.32	-0.86	0.31	0.18	-1.39	0.63	-0.30	1.74	1.08	0.73	1.41
28.84	-1.34	-0.42	1.97	-0.24	0.27	0.06	-1.18	-0.72	0.90	-0.79	1.76
30.53	0.06	-0.48	-2.58	-1.12	0.75	1.75	2.79	-0.15	-0.02	-0.18	0.18
32.34	0.00	-0.33	0.21	0.18	0.10	1.13	0.45	1.64	-0.18	1.63	-0.28
34.27	0.10	2.50	-0.91	-0.01	-0.45	-1.15	0.82	-0.60	0.89	-0.65	1.24
36.34	-0.36	0.13	0.13	0.37	-0.16	0.71	1.36	1.44	-1.62	-0.66	0.18
38.55	0.07	1.83	0.68	1.20	0.80	-0.22	1.80	0.45	1.43	0.50	0.01
40.91	-0.18	-0.37	-0.40	-1.13	0.10	0.43	1.46	0.11	0.40	0.11	0.33
43.43	-0.46	-0.80	0.35	0.66	0.03	-0.27	0.11	0.64	-0.41	-0.51	1.93
46.13	0.21	0.47	2.03	1.78	-0.20	0.00	1.46	1.57	1.17	-1.35	0.54
49.01	1.82	1.07	-2.40	-0.36	0.36	2.16	-0.70	0.19	0.26	-0.78	-1.27
52.09	0.56	-1.22	1.33	0.48	0.98	-0.67	-2.19	0.99	0.14	-0.24	-0.27
55.39	-1.08	0.12	-0.04	-0.97	0.13	-1.75	-0.20	0.15	0.89	-1.08	1.97
58.91	0.32	-1.01	0.11	0.37	-0.62	-0.19	-0.55	0.74	0.45	-0.07	0.81
62.68	-0.70	-0.95	0.84	0.91	0.58	1.36	1.85	0.47	0.45	0.14	-0.34
66.70	0.37	-0.35	-0.35	-1.00	-0.32	-0.16	-0.54	1.53	1.57	0.74	0.93
71.00	-1.25	-0.16	-0.29	-0.25	0.89	-0.80	0.24	-1.25	1.32	-1.59	0.17
75.60	-1.67	0.41	-0.42	-0.66	-0.65	-1.61	2.70	2.21	0.79	0.33	0.14
80.51	1.05	0.33	-0.39	0.60	-0.31	0.01	-1.18	1.51	-0.30	-1.29	0.85
85.77	0.91	2.03	0.92	-1.14	-1.04	0.09	0.01	0.97	1.31	-0.18	-0.10
91.38	0.08	0.25	-1.35	2.13	0.63	1.48	1.05	-1.16	2.02	-0.71	-0.12

97.39	0.10	0.68	-0.55	-0.58	-0.07	3.27	0.27	1.18	0.46	0.09	1.44
103.80	0.05	-1.04	0.96	-0.61	0.38	1.12	0.50	-0.32	1.03	-0.52	0.29
110.66	-0.74	1.64	-1.58	0.44	-0.25	-0.64	2.01	-0.37	0.26	-2.72	-0.06
117.99	0.50	1.00	-1.29	0.82	0.53	-0.24	-0.70	1.19	1.13	-1.22	0.89
125.83	1.57	1.04	0.21	1.26	1.02	-0.74	-0.54	0.64	-0.03	0.92	1.57
134.21	1.25	-0.17	-1.34	0.64	-0.55	-0.97	0.95	1.04	1.25	-1.33	0.64
143.17	0.46	0.07	0.81	-1.39	0.91	0.57	-1.06	-1.93	0.79	-0.30	1.15
152.74	-1.19	0.35	-1.94	-2.18	0.82	0.17	-1.05	1.28	1.20	-1.86	0.15
162.97	-0.46	-1.81	3.09	1.76	-0.93	0.94	2.06	-0.39	-1.09	-1.36	0.82
173.91	-0.23	-0.66	0.51	0.66	0.24	0.77	-1.51	0.35	0.81	-0.03	0.07
185.60	0.45	-0.05	0.90	-0.23	1.53	0.97	-0.58	0.14	2.02	-1.79	1.13
198.10	-0.37	1.12	0.41	0.58	-2.73	-0.01	-1.31	-0.72	0.32	-0.48	1.23
211.46	2.09	-1.44	0.57	0.18	-0.39	-1.69	1.88	-0.05	-1.27	-0.86	1.23
225.75	-1.77	-0.56	1.59	1.20	1.93	-0.09	0.62	0.35	0.83	-1.40	-0.83
241.01	1.06	-1.52	-0.23	-1.06	-1.64	0.26	1.11	0.69	0.97	0.83	0.83
257.33	0.17	1.80	-0.84	-0.13	0.72	1.85	0.87	-0.49	0.98	-0.75	0.68
274.77	0.82	1.32	-0.97	0.01	0.12	0.61	0.36	-0.73	-0.84	-0.14	0.12
293.42	1.16	0.34	0.63	-1.97	0.54	0.25	-1.18	1.32	-0.24	-1.41	-1.00
313.35	-0.81	0.46	0.89	-0.83	-0.12	2.40	1.78	0.38	1.06	1.51	-1.15
334.66	-0.38	-0.12	1.40	-0.29	-1.85	1.58	1.56	0.07	-2.08	1.33	1.23
357.43	0.58	0.58	-1.64	-0.21	-1.42	-1.22	0.03	0.93	1.03	-0.51	1.04
381.78	-1.02	-0.06	-0.23	-1.09	0.12	0.46	0.68	-1.20	1.51	-1.68	0.21
407.80	0.67	-1.11	0.14	2.47	-1.64	-2.34	1.82	3.14	0.18	-0.29	2.67
435.62	-0.43	0.61	1.70	-0.75	-1.84	-0.95	0.98	-0.02	1.58	1.63	-0.66
465.35	1.84	-1.70	-1.33	1.00	-0.54	-1.06	0.52	0.58	-0.13	1.23	1.48
497.14	0.15	1.54	-1.02	0.15	1.84	0.93	-0.64	0.41	2.14	-1.48	0.75
531.11	-0.85	1.13	-0.72	-1.24	-0.25	0.39	-0.32	-1.17	1.74	-0.80	0.22
567.43	0.85	0.78	-0.62	-1.00	-0.86	0.84	0.68	0.57	-0.88	-1.46	1.27
606.26	0.83	-0.62	0.14	0.55	0.82	0.77	0.36	1.37	1.03	0.27	0.44
647.76	1.61	0.35	-1.63	0.58	-0.13	1.45	0.06	0.87	0.12	-1.21	1.85
692.12	0.03	-2.23	0.21	1.94	1.72	2.39	-2.36	1.14	-0.77	1.02	-0.02
739.53	-1.29	-1.40	0.28	-1.00	0.48	2.46	0.21	-0.46	2.05	-0.85	0.84
790.22	1.56	0.54	0.86	1.06	-0.41	0.96	-0.15	0.91	1.31	0.56	1.46
844.40	-0.59	-0.90	-1.46	3.09	-1.12	0.19	1.27	2.37	-0.94	0.42	2.30
902.32	1.21	0.80	2.03	-1.46	-0.28	-0.40	3.32	-0.34	0.18	0.85	2.41
964.23	-0.51	1.17	0.48	-0.23	-0.84	1.24	-0.80	-0.02	0.79	-0.05	-0.75
1030.41	2.30	-1.14	0.35	0.47	-0.69	-2.32	1.19	2.19	-1.54	-1.76	1.83
1101.15	1.81	-1.27	-0.14	-0.19	-0.23	1.03	-2.25	1.65	0.67	0.66	0.08
1176.77	0.90	-1.61	0.65	-0.65	0.14	1.89	-0.20	1.19	0.15	0.04	0.20
1257.60	0.04	3.10	-1.00	-0.90	1.45	-0.08	-1.82	1.35	2.17	-1.32	-0.59
1344.00	-0.40	0.18	0.62	-2.16	0.46	-0.25	0.74	1.86	0.39	-0.12	-0.38
1436.36	0.39	-0.27	1.82	0.43	-1.71	0.82	0.05	0.58	-0.43	0.36	0.74
1535.09	1.28	-0.71	-0.94	-1.02	0.82	0.17	0.77	2.04	-2.02	0.14	0.67
1640.63	0.92	0.46	2.47	-0.91	-0.26	1.77	1.70	-1.66	-0.60	-0.18	0.36
1753.44	0.14	1.13	-0.78	-1.95	-0.93	0.15	-0.15	0.48	0.60	-1.99	0.92
1874.02	0.21	-0.82	-0.42	0.61	0.61	0.37	-0.45	1.49	0.64	-0.18	1.30
2002.92	-2.17	-0.01	0.21	-1.05	0.59	1.08	-0.72	0.70	0.84	0.04	-0.93
2140.71	0.64	1.03	-0.74	-0.79	-0.87	-0.12	0.27	0.59	0.24	-0.23	0.72
2288.00	-0.84	-1.53	1.19	-0.14	0.42	-0.15	-0.49	-0.41	-0.28	-0.53	1.52
2445.44	-0.91	-0.56	0.72	-2.01	-1.12	1.44	0.73	-1.51	1.49	0.56	-0.85

2613.73	1.14	0.29	-0.79	0.00	-2.34	-0.12	-0.29	1.98	0.40	-0.75	0.48
2793.63	-0.34	1.70	0.05	-0.02	0.40	1.16	1.52	-0.09	0.49	-1.15	0.31
2985.93	0.43	-1.90	-1.50	1.14	-1.41	-1.40	2.53	2.83	-0.49	-1.78	1.72
3191.49	-1.74	2.03	1.20	-0.93	-0.74	2.63	0.59	0.45	0.04	0.08	0.23
3411.21	0.34	1.92	-0.21	0.22	0.05	-0.73	-1.15	1.64	0.25	-1.45	1.65
3646.09	-0.54	0.68	0.90	-1.07	-0.55	-0.96	0.29	0.20	0.66	-1.36	1.38
3897.16	2.72	0.00	-2.02	-0.41	1.94	-1.39	-2.86	3.77	-1.77	-2.46	0.97

Wavelength (nm) / Time (ps)	334.09	335.51	336.94	338.36	339.79	341.21	342.63	344.06	345.48	346.91	348.33
-2.74	0.27	0.06	0.19	0.53	0.87	-0.67	-0.31	-0.19	0.14	0.33	-0.11
-2.24	0.27	-0.57	-0.48	0.55	-0.18	0.42	-0.13	-0.26	0.18	0.35	-0.71
-1.74	0.97	0.43	0.40	-0.02	-0.13	0.72	0.05	-0.04	-0.11	-0.15	-0.36
-1.24	-0.97	0.57	-1.80	-0.86	-0.80	0.64	0.53	-0.41	0.21	-0.79	0.71
-0.74	-0.06	-1.13	0.97	-0.33	-0.63	0.08	0.04	0.30	0.21	-0.16	0.33
-0.24	-0.28	0.04	0.40	-0.88	-0.11	-0.45	-1.23	-0.07	0.28	0.55	0.32
-0.14	0.06	0.24	0.32	0.56	0.61	-0.50	0.23	0.29	-0.22	-0.17	0.21
-0.04	-0.26	0.36	-0.01	0.45	0.37	-0.24	0.82	0.40	-0.69	0.06	-0.40
0.06	0.79	-0.27	-0.86	0.43	-0.57	-0.13	-0.47	-0.03	0.52	0.11	-0.01
0.16	0.36	-0.33	-0.44	-0.30	0.77	0.41	0.31	-0.36	0.46	0.05	0.50
0.26	0.95	-0.01	0.22	0.31	1.18	0.22	-0.13	-0.03	0.50	-0.29	0.19
0.31	-1.07	0.62	-1.54	-0.29	0.71	-0.02	-0.08	0.03	-0.32	0.03	-0.24
0.36	-0.56	-0.54	-1.23	-0.26	0.82	0.75	-0.03	-0.60	0.01	-0.14	0.11
0.41	-17.30	-13.70	-5.96	0.67	-0.54	-0.51	-0.40	0.41	0.48	0.12	-0.28
0.46	26.32	-0.54	-31.24	-45.92	-36.70	-20.53	-10.30	-4.67	-0.80	0.32	0.09
0.51	69.34	91.50	100.75	98.50	82.18	45.45	-4.43	-50.56	-69.02	-55.25	-28.14
0.56	-3.24	31.93	66.64	97.57	129.52	159.07	169.66	148.72	101.89	41.41	-13.93
0.61	-47.13	-59.36	-53.84	-29.21	10.93	71.34	137.17	186.99	210.98	215.48	204.26
0.66	3.75	-0.38	-12.01	-40.13	-76.57	-100.90	-92.86	-51.83	6.65	72.84	138.46
0.71	0.90	1.92	1.01	4.78	6.45	6.82	-0.31	-13.76	-44.00	-89.46	-120.07
0.76	0.79	1.80	1.32	4.22	4.32	3.45	2.79	4.23	7.03	11.30	9.20
0.81	1.25	2.74	0.68	4.29	2.06	3.50	4.47	5.66	6.18	5.41	3.46
0.86	0.59	-0.66	1.93	1.74	2.21	4.15	4.24	4.63	3.84	4.39	5.54
0.91	0.89	1.54	0.66	1.92	3.17	3.56	2.92	4.05	4.53	4.20	5.42
0.96	1.39	0.98	0.63	2.87	3.23	2.67	2.42	3.66	4.01	5.42	5.03
1.01	0.74	0.77	1.38	1.55	2.53	2.92	3.42	4.54	4.38	5.25	5.87
1.06	0.74	2.84	0.79	1.19	1.71	3.09	3.05	4.06	4.42	4.97	4.73
1.11	0.52	1.04	0.34	0.51	1.79	1.95	3.02	3.12	4.28	3.50	4.37
1.16	0.69	2.08	1.70	2.63	1.92	2.03	3.45	3.35	2.25	4.06	4.89
1.21	-0.21	0.40	-0.23	1.36	1.66	2.66	2.51	3.79	4.06	4.24	4.12
1.26	0.87	2.51	1.59	2.32	0.87	3.02	3.66	3.22	3.36	3.64	4.00
1.31	0.94	1.05	0.58	2.46	1.33	1.95	1.85	2.73	2.53	2.93	3.24
1.36	-0.09	1.08	1.06	1.10	1.89	1.85	2.44	1.98	3.27	3.57	4.26
1.41	-0.36	1.42	0.38	1.10	1.36	1.60	1.97	2.46	3.17	4.14	3.97
1.46	2.17	1.69	1.91	1.13	1.34	1.88	3.22	3.05	4.54	3.20	3.27
1.51	0.94	0.35	0.70	0.20	0.67	2.63	2.76	2.60	1.94	2.86	3.91
1.56	0.77	0.17	0.14	1.90	0.18	2.77	2.69	2.34	2.28	3.02	4.43
1.61	0.69	0.20	-0.51	2.12	0.92	2.06	1.50	2.29	3.64	2.95	3.82

1.66	-0.91	0.38	0.56	0.60	2.04	2.43	2.83	2.64	3.29	3.34	3.45
1.71	-0.31	0.83	0.79	0.47	0.38	2.14	1.93	1.97	3.30	4.05	3.22
1.76	1.43	1.94	0.35	1.39	2.47	2.14	0.95	2.98	3.42	3.92	3.96
1.81	1.28	1.08	0.89	1.07	0.18	2.14	1.48	2.33	2.86	3.14	3.03
1.86	1.09	0.54	1.45	1.39	0.85	1.42	2.46	2.10	2.73	2.87	3.84
1.91	0.44	0.11	0.46	2.13	1.42	1.65	1.35	2.69	3.02	3.26	2.74
1.96	0.11	0.30	0.46	1.36	1.14	1.77	2.40	1.91	2.43	3.14	3.89
2.01	0.72	0.48	0.80	0.35	1.41	1.33	1.44	2.65	2.87	3.33	2.78
2.06	-0.13	-0.53	0.62	1.25	1.37	0.88	1.72	2.61	3.24	3.49	3.66
2.11	-2.64	2.24	1.96	0.64	-0.38	2.99	1.37	1.62	3.04	2.76	2.62
2.16	-0.72	1.93	-0.18	0.25	1.31	2.56	1.13	1.80	2.77	2.71	2.73
2.21	1.41	0.87	0.65	1.13	1.85	1.72	3.07	2.57	2.23	3.19	3.63
2.26	0.57	-1.31	1.76	0.78	1.24	1.76	2.05	2.51	3.28	3.46	3.91
2.31	0.30	1.27	0.58	1.10	1.32	1.14	1.47	2.59	2.53	2.31	3.57
2.36	2.29	0.62	-0.16	2.28	1.84	1.34	2.11	3.11	2.20	3.41	3.64
2.41	0.65	0.05	1.27	-0.16	0.94	2.17	1.62	2.65	2.72	2.65	3.13
2.46	-0.12	0.55	1.04	0.06	0.71	1.93	1.73	2.10	3.02	3.56	3.71
2.51	2.02	1.23	-0.73	0.57	2.04	2.09	1.61	3.17	3.43	3.57	3.40
2.56	2.94	1.07	-0.69	1.13	2.32	0.90	1.87	3.16	3.22	2.76	3.73
2.61	2.05	1.41	0.23	0.82	1.20	0.89	1.70	1.93	1.76	2.84	2.04
2.66	1.36	0.48	-0.24	1.10	1.22	2.04	1.74	2.10	1.97	3.23	3.76
2.71	0.40	-0.20	1.44	1.60	-0.21	2.11	3.11	1.67	2.16	3.15	3.35
2.76	-1.29	0.65	1.01	1.69	1.05	2.99	2.17	1.96	2.42	3.77	2.86
2.81	0.89	1.31	-0.62	0.36	-0.35	2.09	2.51	2.53	3.11	3.06	2.91
2.86	-0.95	0.01	-0.68	0.44	0.82	1.29	2.51	1.82	2.29	3.33	3.52
2.91	1.55	1.19	1.07	1.26	1.38	0.95	2.51	2.90	2.00	2.88	3.06
2.96	0.41	0.25	-0.75	1.23	1.77	1.70	1.66	2.73	2.82	3.66	4.44
3.01	1.39	1.49	1.21	0.23	1.80	1.63	1.23	1.88	3.43	2.63	3.27
3.06	1.64	0.95	-0.96	0.91	1.04	2.71	1.76	2.58	2.60	2.67	3.57
3.11	1.33	-0.75	0.37	-0.11	0.45	1.15	1.80	2.29	2.38	3.21	3.01
3.16	1.06	0.52	0.43	1.63	0.69	1.33	2.03	3.43	1.94	2.84	3.41
3.21	0.29	0.37	-0.68	-0.49	1.30	1.79	1.73	2.16	2.89	2.71	3.29
3.26	0.35	0.66	1.36	1.87	0.56	0.29	1.53	1.78	2.47	3.07	2.58
3.31	0.55	0.96	0.79	0.37	1.53	2.27	2.89	2.03	3.38	3.19	2.96
3.36	-1.04	1.77	0.90	0.67	2.52	1.60	1.50	2.22	3.64	2.85	3.27
3.41	0.59	1.17	1.00	1.12	0.96	1.99	2.33	2.25	2.53	3.67	2.78
3.46	-0.46	0.44	0.39	0.56	1.45	1.56	2.04	2.39	2.69	3.14	2.89
3.51	0.73	0.00	1.78	0.52	1.86	2.05	0.88	2.76	2.90	3.53	3.23
3.56	1.15	1.50	-0.87	1.40	0.49	1.46	2.52	2.99	2.51	2.31	3.44
3.61	0.45	-1.46	1.32	1.32	0.26	1.86	3.27	2.44	2.04	3.16	2.17
3.66	1.26	-0.40	-0.22	0.39	1.30	1.69	1.97	1.96	2.57	2.95	3.37
3.71	1.43	0.92	-0.40	1.12	2.03	1.32	1.73	2.12	2.08	3.55	3.62
3.76	-0.40	0.76	-0.70	0.90	0.81	1.82	1.70	3.01	3.36	4.07	3.27
3.81	1.08	1.67	-1.34	-0.65	2.50	1.06	0.53	1.21	3.52	2.82	2.88
3.86	-0.21	1.44	0.49	-0.42	1.60	2.23	2.29	2.56	2.08	2.41	3.01
3.91	1.37	-0.73	-0.43	1.39	2.16	0.97	2.54	3.11	2.45	2.85	2.78
3.96	1.71	0.86	-1.26	1.89	2.42	1.90	1.43	3.35	2.87	2.67	3.44
4.01	-1.23	1.20	0.67	0.79	0.75	1.68	1.78	1.75	2.77	3.36	2.54
4.06	1.26	1.05	0.15	1.29	0.05	1.43	2.41	2.51	3.01	2.76	2.99
4.11	0.23	1.01	-1.07	0.23	1.10	2.03	0.51	2.40	2.73	2.51	2.26

4.16	1.08	1.32	0.02	0.17	1.01	2.29	1.16	1.94	2.58	2.68	2.59
4.21	0.29	0.28	1.06	1.07	1.50	2.30	2.12	2.68	3.39	3.52	3.10
4.26	-0.44	0.75	0.91	0.75	1.63	1.60	2.67	2.53	3.29	3.25	3.57
4.31	0.35	0.77	0.58	1.75	0.17	1.44	1.95	2.31	2.28	3.04	2.97
4.36	0.64	1.79	0.49	1.79	1.40	2.07	1.82	1.29	2.43	2.00	2.22
4.41	-0.92	0.46	0.76	1.96	1.78	2.42	2.72	2.40	2.25	2.46	3.60
4.46	0.11	1.84	1.19	0.68	1.34	1.68	1.64	2.01	2.86	3.64	3.47
4.51	1.04	0.83	0.32	1.36	1.57	2.28	2.03	2.01	3.02	3.07	3.37
4.56	-0.02	0.10	-0.67	0.80	0.28	1.65	1.97	1.74	2.18	2.69	2.53
4.61	0.63	0.67	-0.32	1.26	0.93	1.59	2.45	2.40	2.18	2.85	3.04
4.66	1.53	1.54	0.00	1.18	1.39	1.67	2.44	1.44	2.15	2.58	1.78
4.71	1.44	-0.01	0.71	1.21	0.70	1.29	3.28	2.87	2.75	2.55	3.76
4.76	0.87	1.55	0.86	1.30	1.06	2.30	2.02	2.84	2.36	3.46	3.84
4.81	0.58	0.85	-0.87	1.43	0.77	1.90	2.38	2.50	2.75	3.04	3.28
4.86	0.15	0.51	0.93	2.31	1.56	2.11	1.71	2.55	2.21	2.20	2.89
4.91	0.81	-0.15	1.21	1.50	0.84	1.73	1.87	2.25	2.40	2.65	2.41
4.96	-0.32	1.32	-0.34	0.65	0.86	1.30	2.36	2.65	2.74	3.10	3.88
5.01	1.22	1.70	-0.27	1.07	1.03	0.76	1.64	2.09	2.41	3.06	3.50
5.06	0.05	1.25	-0.58	0.90	1.46	1.74	1.77	2.29	2.14	3.05	3.39
5.11	0.51	0.60	-0.40	1.76	0.77	1.32	1.12	2.43	2.79	2.04	2.73
5.16	0.57	0.73	-0.33	0.31	1.30	1.23	1.80	2.61	2.84	3.34	2.06
5.21	1.98	0.30	0.48	0.73	1.66	0.36	2.53	1.69	2.55	2.80	2.98
5.26	0.59	0.58	1.13	0.83	1.71	1.62	2.46	2.73	2.54	3.42	3.01
5.31	0.56	2.04	1.75	0.90	1.07	2.68	2.36	2.31	2.25	3.15	2.81
5.38	1.34	-0.64	0.37	1.16	1.09	1.46	1.15	2.59	2.50	3.03	2.84
5.45	0.92	1.52	0.63	-0.32	1.06	1.90	1.79	3.04	2.55	2.91	3.50
5.53	-0.11	-0.61	0.58	0.04	0.91	1.42	2.15	2.70	2.04	2.58	2.77
5.61	-0.05	-0.30	0.38	0.72	-0.01	1.70	1.62	1.76	2.16	3.55	2.99
5.70	2.01	0.09	0.03	0.88	0.79	1.31	1.61	2.17	2.46	2.36	2.49
5.80	0.45	1.97	0.43	0.79	1.60	1.76	1.61	1.71	2.16	2.59	3.17
5.90	0.18	0.02	1.85	0.76	1.44	1.13	1.65	1.73	2.76	3.04	3.03
6.01	0.26	-0.42	1.19	1.85	1.33	2.61	1.87	1.77	3.10	3.50	3.56
6.13	-0.09	0.11	-0.54	1.57	2.21	2.01	1.86	1.61	2.56	3.11	3.24
6.25	0.14	0.26	-0.45	1.33	0.21	1.71	1.76	2.09	2.00	2.00	2.45
6.39	0.78	0.70	0.12	1.78	0.37	1.61	1.64	1.29	2.28	2.17	1.93
6.53	0.64	0.06	-0.26	0.63	1.03	1.72	1.20	1.35	1.77	2.40	2.85
6.69	0.99	0.27	0.23	1.63	1.06	1.67	1.12	2.41	2.63	3.39	3.17
6.85	-1.38	1.26	0.88	1.27	0.56	1.82	1.47	1.87	2.17	2.30	2.31
7.03	1.09	0.19	0.02	1.54	0.94	0.32	0.72	1.99	1.25	2.22	2.50
7.21	1.00	1.46	0.76	0.82	1.14	1.55	1.83	1.65	1.77	1.73	2.06
7.41	0.23	0.43	-0.12	1.15	0.74	0.92	2.17	1.92	2.11	2.61	2.46
7.63	0.44	0.01	0.64	1.07	1.16	2.33	1.73	2.56	2.01	2.60	2.56
7.86	0.53	-0.07	-0.35	0.64	1.29	0.96	1.69	1.56	1.41	2.33	3.04
8.10	0.37	0.76	-0.81	0.33	0.99	1.82	1.03	2.48	2.16	2.07	2.45
8.36	-1.69	0.26	0.59	0.10	0.69	1.25	1.42	1.24	2.09	1.85	2.43
8.64	0.57	0.49	1.08	-0.06	0.56	1.62	2.04	2.48	1.19	2.61	2.26
8.94	0.11	-0.67	0.49	0.46	0.70	1.20	2.33	2.07	1.90	2.08	1.95
9.26	0.63	-1.34	0.56	1.50	-0.93	1.24	2.43	1.90	2.11	2.09	2.97
9.60	0.01	0.62	0.46	-0.56	0.64	2.55	1.74	0.76	2.40	2.39	2.26
9.97	0.00	1.86	-0.56	-0.65	0.74	0.45	1.50	1.35	1.67	1.15	2.12

10.36	-0.43	1.01	-0.44	-0.76	0.83	1.77	1.24	1.50	1.62	2.82	2.47
10.77	0.79	-0.16	-0.02	0.85	1.12	0.49	0.67	1.57	2.16	2.52	2.12
11.22	0.20	1.57	-0.89	0.28	0.18	0.69	1.07	1.32	1.03	2.03	2.19
11.70	1.21	0.29	-0.81	1.10	1.13	1.00	0.53	2.00	1.97	2.46	2.14
12.21	-0.15	-0.83	0.75	-0.37	0.09	2.06	0.57	1.28	2.90	2.62	0.95
12.75	0.54	0.14	0.49	0.87	0.13	1.23	1.72	0.87	1.49	1.62	1.36
13.33	0.65	0.19	0.85	1.00	0.19	1.25	0.51	1.54	2.48	1.94	2.17
13.95	0.69	0.63	0.97	0.79	0.81	0.66	1.91	1.77	2.04	1.95	2.09
14.62	-0.95	0.08	-0.25	0.06	-0.53	1.46	0.57	1.32	1.42	1.66	1.40
15.33	-0.77	1.03	-1.58	0.07	0.27	-0.15	0.73	0.05	2.18	2.01	2.27
16.09	-0.31	0.46	-1.24	0.75	1.41	0.84	1.33	1.47	1.89	2.19	1.97
16.90	-0.51	0.49	-0.16	-0.24	1.17	1.14	1.02	1.19	2.38	1.90	1.65
17.77	0.70	-0.24	-0.27	1.05	0.11	0.26	1.82	1.37	1.47	1.41	1.88
18.70	-1.56	1.18	0.72	0.39	1.33	1.16	1.05	1.01	1.41	2.20	2.19
19.69	0.97	0.02	-0.01	-0.61	0.22	0.91	0.62	0.68	1.75	1.82	1.43
20.75	-0.27	0.05	0.58	0.52	0.42	1.40	1.91	0.85	1.37	2.08	1.65
21.89	0.51	0.13	-0.77	0.42	0.17	0.25	1.01	0.88	1.64	1.98	2.88
23.10	-0.01	0.72	-1.14	0.59	0.55	-0.13	-0.01	1.74	2.14	2.00	1.78
24.39	-0.05	0.78	-0.47	0.74	-0.12	-0.68	0.82	1.75	1.22	1.82	1.55
25.78	-0.01	0.31	0.68	1.77	-0.85	1.01	1.03	1.82	1.66	1.89	1.31
27.26	0.80	0.61	0.26	-0.09	-0.42	0.41	0.25	1.19	1.93	2.22	1.74
28.84	0.51	-0.14	0.02	0.22	-0.17	0.97	0.35	0.72	0.82	1.92	2.16
30.53	-1.01	-0.04	-0.18	0.10	0.37	0.25	1.32	1.00	0.90	1.86	1.83
32.34	0.35	0.79	-1.35	1.76	0.51	1.00	1.19	0.88	1.52	2.50	2.03
34.27	0.02	0.51	0.80	0.28	0.74	0.08	1.01	0.79	1.21	1.66	1.75
36.34	-0.39	1.29	-0.97	-0.84	-0.17	1.54	0.22	1.38	0.94	1.27	1.70
38.55	-0.22	-0.36	-0.77	0.07	0.80	1.00	0.62	0.54	1.36	1.17	1.70
40.91	1.27	-0.10	0.34	0.34	0.23	1.10	0.27	1.09	0.81	1.05	1.71
43.43	-0.94	0.93	-0.32	0.01	-0.10	0.42	1.21	0.56	1.59	1.64	1.13
46.13	0.31	0.71	0.38	0.82	0.48	0.48	0.90	1.86	1.35	1.45	1.76
49.01	0.21	0.74	0.05	-0.10	0.31	0.41	0.73	1.46	1.39	1.81	1.46
52.09	0.11	0.18	-0.83	0.69	-0.06	1.40	0.75	1.67	0.88	1.78	1.10
55.39	-0.96	1.08	0.56	-0.23	-0.23	0.57	1.04	1.04	1.35	1.36	0.68
58.91	0.96	-0.95	-0.26	0.25	0.43	0.07	1.30	1.40	0.93	1.43	1.11
62.68	0.13	1.72	1.02	0.61	0.46	0.85	0.92	0.72	1.24	1.77	1.44
66.70	-0.03	0.31	0.16	0.35	1.06	1.16	0.18	0.78	2.06	1.77	1.94
71.00	0.24	0.31	-0.17	0.04	0.34	0.43	0.52	0.91	1.67	1.51	1.63
75.60	0.21	0.42	0.45	-0.49	0.61	0.65	0.90	1.24	1.92	0.86	1.52
80.51	0.14	0.72	0.59	0.19	0.05	1.53	1.22	1.39	2.15	2.08	1.28
85.77	0.55	-0.45	-0.61	-0.14	1.03	0.89	0.90	1.62	1.40	2.14	1.41
91.38	0.49	-0.17	-0.99	0.30	0.43	0.18	0.95	0.39	1.70	1.68	1.33
97.39	0.81	0.37	-0.26	0.48	0.14	1.16	0.03	1.25	0.95	1.43	1.46
103.80	-0.68	1.98	-0.54	0.00	0.27	0.53	0.81	1.44	0.79	1.98	1.92
110.66	-0.66	0.19	-0.01	2.08	0.61	0.28	1.55	1.13	2.07	1.28	2.18
117.99	-0.07	-0.42	-1.32	1.46	-0.36	1.12	0.81	1.43	1.09	1.56	1.10
125.83	0.13	-0.13	0.20	0.85	0.57	-0.41	1.25	-0.01	0.50	1.99	1.76
134.21	0.27	0.84	-0.45	-0.19	0.97	1.00	0.93	1.50	1.23	1.71	1.70
143.17	0.26	0.34	-0.55	1.26	1.14	0.72	0.15	0.73	1.90	1.66	1.92
152.74	2.54	0.05	-0.69	1.67	0.23	0.26	1.02	0.64	1.56	1.48	1.60
162.97	-0.03	0.64	0.62	0.14	0.03	0.68	1.21	0.35	0.81	2.30	1.33

173.91	1.37	-0.44	-0.15	0.01	0.87	1.40	-0.46	0.88	1.70	1.09	1.16
185.60	1.44	-0.58	-1.24	1.74	0.72	-0.25	0.85	1.41	0.78	1.76	1.61
198.10	-0.44	1.62	-0.01	-0.60	0.00	0.88	1.03	0.68	0.73	1.05	1.45
211.46	0.48	-0.20	-0.01	0.15	0.46	1.31	0.29	1.72	1.33	1.92	2.07
225.75	1.19	0.78	0.62	0.07	0.74	0.90	0.73	0.74	1.12	2.35	0.94
241.01	-1.58	0.56	0.08	-0.23	1.75	0.27	0.77	1.52	2.21	1.49	0.76
257.33	-0.12	0.02	-0.94	0.50	0.27	0.63	0.79	1.20	1.66	1.70	1.16
274.77	-0.81	-0.01	-0.17	1.25	-0.55	0.96	-0.18	1.21	0.69	1.49	1.44
293.42	1.65	0.81	-0.86	0.50	0.79	0.29	-0.09	1.15	1.61	1.44	2.24
313.35	-0.23	2.07	0.26	-1.66	-0.37	1.48	0.76	0.91	1.03	1.64	0.78
334.66	-0.80	2.32	0.64	0.19	0.26	0.04	0.70	1.07	2.19	0.72	0.96
357.43	-0.66	0.69	-1.42	0.74	0.12	0.92	1.07	1.37	0.58	1.23	1.97
381.78	0.05	0.88	0.31	-0.28	1.15	0.63	-0.02	1.02	1.19	1.57	1.92
407.80	0.75	-1.12	0.28	1.59	-0.66	0.25	0.62	0.51	1.37	1.88	0.79
435.62	0.71	-0.17	-0.82	-1.63	0.47	0.79	-0.19	0.73	0.86	1.47	1.54
465.35	-0.65	-0.12	0.66	0.82	0.36	1.20	2.11	0.58	0.63	1.80	1.95
497.14	1.04	1.16	-1.58	0.67	0.90	-0.17	1.00	1.45	2.15	1.58	2.14
531.11	-1.19	0.36	0.19	-0.10	1.03	-0.21	1.11	0.97	1.39	0.98	0.83
567.43	-0.33	-0.44	0.65	0.36	0.54	0.30	0.60	1.06	1.21	1.08	1.60
606.26	-0.52	0.52	0.18	0.32	1.33	0.45	0.86	0.89	1.29	2.08	1.81
647.76	0.89	0.36	-0.38	1.09	0.62	1.23	0.87	1.67	0.97	1.53	1.97
692.12	-0.43	-0.24	-1.24	0.71	0.64	0.78	1.30	0.90	1.67	1.43	1.79
739.53	-0.31	0.09	1.03	-0.33	0.77	1.51	0.31	1.02	1.15	1.78	1.27
790.22	-1.31	0.74	-0.67	1.03	0.18	0.29	0.17	1.24	1.42	1.19	1.73
844.40	-1.64	0.67	1.13	-0.29	-0.85	1.27	1.46	-0.01	1.66	1.72	1.69
902.32	0.37	0.87	-0.46	0.21	0.96	0.65	1.65	1.04	1.29	1.78	0.85
964.23	-0.17	1.15	-0.71	0.61	1.11	0.38	0.73	0.65	1.66	2.49	1.42
1030.41	-0.31	-0.86	0.83	-0.25	-0.38	0.98	0.86	1.83	1.15	1.84	1.73
1101.15	0.32	-0.10	-0.37	0.67	-0.09	1.35	1.11	1.12	1.41	1.64	1.90
1176.77	-0.65	-0.01	1.13	0.71	-0.21	0.44	0.88	1.54	0.55	1.54	1.35
1257.60	1.61	0.06	-1.17	0.95	1.72	0.38	0.81	1.72	1.09	2.09	1.29
1344.00	0.50	1.61	-0.12	0.36	0.59	0.24	0.89	0.97	1.28	2.19	1.97
1436.36	-0.11	1.31	0.42	-0.52	0.58	1.13	0.98	0.80	1.69	1.50	2.26
1535.09	1.04	0.13	-0.59	0.43	-0.78	0.16	1.03	1.76	1.41	1.30	0.97
1640.63	-0.23	0.26	-1.30	-0.58	1.24	1.75	-0.29	1.37	0.94	1.17	1.10
1753.44	-0.30	0.68	1.46	-0.06	0.41	0.68	0.71	1.04	1.30	1.65	1.42
1874.02	-0.36	0.41	-0.41	0.16	-0.15	0.42	1.19	0.63	1.67	1.53	1.25
2002.92	0.02	1.07	0.73	0.67	-0.21	0.85	0.54	0.27	1.57	1.33	0.72
2140.71	-1.20	0.08	-0.05	0.21	1.15	0.39	1.02	0.91	1.67	2.82	1.61
2288.00	-0.10	0.61	-1.20	1.42	0.67	0.75	0.81	0.87	1.31	2.25	1.78
2445.44	-2.14	-1.11	-0.43	1.11	0.97	0.64	1.04	0.57	1.74	1.75	1.41
2613.73	0.59	-0.19	-0.87	0.30	0.87	0.81	0.21	0.54	0.76	2.00	1.30
2793.63	0.49	0.43	-0.52	0.25	0.66	0.90	1.11	0.46	1.77	1.79	1.29
2985.93	-1.28	-1.56	0.43	0.83	-0.73	1.24	1.14	1.76	1.77	1.49	2.45
3191.49	0.50	1.54	0.54	-0.18	-0.35	0.58	0.37	1.40	2.44	1.98	2.13
3411.21	1.16	0.81	-0.85	1.36	0.21	0.19	2.59	1.99	1.04	2.05	2.74
3646.09	0.90	0.65	0.29	0.26	0.79	2.02	1.48	2.45	1.93	2.33	2.08
3897.16	1.80	-2.02	-0.24	1.88	0.74	0.14	1.55	2.38	1.43	1.70	2.19

Wavelength (nm) / Time (ps)	349.75	351.18	352.60	354.03	355.45	356.87	358.30	359.72	361.15	362.57	363.99
-2.74	0.24	-0.09	-0.38	0.26	-0.65	-0.04	-0.11	-0.29	0.00	0.17	0.33
-2.24	-0.47	0.18	0.04	0.13	0.03	-0.05	0.18	0.10	-0.02	0.22	0.21
-1.74	-0.30	-0.39	-0.37	0.01	0.08	-0.34	-0.04	0.19	-0.18	-0.18	-0.04
-1.24	0.28	0.57	0.40	-0.35	0.71	-0.22	-0.04	-0.17	0.14	0.02	0.03
-0.74	-0.10	-0.09	-0.23	0.31	-0.05	0.24	0.08	0.09	0.02	-0.11	-0.27
-0.24	0.35	-0.10	0.26	0.11	0.05	0.04	0.04	0.15	-0.17	-0.18	-0.21
-0.14	0.05	0.05	0.19	-0.29	0.04	0.22	-0.22	0.18	0.18	0.07	-0.04
-0.04	-0.04	-0.13	0.09	-0.19	-0.22	0.16	0.11	-0.25	0.01	-0.03	-0.02
0.06	-0.43	-0.03	0.05	-0.35	0.06	-0.35	0.05	0.40	-0.22	-0.31	-0.23
0.16	0.51	-0.17	0.74	0.65	0.01	-0.45	-0.01	0.03	0.14	0.33	0.06
0.26	0.28	0.17	0.01	0.17	0.04	-0.19	0.02	0.37	0.08	0.08	-0.10
0.31	0.40	-0.07	0.06	0.33	0.25	-0.28	-0.01	-0.05	-0.19	-0.53	-0.24
0.36	0.04	-0.15	0.06	0.06	-0.41	-0.01	-0.24	-0.10	-0.04	-0.14	-0.38
0.41	0.19	0.08	0.02	0.55	0.21	0.34	0.06	0.14	0.26	-0.05	-0.19
0.46	-0.11	0.16	-0.31	0.50	-0.02	-0.28	0.15	0.10	-0.16	-0.09	-0.40
0.51	-8.52	-1.16	-0.82	0.58	0.16	-0.24	-0.07	0.06	-0.20	0.08	0.13
0.56	-49.64	-64.37	-57.26	-37.01	-16.14	-4.83	-0.72	-0.19	0.24	0.26	0.00
0.61	173.94	120.90	54.34	-10.81	-57.30	-78.07	-73.14	-49.18	-21.59	-2.98	0.98
0.66	201.19	249.08	275.13	273.85	244.44	186.26	105.07	18.88	-50.68	-84.24	-82.54
0.71	-116.10	-74.30	-1.38	89.95	188.43	274.83	330.28	345.36	329.11	297.31	252.72
0.76	1.92	-8.59	-37.51	-83.96	-115.25	-109.66	-64.21	7.25	89.13	168.36	237.34
0.81	4.78	6.39	9.69	12.28	10.27	4.49	-3.18	-29.78	-78.81	-125.01	-141.12
0.86	6.66	7.79	7.93	6.02	6.41	6.88	8.53	10.99	12.60	10.38	7.67
0.91	6.79	6.18	6.42	6.97	6.71	7.46	8.65	9.89	7.92	6.54	6.79
0.96	6.24	5.93	5.98	6.25	7.73	8.02	7.56	7.70	8.01	8.36	8.32
1.01	4.78	5.42	6.71	7.43	7.44	8.34	8.65	7.61	7.85	9.28	10.16
1.06	5.56	6.11	6.13	6.94	6.93	6.26	6.94	7.77	8.32	8.47	8.66
1.11	5.26	5.55	5.28	6.13	6.50	6.63	6.91	7.18	7.36	7.46	7.50
1.16	4.60	4.65	5.66	5.68	5.78	6.70	7.46	7.29	6.70	7.24	7.93
1.21	5.12	4.93	5.16	6.19	6.62	6.64	6.42	6.61	6.90	7.19	7.27
1.26	4.53	4.74	6.02	5.92	6.48	6.65	6.36	6.59	6.68	7.60	7.44
1.31	4.55	4.59	5.55	5.68	5.48	6.09	6.02	7.22	6.79	6.70	6.81
1.36	4.20	4.40	4.44	4.90	5.36	5.93	5.97	5.81	6.24	6.41	6.31
1.41	4.20	4.43	4.90	5.46	5.67	6.00	5.84	5.77	5.86	6.50	6.50
1.46	4.33	4.31	5.06	5.91	5.95	5.52	5.87	6.43	6.35	6.39	5.87
1.51	4.02	4.81	4.82	4.83	5.55	5.70	6.61	5.68	6.70	6.59	6.27
1.56	4.07	4.62	4.83	4.83	5.89	5.61	5.86	6.02	5.62	6.58	6.89
1.61	4.60	3.98	4.22	5.07	5.23	4.88	5.53	5.89	6.08	6.09	5.59
1.66	4.46	4.52	5.19	5.35	5.48	5.12	5.49	6.14	6.47	6.42	6.35
1.71	4.28	4.41	4.72	4.92	5.23	5.59	5.57	6.50	6.09	5.97	5.98
1.76	5.60	4.86	4.30	5.14	6.16	5.65	5.72	5.56	6.29	6.69	6.04
1.81	3.81	3.95	4.51	4.35	5.10	5.22	5.23	4.95	5.36	6.12	5.77
1.86	4.24	3.66	4.88	4.38	5.09	5.07	5.24	5.43	5.52	5.92	5.78
1.91	3.74	4.15	4.03	4.28	4.93	5.02	5.21	5.39	5.21	5.86	5.15
1.96	3.56	3.91	4.54	5.04	5.42	5.60	5.45	5.66	5.56	5.87	5.81

2.01	4.30	4.74	4.51	5.08	5.56	4.95	5.53	5.78	5.70	6.20	5.37
2.06	4.04	4.13	4.59	4.77	5.31	5.18	5.31	5.45	5.28	5.61	6.18
2.11	3.10	4.30	4.11	3.77	5.71	5.35	5.05	5.33	5.58	5.46	5.14
2.16	3.99	3.83	3.20	4.59	5.25	5.10	4.89	5.50	5.40	5.16	5.48
2.21	3.93	3.53	4.71	5.11	5.28	5.15	5.74	5.27	5.56	6.02	6.57
2.26	4.03	4.14	4.53	5.10	5.13	4.90	5.73	5.82	6.23	6.03	6.18
2.31	3.99	3.44	4.54	4.49	4.97	4.89	4.93	5.44	5.11	5.37	5.36
2.36	4.18	3.32	3.96	4.35	4.51	5.15	5.32	4.97	5.06	5.47	5.28
2.41	3.46	3.72	3.98	4.56	4.90	4.93	4.91	5.50	5.82	6.05	5.43
2.46	3.35	4.21	3.81	4.08	4.66	5.05	4.83	4.89	5.73	5.74	5.38
2.51	4.23	4.18	4.08	5.68	5.38	4.89	5.23	5.66	5.44	5.81	5.96
2.56	3.33	3.98	3.96	4.51	4.63	4.57	4.99	5.71	4.97	5.21	5.27
2.61	3.20	3.64	4.14	3.91	5.19	4.70	4.93	4.53	5.24	5.69	5.41
2.66	3.24	4.10	3.83	4.27	4.85	5.10	4.77	4.88	5.02	5.33	5.42
2.71	3.21	4.34	4.42	4.80	5.09	5.51	5.41	5.10	5.34	5.93	5.44
2.76	4.28	4.35	4.11	4.48	5.14	5.29	5.05	5.23	5.53	5.78	5.58
2.81	3.05	4.52	4.25	4.54	4.61	4.46	4.56	4.61	5.45	4.96	5.16
2.86	3.12	3.95	4.14	3.69	5.06	4.62	4.59	4.68	5.18	5.44	4.99
2.91	3.85	3.71	4.31	4.81	4.57	4.80	5.28	5.02	4.97	5.16	5.00
2.96	4.61	4.24	4.39	4.87	4.76	5.02	4.96	5.59	5.17	5.30	5.43
3.01	4.27	3.80	4.90	3.66	5.37	4.98	5.19	5.41	5.13	5.58	5.79
3.06	3.84	3.92	4.30	4.43	4.96	4.72	5.04	5.49	5.14	5.63	5.68
3.11	3.31	3.91	4.31	4.17	4.35	4.71	4.80	4.58	5.35	5.50	4.88
3.16	3.27	4.17	3.96	3.95	4.40	4.48	4.99	4.79	4.84	5.34	5.15
3.21	3.46	3.18	4.20	3.64	4.49	4.95	4.83	5.26	5.48	5.61	5.50
3.26	3.23	3.35	3.47	4.36	4.34	4.63	4.65	5.54	5.04	5.13	5.39
3.31	3.48	4.05	4.37	3.95	4.86	4.30	5.09	4.69	5.07	5.55	5.63
3.36	3.47	3.82	4.38	3.42	5.22	4.46	5.08	5.04	5.62	5.22	4.86
3.41	3.36	3.97	4.32	4.21	4.89	4.77	4.38	5.23	5.38	5.10	5.20
3.46	3.90	3.99	3.33	4.35	5.01	5.12	4.54	5.48	5.17	4.97	5.32
3.51	3.55	3.80	3.99	4.82	4.84	4.68	4.77	4.96	4.94	5.18	5.37
3.56	3.65	3.84	4.14	4.00	4.51	4.15	4.51	4.78	4.78	5.50	5.46
3.61	3.10	3.91	4.34	4.05	4.08	4.57	4.58	4.95	4.68	4.81	4.40
3.66	4.04	4.20	4.37	4.01	5.02	4.79	5.15	4.86	4.84	5.41	5.72
3.71	3.65	4.08	4.40	4.69	5.02	4.43	4.98	5.64	5.06	5.14	5.26
3.76	4.58	3.80	4.06	4.60	4.99	4.37	5.30	5.18	5.42	5.48	5.71
3.81	3.98	3.57	3.19	4.44	4.68	4.52	4.52	4.57	4.75	4.74	4.87
3.86	3.83	3.57	3.97	3.64	4.17	4.41	4.50	4.53	4.76	4.79	4.81
3.91	3.64	3.60	4.10	4.83	4.82	4.31	4.81	5.32	5.39	5.21	5.50
3.96	4.36	3.83	4.27	5.06	4.49	4.41	4.88	5.36	5.33	5.55	5.42
4.01	4.05	3.95	4.73	4.40	4.30	4.93	4.83	5.08	5.33	5.22	5.36
4.06	3.47	3.12	4.32	4.40	4.28	4.63	4.70	4.76	4.45	4.77	5.22
4.11	3.44	3.50	3.90	3.89	4.62	4.26	4.61	5.32	4.61	4.44	4.85
4.16	3.60	3.50	4.02	3.93	5.36	4.23	4.69	4.91	5.00	4.94	5.21
4.21	3.61	4.18	4.59	5.32	5.06	4.95	4.96	5.16	5.65	5.76	5.56
4.26	4.05	4.03	4.44	4.80	5.05	4.85	5.33	5.37	5.33	5.33	4.94
4.31	3.59	3.66	3.27	4.37	4.29	3.95	4.24	4.20	4.83	4.51	4.79
4.36	3.60	3.24	3.01	3.79	4.04	3.67	4.66	4.65	4.65	4.56	4.53
4.41	3.56	3.71	4.01	4.00	4.83	4.51	4.80	4.97	5.04	4.94	4.97
4.46	4.05	3.75	4.64	4.36	4.74	4.78	5.34	4.77	5.31	5.13	5.39

4.51	3.79	4.02	4.19	4.50	4.18	4.96	4.86	5.01	5.18	5.14	4.85
4.56	3.85	4.07	3.99	4.38	4.62	4.01	4.78	4.87	4.92	5.35	5.02
4.61	3.54	3.81	4.13	4.54	4.27	4.08	4.77	4.76	4.54	5.15	4.67
4.66	3.30	3.35	4.08	3.79	3.79	3.90	4.71	4.78	4.87	4.78	4.97
4.71	3.96	3.67	4.63	4.92	5.23	4.69	5.36	5.44	5.66	5.63	5.60
4.76	3.70	3.79	4.41	4.27	4.61	4.43	5.17	5.01	5.21	5.60	5.62
4.81	4.00	3.74	3.90	4.02	4.54	3.81	4.70	4.52	4.66	4.68	5.11
4.86	2.94	3.35	4.10	3.73	4.64	4.44	4.72	4.71	4.72	4.88	4.84
4.91	3.38	3.85	3.80	4.06	4.50	4.62	4.49	4.56	5.33	5.02	5.25
4.96	3.48	3.45	3.65	4.40	4.70	4.52	4.71	4.59	5.08	5.01	4.90
5.01	3.26	2.94	4.24	4.02	4.06	4.71	4.75	5.17	4.87	5.41	5.21
5.06	3.61	3.76	3.94	4.41	4.14	3.89	4.67	4.96	4.65	4.74	5.20
5.11	2.53	3.84	3.46	3.80	4.12	3.83	4.17	4.50	4.53	4.83	4.47
5.16	3.40	4.03	3.26	3.82	4.40	4.34	4.37	4.09	4.60	4.98	4.50
5.21	3.47	3.83	3.59	4.37	4.02	4.54	4.32	4.81	4.41	4.40	4.58
5.26	3.41	3.57	4.29	4.08	4.63	4.81	5.09	4.74	5.00	5.23	4.92
5.31	3.10	3.61	4.05	3.89	4.50	4.13	5.01	4.27	4.69	4.41	4.84
5.38	3.00	3.82	3.48	3.94	4.36	4.01	4.64	4.91	4.52	4.85	4.61
5.45	3.43	3.79	4.25	4.50	5.07	5.00	5.10	4.84	4.96	4.94	4.82
5.53	2.32	3.47	3.61	4.11	4.62	4.20	4.09	4.73	4.90	5.01	4.99
5.61	3.13	4.23	3.81	4.08	4.30	4.51	4.53	4.87	5.64	5.01	5.05
5.70	3.14	3.57	3.60	4.12	4.33	4.06	4.51	4.66	4.68	4.73	5.02
5.80	3.61	3.37	3.05	4.08	4.03	4.03	4.35	5.01	4.80	4.64	5.19
5.90	2.94	3.73	4.21	3.94	3.85	4.23	4.08	3.98	4.28	4.77	4.50
6.01	3.48	3.94	4.17	3.60	3.85	4.59	4.80	5.05	5.01	4.95	5.31
6.13	3.44	3.74	4.10	3.73	4.18	4.79	4.45	4.76	4.64	4.64	4.67
6.25	2.48	2.93	3.40	3.27	4.31	4.12	4.06	4.77	4.31	4.65	4.38
6.39	2.58	3.47	3.50	3.96	3.85	3.98	4.29	4.11	4.20	4.36	4.76
6.53	3.21	3.30	3.44	4.29	3.95	4.09	4.02	4.26	4.32	4.24	4.42
6.69	3.52	3.74	3.79	4.09	4.17	4.62	4.49	4.66	4.93	5.15	4.84
6.85	3.65	3.63	3.63	3.61	3.95	4.74	4.50	4.45	4.25	4.36	4.35
7.03	3.07	2.72	3.56	3.38	3.42	4.14	3.98	4.21	3.68	3.88	4.36
7.21	2.46	3.23	3.67	3.23	3.61	3.99	3.88	3.88	4.26	4.32	4.48
7.41	3.46	3.02	3.74	3.62	3.72	4.14	4.40	4.41	4.52	4.80	4.54
7.63	4.25	3.84	3.74	4.10	4.04	4.26	4.90	4.34	4.20	4.75	4.93
7.86	2.92	3.32	3.84	3.58	3.94	3.97	4.34	4.30	4.38	4.62	4.24
8.10	2.62	2.66	2.62	3.61	4.09	3.59	3.82	4.28	4.14	4.39	4.57
8.36	3.21	2.93	3.00	2.90	3.32	3.16	3.93	3.98	3.80	3.98	3.93
8.64	3.25	3.29	3.11	3.41	3.57	4.15	4.28	4.24	4.22	4.15	4.72
8.94	2.82	3.52	3.81	3.64	4.15	3.53	4.53	4.68	4.51	4.65	4.43
9.26	3.02	3.55	4.01	3.01	4.17	4.42	4.54	4.54	4.05	4.86	4.50
9.60	2.34	2.85	2.74	3.12	3.99	3.71	3.30	4.14	3.87	4.30	3.81
9.97	2.30	3.12	3.09	3.01	3.10	2.86	3.54	3.51	3.81	3.76	3.88
10.36	3.02	3.21	3.23	2.99	3.15	3.83	3.70	4.28	4.19	4.43	4.04
10.77	2.94	2.97	3.56	3.57	3.28	3.36	3.70	4.47	4.25	4.28	4.51
11.22	2.56	2.97	2.83	3.41	3.34	3.75	3.43	4.10	3.87	4.14	4.36
11.70	2.43	2.18	3.63	2.88	2.88	3.40	3.68	3.95	3.97	3.90	4.04
12.21	1.99	1.95	2.70	2.39	3.56	3.00	2.63	3.39	3.38	3.65	3.40
12.75	1.94	2.44	3.12	3.00	3.49	3.51	3.35	3.60	3.79	3.78	3.67
13.33	1.69	2.59	2.65	2.98	3.19	3.27	4.10	3.94	3.93	4.27	3.78

13.95	2.98	2.82	2.90	3.24	3.65	3.42	3.82	3.65	3.83	4.03	4.17
14.62	1.91	2.94	2.67	3.03	3.24	3.57	3.30	3.52	3.12	3.62	3.53
15.33	2.12	2.38	2.59	2.44	3.39	2.96	3.11	3.55	3.48	3.52	3.55
16.09	1.78	2.36	2.50	2.87	3.21	3.22	3.46	3.25	3.55	3.94	3.95
16.90	2.22	3.23	2.56	2.42	3.07	2.88	3.34	3.39	4.02	4.13	3.93
17.77	1.86	2.03	2.13	2.28	3.22	2.99	2.93	3.32	3.64	3.67	3.40
18.70	1.95	3.79	2.44	2.60	3.10	2.99	3.27	3.51	3.65	4.06	3.60
19.69	2.03	1.89	2.43	2.60	3.11	2.91	2.89	2.88	3.25	3.36	3.18
20.75	2.11	2.52	2.16	2.54	3.15	2.64	2.88	3.38	2.99	3.82	3.82
21.89	2.67	2.32	2.82	3.34	3.17	3.17	3.17	3.09	3.43	3.57	3.38
23.10	2.27	2.39	2.89	3.30	3.53	3.18	3.71	4.23	3.73	4.07	4.38
24.39	2.15	2.58	2.38	2.68	2.81	3.10	3.53	3.53	3.33	3.61	3.17
25.78	1.60	2.16	2.59	2.45	2.82	2.61	3.13	3.33	2.95	3.14	3.50
27.26	1.91	2.31	2.58	2.39	2.98	2.91	3.59	3.24	3.08	3.21	2.78
28.84	2.35	2.16	2.67	2.55	3.04	2.88	2.29	2.77	3.35	3.24	3.53
30.53	2.33	2.38	2.59	3.06	3.19	2.92	3.01	3.68	3.51	3.40	3.73
32.34	2.20	2.90	2.42	2.03	2.98	2.88	3.41	3.25	3.00	3.28	3.60
34.27	2.07	2.32	2.28	2.62	3.02	2.61	3.19	3.14	3.40	3.65	3.43
36.34	1.89	1.60	2.29	2.71	2.98	2.95	2.96	2.99	2.93	3.73	3.49
38.55	1.94	2.54	1.81	2.35	2.56	3.00	2.95	3.74	3.46	3.44	3.33
40.91	2.29	2.02	2.30	2.51	3.19	2.56	2.99	3.26	3.43	3.38	3.23
43.43	1.29	1.91	2.13	2.91	2.55	2.57	2.59	2.85	2.90	3.08	3.02
46.13	2.48	2.25	2.57	2.95	2.83	2.55	2.74	3.20	3.34	3.59	3.38
49.01	1.91	2.39	2.50	2.56	2.57	2.87	3.49	3.14	3.40	3.14	3.52
52.09	1.25	2.25	2.64	2.71	2.88	2.44	2.68	2.89	3.18	3.29	3.08
55.39	1.95	2.00	1.57	2.17	2.26	2.32	2.61	3.20	2.74	3.38	3.48
58.91	1.83	2.34	2.61	2.76	2.63	2.97	3.09	3.39	3.37	3.58	3.24
62.68	1.55	1.89	2.17	2.43	2.94	3.02	2.69	2.80	2.66	3.56	2.63
66.70	2.10	2.43	2.22	2.70	3.15	2.80	2.90	3.49	3.03	3.11	3.11
71.00	1.55	2.66	2.40	2.26	2.43	2.90	2.71	2.82	3.30	3.80	3.09
75.60	2.19	1.80	2.46	3.02	2.95	2.35	3.00	3.10	3.11	3.55	3.16
80.51	1.92	2.07	2.32	2.50	2.79	3.18	3.33	3.23	3.34	3.13	3.33
85.77	2.41	2.19	2.70	2.46	2.71	2.82	3.00	2.86	3.05	3.36	3.11
91.38	2.28	2.53	2.06	2.41	2.54	2.80	3.41	2.92	3.19	3.36	3.24
97.39	2.17	1.77	2.43	2.28	2.42	2.96	3.13	3.05	3.03	2.88	2.93
103.80	2.31	2.71	2.65	3.09	2.61	2.54	3.20	3.34	3.56	3.57	3.77
110.66	1.78	2.26	2.25	2.94	3.04	3.11	3.07	2.91	3.52	3.45	3.67
117.99	1.28	1.16	1.62	2.48	2.82	2.32	2.60	2.74	3.23	3.20	2.88
125.83	2.32	2.21	2.21	2.20	2.94	2.70	2.75	3.13	3.80	3.48	3.53
134.21	2.50	1.47	2.29	2.85	2.60	2.68	2.87	2.98	3.24	2.99	2.99
143.17	2.19	2.31	2.79	2.61	3.77	2.73	3.58	3.33	3.37	3.50	3.79
152.74	1.72	2.00	2.30	2.99	2.91	2.75	3.05	3.46	3.07	3.35	3.29
162.97	1.39	2.32	2.38	2.39	3.27	2.46	2.62	3.33	3.62	3.23	2.96
173.91	1.72	2.11	2.10	2.19	2.89	2.50	2.77	2.90	3.14	3.08	3.01
185.60	1.74	1.48	2.27	2.57	2.75	2.67	3.04	2.63	2.95	3.21	3.36
198.10	2.82	2.26	2.56	2.79	2.41	2.60	2.91	3.61	3.21	3.32	3.22
211.46	2.18	2.24	2.30	2.82	2.72	3.01	3.87	3.28	3.39	3.62	3.59
225.75	1.72	1.31	2.29	2.04	2.52	2.56	2.96	3.25	2.59	3.09	2.81
241.01	1.97	1.94	2.79	2.33	2.79	2.29	2.61	2.80	3.03	3.60	2.88
257.33	1.00	1.53	2.33	2.64	2.68	2.48	3.10	2.96	2.71	3.12	3.14

274.77	2.01	1.56	1.93	2.03	2.69	2.42	2.56	2.78	2.81	3.24	2.97
293.42	2.78	1.67	2.74	2.99	2.90	2.08	3.17	3.20	3.33	3.42	3.18
313.35	2.19	1.89	1.83	2.10	3.26	2.69	2.65	3.06	3.27	3.19	3.23
334.66	2.13	2.41	3.02	2.54	2.66	2.97	2.68	2.63	3.20	3.34	3.00
357.43	1.34	2.24	2.14	2.54	2.52	2.48	3.16	2.72	2.76	3.11	2.84
381.78	2.13	1.71	2.41	2.47	2.31	2.77	2.71	2.75	2.80	3.03	2.69
407.80	1.87	2.23	2.09	2.80	2.16	2.66	2.85	2.58	3.11	3.07	2.63
435.62	1.92	2.67	1.59	2.55	2.68	2.08	2.90	2.73	2.74	3.12	3.32
465.35	1.71	1.88	2.54	2.32	2.33	3.20	2.76	2.70	3.10	2.99	3.31
497.14	2.13	1.72	2.30	2.66	2.40	3.00	2.89	2.74	2.76	3.47	3.23
531.11	1.95	1.59	1.46	1.87	2.12	2.41	2.58	2.77	2.92	2.81	2.89
567.43	1.41	1.28	2.66	2.87	2.44	2.31	3.11	2.65	2.69	2.93	2.79
606.26	2.84	2.60	2.26	2.98	2.91	3.11	2.81	2.83	3.16	3.01	3.64
647.76	1.93	2.69	2.44	2.73	2.80	2.94	2.87	3.19	2.93	3.44	3.01
692.12	2.64	2.27	2.46	2.59	2.94	2.81	3.22	3.25	3.06	3.02	3.97
739.53	2.47	1.71	2.15	2.80	2.86	2.25	2.64	3.24	3.27	3.27	3.48
790.22	1.83	1.36	1.74	3.13	2.56	2.10	2.48	3.13	2.99	3.08	2.92
844.40	1.33	1.91	2.15	2.21	2.52	2.57	2.75	2.89	2.49	3.13	2.55
902.32	2.46	2.40	2.49	2.92	3.17	3.20	3.18	3.16	3.40	3.38	3.57
964.23	1.92	2.38	2.15	3.09	2.65	2.59	2.84	2.36	2.71	3.11	3.72
1030.41	2.32	2.31	2.53	2.70	2.64	2.96	2.82	3.13	3.28	3.57	2.83
1101.15	2.43	2.55	2.89	2.46	2.71	2.76	3.33	2.99	3.37	3.43	3.26
1176.77	1.53	2.25	2.49	2.60	2.65	2.87	3.45	3.35	3.07	3.48	3.57
1257.60	1.94	1.77	2.44	2.65	2.37	2.53	2.83	3.12	3.12	3.67	3.74
1344.00	2.64	1.75	2.02	2.85	2.70	2.80	3.21	3.12	3.54	2.62	3.65
1436.36	1.87	1.69	2.23	2.50	3.04	2.56	2.87	3.23	2.82	3.20	3.14
1535.09	1.69	1.73	2.44	2.87	2.60	2.48	3.13	2.94	3.05	3.59	3.44
1640.63	2.35	1.76	2.12	2.80	3.01	2.67	3.03	2.75	3.28	3.24	3.25
1753.44	2.07	2.09	2.21	2.52	2.48	2.53	2.78	3.20	3.17	3.33	3.10
1874.02	2.35	2.44	2.80	2.39	2.41	2.97	3.25	3.10	3.12	3.39	3.15
2002.92	2.36	2.33	2.64	2.36	2.71	2.67	2.79	2.82	3.34	3.34	3.00
2140.71	2.17	2.38	2.21	2.80	3.03	3.23	3.17	3.17	3.20	3.03	3.53
2288.00	1.93	2.17	2.46	2.61	2.65	2.82	3.21	2.96	3.02	3.25	2.97
2445.44	1.92	2.25	2.36	2.31	2.82	2.93	3.07	3.19	3.09	3.26	2.93
2613.73	2.52	2.59	2.62	2.73	2.69	2.51	2.65	2.78	3.40	3.69	3.67
2793.63	1.68	2.59	2.12	2.60	2.55	2.92	2.90	3.41	3.61	3.39	3.39
2985.93	1.57	2.27	3.49	2.60	2.85	3.09	3.09	3.44	3.53	3.79	3.39
3191.49	2.18	2.20	2.58	2.98	3.09	2.93	3.31	3.82	3.11	3.90	3.47
3411.21	2.59	2.05	3.32	3.13	2.90	2.91	3.54	3.38	3.45	3.92	4.12
3646.09	2.74	2.42	3.00	3.52	3.63	3.73	3.53	3.64	3.63	4.10	3.76
3897.16	3.30	2.29	3.61	3.62	3.66	3.29	3.87	3.31	3.69	4.19	4.14

Wavelength (nm) / Time (ps)	365.42	366.84	368.27	369.69	371.11	372.54	373.96	375.39	376.81	378.23	379.66
-2.74	0.34	-0.09	0.21	-0.05	0.00	0.14	0.01	-0.35	0.01	-0.13	0.10
-2.24	0.34	0.18	0.13	0.19	0.02	0.06	0.11	0.30	0.17	0.28	0.31
-1.74	-0.26	0.13	0.12	-0.14	-0.03	-0.03	0.03	0.16	0.20	-0.09	-0.20
-1.24	-0.20	0.02	-0.22	-0.03	-0.05	-0.03	0.02	0.17	-0.16	-0.04	0.14
-0.74	0.11	-0.07	-0.16	0.01	-0.01	-0.01	0.17	0.28	-0.09	0.10	0.00

-0.24	-0.03	-0.02	0.05	-0.02	-0.03	-0.13	-0.16	-0.32	-0.15	0.10	-0.14
-0.14	-0.15	0.07	0.02	0.13	0.16	0.19	-0.02	-0.29	0.14	-0.24	0.14
-0.04	-0.14	-0.22	-0.16	-0.09	-0.08	-0.19	-0.16	0.04	-0.12	0.02	-0.35
0.06	0.23	0.18	0.14	0.08	-0.15	-0.02	-0.24	-0.21	0.26	0.23	0.10
0.16	0.34	-0.05	0.37	-0.01	0.59	0.18	-0.04	0.02	0.52	0.25	0.30
0.26	0.24	0.27	0.20	0.13	-0.06	0.09	-0.18	0.01	0.13	0.17	0.18
0.31	0.40	0.09	0.06	-0.25	-0.19	-0.38	0.07	-0.19	0.03	0.16	0.42
0.36	-0.06	-0.11	-0.12	-0.04	-0.08	-0.13	-0.44	-0.11	-0.09	0.13	0.00
0.41	0.08	0.03	-0.16	0.08	-0.10	0.17	-0.10	-0.33	0.01	0.27	-0.15
0.46	0.03	-0.38	0.10	-0.10	-0.25	0.12	0.10	0.09	-0.18	-0.11	0.06
0.51	-0.15	0.19	0.13	0.21	-0.02	-0.36	-0.43	-0.46	0.04	0.11	0.24
0.56	0.59	0.19	-0.07	0.02	0.16	-0.08	-0.20	0.20	-0.07	-0.11	-0.16
0.61	0.39	0.07	0.16	-0.18	0.15	0.41	-0.23	0.31	0.20	0.04	-0.03
0.66	-57.84	-30.66	-13.86	-7.39	-3.50	-0.10	0.95	-0.03	-0.04	0.18	0.05
0.71	196.98	125.90	47.58	-25.01	-77.37	-96.62	-83.03	-50.45	-20.23	-5.06	-0.60
0.76	300.96	353.66	384.50	380.94	336.74	263.48	174.57	83.67	2.56	-59.16	-95.60
0.81	-116.88	-54.10	42.60	157.29	265.25	344.22	387.81	400.09	390.82	358.75	303.83
0.86	6.16	-8.81	-44.45	-87.84	-114.90	-110.37	-72.38	-3.75	85.08	183.44	274.30
0.91	8.87	9.92	11.23	15.69	13.75	8.08	12.21	15.78	-10.58	-63.24	-111.52
0.96	9.85	10.46	10.79	9.83	9.01	9.09	9.96	10.50	11.76	14.64	15.22
1.01	10.28	9.56	10.29	10.90	10.82	10.66	11.76	12.49	12.91	12.36	11.73
1.06	9.17	9.23	9.36	9.11	9.95	10.92	11.04	10.18	10.81	11.36	11.51
1.11	7.64	7.82	9.15	9.54	9.28	9.30	9.40	9.70	9.80	10.45	10.33
1.16	7.78	8.33	8.47	9.20	8.78	8.30	8.51	9.52	10.10	9.99	9.66
1.21	8.54	8.83	8.67	8.49	9.02	9.56	9.22	8.69	9.29	10.26	10.35
1.26	7.63	7.33	8.19	8.00	8.14	8.12	8.61	9.32	9.51	9.75	9.53
1.31	7.34	7.19	7.81	8.33	8.53	8.64	8.20	8.55	8.89	8.88	8.78
1.36	7.00	7.53	7.48	7.40	7.63	7.90	7.94	7.64	7.89	8.76	8.74
1.41	7.03	6.69	7.60	7.67	7.83	7.61	7.81	7.96	8.38	8.17	8.14
1.46	6.98	6.92	6.98	7.06	7.60	7.83	7.42	7.43	7.91	8.35	8.36
1.51	6.70	6.81	7.37	7.64	7.53	7.51	8.12	8.10	7.79	8.03	8.16
1.56	6.93	7.11	7.37	7.86	7.78	7.44	7.87	7.86	8.17	8.24	7.88
1.61	6.90	7.04	6.44	6.96	7.07	7.08	7.58	7.42	7.94	8.25	8.06
1.66	6.52	6.61	7.07	7.51	7.23	7.63	7.96	7.53	7.61	8.03	8.39
1.71	6.60	6.57	6.78	7.26	7.32	7.31	7.30	7.58	7.92	8.09	7.94
1.76	7.15	6.83	7.26	7.24	7.30	7.77	8.06	7.74	7.85	8.33	8.85
1.81	5.98	5.86	6.35	6.47	6.57	6.73	7.00	6.72	7.15	7.20	7.08
1.86	7.04	6.24	6.30	6.78	6.98	6.85	6.88	7.10	7.40	7.64	7.45
1.91	5.89	6.32	6.55	6.56	6.08	6.48	6.76	7.15	6.98	7.25	7.37
1.96	6.47	6.39	6.84	6.94	7.16	7.22	6.84	6.96	7.55	7.64	7.33
2.01	6.40	6.48	6.26	6.69	7.23	7.27	6.93	7.26	7.41	8.00	7.74
2.06	6.18	6.34	7.12	6.88	7.13	6.93	7.13	7.29	6.89	7.65	7.54
2.11	6.43	5.77	6.20	6.88	6.62	6.15	6.75	6.93	7.17	7.13	7.33
2.16	6.47	6.26	6.36	6.47	6.56	6.75	6.82	6.80	6.83	7.27	7.56
2.21	6.56	6.03	6.90	6.89	6.70	6.93	6.90	7.22	7.47	7.35	7.62
2.26	6.77	6.64	7.20	7.08	7.22	7.15	6.97	7.22	7.56	7.89	7.90
2.31	5.83	5.78	6.29	6.15	6.18	6.21	6.77	6.54	6.88	7.13	7.48
2.36	5.47	5.33	6.07	6.43	5.79	6.51	6.33	6.07	6.40	7.09	6.92
2.41	5.86	6.16	6.20	6.24	6.45	6.35	6.44	6.37	7.00	7.20	7.42

2.46	5.61	5.89	6.17	6.14	6.56	6.63	6.45	6.68	6.93	7.22	7.25
2.51	6.29	6.40	6.42	6.80	6.87	6.95	6.97	6.98	7.38	7.90	7.26
2.56	6.23	5.86	5.96	6.46	6.49	6.64	7.00	6.62	7.09	7.56	7.63
2.61	5.62	5.43	5.97	6.35	6.23	6.34	6.56	6.24	6.42	6.64	6.79
2.66	6.06	5.57	6.03	6.10	6.52	6.39	6.76	6.53	6.52	7.06	7.02
2.71	5.68	6.26	6.30	6.40	6.76	6.61	6.63	6.39	7.01	7.15	7.06
2.76	5.84	5.87	5.97	6.48	6.62	6.76	6.79	6.62	7.00	7.25	7.11
2.81	6.04	5.75	5.87	6.25	6.30	6.27	6.17	6.15	6.49	6.63	6.83
2.86	5.70	5.34	5.50	5.83	6.11	5.94	5.97	6.22	6.50	6.51	6.61
2.91	5.71	5.44	6.17	6.15	6.19	6.07	6.45	6.75	6.65	6.91	6.52
2.96	6.15	5.75	6.55	6.21	6.44	6.67	6.55	6.74	7.23	7.35	7.23
3.01	6.02	5.71	5.84	5.82	6.34	6.48	6.43	6.60	6.71	7.12	6.98
3.06	5.88	5.86	6.05	5.95	6.30	6.61	5.99	6.39	6.61	7.06	6.76
3.11	5.63	5.43	6.30	6.25	6.27	6.23	6.19	6.46	6.76	6.94	6.92
3.16	5.83	5.87	5.77	6.05	6.22	5.94	6.00	6.18	6.21	6.59	6.52
3.21	6.12	5.89	6.28	5.86	6.28	6.88	6.65	6.32	6.88	6.83	7.08
3.26	5.47	5.65	6.09	5.73	6.22	6.24	6.12	6.21	6.56	6.91	6.72
3.31	6.17	5.92	5.72	6.41	6.19	6.40	6.43	6.35	6.77	6.93	6.92
3.36	5.60	5.83	6.00	6.21	6.52	6.71	6.31	6.46	6.95	7.17	6.75
3.41	5.40	5.88	6.22	6.06	6.29	6.16	6.27	6.41	6.35	6.84	6.79
3.46	5.90	5.56	6.17	6.27	6.53	6.27	6.47	6.43	6.84	6.90	6.96
3.51	5.58	5.73	5.80	6.29	5.82	6.66	6.43	6.31	6.25	6.76	7.16
3.56	5.50	5.51	5.84	5.83	5.97	6.07	5.98	5.94	6.52	6.63	6.78
3.61	5.21	5.17	5.62	5.49	5.87	6.06	5.79	6.02	6.53	6.68	6.80
3.66	5.60	6.01	5.79	6.26	6.01	6.26	5.88	6.32	6.71	7.19	6.80
3.71	5.95	5.84	6.35	6.58	6.28	6.25	6.62	6.55	6.99	6.82	7.17
3.76	6.52	6.27	6.11	6.85	6.67	6.52	6.52	6.81	6.79	6.85	7.22
3.81	5.38	5.32	5.88	6.01	6.05	5.73	6.21	6.32	6.14	6.66	6.83
3.86	5.20	4.82	5.68	5.74	5.53	5.86	5.94	5.80	6.18	6.51	6.23
3.91	5.53	5.67	5.97	6.10	6.30	6.30	6.54	6.44	6.78	7.17	6.73
3.96	5.71	5.68	6.48	6.16	6.26	6.28	6.98	6.35	6.56	6.83	6.91
4.01	5.91	5.66	5.59	6.06	6.39	6.57	6.57	6.51	7.08	6.50	6.94
4.06	5.63	5.42	6.14	5.97	5.96	6.41	6.32	6.52	6.50	6.60	6.71
4.11	5.54	5.01	5.88	6.23	5.94	5.76	6.21	6.14	6.27	6.51	6.66
4.16	5.48	5.37	5.88	5.80	6.08	6.11	6.52	6.46	6.84	6.76	6.79
4.21	5.99	6.03	6.33	6.60	6.55	6.44	6.77	6.55	6.64	7.42	7.16
4.26	5.90	5.88	5.90	6.64	6.17	6.65	6.50	6.58	7.03	7.06	6.98
4.31	5.28	5.69	5.77	5.93	5.75	5.56	5.90	6.01	6.01	6.70	6.58
4.36	5.27	5.14	5.59	5.41	5.63	5.95	5.80	5.91	5.82	6.23	6.36
4.41	5.70	5.22	5.61	5.49	5.84	6.07	6.30	6.02	6.31	6.44	6.37
4.46	5.91	6.06	6.20	6.26	6.18	6.46	6.50	6.80	6.63	6.84	7.02
4.51	6.02	5.80	5.81	5.98	6.44	6.48	6.25	6.21	6.64	6.65	6.79
4.56	5.57	5.59	5.43	5.66	6.13	6.20	6.41	6.15	6.11	6.54	6.77
4.61	5.60	5.65	5.69	5.99	5.53	6.06	6.26	6.09	6.40	6.79	6.49
4.66	4.99	5.16	6.06	5.71	5.81	5.81	5.77	5.93	6.49	6.64	6.35
4.71	6.24	6.07	6.22	6.68	6.61	6.69	6.80	6.54	6.78	7.21	7.18
4.76	5.81	5.64	5.76	6.03	6.13	6.21	6.63	6.46	6.46	7.08	7.35
4.81	5.29	5.17	5.54	5.77	5.80	6.16	6.13	6.39	6.51	6.48	6.55
4.86	5.16	5.64	5.26	5.60	5.94	5.99	5.84	6.22	6.37	6.55	6.53
4.91	5.38	5.56	5.50	5.91	6.02	5.80	6.24	6.04	6.27	6.96	6.72

4.96	5.58	5.35	5.92	5.63	6.18	6.07	5.90	6.69	6.99	7.11	6.66
5.01	5.88	5.89	5.92	6.14	5.90	6.25	6.40	6.22	6.77	6.92	6.88
5.06	5.44	5.21	5.66	5.75	5.93	5.77	6.05	6.25	6.16	6.54	6.43
5.11	4.94	4.98	5.62	5.66	5.87	5.74	5.56	5.60	5.76	6.34	6.24
5.16	5.38	4.90	5.48	5.50	5.61	5.63	5.89	5.74	6.28	6.45	6.41
5.21	5.20	5.35	5.18	5.71	5.62	5.68	5.52	6.02	6.23	6.21	6.21
5.26	5.49	5.78	6.15	6.03	6.01	6.33	6.33	6.37	6.57	6.83	6.90
5.31	5.41	5.48	5.57	5.87	6.11	6.08	6.54	6.28	6.41	6.72	6.76
5.38	5.26	5.36	5.60	5.31	5.44	5.76	5.82	5.68	6.14	6.35	6.30
5.45	5.55	5.69	5.99	6.06	5.90	5.88	6.01	6.16	6.14	6.64	6.61
5.53	5.38	5.20	5.52	5.47	5.64	6.00	6.09	6.27	6.34	6.33	6.64
5.61	5.72	5.51	5.66	6.04	5.79	6.05	6.20	6.19	6.52	6.93	6.86
5.70	5.34	5.21	5.46	5.74	5.72	5.74	6.02	5.79	5.98	6.27	6.03
5.80	5.35	5.42	5.36	5.87	5.82	5.74	5.98	5.85	5.81	6.43	6.38
5.90	4.94	5.11	5.18	5.47	5.63	5.46	5.62	5.35	5.71	6.10	6.32
6.01	5.65	5.67	6.02	5.99	5.98	6.17	5.99	6.20	6.34	6.76	6.80
6.13	5.08	5.17	5.28	5.62	5.58	5.89	5.92	5.78	6.05	6.59	6.82
6.25	5.21	4.98	5.21	5.28	5.35	5.51	5.78	5.32	6.06	5.92	5.95
6.39	4.75	4.83	5.10	5.37	5.32	5.35	5.27	5.36	5.89	6.21	6.07
6.53	4.77	5.01	4.97	4.88	5.40	5.61	5.50	5.43	5.80	6.14	6.05
6.69	5.00	5.40	5.55	5.64	6.01	6.02	6.09	6.12	6.40	6.58	6.84
6.85	4.99	5.00	5.00	5.20	5.52	5.54	5.75	5.40	5.80	6.32	6.01
7.03	4.67	4.59	5.17	5.15	5.24	5.71	5.82	5.16	5.69	5.89	5.79
7.21	4.65	4.65	4.81	4.76	5.13	5.20	5.12	5.31	5.41	5.92	5.78
7.41	4.79	4.82	5.41	5.33	5.77	5.77	5.55	5.99	6.13	6.42	6.20
7.63	5.17	5.03	5.25	5.89	5.69	5.49	5.71	5.89	6.43	6.57	6.43
7.86	4.82	5.08	5.08	5.72	5.56	5.71	5.24	5.59	5.85	6.28	6.33
8.10	5.29	4.65	5.15	5.26	5.47	5.31	5.60	5.49	5.59	5.52	5.75
8.36	4.90	4.20	4.92	4.76	5.06	5.12	5.15	5.15	5.08	5.43	5.35
8.64	4.88	4.69	5.31	5.43	5.35	5.45	5.44	5.60	6.15	6.05	6.01
8.94	4.61	5.54	5.59	5.52	5.68	5.77	5.97	5.94	6.39	6.48	6.15
9.26	5.24	5.16	5.64	5.49	5.52	5.54	5.96	5.63	5.94	6.32	6.54
9.60	5.32	4.55	4.90	4.93	5.31	5.38	5.38	5.26	5.26	6.00	5.77
9.97	4.35	4.25	4.55	4.63	4.81	5.08	4.85	5.16	5.51	5.54	5.72
10.36	5.06	4.87	5.12	5.07	5.15	5.29	5.44	5.64	6.04	6.12	6.11
10.77	4.61	4.66	4.76	5.03	5.14	5.55	5.25	5.46	5.77	5.92	5.92
11.22	4.34	4.61	4.88	4.68	4.80	5.26	5.17	5.24	5.36	5.64	5.79
11.70	4.74	4.45	4.81	4.66	5.36	5.37	5.46	5.29	5.77	6.04	6.11
12.21	4.24	4.29	4.19	4.84	4.98	4.74	4.94	4.94	4.97	5.19	5.46
12.75	4.50	4.25	4.79	4.45	4.80	4.96	5.16	5.06	5.34	5.47	5.77
13.33	4.03	4.73	4.85	4.84	5.25	5.27	5.38	5.24	5.53	5.96	5.87
13.95	4.26	4.68	4.63	5.28	5.20	5.03	5.19	5.53	5.91	5.76	5.88
14.62	3.95	4.09	4.44	4.67	4.95	4.72	4.52	5.15	5.15	5.44	5.62
15.33	4.02	3.86	4.12	4.27	4.56	4.47	4.59	4.61	4.72	5.13	5.25
16.09	4.36	4.06	4.31	4.49	4.64	4.71	4.80	5.07	5.29	5.34	5.45
16.90	4.36	4.12	4.85	4.73	4.80	4.89	5.04	5.18	5.48	5.81	5.87
17.77	3.96	4.59	4.68	4.54	4.81	5.20	4.87	5.12	5.57	5.63	5.34
18.70	4.20	4.15	4.26	4.63	4.66	4.73	4.83	4.83	5.08	5.44	5.36
19.69	3.52	3.76	3.69	4.23	4.36	4.41	4.57	4.55	4.93	5.01	5.45
20.75	3.85	3.74	4.37	4.25	4.17	4.57	4.59	4.80	4.61	5.18	5.28

21.89	3.87	3.76	4.56	4.46	4.76	5.04	4.99	5.07	5.16	5.68	5.56
23.10	4.39	4.70	4.51	4.78	4.92	5.01	5.01	5.55	5.55	5.87	6.04
24.39	4.26	3.71	4.47	4.15	4.36	4.80	4.89	4.91	5.03	5.36	5.44
25.78	3.86	4.23	3.92	4.35	4.52	4.59	4.45	5.01	4.81	5.26	5.08
27.26	3.52	3.90	4.60	4.08	4.46	4.61	4.79	5.06	5.07	5.19	5.45
28.84	3.61	3.95	4.12	4.41	4.58	4.66	4.44	5.11	5.07	5.12	5.42
30.53	3.89	3.81	4.40	4.13	4.79	4.72	4.83	4.80	5.01	5.43	5.43
32.34	3.68	3.20	3.76	4.05	4.06	4.30	4.66	4.42	4.73	5.24	5.07
34.27	3.98	3.63	4.34	3.81	4.26	4.77	4.63	4.37	5.21	5.35	5.38
36.34	3.78	4.10	4.45	4.32	4.38	4.41	4.54	4.65	4.83	5.33	5.19
38.55	3.84	3.97	3.93	4.09	4.26	4.60	4.52	4.79	4.72	5.49	5.23
40.91	3.74	3.89	4.49	4.21	4.62	4.44	4.82	4.73	4.92	5.40	5.27
43.43	3.43	3.62	3.77	3.87	4.33	4.16	4.16	4.40	4.36	5.14	4.97
46.13	3.80	4.15	4.17	4.09	5.16	4.60	4.70	4.70	5.23	5.16	5.38
49.01	4.17	3.89	3.62	4.23	4.75	4.71	4.66	4.44	4.90	5.26	5.37
52.09	3.67	3.55	4.08	4.32	4.19	4.46	4.41	4.41	4.81	4.90	5.34
55.39	3.53	3.78	3.82	4.28	4.48	4.03	4.55	4.65	4.94	5.18	5.02
58.91	3.45	3.63	4.00	4.18	4.28	4.50	4.28	4.45	4.84	4.73	4.72
62.68	3.71	3.52	3.85	4.23	4.01	4.41	4.04	4.41	4.95	5.01	4.91
66.70	4.03	3.70	4.39	4.08	4.19	4.35	4.31	4.40	4.74	5.46	4.94
71.00	3.60	3.46	4.18	3.89	4.62	4.59	4.47	4.89	4.98	4.84	5.02
75.60	3.94	3.37	3.72	4.10	4.22	4.28	4.35	4.24	4.71	5.01	4.69
80.51	3.61	3.88	3.90	4.09	4.45	4.36	4.33	4.49	4.91	5.33	5.32
85.77	3.82	3.32	4.07	4.01	4.13	4.42	4.28	4.56	4.41	4.63	4.68
91.38	3.71	3.72	4.18	4.27	3.83	4.28	4.43	4.51	4.97	4.99	5.08
97.39	3.68	3.95	3.76	4.07	4.20	4.15	4.26	4.46	4.89	5.04	4.58
103.80	3.79	4.27	4.11	4.35	4.69	4.69	4.78	4.56	5.08	4.95	5.38
110.66	4.02	3.90	4.22	4.13	4.47	4.34	4.46	4.48	4.96	4.98	5.21
117.99	3.49	3.43	3.71	3.99	4.18	4.11	4.09	4.19	4.54	5.15	4.85
125.83	3.73	3.93	4.32	3.99	4.22	4.51	4.47	4.34	4.51	4.82	4.82
134.21	3.69	3.98	3.86	3.88	4.46	4.40	4.45	4.73	4.72	4.95	4.80
143.17	4.04	3.99	3.97	4.49	4.23	4.17	4.28	4.74	4.93	5.20	5.33
152.74	3.93	3.46	3.66	4.13	4.30	4.40	4.39	4.52	4.64	5.09	4.69
162.97	3.78	3.43	3.97	3.91	4.29	4.15	3.93	4.49	4.65	4.51	5.02
173.91	3.16	3.20	4.03	4.17	4.09	4.14	4.27	4.17	4.47	4.64	4.86
185.60	3.22	3.76	3.96	3.54	4.12	4.57	4.39	4.28	4.72	4.75	4.76
198.10	3.87	3.48	3.90	4.09	4.26	4.45	4.23	4.25	4.38	4.63	4.72
211.46	3.76	4.16	4.03	4.19	4.17	4.39	4.77	4.62	5.33	5.25	5.02
225.75	3.36	3.45	3.57	3.62	4.10	4.05	4.15	4.00	4.23	4.54	4.48
241.01	3.37	3.57	3.88	4.20	4.46	4.24	4.28	4.46	4.34	4.80	5.12
257.33	3.44	3.40	4.00	3.95	4.08	4.45	4.11	4.31	4.57	4.96	4.49
274.77	3.28	3.37	3.64	4.28	3.97	4.23	4.04	4.07	4.37	4.68	4.77
293.42	3.54	3.76	4.14	4.31	4.34	4.41	4.54	4.40	4.80	4.72	4.78
313.35	3.42	3.51	3.57	3.83	3.90	4.02	4.71	4.73	4.53	4.73	4.65
334.66	3.67	3.83	4.01	4.34	4.33	4.29	4.54	4.37	4.67	4.97	4.97
357.43	3.56	3.67	3.90	4.08	3.97	4.22	4.05	4.17	4.41	4.43	4.52
381.78	3.70	3.37	3.54	3.82	3.64	3.93	4.10	4.27	4.59	4.70	4.72
407.80	3.34	3.65	3.69	3.60	4.18	4.06	4.10	3.97	4.63	4.58	4.57
435.62	3.64	3.80	4.00	4.10	4.18	4.25	4.35	4.63	4.49	4.93	4.78
465.35	3.42	3.81	3.65	3.90	4.19	4.30	4.05	4.46	4.51	4.46	4.60

497.14	3.86	3.47	4.10	3.83	4.37	4.37	4.26	4.47	4.37	5.14	4.52
531.11	3.38	3.53	3.74	3.51	3.89	3.84	4.24	3.63	4.47	4.73	4.44
567.43	3.69	3.71	3.92	3.91	4.25	4.27	4.04	4.55	4.45	4.58	4.47
606.26	3.60	3.72	4.11	3.91	4.01	4.45	4.22	4.42	4.40	4.71	4.57
647.76	4.11	3.84	4.00	4.40	4.25	4.57	4.27	4.55	4.56	4.87	4.75
692.12	3.69	3.74	3.91	3.99	4.23	4.25	4.05	4.41	4.59	4.85	4.19
739.53	3.92	3.52	4.15	4.33	4.20	4.33	4.25	4.17	4.85	4.84	4.87
790.22	3.56	3.41	3.76	4.23	3.78	3.79	4.05	4.00	4.31	4.08	4.18
844.40	3.19	3.10	3.56	3.65	3.75	3.98	3.93	4.43	4.41	4.54	4.52
902.32	4.12	3.88	4.05	4.07	4.51	4.33	4.45	4.40	4.41	5.09	5.05
964.23	3.74	3.47	3.80	3.74	3.85	4.12	4.18	4.31	4.14	4.55	4.71
1030.41	3.71	4.08	3.85	3.95	4.42	4.37	4.49	4.43	4.44	4.84	4.51
1101.15	3.74	3.57	4.14	4.16	4.10	4.25	4.05	4.16	4.65	4.59	4.43
1176.77	3.75	3.76	3.89	4.00	4.36	4.31	4.41	4.50	4.55	4.86	4.74
1257.60	3.59	3.43	4.20	3.94	4.31	4.26	4.21	4.66	4.87	4.86	4.48
1344.00	4.14	3.62	4.15	4.19	4.18	4.66	4.48	4.51	4.71	4.88	5.07
1436.36	3.34	3.67	3.69	3.91	4.01	4.21	4.28	3.80	4.64	4.68	4.38
1535.09	4.18	3.97	3.93	4.08	4.31	4.23	4.52	4.16	4.59	4.78	4.45
1640.63	3.82	3.43	3.73	4.44	4.13	4.05	4.35	4.34	4.67	4.59	4.54
1753.44	3.84	3.60	3.99	4.15	4.35	4.24	4.57	4.26	4.56	4.63	4.45
1874.02	3.55	3.76	3.52	3.63	4.18	4.50	3.85	4.09	4.24	4.45	4.14
2002.92	4.00	3.52	4.03	4.16	4.30	4.32	4.87	4.48	4.18	4.54	4.79
2140.71	3.68	3.52	4.00	4.32	4.34	4.56	4.47	4.58	4.40	4.60	4.68
2288.00	3.63	4.12	3.72	3.93	4.21	4.45	3.99	4.52	4.49	4.72	4.64
2445.44	3.53	3.41	3.98	4.17	4.41	4.20	4.40	4.05	4.42	4.58	4.61
2613.73	3.95	3.80	4.03	4.02	3.95	4.88	4.48	4.23	4.65	4.88	4.73
2793.63	3.67	3.74	4.20	4.32	4.27	4.63	4.36	4.02	4.47	4.69	4.40
2985.93	3.44	4.16	4.11	3.94	4.40	4.49	4.51	4.39	4.56	4.40	4.73
3191.49	3.82	4.09	4.07	4.23	4.58	4.36	4.52	4.35	4.53	5.01	5.05
3411.21	4.22	4.36	4.19	4.28	4.76	4.84	4.54	4.51	4.99	5.28	4.56
3646.09	4.58	3.77	4.47	4.68	4.79	4.94	4.74	4.79	4.86	5.11	4.88
3897.16	4.24	4.47	4.70	4.75	4.82	4.96	5.11	4.89	5.27	5.42	5.35

Wavelength (nm) / Time (ps)	381.08	382.51	383.93	385.36	386.78	388.20	389.63	391.05	392.48	393.90	395.32
-2.74	0.35	-0.19	0.05	0.00	-0.10	-0.10	-0.10	-0.09	0.21	0.08	0.07
-2.24	-0.10	0.06	0.11	0.05	0.33	0.01	0.04	0.07	0.41	0.06	0.15
-1.74	-0.07	0.20	0.09	0.11	0.22	-0.05	-0.19	-0.17	-0.31	0.10	0.03
-1.24	-0.02	-0.06	-0.01	-0.16	-0.23	0.38	-0.12	0.01	0.04	-0.05	-0.17
-0.74	-0.17	-0.09	0.04	0.09	-0.02	0.07	0.11	0.05	-0.04	-0.04	0.13
-0.24	0.10	0.28	-0.07	-0.19	0.00	-0.27	-0.08	-0.11	-0.21	-0.26	0.10
-0.14	0.14	-0.04	0.02	0.14	-0.11	-0.25	0.05	0.26	-0.04	0.31	-0.13
-0.04	-0.22	-0.15	-0.22	-0.02	-0.10	0.20	0.29	0.00	-0.07	-0.20	-0.18
0.06	0.07	-0.15	0.02	-0.06	-0.21	-0.06	0.30	0.21	-0.21	-0.09	0.02
0.16	0.03	0.07	0.25	0.21	0.49	0.01	0.38	0.22	0.24	0.41	0.32
0.26	0.13	-0.09	0.06	0.43	0.09	-0.06	0.21	-0.04	-0.09	0.17	0.16
0.31	0.13	-0.15	-0.28	-0.15	-0.13	-0.08	-0.03	-0.12	-0.25	0.03	0.06
0.36	-0.13	0.06	0.04	-0.12	0.20	0.30	0.10	0.19	0.09	-0.06	-0.01
0.41	0.10	-0.07	-0.07	0.11	0.01	0.08	0.09	0.09	-0.01	-0.01	0.19

0.46	0.27	-0.20	-0.17	-0.08	-0.17	-0.19	-0.13	-0.02	-0.08	-0.13	0.14
0.51	0.03	0.15	-0.02	-0.01	0.14	-0.34	0.01	-0.09	-0.19	-0.28	0.24
0.56	0.11	0.08	-0.01	0.07	0.20	0.15	-0.24	0.00	-0.13	0.28	0.18
0.61	0.33	0.04	0.06	0.49	-0.10	-0.07	0.03	-0.09	-0.07	0.07	0.10
0.66	0.34	-0.02	-0.06	0.21	0.11	0.10	0.26	-0.07	-0.01	0.14	0.08
0.71	-0.40	-0.01	0.40	0.24	0.15	-0.11	0.37	0.41	0.12	0.24	-0.01
0.76	-104.29	-87.78	-57.17	-26.28	-7.12	-0.26	0.42	0.28	0.23	0.48	0.31
0.81	229.11	140.69	50.84	-27.93	-83.88	-110.92	-106.38	-77.15	-38.64	-11.43	-2.53
0.86	344.38	384.70	395.67	377.75	337.31	280.65	216.15	150.30	87.84	30.95	-26.63
0.91	-131.08	-113.91	-63.15	15.81	106.34	187.39	247.50	287.59	321.01	355.86	380.86
0.96	12.40	13.64	17.78	10.33	-19.30	-62.40	-100.07	-113.90	-95.52	-42.53	49.67
1.01	12.29	12.61	13.15	13.01	13.46	16.68	15.83	13.16	17.33	24.39	17.37
1.06	11.46	11.41	11.66	12.71	12.82	12.89	12.67	12.19	11.91	11.83	11.37
1.11	11.24	11.31	11.25	11.28	11.37	11.42	12.09	11.61	11.31	12.10	12.09
1.16	10.27	10.86	10.96	11.07	10.61	10.53	11.11	11.32	11.64	12.19	12.17
1.21	10.06	9.81	9.74	10.45	11.16	11.10	11.23	10.94	11.18	11.57	11.22
1.26	9.78	10.35	10.32	10.23	9.97	10.27	10.62	10.63	10.45	10.74	10.82
1.31	9.14	9.22	9.57	10.25	10.35	9.99	10.18	9.95	10.11	10.13	10.53
1.36	9.14	8.78	8.77	9.18	9.34	9.59	9.73	9.61	9.57	10.18	10.07
1.41	9.04	9.17	9.14	8.72	9.18	8.88	9.15	9.07	9.19	9.57	9.65
1.46	8.02	8.10	8.68	9.20	9.19	9.13	8.99	9.32	9.14	9.61	9.50
1.51	8.99	9.01	8.87	9.16	9.05	9.25	9.40	9.67	9.12	9.48	9.59
1.56	8.56	9.15	9.18	8.81	8.83	8.81	9.16	9.10	9.02	9.70	9.71
1.61	8.13	8.16	8.64	8.81	8.59	8.65	8.91	8.83	8.92	9.51	9.23
1.66	8.73	8.53	8.58	8.71	8.76	9.02	8.88	8.95	9.18	9.30	9.41
1.71	7.96	8.23	8.75	8.95	8.80	8.51	8.92	8.77	8.85	8.98	9.26
1.76	8.95	8.48	8.36	8.65	8.65	8.82	8.94	8.79	9.11	9.41	9.47
1.81	7.79	7.89	7.58	7.75	7.94	7.82	8.07	8.37	7.98	8.72	8.41
1.86	7.83	7.79	8.04	8.17	8.39	8.09	8.47	8.59	8.52	8.65	8.80
1.91	7.68	7.59	7.81	7.99	7.80	7.88	8.34	7.93	8.27	8.47	8.40
1.96	7.93	8.02	8.17	8.22	8.21	8.05	8.10	8.35	8.14	8.56	8.71
2.01	7.60	7.87	7.91	8.12	8.48	8.44	8.32	8.37	8.45	8.74	8.73
2.06	7.78	7.84	7.77	8.48	8.23	8.26	8.41	8.37	8.51	8.60	8.84
2.11	7.64	7.58	7.72	7.92	7.96	7.70	7.99	8.15	7.93	8.45	8.58
2.16	7.51	7.22	7.68	8.07	8.00	7.79	8.43	7.99	8.27	8.49	8.43
2.21	7.76	7.96	8.40	8.06	8.05	8.31	8.44	8.36	8.28	8.41	8.57
2.26	8.01	7.74	8.10	8.70	8.67	7.98	8.36	8.51	8.07	8.83	8.75
2.31	7.23	7.29	7.67	7.74	7.80	7.97	8.03	8.14	7.54	8.11	7.96
2.36	7.08	7.24	7.34	7.16	7.28	7.30	7.84	7.73	7.81	7.88	7.71
2.41	7.20	7.27	7.05	7.47	7.73	7.66	7.81	7.77	7.64	7.90	8.04
2.46	7.53	7.26	7.14	7.40	7.37	7.53	8.00	8.03	8.00	7.91	8.13
2.51	7.50	7.59	7.67	8.38	8.23	8.42	8.19	8.02	8.56	8.50	8.49
2.56	7.32	7.75	7.73	7.62	8.04	7.78	8.02	7.90	8.00	8.45	8.16
2.61	7.27	7.07	7.26	7.48	7.36	7.45	7.74	7.39	7.50	7.68	8.01
2.66	7.03	7.31	7.28	7.70	7.48	7.27	7.55	7.72	7.78	7.98	8.36
2.71	7.57	7.72	7.54	7.67	7.84	7.94	7.89	8.15	7.94	8.02	8.34
2.76	7.24	7.61	7.66	7.54	8.05	7.63	7.82	8.10	7.99	8.08	8.45
2.81	7.18	7.10	7.44	7.22	7.43	7.32	7.80	7.57	7.62	7.62	7.72
2.86	7.02	6.63	7.11	7.11	7.11	6.97	7.37	7.19	7.21	7.62	7.68

2.91	7.00	7.28	7.33	7.45	7.43	7.22	7.90	7.35	7.65	8.09	7.95
2.96	7.29	7.21	7.13	7.90	7.67	7.54	7.70	7.54	7.73	8.06	8.24
3.01	7.21	7.52	7.33	7.32	7.25	7.46	7.60	7.82	7.85	7.83	7.76
3.06	6.93	7.17	7.27	7.68	7.49	7.71	7.61	7.53	7.26	7.88	7.67
3.11	7.27	7.12	7.01	7.22	7.57	7.29	7.66	7.55	7.56	7.66	7.73
3.16	6.52	6.97	6.86	7.06	7.16	7.11	7.43	7.41	7.53	7.58	7.73
3.21	7.32	7.28	7.39	7.27	7.38	7.33	7.87	7.66	7.67	7.79	8.15
3.26	7.07	6.98	7.13	7.20	7.51	7.54	7.75	7.73	7.18	7.60	7.81
3.31	6.84	7.34	7.23	7.45	7.56	7.38	7.42	7.68	7.38	7.91	7.78
3.36	6.77	7.29	7.18	7.18	7.45	7.52	7.60	7.44	7.74	7.79	7.90
3.41	7.25	7.14	7.45	7.50	7.17	7.49	7.72	7.74	7.25	7.93	7.87
3.46	7.07	7.44	7.12	7.43	7.56	7.34	7.57	7.74	7.69	7.87	7.80
3.51	6.97	7.10	7.32	7.17	7.23	7.28	7.54	7.45	7.22	7.60	7.77
3.56	7.03	6.92	7.35	7.36	7.34	7.19	7.31	7.25	7.46	7.56	7.76
3.61	6.64	6.37	7.03	7.08	7.05	7.00	7.30	7.23	7.03	7.19	7.32
3.66	6.75	7.01	6.69	7.09	7.25	7.27	7.52	7.41	7.53	7.63	7.61
3.71	7.17	7.22	7.24	7.51	7.53	7.68	7.73	7.85	7.72	8.15	8.07
3.76	7.34	7.82	7.49	7.90	7.59	7.75	7.73	7.80	7.64	8.29	8.26
3.81	6.83	6.86	6.68	6.97	7.00	7.40	7.46	7.11	7.20	7.64	7.55
3.86	6.49	6.56	6.75	6.75	6.83	7.12	7.17	6.93	7.21	7.42	7.11
3.91	7.02	7.10	7.29	7.64	7.62	7.42	7.48	7.53	7.43	7.68	7.91
3.96	6.70	7.52	7.05	7.45	7.33	7.27	7.67	7.62	7.72	8.13	7.80
4.01	7.13	7.23	7.14	7.44	7.60	7.69	7.84	7.77	7.54	8.04	7.89
4.06	7.02	7.11	7.21	7.24	7.47	7.28	7.61	7.48	7.33	7.62	8.02
4.11	7.02	7.10	6.71	7.03	7.01	7.35	7.58	7.37	7.29	7.71	7.48
4.16	7.18	6.97	7.07	7.49	7.37	7.39	7.58	7.11	7.44	7.79	7.64
4.21	7.36	7.55	7.41	7.91	7.71	7.49	8.15	8.02	8.17	7.92	8.14
4.26	7.17	7.36	7.26	7.40	7.71	7.46	7.91	7.30	7.63	7.77	7.98
4.31	6.77	6.66	6.98	6.90	6.97	6.61	7.24	7.09	7.03	7.34	7.46
4.36	6.23	6.92	6.67	6.80	7.08	7.13	7.23	7.13	7.07	7.29	7.45
4.41	6.48	6.91	6.68	6.96	7.24	7.11	7.35	7.13	7.02	7.47	7.48
4.46	7.00	7.19	7.10	7.43	7.46	7.27	7.75	7.86	7.87	7.89	7.89
4.51	6.92	7.08	6.94	7.09	7.49	7.33	7.31	7.25	7.44	7.75	7.77
4.56	6.40	6.86	7.04	6.98	7.15	7.02	7.29	7.30	7.22	7.74	7.52
4.61	6.58	6.74	6.73	7.10	7.04	6.69	6.98	7.10	7.13	7.47	7.53
4.66	6.86	6.73	6.93	7.05	7.05	7.15	7.28	7.15	7.24	7.71	7.86
4.71	7.24	7.33	7.34	7.77	7.71	7.50	7.89	7.81	7.72	8.09	8.20
4.76	7.02	7.06	7.46	7.34	7.68	7.33	7.69	7.39	7.51	7.93	7.95
4.81	6.60	6.77	7.06	7.21	7.06	7.17	7.42	7.39	7.14	7.56	7.73
4.86	6.56	6.41	6.86	7.35	6.99	6.96	7.26	7.12	6.97	7.97	7.58
4.91	6.78	7.05	6.94	7.37	7.28	6.89	7.45	7.24	7.35	7.82	7.92
4.96	6.99	6.89	6.99	6.94	7.19	7.37	7.59	7.12	7.32	7.65	7.64
5.01	6.66	7.06	6.83	7.24	7.60	7.31	7.39	7.44	7.19	7.55	7.62
5.06	6.59	6.82	6.99	6.81	7.23	6.91	7.20	7.11	7.36	7.43	7.47
5.11	6.22	6.71	6.66	7.04	6.74	6.88	6.80	7.02	7.06	7.21	7.38
5.16	6.32	6.82	6.78	6.93	6.87	7.15	6.95	6.94	6.92	7.36	7.49
5.21	6.65	6.61	6.92	6.90	7.05	6.77	7.15	7.14	6.93	7.31	7.29
5.26	6.93	6.98	6.84	7.59	7.56	7.40	7.26	7.59	7.70	7.92	7.76
5.31	6.67	6.69	7.03	7.25	7.38	7.15	7.46	7.22	7.17	7.53	7.46
5.38	6.73	6.47	6.85	6.68	6.83	6.65	6.98	6.74	6.75	7.10	7.14

5.45	6.81	7.15	7.24	7.22	7.30	7.38	7.51	7.14	7.42	7.69	7.66
5.53	6.86	6.99	6.90	7.09	7.24	7.16	7.27	7.36	7.21	7.46	7.73
5.61	7.04	6.99	7.26	7.36	7.38	7.38	7.56	7.56	7.31	7.79	7.93
5.70	6.75	6.75	6.73	6.70	7.10	7.18	7.30	7.37	7.08	7.57	7.78
5.80	6.63	6.74	6.88	7.13	6.99	7.17	7.48	7.26	7.14	7.58	7.32
5.90	6.54	6.54	6.63	6.73	6.80	6.84	6.88	7.17	6.65	7.36	7.53
6.01	6.96	6.98	7.48	7.38	7.43	7.10	7.41	7.39	7.43	7.92	8.20
6.13	6.59	6.60	6.80	7.23	7.18	6.96	7.18	7.16	7.09	7.55	7.40
6.25	6.16	6.54	6.27	6.60	6.71	6.60	7.00	7.11	6.96	7.00	7.24
6.39	6.11	6.26	6.48	6.52	6.32	6.32	6.57	6.60	6.71	6.75	6.98
6.53	6.29	6.32	6.48	6.48	6.22	6.64	6.66	6.72	6.92	6.80	6.87
6.69	6.72	7.17	7.00	7.12	7.30	7.10	7.03	7.06	6.95	7.42	7.60
6.85	6.29	6.20	6.69	6.66	6.55	6.79	7.01	6.99	6.78	7.30	7.16
7.03	6.19	6.22	6.67	6.57	6.73	6.57	6.83	6.75	6.60	7.19	7.24
7.21	6.17	6.25	6.29	6.72	6.72	6.55	6.77	6.69	6.88	7.03	6.98
7.41	6.25	6.16	6.68	6.72	6.80	6.66	7.14	7.01	6.93	7.38	7.36
7.63	6.58	6.89	7.12	6.85	7.10	7.43	7.33	7.35	7.17	7.57	7.52
7.86	6.60	6.45	6.74	6.69	6.56	6.57	6.81	6.95	7.04	7.03	7.22
8.10	6.18	6.11	6.37	6.52	6.66	6.57	6.82	6.67	6.67	6.80	7.12
8.36	5.74	6.08	5.89	5.83	6.10	6.25	6.24	6.29	6.23	6.58	6.50
8.64	5.88	6.49	6.53	6.43	6.33	6.65	6.88	6.64	6.76	7.40	6.94
8.94	6.43	7.07	6.76	7.16	7.23	7.05	7.59	7.11	7.43	7.50	7.50
9.26	6.65	6.57	6.66	7.14	6.97	7.10	7.16	7.16	7.12	7.50	7.72
9.60	6.26	6.30	6.13	6.34	6.64	6.27	6.78	6.95	6.59	6.95	7.08
9.97	6.06	5.54	6.20	6.54	6.24	6.32	6.62	6.48	6.39	6.75	6.73
10.36	6.17	6.49	6.59	6.44	6.61	6.59	7.01	6.95	6.83	7.48	7.29
10.77	6.10	6.29	6.52	6.62	6.57	6.51	6.78	6.90	6.74	7.26	6.94
11.22	5.90	6.07	6.25	6.60	6.33	6.33	7.04	6.76	6.53	7.09	7.00
11.70	5.96	6.15	6.42	6.55	6.54	6.67	6.68	6.75	6.85	7.25	7.02
12.21	5.66	5.70	6.03	5.99	6.02	6.36	6.25	6.30	6.28	6.52	6.71
12.75	5.78	5.78	5.85	6.20	6.01	6.01	6.46	6.54	6.53	6.48	6.86
13.33	6.22	6.28	6.36	6.41	6.54	6.47	6.83	6.66	6.54	7.19	7.46
13.95	6.15	6.23	6.43	6.37	6.56	6.47	6.96	6.61	6.65	6.96	7.11
14.62	5.62	5.79	5.77	6.42	6.03	6.32	6.26	6.56	6.23	6.51	6.52
15.33	5.31	5.81	5.89	5.74	5.76	6.15	6.39	5.94	5.99	6.50	6.75
16.09	5.48	5.83	5.96	6.17	6.06	6.18	6.35	6.19	6.16	6.61	6.48
16.90	5.82	6.09	6.21	6.14	6.14	6.32	6.62	6.58	6.56	6.79	6.69
17.77	5.76	5.92	6.05	6.19	6.01	6.28	6.29	6.65	6.47	6.50	6.70
18.70	5.58	5.72	5.59	6.14	5.89	6.06	6.59	6.19	6.24	6.50	6.38
19.69	5.64	5.56	5.64	5.81	6.11	5.92	6.07	6.16	6.05	6.15	6.19
20.75	5.53	5.43	5.52	5.73	5.94	5.65	6.33	6.37	6.22	6.15	6.19
21.89	5.75	5.94	6.07	6.04	6.35	6.10	6.31	6.40	6.68	6.87	6.86
23.10	6.29	6.22	6.06	6.55	6.57	6.70	6.74	6.67	6.84	7.02	6.97
24.39	5.76	5.87	5.83	5.65	6.12	6.50	6.47	5.98	6.25	6.64	6.54
25.78	5.58	5.58	5.92	5.78	6.07	6.00	6.02	6.11	6.14	6.39	6.24
27.26	5.37	5.44	5.76	5.95	5.74	6.00	6.15	5.93	6.17	6.45	6.53
28.84	5.37	5.45	5.83	5.82	6.00	5.93	6.02	6.04	6.20	6.43	6.45
30.53	5.57	5.73	6.11	6.16	6.12	5.93	6.26	6.45	6.14	6.70	6.54
32.34	5.70	5.57	5.94	5.91	5.93	5.90	5.71	6.02	6.20	6.30	6.47
34.27	5.39	5.53	5.62	6.15	6.05	6.07	6.17	5.96	6.27	6.15	6.46

36.34	5.20	5.29	5.60	5.82	5.86	5.93	6.11	6.17	5.63	6.37	6.24
38.55	5.46	5.80	5.63	6.03	5.97	5.78	6.32	6.12	6.24	6.52	6.18
40.91	5.56	5.49	5.62	5.65	5.91	5.99	6.22	6.02	6.20	6.25	6.70
43.43	5.22	5.07	5.56	5.49	5.63	5.36	5.43	5.86	5.42	5.83	6.03
46.13	5.87	5.73	5.67	5.73	5.67	5.85	6.10	6.11	5.87	6.46	6.36
49.01	5.29	5.23	5.59	5.74	5.79	6.05	6.07	5.89	6.21	6.33	6.27
52.09	5.20	5.27	5.61	5.57	5.58	5.76	6.04	6.14	6.03	6.08	6.07
55.39	5.15	5.37	5.38	5.67	5.66	5.96	5.92	5.56	5.86	6.15	6.24
58.91	5.19	5.27	5.54	5.62	5.50	5.54	5.77	5.86	5.92	5.95	5.97
62.68	5.20	5.20	5.32	5.35	5.41	5.70	5.76	5.42	5.69	6.16	6.06
66.70	4.95	5.32	5.45	5.70	5.80	5.73	5.97	6.19	6.12	6.15	6.12
71.00	4.98	5.31	5.18	5.50	5.84	5.66	5.95	5.96	6.07	5.97	6.18
75.60	5.36	5.14	5.29	5.58	5.53	5.42	5.85	5.73	5.40	6.15	6.09
80.51	5.23	5.47	5.36	5.92	5.76	5.64	5.85	5.92	6.02	6.27	6.07
85.77	5.00	5.19	5.37	5.64	5.59	5.41	5.77	5.83	5.85	6.08	6.15
91.38	5.18	5.28	5.34	5.41	5.74	5.50	5.78	5.90	5.83	6.16	6.05
97.39	5.10	5.01	5.18	5.43	5.74	5.39	5.77	5.64	5.57	5.77	6.02
103.80	5.40	5.59	5.40	5.81	5.83	5.77	6.11	6.03	5.94	6.29	6.60
110.66	5.20	5.01	5.38	5.49	5.77	5.75	6.05	5.95	5.79	6.10	6.29
117.99	4.84	5.15	5.26	5.21	5.55	5.62	5.83	5.50	5.44	5.60	5.93
125.83	4.83	5.30	5.36	5.59	5.58	5.65	6.14	6.01	5.84	6.01	5.82
134.21	5.04	5.22	5.37	5.75	5.51	5.47	5.53	5.70	5.81	5.96	5.87
143.17	5.15	5.46	5.42	5.43	5.52	5.57	5.77	5.47	5.61	5.82	6.23
152.74	4.92	5.12	5.25	5.35	5.46	5.44	5.71	5.45	5.70	5.94	5.78
162.97	5.25	4.96	4.98	5.53	5.30	5.24	5.67	5.53	5.55	6.02	6.11
173.91	4.79	5.00	5.04	5.06	5.24	5.31	5.55	5.44	5.43	5.66	6.10
185.60	4.92	5.24	5.22	5.38	5.39	5.51	5.28	5.43	5.51	5.88	5.76
198.10	5.24	5.25	5.43	5.20	5.26	5.27	5.44	5.66	5.73	5.85	5.91
211.46	5.50	5.49	5.26	5.56	5.35	5.64	6.03	5.95	5.72	6.19	6.22
225.75	4.80	5.22	4.99	5.04	5.51	5.22	5.67	5.46	5.02	5.52	5.53
241.01	5.22	4.82	4.98	5.40	5.41	5.18	5.38	5.16	5.25	5.69	5.81
257.33	4.70	4.98	5.20	5.08	5.27	4.95	5.70	5.64	5.20	5.43	5.66
274.77	4.89	5.05	5.17	5.30	5.06	5.34	5.50	5.43	5.15	5.79	5.59
293.42	5.01	5.08	5.62	5.35	5.69	5.39	5.64	5.46	5.84	5.87	5.71
313.35	5.08	4.83	5.45	5.30	5.27	5.37	5.58	5.59	5.58	5.94	5.72
334.66	5.24	4.97	5.33	5.29	5.59	5.43	5.59	5.52	5.35	5.88	5.90
357.43	4.68	4.73	4.78	5.09	5.34	5.22	5.18	5.46	5.51	5.49	5.60
381.78	4.63	4.81	4.71	4.79	5.02	5.11	5.38	5.06	5.12	5.55	5.26
407.80	4.86	4.81	4.84	4.82	4.87	4.94	5.12	5.47	5.21	5.46	5.40
435.62	4.82	4.91	4.98	5.55	5.48	5.33	5.49	5.33	5.31	5.75	5.51
465.35	4.78	5.06	4.89	5.20	5.25	5.18	5.55	5.36	5.37	5.46	5.55
497.14	4.87	4.74	5.02	5.31	5.06	5.12	5.13	5.22	5.15	5.44	5.69
531.11	4.64	5.00	4.53	4.85	5.02	4.92	4.98	5.20	5.02	5.43	5.11
567.43	4.86	4.70	5.08	4.77	4.93	5.14	5.26	5.30	5.09	5.18	5.56
606.26	5.09	5.00	4.86	5.11	5.32	4.92	5.44	5.35	5.03	5.12	5.36
647.76	4.97	5.25	5.39	5.42	5.38	5.01	5.45	5.44	5.54	5.48	5.81
692.12	4.91	5.14	4.96	5.01	5.15	4.83	5.28	5.46	5.45	5.48	5.45
739.53	4.88	5.19	5.35	5.30	5.14	5.01	5.41	5.25	5.35	5.67	5.65
790.22	4.41	4.59	4.73	4.86	4.86	4.65	5.10	4.88	4.77	5.38	5.07
844.40	4.51	4.88	4.64	5.01	4.94	4.99	5.12	5.09	5.01	5.09	5.38

902.32	4.99	4.95	5.03	5.16	5.48	5.12	5.83	5.42	5.48	5.82	5.81
964.23	4.34	4.34	4.58	4.82	4.88	5.06	5.08	5.09	4.89	5.07	5.11
1030.41	5.02	4.78	5.13	5.11	5.37	5.28	5.40	5.40	5.33	5.36	5.55
1101.15	4.59	5.08	4.91	5.01	5.10	5.07	5.28	5.33	5.15	5.23	5.45
1176.77	4.98	5.13	4.83	4.96	5.37	5.20	5.19	5.18	5.42	5.55	5.34
1257.60	5.11	5.00	5.01	5.00	5.16	5.09	5.38	5.22	5.50	5.52	5.48
1344.00	4.92	4.95	5.18	4.88	5.06	5.40	5.35	5.12	5.36	5.57	5.64
1436.36	4.73	4.60	4.91	5.03	4.94	5.00	5.46	5.18	5.30	5.40	5.51
1535.09	4.72	4.82	4.81	5.26	5.17	5.13	5.31	5.09	5.40	5.62	5.50
1640.63	4.76	4.86	4.54	4.97	4.89	5.02	5.03	5.15	5.13	5.41	5.31
1753.44	4.87	4.86	4.75	4.84	4.93	4.94	5.22	5.02	4.87	5.19	5.35
1874.02	4.24	4.48	4.71	4.76	4.80	4.65	4.78	4.60	4.77	4.91	5.07
2002.92	4.62	4.88	4.86	5.01	4.92	5.12	5.27	5.18	5.17	5.40	5.68
2140.71	4.78	4.60	4.74	5.19	4.91	5.12	5.32	5.13	5.15	5.37	5.31
2288.00	4.78	4.63	4.59	4.78	4.86	4.98	4.94	4.95	5.26	5.52	5.12
2445.44	4.44	4.44	4.62	4.59	4.79	4.93	5.03	5.16	5.03	5.04	5.03
2613.73	4.94	4.80	4.76	5.28	5.04	4.86	5.28	5.31	5.10	5.32	5.57
2793.63	4.60	4.43	4.70	4.88	4.91	4.66	5.09	5.14	4.85	5.52	5.22
2985.93	4.81	4.86	4.97	5.14	5.23	4.61	5.20	5.15	4.95	5.23	5.47
3191.49	4.78	5.06	4.85	5.04	5.39	5.34	5.01	5.34	5.15	5.47	5.56
3411.21	5.27	5.09	5.03	4.95	5.28	5.30	5.37	5.44	5.45	5.55	5.51
3646.09	5.28	5.15	5.30	5.15	5.42	5.31	5.46	5.46	5.27	5.60	5.74
3897.16	5.88	5.70	5.56	5.43	5.94	5.89	5.56	5.59	5.79	5.81	6.30

Wavelength (nm) / Time (ps)	*396.75*	*398.17*	*399.60*	*401.02*	*402.44*	*403.87*	*405.29*	*406.72*	*408.14*	*409.56*	*410.99*
-2.74	*-0.06*	*0.27*	*0.02*	*-0.01*	*0.14*	*0.30*	*0.32*	*0.46*	*0.42*	*0.16*	*0.11*
-2.24	*0.35*	*-0.03*	*0.21*	*0.44*	*-0.18*	*0.14*	*0.36*	*0.03*	*0.49*	*0.20*	*0.40*
-1.74	*-0.29*	*0.03*	*0.16*	*-0.10*	*0.24*	*-0.22*	*-0.06*	*-0.11*	*-0.22*	*-0.20*	*-0.05*
-1.24	*-0.09*	*-0.07*	*-0.14*	*-0.06*	*-0.34*	*0.02*	*-0.18*	*-0.05*	*-0.33*	*-0.17*	*-0.24*
-0.74	*0.07*	*0.08*	*0.07*	*0.11*	*0.47*	*0.17*	*0.35*	*0.11*	*0.13*	*0.28*	*-0.13*
-0.24	*0.11*	*-0.25*	*-0.08*	*-0.22*	*-0.10*	*-0.23*	*-0.52*	*-0.37*	*-0.03*	*-0.13*	*0.14*
-0.14	*0.13*	*0.12*	*-0.05*	*-0.19*	*-0.06*	*0.01*	*-0.21*	*0.26*	*-0.02*	*0.15*	*0.13*
-0.04	*-0.22*	*-0.15*	*-0.19*	*0.03*	*-0.17*	*-0.19*	*-0.06*	*-0.33*	*-0.42*	*-0.30*	*-0.36*
0.06	*0.04*	*0.02*	*0.24*	*0.24*	*0.22*	*0.18*	*0.04*	*0.00*	*-0.08*	*0.04*	*0.19*
0.16	*0.40*	*0.25*	*0.26*	*0.57*	*0.62*	*0.28*	*0.35*	*0.24*	*0.74*	*0.28*	*0.77*
0.26	*-0.02*	*0.23*	*-0.24*	*-0.15*	*0.29*	*0.12*	*-0.01*	*0.23*	*-0.02*	*0.12*	*0.15*
0.31	*0.00*	*0.03*	*-0.11*	*0.07*	*-0.11*	*-0.21*	*-0.33*	*-0.34*	*-0.05*	*-0.06*	*-0.34*
0.36	*-0.08*	*-0.03*	*0.15*	*-0.02*	*-0.16*	*-0.19*	*-0.12*	*-0.13*	*0.00*	*0.07*	*0.04*
0.41	*0.16*	*-0.03*	*0.02*	*0.24*	*0.21*	*0.33*	*-0.47*	*-0.17*	*-0.05*	*-0.08*	*-0.22*
0.46	*-0.04*	*-0.17*	*-0.21*	*0.32*	*0.13*	*-0.09*	*-0.58*	*0.26*	*-0.09*	*-0.14*	*-0.29*
0.51	*-0.22*	*0.05*	*0.10*	*-0.36*	*-0.23*	*-0.19*	*-0.47*	*-0.16*	*-0.33*	*-0.09*	*-0.09*
0.56	*0.21*	*0.46*	*0.38*	*0.16*	*0.31*	*0.33*	*-0.31*	*0.15*	*-0.02*	*-0.07*	*-0.03*
0.61	*0.00*	*0.19*	*0.26*	*-0.14*	*0.16*	*0.37*	*0.07*	*0.07*	*0.16*	*0.20*	*0.22*
0.66	*0.20*	*0.32*	*-0.13*	*0.05*	*0.00*	*0.42*	*-0.32*	*0.14*	*0.02*	*0.19*	*0.35*
0.71	*0.01*	*0.04*	*0.04*	*0.15*	*-0.14*	*0.27*	*0.28*	*-0.31*	*0.33*	*0.06*	*-0.05*
0.76	*-0.49*	*-0.13*	*0.70*	*0.20*	*0.19*	*0.71*	*0.37*	*0.27*	*0.65*	*0.66*	*0.32*
0.81	*-8.62*	*-12.54*	*-3.13*	*5.82*	*2.10*	*-1.67*	*-0.05*	*0.54*	*-0.05*	*0.10*	*-0.18*
0.86	*-85.44*	*-136.46*	*-155.32*	*-123.58*	*-55.71*	*-1.69*	*10.02*	*0.95*	*-2.68*	*-1.14*	*0.16*

0.91	367.78	290.88	171.01	46.08	-52.57	-111.11	-128.07	-111.05	-75.50	-38.54	-12.12
0.96	175.39	314.67	423.64	462.59	439.99	398.06	359.82	334.88	322.06	313.41	299.14
1.01	-15.02	-65.46	-110.70	-133.01	-125.42	-94.19	-50.56	-7.49	29.07	64.41	108.77
1.06	11.41	12.82	12.95	8.50	2.74	2.54	6.37	-0.08	-32.67	-90.69	-157.18
1.11	11.87	11.15	10.73	10.19	9.66	8.16	4.68	0.30	-0.50	4.52	11.44
1.16	12.30	11.32	9.80	8.61	8.33	10.30	13.31	15.11	14.94	11.81	9.99
1.21	11.40	11.48	11.71	11.76	10.56	8.94	8.11	9.93	14.09	17.02	15.25
1.26	10.72	10.00	9.46	9.51	10.81	12.11	13.08	11.41	8.66	6.48	7.62
1.31	10.17	10.40	10.00	9.35	8.09	7.50	8.54	10.49	11.40	11.06	8.73
1.36	9.93	10.27	10.48	10.79	9.68	7.96	6.34	6.26	8.49	11.75	13.09
1.41	9.56	8.70	7.83	7.96	8.89	10.69	11.40	10.53	8.24	6.80	7.72
1.46	9.61	9.80	9.82	9.13	7.78	6.20	6.59	8.92	11.79	12.00	9.46
1.51	9.28	8.84	8.78	9.63	10.22	10.35	9.05	7.25	5.69	6.93	9.08
1.56	9.92	9.40	8.44	8.08	8.32	9.31	10.49	10.71	9.36	7.07	6.57
1.61	9.27	9.49	9.40	8.53	7.70	6.83	7.61	8.82	10.96	10.96	9.43
1.66	9.02	8.53	8.57	9.67	9.78	9.54	8.63	6.95	6.24	7.36	9.18
1.71	9.41	9.24	8.73	7.98	7.35	7.13	8.02	9.35	10.16	9.30	7.35
1.76	9.24	9.23	9.46	9.71	9.49	8.43	7.16	6.51	7.93	9.62	11.21
1.81	8.32	7.72	7.37	7.46	7.81	8.58	8.79	7.87	6.81	6.18	6.96
1.86	8.58	8.82	8.59	8.03	7.49	7.27	8.01	9.33	9.27	8.07	7.08
1.91	7.91	8.36	8.46	8.63	8.55	7.62	6.44	5.59	5.87	7.70	9.33
1.96	8.82	8.38	7.77	7.53	7.39	8.07	8.72	8.76	8.27	7.20	6.85
2.01	8.86	8.83	8.80	8.71	7.60	7.02	6.62	7.04	8.87	10.10	9.46
2.06	8.40	8.42	8.10	8.73	8.91	9.29	8.27	7.40	6.95	6.79	7.91
2.11	8.37	8.27	8.25	7.59	7.26	7.37	8.09	8.20	8.26	7.57	6.74
2.16	8.49	8.58	8.45	8.49	7.89	7.73	6.76	6.69	7.62	9.15	9.54
2.21	8.80	8.22	7.67	7.74	8.47	8.21	8.49	8.21	7.29	6.59	7.23
2.26	8.82	9.10	8.20	8.43	7.13	6.71	7.14	8.28	9.10	9.32	8.01
2.31	8.08	8.05	7.73	7.90	7.84	7.41	6.71	6.59	6.87	7.43	8.44
2.36	7.81	7.81	7.17	7.31	7.55	7.18	7.03	6.73	6.35	6.08	7.21
2.41	8.13	8.32	8.25	7.36	7.05	6.95	6.43	7.31	8.47	8.36	7.59
2.46	8.04	7.41	7.60	8.07	7.67	7.39	7.36	6.43	6.22	6.72	7.61
2.51	8.76	8.98	8.37	7.90	7.11	6.95	6.98	7.98	8.37	8.50	7.19
2.56	8.11	7.99	7.68	8.14	7.56	7.20	6.79	6.95	7.60	8.38	8.68
2.61	7.92	7.85	7.55	7.62	7.59	7.40	7.10	6.20	6.26	6.94	7.69
2.66	7.92	7.91	7.55	6.82	6.78	7.06	7.07	7.91	8.20	7.65	6.83
2.71	8.17	8.31	7.98	8.04	7.81	7.49	6.74	7.18	6.86	8.44	8.82
2.76	8.26	7.86	7.58	7.29	7.40	7.66	7.81	7.37	7.17	6.68	6.69
2.81	7.62	8.02	7.57	7.36	6.54	6.58	6.89	7.15	7.36	7.42	7.09
2.86	7.39	7.53	7.54	7.27	6.71	6.74	6.80	6.17	6.63	7.18	7.43
2.91	8.03	7.72	7.24	7.08	7.19	7.27	7.60	7.40	7.52	6.79	6.70
2.96	7.94	7.94	8.03	7.77	7.31	7.40	6.72	6.90	7.53	8.06	8.00
3.01	8.27	7.81	7.53	7.13	7.18	6.94	7.31	7.31	6.82	6.57	6.77
3.06	7.76	7.55	7.52	7.15	6.88	6.93	6.71	7.41	7.36	7.22	6.93
3.11	7.88	7.47	7.72	7.75	7.06	6.59	6.54	6.42	7.03	7.56	7.68
3.16	7.56	7.25	7.22	6.98	7.09	7.03	7.29	6.93	6.82	6.45	6.65
3.21	8.23	7.97	7.40	7.50	7.04	6.99	7.15	7.57	7.58	7.65	7.45
3.26	7.90	7.92	7.24	7.41	7.24	7.40	6.91	6.53	6.59	6.59	7.54
3.31	7.82	7.60	7.49	7.41	7.24	7.64	7.04	7.26	7.43	6.99	7.01
3.36	7.85	7.99	7.70	7.40	6.90	6.49	6.42	7.18	7.31	7.37	7.25

3.41	7.71	7.50	7.44	7.85	7.12	7.37	7.23	6.77	6.39	7.27	7.44
3.46	8.14	8.02	7.64	7.54	7.06	7.38	7.58	7.20	7.80	7.17	6.96
3.51	7.65	7.70	7.55	7.71	7.29	6.94	6.28	6.44	6.90	7.07	7.41
3.56	7.80	7.65	7.06	7.50	6.91	7.06	7.13	6.55	6.65	7.02	6.91
3.61	7.79	7.17	7.41	6.64	6.33	6.26	6.04	6.72	6.72	6.63	6.41
3.66	7.82	7.62	7.68	7.38	7.39	6.79	6.86	6.93	6.60	7.22	7.41
3.71	8.21	8.05	7.59	7.50	7.13	6.93	6.97	7.23	7.52	6.77	7.07
3.76	8.11	8.15	8.12	7.55	7.85	7.62	7.09	7.15	7.59	7.84	8.13
3.81	7.44	7.57	7.27	7.14	7.14	7.14	6.48	6.56	6.93	7.10	7.23
3.86	7.16	7.15	6.82	6.52	6.63	6.60	6.29	6.31	6.60	6.66	6.47
3.91	7.77	7.80	7.78	7.53	7.49	6.91	6.50	6.87	7.09	7.28	7.52
3.96	7.74	7.77	7.28	7.36	6.95	7.08	6.44	6.72	6.70	6.55	6.94
4.01	8.09	7.91	7.60	7.57	7.36	7.19	7.09	7.25	7.59	7.43	7.04
4.06	7.80	7.80	7.79	7.73	7.37	7.13	6.78	6.59	6.98	7.37	7.41
4.11	7.56	7.53	7.47	6.85	7.08	7.35	6.59	6.98	6.86	7.05	7.01
4.16	7.79	7.75	7.73	7.25	6.87	6.68	6.63	6.63	7.56	7.63	7.18
4.21	7.90	8.30	8.24	7.75	7.71	7.55	7.33	7.08	7.16	7.41	7.60
4.26	7.97	7.90	7.57	7.65	7.34	6.89	7.18	7.31	7.36	7.32	7.46
4.31	7.35	7.34	7.26	7.27	6.99	6.52	6.23	6.62	6.71	6.70	6.94
4.36	7.40	7.22	6.87	6.99	6.97	6.49	6.39	6.40	6.47	6.63	7.00
4.41	7.39	7.27	7.09	6.94	6.65	6.61	6.30	7.04	6.83	6.95	6.71
4.46	8.04	7.85	7.81	7.57	7.47	7.25	6.41	6.63	6.98	7.62	7.37
4.51	7.88	7.82	7.42	7.13	6.76	6.83	6.83	6.52	6.70	6.21	6.66
4.56	7.46	7.48	7.56	7.27	7.04	7.09	6.20	6.75	7.20	6.85	6.93
4.61	7.32	7.32	7.32	7.12	6.98	6.19	6.77	6.61	6.76	7.07	7.07
4.66	7.58	7.74	7.32	6.94	7.22	6.89	6.71	6.85	6.84	6.89	7.01
4.71	7.66	7.80	7.68	7.86	7.32	7.02	6.96	7.31	7.67	7.67	7.92
4.76	7.91	7.79	7.43	7.25	7.22	6.94	6.82	6.63	6.91	6.90	7.24
4.81	7.68	7.30	7.31	7.06	6.88	6.75	6.74	6.70	6.90	7.13	6.67
4.86	7.64	7.67	7.31	7.28	6.61	6.66	6.57	6.43	6.56	7.10	7.00
4.91	7.47	7.60	7.44	7.12	6.67	7.07	6.77	6.65	6.47	6.66	6.66
4.96	7.97	7.71	7.12	6.95	6.85	7.22	7.12	6.88	7.01	6.93	7.05
5.01	7.81	7.62	7.18	7.43	7.33	7.54	6.89	6.60	7.05	6.94	7.09
5.06	7.39	7.38	7.26	7.04	6.64	6.60	6.50	6.49	6.39	6.61	6.42
5.11	7.26	7.07	7.09	6.70	6.44	6.16	6.24	6.15	6.68	6.29	6.54
5.16	7.44	6.96	7.07	7.25	6.62	6.52	6.51	6.46	6.63	6.67	6.89
5.21	7.16	7.22	6.73	7.01	6.47	6.06	6.23	6.14	6.57	6.50	6.24
5.26	7.65	8.16	7.44	7.74	7.00	7.01	6.96	7.26	7.36	7.06	7.43
5.31	7.67	7.39	7.04	7.09	6.66	6.92	6.52	6.62	6.80	6.76	6.82
5.38	7.31	7.10	6.91	6.88	6.75	6.86	5.82	6.55	6.38	6.81	6.45
5.45	7.52	7.57	7.12	6.92	6.73	6.68	6.71	6.30	6.78	7.11	7.17
5.53	7.41	7.61	7.18	7.21	6.76	6.87	6.83	6.69	6.59	7.17	6.74
5.61	7.93	7.69	7.74	7.39	7.21	6.51	6.96	6.70	7.14	7.21	7.12
5.70	7.55	7.50	7.17	7.15	7.05	6.62	6.94	6.73	6.54	7.04	7.08
5.80	7.46	7.46	7.04	6.78	6.80	6.56	6.29	6.58	6.84	7.01	6.76
5.90	7.40	7.00	6.73	6.79	6.86	6.12	6.14	6.06	6.31	6.74	6.50
6.01	8.07	8.07	7.59	7.51	7.05	7.13	6.80	7.14	7.01	7.29	7.34
6.13	7.60	7.31	7.27	6.92	6.49	6.91	6.50	6.66	6.76	7.07	7.21
6.25	6.77	7.16	6.47	6.45	6.59	6.55	6.13	6.53	6.24	7.04	6.65
6.39	6.97	6.84	6.35	6.42	6.15	6.07	5.92	6.11	6.12	6.37	6.48

6.53	7.21	6.92	6.37	6.31	6.41	6.10	5.83	6.11	6.35	6.55	6.40
6.69	7.65	7.37	7.14	7.32	6.91	6.77	6.65	6.78	6.51	6.66	6.90
6.85	7.26	7.02	6.97	6.74	6.48	6.84	6.43	6.25	6.59	6.51	6.42
7.03	7.08	7.06	6.52	6.74	6.43	6.40	5.79	6.30	6.39	6.30	6.40
7.21	6.86	6.82	6.41	6.59	6.09	6.14	6.10	5.77	6.12	6.05	6.34
7.41	7.17	6.83	6.97	6.73	6.95	6.69	6.43	6.34	6.75	6.77	6.94
7.63	7.50	7.33	6.98	7.34	6.69	6.78	6.31	6.30	6.74	6.70	7.16
7.86	7.16	7.08	6.82	6.71	6.91	6.56	6.46	6.42	6.55	6.50	6.88
8.10	7.10	6.75	6.77	6.52	6.37	5.84	6.00	5.70	6.41	6.40	6.57
8.36	6.98	6.60	6.34	6.22	6.14	6.20	5.93	6.18	5.97	5.97	5.94
8.64	7.08	7.20	7.05	6.80	6.36	6.37	6.37	6.44	6.62	6.52	6.63
8.94	7.61	7.52	7.50	7.06	7.21	6.76	7.04	7.17	7.14	7.50	7.10
9.26	7.51	7.31	7.25	7.61	6.97	6.80	6.64	6.87	6.83	7.29	7.23
9.60	6.91	6.79	7.09	6.51	6.69	6.16	6.46	6.21	6.25	6.67	6.61
9.97	6.71	6.67	6.32	6.34	6.35	5.65	6.11	5.84	5.91	5.99	5.67
10.36	7.18	7.26	7.12	6.79	6.77	6.71	6.39	6.53	7.25	6.78	6.82
10.77	7.08	7.43	6.91	6.62	6.69	6.48	6.08	6.27	6.35	6.04	6.86
11.22	6.86	6.60	6.78	6.50	6.01	6.20	6.44	6.02	6.25	6.27	6.34
11.70	7.04	6.99	6.98	6.68	6.71	6.62	6.45	6.40	6.69	6.72	6.86
12.21	6.60	6.57	6.35	6.28	6.01	6.02	5.92	6.02	6.09	6.30	6.10
12.75	6.68	6.84	6.58	6.72	6.17	6.10	5.48	6.04	5.84	6.21	5.97
13.33	7.14	6.92	6.86	6.74	6.61	6.86	5.78	6.51	6.31	6.28	6.54
13.95	6.99	7.04	6.69	6.53	6.55	5.87	6.27	6.51	6.26	6.74	6.45
14.62	6.62	6.47	6.29	6.13	6.37	5.60	5.54	5.58	5.75	6.06	6.08
15.33	6.22	6.09	6.18	6.10	6.21	5.99	5.65	5.34	5.74	5.92	5.88
16.09	6.72	6.51	6.33	6.30	5.86	5.84	6.05	5.71	5.74	6.08	5.96
16.90	6.68	6.48	6.34	6.38	6.04	5.68	5.92	5.88	5.35	6.11	6.13
17.77	7.00	6.89	6.40	6.52	6.29	6.10	6.21	5.76	6.13	6.22	6.17
18.70	6.58	6.38	6.25	6.04	6.04	5.92	5.63	5.85	5.67	6.03	5.92
19.69	6.17	6.52	6.14	6.17	5.89	5.79	5.20	5.18	5.37	5.78	5.61
20.75	6.21	6.34	5.93	5.71	5.63	5.41	5.40	5.50	5.71	5.73	5.54
21.89	6.91	6.69	6.46	6.29	5.77	6.02	5.62	5.89	6.06	6.18	5.93
23.10	7.04	6.83	6.83	6.62	6.41	6.11	6.37	6.46	6.58	6.44	6.44
24.39	6.52	6.49	6.52	6.06	5.65	5.70	5.67	5.73	5.71	6.30	6.03
25.78	6.41	6.43	6.03	5.80	5.83	5.64	5.29	5.14	5.66	5.91	5.50
27.26	6.54	6.31	6.50	6.14	5.91	5.65	5.65	5.76	5.65	6.11	6.25
28.84	6.37	6.39	6.25	5.74	5.92	5.36	5.84	5.49	5.66	5.94	5.89
30.53	6.48	6.63	6.29	6.25	5.80	5.70	6.04	5.58	6.13	5.88	6.36
32.34	6.46	6.40	5.94	5.88	5.59	5.60	5.62	5.55	5.84	5.70	5.93
34.27	6.64	6.47	6.29	6.05	5.65	5.84	5.50	5.68	6.06	5.98	5.79
36.34	6.65	6.29	5.74	6.00	5.64	5.77	5.26	5.67	5.93	5.69	5.98
38.55	6.43	6.35	6.11	5.78	6.12	5.41	5.22	5.52	5.63	5.69	5.77
40.91	6.39	6.36	6.31	6.22	6.20	5.96	5.94	6.10	5.72	5.71	5.85
43.43	6.11	5.91	5.85	5.55	5.18	5.53	4.95	4.83	5.30	5.22	5.33
46.13	6.57	6.37	5.92	5.97	5.75	5.57	5.48	5.70	5.84	5.99	6.00
49.01	6.34	6.42	6.21	6.39	5.63	5.30	5.43	5.80	5.88	6.07	5.97
52.09	6.51	6.07	5.95	5.82	5.41	5.72	5.49	5.04	5.24	5.49	5.48
55.39	6.09	6.00	5.83	5.85	5.56	5.85	5.30	5.57	5.34	5.46	5.43
58.91	6.13	5.77	5.69	5.77	5.55	5.09	5.11	5.24	5.59	5.19	5.60
62.68	5.87	6.05	5.62	5.46	5.48	5.21	5.05	5.05	4.74	5.44	5.10

66.70	6.23	5.96	5.70	5.89	5.48	5.56	5.46	4.99	5.63	5.80	5.52
71.00	6.52	6.00	5.86	5.95	5.60	5.52	5.07	5.02	5.58	5.62	5.70
75.60	5.91	5.68	5.78	5.61	5.19	5.43	5.05	5.11	5.26	5.31	5.62
80.51	6.14	6.23	6.16	5.42	5.48	5.13	5.45	5.48	5.28	5.36	5.63
85.77	5.92	5.74	5.74	5.62	5.32	5.11	4.65	5.19	5.29	5.17	5.29
91.38	5.82	6.23	5.58	5.86	5.52	5.54	4.99	5.16	5.29	5.32	5.54
97.39	5.97	5.74	5.84	5.65	5.28	5.02	5.31	5.11	5.03	5.12	5.20
103.80	6.29	6.22	6.33	5.99	5.87	5.80	5.57	5.79	5.86	5.86	5.73
110.66	6.06	6.25	6.31	5.71	5.56	5.44	5.60	5.32	5.46	5.77	5.65
117.99	5.58	5.58	5.38	5.79	5.31	5.02	4.62	4.94	4.97	4.94	5.04
125.83	5.83	5.82	5.83	5.60	5.79	5.31	5.42	5.53	5.77	5.44	5.73
134.21	5.98	5.72	5.88	5.34	5.18	5.71	4.97	4.95	5.41	5.51	5.33
143.17	5.69	5.89	5.70	5.54	5.24	5.24	4.77	4.88	5.09	5.52	5.21
152.74	6.06	5.79	5.83	5.60	5.30	5.55	5.00	5.38	5.24	5.03	5.27
162.97	5.68	5.50	5.67	5.76	4.97	5.23	4.47	5.11	5.38	4.98	5.29
173.91	5.59	5.48	5.54	5.03	5.24	4.82	4.97	4.78	5.26	5.12	5.32
185.60	5.93	5.67	5.28	5.20	5.47	4.88	5.00	5.18	4.85	4.94	5.08
198.10	5.96	5.90	5.46	5.45	4.81	5.06	5.11	5.12	5.09	4.83	5.21
211.46	6.36	6.20	5.93	5.94	6.04	5.39	5.43	5.10	5.39	5.98	5.56
225.75	5.80	5.68	5.11	5.14	5.13	4.85	4.95	4.48	4.72	4.98	4.83
241.01	5.67	5.74	5.44	5.40	4.59	5.39	5.10	4.94	4.65	4.93	5.13
257.33	5.64	5.59	5.50	5.30	5.18	4.84	4.70	4.56	4.75	5.07	4.67
274.77	5.51	5.64	5.05	5.19	5.15	4.65	4.70	4.70	4.60	4.80	4.75
293.42	5.74	5.84	5.44	5.21	5.69	4.93	4.68	5.03	5.31	5.12	5.17
313.35	5.89	5.72	5.81	5.15	5.32	5.24	5.51	5.07	5.65	5.42	5.53
334.66	5.69	5.36	5.30	5.27	5.39	5.19	4.85	4.66	4.87	4.87	4.85
357.43	5.46	5.37	5.09	4.90	4.88	4.23	4.75	4.91	4.87	4.64	4.82
381.78	5.35	5.50	5.15	5.58	5.08	5.12	4.46	4.30	4.79	4.89	4.72
407.80	5.61	5.17	5.44	5.09	4.89	4.37	4.83	4.67	4.28	4.31	4.61
435.62	5.87	5.72	5.36	5.16	4.97	5.02	4.47	4.43	4.59	4.61	4.65
465.35	5.78	5.46	5.18	5.25	5.08	4.60	4.97	5.01	4.83	5.03	5.04
497.14	5.52	5.35	5.01	4.80	5.05	4.73	4.40	4.84	5.11	5.01	4.68
531.11	5.15	4.86	4.89	5.01	4.94	4.63	4.35	4.48	4.27	4.34	4.77
567.43	5.55	5.13	5.01	4.74	4.69	4.84	4.71	4.42	4.69	4.58	4.65
606.26	5.30	5.30	4.96	4.80	4.91	4.43	4.34	4.66	4.27	4.82	4.85
647.76	5.90	5.94	5.50	5.09	5.16	4.73	5.10	4.88	5.31	4.99	5.20
692.12	5.68	5.24	5.04	5.05	5.07	4.69	4.30	5.01	4.61	5.12	5.28
739.53	5.61	5.90	5.59	5.30	5.31	4.87	4.84	4.84	4.75	5.15	5.01
790.22	5.17	5.19	4.78	4.76	4.71	4.47	3.98	4.14	4.31	4.40	4.50
844.40	5.35	5.02	5.04	5.07	4.76	4.38	4.58	4.45	4.65	4.47	4.51
902.32	5.53	5.58	5.32	5.17	4.64	4.62	4.96	5.00	5.21	5.15	5.18
964.23	5.26	5.11	4.73	4.33	4.38	4.12	3.75	3.97	4.71	4.07	4.13
1030.41	5.75	5.43	5.31	5.56	5.23	4.87	4.46	4.58	4.71	4.99	4.94
1101.15	5.21	5.11	5.23	5.16	5.04	5.03	4.81	4.77	4.72	4.75	4.86
1176.77	5.48	5.26	5.38	4.75	5.08	4.93	4.76	4.37	4.41	4.66	4.83
1257.60	5.58	5.62	5.02	4.89	5.13	4.95	4.33	4.89	4.80	4.68	4.62
1344.00	5.87	5.60	5.45	4.71	4.89	4.76	4.28	4.87	5.24	4.90	5.09
1436.36	5.51	5.53	5.09	5.21	4.87	4.94	4.89	4.68	5.01	4.94	4.82
1535.09	5.52	5.52	5.29	4.94	5.01	4.46	4.48	5.07	4.89	4.78	4.65
1640.63	5.16	5.20	4.85	4.56	4.36	4.08	4.19	4.38	4.35	4.13	4.31

1753.44	5.34	5.22	4.85	5.02	4.67	4.71	4.64	4.79	4.77	4.63	4.90
1874.02	4.96	4.97	4.72	4.82	4.26	3.80	3.47	3.64	3.79	3.98	3.91
2002.92	5.40	5.29	5.27	5.10	5.14	4.61	4.56	4.63	5.09	4.63	4.97
2140.71	5.32	5.46	4.98	5.12	4.51	4.71	4.62	4.68	4.98	4.59	4.70
2288.00	5.22	5.16	5.07	4.69	4.73	4.67	4.52	4.28	4.71	4.45	4.42
2445.44	5.01	5.10	5.07	4.66	4.25	4.53	4.17	4.19	4.24	4.25	4.40
2613.73	5.77	5.57	5.14	5.25	4.55	4.52	4.84	4.58	4.72	4.86	4.72
2793.63	5.53	5.27	5.43	4.79	4.83	4.83	4.45	4.40	4.24	4.61	4.50
2985.93	5.39	5.08	5.10	5.00	4.72	4.50	4.25	4.48	4.55	4.72	4.29
3191.49	5.43	5.38	5.12	4.72	4.53	4.48	4.06	4.41	4.15	4.43	4.25
3411.21	5.59	5.58	5.13	5.17	4.86	4.54	4.93	4.51	4.52	5.01	4.88
3646.09	5.88	5.49	5.25	5.32	4.78	4.80	4.27	4.61	4.64	4.87	4.70
3897.16	6.10	6.01	5.47	5.71	5.31	4.87	4.88	4.58	5.37	4.92	5.60

Wavelength (nm) / Time (ps)	412.41	413.84	415.26	416.68	418.11	419.53	420.96	422.38	423.80	425.23	426.65
-2.74	0.29	0.35	0.25	0.12	0.31	0.13	0.20	0.19	0.22	0.10	0.20
-2.24	0.08	0.19	0.16	0.26	0.22	0.14	0.16	0.11	0.05	0.03	-0.08
-1.74	-0.14	-0.39	-0.35	-0.33	-0.40	-0.38	-0.36	-0.29	-0.41	-0.40	-0.39
-1.24	-0.12	-0.02	-0.19	-0.16	-0.11	-0.06	-0.11	-0.02	-0.03	0.00	0.15
-0.74	0.30	0.45	0.61	0.21	0.20	0.24	0.08	0.03	0.33	0.23	0.21
-0.24	-0.15	-0.09	0.00	-0.01	0.05	0.02	0.04	0.01	0.00	0.09	-0.02
-0.14	0.08	-0.01	-0.10	0.13	0.09	0.09	0.26	0.20	0.13	0.20	0.19
-0.04	-0.35	-0.47	-0.38	-0.23	-0.36	-0.16	-0.28	-0.22	-0.31	-0.26	-0.26
0.06	0.09	0.17	0.16	0.07	0.18	0.36	0.27	0.32	0.32	0.12	0.16
0.16	0.48	0.81	0.52	0.52	0.56	0.60	0.53	0.50	0.38	0.39	0.20
0.26	0.24	0.19	0.18	0.22	0.26	0.30	0.12	0.26	0.14	0.21	0.17
0.31	-0.30	-0.48	-0.40	-0.28	-0.38	-0.35	-0.39	-0.26	-0.46	-0.31	-0.31
0.36	-0.30	-0.21	-0.11	0.05	-0.12	-0.03	-0.02	0.02	-0.14	-0.15	-0.03
0.41	0.13	0.10	0.15	0.07	0.07	0.22	0.18	0.18	0.21	0.23	0.16
0.46	-0.08	-0.14	-0.22	-0.05	-0.08	-0.02	-0.15	-0.12	-0.12	-0.04	-0.12
0.51	-0.09	-0.03	-0.34	-0.38	-0.17	0.00	-0.07	-0.03	-0.09	-0.11	0.01
0.56	0.12	0.15	0.10	0.10	0.27	0.26	0.14	0.22	0.21	-0.02	0.18
0.61	0.04	0.26	0.17	0.33	0.26	0.47	0.34	0.35	0.24	0.09	0.18
0.66	0.21	0.19	0.13	0.16	0.30	0.21	0.31	0.25	0.22	0.15	0.09
0.71	0.39	0.27	-0.11	0.00	0.06	0.34	0.17	0.28	0.23	0.25	0.14
0.76	0.51	0.30	0.48	0.22	0.19	0.12	0.13	0.27	0.14	0.11	0.05
0.81	0.33	0.27	0.16	0.22	0.26	0.31	0.20	0.12	0.09	-0.08	0.05
0.86	-0.10	-0.18	0.38	0.19	-0.32	-0.25	0.15	0.25	0.18	0.12	0.21
0.91	3.03	10.45	10.63	4.35	-4.45	-8.20	-4.71	1.26	2.57	0.38	-0.64
0.96	272.90	233.23	181.58	120.78	55.55	-6.69	-52.60	-72.01	-65.01	-42.34	-18.55
1.01	167.99	244.20	333.76	421.34	482.04	492.06	450.33	375.27	287.02	197.22	114.22
1.06	-209.46	-231.49	-218.29	-171.96	-97.64	-2.64	102.14	205.35	298.32	377.93	441.06
1.11	13.37	11.61	13.58	22.42	29.75	19.72	-13.80	-61.53	-104.33	-125.35	-117.56
1.16	11.82	13.83	13.55	11.40	7.96	4.92	6.50	13.52	18.75	17.28	13.27
1.21	10.76	7.35	8.25	11.57	14.87	15.96	14.80	13.25	12.47	13.06	14.02
1.26	10.88	13.41	13.23	11.61	10.48	11.07	12.77	14.19	14.17	12.11	9.51
1.31	7.91	9.49	11.96	13.51	13.21	11.51	9.83	9.28	10.24	11.41	12.19

1.36	11.95	9.26	8.23	9.20	11.09	12.16	11.41	9.98	8.65	9.01	10.43
1.41	10.62	12.13	11.65	9.64	8.07	8.26	9.99	11.80	12.25	11.44	9.86
1.46	6.92	6.38	8.46	11.24	12.45	11.59	9.66	8.65	9.17	10.57	11.54
1.51	11.34	11.04	9.33	8.33	8.72	10.09	11.26	11.54	9.97	8.62	7.92
1.56	9.40	11.81	12.44	10.97	9.03	8.00	8.71	10.32	11.31	10.97	9.67
1.61	7.11	6.74	8.47	10.20	10.74	10.19	8.66	8.31	8.79	10.02	10.91
1.66	10.36	9.84	8.42	7.83	8.57	10.11	11.02	10.86	9.52	8.13	7.63
1.71	6.71	8.00	9.89	10.88	10.59	9.46	8.28	8.41	9.00	9.75	9.74
1.76	10.56	8.90	7.96	8.51	9.62	10.72	10.17	9.17	8.06	7.82	8.69
1.81	8.80	9.42	8.80	7.72	7.11	7.74	8.59	9.54	9.46	8.72	7.73
1.86	6.96	8.60	9.84	10.40	9.75	8.82	8.30	8.72	9.35	9.63	9.23
1.91	9.62	8.40	7.49	7.38	8.36	9.41	9.45	8.67	7.53	6.97	7.08
1.96	7.61	8.86	9.85	9.23	8.28	7.65	7.88	8.77	9.38	9.30	8.64
2.01	7.61	6.65	7.22	8.72	9.96	10.01	9.08	7.98	7.64	8.24	9.29
2.06	9.14	9.51	8.75	8.03	8.02	8.71	9.43	9.62	9.09	8.16	7.37
2.11	7.43	8.92	9.64	9.46	8.46	7.89	8.01	8.62	9.08	8.83	8.19
2.16	8.73	7.59	7.49	8.07	8.82	9.28	8.84	8.16	7.49	7.56	8.03
2.21	8.34	9.40	9.03	8.30	7.94	8.24	8.89	9.53	9.41	8.59	7.77
2.26	7.40	7.46	8.70	9.80	9.82	9.37	8.45	8.16	8.28	8.78	9.14
2.31	8.83	8.32	7.63	7.45	7.96	8.47	8.65	8.45	7.77	7.44	7.54
2.36	7.81	7.83	7.80	7.40	7.42	7.57	7.98	8.30	7.87	7.23	7.01
2.41	6.71	6.85	7.63	8.56	8.96	8.50	7.70	7.41	7.69	8.10	8.36
2.46	8.57	8.56	8.09	7.47	7.49	8.18	8.56	8.58	7.99	7.42	7.01
2.51	7.00	7.22	8.67	9.28	9.00	8.42	7.68	7.93	8.35	8.71	8.79
2.56	8.21	7.66	7.42	8.22	8.56	8.83	8.32	8.14	7.65	7.63	7.93
2.61	8.27	8.03	7.65	7.26	7.68	8.15	8.37	8.13	7.70	7.13	7.00
2.66	7.04	7.74	8.54	8.77	8.35	7.85	7.48	7.81	8.11	8.32	8.01
2.71	8.55	8.05	7.85	8.04	8.56	9.07	8.86	8.55	7.84	7.64	7.84
2.76	7.37	8.22	8.28	7.96	7.70	7.56	7.87	8.06	8.18	7.92	7.38
2.81	6.67	7.04	7.54	8.02	8.13	7.80	7.45	7.18	7.29	7.37	7.54
2.86	7.34	7.35	6.87	7.07	7.52	7.77	7.85	7.61	7.11	6.89	6.98
2.91	7.16	7.76	8.25	7.95	7.71	7.53	7.35	7.76	7.82	7.77	7.40
2.96	7.56	7.34	7.66	8.15	8.50	8.63	8.19	7.84	7.80	7.66	7.97
3.01	7.53	7.93	8.02	7.67	7.46	7.50	7.70	8.09	7.98	7.53	7.24
3.06	6.99	7.32	7.71	8.18	8.00	7.78	7.46	7.48	7.78	7.77	7.63
3.11	7.23	7.46	7.39	7.61	8.05	8.29	8.03	7.61	7.54	7.47	7.48
3.16	7.27	7.79	7.91	7.42	7.50	7.58	7.72	8.01	7.77	7.55	7.18
3.21	7.35	7.64	8.06	8.13	8.20	7.89	7.70	7.64	7.66	7.63	7.57
3.26	7.55	7.43	7.25	7.11	7.33	7.73	7.73	7.68	7.37	7.01	6.83
3.31	7.34	7.97	8.14	8.13	7.65	7.80	7.76	7.86	7.82	7.60	7.36
3.36	7.21	7.04	7.43	7.90	8.03	8.03	7.75	7.61	7.58	7.30	7.43
3.41	7.89	7.94	7.70	7.50	7.66	7.85	8.22	8.12	7.83	7.39	7.22
3.46	7.39	7.98	8.35	8.31	8.20	7.85	7.83	7.96	8.20	8.01	7.91
3.51	7.45	6.90	7.08	7.30	7.66	7.80	7.70	7.54	7.42	7.15	7.32
3.56	7.43	7.66	7.70	7.36	7.35	7.60	7.68	7.73	7.55	7.18	6.82
3.61	6.59	6.75	7.10	7.16	7.07	7.03	6.95	6.85	7.03	6.85	6.85
3.66	7.62	7.46	7.59	7.47	7.95	8.08	8.10	7.86	7.55	7.36	7.20
3.71	7.31	7.64	7.85	7.91	7.75	7.73	7.69	7.68	7.78	7.64	7.31
3.76	7.68	7.94	8.11	8.23	8.42	8.28	8.09	7.96	8.00	7.87	7.77
3.81	7.24	7.64	7.15	7.30	7.44	7.65	7.63	7.51	7.35	7.10	6.98

3.86	6.54	6.80	7.07	7.18	7.28	7.18	6.98	7.09	7.18	7.01	6.75
3.91	7.55	7.68	7.44	7.90	8.14	8.31	8.14	8.03	7.51	7.56	7.46
3.96	7.16	7.72	7.41	7.48	7.62	7.65	7.65	7.76	7.62	7.30	7.13
4.01	7.19	7.38	7.61	7.92	7.91	7.72	7.41	7.51	7.56	7.55	7.25
4.06	7.41	7.39	7.57	7.76	7.94	7.93	7.82	7.87	7.55	7.35	7.37
4.11	7.45	7.62	7.87	7.93	7.86	7.95	7.94	8.26	8.05	7.74	7.57
4.16	7.05	7.17	7.39	7.69	7.94	7.82	7.65	7.50	7.48	7.53	7.34
4.21	7.79	8.03	8.04	8.10	8.30	8.33	8.38	8.49	8.25	8.11	7.90
4.26	7.20	7.75	7.96	8.09	8.10	8.07	7.99	8.09	7.81	7.85	7.63
4.31	6.62	6.64	6.83	7.10	7.21	7.29	7.41	7.31	6.93	6.99	6.91
4.36	7.02	7.00	7.17	7.22	7.44	7.55	7.56	7.56	7.14	7.06	6.97
4.41	6.82	7.37	7.57	7.63	7.83	7.68	7.57	7.57	7.54	7.44	7.27
4.46	7.52	7.54	7.50	7.62	7.89	7.86	7.75	7.61	7.49	7.13	7.14
4.51	7.13	7.39	7.23	7.39	7.32	7.49	7.29	7.37	7.41	7.29	7.21
4.56	6.99	7.34	7.56	7.66	7.80	7.72	7.60	7.57	7.49	7.25	7.30
4.61	7.18	7.35	7.38	7.36	7.47	7.60	7.65	7.52	7.41	7.27	7.16
4.66	6.89	7.29	7.55	7.56	7.69	7.67	7.56	7.70	7.53	7.23	7.11
4.71	7.49	7.81	7.86	7.99	8.13	8.12	7.84	7.68	7.50	7.45	7.49
4.76	7.24	7.53	7.31	7.55	7.48	7.61	7.56	7.69	7.47	7.40	7.36
4.81	6.88	7.34	7.63	7.58	7.52	7.73	7.46	7.55	7.37	7.29	7.13
4.86	7.40	7.55	7.48	7.63	7.86	7.74	7.70	7.63	7.43	7.35	7.22
4.91	7.17	7.25	7.37	7.31	7.38	7.58	7.57	7.52	7.33	7.23	7.14
4.96	7.23	7.41	7.42	7.69	7.67	7.63	7.66	7.53	7.31	7.23	7.15
5.01	7.22	7.24	7.50	7.47	7.56	7.64	7.50	7.57	7.39	7.07	7.05
5.06	6.79	6.96	7.08	7.17	7.22	7.15	7.14	7.19	7.00	7.01	6.77
5.11	6.81	6.81	6.85	7.01	6.97	6.97	6.84	6.67	6.64	6.63	6.52
5.16	6.85	6.98	7.06	7.18	7.37	7.22	7.10	7.19	6.86	6.78	6.66
5.21	6.47	6.43	6.79	6.98	7.19	7.11	7.02	7.10	6.94	6.77	6.74
5.26	7.37	7.81	7.74	7.94	7.92	7.83	7.72	7.59	7.49	7.37	7.29
5.31	7.11	7.13	7.38	7.48	7.59	7.46	7.52	7.40	7.27	7.04	6.91
5.38	6.91	6.79	7.14	6.94	7.25	7.20	7.29	7.16	6.96	6.79	6.73
5.45	7.37	7.31	7.24	7.56	7.52	7.46	7.58	7.56	7.35	7.18	7.22
5.53	7.20	7.17	7.31	7.46	7.40	7.47	7.38	7.41	7.37	7.02	6.75
5.61	7.09	7.35	7.44	7.65	7.73	7.57	7.52	7.51	7.48	7.30	7.09
5.70	7.32	7.20	7.26	7.38	7.55	7.54	7.42	7.42	7.30	6.98	6.94
5.80	7.01	7.05	7.54	7.56	7.68	7.59	7.59	7.37	7.30	7.29	7.08
5.90	6.82	6.72	6.95	6.92	7.14	6.98	6.83	6.82	6.72	6.62	6.49
6.01	7.37	7.44	7.66	7.76	7.87	7.88	7.74	7.65	7.54	7.34	7.18
6.13	7.32	7.24	7.31	7.55	7.64	7.56	7.39	7.40	7.28	7.17	7.09
6.25	6.78	7.04	7.09	7.15	7.25	7.32	7.20	7.10	7.09	6.86	6.64
6.39	6.68	6.90	6.88	6.95	7.09	7.15	6.94	7.03	6.76	6.84	6.63
6.53	6.64	6.51	6.92	6.81	7.06	7.09	6.99	7.03	6.81	6.80	6.61
6.69	7.19	7.30	7.42	7.42	7.62	7.56	7.47	7.45	7.20	7.16	7.05
6.85	6.64	6.91	7.06	7.32	7.36	7.45	7.18	7.16	7.06	6.87	6.74
7.03	6.67	6.76	6.81	6.92	7.05	7.10	7.07	7.03	6.78	6.78	6.77
7.21	6.17	6.56	6.66	6.63	6.80	6.87	6.82	6.71	6.75	6.55	6.33
7.41	7.00	7.01	7.33	7.29	7.41	7.31	7.26	7.23	7.04	6.88	6.70
7.63	7.00	7.11	7.16	7.28	7.53	7.39	7.43	7.31	7.16	7.04	6.98
7.86	7.06	7.01	7.16	7.13	7.28	7.26	7.12	7.13	7.07	6.80	6.69
8.10	6.46	6.81	6.88	7.00	7.06	6.98	6.90	6.91	6.71	6.58	6.41

8.36	5.97	6.45	6.39	6.56	6.56	6.60	6.57	6.49	6.39	6.32	6.21
8.64	6.74	7.15	7.09	7.13	7.28	7.23	7.15	7.22	7.07	6.87	6.69
8.94	7.23	7.38	7.68	7.65	7.68	7.73	7.60	7.56	7.37	7.26	7.04
9.26	7.28	7.40	7.38	7.51	7.77	7.74	7.51	7.62	7.30	7.42	7.17
9.60	6.75	6.77	7.12	7.08	7.18	7.22	6.91	7.05	6.77	6.71	6.51
9.97	6.17	6.29	6.29	6.44	6.39	6.39	6.40	6.35	6.23	6.05	5.90
10.36	7.26	7.26	7.26	7.27	7.21	7.33	7.28	7.16	6.97	6.77	6.65
10.77	6.59	6.79	6.92	6.86	7.04	7.14	7.15	7.05	6.78	6.84	6.57
11.22	6.29	6.47	6.50	6.70	6.67	6.80	6.77	6.82	6.70	6.54	6.50
11.70	6.83	7.08	7.10	7.40	7.23	7.18	7.19	7.11	6.83	6.87	6.59
12.21	6.17	6.74	6.66	6.75	6.79	6.77	6.59	6.59	6.47	6.29	6.02
12.75	6.08	6.13	6.44	6.55	6.62	6.69	6.66	6.47	6.39	6.26	6.15
13.33	6.43	6.89	6.75	6.70	7.07	7.14	6.97	6.79	6.82	6.38	6.38
13.95	6.73	6.81	6.79	6.79	6.88	6.96	6.95	6.81	6.69	6.53	6.52
14.62	6.26	6.27	6.15	6.29	6.60	6.53	6.49	6.40	6.39	6.18	6.02
15.33	6.21	6.29	6.23	6.55	6.53	6.62	6.53	6.43	6.40	6.15	6.01
16.09	6.18	6.33	6.37	6.36	6.43	6.53	6.51	6.33	6.20	6.05	5.96
16.90	6.16	6.43	6.55	6.70	6.71	6.88	6.62	6.62	6.58	6.38	6.32
17.77	6.32	6.44	6.51	6.55	6.62	6.70	6.63	6.63	6.38	6.36	6.10
18.70	6.23	6.22	6.21	6.48	6.61	6.71	6.66	6.62	6.38	6.47	6.30
19.69	5.55	6.05	6.10	6.06	6.07	6.32	6.27	6.14	6.16	5.89	5.74
20.75	5.98	6.10	5.91	6.01	6.21	6.27	6.18	6.21	5.96	5.86	5.72
21.89	6.14	6.41	6.65	6.51	6.90	6.71	6.71	6.68	6.20	6.15	5.88
23.10	6.77	6.93	6.98	7.07	7.10	7.09	7.04	7.05	6.91	6.80	6.55
24.39	5.97	6.33	6.46	6.41	6.57	6.54	6.54	6.48	6.25	6.01	5.84
25.78	5.60	5.76	5.90	6.01	6.09	6.18	6.10	6.16	5.95	5.79	5.69
27.26	6.33	6.13	6.25	6.38	6.51	6.57	6.40	6.46	6.18	6.14	5.88
28.84	5.81	5.94	6.04	5.78	6.03	6.14	6.08	5.99	5.79	5.81	5.63
30.53	6.03	6.38	6.43	6.42	6.44	6.53	6.64	6.61	6.18	6.12	6.11
32.34	5.90	5.81	5.93	5.97	6.23	6.26	6.00	6.05	5.79	5.64	5.66
34.27	6.22	6.11	6.16	6.10	6.31	6.35	6.03	6.23	5.90	5.73	5.57
36.34	5.85	6.24	6.20	6.40	6.36	6.40	6.28	6.35	6.23	5.88	5.78
38.55	5.77	5.91	5.98	6.20	6.12	6.09	6.11	6.01	5.88	5.81	5.50
40.91	6.05	6.37	6.41	6.47	6.43	6.50	6.39	6.21	6.07	5.92	5.78
43.43	5.51	5.45	5.54	5.62	5.59	5.59	5.53	5.45	5.43	5.14	5.01
46.13	6.10	6.09	6.13	6.37	6.58	6.47	6.65	6.50	6.35	6.11	5.91
49.01	5.79	6.04	6.06	6.14	6.37	6.38	6.23	6.00	6.01	5.80	5.72
52.09	5.68	5.83	5.60	5.65	5.91	5.91	5.91	5.87	5.74	5.56	5.47
55.39	5.51	5.69	5.72	5.73	5.91	6.06	5.72	5.59	5.61	5.29	5.20
58.91	5.38	5.57	5.53	5.71	5.66	5.82	5.60	5.55	5.43	5.28	5.05
62.68	5.25	5.46	5.62	5.79	5.69	5.77	5.54	5.62	5.43	5.18	5.10
66.70	5.90	6.01	6.02	6.20	6.09	6.21	6.00	5.91	5.72	5.66	5.44
71.00	5.71	5.72	6.04	6.07	6.14	6.23	6.05	5.93	5.81	5.71	5.57
75.60	5.47	5.57	5.82	5.86	6.00	6.00	5.76	5.88	5.68	5.65	5.46
80.51	5.76	5.71	5.95	5.93	5.86	5.90	5.70	5.72	5.49	5.41	5.21
85.77	5.06	5.19	5.33	5.56	5.60	5.60	5.60	5.43	5.32	5.23	5.02
91.38	5.52	5.73	5.81	5.77	5.77	5.78	5.68	5.70	5.47	5.28	5.07
97.39	5.29	5.30	5.36	5.56	5.77	5.63	5.61	5.61	5.42	5.28	5.21
103.80	6.01	6.22	6.07	6.54	6.35	6.31	6.31	6.21	5.92	5.77	5.59
110.66	5.61	5.87	5.91	5.98	5.96	6.01	5.89	5.85	5.66	5.47	5.22

117.99	5.15	5.50	5.40	5.51	5.74	5.79	5.70	5.66	5.69	5.46	5.39
125.83	5.58	5.49	5.69	5.75	5.84	5.73	5.70	5.79	5.39	5.28	5.13
134.21	5.36	5.69	5.47	5.58	5.80	5.86	5.71	5.54	5.40	5.40	5.15
143.17	5.48	5.50	5.66	5.93	5.86	5.77	5.77	5.64	5.57	5.45	5.21
152.74	5.62	5.60	5.52	5.66	5.68	5.68	5.65	5.50	5.28	5.00	4.99
162.97	5.40	5.45	5.42	5.39	5.67	5.75	5.35	5.40	5.20	5.05	4.85
173.91	5.21	5.36	5.52	5.41	5.55	5.62	5.25	5.35	5.24	4.86	4.86
185.60	5.26	5.40	5.24	5.44	5.43	5.38	5.38	5.35	5.05	4.91	4.71
198.10	5.43	5.34	5.39	5.44	5.54	5.45	5.60	5.38	5.30	5.07	4.93
211.46	5.95	5.90	6.18	6.17	6.19	6.18	6.05	5.75	5.70	5.46	5.24
225.75	4.77	5.08	4.98	5.13	5.26	5.26	5.21	5.14	5.05	4.91	4.73
241.01	5.14	5.30	5.37	5.41	5.57	5.43	5.28	5.23	5.07	4.84	4.62
257.33	4.91	5.33	5.49	5.43	5.34	5.42	5.39	5.33	5.08	4.92	4.71
274.77	4.87	4.96	5.19	5.19	5.36	5.34	5.17	5.08	4.89	4.77	4.52
293.42	5.53	5.61	5.59	5.74	5.55	5.58	5.30	5.35	5.27	4.87	4.91
313.35	5.50	5.66	5.86	5.72	5.80	5.86	5.75	5.65	5.31	5.09	4.98
334.66	5.18	5.00	5.22	5.02	5.26	5.32	5.23	5.15	4.94	4.76	4.64
357.43	4.85	4.73	4.74	4.85	4.88	4.99	4.86	4.79	4.62	4.36	4.26
381.78	4.95	5.00	4.98	5.08	5.12	5.16	5.03	4.99	4.71	4.45	4.44
407.80	4.80	4.83	4.87	4.81	4.79	4.69	4.80	4.67	4.46	4.27	4.03
435.62	4.72	5.14	5.00	5.13	5.04	5.11	5.18	5.13	4.99	4.80	4.59
465.35	4.96	5.26	5.20	5.31	5.21	5.13	4.92	4.99	4.76	4.43	4.37
497.14	5.13	5.14	4.99	5.29	5.15	5.03	5.06	4.91	4.68	4.55	4.15
531.11	4.27	4.48	4.73	4.50	4.60	4.56	4.51	4.55	4.36	4.16	4.04
567.43	5.04	4.87	4.84	5.07	5.00	5.15	4.89	4.69	4.46	4.35	4.24
606.26	4.63	4.74	4.83	4.85	4.85	4.94	4.66	4.70	4.37	4.21	4.04
647.76	5.24	5.37	5.46	5.75	5.48	5.50	5.38	5.22	4.91	4.62	4.45
692.12	4.95	4.86	5.19	5.29	5.25	5.26	5.03	5.06	4.89	4.66	4.43
739.53	5.13	5.09	5.30	5.50	5.34	5.26	5.18	5.09	4.84	4.61	4.40
790.22	4.29	4.36	4.42	4.39	4.61	4.47	4.28	4.27	4.24	3.96	3.78
844.40	4.49	4.82	4.82	4.85	4.86	4.74	4.64	4.49	4.40	4.06	3.96
902.32	4.89	5.28	5.36	5.26	5.47	5.26	5.02	5.05	4.85	4.63	4.39
964.23	4.32	4.36	4.39	4.55	4.50	4.57	4.24	4.23	4.05	3.88	3.78
1030.41	5.00	5.16	5.28	5.20	5.18	4.94	4.80	4.73	4.46	4.23	4.05
1101.15	4.93	4.91	5.12	5.18	5.22	5.13	4.95	4.91	4.66	4.66	4.29
1176.77	5.05	5.04	5.08	5.07	4.85	4.96	4.65	4.72	4.41	4.35	4.11
1257.60	5.02	5.03	4.90	5.00	5.04	4.90	4.91	4.71	4.39	4.20	4.06
1344.00	5.23	5.34	5.05	5.33	5.30	5.30	5.16	4.96	4.62	4.59	4.27
1436.36	5.09	5.28	5.27	5.17	5.24	5.09	5.12	4.95	4.70	4.51	4.33
1535.09	4.64	4.76	4.83	4.92	4.98	4.90	4.74	4.62	4.46	4.30	4.02
1640.63	4.45	4.62	4.55	4.66	4.55	4.65	4.48	4.50	4.33	4.08	3.86
1753.44	4.95	4.95	5.19	5.03	4.96	4.98	4.94	4.77	4.57	4.29	4.08
1874.02	3.79	3.95	4.02	4.12	4.05	4.06	3.84	3.73	3.74	3.42	3.31
2002.92	5.16	5.37	5.18	5.17	5.17	5.21	4.85	4.86	4.61	4.24	4.16
2140.71	5.00	4.99	5.07	5.02	5.14	4.99	4.81	4.69	4.50	4.22	4.02
2288.00	4.56	4.52	4.57	4.79	4.70	4.66	4.47	4.42	4.22	3.85	3.67
2445.44	4.45	4.41	4.49	4.39	4.56	4.39	4.17	4.09	4.02	3.75	3.61
2613.73	4.87	4.81	4.78	5.07	4.88	4.80	4.68	4.53	4.58	4.24	4.13
2793.63	4.63	4.65	4.64	4.83	4.64	4.58	4.44	4.34	4.13	3.76	3.69
2985.93	4.57	4.76	4.63	4.67	4.61	4.55	4.45	4.27	4.16	3.81	3.61

3191.49	4.46	4.32	4.45	4.63	4.55	4.52	4.48	4.35	4.15	3.85	3.65
3411.21	4.84	5.16	4.92	5.10	5.15	4.96	4.87	4.73	4.51	4.27	4.09
3646.09	4.89	5.02	4.84	4.97	4.97	4.80	4.71	4.63	4.50	4.25	3.92
3897.16	5.45	5.51	5.49	5.65	5.70	5.44	5.55	5.28	5.17	4.96	4.63

Wavelength (nm) / Time (ps)	428.08	429.50	430.92	432.35	433.77	435.20	436.62	438.05	439.47	440.89	442.32
-2.74	0.22	0.23	0.22	0.23	0.27	0.38	0.26	0.29	0.27	0.32	0.23
-2.24	-0.14	-0.06	0.01	-0.09	-0.05	-0.22	-0.11	-0.14	-0.22	-0.20	-0.14
-1.74	-0.39	-0.45	-0.41	-0.37	-0.40	-0.45	-0.39	-0.33	-0.30	-0.21	-0.18
-1.24	-0.07	0.04	0.08	0.07	0.07	0.11	0.14	-0.05	0.11	0.04	0.03
-0.74	0.22	0.15	0.20	0.17	0.17	0.26	0.22	0.20	0.17	0.13	0.14
-0.24	0.09	0.10	0.09	0.16	0.09	-0.07	0.03	0.03	-0.02	0.02	0.01
-0.14	0.20	0.25	0.02	0.13	0.06	0.17	0.04	0.09	0.04	-0.03	0.05
-0.04	-0.15	-0.25	-0.20	-0.30	-0.22	-0.19	-0.19	-0.07	-0.05	-0.06	-0.14
0.06	0.28	0.09	0.27	0.17	0.19	0.13	0.10	0.12	0.04	-0.06	-0.03
0.16	0.16	0.18	0.22	0.01	0.06	-0.01	-0.07	-0.14	-0.19	-0.18	-0.18
0.26	0.13	0.03	0.06	-0.01	0.01	0.00	0.07	-0.08	-0.01	-0.09	0.02
0.31	-0.23	-0.25	-0.15	-0.27	-0.14	-0.14	-0.13	-0.17	-0.22	-0.14	-0.16
0.36	-0.11	-0.07	-0.08	-0.10	-0.07	-0.05	-0.05	-0.05	-0.04	-0.11	-0.11
0.41	0.15	0.16	0.25	0.24	0.14	0.16	0.12	0.06	0.15	0.06	0.16
0.46	-0.14	-0.06	-0.13	-0.16	-0.08	-0.12	-0.12	-0.08	-0.08	-0.13	-0.05
0.51	-0.01	-0.02	0.03	0.11	-0.03	-0.07	-0.02	0.09	-0.05	-0.02	0.02
0.56	-0.01	0.06	0.19	0.03	-0.03	0.02	-0.17	-0.16	-0.17	-0.13	-0.11
0.61	0.23	0.15	0.18	0.12	0.13	0.04	-0.01	0.01	0.08	0.07	0.03
0.66	0.17	0.19	0.12	0.11	0.15	0.01	-0.06	0.02	-0.06	-0.02	-0.08
0.71	0.20	0.28	0.12	0.14	0.17	0.13	0.02	0.07	0.01	0.08	-0.02
0.76	0.20	0.16	0.36	0.27	0.16	0.10	-0.05	-0.15	-0.14	-0.27	-0.20
0.81	0.09	0.00	0.07	0.03	0.22	0.10	0.09	-0.01	0.18	0.09	0.16
0.86	0.27	0.25	0.30	0.22	0.18	0.26	0.34	0.30	0.23	0.10	0.24
0.91	0.01	0.36	0.09	-0.08	0.11	-0.02	-0.11	-0.02	-0.16	-0.05	-0.14
0.96	-3.98	0.62	-0.10	-1.14	-0.97	-0.26	0.19	0.06	-0.35	-0.17	0.04
1.01	40.68	-20.15	-66.67	-96.90	-108.06	-100.91	-78.85	-49.21	-21.52	-3.17	4.07
1.06	482.51	498.06	482.99	439.73	379.11	308.98	235.96	166.11	102.27	46.10	-0.94
1.11	-79.99	-16.37	67.02	163.26	264.19	358.82	440.69	504.64	548.86	572.27	576.22
1.16	14.28	22.04	27.64	17.15	-15.86	-64.64	-115.25	-152.33	-166.98	-155.46	-119.25
1.21	13.07	10.85	8.63	5.56	3.01	5.30	14.02	22.75	23.83	16.60	9.90
1.26	7.99	8.24	9.91	12.27	13.90	14.08	12.60	10.96	9.91	10.19	10.98
1.31	11.82	11.01	10.30	10.37	11.20	12.53	13.15	12.31	9.96	7.05	5.33
1.36	12.17	12.82	12.40	10.29	8.17	6.79	7.12	8.89	10.64	11.34	10.44
1.41	8.63	8.44	9.20	10.32	10.97	10.27	8.56	6.92	6.48	7.67	10.03
1.46	11.30	9.83	8.06	6.62	6.88	8.59	10.72	11.84	11.20	8.73	6.00
1.51	8.23	9.55	10.60	11.16	10.38	8.94	7.41	7.10	7.82	9.07	10.02
1.56	8.44	7.91	8.38	9.64	10.64	10.60	9.02	6.74	4.89	4.84	6.55
1.61	10.56	9.30	7.69	6.43	6.28	7.31	8.73	9.62	8.96	7.38	5.86
1.66	8.03	9.01	9.68	9.81	9.07	7.75	6.84	7.15	8.27	9.43	9.96
1.71	9.06	8.05	7.28	7.31	8.18	9.24	9.89	9.30	7.56	5.63	4.54
1.76	9.74	10.36	10.12	8.89	7.56	6.53	6.48	7.49	8.51	8.82	7.99

1.81	7.18	7.46	8.07	8.53	8.46	7.77	6.57	5.65	5.76	6.54	7.84
1.86	8.48	7.49	6.97	7.21	7.82	8.62	8.76	8.09	6.78	5.46	5.04
1.91	7.83	8.67	8.84	8.46	7.24	6.20	5.83	6.31	7.21	7.83	7.43
1.96	7.77	7.41	7.43	7.96	8.42	8.31	7.71	6.35	5.30	5.07	5.76
2.01	9.65	9.11	8.04	6.94	6.18	6.42	7.57	8.64	8.84	7.76	6.24
2.06	7.31	7.81	8.40	8.53	8.15	7.27	6.32	5.79	6.06	6.89	7.41
2.11	7.59	7.28	7.17	7.37	8.00	8.03	7.30	6.38	5.32	4.94	5.11
2.16	8.58	8.45	7.99	7.04	6.18	5.71	6.01	6.66	7.39	7.12	6.36
2.21	7.27	7.31	7.93	8.20	8.24	7.70	6.76	6.06	5.79	6.28	7.04
2.26	8.88	8.23	7.43	6.96	6.98	7.63	8.06	8.09	7.38	6.20	5.25
2.31	7.94	8.28	8.42	8.06	7.36	6.68	6.12	6.16	6.69	7.00	6.81
2.36	6.97	6.98	7.26	7.25	7.03	6.51	5.98	5.72	5.78	6.03	6.21
2.41	8.25	7.70	6.84	6.38	6.32	6.84	7.40	7.58	7.00	5.94	5.02
2.46	6.96	7.33	7.71	7.66	7.30	6.61	5.90	5.49	5.73	6.20	6.50
2.51	8.22	7.59	7.01	6.79	7.07	7.54	7.96	7.60	6.64	5.56	5.10
2.56	8.05	8.06	7.70	7.04	6.64	6.23	6.57	6.88	6.98	6.82	6.09
2.61	7.13	7.31	7.66	7.33	6.73	6.15	5.71	5.50	5.87	6.09	6.14
2.66	7.54	6.99	6.72	6.78	6.95	7.08	6.97	6.46	5.65	5.07	4.82
2.71	7.83	8.04	7.65	7.23	6.49	6.16	6.03	6.28	6.63	6.59	6.17
2.76	6.90	6.86	6.98	7.07	7.24	7.16	6.48	5.85	5.44	5.44	5.77
2.81	7.50	7.15	6.90	6.51	6.37	6.63	6.91	6.83	6.58	5.79	5.29
2.86	7.25	7.53	7.28	6.97	6.60	6.21	6.06	6.27	6.32	6.33	5.97
2.91	6.92	6.52	6.55	6.60	6.60	6.61	6.36	5.73	5.22	4.93	5.00
2.96	8.01	7.83	7.43	6.94	6.64	6.48	6.63	6.71	6.70	6.22	5.66
3.01	6.94	7.00	7.23	7.20	7.22	6.82	6.28	5.91	5.63	5.74	5.85
3.06	7.40	7.00	6.56	6.43	6.46	6.64	6.65	6.35	5.90	5.09	4.95
3.11	7.52	7.36	6.98	6.58	6.33	6.11	5.95	6.17	6.03	5.86	5.43
3.16	6.94	6.85	6.97	6.99	6.96	6.56	6.07	5.48	5.28	5.24	5.51
3.21	7.26	7.09	6.76	6.42	6.45	6.38	6.24	6.05	5.73	5.23	4.94
3.26	6.95	7.07	6.93	6.81	6.44	6.04	5.65	5.71	5.83	5.85	5.68
3.31	7.01	6.76	6.64	6.61	6.62	6.52	6.19	5.78	5.41	5.29	5.32
3.36	7.26	7.04	6.79	6.40	6.24	6.09	6.11	6.11	6.02	5.64	5.24
3.41	7.20	7.35	7.35	7.16	6.83	6.37	5.96	5.80	5.96	5.95	6.05
3.46	7.36	7.24	6.98	6.85	6.88	6.76	6.48	6.05	5.55	5.17	5.04
3.51	7.26	7.44	7.12	6.83	6.50	6.31	6.31	6.17	6.15	6.08	5.73
3.56	6.71	6.65	6.67	6.62	6.49	6.09	5.75	5.39	5.19	5.18	5.36
3.61	6.60	6.36	6.31	6.22	5.97	5.95	5.82	5.57	5.35	5.16	4.83
3.66	7.24	7.25	7.22	6.86	6.35	6.00	5.71	5.79	5.87	5.75	5.59
3.71	7.12	6.82	6.66	6.65	6.72	6.61	6.44	5.95	5.55	5.13	5.20
3.76	7.68	7.43	7.30	7.00	6.80	6.61	6.49	6.35	6.07	5.78	5.46
3.81	6.90	6.89	6.89	6.55	6.24	5.93	5.67	5.56	5.45	5.43	5.38
3.86	6.70	6.46	6.47	6.41	6.39	6.15	5.92	5.65	5.27	5.06	5.11
3.91	7.49	7.31	7.21	6.93	6.48	6.32	6.23	6.17	6.08	5.91	5.49
3.96	7.02	6.95	7.00	6.92	6.66	6.38	6.08	5.96	5.57	5.45	5.48
4.01	7.19	7.03	6.77	6.45	6.39	6.35	6.12	6.07	5.77	5.34	5.13
4.06	7.23	7.21	7.04	6.74	6.58	6.25	6.16	5.93	5.87	5.66	5.44
4.11	7.38	7.21	7.09	7.04	6.83	6.55	6.25	5.79	5.60	5.44	5.43
4.16	7.31	7.00	6.72	6.47	6.27	6.22	6.12	6.01	5.76	5.33	5.09
4.21	8.04	8.01	7.91	7.73	7.34	7.13	6.86	6.63	6.41	6.29	6.15
4.26	7.47	7.14	6.90	6.82	6.75	6.61	6.35	6.07	5.77	5.49	5.32

4.31	6.98	6.84	6.67	6.25	6.20	6.04	5.89	5.82	5.77	5.49	5.03
4.36	6.92	6.79	6.71	6.49	6.31	6.03	5.67	5.56	5.62	5.38	5.18
4.41	7.10	6.98	6.80	6.53	6.49	6.28	6.10	5.98	5.58	5.25	5.09
4.46	7.02	6.96	6.89	6.54	6.38	6.22	6.36	6.30	5.94	5.90	5.69
4.51	6.91	6.81	6.91	6.65	6.57	6.37	6.05	5.92	5.62	5.42	5.41
4.56	7.07	6.86	6.73	6.51	6.43	6.20	6.03	5.79	5.57	5.33	5.03
4.61	7.14	6.97	6.92	6.54	6.38	6.07	6.04	5.74	5.59	5.34	5.05
4.66	6.86	6.64	6.61	6.55	6.38	6.18	5.87	5.72	5.27	5.08	4.92
4.71	7.17	7.04	6.78	6.46	6.22	6.16	6.00	6.01	5.80	5.51	5.22
4.76	7.09	7.04	7.09	6.97	6.70	6.39	6.18	6.02	5.98	5.84	5.73
4.81	6.82	6.78	6.57	6.53	6.32	6.05	5.96	5.58	5.45	5.19	4.98
4.86	7.12	7.05	6.81	6.43	6.26	6.01	5.91	5.77	5.59	5.32	5.08
4.91	6.92	6.85	6.80	6.54	6.49	6.20	5.95	5.76	5.69	5.39	5.47
4.96	7.05	6.96	6.77	6.54	6.25	6.20	6.04	5.99	5.70	5.38	5.16
5.01	7.06	6.91	6.79	6.54	6.30	6.11	5.84	5.73	5.31	5.46	5.10
5.06	6.62	6.63	6.55	6.35	6.27	6.14	5.90	5.72	5.49	5.40	5.14
5.11	6.37	6.28	6.25	5.92	5.83	5.77	5.59	5.32	5.23	4.84	4.71
5.16	6.49	6.59	6.55	6.16	5.86	5.72	5.51	5.31	5.24	5.08	4.87
5.21	6.79	6.64	6.55	6.37	6.27	6.15	5.90	5.81	5.56	5.28	5.11
5.26	7.11	6.98	6.77	6.65	6.33	6.19	5.99	5.82	5.60	5.40	5.15
5.31	6.83	6.73	6.64	6.45	6.21	6.06	5.74	5.50	5.42	5.28	5.09
5.38	6.62	6.56	6.49	6.25	6.13	5.90	5.76	5.45	5.34	5.24	5.08
5.45	6.92	6.80	6.78	6.49	6.37	6.11	5.91	5.77	5.61	5.44	5.35
5.53	6.62	6.62	6.48	6.37	6.09	5.75	5.61	5.52	5.21	5.04	4.76
5.61	7.02	6.83	6.71	6.44	6.27	6.21	5.94	5.73	5.58	5.27	5.10
5.70	6.78	6.68	6.52	6.31	6.05	5.85	5.58	5.46	5.16	5.15	4.89
5.80	6.91	6.84	6.57	6.41	6.33	6.01	5.88	5.72	5.37	5.22	4.97
5.90	6.31	6.38	6.22	5.94	5.82	5.78	5.50	5.32	5.13	4.98	4.78
6.01	6.96	6.94	6.75	6.52	6.31	6.12	5.82	5.73	5.45	5.17	5.07
6.13	6.84	6.85	6.60	6.36	6.19	5.92	5.64	5.56	5.31	5.03	4.85
6.25	6.51	6.39	6.39	6.13	5.74	5.58	5.39	5.26	4.92	4.90	4.60
6.39	6.50	6.47	6.33	6.06	5.83	5.69	5.49	5.41	5.15	4.86	4.72
6.53	6.52	6.50	6.32	6.21	5.96	5.74	5.59	5.43	5.29	5.02	4.80
6.69	6.91	6.76	6.65	6.42	6.23	5.96	5.73	5.67	5.30	5.19	4.98
6.85	6.70	6.40	6.32	6.12	5.96	5.73	5.59	5.34	5.18	4.94	4.72
7.03	6.39	6.54	6.26	6.06	5.89	5.64	5.47	5.38	4.97	4.83	4.58
7.21	6.29	6.15	6.11	5.83	5.65	5.58	5.33	5.16	5.01	4.79	4.60
7.41	6.57	6.49	6.31	6.05	5.85	5.57	5.50	5.23	5.08	4.85	4.61
7.63	6.83	6.76	6.55	6.36	6.16	5.98	5.77	5.65	5.47	5.19	4.97
7.86	6.57	6.49	6.32	6.04	5.85	5.59	5.41	5.19	5.02	4.82	4.68
8.10	6.23	6.24	5.91	5.85	5.65	5.36	5.24	5.02	4.89	4.69	4.62
8.36	6.06	5.91	5.90	5.65	5.60	5.26	4.95	4.94	4.81	4.64	4.33
8.64	6.55	6.41	6.41	6.12	5.99	5.68	5.47	5.31	5.03	4.75	4.59
8.94	6.86	6.79	6.68	6.44	6.15	5.89	5.75	5.54	5.36	5.07	4.95
9.26	7.00	6.74	6.76	6.49	6.39	6.15	5.80	5.66	5.59	5.28	4.98
9.60	6.25	6.23	5.97	5.74	5.46	5.31	5.09	4.91	4.73	4.47	4.33
9.97	5.80	5.58	5.54	5.34	5.14	5.03	4.82	4.75	4.58	4.35	4.30
10.36	6.46	6.30	6.12	5.77	5.59	5.43	5.22	5.02	4.77	4.52	4.37
10.77	6.47	6.52	6.24	6.16	5.83	5.62	5.42	5.28	4.97	4.89	4.81
11.22	6.37	6.34	6.20	5.98	5.79	5.60	5.33	5.23	4.91	4.88	4.60

11.70	6.44	6.26	6.09	5.92	5.62	5.51	5.24	5.00	4.75	4.66	4.34
12.21	5.92	5.76	5.49	5.35	5.20	4.93	4.68	4.49	4.32	4.03	3.87
12.75	6.03	5.88	5.86	5.60	5.50	5.13	4.88	4.90	4.63	4.43	4.30
13.33	6.20	6.07	5.99	5.73	5.47	5.32	5.03	4.97	4.75	4.54	4.39
13.95	6.17	6.03	5.88	5.75	5.55	5.23	5.03	4.87	4.71	4.39	4.35
14.62	5.85	5.83	5.56	5.45	5.21	5.00	4.93	4.68	4.53	4.36	4.01
15.33	6.00	5.79	5.68	5.44	5.25	5.06	4.86	4.62	4.50	4.31	4.06
16.09	5.82	5.60	5.58	5.29	5.26	4.84	4.63	4.54	4.25	4.17	3.99
16.90	6.25	6.03	6.01	5.73	5.56	5.36	5.23	4.93	4.74	4.50	4.34
17.77	6.02	5.83	5.70	5.50	5.31	5.08	4.83	4.63	4.56	4.28	4.14
18.70	6.20	6.09	5.90	5.73	5.56	5.30	5.09	4.96	4.78	4.47	4.33
19.69	5.68	5.56	5.39	5.09	5.02	4.87	4.62	4.47	4.34	4.13	3.98
20.75	5.57	5.45	5.32	5.18	5.01	4.81	4.57	4.30	4.24	3.91	3.83
21.89	5.85	5.72	5.62	5.42	5.22	5.11	4.99	4.91	4.58	4.41	4.15
23.10	6.29	6.34	6.22	5.98	5.68	5.48	5.26	5.12	4.77	4.62	4.43
24.39	5.92	5.60	5.49	5.30	5.11	4.92	4.59	4.54	4.31	4.15	3.96
25.78	5.68	5.37	5.44	5.24	5.06	4.88	4.70	4.42	4.37	4.07	4.06
27.26	5.91	5.84	5.66	5.37	5.15	5.00	4.66	4.52	4.31	3.97	3.91
28.84	5.37	5.32	5.30	5.04	4.82	4.63	4.34	4.25	4.06	3.82	3.72
30.53	5.95	5.76	5.69	5.42	5.30	4.95	4.77	4.61	4.40	4.11	3.95
32.34	5.45	5.28	5.22	4.94	4.70	4.49	4.26	4.08	4.00	3.87	3.65
34.27	5.36	5.40	5.17	4.98	4.73	4.52	4.33	4.13	4.01	3.80	3.59
36.34	5.72	5.63	5.50	5.15	5.03	4.80	4.43	4.41	4.14	3.96	3.66
38.55	5.47	5.29	5.19	4.86	4.61	4.40	4.23	3.94	3.73	3.53	3.19
40.91	5.66	5.58	5.24	5.01	4.69	4.63	4.41	4.19	3.98	3.82	3.62
43.43	4.95	4.85	4.68	4.51	4.40	4.08	3.94	3.87	3.64	3.50	3.30
46.13	5.66	5.65	5.45	5.14	4.93	4.76	4.55	4.31	4.04	3.84	3.63
49.01	5.54	5.45	5.23	5.10	4.77	4.67	4.32	4.27	3.99	3.71	3.66
52.09	5.38	5.23	5.09	4.90	4.84	4.54	4.42	4.28	4.07	3.95	3.79
55.39	5.02	4.85	4.69	4.57	4.36	4.08	3.92	3.66	3.54	3.34	3.12
58.91	5.09	5.00	4.70	4.50	4.21	4.09	3.95	3.76	3.56	3.50	3.09
62.68	4.92	4.68	4.72	4.49	4.45	4.27	3.94	3.88	3.75	3.50	3.39
66.70	5.24	5.33	4.93	4.80	4.60	4.30	4.05	3.91	3.74	3.46	3.30
71.00	5.40	5.25	5.13	4.79	4.54	4.42	4.12	4.00	3.75	3.60	3.43
75.60	5.37	5.31	5.10	4.93	4.72	4.43	4.31	4.09	3.86	3.65	3.53
80.51	4.94	4.88	4.70	4.39	4.21	3.99	3.78	3.45	3.33	3.11	2.76
85.77	4.95	4.83	4.78	4.54	4.34	4.28	4.13	3.83	3.70	3.60	3.46
91.38	4.96	4.88	4.66	4.50	4.29	4.01	3.88	3.71	3.41	3.30	3.12
97.39	4.95	4.92	4.74	4.43	4.36	4.23	3.88	3.73	3.61	3.33	3.17
103.80	5.56	5.46	5.18	4.95	4.66	4.43	4.22	4.05	3.74	3.58	3.35
110.66	5.02	4.90	4.75	4.44	4.27	4.02	3.85	3.61	3.45	3.15	2.96
117.99	5.18	5.08	4.94	4.70	4.47	4.28	3.95	3.81	3.63	3.37	3.16
125.83	4.95	4.80	4.73	4.37	4.22	3.92	3.73	3.54	3.44	3.18	3.00
134.21	5.09	4.76	4.72	4.31	4.17	4.00	3.78	3.49	3.18	3.09	2.90
143.17	4.91	4.89	4.72	4.62	4.38	4.34	4.02	3.87	3.70	3.33	3.09
152.74	4.76	4.63	4.40	4.09	3.88	3.72	3.32	3.38	2.98	2.88	2.73
162.97	4.76	4.62	4.46	4.21	3.97	3.84	3.48	3.33	3.18	3.06	2.66
173.91	4.59	4.46	4.35	4.11	3.81	3.73	3.52	3.20	2.99	2.85	2.70
185.60	4.57	4.30	4.26	4.03	3.74	3.59	3.36	3.13	3.05	2.72	2.57
198.10	4.73	4.64	4.46	4.23	4.02	3.75	3.56	3.28	3.25	2.94	2.69

211.46	5.19	4.91	4.64	4.39	4.07	3.97	3.71	3.39	3.12	3.04	2.81
225.75	4.53	4.41	4.33	4.05	3.88	3.65	3.45	3.25	3.07	2.93	2.68
241.01	4.47	4.31	4.21	4.09	3.77	3.52	3.31	3.21	2.94	2.70	2.53
257.33	4.64	4.43	4.28	4.04	3.79	3.61	3.34	3.14	2.92	2.64	2.53
274.77	4.53	4.26	4.18	3.84	3.70	3.49	3.10	3.06	2.86	2.68	2.43
293.42	4.63	4.45	4.27	3.95	3.81	3.51	3.30	3.05	2.76	2.61	2.38
313.35	4.71	4.59	4.43	4.13	3.92	3.61	3.29	3.10	2.81	2.58	2.31
334.66	4.54	4.21	4.04	4.03	3.74	3.47	3.26	3.04	2.89	2.69	2.49
357.43	4.14	3.98	3.95	3.71	3.58	3.46	3.17	3.01	2.80	2.56	2.38
381.78	4.25	4.11	3.80	3.59	3.40	3.26	2.92	2.66	2.49	2.24	2.08
407.80	4.01	3.82	3.71	3.50	3.26	3.18	2.82	2.76	2.53	2.31	2.26
435.62	4.58	4.30	4.31	3.94	3.64	3.49	3.27	3.07	2.79	2.56	2.45
465.35	4.20	4.00	3.85	3.55	3.34	2.99	2.87	2.60	2.33	2.22	2.02
497.14	4.11	3.90	3.80	3.51	3.28	2.90	2.73	2.59	2.42	2.15	1.99
531.11	3.92	3.67	3.55	3.27	3.21	2.85	2.80	2.55	2.40	2.09	1.94
567.43	3.98	3.90	3.64	3.38	3.23	3.02	2.72	2.55	2.35	2.13	1.92
606.26	3.86	3.71	3.47	3.26	3.01	2.81	2.55	2.38	2.22	1.94	1.82
647.76	4.25	3.99	3.83	3.53	3.23	2.95	2.70	2.44	2.24	1.95	1.81
692.12	4.11	3.99	3.86	3.46	3.36	3.04	2.81	2.57	2.40	2.04	1.86
739.53	4.24	4.03	3.71	3.57	3.22	2.91	2.68	2.59	2.38	2.09	1.90
790.22	3.65	3.56	3.45	3.15	3.00	2.76	2.59	2.45	2.30	2.07	1.89
844.40	3.79	3.66	3.46	3.32	3.22	2.93	2.66	2.50	2.32	2.10	1.99
902.32	4.03	3.89	3.77	3.41	3.27	2.90	2.76	2.57	2.38	1.97	1.88
964.23	3.61	3.49	3.29	2.98	2.68	2.60	2.51	2.31	2.08	1.94	1.69
1030.41	3.82	3.55	3.30	3.12	2.81	2.59	2.41	2.05	1.89	1.67	1.57
1101.15	4.19	4.04	3.67	3.36	3.06	2.91	2.70	2.40	2.15	1.96	1.73
1176.77	3.89	3.78	3.59	3.49	3.16	2.89	2.53	2.36	2.08	1.84	1.64
1257.60	3.87	3.67	3.39	3.14	2.81	2.72	2.41	2.18	1.89	1.71	1.68
1344.00	4.05	3.92	3.67	3.37	3.16	2.96	2.65	2.47	2.23	1.83	1.62
1436.36	4.00	3.76	3.53	3.21	2.99	2.60	2.40	2.11	2.01	1.67	1.53
1535.09	3.66	3.67	3.35	3.16	2.90	2.53	2.31	2.09	1.82	1.62	1.40
1640.63	3.74	3.55	3.30	3.14	2.89	2.67	2.44	2.13	2.02	1.85	1.60
1753.44	3.88	3.61	3.39	3.10	2.87	2.54	2.33	2.08	1.64	1.60	1.42
1874.02	3.13	3.04	2.83	2.53	2.46	2.19	2.13	1.97	1.73	1.63	1.41
2002.92	3.80	3.61	3.30	2.99	2.78	2.49	2.19	2.06	1.69	1.54	1.26
2140.71	3.85	3.50	3.30	3.12	2.78	2.61	2.22	1.99	1.67	1.47	1.26
2288.00	3.44	3.31	3.02	2.72	2.43	2.20	2.01	1.78	1.55	1.40	1.12
2445.44	3.41	3.16	3.12	2.80	2.56	2.30	2.11	1.87	1.71	1.50	1.36
2613.73	3.91	3.67	3.37	3.22	2.94	2.61	2.19	2.17	1.82	1.47	1.46
2793.63	3.33	3.21	3.10	2.55	2.47	2.08	1.89	1.75	1.41	1.27	1.00
2985.93	3.40	3.23	2.99	2.66	2.52	2.22	1.97	1.73	1.35	1.07	0.97
3191.49	3.55	3.52	3.32	3.00	2.75	2.49	2.29	2.09	1.92	1.66	1.41
3411.21	3.76	3.59	3.46	3.12	2.97	2.66	2.34	2.03	1.89	1.56	1.38
3646.09	3.83	3.77	3.69	3.46	3.30	3.05	2.93	2.72	2.46	2.33	2.13
3897.16	4.32	4.18	4.03	3.69	3.39	3.11	2.88	2.67	2.33	1.99	1.82

Wavelength (nm) / Time (ps)	443.74	445.17	446.59	448.01	449.44	450.86	452.29	453.71	455.13	456.56	457.98
-2.74	0.24	0.25	0.29	0.21	0.17	0.03	0.13	0.10	0.08	-0.06	-0.05

-2.24	-0.20	-0.12	-0.18	-0.16	-0.05	-0.09	-0.07	-0.01	0.06	-0.02	-0.01
-1.74	-0.19	-0.20	-0.14	-0.13	-0.10	0.06	0.12	0.02	-0.01	0.10	0.11
-1.24	0.06	-0.01	0.04	-0.01	0.03	-0.02	-0.04	0.05	-0.04	0.06	-0.04
-0.74	0.16	0.06	0.12	0.11	0.06	0.04	-0.13	-0.01	-0.04	-0.12	-0.17
-0.24	0.03	-0.06	-0.07	0.01	-0.05	0.06	0.02	0.02	-0.01	-0.02	0.08
-0.14	-0.01	0.12	-0.05	0.00	-0.10	-0.02	-0.14	-0.22	-0.09	-0.07	-0.10
-0.04	-0.09	-0.04	-0.02	-0.02	0.04	-0.07	0.09	0.04	0.05	0.14	0.17
0.06	-0.06	-0.09	-0.08	-0.13	-0.09	-0.10	-0.06	-0.06	-0.17	-0.07	-0.15
0.16	-0.19	-0.20	-0.18	-0.21	-0.19	-0.17	-0.19	-0.14	-0.04	-0.13	-0.04
0.26	-0.13	-0.17	-0.14	-0.25	-0.22	-0.18	-0.14	-0.12	-0.02	-0.18	-0.16
0.31	-0.13	-0.08	-0.04	-0.05	0.04	0.08	-0.01	0.00	0.14	0.05	0.00
0.36	-0.17	-0.09	-0.17	-0.07	-0.06	-0.11	0.03	-0.06	-0.08	0.02	-0.02
0.41	0.08	0.03	0.05	-0.04	0.07	0.07	0.05	-0.04	-0.07	-0.10	0.04
0.46	-0.10	-0.18	-0.05	-0.08	-0.13	-0.16	-0.08	-0.07	-0.04	-0.15	-0.12
0.51	-0.07	-0.11	-0.07	-0.13	-0.08	-0.05	-0.08	0.13	0.08	-0.01	-0.03
0.56	-0.16	-0.12	-0.09	-0.18	-0.08	-0.19	-0.10	-0.12	0.04	-0.07	0.02
0.61	-0.02	-0.04	-0.03	-0.09	-0.07	-0.12	0.01	-0.09	-0.05	-0.08	-0.04
0.66	-0.11	-0.16	-0.07	-0.16	-0.12	-0.07	-0.12	-0.07	-0.13	-0.20	-0.16
0.71	0.02	-0.09	-0.04	-0.04	-0.07	-0.12	-0.05	-0.04	-0.01	-0.10	-0.03
0.76	-0.27	-0.34	-0.22	-0.25	-0.23	-0.19	-0.19	-0.19	-0.13	-0.11	-0.05
0.81	0.12	0.06	0.10	0.10	0.10	0.11	0.09	0.04	0.07	-0.01	0.07
0.86	0.19	0.19	0.15	0.25	0.15	0.18	0.12	0.04	0.08	0.09	0.03
0.91	-0.23	-0.21	-0.18	-0.04	-0.14	0.02	-0.14	-0.07	-0.10	-0.13	-0.16
0.96	-0.15	-0.23	-0.01	-0.10	-0.12	0.04	-0.13	-0.04	0.16	-0.11	-0.08
1.01	3.15	-0.38	-2.68	-2.44	-1.15	-0.40	-0.43	-0.58	-0.28	0.04	-0.09
1.06	-38.56	-65.81	-82.69	-89.32	-86.41	-75.47	-58.81	-39.75	-21.83	-7.98	0.01
1.11	562.51	534.34	492.12	438.91	377.49	314.76	253.04	196.26	144.35	97.92	56.33
1.16	-61.91	11.47	94.45	182.34	267.43	345.79	410.84	459.54	487.03	493.90	484.04
1.21	12.69	24.18	32.29	23.20	-8.99	-57.21	-107.21	-145.38	-163.05	-157.22	-129.13
1.26	10.80	9.17	6.69	3.85	2.03	2.53	6.08	12.86	19.82	23.79	21.89
1.31	5.60	7.53	10.39	12.59	13.08	12.03	10.14	8.72	8.04	8.58	9.82
1.36	8.58	6.95	6.67	7.85	9.98	11.58	11.41	9.52	6.28	3.15	1.46
1.41	11.75	12.08	10.35	7.35	4.68	3.26	3.85	5.96	8.47	9.88	9.66
1.46	4.56	4.96	7.00	9.14	9.92	9.01	6.52	3.92	2.58	3.27	5.60
1.51	9.39	7.71	5.58	4.13	4.21	5.94	8.16	9.86	10.04	8.57	6.18
1.56	8.78	10.42	10.40	8.53	5.83	3.97	3.64	4.91	6.90	8.75	9.01
1.61	4.93	5.49	7.17	8.60	9.08	7.98	5.69	3.50	2.18	2.39	4.03
1.66	9.08	7.25	5.12	3.86	4.04	5.24	7.15	8.44	8.41	7.02	5.19
1.71	4.77	6.06	7.48	8.17	7.72	6.02	4.49	3.34	3.74	4.96	6.59
1.76	6.29	4.97	4.64	5.45	7.07	8.43	8.70	7.86	5.82	3.73	2.33
1.81	8.50	8.21	6.93	5.19	3.91	3.63	4.33	5.35	6.32	6.35	5.41
1.86	5.33	6.20	6.84	6.88	5.94	4.46	3.22	2.72	3.37	4.60	5.96
1.91	6.22	4.70	3.65	3.67	4.78	6.10	7.11	7.19	6.11	4.27	2.69
1.96	6.72	7.62	7.58	6.58	5.11	3.82	3.43	3.97	5.08	5.97	6.15
2.01	4.89	4.59	5.15	6.38	7.36	7.32	6.16	4.27	2.59	1.77	2.36
2.06	7.10	6.20	4.89	3.84	3.33	3.96	4.88	5.81	6.18	5.71	4.59
2.11	5.77	6.27	6.43	5.89	4.88	3.87	3.33	3.54	4.23	4.94	5.29
2.16	5.05	4.15	4.25	4.81	5.81	6.42	6.10	5.23	3.86	2.62	1.99
2.21	7.53	7.23	6.26	4.80	3.83	3.63	4.03	4.99	5.92	5.91	5.23

2.26	4.73	5.19	5.96	6.55	6.48	5.93	4.55	3.51	3.06	3.33	4.20
2.31	6.23	5.34	4.44	4.15	4.49	5.10	5.58	5.67	5.09	4.02	2.87
2.36	6.28	5.92	5.20	4.58	4.04	4.01	4.23	4.51	4.61	4.38	3.79
2.41	4.55	4.64	5.39	6.04	6.09	5.43	4.39	3.26	2.63	2.81	3.41
2.46	6.32	5.63	4.74	3.90	3.72	3.98	4.58	5.16	5.22	4.66	3.66
2.51	5.11	5.82	6.67	6.84	6.45	5.17	3.87	3.08	3.12	3.71	4.52
2.56	5.32	4.78	4.55	4.83	5.27	5.51	5.25	4.51	3.60	2.95	2.72
2.61	5.58	5.06	4.26	3.71	3.91	4.18	4.63	4.80	4.58	3.93	3.10
2.66	4.98	5.36	5.69	5.56	4.99	4.23	3.38	3.21	3.26	3.75	4.16
2.71	5.30	4.52	4.15	4.29	4.69	5.04	5.09	4.72	3.93	3.04	2.42
2.76	6.08	6.11	5.72	5.07	4.30	3.81	3.67	4.09	4.52	4.83	4.53
2.81	4.94	4.95	5.37	5.47	5.38	4.94	4.20	3.65	3.16	3.01	3.31
2.86	5.49	4.87	4.58	4.40	4.55	4.82	4.90	4.65	4.13	3.33	2.75
2.91	5.30	5.50	5.43	5.02	4.35	3.69	3.35	3.37	3.63	4.05	4.20
2.96	5.02	4.77	4.78	5.25	5.12	5.22	4.79	4.01	3.31	3.07	2.93
3.01	5.97	5.77	5.21	4.53	4.15	3.96	3.98	4.19	4.46	4.37	3.98
3.06	4.79	4.90	5.33	5.21	4.97	4.34	3.68	3.28	3.21	3.41	3.74
3.11	4.99	4.60	4.22	4.40	4.60	4.52	4.32	3.90	3.33	2.93	2.64
3.16	5.50	5.31	4.89	4.31	3.79	3.55	3.50	3.74	3.98	3.95	3.56
3.21	4.76	4.82	5.03	4.89	4.62	4.24	3.79	3.42	3.47	3.28	3.37
3.26	5.44	4.81	4.43	4.26	4.33	4.42	4.57	4.54	4.16	3.66	3.18
3.31	5.31	5.42	5.16	4.85	4.32	3.95	3.89	3.73	3.90	4.00	3.91
3.36	5.01	4.81	4.88	4.95	4.92	4.68	4.17	3.80	3.45	3.26	3.38
3.41	5.66	5.23	4.63	4.36	4.11	4.33	4.45	4.40	4.34	3.84	3.17
3.46	5.08	5.18	5.23	4.85	4.39	3.95	3.60	3.33	3.47	3.60	3.73
3.51	5.34	4.92	4.66	4.66	4.68	4.74	4.60	4.32	3.82	3.32	3.09
3.56	5.39	5.14	4.72	4.18	3.66	3.61	3.67	3.89	3.86	3.85	3.60
3.61	4.83	4.86	4.82	4.65	4.37	4.13	3.82	3.73	3.38	3.50	3.50
3.66	5.05	4.58	4.31	4.14	4.13	4.36	4.32	4.11	3.72	3.12	2.77
3.71	5.31	5.25	5.19	4.67	4.37	3.98	3.66	3.60	3.79	3.84	3.80
3.76	5.16	4.99	5.01	4.93	4.75	4.56	4.31	3.92	3.73	3.48	3.52
3.81	4.95	4.71	4.45	4.15	3.93	4.05	4.02	3.87	3.60	3.34	2.90
3.86	4.87	4.91	4.78	4.49	4.10	3.72	3.57	3.35	3.31	3.56	3.24
3.91	5.09	4.71	4.56	4.54	4.48	4.49	4.35	4.02	3.50	3.15	2.91
3.96	5.40	5.04	4.92	4.54	4.13	4.05	3.86	3.76	3.79	3.56	3.18
4.01	5.02	4.96	5.07	4.91	4.69	4.37	3.98	3.67	3.56	3.58	3.53
4.06	5.04	4.85	4.59	4.45	4.39	4.27	4.13	4.00	3.57	3.39	3.06
4.11	5.22	4.90	4.62	4.29	3.93	3.71	3.53	3.51	3.55	3.32	3.10
4.16	4.79	4.67	4.66	4.60	4.53	4.15	3.85	3.55	3.27	3.09	3.08
4.21	5.94	5.52	5.17	4.81	4.67	4.52	4.35	4.24	3.94	3.61	3.23
4.26	5.17	5.08	5.01	4.74	4.38	4.13	3.84	3.76	3.57	3.51	3.44
4.31	4.92	4.62	4.59	4.54	4.47	4.25	4.00	3.82	3.34	3.12	2.83
4.36	4.86	4.65	4.41	4.10	4.02	3.99	3.92	3.65	3.46	3.20	2.73
4.41	5.00	4.78	4.69	4.53	4.31	3.87	3.65	3.37	3.25	3.16	3.00
4.46	5.26	5.13	4.91	4.74	4.69	4.54	4.38	4.20	3.92	3.49	3.26
4.51	5.21	5.17	5.06	4.63	4.32	4.03	3.90	3.90	3.83	3.62	3.59
4.56	4.96	4.78	4.77	4.52	4.39	4.15	3.80	3.62	3.53	3.22	3.19
4.61	4.75	4.49	4.35	4.22	4.11	3.97	3.75	3.38	3.12	2.91	2.63
4.66	4.86	4.67	4.59	4.21	3.91	3.76	3.45	3.48	3.30	3.13	3.03
4.71	4.87	4.52	4.58	4.48	4.37	4.16	3.99	3.80	3.46	3.29	3.13

4.76	5.49	5.24	4.89	4.65	4.49	4.36	4.33	4.17	4.08	3.75	3.52
4.81	4.77	4.73	4.55	4.33	4.13	3.88	3.65	3.45	3.33	3.23	3.14
4.86	4.81	4.62	4.26	4.20	4.06	3.98	3.75	3.54	3.21	2.94	2.74
4.91	5.12	4.93	4.67	4.35	4.24	4.05	3.93	3.77	3.68	3.54	3.15
4.96	4.94	4.79	4.69	4.49	4.25	4.09	3.91	3.56	3.42	3.19	3.12
5.01	4.83	4.57	4.41	4.29	4.09	3.93	3.88	3.68	3.36	3.08	2.99
5.06	5.04	4.85	4.76	4.46	4.30	4.09	3.94	3.72	3.70	3.49	3.30
5.11	4.54	4.34	4.33	4.16	4.02	3.71	3.66	3.42	3.17	3.04	2.82
5.16	4.65	4.45	4.25	3.86	3.86	3.66	3.51	3.30	3.25	2.90	2.54
5.21	4.97	4.86	4.70	4.48	4.28	4.01	3.76	3.62	3.47	3.41	3.15
5.26	4.90	4.67	4.48	4.27	4.28	4.00	3.86	3.72	3.43	3.18	3.00
5.31	4.94	4.61	4.44	4.18	3.91	3.74	3.67	3.60	3.42	3.18	2.98
5.38	4.79	4.61	4.51	4.14	3.98	3.88	3.71	3.54	3.42	3.10	2.87
5.45	4.98	4.78	4.53	4.45	4.20	3.93	3.88	3.80	3.55	3.32	3.01
5.53	4.57	4.29	4.23	3.99	3.72	3.56	3.54	3.34	3.32	3.16	3.02
5.61	4.92	4.60	4.54	4.35	4.16	3.88	3.66	3.52	3.33	3.17	3.05
5.70	4.65	4.39	4.26	4.09	3.90	3.79	3.62	3.50	3.29	3.11	2.85
5.80	4.69	4.58	4.32	4.21	4.08	3.78	3.62	3.41	3.18	3.05	2.91
5.90	4.55	4.56	4.40	4.23	4.04	3.80	3.63	3.48	3.18	3.11	2.94
6.01	4.81	4.72	4.53	4.37	4.15	3.93	3.73	3.44	3.36	3.12	2.99
6.13	4.64	4.51	4.24	4.00	3.93	3.71	3.48	3.34	3.21	2.79	2.77
6.25	4.42	4.12	4.05	3.77	3.54	3.54	3.22	3.10	2.87	2.74	2.56
6.39	4.48	4.28	4.17	3.85	3.73	3.53	3.40	3.14	3.01	2.68	2.51
6.53	4.34	4.27	4.17	3.88	3.84	3.66	3.40	3.27	3.01	2.85	2.66
6.69	4.75	4.58	4.38	4.18	4.04	3.87	3.66	3.58	3.31	2.98	2.80
6.85	4.50	4.37	4.13	3.93	3.70	3.50	3.28	3.18	2.96	2.76	2.67
7.03	4.47	4.22	4.13	3.98	3.72	3.63	3.41	3.19	3.05	2.74	2.65
7.21	4.38	4.29	3.97	3.84	3.63	3.57	3.35	3.10	3.00	2.87	2.57
7.41	4.34	4.21	4.04	3.78	3.64	3.46	3.40	3.16	2.85	2.72	2.56
7.63	4.70	4.51	4.34	4.20	4.06	3.79	3.64	3.41	3.24	3.07	2.92
7.86	4.39	4.18	4.12	3.81	3.65	3.48	3.34	3.17	3.01	2.71	2.56
8.10	4.23	4.10	4.00	3.69	3.49	3.30	3.06	2.95	2.88	2.65	2.48
8.36	4.12	3.96	3.72	3.59	3.44	3.18	3.00	2.81	2.67	2.56	2.21
8.64	4.36	4.15	3.99	3.79	3.53	3.48	3.19	2.94	2.84	2.72	2.42
8.94	4.56	4.39	4.05	3.87	3.69	3.47	3.33	3.10	3.02	2.69	2.53
9.26	4.82	4.51	4.42	4.01	3.91	3.67	3.45	3.25	3.08	2.72	2.62
9.60	4.07	3.89	3.71	3.51	3.36	3.16	3.03	2.80	2.72	2.51	2.45
9.97	4.00	3.91	3.80	3.60	3.47	3.21	3.12	2.97	2.84	2.70	2.52
10.36	4.10	3.98	3.62	3.60	3.39	3.25	3.08	2.84	2.69	2.46	2.40
10.77	4.52	4.26	4.13	4.06	3.77	3.65	3.44	3.30	3.01	2.78	2.68
11.22	4.42	4.19	4.12	3.75	3.40	3.26	3.04	2.74	2.67	2.48	2.46
11.70	4.09	3.93	3.78	3.56	3.36	3.13	3.07	2.93	2.71	2.46	2.31
12.21	3.62	3.46	3.32	3.12	2.98	2.96	2.72	2.63	2.44	2.27	2.11
12.75	4.12	3.82	3.76	3.50	3.40	3.26	3.05	2.82	2.71	2.62	2.41
13.33	4.17	3.93	3.86	3.57	3.49	3.37	3.17	3.02	2.77	2.66	2.54
13.95	4.01	4.01	3.63	3.43	3.25	3.23	3.04	2.79	2.68	2.59	2.44
14.62	3.86	3.70	3.58	3.36	3.18	3.04	2.94	2.68	2.51	2.34	2.20
15.33	3.81	3.60	3.39	3.26	3.03	2.87	2.70	2.52	2.27	2.06	1.89
16.09	3.66	3.55	3.53	3.14	3.05	2.92	2.74	2.67	2.48	2.25	2.03
16.90	4.07	3.94	3.67	3.43	3.30	3.16	2.91	2.84	2.63	2.43	2.27

17.77	3.97	3.66	3.52	3.45	3.24	3.04	2.80	2.75	2.50	2.32	2.24
18.70	3.93	3.81	3.68	3.40	3.15	2.99	2.76	2.67	2.47	2.24	2.00
19.69	3.84	3.56	3.49	3.27	3.06	2.86	2.73	2.63	2.49	2.23	2.00
20.75	3.59	3.37	3.30	3.11	2.94	2.85	2.65	2.39	2.20	2.00	1.85
21.89	3.91	3.80	3.58	3.33	3.12	3.01	2.81	2.57	2.48	2.31	2.10
23.10	4.24	3.88	3.77	3.49	3.42	3.23	2.96	2.83	2.67	2.50	2.38
24.39	3.68	3.48	3.29	3.07	2.95	2.76	2.59	2.56	2.26	2.15	2.01
25.78	3.86	3.64	3.37	3.14	3.00	2.82	2.65	2.54	2.39	2.16	2.01
27.26	3.67	3.30	3.34	3.09	2.91	2.80	2.45	2.35	2.18	1.94	1.89
28.84	3.52	3.33	3.21	3.09	3.00	2.79	2.61	2.54	2.37	2.23	2.02
30.53	3.83	3.61	3.31	3.26	3.00	2.83	2.60	2.51	2.29	2.11	2.00
32.34	3.43	3.25	3.10	2.96	2.78	2.59	2.47	2.39	2.19	2.02	1.79
34.27	3.40	3.27	3.15	2.93	2.85	2.67	2.52	2.36	2.24	2.06	1.90
36.34	3.47	3.22	3.01	2.90	2.57	2.63	2.24	2.22	1.97	1.76	1.64
38.55	3.11	3.02	2.81	2.78	2.50	2.44	2.36	2.14	2.09	1.89	1.80
40.91	3.41	3.28	3.09	2.77	2.72	2.52	2.33	2.06	2.04	1.81	1.68
43.43	3.07	2.98	2.79	2.59	2.54	2.32	2.20	2.08	1.89	1.84	1.68
46.13	3.46	3.10	2.91	2.85	2.62	2.47	2.19	2.09	1.87	1.78	1.60
49.01	3.41	3.23	3.03	2.82	2.72	2.42	2.19	2.20	2.11	1.94	1.69
52.09	3.45	3.31	3.15	2.86	2.82	2.66	2.38	2.27	2.22	2.00	1.79
55.39	3.04	2.80	2.73	2.59	2.40	2.20	2.06	2.02	1.86	1.61	1.49
58.91	3.06	2.92	2.66	2.55	2.40	2.31	2.11	1.97	1.72	1.62	1.55
62.68	3.25	2.99	2.89	2.77	2.54	2.39	2.21	2.11	1.88	1.74	1.59
66.70	3.15	2.94	2.72	2.63	2.37	2.24	2.12	1.92	1.79	1.58	1.44
71.00	3.20	2.94	2.76	2.68	2.39	2.25	2.06	1.95	1.73	1.59	1.41
75.60	3.29	3.01	2.79	2.65	2.43	2.29	2.11	1.89	1.69	1.52	1.26
80.51	2.66	2.48	2.39	2.16	2.11	2.03	1.83	1.69	1.59	1.53	1.46
85.77	3.22	3.04	2.82	2.72	2.62	2.43	2.30	2.04	2.06	1.73	1.62
91.38	2.88	2.71	2.61	2.39	2.25	2.09	1.89	1.75	1.67	1.41	1.42
97.39	3.04	2.72	2.57	2.36	2.23	2.06	1.92	1.73	1.58	1.48	1.30
103.80	3.19	2.97	2.73	2.56	2.43	2.28	2.07	1.86	1.68	1.57	1.40
110.66	2.78	2.54	2.46	2.27	2.13	1.96	1.74	1.56	1.51	1.23	1.14
117.99	2.84	2.77	2.51	2.41	2.15	2.01	1.74	1.52	1.32	1.20	1.07
125.83	2.93	2.72	2.47	2.24	2.06	1.99	1.81	1.67	1.54	1.41	1.19
134.21	2.75	2.50	2.36	2.17	1.91	1.79	1.65	1.54	1.37	1.03	0.97
143.17	2.71	2.56	2.42	2.12	1.97	1.70	1.66	1.47	1.37	1.15	0.95
152.74	2.47	2.27	2.10	1.88	1.71	1.59	1.41	1.32	1.23	1.11	0.91
162.97	2.58	2.37	2.17	1.97	1.88	1.73	1.52	1.43	1.24	1.01	0.96
173.91	2.43	2.17	2.07	1.89	1.73	1.56	1.36	1.25	1.07	0.83	0.81
185.60	2.32	2.17	2.01	1.92	1.71	1.63	1.38	1.30	1.26	1.04	0.85
198.10	2.48	2.30	2.15	1.96	1.75	1.63	1.43	1.28	1.04	0.89	0.74
211.46	2.63	2.27	2.21	2.01	1.77	1.55	1.42	1.33	1.07	0.96	0.71
225.75	2.39	2.36	2.05	1.87	1.71	1.48	1.34	1.14	1.12	0.92	0.61
241.01	2.39	2.17	1.97	1.78	1.61	1.33	1.24	1.14	0.99	0.78	0.64
257.33	2.26	2.11	1.88	1.68	1.41	1.24	1.21	1.06	0.87	0.70	0.64
274.77	2.11	2.09	1.93	1.66	1.46	1.36	1.24	1.06	0.95	0.66	0.52
293.42	2.17	2.05	1.80	1.66	1.42	1.35	1.15	0.90	0.84	0.67	0.46
313.35	2.06	1.86	1.67	1.45	1.30	1.12	0.93	0.83	0.57	0.44	0.26
334.66	2.26	2.15	1.90	1.70	1.58	1.37	1.30	1.08	0.90	0.74	0.61
357.43	2.19	2.02	1.77	1.75	1.48	1.33	1.07	1.01	0.90	0.73	0.51

381.78	1.93	1.74	1.54	1.36	1.21	1.09	0.87	0.71	0.57	0.41	0.28
407.80	2.01	1.86	1.62	1.51	1.29	1.20	1.00	0.94	0.79	0.62	0.47
435.62	2.30	1.92	1.70	1.49	1.42	1.16	0.99	0.91	0.73	0.51	0.41
465.35	1.89	1.51	1.47	1.15	1.05	0.79	0.71	0.61	0.44	0.30	0.27
497.14	1.73	1.52	1.49	1.30	1.06	0.96	0.77	0.59	0.39	0.36	0.20
531.11	1.70	1.47	1.31	1.20	0.99	0.77	0.64	0.52	0.48	0.16	0.08
567.43	1.77	1.54	1.33	1.27	1.09	0.91	0.76	0.53	0.50	0.25	0.17
606.26	1.64	1.44	1.40	1.18	0.96	0.83	0.76	0.63	0.38	0.23	0.22
647.76	1.47	1.34	1.14	1.00	0.79	0.69	0.43	0.30	0.31	0.09	-0.02
692.12	1.64	1.36	1.27	1.07	0.92	0.81	0.52	0.35	0.35	-0.01	-0.20
739.53	1.62	1.47	1.26	1.07	0.92	0.76	0.58	0.54	0.36	0.12	0.12
790.22	1.60	1.49	1.40	1.16	0.90	0.87	0.64	0.57	0.39	0.15	0.06
844.40	1.68	1.58	1.40	1.18	0.96	0.89	0.67	0.54	0.41	0.31	0.12
902.32	1.55	1.43	1.25	1.01	0.93	0.72	0.53	0.44	0.22	0.12	-0.05
964.23	1.49	1.32	1.06	1.02	0.90	0.79	0.56	0.50	0.19	0.08	-0.06
1030.41	1.30	1.16	0.98	0.83	0.67	0.56	0.32	0.29	0.01	-0.01	-0.30
1101.15	1.39	1.25	1.03	0.77	0.64	0.36	0.37	0.12	-0.11	-0.26	-0.50
1176.77	1.52	1.26	1.07	0.87	0.66	0.62	0.42	0.28	0.12	-0.08	-0.27
1257.60	1.31	1.23	0.98	0.82	0.58	0.47	0.37	0.13	0.05	0.04	-0.27
1344.00	1.41	1.30	0.94	0.80	0.71	0.48	0.26	0.19	0.09	-0.15	-0.32
1436.36	1.20	0.98	0.83	0.59	0.44	0.21	0.07	0.02	-0.28	-0.47	-0.63
1535.09	1.20	0.99	0.87	0.63	0.46	0.27	0.18	0.13	0.01	-0.30	-0.43
1640.63	1.40	1.26	0.99	0.81	0.58	0.50	0.31	0.16	0.01	-0.23	-0.35
1753.44	1.09	0.87	0.69	0.43	0.23	0.13	-0.06	-0.32	-0.36	-0.58	-0.67
1874.02	1.24	1.07	0.99	0.72	0.53	0.43	0.23	0.06	-0.10	-0.30	-0.40
2002.92	1.05	0.79	0.63	0.32	0.25	0.02	-0.19	-0.29	-0.36	-0.54	-0.64
2140.71	1.03	0.80	0.64	0.31	0.20	0.12	-0.11	-0.34	-0.46	-0.57	-0.73
2288.00	0.80	0.66	0.52	0.23	0.19	0.03	-0.12	-0.31	-0.39	-0.56	-0.74
2445.44	1.16	0.82	0.80	0.46	0.29	0.24	-0.01	-0.19	-0.28	-0.51	-0.61
2613.73	1.18	0.99	0.80	0.67	0.45	0.27	-0.02	-0.13	-0.32	-0.50	-0.54
2793.63	0.91	0.73	0.55	0.32	0.18	-0.08	-0.13	-0.21	-0.39	-0.56	-0.77
2985.93	0.66	0.59	0.36	0.13	-0.03	-0.15	-0.31	-0.48	-0.42	-0.60	-0.70
3191.49	1.23	1.01	0.83	0.71	0.56	0.27	0.18	0.08	-0.09	-0.30	-0.34
3411.21	1.13	0.85	0.70	0.47	0.37	0.32	0.09	-0.08	-0.21	-0.41	-0.40
3646.09	1.82	1.56	1.52	1.18	1.01	0.80	0.61	0.48	0.29	0.08	-0.20
3897.16	1.57	1.34	1.11	0.90	0.77	0.61	0.45	0.31	0.05	-0.01	-0.28

Wavelength (nm) / Time (ps)	459.41	460.83	462.25	463.68	465.10	466.53	467.95	469.37	470.80	472.22	473.65
-2.74	-0.11	-0.14	-0.13	-0.22	-0.17	-0.22	-0.19	-0.19	-0.22	-0.19	-0.22
-2.24	0.00	0.04	0.17	0.09	0.13	0.14	0.12	0.08	0.21	0.18	0.21
-1.74	0.12	0.08	0.17	0.19	0.17	0.20	0.13	0.15	0.14	0.25	0.13
-1.24	-0.07	0.00	-0.03	0.01	-0.03	-0.03	-0.13	-0.05	0.04	-0.03	-0.10
-0.74	-0.04	-0.12	-0.19	-0.17	-0.17	-0.18	-0.18	-0.11	-0.10	-0.07	-0.19
-0.24	0.11	0.06	0.04	0.04	0.08	-0.05	0.00	0.07	-0.05	-0.08	0.10
-0.14	-0.13	-0.14	-0.14	0.00	-0.18	-0.09	0.04	-0.06	-0.13	-0.18	-0.10
-0.04	0.12	0.23	0.12	0.06	0.17	0.23	0.20	0.10	0.10	0.12	0.18
0.06	-0.15	-0.14	-0.08	-0.08	-0.10	-0.15	-0.20	-0.06	-0.10	-0.10	-0.16
0.16	-0.04	-0.01	0.00	0.04	-0.11	0.00	0.12	0.10	0.06	-0.02	0.06

0.26	-0.10	-0.17	-0.11	-0.10	-0.02	-0.05	-0.04	0.01	-0.06	-0.02	-0.06
0.31	0.03	0.06	0.10	0.25	0.01	0.03	0.10	0.00	0.15	0.11	0.17
0.36	0.08	0.16	0.12	0.06	-0.11	-0.07	0.08	0.03	-0.01	0.05	-0.02
0.41	-0.07	0.05	-0.09	-0.07	-0.09	-0.10	-0.10	-0.03	-0.05	0.03	-0.08
0.46	-0.11	0.05	-0.05	-0.07	-0.03	-0.10	-0.09	-0.08	-0.07	0.08	-0.07
0.51	0.07	0.00	-0.01	0.20	0.13	0.02	0.10	0.01	0.09	0.18	0.17
0.56	-0.03	-0.13	0.04	0.06	-0.02	0.01	0.13	0.01	0.01	0.03	-0.01
0.61	-0.06	-0.05	-0.01	0.03	0.04	-0.06	-0.02	0.02	-0.14	-0.04	-0.05
0.66	-0.09	-0.01	-0.09	0.05	-0.11	-0.02	0.01	0.02	-0.04	0.02	-0.09
0.71	0.00	0.03	-0.05	-0.03	-0.13	-0.04	0.00	-0.04	0.02	-0.03	-0.06
0.76	-0.06	-0.03	-0.03	0.03	0.03	-0.04	-0.06	0.03	-0.05	-0.03	-0.03
0.81	-0.07	0.09	0.04	-0.05	-0.14	-0.10	-0.07	0.00	-0.13	-0.05	-0.17
0.86	-0.03	0.06	0.01	0.04	0.08	0.02	-0.02	-0.01	-0.07	0.02	-0.05
0.91	-0.17	0.04	-0.07	0.09	-0.03	0.12	0.04	-0.02	0.04	0.00	0.00
0.96	0.07	-0.03	0.07	0.10	-0.05	-0.07	0.19	0.07	-0.03	0.07	0.00
1.01	-0.27	0.05	0.22	0.12	-0.14	0.06	0.19	0.30	0.07	0.25	0.39
1.06	2.75	1.75	-0.17	-1.46	-1.75	-0.94	-0.34	-0.09	-0.10	-0.22	-0.26
1.11	19.96	-10.01	-34.00	-51.82	-64.04	-68.88	-66.62	-57.91	-44.70	-29.65	-15.98
1.16	464.15	440.85	413.24	380.86	343.11	299.50	252.11	199.90	145.52	93.12	47.85
1.21	-81.78	-19.38	53.86	132.27	213.12	292.41	367.76	430.81	475.21	496.85	501.66
1.26	16.13	11.34	12.07	17.60	21.96	18.44	2.38	-25.16	-59.46	-93.92	-123.98
1.31	10.31	8.99	6.42	3.87	2.26	1.26	0.98	2.73	7.62	14.59	20.72
1.36	2.07	4.55	7.93	10.84	12.23	11.81	10.08	7.69	5.70	5.00	5.33
1.41	8.11	5.96	4.33	4.04	5.08	7.27	9.32	10.27	9.68	7.71	4.62
1.46	8.57	10.72	11.09	9.14	5.82	2.54	0.25	-0.29	1.19	3.81	6.68
1.51	3.78	2.60	2.87	4.26	6.05	7.45	7.68	6.73	4.91	3.03	1.58
1.56	7.52	4.98	2.36	0.81	0.85	2.60	5.35	7.96	9.35	9.19	7.32
1.61	6.45	8.10	8.62	7.64	5.49	3.26	1.86	1.47	2.39	4.22	6.05
1.66	3.46	2.85	3.41	4.88	6.52	7.43	7.50	6.02	4.04	2.13	0.67
1.71	7.80	7.71	6.17	4.09	1.89	0.70	0.68	1.90	3.63	5.43	6.58
1.76	2.35	3.37	5.09	6.43	6.97	6.30	4.97	3.18	1.98	1.70	2.39
1.81	4.00	2.61	1.99	2.16	3.25	4.88	6.01	6.25	5.49	4.05	2.30
1.86	6.60	6.23	4.99	3.22	1.85	1.01	1.22	2.23	3.41	4.48	4.79
1.91	1.83	2.32	3.28	4.51	5.39	5.30	4.47	2.80	1.40	0.49	0.43
1.96	5.42	4.12	2.50	1.52	1.37	2.27	3.63	4.90	5.56	5.53	4.42
2.01	3.75	5.30	6.53	6.37	5.27	3.30	1.62	0.61	0.63	1.57	3.12
2.06	3.27	2.27	2.23	2.72	3.59	4.44	4.77	4.36	3.36	2.01	0.91
2.11	4.95	3.96	2.70	1.57	1.12	1.37	2.22	3.38	4.24	4.50	4.14
2.16	2.43	3.37	4.41	5.05	4.88	4.17	2.96	1.72	1.14	1.18	1.76
2.21	3.99	2.76	2.20	2.19	2.87	3.84	4.68	5.03	4.52	3.57	2.14
2.26	5.09	5.53	5.22	4.29	2.89	1.76	1.10	1.28	2.15	3.15	3.90
2.31	2.28	2.16	2.53	3.15	3.69	3.80	3.53	2.70	1.82	1.27	0.96
2.36	3.17	2.69	2.41	2.46	2.82	3.28	3.55	3.46	3.08	2.50	1.74
2.41	4.35	4.93	4.89	4.13	2.87	1.76	1.02	0.95	1.50	2.30	3.10
2.46	2.60	2.03	1.97	2.44	3.01	3.58	3.74	3.35	2.68	1.70	1.00
2.51	5.24	5.15	4.20	3.03	1.85	1.26	1.41	2.07	3.05	4.09	4.43
2.56	2.87	3.47	3.93	4.08	3.79	3.22	2.38	1.75	1.44	1.50	1.73
2.61	2.41	2.14	2.35	2.73	3.16	3.48	3.28	2.66	1.99	1.33	0.87
2.66	4.37	4.07	3.38	2.39	1.64	1.51	1.67	2.22	2.94	3.27	3.39
2.71	2.28	2.65	3.32	3.71	3.81	3.45	2.77	1.93	1.39	1.09	1.23

2.76	4.12	3.26	2.69	2.20	2.25	2.59	3.18	3.58	3.60	3.17	2.63
2.81	3.78	3.89	3.70	3.25	2.41	1.77	1.35	1.36	1.59	2.11	2.53
2.86	2.44	2.48	2.75	3.06	3.05	2.91	2.42	1.87	1.41	1.11	0.98
2.91	3.91	3.32	2.76	2.10	1.74	1.94	2.21	2.66	3.04	3.01	2.63
2.96	3.28	3.59	3.82	3.59	3.09	2.60	1.79	1.45	1.42	1.79	2.11
3.01	3.32	2.62	2.26	2.19	2.27	2.64	3.06	3.14	2.81	2.50	1.75
3.06	3.77	3.68	3.27	2.66	1.99	1.66	1.74	1.77	2.17	2.50	2.65
3.11	2.69	2.85	2.97	3.15	2.84	2.43	2.01	1.57	1.36	1.40	1.60
3.16	3.10	2.61	2.06	1.88	2.00	2.38	2.61	2.73	2.62	2.32	1.70
3.21	3.50	3.37	3.09	2.68	2.17	1.94	1.92	1.92	2.10	2.34	2.37
3.26	2.79	2.65	2.75	2.98	2.96	2.96	2.70	2.42	2.06	1.69	1.41
3.31	3.64	3.11	2.71	2.34	2.15	2.31	2.57	2.67	2.84	2.66	2.33
3.36	3.45	3.61	3.54	3.31	2.93	2.39	2.19	2.00	2.07	2.41	2.43
3.41	2.81	2.48	2.53	2.61	2.73	2.90	2.83	2.57	2.09	1.77	1.37
3.46	3.51	3.24	2.81	2.27	1.90	1.81	1.94	2.11	2.43	2.62	2.34
3.51	2.97	3.09	3.04	3.19	3.01	2.74	2.43	2.00	1.82	1.81	1.80
3.56	3.16	2.70	2.26	2.15	2.33	2.61	2.74	2.67	2.42	2.15	1.75
3.61	3.47	3.16	2.88	2.61	2.19	2.05	1.95	2.01	2.23	2.18	2.17
3.66	2.52	2.44	2.53	2.73	2.75	2.53	2.30	1.84	1.51	1.31	1.20
3.71	3.61	3.26	2.92	2.55	2.26	2.41	2.45	2.68	2.77	2.65	2.58
3.76	3.45	3.35	3.40	3.07	2.82	2.48	2.37	2.18	2.14	2.20	2.24
3.81	2.60	2.33	2.48	2.45	2.49	2.48	2.30	2.18	1.80	1.62	1.42
3.86	3.15	2.69	2.50	2.08	1.82	1.80	1.85	1.91	2.01	1.98	1.78
3.91	2.83	2.94	2.90	2.93	2.74	2.40	2.15	1.83	1.57	1.68	1.63
3.96	3.00	2.62	2.36	2.28	2.35	2.43	2.50	2.40	2.23	1.93	1.58
4.01	3.59	3.47	3.21	2.83	2.38	2.12	2.03	2.06	2.21	2.28	2.43
4.06	2.86	2.92	2.94	2.90	2.70	2.52	2.27	2.02	1.77	1.86	1.67
4.11	2.72	2.41	2.05	1.98	1.90	1.99	2.04	1.98	1.82	1.69	1.43
4.16	3.12	3.12	3.00	2.77	2.37	2.16	2.00	1.79	1.91	1.92	2.00
4.21	2.94	2.68	2.55	2.55	2.43	2.51	2.36	2.08	1.79	1.72	1.50
4.26	3.28	3.02	2.84	2.44	2.29	2.17	2.03	2.02	2.11	2.13	2.12
4.31	2.82	2.84	2.78	2.59	2.34	2.09	1.93	1.58	1.42	1.57	1.56
4.36	2.63	2.55	2.43	2.35	2.24	2.26	2.06	1.87	1.67	1.58	1.44
4.41	2.96	2.79	2.52	2.15	1.82	1.70	1.67	1.73	1.73	1.89	1.75
4.46	3.12	3.04	3.02	2.95	2.78	2.62	2.44	2.14	1.97	1.88	1.91
4.51	3.27	2.93	2.77	2.61	2.33	2.39	2.37	2.27	2.28	2.14	1.97
4.56	3.18	2.88	2.74	2.53	2.14	2.03	1.86	1.89	1.98	1.89	1.82
4.61	2.52	2.52	2.41	2.27	2.19	1.81	1.71	1.46	1.37	1.32	1.39
4.66	2.93	2.63	2.31	2.17	2.05	1.96	2.01	2.09	1.89	1.86	1.62
4.71	3.05	3.06	3.04	2.84	2.72	2.31	2.22	2.08	1.97	1.98	1.93
4.76	3.17	2.94	2.77	2.69	2.68	2.62	2.47	2.41	2.33	1.98	1.77
4.81	2.89	2.71	2.55	2.32	2.05	1.98	1.96	1.88	1.99	1.81	1.79
4.86	2.65	2.52	2.48	2.30	2.14	1.93	1.70	1.52	1.48	1.47	1.40
4.91	2.99	2.70	2.65	2.54	2.40	2.36	2.25	2.08	1.91	1.90	1.71
4.96	3.09	2.96	2.85	2.63	2.16	2.05	1.90	1.72	1.72	1.77	1.74
5.01	2.83	2.74	2.65	2.43	2.37	2.31	2.16	1.99	1.80	1.72	1.66
5.06	3.12	2.88	2.58	2.45	2.32	2.26	2.28	2.08	2.11	1.92	1.77
5.11	2.78	2.70	2.58	2.29	2.18	1.95	1.79	1.76	1.84	1.78	1.62
5.16	2.44	2.36	2.24	2.25	2.20	1.98	1.91	1.69	1.56	1.51	1.40
5.21	2.98	2.73	2.56	2.24	2.13	1.96	2.01	1.72	1.70	1.67	1.50

5.26	2.75	2.75	2.64	2.54	2.34	2.31	2.22	1.96	1.85	1.80	1.74
5.31	2.78	2.49	2.47	2.21	2.26	2.13	2.16	1.93	1.78	1.72	1.60
5.38	2.82	2.47	2.40	2.24	2.11	2.14	1.90	1.78	1.66	1.52	1.40
5.45	2.88	2.78	2.67	2.43	2.28	2.20	1.95	1.91	1.82	1.77	1.50
5.53	2.88	2.64	2.45	2.40	2.11	2.14	2.00	1.98	1.93	1.88	1.65
5.61	2.92	2.81	2.57	2.45	2.20	2.11	1.99	1.92	1.92	1.87	1.75
5.70	2.74	2.58	2.30	2.29	2.11	2.10	2.03	1.89	1.76	1.68	1.53
5.80	2.76	2.63	2.33	2.24	2.06	1.98	1.68	1.81	1.70	1.62	1.69
5.90	2.80	2.57	2.42	2.25	2.03	1.87	1.74	1.80	1.74	1.65	1.54
6.01	2.89	2.73	2.55	2.44	2.25	2.18	2.07	1.98	1.93	1.98	1.83
6.13	2.66	2.42	2.32	2.19	1.99	1.90	1.81	1.64	1.59	1.63	1.57
6.25	2.31	2.16	2.02	1.87	1.81	1.63	1.69	1.49	1.34	1.32	1.13
6.39	2.38	2.19	2.17	1.94	1.80	1.69	1.56	1.45	1.41	1.38	1.35
6.53	2.56	2.38	2.15	2.05	1.89	1.85	1.56	1.49	1.48	1.46	1.37
6.69	2.63	2.42	2.33	2.26	2.09	1.98	2.00	1.80	1.64	1.57	1.52
6.85	2.47	2.26	2.17	1.93	1.82	1.70	1.61	1.55	1.43	1.37	1.28
7.03	2.41	2.28	2.10	2.02	1.88	1.67	1.69	1.53	1.53	1.47	1.33
7.21	2.48	2.31	2.20	2.12	1.94	1.88	1.72	1.62	1.57	1.57	1.35
7.41	2.41	2.23	2.17	1.93	1.91	1.84	1.84	1.61	1.54	1.44	1.48
7.63	2.64	2.58	2.46	2.26	2.14	2.04	1.93	1.79	1.89	1.78	1.60
7.86	2.43	2.20	2.11	2.03	1.78	1.70	1.71	1.71	1.52	1.52	1.31
8.10	2.36	2.19	1.99	1.92	1.78	1.68	1.65	1.52	1.41	1.35	1.22
8.36	2.13	1.79	1.84	1.64	1.46	1.38	1.24	1.21	1.17	1.12	1.05
8.64	2.25	2.05	2.01	1.89	1.74	1.55	1.56	1.41	1.32	1.26	1.21
8.94	2.53	2.30	2.12	1.95	1.87	1.69	1.61	1.57	1.51	1.49	1.38
9.26	2.55	2.41	2.30	2.04	1.81	1.79	1.76	1.66	1.56	1.58	1.51
9.60	2.32	2.08	1.98	1.95	1.66	1.63	1.51	1.45	1.39	1.39	1.33
9.97	2.35	2.23	2.13	1.87	1.79	1.73	1.71	1.53	1.47	1.42	1.49
10.36	2.22	2.06	2.01	1.77	1.68	1.55	1.48	1.42	1.35	1.32	1.33
10.77	2.45	2.30	2.12	1.93	1.78	1.65	1.64	1.53	1.50	1.53	1.41
11.22	2.20	1.99	1.92	1.87	1.65	1.53	1.57	1.42	1.37	1.31	1.29
11.70	2.25	2.02	1.95	1.76	1.62	1.65	1.53	1.48	1.36	1.35	1.27
12.21	2.02	1.92	1.74	1.71	1.48	1.42	1.41	1.28	1.26	1.19	1.07
12.75	2.27	2.15	2.02	1.78	1.75	1.60	1.54	1.56	1.38	1.37	1.31
13.33	2.33	2.22	2.13	1.95	1.82	1.77	1.62	1.61	1.55	1.52	1.49
13.95	2.22	2.01	1.84	1.93	1.68	1.58	1.48	1.51	1.27	1.41	1.32
14.62	2.02	1.96	1.83	1.73	1.55	1.51	1.37	1.32	1.16	1.30	1.24
15.33	1.81	1.69	1.48	1.47	1.27	1.21	1.13	1.02	1.04	0.95	0.85
16.09	2.02	1.81	1.78	1.60	1.46	1.47	1.31	1.29	1.09	1.09	1.03
16.90	2.20	2.03	1.79	1.80	1.50	1.47	1.42	1.27	1.27	1.24	1.16
17.77	2.06	1.86	1.91	1.70	1.64	1.43	1.43	1.38	1.18	1.20	1.14
18.70	1.80	1.67	1.61	1.45	1.26	1.22	1.21	1.11	0.92	1.03	0.97
19.69	1.99	1.91	1.71	1.64	1.49	1.34	1.31	1.18	1.20	1.20	1.09
20.75	1.82	1.68	1.51	1.38	1.38	1.32	1.08	1.08	1.10	1.01	0.99
21.89	2.00	1.93	1.81	1.66	1.56	1.46	1.34	1.39	1.18	1.20	1.04
23.10	2.18	2.01	1.90	1.86	1.65	1.49	1.51	1.40	1.44	1.40	1.29
24.39	1.85	1.72	1.63	1.48	1.29	1.21	1.16	1.07	1.17	1.07	0.99
25.78	1.94	1.84	1.63	1.62	1.43	1.43	1.30	1.28	1.28	1.14	1.13
27.26	1.72	1.60	1.48	1.35	1.27	1.16	1.21	1.01	1.02	0.98	0.88
28.84	1.84	1.77	1.74	1.59	1.49	1.48	1.44	1.27	1.30	1.27	1.13

30.53	1.88	1.74	1.59	1.47	1.45	1.35	1.29	1.15	1.12	1.11	1.16
32.34	1.83	1.68	1.50	1.46	1.24	1.13	1.15	1.03	0.89	0.99	0.94
34.27	1.84	1.67	1.60	1.49	1.32	1.28	1.22	1.27	1.18	1.15	1.02
36.34	1.42	1.26	1.17	1.16	0.96	0.92	0.82	0.81	0.64	0.75	0.71
38.55	1.76	1.73	1.55	1.49	1.31	1.34	1.21	1.26	1.23	1.21	1.19
40.91	1.64	1.43	1.30	1.35	1.10	1.07	1.03	0.97	0.93	0.95	0.83
43.43	1.55	1.46	1.29	1.31	1.20	1.16	1.10	1.04	0.98	0.93	0.90
46.13	1.39	1.30	1.16	0.97	0.92	0.75	0.86	0.82	0.72	0.71	0.61
49.01	1.57	1.40	1.47	1.10	1.10	1.04	1.05	0.84	0.87	0.97	0.84
52.09	1.58	1.48	1.43	1.24	1.14	1.09	0.90	0.86	0.71	0.87	0.78
55.39	1.38	1.26	1.18	1.10	0.94	0.87	0.94	0.85	0.73	0.75	0.74
58.91	1.45	1.33	1.15	1.06	1.07	1.04	0.92	0.75	0.74	0.76	0.70
62.68	1.54	1.35	1.27	1.15	0.85	0.91	0.92	0.80	0.65	0.66	0.68
66.70	1.37	1.20	1.15	0.92	0.89	0.86	0.71	0.70	0.60	0.72	0.66
71.00	1.19	1.15	1.04	0.94	0.84	0.72	0.66	0.70	0.53	0.60	0.59
75.60	1.26	0.93	0.90	0.83	0.66	0.57	0.49	0.44	0.40	0.50	0.34
80.51	1.38	1.29	1.12	1.05	0.84	0.89	0.96	0.75	0.80	0.80	0.68
85.77	1.37	1.30	1.13	1.12	1.10	0.91	0.89	0.73	0.65	0.64	0.58
91.38	1.24	1.08	0.97	0.84	0.76	0.67	0.64	0.59	0.57	0.57	0.48
97.39	1.18	1.00	0.92	0.74	0.58	0.57	0.63	0.51	0.51	0.46	0.39
103.80	1.29	1.20	1.02	0.93	0.78	0.74	0.77	0.67	0.69	0.60	0.60
110.66	1.10	0.85	0.77	0.69	0.56	0.52	0.51	0.46	0.35	0.32	0.33
117.99	0.74	0.76	0.54	0.37	0.32	0.16	0.18	0.12	0.12	0.10	0.08
125.83	1.17	0.97	0.81	0.75	0.63	0.65	0.56	0.56	0.45	0.48	0.33
134.21	0.94	0.86	0.77	0.69	0.42	0.38	0.43	0.41	0.30	0.32	0.17
143.17	0.85	0.72	0.51	0.46	0.33	0.31	0.36	0.23	0.19	0.28	0.12
152.74	0.81	0.64	0.59	0.47	0.38	0.36	0.29	0.27	0.17	0.25	-0.03
162.97	0.80	0.74	0.73	0.45	0.43	0.25	0.32	0.31	0.38	0.33	0.27
173.91	0.60	0.50	0.46	0.33	0.16	0.10	0.18	0.12	0.09	0.09	0.00
185.60	0.79	0.60	0.71	0.52	0.37	0.31	0.31	0.25	0.30	0.26	0.17
198.10	0.57	0.54	0.55	0.32	0.24	0.10	0.17	0.17	0.02	0.13	-0.01
211.46	0.60	0.47	0.39	0.24	0.20	0.10	0.19	0.13	0.04	0.16	0.02
225.75	0.60	0.50	0.44	0.25	0.22	0.09	-0.01	0.15	0.18	0.14	0.13
241.01	0.53	0.48	0.36	0.20	0.17	0.15	0.12	0.10	0.08	0.08	-0.03
257.33	0.38	0.26	0.21	0.10	-0.02	-0.11	-0.08	-0.23	-0.15	-0.15	-0.10
274.77	0.42	0.37	0.30	0.21	0.05	0.06	0.02	0.07	-0.19	0.04	-0.07
293.42	0.36	0.33	0.18	0.14	0.05	-0.01	-0.08	-0.10	0.01	-0.07	-0.12
313.35	0.19	0.03	-0.01	-0.12	-0.26	-0.18	-0.20	-0.32	-0.23	-0.31	-0.29
334.66	0.52	0.38	0.29	0.19	0.02	-0.01	0.09	-0.01	-0.04	-0.11	0.06
357.43	0.46	0.32	0.19	0.11	0.03	0.05	-0.12	-0.17	-0.30	-0.13	-0.20
381.78	0.21	0.04	0.02	-0.07	-0.26	-0.21	-0.24	-0.27	-0.26	-0.28	-0.48
407.80	0.36	0.28	0.15	-0.06	-0.13	-0.15	-0.23	-0.16	-0.23	-0.24	-0.18
435.62	0.19	0.20	0.05	-0.12	-0.24	-0.26	-0.22	-0.25	-0.39	-0.38	-0.33
465.35	0.02	0.10	-0.11	-0.16	-0.29	-0.28	-0.38	-0.56	-0.42	-0.46	-0.42
497.14	0.04	-0.15	-0.09	-0.19	-0.32	-0.44	-0.32	-0.55	-0.49	-0.55	-0.50
531.11	-0.04	-0.25	-0.33	-0.25	-0.44	-0.47	-0.47	-0.52	-0.63	-0.57	-0.61
567.43	0.07	-0.10	-0.02	-0.10	-0.33	-0.33	-0.29	-0.40	-0.40	-0.32	-0.42
606.26	0.11	-0.04	-0.13	-0.16	-0.26	-0.31	-0.24	-0.34	-0.33	-0.23	-0.39
647.76	-0.05	-0.20	-0.32	-0.36	-0.52	-0.53	-0.42	-0.53	-0.45	-0.56	-0.46
692.12	-0.30	-0.35	-0.44	-0.56	-0.63	-0.68	-0.76	-0.87	-0.74	-0.83	-0.77

739.53	-0.11	-0.16	-0.25	-0.32	-0.43	-0.49	-0.56	-0.50	-0.62	-0.52	-0.60
790.22	-0.03	-0.17	-0.29	-0.42	-0.40	-0.66	-0.60	-0.56	-0.66	-0.65	-0.70
844.40	-0.04	-0.10	-0.29	-0.45	-0.54	-0.60	-0.66	-0.67	-0.70	-0.68	-0.75
902.32	-0.33	-0.25	-0.46	-0.37	-0.55	-0.55	-0.67	-0.61	-0.60	-0.59	-0.64
964.23	-0.24	-0.35	-0.47	-0.47	-0.59	-0.59	-0.63	-0.75	-0.77	-0.70	-0.81
1030.41	-0.32	-0.37	-0.41	-0.57	-0.60	-0.65	-0.73	-0.76	-0.72	-0.60	-0.81
1101.15	-0.50	-0.65	-0.74	-0.77	-0.96	-0.87	-0.96	-0.91	-0.97	-0.90	-1.02
1176.77	-0.27	-0.31	-0.49	-0.69	-0.80	-0.88	-0.78	-0.90	-0.82	-0.92	-0.76
1257.60	-0.40	-0.43	-0.57	-0.68	-0.61	-0.80	-0.74	-0.86	-0.79	-0.75	-0.80
1344.00	-0.51	-0.60	-0.66	-0.83	-0.75	-0.98	-0.92	-0.97	-0.98	-0.91	-0.97
1436.36	-0.55	-0.75	-0.85	-0.94	-1.08	-1.09	-1.03	-1.11	-1.01	-1.02	-1.02
1535.09	-0.49	-0.65	-0.60	-0.81	-0.90	-0.91	-0.97	-0.93	-0.96	-0.86	-0.93
1640.63	-0.52	-0.66	-0.79	-0.87	-1.02	-0.97	-1.08	-1.03	-1.11	-1.00	-1.13
1753.44	-0.75	-0.86	-1.00	-1.01	-1.16	-1.18	-1.18	-1.21	-1.35	-1.36	-1.21
1874.02	-0.49	-0.62	-0.74	-0.81	-0.99	-0.94	-0.98	-1.04	-1.02	-0.96	-0.99
2002.92	-0.79	-0.96	-1.10	-1.16	-1.11	-1.30	-1.20	-1.25	-1.28	-1.21	-1.26
2140.71	-0.93	-0.98	-1.07	-1.14	-1.22	-1.27	-1.31	-1.38	-1.43	-1.33	-1.28
2288.00	-0.79	-0.95	-1.10	-1.11	-1.22	-1.26	-1.33	-1.26	-1.28	-1.29	-1.30
2445.44	-0.64	-0.85	-0.89	-1.06	-1.14	-1.15	-1.35	-1.29	-1.21	-1.24	-1.33
2613.73	-0.75	-0.93	-0.99	-1.00	-1.33	-1.29	-1.25	-1.30	-1.25	-1.26	-1.21
2793.63	-0.83	-0.97	-1.06	-1.08	-1.12	-1.18	-1.18	-1.14	-1.14	-1.11	-0.96
2985.93	-0.85	-0.79	-0.97	-0.92	-0.98	-1.02	-1.07	-0.92	-1.15	-0.91	-1.06
3191.49	-0.56	-0.57	-0.69	-0.85	-0.93	-0.94	-0.92	-1.03	-1.01	-0.99	-0.98
3411.21	-0.45	-0.50	-0.67	-0.71	-0.79	-0.92	-0.87	-0.85	-0.85	-0.84	-0.87
3646.09	-0.21	-0.31	-0.33	-0.64	-0.58	-0.74	-0.77	-0.70	-0.80	-0.74	-0.77
3897.16	-0.32	-0.47	-0.53	-0.52	-0.75	-0.78	-0.72	-0.63	-0.72	-0.68	-0.76

Wavelength (nm) / Time (ps)	475.07	476.49	477.92	479.34	480.77	482.19	483.61	485.04	486.46	487.89	489.31
-2.74	-0.18	-0.22	-0.22	-0.23	-0.26	-0.23	-0.29	-0.23	-0.31	-0.46	-0.41
-2.24	0.15	0.12	0.02	0.16	0.17	0.15	0.24	0.08	0.13	0.04	0.07
-1.74	0.19	0.13	0.13	0.08	0.05	0.14	0.02	0.01	-0.08	0.02	-0.12
-1.24	-0.04	-0.02	0.03	0.01	0.01	0.05	0.03	0.01	0.07	0.03	-0.01
-0.74	-0.13	-0.05	-0.01	0.01	-0.02	-0.13	0.01	0.06	0.10	0.21	0.14
-0.24	-0.08	-0.01	0.03	-0.08	0.01	-0.13	-0.04	0.02	0.02	0.06	0.14
-0.14	-0.09	-0.13	-0.11	-0.08	-0.11	-0.01	-0.02	-0.06	0.01	0.05	0.13
-0.04	0.18	0.16	0.13	0.14	0.14	0.16	0.06	0.12	0.07	0.06	0.05
0.06	-0.18	-0.15	-0.13	-0.12	-0.08	-0.20	-0.14	-0.13	-0.04	-0.07	-0.06
0.16	0.03	0.12	0.05	0.04	0.16	0.11	0.08	0.17	0.32	0.27	0.33
0.26	0.01	-0.05	-0.02	0.01	0.06	0.04	0.10	0.14	0.06	0.21	0.25
0.31	0.11	0.12	0.17	0.14	0.24	0.20	0.21	0.25	0.27	0.23	0.23
0.36	-0.01	-0.03	-0.01	-0.05	-0.03	-0.06	-0.06	-0.11	-0.15	-0.21	-0.17
0.41	-0.05	-0.01	0.00	-0.05	-0.02	-0.03	0.02	-0.03	0.01	0.01	-0.02
0.46	0.07	-0.02	-0.09	-0.05	0.08	-0.01	0.07	0.01	0.10	0.05	0.16
0.51	0.10	0.13	0.08	0.00	0.05	0.06	0.11	0.10	0.01	0.10	0.19
0.56	-0.02	-0.03	-0.04	0.05	0.04	0.06	0.11	0.14	0.04	0.12	0.23
0.61	-0.02	-0.08	-0.07	0.00	-0.01	-0.10	-0.04	-0.09	-0.19	-0.14	-0.08
0.66	0.12	0.01	-0.05	-0.04	0.04	0.05	-0.05	0.02	0.01	-0.04	0.03
0.71	-0.08	-0.08	0.03	0.02	-0.05	-0.04	0.02	-0.01	-0.02	-0.04	0.09

0.76	-0.02	-0.02	-0.01	-0.04	0.05	-0.03	0.10	0.06	0.08	0.12	0.20
0.81	-0.18	-0.17	-0.03	0.01	0.05	0.04	0.00	0.16	0.10	0.04	0.11
0.86	-0.01	-0.13	-0.14	-0.02	-0.12	-0.24	-0.19	-0.22	-0.26	-0.34	-0.28
0.91	-0.07	0.16	-0.03	0.10	0.05	0.11	0.15	0.28	0.24	0.22	0.37
0.96	0.01	-0.13	0.04	0.09	0.07	0.08	0.12	0.10	0.06	-0.03	0.16
1.01	0.24	0.29	0.24	0.29	0.35	0.34	0.39	0.38	0.46	0.40	0.47
1.06	-0.22	-0.11	0.03	0.15	0.05	0.09	0.20	-0.02	-0.06	0.13	0.14
1.11	-6.46	-0.36	1.81	1.72	0.36	-1.18	-2.10	-1.99	-1.20	-0.57	-0.23
1.16	8.31	-23.74	-48.58	-67.30	-80.16	-87.19	-88.56	-85.33	-77.30	-64.83	-49.30
1.21	490.22	466.91	439.58	410.29	383.96	358.30	334.30	307.90	278.33	243.62	205.18
1.26	-141.95	-145.92	-135.20	-110.76	-74.61	-29.68	22.76	79.07	137.83	195.76	250.43
1.31	22.97	20.95	16.11	11.24	8.45	9.24	12.60	16.23	16.82	11.63	-1.20
1.36	6.73	8.04	8.64	8.19	6.22	3.65	0.92	-0.82	-0.85	0.65	3.23
1.41	1.80	-0.08	-0.47	0.95	3.27	6.15	8.69	10.24	10.41	9.59	8.09
1.46	8.72	9.30	8.29	6.21	3.76	2.00	1.73	2.30	4.10	6.32	8.08
1.51	1.61	2.71	4.72	6.84	8.35	8.75	7.96	6.34	4.23	2.24	0.75
1.56	4.59	2.03	0.38	0.15	1.13	3.08	5.19	6.78	7.41	6.82	5.41
1.61	7.20	7.12	5.84	3.93	1.80	0.35	-0.09	0.54	2.06	4.04	5.98
1.66	0.51	1.65	3.37	5.23	6.59	7.20	6.76	5.62	3.93	2.63	1.58
1.71	6.55	5.35	3.72	2.10	0.84	0.83	1.51	2.83	4.32	5.51	6.03
1.76	3.65	5.37	6.55	6.81	6.20	4.72	2.91	1.37	0.49	0.44	1.09
1.81	0.73	-0.04	0.18	1.23	2.57	3.88	4.70	4.83	4.29	3.25	2.10
1.86	4.42	3.30	2.01	1.05	0.64	0.89	1.85	3.06	4.21	5.05	5.32
1.91	1.37	2.86	4.33	5.35	5.60	4.95	3.64	2.28	1.05	0.37	0.28
1.96	3.02	1.54	0.66	0.58	1.03	2.08	3.19	4.14	4.37	4.15	3.36
2.01	4.43	5.13	4.95	3.91	2.40	0.92	-0.02	-0.28	0.40	1.69	3.21
2.06	0.36	0.68	1.51	2.71	3.70	4.45	4.49	4.04	3.17	2.22	1.39
2.11	3.19	2.22	1.15	0.90	1.07	1.63	2.45	3.24	3.67	3.78	3.44
2.16	2.91	3.93	4.46	4.19	3.55	2.42	1.36	0.72	0.39	0.69	1.49
2.21	1.04	0.44	0.76	1.55	2.73	3.71	4.20	4.42	3.83	2.99	2.11
2.26	4.33	4.11	3.16	2.23	1.30	0.79	0.89	1.54	2.43	3.27	4.00
2.31	1.14	1.94	2.61	3.23	3.50	3.21	2.60	1.88	1.14	0.75	0.63
2.36	1.20	1.17	1.18	1.63	2.18	2.56	2.59	2.62	2.25	1.86	1.44
2.41	3.65	3.67	2.96	2.06	1.17	0.50	0.49	0.93	1.64	2.49	3.28
2.46	0.73	0.94	1.62	2.44	3.08	3.43	3.32	2.76	2.07	1.30	0.86
2.51	4.17	3.18	2.09	1.21	0.52	0.70	1.11	2.00	2.93	3.61	3.82
2.56	2.37	2.83	3.04	2.94	2.39	1.90	1.33	1.04	1.13	1.38	1.87
2.61	0.93	1.25	1.90	2.54	2.85	3.08	2.72	2.28	1.78	1.21	1.14
2.66	2.98	2.51	1.79	1.24	0.95	1.24	1.57	2.17	2.81	3.08	3.24
2.71	1.79	2.37	2.88	2.98	2.73	2.29	1.73	1.21	0.85	0.98	1.31
2.76	1.80	1.29	1.16	1.31	1.74	2.31	2.88	3.10	3.08	2.77	2.34
2.81	2.72	2.59	2.26	1.73	1.20	0.81	0.97	1.24	1.54	2.00	2.52
2.86	1.17	1.70	2.03	2.31	2.14	1.97	1.57	1.08	0.90	0.74	0.93
2.91	2.24	1.62	1.16	0.99	1.08	1.32	1.96	2.28	2.67	2.66	2.45
2.96	2.49	2.78	2.72	2.32	1.92	1.51	1.14	1.05	1.17	1.54	1.99
3.01	1.40	1.14	1.20	1.54	2.01	2.40	2.53	2.45	2.38	1.89	1.50
3.06	2.54	2.16	1.56	1.18	0.94	0.88	1.06	1.36	1.74	2.05	2.14
3.11	1.83	2.10	2.20	2.15	1.94	1.56	1.34	1.13	0.96	1.09	1.39
3.16	1.33	1.04	0.87	1.23	1.54	1.96	2.24	2.29	2.27	1.95	1.61

3.21	2.37	2.21	1.83	1.59	1.41	1.41	1.49	1.74	1.95	2.13	2.26
3.26	1.60	1.79	2.03	2.28	2.39	2.23	2.05	1.84	1.51	1.52	1.46
3.31	2.03	1.67	1.45	1.52	1.66	1.89	2.12	2.40	2.46	2.45	2.22
3.36	2.61	2.56	2.40	2.17	1.86	1.60	1.53	1.63	1.69	2.02	2.19
3.41	1.36	1.49	1.81	2.08	2.29	2.35	2.18	2.02	1.66	1.46	1.39
3.46	2.01	1.63	1.41	1.22	1.28	1.45	1.68	2.03	2.28	2.41	2.34
3.51	1.84	2.20	2.39	2.35	2.24	2.01	1.64	1.47	1.43	1.43	1.53
3.56	1.49	1.31	1.32	1.54	1.89	2.14	2.19	2.11	1.98	1.79	1.66
3.61	2.00	1.84	1.60	1.39	1.40	1.39	1.45	1.57	1.70	1.82	1.81
3.66	1.30	1.54	1.81	2.03	2.05	1.87	1.63	1.30	1.18	1.06	1.19
3.71	2.25	1.87	1.67	1.72	1.78	1.92	2.18	2.50	2.46	2.43	2.36
3.76	2.29	2.26	2.18	2.02	1.76	1.76	1.69	1.81	1.82	1.95	2.21
3.81	1.31	1.50	1.65	1.76	1.84	1.88	1.67	1.54	1.38	1.22	1.19
3.86	1.51	1.35	1.11	1.11	1.10	1.19	1.28	1.43	1.53	1.51	1.36
3.91	1.75	1.91	1.92	2.01	1.79	1.65	1.47	1.35	1.25	1.36	1.46
3.96	1.43	1.40	1.49	1.64	1.80	1.98	1.88	1.96	1.83	1.60	1.46
4.01	2.35	2.25	1.86	1.75	1.67	1.61	1.65	1.97	2.08	2.31	2.45
4.06	1.78	1.87	1.96	1.89	1.91	1.79	1.79	1.66	1.52	1.49	1.68
4.11	1.27	1.25	1.18	1.13	1.27	1.54	1.62	1.56	1.52	1.41	1.27
4.16	2.12	2.07	1.95	1.67	1.69	1.46	1.46	1.48	1.66	1.89	2.04
4.21	1.46	1.48	1.59	1.80	1.83	1.99	1.90	1.83	1.70	1.57	1.55
4.26	1.84	1.61	1.48	1.44	1.42	1.38	1.61	1.76	1.76	1.90	1.93
4.31	1.65	1.76	1.75	1.58	1.56	1.42	1.18	1.30	1.21	1.26	1.36
4.36	1.41	1.45	1.58	1.59	1.59	1.58	1.48	1.40	1.32	1.18	1.19
4.41	1.56	1.40	1.26	1.17	1.01	1.12	1.12	1.30	1.46	1.51	1.56
4.46	1.99	2.07	2.01	2.11	1.96	1.86	1.82	1.65	1.58	1.69	1.72
4.51	1.85	1.64	1.49	1.62	1.65	1.64	1.72	1.79	1.75	1.69	1.60
4.56	1.78	1.73	1.54	1.49	1.27	1.33	1.43	1.31	1.39	1.55	1.52
4.61	1.29	1.32	1.27	1.33	1.28	1.08	0.89	0.92	0.90	0.87	0.93
4.66	1.51	1.38	1.36	1.34	1.34	1.52	1.55	1.77	1.63	1.70	1.67
4.71	1.97	1.99	1.88	1.85	1.76	1.66	1.69	1.64	1.62	1.85	1.84
4.76	1.76	1.83	1.88	1.94	2.04	2.03	1.90	1.88	1.81	1.78	1.75
4.81	1.75	1.46	1.44	1.36	1.27	1.39	1.34	1.37	1.49	1.54	1.50
4.86	1.48	1.44	1.43	1.33	1.29	1.20	1.06	1.14	1.01	0.95	1.02
4.91	1.65	1.54	1.63	1.65	1.63	1.77	1.76	1.71	1.68	1.69	1.60
4.96	1.74	1.77	1.64	1.45	1.40	1.39	1.38	1.45	1.41	1.56	1.66
5.01	1.63	1.65	1.76	1.78	1.70	1.79	1.58	1.53	1.44	1.41	1.52
5.06	1.64	1.67	1.51	1.51	1.46	1.47	1.44	1.47	1.47	1.43	1.39
5.11	1.60	1.54	1.54	1.35	1.30	1.31	1.24	1.35	1.29	1.33	1.44
5.16	1.28	1.41	1.45	1.43	1.39	1.43	1.46	1.40	1.32	1.28	1.29
5.21	1.40	1.21	1.22	1.30	1.20	1.28	1.24	1.26	1.23	1.18	1.16
5.26	1.73	1.78	1.84	1.86	1.74	1.69	1.68	1.61	1.55	1.61	1.67
5.31	1.50	1.46	1.34	1.44	1.51	1.47	1.45	1.44	1.32	1.30	1.38
5.38	1.29	1.33	1.29	1.37	1.29	1.36	1.29	1.14	1.18	1.06	0.98
5.45	1.47	1.46	1.60	1.60	1.61	1.64	1.61	1.48	1.49	1.40	1.49
5.53	1.61	1.39	1.49	1.53	1.60	1.60	1.52	1.53	1.39	1.33	1.25
5.61	1.82	1.64	1.69	1.50	1.46	1.54	1.45	1.47	1.42	1.61	1.55
5.70	1.50	1.44	1.44	1.54	1.41	1.40	1.39	1.36	1.24	1.19	1.12
5.80	1.61	1.61	1.50	1.50	1.32	1.39	1.34	1.31	1.27	1.27	1.27
5.90	1.55	1.43	1.45	1.43	1.34	1.40	1.34	1.40	1.35	1.51	1.36

6.01	1.73	1.74	1.78	1.65	1.63	1.65	1.73	1.62	1.69	1.69	1.73
6.13	1.42	1.50	1.41	1.50	1.34	1.33	1.48	1.43	1.32	1.41	1.36
6.25	1.15	1.26	1.14	1.16	1.24	1.14	1.12	1.25	1.17	1.23	1.36
6.39	1.31	1.19	1.20	1.22	1.27	1.18	1.29	1.34	1.35	1.41	1.49
6.53	1.38	1.34	1.26	1.23	1.19	1.20	1.27	1.35	1.35	1.53	1.67
6.69	1.57	1.42	1.46	1.40	1.50	1.54	1.53	1.58	1.69	1.63	1.81
6.85	1.23	1.24	1.22	1.26	1.23	1.24	1.27	1.28	1.38	1.39	1.43
7.03	1.35	1.30	1.28	1.24	1.19	1.27	1.30	1.35	1.42	1.42	1.44
7.21	1.42	1.28	1.24	1.35	1.40	1.45	1.37	1.45	1.51	1.71	1.69
7.41	1.39	1.38	1.41	1.37	1.42	1.36	1.44	1.45	1.58	1.68	1.75
7.63	1.64	1.60	1.51	1.59	1.60	1.66	1.64	1.69	1.69	1.70	1.75
7.86	1.30	1.36	1.28	1.30	1.37	1.32	1.44	1.49	1.50	1.54	1.65
8.10	1.35	1.27	1.13	1.21	1.25	1.24	1.26	1.21	1.25	1.33	1.39
8.36	0.91	0.97	0.92	0.93	1.07	1.00	0.97	1.07	1.04	1.12	1.31
8.64	1.38	1.26	1.20	1.10	1.31	1.23	1.33	1.30	1.38	1.48	1.67
8.94	1.39	1.37	1.36	1.32	1.35	1.39	1.55	1.51	1.53	1.67	1.71
9.26	1.47	1.43	1.44	1.48	1.52	1.47	1.59	1.57	1.69	1.74	1.79
9.60	1.25	1.22	1.21	1.23	1.29	1.22	1.28	1.37	1.35	1.40	1.51
9.97	1.36	1.40	1.23	1.32	1.36	1.35	1.29	1.43	1.40	1.40	1.52
10.36	1.46	1.32	1.34	1.32	1.34	1.40	1.49	1.45	1.62	1.63	1.77
10.77	1.28	1.43	1.36	1.33	1.40	1.33	1.48	1.54	1.42	1.51	1.63
11.22	1.24	1.20	1.19	1.30	1.27	1.38	1.34	1.48	1.56	1.57	1.69
11.70	1.28	1.26	1.21	1.22	1.37	1.38	1.35	1.46	1.51	1.63	1.67
12.21	1.18	1.15	1.22	1.14	1.10	1.25	1.29	1.29	1.27	1.29	1.41
12.75	1.33	1.29	1.25	1.30	1.33	1.35	1.36	1.43	1.42	1.46	1.62
13.33	1.35	1.41	1.45	1.51	1.45	1.53	1.56	1.65	1.60	1.67	1.72
13.95	1.34	1.18	1.26	1.34	1.30	1.41	1.38	1.38	1.43	1.56	1.58
14.62	1.13	1.17	1.06	1.10	1.12	1.27	1.19	1.21	1.34	1.28	1.37
15.33	0.88	0.91	0.84	0.86	0.86	0.93	0.98	1.03	1.01	1.13	1.27
16.09	1.20	1.05	1.05	1.04	1.07	1.11	1.03	1.14	1.15	1.21	1.31
16.90	1.16	1.12	1.16	1.15	1.19	1.20	1.13	1.21	1.16	1.32	1.38
17.77	1.09	1.11	1.12	1.26	1.19	1.23	1.33	1.36	1.32	1.31	1.54
18.70	0.84	0.91	0.87	0.89	0.84	1.01	1.01	1.07	1.16	1.24	1.26
19.69	1.09	1.17	1.09	1.20	1.17	1.17	1.20	1.25	1.26	1.27	1.33
20.75	0.90	0.88	0.96	1.03	1.00	1.00	0.93	1.10	1.13	1.14	1.17
21.89	1.25	1.01	1.06	1.20	1.32	1.27	1.36	1.45	1.45	1.55	1.63
23.10	1.35	1.22	1.38	1.41	1.33	1.50	1.48	1.61	1.66	1.75	1.82
24.39	1.00	1.06	1.15	1.06	1.13	1.17	1.28	1.30	1.40	1.37	1.42
25.78	1.14	1.10	1.06	1.13	1.17	1.24	1.27	1.31	1.28	1.30	1.38
27.26	0.92	0.86	0.98	1.08	0.97	1.09	1.15	1.17	1.28	1.30	1.36
28.84	1.20	1.11	1.14	1.18	1.18	1.27	1.29	1.34	1.41	1.46	1.41
30.53	1.12	1.12	1.04	1.06	1.17	1.18	1.13	1.25	1.30	1.30	1.53
32.34	0.88	0.98	0.94	0.99	1.01	1.02	1.04	1.07	1.10	1.08	1.24
34.27	1.16	1.11	1.05	1.16	1.27	1.27	1.29	1.33	1.40	1.41	1.47
36.34	0.73	0.78	0.80	0.73	0.77	0.78	0.76	1.04	1.04	1.09	1.20
38.55	1.14	1.25	1.28	1.31	1.34	1.31	1.41	1.47	1.47	1.52	1.60
40.91	0.84	0.95	0.90	1.00	1.05	1.01	1.04	1.14	1.26	1.30	1.38
43.43	0.86	0.92	0.97	0.92	0.89	1.06	1.00	1.04	1.16	1.07	1.28
46.13	0.74	0.74	0.75	0.83	0.87	0.83	0.80	0.99	1.01	1.16	1.20
49.01	0.87	0.88	0.95	0.89	1.02	1.00	0.96	1.05	1.18	1.23	1.28

52.09	0.64	0.81	0.62	0.79	0.82	0.77	0.80	0.94	0.89	0.87	0.99
55.39	0.70	0.69	0.76	0.75	0.84	0.83	0.86	0.77	0.92	0.98	1.03
58.91	0.57	0.60	0.68	0.78	0.69	0.72	0.70	0.72	0.69	0.80	0.95
62.68	0.61	0.65	0.55	0.57	0.62	0.59	0.51	0.61	0.55	0.66	0.75
66.70	0.56	0.53	0.49	0.63	0.66	0.69	0.56	0.75	0.74	0.82	0.97
71.00	0.61	0.48	0.54	0.51	0.51	0.51	0.58	0.54	0.67	0.66	0.82
75.60	0.33	0.33	0.34	0.26	0.40	0.36	0.42	0.47	0.40	0.50	0.60
80.51	0.78	0.83	0.68	0.80	0.77	0.86	0.79	0.82	0.86	0.93	1.04
85.77	0.56	0.53	0.46	0.52	0.42	0.47	0.35	0.36	0.43	0.44	0.50
91.38	0.61	0.63	0.56	0.57	0.60	0.64	0.61	0.64	0.56	0.70	0.87
97.39	0.28	0.46	0.32	0.34	0.24	0.30	0.41	0.44	0.37	0.40	0.48
103.80	0.51	0.48	0.41	0.54	0.46	0.44	0.49	0.48	0.42	0.53	0.49
110.66	0.29	0.32	0.25	0.30	0.24	0.24	0.26	0.31	0.42	0.43	0.65
117.99	0.05	0.01	-0.04	-0.04	0.01	-0.15	-0.09	-0.04	-0.02	0.10	0.17
125.83	0.33	0.45	0.35	0.47	0.33	0.19	0.34	0.39	0.35	0.46	0.59
134.21	0.16	0.17	0.14	0.20	0.10	0.03	0.03	0.06	0.02	0.12	0.20
143.17	0.04	0.04	0.03	-0.01	-0.04	-0.17	-0.07	-0.12	0.06	0.09	0.09
152.74	0.19	0.14	0.11	0.17	0.16	0.21	0.17	0.14	0.14	0.34	0.48
162.97	0.22	0.40	0.41	0.40	0.46	0.53	0.64	0.56	0.71	0.69	0.75
173.91	0.10	0.06	0.03	0.16	0.25	0.24	0.26	0.30	0.36	0.30	0.43
185.60	0.26	0.35	0.32	0.46	0.50	0.58	0.61	0.66	0.62	0.64	0.68
198.10	0.08	0.05	0.20	0.28	0.28	0.23	0.33	0.33	0.30	0.30	0.36
211.46	0.09	0.09	0.13	0.27	0.31	0.33	0.42	0.51	0.62	0.70	0.75
225.75	0.04	0.12	0.15	0.18	0.31	0.27	0.34	0.48	0.50	0.53	0.56
241.01	0.07	0.24	0.13	0.12	0.26	0.31	0.34	0.40	0.34	0.38	0.44
257.33	-0.23	-0.09	-0.08	0.00	0.10	0.10	0.21	0.14	0.23	0.18	0.29
274.77	0.07	0.05	-0.04	0.10	0.17	0.20	0.23	0.27	0.34	0.34	0.47
293.42	-0.11	-0.08	-0.09	0.09	0.18	0.25	0.34	0.36	0.47	0.36	0.36
313.35	-0.24	-0.18	-0.20	-0.03	0.00	0.02	0.20	0.18	0.15	0.23	0.34
334.66	-0.04	-0.03	0.10	0.13	0.18	0.18	0.34	0.33	0.47	0.53	0.55
357.43	-0.14	-0.23	-0.16	-0.01	-0.02	0.05	0.04	0.14	0.09	0.08	0.24
381.78	-0.19	-0.23	-0.10	-0.09	-0.02	0.12	0.06	0.31	0.25	0.30	0.41
407.80	-0.27	-0.28	-0.20	-0.11	-0.01	0.06	0.21	0.14	0.29	0.22	0.28
435.62	-0.34	-0.31	-0.17	-0.02	-0.05	0.12	0.11	0.16	0.23	0.26	0.32
465.35	-0.42	-0.21	-0.35	-0.21	-0.24	-0.06	0.01	0.12	0.03	0.21	0.22
497.14	-0.41	-0.30	-0.35	-0.32	-0.14	-0.22	-0.14	-0.09	-0.15	-0.14	0.00
531.11	-0.54	-0.49	-0.43	-0.29	-0.26	-0.19	-0.18	-0.21	-0.08	-0.18	-0.13
567.43	-0.35	-0.26	-0.30	-0.13	-0.10	-0.02	-0.07	0.07	0.06	0.17	0.19
606.26	-0.32	-0.27	-0.32	-0.19	-0.13	-0.17	0.00	-0.08	-0.02	-0.03	0.05
647.76	-0.47	-0.41	-0.35	-0.34	-0.20	-0.13	-0.09	0.06	0.05	0.06	0.03
692.12	-0.84	-0.72	-0.63	-0.63	-0.53	-0.49	-0.39	-0.36	-0.24	-0.24	-0.16
739.53	-0.56	-0.45	-0.46	-0.34	-0.29	-0.25	-0.08	0.07	0.00	0.15	0.16
790.22	-0.62	-0.58	-0.66	-0.55	-0.53	-0.50	-0.38	-0.38	-0.37	-0.34	-0.37
844.40	-0.73	-0.60	-0.69	-0.62	-0.57	-0.43	-0.38	-0.42	-0.40	-0.34	-0.19
902.32	-0.65	-0.66	-0.54	-0.47	-0.45	-0.31	-0.23	-0.12	-0.08	-0.02	0.00
964.23	-0.67	-0.69	-0.55	-0.67	-0.49	-0.42	-0.38	-0.37	-0.43	-0.40	-0.32
1030.41	-0.69	-0.61	-0.56	-0.43	-0.30	-0.22	-0.09	0.01	0.11	0.18	0.31
1101.15	-0.92	-0.88	-0.79	-0.69	-0.51	-0.48	-0.43	-0.31	-0.18	-0.19	-0.14
1176.77	-0.66	-0.68	-0.66	-0.49	-0.29	-0.24	-0.17	0.06	0.04	0.10	0.29
1257.60	-0.72	-0.67	-0.57	-0.53	-0.45	-0.41	-0.17	-0.07	0.01	0.00	0.06

1344.00	-0.88	-0.84	-0.87	-0.65	-0.65	-0.49	-0.44	-0.41	-0.20	-0.31	-0.20
1436.36	-1.04	-0.98	-0.96	-0.84	-0.71	-0.61	-0.50	-0.29	-0.23	-0.18	-0.06
1535.09	-0.80	-0.82	-0.73	-0.64	-0.49	-0.43	-0.35	-0.11	-0.22	-0.05	-0.06
1640.63	-1.08	-0.98	-0.92	-0.83	-0.76	-0.76	-0.51	-0.54	-0.40	-0.42	-0.36
1753.44	-1.14	-1.08	-0.97	-0.96	-0.75	-0.67	-0.61	-0.42	-0.28	-0.21	-0.17
1874.02	-0.97	-0.96	-0.88	-0.86	-0.78	-0.58	-0.54	-0.44	-0.33	-0.36	-0.33
2002.92	-1.15	-1.04	-1.08	-0.99	-0.67	-0.71	-0.54	-0.38	-0.28	-0.18	-0.16
2140.71	-1.25	-1.15	-1.13	-0.94	-0.99	-0.80	-0.70	-0.57	-0.55	-0.44	-0.40
2288.00	-1.04	-1.10	-1.01	-0.94	-0.76	-0.67	-0.53	-0.48	-0.38	-0.36	-0.30
2445.44	-1.21	-1.19	-1.07	-0.99	-0.91	-0.77	-0.71	-0.64	-0.56	-0.56	-0.46
2613.73	-1.18	-1.09	-1.11	-0.94	-0.83	-0.72	-0.64	-0.43	-0.44	-0.31	-0.29
2793.63	-1.06	-0.98	-0.89	-0.69	-0.58	-0.45	-0.33	-0.19	-0.08	-0.11	-0.03
2985.93	-0.93	-0.73	-0.70	-0.63	-0.49	-0.29	-0.25	-0.16	-0.16	-0.24	-0.20
3191.49	-0.88	-0.83	-0.68	-0.67	-0.58	-0.43	-0.27	-0.19	-0.17	-0.05	-0.15
3411.21	-0.78	-0.71	-0.64	-0.45	-0.28	-0.17	-0.07	0.05	0.10	-0.04	0.06
3646.09	-0.73	-0.70	-0.53	-0.41	-0.39	-0.18	-0.07	0.00	0.02	-0.09	0.02
3897.16	-0.56	-0.57	-0.53	-0.29	-0.21	-0.08	0.10	0.16	0.10	0.18	0.09

Wavelength (nm) / Time (ps)	490.74	492.16	493.58	495.01	496.43	497.86	499.28	500.70	502.13	503.55	504.98
-2.74	-0.46	-0.54	-0.49	-0.58	-0.57	-0.58	-0.49	-0.49	-0.41	-0.42	-0.38
-2.24	-0.08	-0.05	-0.06	-0.08	-0.15	-0.06	-0.06	-0.05	-0.07	-0.03	-0.08
-1.74	-0.14	-0.26	-0.23	-0.30	-0.35	-0.28	-0.28	-0.26	-0.36	-0.25	-0.32
-1.24	0.05	0.22	0.19	0.14	0.17	0.16	0.21	0.09	0.15	0.01	0.01
-0.74	0.19	0.24	0.22	0.28	0.38	0.26	0.22	0.32	0.27	0.26	0.31
-0.24	0.17	0.14	0.08	0.19	0.08	0.06	-0.03	0.10	0.12	0.09	0.05
-0.14	0.27	0.29	0.28	0.35	0.32	0.29	0.31	0.23	0.19	0.23	0.30
-0.04	0.01	-0.05	0.00	0.00	0.11	0.13	0.12	0.07	0.10	0.11	0.09
0.06	0.04	-0.06	0.08	0.06	0.02	0.12	0.15	0.15	0.08	0.05	0.04
0.16	0.36	0.44	0.53	0.43	0.38	0.47	0.48	0.49	0.38	0.40	0.38
0.26	0.26	0.30	0.35	0.42	0.44	0.23	0.26	0.32	0.25	0.11	0.19
0.31	0.39	0.35	0.41	0.44	0.43	0.48	0.33	0.36	0.23	0.19	0.40
0.36	-0.16	-0.11	-0.19	-0.10	-0.05	-0.11	-0.06	-0.01	-0.04	-0.11	-0.05
0.41	0.05	0.07	0.18	0.19	0.26	0.29	0.28	0.34	0.17	0.16	0.18
0.46	0.26	0.22	0.19	0.28	0.18	0.29	0.27	0.24	0.28	0.12	0.23
0.51	0.15	0.16	0.17	0.11	0.17	0.02	0.06	0.08	0.01	0.08	0.02
0.56	0.27	0.29	0.33	0.30	0.31	0.27	0.36	0.28	0.34	0.27	0.29
0.61	-0.08	-0.13	0.06	0.06	0.15	0.10	0.18	0.07	0.03	-0.09	0.00
0.66	0.09	0.12	0.11	0.25	0.23	0.31	0.27	0.33	0.17	0.16	0.21
0.71	0.05	0.10	0.15	0.22	0.24	0.11	0.15	0.26	0.24	0.08	0.05
0.76	0.16	0.14	0.33	0.30	0.28	0.17	0.25	0.33	0.25	0.26	0.25
0.81	0.32	0.31	0.29	0.30	0.28	0.33	0.35	0.30	0.30	0.24	0.26
0.86	-0.15	-0.18	-0.18	-0.07	0.04	-0.12	0.03	-0.02	-0.10	-0.11	-0.20
0.91	0.43	0.42	0.61	0.51	0.46	0.56	0.51	0.56	0.42	0.43	0.50
0.96	0.08	0.22	0.16	0.24	0.14	0.25	0.26	0.26	0.19	0.23	0.15
1.01	0.59	0.38	0.60	0.57	0.49	0.50	0.45	0.45	0.32	0.38	0.31
1.06	0.09	0.08	0.29	0.23	0.13	0.28	0.27	0.14	0.23	0.20	0.11
1.11	-0.11	-0.24	-0.51	-0.60	-0.30	-0.08	-0.06	-0.12	-0.10	-0.09	-0.19
1.16	-32.25	-16.25	-3.73	3.90	6.57	5.48	2.51	-0.54	-2.67	-3.37	-2.71

1.21	165.76	126.63	89.95	57.36	28.75	3.15	-19.40	-39.00	-55.59	-69.14	-78.42
1.26	298.95	337.91	367.24	386.73	398.12	401.56	397.93	388.39	374.13	356.46	337.02
1.31	-21.48	-47.33	-75.04	-100.56	-120.68	-132.96	-136.97	-132.28	-119.75	-100.37	-74.50
1.36	6.64	10.28	13.71	16.93	19.38	20.72	20.58	18.36	14.66	10.42	7.13
1.41	6.45	4.90	4.25	4.55	4.99	5.90	6.80	7.23	6.99	6.15	5.14
1.46	9.04	8.84	7.65	5.70	3.52	1.56	0.29	-0.03	0.61	2.14	4.29
1.51	0.23	0.85	2.07	3.84	5.47	6.79	7.53	7.52	6.89	6.02	5.24
1.56	3.67	2.01	1.19	1.14	2.03	3.58	5.30	7.05	8.36	8.92	8.90
1.61	7.33	7.76	7.23	6.13	4.71	3.30	2.14	1.67	1.79	2.49	3.71
1.66	1.58	2.10	3.21	4.46	5.67	6.34	6.52	6.20	5.41	4.29	3.48
1.71	5.81	4.89	3.67	2.26	1.17	0.76	1.00	1.72	3.00	4.49	5.88
1.76	2.43	3.97	5.23	6.03	6.31	5.83	5.15	4.18	3.38	2.81	2.87
1.81	1.33	0.89	1.18	2.04	3.00	4.12	5.14	5.65	5.77	5.34	4.69
1.86	5.00	4.03	3.11	2.03	1.39	1.07	1.29	1.91	2.78	3.87	4.92
1.91	1.00	2.00	3.15	4.15	4.72	4.76	4.54	3.94	3.16	2.67	2.49
1.96	2.37	1.42	0.96	0.87	1.43	2.17	3.24	4.39	5.25	5.71	5.96
2.01	4.57	5.27	5.59	5.04	4.15	3.04	2.08	1.42	1.31	1.73	2.61
2.06	0.96	1.01	1.51	2.41	3.22	4.02	4.47	4.57	4.45	4.04	3.61
2.11	2.78	1.92	1.28	1.02	0.97	1.38	2.20	3.00	3.93	4.64	5.15
2.16	2.65	3.62	4.45	4.70	4.66	4.19	3.72	3.14	2.75	2.54	2.76
2.21	1.42	1.07	1.21	1.75	2.58	3.38	4.25	4.68	4.85	4.63	4.27
2.26	4.28	4.04	3.61	2.88	2.04	1.71	1.58	1.83	2.36	3.14	4.06
2.31	1.03	1.50	2.26	2.96	3.50	3.77	3.72	3.59	3.23	2.96	2.85
2.36	1.27	1.22	1.47	1.81	2.38	2.73	3.23	3.50	3.73	3.70	3.62
2.41	3.90	3.80	3.53	3.08	2.40	1.85	1.59	1.73	2.00	2.63	3.46
2.46	0.62	0.85	1.33	1.90	2.63	3.31	3.62	3.67	3.52	3.24	3.03
2.51	3.74	3.10	2.44	1.93	1.41	1.35	1.68	2.43	3.25	4.00	4.86
2.56	2.53	3.08	3.56	3.55	3.47	3.33	3.01	2.78	2.77	2.74	3.04
2.61	1.17	1.37	1.96	2.58	3.09	3.42	3.45	3.50	3.31	3.11	2.95
2.66	3.05	2.66	2.22	1.96	1.71	1.86	2.15	2.70	3.28	3.95	4.49
2.71	1.94	2.56	3.13	3.42	3.61	3.54	3.41	3.02	2.80	2.74	2.84
2.76	2.02	1.56	1.51	1.60	1.97	2.49	3.05	3.50	3.84	4.11	4.11
2.81	2.71	2.71	2.55	2.26	1.97	1.85	1.81	2.01	2.31	2.80	3.38
2.86	1.12	1.63	2.07	2.41	2.77	2.75	2.73	2.58	2.55	2.50	2.55
2.91	2.18	1.91	1.67	1.54	1.71	1.99	2.41	2.93	3.43	3.92	4.18
2.96	2.40	2.60	2.85	2.83	2.72	2.46	2.38	2.33	2.31	2.54	2.96
3.01	1.35	1.09	1.25	1.49	1.89	2.39	2.93	3.30	3.47	3.54	3.59
3.06	2.14	1.97	1.79	1.54	1.43	1.33	1.64	1.94	2.46	2.79	3.29
3.11	1.69	1.98	2.19	2.46	2.48	2.40	2.42	2.35	2.34	2.45	2.71
3.16	1.32	1.28	1.32	1.53	1.91	2.36	2.77	3.13	3.49	3.53	3.52
3.21	2.38	2.37	2.35	2.16	2.11	2.10	2.34	2.55	2.88	3.16	3.66
3.26	1.61	1.84	2.20	2.60	2.83	2.88	3.03	3.14	3.08	3.01	3.20
3.31	2.13	1.99	1.96	2.13	2.19	2.61	2.82	3.18	3.58	3.77	3.97
3.36	2.52	2.72	2.63	2.69	2.53	2.52	2.51	2.54	2.68	3.07	3.45
3.41	1.49	1.66	1.89	2.30	2.62	2.99	3.19	3.21	3.21	3.25	3.30
3.46	2.28	2.09	1.89	1.91	1.97	2.10	2.54	2.86	3.30	3.59	3.86
3.51	1.88	2.07	2.43	2.65	2.65	2.75	2.72	2.69	2.72	2.86	3.15
3.56	1.35	1.48	1.54	1.90	2.16	2.46	2.89	3.24	3.27	3.44	3.56
3.61	1.84	1.79	1.67	1.68	1.73	1.81	2.08	2.20	2.53	2.79	3.22

3.66	1.42	1.67	2.02	2.22	2.42	2.50	2.70	2.63	2.55	2.60	2.80
3.71	2.25	1.96	1.99	2.05	2.11	2.39	2.68	3.03	3.25	3.57	3.81
3.76	2.31	2.35	2.44	2.51	2.62	2.68	2.75	2.94	3.17	3.47	3.72
3.81	1.31	1.44	1.71	1.98	2.29	2.40	2.55	2.74	2.70	2.84	2.86
3.86	1.36	1.22	1.24	1.24	1.37	1.53	1.77	2.02	2.44	2.67	2.93
3.91	1.74	1.78	2.10	2.32	2.34	2.33	2.42	2.55	2.63	2.82	3.03
3.96	1.52	1.44	1.58	1.82	2.01	2.30	2.67	2.85	3.05	3.16	3.23
4.01	2.56	2.41	2.46	2.47	2.29	2.43	2.63	2.90	3.22	3.46	3.88
4.06	1.81	1.97	2.22	2.32	2.50	2.60	2.68	2.86	2.99	3.08	3.30
4.11	1.28	1.18	1.31	1.52	1.76	2.04	2.31	2.58	2.87	2.96	3.12
4.16	2.14	2.13	2.29	2.39	2.34	2.37	2.46	2.62	2.91	3.16	3.54
4.21	1.62	1.69	1.98	2.19	2.43	2.51	2.78	2.94	3.09	3.12	3.41
4.26	1.93	1.83	1.88	1.82	2.00	2.25	2.41	2.72	2.98	3.36	3.62
4.31	1.50	1.64	1.77	1.88	1.92	2.05	2.08	2.16	2.44	2.47	2.80
4.36	1.32	1.51	1.66	1.92	2.06	2.27	2.54	2.50	2.65	2.75	2.95
4.41	1.56	1.52	1.51	1.71	1.69	1.85	2.07	2.22	2.57	2.90	3.20
4.46	1.93	2.15	2.31	2.50	2.66	2.71	2.75	2.91	3.01	3.17	3.40
4.51	1.56	1.42	1.58	1.62	1.84	2.13	2.34	2.62	2.86	3.05	3.36
4.56	1.60	1.66	1.79	1.79	1.87	1.99	2.20	2.40	2.64	2.83	3.11
4.61	1.12	1.17	1.34	1.47	1.61	1.74	1.87	2.05	2.17	2.28	2.58
4.66	1.59	1.62	1.68	1.75	1.96	2.24	2.46	2.77	2.95	3.20	3.50
4.71	2.01	2.17	2.34	2.39	2.41	2.51	2.62	2.81	2.97	3.13	3.34
4.76	1.74	1.89	2.09	2.32	2.52	2.68	2.86	3.13	3.20	3.27	3.48
4.81	1.61	1.50	1.54	1.70	1.72	1.97	2.17	2.46	2.69	2.93	3.10
4.86	1.16	1.15	1.40	1.48	1.67	1.77	1.99	2.14	2.27	2.45	2.80
4.91	1.63	1.69	1.94	2.09	2.36	2.52	2.73	2.98	3.09	3.23	3.54
4.96	1.75	1.72	1.82	1.84	1.97	2.05	2.23	2.38	2.67	2.87	3.20
5.01	1.66	1.75	1.85	2.04	2.16	2.40	2.54	2.63	2.85	3.05	3.21
5.06	1.39	1.37	1.50	1.69	1.75	1.94	2.21	2.44	2.62	2.86	3.07
5.11	1.58	1.61	1.80	1.84	1.96	2.05	2.20	2.39	2.53	2.71	3.02
5.16	1.27	1.44	1.63	1.74	1.93	2.09	2.27	2.52	2.54	2.84	3.05
5.21	1.07	1.16	1.17	1.38	1.48	1.56	1.81	1.98	2.22	2.49	2.77
5.26	1.73	1.77	1.93	2.08	2.16	2.35	2.51	2.64	2.83	3.10	3.30
5.31	1.43	1.39	1.56	1.79	2.00	2.16	2.40	2.55	2.64	2.79	3.11
5.38	1.12	1.27	1.32	1.45	1.64	1.79	1.96	2.20	2.43	2.55	2.74
5.45	1.51	1.69	1.80	2.06	2.19	2.37	2.52	2.64	2.98	3.03	3.27
5.53	1.26	1.23	1.38	1.53	1.63	1.79	2.04	2.15	2.48	2.71	2.94
5.61	1.59	1.58	1.71	1.82	1.94	2.10	2.26	2.56	2.82	2.98	3.36
5.70	1.17	1.27	1.37	1.57	1.75	2.02	2.25	2.43	2.54	2.81	3.08
5.80	1.28	1.37	1.42	1.58	1.63	1.92	2.12	2.32	2.49	2.66	3.06
5.90	1.51	1.53	1.66	1.73	1.91	2.01	2.18	2.44	2.67	2.84	3.16
6.01	1.86	1.81	1.85	1.94	2.14	2.24	2.54	2.69	2.89	3.12	3.42
6.13	1.45	1.56	1.66	1.84	2.00	2.09	2.32	2.56	2.69	2.91	3.29
6.25	1.39	1.45	1.67	1.81	2.05	2.19	2.38	2.51	2.71	2.92	3.19
6.39	1.69	1.78	2.02	2.14	2.23	2.29	2.59	2.80	2.87	3.09	3.38
6.53	1.72	1.73	1.90	2.05	2.20	2.30	2.57	2.76	2.99	3.10	3.39
6.69	1.94	1.92	2.25	2.35	2.42	2.56	2.78	2.99	3.12	3.35	3.61
6.85	1.62	1.61	1.77	1.99	2.04	2.31	2.44	2.59	2.90	3.09	3.33
7.03	1.56	1.64	1.77	1.92	2.06	2.27	2.49	2.74	2.78	3.00	3.42
7.21	1.78	1.87	2.01	2.21	2.26	2.43	2.69	2.84	3.09	3.23	3.52

7.41	1.89	1.89	2.10	2.23	2.43	2.44	2.73	2.99	3.09	3.32	3.64
7.63	1.82	1.85	2.06	2.09	2.25	2.43	2.63	2.81	2.99	3.26	3.59
7.86	1.80	1.83	1.92	2.11	2.26	2.45	2.58	2.82	3.02	3.24	3.49
8.10	1.52	1.61	1.80	1.90	2.15	2.21	2.50	2.55	2.84	3.00	3.35
8.36	1.38	1.44	1.61	1.65	1.89	2.03	2.06	2.39	2.53	2.79	3.04
8.64	1.73	1.79	1.97	2.21	2.16	2.44	2.65	2.82	3.12	3.21	3.60
8.94	1.95	1.92	2.10	2.18	2.36	2.63	2.77	2.96	3.27	3.43	3.71
9.26	2.04	2.07	2.19	2.46	2.56	2.82	3.06	3.21	3.47	3.72	3.97
9.60	1.61	1.64	1.85	2.05	2.11	2.32	2.59	2.68	2.97	3.22	3.52
9.97	1.58	1.71	1.81	1.99	2.19	2.30	2.56	2.72	2.92	3.09	3.41
10.36	1.81	1.95	2.10	2.18	2.41	2.53	2.78	3.03	3.25	3.47	3.80
10.77	1.79	1.89	2.03	2.11	2.23	2.40	2.74	2.88	3.13	3.33	3.64
11.22	1.71	1.88	2.01	2.11	2.22	2.47	2.62	2.88	3.04	3.40	3.63
11.70	1.96	1.98	2.06	2.30	2.43	2.54	2.85	3.16	3.19	3.44	3.84
12.21	1.42	1.45	1.68	1.86	2.00	2.12	2.37	2.65	2.76	3.08	3.29
12.75	1.67	1.75	1.92	2.06	2.17	2.48	2.53	2.79	3.08	3.28	3.46
13.33	1.80	1.85	2.10	2.23	2.28	2.42	2.83	2.88	3.19	3.38	3.61
13.95	1.67	1.78	1.82	2.04	2.18	2.35	2.56	2.81	3.03	3.28	3.55
14.62	1.58	1.60	1.65	1.88	1.95	2.24	2.45	2.58	2.91	3.05	3.35
15.33	1.39	1.37	1.68	1.79	1.96	2.18	2.34	2.56	2.72	2.96	3.19
16.09	1.52	1.41	1.56	1.85	1.93	2.14	2.40	2.58	2.77	3.02	3.31
16.90	1.45	1.55	1.57	1.77	1.93	2.13	2.30	2.64	2.72	3.04	3.23
17.77	1.64	1.66	1.79	1.95	2.00	2.27	2.48	2.64	2.88	3.11	3.47
18.70	1.47	1.65	1.74	1.93	1.95	2.14	2.44	2.68	2.91	3.12	3.40
19.69	1.41	1.44	1.72	1.80	1.98	2.11	2.36	2.62	2.77	3.07	3.36
20.75	1.24	1.31	1.46	1.62	1.84	2.02	2.22	2.51	2.60	2.95	3.20
21.89	1.69	1.87	2.03	2.19	2.33	2.51	2.75	2.93	3.15	3.34	3.55
23.10	1.83	1.98	2.18	2.33	2.48	2.55	2.78	3.06	3.38	3.58	3.92
24.39	1.67	1.60	1.83	1.91	2.18	2.31	2.59	2.75	2.85	3.24	3.49
25.78	1.56	1.63	1.74	1.86	2.08	2.24	2.48	2.66	2.88	3.09	3.32
27.26	1.54	1.67	1.81	1.95	2.14	2.27	2.59	2.71	2.89	3.14	3.47
28.84	1.60	1.75	1.79	2.01	2.14	2.23	2.50	2.66	2.81	3.14	3.29
30.53	1.67	1.76	1.98	2.13	2.23	2.48	2.65	2.83	3.11	3.30	3.68
32.34	1.33	1.41	1.58	1.81	1.95	2.07	2.34	2.46	2.66	2.95	3.30
34.27	1.58	1.62	1.89	1.89	2.09	2.21	2.46	2.71	2.95	3.24	3.48
36.34	1.35	1.45	1.72	1.91	2.05	2.18	2.46	2.55	2.87	3.02	3.40
38.55	1.67	1.74	1.92	1.99	2.21	2.34	2.57	2.67	2.93	3.14	3.47
40.91	1.50	1.62	1.66	1.92	2.10	2.25	2.49	2.67	2.85	3.12	3.36
43.43	1.31	1.43	1.57	1.72	1.85	2.04	2.25	2.47	2.58	2.82	3.09
46.13	1.35	1.47	1.66	1.79	1.95	2.21	2.50	2.59	2.86	3.04	3.40
49.01	1.42	1.55	1.70	1.87	2.02	2.23	2.38	2.61	2.88	3.11	3.33
52.09	1.18	1.16	1.40	1.45	1.64	1.90	2.09	2.22	2.50	2.75	3.03
55.39	1.04	1.21	1.29	1.43	1.70	1.79	2.04	2.35	2.56	2.77	3.14
58.91	1.00	1.09	1.33	1.45	1.56	1.82	2.04	2.22	2.49	2.75	2.99
62.68	0.81	0.88	1.02	1.20	1.37	1.52	1.77	2.05	2.30	2.46	2.80
66.70	1.06	1.19	1.35	1.56	1.73	1.86	2.16	2.33	2.57	2.85	3.12
71.00	0.88	0.97	1.18	1.46	1.50	1.71	1.79	2.08	2.29	2.48	2.78
75.60	0.74	0.89	1.05	1.20	1.42	1.69	1.98	2.03	2.35	2.58	2.80
80.51	1.18	1.21	1.45	1.65	1.84	1.96	2.20	2.38	2.49	2.77	3.05
85.77	0.54	0.60	0.90	1.00	1.13	1.30	1.62	1.83	1.99	2.23	2.60

91.38	1.00	1.06	1.37	1.50	1.69	1.83	2.19	2.35	2.65	2.78	3.15
97.39	0.56	0.69	0.85	1.00	1.10	1.27	1.62	1.76	2.04	2.19	2.65
103.80	0.81	0.80	1.16	1.28	1.53	1.69	2.07	2.27	2.53	2.68	3.06
110.66	0.84	0.97	1.15	1.40	1.56	1.69	2.10	2.21	2.51	2.67	3.02
117.99	0.20	0.43	0.58	0.75	1.04	1.22	1.50	1.87	1.96	2.23	2.56
125.83	0.84	0.91	1.24	1.42	1.67	1.86	2.13	2.32	2.66	2.86	3.13
134.21	0.38	0.52	0.88	0.99	1.25	1.38	1.71	2.05	2.22	2.43	2.78
143.17	0.47	0.55	0.63	0.98	1.15	1.29	1.59	1.87	2.16	2.29	2.64
152.74	0.60	0.73	0.99	1.18	1.38	1.58	1.92	2.17	2.38	2.58	2.85
162.97	0.80	0.93	1.12	1.31	1.37	1.59	1.88	2.06	2.25	2.59	2.86
173.91	0.52	0.54	0.70	0.94	1.13	1.39	1.50	1.82	2.07	2.37	2.64
185.60	0.87	0.76	1.02	1.27	1.26	1.43	1.87	2.12	2.28	2.48	2.85
198.10	0.50	0.57	0.75	0.91	1.07	1.28	1.57	1.76	2.01	2.33	2.53
211.46	0.91	0.99	1.13	1.27	1.50	1.75	1.82	2.16	2.34	2.55	2.94
225.75	0.73	0.78	0.93	1.17	1.22	1.47	1.71	1.91	2.20	2.42	2.69
241.01	0.64	0.64	0.91	1.08	1.25	1.47	1.61	1.81	2.14	2.31	2.68
257.33	0.45	0.47	0.68	0.87	1.08	1.33	1.56	1.81	1.97	2.24	2.54
274.77	0.62	0.67	0.89	1.01	1.19	1.47	1.63	2.07	2.21	2.47	2.80
293.42	0.65	0.64	0.85	1.02	1.11	1.30	1.65	1.83	2.07	2.32	2.62
313.35	0.43	0.51	0.68	0.89	1.06	1.22	1.42	1.82	1.95	2.16	2.55
334.66	0.63	0.69	0.84	1.05	1.29	1.43	1.70	1.84	2.14	2.39	2.75
357.43	0.27	0.34	0.55	0.71	0.93	1.05	1.39	1.67	1.83	2.09	2.43
381.78	0.60	0.71	0.90	1.08	1.16	1.41	1.70	1.90	2.17	2.49	2.75
407.80	0.36	0.51	0.63	0.86	0.95	1.17	1.39	1.70	1.93	2.16	2.52
435.62	0.44	0.42	0.64	0.90	1.00	1.27	1.48	1.72	2.03	2.28	2.50
465.35	0.33	0.35	0.54	0.79	0.99	1.17	1.50	1.69	1.99	2.23	2.60
497.14	0.00	0.20	0.34	0.48	0.58	0.90	1.11	1.42	1.72	1.97	2.24
531.11	-0.02	0.09	0.21	0.35	0.61	0.79	1.05	1.32	1.65	1.84	2.25
567.43	0.27	0.25	0.55	0.68	0.92	1.11	1.31	1.52	1.83	2.16	2.47
606.26	0.13	0.14	0.35	0.53	0.66	0.90	1.06	1.27	1.68	1.94	2.33
647.76	0.16	0.30	0.38	0.65	0.80	1.01	1.31	1.59	1.87	2.16	2.51
692.12	-0.09	-0.05	0.08	0.24	0.53	0.78	0.94	1.18	1.45	1.67	2.03
739.53	0.27	0.45	0.59	0.80	0.88	1.19	1.45	1.60	1.87	2.21	2.59
790.22	-0.24	-0.16	-0.02	0.02	0.33	0.56	0.83	1.06	1.36	1.66	1.92
844.40	-0.26	-0.16	0.01	0.14	0.38	0.56	0.93	1.16	1.43	1.64	2.05
902.32	0.22	0.35	0.48	0.70	0.76	1.06	1.28	1.49	1.73	2.04	2.31
964.23	-0.30	-0.27	-0.13	0.08	0.16	0.41	0.68	0.98	1.17	1.48	1.85
1030.41	0.43	0.61	0.77	0.93	1.13	1.34	1.61	1.84	2.12	2.49	2.77
1101.15	0.06	0.13	0.16	0.41	0.61	0.82	1.13	1.42	1.65	1.98	2.27
1176.77	0.30	0.44	0.71	0.80	0.94	1.19	1.41	1.59	1.93	2.21	2.46
1257.60	0.33	0.39	0.60	0.68	0.97	1.10	1.33	1.57	1.92	2.20	2.48
1344.00	-0.13	0.02	0.23	0.30	0.49	0.70	1.06	1.20	1.49	1.84	2.14
1436.36	0.05	0.19	0.37	0.50	0.79	1.00	1.26	1.50	1.79	2.14	2.53
1535.09	0.04	0.08	0.21	0.49	0.60	0.77	1.14	1.33	1.66	1.91	2.37
1640.63	-0.35	-0.28	-0.06	0.05	0.19	0.50	0.73	0.95	1.29	1.58	1.87
1753.44	-0.09	-0.02	0.26	0.31	0.53	0.60	1.01	1.27	1.43	1.73	2.06
1874.02	-0.29	-0.11	-0.04	0.07	0.20	0.36	0.70	0.87	1.23	1.52	1.89
2002.92	-0.01	0.15	0.22	0.34	0.41	0.66	1.00	1.29	1.58	1.77	2.11
2140.71	-0.36	-0.32	-0.21	-0.03	0.14	0.35	0.64	0.86	1.19	1.52	1.84
2288.00	-0.20	-0.26	-0.12	0.01	0.16	0.38	0.72	0.93	1.13	1.41	1.87

2445.44	-0.44	-0.43	-0.37	-0.09	0.04	0.17	0.33	0.64	0.93	1.24	1.61
2613.73	-0.24	-0.21	-0.11	-0.01	0.17	0.24	0.48	0.84	1.10	1.29	1.66
2793.63	0.02	0.04	0.23	0.36	0.48	0.54	0.76	1.08	1.32	1.62	1.97
2985.93	-0.22	-0.40	-0.27	-0.27	-0.27	-0.18	0.14	0.35	0.65	0.96	1.28
3191.49	-0.10	-0.14	-0.03	-0.01	0.10	0.14	0.45	0.67	0.98	1.19	1.56
3411.21	0.05	-0.10	-0.08	0.03	0.00	0.21	0.38	0.60	0.91	1.18	1.41
3646.09	0.01	-0.07	-0.02	-0.01	0.06	0.11	0.31	0.50	0.85	1.02	1.34
3897.16	0.03	-0.11	-0.08	0.05	0.05	0.14	0.41	0.67	0.87	1.17	1.52

Wavelength (nm) / Time (ps)	506.40	507.82	509.25	510.67	512.10	513.52	514.94	516.37	517.79	519.22	520.64
-2.74	-0.33	-0.41	-0.42	-0.36	-0.46	-0.42	-0.35	-0.39	-0.43	-0.44	-0.36
-2.24	-0.05	-0.16	0.01	-0.09	-0.13	-0.14	-0.17	-0.09	-0.17	-0.15	-0.12
-1.74	-0.28	-0.42	-0.32	-0.34	-0.32	-0.36	-0.49	-0.42	-0.36	-0.35	-0.33
-1.24	-0.02	0.16	0.07	0.01	-0.01	0.03	0.00	-0.04	-0.02	0.02	-0.07
-0.74	0.31	0.28	0.30	0.28	0.35	0.34	0.36	0.33	0.43	0.33	0.36
-0.24	0.09	0.15	0.08	0.18	0.13	0.17	0.18	0.17	0.17	0.18	0.16
-0.14	0.26	0.22	0.25	0.25	0.27	0.33	0.37	0.38	0.35	0.35	0.30
-0.04	0.02	0.18	0.04	0.06	0.16	0.05	0.11	0.06	0.03	0.07	0.06
0.06	0.07	0.14	0.08	0.07	0.21	0.15	0.18	0.17	0.13	0.16	0.14
0.16	0.42	0.36	0.35	0.28	0.40	0.37	0.35	0.45	0.42	0.42	0.38
0.26	0.12	0.19	0.13	0.16	0.07	0.06	0.08	0.15	0.13	0.12	0.20
0.31	0.30	0.28	0.23	0.13	0.30	0.27	0.26	0.18	0.28	0.31	0.23
0.36	0.00	-0.09	-0.11	-0.09	-0.08	-0.13	-0.07	-0.07	-0.07	-0.03	-0.06
0.41	0.17	0.15	0.14	0.14	0.11	0.22	0.11	0.17	0.16	0.13	0.12
0.46	0.25	0.27	0.16	0.19	0.33	0.29	0.29	0.22	0.31	0.31	0.26
0.51	0.05	0.08	0.05	0.02	0.02	0.01	0.05	0.08	0.06	-0.02	0.03
0.56	0.31	0.33	0.23	0.16	0.35	0.29	0.35	0.33	0.37	0.29	0.33
0.61	0.01	-0.10	-0.01	-0.10	-0.09	-0.12	-0.05	-0.06	-0.01	-0.02	0.00
0.66	0.27	0.27	0.26	0.27	0.19	0.24	0.28	0.27	0.25	0.25	0.17
0.71	0.14	0.16	0.15	0.15	0.20	0.16	0.19	0.25	0.23	0.15	0.17
0.76	0.18	0.22	0.12	0.13	0.14	0.15	0.18	0.15	0.22	0.16	0.14
0.81	0.25	0.28	0.30	0.34	0.39	0.42	0.41	0.35	0.40	0.41	0.26
0.86	-0.11	-0.11	-0.17	-0.19	-0.17	-0.19	-0.18	-0.16	-0.11	-0.05	-0.07
0.91	0.44	0.46	0.41	0.40	0.50	0.45	0.53	0.44	0.47	0.47	0.46
0.96	0.21	0.20	0.19	0.16	0.15	0.24	0.23	0.29	0.29	0.32	0.19
1.01	0.32	0.34	0.27	0.17	0.17	0.24	0.26	0.22	0.25	0.24	0.16
1.06	0.21	0.36	0.11	0.17	0.29	0.23	0.29	0.32	0.21	0.26	0.28
1.11	-0.30	-0.14	-0.16	-0.24	-0.26	-0.20	-0.09	-0.18	-0.15	-0.07	-0.12
1.16	-1.43	0.00	1.01	1.38	0.96	0.34	-0.20	-0.41	-0.09	0.24	0.33
1.21	-83.24	-82.40	-76.36	-65.42	-50.88	-34.95	-19.97	-7.43	1.15	5.55	6.34
1.26	317.95	299.85	279.27	259.04	237.07	212.40	187.42	159.83	132.57	105.25	78.52
1.31	-42.92	-5.55	33.58	75.61	118.64	160.65	201.61	238.26	271.67	299.78	321.39
1.36	5.99	7.26	9.87	12.33	12.70	8.92	0.34	-13.07	-29.96	-48.65	-66.88
1.41	3.89	2.61	1.93	1.83	2.75	4.34	6.58	9.23	12.08	14.68	17.06
1.46	6.62	8.74	10.30	11.09	11.25	10.82	10.18	9.27	8.47	7.88	7.59
1.51	4.63	4.44	4.71	5.43	6.60	7.70	8.80	9.47	9.94	9.88	9.54
1.56	8.10	6.96	5.54	4.46	3.66	3.40	3.85	4.53	5.71	7.03	8.40
1.61	5.02	6.34	7.24	7.50	7.55	7.07	6.71	6.05	5.77	5.62	5.69

1.66	2.99	2.93	3.35	4.22	5.49	6.76	8.02	8.95	9.67	9.99	9.87
1.71	6.97	7.50	7.50	7.18	6.63	5.89	5.35	5.05	5.08	5.28	5.80
1.76	3.30	4.28	5.27	6.37	7.30	8.01	8.24	8.17	7.94	7.48	7.00
1.81	3.98	3.18	2.73	2.72	3.03	3.69	4.55	5.57	6.60	7.26	7.98
1.86	5.69	5.99	6.09	5.95	5.70	5.36	5.11	5.14	5.31	5.79	6.38
1.91	2.69	3.16	3.92	5.03	6.06	6.97	7.59	7.82	7.89	7.66	7.27
1.96	5.62	5.12	4.53	4.00	3.65	3.69	4.00	4.61	5.33	6.05	6.94
2.01	3.75	4.78	5.76	6.46	6.70	6.69	6.41	5.91	5.54	5.29	5.17
2.06	3.26	3.15	3.14	3.55	4.21	4.98	5.97	6.72	7.34	7.82	8.05
2.11	5.25	5.28	5.00	4.77	4.60	4.40	4.50	4.65	5.10	5.58	6.15
2.16	3.36	4.16	4.88	5.72	6.36	6.63	6.79	6.68	6.50	6.25	6.08
2.21	3.77	3.38	3.16	3.22	3.60	4.08	4.90	5.75	6.67	7.33	7.94
2.26	4.89	5.53	6.00	5.92	5.89	5.67	5.43	5.28	5.33	5.56	5.87
2.31	2.92	3.26	3.75	4.40	5.01	5.63	6.33	6.69	6.86	6.91	6.83
2.36	3.53	3.43	3.40	3.54	3.89	4.18	4.78	5.18	5.74	6.22	6.69
2.41	4.20	5.07	5.40	5.60	5.74	5.63	5.54	5.34	5.33	5.37	5.66
2.46	2.87	2.91	3.08	3.55	4.10	4.82	5.39	5.98	6.52	6.77	6.88
2.51	5.32	5.61	5.45	5.31	4.99	4.64	4.54	4.63	4.86	5.37	5.97
2.56	3.56	4.22	4.86	5.33	5.79	6.06	6.25	6.31	6.38	6.39	6.40
2.61	3.01	3.14	3.38	3.95	4.49	5.06	5.62	6.07	6.54	6.83	7.00
2.66	4.85	5.02	5.20	5.20	5.13	4.97	5.03	5.21	5.51	5.81	6.32
2.71	3.08	3.59	4.23	4.80	5.34	5.78	6.07	6.22	6.45	6.34	6.33
2.76	4.09	4.07	3.95	4.00	4.19	4.51	4.91	5.47	6.04	6.50	7.00
2.81	3.97	4.38	4.74	4.93	5.06	5.16	5.09	5.27	5.35	5.52	5.78
2.86	2.75	3.17	3.55	4.04	4.60	5.01	5.36	5.63	5.82	5.99	6.08
2.91	4.34	4.36	4.43	4.42	4.42	4.53	4.76	5.09	5.55	5.96	6.47
2.96	3.48	4.10	4.49	5.03	5.32	5.48	5.59	5.64	5.74	5.84	5.99
3.01	3.64	3.63	3.66	3.83	4.16	4.68	5.05	5.63	6.13	6.57	6.89
3.06	3.78	4.13	4.20	4.40	4.49	4.53	4.66	4.72	5.04	5.37	5.59
3.11	3.17	3.56	3.94	4.36	4.78	5.09	5.32	5.47	5.69	5.82	5.98
3.16	3.68	3.71	3.78	3.97	4.18	4.53	4.95	5.39	5.94	6.27	6.65
3.21	4.08	4.35	4.54	4.77	4.87	4.99	5.04	5.33	5.57	5.83	6.16
3.26	3.23	3.60	4.00	4.34	4.92	5.25	5.65	5.98	6.22	6.36	6.52
3.31	4.07	4.23	4.23	4.32	4.58	4.79	5.12	5.51	5.83	6.26	6.62
3.36	3.79	4.30	4.56	4.89	5.04	5.28	5.38	5.52	5.71	6.01	6.21
3.41	3.37	3.58	3.85	4.25	4.70	5.10	5.54	5.86	6.19	6.48	6.71
3.46	4.05	4.26	4.31	4.42	4.55	4.77	4.92	5.34	5.59	5.98	6.45
3.51	3.43	3.83	4.18	4.66	5.00	5.32	5.61	5.91	6.10	6.21	6.43
3.56	3.62	3.67	3.79	4.06	4.40	4.70	5.10	5.52	5.89	6.25	6.57
3.61	3.54	3.77	3.93	4.14	4.28	4.41	4.66	4.83	5.24	5.49	5.80
3.66	3.04	3.46	3.74	4.16	4.58	4.91	5.26	5.58	5.80	5.99	6.13
3.71	3.92	4.12	4.19	4.29	4.49	4.64	4.97	5.36	5.73	6.08	6.45
3.76	4.11	4.42	4.63	5.01	5.22	5.37	5.67	5.80	6.13	6.33	6.61
3.81	3.18	3.40	3.59	3.99	4.36	4.70	5.01	5.42	5.67	5.95	6.09
3.86	3.31	3.48	3.60	3.85	3.97	4.13	4.37	4.68	5.03	5.45	5.75
3.91	3.43	3.84	4.11	4.52	4.85	5.09	5.47	5.68	5.87	6.08	6.27
3.96	3.42	3.62	3.75	4.02	4.32	4.71	5.02	5.43	5.79	6.11	6.35
4.01	4.18	4.46	4.72	4.92	5.12	5.18	5.42	5.69	5.92	6.21	6.56
4.06	3.51	3.78	4.17	4.45	4.82	5.13	5.50	5.76	6.05	6.28	6.42
4.11	3.31	3.52	3.73	3.92	4.21	4.46	4.91	5.19	5.54	5.93	6.22

4.16	3.92	4.22	4.50	4.81	5.01	5.18	5.44	5.66	5.90	6.13	6.37
4.21	3.67	3.98	4.09	4.40	4.73	5.12	5.50	5.86	6.19	6.43	6.73
4.26	3.89	4.09	4.25	4.47	4.76	4.97	5.15	5.50	5.80	6.17	6.47
4.31	3.16	3.46	3.82	4.12	4.47	4.64	4.99	5.16	5.52	5.64	5.85
4.36	3.18	3.46	3.73	4.07	4.34	4.72	4.94	5.36	5.65	5.83	6.15
4.41	3.56	3.81	3.97	4.28	4.46	4.68	4.95	5.21	5.49	5.76	6.10
4.46	3.67	4.00	4.32	4.67	4.90	5.32	5.58	5.86	6.09	6.26	6.55
4.51	3.55	3.77	3.91	4.15	4.44	4.68	5.08	5.36	5.78	6.11	6.29
4.56	3.41	3.73	4.04	4.17	4.46	4.69	4.92	5.09	5.44	5.68	6.07
4.61	2.87	3.16	3.44	3.75	4.06	4.28	4.64	4.89	5.17	5.42	5.72
4.66	3.67	3.88	4.12	4.37	4.65	4.87	5.11	5.41	5.81	6.13	6.42
4.71	3.75	4.03	4.29	4.59	4.93	5.04	5.40	5.60	5.85	6.09	6.30
4.76	3.65	3.89	4.18	4.54	4.80	5.17	5.50	5.84	6.13	6.46	6.67
4.81	3.49	3.75	3.89	4.03	4.45	4.60	4.86	5.11	5.48	5.72	6.04
4.86	3.06	3.45	3.69	4.03	4.26	4.47	4.85	5.00	5.28	5.50	5.84
4.91	3.75	3.95	4.22	4.43	4.85	5.12	5.48	5.68	6.04	6.29	6.68
4.96	3.42	3.83	4.01	4.27	4.58	4.77	5.09	5.25	5.61	5.87	6.13
5.01	3.46	3.77	4.04	4.30	4.70	4.97	5.30	5.55	5.89	6.10	6.40
5.06	3.32	3.59	3.71	3.88	4.23	4.43	4.71	5.00	5.40	5.78	5.96
5.11	3.35	3.59	3.81	4.12	4.44	4.62	4.99	5.17	5.56	5.79	6.06
5.16	3.22	3.54	3.81	4.10	4.48	4.69	4.98	5.34	5.57	5.87	6.03
5.21	3.07	3.39	3.51	3.77	4.14	4.28	4.68	4.92	5.27	5.48	5.90
5.26	3.70	3.84	4.13	4.49	4.84	5.11	5.35	5.61	5.97	6.13	6.45
5.31	3.29	3.56	3.79	4.00	4.25	4.68	4.96	5.31	5.58	5.92	6.27
5.38	3.04	3.30	3.49	3.80	4.12	4.38	4.76	5.04	5.40	5.69	5.92
5.45	3.61	3.86	4.11	4.50	4.70	4.90	5.26	5.57	5.97	6.22	6.48
5.53	3.12	3.39	3.61	3.91	4.22	4.42	4.68	5.00	5.33	5.72	6.12
5.61	3.54	3.89	4.11	4.41	4.63	4.88	5.23	5.46	5.76	6.13	6.37
5.70	3.35	3.59	3.80	4.05	4.49	4.66	4.97	5.29	5.57	5.89	6.23
5.80	3.28	3.61	3.93	4.22	4.48	4.70	4.96	5.25	5.53	5.83	6.15
5.90	3.47	3.69	3.97	4.24	4.53	4.73	5.01	5.27	5.62	5.92	6.21
6.01	3.72	3.90	4.24	4.43	4.71	4.96	5.27	5.55	5.84	6.13	6.48
6.13	3.59	3.82	4.09	4.34	4.68	4.89	5.09	5.45	5.79	6.01	6.32
6.25	3.43	3.76	3.91	4.24	4.48	4.78	5.07	5.41	5.79	5.96	6.23
6.39	3.64	3.94	4.32	4.56	4.88	5.19	5.48	5.74	5.96	6.27	6.57
6.53	3.63	3.98	4.24	4.52	4.90	5.11	5.36	5.68	5.95	6.24	6.42
6.69	3.88	4.15	4.43	4.70	5.08	5.39	5.64	5.96	6.24	6.50	6.77
6.85	3.65	3.96	4.15	4.40	4.69	4.94	5.28	5.69	5.93	6.15	6.35
7.03	3.68	3.88	4.21	4.48	4.82	5.13	5.32	5.62	5.93	6.18	6.43
7.21	3.77	4.10	4.38	4.68	5.02	5.29	5.57	5.88	6.12	6.37	6.72
7.41	3.83	4.18	4.35	4.65	5.07	5.29	5.53	5.86	6.17	6.49	6.69
7.63	3.72	3.99	4.32	4.61	4.95	5.24	5.53	5.79	6.11	6.47	6.77
7.86	3.86	4.11	4.38	4.62	5.01	5.24	5.55	5.84	6.16	6.42	6.67
8.10	3.61	3.86	4.10	4.41	4.72	5.09	5.28	5.55	5.89	6.14	6.42
8.36	3.37	3.58	3.94	4.21	4.55	4.80	5.16	5.39	5.71	5.98	6.28
8.64	3.84	4.21	4.35	4.74	5.04	5.25	5.64	5.86	6.20	6.48	6.85
8.94	3.99	4.35	4.50	4.82	5.14	5.42	5.76	6.10	6.31	6.68	6.93
9.26	4.24	4.54	4.78	5.07	5.36	5.72	6.03	6.34	6.68	6.94	7.22
9.60	3.75	4.11	4.39	4.71	5.01	5.32	5.60	5.79	6.11	6.52	6.64
9.97	3.63	3.91	4.19	4.53	4.81	5.14	5.39	5.70	6.01	6.29	6.56

10.36	4.07	4.41	4.69	4.93	5.28	5.53	5.80	6.08	6.38	6.67	6.94
10.77	3.92	4.25	4.46	4.78	5.09	5.33	5.71	5.98	6.36	6.59	6.89
11.22	3.98	4.29	4.54	4.86	5.15	5.42	5.75	6.08	6.32	6.57	6.83
11.70	4.06	4.30	4.51	4.84	5.21	5.47	5.93	6.19	6.41	6.68	6.90
12.21	3.55	3.92	4.13	4.45	4.76	5.04	5.32	5.52	5.87	6.09	6.38
12.75	3.87	4.16	4.40	4.75	5.07	5.34	5.62	5.83	6.09	6.42	6.78
13.33	3.99	4.31	4.52	4.78	5.12	5.49	5.76	5.94	6.35	6.62	6.93
13.95	3.82	4.14	4.37	4.67	5.07	5.33	5.69	5.94	6.14	6.48	6.73
14.62	3.60	3.91	4.23	4.52	4.84	5.16	5.44	5.62	6.06	6.20	6.52
15.33	3.54	3.81	4.09	4.38	4.73	4.99	5.34	5.57	5.88	6.25	6.40
16.09	3.59	3.98	4.19	4.46	4.84	5.11	5.35	5.54	5.95	6.23	6.40
16.90	3.58	3.69	4.06	4.35	4.62	4.94	5.29	5.56	5.90	6.18	6.41
17.77	3.80	4.04	4.31	4.57	4.96	5.19	5.56	5.79	6.08	6.40	6.61
18.70	3.71	4.09	4.40	4.64	4.99	5.21	5.56	5.75	6.07	6.31	6.54
19.69	3.62	3.90	4.18	4.46	4.84	4.96	5.39	5.57	5.96	6.15	6.46
20.75	3.60	3.74	4.13	4.42	4.69	5.01	5.31	5.62	5.81	6.08	6.36
21.89	3.91	4.18	4.48	4.72	5.11	5.38	5.70	5.92	6.29	6.59	6.87
23.10	4.17	4.53	4.72	5.00	5.31	5.67	5.88	6.21	6.62	6.80	7.11
24.39	3.76	4.14	4.45	4.70	4.99	5.38	5.57	5.87	6.12	6.42	6.67
25.78	3.68	4.00	4.30	4.51	4.80	5.12	5.45	5.65	6.03	6.33	6.60
27.26	3.78	4.01	4.31	4.54	4.85	5.27	5.43	5.76	6.10	6.41	6.66
28.84	3.64	4.04	4.29	4.54	4.84	5.16	5.48	5.72	6.03	6.35	6.65
30.53	3.80	4.13	4.46	4.79	5.03	5.27	5.59	5.81	6.07	6.34	6.72
32.34	3.57	3.82	4.07	4.36	4.73	4.84	5.19	5.49	5.79	5.92	6.26
34.27	3.74	4.15	4.38	4.68	4.92	5.26	5.45	5.71	5.99	6.30	6.45
36.34	3.61	3.93	4.20	4.49	4.76	5.08	5.34	5.60	5.80	5.99	6.29
38.55	3.63	4.05	4.19	4.53	4.86	5.10	5.39	5.59	5.96	6.17	6.44
40.91	3.74	4.00	4.26	4.51	4.93	5.13	5.45	5.64	6.02	6.31	6.48
43.43	3.39	3.68	3.94	4.21	4.46	4.79	5.06	5.28	5.58	5.85	6.04
46.13	3.66	3.96	4.20	4.52	4.76	5.01	5.38	5.60	5.91	6.13	6.34
49.01	3.66	3.97	4.27	4.54	4.84	5.08	5.34	5.54	5.85	6.13	6.41
52.09	3.30	3.59	3.94	4.25	4.54	4.76	5.07	5.28	5.54	5.81	6.09
55.39	3.52	3.71	4.01	4.30	4.58	4.78	5.09	5.31	5.61	5.80	6.07
58.91	3.29	3.58	3.87	4.17	4.38	4.62	4.98	5.10	5.47	5.70	5.90
62.68	3.11	3.30	3.62	3.85	4.22	4.44	4.69	4.89	5.23	5.48	5.68
66.70	3.33	3.70	3.87	4.18	4.44	4.69	4.95	5.21	5.49	5.72	5.95
71.00	3.05	3.32	3.57	3.91	4.11	4.32	4.58	4.89	5.23	5.43	5.66
75.60	3.20	3.54	3.76	4.10	4.30	4.59	4.77	5.07	5.30	5.62	5.82
80.51	3.32	3.53	3.87	4.07	4.33	4.48	4.72	4.90	5.28	5.46	5.73
85.77	2.82	3.22	3.41	3.71	3.99	4.25	4.45	4.66	4.94	5.28	5.57
91.38	3.48	3.82	3.98	4.30	4.57	4.81	4.99	5.18	5.58	5.78	6.07
97.39	2.90	3.24	3.43	3.66	3.91	4.11	4.36	4.51	4.77	4.95	5.32
103.80	3.33	3.63	3.92	4.12	4.37	4.62	5.02	5.10	5.45	5.74	5.91
110.66	3.29	3.51	3.82	4.02	4.30	4.44	4.73	4.96	5.19	5.44	5.60
117.99	2.87	3.16	3.28	3.54	3.81	4.07	4.29	4.43	4.66	5.09	5.25
125.83	3.53	3.70	3.94	4.25	4.48	4.69	4.86	5.22	5.42	5.58	5.77
134.21	3.04	3.38	3.63	3.85	4.09	4.32	4.52	4.65	4.90	5.09	5.38
143.17	2.88	3.13	3.34	3.61	3.76	3.99	4.21	4.37	4.64	4.89	5.13
152.74	3.16	3.45	3.75	3.94	4.22	4.52	4.76	4.92	5.24	5.57	5.68
162.97	3.14	3.45	3.78	4.06	4.49	4.71	5.14	5.40	5.81	6.09	6.47

173.91	3.00	3.38	3.57	3.93	4.24	4.54	4.93	5.26	5.55	5.88	6.26
185.60	3.16	3.58	3.81	4.17	4.45	4.77	5.17	5.46	5.67	6.12	6.43
198.10	2.92	3.18	3.61	3.90	4.17	4.57	4.92	5.21	5.50	5.80	6.18
211.46	3.18	3.52	3.76	4.00	4.48	4.78	5.16	5.37	5.80	6.17	6.45
225.75	3.05	3.35	3.69	4.05	4.41	4.63	5.06	5.31	5.62	5.93	6.32
241.01	2.98	3.25	3.60	3.92	4.14	4.53	4.80	5.07	5.47	5.74	6.14
257.33	2.81	3.26	3.42	3.79	4.11	4.41	4.78	5.02	5.41	5.75	6.01
274.77	3.17	3.55	3.81	4.22	4.60	4.83	5.19	5.46	5.83	6.13	6.46
293.42	2.98	3.24	3.63	3.90	4.23	4.56	4.90	5.21	5.63	5.97	6.28
313.35	2.98	3.27	3.49	4.00	4.29	4.44	4.88	5.15	5.57	5.89	6.22
334.66	3.09	3.44	3.67	4.08	4.41	4.66	5.08	5.44	5.74	6.05	6.45
357.43	2.79	3.10	3.38	3.72	4.11	4.47	4.76	4.99	5.39	5.72	6.12
381.78	3.11	3.40	3.74	4.09	4.45	4.73	5.10	5.40	5.71	6.04	6.41
407.80	2.81	3.13	3.42	3.78	4.13	4.53	4.76	5.10	5.49	5.80	6.10
435.62	2.82	3.14	3.54	3.88	4.18	4.58	4.87	5.24	5.62	5.99	6.25
465.35	2.93	3.24	3.60	3.88	4.20	4.62	4.92	5.25	5.65	5.91	6.19
497.14	2.73	3.01	3.23	3.55	3.95	4.26	4.58	4.94	5.21	5.60	5.85
531.11	2.61	2.92	3.24	3.61	4.00	4.28	4.62	5.02	5.33	5.61	5.91
567.43	2.77	3.25	3.45	3.81	4.22	4.49	4.86	5.24	5.56	5.87	6.19
606.26	2.49	2.81	3.21	3.58	3.87	4.20	4.55	4.85	5.17	5.59	5.92
647.76	2.80	3.16	3.45	3.80	4.20	4.41	4.84	5.18	5.53	5.85	6.14
692.12	2.36	2.77	3.05	3.36	3.72	4.01	4.39	4.62	5.04	5.32	5.69
739.53	2.84	3.28	3.49	3.92	4.20	4.63	4.91	5.18	5.53	5.86	6.15
790.22	2.26	2.52	2.98	3.29	3.66	3.86	4.30	4.55	4.88	5.24	5.68
844.40	2.42	2.70	3.06	3.37	3.73	4.00	4.35	4.71	5.19	5.44	5.81
902.32	2.67	3.03	3.34	3.71	4.07	4.29	4.70	5.03	5.46	5.71	6.07
964.23	2.13	2.55	2.84	3.21	3.56	3.77	4.18	4.44	4.79	5.18	5.46
1030.41	3.03	3.43	3.70	4.03	4.42	4.72	5.00	5.40	5.73	6.02	6.28
1101.15	2.60	2.95	3.27	3.56	3.97	4.26	4.68	4.93	5.33	5.67	5.94
1176.77	2.78	3.10	3.48	3.77	4.14	4.53	4.88	5.12	5.56	5.85	6.08
1257.60	2.78	3.20	3.45	3.87	4.21	4.57	4.82	5.21	5.52	5.97	6.16
1344.00	2.46	2.78	3.09	3.44	3.90	4.16	4.52	4.82	5.17	5.47	5.86
1436.36	2.83	3.18	3.47	3.82	4.26	4.54	4.99	5.17	5.53	5.79	6.06
1535.09	2.66	2.92	3.24	3.70	3.99	4.29	4.64	5.02	5.37	5.73	5.96
1640.63	2.20	2.61	2.88	3.26	3.53	3.95	4.27	4.63	4.98	5.19	5.58
1753.44	2.49	2.82	3.11	3.44	3.77	4.15	4.51	4.75	5.24	5.51	5.79
1874.02	2.09	2.55	2.75	3.10	3.51	3.82	4.18	4.41	4.86	5.19	5.52
2002.92	2.54	2.83	3.09	3.41	3.83	4.18	4.53	4.88	5.21	5.54	5.85
2140.71	2.02	2.42	2.74	3.11	3.45	3.80	4.16	4.46	4.82	5.16	5.38
2288.00	2.13	2.43	2.76	3.11	3.45	3.82	4.18	4.42	4.76	5.09	5.45
2445.44	1.93	2.25	2.53	2.92	3.23	3.52	3.93	4.19	4.62	4.91	5.20
2613.73	1.97	2.41	2.59	3.01	3.26	3.62	4.01	4.36	4.74	5.04	5.46
2793.63	2.26	2.51	2.76	3.12	3.46	3.80	4.17	4.42	4.83	5.16	5.50
2985.93	1.55	1.84	2.12	2.44	2.72	3.00	3.28	3.73	4.04	4.41	4.78
3191.49	1.74	2.06	2.32	2.61	3.02	3.28	3.71	4.02	4.31	4.65	5.04
3411.21	1.75	1.98	2.13	2.47	2.86	3.11	3.44	3.75	4.17	4.43	4.82
3646.09	1.60	1.88	2.13	2.38	2.71	3.02	3.35	3.65	4.11	4.43	4.83
3897.16	1.71	1.99	2.28	2.48	2.80	3.09	3.47	3.76	4.24	4.46	4.90

Wavelength (nm) / Time (ps)	522.06	523.49	524.91	526.34	527.76	529.18	530.61	532.03	533.46	534.88	536.30
-2.74	-0.39	-0.30	-0.30	-0.26	-0.21	-0.15	-0.10	-0.09	-0.15	-0.03	-0.06
-2.24	-0.07	-0.10	-0.17	-0.07	-0.09	-0.11	-0.08	-0.07	-0.07	-0.09	-0.05
-1.74	-0.28	-0.36	-0.21	-0.18	-0.20	-0.26	-0.20	-0.21	-0.10	-0.12	-0.07
-1.24	-0.02	0.05	0.10	-0.03	0.07	0.01	-0.01	0.07	0.01	-0.03	-0.08
-0.74	0.31	0.25	0.26	0.23	0.22	0.19	0.20	0.22	0.17	0.13	0.15
-0.24	0.19	0.12	0.08	0.19	0.02	0.14	0.03	0.05	0.06	0.00	-0.01
-0.14	0.20	0.28	0.21	0.18	0.17	0.16	0.17	0.02	0.07	0.12	0.09
-0.04	0.05	0.07	0.03	-0.05	0.01	0.03	-0.01	0.01	0.03	0.03	0.02
0.06	0.11	0.12	0.13	0.06	0.06	-0.04	0.00	0.03	0.06	-0.01	0.00
0.16	0.30	0.30	0.26	0.30	0.34	0.20	0.25	0.19	0.18	0.17	0.14
0.26	0.18	0.12	0.17	0.16	0.18	0.13	0.08	0.14	0.19	-0.02	0.02
0.31	0.13	0.21	0.17	0.21	0.23	0.10	0.09	0.18	0.07	0.04	0.09
0.36	-0.01	-0.01	-0.03	-0.08	-0.08	-0.10	-0.07	0.00	-0.04	-0.11	-0.07
0.41	0.13	0.12	0.10	0.02	0.08	-0.03	0.01	0.02	-0.03	-0.11	-0.02
0.46	0.31	0.23	0.32	0.25	0.27	0.25	0.19	0.23	0.21	0.13	0.20
0.51	0.09	0.07	0.04	0.13	0.07	0.06	-0.08	0.10	0.06	0.04	-0.01
0.56	0.25	0.31	0.35	0.25	0.27	0.11	0.07	0.16	0.15	0.17	0.08
0.61	-0.07	0.07	0.03	-0.01	-0.06	-0.04	-0.05	-0.03	0.00	-0.08	-0.07
0.66	0.19	0.17	0.25	0.19	0.19	0.11	0.06	0.17	0.05	0.03	0.09
0.71	0.17	0.24	0.26	0.18	0.15	0.12	0.05	0.08	0.09	0.13	0.03
0.76	0.17	0.12	0.13	0.10	0.23	0.15	0.10	0.10	0.12	0.14	0.06
0.81	0.37	0.31	0.25	0.27	0.23	0.12	0.13	0.21	0.11	0.13	0.08
0.86	-0.07	-0.17	-0.07	-0.08	-0.08	-0.05	-0.17	-0.16	-0.04	-0.08	-0.05
0.91	0.43	0.37	0.36	0.36	0.24	0.22	0.22	0.16	0.19	0.15	0.06
0.96	0.32	0.28	0.29	0.31	0.26	0.19	0.18	0.14	0.26	0.09	0.05
1.01	0.18	0.25	0.21	0.21	0.11	0.12	0.06	0.12	0.08	0.07	0.07
1.06	0.10	0.26	0.20	0.15	0.28	0.11	0.13	0.19	0.12	0.04	0.11
1.11	-0.19	-0.15	-0.06	-0.14	0.01	-0.05	-0.14	-0.16	0.00	0.00	-0.12
1.16	0.39	0.50	0.55	0.41	0.22	0.19	0.25	0.11	0.06	-0.05	-0.02
1.21	4.61	1.64	-1.27	-3.37	-4.26	-4.06	-3.15	-1.80	-0.21	0.83	1.33
1.26	52.69	28.33	5.26	-15.46	-33.07	-48.00	-59.43	-67.28	-71.50	-73.39	-72.24
1.31	335.88	342.59	340.55	329.13	317.61	301.60	281.51	260.42	241.65	224.09	208.33
1.36	-82.53	-94.75	-102.16	-103.87	-102.73	-96.59	-86.31	-72.51	-55.07	-35.09	-11.46
1.41	18.83	20.12	20.69	20.61	19.90	18.89	17.60	16.39	15.19	13.93	13.15
1.46	7.65	8.09	8.96	9.65	10.22	10.58	10.71	10.54	10.08	9.42	9.09
1.51	8.93	8.17	7.37	6.84	6.51	6.50	7.02	7.80	8.79	9.83	10.90
1.56	9.54	10.37	10.95	10.89	10.77	10.41	10.09	9.81	9.60	9.60	9.85
1.61	6.21	6.92	7.92	8.92	9.84	10.60	11.09	11.37	11.23	11.02	10.69
1.66	9.59	9.02	8.47	7.86	7.61	7.49	7.71	8.09	8.62	9.18	9.87
1.71	6.73	7.53	8.37	9.07	9.49	9.64	9.61	9.41	9.21	8.94	8.65
1.76	6.46	6.22	6.32	6.73	7.28	8.04	8.77	9.76	10.42	10.99	11.30
1.81	8.39	8.59	8.47	8.22	7.92	7.59	7.45	7.52	7.79	8.11	8.48
1.86	7.10	7.85	8.43	9.03	9.40	9.61	9.49	9.36	9.21	8.91	8.80
1.91	6.83	6.30	6.12	6.07	6.25	6.57	7.07	7.74	8.33	8.93	9.42
1.96	7.64	8.14	8.40	8.47	8.36	8.20	8.04	8.00	7.97	8.05	8.36
2.01	5.27	5.83	6.55	7.45	8.36	9.00	9.70	10.10	10.22	10.24	10.14

2.06	8.13	8.01	7.72	7.43	7.24	7.13	7.23	7.45	7.93	8.19	8.67
2.11	6.87	7.51	7.89	8.18	8.27	8.39	8.26	8.25	8.18	8.17	8.30
2.16	5.92	5.93	6.23	6.68	7.25	7.82	8.40	9.02	9.49	9.72	9.94
2.21	8.15	8.27	8.18	8.09	7.96	7.74	7.75	7.83	8.14	8.47	8.86
2.26	6.44	6.99	7.67	8.28	8.73	9.06	9.20	9.41	9.32	9.27	9.06
2.31	6.75	6.69	6.62	6.61	6.75	7.00	7.42	7.87	8.33	8.74	9.26
2.36	7.07	7.27	7.39	7.39	7.52	7.57	7.70	7.86	8.00	8.34	8.49
2.41	6.06	6.49	7.09	7.65	8.21	8.64	8.85	8.99	9.11	9.11	9.07
2.46	6.99	6.84	6.79	6.75	6.72	6.77	7.06	7.33	7.74	8.14	8.54
2.51	6.67	7.24	7.98	8.39	8.75	8.90	8.87	8.87	8.77	8.70	8.76
2.56	6.50	6.65	6.96	7.31	7.69	8.06	8.60	9.02	9.27	9.45	9.61
2.61	7.08	7.05	6.97	7.04	7.18	7.18	7.41	7.74	8.03	8.46	8.70
2.66	6.84	7.35	7.78	8.18	8.43	8.58	8.68	8.73	8.78	8.76	8.84
2.71	6.32	6.45	6.66	6.78	7.18	7.58	7.93	8.36	8.82	9.08	9.32
2.76	7.39	7.70	7.88	7.99	8.06	8.03	8.01	8.24	8.32	8.60	8.83
2.81	6.14	6.49	7.01	7.44	7.84	8.16	8.38	8.57	8.74	8.72	8.81
2.86	6.18	6.18	6.32	6.44	6.76	7.07	7.38	7.77	8.16	8.44	8.76
2.91	6.90	7.29	7.63	7.81	7.99	8.17	8.17	8.25	8.38	8.40	8.67
2.96	6.17	6.43	6.88	7.23	7.66	8.04	8.42	8.83	8.97	9.14	9.24
3.01	7.15	7.29	7.53	7.48	7.56	7.71	7.79	8.00	8.22	8.62	8.82
3.06	6.07	6.49	6.98	7.27	7.66	7.83	8.01	8.33	8.30	8.44	8.49
3.11	6.20	6.38	6.61	6.99	7.20	7.58	7.97	8.38	8.66	8.89	9.11
3.16	6.96	7.25	7.42	7.47	7.61	7.71	7.82	7.96	8.19	8.41	8.66
3.21	6.59	6.87	7.29	7.50	7.84	8.06	8.29	8.43	8.60	8.70	8.83
3.26	6.63	6.81	6.90	7.18	7.38	7.57	7.86	8.16	8.50	8.74	8.94
3.31	6.99	7.25	7.51	7.68	7.93	8.03	8.07	8.19	8.35	8.58	8.77
3.36	6.55	6.78	7.18	7.52	7.86	8.22	8.45	8.75	8.91	9.22	9.26
3.41	6.87	6.98	7.15	7.35	7.47	7.61	7.89	8.25	8.52	8.78	9.03
3.46	6.77	7.18	7.51	7.71	7.93	8.05	8.26	8.40	8.58	8.66	8.87
3.51	6.61	6.75	6.97	7.25	7.60	7.84	8.15	8.51	8.75	9.06	9.24
3.56	6.86	7.01	7.23	7.32	7.47	7.61	7.65	7.96	8.26	8.44	8.71
3.61	6.26	6.60	6.84	7.13	7.36	7.65	7.79	8.03	8.21	8.44	8.56
3.66	6.28	6.44	6.63	6.83	7.04	7.35	7.69	8.01	8.29	8.52	8.73
3.71	6.92	7.20	7.41	7.70	7.91	8.03	8.19	8.37	8.60	8.74	8.89
3.76	6.87	7.15	7.50	7.80	8.05	8.32	8.56	8.84	9.07	9.22	9.41
3.81	6.29	6.52	6.63	6.92	7.12	7.32	7.63	7.92	8.19	8.42	8.68
3.86	6.15	6.46	6.78	7.01	7.37	7.56	7.68	7.83	8.10	8.26	8.44
3.91	6.50	6.80	7.05	7.32	7.60	7.98	8.21	8.48	8.82	9.07	9.17
3.96	6.63	6.90	7.10	7.31	7.51	7.67	7.88	8.11	8.32	8.70	8.91
4.01	6.79	7.17	7.57	7.81	8.12	8.31	8.58	8.75	8.89	9.09	9.19
4.06	6.68	6.92	7.21	7.40	7.67	7.90	8.21	8.51	8.69	9.03	9.34
4.11	6.60	6.76	7.10	7.26	7.47	7.57	7.82	8.17	8.34	8.59	8.82
4.16	6.70	7.00	7.26	7.67	7.86	8.10	8.38	8.72	8.82	9.08	9.24
4.21	6.96	7.10	7.31	7.49	7.80	8.06	8.29	8.58	8.85	9.17	9.44
4.26	6.80	7.07	7.31	7.58	7.82	8.05	8.18	8.47	8.57	8.80	8.96
4.31	6.20	6.36	6.79	6.95	7.32	7.59	7.89	8.26	8.43	8.67	8.93
4.36	6.45	6.71	6.90	7.02	7.40	7.55	7.86	8.17	8.40	8.66	8.89
4.41	6.37	6.76	7.07	7.20	7.61	7.79	7.95	8.20	8.44	8.58	8.72
4.46	6.77	7.04	7.24	7.55	7.90	8.16	8.39	8.72	8.98	9.24	9.47
4.51	6.60	6.99	7.15	7.41	7.64	7.89	8.06	8.30	8.60	8.79	9.01

4.56	6.25	6.58	6.93	7.25	7.45	7.69	7.92	8.17	8.38	8.63	8.80
4.61	5.96	6.20	6.51	6.76	7.06	7.29	7.58	7.88	8.14	8.43	8.70
4.66	6.74	7.03	7.29	7.47	7.79	7.99	8.07	8.40	8.64	8.75	9.00
4.71	6.66	6.92	7.17	7.43	7.72	8.08	8.33	8.58	8.78	8.99	9.20
4.76	6.94	7.15	7.48	7.67	7.91	8.15	8.36	8.68	8.98	9.19	9.48
4.81	6.35	6.72	7.00	7.27	7.55	7.72	8.03	8.26	8.45	8.67	8.85
4.86	6.08	6.38	6.61	6.79	7.26	7.53	7.77	8.03	8.35	8.63	8.86
4.91	7.02	7.21	7.48	7.71	7.90	8.09	8.31	8.68	8.87	9.06	9.37
4.96	6.39	6.67	7.02	7.22	7.63	7.83	8.02	8.36	8.60	8.78	9.13
5.01	6.58	6.91	7.21	7.36	7.71	7.84	8.18	8.50	8.68	8.95	9.18
5.06	6.31	6.61	6.93	7.07	7.49	7.60	7.81	8.26	8.47	8.72	8.93
5.11	6.42	6.72	6.91	7.22	7.45	7.80	8.00	8.32	8.54	8.64	8.87
5.16	6.33	6.59	6.84	7.03	7.24	7.58	7.88	8.06	8.43	8.67	8.87
5.21	6.20	6.57	6.84	7.10	7.40	7.63	7.85	8.21	8.44	8.68	8.87
5.26	6.66	6.98	7.25	7.50	7.77	8.01	8.30	8.63	8.89	9.13	9.29
5.31	6.49	6.73	6.97	7.23	7.54	7.78	7.99	8.32	8.52	8.79	9.01
5.38	6.38	6.62	6.87	7.11	7.45	7.61	7.99	8.23	8.42	8.69	9.03
5.45	6.78	7.06	7.38	7.55	7.76	8.00	8.29	8.57	8.85	9.09	9.39
5.53	6.35	6.55	6.82	7.12	7.36	7.66	7.91	8.20	8.55	8.75	9.00
5.61	6.67	6.93	7.21	7.56	7.93	8.17	8.38	8.67	8.97	9.17	9.32
5.70	6.47	6.70	6.96	7.18	7.50	7.68	7.95	8.35	8.60	8.88	9.14
5.80	6.50	6.74	7.12	7.34	7.69	7.95	8.21	8.59	8.86	9.12	9.26
5.90	6.52	6.76	7.10	7.28	7.56	7.86	8.05	8.31	8.54	8.72	8.98
6.01	6.81	7.06	7.44	7.68	7.95	8.14	8.53	8.81	9.05	9.22	9.52
6.13	6.66	6.97	7.22	7.40	7.84	8.03	8.38	8.58	8.78	9.01	9.27
6.25	6.46	6.76	7.07	7.22	7.46	7.68	7.91	8.29	8.48	8.66	8.88
6.39	6.78	7.05	7.27	7.47	7.78	7.99	8.23	8.43	8.68	8.90	9.11
6.53	6.70	6.99	7.26	7.48	7.74	8.03	8.22	8.48	8.68	8.87	9.12
6.69	6.99	7.28	7.51	7.72	7.95	8.29	8.52	8.81	8.97	9.23	9.41
6.85	6.75	6.85	7.18	7.44	7.65	7.97	8.06	8.41	8.61	8.92	9.08
7.03	6.74	6.93	7.23	7.41	7.74	7.96	8.25	8.55	8.80	8.93	9.22
7.21	6.95	7.20	7.51	7.65	7.88	8.18	8.33	8.58	8.83	9.07	9.29
7.41	6.92	7.17	7.48	7.70	7.97	8.20	8.42	8.73	8.94	9.15	9.38
7.63	7.02	7.26	7.60	7.86	8.22	8.38	8.69	9.02	9.33	9.44	9.74
7.86	6.82	7.18	7.36	7.57	7.97	8.16	8.39	8.66	8.95	9.11	9.25
8.10	6.66	6.86	7.22	7.51	7.75	8.03	8.18	8.46	8.73	8.97	9.13
8.36	6.55	6.74	7.01	7.22	7.49	7.74	7.96	8.23	8.50	8.73	8.92
8.64	6.97	7.25	7.48	7.75	7.94	8.23	8.42	8.68	8.88	9.10	9.34
8.94	7.19	7.40	7.68	7.91	8.18	8.46	8.70	9.01	9.20	9.48	9.74
9.26	7.48	7.71	8.05	8.17	8.52	8.76	8.98	9.24	9.43	9.70	9.94
9.60	6.97	7.30	7.47	7.67	8.02	8.22	8.45	8.72	8.98	9.16	9.34
9.97	6.85	7.14	7.39	7.65	7.94	8.07	8.44	8.63	8.98	9.18	9.42
10.36	7.19	7.45	7.71	7.96	8.19	8.55	8.77	8.98	9.25	9.43	9.65
10.77	7.19	7.41	7.61	7.88	8.21	8.48	8.76	8.95	9.36	9.45	9.62
11.22	7.06	7.31	7.55	7.75	8.00	8.34	8.59	8.84	9.06	9.29	9.48
11.70	7.25	7.48	7.75	8.01	8.27	8.56	8.74	8.95	9.24	9.43	9.67
12.21	6.69	7.00	7.21	7.43	7.76	7.99	8.26	8.48	8.85	9.00	9.28
12.75	7.03	7.35	7.59	7.83	8.21	8.38	8.49	8.85	9.19	9.34	9.51
13.33	7.26	7.45	7.77	8.02	8.33	8.58	8.82	9.09	9.41	9.63	9.84
13.95	7.04	7.36	7.66	7.89	8.17	8.36	8.61	8.96	9.12	9.39	9.58

14.62	6.80	7.09	7.29	7.57	7.87	8.07	8.30	8.57	8.87	9.14	9.45
15.33	6.78	7.02	7.29	7.63	7.76	8.03	8.35	8.63	8.81	9.07	9.28
16.09	6.75	6.99	7.31	7.48	7.88	8.16	8.27	8.48	8.90	9.07	9.34
16.90	6.74	7.05	7.36	7.72	7.92	8.31	8.47	8.83	9.08	9.34	9.56
17.77	6.90	7.20	7.50	7.78	8.09	8.23	8.53	8.83	9.09	9.28	9.58
18.70	6.91	7.06	7.35	7.61	7.90	8.14	8.45	8.70	8.97	9.09	9.35
19.69	6.76	7.11	7.31	7.53	7.85	8.12	8.33	8.62	9.01	9.27	9.52
20.75	6.64	6.86	7.18	7.45	7.79	8.01	8.24	8.54	8.88	9.04	9.26
21.89	7.14	7.48	7.73	8.08	8.30	8.55	8.81	9.05	9.35	9.53	9.85
23.10	7.31	7.61	7.88	8.12	8.42	8.65	8.95	9.27	9.53	9.70	10.07
24.39	6.89	7.17	7.48	7.68	8.04	8.22	8.44	8.81	9.07	9.32	9.58
25.78	6.99	7.23	7.56	7.71	8.08	8.30	8.59	8.88	9.17	9.43	9.73
27.26	6.97	7.24	7.49	7.73	8.11	8.37	8.58	8.86	9.15	9.45	9.69
28.84	6.92	7.17	7.43	7.71	8.01	8.27	8.54	8.89	9.16	9.39	9.68
30.53	6.91	7.21	7.59	7.78	7.95	8.38	8.64	8.91	9.24	9.49	9.80
32.34	6.46	6.73	6.95	7.15	7.56	7.80	8.08	8.54	8.75	9.06	9.35
34.27	6.78	7.04	7.28	7.47	7.79	8.11	8.45	8.67	9.08	9.45	9.59
36.34	6.62	6.80	7.10	7.27	7.64	7.96	8.24	8.51	8.89	9.16	9.41
38.55	6.63	7.00	7.23	7.50	7.72	8.05	8.34	8.71	9.07	9.31	9.63
40.91	6.79	7.02	7.28	7.48	7.90	8.11	8.37	8.64	9.08	9.26	9.61
43.43	6.39	6.63	6.87	7.07	7.41	7.67	7.99	8.37	8.69	9.00	9.22
46.13	6.56	6.83	7.11	7.25	7.66	7.82	8.16	8.54	8.78	9.08	9.32
49.01	6.59	6.90	7.19	7.44	7.70	7.92	8.29	8.65	9.04	9.32	9.53
52.09	6.38	6.61	6.84	7.11	7.39	7.70	8.07	8.36	8.80	9.10	9.38
55.39	6.29	6.49	6.75	6.99	7.27	7.57	7.99	8.27	8.65	8.92	9.21
58.91	6.22	6.47	6.79	6.93	7.29	7.61	7.90	8.29	8.59	8.93	9.19
62.68	6.09	6.34	6.57	6.80	7.16	7.51	7.79	8.21	8.52	8.83	9.31
66.70	6.15	6.44	6.66	6.89	7.22	7.48	7.86	8.25	8.59	8.91	9.19
71.00	5.98	6.27	6.64	6.83	7.24	7.62	7.90	8.27	8.63	9.05	9.28
75.60	6.09	6.26	6.51	6.74	7.03	7.42	7.71	8.10	8.46	8.81	9.05
80.51	5.98	6.26	6.43	6.76	7.07	7.38	7.78	8.23	8.61	8.85	9.10
85.77	5.80	6.09	6.34	6.69	6.98	7.40	7.73	8.13	8.57	8.85	9.25
91.38	6.24	6.44	6.83	7.03	7.31	7.67	8.00	8.39	8.79	9.06	9.39
97.39	5.50	5.84	6.04	6.22	6.65	6.99	7.38	7.82	8.19	8.60	8.98
103.80	6.16	6.39	6.60	6.87	7.19	7.55	7.93	8.27	8.70	9.03	9.48
110.66	5.88	6.10	6.41	6.58	6.87	7.24	7.58	8.03	8.48	8.71	8.99
117.99	5.46	5.68	6.02	6.26	6.58	6.92	7.37	7.80	8.23	8.57	8.91
125.83	6.11	6.33	6.53	6.73	7.05	7.42	7.80	8.20	8.61	8.86	9.17
134.21	5.68	5.85	6.13	6.26	6.63	6.98	7.38	7.84	8.22	8.61	9.00
143.17	5.41	5.57	5.89	6.17	6.52	6.80	7.22	7.66	8.13	8.60	8.91
152.74	5.99	6.34	6.56	6.71	7.00	7.37	7.78	8.04	8.59	8.84	9.20
162.97	6.79	7.02	7.32	7.58	7.98	8.25	8.50	8.85	9.15	9.38	9.67
173.91	6.52	6.89	7.16	7.43	7.78	8.01	8.30	8.69	8.97	9.35	9.52
185.60	6.74	6.99	7.33	7.60	7.95	8.26	8.53	8.88	9.13	9.44	9.75
198.10	6.52	6.79	7.12	7.42	7.70	7.99	8.27	8.66	9.01	9.22	9.48
211.46	6.86	7.08	7.41	7.65	7.96	8.24	8.55	8.83	9.13	9.43	9.63
225.75	6.66	6.91	7.21	7.54	7.82	8.15	8.43	8.76	9.09	9.43	9.72
241.01	6.40	6.75	7.00	7.35	7.62	7.86	8.32	8.58	8.85	9.14	9.44
257.33	6.43	6.63	7.03	7.32	7.56	7.96	8.25	8.54	8.87	9.14	9.49
274.77	6.84	7.06	7.48	7.72	7.96	8.25	8.46	8.86	9.18	9.48	9.77

293.42	6.68	6.92	7.28	7.58	7.89	8.18	8.37	8.87	9.21	9.42	9.72
313.35	6.60	6.89	7.15	7.44	7.75	8.14	8.40	8.73	9.06	9.37	9.55
334.66	6.80	7.01	7.36	7.61	7.88	8.24	8.54	8.86	9.13	9.53	9.80
357.43	6.50	6.75	7.10	7.31	7.69	7.99	8.28	8.67	8.97	9.26	9.51
381.78	6.60	6.93	7.19	7.49	7.84	8.08	8.31	8.60	8.94	9.15	9.51
407.80	6.55	6.80	7.06	7.30	7.62	7.93	8.31	8.66	9.01	9.21	9.49
435.62	6.64	6.88	7.14	7.56	7.87	8.13	8.50	8.86	9.09	9.52	9.69
465.35	6.53	6.78	7.06	7.39	7.78	7.95	8.31	8.68	9.01	9.27	9.49
497.14	6.33	6.52	6.80	7.03	7.45	7.69	7.98	8.43	8.81	9.11	9.41
531.11	6.25	6.58	6.83	7.13	7.43	7.78	8.10	8.39	8.67	8.92	9.28
567.43	6.55	6.83	7.10	7.40	7.75	8.04	8.31	8.67	9.06	9.38	9.52
606.26	6.28	6.50	6.80	7.07	7.39	7.80	8.10	8.48	8.87	9.14	9.49
647.76	6.49	6.78	7.00	7.27	7.71	7.97	8.22	8.63	8.93	9.29	9.56
692.12	5.96	6.28	6.55	6.82	7.21	7.53	7.79	8.19	8.56	8.79	9.14
739.53	6.47	6.70	7.07	7.30	7.54	7.94	8.19	8.59	8.97	9.19	9.49
790.22	6.06	6.37	6.59	6.85	7.24	7.48	7.92	8.20	8.61	8.86	9.25
844.40	6.19	6.44	6.70	6.94	7.37	7.68	7.92	8.30	8.73	9.01	9.38
902.32	6.39	6.66	6.96	7.22	7.59	7.86	8.17	8.53	8.85	9.13	9.47
964.23	5.87	6.17	6.40	6.64	7.05	7.39	7.65	8.06	8.45	8.76	9.06
1030.41	6.59	6.92	7.09	7.36	7.63	7.91	8.22	8.52	8.91	9.16	9.45
1101.15	6.28	6.58	6.82	7.06	7.39	7.74	7.92	8.34	8.64	8.97	9.29
1176.77	6.40	6.64	6.97	7.25	7.64	7.89	8.18	8.48	8.85	9.12	9.38
1257.60	6.56	6.73	7.00	7.24	7.49	7.83	8.13	8.49	8.75	9.10	9.28
1344.00	6.20	6.49	6.70	6.87	7.29	7.60	7.87	8.29	8.60	8.84	9.21
1436.36	6.39	6.63	6.86	7.07	7.43	7.65	7.93	8.26	8.66	8.93	9.18
1535.09	6.38	6.67	6.80	7.00	7.34	7.70	8.00	8.34	8.71	8.97	9.25
1640.63	5.92	6.23	6.41	6.68	7.01	7.42	7.56	8.09	8.42	8.68	9.00
1753.44	6.17	6.37	6.67	6.86	7.22	7.38	7.75	8.18	8.43	8.88	9.13
1874.02	5.89	6.10	6.33	6.60	6.93	7.29	7.57	7.85	8.39	8.59	8.98
2002.92	6.15	6.40	6.60	6.87	7.20	7.41	7.79	8.10	8.48	8.86	9.06
2140.71	5.67	6.01	6.17	6.45	6.75	7.05	7.42	7.75	8.22	8.47	8.81
2288.00	5.73	6.04	6.19	6.49	6.83	7.10	7.40	7.78	8.20	8.51	8.89
2445.44	5.65	5.87	6.04	6.36	6.71	7.02	7.30	7.66	8.05	8.32	8.64
2613.73	5.78	6.07	6.35	6.60	6.84	7.25	7.53	7.91	8.32	8.62	8.90
2793.63	5.86	6.09	6.26	6.48	6.83	7.27	7.55	7.78	8.22	8.52	8.77
2985.93	5.17	5.42	5.69	5.94	6.28	6.70	7.00	7.40	7.71	8.10	8.50
3191.49	5.46	5.74	5.96	6.26	6.51	6.94	7.26	7.62	7.90	8.29	8.55
3411.21	5.34	5.58	5.82	6.00	6.40	6.76	7.07	7.54	7.87	8.16	8.46
3646.09	5.24	5.57	5.82	6.02	6.44	6.82	7.05	7.51	7.90	8.22	8.56
3897.16	5.23	5.56	5.83	5.96	6.39	6.69	6.91	7.47	7.84	8.11	8.43

Wavelength (nm) / Time (ps)	537.73	539.15	540.58	542.00	543.43	544.85	546.27	547.70	549.12	550.55	551.97
-2.74	0.00	0.00	-0.02	0.05	-0.01	-0.03	-0.02	-0.03	-0.01	0.05	0.06
-2.24	-0.07	-0.08	-0.07	0.02	0.04	-0.07	-0.07	-0.10	-0.03	-0.09	-0.06
-1.74	-0.15	-0.08	-0.07	-0.11	-0.05	-0.05	-0.07	-0.07	-0.08	-0.06	-0.04
-1.24	0.08	-0.04	0.05	-0.06	-0.09	-0.06	-0.04	0.00	0.04	0.01	-0.01
-0.74	0.11	0.08	0.04	0.07	0.08	0.11	0.13	0.03	0.07	0.06	0.06
-0.24	-0.01	-0.06	-0.07	-0.04	-0.11	0.00	-0.02	-0.02	-0.10	-0.05	-0.03

-0.14	0.01	0.09	0.10	0.00	0.02	0.03	0.07	0.08	0.05	-0.01	-0.02
-0.04	0.03	0.08	0.04	0.06	0.12	0.08	0.02	0.11	0.06	0.08	0.03
0.06	0.00	-0.11	-0.10	-0.05	-0.01	-0.09	-0.01	-0.07	-0.07	-0.01	-0.03
0.16	0.12	0.09	0.01	0.07	0.06	0.00	-0.01	-0.02	0.06	-0.04	-0.01
0.26	0.05	-0.02	0.01	0.01	0.01	-0.06	-0.04	-0.06	-0.10	-0.05	-0.11
0.31	0.00	0.03	0.01	-0.05	0.02	-0.08	-0.10	-0.06	-0.04	0.04	-0.04
0.36	-0.04	-0.12	-0.04	-0.01	0.01	-0.11	-0.12	-0.02	-0.12	-0.02	-0.04
0.41	-0.02	-0.07	-0.09	-0.10	-0.01	-0.05	-0.11	-0.06	-0.03	0.03	-0.07
0.46	0.07	0.07	0.05	0.08	0.07	0.02	0.07	0.03	-0.01	-0.03	0.03
0.51	0.15	0.05	0.07	0.03	0.04	-0.20	-0.08	-0.02	-0.02	0.07	0.00
0.56	0.03	-0.01	0.05	0.02	0.07	-0.04	0.06	-0.05	-0.01	0.03	0.10
0.61	-0.09	-0.04	-0.11	-0.03	-0.05	-0.08	0.09	-0.07	-0.03	0.00	0.00
0.66	0.03	0.02	0.01	0.04	-0.01	-0.05	-0.05	0.02	-0.05	-0.03	-0.04
0.71	0.04	0.03	-0.04	0.03	0.03	-0.04	-0.05	0.01	-0.03	0.01	-0.05
0.76	0.04	-0.02	-0.05	-0.04	0.03	-0.03	-0.10	-0.06	-0.13	-0.08	-0.04
0.81	0.04	0.12	0.07	0.07	0.10	0.03	0.06	0.06	0.00	0.07	0.07
0.86	-0.17	-0.10	-0.17	-0.10	-0.07	-0.14	-0.06	-0.04	-0.07	-0.01	0.02
0.91	0.10	0.05	0.03	0.02	0.12	0.00	-0.01	-0.06	-0.07	0.03	0.04
0.96	0.09	0.02	0.06	0.04	0.06	0.01	-0.04	0.00	-0.05	-0.06	-0.04
1.01	0.02	0.04	0.03	-0.03	0.05	0.00	-0.06	-0.03	-0.04	-0.03	-0.08
1.06	0.06	0.08	0.08	0.03	0.05	-0.05	0.01	0.01	-0.06	0.01	-0.01
1.11	-0.18	0.04	0.06	-0.13	-0.02	-0.06	-0.01	-0.02	-0.03	-0.05	0.08
1.16	0.10	0.04	0.02	0.00	0.04	-0.09	-0.07	-0.03	-0.07	-0.08	-0.04
1.21	1.15	0.51	-0.13	-0.55	-0.67	-0.73	-0.67	-0.53	-0.27	0.05	0.15
1.26	-69.20	-64.58	-58.65	-51.54	-43.69	-35.60	-27.20	-19.21	-11.80	-5.37	-0.55
1.31	195.39	183.90	171.63	160.33	147.99	133.32	117.90	100.18	82.44	64.33	46.93
1.36	15.18	44.69	75.42	108.57	141.81	173.54	203.57	229.00	251.01	267.40	279.21
1.41	12.53	11.88	10.89	9.45	6.97	3.37	-1.51	-7.81	-15.32	-23.71	-32.94
1.46	8.64	8.46	8.50	8.98	9.91	10.88	12.31	13.94	15.57	17.27	18.50
1.51	11.75	12.63	13.41	13.89	14.35	14.45	14.49	14.49	14.40	14.46	14.31
1.56	10.22	10.67	11.16	11.83	12.34	12.70	12.98	13.06	13.08	12.92	12.84
1.61	10.36	9.93	9.67	9.57	9.53	9.69	9.96	10.31	10.67	11.23	11.56
1.66	10.40	10.80	11.08	11.20	11.49	11.48	11.53	11.58	11.66	11.72	11.84
1.71	8.62	8.60	8.87	9.26	9.67	10.17	10.73	11.29	11.74	12.09	12.42
1.76	11.48	11.54	11.49	11.38	11.27	11.03	11.08	11.10	11.24	11.49	11.61
1.81	8.96	9.53	9.97	10.43	10.72	10.95	11.12	11.10	11.06	11.06	11.08
1.86	8.65	8.65	8.72	9.00	9.34	9.63	10.09	10.53	10.88	11.26	11.51
1.91	9.76	9.89	10.12	10.18	10.32	10.32	10.43	10.48	10.50	10.66	10.81
1.96	8.79	9.15	9.62	10.09	10.66	10.86	11.19	11.36	11.57	11.52	11.50
2.01	9.98	9.74	9.52	9.35	9.38	9.38	9.59	9.93	10.22	10.50	10.90
2.06	9.18	9.62	10.03	10.28	10.53	10.64	10.71	10.80	10.78	10.87	10.89
2.11	8.41	8.75	9.03	9.41	9.85	10.18	10.60	10.86	11.16	11.30	11.47
2.16	10.05	9.99	10.02	9.98	9.92	10.01	10.07	10.11	10.26	10.58	10.76
2.21	9.38	9.71	10.06	10.54	10.81	10.89	11.03	11.07	11.11	11.14	11.22
2.26	9.02	9.02	9.05	9.28	9.49	9.93	10.29	10.58	10.97	11.25	11.47
2.31	9.49	9.72	10.05	10.08	10.23	10.38	10.33	10.45	10.52	10.61	10.80
2.36	8.87	9.15	9.48	9.84	10.14	10.34	10.45	10.56	10.70	10.77	10.99
2.41	9.06	9.03	8.98	9.22	9.30	9.53	9.78	10.06	10.39	10.60	10.88
2.46	8.96	9.36	9.58	9.81	10.07	10.22	10.26	10.36	10.44	10.44	10.62
2.51	8.80	8.95	9.17	9.55	9.93	10.26	10.69	11.03	11.25	11.57	11.64

2.56	9.85	9.89	9.89	9.88	10.01	10.06	10.32	10.34	10.62	10.77	10.93
2.61	9.09	9.36	9.62	9.76	9.97	10.12	10.31	10.27	10.45	10.53	10.61
2.66	8.97	9.12	9.39	9.53	9.91	10.14	10.44	10.76	10.91	11.16	11.31
2.71	9.51	9.51	9.75	9.72	9.94	9.87	10.04	10.16	10.26	10.42	10.65
2.76	9.09	9.48	9.71	10.02	10.34	10.52	10.78	10.92	11.01	11.13	11.23
2.81	8.86	8.85	9.09	9.17	9.45	9.65	9.88	10.19	10.46	10.73	10.88
2.86	9.04	9.26	9.35	9.57	9.73	9.82	9.93	10.04	10.15	10.31	10.47
2.91	8.84	9.12	9.34	9.69	9.98	10.19	10.48	10.67	10.76	10.95	11.08
2.96	9.42	9.54	9.53	9.74	9.92	10.01	10.17	10.44	10.64	10.83	11.08
3.01	9.13	9.44	9.70	10.11	10.25	10.48	10.67	10.81	10.86	11.04	11.15
3.06	8.64	8.83	9.01	9.24	9.51	9.78	9.98	10.18	10.41	10.57	10.71
3.11	9.28	9.44	9.56	9.71	9.91	9.99	10.14	10.32	10.45	10.69	10.88
3.16	9.03	9.28	9.54	9.79	10.04	10.22	10.40	10.60	10.75	10.90	11.00
3.21	9.07	9.12	9.45	9.58	9.79	10.01	10.26	10.46	10.67	10.73	10.94
3.26	9.23	9.48	9.74	9.81	10.00	10.11	10.20	10.38	10.58	10.72	10.82
3.31	8.91	9.22	9.47	9.74	9.98	10.22	10.37	10.61	10.68	10.84	10.98
3.36	9.42	9.51	9.72	9.80	10.10	10.22	10.37	10.57	10.84	11.00	11.22
3.41	9.36	9.62	9.80	10.06	10.24	10.34	10.52	10.62	10.75	10.88	11.04
3.46	9.03	9.26	9.48	9.74	9.97	10.19	10.38	10.63	10.80	10.91	11.07
3.51	9.33	9.58	9.77	9.93	10.10	10.23	10.37	10.51	10.73	10.94	10.97
3.56	9.05	9.30	9.57	9.82	10.04	10.18	10.41	10.51	10.64	10.78	10.98
3.61	8.70	8.88	9.08	9.30	9.62	9.75	9.95	10.23	10.40	10.55	10.74
3.66	9.08	9.20	9.41	9.56	9.71	9.81	10.06	10.17	10.25	10.42	10.57
3.71	9.22	9.38	9.68	9.91	10.25	10.42	10.60	10.82	10.89	11.13	11.22
3.76	9.62	9.84	9.96	10.20	10.39	10.62	10.71	10.93	11.10	11.35	11.46
3.81	8.96	9.17	9.41	9.52	9.76	9.95	10.05	10.16	10.32	10.55	10.64
3.86	8.68	8.83	9.05	9.33	9.64	9.82	9.95	10.16	10.34	10.50	10.68
3.91	9.50	9.62	9.79	9.96	10.19	10.28	10.49	10.64	10.86	10.94	11.13
3.96	9.15	9.50	9.74	9.90	10.17	10.29	10.44	10.65	10.79	10.91	10.99
4.01	9.43	9.52	9.71	9.87	10.21	10.35	10.55	10.84	10.97	11.17	11.25
4.06	9.48	9.63	9.86	10.04	10.31	10.29	10.54	10.66	10.83	11.04	11.11
4.11	9.11	9.28	9.49	9.80	10.10	10.26	10.41	10.55	10.72	10.88	10.96
4.16	9.47	9.57	9.79	9.94	10.13	10.27	10.46	10.69	10.88	11.06	11.13
4.21	9.72	9.91	10.18	10.39	10.59	10.74	10.90	10.98	11.20	11.33	11.37
4.26	9.17	9.39	9.66	9.87	10.08	10.32	10.50	10.65	10.83	10.88	11.14
4.31	9.12	9.23	9.41	9.70	9.97	9.98	10.19	10.36	10.48	10.77	10.88
4.36	9.17	9.35	9.58	9.72	10.05	10.17	10.35	10.56	10.73	10.93	10.90
4.41	9.03	9.15	9.38	9.63	9.86	9.97	10.11	10.36	10.58	10.72	10.91
4.46	9.71	9.83	10.00	10.21	10.43	10.51	10.63	10.79	10.98	11.22	11.40
4.51	9.23	9.46	9.72	9.94	10.19	10.38	10.59	10.73	10.94	11.04	11.25
4.56	9.08	9.25	9.42	9.63	9.84	10.04	10.28	10.47	10.61	10.85	10.95
4.61	8.92	9.10	9.31	9.46	9.69	9.93	10.11	10.25	10.42	10.62	10.71
4.66	9.25	9.48	9.64	9.92	10.30	10.31	10.55	10.70	10.93	11.06	11.23
4.71	9.39	9.56	9.74	9.87	10.16	10.29	10.42	10.62	10.87	10.94	11.17
4.76	9.58	9.87	10.06	10.29	10.49	10.67	10.82	10.97	11.12	11.29	11.46
4.81	9.09	9.30	9.53	9.80	10.00	10.23	10.38	10.57	10.76	10.95	11.11
4.86	9.10	9.25	9.44	9.63	9.80	10.01	10.17	10.37	10.52	10.70	10.93
4.91	9.54	9.66	9.96	10.19	10.47	10.58	10.72	10.97	11.04	11.27	11.36
4.96	9.16	9.40	9.60	9.76	10.03	10.19	10.37	10.61	10.69	10.91	11.01
5.01	9.37	9.52	9.72	9.93	10.19	10.29	10.45	10.66	10.81	10.95	11.06

5.06	9.15	9.38	9.65	9.91	10.08	10.36	10.52	10.66	10.81	11.04	11.24
5.11	9.14	9.33	9.49	9.71	9.85	10.04	10.26	10.46	10.57	10.76	11.03
5.16	9.14	9.34	9.50	9.71	9.97	10.06	10.29	10.49	10.63	10.77	10.99
5.21	9.09	9.32	9.50	9.72	10.01	10.26	10.33	10.60	10.72	10.95	11.07
5.26	9.49	9.76	9.95	10.12	10.36	10.45	10.73	10.87	11.09	11.16	11.34
5.31	9.26	9.60	9.76	9.94	10.27	10.41	10.53	10.73	10.85	11.04	11.14
5.38	9.19	9.45	9.57	9.91	10.14	10.29	10.41	10.64	10.77	11.00	11.04
5.45	9.59	9.83	9.95	10.23	10.44	10.69	10.82	11.07	11.07	11.27	11.42
5.53	9.27	9.49	9.72	9.96	10.22	10.32	10.56	10.73	10.95	11.03	11.12
5.61	9.51	9.80	10.00	10.20	10.51	10.62	10.79	10.99	11.22	11.28	11.46
5.70	9.36	9.53	9.83	10.04	10.33	10.47	10.69	10.84	11.00	11.17	11.37
5.80	9.46	9.74	9.92	10.11	10.38	10.57	10.74	10.98	11.18	11.31	11.48
5.90	9.20	9.37	9.64	9.76	10.06	10.15	10.30	10.56	10.68	10.87	10.89
6.01	9.69	9.95	10.17	10.36	10.57	10.72	10.92	11.15	11.33	11.49	11.65
6.13	9.54	9.73	9.93	10.05	10.29	10.50	10.67	10.79	10.96	11.12	11.31
6.25	9.14	9.31	9.54	9.73	10.09	10.21	10.38	10.49	10.68	10.87	10.98
6.39	9.40	9.52	9.77	9.94	10.25	10.28	10.49	10.69	10.80	10.98	11.17
6.53	9.38	9.64	9.76	9.98	10.17	10.35	10.57	10.80	10.91	11.09	11.19
6.69	9.68	9.82	10.03	10.29	10.52	10.61	10.80	11.00	11.09	11.24	11.46
6.85	9.37	9.53	9.74	9.87	10.23	10.36	10.58	10.75	10.84	11.01	11.21
7.03	9.47	9.69	9.95	10.08	10.47	10.55	10.69	10.79	11.03	11.18	11.32
7.21	9.50	9.76	9.92	10.15	10.39	10.53	10.71	10.98	11.00	11.19	11.33
7.41	9.65	9.88	9.91	10.14	10.50	10.66	10.74	10.92	11.21	11.34	11.46
7.63	9.97	10.11	10.34	10.55	10.80	10.97	11.23	11.39	11.51	11.63	11.81
7.86	9.50	9.76	9.97	10.14	10.43	10.57	10.72	10.85	11.09	11.20	11.25
8.10	9.42	9.63	9.92	10.09	10.34	10.43	10.69	10.89	11.05	11.19	11.26
8.36	9.12	9.26	9.55	9.79	9.99	10.17	10.32	10.52	10.69	10.81	11.01
8.64	9.57	9.76	9.94	10.15	10.43	10.54	10.78	10.80	11.05	11.28	11.29
8.94	9.99	10.13	10.38	10.54	10.68	10.89	10.99	11.29	11.44	11.57	11.66
9.26	10.12	10.32	10.54	10.72	11.02	11.13	11.24	11.52	11.69	11.87	11.92
9.60	9.73	9.91	10.16	10.29	10.54	10.66	10.88	11.03	11.19	11.36	11.55
9.97	9.68	9.74	10.06	10.25	10.55	10.66	10.78	11.11	11.17	11.41	11.52
10.36	9.85	10.01	10.36	10.50	10.75	10.93	11.02	11.20	11.33	11.51	11.69
10.77	9.98	10.08	10.22	10.49	10.74	10.97	11.12	11.35	11.39	11.62	11.79
11.22	9.80	9.98	10.24	10.39	10.60	10.71	10.84	11.05	11.28	11.43	11.50
11.70	9.87	10.06	10.26	10.54	10.72	10.92	11.02	11.18	11.40	11.53	11.67
12.21	9.52	9.66	9.91	10.13	10.35	10.50	10.73	10.78	10.96	11.17	11.37
12.75	9.76	10.08	10.23	10.44	10.74	10.85	11.00	11.15	11.36	11.57	11.64
13.33	10.03	10.25	10.46	10.71	10.94	11.04	11.25	11.43	11.63	11.65	11.86
13.95	9.84	10.07	10.24	10.36	10.75	10.78	11.02	11.25	11.32	11.47	11.64
14.62	9.50	9.74	9.95	10.20	10.41	10.52	10.81	11.00	11.06	11.27	11.46
15.33	9.45	9.68	9.87	10.15	10.41	10.53	10.70	10.96	11.05	11.20	11.42
16.09	9.54	9.81	10.01	10.18	10.45	10.63	10.84	10.99	11.14	11.32	11.45
16.90	9.81	10.04	10.34	10.52	10.69	10.92	11.08	11.24	11.40	11.59	11.71
17.77	9.79	10.00	10.28	10.55	10.72	10.81	10.99	11.05	11.28	11.54	11.64
18.70	9.67	9.83	10.00	10.31	10.49	10.54	10.87	11.06	11.13	11.42	11.55
19.69	9.71	9.93	10.18	10.49	10.64	10.80	11.05	11.26	11.36	11.48	11.70
20.75	9.56	9.81	10.04	10.29	10.52	10.68	10.79	11.03	11.14	11.33	11.53
21.89	10.08	10.33	10.53	10.73	10.94	11.18	11.28	11.47	11.65	11.82	11.97
23.10	10.33	10.46	10.63	10.93	11.18	11.21	11.42	11.69	11.88	12.04	12.07

24.39	9.83	10.04	10.28	10.46	10.71	10.84	11.00	11.22	11.35	11.50	11.60
25.78	9.89	10.22	10.47	10.61	10.95	11.05	11.32	11.42	11.59	11.86	11.87
27.26	9.94	10.13	10.34	10.57	10.83	10.95	11.12	11.37	11.48	11.67	11.76
28.84	9.87	10.13	10.31	10.52	10.73	10.94	11.08	11.29	11.50	11.67	11.80
30.53	10.02	10.25	10.49	10.64	10.95	11.15	11.21	11.42	11.60	11.83	11.99
32.34	9.62	9.81	10.13	10.29	10.56	10.70	11.03	11.11	11.21	11.49	11.62
34.27	9.92	10.22	10.37	10.61	10.93	11.04	11.22	11.44	11.66	11.74	11.95
36.34	9.69	9.92	10.14	10.31	10.56	10.72	11.04	11.13	11.33	11.52	11.68
38.55	9.96	10.12	10.33	10.66	10.87	11.07	11.22	11.48	11.58	11.74	11.98
40.91	9.88	10.17	10.29	10.54	10.82	11.00	11.12	11.32	11.48	11.59	11.82
43.43	9.55	9.81	9.97	10.26	10.54	10.66	10.83	11.01	11.24	11.40	11.48
46.13	9.63	9.87	10.11	10.36	10.56	10.74	10.85	11.09	11.28	11.38	11.60
49.01	9.89	10.10	10.39	10.66	10.87	11.05	11.25	11.45	11.56	11.85	11.93
52.09	9.67	9.86	10.21	10.42	10.67	10.83	11.06	11.24	11.38	11.58	11.75
55.39	9.50	9.80	10.03	10.31	10.53	10.72	10.92	11.16	11.30	11.51	11.69
58.91	9.52	9.81	10.06	10.28	10.57	10.76	10.94	11.06	11.31	11.45	11.65
62.68	9.59	9.78	10.06	10.32	10.59	10.80	10.97	11.14	11.36	11.61	11.72
66.70	9.63	9.79	10.11	10.27	10.58	10.76	10.94	11.22	11.37	11.54	11.70
71.00	9.53	9.95	10.10	10.31	10.69	10.74	11.04	11.19	11.40	11.64	11.83
75.60	9.37	9.68	9.91	10.10	10.39	10.59	10.71	10.95	11.11	11.39	11.54
80.51	9.50	9.78	10.06	10.28	10.62	10.76	10.95	11.04	11.35	11.57	11.70
85.77	9.60	9.87	10.27	10.55	10.75	10.98	11.18	11.47	11.62	11.77	11.95
91.38	9.63	9.89	10.19	10.43	10.67	10.84	11.09	11.22	11.39	11.61	11.83
97.39	9.39	9.60	9.90	10.24	10.52	10.80	10.93	11.20	11.32	11.51	11.76
103.80	9.67	9.99	10.25	10.51	10.91	11.08	11.21	11.46	11.72	11.91	12.03
110.66	9.35	9.66	9.96	10.21	10.48	10.70	10.98	11.07	11.38	11.55	11.65
117.99	9.37	9.62	9.99	10.19	10.48	10.72	10.88	11.08	11.34	11.64	11.75
125.83	9.62	9.91	10.22	10.41	10.76	10.94	11.17	11.36	11.50	11.85	11.99
134.21	9.42	9.62	10.05	10.27	10.63	10.78	11.01	11.23	11.44	11.64	11.83
143.17	9.27	9.52	9.91	10.12	10.47	10.72	10.93	11.11	11.35	11.65	11.72
152.74	9.53	9.78	10.16	10.41	10.68	10.85	11.09	11.26	11.43	11.68	11.78
162.97	9.96	10.20	10.33	10.68	10.90	11.12	11.24	11.58	11.64	11.92	12.08
173.91	9.78	10.04	10.28	10.54	10.79	10.99	11.20	11.41	11.59	11.77	11.89
185.60	10.00	10.28	10.46	10.76	11.01	11.22	11.40	11.53	11.82	11.91	12.07
198.10	9.75	10.01	10.27	10.56	10.82	11.05	11.15	11.46	11.65	11.78	12.02
211.46	9.97	10.22	10.35	10.71	10.91	11.07	11.27	11.45	11.72	11.94	12.08
225.75	9.91	10.09	10.51	10.72	11.00	11.14	11.38	11.59	11.80	11.92	12.10
241.01	9.75	10.04	10.14	10.39	10.74	10.90	11.18	11.42	11.60	11.76	11.95
257.33	9.85	10.05	10.32	10.54	10.79	11.05	11.32	11.45	11.69	11.88	12.04
274.77	9.96	10.28	10.53	10.72	11.06	11.18	11.39	11.69	11.85	11.98	12.26
293.42	10.06	10.29	10.47	10.71	11.03	11.19	11.40	11.61	11.90	12.05	12.20
313.35	9.82	10.13	10.49	10.69	10.92	11.11	11.30	11.52	11.73	11.91	12.04
334.66	10.00	10.27	10.50	10.80	11.11	11.23	11.49	11.73	11.90	12.12	12.19
357.43	9.85	10.13	10.36	10.62	10.97	11.13	11.35	11.58	11.82	12.06	12.24
381.78	9.80	10.00	10.27	10.48	10.78	10.97	11.15	11.30	11.55	11.78	11.94
407.80	9.74	10.03	10.22	10.50	10.79	10.95	11.30	11.44	11.63	11.80	11.98
435.62	10.08	10.27	10.61	10.74	11.06	11.31	11.61	11.75	11.98	12.10	12.38
465.35	9.83	10.09	10.39	10.61	10.92	11.00	11.28	11.45	11.66	11.84	12.11
497.14	9.71	10.04	10.24	10.58	10.79	10.98	11.25	11.45	11.68	11.87	12.07
531.11	9.64	9.86	10.07	10.38	10.67	10.83	11.07	11.27	11.48	11.70	11.86

567.43	9.93	10.16	10.41	10.68	10.99	11.19	11.47	11.64	11.80	12.07	12.20
606.26	9.72	10.04	10.35	10.58	10.83	11.05	11.28	11.53	11.74	11.96	12.15
647.76	9.89	10.10	10.38	10.59	10.94	11.05	11.25	11.43	11.77	11.92	12.10
692.12	9.44	9.68	9.98	10.19	10.44	10.70	10.92	11.14	11.28	11.61	11.66
739.53	9.76	10.04	10.25	10.52	10.91	11.06	11.35	11.54	11.66	11.86	12.00
790.22	9.63	9.85	10.03	10.39	10.71	10.88	11.15	11.31	11.50	11.76	11.94
844.40	9.65	9.87	10.17	10.41	10.71	11.02	11.13	11.43	11.56	11.87	11.94
902.32	9.71	10.09	10.33	10.51	10.89	11.09	11.28	11.47	11.71	12.00	12.09
964.23	9.46	9.67	9.95	10.17	10.54	10.66	10.94	11.28	11.48	11.56	11.76
1030.41	9.72	9.94	10.24	10.55	10.82	10.91	11.21	11.45	11.61	11.81	11.91
1101.15	9.64	9.88	10.11	10.39	10.70	10.80	11.15	11.30	11.50	11.74	11.83
1176.77	9.64	9.92	10.18	10.42	10.72	10.96	11.14	11.31	11.56	11.77	11.91
1257.60	9.65	9.95	10.17	10.46	10.78	10.91	11.16	11.32	11.55	11.72	11.96
1344.00	9.49	9.81	10.16	10.41	10.61	10.87	11.15	11.24	11.56	11.68	11.81
1436.36	9.54	9.74	10.01	10.22	10.55	10.70	10.99	11.11	11.37	11.61	11.69
1535.09	9.60	9.91	10.19	10.43	10.75	10.97	11.16	11.34	11.63	11.81	11.94
1640.63	9.32	9.57	9.88	10.22	10.50	10.73	10.94	11.16	11.46	11.62	11.78
1753.44	9.43	9.68	9.96	10.25	10.54	10.69	10.96	11.13	11.38	11.57	11.77
1874.02	9.33	9.52	9.75	10.13	10.32	10.68	10.91	11.08	11.28	11.57	11.66
2002.92	9.46	9.62	9.85	10.20	10.48	10.70	10.92	11.02	11.23	11.51	11.71
2140.71	9.09	9.32	9.70	9.91	10.21	10.43	10.72	10.94	11.02	11.23	11.40
2288.00	9.14	9.44	9.77	10.07	10.38	10.55	10.78	10.98	11.09	11.48	11.61
2445.44	9.03	9.35	9.62	9.96	10.25	10.42	10.58	10.89	11.10	11.28	11.42
2613.73	9.28	9.53	9.78	10.15	10.51	10.66	10.92	11.16	11.26	11.40	11.60
2793.63	9.18	9.37	9.62	9.88	10.27	10.44	10.62	10.87	11.05	11.25	11.39
2985.93	8.80	9.10	9.33	9.65	9.98	10.19	10.41	10.64	10.83	11.12	11.26
3191.49	8.88	9.18	9.43	9.72	9.98	10.30	10.46	10.61	10.85	11.00	11.18
3411.21	8.81	9.10	9.32	9.62	9.94	10.20	10.40	10.53	10.74	10.86	11.12
3646.09	8.85	9.19	9.33	9.65	9.90	10.24	10.38	10.57	10.81	10.82	11.04
3897.16	8.75	9.04	9.34	9.57	9.82	10.06	10.29	10.46	10.64	10.87	10.93

Wavelength (nm) / Time (ps)	553.39	554.82	556.24	557.67	559.09	560.51	561.94	563.36	564.79	566.21	567.63
-2.74	0.04	0.07	0.00	0.04	0.00	-0.07	0.01	0.03	0.10	0.12	0.04
-2.24	-0.11	-0.02	0.00	-0.05	0.00	-0.04	-0.04	-0.06	-0.02	-0.10	-0.12
-1.74	0.00	-0.02	-0.06	-0.05	0.00	-0.04	-0.08	0.00	-0.01	-0.07	-0.03
-1.24	0.00	-0.07	-0.03	0.04	-0.02	-0.01	-0.01	0.03	-0.06	-0.03	-0.05
-0.74	0.09	0.01	0.00	0.03	0.03	0.08	-0.03	-0.03	0.03	0.07	-0.03
-0.24	-0.04	-0.04	-0.02	-0.01	-0.04	0.00	-0.01	0.00	-0.07	-0.02	0.02
-0.14	0.05	0.00	0.03	-0.03	-0.05	0.08	0.03	-0.01	-0.02	-0.01	0.07
-0.04	-0.03	0.07	0.09	0.03	0.07	0.01	0.13	0.05	0.05	0.03	0.09
0.06	-0.07	-0.05	-0.03	-0.03	-0.06	-0.06	-0.01	-0.09	-0.03	-0.06	-0.07
0.16	-0.09	-0.11	0.05	-0.04	0.01	-0.09	-0.06	0.05	-0.07	0.00	-0.01
0.26	0.07	-0.01	-0.03	-0.12	-0.08	-0.04	-0.01	-0.08	-0.02	-0.09	-0.03
0.31	0.03	-0.03	-0.13	-0.08	0.03	0.00	0.01	-0.04	0.11	-0.03	-0.08
0.36	0.05	-0.06	-0.01	-0.06	-0.04	-0.03	0.07	-0.01	0.03	0.01	-0.03
0.41	-0.02	0.04	-0.07	-0.04	-0.07	0.08	-0.08	-0.05	-0.03	-0.02	-0.08
0.46	-0.02	0.01	0.02	0.03	0.00	0.04	0.06	0.03	0.06	-0.03	0.06
0.51	0.00	0.03	0.00	-0.03	-0.07	0.04	0.01	0.04	0.06	-0.02	0.06

0.56	0.02	0.04	0.03	0.02	0.04	0.10	-0.01	-0.06	0.04	-0.03	0.04
0.61	-0.04	0.08	0.02	-0.01	-0.03	-0.05	-0.03	-0.06	0.09	-0.02	-0.06
0.66	0.03	0.00	-0.05	0.01	0.04	0.03	0.00	-0.02	0.03	-0.02	-0.05
0.71	0.01	-0.05	0.02	0.07	-0.02	0.04	0.01	-0.01	0.06	0.01	0.06
0.76	-0.13	-0.06	-0.07	-0.03	-0.07	-0.06	0.00	-0.12	-0.07	-0.12	-0.05
0.81	-0.01	0.01	0.00	0.04	0.10	-0.01	-0.02	0.01	0.06	-0.01	0.00
0.86	-0.03	0.04	0.02	-0.01	-0.03	-0.02	0.00	-0.02	0.08	-0.08	-0.07
0.91	-0.01	0.08	-0.03	-0.01	-0.01	-0.02	-0.05	0.00	0.05	-0.02	0.03
0.96	-0.02	0.09	-0.01	-0.03	0.02	-0.01	0.00	-0.04	0.06	-0.09	0.03
1.01	-0.04	-0.03	-0.11	-0.01	-0.09	-0.11	-0.09	-0.09	-0.01	0.00	-0.07
1.06	-0.06	0.01	-0.11	0.06	0.04	0.03	0.01	-0.04	0.06	0.09	-0.03
1.11	0.13	-0.03	-0.07	0.00	0.01	-0.02	-0.01	-0.01	0.03	-0.02	0.07
1.16	-0.04	0.00	-0.04	-0.24	0.00	0.07	-0.04	-0.02	-0.05	0.07	-0.06
1.21	0.29	0.18	0.14	0.09	0.14	0.12	-0.08	-0.19	-0.11	-0.07	0.02
1.26	2.39	3.87	3.76	2.59	0.92	-0.79	-2.33	-3.36	-3.73	-3.60	-2.98
1.31	30.53	15.54	1.66	-10.68	-21.68	-31.85	-41.46	-50.20	-58.03	-64.57	-70.03
1.36	286.18	288.98	288.48	285.47	281.19	274.42	265.73	256.01	245.12	232.48	219.00
1.41	-42.50	-51.99	-61.39	-70.54	-78.84	-85.73	-91.05	-94.61	-96.27	-95.31	-92.91
1.46	19.68	20.58	21.11	21.48	21.62	21.90	21.95	21.94	21.96	21.95	21.66
1.51	14.31	14.33	14.34	14.26	14.17	14.30	14.27	14.16	14.09	13.96	13.95
1.56	12.71	12.60	12.50	12.59	12.54	12.65	12.71	12.95	13.13	13.28	13.57
1.61	12.04	12.33	12.64	12.90	13.13	13.52	13.68	13.90	14.06	14.15	14.24
1.66	12.13	12.30	12.58	12.85	12.97	13.23	13.41	13.55	13.66	13.75	13.84
1.71	12.53	12.71	12.80	12.90	12.83	12.91	12.79	12.67	12.70	12.66	12.71
1.76	11.76	12.03	12.21	12.40	12.63	12.75	12.85	12.94	13.01	13.02	13.07
1.81	11.13	11.19	11.27	11.29	11.36	11.58	11.68	11.80	12.04	12.18	12.40
1.86	11.75	11.94	12.08	12.21	12.33	12.47	12.51	12.56	12.62	12.60	12.69
1.91	11.10	11.32	11.42	11.51	11.74	11.99	12.02	12.09	12.27	12.28	12.34
1.96	11.53	11.50	11.41	11.43	11.45	11.52	11.56	11.56	11.69	11.78	11.87
2.01	11.26	11.46	11.67	11.89	12.04	12.14	12.20	12.29	12.36	12.39	12.46
2.06	11.05	11.13	11.23	11.50	11.61	11.80	12.00	12.08	12.28	12.35	12.38
2.11	11.68	11.82	11.89	11.92	11.91	12.02	12.05	12.03	12.05	12.12	12.14
2.16	10.90	11.09	11.30	11.50	11.59	11.83	11.85	11.94	12.05	12.13	12.15
2.21	11.22	11.37	11.37	11.46	11.64	11.83	12.00	12.06	12.35	12.36	12.58
2.26	11.78	12.04	12.11	12.19	12.21	12.30	12.36	12.42	12.38	12.32	12.37
2.31	10.96	11.09	11.19	11.37	11.50	11.65	11.73	11.89	11.99	11.99	12.11
2.36	11.01	11.06	11.17	11.26	11.26	11.35	11.44	11.54	11.67	11.76	11.87
2.41	11.06	11.36	11.49	11.56	11.71	11.85	11.96	12.00	12.06	12.12	12.14
2.46	10.66	10.90	10.94	11.17	11.21	11.44	11.52	11.66	11.79	11.88	11.87
2.51	11.80	11.87	11.90	11.90	11.93	11.97	12.07	11.95	12.07	12.05	12.14
2.56	11.29	11.36	11.53	11.70	11.86	11.88	11.95	12.14	12.22	12.24	12.33
2.61	10.79	10.89	10.96	11.19	11.27	11.38	11.46	11.53	11.66	11.71	11.83
2.66	11.46	11.62	11.68	11.73	11.82	11.92	11.93	12.01	12.12	12.01	12.03
2.71	10.87	11.03	11.17	11.42	11.48	11.53	11.66	11.84	11.79	11.80	11.88
2.76	11.29	11.38	11.41	11.53	11.63	11.75	11.87	11.96	11.97	12.09	12.23
2.81	11.03	11.23	11.36	11.41	11.45	11.69	11.67	11.70	11.83	11.77	11.78
2.86	10.67	10.84	10.86	11.10	11.19	11.32	11.41	11.52	11.61	11.70	11.74
2.91	11.26	11.29	11.42	11.51	11.57	11.65	11.73	11.81	11.88	11.94	11.88
2.96	11.33	11.47	11.62	11.73	11.91	12.00	11.94	12.11	12.22	12.21	12.30
3.01	11.22	11.33	11.41	11.52	11.63	11.74	11.88	11.91	12.05	12.09	12.21

3.06	10.95	11.07	11.15	11.26	11.40	11.44	11.37	11.54	11.55	11.56	11.63
3.11	10.96	11.13	11.23	11.43	11.51	11.69	11.73	11.73	11.94	11.95	12.02
3.16	11.05	11.20	11.24	11.42	11.39	11.68	11.67	11.72	11.80	11.86	11.98
3.21	11.15	11.31	11.40	11.39	11.54	11.61	11.70	11.70	11.79	11.82	11.94
3.26	10.92	11.06	11.19	11.33	11.44	11.55	11.67	11.69	11.85	11.86	11.94
3.31	11.08	11.19	11.29	11.43	11.45	11.63	11.66	11.76	11.76	11.81	11.87
3.36	11.33	11.50	11.59	11.72	11.82	11.94	12.02	12.03	12.14	12.21	12.20
3.41	11.12	11.28	11.40	11.48	11.68	11.79	11.80	11.90	12.05	12.12	12.19
3.46	11.22	11.21	11.35	11.48	11.56	11.64	11.76	11.77	11.86	11.73	11.87
3.51	11.12	11.31	11.40	11.53	11.74	11.82	11.94	12.05	12.05	12.08	12.17
3.56	11.09	11.11	11.18	11.31	11.37	11.47	11.52	11.61	11.73	11.74	11.84
3.61	10.98	10.97	11.04	11.17	11.33	11.35	11.43	11.39	11.62	11.57	11.64
3.66	10.84	10.88	11.02	11.11	11.23	11.40	11.56	11.56	11.62	11.69	11.61
3.71	11.41	11.60	11.58	11.62	11.78	11.83	11.99	11.94	12.05	12.00	12.13
3.76	11.60	11.76	11.88	11.94	12.11	12.17	12.27	12.34	12.38	12.36	12.49
3.81	10.77	10.94	11.02	11.17	11.18	11.42	11.54	11.59	11.58	11.65	11.76
3.86	10.85	10.94	11.06	11.11	11.18	11.33	11.33	11.41	11.45	11.50	11.59
3.91	11.36	11.52	11.65	11.67	11.85	11.87	11.91	12.10	12.12	12.24	12.28
3.96	11.20	11.32	11.39	11.55	11.58	11.73	11.84	11.87	11.97	11.97	12.05
4.01	11.40	11.54	11.64	11.76	11.79	11.88	11.99	12.10	12.10	12.16	12.14
4.06	11.30	11.47	11.60	11.65	11.78	11.91	11.98	12.02	12.18	12.06	12.24
4.11	11.15	11.33	11.36	11.49	11.61	11.71	11.77	11.84	11.98	12.03	12.04
4.16	11.34	11.47	11.64	11.67	11.85	11.90	11.94	12.03	12.08	12.15	12.19
4.21	11.62	11.81	11.86	12.11	12.12	12.25	12.28	12.37	12.52	12.43	12.48
4.26	11.31	11.42	11.48	11.57	11.63	11.74	11.84	11.84	11.96	11.97	12.00
4.31	11.03	11.17	11.38	11.49	11.59	11.64	11.69	11.77	11.86	11.97	11.97
4.36	11.13	11.26	11.43	11.52	11.64	11.70	11.77	11.90	11.91	11.91	12.02
4.41	11.03	11.13	11.31	11.44	11.47	11.57	11.64	11.67	11.73	11.82	11.92
4.46	11.45	11.64	11.79	11.88	11.99	12.15	12.12	12.23	12.30	12.35	12.38
4.51	11.36	11.41	11.59	11.67	11.69	11.87	11.92	11.99	12.05	12.10	12.19
4.56	11.07	11.27	11.35	11.42	11.52	11.61	11.68	11.79	11.77	11.91	11.90
4.61	10.91	11.03	11.16	11.30	11.37	11.50	11.60	11.61	11.73	11.70	11.80
4.66	11.30	11.50	11.62	11.68	11.76	11.93	11.94	11.94	12.08	12.08	12.18
4.71	11.36	11.48	11.57	11.69	11.85	11.90	11.95	12.03	12.06	12.03	12.17
4.76	11.62	11.72	11.84	11.94	12.01	12.18	12.21	12.37	12.43	12.43	12.46
4.81	11.17	11.30	11.41	11.56	11.60	11.67	11.77	11.77	11.98	11.88	11.96
4.86	10.99	11.20	11.30	11.43	11.54	11.59	11.69	11.74	11.82	11.85	11.93
4.91	11.55	11.69	11.72	11.81	11.94	12.06	12.10	12.12	12.33	12.28	12.35
4.96	11.26	11.36	11.48	11.63	11.68	11.76	11.85	11.92	12.03	11.98	12.09
5.01	11.20	11.33	11.43	11.57	11.66	11.79	11.85	11.93	12.04	12.09	12.08
5.06	11.35	11.43	11.60	11.65	11.79	11.81	11.90	12.00	12.10	12.11	12.11
5.11	11.18	11.23	11.41	11.49	11.57	11.65	11.77	11.76	11.85	11.89	11.88
5.16	10.99	11.17	11.33	11.40	11.47	11.66	11.64	11.79	11.79	11.75	11.92
5.21	11.16	11.34	11.45	11.59	11.64	11.75	11.88	11.93	12.04	12.03	12.10
5.26	11.58	11.68	11.66	11.88	11.90	12.06	12.12	12.29	12.28	12.27	12.35
5.31	11.34	11.46	11.57	11.70	11.75	11.86	11.98	12.01	12.09	12.12	12.16
5.38	11.19	11.40	11.49	11.57	11.69	11.85	11.92	11.92	11.96	12.04	12.07
5.45	11.58	11.74	11.86	11.97	12.00	12.14	12.22	12.32	12.30	12.45	12.43
5.53	11.41	11.45	11.57	11.67	11.87	11.88	11.99	12.03	12.09	12.12	12.18
5.61	11.58	11.78	11.85	12.00	12.10	12.13	12.30	12.29	12.44	12.45	12.48

5.70	11.43	11.63	11.67	11.82	11.89	12.00	12.05	12.12	12.26	12.22	12.33
5.80	11.57	11.78	11.88	12.01	12.07	12.20	12.26	12.32	12.40	12.40	12.46
5.90	11.16	11.32	11.39	11.52	11.63	11.62	11.74	11.78	11.90	11.89	11.94
6.01	11.77	11.97	12.01	12.13	12.27	12.35	12.37	12.46	12.53	12.48	12.52
6.13	11.43	11.56	11.66	11.77	11.89	11.95	12.08	12.10	12.22	12.13	12.28
6.25	11.15	11.27	11.47	11.44	11.56	11.67	11.77	11.88	11.93	11.97	11.91
6.39	11.26	11.39	11.51	11.57	11.76	11.80	11.95	11.94	12.02	12.00	12.01
6.53	11.31	11.54	11.59	11.64	11.74	11.84	11.91	12.04	12.09	12.06	12.11
6.69	11.55	11.73	11.80	11.86	11.96	12.05	12.23	12.17	12.27	12.24	12.40
6.85	11.37	11.43	11.53	11.73	11.80	11.88	11.87	11.99	12.03	12.08	12.15
7.03	11.54	11.60	11.72	11.83	11.87	11.99	12.05	12.15	12.24	12.28	12.20
7.21	11.50	11.69	11.77	11.86	11.87	12.00	12.13	12.21	12.19	12.22	12.27
7.41	11.60	11.68	11.82	11.92	11.95	12.05	12.14	12.25	12.33	12.22	12.29
7.63	11.98	12.08	12.21	12.26	12.35	12.50	12.52	12.61	12.70	12.72	12.73
7.86	11.48	11.65	11.78	11.80	11.86	11.99	12.01	12.14	12.16	12.23	12.32
8.10	11.50	11.59	11.66	11.75	11.91	11.98	12.09	12.13	12.16	12.15	12.22
8.36	11.20	11.27	11.37	11.46	11.61	11.70	11.78	11.82	11.87	11.87	11.96
8.64	11.48	11.63	11.68	11.85	11.93	12.03	12.03	12.15	12.17	12.13	12.20
8.94	11.85	11.98	12.00	12.19	12.29	12.33	12.42	12.52	12.61	12.52	12.50
9.26	12.10	12.26	12.29	12.37	12.50	12.69	12.63	12.73	12.80	12.81	12.84
9.60	11.67	11.75	11.90	11.96	12.11	12.20	12.23	12.34	12.37	12.37	12.38
9.97	11.68	11.78	11.87	11.98	12.04	12.13	12.26	12.32	12.38	12.34	12.44
10.36	11.87	11.95	12.10	12.17	12.30	12.30	12.44	12.46	12.60	12.56	12.64
10.77	11.88	12.00	12.13	12.21	12.27	12.40	12.42	12.55	12.64	12.62	12.65
11.22	11.68	11.85	11.95	12.11	12.15	12.20	12.30	12.31	12.35	12.40	12.34
11.70	11.81	11.95	12.06	12.19	12.31	12.32	12.42	12.44	12.57	12.45	12.60
12.21	11.46	11.62	11.68	11.83	11.87	11.97	11.99	12.02	12.17	12.16	12.22
12.75	11.79	11.96	12.05	12.16	12.24	12.32	12.34	12.46	12.53	12.54	12.52
13.33	12.02	12.19	12.20	12.40	12.36	12.52	12.53	12.60	12.66	12.72	12.67
13.95	11.82	11.90	12.06	12.18	12.17	12.28	12.36	12.39	12.50	12.33	12.45
14.62	11.55	11.68	11.75	11.93	11.95	12.03	12.13	12.15	12.25	12.26	12.34
15.33	11.47	11.69	11.70	11.92	11.93	12.01	12.16	12.09	12.23	12.20	12.33
16.09	11.61	11.80	11.81	11.89	11.99	12.19	12.23	12.22	12.28	12.31	12.30
16.90	11.94	12.02	12.15	12.19	12.38	12.42	12.49	12.50	12.60	12.62	12.64
17.77	11.77	11.95	12.06	12.10	12.18	12.31	12.34	12.38	12.44	12.47	12.44
18.70	11.69	11.88	11.91	11.99	12.08	12.13	12.28	12.32	12.40	12.40	12.47
19.69	11.77	12.00	12.02	12.15	12.22	12.29	12.41	12.35	12.49	12.56	12.56
20.75	11.57	11.79	11.93	12.01	12.07	12.12	12.25	12.26	12.35	12.37	12.38
21.89	12.14	12.14	12.32	12.38	12.53	12.58	12.66	12.68	12.86	12.79	12.88
23.10	12.32	12.45	12.48	12.67	12.73	12.75	12.86	12.99	13.03	13.00	13.04
24.39	11.79	11.87	12.10	12.11	12.21	12.36	12.39	12.47	12.48	12.49	12.55
25.78	12.02	12.25	12.37	12.43	12.44	12.54	12.59	12.71	12.77	12.69	12.74
27.26	11.95	12.09	12.15	12.31	12.36	12.47	12.53	12.59	12.70	12.60	12.73
28.84	11.99	12.12	12.22	12.26	12.33	12.44	12.55	12.53	12.60	12.59	12.68
30.53	12.11	12.28	12.31	12.39	12.47	12.65	12.71	12.69	12.80	12.80	12.73
32.34	11.74	11.92	12.00	12.10	12.10	12.34	12.35	12.42	12.44	12.50	12.51
34.27	12.15	12.17	12.36	12.47	12.50	12.63	12.62	12.75	12.73	12.84	12.89
36.34	11.76	11.97	11.94	12.20	12.24	12.39	12.45	12.43	12.52	12.56	12.51
38.55	12.10	12.22	12.35	12.43	12.52	12.57	12.66	12.71	12.72	12.69	12.83
40.91	11.96	12.09	12.18	12.24	12.41	12.46	12.56	12.51	12.68	12.61	12.76

43.43	11.76	11.83	11.89	11.96	12.10	12.19	12.23	12.28	12.37	12.36	12.44
46.13	11.81	11.98	11.97	12.19	12.21	12.28	12.28	12.34	12.48	12.49	12.47
49.01	12.08	12.31	12.40	12.49	12.62	12.66	12.72	12.82	12.82	12.86	12.96
52.09	11.82	12.00	12.18	12.30	12.39	12.42	12.48	12.57	12.62	12.67	12.66
55.39	11.85	11.96	12.13	12.14	12.17	12.38	12.50	12.48	12.55	12.51	12.51
58.91	11.75	11.94	12.03	12.15	12.24	12.27	12.43	12.42	12.48	12.34	12.50
62.68	11.86	12.01	12.12	12.27	12.38	12.44	12.51	12.61	12.60	12.60	12.65
66.70	11.84	12.06	12.09	12.29	12.36	12.45	12.51	12.51	12.60	12.60	12.70
71.00	11.95	12.15	12.08	12.37	12.38	12.52	12.60	12.64	12.65	12.71	12.77
75.60	11.75	11.95	12.02	12.23	12.20	12.39	12.43	12.41	12.46	12.54	12.52
80.51	11.84	11.98	12.11	12.30	12.39	12.46	12.51	12.62	12.67	12.65	12.69
85.77	12.14	12.32	12.47	12.58	12.64	12.84	12.87	12.92	13.00	12.95	13.03
91.38	12.03	12.22	12.30	12.43	12.57	12.72	12.69	12.74	12.86	12.82	12.93
97.39	11.98	12.10	12.25	12.37	12.41	12.52	12.68	12.73	12.76	12.82	12.85
103.80	12.26	12.45	12.59	12.72	12.70	12.91	13.06	13.05	13.12	13.07	13.22
110.66	11.86	12.09	12.19	12.32	12.43	12.46	12.60	12.62	12.80	12.72	12.78
117.99	11.94	12.15	12.21	12.34	12.51	12.58	12.59	12.73	12.77	12.83	12.85
125.83	12.18	12.37	12.46	12.59	12.71	12.86	12.81	12.83	13.05	13.04	13.00
134.21	12.05	12.13	12.33	12.47	12.57	12.63	12.71	12.71	12.92	12.85	12.92
143.17	11.94	12.17	12.33	12.47	12.60	12.66	12.72	12.79	12.88	12.93	12.95
152.74	12.02	12.19	12.36	12.51	12.57	12.70	12.74	12.83	12.90	12.97	12.91
162.97	12.22	12.46	12.49	12.63	12.73	12.83	12.86	13.08	13.09	13.15	13.14
173.91	12.07	12.25	12.40	12.46	12.53	12.71	12.75	12.83	12.94	12.96	13.01
185.60	12.24	12.42	12.58	12.69	12.82	12.84	13.00	13.07	13.16	13.17	13.20
198.10	12.19	12.34	12.48	12.58	12.68	12.81	12.83	12.94	13.03	13.04	13.14
211.46	12.25	12.39	12.57	12.70	12.76	12.89	13.04	12.98	13.16	13.10	13.16
225.75	12.25	12.47	12.56	12.70	12.85	12.97	13.05	13.07	13.10	13.10	13.30
241.01	12.09	12.30	12.42	12.59	12.61	12.82	12.85	12.82	12.97	13.06	13.13
257.33	12.29	12.44	12.53	12.66	12.81	12.94	13.02	13.12	13.12	13.18	13.19
274.77	12.49	12.55	12.65	12.81	13.05	13.04	13.17	13.14	13.32	13.26	13.36
293.42	12.42	12.60	12.68	12.80	12.95	13.04	13.04	13.18	13.24	13.29	13.36
313.35	12.25	12.45	12.49	12.69	12.88	12.97	12.89	13.14	13.13	13.08	13.21
334.66	12.46	12.65	12.73	12.97	13.02	13.09	13.24	13.36	13.35	13.36	13.45
357.43	12.37	12.48	12.70	12.89	12.95	13.05	13.13	13.16	13.30	13.34	13.37
381.78	12.15	12.19	12.39	12.51	12.67	12.73	12.85	12.88	13.03	12.99	13.02
407.80	12.23	12.34	12.48	12.64	12.75	12.91	12.94	13.01	13.12	13.06	13.02
435.62	12.49	12.68	12.84	12.82	13.04	13.18	13.38	13.32	13.41	13.41	13.44
465.35	12.28	12.37	12.58	12.71	12.73	12.92	12.95	13.04	13.14	13.18	13.31
497.14	12.28	12.45	12.48	12.64	12.79	12.94	13.06	13.00	13.20	13.11	13.33
531.11	12.10	12.20	12.40	12.52	12.66	12.70	12.90	12.83	12.90	13.01	13.04
567.43	12.38	12.60	12.68	12.88	12.90	13.07	13.17	13.20	13.36	13.32	13.33
606.26	12.36	12.49	12.66	12.77	12.91	12.98	13.10	13.16	13.19	13.27	13.27
647.76	12.21	12.46	12.58	12.66	12.77	12.97	12.91	13.03	13.23	13.16	13.24
692.12	11.88	12.07	12.11	12.30	12.43	12.53	12.73	12.69	12.84	12.86	12.83
739.53	12.22	12.49	12.61	12.75	12.80	12.94	12.95	13.00	13.15	13.17	13.26
790.22	12.11	12.30	12.41	12.50	12.64	12.85	12.93	12.93	13.05	13.17	13.13
844.40	12.22	12.38	12.48	12.67	12.70	12.81	12.94	13.03	13.09	13.08	13.18
902.32	12.19	12.50	12.62	12.69	12.77	12.89	13.05	13.06	13.09	13.20	13.29
964.23	11.98	12.18	12.27	12.38	12.50	12.63	12.68	12.88	12.94	12.91	13.00
1030.41	12.12	12.25	12.35	12.47	12.65	12.80	12.84	12.97	12.96	13.02	13.10

1101.15	12.11	12.29	12.39	12.48	12.62	12.83	12.80	12.92	12.95	13.01	13.02
1176.77	12.19	12.28	12.42	12.48	12.51	12.66	12.89	12.92	13.05	12.98	13.07
1257.60	12.13	12.25	12.49	12.65	12.61	12.75	12.80	12.89	13.04	12.98	13.06
1344.00	12.08	12.12	12.37	12.41	12.58	12.66	12.75	12.88	12.87	12.92	12.98
1436.36	11.97	12.04	12.16	12.36	12.53	12.52	12.63	12.67	12.70	12.73	12.85
1535.09	12.15	12.33	12.49	12.43	12.61	12.71	12.85	12.94	12.97	12.97	13.00
1640.63	11.95	12.10	12.22	12.34	12.54	12.58	12.71	12.80	12.94	12.86	12.90
1753.44	11.86	12.12	12.20	12.32	12.45	12.58	12.65	12.68	12.80	12.81	12.86
1874.02	11.88	12.07	12.18	12.30	12.34	12.55	12.63	12.67	12.77	12.74	12.85
2002.92	11.89	12.05	12.14	12.35	12.34	12.49	12.59	12.64	12.71	12.66	12.76
2140.71	11.59	11.71	11.87	12.16	12.22	12.31	12.43	12.38	12.52	12.47	12.60
2288.00	11.77	11.87	11.98	12.10	12.19	12.36	12.35	12.43	12.58	12.55	12.60
2445.44	11.64	11.79	11.91	12.06	12.21	12.26	12.43	12.40	12.47	12.43	12.51
2613.73	11.83	11.96	12.07	12.18	12.23	12.42	12.50	12.52	12.56	12.52	12.65
2793.63	11.54	11.68	11.79	11.86	11.99	12.03	12.19	12.20	12.23	12.23	12.32
2985.93	11.31	11.49	11.55	11.69	11.81	11.87	11.92	12.03	12.11	12.12	12.21
3191.49	11.36	11.40	11.56	11.65	11.78	11.96	11.90	11.97	12.02	12.12	12.10
3411.21	11.26	11.44	11.50	11.61	11.72	11.71	11.80	11.84	11.87	11.92	11.95
3646.09	11.20	11.33	11.38	11.51	11.68	11.70	11.75	11.89	11.86	11.86	11.94
3897.16	11.09	11.22	11.29	11.37	11.36	11.60	11.67	11.69	11.76	11.65	11.79

Wavelength (nm) / Time (ps)	569.06	570.48	571.91	573.33	574.75	576.18	577.60	579.03	580.45	581.87	583.30
-2.74	0.00	0.03	-0.05	0.08	0.04	-0.04	-0.02	-0.05	-0.04	0.00	-0.15
-2.24	-0.08	-0.02	-0.02	-0.05	-0.04	-0.06	-0.10	-0.11	-0.03	-0.06	-0.12
-1.74	-0.03	-0.06	0.01	-0.02	-0.04	0.00	-0.09	-0.10	0.03	-0.01	0.01
-1.24	-0.01	-0.04	-0.10	-0.09	-0.06	0.06	0.04	0.12	-0.06	-0.05	0.03
-0.74	0.10	0.03	0.03	0.01	0.07	0.00	0.08	-0.03	0.02	0.03	0.06
-0.24	0.03	0.00	0.05	0.02	-0.03	-0.07	0.03	0.05	-0.03	0.04	0.01
-0.14	-0.04	-0.04	0.02	0.02	-0.02	0.07	0.04	0.05	0.05	-0.01	0.12
-0.04	0.02	0.11	0.06	0.03	0.07	0.04	0.01	0.06	0.06	0.07	0.03
0.06	0.01	-0.07	-0.02	0.01	-0.02	-0.05	0.00	0.05	-0.01	0.01	0.00
0.16	0.03	-0.06	0.01	-0.02	-0.03	-0.11	-0.02	-0.06	0.01	-0.05	-0.02
0.26	-0.09	-0.05	-0.07	-0.01	0.00	-0.10	-0.07	-0.04	-0.08	-0.02	-0.06
0.31	-0.02	-0.03	-0.02	0.06	0.03	-0.04	0.00	-0.05	-0.08	0.00	0.10
0.36	0.00	0.04	-0.05	0.07	0.06	-0.02	0.00	0.06	0.00	0.02	0.04
0.41	0.03	0.00	0.02	0.02	0.14	0.07	-0.02	0.01	-0.02	-0.01	0.03
0.46	-0.10	0.03	-0.09	0.07	0.06	-0.06	-0.06	0.05	0.00	0.06	-0.02
0.51	-0.05	0.00	-0.01	0.11	0.11	-0.05	0.02	0.04	0.03	0.00	0.02
0.56	-0.01	0.02	0.01	0.01	0.03	-0.05	0.07	-0.01	0.07	-0.07	-0.03
0.61	-0.07	-0.06	-0.01	0.03	0.08	-0.07	-0.04	0.01	-0.12	-0.01	0.02
0.66	0.01	-0.02	0.05	0.05	0.01	-0.05	-0.07	0.01	0.05	-0.02	0.04
0.71	-0.03	0.00	0.02	0.06	0.07	0.00	-0.05	0.01	0.02	-0.04	0.03
0.76	-0.19	-0.04	-0.02	-0.03	-0.01	-0.07	-0.09	-0.09	-0.05	-0.02	-0.04
0.81	-0.01	-0.01	-0.01	-0.01	0.00	-0.02	-0.06	-0.04	0.05	0.05	0.01
0.86	0.03	-0.06	-0.03	0.00	0.02	-0.01	0.06	0.00	0.00	0.06	0.13
0.91	-0.01	0.06	0.01	0.08	0.03	-0.02	0.00	0.04	0.04	-0.12	0.04
0.96	-0.04	-0.07	-0.07	0.04	0.02	-0.08	-0.01	0.02	0.07	0.00	-0.05
1.01	-0.12	-0.13	-0.07	-0.06	-0.05	-0.10	-0.09	-0.05	-0.09	-0.07	-0.10

1.06	0.02	0.04	0.10	0.02	-0.06	-0.03	-0.02	0.05	0.05	0.05	0.11
1.11	-0.02	-0.01	0.01	-0.01	0.02	-0.03	0.04	0.01	-0.01	-0.02	-0.02
1.16	-0.10	-0.06	0.13	0.10	0.00	-0.07	-0.04	0.07	0.04	-0.13	0.01
1.21	0.01	-0.14	-0.09	-0.01	-0.03	0.21	0.06	-0.10	-0.04	0.04	0.07
1.26	-2.00	-0.96	-0.16	0.56	0.90	0.71	0.37	0.03	-0.28	-0.46	-0.46
1.31	-73.74	-75.39	-74.89	-72.12	-67.09	-60.33	-52.19	-43.44	-34.92	-27.01	-20.23
1.36	205.48	191.08	177.96	165.07	153.41	142.65	133.44	125.43	117.91	110.51	103.24
1.41	-88.60	-82.40	-75.09	-66.09	-55.80	-44.03	-31.05	-16.27	-0.26	16.72	34.38
1.46	21.31	20.66	19.87	18.98	17.91	16.53	15.30	14.18	13.21	12.19	11.33
1.51	13.82	13.69	13.67	13.85	13.98	14.17	14.36	14.83	15.12	15.50	15.94
1.56	13.76	14.03	14.40	14.59	14.85	14.96	15.15	15.46	15.69	15.89	16.20
1.61	14.38	14.50	14.53	14.63	14.66	14.63	14.53	14.38	14.23	14.18	13.97
1.66	13.80	13.89	13.79	13.98	13.91	13.99	14.00	14.10	14.28	14.21	14.36
1.71	12.71	12.73	12.82	12.79	12.84	12.75	12.73	12.71	12.74	12.75	12.87
1.76	13.10	13.21	13.26	13.34	13.44	13.57	13.59	13.72	13.78	13.81	13.88
1.81	12.53	12.59	12.72	13.00	13.06	13.06	13.23	13.35	13.46	13.57	13.68
1.86	12.67	12.71	12.69	12.83	12.74	12.62	12.48	12.46	12.51	12.56	12.61
1.91	12.41	12.44	12.50	12.60	12.66	12.63	12.77	12.77	12.79	12.82	12.78
1.96	11.98	12.12	12.10	12.33	12.40	12.41	12.43	12.62	12.73	12.82	12.97
2.01	12.42	12.50	12.53	12.74	12.89	12.83	12.90	12.88	12.83	12.83	12.79
2.06	12.59	12.57	12.63	12.75	12.82	12.79	12.87	12.95	12.99	13.03	13.06
2.11	12.08	12.13	12.18	12.11	12.18	12.22	12.24	12.21	12.32	12.31	12.35
2.16	12.31	12.31	12.33	12.48	12.43	12.52	12.63	12.64	12.75	12.62	12.68
2.21	12.57	12.63	12.69	12.83	12.94	12.88	13.00	13.03	13.18	13.22	13.19
2.26	12.49	12.54	12.60	12.61	12.58	12.55	12.52	12.53	12.57	12.62	12.57
2.31	12.15	12.19	12.15	12.25	12.39	12.30	12.34	12.41	12.44	12.45	12.45
2.36	11.89	11.95	12.05	12.25	12.21	12.23	12.25	12.39	12.45	12.52	12.56
2.41	12.22	12.25	12.26	12.45	12.41	12.35	12.41	12.36	12.32	12.34	12.35
2.46	11.92	12.00	12.10	12.14	12.20	12.15	12.10	12.22	12.24	12.22	12.38
2.51	12.21	12.25	12.35	12.47	12.44	12.51	12.47	12.51	12.55	12.52	12.64
2.56	12.31	12.44	12.39	12.54	12.58	12.49	12.53	12.61	12.67	12.58	12.62
2.61	11.86	11.94	11.97	12.14	12.12	12.11	12.16	12.29	12.28	12.31	12.30
2.66	12.18	12.20	12.26	12.26	12.35	12.24	12.27	12.36	12.26	12.25	12.36
2.71	11.96	12.05	11.94	12.14	12.23	12.12	12.23	12.21	12.26	12.25	12.19
2.76	12.22	12.35	12.40	12.44	12.38	12.38	12.50	12.55	12.50	12.52	12.61
2.81	11.82	11.95	11.95	12.05	11.95	11.94	12.07	12.01	12.06	12.01	12.08
2.86	11.76	11.82	11.84	11.85	12.04	12.02	12.04	12.10	12.17	12.09	12.20
2.91	12.03	12.03	12.15	12.23	12.21	12.13	12.27	12.32	12.26	12.36	12.33
2.96	12.26	12.36	12.35	12.49	12.54	12.49	12.55	12.53	12.47	12.41	12.50
3.01	12.22	12.23	12.30	12.47	12.47	12.35	12.45	12.47	12.44	12.49	12.52
3.06	11.74	11.73	11.77	11.83	11.84	11.82	11.80	11.85	11.85	11.86	11.82
3.11	12.11	12.18	12.05	12.15	12.30	12.27	12.22	12.27	12.29	12.26	12.33
3.16	12.07	12.04	12.07	12.10	12.13	12.18	12.15	12.28	12.22	12.20	12.32
3.21	11.82	11.92	11.97	12.05	11.99	11.97	11.93	12.09	12.08	12.02	12.09
3.26	11.95	12.02	12.00	12.16	12.16	12.12	12.17	12.22	12.22	12.22	12.25
3.31	11.94	12.03	11.94	12.13	12.10	12.01	12.13	12.16	12.07	12.17	12.29
3.36	12.26	12.17	12.29	12.35	12.30	12.39	12.25	12.33	12.31	12.34	12.31
3.41	12.21	12.20	12.28	12.35	12.37	12.35	12.39	12.43	12.45	12.42	12.43
3.46	11.92	11.97	12.08	12.04	12.06	11.99	12.14	12.11	12.09	12.13	12.16
3.51	12.17	12.26	12.29	12.30	12.31	12.31	12.38	12.38	12.37	12.37	12.40

3.56	11.86	11.94	12.04	12.09	12.10	12.04	11.97	12.06	12.13	12.10	12.10
3.61	11.70	11.76	11.75	11.83	11.72	11.78	11.80	11.82	11.86	11.86	11.82
3.66	11.66	11.76	11.76	11.87	11.91	11.86	11.91	11.82	11.99	11.96	11.93
3.71	12.15	12.22	12.18	12.37	12.33	12.26	12.31	12.31	12.36	12.34	12.38
3.76	12.43	12.48	12.46	12.54	12.58	12.65	12.60	12.60	12.71	12.60	12.65
3.81	11.72	11.76	11.87	11.99	11.96	11.96	11.97	11.98	12.06	11.96	12.04
3.86	11.54	11.68	11.71	11.71	11.81	11.72	11.74	11.81	11.88	11.81	11.86
3.91	12.36	12.37	12.34	12.36	12.46	12.41	12.42	12.42	12.41	12.42	12.49
3.96	12.06	12.15	12.12	12.30	12.30	12.14	12.24	12.28	12.25	12.24	12.29
4.01	12.21	12.27	12.26	12.34	12.39	12.30	12.42	12.32	12.27	12.32	12.37
4.06	12.22	12.28	12.37	12.40	12.44	12.43	12.38	12.53	12.47	12.48	12.50
4.11	12.00	12.09	12.16	12.19	12.20	12.22	12.15	12.34	12.25	12.23	12.29
4.16	12.22	12.19	12.24	12.33	12.42	12.29	12.30	12.35	12.39	12.36	12.41
4.21	12.64	12.63	12.67	12.66	12.72	12.70	12.71	12.76	12.76	12.70	12.78
4.26	12.12	12.11	12.08	12.18	12.26	12.19	12.16	12.27	12.22	12.21	12.23
4.31	11.98	12.02	12.10	12.26	12.08	12.16	12.18	12.10	12.22	12.22	12.17
4.36	12.08	12.09	12.04	12.19	12.18	12.28	12.24	12.17	12.27	12.25	12.22
4.41	11.85	11.85	11.96	12.01	12.03	12.04	12.01	12.21	12.06	12.05	12.00
4.46	12.39	12.38	12.45	12.58	12.51	12.52	12.52	12.49	12.52	12.41	12.42
4.51	12.16	12.21	12.29	12.28	12.34	12.24	12.33	12.36	12.32	12.36	12.34
4.56	11.89	11.99	12.05	12.11	12.13	12.04	12.03	12.09	12.02	12.09	12.12
4.61	11.78	11.86	11.91	12.00	11.93	12.05	11.96	12.03	12.00	11.99	12.05
4.66	12.23	12.18	12.21	12.40	12.40	12.32	12.37	12.35	12.41	12.38	12.42
4.71	12.20	12.18	12.27	12.28	12.39	12.20	12.25	12.31	12.33	12.23	12.23
4.76	12.51	12.56	12.57	12.65	12.67	12.64	12.61	12.63	12.64	12.70	12.76
4.81	12.04	12.00	12.02	12.23	12.08	12.19	12.19	12.14	12.09	12.12	12.20
4.86	11.93	11.94	12.01	12.07	12.06	11.98	12.02	12.11	12.07	12.06	12.08
4.91	12.32	12.35	12.41	12.58	12.52	12.54	12.49	12.63	12.56	12.60	12.51
4.96	12.02	12.09	12.12	12.21	12.19	12.10	12.14	12.25	12.23	12.12	12.22
5.01	12.16	12.16	12.16	12.25	12.27	12.15	12.20	12.21	12.17	12.20	12.22
5.06	12.22	12.24	12.28	12.29	12.35	12.31	12.37	12.35	12.30	12.36	12.36
5.11	11.98	11.99	12.03	12.13	12.20	12.09	12.21	12.16	12.16	12.12	12.08
5.16	12.00	11.99	11.95	12.09	12.08	12.00	12.07	12.07	12.12	12.10	12.09
5.21	12.03	12.09	12.16	12.23	12.20	12.21	12.14	12.28	12.22	12.22	12.22
5.26	12.34	12.43	12.48	12.57	12.54	12.47	12.51	12.50	12.47	12.52	12.61
5.31	12.27	12.26	12.30	12.32	12.31	12.33	12.38	12.37	12.47	12.34	12.30
5.38	12.20	12.05	12.19	12.26	12.32	12.26	12.30	12.33	12.28	12.25	12.31
5.45	12.47	12.54	12.51	12.60	12.67	12.51	12.63	12.58	12.68	12.66	12.69
5.53	12.25	12.09	12.19	12.35	12.32	12.29	12.34	12.22	12.39	12.36	12.40
5.61	12.56	12.58	12.54	12.66	12.58	12.61	12.65	12.60	12.68	12.59	12.56
5.70	12.37	12.40	12.37	12.45	12.42	12.44	12.40	12.38	12.39	12.41	12.46
5.80	12.51	12.49	12.57	12.61	12.57	12.52	12.49	12.62	12.59	12.56	12.62
5.90	11.94	11.91	12.02	12.05	12.04	12.02	12.07	12.08	12.04	12.12	12.14
6.01	12.56	12.68	12.68	12.71	12.72	12.76	12.63	12.77	12.72	12.79	12.75
6.13	12.16	12.22	12.28	12.30	12.41	12.32	12.28	12.37	12.33	12.41	12.40
6.25	11.99	11.95	12.02	12.13	12.15	12.02	12.06	12.14	12.10	12.10	12.20
6.39	12.15	12.06	12.11	12.18	12.24	12.19	12.20	12.21	12.24	12.23	12.21
6.53	12.14	12.21	12.18	12.23	12.31	12.16	12.19	12.32	12.27	12.21	12.31
6.69	12.37	12.34	12.39	12.48	12.40	12.34	12.37	12.47	12.50	12.41	12.39
6.85	12.11	12.12	12.23	12.23	12.42	12.30	12.23	12.30	12.24	12.24	12.28

7.03	12.30	12.31	12.31	12.40	12.44	12.34	12.44	12.39	12.33	12.36	12.36
7.21	12.34	12.28	12.38	12.41	12.45	12.38	12.40	12.45	12.38	12.40	12.38
7.41	12.37	12.43	12.44	12.38	12.44	12.47	12.47	12.48	12.42	12.39	12.47
7.63	12.66	12.76	12.81	12.85	12.85	12.81	12.82	12.89	12.90	12.81	12.80
7.86	12.25	12.27	12.31	12.36	12.33	12.35	12.36	12.37	12.35	12.38	12.32
8.10	12.34	12.35	12.37	12.40	12.40	12.25	12.34	12.34	12.33	12.37	12.34
8.36	11.92	12.02	11.93	12.15	12.14	11.94	12.07	12.00	12.03	11.93	11.93
8.64	12.34	12.27	12.31	12.35	12.32	12.40	12.32	12.32	12.39	12.32	12.27
8.94	12.58	12.61	12.60	12.67	12.59	12.57	12.68	12.66	12.69	12.54	12.50
9.26	12.78	12.82	12.97	12.92	12.94	12.92	12.90	12.93	12.87	12.87	12.90
9.60	12.40	12.45	12.54	12.52	12.56	12.51	12.46	12.47	12.49	12.54	12.38
9.97	12.41	12.40	12.47	12.57	12.53	12.58	12.47	12.55	12.43	12.39	12.49
10.36	12.68	12.64	12.68	12.69	12.72	12.66	12.65	12.62	12.73	12.65	12.66
10.77	12.63	12.67	12.61	12.71	12.69	12.67	12.67	12.69	12.68	12.67	12.70
11.22	12.38	12.36	12.49	12.43	12.48	12.52	12.46	12.46	12.46	12.33	12.42
11.70	12.62	12.59	12.60	12.68	12.68	12.58	12.62	12.57	12.61	12.61	12.58
12.21	12.20	12.18	12.20	12.24	12.23	12.22	12.30	12.24	12.22	12.15	12.20
12.75	12.60	12.59	12.64	12.69	12.71	12.48	12.62	12.66	12.66	12.58	12.51
13.33	12.72	12.74	12.70	12.84	12.82	12.70	12.72	12.71	12.75	12.71	12.68
13.95	12.48	12.56	12.47	12.60	12.59	12.47	12.49	12.49	12.58	12.41	12.47
14.62	12.32	12.31	12.36	12.36	12.44	12.36	12.27	12.34	12.41	12.28	12.27
15.33	12.31	12.31	12.31	12.41	12.30	12.28	12.30	12.22	12.33	12.25	12.17
16.09	12.47	12.29	12.34	12.35	12.40	12.34	12.27	12.35	12.34	12.31	12.35
16.90	12.73	12.63	12.76	12.70	12.72	12.65	12.62	12.64	12.65	12.65	12.65
17.77	12.53	12.53	12.52	12.49	12.51	12.45	12.46	12.49	12.47	12.40	12.43
18.70	12.46	12.46	12.43	12.53	12.54	12.41	12.46	12.37	12.45	12.28	12.36
19.69	12.54	12.60	12.56	12.66	12.67	12.62	12.58	12.63	12.52	12.55	12.47
20.75	12.38	12.34	12.43	12.43	12.49	12.30	12.44	12.37	12.31	12.32	12.35
21.89	12.78	12.84	12.91	12.87	12.87	12.85	12.79	12.79	12.86	12.80	12.74
23.10	13.00	12.99	12.95	13.08	13.13	12.98	12.99	12.94	12.92	12.94	12.87
24.39	12.48	12.47	12.47	12.61	12.48	12.43	12.47	12.44	12.52	12.37	12.41
25.78	12.87	12.90	12.79	12.85	12.82	12.78	12.76	12.73	12.73	12.70	12.75
27.26	12.73	12.70	12.64	12.70	12.72	12.66	12.72	12.65	12.70	12.61	12.66
28.84	12.68	12.76	12.65	12.76	12.70	12.64	12.60	12.70	12.63	12.59	12.65
30.53	12.82	12.82	12.84	12.87	12.84	12.71	12.79	12.78	12.75	12.68	12.67
32.34	12.53	12.47	12.62	12.54	12.51	12.49	12.39	12.50	12.42	12.39	12.38
34.27	12.79	12.84	12.89	12.83	12.84	12.73	12.74	12.79	12.82	12.69	12.62
36.34	12.55	12.62	12.45	12.65	12.59	12.49	12.62	12.52	12.43	12.42	12.46
38.55	12.77	12.85	12.76	12.91	12.88	12.77	12.79	12.77	12.80	12.74	12.66
40.91	12.75	12.66	12.76	12.71	12.72	12.66	12.61	12.62	12.63	12.56	12.57
43.43	12.40	12.42	12.46	12.47	12.47	12.38	12.34	12.36	12.37	12.24	12.32
46.13	12.52	12.49	12.50	12.62	12.52	12.49	12.52	12.51	12.37	12.39	12.38
49.01	12.88	12.93	12.90	12.92	12.90	12.88	12.78	12.93	12.89	12.80	12.83
52.09	12.68	12.65	12.66	12.80	12.73	12.62	12.73	12.62	12.59	12.62	12.53
55.39	12.56	12.54	12.54	12.67	12.61	12.52	12.54	12.48	12.50	12.42	12.45
58.91	12.49	12.48	12.54	12.68	12.55	12.41	12.46	12.47	12.53	12.38	12.41
62.68	12.61	12.65	12.82	12.74	12.71	12.65	12.69	12.68	12.70	12.63	12.59
66.70	12.67	12.70	12.70	12.79	12.81	12.70	12.67	12.70	12.62	12.65	12.60
71.00	12.77	12.71	12.83	12.85	12.83	12.75	12.64	12.76	12.69	12.67	12.55
75.60	12.61	12.58	12.56	12.67	12.65	12.60	12.63	12.54	12.43	12.47	12.46

80.51	12.72	12.70	12.68	12.75	12.75	12.65	12.66	12.70	12.66	12.60	12.71
85.77	13.07	13.06	12.98	13.08	13.11	13.08	13.06	13.04	13.08	13.02	12.94
91.38	12.87	12.93	12.97	12.97	13.02	12.89	12.94	12.97	12.87	12.79	12.82
97.39	12.90	12.86	12.83	12.88	12.86	12.84	12.79	12.86	12.76	12.70	12.77
103.80	13.24	13.23	13.20	13.24	13.19	13.11	13.20	13.22	13.08	13.07	13.03
110.66	12.84	12.81	12.84	12.88	12.87	12.78	12.71	12.69	12.71	12.76	12.71
117.99	12.95	12.89	12.83	12.94	12.86	12.92	12.83	12.77	12.72	12.82	12.82
125.83	13.09	13.04	13.24	13.17	13.15	13.06	13.09	13.02	13.05	12.95	12.99
134.21	12.99	13.00	12.98	12.95	13.00	12.99	12.95	13.04	12.97	12.89	12.88
143.17	12.95	12.98	13.02	13.03	13.09	12.99	12.96	12.89	12.97	12.87	12.86
152.74	12.96	12.95	12.99	13.04	13.09	12.93	12.97	12.97	12.89	12.83	12.87
162.97	13.10	13.26	13.21	13.19	13.26	13.13	13.20	13.14	13.08	13.06	13.10
173.91	13.02	13.07	13.05	13.04	13.10	12.96	13.01	13.03	13.05	12.92	12.97
185.60	13.16	13.28	13.29	13.29	13.30	13.23	13.26	13.23	13.24	13.19	13.20
198.10	13.20	13.12	13.09	13.21	13.13	13.09	13.17	13.23	13.14	13.04	13.00
211.46	13.24	13.25	13.27	13.33	13.27	13.21	13.20	13.22	13.18	13.16	13.21
225.75	13.21	13.28	13.26	13.33	13.39	13.21	13.18	13.28	13.25	13.31	13.21
241.01	13.04	13.14	13.17	13.17	13.31	13.17	13.14	13.11	13.17	13.11	13.12
257.33	13.24	13.30	13.27	13.39	13.31	13.30	13.32	13.35	13.26	13.26	13.17
274.77	13.31	13.38	13.31	13.41	13.42	13.37	13.50	13.42	13.40	13.36	13.33
293.42	13.28	13.43	13.42	13.50	13.44	13.47	13.37	13.40	13.33	13.39	13.35
313.35	13.19	13.25	13.34	13.25	13.29	13.23	13.24	13.19	13.17	13.25	13.11
334.66	13.48	13.54	13.49	13.56	13.50	13.48	13.56	13.55	13.50	13.47	13.46
357.43	13.44	13.40	13.51	13.53	13.49	13.41	13.37	13.48	13.38	13.33	13.41
381.78	13.06	13.18	13.22	13.18	13.13	13.10	13.13	13.09	13.04	13.07	13.08
407.80	13.11	13.17	13.25	13.23	13.28	13.13	13.21	13.15	13.18	13.12	13.15
435.62	13.56	13.49	13.49	13.63	13.59	13.61	13.52	13.45	13.46	13.37	13.42
465.35	13.27	13.26	13.30	13.31	13.28	13.33	13.31	13.24	13.27	13.21	13.31
497.14	13.25	13.40	13.29	13.46	13.30	13.27	13.30	13.34	13.22	13.25	13.25
531.11	12.98	13.02	13.02	13.11	13.05	13.03	13.08	13.06	13.09	13.05	12.96
567.43	13.41	13.46	13.39	13.44	13.49	13.44	13.38	13.44	13.34	13.34	13.38
606.26	13.35	13.41	13.43	13.50	13.46	13.36	13.42	13.50	13.43	13.30	13.33
647.76	13.19	13.30	13.28	13.38	13.30	13.21	13.33	13.22	13.25	13.20	13.14
692.12	12.87	12.84	13.00	12.93	12.94	12.93	12.87	12.85	12.83	12.77	12.90
739.53	13.28	13.26	13.32	13.31	13.30	13.19	13.30	13.29	13.28	13.17	13.23
790.22	13.10	13.20	13.15	13.28	13.26	13.27	13.16	13.24	13.19	13.14	13.07
844.40	13.18	13.23	13.25	13.21	13.18	13.16	13.20	13.09	13.23	13.16	13.10
902.32	13.24	13.24	13.26	13.29	13.25	13.26	13.20	13.24	13.16	13.09	13.13
964.23	12.98	13.05	13.00	13.12	13.04	13.00	12.93	12.95	12.86	12.88	12.89
1030.41	13.13	13.11	13.17	13.24	13.11	13.08	13.12	13.06	13.01	13.05	12.93
1101.15	13.08	13.09	13.05	13.22	13.07	12.97	13.05	13.06	12.97	12.95	12.95
1176.77	13.09	13.13	13.11	13.20	13.15	13.14	13.09	13.05	13.01	12.98	12.97
1257.60	13.04	13.07	13.06	13.18	13.22	13.07	13.02	13.12	13.05	12.95	13.03
1344.00	12.98	13.01	13.03	13.03	13.05	13.00	12.98	12.98	12.97	12.90	12.87
1436.36	12.88	12.89	12.88	12.94	12.85	12.74	12.87	12.85	12.80	12.77	12.72
1535.09	13.00	13.04	13.04	13.14	13.02	13.06	13.03	13.03	12.98	12.82	12.86
1640.63	12.87	12.91	12.96	13.01	12.97	12.83	12.84	12.89	12.94	12.76	12.86
1753.44	12.82	12.92	12.90	12.99	12.93	12.79	12.88	12.80	12.77	12.73	12.69
1874.02	12.78	12.83	12.87	12.86	12.82	12.79	12.82	12.82	12.72	12.68	12.72
2002.92	12.73	12.69	12.78	12.87	12.82	12.69	12.68	12.63	12.59	12.61	12.57

2140.71	12.57	12.53	12.56	12.58	12.58	12.55	12.50	12.46	12.38	12.46	12.35
2288.00	12.62	12.58	12.51	12.62	12.60	12.45	12.52	12.54	12.46	12.43	12.40
2445.44	12.61	12.52	12.54	12.54	12.50	12.50	12.44	12.43	12.44	12.34	12.35
2613.73	12.59	12.52	12.64	12.59	12.59	12.52	12.52	12.53	12.37	12.40	12.29
2793.63	12.25	12.32	12.35	12.39	12.37	12.23	12.19	12.15	12.09	12.02	12.08
2985.93	12.15	12.14	12.09	12.24	12.07	12.00	11.98	12.00	11.83	11.93	11.80
3191.49	12.10	12.02	12.01	12.13	12.11	12.03	11.89	11.90	11.91	11.79	11.75
3411.21	11.94	11.82	11.90	11.86	11.84	11.75	11.71	11.71	11.55	11.58	11.54
3646.09	11.92	11.75	11.84	11.84	11.85	11.76	11.64	11.66	11.46	11.54	11.46
3897.16	11.63	11.63	11.68	11.63	11.66	11.51	11.54	11.51	11.38	11.30	11.25

Wavelength (nm) / Time (ps)	584.72	586.15	587.57	588.99	590.42	591.84	593.27	594.69	596.12	597.54	598.96
-2.74	0.00	0.08	0.01	0.05	0.03	-0.02	0.06	-0.07	0.02	0.05	0.02
-2.24	-0.07	-0.13	-0.09	-0.07	-0.08	0.00	-0.15	-0.12	-0.11	0.02	-0.13
-1.74	0.01	-0.03	0.06	0.05	-0.05	-0.01	0.07	0.07	0.00	-0.04	-0.08
-1.24	-0.01	0.01	0.08	0.06	0.07	0.04	-0.01	0.09	-0.01	0.06	-0.01
-0.74	0.06	-0.03	-0.04	-0.02	-0.03	-0.01	0.06	0.00	-0.03	0.01	0.12
-0.24	0.00	0.01	0.03	-0.03	-0.02	-0.12	-0.08	-0.02	-0.01	-0.02	-0.01
-0.14	0.01	0.06	-0.02	0.01	0.02	0.05	0.03	0.05	0.14	0.04	0.05
-0.04	0.01	0.03	-0.02	-0.05	0.06	0.08	0.02	0.00	0.00	-0.11	0.04
0.06	0.00	0.03	0.01	-0.01	-0.05	-0.05	-0.09	-0.04	0.03	-0.01	0.03
0.16	0.01	0.00	-0.10	-0.07	0.04	-0.02	-0.08	-0.07	0.01	-0.08	-0.02
0.26	-0.03	-0.06	-0.11	-0.01	0.02	0.04	-0.07	0.04	-0.03	-0.08	-0.04
0.31	0.00	-0.03	-0.03	-0.08	0.02	-0.10	0.01	-0.03	-0.02	-0.04	-0.06
0.36	0.04	-0.03	0.06	0.04	0.12	-0.01	0.00	0.01	0.00	-0.06	0.01
0.41	0.04	0.06	0.00	-0.01	0.00	-0.04	0.06	-0.11	0.03	0.02	0.02
0.46	0.01	0.07	0.01	-0.06	0.02	0.13	-0.03	-0.03	-0.01	-0.02	0.03
0.51	0.06	-0.02	-0.03	0.02	-0.07	0.00	0.09	-0.02	0.03	0.00	-0.02
0.56	-0.04	0.04	0.01	-0.01	-0.03	0.05	0.05	-0.05	0.10	0.00	-0.07
0.61	-0.01	-0.04	0.00	-0.01	-0.02	0.03	-0.03	-0.09	-0.03	-0.03	-0.04
0.66	0.01	0.02	0.06	0.04	0.09	0.03	0.10	-0.02	-0.02	-0.09	-0.05
0.71	-0.01	0.00	0.08	0.01	0.04	-0.02	0.04	-0.04	0.01	0.02	0.03
0.76	-0.04	-0.06	-0.09	-0.05	-0.05	-0.05	-0.07	-0.03	-0.04	-0.06	-0.10
0.81	0.03	0.03	0.05	0.04	0.10	0.01	-0.01	0.04	0.01	0.03	-0.05
0.86	0.06	0.01	0.08	-0.02	0.05	0.03	0.10	-0.01	-0.04	0.04	-0.01
0.91	0.04	0.00	-0.03	0.02	0.04	-0.06	-0.06	-0.03	-0.01	-0.06	-0.07
0.96	-0.04	0.06	-0.01	-0.12	-0.06	-0.04	-0.08	0.02	0.02	-0.01	-0.11
1.01	-0.10	-0.06	-0.10	-0.09	-0.07	-0.11	-0.12	-0.10	-0.04	-0.07	-0.09
1.06	0.06	0.00	0.00	0.00	0.03	0.08	-0.04	-0.03	-0.01	-0.01	0.03
1.11	0.03	-0.05	-0.06	0.07	0.02	0.05	-0.02	0.03	-0.13	-0.04	0.04
1.16	0.10	0.11	0.00	-0.01	0.05	0.00	0.02	0.01	-0.02	-0.05	0.01
1.21	0.05	-0.03	0.02	0.02	-0.09	0.00	0.00	0.01	0.07	-0.02	-0.09
1.26	-0.41	-0.54	-0.52	-0.21	-0.20	-0.07	-0.08	-0.08	-0.18	-0.08	0.08
1.31	-14.66	-10.35	-7.25	-5.16	-3.88	-3.13	-2.70	-2.32	-2.03	-1.45	-0.82
1.36	96.31	88.64	80.17	71.51	61.92	52.41	43.09	34.45	26.38	19.37	13.37
1.41	53.24	72.04	89.96	107.36	122.43	135.48	146.09	154.24	159.59	162.57	163.07
1.46	10.40	9.35	7.80	5.86	3.38	0.37	-3.30	-7.56	-12.08	-16.90	-21.72
1.51	16.39	16.83	17.20	17.77	18.19	18.54	18.95	19.21	19.47	19.70	19.92

1.56	16.37	16.63	16.88	17.03	17.06	17.12	17.26	17.33	17.19	17.03	16.84
1.61	13.94	13.83	13.75	13.66	13.67	13.62	13.72	13.78	13.96	14.28	14.51
1.66	14.40	14.32	14.31	14.41	14.27	14.18	14.10	13.87	13.80	13.71	13.57
1.71	12.89	12.95	13.15	13.25	13.42	13.44	13.81	13.94	14.11	14.35	14.58
1.76	13.78	13.76	13.81	13.73	13.67	13.62	13.44	13.50	13.46	13.52	13.46
1.81	13.69	13.92	13.89	13.82	13.86	13.76	13.68	13.62	13.46	13.28	13.07
1.86	12.58	12.68	12.79	12.86	12.87	13.11	13.17	13.40	13.51	13.67	13.86
1.91	12.79	12.67	12.67	12.57	12.60	12.44	12.30	12.30	12.14	12.15	12.14
1.96	13.01	13.12	13.18	13.33	13.48	13.46	13.50	13.52	13.53	13.58	13.53
2.01	12.73	12.72	12.68	12.64	12.61	12.57	12.59	12.62	12.82	12.84	12.88
2.06	13.10	13.04	13.07	12.99	13.03	12.84	12.78	12.72	12.59	12.43	12.29
2.11	12.48	12.59	12.67	12.75	12.84	12.97	13.00	13.12	13.19	13.39	13.38
2.16	12.60	12.60	12.54	12.51	12.54	12.42	12.44	12.45	12.42	12.55	12.52
2.21	13.16	13.24	13.26	13.21	13.27	13.23	13.14	13.17	13.07	12.94	12.76
2.26	12.62	12.59	12.63	12.72	12.70	12.69	12.84	12.92	12.98	12.96	13.12
2.31	12.57	12.51	12.50	12.57	12.38	12.45	12.36	12.28	12.23	12.10	12.06
2.36	12.67	12.65	12.71	12.67	12.75	12.71	12.81	12.73	12.67	12.59	12.53
2.41	12.31	12.32	12.38	12.33	12.40	12.37	12.40	12.53	12.53	12.74	12.74
2.46	12.38	12.38	12.34	12.31	12.25	12.29	12.17	12.23	12.06	12.04	11.88
2.51	12.62	12.68	12.80	12.75	12.82	12.86	12.97	13.09	13.23	13.12	13.19
2.56	12.48	12.61	12.56	12.49	12.37	12.42	12.38	12.34	12.40	12.44	12.47
2.61	12.34	12.29	12.30	12.28	12.29	12.12	12.13	12.01	11.99	11.83	11.81
2.66	12.36	12.43	12.42	12.46	12.47	12.52	12.59	12.73	12.72	12.80	12.82
2.71	12.27	12.13	12.24	12.16	12.14	12.13	12.12	12.05	11.95	11.95	11.94
2.76	12.73	12.56	12.71	12.73	12.68	12.71	12.62	12.71	12.58	12.52	12.50
2.81	12.10	12.04	12.12	12.01	12.14	12.16	12.14	12.18	12.24	12.22	12.33
2.86	12.17	12.19	12.12	12.14	11.99	12.02	11.97	11.83	11.84	11.81	11.78
2.91	12.31	12.40	12.52	12.49	12.54	12.60	12.59	12.68	12.61	12.59	12.54
2.96	12.54	12.50	12.48	12.36	12.44	12.40	12.43	12.36	12.44	12.46	12.57
3.01	12.58	12.58	12.66	12.57	12.61	12.46	12.48	12.46	12.48	12.41	12.43
3.06	11.89	11.97	11.99	11.93	12.01	11.94	12.01	12.13	12.04	12.09	12.12
3.11	12.31	12.13	12.24	12.18	12.18	12.09	12.08	12.16	12.04	12.05	12.08
3.16	12.34	12.45	12.40	12.43	12.41	12.32	12.37	12.45	12.29	12.25	12.20
3.21	12.06	12.05	12.16	12.03	12.17	12.08	12.12	12.15	12.10	12.16	12.11
3.26	12.18	12.16	12.25	12.13	12.11	12.14	12.06	12.00	12.03	11.97	11.91
3.31	12.21	12.30	12.29	12.34	12.32	12.21	12.26	12.29	12.27	12.21	12.19
3.36	12.36	12.23	12.28	12.29	12.21	12.15	12.17	12.22	12.19	12.28	12.25
3.41	12.45	12.42	12.41	12.49	12.38	12.39	12.35	12.29	12.30	12.25	12.25
3.46	12.22	12.16	12.18	12.25	12.20	12.15	12.25	12.20	12.11	12.21	12.05
3.51	12.35	12.39	12.39	12.26	12.28	12.28	12.20	12.22	12.15	12.24	12.19
3.56	12.11	12.21	12.17	12.16	12.16	12.23	12.14	12.12	12.08	11.99	11.96
3.61	11.86	11.91	11.93	11.86	11.92	11.85	11.88	11.87	11.87	11.90	11.86
3.66	11.86	11.90	11.99	11.91	11.86	11.88	11.88	11.76	11.83	11.76	11.70
3.71	12.32	12.48	12.46	12.48	12.48	12.43	12.41	12.42	12.38	12.42	12.39
3.76	12.71	12.67	12.59	12.59	12.59	12.56	12.57	12.64	12.67	12.58	12.65
3.81	12.01	11.99	12.04	11.96	11.89	11.83	11.83	11.83	11.74	11.77	11.71
3.86	11.85	11.85	11.82	11.91	11.79	11.82	11.82	11.87	11.89	11.90	11.89
3.91	12.43	12.36	12.42	12.40	12.43	12.34	12.35	12.37	12.28	12.29	12.40
3.96	12.24	12.17	12.26	12.32	12.26	12.19	12.21	12.06	12.15	12.04	12.08
4.01	12.33	12.28	12.36	12.34	12.37	12.29	12.34	12.38	12.34	12.34	12.39

4.06	12.46	12.45	12.45	12.37	12.39	12.37	12.29	12.30	12.31	12.30	12.28
4.11	12.25	12.31	12.28	12.40	12.24	12.25	12.27	12.19	12.21	12.25	12.18
4.16	12.34	12.33	12.39	12.34	12.35	12.27	12.36	12.40	12.26	12.23	12.22
4.21	12.81	12.77	12.78	12.72	12.76	12.63	12.58	12.62	12.53	12.56	12.53
4.26	12.26	12.23	12.25	12.22	12.23	12.22	12.15	12.23	12.20	12.20	12.24
4.31	12.13	12.09	12.15	12.08	12.05	12.13	12.04	12.05	12.08	11.97	12.03
4.36	12.18	12.16	12.20	12.15	12.20	12.18	12.17	12.10	12.05	12.00	12.02
4.41	12.05	12.04	12.07	12.08	12.06	12.13	12.06	12.15	12.11	12.12	12.13
4.46	12.52	12.56	12.51	12.51	12.52	12.41	12.47	12.37	12.33	12.33	12.37
4.51	12.35	12.33	12.34	12.30	12.37	12.40	12.28	12.39	12.29	12.23	12.19
4.56	12.12	12.10	12.12	12.11	12.09	12.14	12.10	12.04	12.05	11.95	12.01
4.61	12.04	11.94	11.96	11.85	11.93	11.93	11.94	11.90	11.92	11.91	11.91
4.66	12.33	12.39	12.39	12.44	12.47	12.33	12.43	12.32	12.29	12.28	12.20
4.71	12.32	12.28	12.25	12.28	12.28	12.28	12.24	12.17	12.26	12.23	12.15
4.76	12.67	12.63	12.66	12.66	12.73	12.54	12.55	12.59	12.49	12.48	12.46
4.81	12.16	12.20	12.26	12.19	12.19	12.11	12.14	12.08	12.14	12.20	12.03
4.86	12.09	12.05	12.04	12.06	12.12	11.92	12.02	12.10	11.96	11.89	11.90
4.91	12.52	12.62	12.55	12.54	12.51	12.57	12.53	12.45	12.40	12.31	12.42
4.96	12.21	12.12	12.12	12.20	12.28	12.26	12.01	12.12	12.18	12.10	12.13
5.01	12.17	12.25	12.19	12.24	12.21	12.19	12.14	12.14	12.04	12.13	11.93
5.06	12.30	12.50	12.40	12.32	12.37	12.42	12.29	12.35	12.27	12.22	12.26
5.11	12.16	12.08	12.18	12.13	12.06	12.14	12.08	12.07	12.04	12.04	12.05
5.16	12.03	12.04	12.11	12.20	12.03	12.05	11.95	11.95	11.98	11.83	11.89
5.21	12.23	12.18	12.28	12.27	12.18	12.21	12.20	12.14	12.21	12.16	12.06
5.26	12.55	12.50	12.50	12.45	12.42	12.44	12.38	12.47	12.47	12.36	12.38
5.31	12.45	12.43	12.37	12.32	12.31	12.26	12.31	12.31	12.30	12.23	12.23
5.38	12.24	12.31	12.37	12.34	12.21	12.29	12.17	12.22	12.23	12.21	12.09
5.45	12.60	12.64	12.67	12.54	12.68	12.53	12.50	12.55	12.53	12.51	12.54
5.53	12.41	12.37	12.23	12.20	12.28	12.32	12.28	12.25	12.11	12.17	12.07
5.61	12.57	12.60	12.64	12.63	12.57	12.55	12.47	12.60	12.54	12.42	12.47
5.70	12.47	12.41	12.55	12.51	12.42	12.33	12.34	12.33	12.28	12.32	12.20
5.80	12.58	12.57	12.60	12.61	12.50	12.48	12.44	12.42	12.41	12.38	12.39
5.90	12.01	12.07	12.09	12.12	12.17	12.08	12.02	11.98	11.95	11.95	11.91
6.01	12.75	12.71	12.71	12.73	12.69	12.65	12.65	12.60	12.59	12.58	12.58
6.13	12.28	12.24	12.32	12.26	12.32	12.21	12.19	12.17	12.16	12.17	12.11
6.25	12.09	12.12	12.11	12.13	12.07	11.97	12.08	12.00	11.97	11.86	11.88
6.39	12.17	12.10	12.22	12.11	12.10	12.03	12.02	12.07	11.96	12.05	11.86
6.53	12.21	12.23	12.21	12.30	12.15	12.08	12.08	12.08	12.01	11.97	11.97
6.69	12.38	12.34	12.34	12.41	12.32	12.32	12.27	12.21	12.23	12.27	12.18
6.85	12.19	12.19	12.26	12.12	12.17	12.10	12.10	12.03	12.10	12.11	11.96
7.03	12.34	12.27	12.32	12.24	12.25	12.13	12.19	12.05	12.18	12.06	12.15
7.21	12.40	12.38	12.44	12.33	12.30	12.26	12.26	12.22	12.19	12.18	12.17
7.41	12.38	12.39	12.42	12.41	12.34	12.27	12.28	12.26	12.23	12.25	12.21
7.63	12.87	12.87	12.81	12.74	12.74	12.72	12.66	12.65	12.64	12.47	12.56
7.86	12.34	12.33	12.36	12.25	12.30	12.26	12.24	12.20	12.16	12.18	12.09
8.10	12.33	12.40	12.38	12.38	12.26	12.27	12.23	12.19	12.16	12.17	12.15
8.36	11.94	11.93	11.99	11.89	11.95	11.83	11.81	11.87	11.86	11.85	11.73
8.64	12.28	12.28	12.29	12.30	12.19	12.21	12.15	12.16	12.20	12.04	12.03
8.94	12.61	12.57	12.51	12.55	12.54	12.47	12.40	12.39	12.28	12.31	12.37
9.26	12.87	12.90	12.79	12.84	12.78	12.83	12.69	12.67	12.59	12.61	12.61

9.60	12.42	12.37	12.40	12.36	12.30	12.37	12.30	12.28	12.30	12.23	12.16
9.97	12.49	12.43	12.43	12.42	12.36	12.34	12.33	12.26	12.28	12.30	12.21
10.36	12.58	12.60	12.55	12.48	12.55	12.45	12.40	12.48	12.42	12.33	12.35
10.77	12.52	12.61	12.57	12.54	12.55	12.47	12.42	12.45	12.36	12.47	12.37
11.22	12.38	12.35	12.31	12.21	12.27	12.14	12.20	12.23	12.14	12.06	12.08
11.70	12.55	12.48	12.42	12.46	12.42	12.40	12.36	12.35	12.28	12.30	12.22
12.21	12.19	12.12	12.13	12.06	12.13	11.97	11.95	12.07	11.99	11.91	11.87
12.75	12.57	12.53	12.46	12.47	12.47	12.39	12.41	12.29	12.43	12.21	12.34
13.33	12.60	12.63	12.63	12.56	12.47	12.49	12.50	12.48	12.41	12.30	12.36
13.95	12.40	12.45	12.42	12.48	12.38	12.38	12.29	12.30	12.17	12.18	12.07
14.62	12.30	12.32	12.25	12.17	12.17	12.19	12.05	12.10	12.06	12.06	12.02
15.33	12.17	12.13	12.27	12.16	12.06	12.02	11.97	11.96	11.96	11.93	11.87
16.09	12.25	12.21	12.28	12.20	12.14	12.07	12.00	12.01	12.14	12.06	12.02
16.90	12.58	12.51	12.62	12.56	12.39	12.42	12.36	12.33	12.27	12.17	12.20
17.77	12.32	12.35	12.34	12.30	12.27	12.20	12.17	12.17	12.09	12.05	12.04
18.70	12.27	12.22	12.29	12.23	12.21	12.12	12.14	12.12	12.03	12.06	12.03
19.69	12.44	12.50	12.41	12.51	12.39	12.32	12.22	12.23	12.19	12.19	12.19
20.75	12.33	12.28	12.32	12.20	12.17	12.16	12.16	12.07	12.09	12.09	11.97
21.89	12.73	12.65	12.62	12.62	12.60	12.54	12.46	12.49	12.34	12.46	12.28
23.10	12.85	12.86	12.77	12.83	12.69	12.74	12.63	12.56	12.58	12.60	12.60
24.39	12.41	12.27	12.30	12.27	12.29	12.23	12.19	12.18	12.10	12.04	12.04
25.78	12.74	12.65	12.67	12.57	12.55	12.56	12.47	12.41	12.35	12.42	12.26
27.26	12.49	12.55	12.51	12.39	12.40	12.34	12.28	12.28	12.21	12.26	12.15
28.84	12.55	12.57	12.60	12.46	12.50	12.37	12.36	12.31	12.24	12.21	12.21
30.53	12.64	12.63	12.62	12.66	12.50	12.50	12.50	12.45	12.43	12.31	12.22
32.34	12.37	12.39	12.33	12.32	12.26	12.22	12.19	12.09	12.15	12.10	12.00
34.27	12.69	12.65	12.60	12.51	12.59	12.46	12.50	12.38	12.37	12.40	12.34
36.34	12.36	12.39	12.38	12.32	12.25	12.23	12.24	12.17	12.11	12.21	12.10
38.55	12.65	12.54	12.67	12.60	12.53	12.41	12.46	12.37	12.36	12.34	12.29
40.91	12.60	12.47	12.43	12.53	12.42	12.36	12.33	12.38	12.28	12.19	12.14
43.43	12.24	12.27	12.24	12.18	12.14	12.06	12.02	12.00	11.96	11.92	11.95
46.13	12.30	12.34	12.30	12.32	12.19	12.21	12.12	12.10	12.05	11.99	12.00
49.01	12.74	12.78	12.79	12.75	12.63	12.61	12.53	12.57	12.45	12.46	12.48
52.09	12.52	12.57	12.45	12.48	12.36	12.36	12.32	12.38	12.28	12.30	12.18
55.39	12.39	12.32	12.43	12.30	12.25	12.28	12.18	12.26	12.13	12.10	12.07
58.91	12.38	12.33	12.36	12.31	12.32	12.29	12.12	12.13	12.13	12.05	11.92
62.68	12.52	12.48	12.49	12.41	12.47	12.36	12.37	12.32	12.25	12.31	12.18
66.70	12.52	12.50	12.46	12.42	12.42	12.37	12.38	12.32	12.27	12.28	12.26
71.00	12.60	12.54	12.58	12.55	12.49	12.46	12.49	12.43	12.33	12.28	12.26
75.60	12.46	12.47	12.41	12.43	12.36	12.37	12.24	12.16	12.20	12.22	12.12
80.51	12.60	12.59	12.61	12.59	12.45	12.48	12.40	12.35	12.33	12.31	12.25
85.77	12.93	12.87	12.97	12.86	12.76	12.75	12.71	12.73	12.63	12.63	12.55
91.38	12.83	12.79	12.73	12.70	12.78	12.66	12.60	12.61	12.56	12.46	12.52
97.39	12.73	12.75	12.61	12.70	12.54	12.64	12.49	12.48	12.34	12.40	12.36
103.80	13.06	13.06	13.07	12.99	12.96	12.93	12.89	12.90	12.84	12.75	12.68
110.66	12.73	12.68	12.58	12.56	12.56	12.48	12.45	12.37	12.44	12.41	12.38
117.99	12.81	12.75	12.68	12.66	12.69	12.53	12.55	12.48	12.53	12.45	12.39
125.83	13.01	12.94	12.94	12.85	12.83	12.77	12.74	12.72	12.57	12.64	12.68
134.21	12.83	12.78	12.79	12.70	12.76	12.68	12.67	12.61	12.49	12.51	12.49
143.17	12.82	12.75	12.79	12.77	12.74	12.66	12.67	12.63	12.59	12.51	12.48

152.74	12.97	12.80	12.91	12.76	12.74	12.73	12.78	12.74	12.67	12.64	12.55
162.97	12.96	13.01	13.02	12.98	12.91	12.85	12.82	12.83	12.80	12.76	12.76
173.91	12.93	12.87	12.93	12.85	12.94	12.82	12.76	12.64	12.68	12.65	12.59
185.60	13.11	13.19	13.14	13.11	13.10	12.99	12.93	12.92	12.87	12.85	12.80
198.10	12.96	13.02	13.03	12.98	12.94	12.88	12.81	12.84	12.80	12.77	12.72
211.46	13.17	13.13	13.13	13.08	13.04	13.02	12.94	12.98	12.92	12.90	12.84
225.75	13.18	13.16	13.23	13.10	13.03	13.01	12.94	13.00	12.93	12.92	12.86
241.01	13.10	13.01	12.99	13.05	12.97	12.85	12.87	12.83	12.85	12.74	12.76
257.33	13.19	13.19	13.09	13.19	13.04	13.04	12.97	12.97	12.96	12.90	12.89
274.77	13.36	13.33	13.31	13.22	13.23	13.18	13.13	13.06	13.14	12.97	12.98
293.42	13.32	13.24	13.20	13.23	13.18	13.08	13.04	13.05	13.08	12.99	12.95
313.35	13.15	13.10	13.06	13.09	13.07	12.94	12.97	12.91	12.90	12.73	12.76
334.66	13.47	13.39	13.31	13.45	13.31	13.26	13.25	13.17	13.11	13.16	13.11
357.43	13.31	13.35	13.35	13.29	13.22	13.17	13.20	13.12	13.20	13.05	13.01
381.78	13.02	13.01	12.95	12.92	12.94	12.89	12.87	12.82	12.77	12.73	12.69
407.80	13.01	13.14	13.02	13.02	12.94	12.97	12.91	12.84	12.80	12.78	12.65
435.62	13.39	13.37	13.36	13.30	13.31	13.17	13.14	13.12	13.15	13.00	12.97
465.35	13.13	13.17	13.17	13.10	13.13	13.00	12.97	12.93	12.94	12.92	12.84
497.14	13.15	13.20	13.21	13.11	13.00	13.03	12.93	12.91	12.90	12.91	12.90
531.11	12.98	12.95	12.94	12.84	12.90	12.79	12.78	12.76	12.70	12.65	12.59
567.43	13.32	13.28	13.28	13.18	13.19	13.12	13.12	13.12	12.95	12.95	12.93
606.26	13.31	13.29	13.35	13.23	13.14	13.16	13.09	12.97	12.99	12.94	12.94
647.76	13.16	13.16	13.13	13.07	13.09	12.95	12.90	12.89	12.84	12.75	12.75
692.12	12.76	12.77	12.75	12.71	12.63	12.58	12.66	12.56	12.50	12.49	12.48
739.53	13.16	13.14	13.06	13.04	13.08	12.94	12.89	12.89	12.80	12.80	12.82
790.22	13.06	13.11	13.10	12.98	12.94	12.82	12.92	12.86	12.84	12.73	12.68
844.40	13.06	13.01	13.05	12.95	13.04	12.92	12.83	12.83	12.77	12.72	12.63
902.32	13.07	13.06	13.11	12.93	13.01	12.90	12.86	12.87	12.82	12.70	12.73
964.23	12.87	12.80	12.83	12.72	12.69	12.64	12.69	12.57	12.53	12.44	12.43
1030.41	12.89	12.98	12.96	12.89	12.80	12.79	12.76	12.62	12.61	12.56	12.63
1101.15	13.00	12.85	12.94	12.94	12.82	12.76	12.59	12.65	12.57	12.63	12.53
1176.77	12.94	12.88	12.84	12.92	12.76	12.69	12.71	12.68	12.53	12.52	12.50
1257.60	12.99	12.89	12.89	12.87	12.72	12.76	12.67	12.58	12.52	12.61	12.51
1344.00	12.92	12.78	12.74	12.73	12.70	12.65	12.60	12.48	12.49	12.48	12.40
1436.36	12.67	12.64	12.58	12.64	12.51	12.43	12.44	12.45	12.34	12.34	12.30
1535.09	12.83	12.81	12.84	12.63	12.71	12.62	12.58	12.59	12.44	12.40	12.41
1640.63	12.74	12.61	12.75	12.64	12.61	12.52	12.50	12.52	12.35	12.36	12.17
1753.44	12.67	12.69	12.57	12.49	12.50	12.40	12.33	12.34	12.31	12.21	12.09
1874.02	12.68	12.67	12.58	12.50	12.50	12.37	12.44	12.32	12.28	12.28	12.12
2002.92	12.55	12.57	12.46	12.44	12.30	12.27	12.22	12.24	12.10	12.08	12.01
2140.71	12.32	12.24	12.20	12.20	12.10	12.05	11.97	12.03	11.96	11.89	11.83
2288.00	12.30	12.32	12.27	12.38	12.18	12.21	12.06	11.92	11.98	11.88	11.82
2445.44	12.29	12.23	12.18	12.18	12.16	12.19	12.01	12.01	11.92	11.83	11.80
2613.73	12.26	12.21	12.19	12.12	12.11	11.95	11.93	11.95	11.86	11.85	11.75
2793.63	12.01	12.01	11.90	11.92	11.81	11.72	11.69	11.64	11.63	11.45	11.43
2985.93	11.80	11.72	11.69	11.55	11.64	11.56	11.35	11.32	11.31	11.28	11.21
3191.49	11.73	11.67	11.58	11.54	11.53	11.50	11.41	11.30	11.21	11.14	11.06
3411.21	11.47	11.41	11.35	11.28	11.26	11.21	11.14	11.07	11.02	10.90	10.87
3646.09	11.42	11.21	11.28	11.24	11.18	11.02	11.03	10.99	10.90	10.87	10.81
3897.16	11.18	11.04	11.14	10.96	10.96	10.94	10.82	10.74	10.66	10.61	10.52

Time (ps) \ Wavelength (nm)	600.39	601.81	603.24	604.66	606.08	607.51	608.93	610.36	611.78	613.20	614.63
-2.74	-0.03	0.06	-0.05	0.00	-0.03	0.01	0.00	0.03	0.04	0.04	0.07
-2.24	-0.03	-0.07	-0.13	-0.10	-0.14	-0.13	-0.10	-0.13	-0.04	-0.12	-0.05
-1.74	0.00	-0.09	-0.01	-0.09	-0.07	0.00	0.02	0.04	-0.11	0.05	-0.05
-1.24	0.04	0.04	0.08	0.15	0.07	-0.01	-0.01	0.01	0.06	-0.05	-0.01
-0.74	0.02	0.05	0.04	0.03	0.04	-0.02	0.06	0.06	0.02	0.10	0.09
-0.24	-0.05	-0.09	-0.02	0.01	0.03	0.02	0.04	-0.01	0.05	-0.03	0.06
-0.14	0.06	0.03	0.01	-0.06	0.05	0.08	-0.03	0.00	0.00	0.01	0.07
-0.04	0.00	0.06	0.07	0.06	0.04	0.05	0.02	-0.01	-0.02	0.01	-0.18
0.06	0.12	-0.02	-0.02	0.01	-0.06	0.06	-0.03	0.04	0.07	0.00	0.12
0.16	0.08	0.02	-0.05	-0.07	-0.02	-0.07	0.08	0.00	0.01	0.08	0.07
0.26	0.02	-0.05	-0.03	0.04	-0.05	-0.08	-0.09	0.05	-0.02	-0.05	0.02
0.31	0.02	0.01	-0.05	-0.03	-0.06	-0.08	-0.07	0.01	-0.02	-0.13	-0.03
0.36	0.02	0.01	-0.04	0.00	-0.03	-0.01	0.03	-0.05	0.01	0.04	0.06
0.41	-0.07	-0.02	-0.01	-0.07	0.04	0.01	-0.07	0.09	0.07	-0.05	0.00
0.46	0.02	-0.01	-0.02	-0.05	0.02	-0.01	0.02	0.03	-0.05	0.04	0.02
0.51	0.05	0.00	-0.07	-0.05	-0.02	-0.02	-0.07	0.00	0.06	0.10	0.03
0.56	-0.01	-0.07	-0.03	0.01	-0.04	0.07	0.02	0.05	0.01	0.03	0.05
0.61	-0.03	0.06	-0.04	-0.03	-0.01	-0.03	0.00	-0.04	-0.02	0.01	-0.05
0.66	0.03	0.02	0.00	0.03	-0.03	-0.04	0.04	0.08	0.06	0.06	0.05
0.71	-0.01	0.05	-0.07	0.00	0.04	-0.05	0.01	-0.03	-0.06	0.04	-0.03
0.76	-0.02	-0.02	0.00	-0.03	-0.04	-0.09	-0.02	-0.01	0.10	0.03	-0.01
0.81	-0.07	0.06	-0.01	-0.01	0.04	0.11	0.03	0.09	0.01	0.07	0.08
0.86	0.04	-0.02	-0.05	0.04	0.00	0.02	0.02	0.04	-0.02	0.03	0.07
0.91	0.08	-0.05	-0.01	0.05	0.05	0.05	-0.01	0.08	0.12	0.08	0.08
0.96	0.04	-0.01	-0.02	-0.09	0.03	-0.04	-0.07	0.03	-0.05	0.00	0.05
1.01	-0.11	-0.06	-0.09	-0.11	-0.12	-0.07	-0.06	-0.03	-0.03	-0.02	-0.02
1.06	0.04	0.01	0.01	0.00	-0.01	0.01	0.00	0.07	0.00	-0.03	0.04
1.11	0.05	-0.08	-0.08	-0.09	0.03	0.05	-0.07	0.10	0.06	0.03	0.10
1.16	-0.01	0.08	-0.01	-0.01	-0.01	-0.06	-0.01	0.12	-0.01	0.00	0.01
1.21	0.00	0.10	0.03	-0.01	0.02	0.13	-0.07	0.05	0.06	0.10	0.11
1.26	0.13	0.15	-0.03	-0.26	-0.12	-0.09	0.29	0.16	-0.09	-0.35	-0.39
1.31	-0.24	0.07	0.21	0.05	-0.23	-0.54	-0.82	-0.70	-0.60	-0.40	-0.41
1.36	8.74	4.71	0.91	-2.27	-5.94	-9.69	-14.08	-18.92	-24.64	-30.38	-36.70
1.41	162.52	160.54	157.60	155.48	152.97	151.60	150.48	150.19	149.06	148.51	147.49
1.46	-26.53	-30.85	-35.15	-38.52	-41.52	-43.13	-44.30	-44.05	-43.59	-41.45	-39.12
1.51	20.03	20.07	20.14	20.21	20.45	20.69	20.81	21.08	21.06	21.13	21.21
1.56	16.61	16.41	16.04	15.67	15.25	15.10	14.87	14.65	14.31	14.20	14.03
1.61	14.90	15.18	15.54	15.76	15.99	16.27	16.55	16.67	16.67	16.57	16.51
1.66	13.57	13.49	13.41	13.54	13.62	13.88	13.98	14.21	14.54	14.80	15.02
1.71	14.71	14.85	14.95	15.02	15.02	14.97	14.81	14.79	14.69	14.42	14.26
1.76	13.52	13.72	13.81	14.10	14.25	14.52	14.72	15.01	15.08	15.31	15.33
1.81	12.98	12.83	12.60	12.45	12.41	12.28	12.33	12.36	12.34	12.48	12.65
1.86	14.04	14.09	14.15	14.29	14.26	14.16	14.09	14.11	13.96	13.78	13.63
1.91	12.08	12.23	12.34	12.46	12.63	12.86	13.11	13.33	13.51	13.71	13.94
1.96	13.41	13.28	13.20	12.95	12.89	12.79	12.64	12.62	12.55	12.43	12.55
2.01	13.23	13.32	13.42	13.68	13.74	13.83	14.04	14.12	14.10	14.04	14.04

2.06	12.24	12.18	12.08	11.99	12.06	12.21	12.28	12.39	12.47	12.66	12.77
2.11	13.48	13.34	13.35	13.42	13.26	13.29	13.15	13.02	12.97	12.85	12.70
2.16	12.65	12.75	12.86	13.05	13.21	13.37	13.49	13.76	13.71	13.86	13.91
2.21	12.72	12.65	12.44	12.39	12.35	12.35	12.37	12.36	12.47	12.41	12.56
2.26	13.31	13.29	13.37	13.37	13.42	13.42	13.40	13.38	13.45	13.25	13.27
2.31	12.16	12.18	12.18	12.30	12.37	12.40	12.56	12.72	12.76	12.95	13.04
2.36	12.39	12.26	12.24	12.18	12.14	12.10	12.03	12.16	12.04	12.10	12.22
2.41	12.90	12.99	13.11	13.20	13.23	13.35	13.32	13.28	13.24	13.25	13.20
2.46	11.88	11.87	11.79	11.81	11.76	11.83	11.89	12.07	12.13	12.18	12.35
2.51	13.29	13.23	13.22	13.27	13.17	13.16	12.97	13.01	12.82	12.77	12.70
2.56	12.49	12.59	12.67	12.77	12.84	12.84	12.99	13.14	13.12	13.20	13.19
2.61	11.74	11.72	11.69	11.72	11.73	11.79	11.85	12.05	12.01	12.16	12.31
2.66	12.83	12.82	12.88	12.92	12.88	12.86	12.87	12.81	12.77	12.69	12.66
2.71	11.95	12.17	12.13	12.18	12.31	12.37	12.49	12.60	12.61	12.69	12.73
2.76	12.43	12.41	12.33	12.25	12.18	12.18	12.15	12.18	12.29	12.10	12.26
2.81	12.46	12.49	12.44	12.53	12.54	12.53	12.56	12.53	12.59	12.52	12.51
2.86	11.71	11.77	11.82	11.86	11.97	11.99	12.07	12.16	12.23	12.24	12.41
2.91	12.61	12.61	12.54	12.35	12.47	12.37	12.32	12.38	12.32	12.30	12.26
2.96	12.52	12.61	12.70	12.73	12.77	12.81	12.81	12.94	12.90	12.94	13.02
3.01	12.24	12.26	12.21	12.26	12.18	12.25	12.23	12.16	12.16	12.31	12.30
3.06	12.15	12.16	12.08	12.18	12.26	12.15	12.22	12.18	12.17	12.14	11.98
3.11	12.08	12.11	12.05	12.11	12.30	12.32	12.37	12.51	12.46	12.47	12.61
3.16	12.30	12.17	12.10	12.03	12.08	12.03	12.06	12.15	12.06	12.03	12.11
3.21	12.16	12.19	12.15	12.21	12.23	12.23	12.24	12.25	12.23	12.13	12.22
3.26	11.95	11.95	11.95	11.92	11.94	12.03	12.07	12.19	12.22	12.19	12.32
3.31	12.22	12.18	12.10	12.08	12.05	12.04	12.00	12.03	11.98	11.99	11.87
3.36	12.40	12.38	12.28	12.41	12.40	12.48	12.56	12.63	12.51	12.52	12.58
3.41	12.20	12.16	12.08	12.16	12.05	12.29	12.24	12.34	12.35	12.37	12.40
3.46	12.17	12.15	12.12	12.25	12.09	12.08	12.01	12.07	12.04	12.10	12.03
3.51	12.15	12.25	12.15	12.14	12.23	12.31	12.32	12.46	12.40	12.37	12.46
3.56	11.90	11.92	11.87	11.86	11.88	11.85	11.81	11.90	11.87	11.88	11.94
3.61	11.99	11.90	11.89	11.89	11.93	11.95	11.88	11.90	11.84	11.84	11.83
3.66	11.63	11.71	11.60	11.74	11.83	11.83	11.81	11.99	11.95	11.99	12.07
3.71	12.35	12.34	12.29	12.44	12.29	12.26	12.14	12.26	12.22	12.23	12.10
3.76	12.63	12.66	12.62	12.63	12.69	12.67	12.67	12.76	12.76	12.75	12.74
3.81	11.79	11.68	11.73	11.78	11.76	11.72	11.89	11.98	11.92	11.95	12.00
3.86	11.78	11.81	11.85	11.77	11.79	11.75	11.71	11.81	11.82	11.76	11.68
3.91	12.30	12.39	12.27	12.35	12.48	12.41	12.55	12.60	12.53	12.56	12.59
3.96	12.04	12.01	11.96	11.93	11.89	11.88	11.93	12.05	11.95	12.06	12.03
4.01	12.32	12.33	12.34	12.33	12.33	12.36	12.33	12.37	12.22	12.29	12.28
4.06	12.30	12.36	12.37	12.30	12.28	12.37	12.43	12.54	12.50	12.50	12.48
4.11	12.11	12.23	12.15	12.08	12.11	12.12	12.14	12.17	12.07	12.13	12.18
4.16	12.36	12.29	12.37	12.42	12.40	12.36	12.37	12.53	12.45	12.43	12.44
4.21	12.59	12.59	12.51	12.48	12.55	12.57	12.51	12.51	12.61	12.55	12.64
4.26	12.22	12.20	12.17	12.23	12.12	12.17	12.12	12.11	12.17	12.18	12.16
4.31	12.05	12.10	12.01	12.09	12.07	12.12	12.15	12.17	12.15	12.21	12.15
4.36	11.99	12.12	12.05	12.02	12.05	12.08	12.07	12.08	12.06	12.04	12.17
4.41	12.17	12.05	11.99	12.13	12.09	12.14	12.09	12.07	12.11	12.10	12.03
4.46	12.32	12.34	12.29	12.38	12.35	12.41	12.36	12.53	12.54	12.47	12.50
4.51	12.18	12.17	12.13	12.19	12.17	12.11	12.15	12.18	12.08	12.06	12.12

4.56	12.01	12.15	12.05	12.08	12.06	12.04	12.10	12.14	12.16	12.04	12.20
4.61	11.79	11.87	11.81	11.86	11.85	11.87	11.87	11.98	12.03	12.07	12.08
4.66	12.28	12.22	12.25	12.24	12.22	12.25	12.24	12.27	12.24	12.22	12.35
4.71	12.21	12.25	12.16	12.14	12.21	12.28	12.20	12.33	12.33	12.31	12.24
4.76	12.54	12.36	12.35	12.33	12.33	12.39	12.40	12.51	12.42	12.39	12.37
4.81	12.10	12.01	12.09	12.05	12.08	12.05	12.11	12.08	12.06	12.02	12.07
4.86	12.05	11.90	11.87	11.90	11.93	12.06	12.09	12.12	12.08	12.14	12.15
4.91	12.37	12.40	12.39	12.35	12.37	12.25	12.21	12.35	12.37	12.30	12.38
4.96	12.07	12.09	12.09	12.08	12.16	12.09	12.13	12.15	12.16	12.22	12.20
5.01	12.04	12.07	12.06	12.10	11.96	12.00	12.01	12.11	12.10	12.01	12.19
5.06	12.31	12.24	12.16	12.14	12.17	12.17	12.18	12.20	12.22	12.22	12.22
5.11	12.08	12.12	11.98	12.02	11.98	12.06	12.15	12.15	12.13	12.06	12.03
5.16	11.96	11.85	11.83	11.84	11.88	11.88	11.92	11.97	11.97	11.83	11.88
5.21	12.10	12.09	12.01	12.05	11.99	11.97	12.11	12.03	12.14	12.06	11.98
5.26	12.32	12.31	12.39	12.39	12.31	12.41	12.42	12.37	12.44	12.50	12.47
5.31	12.27	12.24	12.15	12.21	12.21	12.17	12.07	12.28	12.24	12.26	12.22
5.38	12.12	12.19	12.07	12.08	12.13	12.06	12.19	12.19	12.20	12.10	12.26
5.45	12.48	12.41	12.42	12.43	12.42	12.40	12.34	12.56	12.43	12.45	12.48
5.53	12.08	12.02	12.05	12.08	12.15	12.00	12.08	12.16	12.21	12.09	12.12
5.61	12.42	12.46	12.36	12.44	12.44	12.42	12.43	12.43	12.42	12.44	12.46
5.70	12.28	12.33	12.23	12.26	12.19	12.27	12.28	12.34	12.27	12.27	12.35
5.80	12.38	12.45	12.32	12.34	12.33	12.40	12.45	12.40	12.45	12.45	12.45
5.90	11.88	11.81	11.86	11.88	11.83	11.88	11.87	11.88	11.92	11.84	11.89
6.01	12.49	12.54	12.52	12.48	12.51	12.56	12.55	12.59	12.57	12.61	12.59
6.13	12.19	12.12	12.05	12.04	12.01	12.10	12.12	12.16	12.16	12.18	12.19
6.25	11.97	11.86	11.87	11.93	11.85	11.88	11.91	11.96	11.95	11.99	12.04
6.39	11.89	11.97	12.03	11.98	11.90	11.94	11.97	11.96	11.95	12.01	11.97
6.53	12.06	12.00	12.01	11.96	12.00	12.00	11.99	12.01	11.94	12.04	11.99
6.69	12.17	12.09	12.08	12.18	12.05	12.14	12.17	12.29	12.20	12.16	12.23
6.85	12.07	11.98	11.96	12.02	11.93	11.92	11.96	12.11	11.95	12.03	12.08
7.03	12.15	12.04	12.06	12.01	12.08	12.10	12.01	12.07	12.11	12.02	12.12
7.21	12.19	12.09	12.07	12.09	12.20	12.17	12.15	12.11	12.16	12.14	12.13
7.41	12.18	12.21	12.04	12.09	12.12	12.18	12.21	12.30	12.26	12.24	12.12
7.63	12.51	12.53	12.42	12.56	12.60	12.52	12.46	12.51	12.53	12.48	12.58
7.86	12.13	12.06	12.10	11.97	12.01	12.07	12.17	12.14	12.16	12.11	12.13
8.10	12.22	12.19	12.05	12.09	12.03	12.09	12.21	12.17	12.11	12.13	12.16
8.36	11.70	11.78	11.82	11.73	11.76	11.65	11.72	11.76	11.74	11.73	11.83
8.64	12.09	12.05	12.06	12.07	12.02	12.03	12.05	12.08	12.12	12.00	12.06
8.94	12.32	12.24	12.24	12.23	12.19	12.25	12.30	12.25	12.31	12.18	12.42
9.26	12.57	12.56	12.51	12.46	12.58	12.57	12.55	12.66	12.59	12.57	12.58
9.60	12.18	12.21	12.23	12.12	12.14	12.18	12.13	12.23	12.22	12.11	12.13
9.97	12.18	12.17	12.17	12.19	12.16	12.18	12.21	12.15	12.18	12.18	12.09
10.36	12.29	12.31	12.27	12.30	12.37	12.32	12.27	12.32	12.25	12.32	12.37
10.77	12.37	12.31	12.26	12.28	12.23	12.20	12.28	12.28	12.28	12.35	12.28
11.22	12.10	12.10	12.04	11.98	11.96	11.97	12.09	12.04	12.07	11.97	11.99
11.70	12.31	12.23	12.17	12.27	12.26	12.20	12.17	12.29	12.28	12.30	12.29
12.21	11.88	11.85	11.87	11.85	11.82	11.89	11.85	11.92	11.84	11.86	11.92
12.75	12.23	12.33	12.22	12.19	12.16	12.17	12.25	12.33	12.21	12.15	12.26
13.33	12.29	12.38	12.26	12.25	12.26	12.20	12.21	12.29	12.25	12.30	12.20
13.95	12.18	12.11	12.09	12.13	12.19	12.05	12.18	12.18	12.12	12.09	12.10

14.62	11.98	11.98	11.87	11.98	11.91	11.89	11.93	11.91	11.96	11.89	11.99
15.33	11.94	12.00	11.85	11.91	11.86	11.88	11.82	11.83	11.85	11.79	11.85
16.09	11.99	11.97	11.86	11.96	11.92	11.91	11.91	12.02	12.01	11.90	11.97
16.90	12.26	12.26	12.12	12.14	12.14	12.12	12.19	12.13	12.13	12.13	12.19
17.77	11.97	11.97	12.00	11.98	11.93	11.99	11.96	12.01	11.88	11.95	11.94
18.70	11.93	11.97	11.87	11.99	11.91	11.87	11.91	11.96	11.90	11.87	11.80
19.69	12.19	12.08	12.06	12.09	12.03	12.16	12.11	12.07	12.04	12.01	12.07
20.75	11.98	11.88	11.92	11.92	11.84	12.03	11.97	11.99	11.96	11.93	11.89
21.89	12.37	12.35	12.28	12.28	12.21	12.28	12.34	12.29	12.32	12.19	12.22
23.10	12.53	12.48	12.52	12.53	12.43	12.44	12.47	12.54	12.47	12.38	12.43
24.39	11.93	12.06	11.93	11.99	11.92	12.02	11.98	11.98	11.97	11.99	12.05
25.78	12.31	12.36	12.21	12.25	12.25	12.27	12.23	12.26	12.26	12.21	12.17
27.26	12.21	12.18	12.11	12.13	12.04	12.06	12.19	12.12	12.16	12.07	12.11
28.84	12.25	12.18	12.18	12.12	12.12	12.20	12.14	12.11	12.17	12.12	12.12
30.53	12.29	12.27	12.15	12.15	12.16	12.30	12.13	12.21	12.23	12.21	12.18
32.34	12.00	12.01	11.97	12.02	12.01	11.98	11.91	12.00	11.99	11.85	11.90
34.27	12.34	12.18	12.24	12.22	12.12	12.20	12.27	12.27	12.28	12.18	12.19
36.34	12.08	12.03	11.91	11.99	11.98	12.02	12.02	12.07	12.07	11.99	12.01
38.55	12.30	12.23	12.23	12.24	12.13	12.22	12.22	12.35	12.25	12.22	12.29
40.91	12.09	12.08	12.04	12.06	12.12	12.02	12.11	12.09	12.04	11.99	12.10
43.43	11.93	11.86	11.85	11.94	11.84	11.82	11.76	11.79	11.73	11.77	11.75
46.13	11.94	11.94	11.94	11.91	11.82	11.92	11.92	12.02	11.96	11.91	11.89
49.01	12.44	12.40	12.25	12.26	12.29	12.33	12.35	12.37	12.48	12.29	12.27
52.09	12.11	12.26	12.14	12.10	12.08	12.06	12.10	12.07	12.14	12.04	12.10
55.39	12.07	12.09	11.88	11.94	11.97	11.99	12.10	12.05	11.97	11.92	11.90
58.91	11.98	12.01	12.00	11.96	11.92	11.94	11.87	12.04	11.97	11.98	12.03
62.68	12.20	12.17	12.12	12.20	12.10	12.05	12.12	12.09	12.23	12.15	12.12
66.70	12.17	12.16	12.18	12.17	12.16	12.12	12.19	12.23	12.21	12.21	12.10
71.00	12.22	12.19	12.16	12.22	12.17	12.11	12.22	12.18	12.17	12.13	12.10
75.60	12.13	12.06	12.06	12.10	12.02	12.09	12.07	12.11	12.10	12.00	12.06
80.51	12.25	12.26	12.20	12.14	12.20	12.18	12.17	12.24	12.20	12.21	12.16
85.77	12.53	12.57	12.47	12.53	12.53	12.53	12.50	12.37	12.42	12.37	12.45
91.38	12.55	12.43	12.33	12.37	12.40	12.42	12.43	12.44	12.47	12.39	12.42
97.39	12.34	12.26	12.26	12.28	12.22	12.32	12.27	12.21	12.31	12.20	12.19
103.80	12.71	12.67	12.67	12.67	12.72	12.72	12.71	12.62	12.71	12.67	12.59
110.66	12.36	12.39	12.24	12.26	12.28	12.27	12.31	12.39	12.28	12.31	12.32
117.99	12.41	12.35	12.34	12.26	12.28	12.26	12.33	12.31	12.31	12.23	12.31
125.83	12.60	12.69	12.62	12.55	12.49	12.56	12.58	12.56	12.49	12.59	12.57
134.21	12.44	12.38	12.42	12.40	12.45	12.35	12.36	12.42	12.39	12.34	12.36
143.17	12.57	12.49	12.51	12.42	12.48	12.50	12.42	12.42	12.47	12.37	12.42
152.74	12.53	12.54	12.41	12.52	12.42	12.51	12.43	12.54	12.52	12.42	12.50
162.97	12.68	12.65	12.62	12.62	12.56	12.59	12.61	12.58	12.62	12.55	12.54
173.91	12.52	12.63	12.47	12.52	12.52	12.52	12.50	12.60	12.51	12.54	12.54
185.60	12.75	12.82	12.78	12.71	12.73	12.67	12.70	12.68	12.74	12.68	12.67
198.10	12.63	12.63	12.59	12.68	12.58	12.66	12.57	12.65	12.63	12.57	12.56
211.46	12.90	12.79	12.73	12.82	12.73	12.79	12.78	12.82	12.78	12.71	12.73
225.75	12.86	12.82	12.76	12.77	12.72	12.78	12.71	12.74	12.80	12.72	12.66
241.01	12.67	12.84	12.67	12.58	12.65	12.68	12.56	12.68	12.61	12.56	12.65
257.33	12.88	12.85	12.82	12.75	12.76	12.81	12.81	12.77	12.79	12.71	12.78
274.77	13.05	13.02	12.93	12.92	12.87	12.93	12.87	12.96	12.82	12.75	12.77

293.42	13.03	12.91	12.88	12.94	12.81	12.87	12.84	12.99	12.85	12.82	12.84
313.35	12.84	12.82	12.66	12.71	12.67	12.71	12.69	12.71	12.58	12.57	12.60
334.66	13.11	13.02	13.04	12.93	12.95	13.02	12.96	13.01	12.93	12.85	12.79
357.43	12.92	12.95	12.93	12.89	12.86	12.85	12.87	12.99	12.90	12.95	12.81
381.78	12.68	12.65	12.51	12.63	12.52	12.66	12.53	12.65	12.59	12.53	12.62
407.80	12.75	12.75	12.70	12.63	12.67	12.67	12.63	12.67	12.63	12.63	12.59
435.62	12.92	13.00	12.93	12.91	12.86	12.87	12.85	12.93	12.84	12.81	12.75
465.35	12.87	12.85	12.84	12.84	12.69	12.85	12.84	12.74	12.78	12.66	12.76
497.14	12.83	12.86	12.80	12.81	12.70	12.72	12.82	12.69	12.75	12.67	12.65
531.11	12.55	12.50	12.60	12.55	12.47	12.49	12.46	12.46	12.45	12.49	12.48
567.43	12.91	12.83	12.93	12.77	12.69	12.76	12.79	12.75	12.69	12.68	12.81
606.26	12.91	12.92	12.90	12.76	12.82	12.85	12.79	12.82	12.73	12.71	12.66
647.76	12.73	12.68	12.65	12.65	12.57	12.65	12.65	12.74	12.66	12.58	12.59
692.12	12.46	12.39	12.27	12.31	12.37	12.31	12.32	12.34	12.26	12.16	12.29
739.53	12.84	12.73	12.66	12.64	12.68	12.73	12.60	12.62	12.58	12.54	12.49
790.22	12.72	12.70	12.61	12.59	12.47	12.55	12.51	12.54	12.41	12.50	12.33
844.40	12.68	12.62	12.60	12.70	12.53	12.54	12.44	12.53	12.53	12.46	12.44
902.32	12.61	12.59	12.53	12.50	12.56	12.55	12.51	12.51	12.52	12.46	12.39
964.23	12.35	12.45	12.31	12.36	12.26	12.26	12.20	12.30	12.19	12.21	12.16
1030.41	12.50	12.45	12.40	12.53	12.44	12.33	12.41	12.45	12.37	12.31	12.31
1101.15	12.51	12.49	12.46	12.36	12.37	12.32	12.28	12.28	12.27	12.25	12.24
1176.77	12.53	12.50	12.36	12.37	12.28	12.32	12.20	12.32	12.25	12.25	12.19
1257.60	12.37	12.45	12.35	12.35	12.39	12.36	12.29	12.24	12.19	12.19	12.15
1344.00	12.31	12.34	12.27	12.27	12.17	12.24	12.23	12.26	12.21	12.18	12.05
1436.36	12.35	12.25	12.09	12.13	12.10	12.07	12.03	12.10	12.09	12.01	11.90
1535.09	12.36	12.37	12.25	12.24	12.27	12.14	12.16	12.18	12.22	12.18	12.04
1640.63	12.19	12.22	12.08	12.10	12.12	12.10	12.13	12.03	12.05	11.89	11.93
1753.44	12.15	12.18	12.05	12.06	12.07	12.05	12.03	12.04	11.94	11.92	11.91
1874.02	12.15	12.13	11.98	11.95	11.95	11.90	11.87	11.90	11.87	11.84	11.82
2002.92	11.97	11.99	11.90	11.86	11.87	11.91	11.91	11.90	11.83	11.76	11.83
2140.71	11.81	11.78	11.76	11.71	11.67	11.75	11.63	11.70	11.71	11.65	11.66
2288.00	11.80	11.83	11.77	11.70	11.71	11.61	11.70	11.62	11.56	11.58	11.51
2445.44	11.83	11.75	11.55	11.65	11.49	11.58	11.58	11.52	11.57	11.45	11.45
2613.73	11.68	11.68	11.61	11.64	11.63	11.51	11.54	11.58	11.51	11.53	11.37
2793.63	11.41	11.41	11.34	11.22	11.26	11.25	11.16	11.18	11.15	11.15	11.15
2985.93	11.19	11.15	11.00	11.03	11.06	10.97	10.93	11.04	10.99	10.87	10.84
3191.49	11.08	11.05	10.94	10.97	10.94	10.90	10.86	10.87	10.76	10.79	10.78
3411.21	10.87	10.86	10.75	10.70	10.62	10.72	10.61	10.67	10.62	10.52	10.51
3646.09	10.74	10.68	10.64	10.61	10.64	10.51	10.49	10.48	10.47	10.38	10.45
3897.16	10.39	10.45	10.39	10.30	10.31	10.29	10.26	10.28	10.26	10.20	10.25

Wavelength (nm) / Time (ps)	616.05	617.48	618.90	620.32	621.75	623.17	624.60	626.02	627.44	628.87	630.29
-2.74	0.03	0.11	0.06	0.03	0.00	0.04	0.06	0.04	0.05	-0.02	0.15
-2.24	-0.07	-0.10	-0.04	0.01	-0.04	0.01	0.00	0.03	-0.04	0.01	-0.01
-1.74	-0.04	0.08	-0.10	0.02	0.05	-0.01	0.02	-0.08	0.09	0.00	-0.01
-1.24	0.02	-0.01	0.02	-0.01	0.03	-0.02	-0.02	0.00	0.05	-0.01	-0.12
-0.74	0.10	0.06	0.09	-0.02	0.11	0.07	0.11	0.13	-0.04	0.07	-0.03
-0.24	0.02	0.03	-0.04	-0.03	-0.02	-0.03	-0.09	-0.11	-0.04	0.00	-0.01

-0.14	0.11	0.00	0.05	0.04	-0.03	-0.03	-0.04	-0.01	-0.04	0.03	-0.05
-0.04	-0.16	-0.17	-0.05	-0.05	-0.10	-0.03	-0.04	0.00	-0.04	-0.08	0.08
0.06	-0.03	-0.01	0.07	0.03	0.00	0.00	-0.08	0.04	0.13	0.09	-0.01
0.16	0.07	0.11	0.09	0.07	0.15	0.18	0.03	0.20	0.14	0.20	0.13
0.26	0.01	0.09	-0.05	0.09	0.01	0.04	-0.01	0.10	0.02	0.07	0.08
0.31	-0.01	-0.09	-0.07	-0.03	0.03	-0.04	-0.03	-0.02	-0.05	0.10	0.09
0.36	0.04	0.07	0.02	0.10	0.05	-0.10	-0.01	0.05	-0.05	-0.05	-0.02
0.41	0.00	-0.13	0.01	0.03	0.06	-0.09	0.12	0.03	0.11	0.09	-0.09
0.46	-0.10	-0.03	0.01	0.02	-0.02	-0.09	0.03	-0.02	0.05	0.10	0.00
0.51	0.00	-0.04	0.01	-0.01	0.02	0.00	0.09	0.05	-0.08	0.03	-0.01
0.56	0.02	0.04	0.00	0.14	-0.01	0.07	0.02	0.12	0.03	0.13	-0.01
0.61	0.04	0.10	0.05	0.12	0.00	0.02	0.10	0.13	0.01	0.10	0.04
0.66	0.09	0.14	0.09	0.03	-0.06	-0.05	0.13	0.14	0.06	0.06	0.08
0.71	-0.04	0.03	-0.04	0.09	0.13	0.04	0.07	0.09	0.09	0.17	0.13
0.76	0.10	0.06	0.03	0.03	0.08	0.07	0.09	0.11	0.15	0.05	0.09
0.81	0.06	0.12	0.06	0.15	0.07	0.17	0.12	0.16	0.11	0.13	0.18
0.86	-0.10	-0.02	-0.08	-0.01	0.05	0.07	0.03	0.01	0.01	0.09	0.01
0.91	0.01	0.10	0.04	0.14	0.05	0.11	0.04	0.07	0.11	0.13	0.09
0.96	-0.01	-0.02	0.02	0.03	0.04	0.10	0.08	0.10	0.05	0.09	0.07
1.01	0.02	0.00	-0.07	-0.02	0.05	0.06	0.09	-0.01	-0.02	0.09	0.07
1.06	-0.07	-0.01	-0.01	0.08	0.07	-0.03	0.07	0.03	0.00	0.13	0.01
1.11	-0.02	0.05	0.09	-0.04	0.02	0.18	0.13	-0.01	0.18	0.14	-0.04
1.16	0.07	0.02	0.02	0.03	0.06	-0.06	0.18	0.05	0.15	0.13	0.13
1.21	-0.11	-0.01	0.07	0.13	0.00	-0.09	0.04	0.24	0.22	0.13	-0.04
1.26	-0.19	0.11	0.26	0.11	-0.19	-0.33	-0.32	-0.04	0.28	0.38	0.13
1.31	-0.64	-0.99	-1.45	-1.81	-1.84	-1.67	-0.87	-0.04	0.76	1.35	1.42
1.36	-43.15	-48.82	-53.69	-57.62	-60.24	-61.39	-60.73	-58.58	-54.84	-50.32	-45.03
1.41	143.78	141.36	137.64	132.51	126.42	119.83	113.07	106.12	99.46	93.23	87.61
1.46	-36.00	-31.76	-27.05	-21.68	-16.09	-10.25	-4.04	2.42	8.75	15.47	22.04
1.51	21.12	21.16	21.28	21.33	21.36	21.39	21.48	21.47	21.36	21.17	20.68
1.56	14.02	14.23	14.29	14.38	14.45	14.81	15.14	15.61	16.08	16.50	17.04
1.61	16.44	16.50	16.27	15.94	15.66	15.59	15.53	15.26	14.78	14.46	14.28
1.66	15.19	15.51	15.82	16.07	16.32	16.58	16.78	16.88	17.08	17.23	17.15
1.71	13.98	13.80	13.63	13.54	13.37	13.21	13.09	13.07	12.98	13.08	13.16
1.76	15.40	15.62	15.67	15.70	15.60	15.57	15.51	15.37	15.16	14.94	14.85
1.81	12.63	12.86	13.15	13.44	13.58	13.87	14.08	14.40	14.55	14.92	15.14
1.86	13.38	13.26	13.13	13.14	12.85	12.76	12.70	12.66	12.55	12.62	12.63
1.91	13.95	14.26	14.26	14.57	14.66	14.74	14.72	14.72	14.62	14.68	14.68
1.96	12.33	12.49	12.49	12.61	12.68	12.84	13.09	13.25	13.46	13.72	13.83
2.01	13.80	13.89	13.75	13.57	13.32	13.30	13.15	12.98	12.74	12.56	12.33
2.06	12.89	13.12	13.38	13.62	13.77	14.00	14.17	14.37	14.47	14.63	14.81
2.11	12.47	12.44	12.45	12.47	12.43	12.42	12.43	12.52	12.61	12.74	12.93
2.16	13.84	13.91	13.94	13.99	13.90	13.85	13.77	13.74	13.61	13.61	13.45
2.21	12.62	12.79	12.89	13.09	13.29	13.42	13.61	13.76	13.95	14.23	14.30
2.26	13.07	13.01	13.00	12.95	12.77	12.64	12.62	12.65	12.57	12.59	12.62
2.31	12.99	13.25	13.36	13.48	13.54	13.53	13.59	13.60	13.65	13.58	13.53
2.36	12.15	12.28	12.42	12.57	12.59	12.75	12.93	13.06	13.17	13.40	13.35
2.41	13.12	13.05	12.97	12.93	12.85	12.74	12.72	12.59	12.50	12.44	12.38
2.46	12.40	12.61	12.78	12.85	12.94	13.06	13.21	13.32	13.33	13.41	13.39
2.51	12.52	12.59	12.46	12.49	12.32	12.38	12.37	12.43	12.43	12.64	12.70

2.56	13.07	13.21	13.13	13.21	13.23	13.13	13.01	12.96	13.03	12.88	12.72
2.61	12.30	12.40	12.51	12.74	12.85	12.95	12.99	13.08	13.22	13.33	13.33
2.66	12.46	12.48	12.51	12.34	12.36	12.29	12.41	12.37	12.36	12.46	12.53
2.71	12.79	12.86	12.87	13.02	13.02	12.87	12.94	13.01	12.88	12.84	12.83
2.76	12.17	12.19	12.24	12.39	12.44	12.57	12.63	12.80	12.86	13.00	13.20
2.81	12.34	12.48	12.33	12.31	12.31	12.22	12.17	12.13	12.16	12.11	12.15
2.86	12.32	12.50	12.52	12.64	12.73	12.70	12.64	12.68	12.73	12.67	12.77
2.91	12.21	12.25	12.25	12.38	12.32	12.37	12.41	12.46	12.51	12.65	12.69
2.96	12.74	12.92	12.89	12.88	12.92	12.75	12.71	12.68	12.71	12.71	12.56
3.01	12.27	12.34	12.47	12.52	12.60	12.76	12.70	12.81	12.88	13.01	13.11
3.06	11.94	12.02	11.85	12.00	11.91	11.94	11.87	11.91	11.85	12.02	12.12
3.11	12.50	12.63	12.59	12.67	12.64	12.71	12.57	12.66	12.62	12.53	12.45
3.16	12.10	12.15	12.24	12.36	12.32	12.42	12.48	12.51	12.65	12.72	12.69
3.21	12.04	12.10	12.08	12.10	12.09	12.13	12.15	12.16	12.13	12.25	12.17
3.26	12.24	12.35	12.36	12.40	12.42	12.51	12.44	12.57	12.44	12.55	12.52
3.31	12.04	11.99	12.08	12.07	12.18	12.24	12.27	12.35	12.33	12.55	12.59
3.36	12.44	12.63	12.53	12.40	12.39	12.25	12.35	12.24	12.25	12.30	12.26
3.41	12.32	12.56	12.56	12.72	12.66	12.78	12.72	12.79	12.79	12.98	13.06
3.46	12.03	11.97	12.00	12.18	12.09	12.10	12.07	12.21	12.13	12.28	12.32
3.51	12.35	12.38	12.45	12.56	12.49	12.48	12.42	12.52	12.42	12.41	12.45
3.56	11.94	12.01	12.04	12.21	12.17	12.34	12.37	12.38	12.51	12.46	12.49
3.61	11.70	11.89	11.80	11.76	11.83	11.85	11.90	11.75	11.83	11.99	11.99
3.66	12.14	12.02	12.13	12.20	12.29	12.19	12.34	12.38	12.33	12.33	12.32
3.71	12.09	12.16	12.12	12.31	12.30	12.29	12.45	12.47	12.53	12.55	12.58
3.76	12.62	12.75	12.72	12.79	12.73	12.74	12.76	12.82	12.80	12.74	12.77
3.81	11.92	11.97	12.05	12.09	12.20	12.14	12.26	12.31	12.39	12.28	12.35
3.86	11.66	11.73	11.65	11.81	11.75	11.81	11.81	11.88	11.83	11.92	11.91
3.91	12.49	12.52	12.61	12.67	12.57	12.62	12.61	12.63	12.59	12.59	12.66
3.96	11.97	12.06	12.12	12.16	12.09	12.22	12.24	12.29	12.37	12.38	12.49
4.01	12.20	12.28	12.25	12.33	12.23	12.27	12.35	12.25	12.31	12.36	12.28
4.06	12.44	12.58	12.53	12.57	12.59	12.60	12.59	12.64	12.63	12.65	12.72
4.11	12.05	12.22	12.19	12.26	12.28	12.37	12.32	12.32	12.31	12.41	12.54
4.16	12.33	12.42	12.47	12.43	12.53	12.49	12.41	12.42	12.46	12.47	12.42
4.21	12.55	12.63	12.65	12.70	12.74	12.73	12.79	12.81	12.75	12.83	12.82
4.26	12.01	12.11	12.10	12.15	12.14	12.23	12.19	12.26	12.20	12.29	12.33
4.31	12.13	12.09	12.27	12.19	12.15	12.17	12.21	12.17	12.28	12.17	12.14
4.36	12.13	12.21	12.26	12.29	12.31	12.28	12.40	12.32	12.37	12.45	12.37
4.41	11.95	11.96	11.99	12.02	12.03	12.05	12.07	12.05	12.09	12.16	12.26
4.46	12.39	12.45	12.43	12.57	12.55	12.55	12.58	12.60	12.57	12.55	12.70
4.51	12.09	12.17	12.16	12.18	12.18	12.17	12.28	12.19	12.28	12.47	12.39
4.56	12.05	12.05	12.17	12.18	12.14	12.18	12.21	12.15	12.24	12.33	12.21
4.61	12.07	12.09	12.10	12.25	12.24	12.11	12.28	12.31	12.30	12.20	12.24
4.66	12.08	12.21	12.25	12.27	12.31	12.37	12.34	12.34	12.44	12.44	12.40
4.71	12.22	12.26	12.35	12.42	12.26	12.37	12.38	12.50	12.46	12.42	12.39
4.76	12.36	12.51	12.50	12.59	12.57	12.63	12.61	12.61	12.57	12.74	12.67
4.81	12.03	12.05	12.15	12.13	12.15	12.17	12.19	12.30	12.18	12.21	12.18
4.86	12.13	12.16	12.16	12.25	12.29	12.26	12.22	12.23	12.21	12.23	12.24
4.91	12.29	12.42	12.45	12.38	12.40	12.55	12.42	12.43	12.54	12.64	12.58
4.96	12.02	12.13	12.13	12.27	12.15	12.20	12.22	12.27	12.22	12.31	12.36
5.01	12.05	12.09	12.19	12.23	12.17	12.15	12.28	12.29	12.25	12.28	12.37

5.06	12.08	12.23	12.17	12.28	12.24	12.21	12.29	12.34	12.35	12.36	12.31
5.11	12.06	12.10	12.09	12.18	12.12	12.14	12.07	12.18	12.22	12.25	12.20
5.16	11.91	11.96	12.00	12.07	12.13	11.96	12.11	12.18	12.13	12.26	12.25
5.21	11.98	11.94	11.97	12.01	12.02	12.09	12.07	12.15	12.12	12.14	12.09
5.26	12.36	12.47	12.49	12.54	12.53	12.55	12.61	12.52	12.45	12.48	12.49
5.31	12.23	12.22	12.39	12.38	12.32	12.43	12.46	12.47	12.44	12.51	12.58
5.38	12.10	12.23	12.21	12.34	12.30	12.35	12.33	12.30	12.28	12.47	12.32
5.45	12.43	12.48	12.54	12.61	12.62	12.55	12.57	12.64	12.66	12.72	12.53
5.53	12.02	12.15	12.14	12.21	12.21	12.25	12.24	12.25	12.31	12.28	12.35
5.61	12.38	12.51	12.40	12.54	12.47	12.53	12.52	12.58	12.54	12.49	12.54
5.70	12.27	12.36	12.38	12.40	12.43	12.48	12.42	12.52	12.49	12.51	12.49
5.80	12.39	12.40	12.42	12.49	12.49	12.47	12.36	12.50	12.42	12.45	12.52
5.90	11.84	11.90	11.87	11.91	11.84	11.89	11.96	11.95	12.00	12.05	12.03
6.01	12.48	12.66	12.56	12.60	12.67	12.63	12.57	12.69	12.66	12.81	12.67
6.13	12.09	12.16	12.24	12.30	12.25	12.11	12.18	12.14	12.20	12.24	12.32
6.25	11.89	12.00	12.07	12.10	12.07	12.08	12.11	12.09	12.06	12.09	12.15
6.39	11.94	12.01	11.99	12.05	11.94	12.07	12.04	12.06	12.00	12.08	12.00
6.53	11.91	11.99	12.01	12.01	12.03	12.02	12.08	12.01	11.96	12.12	12.02
6.69	12.07	12.22	12.18	12.27	12.23	12.30	12.32	12.31	12.28	12.35	12.30
6.85	11.83	12.01	12.03	11.98	11.98	12.07	12.10	12.05	11.99	12.16	12.08
7.03	11.97	12.11	12.09	12.08	12.00	12.12	12.01	12.09	12.10	12.16	12.19
7.21	12.05	12.09	12.21	12.19	12.16	12.27	12.18	12.24	12.28	12.31	12.25
7.41	12.11	12.21	12.23	12.34	12.26	12.25	12.30	12.29	12.33	12.22	12.34
7.63	12.48	12.54	12.49	12.57	12.55	12.49	12.55	12.58	12.44	12.53	12.51
7.86	11.96	12.14	12.17	12.10	12.13	12.22	12.18	12.17	12.20	12.26	12.22
8.10	12.07	12.22	12.17	12.28	12.24	12.23	12.24	12.26	12.16	12.28	12.17
8.36	11.69	11.71	11.81	11.72	11.79	11.70	11.79	11.84	11.73	11.89	11.78
8.64	11.95	12.12	12.03	12.13	12.18	12.12	12.13	12.19	12.17	12.12	12.11
8.94	12.24	12.31	12.35	12.33	12.27	12.32	12.37	12.33	12.39	12.31	12.42
9.26	12.49	12.51	12.43	12.64	12.59	12.53	12.50	12.60	12.50	12.58	12.50
9.60	12.11	12.13	12.19	12.28	12.23	12.14	12.28	12.28	12.24	12.31	12.22
9.97	12.09	12.14	12.09	12.23	12.14	12.12	12.05	12.17	12.12	12.27	12.09
10.36	12.26	12.30	12.37	12.46	12.42	12.39	12.39	12.43	12.43	12.41	12.36
10.77	12.21	12.25	12.28	12.31	12.25	12.33	12.23	12.29	12.26	12.21	12.24
11.22	11.95	11.94	12.01	11.94	12.05	12.02	11.96	11.98	12.03	12.07	11.94
11.70	12.12	12.22	12.28	12.33	12.30	12.29	12.33	12.34	12.24	12.33	12.27
12.21	11.87	11.82	11.92	11.97	11.91	11.96	11.88	12.06	12.00	12.09	11.98
12.75	12.07	12.21	12.16	12.24	12.26	12.25	12.26	12.20	12.25	12.23	12.23
13.33	12.22	12.18	12.22	12.35	12.24	12.33	12.32	12.31	12.29	12.41	12.30
13.95	12.05	12.11	12.10	12.20	12.22	12.08	12.18	12.18	12.09	12.13	12.17
14.62	11.82	11.90	11.93	12.02	11.96	11.99	12.04	12.08	12.05	12.10	12.03
15.33	11.79	11.88	11.87	11.87	11.87	11.77	11.85	11.93	11.92	11.97	11.89
16.09	11.83	11.99	11.93	11.96	11.99	11.99	11.95	11.97	12.00	12.05	12.07
16.90	12.02	11.98	12.10	12.06	12.08	12.08	12.08	12.11	12.12	12.08	12.07
17.77	11.89	11.97	11.93	11.94	11.93	11.93	11.98	12.04	11.92	11.95	11.95
18.70	11.80	11.80	11.87	11.95	11.90	11.71	11.90	11.98	11.88	11.84	11.88
19.69	11.99	11.99	12.00	12.10	12.05	12.10	12.04	12.07	12.06	12.10	12.04
20.75	11.82	11.88	11.94	11.97	12.00	11.93	11.89	11.80	11.93	11.98	11.93
21.89	12.21	12.23	12.19	12.22	12.23	12.15	12.25	12.15	12.26	12.24	12.25
23.10	12.40	12.38	12.44	12.39	12.47	12.37	12.43	12.50	12.50	12.52	12.41

24.39	11.91	11.95	12.04	11.98	12.00	12.06	12.03	11.95	11.98	12.00	11.93
25.78	12.06	12.09	12.23	12.17	12.17	12.15	12.12	12.13	12.19	12.21	12.26
27.26	12.01	12.06	12.01	12.14	12.08	12.09	12.12	12.11	12.00	12.06	12.01
28.84	12.10	12.04	12.13	12.19	12.11	12.12	12.11	12.15	12.05	12.18	12.23
30.53	12.13	12.19	12.15	12.13	12.12	12.14	12.13	12.20	12.10	12.15	12.17
32.34	11.97	11.87	11.95	11.98	12.01	11.95	11.98	11.96	12.03	11.97	11.96
34.27	12.07	12.08	12.12	12.21	12.17	12.17	12.24	12.21	12.21	12.25	12.17
36.34	11.93	11.99	11.93	12.09	12.04	11.91	12.04	11.95	12.01	12.00	12.01
38.55	12.13	12.15	12.17	12.22	12.23	12.16	12.19	12.20	12.15	12.29	12.12
40.91	11.95	12.12	12.14	12.04	12.00	12.05	12.04	12.11	12.04	12.02	12.03
43.43	11.79	11.74	11.76	11.91	11.72	11.79	11.75	11.83	11.72	11.84	11.77
46.13	11.82	11.94	11.90	11.83	11.95	11.92	11.99	11.92	11.95	11.95	11.99
49.01	12.23	12.35	12.31	12.35	12.27	12.30	12.32	12.32	12.28	12.25	12.25
52.09	12.01	11.98	12.11	12.08	12.05	12.05	12.01	12.09	12.03	12.05	11.97
55.39	11.90	11.96	11.98	12.01	11.98	11.88	11.90	12.01	12.07	12.02	11.97
58.91	11.79	11.91	11.91	11.94	11.92	12.01	11.92	11.91	11.97	11.98	11.93
62.68	12.01	12.06	12.05	12.08	12.12	11.95	12.06	12.06	12.12	12.04	12.07
66.70	12.04	12.09	12.14	12.22	12.18	12.12	12.10	12.11	12.07	12.17	12.08
71.00	12.06	12.14	12.11	12.06	12.15	12.07	12.12	12.13	12.19	12.07	12.19
75.60	12.01	12.01	12.01	12.03	12.07	11.97	11.99	12.05	11.90	12.05	11.95
80.51	12.15	12.17	12.19	12.19	12.17	12.14	12.24	12.29	12.23	12.23	12.27
85.77	12.38	12.39	12.38	12.39	12.39	12.44	12.30	12.33	12.30	12.29	12.27
91.38	12.31	12.47	12.32	12.45	12.35	12.39	12.44	12.47	12.39	12.45	12.35
97.39	12.10	12.11	12.19	12.23	12.14	12.21	12.13	12.21	12.13	12.16	12.13
103.80	12.63	12.65	12.60	12.57	12.64	12.72	12.72	12.76	12.60	12.67	12.64
110.66	12.33	12.27	12.26	12.34	12.25	12.23	12.32	12.23	12.26	12.23	12.26
117.99	12.15	12.14	12.21	12.22	12.10	12.04	12.11	12.04	12.07	12.16	12.13
125.83	12.45	12.54	12.43	12.56	12.47	12.55	12.56	12.58	12.53	12.44	12.41
134.21	12.31	12.42	12.31	12.37	12.32	12.29	12.32	12.28	12.34	12.39	12.25
143.17	12.27	12.41	12.33	12.36	12.33	12.34	12.35	12.27	12.24	12.30	12.23
152.74	12.35	12.44	12.43	12.51	12.38	12.39	12.39	12.50	12.40	12.46	12.35
162.97	12.56	12.50	12.57	12.56	12.52	12.51	12.44	12.52	12.45	12.45	12.46
173.91	12.48	12.42	12.49	12.51	12.54	12.49	12.45	12.34	12.28	12.43	12.42
185.60	12.56	12.68	12.67	12.68	12.62	12.67	12.55	12.71	12.64	12.55	12.57
198.10	12.50	12.65	12.50	12.65	12.57	12.50	12.50	12.60	12.39	12.50	12.47
211.46	12.64	12.77	12.70	12.74	12.72	12.70	12.75	12.73	12.71	12.81	12.72
225.75	12.66	12.63	12.65	12.67	12.65	12.61	12.68	12.67	12.49	12.65	12.47
241.01	12.53	12.62	12.67	12.63	12.60	12.67	12.61	12.59	12.49	12.54	12.50
257.33	12.61	12.67	12.67	12.73	12.63	12.65	12.65	12.61	12.60	12.52	12.52
274.77	12.82	12.79	12.74	12.79	12.72	12.73	12.81	12.72	12.65	12.63	12.61
293.42	12.76	12.77	12.79	12.76	12.80	12.74	12.79	12.77	12.72	12.69	12.64
313.35	12.60	12.59	12.61	12.60	12.57	12.62	12.71	12.61	12.43	12.58	12.44
334.66	12.85	12.86	12.89	12.74	12.88	12.80	12.75	12.83	12.62	12.68	12.66
357.43	12.87	12.71	12.75	12.72	12.72	12.71	12.75	12.66	12.62	12.73	12.67
381.78	12.46	12.54	12.48	12.50	12.41	12.41	12.44	12.42	12.39	12.32	12.32
407.80	12.47	12.49	12.55	12.68	12.49	12.39	12.54	12.55	12.44	12.42	12.44
435.62	12.62	12.71	12.68	12.71	12.65	12.55	12.55	12.47	12.46	12.52	12.51
465.35	12.59	12.65	12.60	12.71	12.63	12.63	12.59	12.59	12.51	12.63	12.42
497.14	12.56	12.68	12.67	12.60	12.46	12.51	12.54	12.55	12.45	12.36	12.39
531.11	12.34	12.25	12.31	12.35	12.30	12.28	12.27	12.23	12.16	12.15	12.20

567.43	12.64	12.65	12.63	12.67	12.54	12.52	12.46	12.53	12.44	12.44	12.32
606.26	12.68	12.65	12.58	12.74	12.64	12.52	12.56	12.48	12.55	12.40	12.43
647.76	12.48	12.49	12.52	12.51	12.49	12.52	12.44	12.43	12.47	12.43	12.43
692.12	12.20	12.18	12.29	12.15	12.16	11.99	12.07	11.95	11.99	11.98	11.92
739.53	12.48	12.50	12.57	12.45	12.52	12.48	12.44	12.42	12.41	12.30	12.40
790.22	12.39	12.29	12.32	12.35	12.21	12.18	12.16	12.26	12.06	12.07	12.13
844.40	12.36	12.44	12.33	12.29	12.43	12.29	12.25	12.26	12.24	12.25	12.16
902.32	12.41	12.44	12.37	12.37	12.33	12.24	12.29	12.27	12.17	12.17	12.19
964.23	12.01	12.01	12.13	12.10	12.06	12.03	11.96	11.99	11.81	11.94	11.80
1030.41	12.28	12.26	12.26	12.24	12.29	12.25	12.18	12.18	12.11	12.11	12.03
1101.15	12.16	12.16	12.13	12.25	12.05	12.07	12.01	11.99	11.97	11.90	11.86
1176.77	12.11	12.24	12.04	12.11	12.17	11.98	11.98	11.97	11.98	11.91	11.90
1257.60	12.08	12.23	12.18	12.06	11.99	12.02	12.01	11.96	11.91	12.00	11.88
1344.00	11.99	12.03	12.04	12.03	12.00	11.84	11.77	11.83	11.80	11.80	11.74
1436.36	11.91	11.93	11.82	11.87	11.83	11.91	11.83	11.72	11.64	11.69	11.56
1535.09	12.06	12.06	11.91	11.95	11.95	11.87	11.95	11.76	11.76	11.73	11.64
1640.63	11.80	11.75	11.79	11.76	11.77	11.65	11.63	11.61	11.54	11.60	11.44
1753.44	11.78	11.83	11.84	11.86	11.77	11.69	11.63	11.63	11.49	11.56	11.50
1874.02	11.72	11.68	11.55	11.56	11.47	11.49	11.43	11.48	11.38	11.37	11.26
2002.92	11.75	11.70	11.66	11.77	11.61	11.64	11.57	11.63	11.40	11.43	11.57
2140.71	11.58	11.55	11.49	11.41	11.46	11.45	11.31	11.28	11.26	11.26	11.17
2288.00	11.43	11.50	11.45	11.42	11.42	11.35	11.34	11.22	11.16	11.13	11.09
2445.44	11.40	11.32	11.34	11.41	11.33	11.12	11.17	11.17	11.08	11.09	11.01
2613.73	11.37	11.37	11.42	11.34	11.21	11.25	11.19	11.03	11.10	11.09	10.99
2793.63	11.08	11.07	10.96	10.99	11.01	10.97	10.93	10.92	10.81	10.82	10.66
2985.93	10.81	10.86	10.80	10.75	10.71	10.72	10.64	10.59	10.49	10.56	10.38
3191.49	10.69	10.62	10.61	10.54	10.49	10.47	10.53	10.47	10.32	10.32	10.27
3411.21	10.46	10.42	10.46	10.45	10.27	10.38	10.35	10.27	10.24	10.13	10.06
3646.09	10.34	10.29	10.17	10.28	10.12	10.05	9.95	10.04	9.95	9.94	9.85
3897.16	10.13	10.18	10.08	10.07	9.86	9.86	9.86	9.84	9.68	9.69	9.61

Wavelength (nm) / Time (ps)	631.72	633.14	634.56	635.99	637.41	638.84	640.26	641.68	643.11	644.53	645.96
-2.74	0.06	-0.02	-0.03	0.00	-0.04	0.03	0.02	0.07	0.09	-0.02	-0.02
-2.24	-0.09	0.07	0.06	0.08	0.10	0.04	-0.01	0.05	-0.02	-0.11	0.02
-1.74	0.12	0.06	0.06	0.02	0.22	0.15	0.07	0.15	0.21	0.13	0.10
-1.24	-0.05	-0.08	-0.04	-0.07	-0.01	-0.04	0.01	-0.10	-0.02	-0.03	-0.07
-0.74	0.00	0.04	0.03	0.08	-0.03	-0.03	-0.01	-0.07	-0.06	0.04	-0.11
-0.24	-0.03	-0.08	-0.04	0.01	-0.10	0.00	-0.01	0.04	-0.05	0.06	0.06
-0.14	0.02	0.04	-0.06	0.03	-0.07	-0.06	-0.03	-0.06	-0.07	-0.05	-0.03
-0.04	-0.04	-0.03	0.01	-0.14	-0.07	-0.08	-0.04	-0.08	-0.08	0.00	0.05
0.06	0.06	-0.02	0.03	0.02	0.00	0.05	0.02	-0.06	-0.03	-0.03	0.01
0.16	0.23	0.17	0.23	0.29	0.20	0.18	0.13	0.20	0.18	0.17	0.21
0.26	0.14	-0.01	0.08	0.06	0.04	-0.01	0.12	0.06	0.01	-0.07	0.09
0.31	-0.06	0.06	-0.01	0.06	0.11	0.07	0.07	-0.03	-0.03	-0.06	-0.06
0.36	-0.03	-0.03	-0.05	0.14	0.06	0.06	-0.01	0.00	-0.08	-0.02	-0.02
0.41	-0.02	-0.01	-0.03	0.03	0.01	-0.05	-0.02	0.02	-0.08	0.03	0.00
0.46	0.02	-0.02	0.08	0.02	0.00	-0.07	-0.04	-0.07	-0.07	-0.01	0.03
0.51	-0.07	0.03	0.01	-0.03	0.01	-0.03	0.01	-0.08	-0.10	-0.07	0.00

0.56	0.00	-0.01	0.08	0.07	0.07	0.03	0.04	0.17	0.11	-0.05	-0.04
0.61	0.05	0.01	0.11	0.02	0.05	0.03	0.01	0.04	-0.01	-0.02	-0.09
0.66	0.13	0.06	0.01	0.05	0.06	0.03	0.01	0.07	0.02	0.04	0.03
0.71	0.03	0.03	-0.06	0.07	0.04	0.02	0.04	-0.01	-0.11	0.04	0.03
0.76	0.10	0.03	0.23	0.11	0.13	0.13	0.11	0.14	0.12	0.13	0.14
0.81	0.08	0.18	0.07	0.15	0.05	0.02	0.13	0.22	0.08	0.07	0.06
0.86	0.13	0.05	0.06	0.01	0.17	-0.02	0.17	0.11	0.08	0.17	0.11
0.91	0.10	0.16	0.12	0.05	0.23	0.06	0.18	0.13	0.06	0.05	0.09
0.96	0.06	-0.04	0.15	0.11	0.01	0.12	0.01	0.00	-0.03	0.03	0.06
1.01	0.10	0.01	0.09	0.04	0.17	0.10	0.05	0.08	-0.01	0.06	0.02
1.06	-0.03	0.11	-0.04	0.00	0.13	-0.08	0.03	-0.05	-0.11	0.03	0.06
1.11	0.08	0.02	-0.05	0.07	0.11	-0.06	-0.03	0.02	0.00	-0.11	0.05
1.16	0.14	0.16	-0.03	0.05	0.13	0.07	0.07	0.04	0.08	0.13	-0.01
1.21	0.00	0.31	0.18	-0.04	-0.08	0.12	0.23	0.30	0.05	0.01	0.09
1.26	-0.17	-0.24	-0.19	0.09	0.28	0.17	-0.04	0.00	-0.18	-0.17	-0.17
1.31	1.09	0.51	-0.13	-0.66	-0.92	-1.09	-1.02	-0.68	-0.38	-0.07	0.10
1.36	-39.64	-34.30	-29.25	-24.92	-21.31	-18.33	-16.02	-14.27	-13.02	-12.04	-11.25
1.41	82.94	78.92	75.33	71.98	68.73	65.29	61.51	58.13	54.05	50.29	45.75
1.46	29.30	36.48	43.48	50.31	57.03	63.11	69.13	74.43	78.35	83.60	85.17
1.51	20.03	19.33	18.34	17.17	15.95	14.63	13.12	11.53	10.09	8.39	6.83
1.56	17.62	18.20	18.68	19.22	19.65	20.26	20.61	21.01	21.18	21.49	21.33
1.61	14.27	14.48	14.27	13.90	13.78	13.94	14.40	15.14	15.56	15.55	15.47
1.66	17.25	17.17	17.06	17.01	16.82	16.50	16.17	15.84	15.31	15.17	14.60
1.71	13.20	13.28	13.59	13.79	14.06	14.43	14.75	15.14	15.35	15.87	16.07
1.76	14.56	14.33	14.09	13.80	13.50	13.27	13.05	12.92	12.71	12.66	12.52
1.81	15.33	15.56	15.62	15.65	15.81	15.66	15.58	15.60	15.37	15.18	14.91
1.86	12.73	12.86	13.24	13.36	13.72	13.99	14.20	14.75	14.85	15.37	15.51
1.91	14.49	14.16	14.06	13.90	13.64	13.40	13.01	12.76	12.38	12.25	11.88
1.96	14.12	14.37	14.60	14.71	14.81	15.12	15.23	15.23	15.15	15.09	14.92
2.01	12.27	12.12	12.01	11.98	12.10	12.14	12.25	12.44	12.56	12.86	13.13
2.06	14.80	14.94	14.83	14.76	14.69	14.58	14.36	14.26	13.91	13.73	13.40
2.11	12.93	13.29	13.51	13.62	14.00	14.20	14.32	14.51	14.70	14.95	14.96
2.16	13.22	13.18	12.95	12.92	12.77	12.56	12.49	12.39	12.19	12.14	12.19
2.21	14.43	14.59	14.50	14.68	14.69	14.64	14.42	14.36	14.19	14.06	13.81
2.26	12.60	12.73	12.84	12.88	13.01	13.21	13.42	13.60	13.79	14.00	14.16
2.31	13.58	13.44	13.33	13.10	13.16	12.91	12.81	12.56	12.47	12.29	12.15
2.36	13.48	13.70	13.69	13.83	13.89	13.86	13.82	13.73	13.64	13.56	13.39
2.41	12.29	12.34	12.38	12.37	12.45	12.51	12.70	12.79	12.86	12.99	13.21
2.46	13.36	13.44	13.42	13.35	13.22	13.06	12.97	12.79	12.62	12.47	12.31
2.51	12.88	12.96	13.15	13.29	13.50	13.64	13.84	14.03	14.20	14.53	14.51
2.56	12.73	12.65	12.67	12.52	12.49	12.45	12.35	12.32	12.35	12.30	12.31
2.61	13.29	13.31	13.36	13.25	13.26	13.12	13.04	12.91	12.61	12.70	12.46
2.66	12.56	12.65	12.76	12.81	13.10	13.20	13.29	13.42	13.51	13.51	13.67
2.71	12.72	12.66	12.53	12.52	12.42	12.34	12.20	12.22	12.10	12.04	12.11
2.76	13.17	13.20	13.40	13.46	13.48	13.52	13.54	13.55	13.39	13.25	13.24
2.81	12.10	12.21	12.21	12.26	12.38	12.52	12.52	12.50	12.65	12.86	12.92
2.86	12.51	12.66	12.60	12.44	12.39	12.44	12.37	12.23	12.22	12.21	12.13
2.91	12.81	12.84	12.98	13.11	13.11	13.32	13.43	13.47	13.51	13.62	13.53
2.96	12.52	12.50	12.47	12.47	12.50	12.59	12.52	12.48	12.44	12.53	12.71
3.01	13.09	13.17	13.17	13.22	13.26	13.18	13.18	13.03	13.02	12.99	12.72

3.06	12.02	12.09	12.18	12.35	12.26	12.43	12.58	12.73	12.60	12.79	12.88
3.11	12.41	12.41	12.25	12.21	12.37	12.35	12.22	12.16	12.19	12.16	12.16
3.16	12.76	12.96	12.99	13.03	13.00	13.16	13.07	13.02	12.86	12.97	12.76
3.21	12.11	12.21	12.32	12.22	12.37	12.40	12.45	12.44	12.46	12.57	12.68
3.26	12.38	12.39	12.44	12.33	12.26	12.25	12.22	12.21	12.16	12.16	12.09
3.31	12.58	12.53	12.71	12.81	12.93	12.84	12.88	13.03	12.95	13.01	12.92
3.36	12.21	12.21	12.09	12.11	12.30	12.29	12.30	12.27	12.34	12.49	12.44
3.41	12.91	12.91	12.86	12.74	12.89	12.69	12.71	12.59	12.50	12.42	12.35
3.46	12.35	12.44	12.51	12.52	12.65	12.71	12.71	12.82	12.77	12.72	12.93
3.51	12.44	12.27	12.26	12.32	12.24	12.21	12.19	12.14	12.06	12.15	12.12
3.56	12.48	12.76	12.69	12.75	12.68	12.74	12.71	12.54	12.62	12.52	12.46
3.61	11.98	12.00	12.04	12.11	12.26	12.20	12.30	12.30	12.55	12.56	12.55
3.66	12.39	12.24	12.30	12.19	12.25	12.22	12.15	12.08	12.00	12.00	11.82
3.71	12.58	12.74	12.73	12.93	12.86	12.90	12.98	13.00	12.94	13.00	13.02
3.76	12.74	12.75	12.82	12.84	12.88	12.90	13.00	12.91	12.76	12.80	12.93
3.81	12.33	12.33	12.35	12.34	12.37	12.31	12.25	12.16	12.15	12.09	12.06
3.86	12.08	11.99	12.17	12.17	12.15	12.29	12.29	12.37	12.35	12.46	12.47
3.91	12.65	12.54	12.49	12.52	12.58	12.55	12.43	12.44	12.39	12.35	12.45
3.96	12.40	12.55	12.58	12.37	12.50	12.35	12.39	12.50	12.33	12.50	12.23
4.01	12.40	12.34	12.46	12.41	12.47	12.59	12.69	12.65	12.67	12.69	12.69
4.06	12.64	12.67	12.63	12.73	12.65	12.54	12.60	12.58	12.58	12.37	12.36
4.11	12.53	12.55	12.48	12.54	12.57	12.57	12.74	12.62	12.63	12.50	12.54
4.16	12.45	12.48	12.50	12.49	12.54	12.48	12.61	12.59	12.52	12.63	12.51
4.21	12.75	12.76	12.77	12.81	12.82	12.79	12.77	12.63	12.74	12.61	12.62
4.26	12.39	12.36	12.41	12.42	12.52	12.50	12.44	12.50	12.54	12.60	12.62
4.31	12.04	12.24	12.14	12.18	12.08	12.09	12.01	12.15	12.18	12.13	12.17
4.36	12.28	12.45	12.38	12.34	12.46	12.33	12.39	12.37	12.26	12.27	12.26
4.41	12.14	12.17	12.23	12.27	12.27	12.24	12.33	12.36	12.27	12.34	12.31
4.46	12.49	12.47	12.58	12.55	12.48	12.60	12.47	12.38	12.39	12.43	12.34
4.51	12.44	12.32	12.44	12.47	12.59	12.53	12.63	12.62	12.54	12.42	12.41
4.56	12.32	12.37	12.31	12.33	12.38	12.32	12.40	12.42	12.39	12.39	12.43
4.61	12.25	12.28	12.20	12.26	12.32	12.24	12.16	12.30	12.17	12.29	12.17
4.66	12.51	12.48	12.59	12.57	12.56	12.59	12.60	12.57	12.58	12.52	12.45
4.71	12.34	12.49	12.34	12.50	12.57	12.52	12.37	12.55	12.47	12.42	12.51
4.76	12.56	12.63	12.59	12.62	12.64	12.67	12.53	12.59	12.52	12.54	12.43
4.81	12.26	12.29	12.35	12.45	12.36	12.42	12.37	12.38	12.35	12.36	12.34
4.86	12.23	12.29	12.30	12.34	12.33	12.40	12.29	12.34	12.27	12.27	12.28
4.91	12.53	12.63	12.61	12.70	12.69	12.60	12.54	12.65	12.52	12.56	12.54
4.96	12.21	12.31	12.30	12.33	12.43	12.49	12.54	12.55	12.48	12.50	12.43
5.01	12.23	12.22	12.21	12.22	12.24	12.20	12.22	12.25	12.11	12.09	12.14
5.06	12.29	12.40	12.57	12.51	12.65	12.60	12.58	12.52	12.46	12.62	12.47
5.11	12.21	12.29	12.18	12.29	12.31	12.27	12.35	12.25	12.27	12.25	12.30
5.16	12.16	12.24	12.19	12.12	12.14	12.21	12.24	12.18	12.13	12.16	12.06
5.21	12.09	12.12	12.16	12.14	12.25	12.19	12.22	12.21	12.31	12.37	12.22
5.26	12.49	12.52	12.53	12.50	12.56	12.59	12.44	12.48	12.43	12.47	12.33
5.31	12.47	12.58	12.47	12.60	12.56	12.57	12.56	12.49	12.60	12.60	12.37
5.38	12.32	12.47	12.49	12.55	12.46	12.46	12.46	12.37	12.50	12.47	12.60
5.45	12.59	12.66	12.63	12.62	12.57	12.57	12.64	12.62	12.59	12.59	12.45
5.53	12.24	12.40	12.43	12.41	12.40	12.37	12.42	12.46	12.37	12.36	12.32
5.61	12.55	12.57	12.51	12.60	12.66	12.58	12.66	12.55	12.40	12.57	12.54

5.70	12.60	12.63	12.52	12.53	12.58	12.55	12.56	12.52	12.30	12.46	12.32
5.80	12.42	12.37	12.43	12.43	12.61	12.38	12.50	12.47	12.39	12.45	12.40
5.90	12.04	12.05	12.14	12.14	12.07	12.18	12.31	12.35	12.17	12.28	12.15
6.01	12.72	12.77	12.83	12.83	12.79	12.76	12.72	12.72	12.77	12.77	12.54
6.13	12.27	12.17	12.20	12.26	12.18	12.28	12.29	12.30	12.23	12.24	12.17
6.25	12.17	12.06	12.08	12.24	12.11	12.25	12.11	12.28	12.13	12.20	12.05
6.39	12.07	11.95	11.99	11.99	12.02	12.14	12.05	12.00	11.83	12.00	11.95
6.53	11.98	12.00	11.95	11.98	12.07	12.04	12.09	11.94	11.87	11.98	12.00
6.69	12.33	12.24	12.35	12.22	12.33	12.26	12.29	12.27	12.27	12.22	12.10
6.85	12.03	12.07	12.06	12.07	12.11	12.06	12.18	12.08	11.99	11.95	12.01
7.03	12.09	12.03	12.01	12.13	12.11	12.13	12.10	12.13	12.01	12.26	12.11
7.21	12.31	12.23	12.35	12.22	12.40	12.21	12.29	12.26	12.20	12.21	12.11
7.41	12.35	12.29	12.27	12.27	12.38	12.31	12.28	12.33	12.14	12.20	12.15
7.63	12.48	12.47	12.56	12.44	12.54	12.50	12.58	12.55	12.38	12.49	12.39
7.86	12.20	12.24	12.27	12.19	12.20	12.31	12.20	12.22	12.12	12.29	12.07
8.10	12.27	12.29	12.29	12.21	12.24	12.20	12.25	12.27	12.23	12.10	12.15
8.36	11.73	11.82	11.84	11.94	11.93	11.86	11.91	11.89	11.78	11.78	11.84
8.64	12.10	12.07	12.04	12.15	12.19	12.27	12.20	12.20	12.28	12.12	12.06
8.94	12.39	12.37	12.46	12.43	12.43	12.42	12.37	12.25	12.33	12.25	12.23
9.26	12.49	12.51	12.56	12.56	12.60	12.49	12.51	12.53	12.34	12.46	12.37
9.60	12.25	12.32	12.23	12.22	12.25	12.26	12.30	12.21	12.07	12.19	12.12
9.97	12.21	12.19	12.14	12.28	12.10	12.30	12.30	12.19	12.16	12.31	12.17
10.36	12.33	12.49	12.59	12.45	12.39	12.55	12.45	12.45	12.35	12.34	12.36
10.77	12.17	12.26	12.17	12.43	12.30	12.36	12.28	12.34	12.24	12.37	12.36
11.22	11.91	11.99	12.11	12.06	12.07	12.00	11.93	11.95	11.95	11.94	11.93
11.70	12.26	12.39	12.34	12.25	12.34	12.25	12.27	12.40	12.22	12.27	12.19
12.21	12.04	12.01	12.05	12.10	12.12	12.04	11.97	11.96	11.99	11.87	11.88
12.75	12.20	12.18	12.35	12.21	12.20	12.32	12.32	12.31	12.26	12.29	12.16
13.33	12.36	12.37	12.40	12.40	12.36	12.47	12.55	12.33	12.21	12.28	12.42
13.95	12.16	12.19	11.96	12.16	12.30	12.15	12.13	12.18	12.01	12.08	12.06
14.62	12.05	12.12	12.02	12.14	12.01	11.96	11.99	12.06	12.03	11.99	11.94
15.33	11.83	11.95	11.87	11.90	11.78	11.95	11.91	11.88	11.84	11.97	11.92
16.09	12.14	12.00	12.06	12.03	12.06	11.99	12.13	11.88	12.08	11.92	11.90
16.90	12.12	12.06	12.09	12.11	12.09	12.11	12.07	12.16	12.19	12.13	12.10
17.77	11.90	11.93	11.91	11.95	12.01	12.01	11.97	11.83	11.81	11.79	11.86
18.70	11.88	11.84	11.79	11.87	11.81	11.85	11.88	11.76	11.84	11.85	11.81
19.69	12.05	12.08	12.14	12.08	11.98	12.09	12.07	12.01	12.07	12.10	11.95
20.75	11.91	11.95	11.90	11.96	11.89	11.95	12.00	11.92	11.97	11.92	11.86
21.89	12.26	12.26	12.29	12.30	12.22	12.29	12.23	12.28	12.25	12.16	12.26
23.10	12.52	12.55	12.54	12.51	12.50	12.58	12.48	12.44	12.34	12.46	12.30
24.39	12.00	11.96	12.07	12.03	11.95	12.07	11.86	11.75	11.76	11.80	11.75
25.78	12.17	12.23	12.23	12.12	12.22	12.18	12.15	12.21	12.04	12.06	12.09
27.26	12.12	12.10	12.12	12.09	12.21	12.09	12.18	12.00	11.96	11.95	12.03
28.84	12.20	12.05	12.18	12.20	12.19	12.10	12.12	12.18	12.14	12.10	11.97
30.53	12.12	12.18	12.04	12.09	12.08	12.07	12.12	12.00	12.00	11.96	11.91
32.34	11.96	11.96	11.91	11.97	11.93	11.91	11.94	11.94	11.76	11.83	11.82
34.27	12.09	12.16	12.14	12.01	12.07	12.14	12.08	12.08	12.01	12.04	11.85
36.34	11.95	11.95	12.04	12.02	11.96	12.03	11.98	11.86	11.83	11.94	11.90
38.55	12.25	12.20	12.27	12.16	12.18	12.20	12.16	12.15	12.14	12.05	12.04
40.91	12.02	12.05	11.95	11.98	12.03	11.96	11.86	11.98	11.81	11.80	11.88

43.43	11.80	11.76	11.71	11.89	11.80	11.71	11.78	11.77	11.57	11.62	11.55
46.13	11.92	11.96	11.91	11.89	11.95	11.89	11.86	11.89	11.65	11.74	11.77
49.01	12.16	12.27	12.35	12.23	12.25	12.21	12.14	12.20	12.07	12.07	11.97
52.09	11.94	12.02	12.03	11.90	12.07	11.96	11.94	11.91	11.94	11.88	11.81
55.39	11.95	11.92	11.98	11.87	11.96	12.01	11.98	11.79	11.85	11.77	11.79
58.91	11.88	11.88	11.89	11.85	11.92	11.84	11.86	11.77	11.70	11.81	11.75
62.68	12.04	12.10	12.18	11.95	11.99	12.11	11.99	11.94	11.79	11.90	11.88
66.70	12.14	12.07	12.08	12.09	12.16	12.14	11.99	11.95	11.94	12.00	11.86
71.00	12.10	12.03	12.06	12.02	11.93	12.08	11.98	11.97	11.97	11.89	11.96
75.60	11.88	12.06	11.93	11.95	12.00	11.93	11.91	11.90	11.69	11.81	11.84
80.51	12.31	12.29	12.28	12.20	12.32	12.27	12.28	12.17	12.11	12.18	12.17
85.77	12.28	12.33	12.32	12.23	12.28	12.27	12.20	12.34	12.21	12.25	12.22
91.38	12.34	12.36	12.41	12.34	12.32	12.33	12.37	12.33	12.04	12.13	12.08
97.39	12.07	12.03	12.15	12.10	12.16	12.08	12.08	12.00	11.91	11.96	11.99
103.80	12.53	12.64	12.67	12.61	12.53	12.48	12.45	12.53	12.48	12.45	12.33
110.66	12.25	12.28	12.17	12.17	12.22	12.04	12.03	12.05	11.95	11.87	11.85
117.99	12.02	12.05	12.03	12.07	11.99	12.02	11.99	11.91	11.86	11.74	11.84
125.83	12.42	12.44	12.46	12.37	12.44	12.41	12.34	12.24	12.16	12.20	12.16
134.21	12.26	12.28	12.29	12.11	12.26	12.19	12.20	12.10	12.09	12.04	11.95
143.17	12.19	12.22	12.16	12.15	12.08	12.19	12.11	12.07	11.88	12.03	11.93
152.74	12.36	12.39	12.34	12.36	12.40	12.16	12.26	12.15	12.04	12.14	11.96
162.97	12.35	12.35	12.37	12.36	12.29	12.22	12.26	12.21	12.03	12.18	12.06
173.91	12.36	12.36	12.31	12.39	12.32	12.27	12.20	12.25	12.04	12.14	11.95
185.60	12.59	12.51	12.47	12.35	12.44	12.41	12.38	12.31	12.20	12.18	12.00
198.10	12.40	12.41	12.42	12.35	12.29	12.30	12.20	12.25	12.20	12.16	12.05
211.46	12.63	12.63	12.61	12.60	12.60	12.61	12.51	12.41	12.37	12.40	12.34
225.75	12.51	12.47	12.40	12.50	12.32	12.43	12.36	12.32	12.19	12.21	12.05
241.01	12.44	12.54	12.49	12.40	12.45	12.27	12.35	12.25	12.20	12.16	12.07
257.33	12.43	12.45	12.43	12.35	12.39	12.29	12.41	12.24	12.11	12.19	12.05
274.77	12.52	12.49	12.58	12.47	12.47	12.35	12.30	12.29	12.29	12.23	12.04
293.42	12.58	12.62	12.56	12.48	12.48	12.44	12.47	12.33	12.22	12.17	12.02
313.35	12.37	12.36	12.32	12.39	12.33	12.20	12.10	12.13	12.00	11.96	11.78
334.66	12.62	12.53	12.50	12.52	12.51	12.42	12.34	12.34	12.22	12.07	12.12
357.43	12.61	12.47	12.54	12.66	12.50	12.49	12.43	12.47	12.33	12.29	12.28
381.78	12.23	12.31	12.21	12.24	12.21	12.20	12.02	12.01	11.83	11.95	11.73
407.80	12.42	12.42	12.31	12.25	12.29	12.30	12.17	12.25	11.99	11.93	12.03
435.62	12.36	12.39	12.24	12.20	12.26	12.21	12.13	12.20	11.92	11.86	11.86
465.35	12.44	12.39	12.25	12.32	12.21	12.29	12.12	12.10	12.01	11.93	11.93
497.14	12.34	12.23	12.25	12.17	12.28	12.09	12.18	11.99	11.95	12.08	11.88
531.11	12.09	12.06	11.99	11.90	11.89	11.84	11.80	11.70	11.75	11.68	11.59
567.43	12.16	12.24	12.24	12.16	12.11	12.01	11.88	11.91	11.84	11.70	11.58
606.26	12.30	12.37	12.30	12.20	12.32	12.24	12.18	12.14	12.08	12.03	11.86
647.76	12.27	12.20	12.20	12.26	12.21	12.11	11.94	12.02	11.69	11.85	11.73
692.12	11.91	11.90	11.87	11.76	11.70	11.60	11.65	11.55	11.40	11.45	11.37
739.53	12.24	12.15	12.17	12.18	12.14	12.03	12.01	11.98	11.82	11.75	11.67
790.22	11.96	11.99	11.91	11.82	11.98	11.79	11.90	11.66	11.57	11.62	11.45
844.40	12.16	12.08	12.01	12.07	12.08	11.99	12.05	11.91	11.70	11.85	11.77
902.32	12.05	12.05	12.00	11.88	12.00	11.84	11.82	11.83	11.69	11.63	11.57
964.23	11.75	11.71	11.66	11.53	11.68	11.50	11.55	11.49	11.43	11.23	11.25
1030.41	12.03	12.03	11.92	11.81	11.87	11.86	11.80	11.70	11.53	11.49	11.38

1101.15	11.77	11.71	11.74	11.75	11.65	11.56	11.49	11.50	11.36	11.26	11.15
1176.77	11.80	11.87	11.74	11.67	11.67	11.56	11.52	11.45	11.38	11.24	11.07
1257.60	11.83	11.75	11.84	11.64	11.69	11.60	11.48	11.47	11.26	11.26	11.20
1344.00	11.73	11.72	11.63	11.52	11.48	11.43	11.40	11.26	11.15	11.05	11.08
1436.36	11.57	11.48	11.48	11.51	11.43	11.34	11.31	11.30	11.21	11.02	11.08
1535.09	11.56	11.54	11.56	11.51	11.31	11.31	11.20	11.30	11.14	11.04	10.79
1640.63	11.38	11.47	11.39	11.34	11.23	11.26	11.09	11.09	11.00	11.08	10.91
1753.44	11.37	11.35	11.26	11.28	11.17	11.12	11.03	11.01	10.83	10.79	10.65
1874.02	11.17	11.26	11.14	11.06	11.06	10.97	10.86	10.85	10.79	10.85	10.86
2002.92	11.29	11.29	11.20	11.17	11.19	11.10	10.93	10.99	10.78	10.76	10.66
2140.71	11.14	11.19	11.11	11.05	10.98	10.93	10.89	10.82	10.61	10.65	10.62
2288.00	11.01	11.00	11.01	10.95	10.97	10.78	10.73	10.69	10.58	10.51	10.42
2445.44	10.98	10.96	10.93	10.81	10.82	10.72	10.70	10.60	10.43	10.47	10.35
2613.73	10.91	10.82	10.77	10.91	10.77	10.74	10.61	10.57	10.45	10.40	10.28
2793.63	10.67	10.56	10.54	10.46	10.50	10.30	10.28	10.31	10.16	10.05	10.00
2985.93	10.44	10.27	10.31	10.37	10.31	10.20	10.05	10.05	9.91	9.92	9.83
3191.49	10.16	10.16	10.12	10.06	9.97	10.03	9.93	9.82	9.69	9.63	9.50
3411.21	10.08	9.96	9.84	9.86	9.82	9.74	9.74	9.52	9.30	9.22	9.22
3646.09	9.69	9.83	9.57	9.57	9.54	9.43	9.34	9.32	9.08	9.13	8.99
3897.16	9.57	9.40	9.34	9.39	9.40	9.14	9.12	9.19	8.84	8.81	8.71

Wavelength (nm) / Time (ps)	647.38	648.80	650.23	651.65	653.08	654.50	655.93	657.35	658.77	660.20	661.62
-2.74	-0.06	-0.02	0.01	0.02	-0.04	-0.15	-0.18	-0.06	-0.19	-0.08	-0.06
-2.24	-0.01	-0.04	-0.11	-0.15	-0.08	-0.26	-0.19	-0.27	-0.27	-0.38	-0.37
-1.74	0.09	0.10	0.14	0.12	0.23	0.29	0.27	0.07	0.12	0.08	0.05
-1.24	0.03	0.00	-0.01	0.10	-0.02	0.05	0.04	0.23	0.23	0.23	0.19
-0.74	0.09	0.05	-0.01	-0.08	-0.21	-0.13	0.00	-0.20	-0.21	-0.05	-0.11
-0.24	-0.06	-0.04	0.05	-0.06	0.03	-0.01	-0.03	0.04	0.14	0.03	0.01
-0.14	-0.12	-0.06	-0.02	0.04	0.05	0.14	0.14	0.19	0.18	0.22	0.18
-0.04	0.05	0.00	-0.04	0.02	0.04	0.08	-0.05	-0.01	0.00	-0.05	0.12
0.06	-0.03	0.01	-0.01	-0.07	0.01	0.09	-0.01	-0.03	0.11	0.21	0.19
0.16	0.05	0.07	0.10	0.08	0.08	0.15	-0.05	-0.20	-0.10	-0.19	-0.23
0.26	0.04	-0.12	-0.04	0.02	0.06	-0.04	-0.02	0.05	-0.06	-0.07	-0.16
0.31	0.06	0.04	0.04	0.06	0.04	0.02	0.14	-0.03	0.10	0.04	0.08
0.36	-0.05	-0.04	-0.03	-0.05	-0.13	-0.09	0.01	-0.04	0.02	-0.04	0.01
0.41	-0.02	-0.04	0.00	0.21	0.13	0.00	0.26	0.23	0.16	0.27	0.25
0.46	-0.06	-0.01	-0.12	-0.09	-0.03	0.07	-0.06	-0.11	-0.01	-0.07	0.04
0.51	0.04	-0.07	-0.02	0.15	0.12	0.01	-0.04	-0.02	-0.02	0.12	-0.01
0.56	0.03	0.00	0.01	0.01	0.00	0.04	-0.01	-0.08	-0.01	0.13	0.04
0.61	-0.01	-0.06	-0.08	-0.11	-0.09	-0.11	-0.20	-0.24	-0.18	-0.08	-0.06
0.66	0.06	0.05	-0.07	0.01	-0.02	0.18	-0.01	-0.06	0.08	0.16	0.06
0.71	-0.14	-0.02	0.09	0.02	0.18	-0.04	0.03	0.07	-0.04	0.20	0.10
0.76	0.00	0.06	0.18	0.07	0.01	0.09	-0.05	-0.05	-0.15	-0.12	-0.17
0.81	0.06	-0.03	0.13	0.09	0.05	0.08	-0.07	-0.17	-0.08	0.01	-0.16
0.86	0.18	0.12	0.23	0.23	0.32	0.24	0.25	0.25	0.38	0.32	0.35
0.91	-0.06	0.11	0.07	0.04	-0.01	-0.14	-0.07	-0.16	-0.10	-0.06	-0.11
0.96	0.00	-0.06	0.05	0.01	-0.10	-0.09	-0.10	-0.16	-0.05	-0.15	-0.12
1.01	-0.04	0.11	-0.03	0.03	0.00	-0.05	-0.16	-0.23	-0.21	-0.18	-0.26

1.06	0.03	0.00	0.11	0.08	0.13	0.02	-0.07	0.09	0.11	0.22	0.05
1.11	0.00	-0.08	-0.06	0.04	-0.12	-0.09	-0.14	-0.08	-0.12	-0.09	-0.02
1.16	-0.01	0.06	0.00	0.12	0.17	0.06	-0.01	0.06	-0.09	0.12	-0.06
1.21	0.11	0.31	0.15	0.21	0.15	0.11	0.12	0.13	0.12	0.03	0.09
1.26	-0.06	0.20	0.03	0.03	-0.12	-0.15	-0.07	0.01	0.03	-0.20	-0.27
1.31	0.34	0.40	0.32	0.10	-0.07	-0.08	0.29	0.30	0.30	0.14	0.00
1.36	-10.59	-9.78	-8.62	-6.85	-4.87	-2.63	-0.25	1.54	2.96	3.51	3.38
1.41	41.60	38.32	35.80	34.65	34.38	35.05	37.15	40.39	44.73	49.35	53.49
1.46	86.12	86.75	86.75	87.57	85.92	83.46	80.89	78.61	76.67	76.80	76.18
1.51	5.26	3.30	0.93	-1.54	-4.36	-7.54	-10.89	-14.54	-18.83	-23.24	-27.58
1.56	21.29	21.10	21.05	20.58	20.20	19.57	19.09	18.72	18.95	19.58	20.21
1.61	15.70	16.35	17.69	18.65	19.16	18.98	18.39	17.89	18.04	19.09	20.17
1.66	14.20	13.96	13.63	13.44	13.43	13.42	13.68	14.05	14.38	14.86	15.04
1.71	16.39	16.71	16.89	16.87	16.71	16.78	16.78	16.51	16.23	16.26	16.24
1.76	12.54	12.69	12.78	13.18	13.61	13.87	14.35	14.69	14.96	15.52	15.73
1.81	14.67	14.30	13.97	13.73	13.53	13.30	13.36	13.33	13.45	13.44	13.93
1.86	15.69	15.98	16.03	16.15	16.07	16.20	16.02	15.91	15.75	15.71	15.55
1.91	11.90	11.72	11.70	11.78	11.86	12.14	12.49	12.70	13.13	13.55	13.79
1.96	14.71	14.67	14.50	14.39	14.03	13.94	13.68	13.56	13.57	13.72	13.75
2.01	13.42	13.80	14.19	14.67	14.80	15.11	15.30	15.52	15.56	15.65	15.65
2.06	13.15	13.06	12.76	12.64	12.42	12.38	12.35	12.49	12.67	12.88	13.12
2.11	14.91	15.02	14.91	14.81	14.76	14.53	14.31	14.24	14.29	14.13	14.04
2.16	12.14	12.38	12.50	12.70	12.91	13.07	13.34	13.58	13.70	13.96	14.04
2.21	13.64	13.32	13.17	13.02	12.89	12.85	12.93	12.94	13.10	13.20	13.33
2.26	14.33	14.54	14.54	14.68	14.67	14.74	14.67	14.67	14.68	14.56	14.49
2.31	11.98	11.90	12.04	11.88	12.00	12.22	12.34	12.66	12.81	13.13	13.29
2.36	13.51	13.51	13.33	13.33	13.23	13.24	13.31	13.44	13.58	13.66	13.82
2.41	13.34	13.60	13.89	13.95	14.24	14.27	14.41	14.47	14.58	14.56	14.44
2.46	12.10	12.03	12.07	11.92	11.93	12.01	12.07	12.24	12.37	12.56	12.65
2.51	14.58	14.66	14.71	14.65	14.58	14.55	14.34	14.33	14.27	14.31	14.11
2.56	12.45	12.43	12.60	12.76	12.93	12.97	13.19	13.28	13.34	13.51	13.66
2.61	12.44	12.33	12.41	12.31	12.36	12.27	12.40	12.60	12.69	12.72	12.87
2.66	13.86	13.91	13.78	13.97	13.79	13.75	13.51	13.69	13.50	13.42	13.47
2.71	11.92	12.03	12.08	12.26	12.37	12.40	12.51	12.77	12.83	13.08	13.09
2.76	13.05	13.04	12.97	12.80	12.77	12.84	12.82	12.79	12.76	12.77	12.94
2.81	13.01	13.27	13.27	13.42	13.40	13.56	13.54	13.56	13.63	13.56	13.51
2.86	12.22	12.26	12.32	12.40	12.42	12.58	12.83	12.98	13.13	13.32	13.49
2.91	13.54	13.55	13.37	13.39	13.39	13.18	13.15	13.20	13.05	12.94	12.88
2.96	12.73	12.86	12.92	13.09	13.12	13.18	13.32	13.38	13.49	13.59	13.65
3.01	12.61	12.61	12.58	12.52	12.47	12.48	12.52	12.61	12.68	12.87	13.08
3.06	12.85	12.94	13.06	12.95	12.96	13.07	13.03	13.09	13.05	13.11	13.14
3.11	12.13	12.30	12.27	12.37	12.47	12.61	12.79	12.85	12.97	13.13	13.25
3.16	12.80	12.64	12.68	12.67	12.51	12.42	12.38	12.56	12.43	12.62	12.68
3.21	12.65	12.59	12.50	12.51	12.59	12.58	12.46	12.56	12.49	12.57	12.50
3.26	11.97	12.03	11.95	12.17	12.14	12.12	12.42	12.44	12.64	12.71	12.86
3.31	12.82	12.79	12.70	12.66	12.59	12.52	12.46	12.52	12.60	12.53	12.43
3.36	12.59	12.68	12.58	12.72	12.72	12.85	12.98	12.98	13.07	13.25	13.15
3.41	12.22	12.30	12.24	12.23	12.15	12.18	12.30	12.42	12.42	12.67	12.79
3.46	12.74	12.82	12.74	12.74	12.68	12.55	12.41	12.50	12.43	12.50	12.53
3.51	12.14	12.25	12.29	12.40	12.47	12.57	12.71	12.82	12.97	13.05	13.22

3.56	12.42	12.40	12.31	12.16	12.20	12.02	12.19	12.18	12.19	12.15	12.28
3.61	12.61	12.71	12.70	12.79	12.71	12.92	12.85	12.84	12.92	12.97	13.03
3.66	11.92	11.84	11.84	11.94	12.07	12.16	12.07	12.18	12.29	12.43	12.55
3.71	13.06	12.92	12.84	12.80	12.79	12.72	12.77	12.73	12.84	12.83	12.90
3.76	12.92	12.98	12.81	12.86	12.94	12.97	12.76	12.92	12.95	13.06	12.96
3.81	11.96	11.91	11.91	11.91	12.05	11.88	11.87	11.93	12.11	12.11	12.31
3.86	12.43	12.49	12.42	12.43	12.51	12.47	12.61	12.57	12.57	12.70	12.71
3.91	12.34	12.41	12.43	12.56	12.57	12.57	12.63	12.79	12.97	13.12	13.20
3.96	12.30	12.31	12.32	12.24	12.38	12.39	12.40	12.56	12.60	12.71	12.77
4.01	12.69	12.68	12.76	12.70	12.69	12.85	12.73	12.77	12.76	12.81	12.81
4.06	12.50	12.44	12.55	12.43	12.50	12.57	12.50	12.66	12.68	12.85	12.89
4.11	12.51	12.58	12.35	12.33	12.43	12.44	12.48	12.57	12.67	12.66	12.74
4.16	12.52	12.65	12.62	12.64	12.68	12.65	12.62	12.70	12.79	12.77	12.60
4.21	12.52	12.59	12.63	12.69	12.73	12.79	12.80	12.94	13.11	13.13	13.23
4.26	12.46	12.49	12.39	12.50	12.56	12.50	12.49	12.61	12.62	12.61	12.64
4.31	12.06	12.24	12.32	12.30	12.39	12.47	12.57	12.66	12.80	12.95	13.07
4.36	12.21	12.21	12.29	12.32	12.35	12.35	12.43	12.52	12.62	12.84	12.79
4.41	12.25	12.33	12.43	12.37	12.44	12.44	12.47	12.56	12.60	12.78	12.74
4.46	12.29	12.45	12.36	12.36	12.44	12.50	12.54	12.49	12.55	12.89	12.79
4.51	12.34	12.47	12.42	12.47	12.50	12.50	12.66	12.60	12.70	12.85	12.97
4.56	12.43	12.37	12.50	12.40	12.38	12.43	12.43	12.46	12.51	12.50	12.51
4.61	12.13	12.18	12.18	12.21	12.25	12.28	12.35	12.53	12.53	12.54	12.56
4.66	12.55	12.53	12.42	12.40	12.45	12.41	12.44	12.53	12.59	12.72	12.61
4.71	12.36	12.41	12.47	12.54	12.49	12.47	12.43	12.45	12.42	12.50	12.43
4.76	12.50	12.47	12.48	12.40	12.52	12.53	12.65	12.58	12.74	12.93	12.98
4.81	12.41	12.45	12.36	12.17	12.16	12.25	12.16	12.21	12.31	12.31	12.28
4.86	12.33	12.22	12.44	12.28	12.40	12.38	12.34	12.34	12.53	12.46	12.67
4.91	12.48	12.59	12.55	12.54	12.58	12.66	12.72	12.84	12.92	13.08	13.07
4.96	12.52	12.57	12.47	12.67	12.65	12.61	12.61	12.68	12.76	12.85	12.87
5.01	12.13	11.97	12.08	12.07	12.16	12.11	12.18	12.24	12.24	12.27	12.44
5.06	12.60	12.55	12.54	12.57	12.55	12.51	12.58	12.65	12.79	12.84	12.93
5.11	12.48	12.24	12.23	12.42	12.37	12.39	12.39	12.48	12.46	12.69	12.72
5.16	12.19	12.14	12.17	12.21	12.16	12.32	12.37	12.39	12.48	12.62	12.62
5.21	12.27	12.29	12.28	12.50	12.43	12.56	12.69	12.68	12.83	12.91	13.05
5.26	12.36	12.46	12.40	12.37	12.40	12.35	12.49	12.53	12.66	12.75	12.60
5.31	12.39	12.33	12.33	12.37	12.41	12.37	12.36	12.43	12.57	12.49	12.70
5.38	12.43	12.38	12.50	12.45	12.55	12.58	12.58	12.63	12.73	12.85	12.79
5.45	12.44	12.50	12.56	12.42	12.59	12.67	12.69	12.73	12.70	12.92	13.01
5.53	12.50	12.33	12.36	12.39	12.37	12.38	12.46	12.48	12.56	12.71	12.76
5.61	12.41	12.32	12.50	12.43	12.55	12.47	12.70	12.42	12.55	12.62	12.77
5.70	12.28	12.40	12.36	12.31	12.31	12.44	12.37	12.32	12.51	12.55	12.50
5.80	12.39	12.48	12.52	12.53	12.61	12.59	12.57	12.60	12.77	12.78	13.00
5.90	12.17	12.24	12.22	12.26	12.22	12.36	12.38	12.36	12.40	12.52	12.53
6.01	12.57	12.67	12.63	12.75	12.59	12.64	12.62	12.53	12.71	12.61	12.57
6.13	12.23	12.18	12.13	12.02	12.20	12.17	12.20	12.25	12.36	12.35	12.45
6.25	12.01	12.09	12.04	11.91	12.04	12.01	12.04	12.07	12.07	12.14	12.27
6.39	12.10	11.96	12.04	11.97	12.02	12.06	12.06	12.17	12.39	12.38	12.44
6.53	11.98	11.98	11.97	11.99	12.07	12.20	12.18	12.24	12.33	12.49	12.54
6.69	12.16	12.02	12.12	12.06	11.95	11.88	12.11	12.06	12.03	12.09	12.06
6.85	12.02	12.03	12.08	12.04	12.13	12.13	12.23	12.16	12.33	12.37	12.31

7.03	12.07	12.06	12.16	12.22	12.18	12.32	12.27	12.34	12.48	12.56	12.56
7.21	12.16	12.26	12.27	12.16	12.10	12.18	12.04	12.21	12.34	12.23	12.42
7.41	12.09	12.17	12.16	12.13	12.11	12.22	12.19	12.18	12.21	12.37	12.26
7.63	12.39	12.51	12.39	12.35	12.46	12.43	12.46	12.58	12.71	12.84	12.80
7.86	11.98	12.09	12.01	12.04	12.04	11.98	12.01	12.16	12.20	12.25	12.13
8.10	12.12	12.11	12.18	12.12	12.20	12.32	12.13	12.34	12.37	12.48	12.43
8.36	11.76	11.89	11.94	11.94	11.95	11.83	12.03	11.97	12.25	12.24	12.32
8.64	11.96	12.03	11.96	12.05	12.09	12.12	12.12	12.13	12.20	12.32	12.34
8.94	12.16	12.21	12.14	12.10	12.11	12.22	12.24	12.19	12.26	12.24	12.27
9.26	12.42	12.41	12.31	12.24	12.40	12.36	12.41	12.43	12.45	12.64	12.59
9.60	11.96	12.11	12.09	12.00	12.14	12.14	12.08	12.11	12.16	12.20	12.18
9.97	12.37	12.25	12.26	12.38	12.45	12.42	12.50	12.57	12.61	12.68	12.69
10.36	12.29	12.27	12.30	12.36	12.23	12.15	12.36	12.28	12.33	12.48	12.35
10.77	12.30	12.29	12.29	12.36	12.33	12.41	12.46	12.57	12.64	12.69	12.80
11.22	11.93	11.90	11.84	11.93	11.91	11.84	11.76	11.93	11.97	12.07	12.12
11.70	12.13	12.09	12.13	12.06	12.07	12.02	11.98	12.02	12.04	12.15	12.06
12.21	11.94	11.96	11.93	11.86	11.81	11.79	11.89	11.82	11.76	11.81	11.84
12.75	12.17	12.19	12.19	12.14	12.25	12.26	12.19	12.15	12.28	12.35	12.41
13.33	12.23	12.17	12.30	12.22	12.22	12.28	12.25	12.26	12.43	12.45	12.51
13.95	12.00	11.98	11.98	12.03	11.82	11.80	11.91	12.00	12.02	11.95	12.10
14.62	11.97	12.00	11.99	11.89	11.88	11.89	11.99	11.95	11.98	11.98	12.09
15.33	11.76	11.88	11.84	11.93	11.88	11.95	12.00	11.99	12.08	12.09	12.16
16.09	11.86	11.89	12.02	12.00	11.90	11.83	11.90	11.93	11.95	11.90	12.07
16.90	12.02	12.09	12.20	12.06	12.19	12.36	12.30	12.22	12.47	12.52	12.69
17.77	11.82	11.86	11.81	11.75	11.76	11.72	11.80	11.85	11.91	11.97	11.92
18.70	11.75	11.85	11.78	11.86	11.96	11.85	11.80	11.99	11.99	12.11	12.13
19.69	11.91	12.04	11.94	11.96	11.96	12.00	12.10	12.13	12.28	12.15	12.31
20.75	11.82	11.83	11.81	11.85	11.76	11.85	11.86	12.00	11.92	12.07	12.09
21.89	12.15	12.21	12.20	12.10	12.21	12.18	12.22	12.20	12.35	12.48	12.44
23.10	12.24	12.28	12.33	12.30	12.23	12.24	12.42	12.33	12.29	12.33	12.35
24.39	11.78	11.63	11.76	11.71	11.65	11.77	11.68	11.72	11.77	12.01	11.94
25.78	11.93	12.10	11.97	12.12	12.17	11.98	12.18	12.24	12.38	12.39	12.44
27.26	11.84	11.95	11.89	11.87	11.90	11.95	11.97	12.04	12.05	12.11	12.06
28.84	12.03	12.03	12.06	12.08	12.00	12.22	12.09	12.12	12.31	12.31	12.36
30.53	11.88	11.84	11.93	11.76	11.78	12.00	11.80	11.95	12.05	11.99	12.07
32.34	11.76	11.75	11.70	11.74	11.72	11.75	11.82	11.81	11.85	11.93	12.01
34.27	11.85	11.81	11.84	11.95	11.83	11.87	11.87	11.87	11.95	11.87	12.03
36.34	11.88	11.86	11.75	11.92	11.87	11.87	11.93	12.02	11.98	12.06	12.01
38.55	12.04	12.03	12.06	12.06	12.02	12.07	12.06	12.16	12.33	12.27	12.33
40.91	11.87	11.74	11.87	11.80	11.74	11.78	11.65	11.83	11.87	12.02	11.97
43.43	11.53	11.49	11.47	11.47	11.54	11.33	11.34	11.52	11.43	11.47	11.59
46.13	11.65	11.67	11.64	11.57	11.67	11.58	11.42	11.60	11.69	11.72	11.76
49.01	11.93	12.05	12.05	12.05	12.05	11.99	12.05	12.08	12.14	12.38	12.23
52.09	11.87	11.82	11.95	11.85	11.87	11.94	12.03	12.07	12.16	12.28	12.33
55.39	11.67	11.85	11.64	11.63	11.73	11.65	11.60	11.68	11.68	11.87	11.83
58.91	11.74	11.60	11.69	11.73	11.65	11.64	11.55	11.65	11.58	11.69	11.75
62.68	11.78	11.83	11.84	11.91	11.82	11.81	11.95	11.95	12.02	12.07	12.10
66.70	11.88	12.00	11.88	11.85	11.79	11.88	11.80	11.83	11.86	11.90	12.06
71.00	11.79	11.77	11.93	11.79	11.74	11.89	11.88	11.81	11.93	11.90	11.99
75.60	11.65	11.74	11.66	11.68	11.78	11.73	11.72	11.82	11.87	11.94	12.02

80.51	12.03	12.08	12.02	11.99	11.96	11.86	11.94	11.88	11.95	11.93	11.97
85.77	12.30	12.31	12.24	12.36	12.42	12.48	12.41	12.46	12.40	12.59	12.63
91.38	12.06	11.95	11.95	12.04	11.74	11.89	11.83	11.91	11.91	11.81	11.84
97.39	11.87	12.04	11.89	11.94	12.00	11.89	12.02	12.16	12.09	12.22	12.17
103.80	12.26	12.19	12.26	12.25	12.22	12.05	12.17	12.12	12.09	12.26	12.22
110.66	11.79	11.73	11.65	11.61	11.68	11.67	11.52	11.57	11.59	11.57	11.55
117.99	11.72	11.79	11.85	11.89	11.84	11.81	11.85	11.95	11.97	12.20	12.05
125.83	12.19	11.97	12.01	11.92	11.89	11.91	11.80	11.75	11.75	11.78	11.87
134.21	11.83	11.85	11.70	11.75	11.76	11.59	11.72	11.54	11.59	11.71	11.62
143.17	11.80	11.84	11.81	11.85	11.77	11.85	11.70	11.76	11.80	11.93	11.95
152.74	11.94	11.84	11.73	11.78	11.82	11.61	11.63	11.64	11.66	11.59	11.62
162.97	12.01	11.93	12.00	11.94	11.83	11.87	11.79	11.76	11.98	11.87	11.92
173.91	11.98	11.99	11.97	11.90	11.93	11.96	11.80	11.93	11.89	11.85	11.95
185.60	12.03	11.79	11.86	11.91	11.76	11.67	11.71	11.65	11.55	11.58	11.68
198.10	11.98	12.01	11.95	11.94	11.84	11.80	11.86	11.80	11.91	11.91	11.91
211.46	12.35	12.11	12.09	12.00	12.09	12.05	11.83	11.83	11.88	11.99	11.98
225.75	11.99	11.99	11.91	11.88	11.97	11.95	11.85	12.00	11.99	11.90	12.11
241.01	12.03	11.92	11.85	11.80	11.71	11.72	11.53	11.68	11.56	11.53	11.45
257.33	12.01	12.11	11.94	11.98	11.89	11.92	11.85	11.89	11.99	11.95	11.82
274.77	12.03	11.94	11.82	11.80	11.76	11.67	11.70	11.72	11.67	11.92	11.75
293.42	12.01	11.97	11.97	11.92	11.79	11.68	11.72	11.75	11.76	11.79	11.70
313.35	11.76	11.65	11.67	11.55	11.52	11.44	11.39	11.32	11.39	11.35	11.32
334.66	12.09	11.95	11.97	11.91	11.88	11.81	11.69	11.80	11.79	11.78	11.77
357.43	12.25	12.14	12.09	12.09	12.14	12.05	12.00	12.11	12.03	12.05	12.01
381.78	11.65	11.68	11.52	11.50	11.53	11.26	11.26	11.16	11.14	11.17	11.07
407.80	11.92	11.84	11.87	11.84	11.67	11.64	11.54	11.52	11.48	11.39	11.37
435.62	11.79	11.86	11.67	11.61	11.64	11.62	11.63	11.60	11.56	11.64	11.66
465.35	11.73	11.79	11.59	11.52	11.48	11.38	11.31	11.38	11.25	11.27	11.14
497.14	11.83	11.82	11.65	11.61	11.49	11.48	11.50	11.43	11.53	11.47	11.35
531.11	11.46	11.40	11.27	11.27	11.30	11.25	11.12	11.10	11.13	11.16	11.23
567.43	11.54	11.57	11.55	11.24	11.29	11.32	11.22	11.23	11.18	11.24	11.12
606.26	11.80	11.80	11.87	11.74	11.77	11.57	11.61	11.63	11.68	11.72	11.63
647.76	11.58	11.48	11.40	11.19	11.17	11.15	10.94	10.95	10.82	10.73	10.73
692.12	11.25	11.29	11.14	11.17	11.06	10.92	11.01	10.91	10.93	10.92	10.87
739.53	11.44	11.62	11.44	11.34	11.26	11.18	11.26	11.01	11.22	11.01	10.93
790.22	11.44	11.41	11.47	11.41	11.27	11.23	11.26	11.15	11.22	11.29	11.14
844.40	11.65	11.63	11.62	11.55	11.50	11.28	11.43	11.27	11.24	11.13	11.20
902.32	11.42	11.45	11.36	11.25	11.12	11.13	10.90	10.95	10.88	10.99	10.89
964.23	11.15	11.21	11.11	11.03	11.16	10.95	10.89	10.91	10.85	10.77	10.93
1030.41	11.36	11.22	11.13	11.13	10.97	10.78	10.77	10.69	10.56	10.47	10.38
1101.15	11.03	11.02	11.00	10.94	10.78	10.71	10.59	10.50	10.62	10.54	10.51
1176.77	11.23	11.02	11.09	10.88	10.82	10.73	10.69	10.69	10.54	10.66	10.51
1257.60	11.21	11.04	10.94	10.94	10.82	10.51	10.55	10.65	10.53	10.47	10.41
1344.00	10.98	11.02	10.81	10.81	10.69	10.67	10.58	10.41	10.52	10.33	10.39
1436.36	10.93	10.85	10.80	10.65	10.57	10.56	10.44	10.42	10.35	10.29	10.32
1535.09	10.74	10.66	10.58	10.52	10.47	10.35	10.32	10.20	10.23	10.20	10.08
1640.63	10.88	10.89	10.81	10.82	10.74	10.65	10.71	10.59	10.70	10.66	10.65
1753.44	10.52	10.40	10.45	10.29	10.35	10.08	10.14	10.14	10.19	10.12	10.12
1874.02	10.75	10.61	10.53	10.64	10.59	10.44	10.60	10.43	10.47	10.58	10.57
2002.92	10.49	10.55	10.40	10.30	10.31	10.08	10.01	9.94	9.97	9.80	9.73

2140.71	10.49	10.39	10.30	10.24	10.11	10.10	9.89	9.86	9.71	9.82	9.66
2288.00	10.25	10.18	10.23	10.14	10.01	9.90	9.78	9.77	9.59	9.63	9.55
2445.44	10.36	10.38	10.17	10.18	10.04	9.96	9.94	9.95	9.93	9.88	9.80
2613.73	10.13	10.01	9.91	9.92	9.83	9.61	9.55	9.48	9.49	9.43	9.31
2793.63	9.99	9.79	9.82	9.66	9.61	9.56	9.53	9.43	9.27	9.23	9.08
2985.93	9.82	9.75	9.61	9.50	9.47	9.52	9.30	9.26	9.23	9.28	9.06
3191.49	9.49	9.42	9.35	9.31	9.26	9.25	9.17	9.19	9.27	9.04	9.00
3411.21	9.13	9.03	8.95	8.86	8.74	8.71	8.64	8.60	8.42	8.49	8.39
3646.09	9.01	8.92	8.94	8.92	8.76	8.77	8.64	8.52	8.57	8.73	8.70
3897.16	8.68	8.67	8.51	8.54	8.54	8.53	8.31	8.29	8.23	8.28	8.25

Wavelength (nm) / Time (ps)	663.05	664.47	665.89	667.32	668.74	670.17
-2.74	-0.10	-0.10	-0.17	-0.16	-0.07	-0.12
-2.24	-0.51	-0.47	-0.46	-0.52	-0.59	-0.45
-1.74	0.04	-0.05	-0.03	0.01	0.04	-0.08
-1.24	0.33	0.30	0.35	0.34	0.38	0.32
-0.74	-0.11	-0.12	-0.16	-0.07	-0.15	-0.20
-0.24	0.10	0.13	0.13	0.09	0.09	0.11
-0.14	0.27	0.32	0.23	0.37	0.35	0.48
-0.04	-0.02	-0.02	0.11	-0.06	-0.05	-0.06
0.06	0.23	0.19	0.16	0.10	0.09	0.25
0.16	-0.26	-0.40	-0.35	-0.42	-0.55	-0.45
0.26	-0.11	-0.03	-0.09	-0.15	-0.08	-0.18
0.31	-0.01	0.11	-0.02	0.03	0.06	0.12
0.36	0.01	-0.06	-0.07	-0.13	-0.07	0.00
0.41	0.06	0.34	0.25	0.30	0.28	0.36
0.46	-0.14	-0.04	-0.04	-0.10	0.00	0.02
0.51	-0.12	-0.11	-0.02	-0.05	-0.10	-0.11
0.56	0.10	0.02	0.02	0.00	-0.15	-0.08
0.61	-0.22	-0.15	-0.17	-0.18	-0.07	0.07
0.66	0.10	0.07	0.02	-0.06	0.07	0.05
0.71	0.13	0.04	0.10	0.07	0.07	0.26
0.76	-0.19	-0.30	-0.31	-0.42	-0.44	-0.36
0.81	-0.25	-0.16	-0.34	-0.20	-0.17	-0.21
0.86	0.30	0.32	0.30	0.25	0.26	0.40
0.91	-0.22	-0.19	-0.26	-0.30	-0.37	-0.29
0.96	-0.21	-0.17	-0.24	-0.27	-0.33	-0.35
1.01	-0.40	-0.35	-0.50	-0.51	-0.46	-0.42
1.06	0.04	0.19	0.21	0.14	0.00	0.13
1.11	-0.07	-0.09	-0.10	-0.13	-0.24	-0.07
1.16	-0.02	-0.04	-0.12	-0.03	-0.14	-0.10
1.21	0.11	0.04	0.07	0.09	0.05	0.12
1.26	-0.08	-0.08	-0.11	-0.33	-0.16	-0.01
1.31	-0.13	0.08	0.18	0.25	0.08	-0.10
1.36	2.89	2.33	1.60	1.20	0.51	-0.16
1.41	56.88	59.14	61.08	62.62	64.36	66.97
1.46	74.98	72.87	69.67	68.65	69.19	71.69
1.51	-32.22	-36.65	-41.13	-44.97	-48.31	-51.74

1.56	21.29	21.62	21.59	21.45	21.40	21.84
1.61	21.00	20.53	19.15	17.53	16.93	17.92
1.66	15.47	16.03	16.44	16.95	17.22	17.70
1.71	16.14	16.27	16.29	16.19	16.36	16.72
1.76	15.92	16.19	16.23	16.38	16.56	16.85
1.81	14.21	14.51	14.78	15.06	15.06	15.59
1.86	15.56	15.36	15.39	15.44	15.49	15.85
1.91	13.93	14.26	14.42	14.63	14.74	15.16
1.96	13.80	14.04	14.09	14.31	14.33	14.68
2.01	15.57	15.64	15.52	15.55	15.64	16.04
2.06	13.28	13.57	13.70	13.71	13.90	14.15
2.11	13.98	14.07	14.24	14.04	14.14	14.56
2.16	14.07	14.28	14.08	14.09	14.18	14.60
2.21	13.65	13.72	13.91	13.97	14.19	14.57
2.26	14.42	14.48	14.47	14.46	14.58	14.82
2.31	13.43	13.68	13.86	14.04	13.96	14.30
2.36	13.86	14.05	14.11	14.20	14.28	14.65
2.41	14.39	14.45	14.33	14.30	14.43	14.75
2.46	12.92	13.08	13.20	13.26	13.55	13.82
2.51	14.12	14.03	14.21	14.14	14.20	14.52
2.56	13.57	13.85	13.96	13.91	13.87	14.12
2.61	12.90	13.11	13.24	13.19	13.46	13.59
2.66	13.42	13.37	13.35	13.48	13.53	13.89
2.71	13.21	13.27	13.42	13.29	13.45	13.89
2.76	12.99	13.17	13.29	13.34	13.49	13.72
2.81	13.47	13.62	13.60	13.67	13.61	13.80
2.86	13.61	13.70	13.76	13.82	13.84	14.20
2.91	12.88	13.08	12.95	12.95	13.07	13.43
2.96	13.73	13.84	13.79	13.80	13.85	14.19
3.01	13.15	13.29	13.39	13.54	13.73	14.02
3.06	13.02	13.19	13.18	13.19	13.33	13.62
3.11	13.35	13.44	13.53	13.53	13.64	14.02
3.16	12.80	12.87	12.88	13.02	13.13	13.52
3.21	12.52	12.59	12.64	12.60	12.77	13.10
3.26	12.90	13.18	13.12	13.27	13.17	13.51
3.31	12.52	12.61	12.67	12.69	12.73	12.97
3.36	13.21	13.31	13.33	13.31	13.30	13.76
3.41	12.90	12.99	13.12	13.14	13.33	13.68
3.46	12.42	12.55	12.67	12.56	12.69	12.97
3.51	13.22	13.54	13.59	13.60	13.79	14.09
3.56	12.36	12.47	12.56	12.55	12.69	12.94
3.61	12.97	13.05	13.11	13.18	13.21	13.33
3.66	12.49	12.64	12.66	12.69	12.66	12.98
3.71	12.92	13.05	13.06	13.24	13.23	13.54
3.76	13.07	13.24	13.29	13.30	13.27	13.52
3.81	12.43	12.33	12.46	12.54	12.73	12.86
3.86	12.70	12.85	12.94	12.93	13.03	13.31
3.91	13.18	13.32	13.37	13.42	13.55	13.86
3.96	12.97	13.00	13.07	13.25	13.17	13.49
4.01	12.84	12.80	12.87	12.85	12.93	13.21

4.06	12.95	13.03	13.06	13.12	13.24	13.45
4.11	12.80	13.05	13.09	13.17	13.20	13.58
4.16	12.82	12.81	12.91	12.78	12.93	13.19
4.21	13.40	13.46	13.48	13.53	13.69	13.86
4.26	12.71	12.79	12.80	12.95	12.97	13.33
4.31	12.92	13.15	13.37	13.39	13.37	13.79
4.36	12.93	12.98	13.10	13.17	13.17	13.38
4.41	12.81	12.99	12.97	13.02	13.14	13.57
4.46	12.79	12.95	12.86	12.90	13.14	13.49
4.51	12.98	13.08	13.11	13.25	13.35	13.62
4.56	12.52	12.56	12.53	12.53	12.74	12.87
4.61	12.51	12.79	12.67	12.73	12.81	13.14
4.66	12.77	12.79	12.88	13.06	13.05	13.36
4.71	12.44	12.51	12.46	12.54	12.64	12.88
4.76	13.06	13.32	13.36	13.27	13.50	13.77
4.81	12.44	12.46	12.44	12.56	12.54	12.84
4.86	12.63	12.71	12.72	12.70	12.75	13.04
4.91	13.10	13.31	13.35	13.31	13.48	13.77
4.96	12.76	12.90	12.88	13.04	13.05	13.35
5.01	12.36	12.48	12.64	12.67	12.80	12.98
5.06	12.88	13.07	13.15	13.15	13.23	13.55
5.11	12.71	12.85	12.77	12.94	13.03	13.25
5.16	12.66	12.80	12.90	12.92	12.95	13.32
5.21	13.11	13.23	13.26	13.30	13.32	13.68
5.26	12.79	12.80	12.89	13.04	13.01	13.30
5.31	12.77	12.85	12.82	12.79	12.96	13.32
5.38	12.82	13.00	13.12	12.93	13.17	13.37
5.45	13.10	13.21	13.18	13.24	13.33	13.57
5.53	12.75	12.84	12.95	12.91	13.01	13.17
5.61	13.01	12.94	12.95	13.05	13.07	13.40
5.70	12.62	12.67	12.74	12.79	12.88	13.20
5.80	12.93	13.04	13.19	13.16	13.28	13.54
5.90	12.51	12.60	12.60	12.58	12.77	13.04
6.01	12.60	12.64	12.69	12.71	12.70	13.13
6.13	12.41	12.46	12.45	12.60	12.68	12.88
6.25	12.18	12.34	12.43	12.59	12.56	12.87
6.39	12.60	12.61	12.74	12.70	12.80	13.03
6.53	12.65	12.71	12.74	12.70	12.83	13.08
6.69	12.08	12.27	12.27	12.28	12.38	12.60
6.85	12.51	12.53	12.67	12.66	12.69	12.98
7.03	12.69	12.82	12.79	12.86	13.00	13.22
7.21	12.40	12.44	12.52	12.57	12.61	12.82
7.41	12.32	12.58	12.51	12.55	12.63	12.93
7.63	12.90	12.91	13.12	13.09	13.23	13.50
7.86	12.21	12.23	12.31	12.40	12.53	12.67
8.10	12.49	12.59	12.58	12.62	12.69	12.91
8.36	12.39	12.43	12.47	12.61	12.60	12.75
8.64	12.39	12.50	12.48	12.50	12.62	13.02
8.94	12.18	12.32	12.39	12.44	12.39	12.73
9.26	12.70	12.89	12.84	12.89	13.07	13.34

9.60	12.24	12.23	12.33	12.30	12.46	12.77
9.97	12.80	13.02	12.96	13.01	13.02	13.33
10.36	12.44	12.51	12.39	12.45	12.57	12.93
10.77	12.89	12.90	12.95	13.13	13.15	13.42
11.22	12.25	12.24	12.23	12.30	12.44	12.67
11.70	12.19	12.22	12.32	12.33	12.32	12.50
12.21	11.76	11.98	11.82	11.80	12.02	12.21
12.75	12.45	12.51	12.60	12.55	12.72	12.85
13.33	12.52	12.64	12.58	12.68	12.82	13.07
13.95	12.05	12.21	12.32	12.26	12.39	12.71
14.62	12.04	12.28	12.08	12.14	12.11	12.55
15.33	12.17	12.36	12.40	12.31	12.45	12.76
16.09	12.07	12.14	12.19	12.15	12.30	12.52
16.90	12.77	12.84	12.83	12.85	13.05	13.26
17.77	12.08	12.08	12.21	12.14	12.18	12.38
18.70	12.22	12.40	12.38	12.39	12.57	12.78
19.69	12.41	12.41	12.43	12.41	12.46	12.68
20.75	12.13	12.28	12.26	12.27	12.39	12.73
21.89	12.44	12.67	12.63	12.66	12.70	12.93
23.10	12.45	12.50	12.47	12.70	12.64	12.95
24.39	11.84	12.10	12.20	12.09	12.26	12.48
25.78	12.59	12.61	12.63	12.66	12.72	13.06
27.26	12.23	12.30	12.35	12.29	12.47	12.81
28.84	12.38	12.54	12.45	12.65	12.79	12.98
30.53	12.27	12.31	12.16	12.31	12.37	12.63
32.34	11.99	12.17	12.14	12.13	12.30	12.46
34.27	11.94	12.10	12.17	12.00	12.11	12.48
36.34	12.05	12.27	12.29	12.29	12.38	12.58
38.55	12.43	12.51	12.50	12.44	12.69	12.87
40.91	12.03	11.99	12.03	12.06	12.17	12.39
43.43	11.57	11.54	11.61	11.71	11.66	11.93
46.13	11.76	11.74	11.79	11.84	11.90	12.08
49.01	12.32	12.38	12.54	12.46	12.62	12.78
52.09	12.32	12.37	12.45	12.48	12.50	12.86
55.39	11.70	11.75	11.85	11.76	11.75	12.11
58.91	11.70	11.75	11.88	11.80	11.82	12.09
62.68	12.01	12.31	12.27	12.30	12.42	12.70
66.70	11.92	12.02	12.00	12.11	12.08	12.34
71.00	12.15	12.14	12.23	12.22	12.22	12.46
75.60	11.98	12.20	12.29	12.24	12.27	12.61
80.51	11.92	11.91	11.87	12.03	11.97	12.17
85.77	12.66	12.75	12.73	12.69	12.82	12.96
91.38	11.88	11.95	11.97	11.98	12.00	12.20
97.39	12.20	12.32	12.30	12.14	12.31	12.63
103.80	12.26	12.32	12.34	12.25	12.23	12.47
110.66	11.63	11.55	11.70	11.66	11.60	12.00
117.99	12.19	12.22	12.22	12.22	12.22	12.46
125.83	11.85	11.89	11.88	11.79	11.92	12.12
134.21	11.66	11.75	11.69	11.65	11.72	11.83
143.17	11.87	12.07	12.01	12.01	12.05	12.30

152.74	11.63	11.60	11.56	11.55	11.61	11.90
162.97	11.82	11.88	11.96	12.01	11.96	12.20
173.91	11.86	11.96	12.02	11.99	11.97	12.34
185.60	11.66	11.80	11.71	11.79	11.76	12.01
198.10	11.88	11.96	11.95	12.04	11.99	12.22
211.46	11.84	11.92	11.93	11.83	11.83	12.05
225.75	12.09	12.15	12.11	12.09	12.07	12.38
241.01	11.46	11.65	11.45	11.53	11.48	11.73
257.33	11.87	11.91	12.05	12.00	11.95	12.28
274.77	11.84	11.74	11.85	11.81	12.07	12.29
293.42	11.85	11.84	11.84	11.83	11.77	12.04
313.35	11.28	11.32	11.34	11.32	11.37	11.58
334.66	11.76	11.85	11.89	11.88	11.91	12.22
357.43	11.97	12.02	12.09	11.98	12.11	12.20
381.78	11.05	11.24	11.02	11.07	10.96	11.29
407.80	11.30	11.38	11.33	11.14	11.26	11.36
435.62	11.71	11.71	11.78	11.78	11.73	11.93
465.35	11.24	11.25	11.21	11.22	11.11	11.37
497.14	11.39	11.49	11.34	11.41	11.37	11.60
531.11	11.18	11.14	11.19	11.17	11.23	11.43
567.43	11.11	11.14	11.15	11.18	11.04	11.40
606.26	11.61	11.55	11.56	11.61	11.53	11.71
647.76	10.80	10.62	10.52	10.64	10.46	10.63
692.12	10.84	10.77	10.70	10.76	10.62	10.86
739.53	10.83	10.83	10.81	10.63	10.71	10.74
790.22	11.11	11.24	11.06	11.07	11.12	11.22
844.40	11.20	11.10	11.03	10.96	10.95	11.11
902.32	10.80	10.82	10.66	10.71	10.70	10.88
964.23	10.91	10.78	10.82	10.66	10.74	10.95
1030.41	10.31	10.20	10.10	10.10	10.16	10.22
1101.15	10.42	10.55	10.54	10.42	10.45	10.63
1176.77	10.50	10.44	10.46	10.43	10.27	10.44
1257.60	10.42	10.25	10.21	10.13	10.18	10.30
1344.00	10.31	10.34	10.37	10.23	10.15	10.26
1436.36	10.25	10.16	10.15	10.11	10.06	10.21
1535.09	10.15	10.06	9.99	9.99	9.99	10.08
1640.63	10.67	10.63	10.56	10.49	10.50	10.60
1753.44	9.97	10.05	9.95	10.05	10.12	10.26
1874.02	10.55	10.52	10.49	10.30	10.39	10.48
2002.92	9.67	9.63	9.65	9.54	9.49	9.64
2140.71	9.62	9.65	9.52	9.39	9.33	9.43
2288.00	9.50	9.38	9.40	9.35	9.34	9.47
2445.44	9.72	9.75	9.77	9.71	9.61	9.83
2613.73	9.23	9.17	9.11	9.09	8.96	9.07
2793.63	8.95	9.11	8.97	8.92	8.79	8.96
2985.93	9.02	9.10	9.06	8.92	8.83	9.06
3191.49	8.96	9.02	8.94	8.92	8.88	9.02
3411.21	8.31	8.23	8.26	8.30	8.26	8.29
3646.09	8.54	8.61	8.64	8.56	8.50	8.67
3897.16	8.12	8.12	8.10	8.16	8.16	8.29

S8. TD-DFT and UDFT results of D-RC-A and A-RC-A

Table S9. Cartesian coordinates of the TD-DFT-optimized ground state structure S_0 of D-RC-A.

Rh	0.19319600	-0.17878700	0.03356900
C	-1.11725200	1.38628500	-0.02803000
C	1.53070800	1.34406000	-0.01269000
C	-0.48532800	2.60332200	-0.06533800
C	0.94549100	2.58137200	-0.05614500
C	1.48242600	3.98840900	-0.10234600
C	0.26408300	4.82838700	0.33819200
C	-0.97285200	4.02810200	-0.12283300
O	1.70042000	-1.74072300	0.09776800
C	1.46054500	-2.98160800	0.12681600
C	0.19945400	-3.61069400	0.13365100
C	-1.06662900	-2.99468100	0.11158000
O	-1.31873100	-1.75423800	0.07904100
C	2.68856200	-3.86540100	0.15566400
C	-2.28728400	-3.88919900	0.12878000
C	-2.50228000	1.22928400	-0.03450500
C	2.91062900	1.12385000	-0.01150100
P	0.11934700	-0.06696300	2.36737100
P	0.15717800	-0.21671400	-2.30339600
C	0.60799800	1.49846300	3.17565500
C	-1.54511800	-0.39664800	3.04986900
C	1.17907700	-1.30869200	3.19599900
C	1.22320900	-1.51141500	-3.03813100
C	-1.49618500	-0.58400300	-2.99394700
C	0.66347900	1.29703900	-3.19572400
C	-3.71465300	1.03657200	-0.03982500
C	4.11318200	0.88377500	-0.01905600
[truncated: 34,895 more chars]
